# Structure, Evolution, and Mitochondrial Genome Analysis of Mussel Species (Bivalvia, Mytilidae)

**DOI:** 10.3390/ijms25136902

**Published:** 2024-06-24

**Authors:** Yuri Phedorovich Kartavtsev, Natalia A. Masalkova

**Affiliations:** A.V. Zhirmunsky National Scientific Center of Marine Biology (NSCMB), Far Eastern Branch, Russian Academy of Sciences, 690041 Vladivostok, Russia

**Keywords:** mitogenome structure, evolution, molecular diversity, phylogenomics, multigene phylogenetic reconstructions, divergence time, protein-coding genes (PCGs), genetic distance, coalescent analysis, BEAST, Bayesian simulation, DNA barcoding, systematics, mussel, MRCA, most recent common ancestor

## Abstract

Based on the nucleotide sequences of the mitochondrial genome (mitogenome) of specimens taken from two mussel species (*Arcuatula senhousia* and *Mytilus coruscus*), an investigation was performed by means of the complex approaches of the genomics, molecular phylogenetics, and evolutionary genetics. The mitogenome structure of studied mussels, like in many other invertebrates, appears to be much more variable than in vertebrates and includes changing gene order, duplications, and deletions, which were most frequent for tRNA genes; the mussel species’ mitogenomes also have variable sizes. The results demonstrate some of the very important properties of protein polypeptides, such as hydrophobicity and its determination by the purine and pyrimidine nucleotide ratio. This fact might indirectly indicate the necessity of purifying natural selection for the support of polypeptide functionality. However, in accordance with the widely accepted and logical concept of natural cutoff selection for organisms living in nature, which explains its action against deleterious nucleotide substitutions in the nonsynonymous codons (mutations) and its holding of the active (effective) macromolecules of the polypeptides in a population, we were unable to get unambiguous evidence in favor of this concept in the current paper. Here, the phylogeny and systematics of mussel species from one of the largest taxons of bivalve mollusks are studied, the family known as Mytilidae. The phylogeny for Mytilidae (order Mytilida), which currently has no consensus in terms of systematics, is reconstructed using a data matrix of 26–27 mitogenomes. Initially, a set of 100 sequences from GenBank were downloaded and checked for their gender: whether they were female (F) or male (M) in origin. Our analysis of the new data confirms the known drastic differences between the F/M mitogenome lines in mussels. Phylogenetic reconstructions of the F-lines were performed using the combined set of genetic markers, reconstructing only protein-coding genes (PCGs), only rRNA + tRNA genes, and all genes. Additionally, the analysis includes the usage of nucleotide sequences composed of other data matrices, such as 20–68 mitogenome sequences. The time of divergence from MRCA, estimated via BEAST2, for Mytilidae is close to 293 Mya, suggesting that they originate in the Silurian Period. From all these data, a consensus for the phylogeny of the subfamily of Mytilinae and its systematics is suggested. In particular, the long-debated argument on mussel systematics was resolved as to whether Mytilidae, and the subfamily of Mytilinae, are monophyletic. The topology signal, which was strongly resolved in this paper and in the literature, has refuted the theory regarding the monophyly of Mytilinae.

## 1. Introduction

Genetic and taxonomic studies on mussels are essential from the point of view of the special place of this group among mollusks. Many mussel species are used in laboratory practice as the objects of study. Accordingly, unresolved taxonomic status may lead to errors being introduced in various studies. Also, it is important that most species are used by the fishery industry, are reared in mariculture, or are part of the food industry. Therefore, clarity in the systematics for such taxa is of significant importance. The importance of this study is also associated with the hybridization of species that are found within the *Mytilus* ex. group *edulis* complex, in the World Ocean [1,2,3,4,5,6,7], which opens the need for ecological and genetic monitoring and the definition of taxa ranks by the combined methods of evolutionary genetics, population genetics, and genomics. There are other issues related to the concept of the experimental analysis of a species, the genetic bases of speciation, the formation of a quantitative theory of speciation, and the diversity of the mitochondrial genomes in the water realms, which are still poorly developed. Resolution of the questions raised above will noticeably advance existing generalizations in the literature and in the notion of the importance of molecular polymorphism to molecular science and evolutionary biology.

Mussels (Bivalvia, Mytilidae) are characterized by a large variety of species. According to various estimates, they include from 250 [8] to 400 modern species, currently grouped into around 76 genera [9] (WoRMS 2024; https://www.marinespecies.org/aphia.php?p=taxdetails&id=224589; accessed on 15 April 2024). The evolutionary biology and systematics of mussels have attracted considerable attention from researchers. However, most of the phylogenetic hypotheses put forward during the last century on the basis of classical morphological studies of Mytilidae in general, as well as of subfamilies such as Mytilinae and Modiolinae, remain controversial. One molecular phylogenetic hypothesis concerning mytilids was proposed by Distel in his study based on nuclear DNA (nDNA) sequences, such as the *18S* rRNA gene [10]. This phylogeny included only 12 species belonging to 6 genera, as well as several species from other families that were taken as the outgroup. In his study, the author used the classification proposed by Newell [11], which considered the Mytilinae as polyphyletic. In addition, Distel [10] pointed to a similar divergence rate of the *18S* rRNA gene in the examined mytilid genera. Moreover, the level of this divergence was approximately equal to the differences between the representatives of different families of Bivalvia. In addition, Distel proposed a hypothesis on the multiple origins of the modioloid- and mytiloid-like body structure of mussels. However, this hypothesis was later questioned by Chichvarkhin [12]. Since Distel’s study, mytiloid-like forms other than Mytilinae have not been included in the mussel group, which is also because the shell of the representatives of the genus *Hormomya*, that was the cause of Mytilinae *sensu* Newell polyphyly, can in fact hardly be classified as mytiloid-like. It should also be noted that Distel’s phylogeny exhibited considerable disagreements with the data on spermatozoa morphology in Mytilidae [13]. In addition to the above-mentioned study on nDNA, in recent years, phylogenetic reconstructions of mytilids were carried out with the use of mitochondrial DNA (mtDNA) genes and whole mitochondrial genomes (mitogenomes), which are usually more variable and may be more suitable for analyzing the level of species and order divergence [14]. However, in the case of mussels, their use may be complicated by the considerable divergence of the paternal and maternal genomes, due to the doubly uniparental inheritance of mtDNA [15,16,17,18]. This requires separate analysis of the male and female samples, which is not always possible in practice. Recently, several advanced papers on the molecular systematics and phylogenetics of mussels were published [3,19,20,21,22,23,24,25]. A molecular phylogeny study that included five subfamilies and used nuclear (*18S*, *28S* rDNAs and histone *H3*) and mitochondrial (*cox1* and *16S* rDNA) genes found two major clades, the Modiolinae + Bathymodiolinae clade and the Mytilinae clade (composed of *Septifer*, *Musculus*, and *Brachidontes* species) with polyphyletic Lithophaginae members [24]. In other research [25], the complete mitochondrial genomes of nine Mytilidae species were determined, which represented seven Mytilidae subfamilies and included the sequenced representatives from the subfamilies of Septiferinae and Lithophaginae in the Mytilidae, for a total of 28 mytilid mitogenomes to infer phylogenetic inter-relationships and estimate divergence time among the subfamilies.

In addition to the best-known classification, which is based on paleontological data and is called the Soot-Ryen classification [26], as successively used by Newell and Distel, several other schemes for the classification of mytilids have been proposed. After the first review by Soot-Ryen [27], it became common practice to identify four subfamilies among the mytilids, including Mytilinae Soot-Ryen, 1955, Crenellinae Gray, 1840, Lithophaginae H. Adams et A. Adams, 1857, and Modiolinae G. Termier et H. Termier, 1950. This list continues to expand, reaching 12 subfamilies in recent updates [28] (WoRMS 2024; https://www.marinespecies.org/aphia.php?p=taxdetails&id=211; accessed on 8 April 2024).

A different system that is presently accepted in part for other taxonomic ranks [28] was formerly suggested by Scarlato and Starobogatov [29,30,31], in which the Mytiloidea were divided into four separate families, namely, the Mytilidae, Crenellidae, Septiferidae, and Lithophagidae. The rank of family was also given to Lithophagidae by the authors of [32], although this taxon was not treated later in this rank [33]. Scarlato and Starobogatov distinguished 13 additional subfamilies [34]. This system also implied the polyphyly of Soot-Ryen’s family, Mytilidae. Unfortunately, the diagnoses of their taxa were too short, and the proposed classification scheme had no explanation. Nevertheless, some of their taxa are now accepted but are now in different ranks. Many other literature sources have been considered for taxa classification, as indicated in the introduction above.

For advanced consideration of the subject area, it is obvious that a molecular phylogenetic investigation based on the current and previously tested mitogenomes, as well as on nuclear DNA markers (nDNA) such as *18S* rRNA, *28S* rRNA, histone *H3,* etc., or both, is extremely relevant. It has important and fundamental significance, in terms of clarifying the taxa systematics, genetic divergence patterns, and the extended records on paleontology dating through a reference exchange with molecular clock dating, as well as its potential applications in mariculture and fishery practices in general. In the current research, we employed up to 100 sequences for general analysis and the mitochondrial genomes of 26–27 mytilids (including the 4 newly sequenced specimens) as the basic set and used them to infer mitogenome structure and the factors impacting its variability, the phylogenetic interrelationships, and the divergence times among the taxa of the Mytilidae family. 

## 2. Results

### 2.1. Structure and Variability of the Mitochondrial Genome of Arcuatula senhousia, Mytilus coruscus, and Other Representatives of the Mytilidae Family

**Figure 1:**
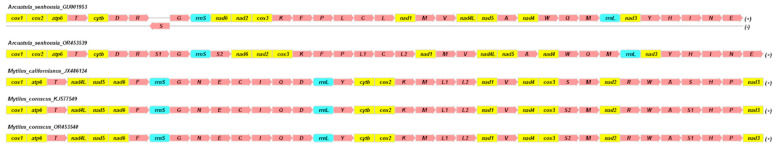
A map of the mitochondrial genomes of five mussel species: *Arcuatula senhousia* (two sequences), *Mytilus coruscus* (two sequences), and *Mytilus californianus* (one sequence), which serves as a brief sample representation of the Mytilidae taxa. More details on mitogenome diversity are given in Figures S1 and S2 in the Supplementary Materials. The composition for the chains of the mitogenomes is given in abbreviated form and in color. The mitogenomes include: 12 protein-coding genes (abbreviated on the map as follows: *atp6*, *cox1*, *cox2*, *cox3*, *cytb*, *nad1*, *nad2*, *nad3*, *nad4*, *nad4L*, *nad5*, and *nad6*; shown by the yellow color), 2 rRNA genes: rrnS, rrnL (abbreviated as *12S* rRNA and *16S* rRNA; shown by the blue color), and 22 tRNA genes that are abbreviated as coding for specific amino acids: T-Trp, C-Cys, E-Glu, Y-Tyr, R-Arg, G-Gly, H-His, L1-Leu1, L2-Leu2, S1-Ser1, S2-Ser2, Q-Glu, F-Phe, M-Met, V-Val, A-Ala, D-Asp, N-Asn, P-Pro, I-Ile, K-Lys, and W-Trp (all are shown by the red color). The genes in the majority are located on the “+“ strand; the genes shown as shifted into a separate line indicate their location on the “−” strand.

The mitogenome of *A. senhousia* (Ar2 specimen) comprises 17,675 bp (GenBank accession No: OR453539). It includes 12 protein-coding genes, 22 tRNAs, 1 *12S* rRNA, and 1 *16S* rRNA gene (Figure 1), along with a control region, CR (Table 1). Another sequenced mitogenome from *Mytilus coruscus* (Kart1 specimen; GenBank accession No: OR453540; Figure 1, Table 1) is generally similar.

Most of the genes of the mitogenome are located on the “+” strand, except for several tRNA genes, which are located on the “−” strand (Figure 1 and Figures S1 and S2 in the Supplementary Materials). For clarity, the data on the structure of the mitogenome sequences in Figure 1 are given only for three members of Mytilinae, including the two specimens for each of the two species that we describe herein (Table 1). Visual map data are extended in the Supplementary Materials for 26–27 mitogenome sequences (Figures S1 and S2). From the maps, the presence of numerous transpositions of the genes is obvious, including tRNA, rRNA, PCGs, and the availability of the *atp8* gene in 14–15 sequences among 26–27 (53.8–57.7%) (Figures S1 and S2). In other words, the order of genes in the studied taxa of Mytilidae is not strictly conserved, as evidenced by the maps (Figure 1, Figures S1 and S2). Contrary to the norm for vertebrates, not a single *nad6* is located on the “−” strand. The most conserved example is the mitogenome of the genus *Mytilus*, plus *Crenomytilus grayanus*, for which 10 compared sequences have the same gene order (Figure S2), excluding the deletion of the Q-block of tRNA coding for the Glu amino acid and the presence-absence of the *atp8* gene (this point will be considered in more detail in Section 3).

**Figure 2:**
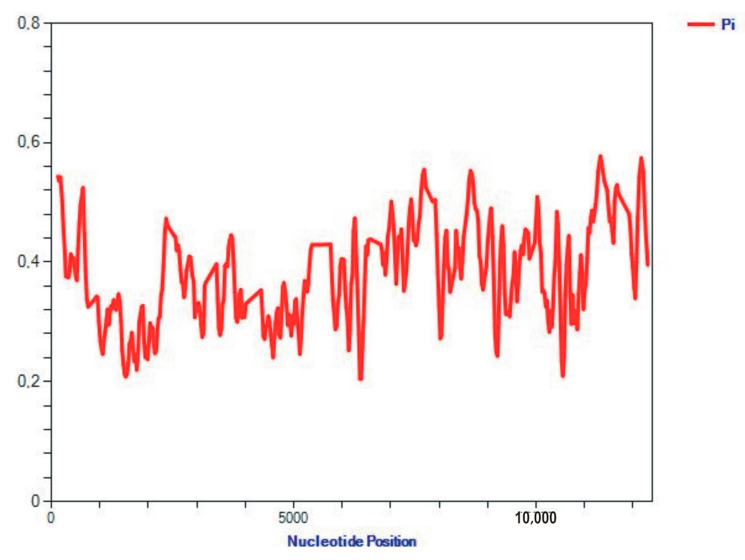
The plot of the distribution of the nucleotide diversity per site (Pi) along the whole length at 12 PCGs of the 26 mussel mitogenomes in the Mytilidae. On the *y*-axis, the diversity scores are given as Pi. The red line represents the Pi variation along the *x*-axis at nucleotide positions along the DNA chain. The window length is 100 and the step size is 25. More details on DNA polymorphism are given in the text.

The majority of the protein-coding genes of mussels use the ATG start codon, but the ATA and ATT are common as start codons as well. For example, the ATG start codon appears in 19 out of 36 (or 52.8%) PCGs of the three species exemplified in Table 1. A typical three-nucleotide stop codon, the TAA, appears in 28 out of 36 (or 77.8%) PCGs, while atypical or incomplete TAG/T stop codons represent the rest, at around 22% (Table 1). The control region (CR; D-loop) is 653 bp long in *A. senhousia* and 60 bp long in *M. coruscus*, but it is undetected in *M. californianus* (Table 1). Our analysis of the sequences showed a very high variability and the informative capacity of the 12–13 PCGs among the investigated members of the mussel family, Mytilidae. The nucleotide diversity per site, Pi (π, Pi for simplicity herein and after), along the sites of the sequences of the 12 PCGs varies widely, as evidenced by the plot built using the software package (SP) DNAsp-6 (Figure 2). The nucleotide diversity, Pi, is the most representative measure of gene variability [35] (equation 10.5) and will be used in many comparisons in this paper.

However, Pi, and especially Hd (see below), were generally similar between genes (Table S5 in the Supplementary Materials). This conclusion is supported by the values of several variables, extracted from Table S5 for illustration: number of polymorphic (segregating) sites, S: 7552; total number of mutations, Eta: 15,557; number of haplotypes, h: 25; haplotype (gene) diversity, Hd: 0.997; variance of Hd: 0.00014; standard deviation of Hd: 0.012; Pi: 0.37673; Theta (per site) from Eta: 0.40557.

The statistical analysis revealed that Pi and Hd did not differ significantly between the 12 PCG of the 26 mitogenomes, averaging at about 38% and 100%: Pi = 0.377 ± 0.004, Hd = 0.997 ± 0.012. According to our analyses, the mitogenome sequences hold significant information capacity to yield the relevant phylogenetic signal, which will be illustrated in more detail with numerical support for the main data sets of mussel species later in this study.

### 2.2. Analysis of the Sequence Properties

As an overview, we provide below several descriptors of variation and nucleotide composition in the set of 26 sequences (following the DNAsp style): sites with alignment gaps or missing data: 2302; invariable (monomorphic) sites: 2500; variable (polymorphic) sites: 7552; total number of mutations: 15,557; singleton variable sites: 408; parsimony informative sites: 7144.

The total variability of the nucleotide frequencies of the four different types shows a slight prevalence of purines (T + C) over pyrimidines (A + G); i.e., T + C = 26.18 ± 1.14% and A + G = 23.82 ± 0.50%. The mean frequencies of the four nucleotides across the three codon positions for 12 PCGs among 26 sequences were as follows: T, 39.18 ± 1.48%; C, 13.18 ± 0.79%; A, 24.67 ± 0.32%; and G, 22.96 ± 0.69%, while for the singleton positions in the same 26 sequences, they were: 46.72 ± 1.48, 10.36 ± 0.79, 21.10 ± 0.32, and 21.82 ± 0.69 (Table 2 and Table S6).

**Figure 3:**
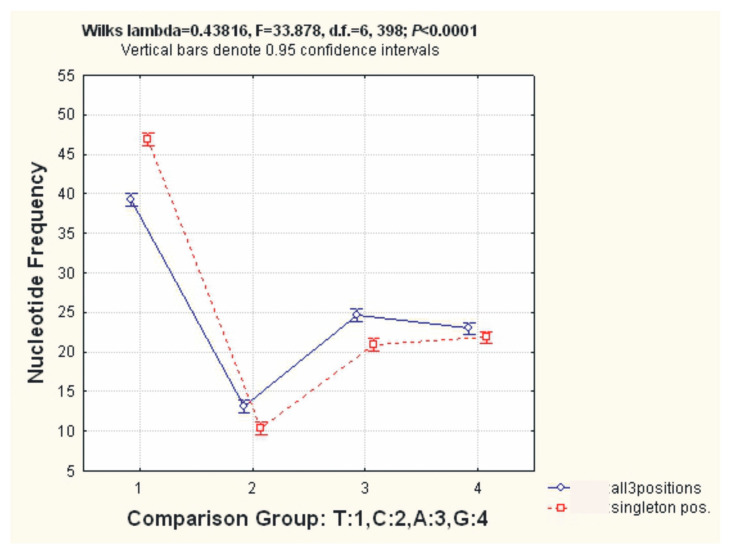
Nucleotide frequency (%) of 12 PCGs in the mitogenomes of 26 mussel specimens. Average nucleotide frequencies are shown for all codon positions and for the singleton positions only. According to the results of the two-way analysis of variance (MANOVA), there are statistically significant differences in the nucleotide content.

The differences in nucleotide frequencies for the three codon positions and for only the singleton positions for 12 PCGs among 26 sequences were visualized after performing a MANOVA using SP Statistica 6 (Figure 3). The heterogeneity of the nucleotide frequencies between the four types of nucleotides was statistically significant for the set of 26 mitogenomes in the two data sets presented: Wilk’s Lambda = 0.43816, F = 33.878, d.f. = 6, *p* < 0.0001.

One of the key points of the sequence properties is their functional significance. The possibility of such an interpretation of the data on nucleotide substitutions is evidenced by 2–3 times higher proportions of synonymous (s) over nonsynonymous (a) substitutions in the codons. For example, the nucleotide diversity of Pi(s) = 0.637, while Pi(a) = 0.288; thus, the Pi(s)/Pi(a) ratio is 2.21. Unfortunately, DNAsp-6 does not give the estimates of the significance of the differences in Pi values. Beyond the point presented in the sentence above, the estimation of the difference between the substitution numbers for synonymous and nonsynonymous sites (K) Ks and Ka, respectively (p. 219, [36]) is possible. Higher values of Ks over Ka are to be expected if there is a natural purifying selection process against deleterious mutations (substitutions) in the polypeptides. Mutations in the synonymous sites are “invisible” to natural selection because they do not lead to amino acid substitutions. In our research on mussels, several tests were made with the set of 26 sequences for 12 PGGs in the entire continuum of the variability of the pairwise Ks/Ka scores in set (i), as well as in the single genus comparisons, within *Mytilus* (ii). The ANOVA results for the set (i) showed highly significant and statistically greater Ks in the set of 26 mitogenomes: Ks = 1.618 ± 0.019, Ka = 0.384 ± 0.019 (*n* = 325), F = 1949.2, d.f. = 1; 648, *p* < 0.0001 (effective hypothesis decomposition). However, an ANOVA may produce biased results, because deviation from the normal distribution was detected in our analysis. Therefore, nonparametric testing for these data was performed. This analysis proved the above conclusion on the Ks/Ka difference: median test with chi-square, *X*^2^ = 535.54, d.f. = 1, *p* < 0.001; Kruskal–Wallis test, *H* = 413.44, *n* = 650, d.f. = 1, *p* < 0.001. For the set (ii) ANOVA, the mean score difference for Ks and Ka was: Ks = 1.069 ± 0.071, Ka = 0.052 ± 0.072 (*n* = 73); F = 100.9, d.f. = 1; 71, *p* < 0.00001. The nonparametric statistics also support the Ks/Ka score differences: median test with chi-square *X*^2^ = 41.47, d.f. = 1, *p* < 0.0001; Kruskal–Wallis test, *H* = 36.50, *n* = 73, d.f. = 1, *p* < 0.0001.

Ending with Section 3.2, let us consider the third sort of estimates regarding the selective value of the data for the molecular markers. Following DNAsp-6, as available from the comparison of all sites for the set of 26 sequences, it is technically possible to apply two basic tests: Fu vs. Li’s D* and the Tajima test statistic for the neutrality of polymorphism. Because these tests are applicable to a single species population, we have compared the most homogeneous data set in our paper that may be suitable for testing, that of the *Mytilus* species group. For the single genus comparisons, within the *Mytilus* results, there were no significant differences in Ks/Ka. Thus, Fu and Li’s D* test statistic was 0.73570, with statistical significance: not significant, *p* > 0.10; Fu and Li’s F* test statistic: 0.68887, with statistical significance: not significant, *p* > 0.10. In other words, all the results above regarding Fu and Li’s statistics do not disprove the hypothesis of the neutral source of sequence variability.

The usage of the Tajima statistics gave a more complex output when testing the neutrality and the differences in Ks/Ka. However, in the single genus comparisons, within *Mytilus*, the results showed no significant scores for neutrality testing, nor for differences in Ks/Ka. Thus, for the coding region, Tajima’s D was 0.30510, not significant, and *p* > 0.10. Analysis of the polymorphic sites included 2840 of them. The results for the synonymous sites were as follows: Tajima’s D(Syn): 0.33587; not significant, *p* > 0.10. Total number of polymorphic sites: 2344 (Changes: 2858; Syn. Changes: 2833); for nonsynonymous sites: Tajima’s D (NonSyn): 0.56510, *p* > 0.10; total number of polymorphic sites: 521 (Changes: 561; NonSyn. Changes: 535); silent sites: Tajima’s D(Sil): 0.35403, *p* > 0.10). More details on nucleotide diversity are provided in the Supplementary Materials (Tables S7 and S8).

### 2.3. Reconstruction of the Gene Trees and Analysis of the Molecular Phylogenetic Relationships

Before reconstruction of the phylogenies, a special investigation was performed to evaluate whether the sequences of mussels from GenBank are homogeneous in terms of sexual diversity between the F- and M-lines. The genetic diversity estimated by DNAsp-6 for the F- and M-lines in the genus *Mytilus* showed similar values for both lines, but the differences were, nevertheless, statistically significant. The measurements of total variability, again following DNAsp-6, were: number of sequences: 20; number of segregating sites, S: 4161; number of haplotypes, h: 19; haplotype diversity, Hd: 0.99474; average number of nucleotide differences, Kt: 1303.37368; nucleotide diversity, Pi: 0.10105. The estimates of genetic differentiation for the F/M lines with slightly modified DNAsp-6 output were: chi-square (table, *X^2^*), *X^2^* = 20.0, *p* = 0.3328, NS (d.f. = 18). HBK 1992, Hs: 0.99176; Hst: 0.00299; PM test; *p*-value of Hs, Hst: 0.5640, NS; HBK 1992, Ks: 739.11077; Kst: 0.43292; permutation (PM) test; *p*-value of Ks, Kst: 0.0000 *** (*** *p* < 0.001; PM test; probability obtained by the PM test with 1000 replicates). The statistics obtained by DNAsp-6 allowed us to estimate the gene flow among F/M lines. Information on the sequence data was combined following the method in [37]: DeltaSt = 0.04682; GammaSt = 0.48770; Nm = 0.53. Following the method in [38] (with Jukes and Cantor correction), the output statistics were: Nst = 0.55342, Nm = 0.40. While following the method in [39], these key statistics are: Fst: 0.54918, Nm: 0.41. Therefore, the parameter of the interline gene flow for the three estimates was averaged to Nm = 0.45. Such a score of Nm indicates a very small amount of gene flow for the intraspecies groups, and it is close to the interspecies scores of the genetic exchange (see more on this point in the Discussion). The performed investigation, which detected a high level of differences between F/M lines, leads us to a conclusion on the necessity of usage in further analyses of the two separate sets of the sequences, with one for the maternal and another for the paternal mitogenome lines. Such a distinction is especially important for reconstructions of the phylogeny and in the building of gene trees.

The sequences of the mitogenome used for the main analysis hold significant information capacity to give a relevant phylogenetic signal. This fact is illustrated below with the numerical data for the mussel species examined in this study (Table S9 in the Supplementary Materials). An important point for such an analysis is to evaluate the saturation effect among the mitogenome sequences in our investigation of mussels. The nucleotide composition saturation was tested by comparing the Iss and Iss.c indices for the 25 mitogenomes of the Mytilidae family: Iss = 0.5985, Iss.c = 0.8432, t = 39.1625, d.f. = 8443, *p* < 0.0001 (two-sided *t*-test, SP DAMBE 7 [40,41]). Analysis was performed on the fully resolved sites only. The significant differences between the Iss and Iss.c indices indicate low or absent composition saturation. Therefore, a minimal impact of saturation on the topology is expected. In this analysis, the two sequences of *M. chilensis* were combined as they are nearly identical. Thus, the sequence set was reduced from the original 26 to 25 in number. The test on the presence of the phylogenetic signal employed the methods of Xia et al. [40,41], and, with modifications, gave a similar conclusion, i.e., the set of 26 sequences tested has a strong phylogenetic signal and it is statistically highly significant (Table S9).

**Figure 4:**
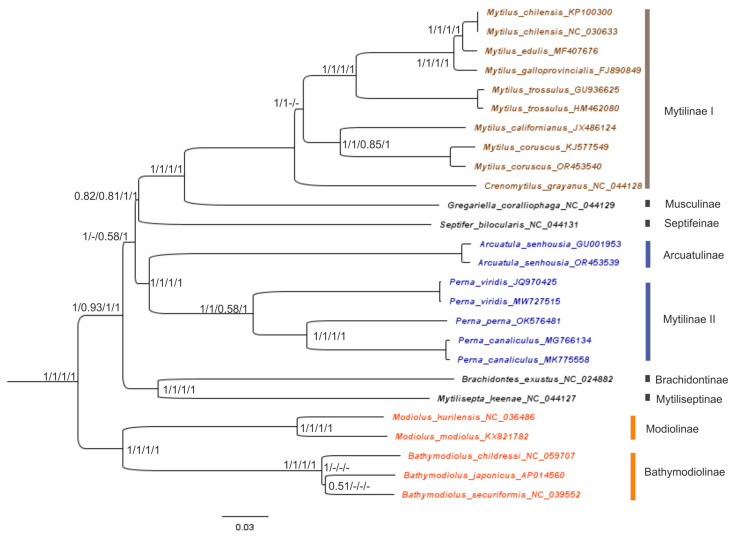
The combined phylogram built using NJ, MP, ML, and BI techniques, and the analyses performed in SP MEGA-11 for NJ, MP, and ML, and in SP MrBayes for BI. The tree reconstruction was based on 12 PCG sequences of 26 mussel representatives of the Mytilidae family. For NJ, MP, and ML trees, support for the topology scores was obtained by a bootstrap test with 1000 replications. The BI inference support for the nodes is defined by the posterior probabilities, estimated with 10^5^ generations. The support scores are shown besides the nodes only for interspecies and higher-rank clades. The NJ tree is basic, for representation of the four topologies. The tree here and trees in Figure 5 and Figure 6 are rooted with the *Modiolus-Bathymodiolus* branch. The scale below the phylogram shows the branch length in distance units. All the scores of the supports for the nodes are given as probabilities, both for bootstrap frequencies and for BI. All the intraspecies support scores are close to 1. For convenience, they are omitted in this and the following phylograms (Figure 5 and Figure 6). The scores of supports are given in this order: NJ/MP/ML/BI. Scores of less than 0.50 are denoted with a dash. The best-fit model, GTR + I + G, was implemented where appropriate. Subfamily divisions, shown on the right, follow the WoRMS resource system (https://www.marinespecies.org/aphia.php?p=taxdetails&id=211; accessed on 2 April 2024).

**Figure 5:**
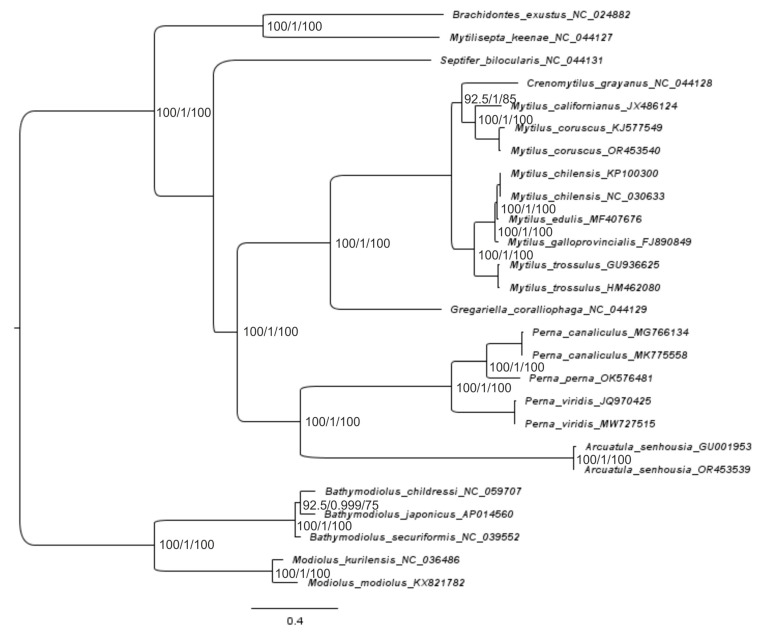
The phylogram built using PhyloSiut Version 1.2.3 software and its IQ-TREE utility. The gene tree is based on 12 PCGs of the mitogenome for 26 mussel representatives of the Mytilidae family. Bootstrap support scores were obtained with 5000 replications for IQ-TREE, performed in the three modes of ultrafast bootstrap, BI-inference, and the SH-aLRT branch test, as defined in the Materials and Methods Section. The support values are shown beside the nodes with a slash, in the following order: ML, %/BI, probability/SH-aLRT, %. Best-fit model: GTR + F + I + G4, chosen according to BIC. The tree was rooted with *Modiolus-Bathymodiolus*. The scale below the phylogram shows the branch length in distance units.

**Figure 6:**
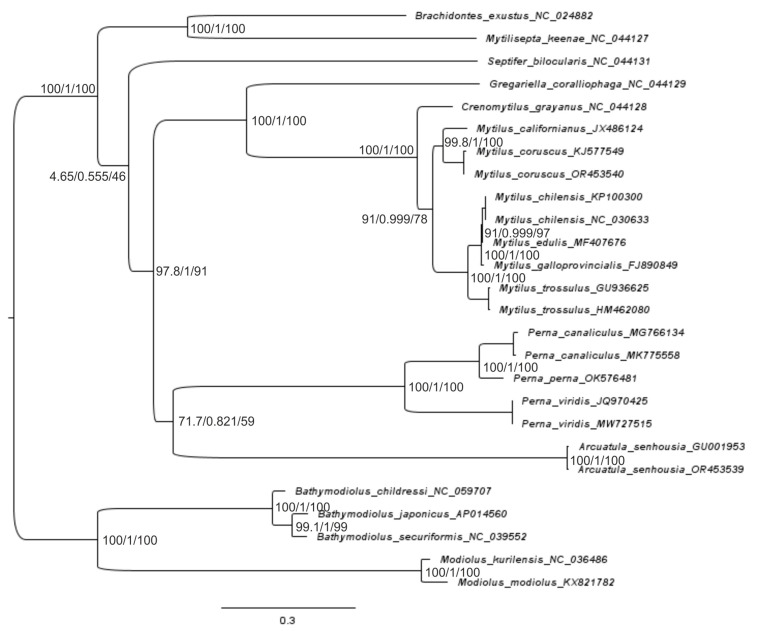
The phylogram, built using PhyloSiut software and its IQ-TREE utility. The gene tree is based on the rRNA and tRNA sequences of 26 analyzed mussel representatives of the Mytilidae family. Bootstrap support scores were obtained with 2000 replications for IQ-TREE, performed in three modes, namely, the ultrafast bootstrap, the BI inference, and the SH-aLRT branch test. Support values are shown beside the nodes with a slash, in the following order: ML, %/BI, probability/SH-aLRT, %. Best-fit model: GTR + F + I + I + R3, chosen according to BIC. The tree was rooted with *Modiolus-Bathymodioulus*. The scale below the phylogram shows the branch length in distance units.

**Figure 7:**
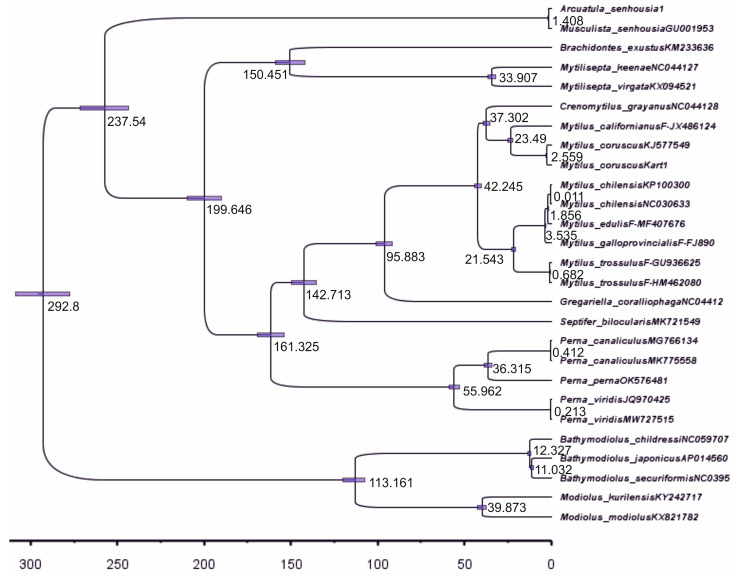
CA-based time-tree reconstruction, created via SP BEAST-2 and with the FigTree utility, using 12 PCGs from the mitogenomes of 27 analyzed mussels of the Mytilidae family. Details of the analysis are given in the text. The estimated ages are given next to the nodes, after rooting the tree with the outgroup taxon *Modiolus* and converting the scale in the nodes to the root age of 292.8 Mya. Blue bars represent CA 95% HDP (high-density probability) for the node ages. This tree was built by running the following file: Mytilidae27sq-no_pat-pt123ps.xml.

An overview of the phylogenetic relationships based on 26–27 mitogenome sequences between the species of the Mytilidae family, along with their time divergence, is presented for 12 PCGs and the rRNA-tRNA of the F-line (Figure 4, Figure 5, Figure 6 and Figure 7 and Figure S3). For the gene trees built with four techniques using the sequences of 12 PCGs of 26 mitogenomes, the topology is clearly resolved for all branches (nodes) except the branch for *Perna + Arcuatula–Gregariella + Crenomytilus + Mytilus*, with supporting scores for this branch: 45% for the SH-aLRT branch test (Figure 5), 58% for the ML test (Figure 4), complete lack of support for MP, and high scores for BI inference of 0.99 (Figure 4).

The gene trees were built with four techniques and used the sequences of 12 PCGs for 26 mussel mitogenomes. The topology is clearly resolved for most branches (nodes) except the branch for *Perna + Arcuatula–Septifer + Gregariella + Crenomytilus + Mytilus*, which has support scores of 1/–/0.58/1, respectively, for the four defined techniques and the highest scores for NJ and BI inference, which are equal to 1.0 (Figure 4). Branch joining *Crenomytilus* and *Mytilus* is not supported well by the probabilistic techniques of ML and BI (Figure 4). Furthermore, discordant taxa joining was obtained by using different techniques of tree building for *Bathymodiolus* species (Figure 4).

Figure 5 shows a gene tree built with three techniques, using the combined sequences of 12 PCGs of 26 mitogenomes. The topology is clearly resolved for all branches except the branch for *Arcuatula + Perna* and the complex branch, *Gregariella + Cremomytilus + Mytilus*, where the support scores are equal to 45.2% for the SH-aLRT branch test, 58% for the ML test, and 0.99 for BI inference (Figure 5).

The topology of the next tree that was built with rRNA + tRNA sequences is well supported statistically for most nodes (Figure 6). Here, the support is weak for the big branch aggregation of the *Gregariella + Crenomytilus + Mytilus* with *Arcuatula + Perna* and is nearly identical in other properties with the NJ/MP part of the tree shown in Figure 4. The gene trees that were built with the same techniques but used PCGs or rRNA + tRNA sequences are quite different. Especially important here, from the taxonomic point of view, is the difference in clustering at the branch for *Crenomytilus–Mytilus* (see Figure 4, Figure 5 and Figure 6). The tree that combined a signal from both 12 PCGs and rRNA + tRNA sequences (Figure S3) has a topology and properties that are similar to the PCG-based tree (Figure 4). Such a result is quite understandable since the information capacity of 12 PCGs for the set of 26 sequences is much higher than that of rRNA and tRNA genes because the latter set is shorter and has fewer variable sites. However, the rRNA + tRNA sequences are more conservative, which makes them useful at the genus-subfamily level. This point will be discussed in a wider context later, when discussing the reconstruction of the gene trees and the analysis of the molecular phylogenetic relationships reported in Section 4.3.

The reconstruction of phylogenies by using comprehensive models and the complicated partitions of nucleotides, such as coding for different PCGs, which are made possible by modern software, does not always appear to be advantageous in the context of obtaining better topology (see Figure 5 and Figure 6), when compared to less comprehensive methods such as NJ/MP (Figure 4). We develop this point more thoroughly in the Discussion Section below. Before that, let us consider the final analyses of the molecular evolution of the mussels, as obtained by coalescent analysis (CA) via SP BEAST-2 v2.6.5–2.6.7 (Figure 7).

Before discussing Figure 7, let us note the distinct names used for the specimens of *Arcuatula* in the figure. One is the laboratory name *Arcuatula sehousia1*, which was used before its registration in GenBank occurred (see the Materials and Methods Section). The second, *Musculista senhousia*, is a synonymous name for *Arcuatula sehousiaGU001953*, corrected in the later releases of GenBank (Table S1 in the Supplementary Materials). This is a simple but illustrative example of the usefulness of DNA barcoding at the mitogenome level, i.e., species–specimen identification by molecular markers. The time tree in Figure 7 reveals the good correspondence of the gene tree topology with the other phylogenies given in Figure 4 and Figure 6. There are certain discrepancies in the branching pattern for *Crenomytilus–Mytilus* and *Bathymodiolus*, as noted above. The reconstruction yields a date close to 293 Mya for the divergence of the sampled representatives of the Mytilidae family from *Modiolus modiolus* and *M. kurilensis* (Figure 7). The MRCA of these two taxa was dated at 292.8 Mya and was calibrated to the time of the divergence of the common ancestor of Mytilidae-Modiolinae (Modiolidae, in some classifications), as noted in the Materials and Methods and the Introduction Sections. For building the tree depicted in Figure 7, a special file with the sequence matrix (Table S3: An example file for the set of 27 mitogenome sequences of the Mytilidae family, named Mytilidae27sq-no_pat-pt123ps.xml), and the set of parameters used for the tree simulation by the CA of the constant population were applied in the different runs. Table S3 was built using BEAUti v2.6.6, the utility of SP BEAST-2, which starts by exporting the BEAUti-made file, Mytilidae27sq-no_pat-pt123ps.xml. The basic model parameters could be read from this file using BEAUti v2.6. After running the main SP BEAST v2.6.6 with implemented *n* = 5 × 10^7^ generations and a variety of other parameters that were appropriate for a successful simulation, the sets of trees and other estimators were obtained. A description of the simulation procedure and the parameters used are given below for the six items. By running SP TreeAnnotator v2.6.4 and loading the SP BEAST output tree file, the total number of trees was defined as 50,002; 25,001 of them were used. Thus, 25,001 trees were processed after discarding the first 50% = 25,000 trees. The final output with the credibility tree contained 28 unique branches (clades in that SP). The maximum credibility tree was constructed as mentioned by using the SP TreeAnnotator, based on the SP BEAST output tree file, Mytilidae27sq12PCGs.trees (available on request). This file is suitable for the next steps of processing in the FigTree 8.1 software, which is produced in cooperation with the SP BEAST-2 authors. 

The quality of the model simulations was estimated through the parameters obtained from special runs of the Tracer v1.7.2 utility of SP BEAST2. The principal files from the Tracer utility for the simulation can be found in the Supplementary Materials in a special folder, with information on the tree height mean (ESS = 852), the Tracer tree likelihood mean (ESS = 211), and the constant population size change (mean = 34.3 ± 0.01, ESS = 2686), which includes .pdf vs. .txt files and the BEAUti .xml file, placed in the folder: Tracer_out_for_Fig7. The sequence partitions of the simulation run that was used for the Figure 7 reconstruction and the main properties of the simulation schedule are as follows and can be retrieved for inspection by running BEAUti: (i) the sequence partitions of 26 specimens for all 12 PCGs comprised the main data set, with the position data matrix and the matrix with the single sequences set; details were implemented in the .xml base file (Mytilidae27sq-no_pat-pt123ps.xml; for sequence partitions, see the menu folder); (ii) the model priors of the coalescent constant population was presented in the same file (Priors); (iii) the tip dates were set as the score in years of the calibration dates, with 292.8 Mya to MRCA for two *Modiolus* representatives, *Modiolus_modiolus*KX82178 and *Modiolus_kurilensis*KY242717 (age 292.8 × 10^6^); (iv) a gamma site model was implemented for the calculations, with GTR + G + I (site model: substitution rate = 2.93 × 10^−5^, G category count = 15, I = 0.126, shape = 1); (v) the clock model was implemented with the following options (strict clock: Clock.rate = 2.93 × 10^−5^); (vi) mcmc setting was performed (MCMC: chain length = 50,000,000). Independent testing supports were prior in item (ii), indicating that the model of the coalescent constant population was appropriate (Supplementary Materials folder: Tracer_out_for_Fig7). The simulated data agreed well with the expectation curve of constant population growth, as demonstrated by the distribution of the set of 27 mitogenome sequences of Mytilidae (mean = 34.3 ± 0.01, ESS = 2686). As noted above, the five tree-building methods (NJ, MP, ML, BA, and CA) basically produced the same topologies for the 26–27 mussel sets (Figure 4, Figure 5, Figure 6 and Figure 7). Data on the branch lengths in Figure 7 are concordant with data on the node probabilities and bootstrap supports given in Figure 4, Figure 5 and Figure 6. Node ages for the sequences of the same species do not differ, judging by the sampling or standard errors (SEs); however, ages for inter-species (39.8–3.0 Mya), inter-genera (113.2–37.3 Mya), and inter-subfamily (family) (292.8–95.8 Mya) levels show an increased dispersion (Figure 7). More details on the lineage divergence are provided in the Discussion Section, along with the representation of data on genetic distance comparisons across taxa ranks. Finally, it is necessary to emphasize the basic concordance of the five molecular genetic reconstructions with simulated lineage diversification in time.

## 3. Discussion

### 3.1. Structure and Variability of the Mitochondrial Genome of Arcuatula senhousia, Mytilus coruscus, and Other Representatives of the Mytilidae Family

The structure of the mitogenome described in the current research (Figure 1, Figures S1 and S2) is basically similar to other Eukarya and to the bivalve mollusks in particular. The mitogenome has a CR with a replication origin, 12–13 PCGs, 2 rRNA genes, and 22 tRNA genes [23,25,42,43]. The distribution of nucleotide diversity per site (Pi) along the whole length of 12 PCGs of mitogenome in mussels for all Mytilidae is shown in Figure 2, together with several variability estimators of genes by DNAsp-6. The numerical scores exemplified above are also given in the Supplementary Materials (Table S5). The statistical analysis revealed that Pi and Hd did not differ significantly between the 12 PCG of 26 sequences, averaging at about 38% and 100%: Pi = 0.377 ± 0.004, Hd = 0.997 ± 0.012. Overall, our analyses show that the structure of the mitogenome of the studied members of Mytilidae is not very conserved and it holds significant information capacity to give a relevant phylogenetic signal. Furthermore, despite high variability, the nucleotide composition does not appear to be saturated according to the Iss and Iss.c. given in the Results. Among 26–27 studied mitogenomes, the most conserved were the mitogenomes of the genus *Mytilus* and *Crenomytilus grayanus*, for which 10 compared sequences have the same gene orders (Figures S1 and S2 in the Supplementary Materials), as was the case in another study [25], excluding the deletion of Q-block of tRNA coding for the Glu amino acid and the presence-absence of the *atp8* gene. Contrary to the norm for vertebrates, not a single *nad6* was located at the “−” strand. The other members of the Mytilidae family have more variable content and a more varied arrangement of the mitogenome, including changing gene locations and the frequent loss of the *atp8* gene (Figures S1 and S2). Increasing the number of investigations indicates that the absence of *atp8* could be caused by annotation difficulties, due to the highly divergent and variable length of this gene [43,44]. In the study [44], the complete mitogenomes of three marine mussels (*Xenostrobus securis*, *Bathymodiolus puteoserpentis*, and *Gigantidas vrijenhoeki*) were newly assembled with estimated lengths of 14,972 bp, 20,482, and 17,786 bp, respectively. The gene *atp8* was annotated in all three mitogenomes, although previous assemblies missed out *atp8.* All newly annotated *atp8* sequences have one predicted transmembrane domain and a similar hydropathy profile, as well as the C-terminal region with positively charged amino acids.

Ending this section, we conclude that for the studied mussels, the changing gene order and variable size of the mitogenome is a norm, unlike in vertebrates, where the gene order is constant and the average genome length is stable at 16,500–17,000 bp.

### 3.2. Analysis of the Properties of Sequences

The obtained variation among the 26 sequences set allows us to pass judgment on their sufficient information signal, as provided in the Results and proved with the statistical tests (Table S9 in the Supplementary Materials).

Our analyses showed the significant heterogeneity of four types of nucleotides in the set of 26 mussel mitogenomes and the bias of purines over pyrimidines, according to Wilk’s test. This fact is widely known regarding PCGs, due to the hydrophobic impact on protein polypeptides, but in the current paper, this was supported by the statistical evaluation (Figure 3, Table 2 and Table S6). Statistical validation is often ignored in molecular science research [36]. Therefore, our result strengthens our knowledge of the very important properties of protein polypeptides, such as their hydrophobicity and its determination by the purine and pyrimidine nucleotide ratio. This finding might indirectly indicate the occurrence of purifying natural selection for supporting the polypeptides’ optimal functionality.

Data on the mitogenome structure and function, in combination with the signal on the topology of threes and including different components of the mitogenome (see Figure 4, Figure 5 and Figure 6), allow us to treat 26–27 PCGs sets as relevant for mussel genome representation and for the phylogenetic reconstructions reported in this paper, in comparison with other relevant research [23,25,43,44]. Let us now move on to an analysis of the sequence properties. The total estimates of synonymous (s) and non-synonymous (a) substitutions in the codons were: Pi(s) = 0.637, while Pi(a) = 0.288; thus, Pi(a)/Pi(s) = 0.452, while Pi(s)/Pi(a) = 2.79. The difference in the substitutions is quite impressive, but no significance is given by DNAsp. The assessment of the degree of this difference could be calculated somewhat differently for Ks and Ka, or as pairwise values between all sequence variants [36]. The evaluation for pairwise estimates of the degree of difference between these variables showed that the variation rows of two variables do not overlap. As has been reported in the Results Section for mussels, twofold tests were made with the 26-sequence set of PGGs: (i) in the entire variability continuum of pairwise Ks/Ka scores, and (ii) for single-genus comparisons, within *Mytilus*. The ANOVA results for set (i) showed highly significant statistically greater Ks in the 26-mitogenome set: Ks = 1.618 ± 0.019, Ka = 0.384 ± 0.019 (*n* = 325). For set (ii), Ks = 1.069 ± 0.071, Ka = 0.052 ± 0.072 (*n* = 73); F = 100.9, d.f. = 1; 71, *p* < 0.00001.

Let us consider a third sort of estimate of the selective value of molecular markers, based on the Fu and Tajima tests presented in Section 3.2, using data from within *Mytilus,* where the results showed Ks/Ka, where *p* > 0.10. If we ignore the genetic heterogeneity for all sites for the 26-sequence set and use only the segregating sites, we find that Fu and Li’s F* test statistics also detected no significant deviation from neutral expectation, where *p* > 0.10. Thus, all the above results for Fu and Li’s statistics are consistent with the neutrality of sequences in our data set. The Tajima statistics gave a similar output when testing neutrality, as illustrated in the Results Section.

In accordance with the widely accepted [36] and logical concept of natural cutoff selection acting upon organisms living in nature, its action against nucleotide substitutions in certain codons (deleterious mutations) may lead to a less active (ineffective) macromolecule in the polypeptide. The typical interpretation is that a high Ks/Ka value (i.e., >1) indicates the action of negative selection. This selection removes mutations in non-synonymous sites because such mutations cause amino acid substitutions. Since the function of mitochondrial genes has been perfected over hundreds of millions of years, the great majority of non-synonymous substitutions are deleterious, and, as a result, are removed by selection. We were unable to source unambiguous evidence in favor of this concept in the current paper. In other words, data on the significance of Ks/Ka might not be evaluated with scientific precision as direct evidence for a normalizing selection process acting against mutations with a phenotypic effect (Ka) in mtDNA sequences, for many reasons. This effect in the current paper was derived from the relatively homogeneous material of the PCG sequences of mussels of a single family and genus. In our testing for the neutrality of molecular polymorphism, we have violated the initial hypothesis with a single species and an infinite population to see the extent of a spurious outcome in an investigation. As we observed, the neutrality tests are relatively robust, providing no detection of spurious selection. Thus, one test of our data supported the hypothesis of the presence of purifying selection for codon-based variation in PCGs, while others did not. A scientifically valid proof of the direct action of natural selection in the molecular markers appears to be a complicated task to achieve, as was noted half a century ago for isozyme polymorphism [35,45,46]. To the author’s best knowledge, the most thorough attempt to detect the impact of molecular variability on the fitness (W) components (W = W_v_ + W_f_, where W_v_ and W_f_ are fitness in terms of viability and fertility, respectively) made in research on allozyme polymorphism in *Drosophila* by Kojima and coauthors [45], where no definite allele effects in the numerous generations were obtained after nearly ten years of laborious laboratory experimentation. The conclusions in favor of selection occurring quite frequently are available in the literature sources on molecular markers [47,48,49], but little attention is paid to the complications, as stressed above.

### 3.3. Reconstruction of Gene Trees and an Analysis of the Molecular Phylogenetic Relationships

Genetic differentiation between the F/M lines, as estimated by DNAsp-6, is presented in the Results Section. In summary, the interline gene flow parameter for three different estimates was averaged to Nm = 0.45. This is a very small amount of gene flow for intraspecies groups, and it is close to the interspecies scores for genetic differentiation [14,50,51]. Thus, there is a high level of differences between the F/M-lines comparable with interspecies divergence in the sequences of mussels, which demands the separate usage of F/M lines in evolutionary comparisons. This is especially important in the reconstruction of phylogeny, gene tree building, and systematics, where erroneous conclusions may be possible.

**Figure 8:**
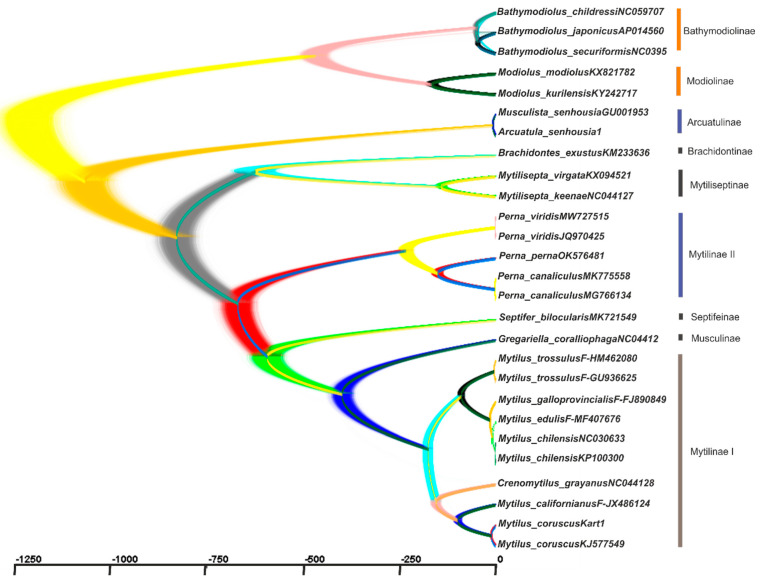
Phylogenetic lineages reconstructed via BEAST-2 and visualized with the DensyTree v.2.2.7 software, based on 27 sequences of 12 PCGs of the Mytilidae family. Simulated lineages are naturally rooted in five presumed ancestral taxa, including the predefined outgroup taxon *Modiolus*. Wider lines depict the consensus trees constructed by computer simulations of the coalescent process of molecular evolution in constant populations during 5 × 10^7^ generations by BEAST-2. Thin lines show all possible trees that occurred during the time span, as depicted in the bottom scale given in the internal calibration provided by DensyTree. DensyTree reconstructed the time-tree interrelationships based on the same BEAST run and the same output tree file as that used for building Figure 7. The source tree file is available for use in Table S2 of the Supplementary Materials (File: Mytilidae27sq-no_pat-pt123ps.xml). The main branches are stained with different colors that depict some differences in the branching patterns.

Data on the mitogenome structure and function, in combination with the signal on the topology of trees [14], allow us to use the set of 12 PCGs as the basis for phylogenetic reconstructions (Figure 4, Figure 5, Figure 6 and Figure 7). We showed, by using up to five different methods of phylogeny reconstruction, that topology is clearly resolved for most branches except the branch joining *Septifer bilocularis–Crenomytilus + Mytilus* in the MP tree, for which the support score was negligible (0.46), and for the branch of *Arcuatula–Perna*, where the support scores were low for some methods (Figure 4, Figure 5, Figure 6 and Figure 7). Reconstruction of the time of divergence of phyletic lineages based on the sequences of 12 PCGs for 27 mussel representatives reveals good matches of gene tree topology with the other four reconstructions given in Figure 4 and Figure 6, although with certain discrepancies in branching pattern, as explained above. It yields a date of close to 293 Mya for divergence between the most basal lineages, *Modiolus modiolus–M. kurilensis*, together with *Bathymodiolus* representatives on the one side and the remaining members of Mytilidae and Mytilinae on the other side (Figure 7). The common root of these late two taxa clusters was dated at 292.8 Mya and was calibrated to the time of divergence of the common ancestor of Mytilidae-Modiolinae (Modiolidae), as noted in the Materials and Methods Section. Another and more visual representation of the variety of the probability of phyletic lineages during the molecular evolution of mussels through time in our data set is exemplified in Figure 8.

The pattern and times of the molecular divergence shown in Figure 7 and Figure 8 are close to those obtained in other papers on mytilids using the mitogenome data [23,25]. However, there are some differences between the inferred trees in our study and those in the cited papers. The major congruence is seen for Mytilinae, which was found to be paraphyletic, as reported in [10,25], and this is definitely established in the current paper (Figure 4, Figure 5, Figure 6, Figure 7 and Figure 8 and Figure S3). In our paper, Mytilinae I includes *Mytilus + Crenomytilus* and should certainly be combined with some other taxa that have been assumed to be present since Soot-Ryen’s work was published [27] and according to current viewpoints [28] (WoRMS 2024; https://www.marinespecies.org/aphia.php?p=taxdetails&id=211; accessed on 8 April 2024), while Mytilinae II includes the representatives of the genus *Perna*, which, topologically (Figure 4, Figure 5, Figure 6, Figure 7 and Figure 8 and Figure S3) and historically (Figure 7 and Figure 8), is definitely an independent lineage that is currently recognized as a different subfamily [28] (Warms 2024; https://www.marinespecies.org/aphia.php?p=taxdetails&id=211; accessed on 8 April 2024) but may have a different status in the future. However, one major difference is also seen in the Mytilinae. For instance, for Mytilinae I, in our study, the two large Pacific mitilids (*M. californianus*, *M. coruscus*) form an internal monophyletic branch within the genus *Mytilus,* with *Crenomytilus grayanus* being more distant from them, while in Figure 1 and Figure 2 [25], *C. grayanus* joins *Mytilus coruscus*, with *M. californianus* being more distant from them. The trees that were reported in [23] on Pteriomorphia and Heterodonta were reconstructed based on the concatenated sequences of 14 shared genes. In this paper, *Bathymodiolus* formed a monophyletic clade with asymbiotic Mytilidae mussels, the vesicomyid clams formed a monophyly sister to the Veneridae, and another taxon branched basally in the Heterodonta. These data do not add much information to the systematics of the mytilids.

Two other studies investigated the molecular phylogeny of Mytilidae; these included different subfamilies using nuclear markers (*18S* rRNA, *28S* rRNA, and histone *H3* [3,24] and mitochondrial genes (*cox1* and *16S* rDNA) [24]. In the paper by Kartavtsev and colleagues [3], the data obtained supported the ranking of Modiolinae G. Termier & H. Termier, 1950 and Bathymodiolinae Kenk & Wilson, 1985 as subfamilies in the Mytilidae family, along with the rank of family for Septiferidae Scarlato et Starobogatov, 1979. The latter taxon is not universally recognized, and this remains to be clarified in future studies. However, our current observations of *Septifer bilocularis* as one of the basal branches of Mytilinae are consistent with such a view (e.g., Figure 4). In [3], Mytilinae was recognized as monophyletic, but the concatenated matrix of four genes used for the tree, which supported such a conclusion, showed no *Perna* (Figure 5, [3]). Liu and coauthors [24] found two major clades: the Modiolinae-Bathymodiolinae node and the Mytilinae branch, which included the *Septifer*, *Musculus*, and *Brachidontes* species, while Lithophaginae was found to be polyphyletic. A close connection with Mytilinae of such taxa as *Septifer*, *Musculus*, and *Brachidontes* is supported by our research (see Figure 4, Figure 5, Figure 6, Figure 7 and Figure 8), but the status of Lithophaginae is yet to be clarified in future investigations. Another paper on several bivalve taxa, with an emphasis on deep-sea bivalves, placed *Bathymodiolus* as a sister taxon to shallow-water mytilid species, based on a mitochondrial gene, *18S* rRNA, and on morphology [21]. Some of the branching patterns of the trees that are based on single genes may support the current results (Figure 1, [52]), but when they are contradictory, it is hardly possible to rule out a weak phylogenetic signal as the source of such a controversy.

Obtained discrepancies are to be expected, simply due to differences in the number and combination of the taxa used in the trees, i.e., due to biased taxa representation. Among other possible sources for curious branching may be the differences between F/M-lines in mitogenome genes, as was stressed at the beginning of this section, and insufficient phylogenetic signals from data matrices for resolving the topology of trees, as presented at the end of Section 3.3 (Table S9). Also, as demonstrated in the results (e.g., Figure 4, Figure 5 and Figure 6), the methods of tree building, the partitioning of sequences, and finding the substitution model are key to obtaining an optimal consensus tree. These complications are convincingly described in the book by Nei and Kumar [53], wherein they noted that complicated substitution models (e.g., those produced by SP PhyloSuit) have drawbacks and they do not always yield a tree with better topology. There are several review papers and even textbooks on this subject, where plenty of advice is shared [54,55,56,57]. This is the case when we see better support for the Mytilinae branch in the simple NJ and MP trees, but not in ML and BI trees constructed with sophisticated GTR + I + G substitution models and partitions for PCGs, in the case of BI (e.g., Figure 4). Another factor that definitely impacts the branching pattern is the properties of the set of markers that are used for reconstruction of the trees. As was shown in the Results, a tree built with more conservative rRNA and tRNA genes has *Crenomytilus* as a basal branch to *Mytilus,* whereas when using only PCGs (see Figure 4 and Figure 5) or PCGs in combination with rRNA-tRNAs (Figure S3), the tree provided a different clustering pattern, where *C. grayanus* is placed within the *Mytilus* branch as a sister-taxon to two large Pacific species, as exemplified above (see Figure 6 and Figure S3). These modern molecular genetic data are supported by an earlier finding on biochemical-genetic observations for the systematics of Mytilidae, at which *Crenomytilus grayanus* was placed as the external branch to the representatives of *Mytilus* (Figure 3, [58]) (this tree uses Dn Nei’s [35] metrics, which are able to quantify molecular evolution, while others in the paper could not). The question concerning a separate place for large Pacific *Mytilus* as *Pacifimytilus* (p. 44, [59]) arose long ago in systematics, and, for *Crenomytilus,* even the tribe status was suggested based on its morphology and zoogeography merits (p. 45, [60]) (cited after Lutaenko [61]). Based on the data that are given below on the genetic distances ranked in the different taxa, and on the gene flow prevalence in the direction *M. trossulus → M. edulis → M. galloprovincialis*, the species rank of *M. trossulus* that was determined in earlier papers for members of *Mytilus* ex. group *edulis* [2,4,62] must be accepted unequivocally. However, the taxa of *M. edulis*, *M. galloprovincialis*, and *M. chilenis* should only be considered as subspecies/semispecies, if the definitions are based on the orthodox BSC [1,14,63]. Such a conclusion is clearly concordant with the age of the taxa, wherein *M. trossulus* is the most ancient member of the *Mytilus* ex. group *edulis* [1,34] (see Figure 7 and Figure 8; see details in [1]). More recent papers on this yang node of mussels have been investigated in a wide area of the World Ocean from the scope of genetic exchange and from the viewpoints of genetic variability and taxa status [1,2,3,6,7,64,65,66].

Obviously, the above information on the molecular evolution of mussels and the supporting zoological data suggest the necessity for revision of the systematics in the Mytilidae family.

It seems appropriate to conclude Section 3.3 by considering genetic divergence by means of the genetic distances between mussels of different taxa ranks. For this purpose, *p*-distances [53] were calculated via SP MEGA-11 by two approaches: (i) estimating the mean scores of several defined sequence arrangements for the three groups: (1) intraspecies comparisons, (2) interspecies comparisons within a single genus, and (3) interspecies comparisons between the different genera of the same family, and (ii) by estimating all the pairwise *p*-distance values for the set of 26 mitogenome sequences (Table S10, afterward making a gradation into groups. The MS Excel spreadsheet included two folders with data matrices, one with averages for the three comparison groups and another for all the pairwise comparisons of the corresponding groupings for intraspecies variation and for the above-defined rankings of taxa variation). When inspecting phylograms that contain a scale representing the length of branches, it is provisionally possible to imagine at a qualitative level that there is an increase in the branch lengths (= genetic distance) in accordance with the level of the taxa hierarchy. It is possible to evaluate the direct interrelationships between these two variables at a quantitative level by using a suitable statistical tool, e.g., ANOVA/MANOVA or correlation analysis. For approach (i), the obtained mean *p*-distance values are as follows: (1) 2.40 ± 0.11%; (2) 15.39 ± 0.27%; (3) 43.89 ± 0.40% (Table S10, folder “Mean + SE”).

**Figure 9:**
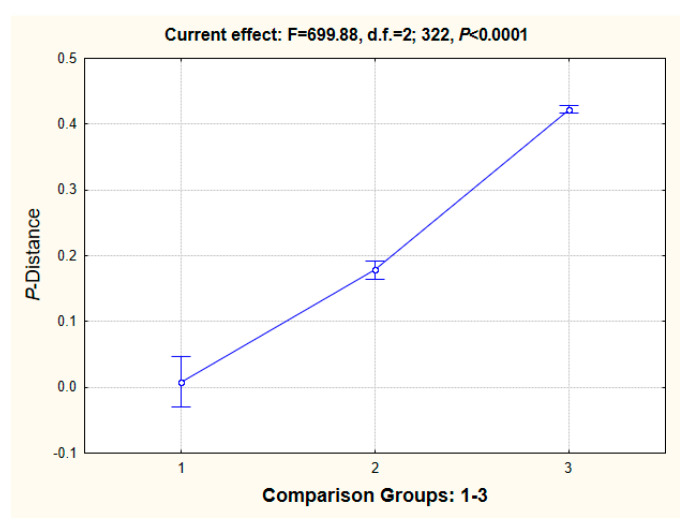
Univariate ANOVA showing the variability of the mean values of the genetic distances *(y*-axis) for the comparison groups *(x*-axis) of the sequences in the sampled taxa for 12 PCGs of 26 mussel mitogenomes in Mytilidae: (1) *p*-distances within the species or between individuals of the same species; (2) interspecies distances within a single genus; (3) between species of different genera in the same family.

The results for all pairwise comparisons of the *p*-distances of 12 PCGS among 26 mitogenomes, i.e., for approach (ii), are: (1) 0.81 ± 1.94%; (2) 17.83 ± 0.71%; (3) 42.24 ± 0.29%. The ANOVA’s current effect is: F = 699.88, d.f. = 2; 322, *p* < 0.0001 (Figure 9). Because of the deviation from normal distribution, nonparametric testing for this approach was performed with the results for the (i) Kruskal–Wallis (K–W) and median (ii) tests: (i) H = 126.35, d.f. = 2, *n* = 325, *p* < 0.001; (ii) *X^2^*= 59.39, d.f. = 2, *p* < 0.0001.

Looking at a comprehensive review of over 20,000 invertebrate and vertebrate specimens, the values of genetic distance estimated at different taxon levels could explain this matter [67,68]. The distance data revealed increasing levels of genetic divergence in the sequences of two genes, cytochrome *b* (*Cyt-b*) and cytochrome *c* oxidase (*Co-1*), in the five groups compared: (i) populations within a species; (ii) subspecies, semi-species, or/and sibling species; (iii) species within a genus; (iv) species from different genera within a family; and (v) species from separate families within an order. The mean unweighted *p*-distances (%) for these five groups for *Cyt-b* were as follows: (i) 1.38 ± 0.30; (ii) 5.10 ± 0.91; (iii) 10.31 ± 0.93; (iv) 17.86 ± 1.36; and (v) 26.36 ± 3.88, respectively; while for *Co-1*, the values were the following: (i) 0.89 ± 0.16; (ii) 3.78 ± 1.18; (iii) 11.06 ± 0.53; (iv) 16.60 ± 0.69; and (v) 20.57 ± 0.57 [67,68]. For the Pleuronectidae flounders, the TrN-distances for *16S* rRNA are: (1) 0.76 ± 0.87%, (2) 3.34 ± 0.85%, and (3) 14.24 ± 0.23% (Figure 7A, [69]); for the representatives of eight different groups of animals, the *p*-distance values were in three corresponding comparison groups: (1) 0.79 ± 0.04%, (2) 8.23 ± 0.22%, (3) 16.47 ± 0.29% [70] (arthropods, chordates, echinoderms, flatworms, mollusks, nematodes, segmented worms, and sponges were included). The values obtained for PCGs of four comparison groups of studied members of the suborder Pleuronectoidei are as follows: (1) 0.54 ± 0.78%, (2) 14.99 ± 0.48%, (3) 16.51 ± 0.22%, (4) 33.57 ± 0.07% (Figure 7B, [69]) (comparison groups represent four different hierarchies of the taxa, excluding semispecies/subspecies).

Evidently, the comparable levels of the genetic distances for the single gene markers and for the mitogenomes tell us that there are nearly two times larger *p*-distances in the (i) intraspecies comparisons, for the two taxa levels, i.e., for the species within a genus (ii), and for the species from different genera within a family (iii)–(iv) for the mussel estimates and for the bulk of other taxa. These genetic distance data agree well with the large ages obtained for the divergence of the mussel taxa in this and in another paper [25] and may explain the increased accumulation of nucleotide substitutions among the lineages in the Mytilidae family throughout their phyletic evolution.

## 4. Materials and Methods

### 4.1. Materials and General Approaches

A total of up to 100 mitogenome sequences belonging to the order Mytilida were analyzed, including 4 obtained by the authors of this paper (see Results) and those retrieved from GenBank [71] (GenBank; https://www.ncbi.nlm.nih.gov/; accessed on 15 March 2023). Additionally, 39 sequences were used for the comparisons, with more precision given in Table S1 (Supplementary Materials). Because of the discordance of the mitogenome in invertebrates, including mussels, the actual number of the sequences included in the data matrices for direct analysis is much lower, and the main data set was established after appropriate alignment as 26–27 mitogenome sequences (treated hereafter as specimens or vectors of scores) of all initial species numbers, as indicated below in the tables and figures. The main data set used in the paper is given in the Supplementary Materials (Table S1). This data set presents the sequences from GenBank of 39 mussel and other bivalve species and the Latin names for the species that are given, in accordance with [33] and [72] (WoRMS, 2024; https://www.marinespecies.org/aphia.php?p=taxdetails&id=211; accessed on 2 April 2024), and includes the four new specimens reported in this study. Supplementary Table S2 includes the example set of 26 PCG sequences of mussels that are presented in the file “myt26sq.nex” and that are used in tree reconstructions by the software packages (SP), SP MEGA, and SP MrBayes. Table S3 comprises data on 27 sequences and was used for major tree reconstructions in SP BEAST2 (Table S3 contains the file “Mytilidae27sq-no_pat-pt123ps.xml”). Table S4 includes the example set of 37 mussel sequences from GenBank, prepared as the file “mytfm-pt.nex”, which was used for the preliminary analyses of the sequences in this paper. (The file mytfm-pt.nex could be used for training and obtaining erroneous evidence on some parts of a tree topology in the case when the initial super matrix is not yet completely and thoroughly assembled). For convenience, all the files are given separately and are placed in the SUPPLEMENT2v folder. There are also MS Word documents with the file name “Figure S1, Figure S2. SUPPLEMENT.docx”, which includes two listed maps of gene order for the 26–27 sequences. Also, a separate documentary file is included in the above file that is named “Gene order for 26 sequnces-info.csv”, which hold csv-based information (MS Excel-compatible) for 26 sequences, and which is provided for those who may want to make an independent investigation of these data.

Two female specimens of *Arcuatula senhousia* (W.H. Benson, 1842) and two female specimens of *Mytilus coruscus* A. Gould, 1861 (current: *Mytilus unguiculatus* Valenciennes, 1858; WoRMS, 2024, https://www.marinespecies.org/aphia.php?p=taxdetails&id=506159; accessed on 16 April 2024) [73] with the adductor tissue were fixed in 95% ethanol; 20–50 mg pieces, each were derived from the collection of the Laboratory of Molecular Systematics at NSCMB FEB RAS (see the affiliation below). The voucher specimens of *Arcuatula senhousia* (laboratory names Ar1 and Ar2, GenBank OR453538 and OR453539, respectively) and *Mytilus coruscus* (laboratory names Kart1 and Kart3, GenBank OR453540 and OR453541, respectively) were collected by scuba-divers in Amursky Bay and Vostok Bay, Peter the Great Bay, and the Sea of Japan in 2021 and 2022, respectively; both are stored at the Museum of the A.V. Zhirmunsky National Scientific Center of Marine Biology of the Far East Center of the Russian Academy of Sciences (NSCMB FEB RAS). DNA was isolated using a commercial kit (DNA Extran-2, Sintol, Moscow, Russia). Then, DNA specimens of *A. senhousia*: Ar. 1, 288 ng/µL, Ar. 2, 392 ng/µland *M. coruscus*: Kart1, 110 ng/µL, Kart3, for which 344 ng/µl of total DNA were sampled and sent on to the Genoanalytica Lab (Moscow, Russia, https://www.genoanalytica.ru/; accessed on 29 January 2024) for sequencing. Sequencing was carried out on the Illumina platform (Novaseq 6000 sequencer, Moscow, Russia).

According to the sequencing technique, the length of the read of the nucleotide fragments along the mitogenome was 150 bp. The fragments were assembled into a complete mitogenome sequence using SP SOAPdenovo2 [74] and SPAdes 3.13.1 [75]. The protein-coding genes, rRNAs, and tRNAs were annotated and mapped using the MITOS WEB bench and several types of software thereon [76] (MITOS web server (uni-leipzig.de); accessed on 17 March 2023).

An analysis of variability and divergence was carried out, starting with such software packages as SP DNAsp-6, Ver. 6.12.03 (or the DNAsp-5 utility release for Fu tests) [77] and SP MEGA-X [78] or SP MEGA-11 [79]. Molecular phylogenetic analysis was performed mainly on the basis of nucleotide sequences (referred to merely as sequences in this paper) of PCGs, using the SP MEGA-X or MEGA-11 and SP MrBayes 3.2.1 or 3.2.7 (http://nbisweden.github.io/MrBayes/download.html; accessed on 16 February 2024) [80,81], and SP BEAST-2 [82,83,84,85,86] (including the later updates at: http://www.beast2.org/; accessed on 20 December 2023). Protein-coding genes were extracted from complete mitochondrial genomes with the MYTOS resource (http://mitos.bioinf.uni-leipzig.de/index.py; accessed on 21 January 2024) or directly from GenBank and were used for further analysis with SP PhyloSuit (http://mitos.bioinf.uni-leipzig.de/index.py; accessed on 21 January 2024) [87], then the sequences were combined into a super-matrix using SequenceMatrix 8.1 SP [88]. In the preliminary steps of the analysis, complete mitogenome sequences were aligned using the ClustalW program in MEGA-11. The gap opening and gap extension penalties were set at 15.0 and 5.0, respectively (for other settings of the alignment program, the default parameters were used). After the first alignment step, any large gaps were manually removed; the final alignment in the second step was performed with reduced penalty levels (5.0 and 0.5 for the two options, respectively). All gaps were then manually removed again.

### 4.2. Analysis of the Sequence Properties

For the analysis of mitogenomes, SP MEGA-11 and SP PhyloSuite were used, with various functional features that are useful for other applications. To complete jobs in SP PhyloSuite, the sequences of mitogenomes used for basic analyses were downloaded from GenBank in the ID.gb format (one of the main GenBank formats). Then, all information on the sequences of PCGs, rRNAs, tRNAs, and the control region (CR) was extracted from the mitogenomes. The resulting sequences were aligned in a program block (utility), MAFFT. Alignment was carried out in two stages. In the first stage, the PCGs were aligned, and in the second stage, rRNAs, tRNAs, and CR were aligned. Next, the resulting fasta files (.fas, .fasta) for protein-coding, rRNA, and tRNA sequences were moved to one folder and were then stitched into a single file using another program block, Concatenate Sequence.

In the next step, the concatenated sequences were analyzed using the software utility of SP PhyloSuit, PartitionFinder, to define the most appropriate mitogenome partition schemes and to choose the optimal models for molecular substitution along sequences. In the subsequent analysis, within this block, the model-fitting options for SP MrBayes were selected (the appropriate option is chosen from the menu window instead of the default option “all”), using the economical (“greedy”) search method. After finishing the PartitionFinder job, the necessary files were sent to SP MrBayes, integrated with PhyloSuite.

When running SP MrBayes in SP PhyloSuite, additional options such as the choice of an outgroup, the number of generations, and others that determine the probabilistic parameters of the tree reconstruction are determined twofold, either using software or manually. This is why, for the last case in the menu window, when starting SP MrBayes, we set the number of generations (*n*) to be equal to *n* = 10^5^ or *n* = 10^6^, and the number of Markov chains in the digital Monte Carlo simulation (mcmc) to be equal to 4. However, in the former case, the special models for each gene were selected by the PartitionFinder utility and were automatically recorded in the command block of MrBayes analysis.

### 4.3. Molecular Phylogenetic Analysis

This analysis is conducted mainly for building the gene trees or phylograms. The phylograms based on the sequences of PCGs were built using several techniques. Initially, the optimal substitution model for nucleotides in the lineages is estimated using the matrices of sequences, which have previously been prepared for the investigation. The best-suited model, as determined using MEGA-X or MEGA-11 software, was the GTR + G + I (general time reversible model, with G representing gamma mode variations across sites and I representing the invariable fraction of nucleotides). The GTR + G + I model was defined as the best for most of the sets of sequences that were chosen for this analysis within the Mytilinae and for the Mytilidae family. Phylogenetic trees were constructed using four methods: neighbor-joining (NJ), maximum parsimony (MP), maximum likelihood (ML), and Bayesian analysis or Bayesian inference (BA or BI). These techniques were performed with an original software package, using MrBayes-3.2.7 for BA, or SP MEGA-X/MEGA-11 for NJ, MP, and ML techniques for the set of 12 PCGs and in 2–3 different sets of sequences. As noted, the additional gene tree reconstructions were performed with SP PhyloSuite and SP BEAST-2.

For the BA analysis, SP MrBayes-3.2.7 was applied as stated above. Before running BA, the SP SequenceMatrix-8.1 [88] was run and the special super-matrix for the BA analysis was formed as its output file, creating the 26-sequence matrix (Myt26sq.nex; given in the Supplementary Materials as Table S2). Then, SP MrBayes-3.2.7 was run to perform a numerical simulation for tree reconstruction. Program settings for BA runs comprised applying either one million generations (*n* = 10^6^) or fewer (*n* = 10^5^) if appropriate, four parallel Markov chains in the ‘mcmc’ program utility, the definitions of partitions for 12 PCGs, descriptors for the coding of nucleotide positions within codons that were previously defined by SequenceMatrix, and a few other options available in SP MrBayes-3.2. The output tree file represents the BA consensus tree, with credibility values for the branches. Three other mentioned techniques of tree building, ML, NJ, and MP, were run with k = 1000 bootstrap replications to provide bootstrap support for topology via the scores derived for the branch nodes in the trees. As an outgroup for the tree rooting for the Mytilidae family, the two taxa, *Bathymodiolus* and *Modiolus,* or solely *Modiolus* were used. For the *Mytilus* genus, *Crenomytilus grayanus* was used as the outgroup in some comparisons. More details on the tree construction and their comparison are given in the Results and Discussion Sections.

The dating of divergence times was made with calibration points based on the paleontological records. The most recent common ancestors (MRCA) comprise the reference points for the calibration of molecular divergence. The calibration points for the molecular divergence are 292.8 million years ago (Mya) for Modiolinae (Table 1, [89]) and 244.6 Mya for *Mytilus* [90] (*Mytilus nativus*, dated 247.2–242 Mya. The definition of the second calibration point in the International Chronostratigraphic Timescale is taken from https://paleobiodb.org/classic/displayTimescale?interval=139; accessed on 19 April 2024). Correspondingly, the first calibration point was applied to Mytilidae from the above taxa in the data matrix. Below, in the third paragraph after this one, more details can be obtained on this point.

As stated above, draft phylogenetic reconstructions for the Mytilidae family were performed largely by SPs MEGA-X/MEGA-11 and MrBayes, involving 12 PCGs and 4 tree reconstruction techniques: NJ, MP, ML, and BA. The topology inference and the molecular divergence in time were obtained using coalescent analysis (CA) by SP BEAST-2, in addition to those 4 for all 12 PCGs and, mostly, for the 27-sequence set in the family, including the outgroup. The CA parameters from three fundamental models were integrated for this purpose: (i) Yule CA (Yule, 1924), (ii) Bayesian Skyline CA, and (iii) CA for a population of constant size [85,86]. For each of the three CA models, additional files that were designed in the BEAUti2.6.6. utility were run, as exemplified below. In one of the SP BEAST-2 simulations, the running file contained partitions and nucleotide positions that had been created with the PhyloSuite software, particularly the PartitionFinder utility.

SP BEAST-2, with the updates v2.6.5–2.6.7 [85,86], was most successfully run on a matrix of 27 sequences of 12 PCGs; it was used principally for the evaluation of node ages in simulated trees but also represents an additional method of tree topology reconstruction. An independent GTR + G + I model of nucleotide substitution with gamma-distributed rate variation across sites was explored (defined previously in MEGA, as described above), with *n* = 5–15 categories and many different options for model settings. The main menu of the BEATY utility of SP BEAST-2 (standard software mode, in this case), where the simulation model parameters are formed, includes 6 main windows: Partitions, Tip Dates, Site Model, Clock Models, Priors, and MCMC. Partitions: This window opens the working data of the sequence matrix, with a specification of its structure. Tip Dates: Here, ages for the tips are inserted, if appropriate. Site Model: i, the gamma site model, and a set of 5 other basic parameters, like: ii, Substitution Rate, iii, Gamma Category Count + Shape, iv, Proportion Invariant, v, Model of substitutions (this was mostly GTR in this case, but in some runs, the simpler Jukes–Cantor model was used). Clock Model: With 2 main settings, i, the uncorrelated relaxed exponential clock or the strict clock (both options were used in different runs) and ii, the Substitution Rate. The prior menu included: i, 1 of the 7 basic models (in our simulations, the basic model was coalescent constant population), ii, clock rates for all PCGs per cite, the mode of distribution (e.g., gamma, normal, etc.), frequency parameters, population size parameters, etc. The MCMC menu includes many critical options for successful runs: i, chain length (in most runs, this is equal to 50,000,000 or 5 × 10^7^ generations), ii, trace log, iii, screenlog, sitelog, treelog (per PCG partition and codon position), etc. [85,86,91]. These settings are differentially selected in the different runs, providing a comprehensive and massive computation job. For a clearer understanding of the process, one of the working files that was built in the BEATY utility is provided in the supplements (Table S3, File: Mytilidae27sq-no_pat-pt123ps.xml).

The reconstruction of the initial tree started with a random option, then the above-defined set of operators was activated to produce simulated gene trees for the set of data of 12 PCGs; e.g., as in the file: Mytilidae27sq-no_pat-pt123ps.xml. Priors that followed a Yule CA branching model, Bayesian Skyline, and CA for a constant size population were employed to test their suitability for the empirical data matrix. The most realistic results were obtained from the constant-size population mode. One or two points for the calibration of molecular clocks were used in this analysis, based on the fossil records. The first point was taken from nearly the oldest fossil finding, which was attributed to the Mytilidae [28,33] (WoRMS, 2024, https://www.marinespecies.org/aphia.php?p=taxdetails&id=211; accessed on 2 February 2024) and was dated at 427–426 Mya [33,92] (Mindat, 2024; https://www.mindat.org/taxon-3476.html; accessed on 19 April 2024). However, the more credible date for molecular calibration is based on fossils dating to the Mesozoic era, 228–201 Mya, which are assumed to be Modiolinae and *Modiolus* representatives [93] (Mindat, 2024; https://www.mindat.org/taxon-2285647.html; accessed on 2 February 2024) and 292.8 Mya [89]. As noted above, the later dating was used as a tip for the time of the most recent common ancestor, TMRCA, to date. Actually, the two calibration points for each of the *Modiolus* species-specimens in our super-matrix were modeled with a normal distribution, with a mean of 292.8 Mya and a standard deviation of 1.0 Mya, respectively, for the Mytilinae subsets. At least 52 different runs for the third basic model (iii, see above) were performed from 4 October 2023 to 20 December 2023, using 50 million generations, sampling every 1000th tree, and applying the variable settings for 6 main menu windows, as defined in BEAUTY. All runs were checked for sufficient mixing, showing stable convergence with unimodal posteriors and tree priors; effective sample sizes (ESS) were accepted with the scores above, to 100–200 (real ESS: 211, 852, and 2686) for three meaningful parameters, using TRACER v1.7 [82,86] (see Supplementary Materials, with the folder Tracer_out_for_Fig7). The TreeAnnotator utility of SP BEAST-2 was applied to the obtained simulated tree sets. By using this software, after the removal of 50% of the resulting trees as burn-in, the remaining trees were combined in a Maximum Clade Credibility consensus tree by running the SP TreeAnnotator v2.6.4 [86]. Together with the SP BEAST-2.6.5–2.6.7, BEAUTY-2.6.5–2.6.7 was involved in the construction of the main xml file for calculations in SP BEAST-2 (i.e., the BEAUTY file was generated in .xml format and is attached in Supplementary Table S3, as reported before). Also, the BEAGLE database (Beagle 5.2; washington.edu) was used in most of the runs, along with recommendations by the team of SP BEAST-2 [85,86].

The phylogenetic trees were visualized and edited, if appropriate, using the SP FigTree 1.4.0 [94] and MEGA-X/MEGA11 programs. As noted, besides the basic five techniques of gene tree reconstructions (BA, ML, NJ, MP-, and CA-trees), the SP IQ-TREE version 2.1.2 (http://www.iqtree.org; Wien, Austria; accessed on 2 February 2024) [95], was used for ML-tree reconstructions that were run with the default parameters, along with the auto-detection of the sequence type and the best-fitting substitution model. IQ-TREE performs on demand the ultrafast bootstrap [96] and the SH-aLRT branch test [97], and gives, as well as BI inference, the chance to estimate the scores for nodes’ support; in the current paper, runs were made where *n* = 2000–5000 replicates.

The four sequences of the mitogenomes obtained in this research for the two mussel species, *Arcuatula senhousia* and *Mytilus coruscus*, have been submitted to GenBank [98] and are represented in Figure 1. They are also given in the two broader comparisons of mitogenomes (Figures S1 and S2 in the Supplementary Materials), measured jointly with the sequences of GenBank that were sampled. For brevity, the structure of the mitogenome is visually represented for only one specimen of each of two species (Ar1 and Kart1, as implemented in the Results Section). However, all the sequences of both species were subjected to molecular and phylogenetic analysis and were included in the comparison of mitogenomic structure with other representatives of the Mytilidae family. The maps of the mitogenomes of the two above-mentioned mussel species were built using the MITOS WEB bench [76]. Also, suitable maps were obtained using SP PhyloSuit, and these are represented in Figures S1 and S2.

## 5. Conclusions

For the studied mussels, the variable gene order and highly variable size of the mitogenome is a norm, unlike in vertebrates, where gene order is a rare event and the average genome length is close to 16,500–17,000 bp. The results provided here will strengthen our knowledge of one very important property of the protein polypeptides, such as their hydrophobicity and its determination by the purine and pyrimidine nucleotide ratio. Thus, this fact might indirectly indicate purifying natural selection for the support of polypeptide functionality. In accordance with the widely accepted and logical concept of natural cutoff selection organisms, which explains its action against nucleotide substitutions in certain codons (deleterious mutations), leading to the less active (ineffective) macromolecule of the polypeptide; we were unable to obtain unambiguous evidence in favor of this concept in the current paper. 

There is a high level of divergence between F/M lines that is comparable with interspecies divergence in the sequences of mussels that require the separate usage of F/M lines for evolutionary comparison. This is especially important in the reconstructions of phylogeny, gene tree building, and systematics, where erroneous conclusions may possibly be drawn. The presented and discussed information above on the molecular evolution of mussels and the supportive zoological data suggest the necessity for a revision of systematics in the Mytilidae family. The comparable levels of genetic distances for single gene markers and for mitogenomes tell us that there is a sufficient difference of *p*-distances, both at the intraspecies level and at two taxa levels, when compared for mussel estimates and in the bulk of other taxa. These genetic distance data agree well with the great age of the divergence of mussel taxa and may explain the increased accumulation of nucleotide substitutions among the lineages in the Mytilidae family throughout phyletic evolution.

## Figures and Tables

**Table 1:** Mitochondrial genome information on the two mussel sequences presented in the current paper (*Arcuatula senhousia*, *Mytilus coruscus*); the third (*Mytilus californianus*) was retrieved from GenBank.

Genome Content/Sequences	*Arcuatula senhousia*, *Ar2* OR453539	*Mytilus coruscus*, *Kart1* OR453540	*Mytilus californianus* JX486124
Size (bp)	17,675	16,629	16,729
Gene number, PCGs	12	12	12
Gene number, rRNA	2	2	2
Gene number, tRNA	22	22	22
AT skew (PCGs, ratio)	0.5795	0.7109	0.7130
GC skew (PCGs, ratio)	1.7258	1.7179	1.7569
T + C:A + G ratio (PCGs, %)	54.02:45.97	50.19:49.81	50.24:49.76
CR (D-loop)	1653–2111 (+)	1–60 (+)	N/A
nad3	43–390 (ATG/TAA, +)	8314–8664 (ATG/TAA, +)	7314–7664 (ATG/TAA, +)
trnY	626–691 (+)	1115–1181 (+)	1–67 (+)
trnH	1235–1299 (+)	8181–8244 (+)	7181–7245 (+)
trnI	1316–1381 (+)	15,154–14,216 (+)	14,150–14,216 (+)
trnN	1393–1456 (+)	14,955–15,019 (+)	13,951–14,015 (+)
trnE	1566–1630 (+)	15,020–15,084 (+)	14,016–14,080 (+)
cox1	1870–3432 (ATT/TAA, +)	8950–10,605 (ATG/TAA, +)	7947–9602 (ATG/TAA, +)
cox2	3763–4470 (ATT/TAA, +)	2493–3221 (ATG/TAA, +)	1378–2109 (ATG/TAA, +)
atp6	4787–5461 (ATG/TAG, +)	10,621–11,331 (ATA/TAA, +)	9612–10,328 (ATG/TAA, +)
trnT	5488–5554 (+)	11,336–11,398 (+)	10,333–10,395 (+)
cytb	5597–6727 (ATT/TAA, +)	1183–2317 (ATG/T, +)	69–1376 (ATG/TAA, +)
trnD	6746–6810 (+)	15,322–15,383 (+)	14,316–14,380 (+)
trnR	6820–6885 (+)	7909–7971 (+)	6909–6973 (+)
trnS1	6887–6944 (+)	8113–8180 (+)	N/A
trnG	6966–7031 (+)	14,892–14,950 (+)	13,885–13,950 (+)
rrnS	7936–8825 (+)	14,885–15,817 (+)	13,884–14,829 (+)
trnS2	7938–7996 (+)	6826–6888 (+)	N/A
nad6	8038–8475 (ATA/TAA, +)	13,406–13,861 (ATA/TAA, +)	12,394–12,858 (ATG/TAG, +)
nad2	8457–7905 (ATT/TAA, +)	6958–7905 (ATG/TAA, +)	5960–6907 (ATG/TAA, +)
cox3	9553–10,407 (ATG/TAA, +)	6028–6810 (ATG/TAG, +)	4877–5812 (ATG/TAA, +)
trnK	10,421–10,487 (+)	3223–3291 (+)	2111–2179 (+)
trnF	10,492–10,559 (+)	13,877–13,944 (+)	12,872–12,939 (+)
trnP	10,575–10,637 (+)	8246–8310 (+)	7247–7310 (+)
trnL1	10,672–10,736 (+)	3366–3431 (+)	2254–2319 (+)
trnC	10,743–10,806 (+)	15,086–15,153 (+)	14,082–14,149 (+)
trnL2	10,854–10,917 (+)	3437–3502 (+)	2325–2390 (+)
nad1	11,011–11,946 (ATG/TAA, +)	3569–4492 (ATA/TAA, +)	2569–3492 (ATA/TAA, +)
trnM	12,080–12,142 (+)	3295–3362 (+)	2183–2250 (+)
trnV	12,182–12,243 (+)	4496–4562 (+)	3497–3562 (+)
nad4L	12,324–12,605 (ATT/TAA, +)	11,399–11,680 (ATG/TAA, +)	10,396–10,677 (ATG/TAA, +)
nad5	12,698–14,432 (ATT/T, +)	11,688–13,397 (ATT/TAA, +)	10,685–12,394 (ATT/TAA, +)
trnA	14,433–14,433 (+)	8046–8046 (+)	7046–7046 (+)

Notes. Numbers denote the start and end positions of a gene. The start/stop codons for PCGs are in brackets, while the “+“ or “−“ signs show the gene location on “+/−“-strands (no “−“ available for the examples in the table). Abbreviations of PCGs, rRNAs (rrn), and tRNAs (trn) coding for proteins, rRNAs, and tRNAs amino acids are as denoted in the Figure 1 caption. N/A means that data are not available.

**Table 2:** The nucleotide content of the 26 mitogenome sequences of PCGs among representatives of the Mytilidae.

Species	Mean Frequencies of Nucleotides (%) Combined for the Three Codon Positions				
	T	C	A	G	Length of Sequence (bp)
All PCG sequences					
*Arcuatula senhousia GU001953*	40.9	12.8	24.0	22.2	11,212
*Arcuatula senhousia OR453539*	41.2	12.8	23.9	22.1	10,971
*Bathymodiolus childressi NC 059707*	40.5	14.5	21.9	23.1	10,866
*Bathymodiolus japonicus AP014560*	41.3	13.7	22.5	22.5	10,842
*Bathymodiolus securiformis NC 039552*	41.4	13.5	22.5	22.5	10,872
*Brachidontes exustus NC 024882*	40.9	13.8	25	20.3	11,100
*Crenomytilus grayanus NC 044128*	36.3	13.8	24.7	25.2	11,298
*Gregariella coralliophaga NC 044129*	40.1	12.1	26.9	20.9	11,181
*Modiolus kurilensis NC 036486*	40.8	12	23.1	24.1	10,929
*Modiolus modiolus KX821782*	40.6	12.1	22.8	24.4	10,839
*Mytilisepta keenae NC 044127*	45.2	9.5	23.1	22.2	10,914
*Mytilus californianus JX486124*	36.8	13.4	26.2	23.6	11,301
*Mytilus chilensis KP100300*	35.4	15	25.2	24.5	11,297
*Mytilus chilensis NC 030633*	35.4	15	25.2	24.5	11,297
*Mytilus coruscus KJ577549*	36.1	14	25.8	24	11,294
*Mytilus coruscus OR453540*	36.3	13.9	25.8	24	10,959
*Mytilus edulis MF407676*	35.3	15	25.2	24.5	11,451
*Mytilus galloprovincialis FJ890849*	35.3	15	25.1	24.6	11,451
*Mytilus trossulus GU936625*	34.5	15.5	24.9	25.1	11,451
*Mytilus trossulus HM462080*	34.5	15.5	25	25.1	11,451
*Perna canaliculus MG766134*	40.5	12.5	26.3	20.8	11,022
*Perna canaliculus MK775558*	40.5	12.5	26.1	20.8	10,914
*Perna perna OK576481*	40.4	12.8	25.9	20.9	10,911
*Perna viridis JQ970425*	42.4	10.2	24.7	22.7	11,004
*Perna viridis MW727515*	42.4	10.2	24.7	22.7	11,004
*Septifer bilocularis NC 044131*	44.8	11.1	24.6	19.5	11,250
Average, *n* = 26	39.18 ± 1.48	13.18 ± 0.79	24.67 ± 0.32	22.96 ± 0.69	11,118
Average, *n* = 26; T + C, A + G	26.18 ± 1.14		23.82 ± 0.50		11,118
PCG sequences for the singleton positions only					
*Arcuatula senhousia GU001953*	47.3	10.41	21.0	21.56	538
*Arcuatula senhousia OR453539*	49.48	9.1	19.92	21.59	477
*Bathymodiolus childressi NC 059707*	49.47	10.53	18.53	21.47	475
*Bathymodiolus japonicus AP014560*	47.68	12.3	18.78	21.52	474
*Bathymodiolus securiformis NC 039552*	50.73	9.15	19.33	20.79	481
*Brachidontes exustus NC 024882*	40.8	15.72	21.2	23.18	509
*Crenomytilus grayanus NC 044128*	42.60	11.59	22.64	23.17	561
*Gregariella coralliophaga NC 044129*	44.26	11.48	23.68	20.58	549
*Modiolus kurilensis NC 036486*	48.91	10.14	19.9	21.87	503
*Modiolus modiolus KX821782*	49.18	9.39	19.39	22.4	490
*Mytilisepta keenae NC 044127*	46.61	10.36	20.12	22.91	502
*Mytilus californianus JX486124*	46.22	9.67	22.67	21.44	569
*Mytilus chilensis KP100300*	46.30	9.15	22.89	21.65	568
*Mytilus chilensis NC 030633*	46.30	9.15	22.89	21.65	568
*Mytilus coruscus KJ577549*	44.1	9.86	23.59	22.54	568
*Mytilus coruscus OR453540*	45.90	9.14	23.24	21.71	525
*Mytilus edulis MF407676*	45.42	9.82	23.13	21.63	601
*Mytilus galloprovincialis FJ890849*	45.42	9.98	22.80	21.80	601
*Mytilus trossulus GU936625*	45.59	9.82	23.13	21.46	601
*Mytilus trossulus HM462080*	45.9	10.32	22.96	21.63	601
*Perna canaliculus MG766134*	50.69	9.43	19.25	20.63	509
*Perna canaliculus MK775558*	50.42	9.49	18.57	21.52	474
*Perna perna OK576481*	48.95	11.76	18.7	21.22	476
*Perna viridis JQ970425*	50.21	9.58	18.75	21.46	480
*Perna viridis MW727515*	50.21	9.58	18.75	21.46	480
*Septifer bilocularis NC 044131*	41.57	13.22	20.50	24.71	522
Average. *n* = 26	46.72 ± 1.48	10.36 ± 0.79	21.10 ± 0.32	21.82 ± 0.69	527
Average. *n* = 26; T + C. A + G	26.18 ± 1.14		23.82 ± 0.50		527

Note. The standard errors for the average frequencies and the heterogeneity of the nucleotide content among the four nucleotide types (T, C, A, G) were estimated by ANOVA/MANOVA.

## Data Availability

Data supporting the reported results can be found in the Supplementary Materials, together with the resources that are given there, including links to publicly archived datasets that were analyzed or generated during the study. Informed consent was obtained from all subjects involved in the study.

## Supplementary Materials

The following supporting information can be downloaded at: https://www.mdpi.com/article/10.3390/ijms25136902/s1.
