# Supplementary material for: Structure, Evolution, and Mitochondrial Genome Analysis of Mussel Species (Bivalvia, Mytilidae)
Source: Int J Mol Sci. 2024 Jun 24;25(13):6902. doi: 10.3390/ijms25136902 (PMC11241113; doi:10.3390/ijms25136902)
Supplement: Supplementary file 1 [file ijms-25-06902-s001.zip › Figure S1, Figure S2. SUPPLEMENT.pdf]

Figure S1. A map of the mitogenome sequences in linear mode for 26 species-specimens of mussels of the family Mytilidae that assembled close to the topological signal of gene trees. The map including 13 PCGs presented in 12 complete sequences, rRNAs and tRNAs obtained in comparisons as basic data set. Structural elements of map are given in different colors: PCGs, yellow; rRNAs, blue; tRNAs, red. Opposite chains given with +/- signs. Most genes are located in “+”-chain. Shifted components of the genome denote location in the “-“-chain. Mitogenomes includes: 12-13 protein-coding genes (abbreviated on the map as follows: *atp6, cox1, cox2, cox3, cytb, nad1, nad2, nad3, nad4, nad4L, nad5,* and *nad6*; designed by yellow color), 2 rRNA genes: rrnS, rrnL (abbreviated for *12S* rRNA and *16S* rRNA; designed by blue color), and 22 tRNA genes that abbreviated as coding for specific amino acids: T-Trp, C-Cys, E-Glu, Y-Tyr, R-Arg, G-Gly, H-His, L1-Leu1, L2-Leu2, S1-Ser1, S2-Ser2, Q-Glu, F-Phe, M-Met, V-Val, A-Ala, D-Asp, N-Asn, P-Pro, I-Ile, K-Lys, and W-Trp (all are designed by red color).

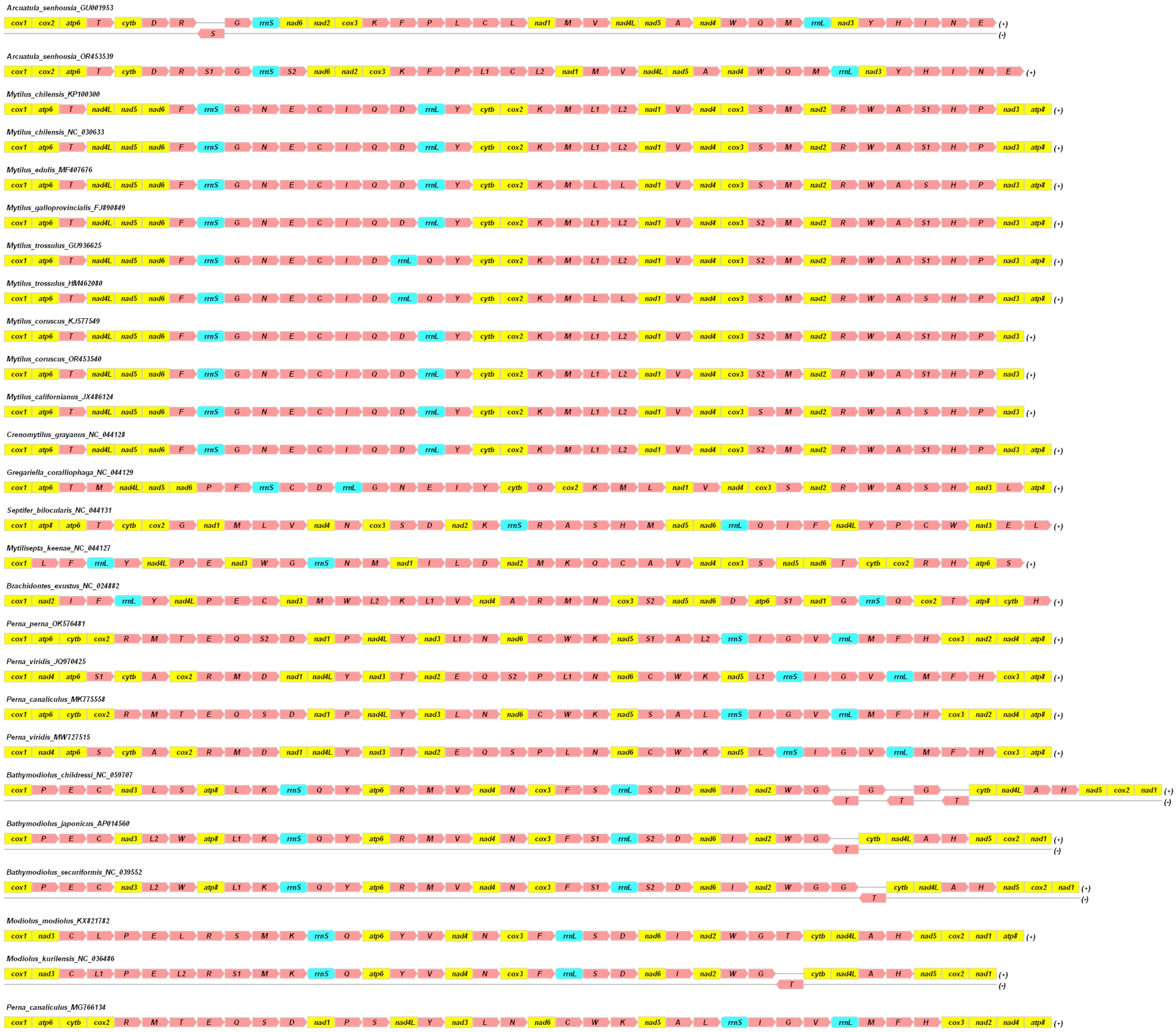

Figure S2. A map of the mitogenome sequences in linear mode for the 27 species of mussels of the family Mytilidae including available 13 PCGs and other genes. Similarly to Fig. S1 structural elements given in different colors. Opposite chains represented with +/- signs. Most genes are located in "+"-chain. Shifted components of the genome denote location in the "-"-chain.

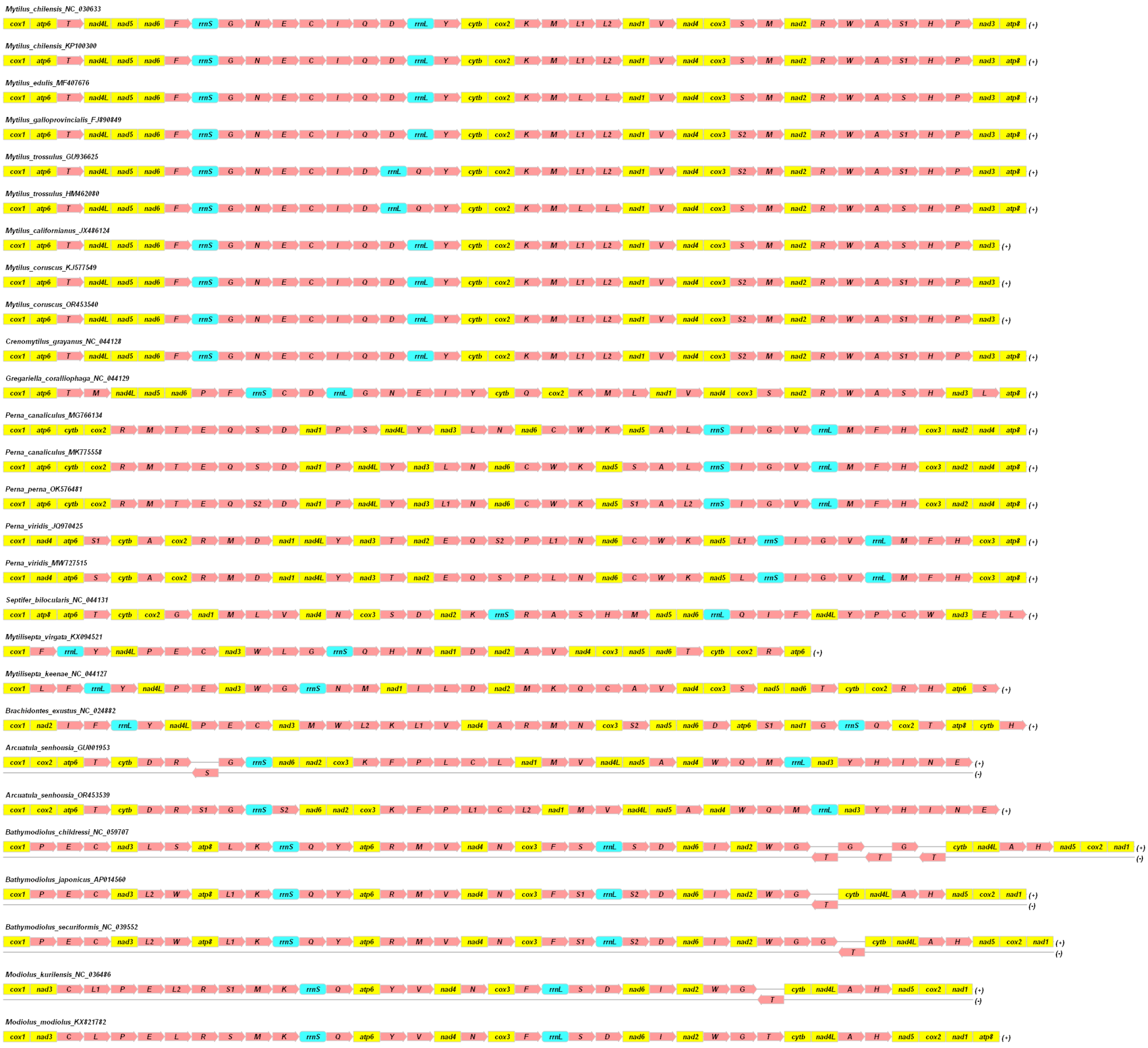

File: Gene order for 26 sequnces-info.csv

<?xml version="1.0" encoding="UTF-8" standalone="no"?> <svg width="771.878mm" height="684.389mm" viewBox="0 0 2188 1940" xmlns="http://www.w3.org/2000/svg" xmlns:xlink="http://www.w3.org/1999/xlink" version="1.2" baseProfile="tiny"> <title>Generated with PhyloSuite http://phylosuite.jushengwu.com/</title> <desc>Generated with PhyloSuite http://phylosuite.jushengwu.com/</desc> <defs> </defs> <g fill="none" stroke="black" stroke-width="1" fill-rule="evenodd" stroke-linecap="square" stroke-linejoin="bevel" > <g fill="none" stroke="#808080" stroke-opacity="1" stroke-width="1" stroke-linecap="square" stroke-linejoin="bevel" transform="matrix(1,0,0,1,0,0)" font-family="MS Shell Dlg 2" font-size="9.75" font-weight="400" font-style="normal" > <polyline fill="none" vector-effect="non-scaling-stroke" points="10,50 1846,50 " /> </g> <g fill="none" stroke="#808080" stroke-opacity="1" stroke-width="1" stroke-linecap="square" stroke-linejoin="bevel" transform="matrix(1,0,0,1,0,0)" font-family="MS Shell Dlg 2" font-size="9.75" font-weight="400" font-style="normal" > <polyline fill="none" vector-effect="non-scaling-stroke" points="10,70 1846,70 " /> </g> <g fill="none" stroke="#808080" stroke-opacity="1" stroke-width="1" stroke-linecap="square" stroke-linejoin="bevel" transform="matrix(1,0,0,1,0,0)" font-family="MS Shell Dlg 2" font-size="9.75" font-weight="400" font-style="normal" > <polyline fill="none" vector-effect="non-scaling-stroke" points="10,140 1897,140 " /> </g> <g fill="none" stroke="#808080" stroke-opacity="1" stroke-width="1" stroke-linecap="square" stroke-linejoin="bevel" transform="matrix(1,0,0,1,0,0)" font-family="MS Shell Dlg 2" font-size="9.75" font-weight="400" font-style="normal" > <polyline fill="none" vector-effect="non-scaling-stroke" points="10,210 1948,210 " /> </g> <g fill="none" stroke="#808080" stroke-opacity="1" stroke-width="1" stroke-linecap="square" stroke-linejoin="bevel" transform="matrix(1,0,0,1,0,0)" font-family="MS Shell Dlg 2" font-size="9.75" font-weight="400" font-style="normal" > <polyline fill="none" vector-effect="non-scaling-stroke" points="10,280 1948,280 " /> </g> <g fill="none" stroke="#808080" stroke-opacity="1" stroke-width="1" stroke-linecap="square" stroke-linejoin="bevel" transform="matrix(1,0,0,1,0,0)" font-family="MS Shell Dlg 2" font-size="9.75" font-weight="400" font-style="normal" > <polyline fill="none" vector-effect="non-scaling-stroke" points="10,350 1948,350 " /> </g> <g fill="none" stroke="#808080" stroke-opacity="1" stroke-width="1" stroke-linecap="square" stroke-linejoin="bevel" transform="matrix(1,0,0,1,0,0)" font-family="MS Shell Dlg 2" font-size="9.75" font-weight="400" font-style="normal" > <polyline fill="none" vector-effect="non-scaling-stroke" points="10,420 1948,420 " /> </g> <g fill="none" stroke="#808080" stroke-opacity="1" stroke-width="1" stroke-linecap="square" stroke-linejoin="bevel" transform="matrix(1,0,0,1,0,0)" font-family="MS Shell Dlg 2" font-size="9.75" font-weight="400" font-style="normal" > <polyline fill="none" vector-effect="non-scaling-stroke" points="10,490 1948,490 " /> </g> <g fill="none" stroke="#808080" stroke-opacity="1" stroke-width="1" stroke-linecap="square" stroke-linejoin="bevel" transform="matrix(1,0,0,1,0,0)" font-family="MS Shell Dlg 2" font-size="9.75" font-weight="400" font-style="normal" > <polyline fill="none" vector-effect="non-scaling-stroke" points="10,560 1948,560 " /> </g> <g fill="none" stroke="#808080" stroke-opacity="1" stroke-width="1" stroke-linecap="square" stroke-linejoin="bevel" transform="matrix(1,0,0,1,0,0)" font-family="MS Shell Dlg 2" font-size="9.75" font-weight="400" font-style="normal" > <polyline fill="none" vector-effect="non-scaling-stroke" points="10,630 1897,630 " /> </g> <g fill="none" stroke="#808080" stroke-opacity="1" stroke-width="1" stroke-linecap="square" stroke-linejoin="bevel" transform="matrix(1,0,0,1,0,0)" font-family="MS Shell Dlg 2" font-size="9.75" font-weight="400" font-style="normal" > <polyline fill="none" vector-effect="non-scaling-stroke" points="10,700 1897,700 " /> </g> <g fill="none" stroke="#808080" stroke-opacity="1" stroke-width="1" stroke-linecap="square" stroke-linejoin="bevel" transform="matrix(1,0,0,1,0,0)" font-family="MS Shell Dlg 2" font-size="9.75" font-weight="400" font-style="normal" > <polyline fill="none" vector-effect="non-scaling-stroke" points="10,770 1897,770 " /> </g> <g fill="none" stroke="#808080" stroke-opacity="1" stroke-width="1" stroke-linecap="square" stroke-linejoin="bevel" transform="matrix(1,0,0,1,0,0)" font-family="MS Shell Dlg 2" font-size="9.75" font-weight="400" font-style="normal" > <polyline fill="none" vector-effect="non-scaling-stroke" points="10,840 1948,840 " /> </g> <g fill="none" stroke="#808080" stroke-opacity="1" stroke-width="1" stroke-linecap="square" stroke-linejoin="bevel" transform="matrix(1,0,0,1,0,0)" font-family="MS Shell Dlg 2" font-size="9.75" font-weight="400" font-style="normal" > <polyline fill="none" vector-effect="non-scaling-stroke" points="10,910 1948,910 " /> </g> <g fill="none" stroke="#808080" stroke-opacity="1" stroke-width="1" stroke-linecap="square" stroke-linejoin="bevel" transform="matrix(1,0,0,1,0,0)" font-family="MS Shell Dlg 2" font-size="9.75" font-weight="400" font-style="normal" > <polyline fill="none" vector-effect="non-scaling-stroke" points="10,980 1948,980 " /> </g> <g fill="none" stroke="#808080" stroke-opacity="1" stroke-width="1" stroke-linecap="square" stroke-linejoin="bevel" transform="matrix(1,0,0,1,0,0)" font-family="MS Shell Dlg 2" font-size="9.75" font-weight="400" font-style="normal" > <polyline fill="none" vector-effect="non-scaling-stroke" points="10,1050 1897,1050 " /> </g> <g fill="none" stroke="#808080" stroke-opacity="1" stroke-width="1" stroke-linecap="square" stroke-linejoin="bevel" transform="matrix(1,0,0,1,0,0)" font-family="MS Shell Dlg 2" font-size="9.75" font-weight="400" font-style="normal" > <polyline fill="none" vector-effect="non-scaling-stroke" points="10,1120 1948,1120 " />

</g> <g fill="none" stroke="#808080" stroke-opacity="1" stroke-width="1" stroke-linecap="square" stroke-linejoin="bevel" transform="matrix(1,0,0,1,0,0)" font-family="MS Shell Dlg 2" font-size="9.75" font-weight="400" font-style="normal" > <polyline fill="none" vector-effect="non-scaling-stroke" points="10,1190 1948,1190 " />
</g> <g fill="none" stroke="#808080" stroke-opacity="1" stroke-width="1" stroke-linecap="square" stroke-linejoin="bevel" transform="matrix(1,0,0,1,0,0)" font-family="MS Shell Dlg 2" font-size="9.75" font-weight="400" font-style="normal" > <polyline fill="none" vector-effect="non-scaling-stroke" points="10,1260 1948,1260 " />
</g> <g fill="none" stroke="#808080" stroke-opacity="1" stroke-width="1" stroke-linecap="square" stroke-linejoin="bevel" transform="matrix(1,0,0,1,0,0)" font-family="MS Shell Dlg 2" font-size="9.75" font-weight="400" font-style="normal" > <polyline fill="none" vector-effect="non-scaling-stroke" points="10,1330 1948,1330 " />
</g> <g fill="none" stroke="#808080" stroke-opacity="1" stroke-width="1" stroke-linecap="square" stroke-linejoin="bevel" transform="matrix(1,0,0,1,0,0)" font-family="MS Shell Dlg 2" font-size="9.75" font-weight="400" font-style="normal" > <polyline fill="none" vector-effect="non-scaling-stroke" points="10,1400 1948,1400 " />
</g> <g fill="none" stroke="#808080" stroke-opacity="1" stroke-width="1" stroke-linecap="square" stroke-linejoin="bevel" transform="matrix(1,0,0,1,0,0)" font-family="MS Shell Dlg 2" font-size="9.75" font-weight="400" font-style="normal" > <polyline fill="none" vector-effect="non-scaling-stroke" points="10,1470 2152,1470 " />
</g> <g fill="none" stroke="#808080" stroke-opacity="1" stroke-width="1" stroke-linecap="square" stroke-linejoin="bevel" transform="matrix(1,0,0,1,0,0)" font-family="MS Shell Dlg 2" font-size="9.75" font-weight="400" font-style="normal" > <polyline fill="none" vector-effect="non-scaling-stroke" points="10,1490 2152,1490 " />
</g> <g fill="none" stroke="#808080" stroke-opacity="1" stroke-width="1" stroke-linecap="square" stroke-linejoin="bevel" transform="matrix(1,0,0,1,0,0)" font-family="MS Shell Dlg 2" font-size="9.75" font-weight="400" font-style="normal" > <polyline fill="none" vector-effect="non-scaling-stroke" points="10,1560 1948,1560 " />
</g> <g fill="none" stroke="#808080" stroke-opacity="1" stroke-width="1" stroke-linecap="square" stroke-linejoin="bevel" transform="matrix(1,0,0,1,0,0)" font-family="MS Shell Dlg 2" font-size="9.75" font-weight="400" font-style="normal" > <polyline fill="none" vector-effect="non-scaling-stroke" points="10,1580 1948,1580 " />
</g> <g fill="none" stroke="#808080" stroke-opacity="1" stroke-width="1" stroke-linecap="square" stroke-linejoin="bevel" transform="matrix(1,0,0,1,0,0)" font-family="MS Shell Dlg 2" font-size="9.75" font-weight="400" font-style="normal" > <polyline fill="none" vector-effect="non-scaling-stroke" points="10,1650 1999,1650 " />
</g> <g fill="none" stroke="#808080" stroke-opacity="1" stroke-width="1" stroke-linecap="square" stroke-linejoin="bevel" transform="matrix(1,0,0,1,0,0)" font-family="MS Shell Dlg 2" font-size="9.75" font-weight="400" font-style="normal" > <polyline fill="none" vector-effect="non-scaling-stroke" points="10,1670 1999,1670 " />
</g> <g fill="none" stroke="#808080" stroke-opacity="1" stroke-width="1" stroke-linecap="square" stroke-linejoin="bevel" transform="matrix(1,0,0,1,0,0)" font-family="MS Shell Dlg 2" font-size="9.75" font-weight="400" font-style="normal" > <polyline fill="none" vector-effect="non-scaling-stroke" points="10,1740 1897,1740 " />
</g> <g fill="none" stroke="#808080" stroke-opacity="1" stroke-width="1" stroke-linecap="square" stroke-linejoin="bevel" transform="matrix(1,0,0,1,0,0)" font-family="MS Shell Dlg 2" font-size="9.75" font-weight="400" font-style="normal" > <polyline fill="none" vector-effect="non-scaling-stroke" points="10,1810 1846,1810 " />
</g> <g fill="none" stroke="#808080" stroke-opacity="1" stroke-width="1" stroke-linecap="square" stroke-linejoin="bevel" transform="matrix(1,0,0,1,0,0)" font-family="MS Shell Dlg 2" font-size="9.75" font-weight="400" font-style="normal" > <polyline fill="none" vector-effect="non-scaling-stroke" points="10,1830 1846,1830 " />
</g> <g fill="none" stroke="#808080" stroke-opacity="1" stroke-width="1" stroke-linecap="square" stroke-linejoin="bevel" transform="matrix(1,0,0,1,0,0)" font-family="MS Shell Dlg 2" font-size="9.75" font-weight="400" font-style="normal" > <polyline fill="none" vector-effect="non-scaling-stroke" points="10,1900 1948,1900 " />
</g> <g fill="none" stroke="#000000" stroke-opacity="1" stroke-width="1" stroke-linecap="square" stroke-linejoin="bevel" transform="matrix(1,0,0,1,10,10)" font-family="MS Shell Dlg 2" font-size="9.75" font-weight="400" font-style="normal" > <text fill="#000000" fill-opacity="1" stroke="none" xml:space="preserve" x="4" y="17" font-family="Arial" font-size="10pt" font-weight="700" font-style="italic" >Arcuatula_senhousia_GU001953</text> </g> <g fill="#ffff33" fill-opacity="1" stroke="#bfbfbf" stroke-opacity="1" stroke-width="1" stroke-linecap="square" stroke-linejoin="bevel" transform="matrix(1,0,0,1,10,40)" font-family="MS Shell Dlg 2" font-size="9.75" font-weight="400" font-style="normal" > <rect x="0" y="0" width="50" height="20"/> </g> <g fill="none" stroke="#000000" stroke-opacity="1" stroke-width="1" stroke-linecap="square" stroke-linejoin="bevel" transform="matrix(1,0,0,1,18,38)" font-family="MS Shell Dlg 2" font-size="9.75" font-weight="400" font-style="normal" > <text fill="#000000" fill-opacity="1" stroke="none" xml:space="preserve" x="4" y="17" font-family="Arial" font-size="10pt" font-weight="700" font-style="italic" >cox1</text> </g> <g fill="#ffff33" fill-opacity="1" stroke="#bfbfbf" stroke-opacity="1" stroke-width="1" stroke-linecap="square" stroke-linejoin="bevel" transform="matrix(1,0,0,1,61,40)" font-family="MS Shell Dlg 2" font-size="9.75" font-weight="400" font-style="normal" > <rect x="0" y="0" width="50" height="20"/> </g>
<g fill="none" stroke="#000000" stroke-opacity="1" stroke-width="1" stroke-linecap="square" stroke-linejoin="bevel" transform="matrix(1,0,0,1,69,38)" font-family="MS Shell Dlg 2" font-size="9.75" font-

weight="400" font-style="normal" > <text fill="#000000" fill-opacity="1" stroke="none" xml:space="preserve" x="4" y="17" font-family="Arial" font-size="10pt" font-weight="700" font-style="italic" >cox2</text> </g> <g fill="#ffff33" fill-opacity="1" stroke="#bfbfbf" stroke-opacity="1" stroke-width="1" stroke-linecap="square" stroke-linejoin="bevel" transform="matrix(1,0,0,1,112,40)" font-family="MS Shell Dlg 2" font-size="9.75" font-weight="400" font-style="normal" > <rect x="0" y="0" width="50" height="20"/> </g> <g fill="none" stroke="#000000" stroke-opacity="1" stroke-width="1" stroke-linecap="square" stroke-linejoin="bevel" transform="matrix(1,0,0,1,120.5,38)" font-family="MS Shell Dlg 2" font-size="9.75" font-weight="400" font-style="normal" > <text fill="#000000" fill-opacity="1" stroke="none" xml:space="preserve" x="4" y="17" font-family="Arial" font-size="10pt" font-weight="700" font-style="italic" >atp6</text> </g> <g fill="#ff9999" fill-opacity="1" stroke="#bfbfbf" stroke-opacity="1" stroke-width="1" stroke-linecap="square" stroke-linejoin="bevel" transform="matrix(1,0,0,1,163,40)" font-family="MS Shell Dlg 2" font-size="9.75" font-weight="400" font-style="normal" > <path vector-effect="none" fill-rule="evenodd" d="M0,1.7 L42.5,1.7 L42.5,0 L50,10 L42.5,20 L42.5,18.3 L0,18.3 L0,1.7"/> </g> <g fill="none" stroke="#000000" stroke-opacity="1" stroke-width="1" stroke-linecap="square" stroke-linejoin="bevel" transform="matrix(1,0,0,1,176.132,38)" font-family="MS Shell Dlg 2" font-size="9.75" font-weight="400" font-style="normal" > <text fill="#000000" fill-opacity="1" stroke="none" xml:space="preserve" x="4" y="17" font-family="Arial" font-size="10pt" font-weight="700" font-style="italic" >T</text> </g> <g fill="#ffff33" fill-opacity="1" stroke="#bfbfbf" stroke-opacity="1" stroke-width="1" stroke-linecap="square" stroke-linejoin="bevel" transform="matrix(1,0,0,1,214,40)" font-family="MS Shell Dlg 2" font-size="9.75" font-weight="400" font-style="normal" > <rect x="0" y="0" width="50" height="20"/> </g> <g fill="none" stroke="#000000" stroke-opacity="1" stroke-width="1" stroke-linecap="square" stroke-linejoin="bevel" transform="matrix(1,0,0,1,223.5,38)" font-family="MS Shell Dlg 2" font-size="9.75" font-weight="400" font-style="normal" > <text fill="#000000" fill-opacity="1" stroke="none" xml:space="preserve" x="4" y="17" font-family="Arial" font-size="10pt" font-weight="700" font-style="italic" >cytb</text> </g> <g fill="#ff9999" fill-opacity="1" stroke="#bfbfbf" stroke-opacity="1" stroke-width="1" stroke-linecap="square" stroke-linejoin="bevel" transform="matrix(1,0,0,1,265,40)" font-family="MS Shell Dlg 2" font-size="9.75" font-weight="400" font-style="normal" > <path vector-effect="none" fill-rule="evenodd" d="M0,1.7 L42.5,1.7 L42.5,0 L50,10 L42.5,20 L42.5,18.3 L0,18.3 L0,1.7"/> </g> <g fill="none" stroke="#000000" stroke-opacity="1" stroke-width="1" stroke-linecap="square" stroke-linejoin="bevel" transform="matrix(1,0,0,1,278.132,38)" font-family="MS Shell Dlg 2" font-size="9.75" font-weight="400" font-style="normal" > <text fill="#000000" fill-opacity="1" stroke="none" xml:space="preserve" x="4" y="17" font-family="Arial" font-size="10pt" font-weight="700" font-style="italic" >D</text> </g> <g fill="#ff9999" fill-opacity="1" stroke="#bfbfbf" stroke-opacity="1" stroke-width="1" stroke-linecap="square" stroke-linejoin="bevel" transform="matrix(1,0,0,1,316,40)" font-family="MS Shell Dlg 2" font-size="9.75" font-weight="400" font-style="normal" > <path vector-effect="none" fill-rule="evenodd" d="M0,1.7 L42.5,1.7 L42.5,0 L50,10 L42.5,20 L42.5,18.3 L0,18.3 L0,1.7"/> </g> <g fill="none" stroke="#000000" stroke-opacity="1" stroke-width="1" stroke-linecap="square" stroke-linejoin="bevel" transform="matrix(1,0,0,1,329.132,38)" font-family="MS Shell Dlg 2" font-size="9.75" font-weight="400" font-style="normal" > <text fill="#000000" fill-opacity="1" stroke="none" xml:space="preserve" x="4" y="17" font-family="Arial" font-size="10pt" font-weight="700" font-style="italic" >R</text> </g> <g fill="#ff9999" fill-opacity="1" stroke="#bfbfbf" stroke-opacity="1" stroke-width="1" stroke-linecap="square" stroke-linejoin="bevel" transform="matrix(1,0,0,1,367,60)" font-family="MS Shell Dlg 2" font-size="9.75" font-weight="400" font-style="normal" > <path vector-effect="none" fill-rule="evenodd" d="M0,10 L7.5,0 L7.5,1.7 L50,1.7 L50,18.3 L7.5,18.3 L7.5,20 L0,10"/> </g> <g fill="none" stroke="#000000" stroke-opacity="1" stroke-width="1" stroke-linecap="square" stroke-linejoin="bevel" transform="matrix(1,0,0,1,387.868,58)" font-family="MS Shell Dlg 2" font-size="9.75" font-weight="400" font-style="normal" > <text fill="#000000" fill-opacity="1" stroke="none" xml:space="preserve" x="4" y="17" font-family="Arial" font-size="10pt" font-weight="700" font-style="italic" >S</text> </g> <g fill="#ff9999" fill-opacity="1" stroke="#bfbfbf" stroke-opacity="1" stroke-width="1" stroke-linecap="square" stroke-linejoin="bevel" transform="matrix(1,0,0,1,418,40)" font-family="MS Shell Dlg 2" font-size="9.75" font-weight="400" font-style="normal" > <path vector-effect="none" fill-rule="evenodd" d="M0,1.7 L42.5,1.7 L42.5,0 L50,10 L42.5,20 L42.5,18.3 L0,18.3 L0,1.7"/> </g> <g fill="none" stroke="#000000" stroke-opacity="1" stroke-width="1" stroke-linecap="square" stroke-linejoin="bevel" transform="matrix(1,0,0,1,431.132,38)" font-family="MS Shell Dlg 2" font-size="9.75" font-weight="400" font-style="normal" > <text fill="#000000" fill-opacity="1" stroke="none" xml:space="preserve" x="4" y="17" font-family="Arial" font-size="10pt" font-weight="700" font-style="italic" >G</text> </g> <g fill="#99ffff" fill-opacity="1" stroke="#bfbfbf" stroke-opacity="1" stroke-width="1" stroke-linecap="square" stroke-linejoin="bevel" transform="matrix(1,0,0,1,469,40)" font-family="MS Shell Dlg 2" font-size="9.75" font-weight="400" font-style="normal" > <path vector-effect="none" fill-rule="evenodd" d="M0,5 C0,2.23858 2.23858,0 5,0 L45,0

C47.7614,0 50,2.23858 50,5 L50,15 C50,17.7614 47.7614,20 45,20 L5,20 C2.23858,20 0,17.7614 0,15 L0,5"/> </g> <g fill="none" stroke="#000000" stroke-opacity="1" stroke-width="1" stroke-linecap="square" stroke-linejoin="bevel" transform="matrix(1,0,0,1,477.5,38)" font-family="MS Shell Dlg 2" font-size="9.75" font-weight="400" font-style="normal" > <text fill="#000000" fill-opacity="1" stroke="none" xml:space="preserve" x="4" y="17" font-family="Arial" font-size="10pt" font-weight="700" font-style="italic" >rrnS</text> </g> <g fill="#ffff33" fill-opacity="1" stroke="#bfbfbf" stroke-opacity="1" stroke-width="1" stroke-linecap="square" stroke-linejoin="bevel" transform="matrix(1,0,0,1,520,40)" font-family="MS Shell Dlg 2" font-size="9.75" font-weight="400" font-style="normal" > <rect x="0" y="0" width="50" height="20"/> </g> <g fill="none" stroke="#000000" stroke-opacity="1" stroke-width="1" stroke-linecap="square" stroke-linejoin="bevel" transform="matrix(1,0,0,1,527,38)" font-family="MS Shell Dlg 2" font-size="9.75" font-weight="400" font-style="normal" > <text fill="#000000" fill-opacity="1" stroke="none" xml:space="preserve" x="4" y="17" font-family="Arial" font-size="10pt" font-weight="700" font-style="italic" >nad6</text> </g> <g fill="#ffff33" fill-opacity="1" stroke="#bfbfbf" stroke-opacity="1" stroke-width="1" stroke-linecap="square" stroke-linejoin="bevel" transform="matrix(1,0,0,1,571,40)" font-family="MS Shell Dlg 2" font-size="9.75" font-weight="400" font-style="normal" > <rect x="0" y="0" width="50" height="20"/> </g> <g fill="none" stroke="#000000" stroke-opacity="1" stroke-width="1" stroke-linecap="square" stroke-linejoin="bevel" transform="matrix(1,0,0,1,578,38)" font-family="MS Shell Dlg 2" font-size="9.75" font-weight="400" font-style="normal" > <text fill="#000000" fill-opacity="1" stroke="none" xml:space="preserve" x="4" y="17" font-family="Arial" font-size="10pt" font-weight="700" font-style="italic" >nad2</text> </g> <g fill="#ffff33" fill-opacity="1" stroke="#bfbfbf" stroke-opacity="1" stroke-width="1" stroke-linecap="square" stroke-linejoin="bevel" transform="matrix(1,0,0,1,622,40)" font-family="MS Shell Dlg 2" font-size="9.75" font-weight="400" font-style="normal" > <rect x="0" y="0" width="50" height="20"/> </g> <g fill="none" stroke="#000000" stroke-opacity="1" stroke-width="1" stroke-linecap="square" stroke-linejoin="bevel" transform="matrix(1,0,0,1,630,38)" font-family="MS Shell Dlg 2" font-size="9.75" font-weight="400" font-style="normal" > <text fill="#000000" fill-opacity="1" stroke="none" xml:space="preserve" x="4" y="17" font-family="Arial" font-size="10pt" font-weight="700" font-style="italic" >cox3</text> </g> <g fill="#ff9999" fill-opacity="1" stroke="#bfbfbf" stroke-opacity="1" stroke-width="1" stroke-linecap="square" stroke-linejoin="bevel" transform="matrix(1,0,0,1,673,40)" font-family="MS Shell Dlg 2" font-size="9.75" font-weight="400" font-style="normal" > <path vector-effect="none" fill-rule="evenodd" d="M0,1.7 L42.5,1.7 L42.5,0 L50,10 L42.5,20 L42.5,18.3 L0,18.3 L0,1.7"/> </g> <g fill="none" stroke="#000000" stroke-opacity="1" stroke-width="1" stroke-linecap="square" stroke-linejoin="bevel" transform="matrix(1,0,0,1,686.633,38)" font-family="MS Shell Dlg 2" font-size="9.75" font-weight="400" font-style="normal" > <text fill="#000000" fill-opacity="1" stroke="none" xml:space="preserve" x="4" y="17" font-family="Arial" font-size="10pt" font-weight="700" font-style="italic" >K</text> </g> <g fill="#ff9999" fill-opacity="1" stroke="#bfbfbf" stroke-opacity="1" stroke-width="1" stroke-linecap="square" stroke-linejoin="bevel" transform="matrix(1,0,0,1,724,40)" font-family="MS Shell Dlg 2" font-size="9.75" font-weight="400" font-style="normal" > <path vector-effect="none" fill-rule="evenodd" d="M0,1.7 L42.5,1.7 L42.5,0 L50,10 L42.5,20 L42.5,18.3 L0,18.3 L0,1.7"/> </g> <g fill="none" stroke="#000000" stroke-opacity="1" stroke-width="1" stroke-linecap="square" stroke-linejoin="bevel" transform="matrix(1,0,0,1,737.633,38)" font-family="MS Shell Dlg 2" font-size="9.75" font-weight="400" font-style="normal" > <text fill="#000000" fill-opacity="1" stroke="none" xml:space="preserve" x="4" y="17" font-family="Arial" font-size="10pt" font-weight="700" font-style="italic" >F</text> </g> <g fill="#ff9999" fill-opacity="1" stroke="#bfbfbf" stroke-opacity="1" stroke-width="1" stroke-linecap="square" stroke-linejoin="bevel" transform="matrix(1,0,0,1,775,40)" font-family="MS Shell Dlg 2" font-size="9.75" font-weight="400" font-style="normal" > <path vector-effect="none" fill-rule="evenodd" d="M0,1.7 L42.5,1.7 L42.5,0 L50,10 L42.5,20 L42.5,18.3 L0,18.3 L0,1.7"/> </g> <g fill="none" stroke="#000000" stroke-opacity="1" stroke-width="1" stroke-linecap="square" stroke-linejoin="bevel" transform="matrix(1,0,0,1,788.633,38)" font-family="MS Shell Dlg 2" font-size="9.75" font-weight="400" font-style="normal" > <text fill="#000000" fill-opacity="1" stroke="none" xml:space="preserve" x="4" y="17" font-family="Arial" font-size="10pt" font-weight="700" font-style="italic" >P</text> </g> <g fill="#ff9999" fill-opacity="1" stroke="#bfbfbf" stroke-opacity="1" stroke-width="1" stroke-linecap="square" stroke-linejoin="bevel" transform="matrix(1,0,0,1,826,40)" font-family="MS Shell Dlg 2" font-size="9.75" font-weight="400" font-style="normal" > <path vector-effect="none" fill-rule="evenodd" d="M0,1.7 L42.5,1.7 L42.5,0 L50,10 L42.5,20 L42.5,18.3 L0,18.3 L0,1.7"/> </g> <g fill="none" stroke="#000000" stroke-opacity="1" stroke-width="1" stroke-linecap="square" stroke-linejoin="bevel" transform="matrix(1,0,0,1,840.133,38)" font-family="MS Shell Dlg 2" font-size="9.75" font-weight="400" font-style="normal" > <text fill="#000000" fill-opacity="1" stroke="none" xml:space="preserve" x="4" y="17" font-family="Arial" font-size="10pt" font-weight="700" font-style="italic" >L</text> </g> <g fill="#ff9999" fill-opacity="1" stroke="#bfbfbf" stroke-opacity="1" stroke-width="1" stroke-linecap="square" stroke-linejoin="bevel"

transform="matrix(1,0,0,1,877,40)" font-family="MS Shell Dlg 2" font-size="9.75" font-weight="400" font-style="normal" > <path vector-effect="none" fill-rule="evenodd" d="M0,1.7 L42.5,1.7 L42.5,0 L50,10 L42.5,20 L42.5,18.3 L0,18.3 L0,1.7"/> </g> <g fill="none" stroke="#000000" stroke-opacity="1" stroke-width="1" stroke-linecap="square" stroke-linejoin="bevel" transform="matrix(1,0,0,1,890.133,38)" font-family="MS Shell Dlg 2" font-size="9.75" font-weight="400" font-style="normal" > <text fill="#000000" fill-opacity="1" stroke="none" xml:space="preserve" x="4" y="17" font-family="Arial" font-size="10pt" font-weight="700" font-style="italic" >C</text> </g> <g fill="#ff9999" fill-opacity="1" stroke="#bfbfbf" stroke-opacity="1" stroke-width="1" stroke-linecap="square" stroke-linejoin="bevel" transform="matrix(1,0,0,1,928,40)" font-family="MS Shell Dlg 2" font-size="9.75" font-weight="400" font-style="normal" > <path vector-effect="none" fill-rule="evenodd" d="M0,1.7 L42.5,1.7 L42.5,0 L50,10 L42.5,20 L42.5,18.3 L0,18.3 L0,1.7"/> </g> <g fill="none" stroke="#000000" stroke-opacity="1" stroke-width="1" stroke-linecap="square" stroke-linejoin="bevel" transform="matrix(1,0,0,1,942.133,38)" font-family="MS Shell Dlg 2" font-size="9.75" font-weight="400" font-style="normal" > <text fill="#000000" fill-opacity="1" stroke="none" xml:space="preserve" x="4" y="17" font-family="Arial" font-size="10pt" font-weight="700" font-style="italic" >L</text> </g> <g fill="#ffff33" fill-opacity="1" stroke="#bfbfbf" stroke-opacity="1" stroke-width="1" stroke-linecap="square" stroke-linejoin="bevel" transform="matrix(1,0,0,1,979,40)" font-family="MS Shell Dlg 2" font-size="9.75" font-weight="400" font-style="normal" > <rect x="0" y="0" width="50" height="20"/> </g> <g fill="none" stroke="#000000" stroke-opacity="1" stroke-width="1" stroke-linecap="square" stroke-linejoin="bevel" transform="matrix(1,0,0,1,986,38)" font-family="MS Shell Dlg 2" font-size="9.75" font-weight="400" font-style="normal" > <text fill="#000000" fill-opacity="1" stroke="none" xml:space="preserve" x="4" y="17" font-family="Arial" font-size="10pt" font-weight="700" font-style="italic" >nad1</text> </g> <g fill="#ff9999" fill-opacity="1" stroke="#bfbfbf" stroke-opacity="1" stroke-width="1" stroke-linecap="square" stroke-linejoin="bevel" transform="matrix(1,0,0,1,1030,40)" font-family="MS Shell Dlg 2" font-size="9.75" font-weight="400" font-style="normal" > <path vector-effect="none" fill-rule="evenodd" d="M0,1.7 L42.5,1.7 L42.5,0 L50,10 L42.5,20 L42.5,18.3 L0,18.3 L0,1.7"/> </g> <g fill="none" stroke="#000000" stroke-opacity="1" stroke-width="1" stroke-linecap="square" stroke-linejoin="bevel" transform="matrix(1,0,0,1,1042.13,38)" font-family="MS Shell Dlg 2" font-size="9.75" font-weight="400" font-style="normal" > <text fill="#000000" fill-opacity="1" stroke="none" xml:space="preserve" x="4" y="17" font-family="Arial" font-size="10pt" font-weight="700" font-style="italic" >M</text> </g> <g fill="#ff9999" fill-opacity="1" stroke="#bfbfbf" stroke-opacity="1" stroke-width="1" stroke-linecap="square" stroke-linejoin="bevel" transform="matrix(1,0,0,1,1081,40)" font-family="MS Shell Dlg 2" font-size="9.75" font-weight="400" font-style="normal" > <path vector-effect="none" fill-rule="evenodd" d="M0,1.7 L42.5,1.7 L42.5,0 L50,10 L42.5,20 L42.5,18.3 L0,18.3 L0,1.7"/> </g> <g fill="none" stroke="#000000" stroke-opacity="1" stroke-width="1" stroke-linecap="square" stroke-linejoin="bevel" transform="matrix(1,0,0,1,1094.13,38)" font-family="MS Shell Dlg 2" font-size="9.75" font-weight="400" font-style="normal" > <text fill="#000000" fill-opacity="1" stroke="none" xml:space="preserve" x="4" y="17" font-family="Arial" font-size="10pt" font-weight="700" font-style="italic" >V</text> </g> <g fill="#ffff33" fill-opacity="1" stroke="#bfbfbf" stroke-opacity="1" stroke-width="1" stroke-linecap="square" stroke-linejoin="bevel" transform="matrix(1,0,0,1,1132,40)" font-family="MS Shell Dlg 2" font-size="9.75" font-weight="400" font-style="normal" > <rect x="0" y="0" width="50" height="20"/> </g> <g fill="none" stroke="#000000" stroke-opacity="1" stroke-width="1" stroke-linecap="square" stroke-linejoin="bevel" transform="matrix(1,0,0,1,1136,38)" font-family="MS Shell Dlg 2" font-size="9.75" font-weight="400" font-style="normal" > <text fill="#000000" fill-opacity="1" stroke="none" xml:space="preserve" x="4" y="17" font-family="Arial" font-size="10pt" font-weight="700" font-style="italic" >nad4L</text> </g> <g fill="#ffff33" fill-opacity="1" stroke="#bfbfbf" stroke-opacity="1" stroke-width="1" stroke-linecap="square" stroke-linejoin="bevel" transform="matrix(1,0,0,1,1183,40)" font-family="MS Shell Dlg 2" font-size="9.75" font-weight="400" font-style="normal" > <rect x="0" y="0" width="50" height="20"/> </g> <g fill="none" stroke="#000000" stroke-opacity="1" stroke-width="1" stroke-linecap="square" stroke-linejoin="bevel" transform="matrix(1,0,0,1,1190,38)" font-family="MS Shell Dlg 2" font-size="9.75" font-weight="400" font-style="normal" > <text fill="#000000" fill-opacity="1" stroke="none" xml:space="preserve" x="4" y="17" font-family="Arial" font-size="10pt" font-weight="700" font-style="italic" >nad5</text> </g> <g fill="#ff9999" fill-opacity="1" stroke="#bfbfbf" stroke-opacity="1" stroke-width="1" stroke-linecap="square" stroke-linejoin="bevel" transform="matrix(1,0,0,1,1234,40)" font-family="MS Shell Dlg 2" font-size="9.75" font-weight="400" font-style="normal" > <path vector-effect="none" fill-rule="evenodd" d="M0,1.7 L42.5,1.7 L42.5,0 L50,10 L42.5,20 L42.5,18.3 L0,18.3 L0,1.7"/> </g> <g fill="none" stroke="#000000" stroke-opacity="1" stroke-width="1" stroke-linecap="square" stroke-linejoin="bevel" transform="matrix(1,0,0,1,1247.13,38)" font-family="MS Shell Dlg 2" font-size="9.75" font-weight="400" font-style="normal" > <text fill="#000000" fill-opacity="1" stroke="none" xml:space="preserve" x="4" y="17" font-family="Arial" font-size="10pt" font-weight="700" font-style="italic" >A</text> </g> <g fill="#ffff33"

fill-opacity="1" stroke="#bfbfbf" stroke-opacity="1" stroke-width="1" stroke-linecap="square" stroke-linejoin="bevel" transform="matrix(1,0,0,1,1285,40)" font-family="MS Shell Dlg 2" font-size="9.75" font-weight="400" font-style="normal" > <rect x="0" y="0" width="50" height="20"/> </g> <g fill="none" stroke="#000000" stroke-opacity="1" stroke-width="1" stroke-linecap="square" stroke-linejoin="bevel" transform="matrix(1,0,0,1,1292,38)" font-family="MS Shell Dlg 2" font-size="9.75" font-weight="400" font-style="normal" > <text fill="#000000" fill-opacity="1" stroke="none" xml:space="preserve" x="4" y="17" font-family="Arial" font-size="10pt" font-weight="700" font-style="italic" >nad4</text> </g> <g fill="#ff9999" fill-opacity="1" stroke="#bfbfbf" stroke-opacity="1" stroke-width="1" stroke-linecap="square" stroke-linejoin="bevel" transform="matrix(1,0,0,1,1336,40)" font-family="MS Shell Dlg 2" font-size="9.75" font-weight="400" font-style="normal" > <path vector-effect="none" fill-rule="evenodd" d="M0,1.7 L42.5,1.7 L42.5,0 L50,10 L42.5,20 L42.5,18.3 L0,18.3 L0,1.7"/> </g> <g fill="none" stroke="#000000" stroke-opacity="1" stroke-width="1" stroke-linecap="square" stroke-linejoin="bevel" transform="matrix(1,0,0,1,1347.13,38)" font-family="MS Shell Dlg 2" font-size="9.75" font-weight="400" font-style="normal" > <text fill="#000000" fill-opacity="1" stroke="none" xml:space="preserve" x="4" y="17" font-family="Arial" font-size="10pt" font-weight="700" font-style="italic" >W</text> </g> <g fill="#ff9999" fill-opacity="1" stroke="#bfbfbf" stroke-opacity="1" stroke-width="1" stroke-linecap="square" stroke-linejoin="bevel" transform="matrix(1,0,0,1,1387,40)" font-family="MS Shell Dlg 2" font-size="9.75" font-weight="400" font-style="normal" > <path vector-effect="none" fill-rule="evenodd" d="M0,1.7 L42.5,1.7 L42.5,0 L50,10 L42.5,20 L42.5,18.3 L0,18.3 L0,1.7"/> </g> <g fill="none" stroke="#000000" stroke-opacity="1" stroke-width="1" stroke-linecap="square" stroke-linejoin="bevel" transform="matrix(1,0,0,1,1399.63,38)" font-family="MS Shell Dlg 2" font-size="9.75" font-weight="400" font-style="normal" > <text fill="#000000" fill-opacity="1" stroke="none" xml:space="preserve" x="4" y="17" font-family="Arial" font-size="10pt" font-weight="700" font-style="italic" >Q</text> </g> <g fill="#ff9999" fill-opacity="1" stroke="#bfbfbf" stroke-opacity="1" stroke-width="1" stroke-linecap="square" stroke-linejoin="bevel" transform="matrix(1,0,0,1,1438,40)" font-family="MS Shell Dlg 2" font-size="9.75" font-weight="400" font-style="normal" > <path vector-effect="none" fill-rule="evenodd" d="M0,1.7 L42.5,1.7 L42.5,0 L50,10 L42.5,20 L42.5,18.3 L0,18.3 L0,1.7"/> </g> <g fill="none" stroke="#000000" stroke-opacity="1" stroke-width="1" stroke-linecap="square" stroke-linejoin="bevel" transform="matrix(1,0,0,1,1450.13,38)" font-family="MS Shell Dlg 2" font-size="9.75" font-weight="400" font-style="normal" > <text fill="#000000" fill-opacity="1" stroke="none" xml:space="preserve" x="4" y="17" font-family="Arial" font-size="10pt" font-weight="700" font-style="italic" >M</text> </g> <g fill="#99ffff" fill-opacity="1" stroke="#bfbfbf" stroke-opacity="1" stroke-width="1" stroke-linecap="square" stroke-linejoin="bevel" transform="matrix(1,0,0,1,1489,40)" font-family="MS Shell Dlg 2" font-size="9.75" font-weight="400" font-style="normal" > <path vector-effect="none" fill-rule="evenodd" d="M0,5 C0,2.23858 2.23858,0 5,0 L45,0 C47.7614,0 50,2.23858 50,5 L50,15 C50,17.7614 47.7614,20 45,20 L5,20 C2.23858,20 0,17.7614 0,15 L0,5"/> </g> <g fill="none" stroke="#000000" stroke-opacity="1" stroke-width="1" stroke-linecap="square" stroke-linejoin="bevel" transform="matrix(1,0,0,1,1498.5,38)" font-family="MS Shell Dlg 2" font-size="9.75" font-weight="400" font-style="normal" > <text fill="#000000" fill-opacity="1" stroke="none" xml:space="preserve" x="4" y="17" font-family="Arial" font-size="10pt" font-weight="700" font-style="italic" >rrnL</text> </g> <g fill="#ffff33" fill-opacity="1" stroke="#bfbfbf" stroke-opacity="1" stroke-width="1" stroke-linecap="square" stroke-linejoin="bevel" transform="matrix(1,0,0,1,1540,40)" font-family="MS Shell Dlg 2" font-size="9.75" font-weight="400" font-style="normal" > <rect x="0" y="0" width="50" height="20"/> </g> <g fill="none" stroke="#000000" stroke-opacity="1" stroke-width="1" stroke-linecap="square" stroke-linejoin="bevel" transform="matrix(1,0,0,1,1547,38)" font-family="MS Shell Dlg 2" font-size="9.75" font-weight="400" font-style="normal" > <text fill="#000000" fill-opacity="1" stroke="none" xml:space="preserve" x="4" y="17" font-family="Arial" font-size="10pt" font-weight="700" font-style="italic" >nad3</text> </g> <g fill="#ff9999" fill-opacity="1" stroke="#bfbfbf" stroke-opacity="1" stroke-width="1" stroke-linecap="square" stroke-linejoin="bevel" transform="matrix(1,0,0,1,1591,40)" font-family="MS Shell Dlg 2" font-size="9.75" font-weight="400" font-style="normal" > <path vector-effect="none" fill-rule="evenodd" d="M0,1.7 L42.5,1.7 L42.5,0 L50,10 L42.5,20 L42.5,18.3 L0,18.3 L0,1.7"/> </g> <g fill="none" stroke="#000000" stroke-opacity="1" stroke-width="1" stroke-linecap="square" stroke-linejoin="bevel" transform="matrix(1,0,0,1,1604.63,38)" font-family="MS Shell Dlg 2" font-size="9.75" font-weight="400" font-style="normal" > <text fill="#000000" fill-opacity="1" stroke="none" xml:space="preserve" x="4" y="17" font-family="Arial" font-size="10pt" font-weight="700" font-style="italic" >Y</text> </g> <g fill="#ff9999" fill-opacity="1" stroke="#bfbfbf" stroke-opacity="1" stroke-width="1" stroke-linecap="square" stroke-linejoin="bevel" transform="matrix(1,0,0,1,1642,40)" font-family="MS Shell Dlg 2" font-size="9.75" font-weight="400" font-style="normal" > <path vector-effect="none" fill-rule="evenodd" d="M0,1.7 L42.5,1.7 L42.5,0 L50,10 L42.5,20 L42.5,18.3 L0,18.3 L0,1.7"/> </g> <g fill="none" stroke="#000000" stroke-opacity="1" stroke-width="1" stroke-linecap="square" stroke-linejoin="bevel" transform="matrix(1,0,0,1,1655.13,38)" font-family="MS Shell

Dlg 2" font-size="9.75" font-weight="400" font-style="normal" > <text fill="#000000" fill-opacity="1" stroke="none" xml:space="preserve" x="4" y="17" font-family="Arial" font-size="10pt" font-weight="700" font-style="italic" >H</text> </g> <g fill="#ff9999" fill-opacity="1" stroke="#bfbfbf" stroke-opacity="1" stroke-width="1" stroke-linecap="square" stroke-linejoin="bevel" transform="matrix(1,0,0,1,1693,40)" font-family="MS Shell Dlg 2" font-size="9.75" font-weight="400" font-style="normal" > <path vector-effect="none" fill-rule="evenodd" d="M0,1.7 L42.5,1.7 L42.5,0 L50,10 L42.5,20 L42.5,18.3 L0,18.3 L0,1.7"/> </g> <g fill="none" stroke="#000000" stroke-opacity="1" stroke-width="1" stroke-linecap="square" stroke-linejoin="bevel" transform="matrix(1,0,0,1,1708.13,38)" font-family="MS Shell Dlg 2" font-size="9.75" font-weight="400" font-style="normal" > <text fill="#000000" fill-opacity="1" stroke="none" xml:space="preserve" x="4" y="17" font-family="Arial" font-size="10pt" font-weight="700" font-style="italic" >I</text> </g> <g fill="#ff9999" fill-opacity="1" stroke="#bfbfbf" stroke-opacity="1" stroke-width="1" stroke-linecap="square" stroke-linejoin="bevel" transform="matrix(1,0,0,1,1744,40)" font-family="MS Shell Dlg 2" font-size="9.75" font-weight="400" font-style="normal" > <path vector-effect="none" fill-rule="evenodd" d="M0,1.7 L42.5,1.7 L42.5,0 L50,10 L42.5,20 L42.5,18.3 L0,18.3 L0,1.7"/> </g> <g fill="none" stroke="#000000" stroke-opacity="1" stroke-width="1" stroke-linecap="square" stroke-linejoin="bevel" transform="matrix(1,0,0,1,1757.13,38)" font-family="MS Shell Dlg 2" font-size="9.75" font-weight="400" font-style="normal" > <text fill="#000000" fill-opacity="1" stroke="none" xml:space="preserve" x="4" y="17" font-family="Arial" font-size="10pt" font-weight="700" font-style="italic" >N</text> </g> <g fill="#ff9999" fill-opacity="1" stroke="#bfbfbf" stroke-opacity="1" stroke-width="1" stroke-linecap="square" stroke-linejoin="bevel" transform="matrix(1,0,0,1,1795,40)" font-family="MS Shell Dlg 2" font-size="9.75" font-weight="400" font-style="normal" > <path vector-effect="none" fill-rule="evenodd" d="M0,1.7 L42.5,1.7 L42.5,0 L50,10 L42.5,20 L42.5,18.3 L0,18.3 L0,1.7"/> </g> <g fill="none" stroke="#000000" stroke-opacity="1" stroke-width="1" stroke-linecap="square" stroke-linejoin="bevel" transform="matrix(1,0,0,1,1808.63,38)" font-family="MS Shell Dlg 2" font-size="9.75" font-weight="400" font-style="normal" > <text fill="#000000" fill-opacity="1" stroke="none" xml:space="preserve" x="4" y="17" font-family="Arial" font-size="10pt" font-weight="700" font-style="italic" >E</text> </g> <g fill="none" stroke="#000000" stroke-opacity="1" stroke-width="1" stroke-linecap="square" stroke-linejoin="bevel" transform="matrix(1,0,0,1,1846,40)" font-family="MS Shell Dlg 2" font-size="9.75" font-weight="400" font-style="normal" > <text fill="#000000" fill-opacity="1" stroke="none" xml:space="preserve" x="4" y="17" font-family="Arial" font-size="10pt" font-weight="700" font-style="italic" >(+)</text> </g> <g fill="none" stroke="#000000" stroke-opacity="1" stroke-width="1" stroke-linecap="square" stroke-linejoin="bevel" transform="matrix(1,0,0,1,1846,60)" font-family="MS Shell Dlg 2" font-size="9.75" font-weight="400" font-style="normal" > <text fill="#000000" fill-opacity="1" stroke="none" xml:space="preserve" x="4" y="17" font-family="Arial" font-size="10pt" font-weight="700" font-style="italic" >(-)</text> </g> <g fill="none" stroke="#000000" stroke-opacity="1" stroke-width="1" stroke-linecap="square" stroke-linejoin="bevel" transform="matrix(1,0,0,1,10,100)" font-family="MS Shell Dlg 2" font-size="9.75" font-weight="400" font-style="normal" > <text fill="#000000" fill-opacity="1" stroke="none" xml:space="preserve" x="4" y="17" font-family="Arial" font-size="10pt" font-weight="700" font-style="italic" >Arcuatula_senhousia_OR453539</text> </g> <g fill="#ffff33" fill-opacity="1" stroke="#bfbfbf" stroke-opacity="1" stroke-width="1" stroke-linecap="square" stroke-linejoin="bevel" transform="matrix(1,0,0,1,10,130)" font-family="MS Shell Dlg 2" font-size="9.75" font-weight="400" font-style="normal" > <rect x="0" y="0" width="50" height="20"/> </g> <g fill="none" stroke="#000000" stroke-opacity="1" stroke-width="1" stroke-linecap="square" stroke-linejoin="bevel" transform="matrix(1,0,0,1,18,128)" font-family="MS Shell Dlg 2" font-size="9.75" font-weight="400" font-style="normal" > <text fill="#000000" fill-opacity="1" stroke="none" xml:space="preserve" x="4" y="17" font-family="Arial" font-size="10pt" font-weight="700" font-style="italic" >cox1</text> </g> <g fill="#ffff33" fill-opacity="1" stroke="#bfbfbf" stroke-opacity="1" stroke-width="1" stroke-linecap="square" stroke-linejoin="bevel" transform="matrix(1,0,0,1,61,130)" font-family="MS Shell Dlg 2" font-size="9.75" font-weight="400" font-style="normal" > <rect x="0" y="0" width="50" height="20"/> </g> <g fill="none" stroke="#000000" stroke-opacity="1" stroke-width="1" stroke-linecap="square" stroke-linejoin="bevel" transform="matrix(1,0,0,1,69,128)" font-family="MS Shell Dlg 2" font-size="9.75" font-weight="400" font-style="normal" > <text fill="#000000" fill-opacity="1" stroke="none" xml:space="preserve" x="4" y="17" font-family="Arial" font-size="10pt" font-weight="700" font-style="italic" >cox2</text> </g> <g fill="#ffff33" fill-opacity="1" stroke="#bfbfbf" stroke-opacity="1" stroke-width="1" stroke-linecap="square" stroke-linejoin="bevel" transform="matrix(1,0,0,1,112,130)" font-family="MS Shell Dlg 2" font-size="9.75" font-weight="400" font-style="normal" > <rect x="0" y="0" width="50" height="20"/> </g> <g fill="none" stroke="#000000" stroke-opacity="1" stroke-width="1" stroke-linecap="square" stroke-linejoin="bevel" transform="matrix(1,0,0,1,120.5,128)" font-family="MS Shell Dlg 2" font-size="9.75" font-weight="400" font-style="normal" > <text fill="#000000" fill-opacity="1" stroke="none" xml:space="preserve" x="4" y="17" font-family="Arial" font-size="10pt" font-weight="700" font-style="italic" >atp6</text> </g> <g

fill="#ff9999" fill-opacity="1" stroke="#bfbfbf" stroke-opacity="1" stroke-width="1" stroke-linecap="square" stroke-linejoin="bevel" transform="matrix(1,0,0,1,163,130)" font-family="MS Shell Dlg 2" font-size="9.75" font-weight="400" font-style="normal" > <path vector-effect="none" fill-rule="evenodd" d="M0,1.7 L42.5,1.7 L42.5,0 L50,10 L42.5,20 L42.5,18.3 L0,18.3 L0,1.7"/> </g> <g fill="none" stroke="#000000" stroke-opacity="1" stroke-width="1" stroke-linecap="square" stroke-linejoin="bevel" transform="matrix(1,0,0,1,176.132,128)" font-family="MS Shell Dlg 2" font-size="9.75" font-weight="400" font-style="normal" > <text fill="#000000" fill-opacity="1" stroke="none" xml:space="preserve" x="4" y="17" font-family="Arial" font-size="10pt" font-weight="700" font-style="italic" >T</text> </g> <g fill="#ffff33" fill-opacity="1" stroke="#bfbfbf" stroke-opacity="1" stroke-width="1" stroke-linecap="square" stroke-linejoin="bevel" transform="matrix(1,0,0,1,214,130)" font-family="MS Shell Dlg 2" font-size="9.75" font-weight="400" font-style="normal" > <rect x="0" y="0" width="50" height="20"/> </g> <g fill="none" stroke="#000000" stroke-opacity="1" stroke-width="1" stroke-linecap="square" stroke-linejoin="bevel" transform="matrix(1,0,0,1,223.5,128)" font-family="MS Shell Dlg 2" font-size="9.75" font-weight="400" font-style="normal" > <text fill="#000000" fill-opacity="1" stroke="none" xml:space="preserve" x="4" y="17" font-family="Arial" font-size="10pt" font-weight="700" font-style="italic" >cytb</text> </g> <g fill="#ff9999" fill-opacity="1" stroke="#bfbfbf" stroke-opacity="1" stroke-width="1" stroke-linecap="square" stroke-linejoin="bevel" transform="matrix(1,0,0,1,265,130)" font-family="MS Shell Dlg 2" font-size="9.75" font-weight="400" font-style="normal" > <path vector-effect="none" fill-rule="evenodd" d="M0,1.7 L42.5,1.7 L42.5,0 L50,10 L42.5,20 L42.5,18.3 L0,18.3 L0,1.7"/> </g> <g fill="none" stroke="#000000" stroke-opacity="1" stroke-width="1" stroke-linecap="square" stroke-linejoin="bevel" transform="matrix(1,0,0,1,278.132,128)" font-family="MS Shell Dlg 2" font-size="9.75" font-weight="400" font-style="normal" > <text fill="#000000" fill-opacity="1" stroke="none" xml:space="preserve" x="4" y="17" font-family="Arial" font-size="10pt" font-weight="700" font-style="italic" >D</text> </g> <g fill="#ff9999" fill-opacity="1" stroke="#bfbfbf" stroke-opacity="1" stroke-width="1" stroke-linecap="square" stroke-linejoin="bevel" transform="matrix(1,0,0,1,316,130)" font-family="MS Shell Dlg 2" font-size="9.75" font-weight="400" font-style="normal" > <path vector-effect="none" fill-rule="evenodd" d="M0,1.7 L42.5,1.7 L42.5,0 L50,10 L42.5,20 L42.5,18.3 L0,18.3 L0,1.7"/> </g> <g fill="none" stroke="#000000" stroke-opacity="1" stroke-width="1" stroke-linecap="square" stroke-linejoin="bevel" transform="matrix(1,0,0,1,329.132,128)" font-family="MS Shell Dlg 2" font-size="9.75" font-weight="400" font-style="normal" > <text fill="#000000" fill-opacity="1" stroke="none" xml:space="preserve" x="4" y="17" font-family="Arial" font-size="10pt" font-weight="700" font-style="italic" >R</text> </g> <g fill="#ff9999" fill-opacity="1" stroke="#bfbfbf" stroke-opacity="1" stroke-width="1" stroke-linecap="square" stroke-linejoin="bevel" transform="matrix(1,0,0,1,367,130)" font-family="MS Shell Dlg 2" font-size="9.75" font-weight="400" font-style="normal" > <path vector-effect="none" fill-rule="evenodd" d="M0,1.7 L42.5,1.7 L42.5,0 L50,10 L42.5,20 L42.5,18.3 L0,18.3 L0,1.7"/> </g> <g fill="none" stroke="#000000" stroke-opacity="1" stroke-width="1" stroke-linecap="square" stroke-linejoin="bevel" transform="matrix(1,0,0,1,376.632,128)" font-family="MS Shell Dlg 2" font-size="9.75" font-weight="400" font-style="normal" > <text fill="#000000" fill-opacity="1" stroke="none" xml:space="preserve" x="4" y="17" font-family="Arial" font-size="10pt" font-weight="700" font-style="italic" >S1</text> </g> <g fill="#ff9999" fill-opacity="1" stroke="#bfbfbf" stroke-opacity="1" stroke-width="1" stroke-linecap="square" stroke-linejoin="bevel" transform="matrix(1,0,0,1,418,130)" font-family="MS Shell Dlg 2" font-size="9.75" font-weight="400" font-style="normal" > <path vector-effect="none" fill-rule="evenodd" d="M0,1.7 L42.5,1.7 L42.5,0 L50,10 L42.5,20 L42.5,18.3 L0,18.3 L0,1.7"/> </g> <g fill="none" stroke="#000000" stroke-opacity="1" stroke-width="1" stroke-linecap="square" stroke-linejoin="bevel" transform="matrix(1,0,0,1,431.132,128)" font-family="MS Shell Dlg 2" font-size="9.75" font-weight="400" font-style="normal" > <text fill="#000000" fill-opacity="1" stroke="none" xml:space="preserve" x="4" y="17" font-family="Arial" font-size="10pt" font-weight="700" font-style="italic" >G</text> </g> <g fill="#99ffff" fill-opacity="1" stroke="#bfbfbf" stroke-opacity="1" stroke-width="1" stroke-linecap="square" stroke-linejoin="bevel" transform="matrix(1,0,0,1,469,130)" font-family="MS Shell Dlg 2" font-size="9.75" font-weight="400" font-style="normal" > <path vector-effect="none" fill-rule="evenodd" d="M0,5 C0,2.23858 2.23858,0 5,0 L45,0 C47.7614,0 50,2.23858 50,5 L50,15 C50,17.7614 47.7614,20 45,20 L5,20 C2.23858,20 0,17.7614 0,15 L0,5"/> </g> <g fill="none" stroke="#000000" stroke-opacity="1" stroke-width="1" stroke-linecap="square" stroke-linejoin="bevel" transform="matrix(1,0,0,1,477.5,128)" font-family="MS Shell Dlg 2" font-size="9.75" font-weight="400" font-style="normal" > <text fill="#000000" fill-opacity="1" stroke="none" xml:space="preserve" x="4" y="17" font-family="Arial" font-size="10pt" font-weight="700" font-style="italic" >rrnS</text> </g> <g fill="#ff9999" fill-opacity="1" stroke="#bfbfbf" stroke-opacity="1" stroke-width="1" stroke-linecap="square" stroke-linejoin="bevel" transform="matrix(1,0,0,1,520,130)" font-family="MS Shell Dlg 2" font-size="9.75" font-weight="400" font-style="normal" > <path vector-effect="none" fill-rule="evenodd" d="M0,1.7 L42.5,1.7 L42.5,0 L50,10 L42.5,20 L42.5,18.3 L0,18.3 L0,1.7"/> </g> <g fill="none" stroke="#000000" stroke-opacity="1"

stroke-width="1" stroke-linecap="square" stroke-linejoin="bevel" transform="matrix(1,0,0,1,529.633,128)" font-family="MS Shell Dlg 2" font-size="9.75" font-weight="400" font-style="normal" > <text fill="#000000" fill-opacity="1" stroke="none" xml:space="preserve" x="4" y="17" font-family="Arial" font-size="10pt" font-weight="700" font-style="italic" >S2</text> </g> <g fill="#ffff33" fill-opacity="1" stroke="#bfbfbf" stroke-opacity="1" stroke-width="1" stroke-linecap="square" stroke-linejoin="bevel" transform="matrix(1,0,0,1,571,130)" font-family="MS Shell Dlg 2" font-size="9.75" font-weight="400" font-style="normal" > <rect x="0" y="0" width="50" height="20"/> </g> <g fill="none" stroke="#000000" stroke-opacity="1" stroke-width="1" stroke-linecap="square" stroke-linejoin="bevel" transform="matrix(1,0,0,1,578,128)" font-family="MS Shell Dlg 2" font-size="9.75" font-weight="400" font-style="normal" > <text fill="#000000" fill-opacity="1" stroke="none" xml:space="preserve" x="4" y="17" font-family="Arial" font-size="10pt" font-weight="700" font-style="italic" >nad6</text> </g> <g fill="#ffff33" fill-opacity="1" stroke="#bfbfbf" stroke-opacity="1" stroke-width="1" stroke-linecap="square" stroke-linejoin="bevel" transform="matrix(1,0,0,1,622,130)" font-family="MS Shell Dlg 2" font-size="9.75" font-weight="400" font-style="normal" > <rect x="0" y="0" width="50" height="20"/> </g> <g fill="none" stroke="#000000" stroke-opacity="1" stroke-width="1" stroke-linecap="square" stroke-linejoin="bevel" transform="matrix(1,0,0,1,629,128)" font-family="MS Shell Dlg 2" font-size="9.75" font-weight="400" font-style="normal" > <text fill="#000000" fill-opacity="1" stroke="none" xml:space="preserve" x="4" y="17" font-family="Arial" font-size="10pt" font-weight="700" font-style="italic" >nad2</text> </g> <g fill="#ffff33" fill-opacity="1" stroke="#bfbfbf" stroke-opacity="1" stroke-width="1" stroke-linecap="square" stroke-linejoin="bevel" transform="matrix(1,0,0,1,673,130)" font-family="MS Shell Dlg 2" font-size="9.75" font-weight="400" font-style="normal" > <rect x="0" y="0" width="50" height="20"/> </g> <g fill="none" stroke="#000000" stroke-opacity="1" stroke-width="1" stroke-linecap="square" stroke-linejoin="bevel" transform="matrix(1,0,0,1,681,128)" font-family="MS Shell Dlg 2" font-size="9.75" font-weight="400" font-style="normal" > <text fill="#000000" fill-opacity="1" stroke="none" xml:space="preserve" x="4" y="17" font-family="Arial" font-size="10pt" font-weight="700" font-style="italic" >cox3</text> </g> <g fill="#ff9999" fill-opacity="1" stroke="#bfbfbf" stroke-opacity="1" stroke-width="1" stroke-linecap="square" stroke-linejoin="bevel" transform="matrix(1,0,0,1,724,130)" font-family="MS Shell Dlg 2" font-size="9.75" font-weight="400" font-style="normal" > <path vector-effect="none" fill-rule="evenodd" d="M0,1.7 L42.5,1.7 L42.5,0 L50,10 L42.5,20 L42.5,18.3 L0,18.3 L0,1.7"/> </g> <g fill="none" stroke="#000000" stroke-opacity="1" stroke-width="1" stroke-linecap="square" stroke-linejoin="bevel" transform="matrix(1,0,0,1,737.633,128)" font-family="MS Shell Dlg 2" font-size="9.75" font-weight="400" font-style="normal" > <text fill="#000000" fill-opacity="1" stroke="none" xml:space="preserve" x="4" y="17" font-family="Arial" font-size="10pt" font-weight="700" font-style="italic" >K</text> </g> <g fill="#ff9999" fill-opacity="1" stroke="#bfbfbf" stroke-opacity="1" stroke-width="1" stroke-linecap="square" stroke-linejoin="bevel" transform="matrix(1,0,0,1,775,130)" font-family="MS Shell Dlg 2" font-size="9.75" font-weight="400" font-style="normal" > <path vector-effect="none" fill-rule="evenodd" d="M0,1.7 L42.5,1.7 L42.5,0 L50,10 L42.5,20 L42.5,18.3 L0,18.3 L0,1.7"/> </g> <g fill="none" stroke="#000000" stroke-opacity="1" stroke-width="1" stroke-linecap="square" stroke-linejoin="bevel" transform="matrix(1,0,0,1,788.633,128)" font-family="MS Shell Dlg 2" font-size="9.75" font-weight="400" font-style="normal" > <text fill="#000000" fill-opacity="1" stroke="none" xml:space="preserve" x="4" y="17" font-family="Arial" font-size="10pt" font-weight="700" font-style="italic" >F</text> </g> <g fill="#ff9999" fill-opacity="1" stroke="#bfbfbf" stroke-opacity="1" stroke-width="1" stroke-linecap="square" stroke-linejoin="bevel" transform="matrix(1,0,0,1,826,130)" font-family="MS Shell Dlg 2" font-size="9.75" font-weight="400" font-style="normal" > <path vector-effect="none" fill-rule="evenodd" d="M0,1.7 L42.5,1.7 L42.5,0 L50,10 L42.5,20 L42.5,18.3 L0,18.3 L0,1.7"/> </g> <g fill="none" stroke="#000000" stroke-opacity="1" stroke-width="1" stroke-linecap="square" stroke-linejoin="bevel" transform="matrix(1,0,0,1,839.633,128)" font-family="MS Shell Dlg 2" font-size="9.75" font-weight="400" font-style="normal" > <text fill="#000000" fill-opacity="1" stroke="none" xml:space="preserve" x="4" y="17" font-family="Arial" font-size="10pt" font-weight="700" font-style="italic" >P</text> </g> <g fill="#ff9999" fill-opacity="1" stroke="#bfbfbf" stroke-opacity="1" stroke-width="1" stroke-linecap="square" stroke-linejoin="bevel" transform="matrix(1,0,0,1,877,130)" font-family="MS Shell Dlg 2" font-size="9.75" font-weight="400" font-style="normal" > <path vector-effect="none" fill-rule="evenodd" d="M0,1.7 L42.5,1.7 L42.5,0 L50,10 L42.5,20 L42.5,18.3 L0,18.3 L0,1.7"/> </g> <g fill="none" stroke="#000000" stroke-opacity="1" stroke-width="1" stroke-linecap="square" stroke-linejoin="bevel" transform="matrix(1,0,0,1,887.633,128)" font-family="MS Shell Dlg 2" font-size="9.75" font-weight="400" font-style="normal" > <text fill="#000000" fill-opacity="1" stroke="none" xml:space="preserve" x="4" y="17" font-family="Arial" font-size="10pt" font-weight="700" font-style="italic" >L1</text> </g> <g fill="#ff9999" fill-opacity="1" stroke="#bfbfbf" stroke-opacity="1" stroke-width="1" stroke-linecap="square" stroke-linejoin="bevel" transform="matrix(1,0,0,1,928,130)" font-family="MS Shell Dlg 2" font-size="9.75" font-weight="400" font-style="normal" > <path vector-effect="none"

fill-rule="evenodd" d="M0,1.7 L42.5,1.7 L42.5,0 L50,10 L42.5,20 L42.5,18.3 L0,18.3 L0,1.7"/> </g> <g fill="none" stroke="#000000" stroke-opacity="1" stroke-width="1" stroke-linecap="square" stroke-linejoin="bevel" transform="matrix(1,0,0,1,941.133,128)" font-family="MS Shell Dlg 2" font-size="9.75" font-weight="400" font-style="normal" > <text fill="#000000" fill-opacity="1" stroke="none" xml:space="preserve" x="4" y="17" font-family="Arial" font-size="10pt" font-weight="700" font-style="italic" >C</text> </g> <g fill="#ff9999" fill-opacity="1" stroke="#bfbfbf" stroke-opacity="1" stroke-width="1" stroke-linecap="square" stroke-linejoin="bevel" transform="matrix(1,0,0,1,979,130)" font-family="MS Shell Dlg 2" font-size="9.75" font-weight="400" font-style="normal" > <path vector-effect="none" fill-rule="evenodd" d="M0,1.7 L42.5,1.7 L42.5,0 L50,10 L42.5,20 L42.5,18.3 L0,18.3 L0,1.7"/> </g> <g fill="none" stroke="#000000" stroke-opacity="1" stroke-width="1" stroke-linecap="square" stroke-linejoin="bevel" transform="matrix(1,0,0,1,989.633,128)" font-family="MS Shell Dlg 2" font-size="9.75" font-weight="400" font-style="normal" > <text fill="#000000" fill-opacity="1" stroke="none" xml:space="preserve" x="4" y="17" font-family="Arial" font-size="10pt" font-weight="700" font-style="italic" >L2</text> </g> <g fill="#ffff33" fill-opacity="1" stroke="#bfbfbf" stroke-opacity="1" stroke-width="1" stroke-linecap="square" stroke-linejoin="bevel" transform="matrix(1,0,0,1,1030,130)" font-family="MS Shell Dlg 2" font-size="9.75" font-weight="400" font-style="normal" > <rect x="0" y="0" width="50" height="20"/> </g> <g fill="none" stroke="#000000" stroke-opacity="1" stroke-width="1" stroke-linecap="square" stroke-linejoin="bevel" transform="matrix(1,0,0,1,1037,128)" font-family="MS Shell Dlg 2" font-size="9.75" font-weight="400" font-style="normal" > <text fill="#000000" fill-opacity="1" stroke="none" xml:space="preserve" x="4" y="17" font-family="Arial" font-size="10pt" font-weight="700" font-style="italic" >nad1</text> </g> <g fill="#ff9999" fill-opacity="1" stroke="#bfbfbf" stroke-opacity="1" stroke-width="1" stroke-linecap="square" stroke-linejoin="bevel" transform="matrix(1,0,0,1,1081,130)" font-family="MS Shell Dlg 2" font-size="9.75" font-weight="400" font-style="normal" > <path vector-effect="none" fill-rule="evenodd" d="M0,1.7 L42.5,1.7 L42.5,0 L50,10 L42.5,20 L42.5,18.3 L0,18.3 L0,1.7"/> </g> <g fill="none" stroke="#000000" stroke-opacity="1" stroke-width="1" stroke-linecap="square" stroke-linejoin="bevel" transform="matrix(1,0,0,1,1093.13,128)" font-family="MS Shell Dlg 2" font-size="9.75" font-weight="400" font-style="normal" > <text fill="#000000" fill-opacity="1" stroke="none" xml:space="preserve" x="4" y="17" font-family="Arial" font-size="10pt" font-weight="700" font-style="italic" >M</text> </g> <g fill="#ff9999" fill-opacity="1" stroke="#bfbfbf" stroke-opacity="1" stroke-width="1" stroke-linecap="square" stroke-linejoin="bevel" transform="matrix(1,0,0,1,1132,130)" font-family="MS Shell Dlg 2" font-size="9.75" font-weight="400" font-style="normal" > <path vector-effect="none" fill-rule="evenodd" d="M0,1.7 L42.5,1.7 L42.5,0 L50,10 L42.5,20 L42.5,18.3 L0,18.3 L0,1.7"/> </g> <g fill="none" stroke="#000000" stroke-opacity="1" stroke-width="1" stroke-linecap="square" stroke-linejoin="bevel" transform="matrix(1,0,0,1,1145.13,128)" font-family="MS Shell Dlg 2" font-size="9.75" font-weight="400" font-style="normal" > <text fill="#000000" fill-opacity="1" stroke="none" xml:space="preserve" x="4" y="17" font-family="Arial" font-size="10pt" font-weight="700" font-style="italic" >V</text> </g> <g fill="#ffff33" fill-opacity="1" stroke="#bfbfbf" stroke-opacity="1" stroke-width="1" stroke-linecap="square" stroke-linejoin="bevel" transform="matrix(1,0,0,1,1183,130)" font-family="MS Shell Dlg 2" font-size="9.75" font-weight="400" font-style="normal" > <rect x="0" y="0" width="50" height="20"/> </g> <g fill="none" stroke="#000000" stroke-opacity="1" stroke-width="1" stroke-linecap="square" stroke-linejoin="bevel" transform="matrix(1,0,0,1,1187,128)" font-family="MS Shell Dlg 2" font-size="9.75" font-weight="400" font-style="normal" > <text fill="#000000" fill-opacity="1" stroke="none" xml:space="preserve" x="4" y="17" font-family="Arial" font-size="10pt" font-weight="700" font-style="italic" >nad4L</text> </g> <g fill="#ffff33" fill-opacity="1" stroke="#bfbfbf" stroke-opacity="1" stroke-width="1" stroke-linecap="square" stroke-linejoin="bevel" transform="matrix(1,0,0,1,1234,130)" font-family="MS Shell Dlg 2" font-size="9.75" font-weight="400" font-style="normal" > <rect x="0" y="0" width="50" height="20"/> </g> <g fill="none" stroke="#000000" stroke-opacity="1" stroke-width="1" stroke-linecap="square" stroke-linejoin="bevel" transform="matrix(1,0,0,1,1241,128)" font-family="MS Shell Dlg 2" font-size="9.75" font-weight="400" font-style="normal" > <text fill="#000000" fill-opacity="1" stroke="none" xml:space="preserve" x="4" y="17" font-family="Arial" font-size="10pt" font-weight="700" font-style="italic" >nad5</text> </g> <g fill="#ff9999" fill-opacity="1" stroke="#bfbfbf" stroke-opacity="1" stroke-width="1" stroke-linecap="square" stroke-linejoin="bevel" transform="matrix(1,0,0,1,1285,130)" font-family="MS Shell Dlg 2" font-size="9.75" font-weight="400" font-style="normal" > <path vector-effect="none" fill-rule="evenodd" d="M0,1.7 L42.5,1.7 L42.5,0 L50,10 L42.5,20 L42.5,18.3 L0,18.3 L0,1.7"/> </g> <g fill="none" stroke="#000000" stroke-opacity="1" stroke-width="1" stroke-linecap="square" stroke-linejoin="bevel" transform="matrix(1,0,0,1,1298.13,128)" font-family="MS Shell Dlg 2" font-size="9.75" font-weight="400" font-style="normal" > <text fill="#000000" fill-opacity="1" stroke="none" xml:space="preserve" x="4" y="17" font-family="Arial" font-size="10pt" font-weight="700" font-style="italic" >A</text> </g> <g fill="#ffff33" fill-opacity="1" stroke="#bfbfbf" stroke-opacity="1" stroke-width="1" stroke-linecap="square" stroke-

linejoin="bevel" transform="matrix(1,0,0,1,1336,130)" font-family="MS Shell Dlg 2" font-size="9.75" font-weight="400" font-style="normal" > <rect x="0" y="0" width="50" height="20"/> </g> <g fill="none" stroke="#000000" stroke-opacity="1" stroke-width="1" stroke-linecap="square" stroke-linejoin="bevel" transform="matrix(1,0,0,1,1343,128)" font-family="MS Shell Dlg 2" font-size="9.75" font-weight="400" font-style="normal" > <text fill="#000000" fill-opacity="1" stroke="none" xml:space="preserve" x="4" y="17" font-family="Arial" font-size="10pt" font-weight="700" font-style="italic" >nad4</text> </g> <g fill="#ff9999" fill-opacity="1" stroke="#bfbfbf" stroke-opacity="1" stroke-width="1" stroke-linecap="square" stroke-linejoin="bevel" transform="matrix(1,0,0,1,1387,130)" font-family="MS Shell Dlg 2" font-size="9.75" font-weight="400" font-style="normal" > <path vector-effect="none" fill-rule="evenodd" d="M0,1.7 L42.5,1.7 L42.5,0 L50,10 L42.5,20 L42.5,18.3 L0,18.3 L0,1.7"/> </g> <g fill="none" stroke="#000000" stroke-opacity="1" stroke-width="1" stroke-linecap="square" stroke-linejoin="bevel" transform="matrix(1,0,0,1,1398.13,128)" font-family="MS Shell Dlg 2" font-size="9.75" font-weight="400" font-style="normal" > <text fill="#000000" fill-opacity="1" stroke="none" xml:space="preserve" x="4" y="17" font-family="Arial" font-size="10pt" font-weight="700" font-style="italic" >W</text> </g> <g fill="#ff9999" fill-opacity="1" stroke="#bfbfbf" stroke-opacity="1" stroke-width="1" stroke-linecap="square" stroke-linejoin="bevel" transform="matrix(1,0,0,1,1438,130)" font-family="MS Shell Dlg 2" font-size="9.75" font-weight="400" font-style="normal" > <path vector-effect="none" fill-rule="evenodd" d="M0,1.7 L42.5,1.7 L42.5,0 L50,10 L42.5,20 L42.5,18.3 L0,18.3 L0,1.7"/> </g> <g fill="none" stroke="#000000" stroke-opacity="1" stroke-width="1" stroke-linecap="square" stroke-linejoin="bevel" transform="matrix(1,0,0,1,1450.63,128)" font-family="MS Shell Dlg 2" font-size="9.75" font-weight="400" font-style="normal" > <text fill="#000000" fill-opacity="1" stroke="none" xml:space="preserve" x="4" y="17" font-family="Arial" font-size="10pt" font-weight="700" font-style="italic" >Q</text> </g> <g fill="#ff9999" fill-opacity="1" stroke="#bfbfbf" stroke-opacity="1" stroke-width="1" stroke-linecap="square" stroke-linejoin="bevel" transform="matrix(1,0,0,1,1489,130)" font-family="MS Shell Dlg 2" font-size="9.75" font-weight="400" font-style="normal" > <path vector-effect="none" fill-rule="evenodd" d="M0,1.7 L42.5,1.7 L42.5,0 L50,10 L42.5,20 L42.5,18.3 L0,18.3 L0,1.7"/> </g> <g fill="none" stroke="#000000" stroke-opacity="1" stroke-width="1" stroke-linecap="square" stroke-linejoin="bevel" transform="matrix(1,0,0,1,1501.13,128)" font-family="MS Shell Dlg 2" font-size="9.75" font-weight="400" font-style="normal" > <text fill="#000000" fill-opacity="1" stroke="none" xml:space="preserve" x="4" y="17" font-family="Arial" font-size="10pt" font-weight="700" font-style="italic" >M</text> </g> <g fill="#99ffff" fill-opacity="1" stroke="#bfbfbf" stroke-opacity="1" stroke-width="1" stroke-linecap="square" stroke-linejoin="bevel" transform="matrix(1,0,0,1,1540,130)" font-family="MS Shell Dlg 2" font-size="9.75" font-weight="400" font-style="normal" > <path vector-effect="none" fill-rule="evenodd" d="M0,5 C0,2.23858 2.23858,0 5,0 L45,0 C47.7614,0 50,2.23858 50,5 L50,15 C50,17.7614 47.7614,20 45,20 L5,20 C2.23858,20 0,17.7614 0,15 L0,5"/> </g> <g fill="none" stroke="#000000" stroke-opacity="1" stroke-width="1" stroke-linecap="square" stroke-linejoin="bevel" transform="matrix(1,0,0,1,1549.5,128)" font-family="MS Shell Dlg 2" font-size="9.75" font-weight="400" font-style="normal" > <text fill="#000000" fill-opacity="1" stroke="none" xml:space="preserve" x="4" y="17" font-family="Arial" font-size="10pt" font-weight="700" font-style="italic" >rrnL</text> </g> <g fill="#ffff33" fill-opacity="1" stroke="#bfbfbf" stroke-opacity="1" stroke-width="1" stroke-linecap="square" stroke-linejoin="bevel" transform="matrix(1,0,0,1,1591,130)" font-family="MS Shell Dlg 2" font-size="9.75" font-weight="400" font-style="normal" > <rect x="0" y="0" width="50" height="20"/> </g> <g fill="none" stroke="#000000" stroke-opacity="1" stroke-width="1" stroke-linecap="square" stroke-linejoin="bevel" transform="matrix(1,0,0,1,1598,128)" font-family="MS Shell Dlg 2" font-size="9.75" font-weight="400" font-style="normal" > <text fill="#000000" fill-opacity="1" stroke="none" xml:space="preserve" x="4" y="17" font-family="Arial" font-size="10pt" font-weight="700" font-style="italic" >nad3</text> </g> <g fill="#ff9999" fill-opacity="1" stroke="#bfbfbf" stroke-opacity="1" stroke-width="1" stroke-linecap="square" stroke-linejoin="bevel" transform="matrix(1,0,0,1,1642,130)" font-family="MS Shell Dlg 2" font-size="9.75" font-weight="400" font-style="normal" > <path vector-effect="none" fill-rule="evenodd" d="M0,1.7 L42.5,1.7 L42.5,0 L50,10 L42.5,20 L42.5,18.3 L0,18.3 L0,1.7"/> </g> <g fill="none" stroke="#000000" stroke-opacity="1" stroke-width="1" stroke-linecap="square" stroke-linejoin="bevel" transform="matrix(1,0,0,1,1655.63,128)" font-family="MS Shell Dlg 2" font-size="9.75" font-weight="400" font-style="normal" > <text fill="#000000" fill-opacity="1" stroke="none" xml:space="preserve" x="4" y="17" font-family="Arial" font-size="10pt" font-weight="700" font-style="italic" >Y</text> </g> <g fill="#ff9999" fill-opacity="1" stroke="#bfbfbf" stroke-opacity="1" stroke-width="1" stroke-linecap="square" stroke-linejoin="bevel" transform="matrix(1,0,0,1,1693,130)" font-family="MS Shell Dlg 2" font-size="9.75" font-weight="400" font-style="normal" > <path vector-effect="none" fill-rule="evenodd" d="M0,1.7 L42.5,1.7 L42.5,0 L50,10 L42.5,20 L42.5,18.3 L0,18.3 L0,1.7"/> </g> <g fill="none" stroke="#000000" stroke-opacity="1" stroke-width="1" stroke-linecap="square" stroke-linejoin="bevel" transform="matrix(1,0,0,1,1706.13,128)" font-family="MS Shell Dlg 2" font-size="9.75" font-weight="400" font-style="normal" > <text fill="#000000" fill-opacity="1"

stroke="none" xml:space="preserve" x="4" y="17" font-family="Arial" font-size="10pt" font-weight="700" font-style="italic" >H</text> </g> <g fill="#ff9999" fill-opacity="1" stroke="#bfbfbf" stroke-opacity="1" stroke-width="1" stroke-linecap="square" stroke-linejoin="bevel" transform="matrix(1,0,0,1,1744,130)" font-family="MS Shell Dlg 2" font-size="9.75" font-weight="400" font-style="normal" > <path vector-effect="none" fill-rule="evenodd" d="M0,1.7 L42.5,1.7 L42.5,0 L50,10 L42.5,20 L42.5,18.3 L0,18.3 L0,1.7"/> </g> <g fill="none" stroke="#000000" stroke-opacity="1" stroke-width="1" stroke-linecap="square" stroke-linejoin="bevel" transform="matrix(1,0,0,1,1759.13,128)" font-family="MS Shell Dlg 2" font-size="9.75" font-weight="400" font-style="normal" > <text fill="#000000" fill-opacity="1" stroke="none" xml:space="preserve" x="4" y="17" font-family="Arial" font-size="10pt" font-weight="700" font-style="italic" >I</text> </g> <g fill="#ff9999" fill-opacity="1" stroke="#bfbfbf" stroke-opacity="1" stroke-width="1" stroke-linecap="square" stroke-linejoin="bevel" transform="matrix(1,0,0,1,1795,130)" font-family="MS Shell Dlg 2" font-size="9.75" font-weight="400" font-style="normal" > <path vector-effect="none" fill-rule="evenodd" d="M0,1.7 L42.5,1.7 L42.5,0 L50,10 L42.5,20 L42.5,18.3 L0,18.3 L0,1.7"/> </g> <g fill="none" stroke="#000000" stroke-opacity="1" stroke-width="1" stroke-linecap="square" stroke-linejoin="bevel" transform="matrix(1,0,0,1,1808.13,128)" font-family="MS Shell Dlg 2" font-size="9.75" font-weight="400" font-style="normal" > <text fill="#000000" fill-opacity="1" stroke="none" xml:space="preserve" x="4" y="17" font-family="Arial" font-size="10pt" font-weight="700" font-style="italic" >N</text> </g> <g fill="#ff9999" fill-opacity="1" stroke="#bfbfbf" stroke-opacity="1" stroke-width="1" stroke-linecap="square" stroke-linejoin="bevel" transform="matrix(1,0,0,1,1846,130)" font-family="MS Shell Dlg 2" font-size="9.75" font-weight="400" font-style="normal" > <path vector-effect="none" fill-rule="evenodd" d="M0,1.7 L42.5,1.7 L42.5,0 L50,10 L42.5,20 L42.5,18.3 L0,18.3 L0,1.7"/> </g> <g fill="none" stroke="#000000" stroke-opacity="1" stroke-width="1" stroke-linecap="square" stroke-linejoin="bevel" transform="matrix(1,0,0,1,1859.63,128)" font-family="MS Shell Dlg 2" font-size="9.75" font-weight="400" font-style="normal" > <text fill="#000000" fill-opacity="1" stroke="none" xml:space="preserve" x="4" y="17" font-family="Arial" font-size="10pt" font-weight="700" font-style="italic" >E</text> </g> <g fill="none" stroke="#000000" stroke-opacity="1" stroke-width="1" stroke-linecap="square" stroke-linejoin="bevel" transform="matrix(1,0,0,1,1897,130)" font-family="MS Shell Dlg 2" font-size="9.75" font-weight="400" font-style="normal" > <text fill="#000000" fill-opacity="1" stroke="none" xml:space="preserve" x="4" y="17" font-family="Arial" font-size="10pt" font-weight="700" font-style="italic" >(+)</text> </g> <g fill="none" stroke="#000000" stroke-opacity="1" stroke-width="1" stroke-linecap="square" stroke-linejoin="bevel" transform="matrix(1,0,0,1,10,170)" font-family="MS Shell Dlg 2" font-size="9.75" font-weight="400" font-style="normal" > <text fill="#000000" fill-opacity="1" stroke="none" xml:space="preserve" x="4" y="17" font-family="Arial" font-size="10pt" font-weight="700" font-style="italic" >Mytilus_chilensis_KP100300</text> </g> <g fill="#ffff33" fill-opacity="1" stroke="#bfbfbf" stroke-opacity="1" stroke-width="1" stroke-linecap="square" stroke-linejoin="bevel" transform="matrix(1,0,0,1,10,200)" font-family="MS Shell Dlg 2" font-size="9.75" font-weight="400" font-style="normal" > <rect x="0" y="0" width="50" height="20"/> </g> <g fill="none" stroke="#000000" stroke-opacity="1" stroke-width="1" stroke-linecap="square" stroke-linejoin="bevel" transform="matrix(1,0,0,1,18,198)" font-family="MS Shell Dlg 2" font-size="9.75" font-weight="400" font-style="normal" > <text fill="#000000" fill-opacity="1" stroke="none" xml:space="preserve" x="4" y="17" font-family="Arial" font-size="10pt" font-weight="700" font-style="italic" >cox1</text> </g> <g fill="#ffff33" fill-opacity="1" stroke="#bfbfbf" stroke-opacity="1" stroke-width="1" stroke-linecap="square" stroke-linejoin="bevel" transform="matrix(1,0,0,1,61,200)" font-family="MS Shell Dlg 2" font-size="9.75" font-weight="400" font-style="normal" > <rect x="0" y="0" width="50" height="20"/> </g> <g fill="none" stroke="#000000" stroke-opacity="1" stroke-width="1" stroke-linecap="square" stroke-linejoin="bevel" transform="matrix(1,0,0,1,69.5,198)" font-family="MS Shell Dlg 2" font-size="9.75" font-weight="400" font-style="normal" > <text fill="#000000" fill-opacity="1" stroke="none" xml:space="preserve" x="4" y="17" font-family="Arial" font-size="10pt" font-weight="700" font-style="italic" >atp6</text> </g> <g fill="#ff9999" fill-opacity="1" stroke="#bfbfbf" stroke-opacity="1" stroke-width="1" stroke-linecap="square" stroke-linejoin="bevel" transform="matrix(1,0,0,1,112,200)" font-family="MS Shell Dlg 2" font-size="9.75" font-weight="400" font-style="normal" > <path vector-effect="none" fill-rule="evenodd" d="M0,1.7 L42.5,1.7 L42.5,0 L50,10 L42.5,20 L42.5,18.3 L0,18.3 L0,1.7"/> </g> <g fill="none" stroke="#000000" stroke-opacity="1" stroke-width="1" stroke-linecap="square" stroke-linejoin="bevel" transform="matrix(1,0,0,1,125.132,198)" font-family="MS Shell Dlg 2" font-size="9.75" font-weight="400" font-style="normal" > <text fill="#000000" fill-opacity="1" stroke="none" xml:space="preserve" x="4" y="17" font-family="Arial" font-size="10pt" font-weight="700" font-style="italic" >T</text> </g> <g fill="#ffff33" fill-opacity="1" stroke="#bfbfbf" stroke-opacity="1" stroke-width="1" stroke-linecap="square" stroke-linejoin="bevel" transform="matrix(1,0,0,1,163,200)" font-family="MS Shell Dlg 2" font-size="9.75" font-weight="400" font-style="normal" > <rect x="0" y="0" width="50" height="20"/> </g> <g fill="none" stroke="#000000" stroke-opacity="1" stroke-width="1" stroke-linecap="square" stroke-linejoin="bevel" transform="matrix(1,0,0,1,167,198)"

font-family="MS Shell Dlg 2" font-size="9.75" font-weight="400" font-style="normal" > <text fill="#000000" fill-opacity="1" stroke="none" xml:space="preserve" x="4" y="17" font-family="Arial" font-size="10pt" font-weight="700" font-style="italic" >nad4L</text> </g> <g fill="#ffff33" fill-opacity="1" stroke="#bfbfbf" stroke-opacity="1" stroke-width="1" stroke-linecap="square" stroke-linejoin="bevel" transform="matrix(1,0,0,1,214,200)" font-family="MS Shell Dlg 2" font-size="9.75" font-weight="400" font-style="normal" > <rect x="0" y="0" width="50" height="20"/> </g> <g fill="none" stroke="#000000" stroke-opacity="1" stroke-width="1" stroke-linecap="square" stroke-linejoin="bevel" transform="matrix(1,0,0,1,221,198)" font-family="MS Shell Dlg 2" font-size="9.75" font-weight="400" font-style="normal" > <text fill="#000000" fill-opacity="1" stroke="none" xml:space="preserve" x="4" y="17" font-family="Arial" font-size="10pt" font-weight="700" font-style="italic" >nad5</text> </g> <g fill="#ffff33" fill-opacity="1" stroke="#bfbfbf" stroke-opacity="1" stroke-width="1" stroke-linecap="square" stroke-linejoin="bevel" transform="matrix(1,0,0,1,265,200)" font-family="MS Shell Dlg 2" font-size="9.75" font-weight="400" font-style="normal" > <rect x="0" y="0" width="50" height="20"/> </g> <g fill="none" stroke="#000000" stroke-opacity="1" stroke-width="1" stroke-linecap="square" stroke-linejoin="bevel" transform="matrix(1,0,0,1,272,198)" font-family="MS Shell Dlg 2" font-size="9.75" font-weight="400" font-style="normal" > <text fill="#000000" fill-opacity="1" stroke="none" xml:space="preserve" x="4" y="17" font-family="Arial" font-size="10pt" font-weight="700" font-style="italic" >nad6</text> </g> <g fill="#ff9999" fill-opacity="1" stroke="#bfbfbf" stroke-opacity="1" stroke-width="1" stroke-linecap="square" stroke-linejoin="bevel" transform="matrix(1,0,0,1,316,200)" font-family="MS Shell Dlg 2" font-size="9.75" font-weight="400" font-style="normal" > <path vector-effect="none" fill-rule="evenodd" d="M0,1.7 L42.5,1.7 L42.5,0 L50,10 L42.5,20 L42.5,18.3 L0,18.3 L0,1.7"/> </g> <g fill="none" stroke="#000000" stroke-opacity="1" stroke-width="1" stroke-linecap="square" stroke-linejoin="bevel" transform="matrix(1,0,0,1,329.632,198)" font-family="MS Shell Dlg 2" font-size="9.75" font-weight="400" font-style="normal" > <text fill="#000000" fill-opacity="1" stroke="none" xml:space="preserve" x="4" y="17" font-family="Arial" font-size="10pt" font-weight="700" font-style="italic" >F</text> </g> <g fill="#99ffff" fill-opacity="1" stroke="#bfbfbf" stroke-opacity="1" stroke-width="1" stroke-linecap="square" stroke-linejoin="bevel" transform="matrix(1,0,0,1,367,200)" font-family="MS Shell Dlg 2" font-size="9.75" font-weight="400" font-style="normal" > <path vector-effect="none" fill-rule="evenodd" d="M0,5 C0,2.23858 2.23858,0 5,0 L45,0 C47.7614,0 50,2.23858 50,5 L50,15 C50,17.7614 47.7614,20 45,20 L5,20 C2.23858,20 0,17.7614 0,15 L0,5"/> </g> <g fill="none" stroke="#000000" stroke-opacity="1" stroke-width="1" stroke-linecap="square" stroke-linejoin="bevel" transform="matrix(1,0,0,1,375.5,198)" font-family="MS Shell Dlg 2" font-size="9.75" font-weight="400" font-style="normal" > <text fill="#000000" fill-opacity="1" stroke="none" xml:space="preserve" x="4" y="17" font-family="Arial" font-size="10pt" font-weight="700" font-style="italic" >rrnS</text> </g> <g fill="#ff9999" fill-opacity="1" stroke="#bfbfbf" stroke-opacity="1" stroke-width="1" stroke-linecap="square" stroke-linejoin="bevel" transform="matrix(1,0,0,1,418,200)" font-family="MS Shell Dlg 2" font-size="9.75" font-weight="400" font-style="normal" > <path vector-effect="none" fill-rule="evenodd" d="M0,1.7 L42.5,1.7 L42.5,0 L50,10 L42.5,20 L42.5,18.3 L0,18.3 L0,1.7"/> </g> <g fill="none" stroke="#000000" stroke-opacity="1" stroke-width="1" stroke-linecap="square" stroke-linejoin="bevel" transform="matrix(1,0,0,1,431.132,198)" font-family="MS Shell Dlg 2" font-size="9.75" font-weight="400" font-style="normal" > <text fill="#000000" fill-opacity="1" stroke="none" xml:space="preserve" x="4" y="17" font-family="Arial" font-size="10pt" font-weight="700" font-style="italic" >G</text> </g> <g fill="#ff9999" fill-opacity="1" stroke="#bfbfbf" stroke-opacity="1" stroke-width="1" stroke-linecap="square" stroke-linejoin="bevel" transform="matrix(1,0,0,1,469,200)" font-family="MS Shell Dlg 2" font-size="9.75" font-weight="400" font-style="normal" > <path vector-effect="none" fill-rule="evenodd" d="M0,1.7 L42.5,1.7 L42.5,0 L50,10 L42.5,20 L42.5,18.3 L0,18.3 L0,1.7"/> </g> <g fill="none" stroke="#000000" stroke-opacity="1" stroke-width="1" stroke-linecap="square" stroke-linejoin="bevel" transform="matrix(1,0,0,1,482.132,198)" font-family="MS Shell Dlg 2" font-size="9.75" font-weight="400" font-style="normal" > <text fill="#000000" fill-opacity="1" stroke="none" xml:space="preserve" x="4" y="17" font-family="Arial" font-size="10pt" font-weight="700" font-style="italic" >N</text> </g> <g fill="#ff9999" fill-opacity="1" stroke="#bfbfbf" stroke-opacity="1" stroke-width="1" stroke-linecap="square" stroke-linejoin="bevel" transform="matrix(1,0,0,1,520,200)" font-family="MS Shell Dlg 2" font-size="9.75" font-weight="400" font-style="normal" > <path vector-effect="none" fill-rule="evenodd" d="M0,1.7 L42.5,1.7 L42.5,0 L50,10 L42.5,20 L42.5,18.3 L0,18.3 L0,1.7"/> </g> <g fill="none" stroke="#000000" stroke-opacity="1" stroke-width="1" stroke-linecap="square" stroke-linejoin="bevel" transform="matrix(1,0,0,1,533.633,198)" font-family="MS Shell Dlg 2" font-size="9.75" font-weight="400" font-style="normal" > <text fill="#000000" fill-opacity="1" stroke="none" xml:space="preserve" x="4" y="17" font-family="Arial" font-size="10pt" font-weight="700" font-style="italic" >E</text> </g> <g fill="#ff9999" fill-opacity="1" stroke="#bfbfbf" stroke-opacity="1" stroke-width="1" stroke-linecap="square" stroke-linejoin="bevel" transform="matrix(1,0,0,1,571,200)" font-family="MS Shell Dlg 2" font-size="9.75" font-

weight="400" font-style="normal" > <path vector-effect="none" fill-rule="evenodd" d="M0,1.7 L42.5,1.7 L42.5,0 L50,10 L42.5,20 L42.5,18.3 L0,18.3 L0,1.7"/> </g> <g fill="none" stroke="#000000" stroke-opacity="1" stroke-width="1" stroke-linecap="square" stroke-linejoin="bevel" transform="matrix(1,0,0,1,584.133,198)" font-family="MS Shell Dlg 2" font-size="9.75" font-weight="400" font-style="normal" > <text fill="#000000" fill-opacity="1" stroke="none" xml:space="preserve" x="4" y="17" font-family="Arial" font-size="10pt" font-weight="700" font-style="italic" >C</text> </g> <g fill="#ff9999" fill-opacity="1" stroke="#bfbfbf" stroke-opacity="1" stroke-width="1" stroke-linecap="square" stroke-linejoin="bevel" transform="matrix(1,0,0,1,622,200)" font-family="MS Shell Dlg 2" font-size="9.75" font-weight="400" font-style="normal" > <path vector-effect="none" fill-rule="evenodd" d="M0,1.7 L42.5,1.7 L42.5,0 L50,10 L42.5,20 L42.5,18.3 L0,18.3 L0,1.7"/> </g> <g fill="none" stroke="#000000" stroke-opacity="1" stroke-width="1" stroke-linecap="square" stroke-linejoin="bevel" transform="matrix(1,0,0,1,637.133,198)" font-family="MS Shell Dlg 2" font-size="9.75" font-weight="400" font-style="normal" > <text fill="#000000" fill-opacity="1" stroke="none" xml:space="preserve" x="4" y="17" font-family="Arial" font-size="10pt" font-weight="700" font-style="italic" >I</text> </g> <g fill="#ff9999" fill-opacity="1" stroke="#bfbfbf" stroke-opacity="1" stroke-width="1" stroke-linecap="square" stroke-linejoin="bevel" transform="matrix(1,0,0,1,673,200)" font-family="MS Shell Dlg 2" font-size="9.75" font-weight="400" font-style="normal" > <path vector-effect="none" fill-rule="evenodd" d="M0,1.7 L42.5,1.7 L42.5,0 L50,10 L42.5,20 L42.5,18.3 L0,18.3 L0,1.7"/> </g> <g fill="none" stroke="#000000" stroke-opacity="1" stroke-width="1" stroke-linecap="square" stroke-linejoin="bevel" transform="matrix(1,0,0,1,685.633,198)" font-family="MS Shell Dlg 2" font-size="9.75" font-weight="400" font-style="normal" > <text fill="#000000" fill-opacity="1" stroke="none" xml:space="preserve" x="4" y="17" font-family="Arial" font-size="10pt" font-weight="700" font-style="italic" >Q</text> </g> <g fill="#ff9999" fill-opacity="1" stroke="#bfbfbf" stroke-opacity="1" stroke-width="1" stroke-linecap="square" stroke-linejoin="bevel" transform="matrix(1,0,0,1,724,200)" font-family="MS Shell Dlg 2" font-size="9.75" font-weight="400" font-style="normal" > <path vector-effect="none" fill-rule="evenodd" d="M0,1.7 L42.5,1.7 L42.5,0 L50,10 L42.5,20 L42.5,18.3 L0,18.3 L0,1.7"/> </g> <g fill="none" stroke="#000000" stroke-opacity="1" stroke-width="1" stroke-linecap="square" stroke-linejoin="bevel" transform="matrix(1,0,0,1,737.133,198)" font-family="MS Shell Dlg 2" font-size="9.75" font-weight="400" font-style="normal" > <text fill="#000000" fill-opacity="1" stroke="none" xml:space="preserve" x="4" y="17" font-family="Arial" font-size="10pt" font-weight="700" font-style="italic" >D</text> </g> <g fill="#99ffff" fill-opacity="1" stroke="#bfbfbf" stroke-opacity="1" stroke-width="1" stroke-linecap="square" stroke-linejoin="bevel" transform="matrix(1,0,0,1,775,200)" font-family="MS Shell Dlg 2" font-size="9.75" font-weight="400" font-style="normal" > <path vector-effect="none" fill-rule="evenodd" d="M0,5 C0,2.23858 2.23858,0 5,0 L45,0 C47.7614,0 50,2.23858 50,5 L50,15 C50,17.7614 47.7614,20 45,20 L5,20 C2.23858,20 0,17.7614 0,15 L0,5"/> </g> <g fill="none" stroke="#000000" stroke-opacity="1" stroke-width="1" stroke-linecap="square" stroke-linejoin="bevel" transform="matrix(1,0,0,1,784.5,198)" font-family="MS Shell Dlg 2" font-size="9.75" font-weight="400" font-style="normal" > <text fill="#000000" fill-opacity="1" stroke="none" xml:space="preserve" x="4" y="17" font-family="Arial" font-size="10pt" font-weight="700" font-style="italic" >rrnL</text> </g> <g fill="#ff9999" fill-opacity="1" stroke="#bfbfbf" stroke-opacity="1" stroke-width="1" stroke-linecap="square" stroke-linejoin="bevel" transform="matrix(1,0,0,1,826,200)" font-family="MS Shell Dlg 2" font-size="9.75" font-weight="400" font-style="normal" > <path vector-effect="none" fill-rule="evenodd" d="M0,1.7 L42.5,1.7 L42.5,0 L50,10 L42.5,20 L42.5,18.3 L0,18.3 L0,1.7"/> </g> <g fill="none" stroke="#000000" stroke-opacity="1" stroke-width="1" stroke-linecap="square" stroke-linejoin="bevel" transform="matrix(1,0,0,1,839.633,198)" font-family="MS Shell Dlg 2" font-size="9.75" font-weight="400" font-style="normal" > <text fill="#000000" fill-opacity="1" stroke="none" xml:space="preserve" x="4" y="17" font-family="Arial" font-size="10pt" font-weight="700" font-style="italic" >Y</text> </g> <g fill="#ffff33" fill-opacity="1" stroke="#bfbfbf" stroke-opacity="1" stroke-width="1" stroke-linecap="square" stroke-linejoin="bevel" transform="matrix(1,0,0,1,877,200)" font-family="MS Shell Dlg 2" font-size="9.75" font-weight="400" font-style="normal" > <rect x="0" y="0" width="50" height="20"/> </g> <g fill="none" stroke="#000000" stroke-opacity="1" stroke-width="1" stroke-linecap="square" stroke-linejoin="bevel" transform="matrix(1,0,0,1,886.5,198)" font-family="MS Shell Dlg 2" font-size="9.75" font-weight="400" font-style="normal" > <text fill="#000000" fill-opacity="1" stroke="none" xml:space="preserve" x="4" y="17" font-family="Arial" font-size="10pt" font-weight="700" font-style="italic" >cytb</text> </g> <g fill="#ffff33" fill-opacity="1" stroke="#bfbfbf" stroke-opacity="1" stroke-width="1" stroke-linecap="square" stroke-linejoin="bevel" transform="matrix(1,0,0,1,928,200)" font-family="MS Shell Dlg 2" font-size="9.75" font-weight="400" font-style="normal" > <rect x="0" y="0" width="50" height="20"/> </g> <g fill="none" stroke="#000000" stroke-opacity="1" stroke-width="1" stroke-linecap="square" stroke-linejoin="bevel" transform="matrix(1,0,0,1,936,198)" font-family="MS Shell Dlg 2" font-size="9.75" font-weight="400" font-style="normal" > <text fill="#000000" fill-opacity="1" stroke="none" xml:space="preserve" x="4" y="17" font-

family="Arial" font-size="10pt" font-weight="700" font-style="italic" >cox2</text> </g> <g fill="#ff9999" fill-opacity="1" stroke="#bfbfbf" stroke-opacity="1" stroke-width="1" stroke-linecap="square" stroke-linejoin="bevel" transform="matrix(1,0,0,1,979,200)" font-family="MS Shell Dlg 2" font-size="9.75" font-weight="400" font-style="normal" > <path vector-effect="none" fill-rule="evenodd" d="M0,1.7 L42.5,1.7 L42.5,0 L50,10 L42.5,20 L42.5,18.3 L0,18.3 L0,1.7"/> </g> <g fill="none" stroke="#000000" stroke-opacity="1" stroke-width="1" stroke-linecap="square" stroke-linejoin="bevel" transform="matrix(1,0,0,1,992.633,198)" font-family="MS Shell Dlg 2" font-size="9.75" font-weight="400" font-style="normal" > <text fill="#000000" fill-opacity="1" stroke="none" xml:space="preserve" x="4" y="17" font-family="Arial" font-size="10pt" font-weight="700" font-style="italic" >K</text> </g> <g fill="#ff9999" fill-opacity="1" stroke="#bfbfbf" stroke-opacity="1" stroke-width="1" stroke-linecap="square" stroke-linejoin="bevel" transform="matrix(1,0,0,1,1030,200)" font-family="MS Shell Dlg 2" font-size="9.75" font-weight="400" font-style="normal" > <path vector-effect="none" fill-rule="evenodd" d="M0,1.7 L42.5,1.7 L42.5,0 L50,10 L42.5,20 L42.5,18.3 L0,18.3 L0,1.7"/> </g> <g fill="none" stroke="#000000" stroke-opacity="1" stroke-width="1" stroke-linecap="square" stroke-linejoin="bevel" transform="matrix(1,0,0,1,1042.13,198)" font-family="MS Shell Dlg 2" font-size="9.75" font-weight="400" font-style="normal" > <text fill="#000000" fill-opacity="1" stroke="none" xml:space="preserve" x="4" y="17" font-family="Arial" font-size="10pt" font-weight="700" font-style="italic" >M</text> </g> <g fill="#ff9999" fill-opacity="1" stroke="#bfbfbf" stroke-opacity="1" stroke-width="1" stroke-linecap="square" stroke-linejoin="bevel" transform="matrix(1,0,0,1,1081,200)" font-family="MS Shell Dlg 2" font-size="9.75" font-weight="400" font-style="normal" > <path vector-effect="none" fill-rule="evenodd" d="M0,1.7 L42.5,1.7 L42.5,0 L50,10 L42.5,20 L42.5,18.3 L0,18.3 L0,1.7"/> </g> <g fill="none" stroke="#000000" stroke-opacity="1" stroke-width="1" stroke-linecap="square" stroke-linejoin="bevel" transform="matrix(1,0,0,1,1091.63,198)" font-family="MS Shell Dlg 2" font-size="9.75" font-weight="400" font-style="normal" > <text fill="#000000" fill-opacity="1" stroke="none" xml:space="preserve" x="4" y="17" font-family="Arial" font-size="10pt" font-weight="700" font-style="italic" >L1</text> </g> <g fill="#ff9999" fill-opacity="1" stroke="#bfbfbf" stroke-opacity="1" stroke-width="1" stroke-linecap="square" stroke-linejoin="bevel" transform="matrix(1,0,0,1,1132,200)" font-family="MS Shell Dlg 2" font-size="9.75" font-weight="400" font-style="normal" > <path vector-effect="none" fill-rule="evenodd" d="M0,1.7 L42.5,1.7 L42.5,0 L50,10 L42.5,20 L42.5,18.3 L0,18.3 L0,1.7"/> </g> <g fill="none" stroke="#000000" stroke-opacity="1" stroke-width="1" stroke-linecap="square" stroke-linejoin="bevel" transform="matrix(1,0,0,1,1142.63,198)" font-family="MS Shell Dlg 2" font-size="9.75" font-weight="400" font-style="normal" > <text fill="#000000" fill-opacity="1" stroke="none" xml:space="preserve" x="4" y="17" font-family="Arial" font-size="10pt" font-weight="700" font-style="italic" >L2</text> </g> <g fill="#ffff33" fill-opacity="1" stroke="#bfbfbf" stroke-opacity="1" stroke-width="1" stroke-linecap="square" stroke-linejoin="bevel" transform="matrix(1,0,0,1,1183,200)" font-family="MS Shell Dlg 2" font-size="9.75" font-weight="400" font-style="normal" > <rect x="0" y="0" width="50" height="20"/> </g> <g fill="none" stroke="#000000" stroke-opacity="1" stroke-width="1" stroke-linecap="square" stroke-linejoin="bevel" transform="matrix(1,0,0,1,1190,198)" font-family="MS Shell Dlg 2" font-size="9.75" font-weight="400" font-style="normal" > <text fill="#000000" fill-opacity="1" stroke="none" xml:space="preserve" x="4" y="17" font-family="Arial" font-size="10pt" font-weight="700" font-style="italic" >nad1</text> </g> <g fill="#ff9999" fill-opacity="1" stroke="#bfbfbf" stroke-opacity="1" stroke-width="1" stroke-linecap="square" stroke-linejoin="bevel" transform="matrix(1,0,0,1,1234,200)" font-family="MS Shell Dlg 2" font-size="9.75" font-weight="400" font-style="normal" > <path vector-effect="none" fill-rule="evenodd" d="M0,1.7 L42.5,1.7 L42.5,0 L50,10 L42.5,20 L42.5,18.3 L0,18.3 L0,1.7"/> </g> <g fill="none" stroke="#000000" stroke-opacity="1" stroke-width="1" stroke-linecap="square" stroke-linejoin="bevel" transform="matrix(1,0,0,1,1247.13,198)" font-family="MS Shell Dlg 2" font-size="9.75" font-weight="400" font-style="normal" > <text fill="#000000" fill-opacity="1" stroke="none" xml:space="preserve" x="4" y="17" font-family="Arial" font-size="10pt" font-weight="700" font-style="italic" >V</text> </g> <g fill="#ffff33" fill-opacity="1" stroke="#bfbfbf" stroke-opacity="1" stroke-width="1" stroke-linecap="square" stroke-linejoin="bevel" transform="matrix(1,0,0,1,1285,200)" font-family="MS Shell Dlg 2" font-size="9.75" font-weight="400" font-style="normal" > <rect x="0" y="0" width="50" height="20"/> </g> <g fill="none" stroke="#000000" stroke-opacity="1" stroke-width="1" stroke-linecap="square" stroke-linejoin="bevel" transform="matrix(1,0,0,1,1292,198)" font-family="MS Shell Dlg 2" font-size="9.75" font-weight="400" font-style="normal" > <text fill="#000000" fill-opacity="1" stroke="none" xml:space="preserve" x="4" y="17" font-family="Arial" font-size="10pt" font-weight="700" font-style="italic" >nad4</text> </g> <g fill="#ffff33" fill-opacity="1" stroke="#bfbfbf" stroke-opacity="1" stroke-width="1" stroke-linecap="square" stroke-linejoin="bevel" transform="matrix(1,0,0,1,1336,200)" font-family="MS Shell Dlg 2" font-size="9.75" font-weight="400" font-style="normal" > <rect x="0" y="0" width="50" height="20"/> </g> <g fill="none" stroke="#000000" stroke-opacity="1" stroke-width="1" stroke-linecap="square" stroke-linejoin="bevel"

transform="matrix(1,0,0,1,1344,198)" font-family="MS Shell Dlg 2" font-size="9.75" font-weight="400" font-style="normal" > <text fill="#000000" fill-opacity="1" stroke="none" xml:space="preserve" x="4" y="17" font-family="Arial" font-size="10pt" font-weight="700" font-style="italic" >cox3</text> </g> <g fill="#ff9999" fill-opacity="1" stroke="#bfbfbf" stroke-opacity="1" stroke-width="1" stroke-linecap="square" stroke-linejoin="bevel" transform="matrix(1,0,0,1,1387,200)" font-family="MS Shell Dlg 2" font-size="9.75" font-weight="400" font-style="normal" > <path vector-effect="none" fill-rule="evenodd" d="M0,1.7 L42.5,1.7 L42.5,0 L50,10 L42.5,20 L42.5,18.3 L0,18.3 L0,1.7"/> </g> <g fill="none" stroke="#000000" stroke-opacity="1" stroke-width="1" stroke-linecap="square" stroke-linejoin="bevel" transform="matrix(1,0,0,1,1400.13,198)" font-family="MS Shell Dlg 2" font-size="9.75" font-weight="400" font-style="normal" > <text fill="#000000" fill-opacity="1" stroke="none" xml:space="preserve" x="4" y="17" font-family="Arial" font-size="10pt" font-weight="700" font-style="italic" >S</text> </g> <g fill="#ff9999" fill-opacity="1" stroke="#bfbfbf" stroke-opacity="1" stroke-width="1" stroke-linecap="square" stroke-linejoin="bevel" transform="matrix(1,0,0,1,1438,200)" font-family="MS Shell Dlg 2" font-size="9.75" font-weight="400" font-style="normal" > <path vector-effect="none" fill-rule="evenodd" d="M0,1.7 L42.5,1.7 L42.5,0 L50,10 L42.5,20 L42.5,18.3 L0,18.3 L0,1.7"/> </g> <g fill="none" stroke="#000000" stroke-opacity="1" stroke-width="1" stroke-linecap="square" stroke-linejoin="bevel" transform="matrix(1,0,0,1,1450.13,198)" font-family="MS Shell Dlg 2" font-size="9.75" font-weight="400" font-style="normal" > <text fill="#000000" fill-opacity="1" stroke="none" xml:space="preserve" x="4" y="17" font-family="Arial" font-size="10pt" font-weight="700" font-style="italic" >M</text> </g> <g fill="#ffff33" fill-opacity="1" stroke="#bfbfbf" stroke-opacity="1" stroke-width="1" stroke-linecap="square" stroke-linejoin="bevel" transform="matrix(1,0,0,1,1489,200)" font-family="MS Shell Dlg 2" font-size="9.75" font-weight="400" font-style="normal" > <rect x="0" y="0" width="50" height="20"/> </g> <g fill="none" stroke="#000000" stroke-opacity="1" stroke-width="1" stroke-linecap="square" stroke-linejoin="bevel" transform="matrix(1,0,0,1,1496,198)" font-family="MS Shell Dlg 2" font-size="9.75" font-weight="400" font-style="normal" > <text fill="#000000" fill-opacity="1" stroke="none" xml:space="preserve" x="4" y="17" font-family="Arial" font-size="10pt" font-weight="700" font-style="italic" >nad2</text> </g> <g fill="#ff9999" fill-opacity="1" stroke="#bfbfbf" stroke-opacity="1" stroke-width="1" stroke-linecap="square" stroke-linejoin="bevel" transform="matrix(1,0,0,1,1540,200)" font-family="MS Shell Dlg 2" font-size="9.75" font-weight="400" font-style="normal" > <path vector-effect="none" fill-rule="evenodd" d="M0,1.7 L42.5,1.7 L42.5,0 L50,10 L42.5,20 L42.5,18.3 L0,18.3 L0,1.7"/> </g> <g fill="none" stroke="#000000" stroke-opacity="1" stroke-width="1" stroke-linecap="square" stroke-linejoin="bevel" transform="matrix(1,0,0,1,1553.13,198)" font-family="MS Shell Dlg 2" font-size="9.75" font-weight="400" font-style="normal" > <text fill="#000000" fill-opacity="1" stroke="none" xml:space="preserve" x="4" y="17" font-family="Arial" font-size="10pt" font-weight="700" font-style="italic" >R</text> </g> <g fill="#ff9999" fill-opacity="1" stroke="#bfbfbf" stroke-opacity="1" stroke-width="1" stroke-linecap="square" stroke-linejoin="bevel" transform="matrix(1,0,0,1,1591,200)" font-family="MS Shell Dlg 2" font-size="9.75" font-weight="400" font-style="normal" > <path vector-effect="none" fill-rule="evenodd" d="M0,1.7 L42.5,1.7 L42.5,0 L50,10 L42.5,20 L42.5,18.3 L0,18.3 L0,1.7"/> </g> <g fill="none" stroke="#000000" stroke-opacity="1" stroke-width="1" stroke-linecap="square" stroke-linejoin="bevel" transform="matrix(1,0,0,1,1602.13,198)" font-family="MS Shell Dlg 2" font-size="9.75" font-weight="400" font-style="normal" > <text fill="#000000" fill-opacity="1" stroke="none" xml:space="preserve" x="4" y="17" font-family="Arial" font-size="10pt" font-weight="700" font-style="italic" >W</text> </g> <g fill="#ff9999" fill-opacity="1" stroke="#bfbfbf" stroke-opacity="1" stroke-width="1" stroke-linecap="square" stroke-linejoin="bevel" transform="matrix(1,0,0,1,1642,200)" font-family="MS Shell Dlg 2" font-size="9.75" font-weight="400" font-style="normal" > <path vector-effect="none" fill-rule="evenodd" d="M0,1.7 L42.5,1.7 L42.5,0 L50,10 L42.5,20 L42.5,18.3 L0,18.3 L0,1.7"/> </g> <g fill="none" stroke="#000000" stroke-opacity="1" stroke-width="1" stroke-linecap="square" stroke-linejoin="bevel" transform="matrix(1,0,0,1,1655.13,198)" font-family="MS Shell Dlg 2" font-size="9.75" font-weight="400" font-style="normal" > <text fill="#000000" fill-opacity="1" stroke="none" xml:space="preserve" x="4" y="17" font-family="Arial" font-size="10pt" font-weight="700" font-style="italic" >A</text> </g> <g fill="#ff9999" fill-opacity="1" stroke="#bfbfbf" stroke-opacity="1" stroke-width="1" stroke-linecap="square" stroke-linejoin="bevel" transform="matrix(1,0,0,1,1693,200)" font-family="MS Shell Dlg 2" font-size="9.75" font-weight="400" font-style="normal" > <path vector-effect="none" fill-rule="evenodd" d="M0,1.7 L42.5,1.7 L42.5,0 L50,10 L42.5,20 L42.5,18.3 L0,18.3 L0,1.7"/> </g> <g fill="none" stroke="#000000" stroke-opacity="1" stroke-width="1" stroke-linecap="square" stroke-linejoin="bevel" transform="matrix(1,0,0,1,1702.63,198)" font-family="MS Shell Dlg 2" font-size="9.75" font-weight="400" font-style="normal" > <text fill="#000000" fill-opacity="1" stroke="none" xml:space="preserve" x="4" y="17" font-family="Arial" font-size="10pt" font-weight="700" font-style="italic" >S1</text> </g> <g fill="#ff9999" fill-opacity="1" stroke="#bfbfbf" stroke-opacity="1" stroke-width="1" stroke-linecap="square" stroke-linejoin="bevel" transform="matrix(1,0,0,1,1744,200)" font-family="MS Shell Dlg 2" font-size="9.75" font-

weight="400" font-style="normal" > <path vector-effect="none" fill-rule="evenodd" d="M0,1.7 L42.5,1.7 L42.5,0 L50,10 L42.5,20 L42.5,18.3 L0,18.3 L0,1.7"/> </g> <g fill="none" stroke="#000000" stroke-opacity="1" stroke-width="1" stroke-linecap="square" stroke-linejoin="bevel" transform="matrix(1,0,0,1,1757.13,198)" font-family="MS Shell Dlg 2" font-size="9.75" font-weight="400" font-style="normal" > <text fill="#000000" fill-opacity="1" stroke="none" xml:space="preserve" x="4" y="17" font-family="Arial" font-size="10pt" font-weight="700" font-style="italic" >H</text> </g> <g fill="#ff9999" fill-opacity="1" stroke="#bfbfbf" stroke-opacity="1" stroke-width="1" stroke-linecap="square" stroke-linejoin="bevel" transform="matrix(1,0,0,1,1795,200)" font-family="MS Shell Dlg 2" font-size="9.75" font-weight="400" font-style="normal" > <path vector-effect="none" fill-rule="evenodd" d="M0,1.7 L42.5,1.7 L42.5,0 L50,10 L42.5,20 L42.5,18.3 L0,18.3 L0,1.7"/> </g> <g fill="none" stroke="#000000" stroke-opacity="1" stroke-width="1" stroke-linecap="square" stroke-linejoin="bevel" transform="matrix(1,0,0,1,1808.63,198)" font-family="MS Shell Dlg 2" font-size="9.75" font-weight="400" font-style="normal" > <text fill="#000000" fill-opacity="1" stroke="none" xml:space="preserve" x="4" y="17" font-family="Arial" font-size="10pt" font-weight="700" font-style="italic" >P</text> </g> <g fill="#ffff33" fill-opacity="1" stroke="#bfbfbf" stroke-opacity="1" stroke-width="1" stroke-linecap="square" stroke-linejoin="bevel" transform="matrix(1,0,0,1,1846,200)" font-family="MS Shell Dlg 2" font-size="9.75" font-weight="400" font-style="normal" > <rect x="0" y="0" width="50" height="20"/> </g> <g fill="none" stroke="#000000" stroke-opacity="1" stroke-width="1" stroke-linecap="square" stroke-linejoin="bevel" transform="matrix(1,0,0,1,1853,198)" font-family="MS Shell Dlg 2" font-size="9.75" font-weight="400" font-style="normal" > <text fill="#000000" fill-opacity="1" stroke="none" xml:space="preserve" x="4" y="17" font-family="Arial" font-size="10pt" font-weight="700" font-style="italic" >nad3</text> </g> <g fill="#ffff33" fill-opacity="1" stroke="#bfbfbf" stroke-opacity="1" stroke-width="1" stroke-linecap="square" stroke-linejoin="bevel" transform="matrix(1,0,0,1,1897,200)" font-family="MS Shell Dlg 2" font-size="9.75" font-weight="400" font-style="normal" > <rect x="0" y="0" width="50" height="20"/> </g> <g fill="none" stroke="#000000" stroke-opacity="1" stroke-width="1" stroke-linecap="square" stroke-linejoin="bevel" transform="matrix(1,0,0,1,1905.5,198)" font-family="MS Shell Dlg 2" font-size="9.75" font-weight="400" font-style="normal" > <text fill="#000000" fill-opacity="1" stroke="none" xml:space="preserve" x="4" y="17" font-family="Arial" font-size="10pt" font-weight="700" font-style="italic" >atp8</text> </g> <g fill="none" stroke="#000000" stroke-opacity="1" stroke-width="1" stroke-linecap="square" stroke-linejoin="bevel" transform="matrix(1,0,0,1,1948,200)" font-family="MS Shell Dlg 2" font-size="9.75" font-weight="400" font-style="normal" > <text fill="#000000" fill-opacity="1" stroke="none" xml:space="preserve" x="4" y="17" font-family="Arial" font-size="10pt" font-weight="700" font-style="italic" >(+)</text> </g> <g fill="none" stroke="#000000" stroke-opacity="1" stroke-width="1" stroke-linecap="square" stroke-linejoin="bevel" transform="matrix(1,0,0,1,10,240)" font-family="MS Shell Dlg 2" font-size="9.75" font-weight="400" font-style="normal" > <text fill="#000000" fill-opacity="1" stroke="none" xml:space="preserve" x="4" y="17" font-family="Arial" font-size="10pt" font-weight="700" font-style="italic" >Mytilus_chilensis_NC_030633</text> </g> <g fill="#ffff33" fill-opacity="1" stroke="#bfbfbf" stroke-opacity="1" stroke-width="1" stroke-linecap="square" stroke-linejoin="bevel" transform="matrix(1,0,0,1,10,270)" font-family="MS Shell Dlg 2" font-size="9.75" font-weight="400" font-style="normal" > <rect x="0" y="0" width="50" height="20"/> </g> <g fill="none" stroke="#000000" stroke-opacity="1" stroke-width="1" stroke-linecap="square" stroke-linejoin="bevel" transform="matrix(1,0,0,1,18,268)" font-family="MS Shell Dlg 2" font-size="9.75" font-weight="400" font-style="normal" > <text fill="#000000" fill-opacity="1" stroke="none" xml:space="preserve" x="4" y="17" font-family="Arial" font-size="10pt" font-weight="700" font-style="italic" >cox1</text> </g> <g fill="#ffff33" fill-opacity="1" stroke="#bfbfbf" stroke-opacity="1" stroke-width="1" stroke-linecap="square" stroke-linejoin="bevel" transform="matrix(1,0,0,1,61,270)" font-family="MS Shell Dlg 2" font-size="9.75" font-weight="400" font-style="normal" > <rect x="0" y="0" width="50" height="20"/> </g> <g fill="none" stroke="#000000" stroke-opacity="1" stroke-width="1" stroke-linecap="square" stroke-linejoin="bevel" transform="matrix(1,0,0,1,69.5,268)" font-family="MS Shell Dlg 2" font-size="9.75" font-weight="400" font-style="normal" > <text fill="#000000" fill-opacity="1" stroke="none" xml:space="preserve" x="4" y="17" font-family="Arial" font-size="10pt" font-weight="700" font-style="italic" >atp6</text> </g> <g fill="#ff9999" fill-opacity="1" stroke="#bfbfbf" stroke-opacity="1" stroke-width="1" stroke-linecap="square" stroke-linejoin="bevel" transform="matrix(1,0,0,1,112,270)" font-family="MS Shell Dlg 2" font-size="9.75" font-weight="400" font-style="normal" > <path vector-effect="none" fill-rule="evenodd" d="M0,1.7 L42.5,1.7 L42.5,0 L50,10 L42.5,20 L42.5,18.3 L0,18.3 L0,1.7"/> </g> <g fill="none" stroke="#000000" stroke-opacity="1" stroke-width="1" stroke-linecap="square" stroke-linejoin="bevel" transform="matrix(1,0,0,1,125.132,268)" font-family="MS Shell Dlg 2" font-size="9.75" font-weight="400" font-style="normal" > <text fill="#000000" fill-opacity="1" stroke="none" xml:space="preserve" x="4" y="17" font-family="Arial" font-size="10pt" font-weight="700" font-style="italic" >T</text> </g> <g fill="#ffff33" fill-opacity="1" stroke="#bfbfbf" stroke-opacity="1" stroke-width="1" stroke-linecap="square" stroke-linejoin="bevel"

transform="matrix(1,0,0,1,163,270)" font-family="MS Shell Dlg 2" font-size="9.75" font-weight="400" font-style="normal" > <rect x="0" y="0" width="50" height="20"/> </g> <g fill="none" stroke="#000000" stroke-opacity="1" stroke-width="1" stroke-linecap="square" stroke-linejoin="bevel" transform="matrix(1,0,0,1,167,268)" font-family="MS Shell Dlg 2" font-size="9.75" font-weight="400" font-style="normal" > <text fill="#000000" fill-opacity="1" stroke="none" xml:space="preserve" x="4" y="17" font-family="Arial" font-size="10pt" font-weight="700" font-style="italic" >nad4L</text> </g> <g fill="#ffff33" fill-opacity="1" stroke="#bfbfbf" stroke-opacity="1" stroke-width="1" stroke-linecap="square" stroke-linejoin="bevel" transform="matrix(1,0,0,1,214,270)" font-family="MS Shell Dlg 2" font-size="9.75" font-weight="400" font-style="normal" > <rect x="0" y="0" width="50" height="20"/> </g> <g fill="none" stroke="#000000" stroke-opacity="1" stroke-width="1" stroke-linecap="square" stroke-linejoin="bevel" transform="matrix(1,0,0,1,221,268)" font-family="MS Shell Dlg 2" font-size="9.75" font-weight="400" font-style="normal" > <text fill="#000000" fill-opacity="1" stroke="none" xml:space="preserve" x="4" y="17" font-family="Arial" font-size="10pt" font-weight="700" font-style="italic" >nad5</text> </g> <g fill="#ffff33" fill-opacity="1" stroke="#bfbfbf" stroke-opacity="1" stroke-width="1" stroke-linecap="square" stroke-linejoin="bevel" transform="matrix(1,0,0,1,265,270)" font-family="MS Shell Dlg 2" font-size="9.75" font-weight="400" font-style="normal" > <rect x="0" y="0" width="50" height="20"/> </g> <g fill="none" stroke="#000000" stroke-opacity="1" stroke-width="1" stroke-linecap="square" stroke-linejoin="bevel" transform="matrix(1,0,0,1,272,268)" font-family="MS Shell Dlg 2" font-size="9.75" font-weight="400" font-style="normal" > <text fill="#000000" fill-opacity="1" stroke="none" xml:space="preserve" x="4" y="17" font-family="Arial" font-size="10pt" font-weight="700" font-style="italic" >nad6</text> </g> <g fill="#ff9999" fill-opacity="1" stroke="#bfbfbf" stroke-opacity="1" stroke-width="1" stroke-linecap="square" stroke-linejoin="bevel" transform="matrix(1,0,0,1,316,270)" font-family="MS Shell Dlg 2" font-size="9.75" font-weight="400" font-style="normal" > <path vector-effect="none" fill-rule="evenodd" d="M0,1.7 L42.5,1.7 L42.5,0 L50,10 L42.5,20 L42.5,18.3 L0,18.3 L0,1.7"/> </g> <g fill="none" stroke="#000000" stroke-opacity="1" stroke-width="1" stroke-linecap="square" stroke-linejoin="bevel" transform="matrix(1,0,0,1,329.632,268)" font-family="MS Shell Dlg 2" font-size="9.75" font-weight="400" font-style="normal" > <text fill="#000000" fill-opacity="1" stroke="none" xml:space="preserve" x="4" y="17" font-family="Arial" font-size="10pt" font-weight="700" font-style="italic" >F</text> </g> <g fill="#99ffff" fill-opacity="1" stroke="#bfbfbf" stroke-opacity="1" stroke-width="1" stroke-linecap="square" stroke-linejoin="bevel" transform="matrix(1,0,0,1,367,270)" font-family="MS Shell Dlg 2" font-size="9.75" font-weight="400" font-style="normal" > <path vector-effect="none" fill-rule="evenodd" d="M0,5 C0,2.23858 2.23858,0 5,0 L45,0 C47.7614,0 50,2.23858 50,5 L50,15 C50,17.7614 47.7614,20 45,20 L5,20 C2.23858,20 0,17.7614 0,15 L0,5"/> </g> <g fill="none" stroke="#000000" stroke-opacity="1" stroke-width="1" stroke-linecap="square" stroke-linejoin="bevel" transform="matrix(1,0,0,1,375.5,268)" font-family="MS Shell Dlg 2" font-size="9.75" font-weight="400" font-style="normal" > <text fill="#000000" fill-opacity="1" stroke="none" xml:space="preserve" x="4" y="17" font-family="Arial" font-size="10pt" font-weight="700" font-style="italic" >rrnS</text> </g> <g fill="#ff9999" fill-opacity="1" stroke="#bfbfbf" stroke-opacity="1" stroke-width="1" stroke-linecap="square" stroke-linejoin="bevel" transform="matrix(1,0,0,1,418,270)" font-family="MS Shell Dlg 2" font-size="9.75" font-weight="400" font-style="normal" > <path vector-effect="none" fill-rule="evenodd" d="M0,1.7 L42.5,1.7 L42.5,0 L50,10 L42.5,20 L42.5,18.3 L0,18.3 L0,1.7"/> </g> <g fill="none" stroke="#000000" stroke-opacity="1" stroke-width="1" stroke-linecap="square" stroke-linejoin="bevel" transform="matrix(1,0,0,1,431.132,268)" font-family="MS Shell Dlg 2" font-size="9.75" font-weight="400" font-style="normal" > <text fill="#000000" fill-opacity="1" stroke="none" xml:space="preserve" x="4" y="17" font-family="Arial" font-size="10pt" font-weight="700" font-style="italic" >G</text> </g> <g fill="#ff9999" fill-opacity="1" stroke="#bfbfbf" stroke-opacity="1" stroke-width="1" stroke-linecap="square" stroke-linejoin="bevel" transform="matrix(1,0,0,1,469,270)" font-family="MS Shell Dlg 2" font-size="9.75" font-weight="400" font-style="normal" > <path vector-effect="none" fill-rule="evenodd" d="M0,1.7 L42.5,1.7 L42.5,0 L50,10 L42.5,20 L42.5,18.3 L0,18.3 L0,1.7"/> </g> <g fill="none" stroke="#000000" stroke-opacity="1" stroke-width="1" stroke-linecap="square" stroke-linejoin="bevel" transform="matrix(1,0,0,1,482.132,268)" font-family="MS Shell Dlg 2" font-size="9.75" font-weight="400" font-style="normal" > <text fill="#000000" fill-opacity="1" stroke="none" xml:space="preserve" x="4" y="17" font-family="Arial" font-size="10pt" font-weight="700" font-style="italic" >N</text> </g> <g fill="#ff9999" fill-opacity="1" stroke="#bfbfbf" stroke-opacity="1" stroke-width="1" stroke-linecap="square" stroke-linejoin="bevel" transform="matrix(1,0,0,1,520,270)" font-family="MS Shell Dlg 2" font-size="9.75" font-weight="400" font-style="normal" > <path vector-effect="none" fill-rule="evenodd" d="M0,1.7 L42.5,1.7 L42.5,0 L50,10 L42.5,20 L42.5,18.3 L0,18.3 L0,1.7"/> </g> <g fill="none" stroke="#000000" stroke-opacity="1" stroke-width="1" stroke-linecap="square" stroke-linejoin="bevel" transform="matrix(1,0,0,1,533.633,268)" font-family="MS Shell Dlg 2" font-size="9.75" font-weight="400" font-style="normal" > <text fill="#000000" fill-opacity="1" stroke="none" xml:space="preserve" x="4" y="17" font-

family="Arial" font-size="10pt" font-weight="700" font-style="italic" >E</text> </g> <g fill="#ff9999" fill-opacity="1" stroke="#bfbfbf" stroke-opacity="1" stroke-width="1" stroke-linecap="square" stroke-linejoin="bevel" transform="matrix(1,0,0,1,571,270)" font-family="MS Shell Dlg 2" font-size="9.75" font-weight="400" font-style="normal" > <path vector-effect="none" fill-rule="evenodd" d="M0,1.7 L42.5,1.7 L42.5,0 L50,10 L42.5,20 L42.5,18.3 L0,18.3 L0,1.7"/> </g> <g fill="none" stroke="#000000" stroke-opacity="1" stroke-width="1" stroke-linecap="square" stroke-linejoin="bevel" transform="matrix(1,0,0,1,584.133,268)" font-family="MS Shell Dlg 2" font-size="9.75" font-weight="400" font-style="normal" > <text fill="#000000" fill-opacity="1" stroke="none" xml:space="preserve" x="4" y="17" font-family="Arial" font-size="10pt" font-weight="700" font-style="italic" >C</text> </g> <g fill="#ff9999" fill-opacity="1" stroke="#bfbfbf" stroke-opacity="1" stroke-width="1" stroke-linecap="square" stroke-linejoin="bevel" transform="matrix(1,0,0,1,622,270)" font-family="MS Shell Dlg 2" font-size="9.75" font-weight="400" font-style="normal" > <path vector-effect="none" fill-rule="evenodd" d="M0,1.7 L42.5,1.7 L42.5,0 L50,10 L42.5,20 L42.5,18.3 L0,18.3 L0,1.7"/> </g> <g fill="none" stroke="#000000" stroke-opacity="1" stroke-width="1" stroke-linecap="square" stroke-linejoin="bevel" transform="matrix(1,0,0,1,637.133,268)" font-family="MS Shell Dlg 2" font-size="9.75" font-weight="400" font-style="normal" > <text fill="#000000" fill-opacity="1" stroke="none" xml:space="preserve" x="4" y="17" font-family="Arial" font-size="10pt" font-weight="700" font-style="italic" >I</text> </g> <g fill="#ff9999" fill-opacity="1" stroke="#bfbfbf" stroke-opacity="1" stroke-width="1" stroke-linecap="square" stroke-linejoin="bevel" transform="matrix(1,0,0,1,673,270)" font-family="MS Shell Dlg 2" font-size="9.75" font-weight="400" font-style="normal" > <path vector-effect="none" fill-rule="evenodd" d="M0,1.7 L42.5,1.7 L42.5,0 L50,10 L42.5,20 L42.5,18.3 L0,18.3 L0,1.7"/> </g> <g fill="none" stroke="#000000" stroke-opacity="1" stroke-width="1" stroke-linecap="square" stroke-linejoin="bevel" transform="matrix(1,0,0,1,685.633,268)" font-family="MS Shell Dlg 2" font-size="9.75" font-weight="400" font-style="normal" > <text fill="#000000" fill-opacity="1" stroke="none" xml:space="preserve" x="4" y="17" font-family="Arial" font-size="10pt" font-weight="700" font-style="italic" >Q</text> </g> <g fill="#ff9999" fill-opacity="1" stroke="#bfbfbf" stroke-opacity="1" stroke-width="1" stroke-linecap="square" stroke-linejoin="bevel" transform="matrix(1,0,0,1,724,270)" font-family="MS Shell Dlg 2" font-size="9.75" font-weight="400" font-style="normal" > <path vector-effect="none" fill-rule="evenodd" d="M0,1.7 L42.5,1.7 L42.5,0 L50,10 L42.5,20 L42.5,18.3 L0,18.3 L0,1.7"/> </g> <g fill="none" stroke="#000000" stroke-opacity="1" stroke-width="1" stroke-linecap="square" stroke-linejoin="bevel" transform="matrix(1,0,0,1,737.133,268)" font-family="MS Shell Dlg 2" font-size="9.75" font-weight="400" font-style="normal" > <text fill="#000000" fill-opacity="1" stroke="none" xml:space="preserve" x="4" y="17" font-family="Arial" font-size="10pt" font-weight="700" font-style="italic" >D</text> </g> <g fill="#99ffff" fill-opacity="1" stroke="#bfbfbf" stroke-opacity="1" stroke-width="1" stroke-linecap="square" stroke-linejoin="bevel" transform="matrix(1,0,0,1,775,270)" font-family="MS Shell Dlg 2" font-size="9.75" font-weight="400" font-style="normal" > <path vector-effect="none" fill-rule="evenodd" d="M0,5 C0,2.23858 2.23858,0 5,0 L45,0 C47.7614,0 50,2.23858 50,5 L50,15 C50,17.7614 47.7614,20 45,20 L5,20 C2.23858,20 0,17.7614 0,15 L0,5"/> </g> <g fill="none" stroke="#000000" stroke-opacity="1" stroke-width="1" stroke-linecap="square" stroke-linejoin="bevel" transform="matrix(1,0,0,1,784.5,268)" font-family="MS Shell Dlg 2" font-size="9.75" font-weight="400" font-style="normal" > <text fill="#000000" fill-opacity="1" stroke="none" xml:space="preserve" x="4" y="17" font-family="Arial" font-size="10pt" font-weight="700" font-style="italic" >rrnL</text> </g> <g fill="#ff9999" fill-opacity="1" stroke="#bfbfbf" stroke-opacity="1" stroke-width="1" stroke-linecap="square" stroke-linejoin="bevel" transform="matrix(1,0,0,1,826,270)" font-family="MS Shell Dlg 2" font-size="9.75" font-weight="400" font-style="normal" > <path vector-effect="none" fill-rule="evenodd" d="M0,1.7 L42.5,1.7 L42.5,0 L50,10 L42.5,20 L42.5,18.3 L0,18.3 L0,1.7"/> </g> <g fill="none" stroke="#000000" stroke-opacity="1" stroke-width="1" stroke-linecap="square" stroke-linejoin="bevel" transform="matrix(1,0,0,1,839.633,268)" font-family="MS Shell Dlg 2" font-size="9.75" font-weight="400" font-style="normal" > <text fill="#000000" fill-opacity="1" stroke="none" xml:space="preserve" x="4" y="17" font-family="Arial" font-size="10pt" font-weight="700" font-style="italic" >Y</text> </g> <g fill="#ffff33" fill-opacity="1" stroke="#bfbfbf" stroke-opacity="1" stroke-width="1" stroke-linecap="square" stroke-linejoin="bevel" transform="matrix(1,0,0,1,877,270)" font-family="MS Shell Dlg 2" font-size="9.75" font-weight="400" font-style="normal" > <rect x="0" y="0" width="50" height="20"/> </g> <g fill="none" stroke="#000000" stroke-opacity="1" stroke-width="1" stroke-linecap="square" stroke-linejoin="bevel" transform="matrix(1,0,0,1,886.5,268)" font-family="MS Shell Dlg 2" font-size="9.75" font-weight="400" font-style="normal" > <text fill="#000000" fill-opacity="1" stroke="none" xml:space="preserve" x="4" y="17" font-family="Arial" font-size="10pt" font-weight="700" font-style="italic" >cytb</text> </g> <g fill="#ffff33" fill-opacity="1" stroke="#bfbfbf" stroke-opacity="1" stroke-width="1" stroke-linecap="square" stroke-linejoin="bevel" transform="matrix(1,0,0,1,928,270)" font-family="MS Shell Dlg 2" font-size="9.75" font-weight="400" font-style="normal" > <rect x="0" y="0" width="50" height="20"/> </g> <g fill="none"

stroke="#000000" stroke-opacity="1" stroke-width="1" stroke-linecap="square" stroke-linejoin="bevel" transform="matrix(1,0,0,1,936,268)" font-family="MS Shell Dlg 2" font-size="9.75" font-weight="400" font-style="normal" > <text fill="#000000" fill-opacity="1" stroke="none" xml:space="preserve" x="4" y="17" font-family="Arial" font-size="10pt" font-weight="700" font-style="italic" >cox2</text> </g> <g fill="#ff9999" fill-opacity="1" stroke="#bfbfbf" stroke-opacity="1" stroke-width="1" stroke-linecap="square" stroke-linejoin="bevel" transform="matrix(1,0,0,1,979,270)" font-family="MS Shell Dlg 2" font-size="9.75" font-weight="400" font-style="normal" > <path vector-effect="none" fill-rule="evenodd" d="M0,1.7 L42.5,1.7 L42.5,0 L50,10 L42.5,20 L42.5,18.3 L0,18.3 L0,1.7"/> </g> <g fill="none" stroke="#000000" stroke-opacity="1" stroke-width="1" stroke-linecap="square" stroke-linejoin="bevel" transform="matrix(1,0,0,1,992.633,268)" font-family="MS Shell Dlg 2" font-size="9.75" font-weight="400" font-style="normal" > <text fill="#000000" fill-opacity="1" stroke="none" xml:space="preserve" x="4" y="17" font-family="Arial" font-size="10pt" font-weight="700" font-style="italic" >K</text> </g> <g fill="#ff9999" fill-opacity="1" stroke="#bfbfbf" stroke-opacity="1" stroke-width="1" stroke-linecap="square" stroke-linejoin="bevel" transform="matrix(1,0,0,1,1030,270)" font-family="MS Shell Dlg 2" font-size="9.75" font-weight="400" font-style="normal" > <path vector-effect="none" fill-rule="evenodd" d="M0,1.7 L42.5,1.7 L42.5,0 L50,10 L42.5,20 L42.5,18.3 L0,18.3 L0,1.7"/> </g> <g fill="none" stroke="#000000" stroke-opacity="1" stroke-width="1" stroke-linecap="square" stroke-linejoin="bevel" transform="matrix(1,0,0,1,1042.13,268)" font-family="MS Shell Dlg 2" font-size="9.75" font-weight="400" font-style="normal" > <text fill="#000000" fill-opacity="1" stroke="none" xml:space="preserve" x="4" y="17" font-family="Arial" font-size="10pt" font-weight="700" font-style="italic" >M</text> </g> <g fill="#ff9999" fill-opacity="1" stroke="#bfbfbf" stroke-opacity="1" stroke-width="1" stroke-linecap="square" stroke-linejoin="bevel" transform="matrix(1,0,0,1,1081,270)" font-family="MS Shell Dlg 2" font-size="9.75" font-weight="400" font-style="normal" > <path vector-effect="none" fill-rule="evenodd" d="M0,1.7 L42.5,1.7 L42.5,0 L50,10 L42.5,20 L42.5,18.3 L0,18.3 L0,1.7"/> </g> <g fill="none" stroke="#000000" stroke-opacity="1" stroke-width="1" stroke-linecap="square" stroke-linejoin="bevel" transform="matrix(1,0,0,1,1091.63,268)" font-family="MS Shell Dlg 2" font-size="9.75" font-weight="400" font-style="normal" > <text fill="#000000" fill-opacity="1" stroke="none" xml:space="preserve" x="4" y="17" font-family="Arial" font-size="10pt" font-weight="700" font-style="italic" >L1</text> </g> <g fill="#ff9999" fill-opacity="1" stroke="#bfbfbf" stroke-opacity="1" stroke-width="1" stroke-linecap="square" stroke-linejoin="bevel" transform="matrix(1,0,0,1,1132,270)" font-family="MS Shell Dlg 2" font-size="9.75" font-weight="400" font-style="normal" > <path vector-effect="none" fill-rule="evenodd" d="M0,1.7 L42.5,1.7 L42.5,0 L50,10 L42.5,20 L42.5,18.3 L0,18.3 L0,1.7"/> </g> <g fill="none" stroke="#000000" stroke-opacity="1" stroke-width="1" stroke-linecap="square" stroke-linejoin="bevel" transform="matrix(1,0,0,1,1142.63,268)" font-family="MS Shell Dlg 2" font-size="9.75" font-weight="400" font-style="normal" > <text fill="#000000" fill-opacity="1" stroke="none" xml:space="preserve" x="4" y="17" font-family="Arial" font-size="10pt" font-weight="700" font-style="italic" >L2</text> </g> <g fill="#ffff33" fill-opacity="1" stroke="#bfbfbf" stroke-opacity="1" stroke-width="1" stroke-linecap="square" stroke-linejoin="bevel" transform="matrix(1,0,0,1,1183,270)" font-family="MS Shell Dlg 2" font-size="9.75" font-weight="400" font-style="normal" > <rect x="0" y="0" width="50" height="20"/> </g> <g fill="none" stroke="#000000" stroke-opacity="1" stroke-width="1" stroke-linecap="square" stroke-linejoin="bevel" transform="matrix(1,0,0,1,1190,268)" font-family="MS Shell Dlg 2" font-size="9.75" font-weight="400" font-style="normal" > <text fill="#000000" fill-opacity="1" stroke="none" xml:space="preserve" x="4" y="17" font-family="Arial" font-size="10pt" font-weight="700" font-style="italic" >nad1</text> </g> <g fill="#ff9999" fill-opacity="1" stroke="#bfbfbf" stroke-opacity="1" stroke-width="1" stroke-linecap="square" stroke-linejoin="bevel" transform="matrix(1,0,0,1,1234,270)" font-family="MS Shell Dlg 2" font-size="9.75" font-weight="400" font-style="normal" > <path vector-effect="none" fill-rule="evenodd" d="M0,1.7 L42.5,1.7 L42.5,0 L50,10 L42.5,20 L42.5,18.3 L0,18.3 L0,1.7"/> </g> <g fill="none" stroke="#000000" stroke-opacity="1" stroke-width="1" stroke-linecap="square" stroke-linejoin="bevel" transform="matrix(1,0,0,1,1247.13,268)" font-family="MS Shell Dlg 2" font-size="9.75" font-weight="400" font-style="normal" > <text fill="#000000" fill-opacity="1" stroke="none" xml:space="preserve" x="4" y="17" font-family="Arial" font-size="10pt" font-weight="700" font-style="italic" >V</text> </g> <g fill="#ffff33" fill-opacity="1" stroke="#bfbfbf" stroke-opacity="1" stroke-width="1" stroke-linecap="square" stroke-linejoin="bevel" transform="matrix(1,0,0,1,1285,270)" font-family="MS Shell Dlg 2" font-size="9.75" font-weight="400" font-style="normal" > <rect x="0" y="0" width="50" height="20"/> </g> <g fill="none" stroke="#000000" stroke-opacity="1" stroke-width="1" stroke-linecap="square" stroke-linejoin="bevel" transform="matrix(1,0,0,1,1292,268)" font-family="MS Shell Dlg 2" font-size="9.75" font-weight="400" font-style="normal" > <text fill="#000000" fill-opacity="1" stroke="none" xml:space="preserve" x="4" y="17" font-family="Arial" font-size="10pt" font-weight="700" font-style="italic" >nad4</text> </g> <g fill="#ffff33" fill-opacity="1" stroke="#bfbfbf" stroke-opacity="1" stroke-width="1" stroke-linecap="square" stroke-

linejoin="bevel" transform="matrix(1,0,0,1,1336,270)" font-family="MS Shell Dlg 2" font-size="9.75" font-weight="400" font-style="normal" > <rect x="0" y="0" width="50" height="20"/> </g> <g fill="none" stroke="#000000" stroke-opacity="1" stroke-width="1" stroke-linecap="square" stroke-linejoin="bevel" transform="matrix(1,0,0,1,1344,268)" font-family="MS Shell Dlg 2" font-size="9.75" font-weight="400" font-style="normal" > <text fill="#000000" fill-opacity="1" stroke="none" xml:space="preserve" x="4" y="17" font-family="Arial" font-size="10pt" font-weight="700" font-style="italic" >cox3</text> </g> <g fill="#ff9999" fill-opacity="1" stroke="#bfbfbf" stroke-opacity="1" stroke-width="1" stroke-linecap="square" stroke-linejoin="bevel" transform="matrix(1,0,0,1,1387,270)" font-family="MS Shell Dlg 2" font-size="9.75" font-weight="400" font-style="normal" > <path vector-effect="none" fill-rule="evenodd" d="M0,1.7 L42.5,1.7 L42.5,0 L50,10 L42.5,20 L42.5,18.3 L0,18.3 L0,1.7"/> </g> <g fill="none" stroke="#000000" stroke-opacity="1" stroke-width="1" stroke-linecap="square" stroke-linejoin="bevel" transform="matrix(1,0,0,1,1400.13,268)" font-family="MS Shell Dlg 2" font-size="9.75" font-weight="400" font-style="normal" > <text fill="#000000" fill-opacity="1" stroke="none" xml:space="preserve" x="4" y="17" font-family="Arial" font-size="10pt" font-weight="700" font-style="italic" >S</text> </g> <g fill="#ff9999" fill-opacity="1" stroke="#bfbfbf" stroke-opacity="1" stroke-width="1" stroke-linecap="square" stroke-linejoin="bevel" transform="matrix(1,0,0,1,1438,270)" font-family="MS Shell Dlg 2" font-size="9.75" font-weight="400" font-style="normal" > <path vector-effect="none" fill-rule="evenodd" d="M0,1.7 L42.5,1.7 L42.5,0 L50,10 L42.5,20 L42.5,18.3 L0,18.3 L0,1.7"/> </g> <g fill="none" stroke="#000000" stroke-opacity="1" stroke-width="1" stroke-linecap="square" stroke-linejoin="bevel" transform="matrix(1,0,0,1,1450.13,268)" font-family="MS Shell Dlg 2" font-size="9.75" font-weight="400" font-style="normal" > <text fill="#000000" fill-opacity="1" stroke="none" xml:space="preserve" x="4" y="17" font-family="Arial" font-size="10pt" font-weight="700" font-style="italic" >M</text> </g> <g fill="#ffff33" fill-opacity="1" stroke="#bfbfbf" stroke-opacity="1" stroke-width="1" stroke-linecap="square" stroke-linejoin="bevel" transform="matrix(1,0,0,1,1489,270)" font-family="MS Shell Dlg 2" font-size="9.75" font-weight="400" font-style="normal" > <rect x="0" y="0" width="50" height="20"/> </g> <g fill="none" stroke="#000000" stroke-opacity="1" stroke-width="1" stroke-linecap="square" stroke-linejoin="bevel" transform="matrix(1,0,0,1,1496,268)" font-family="MS Shell Dlg 2" font-size="9.75" font-weight="400" font-style="normal" > <text fill="#000000" fill-opacity="1" stroke="none" xml:space="preserve" x="4" y="17" font-family="Arial" font-size="10pt" font-weight="700" font-style="italic" >nad2</text> </g> <g fill="#ff9999" fill-opacity="1" stroke="#bfbfbf" stroke-opacity="1" stroke-width="1" stroke-linecap="square" stroke-linejoin="bevel" transform="matrix(1,0,0,1,1540,270)" font-family="MS Shell Dlg 2" font-size="9.75" font-weight="400" font-style="normal" > <path vector-effect="none" fill-rule="evenodd" d="M0,1.7 L42.5,1.7 L42.5,0 L50,10 L42.5,20 L42.5,18.3 L0,18.3 L0,1.7"/> </g> <g fill="none" stroke="#000000" stroke-opacity="1" stroke-width="1" stroke-linecap="square" stroke-linejoin="bevel" transform="matrix(1,0,0,1,1553.13,268)" font-family="MS Shell Dlg 2" font-size="9.75" font-weight="400" font-style="normal" > <text fill="#000000" fill-opacity="1" stroke="none" xml:space="preserve" x="4" y="17" font-family="Arial" font-size="10pt" font-weight="700" font-style="italic" >R</text> </g> <g fill="#ff9999" fill-opacity="1" stroke="#bfbfbf" stroke-opacity="1" stroke-width="1" stroke-linecap="square" stroke-linejoin="bevel" transform="matrix(1,0,0,1,1591,270)" font-family="MS Shell Dlg 2" font-size="9.75" font-weight="400" font-style="normal" > <path vector-effect="none" fill-rule="evenodd" d="M0,1.7 L42.5,1.7 L42.5,0 L50,10 L42.5,20 L42.5,18.3 L0,18.3 L0,1.7"/> </g> <g fill="none" stroke="#000000" stroke-opacity="1" stroke-width="1" stroke-linecap="square" stroke-linejoin="bevel" transform="matrix(1,0,0,1,1602.13,268)" font-family="MS Shell Dlg 2" font-size="9.75" font-weight="400" font-style="normal" > <text fill="#000000" fill-opacity="1" stroke="none" xml:space="preserve" x="4" y="17" font-family="Arial" font-size="10pt" font-weight="700" font-style="italic" >W</text> </g> <g fill="#ff9999" fill-opacity="1" stroke="#bfbfbf" stroke-opacity="1" stroke-width="1" stroke-linecap="square" stroke-linejoin="bevel" transform="matrix(1,0,0,1,1642,270)" font-family="MS Shell Dlg 2" font-size="9.75" font-weight="400" font-style="normal" > <path vector-effect="none" fill-rule="evenodd" d="M0,1.7 L42.5,1.7 L42.5,0 L50,10 L42.5,20 L42.5,18.3 L0,18.3 L0,1.7"/> </g> <g fill="none" stroke="#000000" stroke-opacity="1" stroke-width="1" stroke-linecap="square" stroke-linejoin="bevel" transform="matrix(1,0,0,1,1655.13,268)" font-family="MS Shell Dlg 2" font-size="9.75" font-weight="400" font-style="normal" > <text fill="#000000" fill-opacity="1" stroke="none" xml:space="preserve" x="4" y="17" font-family="Arial" font-size="10pt" font-weight="700" font-style="italic" >A</text> </g> <g fill="#ff9999" fill-opacity="1" stroke="#bfbfbf" stroke-opacity="1" stroke-width="1" stroke-linecap="square" stroke-linejoin="bevel" transform="matrix(1,0,0,1,1693,270)" font-family="MS Shell Dlg 2" font-size="9.75" font-weight="400" font-style="normal" > <path vector-effect="none" fill-rule="evenodd" d="M0,1.7 L42.5,1.7 L42.5,0 L50,10 L42.5,20 L42.5,18.3 L0,18.3 L0,1.7"/> </g> <g fill="none" stroke="#000000" stroke-opacity="1" stroke-width="1" stroke-linecap="square" stroke-linejoin="bevel" transform="matrix(1,0,0,1,1702.63,268)" font-family="MS Shell Dlg 2" font-size="9.75" font-weight="400" font-style="normal" > <text fill="#000000" fill-opacity="1" stroke="none" xml:space="preserve" x="4" y="17" font-

family="Arial" font-size="10pt" font-weight="700" font-style="italic" >S1</text> </g> <g fill="#ff9999"
fill-opacity="1" stroke="#bfbfbf" stroke-opacity="1" stroke-width="1" stroke-linecap="square" stroke-
linejoin="bevel" transform="matrix(1,0,0,1,1744,270)" font-family="MS Shell Dlg 2" font-size="9.75" font-
weight="400" font-style="normal" > <path vector-effect="none" fill-rule="evenodd" d="M0,1.7 L42.5,1.7 L42.5,0
L50,10 L42.5,20 L42.5,18.3 L0,18.3 L0,1.7"/> </g> <g fill="none" stroke="#000000" stroke-opacity="1"
stroke-width="1" stroke-linecap="square" stroke-linejoin="bevel" transform="matrix(1,0,0,1,1757.13,268)" font-
family="MS Shell Dlg 2" font-size="9.75" font-weight="400" font-style="normal" > <text fill="#000000" fill-
opacity="1" stroke="none" xml:space="preserve" x="4" y="17" font-family="Arial" font-size="10pt" font-
weight="700" font-style="italic" >H</text> </g> <g fill="#ff9999" fill-opacity="1" stroke="#bfbfbf"
stroke-opacity="1" stroke-width="1" stroke-linecap="square" stroke-linejoin="bevel"
transform="matrix(1,0,0,1,1795,270)" font-family="MS Shell Dlg 2" font-size="9.75" font-weight="400" font-
style="normal" > <path vector-effect="none" fill-rule="evenodd" d="M0,1.7 L42.5,1.7 L42.5,0 L50,10 L42.5,20
L42.5,18.3 L0,18.3 L0,1.7"/> </g> <g fill="none" stroke="#000000" stroke-opacity="1" stroke-width="1"
stroke-linecap="square" stroke-linejoin="bevel" transform="matrix(1,0,0,1,1808.63,268)" font-family="MS Shell
Dlg 2" font-size="9.75" font-weight="400" font-style="normal" > <text fill="#000000" fill-opacity="1"
stroke="none" xml:space="preserve" x="4" y="17" font-family="Arial" font-size="10pt" font-weight="700" font-
style="italic" >P</text> </g> <g fill="#ffff33" fill-opacity="1" stroke="#bfbfbf" stroke-opacity="1"
stroke-width="1" stroke-linecap="square" stroke-linejoin="bevel" transform="matrix(1,0,0,1,1846,270)" font-
family="MS Shell Dlg 2" font-size="9.75" font-weight="400" font-style="normal" > <rect x="0" y="0" width="50"
height="20"/> </g> <g fill="none" stroke="#000000" stroke-opacity="1" stroke-width="1" stroke-
linecap="square" stroke-linejoin="bevel" transform="matrix(1,0,0,1,1853,268)" font-family="MS Shell Dlg 2" font-
size="9.75" font-weight="400" font-style="normal" > <text fill="#000000" fill-opacity="1" stroke="none"
xml:space="preserve" x="4" y="17" font-family="Arial" font-size="10pt" font-weight="700" font-style="italic"
>nad3</text> </g> <g fill="#ffff33" fill-opacity="1" stroke="#bfbfbf" stroke-opacity="1" stroke-width="1"
stroke-linecap="square" stroke-linejoin="bevel" transform="matrix(1,0,0,1,1897,270)" font-family="MS Shell Dlg
2" font-size="9.75" font-weight="400" font-style="normal" > <rect x="0" y="0" width="50" height="20"/> </g>
<g fill="none" stroke="#000000" stroke-opacity="1" stroke-width="1" stroke-linecap="square" stroke-
linejoin="bevel" transform="matrix(1,0,0,1,1905.5,268)" font-family="MS Shell Dlg 2" font-size="9.75" font-
weight="400" font-style="normal" > <text fill="#000000" fill-opacity="1" stroke="none" xml:space="preserve"
x="4" y="17" font-family="Arial" font-size="10pt" font-weight="700" font-style="italic" >atp8</text> </g>
<g fill="none" stroke="#000000" stroke-opacity="1" stroke-width="1" stroke-linecap="square" stroke-
linejoin="bevel" transform="matrix(1,0,0,1,1948,270)" font-family="MS Shell Dlg 2" font-size="9.75" font-
weight="400" font-style="normal" > <text fill="#000000" fill-opacity="1" stroke="none" xml:space="preserve"
x="4" y="17" font-family="Arial" font-size="10pt" font-weight="700" font-style="italic" >(+)</text> </g>
<g fill="none" stroke="#000000" stroke-opacity="1" stroke-width="1" stroke-linecap="square" stroke-
linejoin="bevel" transform="matrix(1,0,0,1,10,310)" font-family="MS Shell Dlg 2" font-size="9.75" font-
weight="400" font-style="normal" > <text fill="#000000" fill-opacity="1" stroke="none" xml:space="preserve"
x="4" y="17" font-family="Arial" font-size="10pt" font-weight="700" font-style="italic"
>Mytilus_edulis_MF407676</text> </g> <g fill="#ffff33" fill-opacity="1" stroke="#bfbfbf" stroke-
opacity="1" stroke-width="1" stroke-linecap="square" stroke-linejoin="bevel" transform="matrix(1,0,0,1,10,340)"
font-family="MS Shell Dlg 2" font-size="9.75" font-weight="400" font-style="normal" > <rect x="0" y="0"
width="50" height="20"/> </g> <g fill="none" stroke="#000000" stroke-opacity="1" stroke-width="1" stroke-
linecap="square" stroke-linejoin="bevel" transform="matrix(1,0,0,1,18,338)" font-family="MS Shell Dlg 2" font-
size="9.75" font-weight="400" font-style="normal" > <text fill="#000000" fill-opacity="1" stroke="none"
xml:space="preserve" x="4" y="17" font-family="Arial" font-size="10pt" font-weight="700" font-style="italic"
>cox1</text> </g> <g fill="#ffff33" fill-opacity="1" stroke="#bfbfbf" stroke-opacity="1" stroke-width="1"
stroke-linecap="square" stroke-linejoin="bevel" transform="matrix(1,0,0,1,61,340)" font-family="MS Shell Dlg 2"
font-size="9.75" font-weight="400" font-style="normal" > <rect x="0" y="0" width="50" height="20"/> </g>
<g fill="none" stroke="#000000" stroke-opacity="1" stroke-width="1" stroke-linecap="square" stroke-
linejoin="bevel" transform="matrix(1,0,0,1,69.5,338)" font-family="MS Shell Dlg 2" font-size="9.75" font-
weight="400" font-style="normal" > <text fill="#000000" fill-opacity="1" stroke="none" xml:space="preserve"
x="4" y="17" font-family="Arial" font-size="10pt" font-weight="700" font-style="italic" >atp6</text> </g>
<g fill="#ff9999" fill-opacity="1" stroke="#bfbfbf" stroke-opacity="1" stroke-width="1" stroke-linecap="square"
stroke-linejoin="bevel" transform="matrix(1,0,0,1,112,340)" font-family="MS Shell Dlg 2" font-size="9.75" font-
weight="400" font-style="normal" > <path vector-effect="none" fill-rule="evenodd" d="M0,1.7 L42.5,1.7 L42.5,0
L50,10 L42.5,20 L42.5,18.3 L0,18.3 L0,1.7"/> </g> <g fill="none" stroke="#000000" stroke-opacity="1"
stroke-width="1" stroke-linecap="square" stroke-linejoin="bevel" transform="matrix(1,0,0,1,125.132,338)" font-
family="MS Shell Dlg 2" font-size="9.75" font-weight="400" font-style="normal" > <text fill="#000000" fill-

opacity="1" stroke="none" xml:space="preserve" x="4" y="17" font-family="Arial" font-size="10pt" font-weight="700" font-style="italic" >T</text> </g> <g fill="#ffff33" fill-opacity="1" stroke="#bfbfbf" stroke-opacity="1" stroke-width="1" stroke-linecap="square" stroke-linejoin="bevel" transform="matrix(1,0,0,1,163,340)" font-family="MS Shell Dlg 2" font-size="9.75" font-weight="400" font-style="normal" > <rect x="0" y="0" width="50" height="20"/> </g> <g fill="none" stroke="#000000" stroke-opacity="1" stroke-width="1" stroke-linecap="square" stroke-linejoin="bevel" transform="matrix(1,0,0,1,167,338)" font-family="MS Shell Dlg 2" font-size="9.75" font-weight="400" font-style="normal" > <text fill="#000000" fill-opacity="1" stroke="none" xml:space="preserve" x="4" y="17" font-family="Arial" font-size="10pt" font-weight="700" font-style="italic" >nad4L</text> </g> <g fill="#ffff33" fill-opacity="1" stroke="#bfbfbf" stroke-opacity="1" stroke-width="1" stroke-linecap="square" stroke-linejoin="bevel" transform="matrix(1,0,0,1,214,340)" font-family="MS Shell Dlg 2" font-size="9.75" font-weight="400" font-style="normal" > <rect x="0" y="0" width="50" height="20"/> </g> <g fill="none" stroke="#000000" stroke-opacity="1" stroke-width="1" stroke-linecap="square" stroke-linejoin="bevel" transform="matrix(1,0,0,1,221,338)" font-family="MS Shell Dlg 2" font-size="9.75" font-weight="400" font-style="normal" > <text fill="#000000" fill-opacity="1" stroke="none" xml:space="preserve" x="4" y="17" font-family="Arial" font-size="10pt" font-weight="700" font-style="italic" >nad5</text> </g> <g fill="#ffff33" fill-opacity="1" stroke="#bfbfbf" stroke-opacity="1" stroke-width="1" stroke-linecap="square" stroke-linejoin="bevel" transform="matrix(1,0,0,1,265,340)" font-family="MS Shell Dlg 2" font-size="9.75" font-weight="400" font-style="normal" > <rect x="0" y="0" width="50" height="20"/> </g> <g fill="none" stroke="#000000" stroke-opacity="1" stroke-width="1" stroke-linecap="square" stroke-linejoin="bevel" transform="matrix(1,0,0,1,272,338)" font-family="MS Shell Dlg 2" font-size="9.75" font-weight="400" font-style="normal" > <text fill="#000000" fill-opacity="1" stroke="none" xml:space="preserve" x="4" y="17" font-family="Arial" font-size="10pt" font-weight="700" font-style="italic" >nad6</text> </g> <g fill="#ff9999" fill-opacity="1" stroke="#bfbfbf" stroke-opacity="1" stroke-width="1" stroke-linecap="square" stroke-linejoin="bevel" transform="matrix(1,0,0,1,316,340)" font-family="MS Shell Dlg 2" font-size="9.75" font-weight="400" font-style="normal" > <path vector-effect="none" fill-rule="evenodd" d="M0,1.7 L42.5,1.7 L42.5,0 L50,10 L42.5,20 L42.5,18.3 L0,18.3 L0,1.7"/> </g> <g fill="none" stroke="#000000" stroke-opacity="1" stroke-width="1" stroke-linecap="square" stroke-linejoin="bevel" transform="matrix(1,0,0,1,329.632,338)" font-family="MS Shell Dlg 2" font-size="9.75" font-weight="400" font-style="normal" > <text fill="#000000" fill-opacity="1" stroke="none" xml:space="preserve" x="4" y="17" font-family="Arial" font-size="10pt" font-weight="700" font-style="italic" >F</text> </g> <g fill="#99ffff" fill-opacity="1" stroke="#bfbfbf" stroke-opacity="1" stroke-width="1" stroke-linecap="square" stroke-linejoin="bevel" transform="matrix(1,0,0,1,367,340)" font-family="MS Shell Dlg 2" font-size="9.75" font-weight="400" font-style="normal" > <path vector-effect="none" fill-rule="evenodd" d="M0,5 C0,2.23858 2.23858,0 5,0 L45,0 C47.7614,0 50,2.23858 50,5 L50,15 C50,17.7614 47.7614,20 45,20 L5,20 C2.23858,20 0,17.7614 0,15 L0,5"/> </g> <g fill="none" stroke="#000000" stroke-opacity="1" stroke-width="1" stroke-linecap="square" stroke-linejoin="bevel" transform="matrix(1,0,0,1,375.5,338)" font-family="MS Shell Dlg 2" font-size="9.75" font-weight="400" font-style="normal" > <text fill="#000000" fill-opacity="1" stroke="none" xml:space="preserve" x="4" y="17" font-family="Arial" font-size="10pt" font-weight="700" font-style="italic" >rrnS</text> </g> <g fill="#ff9999" fill-opacity="1" stroke="#bfbfbf" stroke-opacity="1" stroke-width="1" stroke-linecap="square" stroke-linejoin="bevel" transform="matrix(1,0,0,1,418,340)" font-family="MS Shell Dlg 2" font-size="9.75" font-weight="400" font-style="normal" > <path vector-effect="none" fill-rule="evenodd" d="M0,1.7 L42.5,1.7 L42.5,0 L50,10 L42.5,20 L42.5,18.3 L0,18.3 L0,1.7"/> </g> <g fill="none" stroke="#000000" stroke-opacity="1" stroke-width="1" stroke-linecap="square" stroke-linejoin="bevel" transform="matrix(1,0,0,1,431.132,338)" font-family="MS Shell Dlg 2" font-size="9.75" font-weight="400" font-style="normal" > <text fill="#000000" fill-opacity="1" stroke="none" xml:space="preserve" x="4" y="17" font-family="Arial" font-size="10pt" font-weight="700" font-style="italic" >G</text> </g> <g fill="#ff9999" fill-opacity="1" stroke="#bfbfbf" stroke-opacity="1" stroke-width="1" stroke-linecap="square" stroke-linejoin="bevel" transform="matrix(1,0,0,1,469,340)" font-family="MS Shell Dlg 2" font-size="9.75" font-weight="400" font-style="normal" > <path vector-effect="none" fill-rule="evenodd" d="M0,1.7 L42.5,1.7 L42.5,0 L50,10 L42.5,20 L42.5,18.3 L0,18.3 L0,1.7"/> </g> <g fill="none" stroke="#000000" stroke-opacity="1" stroke-width="1" stroke-linecap="square" stroke-linejoin="bevel" transform="matrix(1,0,0,1,482.132,338)" font-family="MS Shell Dlg 2" font-size="9.75" font-weight="400" font-style="normal" > <text fill="#000000" fill-opacity="1" stroke="none" xml:space="preserve" x="4" y="17" font-family="Arial" font-size="10pt" font-weight="700" font-style="italic" >N</text> </g> <g fill="#ff9999" fill-opacity="1" stroke="#bfbfbf" stroke-opacity="1" stroke-width="1" stroke-linecap="square" stroke-linejoin="bevel" transform="matrix(1,0,0,1,520,340)" font-family="MS Shell Dlg 2" font-size="9.75" font-weight="400" font-style="normal" > <path vector-effect="none" fill-rule="evenodd" d="M0,1.7 L42.5,1.7 L42.5,0 L50,10 L42.5,20 L42.5,18.3 L0,18.3 L0,1.7"/> </g> <g

fill="none" stroke="#000000" stroke-opacity="1" stroke-width="1" stroke-linecap="square" stroke-linejoin="bevel" transform="matrix(1,0,0,1,533.633,338)" font-family="MS Shell Dlg 2" font-size="9.75" font-weight="400" font-style="normal" > <text fill="#000000" fill-opacity="1" stroke="none" xml:space="preserve" x="4" y="17" font-family="Arial" font-size="10pt" font-weight="700" font-style="italic" >E</text> </g> <g fill="#ff9999" fill-opacity="1" stroke="#bfbfbf" stroke-opacity="1" stroke-width="1" stroke-linecap="square" stroke-linejoin="bevel" transform="matrix(1,0,0,1,571,340)" font-family="MS Shell Dlg 2" font-size="9.75" font-weight="400" font-style="normal" > <path vector-effect="none" fill-rule="evenodd" d="M0,1.7 L42.5,1.7 L42.5,0 L50,10 L42.5,20 L42.5,18.3 L0,18.3 L0,1.7"/> </g> <g fill="none" stroke="#000000" stroke-opacity="1" stroke-width="1" stroke-linecap="square" stroke-linejoin="bevel" transform="matrix(1,0,0,1,584.133,338)" font-family="MS Shell Dlg 2" font-size="9.75" font-weight="400" font-style="normal" > <text fill="#000000" fill-opacity="1" stroke="none" xml:space="preserve" x="4" y="17" font-family="Arial" font-size="10pt" font-weight="700" font-style="italic" >C</text> </g> <g fill="#ff9999" fill-opacity="1" stroke="#bfbfbf" stroke-opacity="1" stroke-width="1" stroke-linecap="square" stroke-linejoin="bevel" transform="matrix(1,0,0,1,622,340)" font-family="MS Shell Dlg 2" font-size="9.75" font-weight="400" font-style="normal" > <path vector-effect="none" fill-rule="evenodd" d="M0,1.7 L42.5,1.7 L42.5,0 L50,10 L42.5,20 L42.5,18.3 L0,18.3 L0,1.7"/> </g> <g fill="none" stroke="#000000" stroke-opacity="1" stroke-width="1" stroke-linecap="square" stroke-linejoin="bevel" transform="matrix(1,0,0,1,637.133,338)" font-family="MS Shell Dlg 2" font-size="9.75" font-weight="400" font-style="normal" > <text fill="#000000" fill-opacity="1" stroke="none" xml:space="preserve" x="4" y="17" font-family="Arial" font-size="10pt" font-weight="700" font-style="italic" >I</text> </g> <g fill="#ff9999" fill-opacity="1" stroke="#bfbfbf" stroke-opacity="1" stroke-width="1" stroke-linecap="square" stroke-linejoin="bevel" transform="matrix(1,0,0,1,673,340)" font-family="MS Shell Dlg 2" font-size="9.75" font-weight="400" font-style="normal" > <path vector-effect="none" fill-rule="evenodd" d="M0,1.7 L42.5,1.7 L42.5,0 L50,10 L42.5,20 L42.5,18.3 L0,18.3 L0,1.7"/> </g> <g fill="none" stroke="#000000" stroke-opacity="1" stroke-width="1" stroke-linecap="square" stroke-linejoin="bevel" transform="matrix(1,0,0,1,685.633,338)" font-family="MS Shell Dlg 2" font-size="9.75" font-weight="400" font-style="normal" > <text fill="#000000" fill-opacity="1" stroke="none" xml:space="preserve" x="4" y="17" font-family="Arial" font-size="10pt" font-weight="700" font-style="italic" >Q</text> </g> <g fill="#ff9999" fill-opacity="1" stroke="#bfbfbf" stroke-opacity="1" stroke-width="1" stroke-linecap="square" stroke-linejoin="bevel" transform="matrix(1,0,0,1,724,340)" font-family="MS Shell Dlg 2" font-size="9.75" font-weight="400" font-style="normal" > <path vector-effect="none" fill-rule="evenodd" d="M0,1.7 L42.5,1.7 L42.5,0 L50,10 L42.5,20 L42.5,18.3 L0,18.3 L0,1.7"/> </g> <g fill="none" stroke="#000000" stroke-opacity="1" stroke-width="1" stroke-linecap="square" stroke-linejoin="bevel" transform="matrix(1,0,0,1,737.133,338)" font-family="MS Shell Dlg 2" font-size="9.75" font-weight="400" font-style="normal" > <text fill="#000000" fill-opacity="1" stroke="none" xml:space="preserve" x="4" y="17" font-family="Arial" font-size="10pt" font-weight="700" font-style="italic" >D</text> </g> <g fill="#99ffff" fill-opacity="1" stroke="#bfbfbf" stroke-opacity="1" stroke-width="1" stroke-linecap="square" stroke-linejoin="bevel" transform="matrix(1,0,0,1,775,340)" font-family="MS Shell Dlg 2" font-size="9.75" font-weight="400" font-style="normal" > <path vector-effect="none" fill-rule="evenodd" d="M0,5 C0,2.23858 2.23858,0 5,0 L45,0 C47.7614,0 50,2.23858 50,5 L50,15 C50,17.7614 47.7614,20 45,20 L5,20 C2.23858,20 0,17.7614 0,15 L0,5"/> </g> <g fill="none" stroke="#000000" stroke-opacity="1" stroke-width="1" stroke-linecap="square" stroke-linejoin="bevel" transform="matrix(1,0,0,1,784.5,338)" font-family="MS Shell Dlg 2" font-size="9.75" font-weight="400" font-style="normal" > <text fill="#000000" fill-opacity="1" stroke="none" xml:space="preserve" x="4" y="17" font-family="Arial" font-size="10pt" font-weight="700" font-style="italic" >rrnL</text> </g> <g fill="#ff9999" fill-opacity="1" stroke="#bfbfbf" stroke-opacity="1" stroke-width="1" stroke-linecap="square" stroke-linejoin="bevel" transform="matrix(1,0,0,1,826,340)" font-family="MS Shell Dlg 2" font-size="9.75" font-weight="400" font-style="normal" > <path vector-effect="none" fill-rule="evenodd" d="M0,1.7 L42.5,1.7 L42.5,0 L50,10 L42.5,20 L42.5,18.3 L0,18.3 L0,1.7"/> </g> <g fill="none" stroke="#000000" stroke-opacity="1" stroke-width="1" stroke-linecap="square" stroke-linejoin="bevel" transform="matrix(1,0,0,1,839.633,338)" font-family="MS Shell Dlg 2" font-size="9.75" font-weight="400" font-style="normal" > <text fill="#000000" fill-opacity="1" stroke="none" xml:space="preserve" x="4" y="17" font-family="Arial" font-size="10pt" font-weight="700" font-style="italic" >Y</text> </g> <g fill="#ffff33" fill-opacity="1" stroke="#bfbfbf" stroke-opacity="1" stroke-width="1" stroke-linecap="square" stroke-linejoin="bevel" transform="matrix(1,0,0,1,877,340)" font-family="MS Shell Dlg 2" font-size="9.75" font-weight="400" font-style="normal" > <rect x="0" y="0" width="50" height="20"/> </g> <g fill="none" stroke="#000000" stroke-opacity="1" stroke-width="1" stroke-linecap="square" stroke-linejoin="bevel" transform="matrix(1,0,0,1,886.5,338)" font-family="MS Shell Dlg 2" font-size="9.75" font-weight="400" font-style="normal" > <text fill="#000000" fill-opacity="1" stroke="none" xml:space="preserve" x="4" y="17" font-family="Arial" font-size="10pt" font-weight="700" font-style="italic" >cytb</text> </g> <g fill="#ffff33"

fill-opacity="1" stroke="#bfbfbf" stroke-opacity="1" stroke-width="1" stroke-linecap="square" stroke-
linejoin="bevel" transform="matrix(1,0,0,1,928,340)" font-family="MS Shell Dlg 2" font-size="9.75" font-
weight="400" font-style="normal" > <rect x="0" y="0" width="50" height="20"/> </g> <g fill="none"
stroke="#000000" stroke-opacity="1" stroke-width="1" stroke-linecap="square" stroke-linejoin="bevel"
transform="matrix(1,0,0,1,936,338)" font-family="MS Shell Dlg 2" font-size="9.75" font-weight="400" font-
style="normal" > <text fill="#000000" fill-opacity="1" stroke="none" xml:space="preserve" x="4" y="17" font-
family="Arial" font-size="10pt" font-weight="700" font-style="italic" >cox2</text> </g> <g
fill="#ff9999" fill-opacity="1" stroke="#bfbfbf" stroke-opacity="1" stroke-width="1" stroke-linecap="square"
stroke-linejoin="bevel" transform="matrix(1,0,0,1,979,340)" font-family="MS Shell Dlg 2" font-size="9.75" font-
weight="400" font-style="normal" > <path vector-effect="none" fill-rule="evenodd" d="M0,1.7 L42.5,1.7 L42.5,0
L50,10 L42.5,20 L42.5,18.3 L0,18.3 L0,1.7"/> </g> <g fill="none" stroke="#000000" stroke-opacity="1"
stroke-width="1" stroke-linecap="square" stroke-linejoin="bevel" transform="matrix(1,0,0,1,992.633,338)" font-
family="MS Shell Dlg 2" font-size="9.75" font-weight="400" font-style="normal" > <text fill="#000000" fill-
opacity="1" stroke="none" xml:space="preserve" x="4" y="17" font-family="Arial" font-size="10pt" font-
weight="700" font-style="italic" >K</text> </g> <g fill="#ff9999" fill-opacity="1" stroke="#bfbfbf"
stroke-opacity="1" stroke-width="1" stroke-linecap="square" stroke-linejoin="bevel"
transform="matrix(1,0,0,1,1030,340)" font-family="MS Shell Dlg 2" font-size="9.75" font-weight="400" font-
style="normal" > <path vector-effect="none" fill-rule="evenodd" d="M0,1.7 L42.5,1.7 L42.5,0 L50,10 L42.5,20
L42.5,18.3 L0,18.3 L0,1.7"/> </g> <g fill="none" stroke="#000000" stroke-opacity="1" stroke-width="1"
stroke-linecap="square" stroke-linejoin="bevel" transform="matrix(1,0,0,1,1042.13,338)" font-family="MS Shell
Dlg 2" font-size="9.75" font-weight="400" font-style="normal" > <text fill="#000000" fill-opacity="1"
stroke="none" xml:space="preserve" x="4" y="17" font-family="Arial" font-size="10pt" font-weight="700" font-
style="italic" >M</text> </g> <g fill="#ff9999" fill-opacity="1" stroke="#bfbfbf" stroke-opacity="1"
stroke-width="1" stroke-linecap="square" stroke-linejoin="bevel" transform="matrix(1,0,0,1,1081,340)" font-
family="MS Shell Dlg 2" font-size="9.75" font-weight="400" font-style="normal" > <path vector-effect="none"
fill-rule="evenodd" d="M0,1.7 L42.5,1.7 L42.5,0 L50,10 L42.5,20 L42.5,18.3 L0,18.3 L0,1.7"/> </g> <g
fill="none" stroke="#000000" stroke-opacity="1" stroke-width="1" stroke-linecap="square" stroke-linejoin="bevel"
transform="matrix(1,0,0,1,1095.13,338)" font-family="MS Shell Dlg 2" font-size="9.75" font-weight="400" font-
style="normal" > <text fill="#000000" fill-opacity="1" stroke="none" xml:space="preserve" x="4" y="17" font-
family="Arial" font-size="10pt" font-weight="700" font-style="italic" >L</text> </g> <g fill="#ff9999"
fill-opacity="1" stroke="#bfbfbf" stroke-opacity="1" stroke-width="1" stroke-linecap="square" stroke-
linejoin="bevel" transform="matrix(1,0,0,1,1132,340)" font-family="MS Shell Dlg 2" font-size="9.75" font-
weight="400" font-style="normal" > <path vector-effect="none" fill-rule="evenodd" d="M0,1.7 L42.5,1.7 L42.5,0
L50,10 L42.5,20 L42.5,18.3 L0,18.3 L0,1.7"/> </g> <g fill="none" stroke="#000000" stroke-opacity="1"
stroke-width="1" stroke-linecap="square" stroke-linejoin="bevel" transform="matrix(1,0,0,1,1146.13,338)" font-
family="MS Shell Dlg 2" font-size="9.75" font-weight="400" font-style="normal" > <text fill="#000000" fill-
opacity="1" stroke="none" xml:space="preserve" x="4" y="17" font-family="Arial" font-size="10pt" font-
weight="700" font-style="italic" >L</text> </g> <g fill="#ffff33" fill-opacity="1" stroke="#bfbfbf"
stroke-opacity="1" stroke-width="1" stroke-linecap="square" stroke-linejoin="bevel"
transform="matrix(1,0,0,1,1183,340)" font-family="MS Shell Dlg 2" font-size="9.75" font-weight="400" font-
style="normal" > <rect x="0" y="0" width="50" height="20"/> </g> <g fill="none" stroke="#000000" stroke-
opacity="1" stroke-width="1" stroke-linecap="square" stroke-linejoin="bevel"
transform="matrix(1,0,0,1,1190,338)" font-family="MS Shell Dlg 2" font-size="9.75" font-weight="400" font-
style="normal" > <text fill="#000000" fill-opacity="1" stroke="none" xml:space="preserve" x="4" y="17" font-
family="Arial" font-size="10pt" font-weight="700" font-style="italic" >nad1</text> </g> <g
fill="#ff9999" fill-opacity="1" stroke="#bfbfbf" stroke-opacity="1" stroke-width="1" stroke-linecap="square"
stroke-linejoin="bevel" transform="matrix(1,0,0,1,1234,340)" font-family="MS Shell Dlg 2" font-size="9.75" font-
weight="400" font-style="normal" > <path vector-effect="none" fill-rule="evenodd" d="M0,1.7 L42.5,1.7 L42.5,0
L50,10 L42.5,20 L42.5,18.3 L0,18.3 L0,1.7"/> </g> <g fill="none" stroke="#000000" stroke-opacity="1"
stroke-width="1" stroke-linecap="square" stroke-linejoin="bevel" transform="matrix(1,0,0,1,1247.13,338)" font-
family="MS Shell Dlg 2" font-size="9.75" font-weight="400" font-style="normal" > <text fill="#000000" fill-
opacity="1" stroke="none" xml:space="preserve" x="4" y="17" font-family="Arial" font-size="10pt" font-
weight="700" font-style="italic" >V</text> </g> <g fill="#ffff33" fill-opacity="1" stroke="#bfbfbf"
stroke-opacity="1" stroke-width="1" stroke-linecap="square" stroke-linejoin="bevel"
transform="matrix(1,0,0,1,1285,340)" font-family="MS Shell Dlg 2" font-size="9.75" font-weight="400" font-
style="normal" > <rect x="0" y="0" width="50" height="20"/> </g> <g fill="none" stroke="#000000" stroke-
opacity="1" stroke-width="1" stroke-linecap="square" stroke-linejoin="bevel"
transform="matrix(1,0,0,1,1292,338)" font-family="MS Shell Dlg 2" font-size="9.75" font-weight="400" font-

style="normal" > <text fill="#000000" fill-opacity="1" stroke="none" xml:space="preserve" x="4" y="17" font-family="Arial" font-size="10pt" font-weight="700" font-style="italic"   >nad4</text> </g>   <g fill="#ffff33" fill-opacity="1" stroke="#bfbfbf" stroke-opacity="1" stroke-width="1" stroke-linecap="square" stroke-linejoin="bevel" transform="matrix(1,0,0,1,1336,340)" font-family="MS Shell Dlg 2" font-size="9.75" font-weight="400" font-style="normal" > <rect x="0" y="0" width="50" height="20"/> </g>   <g fill="none" stroke="#000000" stroke-opacity="1" stroke-width="1" stroke-linecap="square" stroke-linejoin="bevel" transform="matrix(1,0,0,1,1344,338)" font-family="MS Shell Dlg 2" font-size="9.75" font-weight="400" font-style="normal" > <text fill="#000000" fill-opacity="1" stroke="none" xml:space="preserve" x="4" y="17" font-family="Arial" font-size="10pt" font-weight="700" font-style="italic"   >cox3</text> </g>   <g fill="#ff9999" fill-opacity="1" stroke="#bfbfbf" stroke-opacity="1" stroke-width="1" stroke-linecap="square" stroke-linejoin="bevel" transform="matrix(1,0,0,1,1387,340)" font-family="MS Shell Dlg 2" font-size="9.75" font-weight="400" font-style="normal" > <path vector-effect="none" fill-rule="evenodd" d="M0,1.7 L42.5,1.7 L42.5,0 L50,10 L42.5,20 L42.5,18.3 L0,18.3 L0,1.7"/> </g>   <g fill="none" stroke="#000000" stroke-opacity="1" stroke-width="1" stroke-linecap="square" stroke-linejoin="bevel" transform="matrix(1,0,0,1,1400.13,338)" font-family="MS Shell Dlg 2" font-size="9.75" font-weight="400" font-style="normal" > <text fill="#000000" fill-opacity="1" stroke="none" xml:space="preserve" x="4" y="17" font-family="Arial" font-size="10pt" font-weight="700" font-style="italic"   >S</text> </g>   <g fill="#ff9999" fill-opacity="1" stroke="#bfbfbf" stroke-opacity="1" stroke-width="1" stroke-linecap="square" stroke-linejoin="bevel" transform="matrix(1,0,0,1,1438,340)" font-family="MS Shell Dlg 2" font-size="9.75" font-weight="400" font-style="normal" > <path vector-effect="none" fill-rule="evenodd" d="M0,1.7 L42.5,1.7 L42.5,0 L50,10 L42.5,20 L42.5,18.3 L0,18.3 L0,1.7"/> </g>   <g fill="none" stroke="#000000" stroke-opacity="1" stroke-width="1" stroke-linecap="square" stroke-linejoin="bevel" transform="matrix(1,0,0,1,1450.13,338)" font-family="MS Shell Dlg 2" font-size="9.75" font-weight="400" font-style="normal" > <text fill="#000000" fill-opacity="1" stroke="none" xml:space="preserve" x="4" y="17" font-family="Arial" font-size="10pt" font-weight="700" font-style="italic"   >M</text> </g>   <g fill="#ffff33" fill-opacity="1" stroke="#bfbfbf" stroke-opacity="1" stroke-width="1" stroke-linecap="square" stroke-linejoin="bevel" transform="matrix(1,0,0,1,1489,340)" font-family="MS Shell Dlg 2" font-size="9.75" font-weight="400" font-style="normal" > <rect x="0" y="0" width="50" height="20"/> </g>   <g fill="none" stroke="#000000" stroke-opacity="1" stroke-width="1" stroke-linecap="square" stroke-linejoin="bevel" transform="matrix(1,0,0,1,1496,338)" font-family="MS Shell Dlg 2" font-size="9.75" font-weight="400" font-style="normal" > <text fill="#000000" fill-opacity="1" stroke="none" xml:space="preserve" x="4" y="17" font-family="Arial" font-size="10pt" font-weight="700" font-style="italic"   >nad2</text> </g>   <g fill="#ff9999" fill-opacity="1" stroke="#bfbfbf" stroke-opacity="1" stroke-width="1" stroke-linecap="square" stroke-linejoin="bevel" transform="matrix(1,0,0,1,1540,340)" font-family="MS Shell Dlg 2" font-size="9.75" font-weight="400" font-style="normal" > <path vector-effect="none" fill-rule="evenodd" d="M0,1.7 L42.5,1.7 L42.5,0 L50,10 L42.5,20 L42.5,18.3 L0,18.3 L0,1.7"/> </g>   <g fill="none" stroke="#000000" stroke-opacity="1" stroke-width="1" stroke-linecap="square" stroke-linejoin="bevel" transform="matrix(1,0,0,1,1553.13,338)" font-family="MS Shell Dlg 2" font-size="9.75" font-weight="400" font-style="normal" > <text fill="#000000" fill-opacity="1" stroke="none" xml:space="preserve" x="4" y="17" font-family="Arial" font-size="10pt" font-weight="700" font-style="italic"   >R</text> </g>   <g fill="#ff9999" fill-opacity="1" stroke="#bfbfbf" stroke-opacity="1" stroke-width="1" stroke-linecap="square" stroke-linejoin="bevel" transform="matrix(1,0,0,1,1591,340)" font-family="MS Shell Dlg 2" font-size="9.75" font-weight="400" font-style="normal" > <path vector-effect="none" fill-rule="evenodd" d="M0,1.7 L42.5,1.7 L42.5,0 L50,10 L42.5,20 L42.5,18.3 L0,18.3 L0,1.7"/> </g>   <g fill="none" stroke="#000000" stroke-opacity="1" stroke-width="1" stroke-linecap="square" stroke-linejoin="bevel" transform="matrix(1,0,0,1,1602.13,338)" font-family="MS Shell Dlg 2" font-size="9.75" font-weight="400" font-style="normal" > <text fill="#000000" fill-opacity="1" stroke="none" xml:space="preserve" x="4" y="17" font-family="Arial" font-size="10pt" font-weight="700" font-style="italic"   >W</text> </g>   <g fill="#ff9999" fill-opacity="1" stroke="#bfbfbf" stroke-opacity="1" stroke-width="1" stroke-linecap="square" stroke-linejoin="bevel" transform="matrix(1,0,0,1,1642,340)" font-family="MS Shell Dlg 2" font-size="9.75" font-weight="400" font-style="normal" > <path vector-effect="none" fill-rule="evenodd" d="M0,1.7 L42.5,1.7 L42.5,0 L50,10 L42.5,20 L42.5,18.3 L0,18.3 L0,1.7"/> </g>   <g fill="none" stroke="#000000" stroke-opacity="1" stroke-width="1" stroke-linecap="square" stroke-linejoin="bevel" transform="matrix(1,0,0,1,1655.13,338)" font-family="MS Shell Dlg 2" font-size="9.75" font-weight="400" font-style="normal" > <text fill="#000000" fill-opacity="1" stroke="none" xml:space="preserve" x="4" y="17" font-family="Arial" font-size="10pt" font-weight="700" font-style="italic"   >A</text> </g>   <g fill="#ff9999" fill-opacity="1" stroke="#bfbfbf" stroke-opacity="1" stroke-width="1" stroke-linecap="square" stroke-linejoin="bevel" transform="matrix(1,0,0,1,1693,340)" font-family="MS Shell Dlg 2" font-size="9.75" font-weight="400" font-style="normal" > <path vector-effect="none" fill-rule="evenodd" d="M0,1.7 L42.5,1.7 L42.5,0 L50,10 L42.5,20 L42.5,18.3 L0,18.3 L0,1.7"/> </g>   <g

fill="none" stroke="#000000" stroke-opacity="1" stroke-width="1" stroke-linecap="square" stroke-linejoin="bevel" transform="matrix(1,0,0,1,1706.13,338)" font-family="MS Shell Dlg 2" font-size="9.75" font-weight="400" font-style="normal" > <text fill="#000000" fill-opacity="1" stroke="none" xml:space="preserve" x="4" y="17" font-family="Arial" font-size="10pt" font-weight="700" font-style="italic" >S</text> </g> <g fill="#ff9999" fill-opacity="1" stroke="#bfbfbf" stroke-opacity="1" stroke-width="1" stroke-linecap="square" stroke-linejoin="bevel" transform="matrix(1,0,0,1,1744,340)" font-family="MS Shell Dlg 2" font-size="9.75" font-weight="400" font-style="normal" > <path vector-effect="none" fill-rule="evenodd" d="M0,1.7 L42.5,1.7 L42.5,0 L50,10 L42.5,20 L42.5,18.3 L0,18.3 L0,1.7"/> </g> <g fill="none" stroke="#000000" stroke-opacity="1" stroke-width="1" stroke-linecap="square" stroke-linejoin="bevel" transform="matrix(1,0,0,1,1757.13,338)" font-family="MS Shell Dlg 2" font-size="9.75" font-weight="400" font-style="normal" > <text fill="#000000" fill-opacity="1" stroke="none" xml:space="preserve" x="4" y="17" font-family="Arial" font-size="10pt" font-weight="700" font-style="italic" >H</text> </g> <g fill="#ff9999" fill-opacity="1" stroke="#bfbfbf" stroke-opacity="1" stroke-width="1" stroke-linecap="square" stroke-linejoin="bevel" transform="matrix(1,0,0,1,1795,340)" font-family="MS Shell Dlg 2" font-size="9.75" font-weight="400" font-style="normal" > <path vector-effect="none" fill-rule="evenodd" d="M0,1.7 L42.5,1.7 L42.5,0 L50,10 L42.5,20 L42.5,18.3 L0,18.3 L0,1.7"/> </g> <g fill="none" stroke="#000000" stroke-opacity="1" stroke-width="1" stroke-linecap="square" stroke-linejoin="bevel" transform="matrix(1,0,0,1,1808.63,338)" font-family="MS Shell Dlg 2" font-size="9.75" font-weight="400" font-style="normal" > <text fill="#000000" fill-opacity="1" stroke="none" xml:space="preserve" x="4" y="17" font-family="Arial" font-size="10pt" font-weight="700" font-style="italic" >P</text> </g> <g fill="#ffff33" fill-opacity="1" stroke="#bfbfbf" stroke-opacity="1" stroke-width="1" stroke-linecap="square" stroke-linejoin="bevel" transform="matrix(1,0,0,1,1846,340)" font-family="MS Shell Dlg 2" font-size="9.75" font-weight="400" font-style="normal" > <rect x="0" y="0" width="50" height="20"/> </g> <g fill="none" stroke="#000000" stroke-opacity="1" stroke-width="1" stroke-linecap="square" stroke-linejoin="bevel" transform="matrix(1,0,0,1,1853,338)" font-family="MS Shell Dlg 2" font-size="9.75" font-weight="400" font-style="normal" > <text fill="#000000" fill-opacity="1" stroke="none" xml:space="preserve" x="4" y="17" font-family="Arial" font-size="10pt" font-weight="700" font-style="italic" >nad3</text> </g> <g fill="#ffff33" fill-opacity="1" stroke="#bfbfbf" stroke-opacity="1" stroke-width="1" stroke-linecap="square" stroke-linejoin="bevel" transform="matrix(1,0,0,1,1897,340)" font-family="MS Shell Dlg 2" font-size="9.75" font-weight="400" font-style="normal" > <rect x="0" y="0" width="50" height="20"/> </g> <g fill="none" stroke="#000000" stroke-opacity="1" stroke-width="1" stroke-linecap="square" stroke-linejoin="bevel" transform="matrix(1,0,0,1,1905.5,338)" font-family="MS Shell Dlg 2" font-size="9.75" font-weight="400" font-style="normal" > <text fill="#000000" fill-opacity="1" stroke="none" xml:space="preserve" x="4" y="17" font-family="Arial" font-size="10pt" font-weight="700" font-style="italic" >atp8</text> </g> <g fill="none" stroke="#000000" stroke-opacity="1" stroke-width="1" stroke-linecap="square" stroke-linejoin="bevel" transform="matrix(1,0,0,1,1948,340)" font-family="MS Shell Dlg 2" font-size="9.75" font-weight="400" font-style="normal" > <text fill="#000000" fill-opacity="1" stroke="none" xml:space="preserve" x="4" y="17" font-family="Arial" font-size="10pt" font-weight="700" font-style="italic" >(+)</text> </g> <g fill="none" stroke="#000000" stroke-opacity="1" stroke-width="1" stroke-linecap="square" stroke-linejoin="bevel" transform="matrix(1,0,0,1,10,380)" font-family="MS Shell Dlg 2" font-size="9.75" font-weight="400" font-style="normal" > <text fill="#000000" fill-opacity="1" stroke="none" xml:space="preserve" x="4" y="17" font-family="Arial" font-size="10pt" font-weight="700" font-style="italic" >Mytilus_galloprovincialis_FJ890849</text> </g> <g fill="#ffff33" fill-opacity="1" stroke="#bfbfbf" stroke-opacity="1" stroke-width="1" stroke-linecap="square" stroke-linejoin="bevel" transform="matrix(1,0,0,1,10,410)" font-family="MS Shell Dlg 2" font-size="9.75" font-weight="400" font-style="normal" > <rect x="0" y="0" width="50" height="20"/> </g> <g fill="none" stroke="#000000" stroke-opacity="1" stroke-width="1" stroke-linecap="square" stroke-linejoin="bevel" transform="matrix(1,0,0,1,18,408)" font-family="MS Shell Dlg 2" font-size="9.75" font-weight="400" font-style="normal" > <text fill="#000000" fill-opacity="1" stroke="none" xml:space="preserve" x="4" y="17" font-family="Arial" font-size="10pt" font-weight="700" font-style="italic" >cox1</text> </g> <g fill="#ffff33" fill-opacity="1" stroke="#bfbfbf" stroke-opacity="1" stroke-width="1" stroke-linecap="square" stroke-linejoin="bevel" transform="matrix(1,0,0,1,61,410)" font-family="MS Shell Dlg 2" font-size="9.75" font-weight="400" font-style="normal" > <rect x="0" y="0" width="50" height="20"/> </g> <g fill="none" stroke="#000000" stroke-opacity="1" stroke-width="1" stroke-linecap="square" stroke-linejoin="bevel" transform="matrix(1,0,0,1,69.5,408)" font-family="MS Shell Dlg 2" font-size="9.75" font-weight="400" font-style="normal" > <text fill="#000000" fill-opacity="1" stroke="none" xml:space="preserve" x="4" y="17" font-family="Arial" font-size="10pt" font-weight="700" font-style="italic" >atp6</text> </g> <g fill="#ff9999" fill-opacity="1" stroke="#bfbfbf" stroke-opacity="1" stroke-width="1" stroke-linecap="square" stroke-linejoin="bevel" transform="matrix(1,0,0,1,112,410)" font-family="MS Shell Dlg 2" font-size="9.75" font-

weight="400" font-style="normal" > <path vector-effect="none" fill-rule="evenodd" d="M0,1.7 L42.5,1.7 L42.5,0 L50,10 L42.5,20 L42.5,18.3 L0,18.3 L0,1.7"/> </g> <g fill="none" stroke="#000000" stroke-opacity="1" stroke-width="1" stroke-linecap="square" stroke-linejoin="bevel" transform="matrix(1,0,0,1,125.132,408)" font-family="MS Shell Dlg 2" font-size="9.75" font-weight="400" font-style="normal" > <text fill="#000000" fill-opacity="1" stroke="none" xml:space="preserve" x="4" y="17" font-family="Arial" font-size="10pt" font-weight="700" font-style="italic" >T</text> </g> <g fill="#ffff33" fill-opacity="1" stroke="#bfbfbf" stroke-opacity="1" stroke-width="1" stroke-linecap="square" stroke-linejoin="bevel" transform="matrix(1,0,0,1,163,410)" font-family="MS Shell Dlg 2" font-size="9.75" font-weight="400" font-style="normal" > <rect x="0" y="0" width="50" height="20"/> </g> <g fill="none" stroke="#000000" stroke-opacity="1" stroke-width="1" stroke-linecap="square" stroke-linejoin="bevel" transform="matrix(1,0,0,1,167,408)" font-family="MS Shell Dlg 2" font-size="9.75" font-weight="400" font-style="normal" > <text fill="#000000" fill-opacity="1" stroke="none" xml:space="preserve" x="4" y="17" font-family="Arial" font-size="10pt" font-weight="700" font-style="italic" >nad4L</text> </g> <g fill="#ffff33" fill-opacity="1" stroke="#bfbfbf" stroke-opacity="1" stroke-width="1" stroke-linecap="square" stroke-linejoin="bevel" transform="matrix(1,0,0,1,214,410)" font-family="MS Shell Dlg 2" font-size="9.75" font-weight="400" font-style="normal" > <rect x="0" y="0" width="50" height="20"/> </g> <g fill="none" stroke="#000000" stroke-opacity="1" stroke-width="1" stroke-linecap="square" stroke-linejoin="bevel" transform="matrix(1,0,0,1,221,408)" font-family="MS Shell Dlg 2" font-size="9.75" font-weight="400" font-style="normal" > <text fill="#000000" fill-opacity="1" stroke="none" xml:space="preserve" x="4" y="17" font-family="Arial" font-size="10pt" font-weight="700" font-style="italic" >nad5</text> </g> <g fill="#ffff33" fill-opacity="1" stroke="#bfbfbf" stroke-opacity="1" stroke-width="1" stroke-linecap="square" stroke-linejoin="bevel" transform="matrix(1,0,0,1,265,410)" font-family="MS Shell Dlg 2" font-size="9.75" font-weight="400" font-style="normal" > <rect x="0" y="0" width="50" height="20"/> </g> <g fill="none" stroke="#000000" stroke-opacity="1" stroke-width="1" stroke-linecap="square" stroke-linejoin="bevel" transform="matrix(1,0,0,1,272,408)" font-family="MS Shell Dlg 2" font-size="9.75" font-weight="400" font-style="normal" > <text fill="#000000" fill-opacity="1" stroke="none" xml:space="preserve" x="4" y="17" font-family="Arial" font-size="10pt" font-weight="700" font-style="italic" >nad6</text> </g> <g fill="#ff9999" fill-opacity="1" stroke="#bfbfbf" stroke-opacity="1" stroke-width="1" stroke-linecap="square" stroke-linejoin="bevel" transform="matrix(1,0,0,1,316,410)" font-family="MS Shell Dlg 2" font-size="9.75" font-weight="400" font-style="normal" > <path vector-effect="none" fill-rule="evenodd" d="M0,1.7 L42.5,1.7 L42.5,0 L50,10 L42.5,20 L42.5,18.3 L0,18.3 L0,1.7"/> </g> <g fill="none" stroke="#000000" stroke-opacity="1" stroke-width="1" stroke-linecap="square" stroke-linejoin="bevel" transform="matrix(1,0,0,1,329.632,408)" font-family="MS Shell Dlg 2" font-size="9.75" font-weight="400" font-style="normal" > <text fill="#000000" fill-opacity="1" stroke="none" xml:space="preserve" x="4" y="17" font-family="Arial" font-size="10pt" font-weight="700" font-style="italic" >F</text> </g> <g fill="#99ffff" fill-opacity="1" stroke="#bfbfbf" stroke-opacity="1" stroke-width="1" stroke-linecap="square" stroke-linejoin="bevel" transform="matrix(1,0,0,1,367,410)" font-family="MS Shell Dlg 2" font-size="9.75" font-weight="400" font-style="normal" > <path vector-effect="none" fill-rule="evenodd" d="M0,5 C0,2.23858 2.23858,0 5,0 L45,0 C47.7614,0 50,2.23858 50,5 L50,15 C50,17.7614 47.7614,20 45,20 L5,20 C2.23858,20 0,17.7614 0,15 L0,5"/> </g> <g fill="none" stroke="#000000" stroke-opacity="1" stroke-width="1" stroke-linecap="square" stroke-linejoin="bevel" transform="matrix(1,0,0,1,375.5,408)" font-family="MS Shell Dlg 2" font-size="9.75" font-weight="400" font-style="normal" > <text fill="#000000" fill-opacity="1" stroke="none" xml:space="preserve" x="4" y="17" font-family="Arial" font-size="10pt" font-weight="700" font-style="italic" >rrnS</text> </g> <g fill="#ff9999" fill-opacity="1" stroke="#bfbfbf" stroke-opacity="1" stroke-width="1" stroke-linecap="square" stroke-linejoin="bevel" transform="matrix(1,0,0,1,418,410)" font-family="MS Shell Dlg 2" font-size="9.75" font-weight="400" font-style="normal" > <path vector-effect="none" fill-rule="evenodd" d="M0,1.7 L42.5,1.7 L42.5,0 L50,10 L42.5,20 L42.5,18.3 L0,18.3 L0,1.7"/> </g> <g fill="none" stroke="#000000" stroke-opacity="1" stroke-width="1" stroke-linecap="square" stroke-linejoin="bevel" transform="matrix(1,0,0,1,431.132,408)" font-family="MS Shell Dlg 2" font-size="9.75" font-weight="400" font-style="normal" > <text fill="#000000" fill-opacity="1" stroke="none" xml:space="preserve" x="4" y="17" font-family="Arial" font-size="10pt" font-weight="700" font-style="italic" >G</text> </g> <g fill="#ff9999" fill-opacity="1" stroke="#bfbfbf" stroke-opacity="1" stroke-width="1" stroke-linecap="square" stroke-linejoin="bevel" transform="matrix(1,0,0,1,469,410)" font-family="MS Shell Dlg 2" font-size="9.75" font-weight="400" font-style="normal" > <path vector-effect="none" fill-rule="evenodd" d="M0,1.7 L42.5,1.7 L42.5,0 L50,10 L42.5,20 L42.5,18.3 L0,18.3 L0,1.7"/> </g> <g fill="none" stroke="#000000" stroke-opacity="1" stroke-width="1" stroke-linecap="square" stroke-linejoin="bevel" transform="matrix(1,0,0,1,482.132,408)" font-family="MS Shell Dlg 2" font-size="9.75" font-weight="400" font-style="normal" > <text fill="#000000" fill-opacity="1" stroke="none" xml:space="preserve" x="4" y="17" font-family="Arial" font-size="10pt" font-weight="700" font-

style="italic" >N</text> </g> <g fill="#ff9999" fill-opacity="1" stroke="#bfbfbf" stroke-opacity="1" stroke-width="1" stroke-linecap="square" stroke-linejoin="bevel" transform="matrix(1,0,0,1,520,410)" font-family="MS Shell Dlg 2" font-size="9.75" font-weight="400" font-style="normal" > <path vector-effect="none" fill-rule="evenodd" d="M0,1.7 L42.5,1.7 L42.5,0 L50,10 L42.5,20 L42.5,18.3 L0,18.3 L0,1.7"/> </g> <g fill="none" stroke="#000000" stroke-opacity="1" stroke-width="1" stroke-linecap="square" stroke-linejoin="bevel" transform="matrix(1,0,0,1,533.633,408)" font-family="MS Shell Dlg 2" font-size="9.75" font-weight="400" font-style="normal" > <text fill="#000000" fill-opacity="1" stroke="none" xml:space="preserve" x="4" y="17" font-family="Arial" font-size="10pt" font-weight="700" font-style="italic" >E</text> </g> <g fill="#ff9999" fill-opacity="1" stroke="#bfbfbf" stroke-opacity="1" stroke-width="1" stroke-linecap="square" stroke-linejoin="bevel" transform="matrix(1,0,0,1,571,410)" font-family="MS Shell Dlg 2" font-size="9.75" font-weight="400" font-style="normal" > <path vector-effect="none" fill-rule="evenodd" d="M0,1.7 L42.5,1.7 L42.5,0 L50,10 L42.5,20 L42.5,18.3 L0,18.3 L0,1.7"/> </g> <g fill="none" stroke="#000000" stroke-opacity="1" stroke-width="1" stroke-linecap="square" stroke-linejoin="bevel" transform="matrix(1,0,0,1,584.133,408)" font-family="MS Shell Dlg 2" font-size="9.75" font-weight="400" font-style="normal" > <text fill="#000000" fill-opacity="1" stroke="none" xml:space="preserve" x="4" y="17" font-family="Arial" font-size="10pt" font-weight="700" font-style="italic" >C</text> </g> <g fill="#ff9999" fill-opacity="1" stroke="#bfbfbf" stroke-opacity="1" stroke-width="1" stroke-linecap="square" stroke-linejoin="bevel" transform="matrix(1,0,0,1,622,410)" font-family="MS Shell Dlg 2" font-size="9.75" font-weight="400" font-style="normal" > <path vector-effect="none" fill-rule="evenodd" d="M0,1.7 L42.5,1.7 L42.5,0 L50,10 L42.5,20 L42.5,18.3 L0,18.3 L0,1.7"/> </g> <g fill="none" stroke="#000000" stroke-opacity="1" stroke-width="1" stroke-linecap="square" stroke-linejoin="bevel" transform="matrix(1,0,0,1,637.133,408)" font-family="MS Shell Dlg 2" font-size="9.75" font-weight="400" font-style="normal" > <text fill="#000000" fill-opacity="1" stroke="none" xml:space="preserve" x="4" y="17" font-family="Arial" font-size="10pt" font-weight="700" font-style="italic" >I</text> </g> <g fill="#ff9999" fill-opacity="1" stroke="#bfbfbf" stroke-opacity="1" stroke-width="1" stroke-linecap="square" stroke-linejoin="bevel" transform="matrix(1,0,0,1,673,410)" font-family="MS Shell Dlg 2" font-size="9.75" font-weight="400" font-style="normal" > <path vector-effect="none" fill-rule="evenodd" d="M0,1.7 L42.5,1.7 L42.5,0 L50,10 L42.5,20 L42.5,18.3 L0,18.3 L0,1.7"/> </g> <g fill="none" stroke="#000000" stroke-opacity="1" stroke-width="1" stroke-linecap="square" stroke-linejoin="bevel" transform="matrix(1,0,0,1,685.633,408)" font-family="MS Shell Dlg 2" font-size="9.75" font-weight="400" font-style="normal" > <text fill="#000000" fill-opacity="1" stroke="none" xml:space="preserve" x="4" y="17" font-family="Arial" font-size="10pt" font-weight="700" font-style="italic" >Q</text> </g> <g fill="#ff9999" fill-opacity="1" stroke="#bfbfbf" stroke-opacity="1" stroke-width="1" stroke-linecap="square" stroke-linejoin="bevel" transform="matrix(1,0,0,1,724,410)" font-family="MS Shell Dlg 2" font-size="9.75" font-weight="400" font-style="normal" > <path vector-effect="none" fill-rule="evenodd" d="M0,1.7 L42.5,1.7 L42.5,0 L50,10 L42.5,20 L42.5,18.3 L0,18.3 L0,1.7"/> </g> <g fill="none" stroke="#000000" stroke-opacity="1" stroke-width="1" stroke-linecap="square" stroke-linejoin="bevel" transform="matrix(1,0,0,1,737.133,408)" font-family="MS Shell Dlg 2" font-size="9.75" font-weight="400" font-style="normal" > <text fill="#000000" fill-opacity="1" stroke="none" xml:space="preserve" x="4" y="17" font-family="Arial" font-size="10pt" font-weight="700" font-style="italic" >D</text> </g> <g fill="#99ffff" fill-opacity="1" stroke="#bfbfbf" stroke-opacity="1" stroke-width="1" stroke-linecap="square" stroke-linejoin="bevel" transform="matrix(1,0,0,1,775,410)" font-family="MS Shell Dlg 2" font-size="9.75" font-weight="400" font-style="normal" > <path vector-effect="none" fill-rule="evenodd" d="M0,5 C0,2.23858 2.23858,0 5,0 L45,0 C47.7614,0 50,2.23858 50,5 L50,15 C50,17.7614 47.7614,20 45,20 L5,20 C2.23858,20 0,17.7614 0,15 L0,5"/> </g> <g fill="none" stroke="#000000" stroke-opacity="1" stroke-width="1" stroke-linecap="square" stroke-linejoin="bevel" transform="matrix(1,0,0,1,784.5,408)" font-family="MS Shell Dlg 2" font-size="9.75" font-weight="400" font-style="normal" > <text fill="#000000" fill-opacity="1" stroke="none" xml:space="preserve" x="4" y="17" font-family="Arial" font-size="10pt" font-weight="700" font-style="italic" >rrnL</text> </g> <g fill="#ff9999" fill-opacity="1" stroke="#bfbfbf" stroke-opacity="1" stroke-width="1" stroke-linecap="square" stroke-linejoin="bevel" transform="matrix(1,0,0,1,826,410)" font-family="MS Shell Dlg 2" font-size="9.75" font-weight="400" font-style="normal" > <path vector-effect="none" fill-rule="evenodd" d="M0,1.7 L42.5,1.7 L42.5,0 L50,10 L42.5,20 L42.5,18.3 L0,18.3 L0,1.7"/> </g> <g fill="none" stroke="#000000" stroke-opacity="1" stroke-width="1" stroke-linecap="square" stroke-linejoin="bevel" transform="matrix(1,0,0,1,839.633,408)" font-family="MS Shell Dlg 2" font-size="9.75" font-weight="400" font-style="normal" > <text fill="#000000" fill-opacity="1" stroke="none" xml:space="preserve" x="4" y="17" font-family="Arial" font-size="10pt" font-weight="700" font-style="italic" >Y</text> </g> <g fill="#ffff33" fill-opacity="1" stroke="#bfbfbf" stroke-opacity="1" stroke-width="1" stroke-linecap="square" stroke-linejoin="bevel" transform="matrix(1,0,0,1,877,410)" font-family="MS Shell Dlg 2" font-size="9.75" font-weight="400" font-style="normal" > <rect x="0" y="0" width="50" height="20"/> </g> <g fill="none" stroke="#000000" stroke-

opacity="1" stroke-width="1" stroke-linecap="square" stroke-linejoin="bevel" transform="matrix(1,0,0,1,886.5,408)" font-family="MS Shell Dlg 2" font-size="9.75" font-weight="400" font-style="normal" > <text fill="#000000" fill-opacity="1" stroke="none" xml:space="preserve" x="4" y="17" font-family="Arial" font-size="10pt" font-weight="700" font-style="italic" >cytb</text> </g> <g fill="#ffff33" fill-opacity="1" stroke="#bfbfbf" stroke-opacity="1" stroke-width="1" stroke-linecap="square" stroke-linejoin="bevel" transform="matrix(1,0,0,1,928,410)" font-family="MS Shell Dlg 2" font-size="9.75" font-weight="400" font-style="normal" > <rect x="0" y="0" width="50" height="20"/> </g> <g fill="none" stroke="#000000" stroke-opacity="1" stroke-width="1" stroke-linecap="square" stroke-linejoin="bevel" transform="matrix(1,0,0,1,936,408)" font-family="MS Shell Dlg 2" font-size="9.75" font-weight="400" font-style="normal" > <text fill="#000000" fill-opacity="1" stroke="none" xml:space="preserve" x="4" y="17" font-family="Arial" font-size="10pt" font-weight="700" font-style="italic" >cox2</text> </g> <g fill="#ff9999" fill-opacity="1" stroke="#bfbfbf" stroke-opacity="1" stroke-width="1" stroke-linecap="square" stroke-linejoin="bevel" transform="matrix(1,0,0,1,979,410)" font-family="MS Shell Dlg 2" font-size="9.75" font-weight="400" font-style="normal" > <path vector-effect="none" fill-rule="evenodd" d="M0,1.7 L42.5,1.7 L42.5,0 L50,10 L42.5,20 L42.5,18.3 L0,18.3 L0,1.7"/> </g> <g fill="none" stroke="#000000" stroke-opacity="1" stroke-width="1" stroke-linecap="square" stroke-linejoin="bevel" transform="matrix(1,0,0,1,992.633,408)" font-family="MS Shell Dlg 2" font-size="9.75" font-weight="400" font-style="normal" > <text fill="#000000" fill-opacity="1" stroke="none" xml:space="preserve" x="4" y="17" font-family="Arial" font-size="10pt" font-weight="700" font-style="italic" >K</text> </g> <g fill="#ff9999" fill-opacity="1" stroke="#bfbfbf" stroke-opacity="1" stroke-width="1" stroke-linecap="square" stroke-linejoin="bevel" transform="matrix(1,0,0,1,1030,410)" font-family="MS Shell Dlg 2" font-size="9.75" font-weight="400" font-style="normal" > <path vector-effect="none" fill-rule="evenodd" d="M0,1.7 L42.5,1.7 L42.5,0 L50,10 L42.5,20 L42.5,18.3 L0,18.3 L0,1.7"/> </g> <g fill="none" stroke="#000000" stroke-opacity="1" stroke-width="1" stroke-linecap="square" stroke-linejoin="bevel" transform="matrix(1,0,0,1,1042.13,408)" font-family="MS Shell Dlg 2" font-size="9.75" font-weight="400" font-style="normal" > <text fill="#000000" fill-opacity="1" stroke="none" xml:space="preserve" x="4" y="17" font-family="Arial" font-size="10pt" font-weight="700" font-style="italic" >M</text> </g> <g fill="#ff9999" fill-opacity="1" stroke="#bfbfbf" stroke-opacity="1" stroke-width="1" stroke-linecap="square" stroke-linejoin="bevel" transform="matrix(1,0,0,1,1081,410)" font-family="MS Shell Dlg 2" font-size="9.75" font-weight="400" font-style="normal" > <path vector-effect="none" fill-rule="evenodd" d="M0,1.7 L42.5,1.7 L42.5,0 L50,10 L42.5,20 L42.5,18.3 L0,18.3 L0,1.7"/> </g> <g fill="none" stroke="#000000" stroke-opacity="1" stroke-width="1" stroke-linecap="square" stroke-linejoin="bevel" transform="matrix(1,0,0,1,1091.63,408)" font-family="MS Shell Dlg 2" font-size="9.75" font-weight="400" font-style="normal" > <text fill="#000000" fill-opacity="1" stroke="none" xml:space="preserve" x="4" y="17" font-family="Arial" font-size="10pt" font-weight="700" font-style="italic" >L1</text> </g> <g fill="#ff9999" fill-opacity="1" stroke="#bfbfbf" stroke-opacity="1" stroke-width="1" stroke-linecap="square" stroke-linejoin="bevel" transform="matrix(1,0,0,1,1132,410)" font-family="MS Shell Dlg 2" font-size="9.75" font-weight="400" font-style="normal" > <path vector-effect="none" fill-rule="evenodd" d="M0,1.7 L42.5,1.7 L42.5,0 L50,10 L42.5,20 L42.5,18.3 L0,18.3 L0,1.7"/> </g> <g fill="none" stroke="#000000" stroke-opacity="1" stroke-width="1" stroke-linecap="square" stroke-linejoin="bevel" transform="matrix(1,0,0,1,1142.63,408)" font-family="MS Shell Dlg 2" font-size="9.75" font-weight="400" font-style="normal" > <text fill="#000000" fill-opacity="1" stroke="none" xml:space="preserve" x="4" y="17" font-family="Arial" font-size="10pt" font-weight="700" font-style="italic" >L2</text> </g> <g fill="#ffff33" fill-opacity="1" stroke="#bfbfbf" stroke-opacity="1" stroke-width="1" stroke-linecap="square" stroke-linejoin="bevel" transform="matrix(1,0,0,1,1183,410)" font-family="MS Shell Dlg 2" font-size="9.75" font-weight="400" font-style="normal" > <rect x="0" y="0" width="50" height="20"/> </g> <g fill="none" stroke="#000000" stroke-opacity="1" stroke-width="1" stroke-linecap="square" stroke-linejoin="bevel" transform="matrix(1,0,0,1,1190,408)" font-family="MS Shell Dlg 2" font-size="9.75" font-weight="400" font-style="normal" > <text fill="#000000" fill-opacity="1" stroke="none" xml:space="preserve" x="4" y="17" font-family="Arial" font-size="10pt" font-weight="700" font-style="italic" >nad1</text> </g> <g fill="#ff9999" fill-opacity="1" stroke="#bfbfbf" stroke-opacity="1" stroke-width="1" stroke-linecap="square" stroke-linejoin="bevel" transform="matrix(1,0,0,1,1234,410)" font-family="MS Shell Dlg 2" font-size="9.75" font-weight="400" font-style="normal" > <path vector-effect="none" fill-rule="evenodd" d="M0,1.7 L42.5,1.7 L42.5,0 L50,10 L42.5,20 L42.5,18.3 L0,18.3 L0,1.7"/> </g> <g fill="none" stroke="#000000" stroke-opacity="1" stroke-width="1" stroke-linecap="square" stroke-linejoin="bevel" transform="matrix(1,0,0,1,1247.13,408)" font-family="MS Shell Dlg 2" font-size="9.75" font-weight="400" font-style="normal" > <text fill="#000000" fill-opacity="1" stroke="none" xml:space="preserve" x="4" y="17" font-family="Arial" font-size="10pt" font-weight="700" font-style="italic" >V</text> </g> <g fill="#ffff33" fill-opacity="1" stroke="#bfbfbf" stroke-opacity="1" stroke-width="1" stroke-linecap="square" stroke-linejoin="bevel"

transform="matrix(1,0,0,1,1285,410)" font-family="MS Shell Dlg 2" font-size="9.75" font-weight="400" font-style="normal" > <rect x="0" y="0" width="50" height="20"/> </g> <g fill="none" stroke="#000000" stroke-opacity="1" stroke-width="1" stroke-linecap="square" stroke-linejoin="bevel" transform="matrix(1,0,0,1,1292,408)" font-family="MS Shell Dlg 2" font-size="9.75" font-weight="400" font-style="normal" > <text fill="#000000" fill-opacity="1" stroke="none" xml:space="preserve" x="4" y="17" font-family="Arial" font-size="10pt" font-weight="700" font-style="italic" >nad4</text> </g> <g fill="#ffff33" fill-opacity="1" stroke="#bfbfbf" stroke-opacity="1" stroke-width="1" stroke-linecap="square" stroke-linejoin="bevel" transform="matrix(1,0,0,1,1336,410)" font-family="MS Shell Dlg 2" font-size="9.75" font-weight="400" font-style="normal" > <rect x="0" y="0" width="50" height="20"/> </g> <g fill="none" stroke="#000000" stroke-opacity="1" stroke-width="1" stroke-linecap="square" stroke-linejoin="bevel" transform="matrix(1,0,0,1,1344,408)" font-family="MS Shell Dlg 2" font-size="9.75" font-weight="400" font-style="normal" > <text fill="#000000" fill-opacity="1" stroke="none" xml:space="preserve" x="4" y="17" font-family="Arial" font-size="10pt" font-weight="700" font-style="italic" >cox3</text> </g> <g fill="#ff9999" fill-opacity="1" stroke="#bfbfbf" stroke-opacity="1" stroke-width="1" stroke-linecap="square" stroke-linejoin="bevel" transform="matrix(1,0,0,1,1387,410)" font-family="MS Shell Dlg 2" font-size="9.75" font-weight="400" font-style="normal" > <path vector-effect="none" fill-rule="evenodd" d="M0,1.7 L42.5,1.7 L42.5,0 L50,10 L42.5,20 L42.5,18.3 L0,18.3 L0,1.7"/> </g> <g fill="none" stroke="#000000" stroke-opacity="1" stroke-width="1" stroke-linecap="square" stroke-linejoin="bevel" transform="matrix(1,0,0,1,1396.63,408)" font-family="MS Shell Dlg 2" font-size="9.75" font-weight="400" font-style="normal" > <text fill="#000000" fill-opacity="1" stroke="none" xml:space="preserve" x="4" y="17" font-family="Arial" font-size="10pt" font-weight="700" font-style="italic" >S2</text> </g> <g fill="#ff9999" fill-opacity="1" stroke="#bfbfbf" stroke-opacity="1" stroke-width="1" stroke-linecap="square" stroke-linejoin="bevel" transform="matrix(1,0,0,1,1438,410)" font-family="MS Shell Dlg 2" font-size="9.75" font-weight="400" font-style="normal" > <path vector-effect="none" fill-rule="evenodd" d="M0,1.7 L42.5,1.7 L42.5,0 L50,10 L42.5,20 L42.5,18.3 L0,18.3 L0,1.7"/> </g> <g fill="none" stroke="#000000" stroke-opacity="1" stroke-width="1" stroke-linecap="square" stroke-linejoin="bevel" transform="matrix(1,0,0,1,1450.13,408)" font-family="MS Shell Dlg 2" font-size="9.75" font-weight="400" font-style="normal" > <text fill="#000000" fill-opacity="1" stroke="none" xml:space="preserve" x="4" y="17" font-family="Arial" font-size="10pt" font-weight="700" font-style="italic" >M</text> </g> <g fill="#ffff33" fill-opacity="1" stroke="#bfbfbf" stroke-opacity="1" stroke-width="1" stroke-linecap="square" stroke-linejoin="bevel" transform="matrix(1,0,0,1,1489,410)" font-family="MS Shell Dlg 2" font-size="9.75" font-weight="400" font-style="normal" > <rect x="0" y="0" width="50" height="20"/> </g> <g fill="none" stroke="#000000" stroke-opacity="1" stroke-width="1" stroke-linecap="square" stroke-linejoin="bevel" transform="matrix(1,0,0,1,1496,408)" font-family="MS Shell Dlg 2" font-size="9.75" font-weight="400" font-style="normal" > <text fill="#000000" fill-opacity="1" stroke="none" xml:space="preserve" x="4" y="17" font-family="Arial" font-size="10pt" font-weight="700" font-style="italic" >nad2</text> </g> <g fill="#ff9999" fill-opacity="1" stroke="#bfbfbf" stroke-opacity="1" stroke-width="1" stroke-linecap="square" stroke-linejoin="bevel" transform="matrix(1,0,0,1,1540,410)" font-family="MS Shell Dlg 2" font-size="9.75" font-weight="400" font-style="normal" > <path vector-effect="none" fill-rule="evenodd" d="M0,1.7 L42.5,1.7 L42.5,0 L50,10 L42.5,20 L42.5,18.3 L0,18.3 L0,1.7"/> </g> <g fill="none" stroke="#000000" stroke-opacity="1" stroke-width="1" stroke-linecap="square" stroke-linejoin="bevel" transform="matrix(1,0,0,1,1553.13,408)" font-family="MS Shell Dlg 2" font-size="9.75" font-weight="400" font-style="normal" > <text fill="#000000" fill-opacity="1" stroke="none" xml:space="preserve" x="4" y="17" font-family="Arial" font-size="10pt" font-weight="700" font-style="italic" >R</text> </g> <g fill="#ff9999" fill-opacity="1" stroke="#bfbfbf" stroke-opacity="1" stroke-width="1" stroke-linecap="square" stroke-linejoin="bevel" transform="matrix(1,0,0,1,1591,410)" font-family="MS Shell Dlg 2" font-size="9.75" font-weight="400" font-style="normal" > <path vector-effect="none" fill-rule="evenodd" d="M0,1.7 L42.5,1.7 L42.5,0 L50,10 L42.5,20 L42.5,18.3 L0,18.3 L0,1.7"/> </g> <g fill="none" stroke="#000000" stroke-opacity="1" stroke-width="1" stroke-linecap="square" stroke-linejoin="bevel" transform="matrix(1,0,0,1,1602.13,408)" font-family="MS Shell Dlg 2" font-size="9.75" font-weight="400" font-style="normal" > <text fill="#000000" fill-opacity="1" stroke="none" xml:space="preserve" x="4" y="17" font-family="Arial" font-size="10pt" font-weight="700" font-style="italic" >W</text> </g> <g fill="#ff9999" fill-opacity="1" stroke="#bfbfbf" stroke-opacity="1" stroke-width="1" stroke-linecap="square" stroke-linejoin="bevel" transform="matrix(1,0,0,1,1642,410)" font-family="MS Shell Dlg 2" font-size="9.75" font-weight="400" font-style="normal" > <path vector-effect="none" fill-rule="evenodd" d="M0,1.7 L42.5,1.7 L42.5,0 L50,10 L42.5,20 L42.5,18.3 L0,18.3 L0,1.7"/> </g> <g fill="none" stroke="#000000" stroke-opacity="1" stroke-width="1" stroke-linecap="square" stroke-linejoin="bevel" transform="matrix(1,0,0,1,1655.13,408)" font-family="MS Shell Dlg 2" font-size="9.75" font-weight="400" font-style="normal" > <text fill="#000000" fill-opacity="1" stroke="none" xml:space="preserve" x="4" y="17" font-family="Arial" font-size="10pt" font-weight="700" font-

style="italic" >A</text> </g> <g fill="#ff9999" fill-opacity="1" stroke="#bfbfbf" stroke-opacity="1" stroke-width="1" stroke-linecap="square" stroke-linejoin="bevel" transform="matrix(1,0,0,1,1693,410)" font-family="MS Shell Dlg 2" font-size="9.75" font-weight="400" font-style="normal" > <path vector-effect="none" fill-rule="evenodd" d="M0,1.7 L42.5,1.7 L42.5,0 L50,10 L42.5,20 L42.5,18.3 L0,18.3 L0,1.7"/> </g> <g fill="none" stroke="#000000" stroke-opacity="1" stroke-width="1" stroke-linecap="square" stroke-linejoin="bevel" transform="matrix(1,0,0,1,1702.63,408)" font-family="MS Shell Dlg 2" font-size="9.75" font-weight="400" font-style="normal" > <text fill="#000000" fill-opacity="1" stroke="none" xml:space="preserve" x="4" y="17" font-family="Arial" font-size="10pt" font-weight="700" font-style="italic" >S1</text> </g> <g fill="#ff9999" fill-opacity="1" stroke="#bfbfbf" stroke-opacity="1" stroke-width="1" stroke-linecap="square" stroke-linejoin="bevel" transform="matrix(1,0,0,1,1744,410)" font-family="MS Shell Dlg 2" font-size="9.75" font-weight="400" font-style="normal" > <path vector-effect="none" fill-rule="evenodd" d="M0,1.7 L42.5,1.7 L42.5,0 L50,10 L42.5,20 L42.5,18.3 L0,18.3 L0,1.7"/> </g> <g fill="none" stroke="#000000" stroke-opacity="1" stroke-width="1" stroke-linecap="square" stroke-linejoin="bevel" transform="matrix(1,0,0,1,1757.13,408)" font-family="MS Shell Dlg 2" font-size="9.75" font-weight="400" font-style="normal" > <text fill="#000000" fill-opacity="1" stroke="none" xml:space="preserve" x="4" y="17" font-family="Arial" font-size="10pt" font-weight="700" font-style="italic" >H</text> </g> <g fill="#ff9999" fill-opacity="1" stroke="#bfbfbf" stroke-opacity="1" stroke-width="1" stroke-linecap="square" stroke-linejoin="bevel" transform="matrix(1,0,0,1,1795,410)" font-family="MS Shell Dlg 2" font-size="9.75" font-weight="400" font-style="normal" > <path vector-effect="none" fill-rule="evenodd" d="M0,1.7 L42.5,1.7 L42.5,0 L50,10 L42.5,20 L42.5,18.3 L0,18.3 L0,1.7"/> </g> <g fill="none" stroke="#000000" stroke-opacity="1" stroke-width="1" stroke-linecap="square" stroke-linejoin="bevel" transform="matrix(1,0,0,1,1808.63,408)" font-family="MS Shell Dlg 2" font-size="9.75" font-weight="400" font-style="normal" > <text fill="#000000" fill-opacity="1" stroke="none" xml:space="preserve" x="4" y="17" font-family="Arial" font-size="10pt" font-weight="700" font-style="italic" >P</text> </g> <g fill="#ffff33" fill-opacity="1" stroke="#bfbfbf" stroke-opacity="1" stroke-width="1" stroke-linecap="square" stroke-linejoin="bevel" transform="matrix(1,0,0,1,1846,410)" font-family="MS Shell Dlg 2" font-size="9.75" font-weight="400" font-style="normal" > <rect x="0" y="0" width="50" height="20"/> </g> <g fill="none" stroke="#000000" stroke-opacity="1" stroke-width="1" stroke-linecap="square" stroke-linejoin="bevel" transform="matrix(1,0,0,1,1853,408)" font-family="MS Shell Dlg 2" font-size="9.75" font-weight="400" font-style="normal" > <text fill="#000000" fill-opacity="1" stroke="none" xml:space="preserve" x="4" y="17" font-family="Arial" font-size="10pt" font-weight="700" font-style="italic" >nad3</text> </g> <g fill="#ffff33" fill-opacity="1" stroke="#bfbfbf" stroke-opacity="1" stroke-width="1" stroke-linecap="square" stroke-linejoin="bevel" transform="matrix(1,0,0,1,1897,410)" font-family="MS Shell Dlg 2" font-size="9.75" font-weight="400" font-style="normal" > <rect x="0" y="0" width="50" height="20"/> </g> <g fill="none" stroke="#000000" stroke-opacity="1" stroke-width="1" stroke-linecap="square" stroke-linejoin="bevel" transform="matrix(1,0,0,1,1905.5,408)" font-family="MS Shell Dlg 2" font-size="9.75" font-weight="400" font-style="normal" > <text fill="#000000" fill-opacity="1" stroke="none" xml:space="preserve" x="4" y="17" font-family="Arial" font-size="10pt" font-weight="700" font-style="italic" >atp8</text> </g> <g fill="none" stroke="#000000" stroke-opacity="1" stroke-width="1" stroke-linecap="square" stroke-linejoin="bevel" transform="matrix(1,0,0,1,1948,410)" font-family="MS Shell Dlg 2" font-size="9.75" font-weight="400" font-style="normal" > <text fill="#000000" fill-opacity="1" stroke="none" xml:space="preserve" x="4" y="17" font-family="Arial" font-size="10pt" font-weight="700" font-style="italic" >(+)</text> </g> <g fill="none" stroke="#000000" stroke-opacity="1" stroke-width="1" stroke-linecap="square" stroke-linejoin="bevel" transform="matrix(1,0,0,1,10,450)" font-family="MS Shell Dlg 2" font-size="9.75" font-weight="400" font-style="normal" > <text fill="#000000" fill-opacity="1" stroke="none" xml:space="preserve" x="4" y="17" font-family="Arial" font-size="10pt" font-weight="700" font-style="italic" >Mytilus_trossulus_GU936625</text> </g> <g fill="#ffff33" fill-opacity="1" stroke="#bfbfbf" stroke-opacity="1" stroke-width="1" stroke-linecap="square" stroke-linejoin="bevel" transform="matrix(1,0,0,1,10,480)" font-family="MS Shell Dlg 2" font-size="9.75" font-weight="400" font-style="normal" > <rect x="0" y="0" width="50" height="20"/> </g> <g fill="none" stroke="#000000" stroke-opacity="1" stroke-width="1" stroke-linecap="square" stroke-linejoin="bevel" transform="matrix(1,0,0,1,18,478)" font-family="MS Shell Dlg 2" font-size="9.75" font-weight="400" font-style="normal" > <text fill="#000000" fill-opacity="1" stroke="none" xml:space="preserve" x="4" y="17" font-family="Arial" font-size="10pt" font-weight="700" font-style="italic" >cox1</text> </g> <g fill="#ffff33" fill-opacity="1" stroke="#bfbfbf" stroke-opacity="1" stroke-width="1" stroke-linecap="square" stroke-linejoin="bevel" transform="matrix(1,0,0,1,61,480)" font-family="MS Shell Dlg 2" font-size="9.75" font-weight="400" font-style="normal" > <rect x="0" y="0" width="50" height="20"/> </g> <g fill="none" stroke="#000000" stroke-opacity="1" stroke-width="1" stroke-linecap="square" stroke-linejoin="bevel" transform="matrix(1,0,0,1,69.5,478)" font-family="MS Shell Dlg 2" font-size="9.75" font-weight="400" font-style="normal" > <text fill="#000000" fill-opacity="1" stroke="none" xml:space="preserve"

x="4" y="17" font-family="Arial" font-size="10pt" font-weight="700" font-style="italic" >atp6</text> </g>
<g fill="#ff9999" fill-opacity="1" stroke="#bfbfbf" stroke-opacity="1" stroke-width="1" stroke-linecap="square" stroke-linejoin="bevel" transform="matrix(1,0,0,1,112,480)" font-family="MS Shell Dlg 2" font-size="9.75" font-weight="400" font-style="normal" > <path vector-effect="none" fill-rule="evenodd" d="M0,1.7 L42.5,1.7 L42.5,0 L50,10 L42.5,20 L42.5,18.3 L0,18.3 L0,1.7"/> </g> <g fill="none" stroke="#000000" stroke-opacity="1" stroke-width="1" stroke-linecap="square" stroke-linejoin="bevel" transform="matrix(1,0,0,1,125.132,478)" font-family="MS Shell Dlg 2" font-size="9.75" font-weight="400" font-style="normal" > <text fill="#000000" fill-opacity="1" stroke="none" xml:space="preserve" x="4" y="17" font-family="Arial" font-size="10pt" font-weight="700" font-style="italic" >T</text> </g> <g fill="#ffff33" fill-opacity="1" stroke="#bfbfbf" stroke-opacity="1" stroke-width="1" stroke-linecap="square" stroke-linejoin="bevel" transform="matrix(1,0,0,1,163,480)" font-family="MS Shell Dlg 2" font-size="9.75" font-weight="400" font-style="normal" > <rect x="0" y="0" width="50" height="20"/> </g> <g fill="none" stroke="#000000" stroke-opacity="1" stroke-width="1" stroke-linecap="square" stroke-linejoin="bevel" transform="matrix(1,0,0,1,167,478)" font-family="MS Shell Dlg 2" font-size="9.75" font-weight="400" font-style="normal" > <text fill="#000000" fill-opacity="1" stroke="none" xml:space="preserve" x="4" y="17" font-family="Arial" font-size="10pt" font-weight="700" font-style="italic" >nad4L</text> </g> <g fill="#ffff33" fill-opacity="1" stroke="#bfbfbf" stroke-opacity="1" stroke-width="1" stroke-linecap="square" stroke-linejoin="bevel" transform="matrix(1,0,0,1,214,480)" font-family="MS Shell Dlg 2" font-size="9.75" font-weight="400" font-style="normal" > <rect x="0" y="0" width="50" height="20"/> </g> <g fill="none" stroke="#000000" stroke-opacity="1" stroke-width="1" stroke-linecap="square" stroke-linejoin="bevel" transform="matrix(1,0,0,1,221,478)" font-family="MS Shell Dlg 2" font-size="9.75" font-weight="400" font-style="normal" > <text fill="#000000" fill-opacity="1" stroke="none" xml:space="preserve" x="4" y="17" font-family="Arial" font-size="10pt" font-weight="700" font-style="italic" >nad5</text> </g> <g fill="#ffff33" fill-opacity="1" stroke="#bfbfbf" stroke-opacity="1" stroke-width="1" stroke-linecap="square" stroke-linejoin="bevel" transform="matrix(1,0,0,1,265,480)" font-family="MS Shell Dlg 2" font-size="9.75" font-weight="400" font-style="normal" > <rect x="0" y="0" width="50" height="20"/> </g> <g fill="none" stroke="#000000" stroke-opacity="1" stroke-width="1" stroke-linecap="square" stroke-linejoin="bevel" transform="matrix(1,0,0,1,272,478)" font-family="MS Shell Dlg 2" font-size="9.75" font-weight="400" font-style="normal" > <text fill="#000000" fill-opacity="1" stroke="none" xml:space="preserve" x="4" y="17" font-family="Arial" font-size="10pt" font-weight="700" font-style="italic" >nad6</text> </g> <g fill="#ff9999" fill-opacity="1" stroke="#bfbfbf" stroke-opacity="1" stroke-width="1" stroke-linecap="square" stroke-linejoin="bevel" transform="matrix(1,0,0,1,316,480)" font-family="MS Shell Dlg 2" font-size="9.75" font-weight="400" font-style="normal" > <path vector-effect="none" fill-rule="evenodd" d="M0,1.7 L42.5,1.7 L42.5,0 L50,10 L42.5,20 L42.5,18.3 L0,18.3 L0,1.7"/> </g> <g fill="none" stroke="#000000" stroke-opacity="1" stroke-width="1" stroke-linecap="square" stroke-linejoin="bevel" transform="matrix(1,0,0,1,329.632,478)" font-family="MS Shell Dlg 2" font-size="9.75" font-weight="400" font-style="normal" > <text fill="#000000" fill-opacity="1" stroke="none" xml:space="preserve" x="4" y="17" font-family="Arial" font-size="10pt" font-weight="700" font-style="italic" >F</text> </g> <g fill="#99ffff" fill-opacity="1" stroke="#bfbfbf" stroke-opacity="1" stroke-width="1" stroke-linecap="square" stroke-linejoin="bevel" transform="matrix(1,0,0,1,367,480)" font-family="MS Shell Dlg 2" font-size="9.75" font-weight="400" font-style="normal" > <path vector-effect="none" fill-rule="evenodd" d="M0,5 C0,2.23858 2.23858,0 5,0 L45,0 C47.7614,0 50,2.23858 50,5 L50,15 C50,17.7614 47.7614,20 45,20 L5,20 C2.23858,20 0,17.7614 0,15 L0,5"/> </g> <g fill="none" stroke="#000000" stroke-opacity="1" stroke-width="1" stroke-linecap="square" stroke-linejoin="bevel" transform="matrix(1,0,0,1,375.5,478)" font-family="MS Shell Dlg 2" font-size="9.75" font-weight="400" font-style="normal" > <text fill="#000000" fill-opacity="1" stroke="none" xml:space="preserve" x="4" y="17" font-family="Arial" font-size="10pt" font-weight="700" font-style="italic" >rrnS</text> </g> <g fill="#ff9999" fill-opacity="1" stroke="#bfbfbf" stroke-opacity="1" stroke-width="1" stroke-linecap="square" stroke-linejoin="bevel" transform="matrix(1,0,0,1,418,480)" font-family="MS Shell Dlg 2" font-size="9.75" font-weight="400" font-style="normal" > <path vector-effect="none" fill-rule="evenodd" d="M0,1.7 L42.5,1.7 L42.5,0 L50,10 L42.5,20 L42.5,18.3 L0,18.3 L0,1.7"/> </g> <g fill="none" stroke="#000000" stroke-opacity="1" stroke-width="1" stroke-linecap="square" stroke-linejoin="bevel" transform="matrix(1,0,0,1,431.132,478)" font-family="MS Shell Dlg 2" font-size="9.75" font-weight="400" font-style="normal" > <text fill="#000000" fill-opacity="1" stroke="none" xml:space="preserve" x="4" y="17" font-family="Arial" font-size="10pt" font-weight="700" font-style="italic" >G</text> </g> <g fill="#ff9999" fill-opacity="1" stroke="#bfbfbf" stroke-opacity="1" stroke-width="1" stroke-linecap="square" stroke-linejoin="bevel" transform="matrix(1,0,0,1,469,480)" font-family="MS Shell Dlg 2" font-size="9.75" font-weight="400" font-style="normal" > <path vector-effect="none" fill-rule="evenodd" d="M0,1.7 L42.5,1.7 L42.5,0 L50,10 L42.5,20 L42.5,18.3 L0,18.3 L0,1.7"/> </g> <g fill="none" stroke="#000000" stroke-opacity="1" stroke-width="1"

stroke-linecap="square" stroke-linejoin="bevel" transform="matrix(1,0,0,1,482.132,478)" font-family="MS Shell Dlg 2" font-size="9.75" font-weight="400" font-style="normal" > <text fill="#000000" fill-opacity="1" stroke="none" xml:space="preserve" x="4" y="17" font-family="Arial" font-size="10pt" font-weight="700" font-style="italic" >N</text> </g> <g fill="#ff9999" fill-opacity="1" stroke="#bfbfbf" stroke-opacity="1" stroke-width="1" stroke-linecap="square" stroke-linejoin="bevel" transform="matrix(1,0,0,1,520,480)" font-family="MS Shell Dlg 2" font-size="9.75" font-weight="400" font-style="normal" > <path vector-effect="none" fill-rule="evenodd" d="M0,1.7 L42.5,1.7 L42.5,0 L50,10 L42.5,20 L42.5,18.3 L0,18.3 L0,1.7"/> </g> <g fill="none" stroke="#000000" stroke-opacity="1" stroke-width="1" stroke-linecap="square" stroke-linejoin="bevel" transform="matrix(1,0,0,1,533.633,478)" font-family="MS Shell Dlg 2" font-size="9.75" font-weight="400" font-style="normal" > <text fill="#000000" fill-opacity="1" stroke="none" xml:space="preserve" x="4" y="17" font-family="Arial" font-size="10pt" font-weight="700" font-style="italic" >E</text> </g> <g fill="#ff9999" fill-opacity="1" stroke="#bfbfbf" stroke-opacity="1" stroke-width="1" stroke-linecap="square" stroke-linejoin="bevel" transform="matrix(1,0,0,1,571,480)" font-family="MS Shell Dlg 2" font-size="9.75" font-weight="400" font-style="normal" > <path vector-effect="none" fill-rule="evenodd" d="M0,1.7 L42.5,1.7 L42.5,0 L50,10 L42.5,20 L42.5,18.3 L0,18.3 L0,1.7"/> </g> <g fill="none" stroke="#000000" stroke-opacity="1" stroke-width="1" stroke-linecap="square" stroke-linejoin="bevel" transform="matrix(1,0,0,1,584.133,478)" font-family="MS Shell Dlg 2" font-size="9.75" font-weight="400" font-style="normal" > <text fill="#000000" fill-opacity="1" stroke="none" xml:space="preserve" x="4" y="17" font-family="Arial" font-size="10pt" font-weight="700" font-style="italic" >C</text> </g> <g fill="#ff9999" fill-opacity="1" stroke="#bfbfbf" stroke-opacity="1" stroke-width="1" stroke-linecap="square" stroke-linejoin="bevel" transform="matrix(1,0,0,1,622,480)" font-family="MS Shell Dlg 2" font-size="9.75" font-weight="400" font-style="normal" > <path vector-effect="none" fill-rule="evenodd" d="M0,1.7 L42.5,1.7 L42.5,0 L50,10 L42.5,20 L42.5,18.3 L0,18.3 L0,1.7"/> </g> <g fill="none" stroke="#000000" stroke-opacity="1" stroke-width="1" stroke-linecap="square" stroke-linejoin="bevel" transform="matrix(1,0,0,1,637.133,478)" font-family="MS Shell Dlg 2" font-size="9.75" font-weight="400" font-style="normal" > <text fill="#000000" fill-opacity="1" stroke="none" xml:space="preserve" x="4" y="17" font-family="Arial" font-size="10pt" font-weight="700" font-style="italic" >I</text> </g> <g fill="#ff9999" fill-opacity="1" stroke="#bfbfbf" stroke-opacity="1" stroke-width="1" stroke-linecap="square" stroke-linejoin="bevel" transform="matrix(1,0,0,1,673,480)" font-family="MS Shell Dlg 2" font-size="9.75" font-weight="400" font-style="normal" > <path vector-effect="none" fill-rule="evenodd" d="M0,1.7 L42.5,1.7 L42.5,0 L50,10 L42.5,20 L42.5,18.3 L0,18.3 L0,1.7"/> </g> <g fill="none" stroke="#000000" stroke-opacity="1" stroke-width="1" stroke-linecap="square" stroke-linejoin="bevel" transform="matrix(1,0,0,1,686.133,478)" font-family="MS Shell Dlg 2" font-size="9.75" font-weight="400" font-style="normal" > <text fill="#000000" fill-opacity="1" stroke="none" xml:space="preserve" x="4" y="17" font-family="Arial" font-size="10pt" font-weight="700" font-style="italic" >D</text> </g> <g fill="#99ffff" fill-opacity="1" stroke="#bfbfbf" stroke-opacity="1" stroke-width="1" stroke-linecap="square" stroke-linejoin="bevel" transform="matrix(1,0,0,1,724,480)" font-family="MS Shell Dlg 2" font-size="9.75" font-weight="400" font-style="normal" > <path vector-effect="none" fill-rule="evenodd" d="M0,5 C0,2.23858 2.23858,0 5,0 L45,0 C47.7614,0 50,2.23858 50,5 L50,15 C50,17.7614 47.7614,20 45,20 L5,20 C2.23858,20 0,17.7614 0,15 L0,5"/> </g> <g fill="none" stroke="#000000" stroke-opacity="1" stroke-width="1" stroke-linecap="square" stroke-linejoin="bevel" transform="matrix(1,0,0,1,733.5,478)" font-family="MS Shell Dlg 2" font-size="9.75" font-weight="400" font-style="normal" > <text fill="#000000" fill-opacity="1" stroke="none" xml:space="preserve" x="4" y="17" font-family="Arial" font-size="10pt" font-weight="700" font-style="italic" >rrnL</text> </g> <g fill="#ff9999" fill-opacity="1" stroke="#bfbfbf" stroke-opacity="1" stroke-width="1" stroke-linecap="square" stroke-linejoin="bevel" transform="matrix(1,0,0,1,775,480)" font-family="MS Shell Dlg 2" font-size="9.75" font-weight="400" font-style="normal" > <path vector-effect="none" fill-rule="evenodd" d="M0,1.7 L42.5,1.7 L42.5,0 L50,10 L42.5,20 L42.5,18.3 L0,18.3 L0,1.7"/> </g> <g fill="none" stroke="#000000" stroke-opacity="1" stroke-width="1" stroke-linecap="square" stroke-linejoin="bevel" transform="matrix(1,0,0,1,787.633,478)" font-family="MS Shell Dlg 2" font-size="9.75" font-weight="400" font-style="normal" > <text fill="#000000" fill-opacity="1" stroke="none" xml:space="preserve" x="4" y="17" font-family="Arial" font-size="10pt" font-weight="700" font-style="italic" >Q</text> </g> <g fill="#ff9999" fill-opacity="1" stroke="#bfbfbf" stroke-opacity="1" stroke-width="1" stroke-linecap="square" stroke-linejoin="bevel" transform="matrix(1,0,0,1,826,480)" font-family="MS Shell Dlg 2" font-size="9.75" font-weight="400" font-style="normal" > <path vector-effect="none" fill-rule="evenodd" d="M0,1.7 L42.5,1.7 L42.5,0 L50,10 L42.5,20 L42.5,18.3 L0,18.3 L0,1.7"/> </g> <g fill="none" stroke="#000000" stroke-opacity="1" stroke-width="1" stroke-linecap="square" stroke-linejoin="bevel" transform="matrix(1,0,0,1,839.633,478)" font-family="MS Shell Dlg 2" font-size="9.75" font-weight="400" font-style="normal" > <text fill="#000000" fill-opacity="1" stroke="none" xml:space="preserve" x="4" y="17" font-family="Arial" font-size="10pt" font-weight="700" font-style="italic" >Y</text> </g> <g fill="#ffff33" fill-opacity="1" stroke="#bfbfbf"

stroke-opacity="1" stroke-width="1" stroke-linecap="square" stroke-linejoin="bevel" transform="matrix(1,0,0,1,877,480)" font-family="MS Shell Dlg 2" font-size="9.75" font-weight="400" font-style="normal" > <rect x="0" y="0" width="50" height="20"/> </g> <g fill="none" stroke="#000000" stroke-opacity="1" stroke-width="1" stroke-linecap="square" stroke-linejoin="bevel" transform="matrix(1,0,0,1,886.5,478)" font-family="MS Shell Dlg 2" font-size="9.75" font-weight="400" font-style="normal" > <text fill="#000000" fill-opacity="1" stroke="none" xml:space="preserve" x="4" y="17" font-family="Arial" font-size="10pt" font-weight="700" font-style="italic" >cytb</text> </g> <g fill="#ffff33" fill-opacity="1" stroke="#bfbfbf" stroke-opacity="1" stroke-width="1" stroke-linecap="square" stroke-linejoin="bevel" transform="matrix(1,0,0,1,928,480)" font-family="MS Shell Dlg 2" font-size="9.75" font-weight="400" font-style="normal" > <rect x="0" y="0" width="50" height="20"/> </g> <g fill="none" stroke="#000000" stroke-opacity="1" stroke-width="1" stroke-linecap="square" stroke-linejoin="bevel" transform="matrix(1,0,0,1,936,478)" font-family="MS Shell Dlg 2" font-size="9.75" font-weight="400" font-style="normal" > <text fill="#000000" fill-opacity="1" stroke="none" xml:space="preserve" x="4" y="17" font-family="Arial" font-size="10pt" font-weight="700" font-style="italic" >cox2</text> </g> <g fill="#ff9999" fill-opacity="1" stroke="#bfbfbf" stroke-opacity="1" stroke-width="1" stroke-linecap="square" stroke-linejoin="bevel" transform="matrix(1,0,0,1,979,480)" font-family="MS Shell Dlg 2" font-size="9.75" font-weight="400" font-style="normal" > <path vector-effect="none" fill-rule="evenodd" d="M0,1.7 L42.5,1.7 L42.5,0 L50,10 L42.5,20 L42.5,18.3 L0,18.3 L0,1.7"/> </g> <g fill="none" stroke="#000000" stroke-opacity="1" stroke-width="1" stroke-linecap="square" stroke-linejoin="bevel" transform="matrix(1,0,0,1,992.633,478)" font-family="MS Shell Dlg 2" font-size="9.75" font-weight="400" font-style="normal" > <text fill="#000000" fill-opacity="1" stroke="none" xml:space="preserve" x="4" y="17" font-family="Arial" font-size="10pt" font-weight="700" font-style="italic" >K</text> </g> <g fill="#ff9999" fill-opacity="1" stroke="#bfbfbf" stroke-opacity="1" stroke-width="1" stroke-linecap="square" stroke-linejoin="bevel" transform="matrix(1,0,0,1,1030,480)" font-family="MS Shell Dlg 2" font-size="9.75" font-weight="400" font-style="normal" > <path vector-effect="none" fill-rule="evenodd" d="M0,1.7 L42.5,1.7 L42.5,0 L50,10 L42.5,20 L42.5,18.3 L0,18.3 L0,1.7"/> </g> <g fill="none" stroke="#000000" stroke-opacity="1" stroke-width="1" stroke-linecap="square" stroke-linejoin="bevel" transform="matrix(1,0,0,1,1042.13,478)" font-family="MS Shell Dlg 2" font-size="9.75" font-weight="400" font-style="normal" > <text fill="#000000" fill-opacity="1" stroke="none" xml:space="preserve" x="4" y="17" font-family="Arial" font-size="10pt" font-weight="700" font-style="italic" >M</text> </g> <g fill="#ff9999" fill-opacity="1" stroke="#bfbfbf" stroke-opacity="1" stroke-width="1" stroke-linecap="square" stroke-linejoin="bevel" transform="matrix(1,0,0,1,1081,480)" font-family="MS Shell Dlg 2" font-size="9.75" font-weight="400" font-style="normal" > <path vector-effect="none" fill-rule="evenodd" d="M0,1.7 L42.5,1.7 L42.5,0 L50,10 L42.5,20 L42.5,18.3 L0,18.3 L0,1.7"/> </g> <g fill="none" stroke="#000000" stroke-opacity="1" stroke-width="1" stroke-linecap="square" stroke-linejoin="bevel" transform="matrix(1,0,0,1,1091.63,478)" font-family="MS Shell Dlg 2" font-size="9.75" font-weight="400" font-style="normal" > <text fill="#000000" fill-opacity="1" stroke="none" xml:space="preserve" x="4" y="17" font-family="Arial" font-size="10pt" font-weight="700" font-style="italic" >L1</text> </g> <g fill="#ff9999" fill-opacity="1" stroke="#bfbfbf" stroke-opacity="1" stroke-width="1" stroke-linecap="square" stroke-linejoin="bevel" transform="matrix(1,0,0,1,1132,480)" font-family="MS Shell Dlg 2" font-size="9.75" font-weight="400" font-style="normal" > <path vector-effect="none" fill-rule="evenodd" d="M0,1.7 L42.5,1.7 L42.5,0 L50,10 L42.5,20 L42.5,18.3 L0,18.3 L0,1.7"/> </g> <g fill="none" stroke="#000000" stroke-opacity="1" stroke-width="1" stroke-linecap="square" stroke-linejoin="bevel" transform="matrix(1,0,0,1,1142.63,478)" font-family="MS Shell Dlg 2" font-size="9.75" font-weight="400" font-style="normal" > <text fill="#000000" fill-opacity="1" stroke="none" xml:space="preserve" x="4" y="17" font-family="Arial" font-size="10pt" font-weight="700" font-style="italic" >L2</text> </g> <g fill="#ffff33" fill-opacity="1" stroke="#bfbfbf" stroke-opacity="1" stroke-width="1" stroke-linecap="square" stroke-linejoin="bevel" transform="matrix(1,0,0,1,1183,480)" font-family="MS Shell Dlg 2" font-size="9.75" font-weight="400" font-style="normal" > <rect x="0" y="0" width="50" height="20"/> </g> <g fill="none" stroke="#000000" stroke-opacity="1" stroke-width="1" stroke-linecap="square" stroke-linejoin="bevel" transform="matrix(1,0,0,1,1190,478)" font-family="MS Shell Dlg 2" font-size="9.75" font-weight="400" font-style="normal" > <text fill="#000000" fill-opacity="1" stroke="none" xml:space="preserve" x="4" y="17" font-family="Arial" font-size="10pt" font-weight="700" font-style="italic" >nad1</text> </g> <g fill="#ff9999" fill-opacity="1" stroke="#bfbfbf" stroke-opacity="1" stroke-width="1" stroke-linecap="square" stroke-linejoin="bevel" transform="matrix(1,0,0,1,1234,480)" font-family="MS Shell Dlg 2" font-size="9.75" font-weight="400" font-style="normal" > <path vector-effect="none" fill-rule="evenodd" d="M0,1.7 L42.5,1.7 L42.5,0 L50,10 L42.5,20 L42.5,18.3 L0,18.3 L0,1.7"/> </g> <g fill="none" stroke="#000000" stroke-opacity="1" stroke-width="1" stroke-linecap="square" stroke-linejoin="bevel" transform="matrix(1,0,0,1,1247.13,478)" font-family="MS Shell Dlg 2" font-size="9.75" font-weight="400" font-style="normal" > <text fill="#000000" fill-

opacity="1" stroke="none" xml:space="preserve" x="4" y="17" font-family="Arial" font-size="10pt" font-weight="700" font-style="italic" >V</text> </g> <g fill="#ffff33" fill-opacity="1" stroke="#bfbfbf" stroke-opacity="1" stroke-width="1" stroke-linecap="square" stroke-linejoin="bevel" transform="matrix(1,0,0,1,1285,480)" font-family="MS Shell Dlg 2" font-size="9.75" font-weight="400" font-style="normal" > <rect x="0" y="0" width="50" height="20"/> </g> <g fill="none" stroke="#000000" stroke-opacity="1" stroke-width="1" stroke-linecap="square" stroke-linejoin="bevel" transform="matrix(1,0,0,1,1292,478)" font-family="MS Shell Dlg 2" font-size="9.75" font-weight="400" font-style="normal" > <text fill="#000000" fill-opacity="1" stroke="none" xml:space="preserve" x="4" y="17" font-family="Arial" font-size="10pt" font-weight="700" font-style="italic" >nad4</text> </g> <g fill="#ffff33" fill-opacity="1" stroke="#bfbfbf" stroke-opacity="1" stroke-width="1" stroke-linecap="square" stroke-linejoin="bevel" transform="matrix(1,0,0,1,1336,480)" font-family="MS Shell Dlg 2" font-size="9.75" font-weight="400" font-style="normal" > <rect x="0" y="0" width="50" height="20"/> </g> <g fill="none" stroke="#000000" stroke-opacity="1" stroke-width="1" stroke-linecap="square" stroke-linejoin="bevel" transform="matrix(1,0,0,1,1344,478)" font-family="MS Shell Dlg 2" font-size="9.75" font-weight="400" font-style="normal" > <text fill="#000000" fill-opacity="1" stroke="none" xml:space="preserve" x="4" y="17" font-family="Arial" font-size="10pt" font-weight="700" font-style="italic" >cox3</text> </g> <g fill="#ff9999" fill-opacity="1" stroke="#bfbfbf" stroke-opacity="1" stroke-width="1" stroke-linecap="square" stroke-linejoin="bevel" transform="matrix(1,0,0,1,1387,480)" font-family="MS Shell Dlg 2" font-size="9.75" font-weight="400" font-style="normal" > <path vector-effect="none" fill-rule="evenodd" d="M0,1.7 L42.5,1.7 L42.5,0 L50,10 L42.5,20 L42.5,18.3 L0,18.3 L0,1.7"/> </g> <g fill="none" stroke="#000000" stroke-opacity="1" stroke-width="1" stroke-linecap="square" stroke-linejoin="bevel" transform="matrix(1,0,0,1,1396.63,478)" font-family="MS Shell Dlg 2" font-size="9.75" font-weight="400" font-style="normal" > <text fill="#000000" fill-opacity="1" stroke="none" xml:space="preserve" x="4" y="17" font-family="Arial" font-size="10pt" font-weight="700" font-style="italic" >S2</text> </g> <g fill="#ff9999" fill-opacity="1" stroke="#bfbfbf" stroke-opacity="1" stroke-width="1" stroke-linecap="square" stroke-linejoin="bevel" transform="matrix(1,0,0,1,1438,480)" font-family="MS Shell Dlg 2" font-size="9.75" font-weight="400" font-style="normal" > <path vector-effect="none" fill-rule="evenodd" d="M0,1.7 L42.5,1.7 L42.5,0 L50,10 L42.5,20 L42.5,18.3 L0,18.3 L0,1.7"/> </g> <g fill="none" stroke="#000000" stroke-opacity="1" stroke-width="1" stroke-linecap="square" stroke-linejoin="bevel" transform="matrix(1,0,0,1,1450.13,478)" font-family="MS Shell Dlg 2" font-size="9.75" font-weight="400" font-style="normal" > <text fill="#000000" fill-opacity="1" stroke="none" xml:space="preserve" x="4" y="17" font-family="Arial" font-size="10pt" font-weight="700" font-style="italic" >M</text> </g> <g fill="#ffff33" fill-opacity="1" stroke="#bfbfbf" stroke-opacity="1" stroke-width="1" stroke-linecap="square" stroke-linejoin="bevel" transform="matrix(1,0,0,1,1489,480)" font-family="MS Shell Dlg 2" font-size="9.75" font-weight="400" font-style="normal" > <rect x="0" y="0" width="50" height="20"/> </g> <g fill="none" stroke="#000000" stroke-opacity="1" stroke-width="1" stroke-linecap="square" stroke-linejoin="bevel" transform="matrix(1,0,0,1,1496,478)" font-family="MS Shell Dlg 2" font-size="9.75" font-weight="400" font-style="normal" > <text fill="#000000" fill-opacity="1" stroke="none" xml:space="preserve" x="4" y="17" font-family="Arial" font-size="10pt" font-weight="700" font-style="italic" >nad2</text> </g> <g fill="#ff9999" fill-opacity="1" stroke="#bfbfbf" stroke-opacity="1" stroke-width="1" stroke-linecap="square" stroke-linejoin="bevel" transform="matrix(1,0,0,1,1540,480)" font-family="MS Shell Dlg 2" font-size="9.75" font-weight="400" font-style="normal" > <path vector-effect="none" fill-rule="evenodd" d="M0,1.7 L42.5,1.7 L42.5,0 L50,10 L42.5,20 L42.5,18.3 L0,18.3 L0,1.7"/> </g> <g fill="none" stroke="#000000" stroke-opacity="1" stroke-width="1" stroke-linecap="square" stroke-linejoin="bevel" transform="matrix(1,0,0,1,1553.13,478)" font-family="MS Shell Dlg 2" font-size="9.75" font-weight="400" font-style="normal" > <text fill="#000000" fill-opacity="1" stroke="none" xml:space="preserve" x="4" y="17" font-family="Arial" font-size="10pt" font-weight="700" font-style="italic" >R</text> </g> <g fill="#ff9999" fill-opacity="1" stroke="#bfbfbf" stroke-opacity="1" stroke-width="1" stroke-linecap="square" stroke-linejoin="bevel" transform="matrix(1,0,0,1,1591,480)" font-family="MS Shell Dlg 2" font-size="9.75" font-weight="400" font-style="normal" > <path vector-effect="none" fill-rule="evenodd" d="M0,1.7 L42.5,1.7 L42.5,0 L50,10 L42.5,20 L42.5,18.3 L0,18.3 L0,1.7"/> </g> <g fill="none" stroke="#000000" stroke-opacity="1" stroke-width="1" stroke-linecap="square" stroke-linejoin="bevel" transform="matrix(1,0,0,1,1602.13,478)" font-family="MS Shell Dlg 2" font-size="9.75" font-weight="400" font-style="normal" > <text fill="#000000" fill-opacity="1" stroke="none" xml:space="preserve" x="4" y="17" font-family="Arial" font-size="10pt" font-weight="700" font-style="italic" >W</text> </g> <g fill="#ff9999" fill-opacity="1" stroke="#bfbfbf" stroke-opacity="1" stroke-width="1" stroke-linecap="square" stroke-linejoin="bevel" transform="matrix(1,0,0,1,1642,480)" font-family="MS Shell Dlg 2" font-size="9.75" font-weight="400" font-style="normal" > <path vector-effect="none" fill-rule="evenodd" d="M0,1.7 L42.5,1.7 L42.5,0 L50,10 L42.5,20 L42.5,18.3 L0,18.3 L0,1.7"/> </g> <g fill="none" stroke="#000000" stroke-opacity="1" stroke-width="1"

stroke-linecap="square" stroke-linejoin="bevel" transform="matrix(1,0,0,1,1655.13,478)" font-family="MS Shell Dlg 2" font-size="9.75" font-weight="400" font-style="normal" > <text fill="#000000" fill-opacity="1" stroke="none" xml:space="preserve" x="4" y="17" font-family="Arial" font-size="10pt" font-weight="700" font-style="italic" >A</text> </g> <g fill="#ff9999" fill-opacity="1" stroke="#bfbfbf" stroke-opacity="1" stroke-width="1" stroke-linecap="square" stroke-linejoin="bevel" transform="matrix(1,0,0,1,1693,480)" font-family="MS Shell Dlg 2" font-size="9.75" font-weight="400" font-style="normal" > <path vector-effect="none" fill-rule="evenodd" d="M0,1.7 L42.5,1.7 L42.5,0 L50,10 L42.5,20 L42.5,18.3 L0,18.3 L0,1.7"/> </g> <g fill="none" stroke="#000000" stroke-opacity="1" stroke-width="1" stroke-linecap="square" stroke-linejoin="bevel" transform="matrix(1,0,0,1,1702.63,478)" font-family="MS Shell Dlg 2" font-size="9.75" font-weight="400" font-style="normal" > <text fill="#000000" fill-opacity="1" stroke="none" xml:space="preserve" x="4" y="17" font-family="Arial" font-size="10pt" font-weight="700" font-style="italic" >S1</text> </g> <g fill="#ff9999" fill-opacity="1" stroke="#bfbfbf" stroke-opacity="1" stroke-width="1" stroke-linecap="square" stroke-linejoin="bevel" transform="matrix(1,0,0,1,1744,480)" font-family="MS Shell Dlg 2" font-size="9.75" font-weight="400" font-style="normal" > <path vector-effect="none" fill-rule="evenodd" d="M0,1.7 L42.5,1.7 L42.5,0 L50,10 L42.5,20 L42.5,18.3 L0,18.3 L0,1.7"/> </g> <g fill="none" stroke="#000000" stroke-opacity="1" stroke-width="1" stroke-linecap="square" stroke-linejoin="bevel" transform="matrix(1,0,0,1,1757.13,478)" font-family="MS Shell Dlg 2" font-size="9.75" font-weight="400" font-style="normal" > <text fill="#000000" fill-opacity="1" stroke="none" xml:space="preserve" x="4" y="17" font-family="Arial" font-size="10pt" font-weight="700" font-style="italic" >H</text> </g> <g fill="#ff9999" fill-opacity="1" stroke="#bfbfbf" stroke-opacity="1" stroke-width="1" stroke-linecap="square" stroke-linejoin="bevel" transform="matrix(1,0,0,1,1795,480)" font-family="MS Shell Dlg 2" font-size="9.75" font-weight="400" font-style="normal" > <path vector-effect="none" fill-rule="evenodd" d="M0,1.7 L42.5,1.7 L42.5,0 L50,10 L42.5,20 L42.5,18.3 L0,18.3 L0,1.7"/> </g> <g fill="none" stroke="#000000" stroke-opacity="1" stroke-width="1" stroke-linecap="square" stroke-linejoin="bevel" transform="matrix(1,0,0,1,1808.63,478)" font-family="MS Shell Dlg 2" font-size="9.75" font-weight="400" font-style="normal" > <text fill="#000000" fill-opacity="1" stroke="none" xml:space="preserve" x="4" y="17" font-family="Arial" font-size="10pt" font-weight="700" font-style="italic" >P</text> </g> <g fill="#ffff33" fill-opacity="1" stroke="#bfbfbf" stroke-opacity="1" stroke-width="1" stroke-linecap="square" stroke-linejoin="bevel" transform="matrix(1,0,0,1,1846,480)" font-family="MS Shell Dlg 2" font-size="9.75" font-weight="400" font-style="normal" > <rect x="0" y="0" width="50" height="20"/> </g> <g fill="none" stroke="#000000" stroke-opacity="1" stroke-width="1" stroke-linecap="square" stroke-linejoin="bevel" transform="matrix(1,0,0,1,1853,478)" font-family="MS Shell Dlg 2" font-size="9.75" font-weight="400" font-style="normal" > <text fill="#000000" fill-opacity="1" stroke="none" xml:space="preserve" x="4" y="17" font-family="Arial" font-size="10pt" font-weight="700" font-style="italic" >nad3</text> </g> <g fill="#ffff33" fill-opacity="1" stroke="#bfbfbf" stroke-opacity="1" stroke-width="1" stroke-linecap="square" stroke-linejoin="bevel" transform="matrix(1,0,0,1,1897,480)" font-family="MS Shell Dlg 2" font-size="9.75" font-weight="400" font-style="normal" > <rect x="0" y="0" width="50" height="20"/> </g> <g fill="none" stroke="#000000" stroke-opacity="1" stroke-width="1" stroke-linecap="square" stroke-linejoin="bevel" transform="matrix(1,0,0,1,1905.5,478)" font-family="MS Shell Dlg 2" font-size="9.75" font-weight="400" font-style="normal" > <text fill="#000000" fill-opacity="1" stroke="none" xml:space="preserve" x="4" y="17" font-family="Arial" font-size="10pt" font-weight="700" font-style="italic" >atp8</text> </g> <g fill="none" stroke="#000000" stroke-opacity="1" stroke-width="1" stroke-linecap="square" stroke-linejoin="bevel" transform="matrix(1,0,0,1,1948,480)" font-family="MS Shell Dlg 2" font-size="9.75" font-weight="400" font-style="normal" > <text fill="#000000" fill-opacity="1" stroke="none" xml:space="preserve" x="4" y="17" font-family="Arial" font-size="10pt" font-weight="700" font-style="italic" >(+)</text> </g> <g fill="none" stroke="#000000" stroke-opacity="1" stroke-width="1" stroke-linecap="square" stroke-linejoin="bevel" transform="matrix(1,0,0,1,10,520)" font-family="MS Shell Dlg 2" font-size="9.75" font-weight="400" font-style="normal" > <text fill="#000000" fill-opacity="1" stroke="none" xml:space="preserve" x="4" y="17" font-family="Arial" font-size="10pt" font-weight="700" font-style="italic" >Mytilus_trossulus_HM462080</text> </g> <g fill="#ffff33" fill-opacity="1" stroke="#bfbfbf" stroke-opacity="1" stroke-width="1" stroke-linecap="square" stroke-linejoin="bevel" transform="matrix(1,0,0,1,10,550)" font-family="MS Shell Dlg 2" font-size="9.75" font-weight="400" font-style="normal" > <rect x="0" y="0" width="50" height="20"/> </g> <g fill="none" stroke="#000000" stroke-opacity="1" stroke-width="1" stroke-linecap="square" stroke-linejoin="bevel" transform="matrix(1,0,0,1,18,548)" font-family="MS Shell Dlg 2" font-size="9.75" font-weight="400" font-style="normal" > <text fill="#000000" fill-opacity="1" stroke="none" xml:space="preserve" x="4" y="17" font-family="Arial" font-size="10pt" font-weight="700" font-style="italic" >cox1</text> </g> <g fill="#ffff33" fill-opacity="1" stroke="#bfbfbf" stroke-opacity="1" stroke-width="1" stroke-linecap="square" stroke-linejoin="bevel" transform="matrix(1,0,0,1,61,550)" font-family="MS Shell Dlg 2" font-size="9.75" font-weight="400" font-style="normal" > <rect x="0" y="0" width="50" height="20"/> </g>

<g fill="none" stroke="#000000" stroke-opacity="1" stroke-width="1" stroke-linecap="square" stroke-linejoin="bevel" transform="matrix(1,0,0,1,69.5,548)" font-family="MS Shell Dlg 2" font-size="9.75" font-weight="400" font-style="normal" > <text fill="#000000" fill-opacity="1" stroke="none" xml:space="preserve" x="4" y="17" font-family="Arial" font-size="10pt" font-weight="700" font-style="italic" >atp6</text> </g> <g fill="#ff9999" fill-opacity="1" stroke="#bfbfbf" stroke-opacity="1" stroke-width="1" stroke-linecap="square" stroke-linejoin="bevel" transform="matrix(1,0,0,1,112,550)" font-family="MS Shell Dlg 2" font-size="9.75" font-weight="400" font-style="normal" > <path vector-effect="none" fill-rule="evenodd" d="M0,1.7 L42.5,1.7 L42.5,0 L50,10 L42.5,20 L42.5,18.3 L0,18.3 L0,1.7"/> </g> <g fill="none" stroke="#000000" stroke-opacity="1" stroke-width="1" stroke-linecap="square" stroke-linejoin="bevel" transform="matrix(1,0,0,1,125.132,548)" font-family="MS Shell Dlg 2" font-size="9.75" font-weight="400" font-style="normal" > <text fill="#000000" fill-opacity="1" stroke="none" xml:space="preserve" x="4" y="17" font-family="Arial" font-size="10pt" font-weight="700" font-style="italic" >T</text> </g> <g fill="#ffff33" fill-opacity="1" stroke="#bfbfbf" stroke-opacity="1" stroke-width="1" stroke-linecap="square" stroke-linejoin="bevel" transform="matrix(1,0,0,1,163,550)" font-family="MS Shell Dlg 2" font-size="9.75" font-weight="400" font-style="normal" > <rect x="0" y="0" width="50" height="20"/> </g> <g fill="none" stroke="#000000" stroke-opacity="1" stroke-width="1" stroke-linecap="square" stroke-linejoin="bevel" transform="matrix(1,0,0,1,167,548)" font-family="MS Shell Dlg 2" font-size="9.75" font-weight="400" font-style="normal" > <text fill="#000000" fill-opacity="1" stroke="none" xml:space="preserve" x="4" y="17" font-family="Arial" font-size="10pt" font-weight="700" font-style="italic" >nad4L</text> </g> <g fill="#ffff33" fill-opacity="1" stroke="#bfbfbf" stroke-opacity="1" stroke-width="1" stroke-linecap="square" stroke-linejoin="bevel" transform="matrix(1,0,0,1,214,550)" font-family="MS Shell Dlg 2" font-size="9.75" font-weight="400" font-style="normal" > <rect x="0" y="0" width="50" height="20"/> </g> <g fill="none" stroke="#000000" stroke-opacity="1" stroke-width="1" stroke-linecap="square" stroke-linejoin="bevel" transform="matrix(1,0,0,1,221,548)" font-family="MS Shell Dlg 2" font-size="9.75" font-weight="400" font-style="normal" > <text fill="#000000" fill-opacity="1" stroke="none" xml:space="preserve" x="4" y="17" font-family="Arial" font-size="10pt" font-weight="700" font-style="italic" >nad5</text> </g> <g fill="#ffff33" fill-opacity="1" stroke="#bfbfbf" stroke-opacity="1" stroke-width="1" stroke-linecap="square" stroke-linejoin="bevel" transform="matrix(1,0,0,1,265,550)" font-family="MS Shell Dlg 2" font-size="9.75" font-weight="400" font-style="normal" > <rect x="0" y="0" width="50" height="20"/> </g> <g fill="none" stroke="#000000" stroke-opacity="1" stroke-width="1" stroke-linecap="square" stroke-linejoin="bevel" transform="matrix(1,0,0,1,272,548)" font-family="MS Shell Dlg 2" font-size="9.75" font-weight="400" font-style="normal" > <text fill="#000000" fill-opacity="1" stroke="none" xml:space="preserve" x="4" y="17" font-family="Arial" font-size="10pt" font-weight="700" font-style="italic" >nad6</text> </g> <g fill="#ff9999" fill-opacity="1" stroke="#bfbfbf" stroke-opacity="1" stroke-width="1" stroke-linecap="square" stroke-linejoin="bevel" transform="matrix(1,0,0,1,316,550)" font-family="MS Shell Dlg 2" font-size="9.75" font-weight="400" font-style="normal" > <path vector-effect="none" fill-rule="evenodd" d="M0,1.7 L42.5,1.7 L42.5,0 L50,10 L42.5,20 L42.5,18.3 L0,18.3 L0,1.7"/> </g> <g fill="none" stroke="#000000" stroke-opacity="1" stroke-width="1" stroke-linecap="square" stroke-linejoin="bevel" transform="matrix(1,0,0,1,329.632,548)" font-family="MS Shell Dlg 2" font-size="9.75" font-weight="400" font-style="normal" > <text fill="#000000" fill-opacity="1" stroke="none" xml:space="preserve" x="4" y="17" font-family="Arial" font-size="10pt" font-weight="700" font-style="italic" >F</text> </g> <g fill="#99ffff" fill-opacity="1" stroke="#bfbfbf" stroke-opacity="1" stroke-width="1" stroke-linecap="square" stroke-linejoin="bevel" transform="matrix(1,0,0,1,367,550)" font-family="MS Shell Dlg 2" font-size="9.75" font-weight="400" font-style="normal" > <path vector-effect="none" fill-rule="evenodd" d="M0,5 C0,2.23858 2.23858,0 5,0 L45,0 C47.7614,0 50,2.23858 50,5 L50,15 C50,17.7614 47.7614,20 45,20 L5,20 C2.23858,20 0,17.7614 0,15 L0,5"/> </g> <g fill="none" stroke="#000000" stroke-opacity="1" stroke-width="1" stroke-linecap="square" stroke-linejoin="bevel" transform="matrix(1,0,0,1,375.5,548)" font-family="MS Shell Dlg 2" font-size="9.75" font-weight="400" font-style="normal" > <text fill="#000000" fill-opacity="1" stroke="none" xml:space="preserve" x="4" y="17" font-family="Arial" font-size="10pt" font-weight="700" font-style="italic" >rrnS</text> </g> <g fill="#ff9999" fill-opacity="1" stroke="#bfbfbf" stroke-opacity="1" stroke-width="1" stroke-linecap="square" stroke-linejoin="bevel" transform="matrix(1,0,0,1,418,550)" font-family="MS Shell Dlg 2" font-size="9.75" font-weight="400" font-style="normal" > <path vector-effect="none" fill-rule="evenodd" d="M0,1.7 L42.5,1.7 L42.5,0 L50,10 L42.5,20 L42.5,18.3 L0,18.3 L0,1.7"/> </g> <g fill="none" stroke="#000000" stroke-opacity="1" stroke-width="1" stroke-linecap="square" stroke-linejoin="bevel" transform="matrix(1,0,0,1,431.132,548)" font-family="MS Shell Dlg 2" font-size="9.75" font-weight="400" font-style="normal" > <text fill="#000000" fill-opacity="1" stroke="none" xml:space="preserve" x="4" y="17" font-family="Arial" font-size="10pt" font-weight="700" font-style="italic" >G</text> </g> <g fill="#ff9999" fill-opacity="1" stroke="#bfbfbf" stroke-opacity="1" stroke-width="1" stroke-linecap="square" stroke-linejoin="bevel"

transform="matrix(1,0,0,1,469,550)" font-family="MS Shell Dlg 2" font-size="9.75" font-weight="400" font-style="normal" > <path vector-effect="none" fill-rule="evenodd" d="M0,1.7 L42.5,1.7 L42.5,0 L50,10 L42.5,20 L42.5,18.3 L0,18.3 L0,1.7"/> </g> <g fill="none" stroke="#000000" stroke-opacity="1" stroke-width="1" stroke-linecap="square" stroke-linejoin="bevel" transform="matrix(1,0,0,1,482.132,548)" font-family="MS Shell Dlg 2" font-size="9.75" font-weight="400" font-style="normal" > <text fill="#000000" fill-opacity="1" stroke="none" xml:space="preserve" x="4" y="17" font-family="Arial" font-size="10pt" font-weight="700" font-style="italic" >N</text> </g> <g fill="#ff9999" fill-opacity="1" stroke="#bfbfbf" stroke-opacity="1" stroke-width="1" stroke-linecap="square" stroke-linejoin="bevel" transform="matrix(1,0,0,1,520,550)" font-family="MS Shell Dlg 2" font-size="9.75" font-weight="400" font-style="normal" > <path vector-effect="none" fill-rule="evenodd" d="M0,1.7 L42.5,1.7 L42.5,0 L50,10 L42.5,20 L42.5,18.3 L0,18.3 L0,1.7"/> </g> <g fill="none" stroke="#000000" stroke-opacity="1" stroke-width="1" stroke-linecap="square" stroke-linejoin="bevel" transform="matrix(1,0,0,1,533.633,548)" font-family="MS Shell Dlg 2" font-size="9.75" font-weight="400" font-style="normal" > <text fill="#000000" fill-opacity="1" stroke="none" xml:space="preserve" x="4" y="17" font-family="Arial" font-size="10pt" font-weight="700" font-style="italic" >E</text> </g> <g fill="#ff9999" fill-opacity="1" stroke="#bfbfbf" stroke-opacity="1" stroke-width="1" stroke-linecap="square" stroke-linejoin="bevel" transform="matrix(1,0,0,1,571,550)" font-family="MS Shell Dlg 2" font-size="9.75" font-weight="400" font-style="normal" > <path vector-effect="none" fill-rule="evenodd" d="M0,1.7 L42.5,1.7 L42.5,0 L50,10 L42.5,20 L42.5,18.3 L0,18.3 L0,1.7"/> </g> <g fill="none" stroke="#000000" stroke-opacity="1" stroke-width="1" stroke-linecap="square" stroke-linejoin="bevel" transform="matrix(1,0,0,1,584.133,548)" font-family="MS Shell Dlg 2" font-size="9.75" font-weight="400" font-style="normal" > <text fill="#000000" fill-opacity="1" stroke="none" xml:space="preserve" x="4" y="17" font-family="Arial" font-size="10pt" font-weight="700" font-style="italic" >C</text> </g> <g fill="#ff9999" fill-opacity="1" stroke="#bfbfbf" stroke-opacity="1" stroke-width="1" stroke-linecap="square" stroke-linejoin="bevel" transform="matrix(1,0,0,1,622,550)" font-family="MS Shell Dlg 2" font-size="9.75" font-weight="400" font-style="normal" > <path vector-effect="none" fill-rule="evenodd" d="M0,1.7 L42.5,1.7 L42.5,0 L50,10 L42.5,20 L42.5,18.3 L0,18.3 L0,1.7"/> </g> <g fill="none" stroke="#000000" stroke-opacity="1" stroke-width="1" stroke-linecap="square" stroke-linejoin="bevel" transform="matrix(1,0,0,1,637.133,548)" font-family="MS Shell Dlg 2" font-size="9.75" font-weight="400" font-style="normal" > <text fill="#000000" fill-opacity="1" stroke="none" xml:space="preserve" x="4" y="17" font-family="Arial" font-size="10pt" font-weight="700" font-style="italic" >I</text> </g> <g fill="#ff9999" fill-opacity="1" stroke="#bfbfbf" stroke-opacity="1" stroke-width="1" stroke-linecap="square" stroke-linejoin="bevel" transform="matrix(1,0,0,1,673,550)" font-family="MS Shell Dlg 2" font-size="9.75" font-weight="400" font-style="normal" > <path vector-effect="none" fill-rule="evenodd" d="M0,1.7 L42.5,1.7 L42.5,0 L50,10 L42.5,20 L42.5,18.3 L0,18.3 L0,1.7"/> </g> <g fill="none" stroke="#000000" stroke-opacity="1" stroke-width="1" stroke-linecap="square" stroke-linejoin="bevel" transform="matrix(1,0,0,1,686.133,548)" font-family="MS Shell Dlg 2" font-size="9.75" font-weight="400" font-style="normal" > <text fill="#000000" fill-opacity="1" stroke="none" xml:space="preserve" x="4" y="17" font-family="Arial" font-size="10pt" font-weight="700" font-style="italic" >D</text> </g> <g fill="#99ffff" fill-opacity="1" stroke="#bfbfbf" stroke-opacity="1" stroke-width="1" stroke-linecap="square" stroke-linejoin="bevel" transform="matrix(1,0,0,1,724,550)" font-family="MS Shell Dlg 2" font-size="9.75" font-weight="400" font-style="normal" > <path vector-effect="none" fill-rule="evenodd" d="M0,5 C0,2.23858 2.23858,0 5,0 L45,0 C47.7614,0 50,2.23858 50,5 L50,15 C50,17.7614 47.7614,20 45,20 L5,20 C2.23858,20 0,17.7614 0,15 L0,5"/> </g> <g fill="none" stroke="#000000" stroke-opacity="1" stroke-width="1" stroke-linecap="square" stroke-linejoin="bevel" transform="matrix(1,0,0,1,733.5,548)" font-family="MS Shell Dlg 2" font-size="9.75" font-weight="400" font-style="normal" > <text fill="#000000" fill-opacity="1" stroke="none" xml:space="preserve" x="4" y="17" font-family="Arial" font-size="10pt" font-weight="700" font-style="italic" >rrnL</text> </g> <g fill="#ff9999" fill-opacity="1" stroke="#bfbfbf" stroke-opacity="1" stroke-width="1" stroke-linecap="square" stroke-linejoin="bevel" transform="matrix(1,0,0,1,775,550)" font-family="MS Shell Dlg 2" font-size="9.75" font-weight="400" font-style="normal" > <path vector-effect="none" fill-rule="evenodd" d="M0,1.7 L42.5,1.7 L42.5,0 L50,10 L42.5,20 L42.5,18.3 L0,18.3 L0,1.7"/> </g> <g fill="none" stroke="#000000" stroke-opacity="1" stroke-width="1" stroke-linecap="square" stroke-linejoin="bevel" transform="matrix(1,0,0,1,787.633,548)" font-family="MS Shell Dlg 2" font-size="9.75" font-weight="400" font-style="normal" > <text fill="#000000" fill-opacity="1" stroke="none" xml:space="preserve" x="4" y="17" font-family="Arial" font-size="10pt" font-weight="700" font-style="italic" >Q</text> </g> <g fill="#ff9999" fill-opacity="1" stroke="#bfbfbf" stroke-opacity="1" stroke-width="1" stroke-linecap="square" stroke-linejoin="bevel" transform="matrix(1,0,0,1,826,550)" font-family="MS Shell Dlg 2" font-size="9.75" font-weight="400" font-style="normal" > <path vector-effect="none" fill-rule="evenodd" d="M0,1.7 L42.5,1.7 L42.5,0 L50,10 L42.5,20 L42.5,18.3 L0,18.3 L0,1.7"/> </g> <g fill="none" stroke="#000000" stroke-opacity="1" stroke-width="1" stroke-linecap="square" stroke-linejoin="bevel" transform="matrix(1,0,0,1,839.633,548)" font-

family="MS Shell Dlg 2" font-size="9.75" font-weight="400" font-style="normal" > <text fill="#000000" fill-opacity="1" stroke="none" xml:space="preserve" x="4" y="17" font-family="Arial" font-size="10pt" font-weight="700" font-style="italic" >Y</text> </g> <g fill="#ffff33" fill-opacity="1" stroke="#bfbfbf" stroke-opacity="1" stroke-width="1" stroke-linecap="square" stroke-linejoin="bevel" transform="matrix(1,0,0,1,877,550)" font-family="MS Shell Dlg 2" font-size="9.75" font-weight="400" font-style="normal" > <rect x="0" y="0" width="50" height="20"/> </g> <g fill="none" stroke="#000000" stroke-opacity="1" stroke-width="1" stroke-linecap="square" stroke-linejoin="bevel" transform="matrix(1,0,0,1,886.5,548)" font-family="MS Shell Dlg 2" font-size="9.75" font-weight="400" font-style="normal" > <text fill="#000000" fill-opacity="1" stroke="none" xml:space="preserve" x="4" y="17" font-family="Arial" font-size="10pt" font-weight="700" font-style="italic" >cytb</text> </g> <g fill="#ffff33" fill-opacity="1" stroke="#bfbfbf" stroke-opacity="1" stroke-width="1" stroke-linecap="square" stroke-linejoin="bevel" transform="matrix(1,0,0,1,928,550)" font-family="MS Shell Dlg 2" font-size="9.75" font-weight="400" font-style="normal" > <rect x="0" y="0" width="50" height="20"/> </g> <g fill="none" stroke="#000000" stroke-opacity="1" stroke-width="1" stroke-linecap="square" stroke-linejoin="bevel" transform="matrix(1,0,0,1,936,548)" font-family="MS Shell Dlg 2" font-size="9.75" font-weight="400" font-style="normal" > <text fill="#000000" fill-opacity="1" stroke="none" xml:space="preserve" x="4" y="17" font-family="Arial" font-size="10pt" font-weight="700" font-style="italic" >cox2</text> </g> <g fill="#ff9999" fill-opacity="1" stroke="#bfbfbf" stroke-opacity="1" stroke-width="1" stroke-linecap="square" stroke-linejoin="bevel" transform="matrix(1,0,0,1,979,550)" font-family="MS Shell Dlg 2" font-size="9.75" font-weight="400" font-style="normal" > <path vector-effect="none" fill-rule="evenodd" d="M0,1.7 L42.5,1.7 L42.5,0 L50,10 L42.5,20 L42.5,18.3 L0,18.3 L0,1.7"/> </g> <g fill="none" stroke="#000000" stroke-opacity="1" stroke-width="1" stroke-linecap="square" stroke-linejoin="bevel" transform="matrix(1,0,0,1,992.633,548)" font-family="MS Shell Dlg 2" font-size="9.75" font-weight="400" font-style="normal" > <text fill="#000000" fill-opacity="1" stroke="none" xml:space="preserve" x="4" y="17" font-family="Arial" font-size="10pt" font-weight="700" font-style="italic" >K</text> </g> <g fill="#ff9999" fill-opacity="1" stroke="#bfbfbf" stroke-opacity="1" stroke-width="1" stroke-linecap="square" stroke-linejoin="bevel" transform="matrix(1,0,0,1,1030,550)" font-family="MS Shell Dlg 2" font-size="9.75" font-weight="400" font-style="normal" > <path vector-effect="none" fill-rule="evenodd" d="M0,1.7 L42.5,1.7 L42.5,0 L50,10 L42.5,20 L42.5,18.3 L0,18.3 L0,1.7"/> </g> <g fill="none" stroke="#000000" stroke-opacity="1" stroke-width="1" stroke-linecap="square" stroke-linejoin="bevel" transform="matrix(1,0,0,1,1042.13,548)" font-family="MS Shell Dlg 2" font-size="9.75" font-weight="400" font-style="normal" > <text fill="#000000" fill-opacity="1" stroke="none" xml:space="preserve" x="4" y="17" font-family="Arial" font-size="10pt" font-weight="700" font-style="italic" >M</text> </g> <g fill="#ff9999" fill-opacity="1" stroke="#bfbfbf" stroke-opacity="1" stroke-width="1" stroke-linecap="square" stroke-linejoin="bevel" transform="matrix(1,0,0,1,1081,550)" font-family="MS Shell Dlg 2" font-size="9.75" font-weight="400" font-style="normal" > <path vector-effect="none" fill-rule="evenodd" d="M0,1.7 L42.5,1.7 L42.5,0 L50,10 L42.5,20 L42.5,18.3 L0,18.3 L0,1.7"/> </g> <g fill="none" stroke="#000000" stroke-opacity="1" stroke-width="1" stroke-linecap="square" stroke-linejoin="bevel" transform="matrix(1,0,0,1,1095.13,548)" font-family="MS Shell Dlg 2" font-size="9.75" font-weight="400" font-style="normal" > <text fill="#000000" fill-opacity="1" stroke="none" xml:space="preserve" x="4" y="17" font-family="Arial" font-size="10pt" font-weight="700" font-style="italic" >L</text> </g> <g fill="#ff9999" fill-opacity="1" stroke="#bfbfbf" stroke-opacity="1" stroke-width="1" stroke-linecap="square" stroke-linejoin="bevel" transform="matrix(1,0,0,1,1132,550)" font-family="MS Shell Dlg 2" font-size="9.75" font-weight="400" font-style="normal" > <path vector-effect="none" fill-rule="evenodd" d="M0,1.7 L42.5,1.7 L42.5,0 L50,10 L42.5,20 L42.5,18.3 L0,18.3 L0,1.7"/> </g> <g fill="none" stroke="#000000" stroke-opacity="1" stroke-width="1" stroke-linecap="square" stroke-linejoin="bevel" transform="matrix(1,0,0,1,1146.13,548)" font-family="MS Shell Dlg 2" font-size="9.75" font-weight="400" font-style="normal" > <text fill="#000000" fill-opacity="1" stroke="none" xml:space="preserve" x="4" y="17" font-family="Arial" font-size="10pt" font-weight="700" font-style="italic" >L</text> </g> <g fill="#ffff33" fill-opacity="1" stroke="#bfbfbf" stroke-opacity="1" stroke-width="1" stroke-linecap="square" stroke-linejoin="bevel" transform="matrix(1,0,0,1,1183,550)" font-family="MS Shell Dlg 2" font-size="9.75" font-weight="400" font-style="normal" > <rect x="0" y="0" width="50" height="20"/> </g> <g fill="none" stroke="#000000" stroke-opacity="1" stroke-width="1" stroke-linecap="square" stroke-linejoin="bevel" transform="matrix(1,0,0,1,1190,548)" font-family="MS Shell Dlg 2" font-size="9.75" font-weight="400" font-style="normal" > <text fill="#000000" fill-opacity="1" stroke="none" xml:space="preserve" x="4" y="17" font-family="Arial" font-size="10pt" font-weight="700" font-style="italic" >nad1</text> </g> <g fill="#ff9999" fill-opacity="1" stroke="#bfbfbf" stroke-opacity="1" stroke-width="1" stroke-linecap="square" stroke-linejoin="bevel" transform="matrix(1,0,0,1,1234,550)" font-family="MS Shell Dlg 2" font-size="9.75" font-weight="400" font-style="normal" > <path vector-effect="none" fill-rule="evenodd" d="M0,1.7 L42.5,1.7 L42.5,0

L50,10 L42.5,20 L42.5,18.3 L0,18.3 L0,1.7"/> </g> <g fill="none" stroke="#000000" stroke-opacity="1" stroke-width="1" stroke-linecap="square" stroke-linejoin="bevel" transform="matrix(1,0,0,1,1247.13,548)" font-family="MS Shell Dlg 2" font-size="9.75" font-weight="400" font-style="normal" > <text fill="#000000" fill-opacity="1" stroke="none" xml:space="preserve" x="4" y="17" font-family="Arial" font-size="10pt" font-weight="700" font-style="italic" >V</text> </g> <g fill="#ffff33" fill-opacity="1" stroke="#bfbfbf" stroke-opacity="1" stroke-width="1" stroke-linecap="square" stroke-linejoin="bevel" transform="matrix(1,0,0,1,1285,550)" font-family="MS Shell Dlg 2" font-size="9.75" font-weight="400" font-style="normal" > <rect x="0" y="0" width="50" height="20"/> </g> <g fill="none" stroke="#000000" stroke-opacity="1" stroke-width="1" stroke-linecap="square" stroke-linejoin="bevel" transform="matrix(1,0,0,1,1292,548)" font-family="MS Shell Dlg 2" font-size="9.75" font-weight="400" font-style="normal" > <text fill="#000000" fill-opacity="1" stroke="none" xml:space="preserve" x="4" y="17" font-family="Arial" font-size="10pt" font-weight="700" font-style="italic" >nad4</text> </g> <g fill="#ffff33" fill-opacity="1" stroke="#bfbfbf" stroke-opacity="1" stroke-width="1" stroke-linecap="square" stroke-linejoin="bevel" transform="matrix(1,0,0,1,1336,550)" font-family="MS Shell Dlg 2" font-size="9.75" font-weight="400" font-style="normal" > <rect x="0" y="0" width="50" height="20"/> </g> <g fill="none" stroke="#000000" stroke-opacity="1" stroke-width="1" stroke-linecap="square" stroke-linejoin="bevel" transform="matrix(1,0,0,1,1344,548)" font-family="MS Shell Dlg 2" font-size="9.75" font-weight="400" font-style="normal" > <text fill="#000000" fill-opacity="1" stroke="none" xml:space="preserve" x="4" y="17" font-family="Arial" font-size="10pt" font-weight="700" font-style="italic" >cox3</text> </g> <g fill="#ff9999" fill-opacity="1" stroke="#bfbfbf" stroke-opacity="1" stroke-width="1" stroke-linecap="square" stroke-linejoin="bevel" transform="matrix(1,0,0,1,1387,550)" font-family="MS Shell Dlg 2" font-size="9.75" font-weight="400" font-style="normal" > <path vector-effect="none" fill-rule="evenodd" d="M0,1.7 L42.5,1.7 L42.5,0 L50,10 L42.5,20 L42.5,18.3 L0,18.3 L0,1.7"/> </g> <g fill="none" stroke="#000000" stroke-opacity="1" stroke-width="1" stroke-linecap="square" stroke-linejoin="bevel" transform="matrix(1,0,0,1,1400.13,548)" font-family="MS Shell Dlg 2" font-size="9.75" font-weight="400" font-style="normal" > <text fill="#000000" fill-opacity="1" stroke="none" xml:space="preserve" x="4" y="17" font-family="Arial" font-size="10pt" font-weight="700" font-style="italic" >S</text> </g> <g fill="#ff9999" fill-opacity="1" stroke="#bfbfbf" stroke-opacity="1" stroke-width="1" stroke-linecap="square" stroke-linejoin="bevel" transform="matrix(1,0,0,1,1438,550)" font-family="MS Shell Dlg 2" font-size="9.75" font-weight="400" font-style="normal" > <path vector-effect="none" fill-rule="evenodd" d="M0,1.7 L42.5,1.7 L42.5,0 L50,10 L42.5,20 L42.5,18.3 L0,18.3 L0,1.7"/> </g> <g fill="none" stroke="#000000" stroke-opacity="1" stroke-width="1" stroke-linecap="square" stroke-linejoin="bevel" transform="matrix(1,0,0,1,1450.13,548)" font-family="MS Shell Dlg 2" font-size="9.75" font-weight="400" font-style="normal" > <text fill="#000000" fill-opacity="1" stroke="none" xml:space="preserve" x="4" y="17" font-family="Arial" font-size="10pt" font-weight="700" font-style="italic" >M</text> </g> <g fill="#ffff33" fill-opacity="1" stroke="#bfbfbf" stroke-opacity="1" stroke-width="1" stroke-linecap="square" stroke-linejoin="bevel" transform="matrix(1,0,0,1,1489,550)" font-family="MS Shell Dlg 2" font-size="9.75" font-weight="400" font-style="normal" > <rect x="0" y="0" width="50" height="20"/> </g> <g fill="none" stroke="#000000" stroke-opacity="1" stroke-width="1" stroke-linecap="square" stroke-linejoin="bevel" transform="matrix(1,0,0,1,1496,548)" font-family="MS Shell Dlg 2" font-size="9.75" font-weight="400" font-style="normal" > <text fill="#000000" fill-opacity="1" stroke="none" xml:space="preserve" x="4" y="17" font-family="Arial" font-size="10pt" font-weight="700" font-style="italic" >nad2</text> </g> <g fill="#ff9999" fill-opacity="1" stroke="#bfbfbf" stroke-opacity="1" stroke-width="1" stroke-linecap="square" stroke-linejoin="bevel" transform="matrix(1,0,0,1,1540,550)" font-family="MS Shell Dlg 2" font-size="9.75" font-weight="400" font-style="normal" > <path vector-effect="none" fill-rule="evenodd" d="M0,1.7 L42.5,1.7 L42.5,0 L50,10 L42.5,20 L42.5,18.3 L0,18.3 L0,1.7"/> </g> <g fill="none" stroke="#000000" stroke-opacity="1" stroke-width="1" stroke-linecap="square" stroke-linejoin="bevel" transform="matrix(1,0,0,1,1553.13,548)" font-family="MS Shell Dlg 2" font-size="9.75" font-weight="400" font-style="normal" > <text fill="#000000" fill-opacity="1" stroke="none" xml:space="preserve" x="4" y="17" font-family="Arial" font-size="10pt" font-weight="700" font-style="italic" >R</text> </g> <g fill="#ff9999" fill-opacity="1" stroke="#bfbfbf" stroke-opacity="1" stroke-width="1" stroke-linecap="square" stroke-linejoin="bevel" transform="matrix(1,0,0,1,1591,550)" font-family="MS Shell Dlg 2" font-size="9.75" font-weight="400" font-style="normal" > <path vector-effect="none" fill-rule="evenodd" d="M0,1.7 L42.5,1.7 L42.5,0 L50,10 L42.5,20 L42.5,18.3 L0,18.3 L0,1.7"/> </g> <g fill="none" stroke="#000000" stroke-opacity="1" stroke-width="1" stroke-linecap="square" stroke-linejoin="bevel" transform="matrix(1,0,0,1,1602.13,548)" font-family="MS Shell Dlg 2" font-size="9.75" font-weight="400" font-style="normal" > <text fill="#000000" fill-opacity="1" stroke="none" xml:space="preserve" x="4" y="17" font-family="Arial" font-size="10pt" font-weight="700" font-style="italic" >W</text> </g> <g fill="#ff9999" fill-opacity="1" stroke="#bfbfbf" stroke-opacity="1" stroke-width="1" stroke-linecap="square" stroke-linejoin="bevel"

transform="matrix(1,0,0,1,1642,550)" font-family="MS Shell Dlg 2" font-size="9.75" font-weight="400" font-style="normal" > <path vector-effect="none" fill-rule="evenodd" d="M0,1.7 L42.5,1.7 L42.5,0 L50,10 L42.5,20 L42.5,18.3 L0,18.3 L0,1.7"/> </g> <g fill="none" stroke="#000000" stroke-opacity="1" stroke-width="1" stroke-linecap="square" stroke-linejoin="bevel" transform="matrix(1,0,0,1,1655.13,548)" font-family="MS Shell Dlg 2" font-size="9.75" font-weight="400" font-style="normal" > <text fill="#000000" fill-opacity="1" stroke="none" xml:space="preserve" x="4" y="17" font-family="Arial" font-size="10pt" font-weight="700" font-style="italic" >A</text> </g> <g fill="#ff9999" fill-opacity="1" stroke="#bfbfbf" stroke-opacity="1" stroke-width="1" stroke-linecap="square" stroke-linejoin="bevel" transform="matrix(1,0,0,1,1693,550)" font-family="MS Shell Dlg 2" font-size="9.75" font-weight="400" font-style="normal" > <path vector-effect="none" fill-rule="evenodd" d="M0,1.7 L42.5,1.7 L42.5,0 L50,10 L42.5,20 L42.5,18.3 L0,18.3 L0,1.7"/> </g> <g fill="none" stroke="#000000" stroke-opacity="1" stroke-width="1" stroke-linecap="square" stroke-linejoin="bevel" transform="matrix(1,0,0,1,1706.13,548)" font-family="MS Shell Dlg 2" font-size="9.75" font-weight="400" font-style="normal" > <text fill="#000000" fill-opacity="1" stroke="none" xml:space="preserve" x="4" y="17" font-family="Arial" font-size="10pt" font-weight="700" font-style="italic" >S</text> </g> <g fill="#ff9999" fill-opacity="1" stroke="#bfbfbf" stroke-opacity="1" stroke-width="1" stroke-linecap="square" stroke-linejoin="bevel" transform="matrix(1,0,0,1,1744,550)" font-family="MS Shell Dlg 2" font-size="9.75" font-weight="400" font-style="normal" > <path vector-effect="none" fill-rule="evenodd" d="M0,1.7 L42.5,1.7 L42.5,0 L50,10 L42.5,20 L42.5,18.3 L0,18.3 L0,1.7"/> </g> <g fill="none" stroke="#000000" stroke-opacity="1" stroke-width="1" stroke-linecap="square" stroke-linejoin="bevel" transform="matrix(1,0,0,1,1757.13,548)" font-family="MS Shell Dlg 2" font-size="9.75" font-weight="400" font-style="normal" > <text fill="#000000" fill-opacity="1" stroke="none" xml:space="preserve" x="4" y="17" font-family="Arial" font-size="10pt" font-weight="700" font-style="italic" >H</text> </g> <g fill="#ff9999" fill-opacity="1" stroke="#bfbfbf" stroke-opacity="1" stroke-width="1" stroke-linecap="square" stroke-linejoin="bevel" transform="matrix(1,0,0,1,1795,550)" font-family="MS Shell Dlg 2" font-size="9.75" font-weight="400" font-style="normal" > <path vector-effect="none" fill-rule="evenodd" d="M0,1.7 L42.5,1.7 L42.5,0 L50,10 L42.5,20 L42.5,18.3 L0,18.3 L0,1.7"/> </g> <g fill="none" stroke="#000000" stroke-opacity="1" stroke-width="1" stroke-linecap="square" stroke-linejoin="bevel" transform="matrix(1,0,0,1,1808.63,548)" font-family="MS Shell Dlg 2" font-size="9.75" font-weight="400" font-style="normal" > <text fill="#000000" fill-opacity="1" stroke="none" xml:space="preserve" x="4" y="17" font-family="Arial" font-size="10pt" font-weight="700" font-style="italic" >P</text> </g> <g fill="#ffff33" fill-opacity="1" stroke="#bfbfbf" stroke-opacity="1" stroke-width="1" stroke-linecap="square" stroke-linejoin="bevel" transform="matrix(1,0,0,1,1846,550)" font-family="MS Shell Dlg 2" font-size="9.75" font-weight="400" font-style="normal" > <rect x="0" y="0" width="50" height="20"/> </g> <g fill="none" stroke="#000000" stroke-opacity="1" stroke-width="1" stroke-linecap="square" stroke-linejoin="bevel" transform="matrix(1,0,0,1,1853,548)" font-family="MS Shell Dlg 2" font-size="9.75" font-weight="400" font-style="normal" > <text fill="#000000" fill-opacity="1" stroke="none" xml:space="preserve" x="4" y="17" font-family="Arial" font-size="10pt" font-weight="700" font-style="italic" >nad3</text> </g> <g fill="#ffff33" fill-opacity="1" stroke="#bfbfbf" stroke-opacity="1" stroke-width="1" stroke-linecap="square" stroke-linejoin="bevel" transform="matrix(1,0,0,1,1897,550)" font-family="MS Shell Dlg 2" font-size="9.75" font-weight="400" font-style="normal" > <rect x="0" y="0" width="50" height="20"/> </g> <g fill="none" stroke="#000000" stroke-opacity="1" stroke-width="1" stroke-linecap="square" stroke-linejoin="bevel" transform="matrix(1,0,0,1,1905.5,548)" font-family="MS Shell Dlg 2" font-size="9.75" font-weight="400" font-style="normal" > <text fill="#000000" fill-opacity="1" stroke="none" xml:space="preserve" x="4" y="17" font-family="Arial" font-size="10pt" font-weight="700" font-style="italic" >atp8</text> </g> <g fill="none" stroke="#000000" stroke-opacity="1" stroke-width="1" stroke-linecap="square" stroke-linejoin="bevel" transform="matrix(1,0,0,1,1948,550)" font-family="MS Shell Dlg 2" font-size="9.75" font-weight="400" font-style="normal" > <text fill="#000000" fill-opacity="1" stroke="none" xml:space="preserve" x="4" y="17" font-family="Arial" font-size="10pt" font-weight="700" font-style="italic" >(+)</text> </g> <g fill="none" stroke="#000000" stroke-opacity="1" stroke-width="1" stroke-linecap="square" stroke-linejoin="bevel" transform="matrix(1,0,0,1,10,590)" font-family="MS Shell Dlg 2" font-size="9.75" font-weight="400" font-style="normal" > <text fill="#000000" fill-opacity="1" stroke="none" xml:space="preserve" x="4" y="17" font-family="Arial" font-size="10pt" font-weight="700" font-style="italic" >Mytilus_coruscus_KJ577549</text> </g> <g fill="#ffff33" fill-opacity="1" stroke="#bfbfbf" stroke-opacity="1" stroke-width="1" stroke-linecap="square" stroke-linejoin="bevel" transform="matrix(1,0,0,1,10,620)" font-family="MS Shell Dlg 2" font-size="9.75" font-weight="400" font-style="normal" > <rect x="0" y="0" width="50" height="20"/> </g> <g fill="none" stroke="#000000" stroke-opacity="1" stroke-width="1" stroke-linecap="square" stroke-linejoin="bevel" transform="matrix(1,0,0,1,18,618)" font-family="MS Shell Dlg 2" font-size="9.75" font-weight="400" font-style="normal" > <text fill="#000000" fill-opacity="1" stroke="none" xml:space="preserve" x="4" y="17" font-family="Arial" font-size="10pt" font-weight="700" font-style="italic"

>cox1</text> </g> <g fill="#ffff33" fill-opacity="1" stroke="#bfbfbf" stroke-opacity="1" stroke-width="1" stroke-linecap="square" stroke-linejoin="bevel" transform="matrix(1,0,0,1,61,620)" font-family="MS Shell Dlg 2" font-size="9.75" font-weight="400" font-style="normal" > <rect x="0" y="0" width="50" height="20"/> </g> <g fill="none" stroke="#000000" stroke-opacity="1" stroke-width="1" stroke-linecap="square" stroke-linejoin="bevel" transform="matrix(1,0,0,1,69.5,618)" font-family="MS Shell Dlg 2" font-size="9.75" font-weight="400" font-style="normal" > <text fill="#000000" fill-opacity="1" stroke="none" xml:space="preserve" x="4" y="17" font-family="Arial" font-size="10pt" font-weight="700" font-style="italic" >atp6</text> </g> <g fill="#ff9999" fill-opacity="1" stroke="#bfbfbf" stroke-opacity="1" stroke-width="1" stroke-linecap="square" stroke-linejoin="bevel" transform="matrix(1,0,0,1,112,620)" font-family="MS Shell Dlg 2" font-size="9.75" font-weight="400" font-style="normal" > <path vector-effect="none" fill-rule="evenodd" d="M0,1.7 L42.5,1.7 L42.5,0 L50,10 L42.5,20 L42.5,18.3 L0,18.3 L0,1.7"/> </g> <g fill="none" stroke="#000000" stroke-opacity="1" stroke-width="1" stroke-linecap="square" stroke-linejoin="bevel" transform="matrix(1,0,0,1,125.132,618)" font-family="MS Shell Dlg 2" font-size="9.75" font-weight="400" font-style="normal" > <text fill="#000000" fill-opacity="1" stroke="none" xml:space="preserve" x="4" y="17" font-family="Arial" font-size="10pt" font-weight="700" font-style="italic" >T</text> </g> <g fill="#ffff33" fill-opacity="1" stroke="#bfbfbf" stroke-opacity="1" stroke-width="1" stroke-linecap="square" stroke-linejoin="bevel" transform="matrix(1,0,0,1,163,620)" font-family="MS Shell Dlg 2" font-size="9.75" font-weight="400" font-style="normal" > <rect x="0" y="0" width="50" height="20"/> </g> <g fill="none" stroke="#000000" stroke-opacity="1" stroke-width="1" stroke-linecap="square" stroke-linejoin="bevel" transform="matrix(1,0,0,1,167,618)" font-family="MS Shell Dlg 2" font-size="9.75" font-weight="400" font-style="normal" > <text fill="#000000" fill-opacity="1" stroke="none" xml:space="preserve" x="4" y="17" font-family="Arial" font-size="10pt" font-weight="700" font-style="italic" >nad4L</text> </g> <g fill="#ffff33" fill-opacity="1" stroke="#bfbfbf" stroke-opacity="1" stroke-width="1" stroke-linecap="square" stroke-linejoin="bevel" transform="matrix(1,0,0,1,214,620)" font-family="MS Shell Dlg 2" font-size="9.75" font-weight="400" font-style="normal" > <rect x="0" y="0" width="50" height="20"/> </g> <g fill="none" stroke="#000000" stroke-opacity="1" stroke-width="1" stroke-linecap="square" stroke-linejoin="bevel" transform="matrix(1,0,0,1,221,618)" font-family="MS Shell Dlg 2" font-size="9.75" font-weight="400" font-style="normal" > <text fill="#000000" fill-opacity="1" stroke="none" xml:space="preserve" x="4" y="17" font-family="Arial" font-size="10pt" font-weight="700" font-style="italic" >nad5</text> </g> <g fill="#ffff33" fill-opacity="1" stroke="#bfbfbf" stroke-opacity="1" stroke-width="1" stroke-linecap="square" stroke-linejoin="bevel" transform="matrix(1,0,0,1,265,620)" font-family="MS Shell Dlg 2" font-size="9.75" font-weight="400" font-style="normal" > <rect x="0" y="0" width="50" height="20"/> </g> <g fill="none" stroke="#000000" stroke-opacity="1" stroke-width="1" stroke-linecap="square" stroke-linejoin="bevel" transform="matrix(1,0,0,1,272,618)" font-family="MS Shell Dlg 2" font-size="9.75" font-weight="400" font-style="normal" > <text fill="#000000" fill-opacity="1" stroke="none" xml:space="preserve" x="4" y="17" font-family="Arial" font-size="10pt" font-weight="700" font-style="italic" >nad6</text> </g> <g fill="#ff9999" fill-opacity="1" stroke="#bfbfbf" stroke-opacity="1" stroke-width="1" stroke-linecap="square" stroke-linejoin="bevel" transform="matrix(1,0,0,1,316,620)" font-family="MS Shell Dlg 2" font-size="9.75" font-weight="400" font-style="normal" > <path vector-effect="none" fill-rule="evenodd" d="M0,1.7 L42.5,1.7 L42.5,0 L50,10 L42.5,20 L42.5,18.3 L0,18.3 L0,1.7"/> </g> <g fill="none" stroke="#000000" stroke-opacity="1" stroke-width="1" stroke-linecap="square" stroke-linejoin="bevel" transform="matrix(1,0,0,1,329.632,618)" font-family="MS Shell Dlg 2" font-size="9.75" font-weight="400" font-style="normal" > <text fill="#000000" fill-opacity="1" stroke="none" xml:space="preserve" x="4" y="17" font-family="Arial" font-size="10pt" font-weight="700" font-style="italic" >F</text> </g> <g fill="#99ffff" fill-opacity="1" stroke="#bfbfbf" stroke-opacity="1" stroke-width="1" stroke-linecap="square" stroke-linejoin="bevel" transform="matrix(1,0,0,1,367,620)" font-family="MS Shell Dlg 2" font-size="9.75" font-weight="400" font-style="normal" > <path vector-effect="none" fill-rule="evenodd" d="M0,5 C0,2.23858 2.23858,0 5,0 L45,0 C47.7614,0 50,2.23858 50,5 L50,15 C50,17.7614 47.7614,20 45,20 L5,20 C2.23858,20 0,17.7614 0,15 L0,5"/> </g> <g fill="none" stroke="#000000" stroke-opacity="1" stroke-width="1" stroke-linecap="square" stroke-linejoin="bevel" transform="matrix(1,0,0,1,375.5,618)" font-family="MS Shell Dlg 2" font-size="9.75" font-weight="400" font-style="normal" > <text fill="#000000" fill-opacity="1" stroke="none" xml:space="preserve" x="4" y="17" font-family="Arial" font-size="10pt" font-weight="700" font-style="italic" >rrnS</text> </g> <g fill="#ff9999" fill-opacity="1" stroke="#bfbfbf" stroke-opacity="1" stroke-width="1" stroke-linecap="square" stroke-linejoin="bevel" transform="matrix(1,0,0,1,418,620)" font-family="MS Shell Dlg 2" font-size="9.75" font-weight="400" font-style="normal" > <path vector-effect="none" fill-rule="evenodd" d="M0,1.7 L42.5,1.7 L42.5,0 L50,10 L42.5,20 L42.5,18.3 L0,18.3 L0,1.7"/> </g> <g fill="none" stroke="#000000" stroke-opacity="1" stroke-width="1" stroke-linecap="square" stroke-linejoin="bevel" transform="matrix(1,0,0,1,431.132,618)" font-family="MS Shell Dlg 2" font-size="9.75" font-weight="400" font-style="normal" > <text fill="#000000" fill-

opacity="1" stroke="none" xml:space="preserve" x="4" y="17" font-family="Arial" font-size="10pt" font-weight="700" font-style="italic" >G</text> </g> <g fill="#ff9999" fill-opacity="1" stroke="#bfbfbf" stroke-opacity="1" stroke-width="1" stroke-linecap="square" stroke-linejoin="bevel" transform="matrix(1,0,0,1,469,620)" font-family="MS Shell Dlg 2" font-size="9.75" font-weight="400" font-style="normal" > <path vector-effect="none" fill-rule="evenodd" d="M0,1.7 L42.5,1.7 L42.5,0 L50,10 L42.5,20 L42.5,18.3 L0,18.3 L0,1.7"/> </g> <g fill="none" stroke="#000000" stroke-opacity="1" stroke-width="1" stroke-linecap="square" stroke-linejoin="bevel" transform="matrix(1,0,0,1,482.132,618)" font-family="MS Shell Dlg 2" font-size="9.75" font-weight="400" font-style="normal" > <text fill="#000000" fill-opacity="1" stroke="none" xml:space="preserve" x="4" y="17" font-family="Arial" font-size="10pt" font-weight="700" font-style="italic" >N</text> </g> <g fill="#ff9999" fill-opacity="1" stroke="#bfbfbf" stroke-opacity="1" stroke-width="1" stroke-linecap="square" stroke-linejoin="bevel" transform="matrix(1,0,0,1,520,620)" font-family="MS Shell Dlg 2" font-size="9.75" font-weight="400" font-style="normal" > <path vector-effect="none" fill-rule="evenodd" d="M0,1.7 L42.5,1.7 L42.5,0 L50,10 L42.5,20 L42.5,18.3 L0,18.3 L0,1.7"/> </g> <g fill="none" stroke="#000000" stroke-opacity="1" stroke-width="1" stroke-linecap="square" stroke-linejoin="bevel" transform="matrix(1,0,0,1,533.633,618)" font-family="MS Shell Dlg 2" font-size="9.75" font-weight="400" font-style="normal" > <text fill="#000000" fill-opacity="1" stroke="none" xml:space="preserve" x="4" y="17" font-family="Arial" font-size="10pt" font-weight="700" font-style="italic" >E</text> </g> <g fill="#ff9999" fill-opacity="1" stroke="#bfbfbf" stroke-opacity="1" stroke-width="1" stroke-linecap="square" stroke-linejoin="bevel" transform="matrix(1,0,0,1,571,620)" font-family="MS Shell Dlg 2" font-size="9.75" font-weight="400" font-style="normal" > <path vector-effect="none" fill-rule="evenodd" d="M0,1.7 L42.5,1.7 L42.5,0 L50,10 L42.5,20 L42.5,18.3 L0,18.3 L0,1.7"/> </g> <g fill="none" stroke="#000000" stroke-opacity="1" stroke-width="1" stroke-linecap="square" stroke-linejoin="bevel" transform="matrix(1,0,0,1,584.133,618)" font-family="MS Shell Dlg 2" font-size="9.75" font-weight="400" font-style="normal" > <text fill="#000000" fill-opacity="1" stroke="none" xml:space="preserve" x="4" y="17" font-family="Arial" font-size="10pt" font-weight="700" font-style="italic" >C</text> </g> <g fill="#ff9999" fill-opacity="1" stroke="#bfbfbf" stroke-opacity="1" stroke-width="1" stroke-linecap="square" stroke-linejoin="bevel" transform="matrix(1,0,0,1,622,620)" font-family="MS Shell Dlg 2" font-size="9.75" font-weight="400" font-style="normal" > <path vector-effect="none" fill-rule="evenodd" d="M0,1.7 L42.5,1.7 L42.5,0 L50,10 L42.5,20 L42.5,18.3 L0,18.3 L0,1.7"/> </g> <g fill="none" stroke="#000000" stroke-opacity="1" stroke-width="1" stroke-linecap="square" stroke-linejoin="bevel" transform="matrix(1,0,0,1,637.133,618)" font-family="MS Shell Dlg 2" font-size="9.75" font-weight="400" font-style="normal" > <text fill="#000000" fill-opacity="1" stroke="none" xml:space="preserve" x="4" y="17" font-family="Arial" font-size="10pt" font-weight="700" font-style="italic" >I</text> </g> <g fill="#ff9999" fill-opacity="1" stroke="#bfbfbf" stroke-opacity="1" stroke-width="1" stroke-linecap="square" stroke-linejoin="bevel" transform="matrix(1,0,0,1,673,620)" font-family="MS Shell Dlg 2" font-size="9.75" font-weight="400" font-style="normal" > <path vector-effect="none" fill-rule="evenodd" d="M0,1.7 L42.5,1.7 L42.5,0 L50,10 L42.5,20 L42.5,18.3 L0,18.3 L0,1.7"/> </g> <g fill="none" stroke="#000000" stroke-opacity="1" stroke-width="1" stroke-linecap="square" stroke-linejoin="bevel" transform="matrix(1,0,0,1,685.633,618)" font-family="MS Shell Dlg 2" font-size="9.75" font-weight="400" font-style="normal" > <text fill="#000000" fill-opacity="1" stroke="none" xml:space="preserve" x="4" y="17" font-family="Arial" font-size="10pt" font-weight="700" font-style="italic" >Q</text> </g> <g fill="#ff9999" fill-opacity="1" stroke="#bfbfbf" stroke-opacity="1" stroke-width="1" stroke-linecap="square" stroke-linejoin="bevel" transform="matrix(1,0,0,1,724,620)" font-family="MS Shell Dlg 2" font-size="9.75" font-weight="400" font-style="normal" > <path vector-effect="none" fill-rule="evenodd" d="M0,1.7 L42.5,1.7 L42.5,0 L50,10 L42.5,20 L42.5,18.3 L0,18.3 L0,1.7"/> </g> <g fill="none" stroke="#000000" stroke-opacity="1" stroke-width="1" stroke-linecap="square" stroke-linejoin="bevel" transform="matrix(1,0,0,1,737.133,618)" font-family="MS Shell Dlg 2" font-size="9.75" font-weight="400" font-style="normal" > <text fill="#000000" fill-opacity="1" stroke="none" xml:space="preserve" x="4" y="17" font-family="Arial" font-size="10pt" font-weight="700" font-style="italic" >D</text> </g> <g fill="#99ffff" fill-opacity="1" stroke="#bfbfbf" stroke-opacity="1" stroke-width="1" stroke-linecap="square" stroke-linejoin="bevel" transform="matrix(1,0,0,1,775,620)" font-family="MS Shell Dlg 2" font-size="9.75" font-weight="400" font-style="normal" > <path vector-effect="none" fill-rule="evenodd" d="M0,5 C0,2.23858 2.23858,0 5,0 L45,0 C47.7614,0 50,2.23858 50,5 L50,15 C50,17.7614 47.7614,20 45,20 L5,20 C2.23858,20 0,17.7614 0,15 L0,5"/> </g> <g fill="none" stroke="#000000" stroke-opacity="1" stroke-width="1" stroke-linecap="square" stroke-linejoin="bevel" transform="matrix(1,0,0,1,784.5,618)" font-family="MS Shell Dlg 2" font-size="9.75" font-weight="400" font-style="normal" > <text fill="#000000" fill-opacity="1" stroke="none" xml:space="preserve" x="4" y="17" font-family="Arial" font-size="10pt" font-weight="700" font-style="italic" >rrnL</text> </g> <g fill="#ff9999" fill-opacity="1" stroke="#bfbfbf" stroke-opacity="1" stroke-width="1" stroke-linecap="square" stroke-linejoin="bevel" transform="matrix(1,0,0,1,826,620)" font-family="MS Shell Dlg 2" font-size="9.75" font-

weight="400" font-style="normal" > <path vector-effect="none" fill-rule="evenodd" d="M0,1.7 L42.5,1.7 L42.5,0 L50,10 L42.5,20 L42.5,18.3 L0,18.3 L0,1.7"/> </g> <g fill="none" stroke="#000000" stroke-opacity="1" stroke-width="1" stroke-linecap="square" stroke-linejoin="bevel" transform="matrix(1,0,0,1,839.633,618)" font-family="MS Shell Dlg 2" font-size="9.75" font-weight="400" font-style="normal" > <text fill="#000000" fill-opacity="1" stroke="none" xml:space="preserve" x="4" y="17" font-family="Arial" font-size="10pt" font-weight="700" font-style="italic" >Y</text> </g> <g fill="#ffff33" fill-opacity="1" stroke="#bfbfbf" stroke-opacity="1" stroke-width="1" stroke-linecap="square" stroke-linejoin="bevel" transform="matrix(1,0,0,1,877,620)" font-family="MS Shell Dlg 2" font-size="9.75" font-weight="400" font-style="normal" > <rect x="0" y="0" width="50" height="20"/> </g> <g fill="none" stroke="#000000" stroke-opacity="1" stroke-width="1" stroke-linecap="square" stroke-linejoin="bevel" transform="matrix(1,0,0,1,886.5,618)" font-family="MS Shell Dlg 2" font-size="9.75" font-weight="400" font-style="normal" > <text fill="#000000" fill-opacity="1" stroke="none" xml:space="preserve" x="4" y="17" font-family="Arial" font-size="10pt" font-weight="700" font-style="italic" >cytb</text> </g> <g fill="#ffff33" fill-opacity="1" stroke="#bfbfbf" stroke-opacity="1" stroke-width="1" stroke-linecap="square" stroke-linejoin="bevel" transform="matrix(1,0,0,1,928,620)" font-family="MS Shell Dlg 2" font-size="9.75" font-weight="400" font-style="normal" > <rect x="0" y="0" width="50" height="20"/> </g> <g fill="none" stroke="#000000" stroke-opacity="1" stroke-width="1" stroke-linecap="square" stroke-linejoin="bevel" transform="matrix(1,0,0,1,936,618)" font-family="MS Shell Dlg 2" font-size="9.75" font-weight="400" font-style="normal" > <text fill="#000000" fill-opacity="1" stroke="none" xml:space="preserve" x="4" y="17" font-family="Arial" font-size="10pt" font-weight="700" font-style="italic" >cox2</text> </g> <g fill="#ff9999" fill-opacity="1" stroke="#bfbfbf" stroke-opacity="1" stroke-width="1" stroke-linecap="square" stroke-linejoin="bevel" transform="matrix(1,0,0,1,979,620)" font-family="MS Shell Dlg 2" font-size="9.75" font-weight="400" font-style="normal" > <path vector-effect="none" fill-rule="evenodd" d="M0,1.7 L42.5,1.7 L42.5,0 L50,10 L42.5,20 L42.5,18.3 L0,18.3 L0,1.7"/> </g> <g fill="none" stroke="#000000" stroke-opacity="1" stroke-width="1" stroke-linecap="square" stroke-linejoin="bevel" transform="matrix(1,0,0,1,992.633,618)" font-family="MS Shell Dlg 2" font-size="9.75" font-weight="400" font-style="normal" > <text fill="#000000" fill-opacity="1" stroke="none" xml:space="preserve" x="4" y="17" font-family="Arial" font-size="10pt" font-weight="700" font-style="italic" >K</text> </g> <g fill="#ff9999" fill-opacity="1" stroke="#bfbfbf" stroke-opacity="1" stroke-width="1" stroke-linecap="square" stroke-linejoin="bevel" transform="matrix(1,0,0,1,1030,620)" font-family="MS Shell Dlg 2" font-size="9.75" font-weight="400" font-style="normal" > <path vector-effect="none" fill-rule="evenodd" d="M0,1.7 L42.5,1.7 L42.5,0 L50,10 L42.5,20 L42.5,18.3 L0,18.3 L0,1.7"/> </g> <g fill="none" stroke="#000000" stroke-opacity="1" stroke-width="1" stroke-linecap="square" stroke-linejoin="bevel" transform="matrix(1,0,0,1,1042.13,618)" font-family="MS Shell Dlg 2" font-size="9.75" font-weight="400" font-style="normal" > <text fill="#000000" fill-opacity="1" stroke="none" xml:space="preserve" x="4" y="17" font-family="Arial" font-size="10pt" font-weight="700" font-style="italic" >M</text> </g> <g fill="#ff9999" fill-opacity="1" stroke="#bfbfbf" stroke-opacity="1" stroke-width="1" stroke-linecap="square" stroke-linejoin="bevel" transform="matrix(1,0,0,1,1081,620)" font-family="MS Shell Dlg 2" font-size="9.75" font-weight="400" font-style="normal" > <path vector-effect="none" fill-rule="evenodd" d="M0,1.7 L42.5,1.7 L42.5,0 L50,10 L42.5,20 L42.5,18.3 L0,18.3 L0,1.7"/> </g> <g fill="none" stroke="#000000" stroke-opacity="1" stroke-width="1" stroke-linecap="square" stroke-linejoin="bevel" transform="matrix(1,0,0,1,1091.63,618)" font-family="MS Shell Dlg 2" font-size="9.75" font-weight="400" font-style="normal" > <text fill="#000000" fill-opacity="1" stroke="none" xml:space="preserve" x="4" y="17" font-family="Arial" font-size="10pt" font-weight="700" font-style="italic" >L1</text> </g> <g fill="#ff9999" fill-opacity="1" stroke="#bfbfbf" stroke-opacity="1" stroke-width="1" stroke-linecap="square" stroke-linejoin="bevel" transform="matrix(1,0,0,1,1132,620)" font-family="MS Shell Dlg 2" font-size="9.75" font-weight="400" font-style="normal" > <path vector-effect="none" fill-rule="evenodd" d="M0,1.7 L42.5,1.7 L42.5,0 L50,10 L42.5,20 L42.5,18.3 L0,18.3 L0,1.7"/> </g> <g fill="none" stroke="#000000" stroke-opacity="1" stroke-width="1" stroke-linecap="square" stroke-linejoin="bevel" transform="matrix(1,0,0,1,1142.63,618)" font-family="MS Shell Dlg 2" font-size="9.75" font-weight="400" font-style="normal" > <text fill="#000000" fill-opacity="1" stroke="none" xml:space="preserve" x="4" y="17" font-family="Arial" font-size="10pt" font-weight="700" font-style="italic" >L2</text> </g> <g fill="#ffff33" fill-opacity="1" stroke="#bfbfbf" stroke-opacity="1" stroke-width="1" stroke-linecap="square" stroke-linejoin="bevel" transform="matrix(1,0,0,1,1183,620)" font-family="MS Shell Dlg 2" font-size="9.75" font-weight="400" font-style="normal" > <rect x="0" y="0" width="50" height="20"/> </g> <g fill="none" stroke="#000000" stroke-opacity="1" stroke-width="1" stroke-linecap="square" stroke-linejoin="bevel" transform="matrix(1,0,0,1,1190,618)" font-family="MS Shell Dlg 2" font-size="9.75" font-weight="400" font-style="normal" > <text fill="#000000" fill-opacity="1" stroke="none" xml:space="preserve" x="4" y="17" font-family="Arial" font-size="10pt" font-weight="700" font-style="italic" >nad1</text> </g> <g

fill="#ff9999" fill-opacity="1" stroke="#bfbfbf" stroke-opacity="1" stroke-width="1" stroke-linecap="square"
stroke-linejoin="bevel" transform="matrix(1,0,0,1,1234,620)" font-family="MS Shell Dlg 2" font-size="9.75" font-
weight="400" font-style="normal" > <path vector-effect="none" fill-rule="evenodd" d="M0,1.7 L42.5,1.7 L42.5,0
L50,10 L42.5,20 L42.5,18.3 L0,18.3 L0,1.7"/> </g> <g fill="none" stroke="#000000" stroke-opacity="1"
stroke-width="1" stroke-linecap="square" stroke-linejoin="bevel" transform="matrix(1,0,0,1,1247.13,618)" font-
family="MS Shell Dlg 2" font-size="9.75" font-weight="400" font-style="normal" > <text fill="#000000" fill-
opacity="1" stroke="none" xml:space="preserve" x="4" y="17" font-family="Arial" font-size="10pt" font-
weight="700" font-style="italic" >V</text> </g> <g fill="#ffff33" fill-opacity="1" stroke="#bfbfbf"
stroke-opacity="1" stroke-width="1" stroke-linecap="square" stroke-linejoin="bevel"
transform="matrix(1,0,0,1,1285,620)" font-family="MS Shell Dlg 2" font-size="9.75" font-weight="400" font-
style="normal" > <rect x="0" y="0" width="50" height="20"/> </g> <g fill="none" stroke="#000000" stroke-
opacity="1" stroke-width="1" stroke-linecap="square" stroke-linejoin="bevel"
transform="matrix(1,0,0,1,1292,618)" font-family="MS Shell Dlg 2" font-size="9.75" font-weight="400" font-
style="normal" > <text fill="#000000" fill-opacity="1" stroke="none" xml:space="preserve" x="4" y="17" font-
family="Arial" font-size="10pt" font-weight="700" font-style="italic" >nad4</text> </g> <g fill="#ffff33"
fill-opacity="1" stroke="#bfbfbf" stroke-opacity="1" stroke-width="1" stroke-linecap="square" stroke-
linejoin="bevel" transform="matrix(1,0,0,1,1336,620)" font-family="MS Shell Dlg 2" font-size="9.75" font-
weight="400" font-style="normal" > <rect x="0" y="0" width="50" height="20"/> </g> <g fill="none"
stroke="#000000" stroke-opacity="1" stroke-width="1" stroke-linecap="square" stroke-linejoin="bevel"
transform="matrix(1,0,0,1,1344,618)" font-family="MS Shell Dlg 2" font-size="9.75" font-weight="400" font-
style="normal" > <text fill="#000000" fill-opacity="1" stroke="none" xml:space="preserve" x="4" y="17" font-
family="Arial" font-size="10pt" font-weight="700" font-style="italic" >cox3</text> </g> <g
fill="#ff9999" fill-opacity="1" stroke="#bfbfbf" stroke-opacity="1" stroke-width="1" stroke-linecap="square"
stroke-linejoin="bevel" transform="matrix(1,0,0,1,1387,620)" font-family="MS Shell Dlg 2" font-size="9.75" font-
weight="400" font-style="normal" > <path vector-effect="none" fill-rule="evenodd" d="M0,1.7 L42.5,1.7 L42.5,0
L50,10 L42.5,20 L42.5,18.3 L0,18.3 L0,1.7"/> </g> <g fill="none" stroke="#000000" stroke-opacity="1"
stroke-width="1" stroke-linecap="square" stroke-linejoin="bevel" transform="matrix(1,0,0,1,1396.63,618)" font-
family="MS Shell Dlg 2" font-size="9.75" font-weight="400" font-style="normal" > <text fill="#000000" fill-
opacity="1" stroke="none" xml:space="preserve" x="4" y="17" font-family="Arial" font-size="10pt" font-
weight="700" font-style="italic" >S2</text> </g> <g fill="#ff9999" fill-opacity="1" stroke="#bfbfbf"
stroke-opacity="1" stroke-width="1" stroke-linecap="square" stroke-linejoin="bevel"
transform="matrix(1,0,0,1,1438,620)" font-family="MS Shell Dlg 2" font-size="9.75" font-weight="400" font-
style="normal" > <path vector-effect="none" fill-rule="evenodd" d="M0,1.7 L42.5,1.7 L42.5,0 L50,10 L42.5,20
L42.5,18.3 L0,18.3 L0,1.7"/> </g> <g fill="none" stroke="#000000" stroke-opacity="1" stroke-width="1"
stroke-linecap="square" stroke-linejoin="bevel" transform="matrix(1,0,0,1,1450.13,618)" font-family="MS Shell
Dlg 2" font-size="9.75" font-weight="400" font-style="normal" > <text fill="#000000" fill-opacity="1"
stroke="none" xml:space="preserve" x="4" y="17" font-family="Arial" font-size="10pt" font-weight="700" font-
style="italic" >M</text> </g> <g fill="#ffff33" fill-opacity="1" stroke="#bfbfbf" stroke-opacity="1"
stroke-width="1" stroke-linecap="square" stroke-linejoin="bevel" transform="matrix(1,0,0,1,1489,620)" font-
family="MS Shell Dlg 2" font-size="9.75" font-weight="400" font-style="normal" > <rect x="0" y="0" width="50"
height="20"/> </g> <g fill="none" stroke="#000000" stroke-opacity="1" stroke-width="1" stroke-
linecap="square" stroke-linejoin="bevel" transform="matrix(1,0,0,1,1496,618)" font-family="MS Shell Dlg 2" font-
size="9.75" font-weight="400" font-style="normal" > <text fill="#000000" fill-opacity="1" stroke="none"
xml:space="preserve" x="4" y="17" font-family="Arial" font-size="10pt" font-weight="700" font-style="italic"
>nad2</text> </g> <g fill="#ff9999" fill-opacity="1" stroke="#bfbfbf" stroke-opacity="1" stroke-width="1"
stroke-linecap="square" stroke-linejoin="bevel" transform="matrix(1,0,0,1,1540,620)" font-family="MS Shell Dlg
2" font-size="9.75" font-weight="400" font-style="normal" > <path vector-effect="none" fill-rule="evenodd"
d="M0,1.7 L42.5,1.7 L42.5,0 L50,10 L42.5,20 L42.5,18.3 L0,18.3 L0,1.7"/> </g> <g fill="none"
stroke="#000000" stroke-opacity="1" stroke-width="1" stroke-linecap="square" stroke-linejoin="bevel"
transform="matrix(1,0,0,1,1553.13,618)" font-family="MS Shell Dlg 2" font-size="9.75" font-weight="400" font-
style="normal" > <text fill="#000000" fill-opacity="1" stroke="none" xml:space="preserve" x="4" y="17" font-
family="Arial" font-size="10pt" font-weight="700" font-style="italic" >R</text> </g> <g fill="#ff9999"
fill-opacity="1" stroke="#bfbfbf" stroke-opacity="1" stroke-width="1" stroke-linecap="square" stroke-
linejoin="bevel" transform="matrix(1,0,0,1,1591,620)" font-family="MS Shell Dlg 2" font-size="9.75" font-
weight="400" font-style="normal" > <path vector-effect="none" fill-rule="evenodd" d="M0,1.7 L42.5,1.7 L42.5,0
L50,10 L42.5,20 L42.5,18.3 L0,18.3 L0,1.7"/> </g> <g fill="none" stroke="#000000" stroke-opacity="1"
stroke-width="1" stroke-linecap="square" stroke-linejoin="bevel" transform="matrix(1,0,0,1,1602.13,618)" font-
family="MS Shell Dlg 2" font-size="9.75" font-weight="400" font-style="normal" > <text fill="#000000" fill-

opacity="1" stroke="none" xml:space="preserve" x="4" y="17" font-family="Arial" font-size="10pt" font-weight="700" font-style="italic" >W</text> </g> <g fill="#ff9999" fill-opacity="1" stroke="#bfbfbf" stroke-opacity="1" stroke-width="1" stroke-linecap="square" stroke-linejoin="bevel" transform="matrix(1,0,0,1,1642,620)" font-family="MS Shell Dlg 2" font-size="9.75" font-weight="400" font-style="normal" > <path vector-effect="none" fill-rule="evenodd" d="M0,1.7 L42.5,1.7 L42.5,0 L50,10 L42.5,20 L42.5,18.3 L0,18.3 L0,1.7"/> </g> <g fill="none" stroke="#000000" stroke-opacity="1" stroke-width="1" stroke-linecap="square" stroke-linejoin="bevel" transform="matrix(1,0,0,1,1655.13,618)" font-family="MS Shell Dlg 2" font-size="9.75" font-weight="400" font-style="normal" > <text fill="#000000" fill-opacity="1" stroke="none" xml:space="preserve" x="4" y="17" font-family="Arial" font-size="10pt" font-weight="700" font-style="italic" >A</text> </g> <g fill="#ff9999" fill-opacity="1" stroke="#bfbfbf" stroke-opacity="1" stroke-width="1" stroke-linecap="square" stroke-linejoin="bevel" transform="matrix(1,0,0,1,1693,620)" font-family="MS Shell Dlg 2" font-size="9.75" font-weight="400" font-style="normal" > <path vector-effect="none" fill-rule="evenodd" d="M0,1.7 L42.5,1.7 L42.5,0 L50,10 L42.5,20 L42.5,18.3 L0,18.3 L0,1.7"/> </g> <g fill="none" stroke="#000000" stroke-opacity="1" stroke-width="1" stroke-linecap="square" stroke-linejoin="bevel" transform="matrix(1,0,0,1,1702.63,618)" font-family="MS Shell Dlg 2" font-size="9.75" font-weight="400" font-style="normal" > <text fill="#000000" fill-opacity="1" stroke="none" xml:space="preserve" x="4" y="17" font-family="Arial" font-size="10pt" font-weight="700" font-style="italic" >S1</text> </g> <g fill="#ff9999" fill-opacity="1" stroke="#bfbfbf" stroke-opacity="1" stroke-width="1" stroke-linecap="square" stroke-linejoin="bevel" transform="matrix(1,0,0,1,1744,620)" font-family="MS Shell Dlg 2" font-size="9.75" font-weight="400" font-style="normal" > <path vector-effect="none" fill-rule="evenodd" d="M0,1.7 L42.5,1.7 L42.5,0 L50,10 L42.5,20 L42.5,18.3 L0,18.3 L0,1.7"/> </g> <g fill="none" stroke="#000000" stroke-opacity="1" stroke-width="1" stroke-linecap="square" stroke-linejoin="bevel" transform="matrix(1,0,0,1,1757.13,618)" font-family="MS Shell Dlg 2" font-size="9.75" font-weight="400" font-style="normal" > <text fill="#000000" fill-opacity="1" stroke="none" xml:space="preserve" x="4" y="17" font-family="Arial" font-size="10pt" font-weight="700" font-style="italic" >H</text> </g> <g fill="#ff9999" fill-opacity="1" stroke="#bfbfbf" stroke-opacity="1" stroke-width="1" stroke-linecap="square" stroke-linejoin="bevel" transform="matrix(1,0,0,1,1795,620)" font-family="MS Shell Dlg 2" font-size="9.75" font-weight="400" font-style="normal" > <path vector-effect="none" fill-rule="evenodd" d="M0,1.7 L42.5,1.7 L42.5,0 L50,10 L42.5,20 L42.5,18.3 L0,18.3 L0,1.7"/> </g> <g fill="none" stroke="#000000" stroke-opacity="1" stroke-width="1" stroke-linecap="square" stroke-linejoin="bevel" transform="matrix(1,0,0,1,1808.63,618)" font-family="MS Shell Dlg 2" font-size="9.75" font-weight="400" font-style="normal" > <text fill="#000000" fill-opacity="1" stroke="none" xml:space="preserve" x="4" y="17" font-family="Arial" font-size="10pt" font-weight="700" font-style="italic" >P</text> </g> <g fill="#ffff33" fill-opacity="1" stroke="#bfbfbf" stroke-opacity="1" stroke-width="1" stroke-linecap="square" stroke-linejoin="bevel" transform="matrix(1,0,0,1,1846,620)" font-family="MS Shell Dlg 2" font-size="9.75" font-weight="400" font-style="normal" > <rect x="0" y="0" width="50" height="20"/> </g> <g fill="none" stroke="#000000" stroke-opacity="1" stroke-width="1" stroke-linecap="square" stroke-linejoin="bevel" transform="matrix(1,0,0,1,1853,618)" font-family="MS Shell Dlg 2" font-size="9.75" font-weight="400" font-style="normal" > <text fill="#000000" fill-opacity="1" stroke="none" xml:space="preserve" x="4" y="17" font-family="Arial" font-size="10pt" font-weight="700" font-style="italic" >nad3</text> </g> <g fill="none" stroke="#000000" stroke-opacity="1" stroke-width="1" stroke-linecap="square" stroke-linejoin="bevel" transform="matrix(1,0,0,1,1897,620)" font-family="MS Shell Dlg 2" font-size="9.75" font-weight="400" font-style="normal" > <text fill="#000000" fill-opacity="1" stroke="none" xml:space="preserve" x="4" y="17" font-family="Arial" font-size="10pt" font-weight="700" font-style="italic" >(+)</text> </g> <g fill="none" stroke="#000000" stroke-opacity="1" stroke-width="1" stroke-linecap="square" stroke-linejoin="bevel" transform="matrix(1,0,0,1,10,660)" font-family="MS Shell Dlg 2" font-size="9.75" font-weight="400" font-style="normal" > <text fill="#000000" fill-opacity="1" stroke="none" xml:space="preserve" x="4" y="17" font-family="Arial" font-size="10pt" font-weight="700" font-style="italic" >Mytilus_coruscus_OR453540</text> </g> <g fill="#ffff33" fill-opacity="1" stroke="#bfbfbf" stroke-opacity="1" stroke-width="1" stroke-linecap="square" stroke-linejoin="bevel" transform="matrix(1,0,0,1,10,690)" font-family="MS Shell Dlg 2" font-size="9.75" font-weight="400" font-style="normal" > <rect x="0" y="0" width="50" height="20"/> </g> <g fill="none" stroke="#000000" stroke-opacity="1" stroke-width="1" stroke-linecap="square" stroke-linejoin="bevel" transform="matrix(1,0,0,1,18,688)" font-family="MS Shell Dlg 2" font-size="9.75" font-weight="400" font-style="normal" > <text fill="#000000" fill-opacity="1" stroke="none" xml:space="preserve" x="4" y="17" font-family="Arial" font-size="10pt" font-weight="700" font-style="italic" >cox1</text> </g> <g fill="#ffff33" fill-opacity="1" stroke="#bfbfbf" stroke-opacity="1" stroke-width="1" stroke-linecap="square" stroke-linejoin="bevel" transform="matrix(1,0,0,1,61,690)" font-family="MS Shell Dlg 2" font-size="9.75" font-weight="400" font-style="normal" > <rect x="0" y="0" width="50" height="20"/> </g> <g fill="none" stroke="#000000" stroke-opacity="1" stroke-width="1" stroke-linecap="square" stroke-

linejoin="bevel" transform="matrix(1,0,0,1,69.5,688)" font-family="MS Shell Dlg 2" font-size="9.75" font-weight="400" font-style="normal" > <text fill="#000000" fill-opacity="1" stroke="none" xml:space="preserve" x="4" y="17" font-family="Arial" font-size="10pt" font-weight="700" font-style="italic" >atp6</text> </g> <g fill="#ff9999" fill-opacity="1" stroke="#bfbfbf" stroke-opacity="1" stroke-width="1" stroke-linecap="square" stroke-linejoin="bevel" transform="matrix(1,0,0,1,112,690)" font-family="MS Shell Dlg 2" font-size="9.75" font-weight="400" font-style="normal" > <path vector-effect="none" fill-rule="evenodd" d="M0,1.7 L42.5,1.7 L42.5,0 L50,10 L42.5,20 L42.5,18.3 L0,18.3 L0,1.7"/> </g> <g fill="none" stroke="#000000" stroke-opacity="1" stroke-width="1" stroke-linecap="square" stroke-linejoin="bevel" transform="matrix(1,0,0,1,125.132,688)" font-family="MS Shell Dlg 2" font-size="9.75" font-weight="400" font-style="normal" > <text fill="#000000" fill-opacity="1" stroke="none" xml:space="preserve" x="4" y="17" font-family="Arial" font-size="10pt" font-weight="700" font-style="italic" >T</text> </g> <g fill="#ffff33" fill-opacity="1" stroke="#bfbfbf" stroke-opacity="1" stroke-width="1" stroke-linecap="square" stroke-linejoin="bevel" transform="matrix(1,0,0,1,163,690)" font-family="MS Shell Dlg 2" font-size="9.75" font-weight="400" font-style="normal" > <rect x="0" y="0" width="50" height="20"/> </g> <g fill="none" stroke="#000000" stroke-opacity="1" stroke-width="1" stroke-linecap="square" stroke-linejoin="bevel" transform="matrix(1,0,0,1,167,688)" font-family="MS Shell Dlg 2" font-size="9.75" font-weight="400" font-style="normal" > <text fill="#000000" fill-opacity="1" stroke="none" xml:space="preserve" x="4" y="17" font-family="Arial" font-size="10pt" font-weight="700" font-style="italic" >nad4L</text> </g> <g fill="#ffff33" fill-opacity="1" stroke="#bfbfbf" stroke-opacity="1" stroke-width="1" stroke-linecap="square" stroke-linejoin="bevel" transform="matrix(1,0,0,1,214,690)" font-family="MS Shell Dlg 2" font-size="9.75" font-weight="400" font-style="normal" > <rect x="0" y="0" width="50" height="20"/> </g> <g fill="none" stroke="#000000" stroke-opacity="1" stroke-width="1" stroke-linecap="square" stroke-linejoin="bevel" transform="matrix(1,0,0,1,221,688)" font-family="MS Shell Dlg 2" font-size="9.75" font-weight="400" font-style="normal" > <text fill="#000000" fill-opacity="1" stroke="none" xml:space="preserve" x="4" y="17" font-family="Arial" font-size="10pt" font-weight="700" font-style="italic" >nad5</text> </g> <g fill="#ffff33" fill-opacity="1" stroke="#bfbfbf" stroke-opacity="1" stroke-width="1" stroke-linecap="square" stroke-linejoin="bevel" transform="matrix(1,0,0,1,265,690)" font-family="MS Shell Dlg 2" font-size="9.75" font-weight="400" font-style="normal" > <rect x="0" y="0" width="50" height="20"/> </g> <g fill="none" stroke="#000000" stroke-opacity="1" stroke-width="1" stroke-linecap="square" stroke-linejoin="bevel" transform="matrix(1,0,0,1,272,688)" font-family="MS Shell Dlg 2" font-size="9.75" font-weight="400" font-style="normal" > <text fill="#000000" fill-opacity="1" stroke="none" xml:space="preserve" x="4" y="17" font-family="Arial" font-size="10pt" font-weight="700" font-style="italic" >nad6</text> </g> <g fill="#ff9999" fill-opacity="1" stroke="#bfbfbf" stroke-opacity="1" stroke-width="1" stroke-linecap="square" stroke-linejoin="bevel" transform="matrix(1,0,0,1,316,690)" font-family="MS Shell Dlg 2" font-size="9.75" font-weight="400" font-style="normal" > <path vector-effect="none" fill-rule="evenodd" d="M0,1.7 L42.5,1.7 L42.5,0 L50,10 L42.5,20 L42.5,18.3 L0,18.3 L0,1.7"/> </g> <g fill="none" stroke="#000000" stroke-opacity="1" stroke-width="1" stroke-linecap="square" stroke-linejoin="bevel" transform="matrix(1,0,0,1,329.632,688)" font-family="MS Shell Dlg 2" font-size="9.75" font-weight="400" font-style="normal" > <text fill="#000000" fill-opacity="1" stroke="none" xml:space="preserve" x="4" y="17" font-family="Arial" font-size="10pt" font-weight="700" font-style="italic" >F</text> </g> <g fill="#99ffff" fill-opacity="1" stroke="#bfbfbf" stroke-opacity="1" stroke-width="1" stroke-linecap="square" stroke-linejoin="bevel" transform="matrix(1,0,0,1,367,690)" font-family="MS Shell Dlg 2" font-size="9.75" font-weight="400" font-style="normal" > <path vector-effect="none" fill-rule="evenodd" d="M0,5 C0,2.23858 2.23858,0 5,0 L45,0 C47.7614,0 50,2.23858 50,5 L50,15 C50,17.7614 47.7614,20 45,20 L5,20 C2.23858,20 0,17.7614 0,15 L0,5"/> </g> <g fill="none" stroke="#000000" stroke-opacity="1" stroke-width="1" stroke-linecap="square" stroke-linejoin="bevel" transform="matrix(1,0,0,1,375.5,688)" font-family="MS Shell Dlg 2" font-size="9.75" font-weight="400" font-style="normal" > <text fill="#000000" fill-opacity="1" stroke="none" xml:space="preserve" x="4" y="17" font-family="Arial" font-size="10pt" font-weight="700" font-style="italic" >rrnS</text> </g> <g fill="#ff9999" fill-opacity="1" stroke="#bfbfbf" stroke-opacity="1" stroke-width="1" stroke-linecap="square" stroke-linejoin="bevel" transform="matrix(1,0,0,1,418,690)" font-family="MS Shell Dlg 2" font-size="9.75" font-weight="400" font-style="normal" > <path vector-effect="none" fill-rule="evenodd" d="M0,1.7 L42.5,1.7 L42.5,0 L50,10 L42.5,20 L42.5,18.3 L0,18.3 L0,1.7"/> </g> <g fill="none" stroke="#000000" stroke-opacity="1" stroke-width="1" stroke-linecap="square" stroke-linejoin="bevel" transform="matrix(1,0,0,1,431.132,688)" font-family="MS Shell Dlg 2" font-size="9.75" font-weight="400" font-style="normal" > <text fill="#000000" fill-opacity="1" stroke="none" xml:space="preserve" x="4" y="17" font-family="Arial" font-size="10pt" font-weight="700" font-style="italic" >G</text> </g> <g fill="#ff9999" fill-opacity="1" stroke="#bfbfbf" stroke-opacity="1" stroke-width="1" stroke-linecap="square" stroke-linejoin="bevel" transform="matrix(1,0,0,1,469,690)" font-family="MS Shell Dlg 2" font-size="9.75" font-weight="400" font-

style="normal" > <path vector-effect="none" fill-rule="evenodd" d="M0,1.7 L42.5,1.7 L42.5,0 L50,10 L42.5,20 L42.5,18.3 L0,18.3 L0,1.7"/> </g> <g fill="none" stroke="#000000" stroke-opacity="1" stroke-width="1" stroke-linecap="square" stroke-linejoin="bevel" transform="matrix(1,0,0,1,482.132,688)" font-family="MS Shell Dlg 2" font-size="9.75" font-weight="400" font-style="normal" > <text fill="#000000" fill-opacity="1" stroke="none" xml:space="preserve" x="4" y="17" font-family="Arial" font-size="10pt" font-weight="700" font-style="italic" >N</text> </g> <g fill="#ff9999" fill-opacity="1" stroke="#bfbfbf" stroke-opacity="1" stroke-width="1" stroke-linecap="square" stroke-linejoin="bevel" transform="matrix(1,0,0,1,520,690)" font-family="MS Shell Dlg 2" font-size="9.75" font-weight="400" font-style="normal" > <path vector-effect="none" fill-rule="evenodd" d="M0,1.7 L42.5,1.7 L42.5,0 L50,10 L42.5,20 L42.5,18.3 L0,18.3 L0,1.7"/> </g> <g fill="none" stroke="#000000" stroke-opacity="1" stroke-width="1" stroke-linecap="square" stroke-linejoin="bevel" transform="matrix(1,0,0,1,533.633,688)" font-family="MS Shell Dlg 2" font-size="9.75" font-weight="400" font-style="normal" > <text fill="#000000" fill-opacity="1" stroke="none" xml:space="preserve" x="4" y="17" font-family="Arial" font-size="10pt" font-weight="700" font-style="italic" >E</text> </g> <g fill="#ff9999" fill-opacity="1" stroke="#bfbfbf" stroke-opacity="1" stroke-width="1" stroke-linecap="square" stroke-linejoin="bevel" transform="matrix(1,0,0,1,571,690)" font-family="MS Shell Dlg 2" font-size="9.75" font-weight="400" font-style="normal" > <path vector-effect="none" fill-rule="evenodd" d="M0,1.7 L42.5,1.7 L42.5,0 L50,10 L42.5,20 L42.5,18.3 L0,18.3 L0,1.7"/> </g> <g fill="none" stroke="#000000" stroke-opacity="1" stroke-width="1" stroke-linecap="square" stroke-linejoin="bevel" transform="matrix(1,0,0,1,584.133,688)" font-family="MS Shell Dlg 2" font-size="9.75" font-weight="400" font-style="normal" > <text fill="#000000" fill-opacity="1" stroke="none" xml:space="preserve" x="4" y="17" font-family="Arial" font-size="10pt" font-weight="700" font-style="italic" >C</text> </g> <g fill="#ff9999" fill-opacity="1" stroke="#bfbfbf" stroke-opacity="1" stroke-width="1" stroke-linecap="square" stroke-linejoin="bevel" transform="matrix(1,0,0,1,622,690)" font-family="MS Shell Dlg 2" font-size="9.75" font-weight="400" font-style="normal" > <path vector-effect="none" fill-rule="evenodd" d="M0,1.7 L42.5,1.7 L42.5,0 L50,10 L42.5,20 L42.5,18.3 L0,18.3 L0,1.7"/> </g> <g fill="none" stroke="#000000" stroke-opacity="1" stroke-width="1" stroke-linecap="square" stroke-linejoin="bevel" transform="matrix(1,0,0,1,637.133,688)" font-family="MS Shell Dlg 2" font-size="9.75" font-weight="400" font-style="normal" > <text fill="#000000" fill-opacity="1" stroke="none" xml:space="preserve" x="4" y="17" font-family="Arial" font-size="10pt" font-weight="700" font-style="italic" >I</text> </g> <g fill="#ff9999" fill-opacity="1" stroke="#bfbfbf" stroke-opacity="1" stroke-width="1" stroke-linecap="square" stroke-linejoin="bevel" transform="matrix(1,0,0,1,673,690)" font-family="MS Shell Dlg 2" font-size="9.75" font-weight="400" font-style="normal" > <path vector-effect="none" fill-rule="evenodd" d="M0,1.7 L42.5,1.7 L42.5,0 L50,10 L42.5,20 L42.5,18.3 L0,18.3 L0,1.7"/> </g> <g fill="none" stroke="#000000" stroke-opacity="1" stroke-width="1" stroke-linecap="square" stroke-linejoin="bevel" transform="matrix(1,0,0,1,685.633,688)" font-family="MS Shell Dlg 2" font-size="9.75" font-weight="400" font-style="normal" > <text fill="#000000" fill-opacity="1" stroke="none" xml:space="preserve" x="4" y="17" font-family="Arial" font-size="10pt" font-weight="700" font-style="italic" >Q</text> </g> <g fill="#ff9999" fill-opacity="1" stroke="#bfbfbf" stroke-opacity="1" stroke-width="1" stroke-linecap="square" stroke-linejoin="bevel" transform="matrix(1,0,0,1,724,690)" font-family="MS Shell Dlg 2" font-size="9.75" font-weight="400" font-style="normal" > <path vector-effect="none" fill-rule="evenodd" d="M0,1.7 L42.5,1.7 L42.5,0 L50,10 L42.5,20 L42.5,18.3 L0,18.3 L0,1.7"/> </g> <g fill="none" stroke="#000000" stroke-opacity="1" stroke-width="1" stroke-linecap="square" stroke-linejoin="bevel" transform="matrix(1,0,0,1,737.133,688)" font-family="MS Shell Dlg 2" font-size="9.75" font-weight="400" font-style="normal" > <text fill="#000000" fill-opacity="1" stroke="none" xml:space="preserve" x="4" y="17" font-family="Arial" font-size="10pt" font-weight="700" font-style="italic" >D</text> </g> <g fill="#99ffff" fill-opacity="1" stroke="#bfbfbf" stroke-opacity="1" stroke-width="1" stroke-linecap="square" stroke-linejoin="bevel" transform="matrix(1,0,0,1,775,690)" font-family="MS Shell Dlg 2" font-size="9.75" font-weight="400" font-style="normal" > <path vector-effect="none" fill-rule="evenodd" d="M0,5 C0,2.23858 2.23858,0 5,0 L45,0 C47.7614,0 50,2.23858 50,5 L50,15 C50,17.7614 47.7614,20 45,20 L5,20 C2.23858,20 0,17.7614 0,15 L0,5"/> </g> <g fill="none" stroke="#000000" stroke-opacity="1" stroke-width="1" stroke-linecap="square" stroke-linejoin="bevel" transform="matrix(1,0,0,1,784.5,688)" font-family="MS Shell Dlg 2" font-size="9.75" font-weight="400" font-style="normal" > <text fill="#000000" fill-opacity="1" stroke="none" xml:space="preserve" x="4" y="17" font-family="Arial" font-size="10pt" font-weight="700" font-style="italic" >rrnL</text> </g> <g fill="#ff9999" fill-opacity="1" stroke="#bfbfbf" stroke-opacity="1" stroke-width="1" stroke-linecap="square" stroke-linejoin="bevel" transform="matrix(1,0,0,1,826,690)" font-family="MS Shell Dlg 2" font-size="9.75" font-weight="400" font-style="normal" > <path vector-effect="none" fill-rule="evenodd" d="M0,1.7 L42.5,1.7 L42.5,0 L50,10 L42.5,20 L42.5,18.3 L0,18.3 L0,1.7"/> </g> <g fill="none" stroke="#000000" stroke-opacity="1" stroke-width="1" stroke-linecap="square" stroke-linejoin="bevel" transform="matrix(1,0,0,1,839.633,688)" font-family="MS Shell Dlg 2" font-size="9.75" font-weight="400" font-style="normal" > <text fill="#000000" fill-

opacity="1" stroke="none" xml:space="preserve" x="4" y="17" font-family="Arial" font-size="10pt" font-weight="700" font-style="italic" >Y</text> </g> <g fill="#ffff33" fill-opacity="1" stroke="#bfbfbf" stroke-opacity="1" stroke-width="1" stroke-linecap="square" stroke-linejoin="bevel" transform="matrix(1,0,0,1,877,690)" font-family="MS Shell Dlg 2" font-size="9.75" font-weight="400" font-style="normal" > <rect x="0" y="0" width="50" height="20"/> </g> <g fill="none" stroke="#000000" stroke-opacity="1" stroke-width="1" stroke-linecap="square" stroke-linejoin="bevel" transform="matrix(1,0,0,1,886.5,688)" font-family="MS Shell Dlg 2" font-size="9.75" font-weight="400" font-style="normal" > <text fill="#000000" fill-opacity="1" stroke="none" xml:space="preserve" x="4" y="17" font-family="Arial" font-size="10pt" font-weight="700" font-style="italic" >cytb</text> </g> <g fill="#ffff33" fill-opacity="1" stroke="#bfbfbf" stroke-opacity="1" stroke-width="1" stroke-linecap="square" stroke-linejoin="bevel" transform="matrix(1,0,0,1,928,690)" font-family="MS Shell Dlg 2" font-size="9.75" font-weight="400" font-style="normal" > <rect x="0" y="0" width="50" height="20"/> </g> <g fill="none" stroke="#000000" stroke-opacity="1" stroke-width="1" stroke-linecap="square" stroke-linejoin="bevel" transform="matrix(1,0,0,1,936,688)" font-family="MS Shell Dlg 2" font-size="9.75" font-weight="400" font-style="normal" > <text fill="#000000" fill-opacity="1" stroke="none" xml:space="preserve" x="4" y="17" font-family="Arial" font-size="10pt" font-weight="700" font-style="italic" >cox2</text> </g> <g fill="#ff9999" fill-opacity="1" stroke="#bfbfbf" stroke-opacity="1" stroke-width="1" stroke-linecap="square" stroke-linejoin="bevel" transform="matrix(1,0,0,1,979,690)" font-family="MS Shell Dlg 2" font-size="9.75" font-weight="400" font-style="normal" > <path vector-effect="none" fill-rule="evenodd" d="M0,1.7 L42.5,1.7 L42.5,0 L50,10 L42.5,20 L42.5,18.3 L0,18.3 L0,1.7"/> </g> <g fill="none" stroke="#000000" stroke-opacity="1" stroke-width="1" stroke-linecap="square" stroke-linejoin="bevel" transform="matrix(1,0,0,1,992.633,688)" font-family="MS Shell Dlg 2" font-size="9.75" font-weight="400" font-style="normal" > <text fill="#000000" fill-opacity="1" stroke="none" xml:space="preserve" x="4" y="17" font-family="Arial" font-size="10pt" font-weight="700" font-style="italic" >K</text> </g> <g fill="#ff9999" fill-opacity="1" stroke="#bfbfbf" stroke-opacity="1" stroke-width="1" stroke-linecap="square" stroke-linejoin="bevel" transform="matrix(1,0,0,1,1030,690)" font-family="MS Shell Dlg 2" font-size="9.75" font-weight="400" font-style="normal" > <path vector-effect="none" fill-rule="evenodd" d="M0,1.7 L42.5,1.7 L42.5,0 L50,10 L42.5,20 L42.5,18.3 L0,18.3 L0,1.7"/> </g> <g fill="none" stroke="#000000" stroke-opacity="1" stroke-width="1" stroke-linecap="square" stroke-linejoin="bevel" transform="matrix(1,0,0,1,1042.13,688)" font-family="MS Shell Dlg 2" font-size="9.75" font-weight="400" font-style="normal" > <text fill="#000000" fill-opacity="1" stroke="none" xml:space="preserve" x="4" y="17" font-family="Arial" font-size="10pt" font-weight="700" font-style="italic" >M</text> </g> <g fill="#ff9999" fill-opacity="1" stroke="#bfbfbf" stroke-opacity="1" stroke-width="1" stroke-linecap="square" stroke-linejoin="bevel" transform="matrix(1,0,0,1,1081,690)" font-family="MS Shell Dlg 2" font-size="9.75" font-weight="400" font-style="normal" > <path vector-effect="none" fill-rule="evenodd" d="M0,1.7 L42.5,1.7 L42.5,0 L50,10 L42.5,20 L42.5,18.3 L0,18.3 L0,1.7"/> </g> <g fill="none" stroke="#000000" stroke-opacity="1" stroke-width="1" stroke-linecap="square" stroke-linejoin="bevel" transform="matrix(1,0,0,1,1091.63,688)" font-family="MS Shell Dlg 2" font-size="9.75" font-weight="400" font-style="normal" > <text fill="#000000" fill-opacity="1" stroke="none" xml:space="preserve" x="4" y="17" font-family="Arial" font-size="10pt" font-weight="700" font-style="italic" >L1</text> </g> <g fill="#ff9999" fill-opacity="1" stroke="#bfbfbf" stroke-opacity="1" stroke-width="1" stroke-linecap="square" stroke-linejoin="bevel" transform="matrix(1,0,0,1,1132,690)" font-family="MS Shell Dlg 2" font-size="9.75" font-weight="400" font-style="normal" > <path vector-effect="none" fill-rule="evenodd" d="M0,1.7 L42.5,1.7 L42.5,0 L50,10 L42.5,20 L42.5,18.3 L0,18.3 L0,1.7"/> </g> <g fill="none" stroke="#000000" stroke-opacity="1" stroke-width="1" stroke-linecap="square" stroke-linejoin="bevel" transform="matrix(1,0,0,1,1142.63,688)" font-family="MS Shell Dlg 2" font-size="9.75" font-weight="400" font-style="normal" > <text fill="#000000" fill-opacity="1" stroke="none" xml:space="preserve" x="4" y="17" font-family="Arial" font-size="10pt" font-weight="700" font-style="italic" >L2</text> </g> <g fill="#ffff33" fill-opacity="1" stroke="#bfbfbf" stroke-opacity="1" stroke-width="1" stroke-linecap="square" stroke-linejoin="bevel" transform="matrix(1,0,0,1,1183,690)" font-family="MS Shell Dlg 2" font-size="9.75" font-weight="400" font-style="normal" > <rect x="0" y="0" width="50" height="20"/> </g> <g fill="none" stroke="#000000" stroke-opacity="1" stroke-width="1" stroke-linecap="square" stroke-linejoin="bevel" transform="matrix(1,0,0,1,1190,688)" font-family="MS Shell Dlg 2" font-size="9.75" font-weight="400" font-style="normal" > <text fill="#000000" fill-opacity="1" stroke="none" xml:space="preserve" x="4" y="17" font-family="Arial" font-size="10pt" font-weight="700" font-style="italic" >nad1</text> </g> <g fill="#ff9999" fill-opacity="1" stroke="#bfbfbf" stroke-opacity="1" stroke-width="1" stroke-linecap="square" stroke-linejoin="bevel" transform="matrix(1,0,0,1,1234,690)" font-family="MS Shell Dlg 2" font-size="9.75" font-weight="400" font-style="normal" > <path vector-effect="none" fill-rule="evenodd" d="M0,1.7 L42.5,1.7 L42.5,0 L50,10 L42.5,20 L42.5,18.3 L0,18.3 L0,1.7"/> </g> <g fill="none" stroke="#000000" stroke-opacity="1"

stroke-width="1" stroke-linecap="square" stroke-linejoin="bevel" transform="matrix(1,0,0,1,1247.13,688)" font-family="MS Shell Dlg 2" font-size="9.75" font-weight="400" font-style="normal" > <text fill="#000000" fill-opacity="1" stroke="none" xml:space="preserve" x="4" y="17" font-family="Arial" font-size="10pt" font-weight="700" font-style="italic" >V</text> </g> <g fill="#ffff33" fill-opacity="1" stroke="#bfbfbf" stroke-opacity="1" stroke-width="1" stroke-linecap="square" stroke-linejoin="bevel" transform="matrix(1,0,0,1,1285,690)" font-family="MS Shell Dlg 2" font-size="9.75" font-weight="400" font-style="normal" > <rect x="0" y="0" width="50" height="20"/> </g> <g fill="none" stroke="#000000" stroke-opacity="1" stroke-width="1" stroke-linecap="square" stroke-linejoin="bevel" transform="matrix(1,0,0,1,1292,688)" font-family="MS Shell Dlg 2" font-size="9.75" font-weight="400" font-style="normal" > <text fill="#000000" fill-opacity="1" stroke="none" xml:space="preserve" x="4" y="17" font-family="Arial" font-size="10pt" font-weight="700" font-style="italic" >nad4</text> </g> <g fill="#ffff33" fill-opacity="1" stroke="#bfbfbf" stroke-opacity="1" stroke-width="1" stroke-linecap="square" stroke-linejoin="bevel" transform="matrix(1,0,0,1,1336,690)" font-family="MS Shell Dlg 2" font-size="9.75" font-weight="400" font-style="normal" > <rect x="0" y="0" width="50" height="20"/> </g> <g fill="none" stroke="#000000" stroke-opacity="1" stroke-width="1" stroke-linecap="square" stroke-linejoin="bevel" transform="matrix(1,0,0,1,1344,688)" font-family="MS Shell Dlg 2" font-size="9.75" font-weight="400" font-style="normal" > <text fill="#000000" fill-opacity="1" stroke="none" xml:space="preserve" x="4" y="17" font-family="Arial" font-size="10pt" font-weight="700" font-style="italic" >cox3</text> </g> <g fill="#ff9999" fill-opacity="1" stroke="#bfbfbf" stroke-opacity="1" stroke-width="1" stroke-linecap="square" stroke-linejoin="bevel" transform="matrix(1,0,0,1,1387,690)" font-family="MS Shell Dlg 2" font-size="9.75" font-weight="400" font-style="normal" > <path vector-effect="none" fill-rule="evenodd" d="M0,1.7 L42.5,1.7 L42.5,0 L50,10 L42.5,20 L42.5,18.3 L0,18.3 L0,1.7"/> </g> <g fill="none" stroke="#000000" stroke-opacity="1" stroke-width="1" stroke-linecap="square" stroke-linejoin="bevel" transform="matrix(1,0,0,1,1396.63,688)" font-family="MS Shell Dlg 2" font-size="9.75" font-weight="400" font-style="normal" > <text fill="#000000" fill-opacity="1" stroke="none" xml:space="preserve" x="4" y="17" font-family="Arial" font-size="10pt" font-weight="700" font-style="italic" >S2</text> </g> <g fill="#ff9999" fill-opacity="1" stroke="#bfbfbf" stroke-opacity="1" stroke-width="1" stroke-linecap="square" stroke-linejoin="bevel" transform="matrix(1,0,0,1,1438,690)" font-family="MS Shell Dlg 2" font-size="9.75" font-weight="400" font-style="normal" > <path vector-effect="none" fill-rule="evenodd" d="M0,1.7 L42.5,1.7 L42.5,0 L50,10 L42.5,20 L42.5,18.3 L0,18.3 L0,1.7"/> </g> <g fill="none" stroke="#000000" stroke-opacity="1" stroke-width="1" stroke-linecap="square" stroke-linejoin="bevel" transform="matrix(1,0,0,1,1450.13,688)" font-family="MS Shell Dlg 2" font-size="9.75" font-weight="400" font-style="normal" > <text fill="#000000" fill-opacity="1" stroke="none" xml:space="preserve" x="4" y="17" font-family="Arial" font-size="10pt" font-weight="700" font-style="italic" >M</text> </g> <g fill="#ffff33" fill-opacity="1" stroke="#bfbfbf" stroke-opacity="1" stroke-width="1" stroke-linecap="square" stroke-linejoin="bevel" transform="matrix(1,0,0,1,1489,690)" font-family="MS Shell Dlg 2" font-size="9.75" font-weight="400" font-style="normal" > <rect x="0" y="0" width="50" height="20"/> </g> <g fill="none" stroke="#000000" stroke-opacity="1" stroke-width="1" stroke-linecap="square" stroke-linejoin="bevel" transform="matrix(1,0,0,1,1496,688)" font-family="MS Shell Dlg 2" font-size="9.75" font-weight="400" font-style="normal" > <text fill="#000000" fill-opacity="1" stroke="none" xml:space="preserve" x="4" y="17" font-family="Arial" font-size="10pt" font-weight="700" font-style="italic" >nad2</text> </g> <g fill="#ff9999" fill-opacity="1" stroke="#bfbfbf" stroke-opacity="1" stroke-width="1" stroke-linecap="square" stroke-linejoin="bevel" transform="matrix(1,0,0,1,1540,690)" font-family="MS Shell Dlg 2" font-size="9.75" font-weight="400" font-style="normal" > <path vector-effect="none" fill-rule="evenodd" d="M0,1.7 L42.5,1.7 L42.5,0 L50,10 L42.5,20 L42.5,18.3 L0,18.3 L0,1.7"/> </g> <g fill="none" stroke="#000000" stroke-opacity="1" stroke-width="1" stroke-linecap="square" stroke-linejoin="bevel" transform="matrix(1,0,0,1,1553.13,688)" font-family="MS Shell Dlg 2" font-size="9.75" font-weight="400" font-style="normal" > <text fill="#000000" fill-opacity="1" stroke="none" xml:space="preserve" x="4" y="17" font-family="Arial" font-size="10pt" font-weight="700" font-style="italic" >R</text> </g> <g fill="#ff9999" fill-opacity="1" stroke="#bfbfbf" stroke-opacity="1" stroke-width="1" stroke-linecap="square" stroke-linejoin="bevel" transform="matrix(1,0,0,1,1591,690)" font-family="MS Shell Dlg 2" font-size="9.75" font-weight="400" font-style="normal" > <path vector-effect="none" fill-rule="evenodd" d="M0,1.7 L42.5,1.7 L42.5,0 L50,10 L42.5,20 L42.5,18.3 L0,18.3 L0,1.7"/> </g> <g fill="none" stroke="#000000" stroke-opacity="1" stroke-width="1" stroke-linecap="square" stroke-linejoin="bevel" transform="matrix(1,0,0,1,1602.13,688)" font-family="MS Shell Dlg 2" font-size="9.75" font-weight="400" font-style="normal" > <text fill="#000000" fill-opacity="1" stroke="none" xml:space="preserve" x="4" y="17" font-family="Arial" font-size="10pt" font-weight="700" font-style="italic" >W</text> </g> <g fill="#ff9999" fill-opacity="1" stroke="#bfbfbf" stroke-opacity="1" stroke-width="1" stroke-linecap="square" stroke-linejoin="bevel" transform="matrix(1,0,0,1,1642,690)" font-family="MS Shell Dlg 2" font-size="9.75" font-weight="400" font-

style="normal" > <path vector-effect="none" fill-rule="evenodd" d="M0,1.7 L42.5,1.7 L42.5,0 L50,10 L42.5,20 L42.5,18.3 L0,18.3 L0,1.7"/> </g> <g fill="none" stroke="#000000" stroke-opacity="1" stroke-width="1" stroke-linecap="square" stroke-linejoin="bevel" transform="matrix(1,0,0,1,1655.13,688)" font-family="MS Shell Dlg 2" font-size="9.75" font-weight="400" font-style="normal" > <text fill="#000000" fill-opacity="1" stroke="none" xml:space="preserve" x="4" y="17" font-family="Arial" font-size="10pt" font-weight="700" font-style="italic" >A</text> </g> <g fill="#ff9999" fill-opacity="1" stroke="#bfbfbf" stroke-opacity="1" stroke-width="1" stroke-linecap="square" stroke-linejoin="bevel" transform="matrix(1,0,0,1,1693,690)" font-family="MS Shell Dlg 2" font-size="9.75" font-weight="400" font-style="normal" > <path vector-effect="none" fill-rule="evenodd" d="M0,1.7 L42.5,1.7 L42.5,0 L50,10 L42.5,20 L42.5,18.3 L0,18.3 L0,1.7"/> </g> <g fill="none" stroke="#000000" stroke-opacity="1" stroke-width="1" stroke-linecap="square" stroke-linejoin="bevel" transform="matrix(1,0,0,1,1702.63,688)" font-family="MS Shell Dlg 2" font-size="9.75" font-weight="400" font-style="normal" > <text fill="#000000" fill-opacity="1" stroke="none" xml:space="preserve" x="4" y="17" font-family="Arial" font-size="10pt" font-weight="700" font-style="italic" >S1</text> </g> <g fill="#ff9999" fill-opacity="1" stroke="#bfbfbf" stroke-opacity="1" stroke-width="1" stroke-linecap="square" stroke-linejoin="bevel" transform="matrix(1,0,0,1,1744,690)" font-family="MS Shell Dlg 2" font-size="9.75" font-weight="400" font-style="normal" > <path vector-effect="none" fill-rule="evenodd" d="M0,1.7 L42.5,1.7 L42.5,0 L50,10 L42.5,20 L42.5,18.3 L0,18.3 L0,1.7"/> </g> <g fill="none" stroke="#000000" stroke-opacity="1" stroke-width="1" stroke-linecap="square" stroke-linejoin="bevel" transform="matrix(1,0,0,1,1757.13,688)" font-family="MS Shell Dlg 2" font-size="9.75" font-weight="400" font-style="normal" > <text fill="#000000" fill-opacity="1" stroke="none" xml:space="preserve" x="4" y="17" font-family="Arial" font-size="10pt" font-weight="700" font-style="italic" >H</text> </g> <g fill="#ff9999" fill-opacity="1" stroke="#bfbfbf" stroke-opacity="1" stroke-width="1" stroke-linecap="square" stroke-linejoin="bevel" transform="matrix(1,0,0,1,1795,690)" font-family="MS Shell Dlg 2" font-size="9.75" font-weight="400" font-style="normal" > <path vector-effect="none" fill-rule="evenodd" d="M0,1.7 L42.5,1.7 L42.5,0 L50,10 L42.5,20 L42.5,18.3 L0,18.3 L0,1.7"/> </g> <g fill="none" stroke="#000000" stroke-opacity="1" stroke-width="1" stroke-linecap="square" stroke-linejoin="bevel" transform="matrix(1,0,0,1,1808.63,688)" font-family="MS Shell Dlg 2" font-size="9.75" font-weight="400" font-style="normal" > <text fill="#000000" fill-opacity="1" stroke="none" xml:space="preserve" x="4" y="17" font-family="Arial" font-size="10pt" font-weight="700" font-style="italic" >P</text> </g> <g fill="#ffff33" fill-opacity="1" stroke="#bfbfbf" stroke-opacity="1" stroke-width="1" stroke-linecap="square" stroke-linejoin="bevel" transform="matrix(1,0,0,1,1846,690)" font-family="MS Shell Dlg 2" font-size="9.75" font-weight="400" font-style="normal" > <rect x="0" y="0" width="50" height="20"/> </g> <g fill="none" stroke="#000000" stroke-opacity="1" stroke-width="1" stroke-linecap="square" stroke-linejoin="bevel" transform="matrix(1,0,0,1,1853,688)" font-family="MS Shell Dlg 2" font-size="9.75" font-weight="400" font-style="normal" > <text fill="#000000" fill-opacity="1" stroke="none" xml:space="preserve" x="4" y="17" font-family="Arial" font-size="10pt" font-weight="700" font-style="italic" >nad3</text> </g> <g fill="none" stroke="#000000" stroke-opacity="1" stroke-width="1" stroke-linecap="square" stroke-linejoin="bevel" transform="matrix(1,0,0,1,1897,690)" font-family="MS Shell Dlg 2" font-size="9.75" font-weight="400" font-style="normal" > <text fill="#000000" fill-opacity="1" stroke="none" xml:space="preserve" x="4" y="17" font-family="Arial" font-size="10pt" font-weight="700" font-style="italic" >(+)</text> </g> <g fill="none" stroke="#000000" stroke-opacity="1" stroke-width="1" stroke-linecap="square" stroke-linejoin="bevel" transform="matrix(1,0,0,1,10,730)" font-family="MS Shell Dlg 2" font-size="9.75" font-weight="400" font-style="normal" > <text fill="#000000" fill-opacity="1" stroke="none" xml:space="preserve" x="4" y="17" font-family="Arial" font-size="10pt" font-weight="700" font-style="italic" >Mytilus_californianus_JX486124</text> </g> <g fill="#ffff33" fill-opacity="1" stroke="#bfbfbf" stroke-opacity="1" stroke-width="1" stroke-linecap="square" stroke-linejoin="bevel" transform="matrix(1,0,0,1,10,760)" font-family="MS Shell Dlg 2" font-size="9.75" font-weight="400" font-style="normal" > <rect x="0" y="0" width="50" height="20"/> </g> <g fill="none" stroke="#000000" stroke-opacity="1" stroke-width="1" stroke-linecap="square" stroke-linejoin="bevel" transform="matrix(1,0,0,1,18,758)" font-family="MS Shell Dlg 2" font-size="9.75" font-weight="400" font-style="normal" > <text fill="#000000" fill-opacity="1" stroke="none" xml:space="preserve" x="4" y="17" font-family="Arial" font-size="10pt" font-weight="700" font-style="italic" >cox1</text> </g> <g fill="#ffff33" fill-opacity="1" stroke="#bfbfbf" stroke-opacity="1" stroke-width="1" stroke-linecap="square" stroke-linejoin="bevel" transform="matrix(1,0,0,1,61,760)" font-family="MS Shell Dlg 2" font-size="9.75" font-weight="400" font-style="normal" > <rect x="0" y="0" width="50" height="20"/> </g> <g fill="none" stroke="#000000" stroke-opacity="1" stroke-width="1" stroke-linecap="square" stroke-linejoin="bevel" transform="matrix(1,0,0,1,69.5,758)" font-family="MS Shell Dlg 2" font-size="9.75" font-weight="400" font-style="normal" > <text fill="#000000" fill-opacity="1" stroke="none" xml:space="preserve" x="4" y="17" font-family="Arial" font-size="10pt" font-weight="700" font-style="italic" >atp6</text> </g> <g fill="#ff9999" fill-opacity="1" stroke="#bfbfbf" stroke-opacity="1" stroke-width="1" stroke-linecap="square"

stroke-linejoin="bevel" transform="matrix(1,0,0,1,112,760)" font-family="MS Shell Dlg 2" font-size="9.75" font-weight="400" font-style="normal" > <path vector-effect="none" fill-rule="evenodd" d="M0,1.7 L42.5,1.7 L42.5,0 L50,10 L42.5,20 L42.5,18.3 L0,18.3 L0,1.7"/> </g> <g fill="none" stroke="#000000" stroke-opacity="1" stroke-width="1" stroke-linecap="square" stroke-linejoin="bevel" transform="matrix(1,0,0,1,125.132,758)" font-family="MS Shell Dlg 2" font-size="9.75" font-weight="400" font-style="normal" > <text fill="#000000" fill-opacity="1" stroke="none" xml:space="preserve" x="4" y="17" font-family="Arial" font-size="10pt" font-weight="700" font-style="italic" >T</text> </g> <g fill="#ffff33" fill-opacity="1" stroke="#bfbfbf" stroke-opacity="1" stroke-width="1" stroke-linecap="square" stroke-linejoin="bevel" transform="matrix(1,0,0,1,163,760)" font-family="MS Shell Dlg 2" font-size="9.75" font-weight="400" font-style="normal" > <rect x="0" y="0" width="50" height="20"/> </g> <g fill="none" stroke="#000000" stroke-opacity="1" stroke-width="1" stroke-linecap="square" stroke-linejoin="bevel" transform="matrix(1,0,0,1,167,758)" font-family="MS Shell Dlg 2" font-size="9.75" font-weight="400" font-style="normal" > <text fill="#000000" fill-opacity="1" stroke="none" xml:space="preserve" x="4" y="17" font-family="Arial" font-size="10pt" font-weight="700" font-style="italic" >nad4L</text> </g> <g fill="#ffff33" fill-opacity="1" stroke="#bfbfbf" stroke-opacity="1" stroke-width="1" stroke-linecap="square" stroke-linejoin="bevel" transform="matrix(1,0,0,1,214,760)" font-family="MS Shell Dlg 2" font-size="9.75" font-weight="400" font-style="normal" > <rect x="0" y="0" width="50" height="20"/> </g> <g fill="none" stroke="#000000" stroke-opacity="1" stroke-width="1" stroke-linecap="square" stroke-linejoin="bevel" transform="matrix(1,0,0,1,221,758)" font-family="MS Shell Dlg 2" font-size="9.75" font-weight="400" font-style="normal" > <text fill="#000000" fill-opacity="1" stroke="none" xml:space="preserve" x="4" y="17" font-family="Arial" font-size="10pt" font-weight="700" font-style="italic" >nad5</text> </g> <g fill="#ffff33" fill-opacity="1" stroke="#bfbfbf" stroke-opacity="1" stroke-width="1" stroke-linecap="square" stroke-linejoin="bevel" transform="matrix(1,0,0,1,265,760)" font-family="MS Shell Dlg 2" font-size="9.75" font-weight="400" font-style="normal" > <rect x="0" y="0" width="50" height="20"/> </g> <g fill="none" stroke="#000000" stroke-opacity="1" stroke-width="1" stroke-linecap="square" stroke-linejoin="bevel" transform="matrix(1,0,0,1,272,758)" font-family="MS Shell Dlg 2" font-size="9.75" font-weight="400" font-style="normal" > <text fill="#000000" fill-opacity="1" stroke="none" xml:space="preserve" x="4" y="17" font-family="Arial" font-size="10pt" font-weight="700" font-style="italic" >nad6</text> </g> <g fill="#ff9999" fill-opacity="1" stroke="#bfbfbf" stroke-opacity="1" stroke-width="1" stroke-linecap="square" stroke-linejoin="bevel" transform="matrix(1,0,0,1,316,760)" font-family="MS Shell Dlg 2" font-size="9.75" font-weight="400" font-style="normal" > <path vector-effect="none" fill-rule="evenodd" d="M0,1.7 L42.5,1.7 L42.5,0 L50,10 L42.5,20 L42.5,18.3 L0,18.3 L0,1.7"/> </g> <g fill="none" stroke="#000000" stroke-opacity="1" stroke-width="1" stroke-linecap="square" stroke-linejoin="bevel" transform="matrix(1,0,0,1,329.632,758)" font-family="MS Shell Dlg 2" font-size="9.75" font-weight="400" font-style="normal" > <text fill="#000000" fill-opacity="1" stroke="none" xml:space="preserve" x="4" y="17" font-family="Arial" font-size="10pt" font-weight="700" font-style="italic" >F</text> </g> <g fill="#99ffff" fill-opacity="1" stroke="#bfbfbf" stroke-opacity="1" stroke-width="1" stroke-linecap="square" stroke-linejoin="bevel" transform="matrix(1,0,0,1,367,760)" font-family="MS Shell Dlg 2" font-size="9.75" font-weight="400" font-style="normal" > <path vector-effect="none" fill-rule="evenodd" d="M0,5 C0,2.23858 2.23858,0 5,0 L45,0 C47.7614,0 50,2.23858 50,5 L50,15 C50,17.7614 47.7614,20 45,20 L5,20 C2.23858,20 0,17.7614 0,15 L0,5"/> </g> <g fill="none" stroke="#000000" stroke-opacity="1" stroke-width="1" stroke-linecap="square" stroke-linejoin="bevel" transform="matrix(1,0,0,1,375.5,758)" font-family="MS Shell Dlg 2" font-size="9.75" font-weight="400" font-style="normal" > <text fill="#000000" fill-opacity="1" stroke="none" xml:space="preserve" x="4" y="17" font-family="Arial" font-size="10pt" font-weight="700" font-style="italic" >rrnS</text> </g> <g fill="#ff9999" fill-opacity="1" stroke="#bfbfbf" stroke-opacity="1" stroke-width="1" stroke-linecap="square" stroke-linejoin="bevel" transform="matrix(1,0,0,1,418,760)" font-family="MS Shell Dlg 2" font-size="9.75" font-weight="400" font-style="normal" > <path vector-effect="none" fill-rule="evenodd" d="M0,1.7 L42.5,1.7 L42.5,0 L50,10 L42.5,20 L42.5,18.3 L0,18.3 L0,1.7"/> </g> <g fill="none" stroke="#000000" stroke-opacity="1" stroke-width="1" stroke-linecap="square" stroke-linejoin="bevel" transform="matrix(1,0,0,1,431.132,758)" font-family="MS Shell Dlg 2" font-size="9.75" font-weight="400" font-style="normal" > <text fill="#000000" fill-opacity="1" stroke="none" xml:space="preserve" x="4" y="17" font-family="Arial" font-size="10pt" font-weight="700" font-style="italic" >G</text> </g> <g fill="#ff9999" fill-opacity="1" stroke="#bfbfbf" stroke-opacity="1" stroke-width="1" stroke-linecap="square" stroke-linejoin="bevel" transform="matrix(1,0,0,1,469,760)" font-family="MS Shell Dlg 2" font-size="9.75" font-weight="400" font-style="normal" > <path vector-effect="none" fill-rule="evenodd" d="M0,1.7 L42.5,1.7 L42.5,0 L50,10 L42.5,20 L42.5,18.3 L0,18.3 L0,1.7"/> </g> <g fill="none" stroke="#000000" stroke-opacity="1" stroke-width="1" stroke-linecap="square" stroke-linejoin="bevel" transform="matrix(1,0,0,1,482.132,758)" font-family="MS Shell Dlg 2" font-size="9.75" font-weight="400" font-style="normal" > <text fill="#000000" fill-opacity="1"

stroke="none" xml:space="preserve" x="4" y="17" font-family="Arial" font-size="10pt" font-weight="700" font-
style="italic" >N</text> </g> <g fill="#ff9999" fill-opacity="1" stroke="#bfbfbf" stroke-opacity="1"
stroke-width="1" stroke-linecap="square" stroke-linejoin="bevel" transform="matrix(1,0,0,1,520,760)" font-
family="MS Shell Dlg 2" font-size="9.75" font-weight="400" font-style="normal" > <path vector-effect="none"
fill-rule="evenodd" d="M0,1.7 L42.5,1.7 L42.5,0 L50,10 L42.5,20 L42.5,18.3 L0,18.3 L0,1.7"/> </g> <g
fill="none" stroke="#000000" stroke-opacity="1" stroke-width="1" stroke-linecap="square" stroke-linejoin="bevel"
transform="matrix(1,0,0,1,533.633,758)" font-family="MS Shell Dlg 2" font-size="9.75" font-weight="400" font-
style="normal" > <text fill="#000000" fill-opacity="1" stroke="none" xml:space="preserve" x="4" y="17" font-
family="Arial" font-size="10pt" font-weight="700" font-style="italic" >E</text> </g> <g fill="#ff9999"
fill-opacity="1" stroke="#bfbfbf" stroke-opacity="1" stroke-width="1" stroke-linecap="square" stroke-
linejoin="bevel" transform="matrix(1,0,0,1,571,760)" font-family="MS Shell Dlg 2" font-size="9.75" font-
weight="400" font-style="normal" > <path vector-effect="none" fill-rule="evenodd" d="M0,1.7 L42.5,1.7 L42.5,0
L50,10 L42.5,20 L42.5,18.3 L0,18.3 L0,1.7"/> </g> <g fill="none" stroke="#000000" stroke-opacity="1"
stroke-width="1" stroke-linecap="square" stroke-linejoin="bevel" transform="matrix(1,0,0,1,584.133,758)" font-
family="MS Shell Dlg 2" font-size="9.75" font-weight="400" font-style="normal" > <text fill="#000000" fill-
opacity="1" stroke="none" xml:space="preserve" x="4" y="17" font-family="Arial" font-size="10pt" font-
weight="700" font-style="italic" >C</text> </g> <g fill="#ff9999" fill-opacity="1" stroke="#bfbfbf"
stroke-opacity="1" stroke-width="1" stroke-linecap="square" stroke-linejoin="bevel"
transform="matrix(1,0,0,1,622,760)" font-family="MS Shell Dlg 2" font-size="9.75" font-weight="400" font-
style="normal" > <path vector-effect="none" fill-rule="evenodd" d="M0,1.7 L42.5,1.7 L42.5,0 L50,10 L42.5,20
L42.5,18.3 L0,18.3 L0,1.7"/> </g> <g fill="none" stroke="#000000" stroke-opacity="1" stroke-width="1"
stroke-linecap="square" stroke-linejoin="bevel" transform="matrix(1,0,0,1,637.133,758)" font-family="MS Shell
Dlg 2" font-size="9.75" font-weight="400" font-style="normal" > <text fill="#000000" fill-opacity="1"
stroke="none" xml:space="preserve" x="4" y="17" font-family="Arial" font-size="10pt" font-weight="700" font-
style="italic" >I</text> </g> <g fill="#ff9999" fill-opacity="1" stroke="#bfbfbf" stroke-opacity="1"
stroke-width="1" stroke-linecap="square" stroke-linejoin="bevel" transform="matrix(1,0,0,1,673,760)" font-
family="MS Shell Dlg 2" font-size="9.75" font-weight="400" font-style="normal" > <path vector-effect="none"
fill-rule="evenodd" d="M0,1.7 L42.5,1.7 L42.5,0 L50,10 L42.5,20 L42.5,18.3 L0,18.3 L0,1.7"/> </g> <g
fill="none" stroke="#000000" stroke-opacity="1" stroke-width="1" stroke-linecap="square" stroke-linejoin="bevel"
transform="matrix(1,0,0,1,685.633,758)" font-family="MS Shell Dlg 2" font-size="9.75" font-weight="400" font-
style="normal" > <text fill="#000000" fill-opacity="1" stroke="none" xml:space="preserve" x="4" y="17" font-
family="Arial" font-size="10pt" font-weight="700" font-style="italic" >Q</text> </g> <g fill="#ff9999"
fill-opacity="1" stroke="#bfbfbf" stroke-opacity="1" stroke-width="1" stroke-linecap="square" stroke-
linejoin="bevel" transform="matrix(1,0,0,1,724,760)" font-family="MS Shell Dlg 2" font-size="9.75" font-
weight="400" font-style="normal" > <path vector-effect="none" fill-rule="evenodd" d="M0,1.7 L42.5,1.7 L42.5,0
L50,10 L42.5,20 L42.5,18.3 L0,18.3 L0,1.7"/> </g> <g fill="none" stroke="#000000" stroke-opacity="1"
stroke-width="1" stroke-linecap="square" stroke-linejoin="bevel" transform="matrix(1,0,0,1,737.133,758)" font-
family="MS Shell Dlg 2" font-size="9.75" font-weight="400" font-style="normal" > <text fill="#000000" fill-
opacity="1" stroke="none" xml:space="preserve" x="4" y="17" font-family="Arial" font-size="10pt" font-
weight="700" font-style="italic" >D</text> </g> <g fill="#99ffff" fill-opacity="1" stroke="#bfbfbf"
stroke-opacity="1" stroke-width="1" stroke-linecap="square" stroke-linejoin="bevel"
transform="matrix(1,0,0,1,775,760)" font-family="MS Shell Dlg 2" font-size="9.75" font-weight="400" font-
style="normal" > <path vector-effect="none" fill-rule="evenodd" d="M0,5 C0,2.23858 2.23858,0 5,0 L45,0
C47.7614,0 50,2.23858 50,5 L50,15 C50,17.7614 47.7614,20 45,20 L5,20 C2.23858,20 0,17.7614 0,15 L0,5"/>
</g> <g fill="none" stroke="#000000" stroke-opacity="1" stroke-width="1" stroke-linecap="square" stroke-
linejoin="bevel" transform="matrix(1,0,0,1,784.5,758)" font-family="MS Shell Dlg 2" font-size="9.75" font-
weight="400" font-style="normal" > <text fill="#000000" fill-opacity="1" stroke="none" xml:space="preserve"
x="4" y="17" font-family="Arial" font-size="10pt" font-weight="700" font-style="italic" >rrnL</text> </g>
<g fill="#ff9999" fill-opacity="1" stroke="#bfbfbf" stroke-opacity="1" stroke-width="1" stroke-linecap="square"
stroke-linejoin="bevel" transform="matrix(1,0,0,1,826,760)" font-family="MS Shell Dlg 2" font-size="9.75" font-
weight="400" font-style="normal" > <path vector-effect="none" fill-rule="evenodd" d="M0,1.7 L42.5,1.7 L42.5,0
L50,10 L42.5,20 L42.5,18.3 L0,18.3 L0,1.7"/> </g> <g fill="none" stroke="#000000" stroke-opacity="1"
stroke-width="1" stroke-linecap="square" stroke-linejoin="bevel" transform="matrix(1,0,0,1,839.633,758)" font-
family="MS Shell Dlg 2" font-size="9.75" font-weight="400" font-style="normal" > <text fill="#000000" fill-
opacity="1" stroke="none" xml:space="preserve" x="4" y="17" font-family="Arial" font-size="10pt" font-
weight="700" font-style="italic" >Y</text> </g> <g fill="#ffff33" fill-opacity="1" stroke="#bfbfbf"
stroke-opacity="1" stroke-width="1" stroke-linecap="square" stroke-linejoin="bevel"
transform="matrix(1,0,0,1,877,760)" font-family="MS Shell Dlg 2" font-size="9.75" font-weight="400" font-

style="normal" > <rect x="0" y="0" width="50" height="20"/> </g> <g fill="none" stroke="#000000" stroke-opacity="1" stroke-width="1" stroke-linecap="square" stroke-linejoin="bevel" transform="matrix(1,0,0,1,886.5,758)" font-family="MS Shell Dlg 2" font-size="9.75" font-weight="400" font-style="normal" > <text fill="#000000" fill-opacity="1" stroke="none" xml:space="preserve" x="4" y="17" font-family="Arial" font-size="10pt" font-weight="700" font-style="italic" >cytb</text> </g> <g fill="#ffff33" fill-opacity="1" stroke="#bfbfbf" stroke-opacity="1" stroke-width="1" stroke-linecap="square" stroke-linejoin="bevel" transform="matrix(1,0,0,1,928,760)" font-family="MS Shell Dlg 2" font-size="9.75" font-weight="400" font-style="normal" > <rect x="0" y="0" width="50" height="20"/> </g> <g fill="none" stroke="#000000" stroke-opacity="1" stroke-width="1" stroke-linecap="square" stroke-linejoin="bevel" transform="matrix(1,0,0,1,936,758)" font-family="MS Shell Dlg 2" font-size="9.75" font-weight="400" font-style="normal" > <text fill="#000000" fill-opacity="1" stroke="none" xml:space="preserve" x="4" y="17" font-family="Arial" font-size="10pt" font-weight="700" font-style="italic" >cox2</text> </g> <g fill="#ff9999" fill-opacity="1" stroke="#bfbfbf" stroke-opacity="1" stroke-width="1" stroke-linecap="square" stroke-linejoin="bevel" transform="matrix(1,0,0,1,979,760)" font-family="MS Shell Dlg 2" font-size="9.75" font-weight="400" font-style="normal" > <path vector-effect="none" fill-rule="evenodd" d="M0,1.7 L42.5,1.7 L42.5,0 L50,10 L42.5,20 L42.5,18.3 L0,18.3 L0,1.7"/> </g> <g fill="none" stroke="#000000" stroke-opacity="1" stroke-width="1" stroke-linecap="square" stroke-linejoin="bevel" transform="matrix(1,0,0,1,992.633,758)" font-family="MS Shell Dlg 2" font-size="9.75" font-weight="400" font-style="normal" > <text fill="#000000" fill-opacity="1" stroke="none" xml:space="preserve" x="4" y="17" font-family="Arial" font-size="10pt" font-weight="700" font-style="italic" >K</text> </g> <g fill="#ff9999" fill-opacity="1" stroke="#bfbfbf" stroke-opacity="1" stroke-width="1" stroke-linecap="square" stroke-linejoin="bevel" transform="matrix(1,0,0,1,1030,760)" font-family="MS Shell Dlg 2" font-size="9.75" font-weight="400" font-style="normal" > <path vector-effect="none" fill-rule="evenodd" d="M0,1.7 L42.5,1.7 L42.5,0 L50,10 L42.5,20 L42.5,18.3 L0,18.3 L0,1.7"/> </g> <g fill="none" stroke="#000000" stroke-opacity="1" stroke-width="1" stroke-linecap="square" stroke-linejoin="bevel" transform="matrix(1,0,0,1,1042.13,758)" font-family="MS Shell Dlg 2" font-size="9.75" font-weight="400" font-style="normal" > <text fill="#000000" fill-opacity="1" stroke="none" xml:space="preserve" x="4" y="17" font-family="Arial" font-size="10pt" font-weight="700" font-style="italic" >M</text> </g> <g fill="#ff9999" fill-opacity="1" stroke="#bfbfbf" stroke-opacity="1" stroke-width="1" stroke-linecap="square" stroke-linejoin="bevel" transform="matrix(1,0,0,1,1081,760)" font-family="MS Shell Dlg 2" font-size="9.75" font-weight="400" font-style="normal" > <path vector-effect="none" fill-rule="evenodd" d="M0,1.7 L42.5,1.7 L42.5,0 L50,10 L42.5,20 L42.5,18.3 L0,18.3 L0,1.7"/> </g> <g fill="none" stroke="#000000" stroke-opacity="1" stroke-width="1" stroke-linecap="square" stroke-linejoin="bevel" transform="matrix(1,0,0,1,1091.63,758)" font-family="MS Shell Dlg 2" font-size="9.75" font-weight="400" font-style="normal" > <text fill="#000000" fill-opacity="1" stroke="none" xml:space="preserve" x="4" y="17" font-family="Arial" font-size="10pt" font-weight="700" font-style="italic" >L1</text> </g> <g fill="#ff9999" fill-opacity="1" stroke="#bfbfbf" stroke-opacity="1" stroke-width="1" stroke-linecap="square" stroke-linejoin="bevel" transform="matrix(1,0,0,1,1132,760)" font-family="MS Shell Dlg 2" font-size="9.75" font-weight="400" font-style="normal" > <path vector-effect="none" fill-rule="evenodd" d="M0,1.7 L42.5,1.7 L42.5,0 L50,10 L42.5,20 L42.5,18.3 L0,18.3 L0,1.7"/> </g> <g fill="none" stroke="#000000" stroke-opacity="1" stroke-width="1" stroke-linecap="square" stroke-linejoin="bevel" transform="matrix(1,0,0,1,1142.63,758)" font-family="MS Shell Dlg 2" font-size="9.75" font-weight="400" font-style="normal" > <text fill="#000000" fill-opacity="1" stroke="none" xml:space="preserve" x="4" y="17" font-family="Arial" font-size="10pt" font-weight="700" font-style="italic" >L2</text> </g> <g fill="#ffff33" fill-opacity="1" stroke="#bfbfbf" stroke-opacity="1" stroke-width="1" stroke-linecap="square" stroke-linejoin="bevel" transform="matrix(1,0,0,1,1183,760)" font-family="MS Shell Dlg 2" font-size="9.75" font-weight="400" font-style="normal" > <rect x="0" y="0" width="50" height="20"/> </g> <g fill="none" stroke="#000000" stroke-opacity="1" stroke-width="1" stroke-linecap="square" stroke-linejoin="bevel" transform="matrix(1,0,0,1,1190,758)" font-family="MS Shell Dlg 2" font-size="9.75" font-weight="400" font-style="normal" > <text fill="#000000" fill-opacity="1" stroke="none" xml:space="preserve" x="4" y="17" font-family="Arial" font-size="10pt" font-weight="700" font-style="italic" >nad1</text> </g> <g fill="#ff9999" fill-opacity="1" stroke="#bfbfbf" stroke-opacity="1" stroke-width="1" stroke-linecap="square" stroke-linejoin="bevel" transform="matrix(1,0,0,1,1234,760)" font-family="MS Shell Dlg 2" font-size="9.75" font-weight="400" font-style="normal" > <path vector-effect="none" fill-rule="evenodd" d="M0,1.7 L42.5,1.7 L42.5,0 L50,10 L42.5,20 L42.5,18.3 L0,18.3 L0,1.7"/> </g> <g fill="none" stroke="#000000" stroke-opacity="1" stroke-width="1" stroke-linecap="square" stroke-linejoin="bevel" transform="matrix(1,0,0,1,1247.13,758)" font-family="MS Shell Dlg 2" font-size="9.75" font-weight="400" font-style="normal" > <text fill="#000000" fill-opacity="1" stroke="none" xml:space="preserve" x="4" y="17" font-family="Arial" font-size="10pt" font-weight="700" font-style="italic" >V</text> </g> <g fill="#ffff33" fill-opacity="1" stroke="#bfbfbf"

stroke-opacity="1" stroke-width="1" stroke-linecap="square" stroke-linejoin="bevel" transform="matrix(1,0,0,1,1285,760)" font-family="MS Shell Dlg 2" font-size="9.75" font-weight="400" font-style="normal" > <rect x="0" y="0" width="50" height="20"/> </g> <g fill="none" stroke="#000000" stroke-opacity="1" stroke-width="1" stroke-linecap="square" stroke-linejoin="bevel" transform="matrix(1,0,0,1,1292,758)" font-family="MS Shell Dlg 2" font-size="9.75" font-weight="400" font-style="normal" > <text fill="#000000" fill-opacity="1" stroke="none" xml:space="preserve" x="4" y="17" font-family="Arial" font-size="10pt" font-weight="700" font-style="italic" >nad4</text> </g> <g fill="#ffff33" fill-opacity="1" stroke="#bfbfbf" stroke-opacity="1" stroke-width="1" stroke-linecap="square" stroke-linejoin="bevel" transform="matrix(1,0,0,1,1336,760)" font-family="MS Shell Dlg 2" font-size="9.75" font-weight="400" font-style="normal" > <rect x="0" y="0" width="50" height="20"/> </g> <g fill="none" stroke="#000000" stroke-opacity="1" stroke-width="1" stroke-linecap="square" stroke-linejoin="bevel" transform="matrix(1,0,0,1,1344,758)" font-family="MS Shell Dlg 2" font-size="9.75" font-weight="400" font-style="normal" > <text fill="#000000" fill-opacity="1" stroke="none" xml:space="preserve" x="4" y="17" font-family="Arial" font-size="10pt" font-weight="700" font-style="italic" >cox3</text> </g> <g fill="#ff9999" fill-opacity="1" stroke="#bfbfbf" stroke-opacity="1" stroke-width="1" stroke-linecap="square" stroke-linejoin="bevel" transform="matrix(1,0,0,1,1387,760)" font-family="MS Shell Dlg 2" font-size="9.75" font-weight="400" font-style="normal" > <path vector-effect="none" fill-rule="evenodd" d="M0,1.7 L42.5,1.7 L42.5,0 L50,10 L42.5,20 L42.5,18.3 L0,18.3 L0,1.7"/> </g> <g fill="none" stroke="#000000" stroke-opacity="1" stroke-width="1" stroke-linecap="square" stroke-linejoin="bevel" transform="matrix(1,0,0,1,1400.13,758)" font-family="MS Shell Dlg 2" font-size="9.75" font-weight="400" font-style="normal" > <text fill="#000000" fill-opacity="1" stroke="none" xml:space="preserve" x="4" y="17" font-family="Arial" font-size="10pt" font-weight="700" font-style="italic" >S</text> </g> <g fill="#ff9999" fill-opacity="1" stroke="#bfbfbf" stroke-opacity="1" stroke-width="1" stroke-linecap="square" stroke-linejoin="bevel" transform="matrix(1,0,0,1,1438,760)" font-family="MS Shell Dlg 2" font-size="9.75" font-weight="400" font-style="normal" > <path vector-effect="none" fill-rule="evenodd" d="M0,1.7 L42.5,1.7 L42.5,0 L50,10 L42.5,20 L42.5,18.3 L0,18.3 L0,1.7"/> </g> <g fill="none" stroke="#000000" stroke-opacity="1" stroke-width="1" stroke-linecap="square" stroke-linejoin="bevel" transform="matrix(1,0,0,1,1450.13,758)" font-family="MS Shell Dlg 2" font-size="9.75" font-weight="400" font-style="normal" > <text fill="#000000" fill-opacity="1" stroke="none" xml:space="preserve" x="4" y="17" font-family="Arial" font-size="10pt" font-weight="700" font-style="italic" >M</text> </g> <g fill="#ffff33" fill-opacity="1" stroke="#bfbfbf" stroke-opacity="1" stroke-width="1" stroke-linecap="square" stroke-linejoin="bevel" transform="matrix(1,0,0,1,1489,760)" font-family="MS Shell Dlg 2" font-size="9.75" font-weight="400" font-style="normal" > <rect x="0" y="0" width="50" height="20"/> </g> <g fill="none" stroke="#000000" stroke-opacity="1" stroke-width="1" stroke-linecap="square" stroke-linejoin="bevel" transform="matrix(1,0,0,1,1496,758)" font-family="MS Shell Dlg 2" font-size="9.75" font-weight="400" font-style="normal" > <text fill="#000000" fill-opacity="1" stroke="none" xml:space="preserve" x="4" y="17" font-family="Arial" font-size="10pt" font-weight="700" font-style="italic" >nad2</text> </g> <g fill="#ff9999" fill-opacity="1" stroke="#bfbfbf" stroke-opacity="1" stroke-width="1" stroke-linecap="square" stroke-linejoin="bevel" transform="matrix(1,0,0,1,1540,760)" font-family="MS Shell Dlg 2" font-size="9.75" font-weight="400" font-style="normal" > <path vector-effect="none" fill-rule="evenodd" d="M0,1.7 L42.5,1.7 L42.5,0 L50,10 L42.5,20 L42.5,18.3 L0,18.3 L0,1.7"/> </g> <g fill="none" stroke="#000000" stroke-opacity="1" stroke-width="1" stroke-linecap="square" stroke-linejoin="bevel" transform="matrix(1,0,0,1,1553.13,758)" font-family="MS Shell Dlg 2" font-size="9.75" font-weight="400" font-style="normal" > <text fill="#000000" fill-opacity="1" stroke="none" xml:space="preserve" x="4" y="17" font-family="Arial" font-size="10pt" font-weight="700" font-style="italic" >R</text> </g> <g fill="#ff9999" fill-opacity="1" stroke="#bfbfbf" stroke-opacity="1" stroke-width="1" stroke-linecap="square" stroke-linejoin="bevel" transform="matrix(1,0,0,1,1591,760)" font-family="MS Shell Dlg 2" font-size="9.75" font-weight="400" font-style="normal" > <path vector-effect="none" fill-rule="evenodd" d="M0,1.7 L42.5,1.7 L42.5,0 L50,10 L42.5,20 L42.5,18.3 L0,18.3 L0,1.7"/> </g> <g fill="none" stroke="#000000" stroke-opacity="1" stroke-width="1" stroke-linecap="square" stroke-linejoin="bevel" transform="matrix(1,0,0,1,1602.13,758)" font-family="MS Shell Dlg 2" font-size="9.75" font-weight="400" font-style="normal" > <text fill="#000000" fill-opacity="1" stroke="none" xml:space="preserve" x="4" y="17" font-family="Arial" font-size="10pt" font-weight="700" font-style="italic" >W</text> </g> <g fill="#ff9999" fill-opacity="1" stroke="#bfbfbf" stroke-opacity="1" stroke-width="1" stroke-linecap="square" stroke-linejoin="bevel" transform="matrix(1,0,0,1,1642,760)" font-family="MS Shell Dlg 2" font-size="9.75" font-weight="400" font-style="normal" > <path vector-effect="none" fill-rule="evenodd" d="M0,1.7 L42.5,1.7 L42.5,0 L50,10 L42.5,20 L42.5,18.3 L0,18.3 L0,1.7"/> </g> <g fill="none" stroke="#000000" stroke-opacity="1" stroke-width="1" stroke-linecap="square" stroke-linejoin="bevel" transform="matrix(1,0,0,1,1655.13,758)" font-family="MS Shell Dlg 2" font-size="9.75" font-weight="400" font-style="normal" > <text fill="#000000" fill-opacity="1"

stroke="none" xml:space="preserve" x="4" y="17" font-family="Arial" font-size="10pt" font-weight="700" font-style="italic"   >A</text> </g>   <g fill="#ff9999" fill-opacity="1" stroke="#bfbfbf" stroke-opacity="1" stroke-width="1" stroke-linecap="square" stroke-linejoin="bevel" transform="matrix(1,0,0,1,1693,760)" font-family="MS Shell Dlg 2" font-size="9.75" font-weight="400" font-style="normal" > <path vector-effect="none" fill-rule="evenodd" d="M0,1.7 L42.5,1.7 L42.5,0 L50,10 L42.5,20 L42.5,18.3 L0,18.3 L0,1.7"/> </g>   <g fill="none" stroke="#000000" stroke-opacity="1" stroke-width="1" stroke-linecap="square" stroke-linejoin="bevel" transform="matrix(1,0,0,1,1706.13,758)" font-family="MS Shell Dlg 2" font-size="9.75" font-weight="400" font-style="normal" > <text fill="#000000" fill-opacity="1" stroke="none" xml:space="preserve" x="4" y="17" font-family="Arial" font-size="10pt" font-weight="700" font-style="italic"   >S</text> </g>   <g fill="#ff9999" fill-opacity="1" stroke="#bfbfbf" stroke-opacity="1" stroke-width="1" stroke-linecap="square" stroke-linejoin="bevel" transform="matrix(1,0,0,1,1744,760)" font-family="MS Shell Dlg 2" font-size="9.75" font-weight="400" font-style="normal" > <path vector-effect="none" fill-rule="evenodd" d="M0,1.7 L42.5,1.7 L42.5,0 L50,10 L42.5,20 L42.5,18.3 L0,18.3 L0,1.7"/> </g>   <g fill="none" stroke="#000000" stroke-opacity="1" stroke-width="1" stroke-linecap="square" stroke-linejoin="bevel" transform="matrix(1,0,0,1,1757.13,758)" font-family="MS Shell Dlg 2" font-size="9.75" font-weight="400" font-style="normal" > <text fill="#000000" fill-opacity="1" stroke="none" xml:space="preserve" x="4" y="17" font-family="Arial" font-size="10pt" font-weight="700" font-style="italic"   >H</text> </g>   <g fill="#ff9999" fill-opacity="1" stroke="#bfbfbf" stroke-opacity="1" stroke-width="1" stroke-linecap="square" stroke-linejoin="bevel" transform="matrix(1,0,0,1,1795,760)" font-family="MS Shell Dlg 2" font-size="9.75" font-weight="400" font-style="normal" > <path vector-effect="none" fill-rule="evenodd" d="M0,1.7 L42.5,1.7 L42.5,0 L50,10 L42.5,20 L42.5,18.3 L0,18.3 L0,1.7"/> </g>   <g fill="none" stroke="#000000" stroke-opacity="1" stroke-width="1" stroke-linecap="square" stroke-linejoin="bevel" transform="matrix(1,0,0,1,1808.63,758)" font-family="MS Shell Dlg 2" font-size="9.75" font-weight="400" font-style="normal" > <text fill="#000000" fill-opacity="1" stroke="none" xml:space="preserve" x="4" y="17" font-family="Arial" font-size="10pt" font-weight="700" font-style="italic"   >P</text> </g>   <g fill="#ffff33" fill-opacity="1" stroke="#bfbfbf" stroke-opacity="1" stroke-width="1" stroke-linecap="square" stroke-linejoin="bevel" transform="matrix(1,0,0,1,1846,760)" font-family="MS Shell Dlg 2" font-size="9.75" font-weight="400" font-style="normal" > <rect x="0" y="0" width="50" height="20"/> </g>   <g fill="none" stroke="#000000" stroke-opacity="1" stroke-width="1" stroke-linecap="square" stroke-linejoin="bevel" transform="matrix(1,0,0,1,1853,758)" font-family="MS Shell Dlg 2" font-size="9.75" font-weight="400" font-style="normal" > <text fill="#000000" fill-opacity="1" stroke="none" xml:space="preserve" x="4" y="17" font-family="Arial" font-size="10pt" font-weight="700" font-style="italic" >nad3</text> </g>   <g fill="none" stroke="#000000" stroke-opacity="1" stroke-width="1" stroke-linecap="square" stroke-linejoin="bevel" transform="matrix(1,0,0,1,1897,760)" font-family="MS Shell Dlg 2" font-size="9.75" font-weight="400" font-style="normal" > <text fill="#000000" fill-opacity="1" stroke="none" xml:space="preserve" x="4" y="17" font-family="Arial" font-size="10pt" font-weight="700" font-style="italic" >(+)</text> </g>   <g fill="none" stroke="#000000" stroke-opacity="1" stroke-width="1" stroke-linecap="square" stroke-linejoin="bevel" transform="matrix(1,0,0,1,10,800)" font-family="MS Shell Dlg 2" font-size="9.75" font-weight="400" font-style="normal" > <text fill="#000000" fill-opacity="1" stroke="none" xml:space="preserve" x="4" y="17" font-family="Arial" font-size="10pt" font-weight="700" font-style="italic" >Crenomytilus_grayanus_NC_044128</text> </g>   <g fill="#ffff33" fill-opacity="1" stroke="#bfbfbf" stroke-opacity="1" stroke-width="1" stroke-linecap="square" stroke-linejoin="bevel" transform="matrix(1,0,0,1,10,830)" font-family="MS Shell Dlg 2" font-size="9.75" font-weight="400" font-style="normal" > <rect x="0" y="0" width="50" height="20"/> </g>   <g fill="none" stroke="#000000" stroke-opacity="1" stroke-width="1" stroke-linecap="square" stroke-linejoin="bevel" transform="matrix(1,0,0,1,18,828)" font-family="MS Shell Dlg 2" font-size="9.75" font-weight="400" font-style="normal" > <text fill="#000000" fill-opacity="1" stroke="none" xml:space="preserve" x="4" y="17" font-family="Arial" font-size="10pt" font-weight="700" font-style="italic"   >cox1</text> </g>   <g fill="#ffff33" fill-opacity="1" stroke="#bfbfbf" stroke-opacity="1" stroke-width="1" stroke-linecap="square" stroke-linejoin="bevel" transform="matrix(1,0,0,1,61,830)" font-family="MS Shell Dlg 2" font-size="9.75" font-weight="400" font-style="normal" > <rect x="0" y="0" width="50" height="20"/> </g>   <g fill="none" stroke="#000000" stroke-opacity="1" stroke-width="1" stroke-linecap="square" stroke-linejoin="bevel" transform="matrix(1,0,0,1,69.5,828)" font-family="MS Shell Dlg 2" font-size="9.75" font-weight="400" font-style="normal" > <text fill="#000000" fill-opacity="1" stroke="none" xml:space="preserve" x="4" y="17" font-family="Arial" font-size="10pt" font-weight="700" font-style="italic"   >atp6</text> </g>   <g fill="#ff9999" fill-opacity="1" stroke="#bfbfbf" stroke-opacity="1" stroke-width="1" stroke-linecap="square" stroke-linejoin="bevel" transform="matrix(1,0,0,1,112,830)" font-family="MS Shell Dlg 2" font-size="9.75" font-weight="400" font-style="normal" > <path vector-effect="none" fill-rule="evenodd" d="M0,1.7 L42.5,1.7 L42.5,0 L50,10 L42.5,20 L42.5,18.3 L0,18.3 L0,1.7"/> </g>   <g fill="none" stroke="#000000" stroke-opacity="1"

stroke-width="1" stroke-linecap="square" stroke-linejoin="bevel" transform="matrix(1,0,0,1,125.132,828)" font-family="MS Shell Dlg 2" font-size="9.75" font-weight="400" font-style="normal" > <text fill="#000000" fill-opacity="1" stroke="none" xml:space="preserve" x="4" y="17" font-family="Arial" font-size="10pt" font-weight="700" font-style="italic" >T</text> </g> <g fill="#ffff33" fill-opacity="1" stroke="#bfbfbf" stroke-opacity="1" stroke-width="1" stroke-linecap="square" stroke-linejoin="bevel" transform="matrix(1,0,0,1,163,830)" font-family="MS Shell Dlg 2" font-size="9.75" font-weight="400" font-style="normal" > <rect x="0" y="0" width="50" height="20"/> </g> <g fill="none" stroke="#000000" stroke-opacity="1" stroke-width="1" stroke-linecap="square" stroke-linejoin="bevel" transform="matrix(1,0,0,1,167,828)" font-family="MS Shell Dlg 2" font-size="9.75" font-weight="400" font-style="normal" > <text fill="#000000" fill-opacity="1" stroke="none" xml:space="preserve" x="4" y="17" font-family="Arial" font-size="10pt" font-weight="700" font-style="italic" >nad4L</text> </g> <g fill="#ffff33" fill-opacity="1" stroke="#bfbfbf" stroke-opacity="1" stroke-width="1" stroke-linecap="square" stroke-linejoin="bevel" transform="matrix(1,0,0,1,214,830)" font-family="MS Shell Dlg 2" font-size="9.75" font-weight="400" font-style="normal" > <rect x="0" y="0" width="50" height="20"/> </g> <g fill="none" stroke="#000000" stroke-opacity="1" stroke-width="1" stroke-linecap="square" stroke-linejoin="bevel" transform="matrix(1,0,0,1,221,828)" font-family="MS Shell Dlg 2" font-size="9.75" font-weight="400" font-style="normal" > <text fill="#000000" fill-opacity="1" stroke="none" xml:space="preserve" x="4" y="17" font-family="Arial" font-size="10pt" font-weight="700" font-style="italic" >nad5</text> </g> <g fill="#ffff33" fill-opacity="1" stroke="#bfbfbf" stroke-opacity="1" stroke-width="1" stroke-linecap="square" stroke-linejoin="bevel" transform="matrix(1,0,0,1,265,830)" font-family="MS Shell Dlg 2" font-size="9.75" font-weight="400" font-style="normal" > <rect x="0" y="0" width="50" height="20"/> </g> <g fill="none" stroke="#000000" stroke-opacity="1" stroke-width="1" stroke-linecap="square" stroke-linejoin="bevel" transform="matrix(1,0,0,1,272,828)" font-family="MS Shell Dlg 2" font-size="9.75" font-weight="400" font-style="normal" > <text fill="#000000" fill-opacity="1" stroke="none" xml:space="preserve" x="4" y="17" font-family="Arial" font-size="10pt" font-weight="700" font-style="italic" >nad6</text> </g> <g fill="#ff9999" fill-opacity="1" stroke="#bfbfbf" stroke-opacity="1" stroke-width="1" stroke-linecap="square" stroke-linejoin="bevel" transform="matrix(1,0,0,1,316,830)" font-family="MS Shell Dlg 2" font-size="9.75" font-weight="400" font-style="normal" > <path vector-effect="none" fill-rule="evenodd" d="M0,1.7 L42.5,1.7 L42.5,0 L50,10 L42.5,20 L42.5,18.3 L0,18.3 L0,1.7"/> </g> <g fill="none" stroke="#000000" stroke-opacity="1" stroke-width="1" stroke-linecap="square" stroke-linejoin="bevel" transform="matrix(1,0,0,1,329.632,828)" font-family="MS Shell Dlg 2" font-size="9.75" font-weight="400" font-style="normal" > <text fill="#000000" fill-opacity="1" stroke="none" xml:space="preserve" x="4" y="17" font-family="Arial" font-size="10pt" font-weight="700" font-style="italic" >F</text> </g> <g fill="#99ffff" fill-opacity="1" stroke="#bfbfbf" stroke-opacity="1" stroke-width="1" stroke-linecap="square" stroke-linejoin="bevel" transform="matrix(1,0,0,1,367,830)" font-family="MS Shell Dlg 2" font-size="9.75" font-weight="400" font-style="normal" > <path vector-effect="none" fill-rule="evenodd" d="M0,5 C0,2.23858 2.23858,0 5,0 L45,0 C47.7614,0 50,2.23858 50,5 L50,15 C50,17.7614 47.7614,20 45,20 L5,20 C2.23858,20 0,17.7614 0,15 L0,5"/> </g> <g fill="none" stroke="#000000" stroke-opacity="1" stroke-width="1" stroke-linecap="square" stroke-linejoin="bevel" transform="matrix(1,0,0,1,375.5,828)" font-family="MS Shell Dlg 2" font-size="9.75" font-weight="400" font-style="normal" > <text fill="#000000" fill-opacity="1" stroke="none" xml:space="preserve" x="4" y="17" font-family="Arial" font-size="10pt" font-weight="700" font-style="italic" >rrnS</text> </g> <g fill="#ff9999" fill-opacity="1" stroke="#bfbfbf" stroke-opacity="1" stroke-width="1" stroke-linecap="square" stroke-linejoin="bevel" transform="matrix(1,0,0,1,418,830)" font-family="MS Shell Dlg 2" font-size="9.75" font-weight="400" font-style="normal" > <path vector-effect="none" fill-rule="evenodd" d="M0,1.7 L42.5,1.7 L42.5,0 L50,10 L42.5,20 L42.5,18.3 L0,18.3 L0,1.7"/> </g> <g fill="none" stroke="#000000" stroke-opacity="1" stroke-width="1" stroke-linecap="square" stroke-linejoin="bevel" transform="matrix(1,0,0,1,431.132,828)" font-family="MS Shell Dlg 2" font-size="9.75" font-weight="400" font-style="normal" > <text fill="#000000" fill-opacity="1" stroke="none" xml:space="preserve" x="4" y="17" font-family="Arial" font-size="10pt" font-weight="700" font-style="italic" >G</text> </g> <g fill="#ff9999" fill-opacity="1" stroke="#bfbfbf" stroke-opacity="1" stroke-width="1" stroke-linecap="square" stroke-linejoin="bevel" transform="matrix(1,0,0,1,469,830)" font-family="MS Shell Dlg 2" font-size="9.75" font-weight="400" font-style="normal" > <path vector-effect="none" fill-rule="evenodd" d="M0,1.7 L42.5,1.7 L42.5,0 L50,10 L42.5,20 L42.5,18.3 L0,18.3 L0,1.7"/> </g> <g fill="none" stroke="#000000" stroke-opacity="1" stroke-width="1" stroke-linecap="square" stroke-linejoin="bevel" transform="matrix(1,0,0,1,482.132,828)" font-family="MS Shell Dlg 2" font-size="9.75" font-weight="400" font-style="normal" > <text fill="#000000" fill-opacity="1" stroke="none" xml:space="preserve" x="4" y="17" font-family="Arial" font-size="10pt" font-weight="700" font-style="italic" >N</text> </g> <g fill="#ff9999" fill-opacity="1" stroke="#bfbfbf" stroke-opacity="1" stroke-width="1" stroke-linecap="square" stroke-linejoin="bevel" transform="matrix(1,0,0,1,520,830)" font-

family="MS Shell Dlg 2" font-size="9.75" font-weight="400" font-style="normal" > <path vector-effect="none" fill-rule="evenodd" d="M0,1.7 L42.5,1.7 L42.5,0 L50,10 L42.5,20 L42.5,18.3 L0,18.3 L0,1.7"/> </g> <g fill="none" stroke="#000000" stroke-opacity="1" stroke-width="1" stroke-linecap="square" stroke-linejoin="bevel" transform="matrix(1,0,0,1,533.633,828)" font-family="MS Shell Dlg 2" font-size="9.75" font-weight="400" font-style="normal" > <text fill="#000000" fill-opacity="1" stroke="none" xml:space="preserve" x="4" y="17" font-family="Arial" font-size="10pt" font-weight="700" font-style="italic" >E</text> </g> <g fill="#ff9999" fill-opacity="1" stroke="#bfbfbf" stroke-opacity="1" stroke-width="1" stroke-linecap="square" stroke-linejoin="bevel" transform="matrix(1,0,0,1,571,830)" font-family="MS Shell Dlg 2" font-size="9.75" font-weight="400" font-style="normal" > <path vector-effect="none" fill-rule="evenodd" d="M0,1.7 L42.5,1.7 L42.5,0 L50,10 L42.5,20 L42.5,18.3 L0,18.3 L0,1.7"/> </g> <g fill="none" stroke="#000000" stroke-opacity="1" stroke-width="1" stroke-linecap="square" stroke-linejoin="bevel" transform="matrix(1,0,0,1,584.133,828)" font-family="MS Shell Dlg 2" font-size="9.75" font-weight="400" font-style="normal" > <text fill="#000000" fill-opacity="1" stroke="none" xml:space="preserve" x="4" y="17" font-family="Arial" font-size="10pt" font-weight="700" font-style="italic" >C</text> </g> <g fill="#ff9999" fill-opacity="1" stroke="#bfbfbf" stroke-opacity="1" stroke-width="1" stroke-linecap="square" stroke-linejoin="bevel" transform="matrix(1,0,0,1,622,830)" font-family="MS Shell Dlg 2" font-size="9.75" font-weight="400" font-style="normal" > <path vector-effect="none" fill-rule="evenodd" d="M0,1.7 L42.5,1.7 L42.5,0 L50,10 L42.5,20 L42.5,18.3 L0,18.3 L0,1.7"/> </g> <g fill="none" stroke="#000000" stroke-opacity="1" stroke-width="1" stroke-linecap="square" stroke-linejoin="bevel" transform="matrix(1,0,0,1,637.133,828)" font-family="MS Shell Dlg 2" font-size="9.75" font-weight="400" font-style="normal" > <text fill="#000000" fill-opacity="1" stroke="none" xml:space="preserve" x="4" y="17" font-family="Arial" font-size="10pt" font-weight="700" font-style="italic" >I</text> </g> <g fill="#ff9999" fill-opacity="1" stroke="#bfbfbf" stroke-opacity="1" stroke-width="1" stroke-linecap="square" stroke-linejoin="bevel" transform="matrix(1,0,0,1,673,830)" font-family="MS Shell Dlg 2" font-size="9.75" font-weight="400" font-style="normal" > <path vector-effect="none" fill-rule="evenodd" d="M0,1.7 L42.5,1.7 L42.5,0 L50,10 L42.5,20 L42.5,18.3 L0,18.3 L0,1.7"/> </g> <g fill="none" stroke="#000000" stroke-opacity="1" stroke-width="1" stroke-linecap="square" stroke-linejoin="bevel" transform="matrix(1,0,0,1,685.633,828)" font-family="MS Shell Dlg 2" font-size="9.75" font-weight="400" font-style="normal" > <text fill="#000000" fill-opacity="1" stroke="none" xml:space="preserve" x="4" y="17" font-family="Arial" font-size="10pt" font-weight="700" font-style="italic" >Q</text> </g> <g fill="#ff9999" fill-opacity="1" stroke="#bfbfbf" stroke-opacity="1" stroke-width="1" stroke-linecap="square" stroke-linejoin="bevel" transform="matrix(1,0,0,1,724,830)" font-family="MS Shell Dlg 2" font-size="9.75" font-weight="400" font-style="normal" > <path vector-effect="none" fill-rule="evenodd" d="M0,1.7 L42.5,1.7 L42.5,0 L50,10 L42.5,20 L42.5,18.3 L0,18.3 L0,1.7"/> </g> <g fill="none" stroke="#000000" stroke-opacity="1" stroke-width="1" stroke-linecap="square" stroke-linejoin="bevel" transform="matrix(1,0,0,1,737.133,828)" font-family="MS Shell Dlg 2" font-size="9.75" font-weight="400" font-style="normal" > <text fill="#000000" fill-opacity="1" stroke="none" xml:space="preserve" x="4" y="17" font-family="Arial" font-size="10pt" font-weight="700" font-style="italic" >D</text> </g> <g fill="#99ffff" fill-opacity="1" stroke="#bfbfbf" stroke-opacity="1" stroke-width="1" stroke-linecap="square" stroke-linejoin="bevel" transform="matrix(1,0,0,1,775,830)" font-family="MS Shell Dlg 2" font-size="9.75" font-weight="400" font-style="normal" > <path vector-effect="none" fill-rule="evenodd" d="M0,5 C0,2.23858 2.23858,0 5,0 L45,0 C47.7614,0 50,2.23858 50,5 L50,15 C50,17.7614 47.7614,20 45,20 L5,20 C2.23858,20 0,17.7614 0,15 L0,5"/> </g> <g fill="none" stroke="#000000" stroke-opacity="1" stroke-width="1" stroke-linecap="square" stroke-linejoin="bevel" transform="matrix(1,0,0,1,784.5,828)" font-family="MS Shell Dlg 2" font-size="9.75" font-weight="400" font-style="normal" > <text fill="#000000" fill-opacity="1" stroke="none" xml:space="preserve" x="4" y="17" font-family="Arial" font-size="10pt" font-weight="700" font-style="italic" >rrnL</text> </g> <g fill="#ff9999" fill-opacity="1" stroke="#bfbfbf" stroke-opacity="1" stroke-width="1" stroke-linecap="square" stroke-linejoin="bevel" transform="matrix(1,0,0,1,826,830)" font-family="MS Shell Dlg 2" font-size="9.75" font-weight="400" font-style="normal" > <path vector-effect="none" fill-rule="evenodd" d="M0,1.7 L42.5,1.7 L42.5,0 L50,10 L42.5,20 L42.5,18.3 L0,18.3 L0,1.7"/> </g> <g fill="none" stroke="#000000" stroke-opacity="1" stroke-width="1" stroke-linecap="square" stroke-linejoin="bevel" transform="matrix(1,0,0,1,839.633,828)" font-family="MS Shell Dlg 2" font-size="9.75" font-weight="400" font-style="normal" > <text fill="#000000" fill-opacity="1" stroke="none" xml:space="preserve" x="4" y="17" font-family="Arial" font-size="10pt" font-weight="700" font-style="italic" >Y</text> </g> <g fill="#ffff33" fill-opacity="1" stroke="#bfbfbf" stroke-opacity="1" stroke-width="1" stroke-linecap="square" stroke-linejoin="bevel" transform="matrix(1,0,0,1,877,830)" font-family="MS Shell Dlg 2" font-size="9.75" font-weight="400" font-style="normal" > <rect x="0" y="0" width="50" height="20"/> </g> <g fill="none" stroke="#000000" stroke-opacity="1" stroke-width="1" stroke-linecap="square" stroke-linejoin="bevel" transform="matrix(1,0,0,1,886.5,828)" font-family="MS Shell Dlg 2" font-size="9.75" font-weight="400" font-

style="normal" > <text fill="#000000" fill-opacity="1" stroke="none" xml:space="preserve" x="4" y="17" font-
family="Arial" font-size="10pt" font-weight="700" font-style="italic"   >cytb</text> </g>   <g fill="#ffff33"
fill-opacity="1" stroke="#bfbfbf" stroke-opacity="1" stroke-width="1" stroke-linecap="square" stroke-
linejoin="bevel" transform="matrix(1,0,0,1,928,830)" font-family="MS Shell Dlg 2" font-size="9.75" font-
weight="400" font-style="normal" > <rect x="0" y="0" width="50" height="20"/> </g>   <g fill="none"
stroke="#000000" stroke-opacity="1" stroke-width="1" stroke-linecap="square" stroke-linejoin="bevel"
transform="matrix(1,0,0,1,936,828)" font-family="MS Shell Dlg 2" font-size="9.75" font-weight="400" font-
style="normal" > <text fill="#000000" fill-opacity="1" stroke="none" xml:space="preserve" x="4" y="17" font-
family="Arial" font-size="10pt" font-weight="700" font-style="italic"   >cox2</text> </g>   <g
fill="#ff9999" fill-opacity="1" stroke="#bfbfbf" stroke-opacity="1" stroke-width="1" stroke-linecap="square"
stroke-linejoin="bevel" transform="matrix(1,0,0,1,979,830)" font-family="MS Shell Dlg 2" font-size="9.75" font-
weight="400" font-style="normal" > <path vector-effect="none" fill-rule="evenodd" d="M0,1.7 L42.5,1.7 L42.5,0
L50,10 L42.5,20 L42.5,18.3 L0,18.3 L0,1.7"/> </g>   <g fill="none" stroke="#000000" stroke-opacity="1"
stroke-width="1" stroke-linecap="square" stroke-linejoin="bevel" transform="matrix(1,0,0,1,992.633,828)" font-
family="MS Shell Dlg 2" font-size="9.75" font-weight="400" font-style="normal" > <text fill="#000000" fill-
opacity="1" stroke="none" xml:space="preserve" x="4" y="17" font-family="Arial" font-size="10pt" font-
weight="700" font-style="italic"   >K</text> </g>   <g fill="#ff9999" fill-opacity="1" stroke="#bfbfbf"
stroke-opacity="1" stroke-width="1" stroke-linecap="square" stroke-linejoin="bevel"
transform="matrix(1,0,0,1,1030,830)" font-family="MS Shell Dlg 2" font-size="9.75" font-weight="400" font-
style="normal" > <path vector-effect="none" fill-rule="evenodd" d="M0,1.7 L42.5,1.7 L42.5,0 L50,10 L42.5,20
L42.5,18.3 L0,18.3 L0,1.7"/> </g>   <g fill="none" stroke="#000000" stroke-opacity="1" stroke-width="1"
stroke-linecap="square" stroke-linejoin="bevel" transform="matrix(1,0,0,1,1042.13,828)" font-family="MS Shell
Dlg 2" font-size="9.75" font-weight="400" font-style="normal" > <text fill="#000000" fill-opacity="1"
stroke="none" xml:space="preserve" x="4" y="17" font-family="Arial" font-size="10pt" font-weight="700" font-
style="italic"   >M</text> </g>   <g fill="#ff9999" fill-opacity="1" stroke="#bfbfbf" stroke-opacity="1"
stroke-width="1" stroke-linecap="square" stroke-linejoin="bevel" transform="matrix(1,0,0,1,1081,830)" font-
family="MS Shell Dlg 2" font-size="9.75" font-weight="400" font-style="normal" > <path vector-effect="none"
fill-rule="evenodd" d="M0,1.7 L42.5,1.7 L42.5,0 L50,10 L42.5,20 L42.5,18.3 L0,18.3 L0,1.7"/> </g>   <g
fill="none" stroke="#000000" stroke-opacity="1" stroke-width="1" stroke-linecap="square" stroke-linejoin="bevel"
transform="matrix(1,0,0,1,1091.63,828)" font-family="MS Shell Dlg 2" font-size="9.75" font-weight="400" font-
style="normal" > <text fill="#000000" fill-opacity="1" stroke="none" xml:space="preserve" x="4" y="17" font-
family="Arial" font-size="10pt" font-weight="700" font-style="italic"   >L1</text> </g>   <g fill="#ff9999"
fill-opacity="1" stroke="#bfbfbf" stroke-opacity="1" stroke-width="1" stroke-linecap="square" stroke-
linejoin="bevel" transform="matrix(1,0,0,1,1132,830)" font-family="MS Shell Dlg 2" font-size="9.75" font-
weight="400" font-style="normal" > <path vector-effect="none" fill-rule="evenodd" d="M0,1.7 L42.5,1.7 L42.5,0
L50,10 L42.5,20 L42.5,18.3 L0,18.3 L0,1.7"/> </g>   <g fill="none" stroke="#000000" stroke-opacity="1"
stroke-width="1" stroke-linecap="square" stroke-linejoin="bevel" transform="matrix(1,0,0,1,1142.63,828)" font-
family="MS Shell Dlg 2" font-size="9.75" font-weight="400" font-style="normal" > <text fill="#000000" fill-
opacity="1" stroke="none" xml:space="preserve" x="4" y="17" font-family="Arial" font-size="10pt" font-
weight="700" font-style="italic"   >L2</text> </g>   <g fill="#ffff33" fill-opacity="1" stroke="#bfbfbf"
stroke-opacity="1" stroke-width="1" stroke-linecap="square" stroke-linejoin="bevel"
transform="matrix(1,0,0,1,1183,830)" font-family="MS Shell Dlg 2" font-size="9.75" font-weight="400" font-
style="normal" > <rect x="0" y="0" width="50" height="20"/> </g>   <g fill="none" stroke="#000000" stroke-
opacity="1" stroke-width="1" stroke-linecap="square" stroke-linejoin="bevel"
transform="matrix(1,0,0,1,1190,828)" font-family="MS Shell Dlg 2" font-size="9.75" font-weight="400" font-
style="normal" > <text fill="#000000" fill-opacity="1" stroke="none" xml:space="preserve" x="4" y="17" font-
family="Arial" font-size="10pt" font-weight="700" font-style="italic"   >nad1</text> </g>   <g
fill="#ff9999" fill-opacity="1" stroke="#bfbfbf" stroke-opacity="1" stroke-width="1" stroke-linecap="square"
stroke-linejoin="bevel" transform="matrix(1,0,0,1,1234,830)" font-family="MS Shell Dlg 2" font-size="9.75" font-
weight="400" font-style="normal" > <path vector-effect="none" fill-rule="evenodd" d="M0,1.7 L42.5,1.7 L42.5,0
L50,10 L42.5,20 L42.5,18.3 L0,18.3 L0,1.7"/> </g>   <g fill="none" stroke="#000000" stroke-opacity="1"
stroke-width="1" stroke-linecap="square" stroke-linejoin="bevel" transform="matrix(1,0,0,1,1247.13,828)" font-
family="MS Shell Dlg 2" font-size="9.75" font-weight="400" font-style="normal" > <text fill="#000000" fill-
opacity="1" stroke="none" xml:space="preserve" x="4" y="17" font-family="Arial" font-size="10pt" font-
weight="700" font-style="italic"   >V</text> </g>   <g fill="#ffff33" fill-opacity="1" stroke="#bfbfbf"
stroke-opacity="1" stroke-width="1" stroke-linecap="square" stroke-linejoin="bevel"
transform="matrix(1,0,0,1,1285,830)" font-family="MS Shell Dlg 2" font-size="9.75" font-weight="400" font-
style="normal" > <rect x="0" y="0" width="50" height="20"/> </g>   <g fill="none" stroke="#000000" stroke-

opacity="1" stroke-width="1" stroke-linecap="square" stroke-linejoin="bevel"
transform="matrix(1,0,0,1,1292,828)" font-family="MS Shell Dlg 2" font-size="9.75" font-weight="400" font-
style="normal" > <text fill="#000000" fill-opacity="1" stroke="none" xml:space="preserve" x="4" y="17" font-
family="Arial" font-size="10pt" font-weight="700" font-style="italic" >nad4</text> </g> <g fill="#ffff33"
fill-opacity="1" stroke="#bfbfbf" stroke-opacity="1" stroke-width="1" stroke-linecap="square" stroke-
linejoin="bevel" transform="matrix(1,0,0,1,1336,830)" font-family="MS Shell Dlg 2" font-size="9.75" font-
weight="400" font-style="normal" > <rect x="0" y="0" width="50" height="20"/> </g> <g fill="none"
stroke="#000000" stroke-opacity="1" stroke-width="1" stroke-linecap="square" stroke-linejoin="bevel"
transform="matrix(1,0,0,1,1344,828)" font-family="MS Shell Dlg 2" font-size="9.75" font-weight="400" font-
style="normal" > <text fill="#000000" fill-opacity="1" stroke="none" xml:space="preserve" x="4" y="17" font-
family="Arial" font-size="10pt" font-weight="700" font-style="italic" >cox3</text> </g> <g
fill="#ff9999" fill-opacity="1" stroke="#bfbfbf" stroke-opacity="1" stroke-width="1" stroke-linecap="square"
stroke-linejoin="bevel" transform="matrix(1,0,0,1,1387,830)" font-family="MS Shell Dlg 2" font-size="9.75" font-
weight="400" font-style="normal" > <path vector-effect="none" fill-rule="evenodd" d="M0,1.7 L42.5,1.7 L42.5,0
L50,10 L42.5,20 L42.5,18.3 L0,18.3 L0,1.7"/> </g> <g fill="none" stroke="#000000" stroke-opacity="1"
stroke-width="1" stroke-linecap="square" stroke-linejoin="bevel" transform="matrix(1,0,0,1,1396.63,828)" font-
family="MS Shell Dlg 2" font-size="9.75" font-weight="400" font-style="normal" > <text fill="#000000" fill-
opacity="1" stroke="none" xml:space="preserve" x="4" y="17" font-family="Arial" font-size="10pt" font-
weight="700" font-style="italic" >S2</text> </g> <g fill="#ff9999" fill-opacity="1" stroke="#bfbfbf"
stroke-opacity="1" stroke-width="1" stroke-linecap="square" stroke-linejoin="bevel"
transform="matrix(1,0,0,1,1438,830)" font-family="MS Shell Dlg 2" font-size="9.75" font-weight="400" font-
style="normal" > <path vector-effect="none" fill-rule="evenodd" d="M0,1.7 L42.5,1.7 L42.5,0 L50,10 L42.5,20
L42.5,18.3 L0,18.3 L0,1.7"/> </g> <g fill="none" stroke="#000000" stroke-opacity="1" stroke-width="1"
stroke-linecap="square" stroke-linejoin="bevel" transform="matrix(1,0,0,1,1450.13,828)" font-family="MS Shell
Dlg 2" font-size="9.75" font-weight="400" font-style="normal" > <text fill="#000000" fill-opacity="1"
stroke="none" xml:space="preserve" x="4" y="17" font-family="Arial" font-size="10pt" font-weight="700" font-
style="italic" >M</text> </g> <g fill="#ffff33" fill-opacity="1" stroke="#bfbfbf" stroke-opacity="1"
stroke-width="1" stroke-linecap="square" stroke-linejoin="bevel" transform="matrix(1,0,0,1,1489,830)" font-
family="MS Shell Dlg 2" font-size="9.75" font-weight="400" font-style="normal" > <rect x="0" y="0" width="50"
height="20"/> </g> <g fill="none" stroke="#000000" stroke-opacity="1" stroke-width="1" stroke-
linecap="square" stroke-linejoin="bevel" transform="matrix(1,0,0,1,1496,828)" font-family="MS Shell Dlg 2" font-
size="9.75" font-weight="400" font-style="normal" > <text fill="#000000" fill-opacity="1" stroke="none"
xml:space="preserve" x="4" y="17" font-family="Arial" font-size="10pt" font-weight="700" font-style="italic"
>nad2</text> </g> <g fill="#ff9999" fill-opacity="1" stroke="#bfbfbf" stroke-opacity="1" stroke-width="1"
stroke-linecap="square" stroke-linejoin="bevel" transform="matrix(1,0,0,1,1540,830)" font-family="MS Shell Dlg
2" font-size="9.75" font-weight="400" font-style="normal" > <path vector-effect="none" fill-rule="evenodd"
d="M0,1.7 L42.5,1.7 L42.5,0 L50,10 L42.5,20 L42.5,18.3 L0,18.3 L0,1.7"/> </g> <g fill="none"
stroke="#000000" stroke-opacity="1" stroke-width="1" stroke-linecap="square" stroke-linejoin="bevel"
transform="matrix(1,0,0,1,1553.13,828)" font-family="MS Shell Dlg 2" font-size="9.75" font-weight="400" font-
style="normal" > <text fill="#000000" fill-opacity="1" stroke="none" xml:space="preserve" x="4" y="17" font-
family="Arial" font-size="10pt" font-weight="700" font-style="italic" >R</text> </g> <g fill="#ff9999"
fill-opacity="1" stroke="#bfbfbf" stroke-opacity="1" stroke-width="1" stroke-linecap="square" stroke-
linejoin="bevel" transform="matrix(1,0,0,1,1591,830)" font-family="MS Shell Dlg 2" font-size="9.75" font-
weight="400" font-style="normal" > <path vector-effect="none" fill-rule="evenodd" d="M0,1.7 L42.5,1.7 L42.5,0
L50,10 L42.5,20 L42.5,18.3 L0,18.3 L0,1.7"/> </g> <g fill="none" stroke="#000000" stroke-opacity="1"
stroke-width="1" stroke-linecap="square" stroke-linejoin="bevel" transform="matrix(1,0,0,1,1602.13,828)" font-
family="MS Shell Dlg 2" font-size="9.75" font-weight="400" font-style="normal" > <text fill="#000000" fill-
opacity="1" stroke="none" xml:space="preserve" x="4" y="17" font-family="Arial" font-size="10pt" font-
weight="700" font-style="italic" >W</text> </g> <g fill="#ff9999" fill-opacity="1" stroke="#bfbfbf"
stroke-opacity="1" stroke-width="1" stroke-linecap="square" stroke-linejoin="bevel"
transform="matrix(1,0,0,1,1642,830)" font-family="MS Shell Dlg 2" font-size="9.75" font-weight="400" font-
style="normal" > <path vector-effect="none" fill-rule="evenodd" d="M0,1.7 L42.5,1.7 L42.5,0 L50,10 L42.5,20
L42.5,18.3 L0,18.3 L0,1.7"/> </g> <g fill="none" stroke="#000000" stroke-opacity="1" stroke-width="1"
stroke-linecap="square" stroke-linejoin="bevel" transform="matrix(1,0,0,1,1655.13,828)" font-family="MS Shell
Dlg 2" font-size="9.75" font-weight="400" font-style="normal" > <text fill="#000000" fill-opacity="1"
stroke="none" xml:space="preserve" x="4" y="17" font-family="Arial" font-size="10pt" font-weight="700" font-
style="italic" >A</text> </g> <g fill="#ff9999" fill-opacity="1" stroke="#bfbfbf" stroke-opacity="1"
stroke-width="1" stroke-linecap="square" stroke-linejoin="bevel" transform="matrix(1,0,0,1,1693,830)" font-

family="MS Shell Dlg 2" font-size="9.75" font-weight="400" font-style="normal" > <path vector-effect="none" fill-rule="evenodd" d="M0,1.7 L42.5,1.7 L42.5,0 L50,10 L42.5,20 L42.5,18.3 L0,18.3 L0,1.7"/> </g> <g fill="none" stroke="#000000" stroke-opacity="1" stroke-width="1" stroke-linecap="square" stroke-linejoin="bevel" transform="matrix(1,0,0,1,1702.63,828)" font-family="MS Shell Dlg 2" font-size="9.75" font-weight="400" font-style="normal" > <text fill="#000000" fill-opacity="1" stroke="none" xml:space="preserve" x="4" y="17" font-family="Arial" font-size="10pt" font-weight="700" font-style="italic" >S1</text> </g> <g fill="#ff9999" fill-opacity="1" stroke="#bfbfbf" stroke-opacity="1" stroke-width="1" stroke-linecap="square" stroke-linejoin="bevel" transform="matrix(1,0,0,1,1744,830)" font-family="MS Shell Dlg 2" font-size="9.75" font-weight="400" font-style="normal" > <path vector-effect="none" fill-rule="evenodd" d="M0,1.7 L42.5,1.7 L42.5,0 L50,10 L42.5,20 L42.5,18.3 L0,18.3 L0,1.7"/> </g> <g fill="none" stroke="#000000" stroke-opacity="1" stroke-width="1" stroke-linecap="square" stroke-linejoin="bevel" transform="matrix(1,0,0,1,1757.13,828)" font-family="MS Shell Dlg 2" font-size="9.75" font-weight="400" font-style="normal" > <text fill="#000000" fill-opacity="1" stroke="none" xml:space="preserve" x="4" y="17" font-family="Arial" font-size="10pt" font-weight="700" font-style="italic" >H</text> </g> <g fill="#ff9999" fill-opacity="1" stroke="#bfbfbf" stroke-opacity="1" stroke-width="1" stroke-linecap="square" stroke-linejoin="bevel" transform="matrix(1,0,0,1,1795,830)" font-family="MS Shell Dlg 2" font-size="9.75" font-weight="400" font-style="normal" > <path vector-effect="none" fill-rule="evenodd" d="M0,1.7 L42.5,1.7 L42.5,0 L50,10 L42.5,20 L42.5,18.3 L0,18.3 L0,1.7"/> </g> <g fill="none" stroke="#000000" stroke-opacity="1" stroke-width="1" stroke-linecap="square" stroke-linejoin="bevel" transform="matrix(1,0,0,1,1808.63,828)" font-family="MS Shell Dlg 2" font-size="9.75" font-weight="400" font-style="normal" > <text fill="#000000" fill-opacity="1" stroke="none" xml:space="preserve" x="4" y="17" font-family="Arial" font-size="10pt" font-weight="700" font-style="italic" >P</text> </g> <g fill="#ffff33" fill-opacity="1" stroke="#bfbfbf" stroke-opacity="1" stroke-width="1" stroke-linecap="square" stroke-linejoin="bevel" transform="matrix(1,0,0,1,1846,830)" font-family="MS Shell Dlg 2" font-size="9.75" font-weight="400" font-style="normal" > <rect x="0" y="0" width="50" height="20"/> </g> <g fill="none" stroke="#000000" stroke-opacity="1" stroke-width="1" stroke-linecap="square" stroke-linejoin="bevel" transform="matrix(1,0,0,1,1853,828)" font-family="MS Shell Dlg 2" font-size="9.75" font-weight="400" font-style="normal" > <text fill="#000000" fill-opacity="1" stroke="none" xml:space="preserve" x="4" y="17" font-family="Arial" font-size="10pt" font-weight="700" font-style="italic" >nad3</text> </g> <g fill="#ffff33" fill-opacity="1" stroke="#bfbfbf" stroke-opacity="1" stroke-width="1" stroke-linecap="square" stroke-linejoin="bevel" transform="matrix(1,0,0,1,1897,830)" font-family="MS Shell Dlg 2" font-size="9.75" font-weight="400" font-style="normal" > <rect x="0" y="0" width="50" height="20"/> </g> <g fill="none" stroke="#000000" stroke-opacity="1" stroke-width="1" stroke-linecap="square" stroke-linejoin="bevel" transform="matrix(1,0,0,1,1905.5,828)" font-family="MS Shell Dlg 2" font-size="9.75" font-weight="400" font-style="normal" > <text fill="#000000" fill-opacity="1" stroke="none" xml:space="preserve" x="4" y="17" font-family="Arial" font-size="10pt" font-weight="700" font-style="italic" >atp8</text> </g> <g fill="none" stroke="#000000" stroke-opacity="1" stroke-width="1" stroke-linecap="square" stroke-linejoin="bevel" transform="matrix(1,0,0,1,1948,830)" font-family="MS Shell Dlg 2" font-size="9.75" font-weight="400" font-style="normal" > <text fill="#000000" fill-opacity="1" stroke="none" xml:space="preserve" x="4" y="17" font-family="Arial" font-size="10pt" font-weight="700" font-style="italic" >(+)</text> </g> <g fill="none" stroke="#000000" stroke-opacity="1" stroke-width="1" stroke-linecap="square" stroke-linejoin="bevel" transform="matrix(1,0,0,1,10,870)" font-family="MS Shell Dlg 2" font-size="9.75" font-weight="400" font-style="normal" > <text fill="#000000" fill-opacity="1" stroke="none" xml:space="preserve" x="4" y="17" font-family="Arial" font-size="10pt" font-weight="700" font-style="italic" >Gregariella_coralliophaga_NC_044129</text> </g> <g fill="#ffff33" fill-opacity="1" stroke="#bfbfbf" stroke-opacity="1" stroke-width="1" stroke-linecap="square" stroke-linejoin="bevel" transform="matrix(1,0,0,1,10,900)" font-family="MS Shell Dlg 2" font-size="9.75" font-weight="400" font-style="normal" > <rect x="0" y="0" width="50" height="20"/> </g> <g fill="none" stroke="#000000" stroke-opacity="1" stroke-width="1" stroke-linecap="square" stroke-linejoin="bevel" transform="matrix(1,0,0,1,18,898)" font-family="MS Shell Dlg 2" font-size="9.75" font-weight="400" font-style="normal" > <text fill="#000000" fill-opacity="1" stroke="none" xml:space="preserve" x="4" y="17" font-family="Arial" font-size="10pt" font-weight="700" font-style="italic" >cox1</text> </g> <g fill="#ffff33" fill-opacity="1" stroke="#bfbfbf" stroke-opacity="1" stroke-width="1" stroke-linecap="square" stroke-linejoin="bevel" transform="matrix(1,0,0,1,61,900)" font-family="MS Shell Dlg 2" font-size="9.75" font-weight="400" font-style="normal" > <rect x="0" y="0" width="50" height="20"/> </g> <g fill="none" stroke="#000000" stroke-opacity="1" stroke-width="1" stroke-linecap="square" stroke-linejoin="bevel" transform="matrix(1,0,0,1,69.5,898)" font-family="MS Shell Dlg 2" font-size="9.75" font-weight="400" font-style="normal" > <text fill="#000000" fill-opacity="1" stroke="none" xml:space="preserve" x="4" y="17" font-family="Arial" font-size="10pt" font-weight="700" font-style="italic" >atp6</text> </g> <g

fill="#ff9999" fill-opacity="1" stroke="#bfbfbf" stroke-opacity="1" stroke-width="1" stroke-linecap="square" stroke-linejoin="bevel" transform="matrix(1,0,0,1,112,900)" font-family="MS Shell Dlg 2" font-size="9.75" font-weight="400" font-style="normal" > <path vector-effect="none" fill-rule="evenodd" d="M0,1.7 L42.5,1.7 L42.5,0 L50,10 L42.5,20 L42.5,18.3 L0,18.3 L0,1.7"/> </g> <g fill="none" stroke="#000000" stroke-opacity="1" stroke-width="1" stroke-linecap="square" stroke-linejoin="bevel" transform="matrix(1,0,0,1,125.132,898)" font-family="MS Shell Dlg 2" font-size="9.75" font-weight="400" font-style="normal" > <text fill="#000000" fill-opacity="1" stroke="none" xml:space="preserve" x="4" y="17" font-family="Arial" font-size="10pt" font-weight="700" font-style="italic" >T</text> </g> <g fill="#ff9999" fill-opacity="1" stroke="#bfbfbf" stroke-opacity="1" stroke-width="1" stroke-linecap="square" stroke-linejoin="bevel" transform="matrix(1,0,0,1,163,900)" font-family="MS Shell Dlg 2" font-size="9.75" font-weight="400" font-style="normal" > <path vector-effect="none" fill-rule="evenodd" d="M0,1.7 L42.5,1.7 L42.5,0 L50,10 L42.5,20 L42.5,18.3 L0,18.3 L0,1.7"/> </g> <g fill="none" stroke="#000000" stroke-opacity="1" stroke-width="1" stroke-linecap="square" stroke-linejoin="bevel" transform="matrix(1,0,0,1,175.132,898)" font-family="MS Shell Dlg 2" font-size="9.75" font-weight="400" font-style="normal" > <text fill="#000000" fill-opacity="1" stroke="none" xml:space="preserve" x="4" y="17" font-family="Arial" font-size="10pt" font-weight="700" font-style="italic" >M</text> </g> <g fill="#ffff33" fill-opacity="1" stroke="#bfbfbf" stroke-opacity="1" stroke-width="1" stroke-linecap="square" stroke-linejoin="bevel" transform="matrix(1,0,0,1,214,900)" font-family="MS Shell Dlg 2" font-size="9.75" font-weight="400" font-style="normal" > <rect x="0" y="0" width="50" height="20"/> </g> <g fill="none" stroke="#000000" stroke-opacity="1" stroke-width="1" stroke-linecap="square" stroke-linejoin="bevel" transform="matrix(1,0,0,1,218,898)" font-family="MS Shell Dlg 2" font-size="9.75" font-weight="400" font-style="normal" > <text fill="#000000" fill-opacity="1" stroke="none" xml:space="preserve" x="4" y="17" font-family="Arial" font-size="10pt" font-weight="700" font-style="italic" >nad4L</text> </g> <g fill="#ffff33" fill-opacity="1" stroke="#bfbfbf" stroke-opacity="1" stroke-width="1" stroke-linecap="square" stroke-linejoin="bevel" transform="matrix(1,0,0,1,265,900)" font-family="MS Shell Dlg 2" font-size="9.75" font-weight="400" font-style="normal" > <rect x="0" y="0" width="50" height="20"/> </g> <g fill="none" stroke="#000000" stroke-opacity="1" stroke-width="1" stroke-linecap="square" stroke-linejoin="bevel" transform="matrix(1,0,0,1,272,898)" font-family="MS Shell Dlg 2" font-size="9.75" font-weight="400" font-style="normal" > <text fill="#000000" fill-opacity="1" stroke="none" xml:space="preserve" x="4" y="17" font-family="Arial" font-size="10pt" font-weight="700" font-style="italic" >nad5</text> </g> <g fill="#ffff33" fill-opacity="1" stroke="#bfbfbf" stroke-opacity="1" stroke-width="1" stroke-linecap="square" stroke-linejoin="bevel" transform="matrix(1,0,0,1,316,900)" font-family="MS Shell Dlg 2" font-size="9.75" font-weight="400" font-style="normal" > <rect x="0" y="0" width="50" height="20"/> </g> <g fill="none" stroke="#000000" stroke-opacity="1" stroke-width="1" stroke-linecap="square" stroke-linejoin="bevel" transform="matrix(1,0,0,1,323,898)" font-family="MS Shell Dlg 2" font-size="9.75" font-weight="400" font-style="normal" > <text fill="#000000" fill-opacity="1" stroke="none" xml:space="preserve" x="4" y="17" font-family="Arial" font-size="10pt" font-weight="700" font-style="italic" >nad6</text> </g> <g fill="#ff9999" fill-opacity="1" stroke="#bfbfbf" stroke-opacity="1" stroke-width="1" stroke-linecap="square" stroke-linejoin="bevel" transform="matrix(1,0,0,1,367,900)" font-family="MS Shell Dlg 2" font-size="9.75" font-weight="400" font-style="normal" > <path vector-effect="none" fill-rule="evenodd" d="M0,1.7 L42.5,1.7 L42.5,0 L50,10 L42.5,20 L42.5,18.3 L0,18.3 L0,1.7"/> </g> <g fill="none" stroke="#000000" stroke-opacity="1" stroke-width="1" stroke-linecap="square" stroke-linejoin="bevel" transform="matrix(1,0,0,1,380.632,898)" font-family="MS Shell Dlg 2" font-size="9.75" font-weight="400" font-style="normal" > <text fill="#000000" fill-opacity="1" stroke="none" xml:space="preserve" x="4" y="17" font-family="Arial" font-size="10pt" font-weight="700" font-style="italic" >P</text> </g> <g fill="#ff9999" fill-opacity="1" stroke="#bfbfbf" stroke-opacity="1" stroke-width="1" stroke-linecap="square" stroke-linejoin="bevel" transform="matrix(1,0,0,1,418,900)" font-family="MS Shell Dlg 2" font-size="9.75" font-weight="400" font-style="normal" > <path vector-effect="none" fill-rule="evenodd" d="M0,1.7 L42.5,1.7 L42.5,0 L50,10 L42.5,20 L42.5,18.3 L0,18.3 L0,1.7"/> </g> <g fill="none" stroke="#000000" stroke-opacity="1" stroke-width="1" stroke-linecap="square" stroke-linejoin="bevel" transform="matrix(1,0,0,1,431.632,898)" font-family="MS Shell Dlg 2" font-size="9.75" font-weight="400" font-style="normal" > <text fill="#000000" fill-opacity="1" stroke="none" xml:space="preserve" x="4" y="17" font-family="Arial" font-size="10pt" font-weight="700" font-style="italic" >F</text> </g> <g fill="#99ffff" fill-opacity="1" stroke="#bfbfbf" stroke-opacity="1" stroke-width="1" stroke-linecap="square" stroke-linejoin="bevel" transform="matrix(1,0,0,1,469,900)" font-family="MS Shell Dlg 2" font-size="9.75" font-weight="400" font-style="normal" > <path vector-effect="none" fill-rule="evenodd" d="M0,5 C0,2.23858 2.23858,0 5,0 L45,0 C47.7614,0 50,2.23858 50,5 L50,15 C50,17.7614 47.7614,20 45,20 L5,20 C2.23858,20 0,17.7614 0,15 L0,5"/> </g> <g fill="none" stroke="#000000" stroke-opacity="1" stroke-width="1" stroke-linecap="square" stroke-linejoin="bevel" transform="matrix(1,0,0,1,477.5,898)" font-family="MS Shell Dlg 2" font-size="9.75" font-weight="400" font-

style="normal" > <text fill="#000000" fill-opacity="1" stroke="none" xml:space="preserve" x="4" y="17" font-family="Arial" font-size="10pt" font-weight="700" font-style="italic" >rrnS</text> </g> <g fill="#ff9999" fill-opacity="1" stroke="#bfbfbf" stroke-opacity="1" stroke-width="1" stroke-linecap="square" stroke-linejoin="bevel" transform="matrix(1,0,0,1,520,900)" font-family="MS Shell Dlg 2" font-size="9.75" font-weight="400" font-style="normal" > <path vector-effect="none" fill-rule="evenodd" d="M0,1.7 L42.5,1.7 L42.5,0 L50,10 L42.5,20 L42.5,18.3 L0,18.3 L0,1.7"/> </g> <g fill="none" stroke="#000000" stroke-opacity="1" stroke-width="1" stroke-linecap="square" stroke-linejoin="bevel" transform="matrix(1,0,0,1,533.133,898)" font-family="MS Shell Dlg 2" font-size="9.75" font-weight="400" font-style="normal" > <text fill="#000000" fill-opacity="1" stroke="none" xml:space="preserve" x="4" y="17" font-family="Arial" font-size="10pt" font-weight="700" font-style="italic" >C</text> </g> <g fill="#ff9999" fill-opacity="1" stroke="#bfbfbf" stroke-opacity="1" stroke-width="1" stroke-linecap="square" stroke-linejoin="bevel" transform="matrix(1,0,0,1,571,900)" font-family="MS Shell Dlg 2" font-size="9.75" font-weight="400" font-style="normal" > <path vector-effect="none" fill-rule="evenodd" d="M0,1.7 L42.5,1.7 L42.5,0 L50,10 L42.5,20 L42.5,18.3 L0,18.3 L0,1.7"/> </g> <g fill="none" stroke="#000000" stroke-opacity="1" stroke-width="1" stroke-linecap="square" stroke-linejoin="bevel" transform="matrix(1,0,0,1,584.133,898)" font-family="MS Shell Dlg 2" font-size="9.75" font-weight="400" font-style="normal" > <text fill="#000000" fill-opacity="1" stroke="none" xml:space="preserve" x="4" y="17" font-family="Arial" font-size="10pt" font-weight="700" font-style="italic" >D</text> </g> <g fill="#99ffff" fill-opacity="1" stroke="#bfbfbf" stroke-opacity="1" stroke-width="1" stroke-linecap="square" stroke-linejoin="bevel" transform="matrix(1,0,0,1,622,900)" font-family="MS Shell Dlg 2" font-size="9.75" font-weight="400" font-style="normal" > <path vector-effect="none" fill-rule="evenodd" d="M0,5 C0,2.23858 2.23858,0 5,0 L45,0 C47.7614,0 50,2.23858 50,5 L50,15 C50,17.7614 47.7614,20 45,20 L5,20 C2.23858,20 0,17.7614 0,15 L0,5"/> </g> <g fill="none" stroke="#000000" stroke-opacity="1" stroke-width="1" stroke-linecap="square" stroke-linejoin="bevel" transform="matrix(1,0,0,1,631.5,898)" font-family="MS Shell Dlg 2" font-size="9.75" font-weight="400" font-style="normal" > <text fill="#000000" fill-opacity="1" stroke="none" xml:space="preserve" x="4" y="17" font-family="Arial" font-size="10pt" font-weight="700" font-style="italic" >rrnL</text> </g> <g fill="#ff9999" fill-opacity="1" stroke="#bfbfbf" stroke-opacity="1" stroke-width="1" stroke-linecap="square" stroke-linejoin="bevel" transform="matrix(1,0,0,1,673,900)" font-family="MS Shell Dlg 2" font-size="9.75" font-weight="400" font-style="normal" > <path vector-effect="none" fill-rule="evenodd" d="M0,1.7 L42.5,1.7 L42.5,0 L50,10 L42.5,20 L42.5,18.3 L0,18.3 L0,1.7"/> </g> <g fill="none" stroke="#000000" stroke-opacity="1" stroke-width="1" stroke-linecap="square" stroke-linejoin="bevel" transform="matrix(1,0,0,1,686.133,898)" font-family="MS Shell Dlg 2" font-size="9.75" font-weight="400" font-style="normal" > <text fill="#000000" fill-opacity="1" stroke="none" xml:space="preserve" x="4" y="17" font-family="Arial" font-size="10pt" font-weight="700" font-style="italic" >G</text> </g> <g fill="#ff9999" fill-opacity="1" stroke="#bfbfbf" stroke-opacity="1" stroke-width="1" stroke-linecap="square" stroke-linejoin="bevel" transform="matrix(1,0,0,1,724,900)" font-family="MS Shell Dlg 2" font-size="9.75" font-weight="400" font-style="normal" > <path vector-effect="none" fill-rule="evenodd" d="M0,1.7 L42.5,1.7 L42.5,0 L50,10 L42.5,20 L42.5,18.3 L0,18.3 L0,1.7"/> </g> <g fill="none" stroke="#000000" stroke-opacity="1" stroke-width="1" stroke-linecap="square" stroke-linejoin="bevel" transform="matrix(1,0,0,1,737.133,898)" font-family="MS Shell Dlg 2" font-size="9.75" font-weight="400" font-style="normal" > <text fill="#000000" fill-opacity="1" stroke="none" xml:space="preserve" x="4" y="17" font-family="Arial" font-size="10pt" font-weight="700" font-style="italic" >N</text> </g> <g fill="#ff9999" fill-opacity="1" stroke="#bfbfbf" stroke-opacity="1" stroke-width="1" stroke-linecap="square" stroke-linejoin="bevel" transform="matrix(1,0,0,1,775,900)" font-family="MS Shell Dlg 2" font-size="9.75" font-weight="400" font-style="normal" > <path vector-effect="none" fill-rule="evenodd" d="M0,1.7 L42.5,1.7 L42.5,0 L50,10 L42.5,20 L42.5,18.3 L0,18.3 L0,1.7"/> </g> <g fill="none" stroke="#000000" stroke-opacity="1" stroke-width="1" stroke-linecap="square" stroke-linejoin="bevel" transform="matrix(1,0,0,1,788.633,898)" font-family="MS Shell Dlg 2" font-size="9.75" font-weight="400" font-style="normal" > <text fill="#000000" fill-opacity="1" stroke="none" xml:space="preserve" x="4" y="17" font-family="Arial" font-size="10pt" font-weight="700" font-style="italic" >E</text> </g> <g fill="#ff9999" fill-opacity="1" stroke="#bfbfbf" stroke-opacity="1" stroke-width="1" stroke-linecap="square" stroke-linejoin="bevel" transform="matrix(1,0,0,1,826,900)" font-family="MS Shell Dlg 2" font-size="9.75" font-weight="400" font-style="normal" > <path vector-effect="none" fill-rule="evenodd" d="M0,1.7 L42.5,1.7 L42.5,0 L50,10 L42.5,20 L42.5,18.3 L0,18.3 L0,1.7"/> </g> <g fill="none" stroke="#000000" stroke-opacity="1" stroke-width="1" stroke-linecap="square" stroke-linejoin="bevel" transform="matrix(1,0,0,1,841.133,898)" font-family="MS Shell Dlg 2" font-size="9.75" font-weight="400" font-style="normal" > <text fill="#000000" fill-opacity="1" stroke="none" xml:space="preserve" x="4" y="17" font-family="Arial" font-size="10pt" font-weight="700" font-style="italic" >I</text> </g> <g fill="#ff9999" fill-opacity="1" stroke="#bfbfbf" stroke-opacity="1" stroke-width="1" stroke-linecap="square" stroke-linejoin="bevel"

transform="matrix(1,0,0,1,877,900)" font-family="MS Shell Dlg 2" font-size="9.75" font-weight="400" font-style="normal" > <path vector-effect="none" fill-rule="evenodd" d="M0,1.7 L42.5,1.7 L42.5,0 L50,10 L42.5,20 L42.5,18.3 L0,18.3 L0,1.7"/> </g> <g fill="none" stroke="#000000" stroke-opacity="1" stroke-width="1" stroke-linecap="square" stroke-linejoin="bevel" transform="matrix(1,0,0,1,890.633,898)" font-family="MS Shell Dlg 2" font-size="9.75" font-weight="400" font-style="normal" > <text fill="#000000" fill-opacity="1" stroke="none" xml:space="preserve" x="4" y="17" font-family="Arial" font-size="10pt" font-weight="700" font-style="italic" >Y</text> </g> <g fill="#ffff33" fill-opacity="1" stroke="#bfbfbf" stroke-opacity="1" stroke-width="1" stroke-linecap="square" stroke-linejoin="bevel" transform="matrix(1,0,0,1,928,900)" font-family="MS Shell Dlg 2" font-size="9.75" font-weight="400" font-style="normal" > <rect x="0" y="0" width="50" height="20"/> </g> <g fill="none" stroke="#000000" stroke-opacity="1" stroke-width="1" stroke-linecap="square" stroke-linejoin="bevel" transform="matrix(1,0,0,1,937.5,898)" font-family="MS Shell Dlg 2" font-size="9.75" font-weight="400" font-style="normal" > <text fill="#000000" fill-opacity="1" stroke="none" xml:space="preserve" x="4" y="17" font-family="Arial" font-size="10pt" font-weight="700" font-style="italic" >cytb</text> </g> <g fill="#ff9999" fill-opacity="1" stroke="#bfbfbf" stroke-opacity="1" stroke-width="1" stroke-linecap="square" stroke-linejoin="bevel" transform="matrix(1,0,0,1,979,900)" font-family="MS Shell Dlg 2" font-size="9.75" font-weight="400" font-style="normal" > <path vector-effect="none" fill-rule="evenodd" d="M0,1.7 L42.5,1.7 L42.5,0 L50,10 L42.5,20 L42.5,18.3 L0,18.3 L0,1.7"/> </g> <g fill="none" stroke="#000000" stroke-opacity="1" stroke-width="1" stroke-linecap="square" stroke-linejoin="bevel" transform="matrix(1,0,0,1,991.633,898)" font-family="MS Shell Dlg 2" font-size="9.75" font-weight="400" font-style="normal" > <text fill="#000000" fill-opacity="1" stroke="none" xml:space="preserve" x="4" y="17" font-family="Arial" font-size="10pt" font-weight="700" font-style="italic" >Q</text> </g> <g fill="#ffff33" fill-opacity="1" stroke="#bfbfbf" stroke-opacity="1" stroke-width="1" stroke-linecap="square" stroke-linejoin="bevel" transform="matrix(1,0,0,1,1030,900)" font-family="MS Shell Dlg 2" font-size="9.75" font-weight="400" font-style="normal" > <rect x="0" y="0" width="50" height="20"/> </g> <g fill="none" stroke="#000000" stroke-opacity="1" stroke-width="1" stroke-linecap="square" stroke-linejoin="bevel" transform="matrix(1,0,0,1,1038,898)" font-family="MS Shell Dlg 2" font-size="9.75" font-weight="400" font-style="normal" > <text fill="#000000" fill-opacity="1" stroke="none" xml:space="preserve" x="4" y="17" font-family="Arial" font-size="10pt" font-weight="700" font-style="italic" >cox2</text> </g> <g fill="#ff9999" fill-opacity="1" stroke="#bfbfbf" stroke-opacity="1" stroke-width="1" stroke-linecap="square" stroke-linejoin="bevel" transform="matrix(1,0,0,1,1081,900)" font-family="MS Shell Dlg 2" font-size="9.75" font-weight="400" font-style="normal" > <path vector-effect="none" fill-rule="evenodd" d="M0,1.7 L42.5,1.7 L42.5,0 L50,10 L42.5,20 L42.5,18.3 L0,18.3 L0,1.7"/> </g> <g fill="none" stroke="#000000" stroke-opacity="1" stroke-width="1" stroke-linecap="square" stroke-linejoin="bevel" transform="matrix(1,0,0,1,1094.63,898)" font-family="MS Shell Dlg 2" font-size="9.75" font-weight="400" font-style="normal" > <text fill="#000000" fill-opacity="1" stroke="none" xml:space="preserve" x="4" y="17" font-family="Arial" font-size="10pt" font-weight="700" font-style="italic" >K</text> </g> <g fill="#ff9999" fill-opacity="1" stroke="#bfbfbf" stroke-opacity="1" stroke-width="1" stroke-linecap="square" stroke-linejoin="bevel" transform="matrix(1,0,0,1,1132,900)" font-family="MS Shell Dlg 2" font-size="9.75" font-weight="400" font-style="normal" > <path vector-effect="none" fill-rule="evenodd" d="M0,1.7 L42.5,1.7 L42.5,0 L50,10 L42.5,20 L42.5,18.3 L0,18.3 L0,1.7"/> </g> <g fill="none" stroke="#000000" stroke-opacity="1" stroke-width="1" stroke-linecap="square" stroke-linejoin="bevel" transform="matrix(1,0,0,1,1144.13,898)" font-family="MS Shell Dlg 2" font-size="9.75" font-weight="400" font-style="normal" > <text fill="#000000" fill-opacity="1" stroke="none" xml:space="preserve" x="4" y="17" font-family="Arial" font-size="10pt" font-weight="700" font-style="italic" >M</text> </g> <g fill="#ff9999" fill-opacity="1" stroke="#bfbfbf" stroke-opacity="1" stroke-width="1" stroke-linecap="square" stroke-linejoin="bevel" transform="matrix(1,0,0,1,1183,900)" font-family="MS Shell Dlg 2" font-size="9.75" font-weight="400" font-style="normal" > <path vector-effect="none" fill-rule="evenodd" d="M0,1.7 L42.5,1.7 L42.5,0 L50,10 L42.5,20 L42.5,18.3 L0,18.3 L0,1.7"/> </g> <g fill="none" stroke="#000000" stroke-opacity="1" stroke-width="1" stroke-linecap="square" stroke-linejoin="bevel" transform="matrix(1,0,0,1,1197.13,898)" font-family="MS Shell Dlg 2" font-size="9.75" font-weight="400" font-style="normal" > <text fill="#000000" fill-opacity="1" stroke="none" xml:space="preserve" x="4" y="17" font-family="Arial" font-size="10pt" font-weight="700" font-style="italic" >L</text> </g> <g fill="#ffff33" fill-opacity="1" stroke="#bfbfbf" stroke-opacity="1" stroke-width="1" stroke-linecap="square" stroke-linejoin="bevel" transform="matrix(1,0,0,1,1234,900)" font-family="MS Shell Dlg 2" font-size="9.75" font-weight="400" font-style="normal" > <rect x="0" y="0" width="50" height="20"/> </g> <g fill="none" stroke="#000000" stroke-opacity="1" stroke-width="1" stroke-linecap="square" stroke-linejoin="bevel" transform="matrix(1,0,0,1,1241,898)" font-family="MS Shell Dlg 2" font-size="9.75" font-weight="400" font-style="normal" > <text fill="#000000" fill-opacity="1" stroke="none" xml:space="preserve" x="4" y="17" font-family="Arial" font-size="10pt" font-weight="700" font-style="italic" >nad1</text> </g> <g

fill="#ff9999" fill-opacity="1" stroke="#bfbfbf" stroke-opacity="1" stroke-width="1" stroke-linecap="square" stroke-linejoin="bevel" transform="matrix(1,0,0,1,1285,900)" font-family="MS Shell Dlg 2" font-size="9.75" font-weight="400" font-style="normal" > <path vector-effect="none" fill-rule="evenodd" d="M0,1.7 L42.5,1.7 L42.5,0 L50,10 L42.5,20 L42.5,18.3 L0,18.3 L0,1.7"/> </g> <g fill="none" stroke="#000000" stroke-opacity="1" stroke-width="1" stroke-linecap="square" stroke-linejoin="bevel" transform="matrix(1,0,0,1,1298.13,898)" font-family="MS Shell Dlg 2" font-size="9.75" font-weight="400" font-style="normal" > <text fill="#000000" fill-opacity="1" stroke="none" xml:space="preserve" x="4" y="17" font-family="Arial" font-size="10pt" font-weight="700" font-style="italic" >V</text> </g> <g fill="#ffff33" fill-opacity="1" stroke="#bfbfbf" stroke-opacity="1" stroke-width="1" stroke-linecap="square" stroke-linejoin="bevel" transform="matrix(1,0,0,1,1336,900)" font-family="MS Shell Dlg 2" font-size="9.75" font-weight="400" font-style="normal" > <rect x="0" y="0" width="50" height="20"/> </g> <g fill="none" stroke="#000000" stroke-opacity="1" stroke-width="1" stroke-linecap="square" stroke-linejoin="bevel" transform="matrix(1,0,0,1,1343,898)" font-family="MS Shell Dlg 2" font-size="9.75" font-weight="400" font-style="normal" > <text fill="#000000" fill-opacity="1" stroke="none" xml:space="preserve" x="4" y="17" font-family="Arial" font-size="10pt" font-weight="700" font-style="italic" >nad4</text> </g> <g fill="#ffff33" fill-opacity="1" stroke="#bfbfbf" stroke-opacity="1" stroke-width="1" stroke-linecap="square" stroke-linejoin="bevel" transform="matrix(1,0,0,1,1387,900)" font-family="MS Shell Dlg 2" font-size="9.75" font-weight="400" font-style="normal" > <rect x="0" y="0" width="50" height="20"/> </g> <g fill="none" stroke="#000000" stroke-opacity="1" stroke-width="1" stroke-linecap="square" stroke-linejoin="bevel" transform="matrix(1,0,0,1,1395,898)" font-family="MS Shell Dlg 2" font-size="9.75" font-weight="400" font-style="normal" > <text fill="#000000" fill-opacity="1" stroke="none" xml:space="preserve" x="4" y="17" font-family="Arial" font-size="10pt" font-weight="700" font-style="italic" >cox3</text> </g> <g fill="#ff9999" fill-opacity="1" stroke="#bfbfbf" stroke-opacity="1" stroke-width="1" stroke-linecap="square" stroke-linejoin="bevel" transform="matrix(1,0,0,1,1438,900)" font-family="MS Shell Dlg 2" font-size="9.75" font-weight="400" font-style="normal" > <path vector-effect="none" fill-rule="evenodd" d="M0,1.7 L42.5,1.7 L42.5,0 L50,10 L42.5,20 L42.5,18.3 L0,18.3 L0,1.7"/> </g> <g fill="none" stroke="#000000" stroke-opacity="1" stroke-width="1" stroke-linecap="square" stroke-linejoin="bevel" transform="matrix(1,0,0,1,1451.13,898)" font-family="MS Shell Dlg 2" font-size="9.75" font-weight="400" font-style="normal" > <text fill="#000000" fill-opacity="1" stroke="none" xml:space="preserve" x="4" y="17" font-family="Arial" font-size="10pt" font-weight="700" font-style="italic" >S</text> </g> <g fill="#ffff33" fill-opacity="1" stroke="#bfbfbf" stroke-opacity="1" stroke-width="1" stroke-linecap="square" stroke-linejoin="bevel" transform="matrix(1,0,0,1,1489,900)" font-family="MS Shell Dlg 2" font-size="9.75" font-weight="400" font-style="normal" > <rect x="0" y="0" width="50" height="20"/> </g> <g fill="none" stroke="#000000" stroke-opacity="1" stroke-width="1" stroke-linecap="square" stroke-linejoin="bevel" transform="matrix(1,0,0,1,1496,898)" font-family="MS Shell Dlg 2" font-size="9.75" font-weight="400" font-style="normal" > <text fill="#000000" fill-opacity="1" stroke="none" xml:space="preserve" x="4" y="17" font-family="Arial" font-size="10pt" font-weight="700" font-style="italic" >nad2</text> </g> <g fill="#ff9999" fill-opacity="1" stroke="#bfbfbf" stroke-opacity="1" stroke-width="1" stroke-linecap="square" stroke-linejoin="bevel" transform="matrix(1,0,0,1,1540,900)" font-family="MS Shell Dlg 2" font-size="9.75" font-weight="400" font-style="normal" > <path vector-effect="none" fill-rule="evenodd" d="M0,1.7 L42.5,1.7 L42.5,0 L50,10 L42.5,20 L42.5,18.3 L0,18.3 L0,1.7"/> </g> <g fill="none" stroke="#000000" stroke-opacity="1" stroke-width="1" stroke-linecap="square" stroke-linejoin="bevel" transform="matrix(1,0,0,1,1553.13,898)" font-family="MS Shell Dlg 2" font-size="9.75" font-weight="400" font-style="normal" > <text fill="#000000" fill-opacity="1" stroke="none" xml:space="preserve" x="4" y="17" font-family="Arial" font-size="10pt" font-weight="700" font-style="italic" >R</text> </g> <g fill="#ff9999" fill-opacity="1" stroke="#bfbfbf" stroke-opacity="1" stroke-width="1" stroke-linecap="square" stroke-linejoin="bevel" transform="matrix(1,0,0,1,1591,900)" font-family="MS Shell Dlg 2" font-size="9.75" font-weight="400" font-style="normal" > <path vector-effect="none" fill-rule="evenodd" d="M0,1.7 L42.5,1.7 L42.5,0 L50,10 L42.5,20 L42.5,18.3 L0,18.3 L0,1.7"/> </g> <g fill="none" stroke="#000000" stroke-opacity="1" stroke-width="1" stroke-linecap="square" stroke-linejoin="bevel" transform="matrix(1,0,0,1,1602.13,898)" font-family="MS Shell Dlg 2" font-size="9.75" font-weight="400" font-style="normal" > <text fill="#000000" fill-opacity="1" stroke="none" xml:space="preserve" x="4" y="17" font-family="Arial" font-size="10pt" font-weight="700" font-style="italic" >W</text> </g> <g fill="#ff9999" fill-opacity="1" stroke="#bfbfbf" stroke-opacity="1" stroke-width="1" stroke-linecap="square" stroke-linejoin="bevel" transform="matrix(1,0,0,1,1642,900)" font-family="MS Shell Dlg 2" font-size="9.75" font-weight="400" font-style="normal" > <path vector-effect="none" fill-rule="evenodd" d="M0,1.7 L42.5,1.7 L42.5,0 L50,10 L42.5,20 L42.5,18.3 L0,18.3 L0,1.7"/> </g> <g fill="none" stroke="#000000" stroke-opacity="1" stroke-width="1" stroke-linecap="square" stroke-linejoin="bevel" transform="matrix(1,0,0,1,1655.13,898)" font-family="MS Shell Dlg 2" font-size="9.75" font-weight="400" font-

style="normal" > <text fill="#000000" fill-opacity="1" stroke="none" xml:space="preserve" x="4" y="17" font-family="Arial" font-size="10pt" font-weight="700" font-style="italic" >A</text> </g> <g fill="#ff9999" fill-opacity="1" stroke="#bfbfbf" stroke-opacity="1" stroke-width="1" stroke-linecap="square" stroke-linejoin="bevel" transform="matrix(1,0,0,1,1693,900)" font-family="MS Shell Dlg 2" font-size="9.75" font-weight="400" font-style="normal" > <path vector-effect="none" fill-rule="evenodd" d="M0,1.7 L42.5,1.7 L42.5,0 L50,10 L42.5,20 L42.5,18.3 L0,18.3 L0,1.7"/> </g> <g fill="none" stroke="#000000" stroke-opacity="1" stroke-width="1" stroke-linecap="square" stroke-linejoin="bevel" transform="matrix(1,0,0,1,1706.13,898)" font-family="MS Shell Dlg 2" font-size="9.75" font-weight="400" font-style="normal" > <text fill="#000000" fill-opacity="1" stroke="none" xml:space="preserve" x="4" y="17" font-family="Arial" font-size="10pt" font-weight="700" font-style="italic" >S</text> </g> <g fill="#ff9999" fill-opacity="1" stroke="#bfbfbf" stroke-opacity="1" stroke-width="1" stroke-linecap="square" stroke-linejoin="bevel" transform="matrix(1,0,0,1,1744,900)" font-family="MS Shell Dlg 2" font-size="9.75" font-weight="400" font-style="normal" > <path vector-effect="none" fill-rule="evenodd" d="M0,1.7 L42.5,1.7 L42.5,0 L50,10 L42.5,20 L42.5,18.3 L0,18.3 L0,1.7"/> </g> <g fill="none" stroke="#000000" stroke-opacity="1" stroke-width="1" stroke-linecap="square" stroke-linejoin="bevel" transform="matrix(1,0,0,1,1757.13,898)" font-family="MS Shell Dlg 2" font-size="9.75" font-weight="400" font-style="normal" > <text fill="#000000" fill-opacity="1" stroke="none" xml:space="preserve" x="4" y="17" font-family="Arial" font-size="10pt" font-weight="700" font-style="italic" >H</text> </g> <g fill="#ffff33" fill-opacity="1" stroke="#bfbfbf" stroke-opacity="1" stroke-width="1" stroke-linecap="square" stroke-linejoin="bevel" transform="matrix(1,0,0,1,1795,900)" font-family="MS Shell Dlg 2" font-size="9.75" font-weight="400" font-style="normal" > <rect x="0" y="0" width="50" height="20"/> </g> <g fill="none" stroke="#000000" stroke-opacity="1" stroke-width="1" stroke-linecap="square" stroke-linejoin="bevel" transform="matrix(1,0,0,1,1802,898)" font-family="MS Shell Dlg 2" font-size="9.75" font-weight="400" font-style="normal" > <text fill="#000000" fill-opacity="1" stroke="none" xml:space="preserve" x="4" y="17" font-family="Arial" font-size="10pt" font-weight="700" font-style="italic" >nad3</text> </g> <g fill="#ff9999" fill-opacity="1" stroke="#bfbfbf" stroke-opacity="1" stroke-width="1" stroke-linecap="square" stroke-linejoin="bevel" transform="matrix(1,0,0,1,1846,900)" font-family="MS Shell Dlg 2" font-size="9.75" font-weight="400" font-style="normal" > <path vector-effect="none" fill-rule="evenodd" d="M0,1.7 L42.5,1.7 L42.5,0 L50,10 L42.5,20 L42.5,18.3 L0,18.3 L0,1.7"/> </g> <g fill="none" stroke="#000000" stroke-opacity="1" stroke-width="1" stroke-linecap="square" stroke-linejoin="bevel" transform="matrix(1,0,0,1,1860.13,898)" font-family="MS Shell Dlg 2" font-size="9.75" font-weight="400" font-style="normal" > <text fill="#000000" fill-opacity="1" stroke="none" xml:space="preserve" x="4" y="17" font-family="Arial" font-size="10pt" font-weight="700" font-style="italic" >L</text> </g> <g fill="#ffff33" fill-opacity="1" stroke="#bfbfbf" stroke-opacity="1" stroke-width="1" stroke-linecap="square" stroke-linejoin="bevel" transform="matrix(1,0,0,1,1897,900)" font-family="MS Shell Dlg 2" font-size="9.75" font-weight="400" font-style="normal" > <rect x="0" y="0" width="50" height="20"/> </g> <g fill="none" stroke="#000000" stroke-opacity="1" stroke-width="1" stroke-linecap="square" stroke-linejoin="bevel" transform="matrix(1,0,0,1,1905.5,898)" font-family="MS Shell Dlg 2" font-size="9.75" font-weight="400" font-style="normal" > <text fill="#000000" fill-opacity="1" stroke="none" xml:space="preserve" x="4" y="17" font-family="Arial" font-size="10pt" font-weight="700" font-style="italic" >atp8</text> </g> <g fill="none" stroke="#000000" stroke-opacity="1" stroke-width="1" stroke-linecap="square" stroke-linejoin="bevel" transform="matrix(1,0,0,1,1948,900)" font-family="MS Shell Dlg 2" font-size="9.75" font-weight="400" font-style="normal" > <text fill="#000000" fill-opacity="1" stroke="none" xml:space="preserve" x="4" y="17" font-family="Arial" font-size="10pt" font-weight="700" font-style="italic" >(+)</text> </g> <g fill="none" stroke="#000000" stroke-opacity="1" stroke-width="1" stroke-linecap="square" stroke-linejoin="bevel" transform="matrix(1,0,0,1,10,940)" font-family="MS Shell Dlg 2" font-size="9.75" font-weight="400" font-style="normal" > <text fill="#000000" fill-opacity="1" stroke="none" xml:space="preserve" x="4" y="17" font-family="Arial" font-size="10pt" font-weight="700" font-style="italic" >Septifer_bilocularis_NC_044131</text> </g> <g fill="#ffff33" fill-opacity="1" stroke="#bfbfbf" stroke-opacity="1" stroke-width="1" stroke-linecap="square" stroke-linejoin="bevel" transform="matrix(1,0,0,1,10,970)" font-family="MS Shell Dlg 2" font-size="9.75" font-weight="400" font-style="normal" > <rect x="0" y="0" width="50" height="20"/> </g> <g fill="none" stroke="#000000" stroke-opacity="1" stroke-width="1" stroke-linecap="square" stroke-linejoin="bevel" transform="matrix(1,0,0,1,18,968)" font-family="MS Shell Dlg 2" font-size="9.75" font-weight="400" font-style="normal" > <text fill="#000000" fill-opacity="1" stroke="none" xml:space="preserve" x="4" y="17" font-family="Arial" font-size="10pt" font-weight="700" font-style="italic" >cox1</text> </g> <g fill="#ffff33" fill-opacity="1" stroke="#bfbfbf" stroke-opacity="1" stroke-width="1" stroke-linecap="square" stroke-linejoin="bevel" transform="matrix(1,0,0,1,61,970)" font-family="MS Shell Dlg 2" font-size="9.75" font-weight="400" font-style="normal" > <rect x="0" y="0" width="50" height="20"/> </g> <g fill="none" stroke="#000000" stroke-opacity="1" stroke-width="1" stroke-linecap="square" stroke-linejoin="bevel"

transform="matrix(1,0,0,1,69.5,968)" font-family="MS Shell Dlg 2" font-size="9.75" font-weight="400" font-style="normal" > <text fill="#000000" fill-opacity="1" stroke="none" xml:space="preserve" x="4" y="17" font-family="Arial" font-size="10pt" font-weight="700" font-style="italic" >atp8</text> </g> <g fill="#ffff33" fill-opacity="1" stroke="#bfbfbf" stroke-opacity="1" stroke-width="1" stroke-linecap="square" stroke-linejoin="bevel" transform="matrix(1,0,0,1,112,970)" font-family="MS Shell Dlg 2" font-size="9.75" font-weight="400" font-style="normal" > <rect x="0" y="0" width="50" height="20"/> </g> <g fill="none" stroke="#000000" stroke-opacity="1" stroke-width="1" stroke-linecap="square" stroke-linejoin="bevel" transform="matrix(1,0,0,1,120.5,968)" font-family="MS Shell Dlg 2" font-size="9.75" font-weight="400" font-style="normal" > <text fill="#000000" fill-opacity="1" stroke="none" xml:space="preserve" x="4" y="17" font-family="Arial" font-size="10pt" font-weight="700" font-style="italic" >atp6</text> </g> <g fill="#ff9999" fill-opacity="1" stroke="#bfbfbf" stroke-opacity="1" stroke-width="1" stroke-linecap="square" stroke-linejoin="bevel" transform="matrix(1,0,0,1,163,970)" font-family="MS Shell Dlg 2" font-size="9.75" font-weight="400" font-style="normal" > <path vector-effect="none" fill-rule="evenodd" d="M0,1.7 L42.5,1.7 L42.5,0 L50,10 L42.5,20 L42.5,18.3 L0,18.3 L0,1.7"/> </g> <g fill="none" stroke="#000000" stroke-opacity="1" stroke-width="1" stroke-linecap="square" stroke-linejoin="bevel" transform="matrix(1,0,0,1,176.132,968)" font-family="MS Shell Dlg 2" font-size="9.75" font-weight="400" font-style="normal" > <text fill="#000000" fill-opacity="1" stroke="none" xml:space="preserve" x="4" y="17" font-family="Arial" font-size="10pt" font-weight="700" font-style="italic" >T</text> </g> <g fill="#ffff33" fill-opacity="1" stroke="#bfbfbf" stroke-opacity="1" stroke-width="1" stroke-linecap="square" stroke-linejoin="bevel" transform="matrix(1,0,0,1,214,970)" font-family="MS Shell Dlg 2" font-size="9.75" font-weight="400" font-style="normal" > <rect x="0" y="0" width="50" height="20"/> </g> <g fill="none" stroke="#000000" stroke-opacity="1" stroke-width="1" stroke-linecap="square" stroke-linejoin="bevel" transform="matrix(1,0,0,1,223.5,968)" font-family="MS Shell Dlg 2" font-size="9.75" font-weight="400" font-style="normal" > <text fill="#000000" fill-opacity="1" stroke="none" xml:space="preserve" x="4" y="17" font-family="Arial" font-size="10pt" font-weight="700" font-style="italic" >cytb</text> </g> <g fill="#ffff33" fill-opacity="1" stroke="#bfbfbf" stroke-opacity="1" stroke-width="1" stroke-linecap="square" stroke-linejoin="bevel" transform="matrix(1,0,0,1,265,970)" font-family="MS Shell Dlg 2" font-size="9.75" font-weight="400" font-style="normal" > <rect x="0" y="0" width="50" height="20"/> </g> <g fill="none" stroke="#000000" stroke-opacity="1" stroke-width="1" stroke-linecap="square" stroke-linejoin="bevel" transform="matrix(1,0,0,1,273,968)" font-family="MS Shell Dlg 2" font-size="9.75" font-weight="400" font-style="normal" > <text fill="#000000" fill-opacity="1" stroke="none" xml:space="preserve" x="4" y="17" font-family="Arial" font-size="10pt" font-weight="700" font-style="italic" >cox2</text> </g> <g fill="#ff9999" fill-opacity="1" stroke="#bfbfbf" stroke-opacity="1" stroke-width="1" stroke-linecap="square" stroke-linejoin="bevel" transform="matrix(1,0,0,1,316,970)" font-family="MS Shell Dlg 2" font-size="9.75" font-weight="400" font-style="normal" > <path vector-effect="none" fill-rule="evenodd" d="M0,1.7 L42.5,1.7 L42.5,0 L50,10 L42.5,20 L42.5,18.3 L0,18.3 L0,1.7"/> </g> <g fill="none" stroke="#000000" stroke-opacity="1" stroke-width="1" stroke-linecap="square" stroke-linejoin="bevel" transform="matrix(1,0,0,1,329.132,968)" font-family="MS Shell Dlg 2" font-size="9.75" font-weight="400" font-style="normal" > <text fill="#000000" fill-opacity="1" stroke="none" xml:space="preserve" x="4" y="17" font-family="Arial" font-size="10pt" font-weight="700" font-style="italic" >G</text> </g> <g fill="#ffff33" fill-opacity="1" stroke="#bfbfbf" stroke-opacity="1" stroke-width="1" stroke-linecap="square" stroke-linejoin="bevel" transform="matrix(1,0,0,1,367,970)" font-family="MS Shell Dlg 2" font-size="9.75" font-weight="400" font-style="normal" > <rect x="0" y="0" width="50" height="20"/> </g> <g fill="none" stroke="#000000" stroke-opacity="1" stroke-width="1" stroke-linecap="square" stroke-linejoin="bevel" transform="matrix(1,0,0,1,374,968)" font-family="MS Shell Dlg 2" font-size="9.75" font-weight="400" font-style="normal" > <text fill="#000000" fill-opacity="1" stroke="none" xml:space="preserve" x="4" y="17" font-family="Arial" font-size="10pt" font-weight="700" font-style="italic" >nad1</text> </g> <g fill="#ff9999" fill-opacity="1" stroke="#bfbfbf" stroke-opacity="1" stroke-width="1" stroke-linecap="square" stroke-linejoin="bevel" transform="matrix(1,0,0,1,418,970)" font-family="MS Shell Dlg 2" font-size="9.75" font-weight="400" font-style="normal" > <path vector-effect="none" fill-rule="evenodd" d="M0,1.7 L42.5,1.7 L42.5,0 L50,10 L42.5,20 L42.5,18.3 L0,18.3 L0,1.7"/> </g> <g fill="none" stroke="#000000" stroke-opacity="1" stroke-width="1" stroke-linecap="square" stroke-linejoin="bevel" transform="matrix(1,0,0,1,430.132,968)" font-family="MS Shell Dlg 2" font-size="9.75" font-weight="400" font-style="normal" > <text fill="#000000" fill-opacity="1" stroke="none" xml:space="preserve" x="4" y="17" font-family="Arial" font-size="10pt" font-weight="700" font-style="italic" >M</text> </g> <g fill="#ff9999" fill-opacity="1" stroke="#bfbfbf" stroke-opacity="1" stroke-width="1" stroke-linecap="square" stroke-linejoin="bevel" transform="matrix(1,0,0,1,469,970)" font-family="MS Shell Dlg 2" font-size="9.75" font-weight="400" font-style="normal" > <path vector-effect="none" fill-rule="evenodd" d="M0,1.7 L42.5,1.7 L42.5,0 L50,10 L42.5,20 L42.5,18.3 L0,18.3 L0,1.7"/> </g> <g

fill="none" stroke="#000000" stroke-opacity="1" stroke-width="1" stroke-linecap="square" stroke-linejoin="bevel" transform="matrix(1,0,0,1,483.132,968)" font-family="MS Shell Dlg 2" font-size="9.75" font-weight="400" font-style="normal" > <text fill="#000000" fill-opacity="1" stroke="none" xml:space="preserve" x="4" y="17" font-family="Arial" font-size="10pt" font-weight="700" font-style="italic" >L</text> </g> <g fill="#ff9999" fill-opacity="1" stroke="#bfbfbf" stroke-opacity="1" stroke-width="1" stroke-linecap="square" stroke-linejoin="bevel" transform="matrix(1,0,0,1,520,970)" font-family="MS Shell Dlg 2" font-size="9.75" font-weight="400" font-style="normal" > <path vector-effect="none" fill-rule="evenodd" d="M0,1.7 L42.5,1.7 L42.5,0 L50,10 L42.5,20 L42.5,18.3 L0,18.3 L0,1.7"/> </g> <g fill="none" stroke="#000000" stroke-opacity="1" stroke-width="1" stroke-linecap="square" stroke-linejoin="bevel" transform="matrix(1,0,0,1,533.133,968)" font-family="MS Shell Dlg 2" font-size="9.75" font-weight="400" font-style="normal" > <text fill="#000000" fill-opacity="1" stroke="none" xml:space="preserve" x="4" y="17" font-family="Arial" font-size="10pt" font-weight="700" font-style="italic" >V</text> </g> <g fill="#ffff33" fill-opacity="1" stroke="#bfbfbf" stroke-opacity="1" stroke-width="1" stroke-linecap="square" stroke-linejoin="bevel" transform="matrix(1,0,0,1,571,970)" font-family="MS Shell Dlg 2" font-size="9.75" font-weight="400" font-style="normal" > <rect x="0" y="0" width="50" height="20"/> </g> <g fill="none" stroke="#000000" stroke-opacity="1" stroke-width="1" stroke-linecap="square" stroke-linejoin="bevel" transform="matrix(1,0,0,1,578,968)" font-family="MS Shell Dlg 2" font-size="9.75" font-weight="400" font-style="normal" > <text fill="#000000" fill-opacity="1" stroke="none" xml:space="preserve" x="4" y="17" font-family="Arial" font-size="10pt" font-weight="700" font-style="italic" >nad4</text> </g> <g fill="#ff9999" fill-opacity="1" stroke="#bfbfbf" stroke-opacity="1" stroke-width="1" stroke-linecap="square" stroke-linejoin="bevel" transform="matrix(1,0,0,1,622,970)" font-family="MS Shell Dlg 2" font-size="9.75" font-weight="400" font-style="normal" > <path vector-effect="none" fill-rule="evenodd" d="M0,1.7 L42.5,1.7 L42.5,0 L50,10 L42.5,20 L42.5,18.3 L0,18.3 L0,1.7"/> </g> <g fill="none" stroke="#000000" stroke-opacity="1" stroke-width="1" stroke-linecap="square" stroke-linejoin="bevel" transform="matrix(1,0,0,1,635.133,968)" font-family="MS Shell Dlg 2" font-size="9.75" font-weight="400" font-style="normal" > <text fill="#000000" fill-opacity="1" stroke="none" xml:space="preserve" x="4" y="17" font-family="Arial" font-size="10pt" font-weight="700" font-style="italic" >N</text> </g> <g fill="#ffff33" fill-opacity="1" stroke="#bfbfbf" stroke-opacity="1" stroke-width="1" stroke-linecap="square" stroke-linejoin="bevel" transform="matrix(1,0,0,1,673,970)" font-family="MS Shell Dlg 2" font-size="9.75" font-weight="400" font-style="normal" > <rect x="0" y="0" width="50" height="20"/> </g> <g fill="none" stroke="#000000" stroke-opacity="1" stroke-width="1" stroke-linecap="square" stroke-linejoin="bevel" transform="matrix(1,0,0,1,681,968)" font-family="MS Shell Dlg 2" font-size="9.75" font-weight="400" font-style="normal" > <text fill="#000000" fill-opacity="1" stroke="none" xml:space="preserve" x="4" y="17" font-family="Arial" font-size="10pt" font-weight="700" font-style="italic" >cox3</text> </g> <g fill="#ff9999" fill-opacity="1" stroke="#bfbfbf" stroke-opacity="1" stroke-width="1" stroke-linecap="square" stroke-linejoin="bevel" transform="matrix(1,0,0,1,724,970)" font-family="MS Shell Dlg 2" font-size="9.75" font-weight="400" font-style="normal" > <path vector-effect="none" fill-rule="evenodd" d="M0,1.7 L42.5,1.7 L42.5,0 L50,10 L42.5,20 L42.5,18.3 L0,18.3 L0,1.7"/> </g> <g fill="none" stroke="#000000" stroke-opacity="1" stroke-width="1" stroke-linecap="square" stroke-linejoin="bevel" transform="matrix(1,0,0,1,737.133,968)" font-family="MS Shell Dlg 2" font-size="9.75" font-weight="400" font-style="normal" > <text fill="#000000" fill-opacity="1" stroke="none" xml:space="preserve" x="4" y="17" font-family="Arial" font-size="10pt" font-weight="700" font-style="italic" >S</text> </g> <g fill="#ff9999" fill-opacity="1" stroke="#bfbfbf" stroke-opacity="1" stroke-width="1" stroke-linecap="square" stroke-linejoin="bevel" transform="matrix(1,0,0,1,775,970)" font-family="MS Shell Dlg 2" font-size="9.75" font-weight="400" font-style="normal" > <path vector-effect="none" fill-rule="evenodd" d="M0,1.7 L42.5,1.7 L42.5,0 L50,10 L42.5,20 L42.5,18.3 L0,18.3 L0,1.7"/> </g> <g fill="none" stroke="#000000" stroke-opacity="1" stroke-width="1" stroke-linecap="square" stroke-linejoin="bevel" transform="matrix(1,0,0,1,788.133,968)" font-family="MS Shell Dlg 2" font-size="9.75" font-weight="400" font-style="normal" > <text fill="#000000" fill-opacity="1" stroke="none" xml:space="preserve" x="4" y="17" font-family="Arial" font-size="10pt" font-weight="700" font-style="italic" >D</text> </g> <g fill="#ffff33" fill-opacity="1" stroke="#bfbfbf" stroke-opacity="1" stroke-width="1" stroke-linecap="square" stroke-linejoin="bevel" transform="matrix(1,0,0,1,826,970)" font-family="MS Shell Dlg 2" font-size="9.75" font-weight="400" font-style="normal" > <rect x="0" y="0" width="50" height="20"/> </g> <g fill="none" stroke="#000000" stroke-opacity="1" stroke-width="1" stroke-linecap="square" stroke-linejoin="bevel" transform="matrix(1,0,0,1,833,968)" font-family="MS Shell Dlg 2" font-size="9.75" font-weight="400" font-style="normal" > <text fill="#000000" fill-opacity="1" stroke="none" xml:space="preserve" x="4" y="17" font-family="Arial" font-size="10pt" font-weight="700" font-style="italic" >nad2</text> </g> <g fill="#ff9999" fill-opacity="1" stroke="#bfbfbf" stroke-opacity="1" stroke-width="1" stroke-linecap="square" stroke-linejoin="bevel" transform="matrix(1,0,0,1,877,970)" font-family="MS Shell Dlg 2" font-size="9.75" font-weight="400" font-

style="normal" > <path vector-effect="none" fill-rule="evenodd" d="M0,1.7 L42.5,1.7 L42.5,0 L50,10 L42.5,20 L42.5,18.3 L0,18.3 L0,1.7"/> </g>   <g fill="none" stroke="#000000" stroke-opacity="1" stroke-width="1" stroke-linecap="square" stroke-linejoin="bevel" transform="matrix(1,0,0,1,890.633,968)" font-family="MS Shell Dlg 2" font-size="9.75" font-weight="400" font-style="normal" > <text fill="#000000" fill-opacity="1" stroke="none" xml:space="preserve" x="4" y="17" font-family="Arial" font-size="10pt" font-weight="700" font-style="italic"   >K</text> </g>   <g fill="#99ffff" fill-opacity="1" stroke="#bfbfbf" stroke-opacity="1" stroke-width="1" stroke-linecap="square" stroke-linejoin="bevel" transform="matrix(1,0,0,1,928,970)" font-family="MS Shell Dlg 2" font-size="9.75" font-weight="400" font-style="normal" > <path vector-effect="none" fill-rule="evenodd" d="M0,5 C0,2.23858 2.23858,0 5,0 L45,0 C47.7614,0 50,2.23858 50,5 L50,15 C50,17.7614 47.7614,20 45,20 L5,20 C2.23858,20 0,17.7614 0,15 L0,5"/> </g>   <g fill="none" stroke="#000000" stroke-opacity="1" stroke-width="1" stroke-linecap="square" stroke-linejoin="bevel" transform="matrix(1,0,0,1,936.5,968)" font-family="MS Shell Dlg 2" font-size="9.75" font-weight="400" font-style="normal" > <text fill="#000000" fill-opacity="1" stroke="none" xml:space="preserve" x="4" y="17" font-family="Arial" font-size="10pt" font-weight="700" font-style="italic"   >rrnS</text> </g>   <g fill="#ff9999" fill-opacity="1" stroke="#bfbfbf" stroke-opacity="1" stroke-width="1" stroke-linecap="square" stroke-linejoin="bevel" transform="matrix(1,0,0,1,979,970)" font-family="MS Shell Dlg 2" font-size="9.75" font-weight="400" font-style="normal" > <path vector-effect="none" fill-rule="evenodd" d="M0,1.7 L42.5,1.7 L42.5,0 L50,10 L42.5,20 L42.5,18.3 L0,18.3 L0,1.7"/> </g>   <g fill="none" stroke="#000000" stroke-opacity="1" stroke-width="1" stroke-linecap="square" stroke-linejoin="bevel" transform="matrix(1,0,0,1,992.133,968)" font-family="MS Shell Dlg 2" font-size="9.75" font-weight="400" font-style="normal" > <text fill="#000000" fill-opacity="1" stroke="none" xml:space="preserve" x="4" y="17" font-family="Arial" font-size="10pt" font-weight="700" font-style="italic"   >R</text> </g>   <g fill="#ff9999" fill-opacity="1" stroke="#bfbfbf" stroke-opacity="1" stroke-width="1" stroke-linecap="square" stroke-linejoin="bevel" transform="matrix(1,0,0,1,1030,970)" font-family="MS Shell Dlg 2" font-size="9.75" font-weight="400" font-style="normal" > <path vector-effect="none" fill-rule="evenodd" d="M0,1.7 L42.5,1.7 L42.5,0 L50,10 L42.5,20 L42.5,18.3 L0,18.3 L0,1.7"/> </g>   <g fill="none" stroke="#000000" stroke-opacity="1" stroke-width="1" stroke-linecap="square" stroke-linejoin="bevel" transform="matrix(1,0,0,1,1043.13,968)" font-family="MS Shell Dlg 2" font-size="9.75" font-weight="400" font-style="normal" > <text fill="#000000" fill-opacity="1" stroke="none" xml:space="preserve" x="4" y="17" font-family="Arial" font-size="10pt" font-weight="700" font-style="italic"   >A</text> </g>   <g fill="#ff9999" fill-opacity="1" stroke="#bfbfbf" stroke-opacity="1" stroke-width="1" stroke-linecap="square" stroke-linejoin="bevel" transform="matrix(1,0,0,1,1081,970)" font-family="MS Shell Dlg 2" font-size="9.75" font-weight="400" font-style="normal" > <path vector-effect="none" fill-rule="evenodd" d="M0,1.7 L42.5,1.7 L42.5,0 L50,10 L42.5,20 L42.5,18.3 L0,18.3 L0,1.7"/> </g>   <g fill="none" stroke="#000000" stroke-opacity="1" stroke-width="1" stroke-linecap="square" stroke-linejoin="bevel" transform="matrix(1,0,0,1,1094.13,968)" font-family="MS Shell Dlg 2" font-size="9.75" font-weight="400" font-style="normal" > <text fill="#000000" fill-opacity="1" stroke="none" xml:space="preserve" x="4" y="17" font-family="Arial" font-size="10pt" font-weight="700" font-style="italic"   >S</text> </g>   <g fill="#ff9999" fill-opacity="1" stroke="#bfbfbf" stroke-opacity="1" stroke-width="1" stroke-linecap="square" stroke-linejoin="bevel" transform="matrix(1,0,0,1,1132,970)" font-family="MS Shell Dlg 2" font-size="9.75" font-weight="400" font-style="normal" > <path vector-effect="none" fill-rule="evenodd" d="M0,1.7 L42.5,1.7 L42.5,0 L50,10 L42.5,20 L42.5,18.3 L0,18.3 L0,1.7"/> </g>   <g fill="none" stroke="#000000" stroke-opacity="1" stroke-width="1" stroke-linecap="square" stroke-linejoin="bevel" transform="matrix(1,0,0,1,1145.13,968)" font-family="MS Shell Dlg 2" font-size="9.75" font-weight="400" font-style="normal" > <text fill="#000000" fill-opacity="1" stroke="none" xml:space="preserve" x="4" y="17" font-family="Arial" font-size="10pt" font-weight="700" font-style="italic"   >H</text> </g>   <g fill="#ff9999" fill-opacity="1" stroke="#bfbfbf" stroke-opacity="1" stroke-width="1" stroke-linecap="square" stroke-linejoin="bevel" transform="matrix(1,0,0,1,1183,970)" font-family="MS Shell Dlg 2" font-size="9.75" font-weight="400" font-style="normal" > <path vector-effect="none" fill-rule="evenodd" d="M0,1.7 L42.5,1.7 L42.5,0 L50,10 L42.5,20 L42.5,18.3 L0,18.3 L0,1.7"/> </g>   <g fill="none" stroke="#000000" stroke-opacity="1" stroke-width="1" stroke-linecap="square" stroke-linejoin="bevel" transform="matrix(1,0,0,1,1195.13,968)" font-family="MS Shell Dlg 2" font-size="9.75" font-weight="400" font-style="normal" > <text fill="#000000" fill-opacity="1" stroke="none" xml:space="preserve" x="4" y="17" font-family="Arial" font-size="10pt" font-weight="700" font-style="italic"   >M</text> </g>   <g fill="#ffff33" fill-opacity="1" stroke="#bfbfbf" stroke-opacity="1" stroke-width="1" stroke-linecap="square" stroke-linejoin="bevel" transform="matrix(1,0,0,1,1234,970)" font-family="MS Shell Dlg 2" font-size="9.75" font-weight="400" font-style="normal" > <rect x="0" y="0" width="50" height="20"/> </g>   <g fill="none" stroke="#000000" stroke-opacity="1" stroke-width="1" stroke-linecap="square" stroke-linejoin="bevel" transform="matrix(1,0,0,1,1241,968)" font-family="MS Shell Dlg 2" font-size="9.75" font-weight="400" font-style="normal" > <text fill="#000000" fill-opacity="1" stroke="none"

xml:space="preserve" x="4" y="17" font-family="Arial" font-size="10pt" font-weight="700" font-style="italic" >nad5</text> </g> <g fill="#ffff33" fill-opacity="1" stroke="#bfbfbf" stroke-opacity="1" stroke-width="1" stroke-linecap="square" stroke-linejoin="bevel" transform="matrix(1,0,0,1,1285,970)" font-family="MS Shell Dlg 2" font-size="9.75" font-weight="400" font-style="normal" > <rect x="0" y="0" width="50" height="20"/> </g> <g fill="none" stroke="#000000" stroke-opacity="1" stroke-width="1" stroke-linecap="square" stroke-linejoin="bevel" transform="matrix(1,0,0,1,1292,968)" font-family="MS Shell Dlg 2" font-size="9.75" font-weight="400" font-style="normal" > <text fill="#000000" fill-opacity="1" stroke="none" xml:space="preserve" x="4" y="17" font-family="Arial" font-size="10pt" font-weight="700" font-style="italic" >nad6</text> </g> <g fill="#99ffff" fill-opacity="1" stroke="#bfbfbf" stroke-opacity="1" stroke-width="1" stroke-linecap="square" stroke-linejoin="bevel" transform="matrix(1,0,0,1,1336,970)" font-family="MS Shell Dlg 2" font-size="9.75" font-weight="400" font-style="normal" > <path vector-effect="none" fill-rule="evenodd" d="M0,5 C0,2.23858 2.23858,0 5,0 L45,0 C47.7614,0 50,2.23858 50,5 L50,15 C50,17.7614 47.7614,20 45,20 L5,20 C2.23858,20 0,17.7614 0,15 L0,5"/> </g> <g fill="none" stroke="#000000" stroke-opacity="1" stroke-width="1" stroke-linecap="square" stroke-linejoin="bevel" transform="matrix(1,0,0,1,1345.5,968)" font-family="MS Shell Dlg 2" font-size="9.75" font-weight="400" font-style="normal" > <text fill="#000000" fill-opacity="1" stroke="none" xml:space="preserve" x="4" y="17" font-family="Arial" font-size="10pt" font-weight="700" font-style="italic" >rrnL</text> </g> <g fill="#ff9999" fill-opacity="1" stroke="#bfbfbf" stroke-opacity="1" stroke-width="1" stroke-linecap="square" stroke-linejoin="bevel" transform="matrix(1,0,0,1,1387,970)" font-family="MS Shell Dlg 2" font-size="9.75" font-weight="400" font-style="normal" > <path vector-effect="none" fill-rule="evenodd" d="M0,1.7 L42.5,1.7 L42.5,0 L50,10 L42.5,20 L42.5,18.3 L0,18.3 L0,1.7"/> </g> <g fill="none" stroke="#000000" stroke-opacity="1" stroke-width="1" stroke-linecap="square" stroke-linejoin="bevel" transform="matrix(1,0,0,1,1399.63,968)" font-family="MS Shell Dlg 2" font-size="9.75" font-weight="400" font-style="normal" > <text fill="#000000" fill-opacity="1" stroke="none" xml:space="preserve" x="4" y="17" font-family="Arial" font-size="10pt" font-weight="700" font-style="italic" >Q</text> </g> <g fill="#ff9999" fill-opacity="1" stroke="#bfbfbf" stroke-opacity="1" stroke-width="1" stroke-linecap="square" stroke-linejoin="bevel" transform="matrix(1,0,0,1,1438,970)" font-family="MS Shell Dlg 2" font-size="9.75" font-weight="400" font-style="normal" > <path vector-effect="none" fill-rule="evenodd" d="M0,1.7 L42.5,1.7 L42.5,0 L50,10 L42.5,20 L42.5,18.3 L0,18.3 L0,1.7"/> </g> <g fill="none" stroke="#000000" stroke-opacity="1" stroke-width="1" stroke-linecap="square" stroke-linejoin="bevel" transform="matrix(1,0,0,1,1453.13,968)" font-family="MS Shell Dlg 2" font-size="9.75" font-weight="400" font-style="normal" > <text fill="#000000" fill-opacity="1" stroke="none" xml:space="preserve" x="4" y="17" font-family="Arial" font-size="10pt" font-weight="700" font-style="italic" >I</text> </g> <g fill="#ff9999" fill-opacity="1" stroke="#bfbfbf" stroke-opacity="1" stroke-width="1" stroke-linecap="square" stroke-linejoin="bevel" transform="matrix(1,0,0,1,1489,970)" font-family="MS Shell Dlg 2" font-size="9.75" font-weight="400" font-style="normal" > <path vector-effect="none" fill-rule="evenodd" d="M0,1.7 L42.5,1.7 L42.5,0 L50,10 L42.5,20 L42.5,18.3 L0,18.3 L0,1.7"/> </g> <g fill="none" stroke="#000000" stroke-opacity="1" stroke-width="1" stroke-linecap="square" stroke-linejoin="bevel" transform="matrix(1,0,0,1,1502.63,968)" font-family="MS Shell Dlg 2" font-size="9.75" font-weight="400" font-style="normal" > <text fill="#000000" fill-opacity="1" stroke="none" xml:space="preserve" x="4" y="17" font-family="Arial" font-size="10pt" font-weight="700" font-style="italic" >F</text> </g> <g fill="#ffff33" fill-opacity="1" stroke="#bfbfbf" stroke-opacity="1" stroke-width="1" stroke-linecap="square" stroke-linejoin="bevel" transform="matrix(1,0,0,1,1540,970)" font-family="MS Shell Dlg 2" font-size="9.75" font-weight="400" font-style="normal" > <rect x="0" y="0" width="50" height="20"/> </g> <g fill="none" stroke="#000000" stroke-opacity="1" stroke-width="1" stroke-linecap="square" stroke-linejoin="bevel" transform="matrix(1,0,0,1,1544,968)" font-family="MS Shell Dlg 2" font-size="9.75" font-weight="400" font-style="normal" > <text fill="#000000" fill-opacity="1" stroke="none" xml:space="preserve" x="4" y="17" font-family="Arial" font-size="10pt" font-weight="700" font-style="italic" >nad4L</text> </g> <g fill="#ff9999" fill-opacity="1" stroke="#bfbfbf" stroke-opacity="1" stroke-width="1" stroke-linecap="square" stroke-linejoin="bevel" transform="matrix(1,0,0,1,1591,970)" font-family="MS Shell Dlg 2" font-size="9.75" font-weight="400" font-style="normal" > <path vector-effect="none" fill-rule="evenodd" d="M0,1.7 L42.5,1.7 L42.5,0 L50,10 L42.5,20 L42.5,18.3 L0,18.3 L0,1.7"/> </g> <g fill="none" stroke="#000000" stroke-opacity="1" stroke-width="1" stroke-linecap="square" stroke-linejoin="bevel" transform="matrix(1,0,0,1,1604.63,968)" font-family="MS Shell Dlg 2" font-size="9.75" font-weight="400" font-style="normal" > <text fill="#000000" fill-opacity="1" stroke="none" xml:space="preserve" x="4" y="17" font-family="Arial" font-size="10pt" font-weight="700" font-style="italic" >Y</text> </g> <g fill="#ff9999" fill-opacity="1" stroke="#bfbfbf" stroke-opacity="1" stroke-width="1" stroke-linecap="square" stroke-linejoin="bevel" transform="matrix(1,0,0,1,1642,970)" font-family="MS Shell Dlg 2" font-size="9.75" font-weight="400" font-style="normal" > <path vector-effect="none" fill-rule="evenodd" d="M0,1.7 L42.5,1.7 L42.5,0 L50,10 L42.5,20 L42.5,18.3 L0,18.3 L0,1.7"/> </g> <g fill="none" stroke="#000000" stroke-opacity="1"

stroke-width="1" stroke-linecap="square" stroke-linejoin="bevel" transform="matrix(1,0,0,1,1655.63,968)" font-family="MS Shell Dlg 2" font-size="9.75" font-weight="400" font-style="normal" > <text fill="#000000" fill-opacity="1" stroke="none" xml:space="preserve" x="4" y="17" font-family="Arial" font-size="10pt" font-weight="700" font-style="italic" >P</text> </g> <g fill="#ff9999" fill-opacity="1" stroke="#bfbfbf" stroke-opacity="1" stroke-width="1" stroke-linecap="square" stroke-linejoin="bevel" transform="matrix(1,0,0,1,1693,970)" font-family="MS Shell Dlg 2" font-size="9.75" font-weight="400" font-style="normal" > <path vector-effect="none" fill-rule="evenodd" d="M0,1.7 L42.5,1.7 L42.5,0 L50,10 L42.5,20 L42.5,18.3 L0,18.3 L0,1.7"/> </g> <g fill="none" stroke="#000000" stroke-opacity="1" stroke-width="1" stroke-linecap="square" stroke-linejoin="bevel" transform="matrix(1,0,0,1,1706.13,968)" font-family="MS Shell Dlg 2" font-size="9.75" font-weight="400" font-style="normal" > <text fill="#000000" fill-opacity="1" stroke="none" xml:space="preserve" x="4" y="17" font-family="Arial" font-size="10pt" font-weight="700" font-style="italic" >C</text> </g> <g fill="#ff9999" fill-opacity="1" stroke="#bfbfbf" stroke-opacity="1" stroke-width="1" stroke-linecap="square" stroke-linejoin="bevel" transform="matrix(1,0,0,1,1744,970)" font-family="MS Shell Dlg 2" font-size="9.75" font-weight="400" font-style="normal" > <path vector-effect="none" fill-rule="evenodd" d="M0,1.7 L42.5,1.7 L42.5,0 L50,10 L42.5,20 L42.5,18.3 L0,18.3 L0,1.7"/> </g> <g fill="none" stroke="#000000" stroke-opacity="1" stroke-width="1" stroke-linecap="square" stroke-linejoin="bevel" transform="matrix(1,0,0,1,1755.13,968)" font-family="MS Shell Dlg 2" font-size="9.75" font-weight="400" font-style="normal" > <text fill="#000000" fill-opacity="1" stroke="none" xml:space="preserve" x="4" y="17" font-family="Arial" font-size="10pt" font-weight="700" font-style="italic" >W</text> </g> <g fill="#ffff33" fill-opacity="1" stroke="#bfbfbf" stroke-opacity="1" stroke-width="1" stroke-linecap="square" stroke-linejoin="bevel" transform="matrix(1,0,0,1,1795,970)" font-family="MS Shell Dlg 2" font-size="9.75" font-weight="400" font-style="normal" > <rect x="0" y="0" width="50" height="20"/> </g> <g fill="none" stroke="#000000" stroke-opacity="1" stroke-width="1" stroke-linecap="square" stroke-linejoin="bevel" transform="matrix(1,0,0,1,1802,968)" font-family="MS Shell Dlg 2" font-size="9.75" font-weight="400" font-style="normal" > <text fill="#000000" fill-opacity="1" stroke="none" xml:space="preserve" x="4" y="17" font-family="Arial" font-size="10pt" font-weight="700" font-style="italic" >nad3</text> </g> <g fill="#ff9999" fill-opacity="1" stroke="#bfbfbf" stroke-opacity="1" stroke-width="1" stroke-linecap="square" stroke-linejoin="bevel" transform="matrix(1,0,0,1,1846,970)" font-family="MS Shell Dlg 2" font-size="9.75" font-weight="400" font-style="normal" > <path vector-effect="none" fill-rule="evenodd" d="M0,1.7 L42.5,1.7 L42.5,0 L50,10 L42.5,20 L42.5,18.3 L0,18.3 L0,1.7"/> </g> <g fill="none" stroke="#000000" stroke-opacity="1" stroke-width="1" stroke-linecap="square" stroke-linejoin="bevel" transform="matrix(1,0,0,1,1859.63,968)" font-family="MS Shell Dlg 2" font-size="9.75" font-weight="400" font-style="normal" > <text fill="#000000" fill-opacity="1" stroke="none" xml:space="preserve" x="4" y="17" font-family="Arial" font-size="10pt" font-weight="700" font-style="italic" >E</text> </g> <g fill="#ff9999" fill-opacity="1" stroke="#bfbfbf" stroke-opacity="1" stroke-width="1" stroke-linecap="square" stroke-linejoin="bevel" transform="matrix(1,0,0,1,1897,970)" font-family="MS Shell Dlg 2" font-size="9.75" font-weight="400" font-style="normal" > <path vector-effect="none" fill-rule="evenodd" d="M0,1.7 L42.5,1.7 L42.5,0 L50,10 L42.5,20 L42.5,18.3 L0,18.3 L0,1.7"/> </g> <g fill="none" stroke="#000000" stroke-opacity="1" stroke-width="1" stroke-linecap="square" stroke-linejoin="bevel" transform="matrix(1,0,0,1,1911.13,968)" font-family="MS Shell Dlg 2" font-size="9.75" font-weight="400" font-style="normal" > <text fill="#000000" fill-opacity="1" stroke="none" xml:space="preserve" x="4" y="17" font-family="Arial" font-size="10pt" font-weight="700" font-style="italic" >L</text> </g> <g fill="none" stroke="#000000" stroke-opacity="1" stroke-width="1" stroke-linecap="square" stroke-linejoin="bevel" transform="matrix(1,0,0,1,1948,970)" font-family="MS Shell Dlg 2" font-size="9.75" font-weight="400" font-style="normal" > <text fill="#000000" fill-opacity="1" stroke="none" xml:space="preserve" x="4" y="17" font-family="Arial" font-size="10pt" font-weight="700" font-style="italic" >(+)</text> </g> <g fill="none" stroke="#000000" stroke-opacity="1" stroke-width="1" stroke-linecap="square" stroke-linejoin="bevel" transform="matrix(1,0,0,1,10,1010)" font-family="MS Shell Dlg 2" font-size="9.75" font-weight="400" font-style="normal" > <text fill="#000000" fill-opacity="1" stroke="none" xml:space="preserve" x="4" y="17" font-family="Arial" font-size="10pt" font-weight="700" font-style="italic" >Mytilisepta_keenae_NC_044127</text> </g> <g fill="#ffff33" fill-opacity="1" stroke="#bfbfbf" stroke-opacity="1" stroke-width="1" stroke-linecap="square" stroke-linejoin="bevel" transform="matrix(1,0,0,1,10,1040)" font-family="MS Shell Dlg 2" font-size="9.75" font-weight="400" font-style="normal" > <rect x="0" y="0" width="50" height="20"/> </g> <g fill="none" stroke="#000000" stroke-opacity="1" stroke-width="1" stroke-linecap="square" stroke-linejoin="bevel" transform="matrix(1,0,0,1,18,1038)" font-family="MS Shell Dlg 2" font-size="9.75" font-weight="400" font-style="normal" > <text fill="#000000" fill-opacity="1" stroke="none" xml:space="preserve" x="4" y="17" font-family="Arial" font-size="10pt" font-weight="700" font-style="italic" >cox1</text> </g> <g fill="#ff9999" fill-opacity="1" stroke="#bfbfbf" stroke-opacity="1" stroke-width="1" stroke-linecap="square" stroke-linejoin="bevel" transform="matrix(1,0,0,1,61,1040)" font-family="MS Shell Dlg 2"

font-size="9.75" font-weight="400" font-style="normal" > <path vector-effect="none" fill-rule="evenodd" d="M0,1.7 L42.5,1.7 L42.5,0 L50,10 L42.5,20 L42.5,18.3 L0,18.3 L0,1.7"/> </g> <g fill="none" stroke="#000000" stroke-opacity="1" stroke-width="1" stroke-linecap="square" stroke-linejoin="bevel" transform="matrix(1,0,0,1,75.1325,1038)" font-family="MS Shell Dlg 2" font-size="9.75" font-weight="400" font-style="normal" > <text fill="#000000" fill-opacity="1" stroke="none" xml:space="preserve" x="4" y="17" font-family="Arial" font-size="10pt" font-weight="700" font-style="italic" >L</text> </g> <g fill="#ff9999" fill-opacity="1" stroke="#bfbfbf" stroke-opacity="1" stroke-width="1" stroke-linecap="square" stroke-linejoin="bevel" transform="matrix(1,0,0,1,112,1040)" font-family="MS Shell Dlg 2" font-size="9.75" font-weight="400" font-style="normal" > <path vector-effect="none" fill-rule="evenodd" d="M0,1.7 L42.5,1.7 L42.5,0 L50,10 L42.5,20 L42.5,18.3 L0,18.3 L0,1.7"/> </g> <g fill="none" stroke="#000000" stroke-opacity="1" stroke-width="1" stroke-linecap="square" stroke-linejoin="bevel" transform="matrix(1,0,0,1,125.632,1038)" font-family="MS Shell Dlg 2" font-size="9.75" font-weight="400" font-style="normal" > <text fill="#000000" fill-opacity="1" stroke="none" xml:space="preserve" x="4" y="17" font-family="Arial" font-size="10pt" font-weight="700" font-style="italic" >F</text> </g> <g fill="#99ffff" fill-opacity="1" stroke="#bfbfbf" stroke-opacity="1" stroke-width="1" stroke-linecap="square" stroke-linejoin="bevel" transform="matrix(1,0,0,1,163,1040)" font-family="MS Shell Dlg 2" font-size="9.75" font-weight="400" font-style="normal" > <path vector-effect="none" fill-rule="evenodd" d="M0,5 C0,2.23858 2.23858,0 5,0 L45,0 C47.7614,0 50,2.23858 50,5 L50,15 C50,17.7614 47.7614,20 45,20 L5,20 C2.23858,20 0,17.7614 0,15 L0,5"/> </g> <g fill="none" stroke="#000000" stroke-opacity="1" stroke-width="1" stroke-linecap="square" stroke-linejoin="bevel" transform="matrix(1,0,0,1,172.5,1038)" font-family="MS Shell Dlg 2" font-size="9.75" font-weight="400" font-style="normal" > <text fill="#000000" fill-opacity="1" stroke="none" xml:space="preserve" x="4" y="17" font-family="Arial" font-size="10pt" font-weight="700" font-style="italic" >rrnL</text> </g> <g fill="#ff9999" fill-opacity="1" stroke="#bfbfbf" stroke-opacity="1" stroke-width="1" stroke-linecap="square" stroke-linejoin="bevel" transform="matrix(1,0,0,1,214,1040)" font-family="MS Shell Dlg 2" font-size="9.75" font-weight="400" font-style="normal" > <path vector-effect="none" fill-rule="evenodd" d="M0,1.7 L42.5,1.7 L42.5,0 L50,10 L42.5,20 L42.5,18.3 L0,18.3 L0,1.7"/> </g> <g fill="none" stroke="#000000" stroke-opacity="1" stroke-width="1" stroke-linecap="square" stroke-linejoin="bevel" transform="matrix(1,0,0,1,227.632,1038)" font-family="MS Shell Dlg 2" font-size="9.75" font-weight="400" font-style="normal" > <text fill="#000000" fill-opacity="1" stroke="none" xml:space="preserve" x="4" y="17" font-family="Arial" font-size="10pt" font-weight="700" font-style="italic" >Y</text> </g> <g fill="#ffff33" fill-opacity="1" stroke="#bfbfbf" stroke-opacity="1" stroke-width="1" stroke-linecap="square" stroke-linejoin="bevel" transform="matrix(1,0,0,1,265,1040)" font-family="MS Shell Dlg 2" font-size="9.75" font-weight="400" font-style="normal" > <rect x="0" y="0" width="50" height="20"/> </g> <g fill="none" stroke="#000000" stroke-opacity="1" stroke-width="1" stroke-linecap="square" stroke-linejoin="bevel" transform="matrix(1,0,0,1,269,1038)" font-family="MS Shell Dlg 2" font-size="9.75" font-weight="400" font-style="normal" > <text fill="#000000" fill-opacity="1" stroke="none" xml:space="preserve" x="4" y="17" font-family="Arial" font-size="10pt" font-weight="700" font-style="italic" >nad4L</text> </g> <g fill="#ff9999" fill-opacity="1" stroke="#bfbfbf" stroke-opacity="1" stroke-width="1" stroke-linecap="square" stroke-linejoin="bevel" transform="matrix(1,0,0,1,316,1040)" font-family="MS Shell Dlg 2" font-size="9.75" font-weight="400" font-style="normal" > <path vector-effect="none" fill-rule="evenodd" d="M0,1.7 L42.5,1.7 L42.5,0 L50,10 L42.5,20 L42.5,18.3 L0,18.3 L0,1.7"/> </g> <g fill="none" stroke="#000000" stroke-opacity="1" stroke-width="1" stroke-linecap="square" stroke-linejoin="bevel" transform="matrix(1,0,0,1,329.632,1038)" font-family="MS Shell Dlg 2" font-size="9.75" font-weight="400" font-style="normal" > <text fill="#000000" fill-opacity="1" stroke="none" xml:space="preserve" x="4" y="17" font-family="Arial" font-size="10pt" font-weight="700" font-style="italic" >P</text> </g> <g fill="#ff9999" fill-opacity="1" stroke="#bfbfbf" stroke-opacity="1" stroke-width="1" stroke-linecap="square" stroke-linejoin="bevel" transform="matrix(1,0,0,1,367,1040)" font-family="MS Shell Dlg 2" font-size="9.75" font-weight="400" font-style="normal" > <path vector-effect="none" fill-rule="evenodd" d="M0,1.7 L42.5,1.7 L42.5,0 L50,10 L42.5,20 L42.5,18.3 L0,18.3 L0,1.7"/> </g> <g fill="none" stroke="#000000" stroke-opacity="1" stroke-width="1" stroke-linecap="square" stroke-linejoin="bevel" transform="matrix(1,0,0,1,380.632,1038)" font-family="MS Shell Dlg 2" font-size="9.75" font-weight="400" font-style="normal" > <text fill="#000000" fill-opacity="1" stroke="none" xml:space="preserve" x="4" y="17" font-family="Arial" font-size="10pt" font-weight="700" font-style="italic" >E</text> </g> <g fill="#ffff33" fill-opacity="1" stroke="#bfbfbf" stroke-opacity="1" stroke-width="1" stroke-linecap="square" stroke-linejoin="bevel" transform="matrix(1,0,0,1,418,1040)" font-family="MS Shell Dlg 2" font-size="9.75" font-weight="400" font-style="normal" > <rect x="0" y="0" width="50" height="20"/> </g> <g fill="none" stroke="#000000" stroke-opacity="1" stroke-width="1" stroke-linecap="square" stroke-linejoin="bevel" transform="matrix(1,0,0,1,425,1038)" font-family="MS Shell Dlg 2" font-size="9.75" font-weight="400" font-style="normal" > <text fill="#000000" fill-opacity="1" stroke="none"

xml:space="preserve" x="4" y="17" font-family="Arial" font-size="10pt" font-weight="700" font-style="italic" >nad3</text> </g> <g fill="#ff9999" fill-opacity="1" stroke="#bfbfbf" stroke-opacity="1" stroke-width="1" stroke-linecap="square" stroke-linejoin="bevel" transform="matrix(1,0,0,1,469,1040)" font-family="MS Shell Dlg 2" font-size="9.75" font-weight="400" font-style="normal" > <path vector-effect="none" fill-rule="evenodd" d="M0,1.7 L42.5,1.7 L42.5,0 L50,10 L42.5,20 L42.5,18.3 L0,18.3 L0,1.7"/> </g> <g fill="none" stroke="#000000" stroke-opacity="1" stroke-width="1" stroke-linecap="square" stroke-linejoin="bevel" transform="matrix(1,0,0,1,480.132,1038)" font-family="MS Shell Dlg 2" font-size="9.75" font-weight="400" font-style="normal" > <text fill="#000000" fill-opacity="1" stroke="none" xml:space="preserve" x="4" y="17" font-family="Arial" font-size="10pt" font-weight="700" font-style="italic" >W</text> </g> <g fill="#ff9999" fill-opacity="1" stroke="#bfbfbf" stroke-opacity="1" stroke-width="1" stroke-linecap="square" stroke-linejoin="bevel" transform="matrix(1,0,0,1,520,1040)" font-family="MS Shell Dlg 2" font-size="9.75" font-weight="400" font-style="normal" > <path vector-effect="none" fill-rule="evenodd" d="M0,1.7 L42.5,1.7 L42.5,0 L50,10 L42.5,20 L42.5,18.3 L0,18.3 L0,1.7"/> </g> <g fill="none" stroke="#000000" stroke-opacity="1" stroke-width="1" stroke-linecap="square" stroke-linejoin="bevel" transform="matrix(1,0,0,1,533.133,1038)" font-family="MS Shell Dlg 2" font-size="9.75" font-weight="400" font-style="normal" > <text fill="#000000" fill-opacity="1" stroke="none" xml:space="preserve" x="4" y="17" font-family="Arial" font-size="10pt" font-weight="700" font-style="italic" >G</text> </g> <g fill="#99ffff" fill-opacity="1" stroke="#bfbfbf" stroke-opacity="1" stroke-width="1" stroke-linecap="square" stroke-linejoin="bevel" transform="matrix(1,0,0,1,571,1040)" font-family="MS Shell Dlg 2" font-size="9.75" font-weight="400" font-style="normal" > <path vector-effect="none" fill-rule="evenodd" d="M0,5 C0,2.23858 2.23858,0 5,0 L45,0 C47.7614,0 50,2.23858 50,5 L50,15 C50,17.7614 47.7614,20 45,20 L5,20 C2.23858,20 0,17.7614 0,15 L0,5"/> </g> <g fill="none" stroke="#000000" stroke-opacity="1" stroke-width="1" stroke-linecap="square" stroke-linejoin="bevel" transform="matrix(1,0,0,1,579.5,1038)" font-family="MS Shell Dlg 2" font-size="9.75" font-weight="400" font-style="normal" > <text fill="#000000" fill-opacity="1" stroke="none" xml:space="preserve" x="4" y="17" font-family="Arial" font-size="10pt" font-weight="700" font-style="italic" >rrnS</text> </g> <g fill="#ff9999" fill-opacity="1" stroke="#bfbfbf" stroke-opacity="1" stroke-width="1" stroke-linecap="square" stroke-linejoin="bevel" transform="matrix(1,0,0,1,622,1040)" font-family="MS Shell Dlg 2" font-size="9.75" font-weight="400" font-style="normal" > <path vector-effect="none" fill-rule="evenodd" d="M0,1.7 L42.5,1.7 L42.5,0 L50,10 L42.5,20 L42.5,18.3 L0,18.3 L0,1.7"/> </g> <g fill="none" stroke="#000000" stroke-opacity="1" stroke-width="1" stroke-linecap="square" stroke-linejoin="bevel" transform="matrix(1,0,0,1,635.133,1038)" font-family="MS Shell Dlg 2" font-size="9.75" font-weight="400" font-style="normal" > <text fill="#000000" fill-opacity="1" stroke="none" xml:space="preserve" x="4" y="17" font-family="Arial" font-size="10pt" font-weight="700" font-style="italic" >N</text> </g> <g fill="#ff9999" fill-opacity="1" stroke="#bfbfbf" stroke-opacity="1" stroke-width="1" stroke-linecap="square" stroke-linejoin="bevel" transform="matrix(1,0,0,1,673,1040)" font-family="MS Shell Dlg 2" font-size="9.75" font-weight="400" font-style="normal" > <path vector-effect="none" fill-rule="evenodd" d="M0,1.7 L42.5,1.7 L42.5,0 L50,10 L42.5,20 L42.5,18.3 L0,18.3 L0,1.7"/> </g> <g fill="none" stroke="#000000" stroke-opacity="1" stroke-width="1" stroke-linecap="square" stroke-linejoin="bevel" transform="matrix(1,0,0,1,685.133,1038)" font-family="MS Shell Dlg 2" font-size="9.75" font-weight="400" font-style="normal" > <text fill="#000000" fill-opacity="1" stroke="none" xml:space="preserve" x="4" y="17" font-family="Arial" font-size="10pt" font-weight="700" font-style="italic" >M</text> </g> <g fill="#ffff33" fill-opacity="1" stroke="#bfbfbf" stroke-opacity="1" stroke-width="1" stroke-linecap="square" stroke-linejoin="bevel" transform="matrix(1,0,0,1,724,1040)" font-family="MS Shell Dlg 2" font-size="9.75" font-weight="400" font-style="normal" > <rect x="0" y="0" width="50" height="20"/> </g> <g fill="none" stroke="#000000" stroke-opacity="1" stroke-width="1" stroke-linecap="square" stroke-linejoin="bevel" transform="matrix(1,0,0,1,731,1038)" font-family="MS Shell Dlg 2" font-size="9.75" font-weight="400" font-style="normal" > <text fill="#000000" fill-opacity="1" stroke="none" xml:space="preserve" x="4" y="17" font-family="Arial" font-size="10pt" font-weight="700" font-style="italic" >nad1</text> </g> <g fill="#ff9999" fill-opacity="1" stroke="#bfbfbf" stroke-opacity="1" stroke-width="1" stroke-linecap="square" stroke-linejoin="bevel" transform="matrix(1,0,0,1,775,1040)" font-family="MS Shell Dlg 2" font-size="9.75" font-weight="400" font-style="normal" > <path vector-effect="none" fill-rule="evenodd" d="M0,1.7 L42.5,1.7 L42.5,0 L50,10 L42.5,20 L42.5,18.3 L0,18.3 L0,1.7"/> </g> <g fill="none" stroke="#000000" stroke-opacity="1" stroke-width="1" stroke-linecap="square" stroke-linejoin="bevel" transform="matrix(1,0,0,1,790.133,1038)" font-family="MS Shell Dlg 2" font-size="9.75" font-weight="400" font-style="normal" > <text fill="#000000" fill-opacity="1" stroke="none" xml:space="preserve" x="4" y="17" font-family="Arial" font-size="10pt" font-weight="700" font-style="italic" >I</text> </g> <g fill="#ff9999" fill-opacity="1" stroke="#bfbfbf" stroke-opacity="1" stroke-width="1" stroke-linecap="square" stroke-linejoin="bevel" transform="matrix(1,0,0,1,826,1040)" font-family="MS Shell Dlg 2" font-size="9.75" font-weight="400" font-style="normal" > <path vector-effect="none" fill-rule="evenodd" d="M0,1.7 L42.5,1.7 L42.5,0

L50,10 L42.5,20 L42.5,18.3 L0,18.3 L0,1.7"/> </g> <g fill="none" stroke="#000000" stroke-opacity="1" stroke-width="1" stroke-linecap="square" stroke-linejoin="bevel" transform="matrix(1,0,0,1,840.133,1038)" font-family="MS Shell Dlg 2" font-size="9.75" font-weight="400" font-style="normal" > <text fill="#000000" fill-opacity="1" stroke="none" xml:space="preserve" x="4" y="17" font-family="Arial" font-size="10pt" font-weight="700" font-style="italic" >L</text> </g> <g fill="#ff9999" fill-opacity="1" stroke="#bfbfbf" stroke-opacity="1" stroke-width="1" stroke-linecap="square" stroke-linejoin="bevel" transform="matrix(1,0,0,1,877,1040)" font-family="MS Shell Dlg 2" font-size="9.75" font-weight="400" font-style="normal" > <path vector-effect="none" fill-rule="evenodd" d="M0,1.7 L42.5,1.7 L42.5,0 L50,10 L42.5,20 L42.5,18.3 L0,18.3 L0,1.7"/> </g> <g fill="none" stroke="#000000" stroke-opacity="1" stroke-width="1" stroke-linecap="square" stroke-linejoin="bevel" transform="matrix(1,0,0,1,890.133,1038)" font-family="MS Shell Dlg 2" font-size="9.75" font-weight="400" font-style="normal" > <text fill="#000000" fill-opacity="1" stroke="none" xml:space="preserve" x="4" y="17" font-family="Arial" font-size="10pt" font-weight="700" font-style="italic" >D</text> </g> <g fill="#ffff33" fill-opacity="1" stroke="#bfbfbf" stroke-opacity="1" stroke-width="1" stroke-linecap="square" stroke-linejoin="bevel" transform="matrix(1,0,0,1,928,1040)" font-family="MS Shell Dlg 2" font-size="9.75" font-weight="400" font-style="normal" > <rect x="0" y="0" width="50" height="20"/> </g> <g fill="none" stroke="#000000" stroke-opacity="1" stroke-width="1" stroke-linecap="square" stroke-linejoin="bevel" transform="matrix(1,0,0,1,935,1038)" font-family="MS Shell Dlg 2" font-size="9.75" font-weight="400" font-style="normal" > <text fill="#000000" fill-opacity="1" stroke="none" xml:space="preserve" x="4" y="17" font-family="Arial" font-size="10pt" font-weight="700" font-style="italic" >nad2</text> </g> <g fill="#ff9999" fill-opacity="1" stroke="#bfbfbf" stroke-opacity="1" stroke-width="1" stroke-linecap="square" stroke-linejoin="bevel" transform="matrix(1,0,0,1,979,1040)" font-family="MS Shell Dlg 2" font-size="9.75" font-weight="400" font-style="normal" > <path vector-effect="none" fill-rule="evenodd" d="M0,1.7 L42.5,1.7 L42.5,0 L50,10 L42.5,20 L42.5,18.3 L0,18.3 L0,1.7"/> </g> <g fill="none" stroke="#000000" stroke-opacity="1" stroke-width="1" stroke-linecap="square" stroke-linejoin="bevel" transform="matrix(1,0,0,1,991.133,1038)" font-family="MS Shell Dlg 2" font-size="9.75" font-weight="400" font-style="normal" > <text fill="#000000" fill-opacity="1" stroke="none" xml:space="preserve" x="4" y="17" font-family="Arial" font-size="10pt" font-weight="700" font-style="italic" >M</text> </g> <g fill="#ff9999" fill-opacity="1" stroke="#bfbfbf" stroke-opacity="1" stroke-width="1" stroke-linecap="square" stroke-linejoin="bevel" transform="matrix(1,0,0,1,1030,1040)" font-family="MS Shell Dlg 2" font-size="9.75" font-weight="400" font-style="normal" > <path vector-effect="none" fill-rule="evenodd" d="M0,1.7 L42.5,1.7 L42.5,0 L50,10 L42.5,20 L42.5,18.3 L0,18.3 L0,1.7"/> </g> <g fill="none" stroke="#000000" stroke-opacity="1" stroke-width="1" stroke-linecap="square" stroke-linejoin="bevel" transform="matrix(1,0,0,1,1043.63,1038)" font-family="MS Shell Dlg 2" font-size="9.75" font-weight="400" font-style="normal" > <text fill="#000000" fill-opacity="1" stroke="none" xml:space="preserve" x="4" y="17" font-family="Arial" font-size="10pt" font-weight="700" font-style="italic" >K</text> </g> <g fill="#ff9999" fill-opacity="1" stroke="#bfbfbf" stroke-opacity="1" stroke-width="1" stroke-linecap="square" stroke-linejoin="bevel" transform="matrix(1,0,0,1,1081,1040)" font-family="MS Shell Dlg 2" font-size="9.75" font-weight="400" font-style="normal" > <path vector-effect="none" fill-rule="evenodd" d="M0,1.7 L42.5,1.7 L42.5,0 L50,10 L42.5,20 L42.5,18.3 L0,18.3 L0,1.7"/> </g> <g fill="none" stroke="#000000" stroke-opacity="1" stroke-width="1" stroke-linecap="square" stroke-linejoin="bevel" transform="matrix(1,0,0,1,1093.63,1038)" font-family="MS Shell Dlg 2" font-size="9.75" font-weight="400" font-style="normal" > <text fill="#000000" fill-opacity="1" stroke="none" xml:space="preserve" x="4" y="17" font-family="Arial" font-size="10pt" font-weight="700" font-style="italic" >Q</text> </g> <g fill="#ff9999" fill-opacity="1" stroke="#bfbfbf" stroke-opacity="1" stroke-width="1" stroke-linecap="square" stroke-linejoin="bevel" transform="matrix(1,0,0,1,1132,1040)" font-family="MS Shell Dlg 2" font-size="9.75" font-weight="400" font-style="normal" > <path vector-effect="none" fill-rule="evenodd" d="M0,1.7 L42.5,1.7 L42.5,0 L50,10 L42.5,20 L42.5,18.3 L0,18.3 L0,1.7"/> </g> <g fill="none" stroke="#000000" stroke-opacity="1" stroke-width="1" stroke-linecap="square" stroke-linejoin="bevel" transform="matrix(1,0,0,1,1145.13,1038)" font-family="MS Shell Dlg 2" font-size="9.75" font-weight="400" font-style="normal" > <text fill="#000000" fill-opacity="1" stroke="none" xml:space="preserve" x="4" y="17" font-family="Arial" font-size="10pt" font-weight="700" font-style="italic" >C</text> </g> <g fill="#ff9999" fill-opacity="1" stroke="#bfbfbf" stroke-opacity="1" stroke-width="1" stroke-linecap="square" stroke-linejoin="bevel" transform="matrix(1,0,0,1,1183,1040)" font-family="MS Shell Dlg 2" font-size="9.75" font-weight="400" font-style="normal" > <path vector-effect="none" fill-rule="evenodd" d="M0,1.7 L42.5,1.7 L42.5,0 L50,10 L42.5,20 L42.5,18.3 L0,18.3 L0,1.7"/> </g> <g fill="none" stroke="#000000" stroke-opacity="1" stroke-width="1" stroke-linecap="square" stroke-linejoin="bevel" transform="matrix(1,0,0,1,1196.13,1038)" font-family="MS Shell Dlg 2" font-size="9.75" font-weight="400" font-style="normal" > <text fill="#000000" fill-opacity="1" stroke="none" xml:space="preserve" x="4" y="17" font-family="Arial" font-size="10pt" font-weight="700" font-style="italic" >A</text> </g> <g fill="#ff9999" fill-opacity="1" stroke="#bfbfbf"

stroke-opacity="1" stroke-width="1" stroke-linecap="square" stroke-linejoin="bevel" transform="matrix(1,0,0,1,1234,1040)" font-family="MS Shell Dlg 2" font-size="9.75" font-weight="400" font-style="normal" > <path vector-effect="none" fill-rule="evenodd" d="M0,1.7 L42.5,1.7 L42.5,0 L50,10 L42.5,20 L42.5,18.3 L0,18.3 L0,1.7"/> </g> <g fill="none" stroke="#000000" stroke-opacity="1" stroke-width="1" stroke-linecap="square" stroke-linejoin="bevel" transform="matrix(1,0,0,1,1247.13,1038)" font-family="MS Shell Dlg 2" font-size="9.75" font-weight="400" font-style="normal" > <text fill="#000000" fill-opacity="1" stroke="none" xml:space="preserve" x="4" y="17" font-family="Arial" font-size="10pt" font-weight="700" font-style="italic" >V</text> </g> <g fill="#ffff33" fill-opacity="1" stroke="#bfbfbf" stroke-opacity="1" stroke-width="1" stroke-linecap="square" stroke-linejoin="bevel" transform="matrix(1,0,0,1,1285,1040)" font-family="MS Shell Dlg 2" font-size="9.75" font-weight="400" font-style="normal" > <rect x="0" y="0" width="50" height="20"/> </g> <g fill="none" stroke="#000000" stroke-opacity="1" stroke-width="1" stroke-linecap="square" stroke-linejoin="bevel" transform="matrix(1,0,0,1,1292,1038)" font-family="MS Shell Dlg 2" font-size="9.75" font-weight="400" font-style="normal" > <text fill="#000000" fill-opacity="1" stroke="none" xml:space="preserve" x="4" y="17" font-family="Arial" font-size="10pt" font-weight="700" font-style="italic" >nad4</text> </g> <g fill="#ffff33" fill-opacity="1" stroke="#bfbfbf" stroke-opacity="1" stroke-width="1" stroke-linecap="square" stroke-linejoin="bevel" transform="matrix(1,0,0,1,1336,1040)" font-family="MS Shell Dlg 2" font-size="9.75" font-weight="400" font-style="normal" > <rect x="0" y="0" width="50" height="20"/> </g> <g fill="none" stroke="#000000" stroke-opacity="1" stroke-width="1" stroke-linecap="square" stroke-linejoin="bevel" transform="matrix(1,0,0,1,1344,1038)" font-family="MS Shell Dlg 2" font-size="9.75" font-weight="400" font-style="normal" > <text fill="#000000" fill-opacity="1" stroke="none" xml:space="preserve" x="4" y="17" font-family="Arial" font-size="10pt" font-weight="700" font-style="italic" >cox3</text> </g> <g fill="#ff9999" fill-opacity="1" stroke="#bfbfbf" stroke-opacity="1" stroke-width="1" stroke-linecap="square" stroke-linejoin="bevel" transform="matrix(1,0,0,1,1387,1040)" font-family="MS Shell Dlg 2" font-size="9.75" font-weight="400" font-style="normal" > <path vector-effect="none" fill-rule="evenodd" d="M0,1.7 L42.5,1.7 L42.5,0 L50,10 L42.5,20 L42.5,18.3 L0,18.3 L0,1.7"/> </g> <g fill="none" stroke="#000000" stroke-opacity="1" stroke-width="1" stroke-linecap="square" stroke-linejoin="bevel" transform="matrix(1,0,0,1,1400.13,1038)" font-family="MS Shell Dlg 2" font-size="9.75" font-weight="400" font-style="normal" > <text fill="#000000" fill-opacity="1" stroke="none" xml:space="preserve" x="4" y="17" font-family="Arial" font-size="10pt" font-weight="700" font-style="italic" >S</text> </g> <g fill="#ffff33" fill-opacity="1" stroke="#bfbfbf" stroke-opacity="1" stroke-width="1" stroke-linecap="square" stroke-linejoin="bevel" transform="matrix(1,0,0,1,1438,1040)" font-family="MS Shell Dlg 2" font-size="9.75" font-weight="400" font-style="normal" > <rect x="0" y="0" width="50" height="20"/> </g> <g fill="none" stroke="#000000" stroke-opacity="1" stroke-width="1" stroke-linecap="square" stroke-linejoin="bevel" transform="matrix(1,0,0,1,1445,1038)" font-family="MS Shell Dlg 2" font-size="9.75" font-weight="400" font-style="normal" > <text fill="#000000" fill-opacity="1" stroke="none" xml:space="preserve" x="4" y="17" font-family="Arial" font-size="10pt" font-weight="700" font-style="italic" >nad5</text> </g> <g fill="#ffff33" fill-opacity="1" stroke="#bfbfbf" stroke-opacity="1" stroke-width="1" stroke-linecap="square" stroke-linejoin="bevel" transform="matrix(1,0,0,1,1489,1040)" font-family="MS Shell Dlg 2" font-size="9.75" font-weight="400" font-style="normal" > <rect x="0" y="0" width="50" height="20"/> </g> <g fill="none" stroke="#000000" stroke-opacity="1" stroke-width="1" stroke-linecap="square" stroke-linejoin="bevel" transform="matrix(1,0,0,1,1496,1038)" font-family="MS Shell Dlg 2" font-size="9.75" font-weight="400" font-style="normal" > <text fill="#000000" fill-opacity="1" stroke="none" xml:space="preserve" x="4" y="17" font-family="Arial" font-size="10pt" font-weight="700" font-style="italic" >nad6</text> </g> <g fill="#ff9999" fill-opacity="1" stroke="#bfbfbf" stroke-opacity="1" stroke-width="1" stroke-linecap="square" stroke-linejoin="bevel" transform="matrix(1,0,0,1,1540,1040)" font-family="MS Shell Dlg 2" font-size="9.75" font-weight="400" font-style="normal" > <path vector-effect="none" fill-rule="evenodd" d="M0,1.7 L42.5,1.7 L42.5,0 L50,10 L42.5,20 L42.5,18.3 L0,18.3 L0,1.7"/> </g> <g fill="none" stroke="#000000" stroke-opacity="1" stroke-width="1" stroke-linecap="square" stroke-linejoin="bevel" transform="matrix(1,0,0,1,1553.13,1038)" font-family="MS Shell Dlg 2" font-size="9.75" font-weight="400" font-style="normal" > <text fill="#000000" fill-opacity="1" stroke="none" xml:space="preserve" x="4" y="17" font-family="Arial" font-size="10pt" font-weight="700" font-style="italic" >T</text> </g> <g fill="#ffff33" fill-opacity="1" stroke="#bfbfbf" stroke-opacity="1" stroke-width="1" stroke-linecap="square" stroke-linejoin="bevel" transform="matrix(1,0,0,1,1591,1040)" font-family="MS Shell Dlg 2" font-size="9.75" font-weight="400" font-style="normal" > <rect x="0" y="0" width="50" height="20"/> </g> <g fill="none" stroke="#000000" stroke-opacity="1" stroke-width="1" stroke-linecap="square" stroke-linejoin="bevel" transform="matrix(1,0,0,1,1600.5,1038)" font-family="MS Shell Dlg 2" font-size="9.75" font-weight="400" font-style="normal" > <text fill="#000000" fill-opacity="1" stroke="none" xml:space="preserve" x="4" y="17" font-family="Arial" font-size="10pt" font-weight="700" font-style="italic" >cytb</text> </g> <g fill="#ffff33"

fill-opacity="1" stroke="#bfbfbf" stroke-opacity="1" stroke-width="1" stroke-linecap="square" stroke-linejoin="bevel" transform="matrix(1,0,0,1,1642,1040)" font-family="MS Shell Dlg 2" font-size="9.75" font-weight="400" font-style="normal" > <rect x="0" y="0" width="50" height="20"/> </g> <g fill="none" stroke="#000000" stroke-opacity="1" stroke-width="1" stroke-linecap="square" stroke-linejoin="bevel" transform="matrix(1,0,0,1,1650,1038)" font-family="MS Shell Dlg 2" font-size="9.75" font-weight="400" font-style="normal" > <text fill="#000000" fill-opacity="1" stroke="none" xml:space="preserve" x="4" y="17" font-family="Arial" font-size="10pt" font-weight="700" font-style="italic" >cox2</text> </g> <g fill="#ff9999" fill-opacity="1" stroke="#bfbfbf" stroke-opacity="1" stroke-width="1" stroke-linecap="square" stroke-linejoin="bevel" transform="matrix(1,0,0,1,1693,1040)" font-family="MS Shell Dlg 2" font-size="9.75" font-weight="400" font-style="normal" > <path vector-effect="none" fill-rule="evenodd" d="M0,1.7 L42.5,1.7 L42.5,0 L50,10 L42.5,20 L42.5,18.3 L0,18.3 L0,1.7"/> </g> <g fill="none" stroke="#000000" stroke-opacity="1" stroke-width="1" stroke-linecap="square" stroke-linejoin="bevel" transform="matrix(1,0,0,1,1706.13,1038)" font-family="MS Shell Dlg 2" font-size="9.75" font-weight="400" font-style="normal" > <text fill="#000000" fill-opacity="1" stroke="none" xml:space="preserve" x="4" y="17" font-family="Arial" font-size="10pt" font-weight="700" font-style="italic" >R</text> </g> <g fill="#ff9999" fill-opacity="1" stroke="#bfbfbf" stroke-opacity="1" stroke-width="1" stroke-linecap="square" stroke-linejoin="bevel" transform="matrix(1,0,0,1,1744,1040)" font-family="MS Shell Dlg 2" font-size="9.75" font-weight="400" font-style="normal" > <path vector-effect="none" fill-rule="evenodd" d="M0,1.7 L42.5,1.7 L42.5,0 L50,10 L42.5,20 L42.5,18.3 L0,18.3 L0,1.7"/> </g> <g fill="none" stroke="#000000" stroke-opacity="1" stroke-width="1" stroke-linecap="square" stroke-linejoin="bevel" transform="matrix(1,0,0,1,1757.13,1038)" font-family="MS Shell Dlg 2" font-size="9.75" font-weight="400" font-style="normal" > <text fill="#000000" fill-opacity="1" stroke="none" xml:space="preserve" x="4" y="17" font-family="Arial" font-size="10pt" font-weight="700" font-style="italic" >H</text> </g> <g fill="#ffff33" fill-opacity="1" stroke="#bfbfbf" stroke-opacity="1" stroke-width="1" stroke-linecap="square" stroke-linejoin="bevel" transform="matrix(1,0,0,1,1795,1040)" font-family="MS Shell Dlg 2" font-size="9.75" font-weight="400" font-style="normal" > <rect x="0" y="0" width="50" height="20"/> </g> <g fill="none" stroke="#000000" stroke-opacity="1" stroke-width="1" stroke-linecap="square" stroke-linejoin="bevel" transform="matrix(1,0,0,1,1803.5,1038)" font-family="MS Shell Dlg 2" font-size="9.75" font-weight="400" font-style="normal" > <text fill="#000000" fill-opacity="1" stroke="none" xml:space="preserve" x="4" y="17" font-family="Arial" font-size="10pt" font-weight="700" font-style="italic" >atp6</text> </g> <g fill="#ff9999" fill-opacity="1" stroke="#bfbfbf" stroke-opacity="1" stroke-width="1" stroke-linecap="square" stroke-linejoin="bevel" transform="matrix(1,0,0,1,1846,1040)" font-family="MS Shell Dlg 2" font-size="9.75" font-weight="400" font-style="normal" > <path vector-effect="none" fill-rule="evenodd" d="M0,1.7 L42.5,1.7 L42.5,0 L50,10 L42.5,20 L42.5,18.3 L0,18.3 L0,1.7"/> </g> <g fill="none" stroke="#000000" stroke-opacity="1" stroke-width="1" stroke-linecap="square" stroke-linejoin="bevel" transform="matrix(1,0,0,1,1859.13,1038)" font-family="MS Shell Dlg 2" font-size="9.75" font-weight="400" font-style="normal" > <text fill="#000000" fill-opacity="1" stroke="none" xml:space="preserve" x="4" y="17" font-family="Arial" font-size="10pt" font-weight="700" font-style="italic" >S</text> </g> <g fill="none" stroke="#000000" stroke-opacity="1" stroke-width="1" stroke-linecap="square" stroke-linejoin="bevel" transform="matrix(1,0,0,1,1897,1040)" font-family="MS Shell Dlg 2" font-size="9.75" font-weight="400" font-style="normal" > <text fill="#000000" fill-opacity="1" stroke="none" xml:space="preserve" x="4" y="17" font-family="Arial" font-size="10pt" font-weight="700" font-style="italic" >(+)</text> </g> <g fill="none" stroke="#000000" stroke-opacity="1" stroke-width="1" stroke-linecap="square" stroke-linejoin="bevel" transform="matrix(1,0,0,1,10,1080)" font-family="MS Shell Dlg 2" font-size="9.75" font-weight="400" font-style="normal" > <text fill="#000000" fill-opacity="1" stroke="none" xml:space="preserve" x="4" y="17" font-family="Arial" font-size="10pt" font-weight="700" font-style="italic" >Brachidontes_exustus_NC_024882</text> </g> <g fill="#ffff33" fill-opacity="1" stroke="#bfbfbf" stroke-opacity="1" stroke-width="1" stroke-linecap="square" stroke-linejoin="bevel" transform="matrix(1,0,0,1,10,1110)" font-family="MS Shell Dlg 2" font-size="9.75" font-weight="400" font-style="normal" > <rect x="0" y="0" width="50" height="20"/> </g> <g fill="none" stroke="#000000" stroke-opacity="1" stroke-width="1" stroke-linecap="square" stroke-linejoin="bevel" transform="matrix(1,0,0,1,18,1108)" font-family="MS Shell Dlg 2" font-size="9.75" font-weight="400" font-style="normal" > <text fill="#000000" fill-opacity="1" stroke="none" xml:space="preserve" x="4" y="17" font-family="Arial" font-size="10pt" font-weight="700" font-style="italic" >cox1</text> </g> <g fill="#ffff33" fill-opacity="1" stroke="#bfbfbf" stroke-opacity="1" stroke-width="1" stroke-linecap="square" stroke-linejoin="bevel" transform="matrix(1,0,0,1,61,1110)" font-family="MS Shell Dlg 2" font-size="9.75" font-weight="400" font-style="normal" > <rect x="0" y="0" width="50" height="20"/> </g> <g fill="none" stroke="#000000" stroke-opacity="1" stroke-width="1" stroke-linecap="square" stroke-linejoin="bevel" transform="matrix(1,0,0,1,68,1108)" font-family="MS Shell Dlg 2" font-size="9.75" font-weight="400" font-

style="normal" > <text fill="#000000" fill-opacity="1" stroke="none" xml:space="preserve" x="4" y="17" font-family="Arial" font-size="10pt" font-weight="700" font-style="italic"   >nad2</text> </g>   <g fill="#ff9999" fill-opacity="1" stroke="#bfbfbf" stroke-opacity="1" stroke-width="1" stroke-linecap="square" stroke-linejoin="bevel" transform="matrix(1,0,0,1,112,1110)" font-family="MS Shell Dlg 2" font-size="9.75" font-weight="400" font-style="normal" > <path vector-effect="none" fill-rule="evenodd" d="M0,1.7 L42.5,1.7 L42.5,0 L50,10 L42.5,20 L42.5,18.3 L0,18.3 L0,1.7"/> </g>   <g fill="none" stroke="#000000" stroke-opacity="1" stroke-width="1" stroke-linecap="square" stroke-linejoin="bevel" transform="matrix(1,0,0,1,127.132,1108)" font-family="MS Shell Dlg 2" font-size="9.75" font-weight="400" font-style="normal" > <text fill="#000000" fill-opacity="1" stroke="none" xml:space="preserve" x="4" y="17" font-family="Arial" font-size="10pt" font-weight="700" font-style="italic"   >I</text> </g>   <g fill="#ff9999" fill-opacity="1" stroke="#bfbfbf" stroke-opacity="1" stroke-width="1" stroke-linecap="square" stroke-linejoin="bevel" transform="matrix(1,0,0,1,163,1110)" font-family="MS Shell Dlg 2" font-size="9.75" font-weight="400" font-style="normal" > <path vector-effect="none" fill-rule="evenodd" d="M0,1.7 L42.5,1.7 L42.5,0 L50,10 L42.5,20 L42.5,18.3 L0,18.3 L0,1.7"/> </g>   <g fill="none" stroke="#000000" stroke-opacity="1" stroke-width="1" stroke-linecap="square" stroke-linejoin="bevel" transform="matrix(1,0,0,1,176.632,1108)" font-family="MS Shell Dlg 2" font-size="9.75" font-weight="400" font-style="normal" > <text fill="#000000" fill-opacity="1" stroke="none" xml:space="preserve" x="4" y="17" font-family="Arial" font-size="10pt" font-weight="700" font-style="italic"   >F</text> </g>   <g fill="#99ffff" fill-opacity="1" stroke="#bfbfbf" stroke-opacity="1" stroke-width="1" stroke-linecap="square" stroke-linejoin="bevel" transform="matrix(1,0,0,1,214,1110)" font-family="MS Shell Dlg 2" font-size="9.75" font-weight="400" font-style="normal" > <path vector-effect="none" fill-rule="evenodd" d="M0,5 C0,2.23858 2.23858,0 5,0 L45,0 C47.7614,0 50,2.23858 50,5 L50,15 C50,17.7614 47.7614,20 45,20 L5,20 C2.23858,20 0,17.7614 0,15 L0,5"/> </g>   <g fill="none" stroke="#000000" stroke-opacity="1" stroke-width="1" stroke-linecap="square" stroke-linejoin="bevel" transform="matrix(1,0,0,1,223.5,1108)" font-family="MS Shell Dlg 2" font-size="9.75" font-weight="400" font-style="normal" > <text fill="#000000" fill-opacity="1" stroke="none" xml:space="preserve" x="4" y="17" font-family="Arial" font-size="10pt" font-weight="700" font-style="italic"   >rrnL</text> </g>   <g fill="#ff9999" fill-opacity="1" stroke="#bfbfbf" stroke-opacity="1" stroke-width="1" stroke-linecap="square" stroke-linejoin="bevel" transform="matrix(1,0,0,1,265,1110)" font-family="MS Shell Dlg 2" font-size="9.75" font-weight="400" font-style="normal" > <path vector-effect="none" fill-rule="evenodd" d="M0,1.7 L42.5,1.7 L42.5,0 L50,10 L42.5,20 L42.5,18.3 L0,18.3 L0,1.7"/> </g>   <g fill="none" stroke="#000000" stroke-opacity="1" stroke-width="1" stroke-linecap="square" stroke-linejoin="bevel" transform="matrix(1,0,0,1,278.632,1108)" font-family="MS Shell Dlg 2" font-size="9.75" font-weight="400" font-style="normal" > <text fill="#000000" fill-opacity="1" stroke="none" xml:space="preserve" x="4" y="17" font-family="Arial" font-size="10pt" font-weight="700" font-style="italic"   >Y</text> </g>   <g fill="#ffff33" fill-opacity="1" stroke="#bfbfbf" stroke-opacity="1" stroke-width="1" stroke-linecap="square" stroke-linejoin="bevel" transform="matrix(1,0,0,1,316,1110)" font-family="MS Shell Dlg 2" font-size="9.75" font-weight="400" font-style="normal" > <rect x="0" y="0" width="50" height="20"/> </g>   <g fill="none" stroke="#000000" stroke-opacity="1" stroke-width="1" stroke-linecap="square" stroke-linejoin="bevel" transform="matrix(1,0,0,1,320,1108)" font-family="MS Shell Dlg 2" font-size="9.75" font-weight="400" font-style="normal" > <text fill="#000000" fill-opacity="1" stroke="none" xml:space="preserve" x="4" y="17" font-family="Arial" font-size="10pt" font-weight="700" font-style="italic"   >nad4L</text> </g>   <g fill="#ff9999" fill-opacity="1" stroke="#bfbfbf" stroke-opacity="1" stroke-width="1" stroke-linecap="square" stroke-linejoin="bevel" transform="matrix(1,0,0,1,367,1110)" font-family="MS Shell Dlg 2" font-size="9.75" font-weight="400" font-style="normal" > <path vector-effect="none" fill-rule="evenodd" d="M0,1.7 L42.5,1.7 L42.5,0 L50,10 L42.5,20 L42.5,18.3 L0,18.3 L0,1.7"/> </g>   <g fill="none" stroke="#000000" stroke-opacity="1" stroke-width="1" stroke-linecap="square" stroke-linejoin="bevel" transform="matrix(1,0,0,1,380.632,1108)" font-family="MS Shell Dlg 2" font-size="9.75" font-weight="400" font-style="normal" > <text fill="#000000" fill-opacity="1" stroke="none" xml:space="preserve" x="4" y="17" font-family="Arial" font-size="10pt" font-weight="700" font-style="italic"   >P</text> </g>   <g fill="#ff9999" fill-opacity="1" stroke="#bfbfbf" stroke-opacity="1" stroke-width="1" stroke-linecap="square" stroke-linejoin="bevel" transform="matrix(1,0,0,1,418,1110)" font-family="MS Shell Dlg 2" font-size="9.75" font-weight="400" font-style="normal" > <path vector-effect="none" fill-rule="evenodd" d="M0,1.7 L42.5,1.7 L42.5,0 L50,10 L42.5,20 L42.5,18.3 L0,18.3 L0,1.7"/> </g>   <g fill="none" stroke="#000000" stroke-opacity="1" stroke-width="1" stroke-linecap="square" stroke-linejoin="bevel" transform="matrix(1,0,0,1,431.632,1108)" font-family="MS Shell Dlg 2" font-size="9.75" font-weight="400" font-style="normal" > <text fill="#000000" fill-opacity="1" stroke="none" xml:space="preserve" x="4" y="17" font-family="Arial" font-size="10pt" font-weight="700" font-style="italic"   >E</text> </g>   <g fill="#ff9999" fill-opacity="1" stroke="#bfbfbf" stroke-opacity="1" stroke-width="1" stroke-linecap="square" stroke-linejoin="bevel" transform="matrix(1,0,0,1,469,1110)" font-

family="MS Shell Dlg 2" font-size="9.75" font-weight="400" font-style="normal" > <path vector-effect="none" fill-rule="evenodd" d="M0,1.7 L42.5,1.7 L42.5,0 L50,10 L42.5,20 L42.5,18.3 L0,18.3 L0,1.7"/> </g> <g fill="none" stroke="#000000" stroke-opacity="1" stroke-width="1" stroke-linecap="square" stroke-linejoin="bevel" transform="matrix(1,0,0,1,482.132,1108)" font-family="MS Shell Dlg 2" font-size="9.75" font-weight="400" font-style="normal" > <text fill="#000000" fill-opacity="1" stroke="none" xml:space="preserve" x="4" y="17" font-family="Arial" font-size="10pt" font-weight="700" font-style="italic" >C</text> </g> <g fill="#ffff33" fill-opacity="1" stroke="#bfbfbf" stroke-opacity="1" stroke-width="1" stroke-linecap="square" stroke-linejoin="bevel" transform="matrix(1,0,0,1,520,1110)" font-family="MS Shell Dlg 2" font-size="9.75" font-weight="400" font-style="normal" > <rect x="0" y="0" width="50" height="20"/> </g> <g fill="none" stroke="#000000" stroke-opacity="1" stroke-width="1" stroke-linecap="square" stroke-linejoin="bevel" transform="matrix(1,0,0,1,527,1108)" font-family="MS Shell Dlg 2" font-size="9.75" font-weight="400" font-style="normal" > <text fill="#000000" fill-opacity="1" stroke="none" xml:space="preserve" x="4" y="17" font-family="Arial" font-size="10pt" font-weight="700" font-style="italic" >nad3</text> </g> <g fill="#ff9999" fill-opacity="1" stroke="#bfbfbf" stroke-opacity="1" stroke-width="1" stroke-linecap="square" stroke-linejoin="bevel" transform="matrix(1,0,0,1,571,1110)" font-family="MS Shell Dlg 2" font-size="9.75" font-weight="400" font-style="normal" > <path vector-effect="none" fill-rule="evenodd" d="M0,1.7 L42.5,1.7 L42.5,0 L50,10 L42.5,20 L42.5,18.3 L0,18.3 L0,1.7"/> </g> <g fill="none" stroke="#000000" stroke-opacity="1" stroke-width="1" stroke-linecap="square" stroke-linejoin="bevel" transform="matrix(1,0,0,1,583.133,1108)" font-family="MS Shell Dlg 2" font-size="9.75" font-weight="400" font-style="normal" > <text fill="#000000" fill-opacity="1" stroke="none" xml:space="preserve" x="4" y="17" font-family="Arial" font-size="10pt" font-weight="700" font-style="italic" >M</text> </g> <g fill="#ff9999" fill-opacity="1" stroke="#bfbfbf" stroke-opacity="1" stroke-width="1" stroke-linecap="square" stroke-linejoin="bevel" transform="matrix(1,0,0,1,622,1110)" font-family="MS Shell Dlg 2" font-size="9.75" font-weight="400" font-style="normal" > <path vector-effect="none" fill-rule="evenodd" d="M0,1.7 L42.5,1.7 L42.5,0 L50,10 L42.5,20 L42.5,18.3 L0,18.3 L0,1.7"/> </g> <g fill="none" stroke="#000000" stroke-opacity="1" stroke-width="1" stroke-linecap="square" stroke-linejoin="bevel" transform="matrix(1,0,0,1,633.133,1108)" font-family="MS Shell Dlg 2" font-size="9.75" font-weight="400" font-style="normal" > <text fill="#000000" fill-opacity="1" stroke="none" xml:space="preserve" x="4" y="17" font-family="Arial" font-size="10pt" font-weight="700" font-style="italic" >W</text> </g> <g fill="#ff9999" fill-opacity="1" stroke="#bfbfbf" stroke-opacity="1" stroke-width="1" stroke-linecap="square" stroke-linejoin="bevel" transform="matrix(1,0,0,1,673,1110)" font-family="MS Shell Dlg 2" font-size="9.75" font-weight="400" font-style="normal" > <path vector-effect="none" fill-rule="evenodd" d="M0,1.7 L42.5,1.7 L42.5,0 L50,10 L42.5,20 L42.5,18.3 L0,18.3 L0,1.7"/> </g> <g fill="none" stroke="#000000" stroke-opacity="1" stroke-width="1" stroke-linecap="square" stroke-linejoin="bevel" transform="matrix(1,0,0,1,683.633,1108)" font-family="MS Shell Dlg 2" font-size="9.75" font-weight="400" font-style="normal" > <text fill="#000000" fill-opacity="1" stroke="none" xml:space="preserve" x="4" y="17" font-family="Arial" font-size="10pt" font-weight="700" font-style="italic" >L2</text> </g> <g fill="#ff9999" fill-opacity="1" stroke="#bfbfbf" stroke-opacity="1" stroke-width="1" stroke-linecap="square" stroke-linejoin="bevel" transform="matrix(1,0,0,1,724,1110)" font-family="MS Shell Dlg 2" font-size="9.75" font-weight="400" font-style="normal" > <path vector-effect="none" fill-rule="evenodd" d="M0,1.7 L42.5,1.7 L42.5,0 L50,10 L42.5,20 L42.5,18.3 L0,18.3 L0,1.7"/> </g> <g fill="none" stroke="#000000" stroke-opacity="1" stroke-width="1" stroke-linecap="square" stroke-linejoin="bevel" transform="matrix(1,0,0,1,737.633,1108)" font-family="MS Shell Dlg 2" font-size="9.75" font-weight="400" font-style="normal" > <text fill="#000000" fill-opacity="1" stroke="none" xml:space="preserve" x="4" y="17" font-family="Arial" font-size="10pt" font-weight="700" font-style="italic" >K</text> </g> <g fill="#ff9999" fill-opacity="1" stroke="#bfbfbf" stroke-opacity="1" stroke-width="1" stroke-linecap="square" stroke-linejoin="bevel" transform="matrix(1,0,0,1,775,1110)" font-family="MS Shell Dlg 2" font-size="9.75" font-weight="400" font-style="normal" > <path vector-effect="none" fill-rule="evenodd" d="M0,1.7 L42.5,1.7 L42.5,0 L50,10 L42.5,20 L42.5,18.3 L0,18.3 L0,1.7"/> </g> <g fill="none" stroke="#000000" stroke-opacity="1" stroke-width="1" stroke-linecap="square" stroke-linejoin="bevel" transform="matrix(1,0,0,1,785.633,1108)" font-family="MS Shell Dlg 2" font-size="9.75" font-weight="400" font-style="normal" > <text fill="#000000" fill-opacity="1" stroke="none" xml:space="preserve" x="4" y="17" font-family="Arial" font-size="10pt" font-weight="700" font-style="italic" >L1</text> </g> <g fill="#ff9999" fill-opacity="1" stroke="#bfbfbf" stroke-opacity="1" stroke-width="1" stroke-linecap="square" stroke-linejoin="bevel" transform="matrix(1,0,0,1,826,1110)" font-family="MS Shell Dlg 2" font-size="9.75" font-weight="400" font-style="normal" > <path vector-effect="none" fill-rule="evenodd" d="M0,1.7 L42.5,1.7 L42.5,0 L50,10 L42.5,20 L42.5,18.3 L0,18.3 L0,1.7"/> </g> <g fill="none" stroke="#000000" stroke-opacity="1" stroke-width="1" stroke-linecap="square" stroke-linejoin="bevel" transform="matrix(1,0,0,1,839.133,1108)" font-family="MS Shell Dlg 2" font-size="9.75" font-weight="400" font-style="normal" > <text fill="#000000" fill-opacity="1" stroke="none" xml:space="preserve" x="4" y="17" font-

family="Arial" font-size="10pt" font-weight="700" font-style="italic" >V</text> </g> <g fill="#ffff33"
fill-opacity="1" stroke="#bfbfbf" stroke-opacity="1" stroke-width="1" stroke-linecap="square" stroke-
linejoin="bevel" transform="matrix(1,0,0,1,877,1110)" font-family="MS Shell Dlg 2" font-size="9.75" font-
weight="400" font-style="normal" > <rect x="0" y="0" width="50" height="20"/> </g> <g fill="none"
stroke="#000000" stroke-opacity="1" stroke-width="1" stroke-linecap="square" stroke-linejoin="bevel"
transform="matrix(1,0,0,1,884,1108)" font-family="MS Shell Dlg 2" font-size="9.75" font-weight="400" font-
style="normal" > <text fill="#000000" fill-opacity="1" stroke="none" xml:space="preserve" x="4" y="17" font-
family="Arial" font-size="10pt" font-weight="700" font-style="italic" >nad4</text> </g> <g
fill="#ff9999" fill-opacity="1" stroke="#bfbfbf" stroke-opacity="1" stroke-width="1" stroke-linecap="square"
stroke-linejoin="bevel" transform="matrix(1,0,0,1,928,1110)" font-family="MS Shell Dlg 2" font-size="9.75" font-
weight="400" font-style="normal" > <path vector-effect="none" fill-rule="evenodd" d="M0,1.7 L42.5,1.7 L42.5,0
L50,10 L42.5,20 L42.5,18.3 L0,18.3 L0,1.7"/> </g> <g fill="none" stroke="#000000" stroke-opacity="1"
stroke-width="1" stroke-linecap="square" stroke-linejoin="bevel" transform="matrix(1,0,0,1,941.133,1108)" font-
family="MS Shell Dlg 2" font-size="9.75" font-weight="400" font-style="normal" > <text fill="#000000" fill-
opacity="1" stroke="none" xml:space="preserve" x="4" y="17" font-family="Arial" font-size="10pt" font-
weight="700" font-style="italic" >A</text> </g> <g fill="#ff9999" fill-opacity="1" stroke="#bfbfbf"
stroke-opacity="1" stroke-width="1" stroke-linecap="square" stroke-linejoin="bevel"
transform="matrix(1,0,0,1,979,1110)" font-family="MS Shell Dlg 2" font-size="9.75" font-weight="400" font-
style="normal" > <path vector-effect="none" fill-rule="evenodd" d="M0,1.7 L42.5,1.7 L42.5,0 L50,10 L42.5,20
L42.5,18.3 L0,18.3 L0,1.7"/> </g> <g fill="none" stroke="#000000" stroke-opacity="1" stroke-width="1"
stroke-linecap="square" stroke-linejoin="bevel" transform="matrix(1,0,0,1,992.133,1108)" font-family="MS Shell
Dlg 2" font-size="9.75" font-weight="400" font-style="normal" > <text fill="#000000" fill-opacity="1"
stroke="none" xml:space="preserve" x="4" y="17" font-family="Arial" font-size="10pt" font-weight="700" font-
style="italic" >R</text> </g> <g fill="#ff9999" fill-opacity="1" stroke="#bfbfbf" stroke-opacity="1"
stroke-width="1" stroke-linecap="square" stroke-linejoin="bevel" transform="matrix(1,0,0,1,1030,1110)" font-
family="MS Shell Dlg 2" font-size="9.75" font-weight="400" font-style="normal" > <path vector-effect="none"
fill-rule="evenodd" d="M0,1.7 L42.5,1.7 L42.5,0 L50,10 L42.5,20 L42.5,18.3 L0,18.3 L0,1.7"/> </g> <g
fill="none" stroke="#000000" stroke-opacity="1" stroke-width="1" stroke-linecap="square" stroke-linejoin="bevel"
transform="matrix(1,0,0,1,1042.13,1108)" font-family="MS Shell Dlg 2" font-size="9.75" font-weight="400" font-
style="normal" > <text fill="#000000" fill-opacity="1" stroke="none" xml:space="preserve" x="4" y="17" font-
family="Arial" font-size="10pt" font-weight="700" font-style="italic" >M</text> </g> <g fill="#ff9999"
fill-opacity="1" stroke="#bfbfbf" stroke-opacity="1" stroke-width="1" stroke-linecap="square" stroke-
linejoin="bevel" transform="matrix(1,0,0,1,1081,1110)" font-family="MS Shell Dlg 2" font-size="9.75" font-
weight="400" font-style="normal" > <path vector-effect="none" fill-rule="evenodd" d="M0,1.7 L42.5,1.7 L42.5,0
L50,10 L42.5,20 L42.5,18.3 L0,18.3 L0,1.7"/> </g> <g fill="none" stroke="#000000" stroke-opacity="1"
stroke-width="1" stroke-linecap="square" stroke-linejoin="bevel" transform="matrix(1,0,0,1,1094.13,1108)" font-
family="MS Shell Dlg 2" font-size="9.75" font-weight="400" font-style="normal" > <text fill="#000000" fill-
opacity="1" stroke="none" xml:space="preserve" x="4" y="17" font-family="Arial" font-size="10pt" font-
weight="700" font-style="italic" >N</text> </g> <g fill="#ffff33" fill-opacity="1" stroke="#bfbfbf"
stroke-opacity="1" stroke-width="1" stroke-linecap="square" stroke-linejoin="bevel"
transform="matrix(1,0,0,1,1132,1110)" font-family="MS Shell Dlg 2" font-size="9.75" font-weight="400" font-
style="normal" > <rect x="0" y="0" width="50" height="20"/> </g> <g fill="none" stroke="#000000" stroke-
opacity="1" stroke-width="1" stroke-linecap="square" stroke-linejoin="bevel"
transform="matrix(1,0,0,1,1140,1108)" font-family="MS Shell Dlg 2" font-size="9.75" font-weight="400" font-
style="normal" > <text fill="#000000" fill-opacity="1" stroke="none" xml:space="preserve" x="4" y="17" font-
family="Arial" font-size="10pt" font-weight="700" font-style="italic" >cox3</text> </g> <g
fill="#ff9999" fill-opacity="1" stroke="#bfbfbf" stroke-opacity="1" stroke-width="1" stroke-linecap="square"
stroke-linejoin="bevel" transform="matrix(1,0,0,1,1183,1110)" font-family="MS Shell Dlg 2" font-size="9.75"
font-weight="400" font-style="normal" > <path vector-effect="none" fill-rule="evenodd" d="M0,1.7 L42.5,1.7
L42.5,0 L50,10 L42.5,20 L42.5,18.3 L0,18.3 L0,1.7"/> </g> <g fill="none" stroke="#000000" stroke-
opacity="1" stroke-width="1" stroke-linecap="square" stroke-linejoin="bevel"
transform="matrix(1,0,0,1,1192.63,1108)" font-family="MS Shell Dlg 2" font-size="9.75" font-weight="400" font-
style="normal" > <text fill="#000000" fill-opacity="1" stroke="none" xml:space="preserve" x="4" y="17" font-
family="Arial" font-size="10pt" font-weight="700" font-style="italic" >S2</text> </g> <g fill="#ffff33"
fill-opacity="1" stroke="#bfbfbf" stroke-opacity="1" stroke-width="1" stroke-linecap="square" stroke-
linejoin="bevel" transform="matrix(1,0,0,1,1234,1110)" font-family="MS Shell Dlg 2" font-size="9.75" font-
weight="400" font-style="normal" > <rect x="0" y="0" width="50" height="20"/> </g> <g fill="none"
stroke="#000000" stroke-opacity="1" stroke-width="1" stroke-linecap="square" stroke-linejoin="bevel"

transform="matrix(1,0,0,1,1241,1108)" font-family="MS Shell Dlg 2" font-size="9.75" font-weight="400" font-style="normal" > <text fill="#000000" fill-opacity="1" stroke="none" xml:space="preserve" x="4" y="17" font-family="Arial" font-size="10pt" font-weight="700" font-style="italic" >nad5</text> </g> <g fill="#ffff33" fill-opacity="1" stroke="#bfbfbf" stroke-opacity="1" stroke-width="1" stroke-linecap="square" stroke-linejoin="bevel" transform="matrix(1,0,0,1,1285,1110)" font-family="MS Shell Dlg 2" font-size="9.75" font-weight="400" font-style="normal" > <rect x="0" y="0" width="50" height="20"/> </g> <g fill="none" stroke="#000000" stroke-opacity="1" stroke-width="1" stroke-linecap="square" stroke-linejoin="bevel" transform="matrix(1,0,0,1,1292,1108)" font-family="MS Shell Dlg 2" font-size="9.75" font-weight="400" font-style="normal" > <text fill="#000000" fill-opacity="1" stroke="none" xml:space="preserve" x="4" y="17" font-family="Arial" font-size="10pt" font-weight="700" font-style="italic" >nad6</text> </g> <g fill="#ff9999" fill-opacity="1" stroke="#bfbfbf" stroke-opacity="1" stroke-width="1" stroke-linecap="square" stroke-linejoin="bevel" transform="matrix(1,0,0,1,1336,1110)" font-family="MS Shell Dlg 2" font-size="9.75" font-weight="400" font-style="normal" > <path vector-effect="none" fill-rule="evenodd" d="M0,1.7 L42.5,1.7 L42.5,0 L50,10 L42.5,20 L42.5,18.3 L0,18.3 L0,1.7"/> </g> <g fill="none" stroke="#000000" stroke-opacity="1" stroke-width="1" stroke-linecap="square" stroke-linejoin="bevel" transform="matrix(1,0,0,1,1349.13,1108)" font-family="MS Shell Dlg 2" font-size="9.75" font-weight="400" font-style="normal" > <text fill="#000000" fill-opacity="1" stroke="none" xml:space="preserve" x="4" y="17" font-family="Arial" font-size="10pt" font-weight="700" font-style="italic" >D</text> </g> <g fill="#ffff33" fill-opacity="1" stroke="#bfbfbf" stroke-opacity="1" stroke-width="1" stroke-linecap="square" stroke-linejoin="bevel" transform="matrix(1,0,0,1,1387,1110)" font-family="MS Shell Dlg 2" font-size="9.75" font-weight="400" font-style="normal" > <rect x="0" y="0" width="50" height="20"/> </g> <g fill="none" stroke="#000000" stroke-opacity="1" stroke-width="1" stroke-linecap="square" stroke-linejoin="bevel" transform="matrix(1,0,0,1,1395.5,1108)" font-family="MS Shell Dlg 2" font-size="9.75" font-weight="400" font-style="normal" > <text fill="#000000" fill-opacity="1" stroke="none" xml:space="preserve" x="4" y="17" font-family="Arial" font-size="10pt" font-weight="700" font-style="italic" >atp6</text> </g> <g fill="#ff9999" fill-opacity="1" stroke="#bfbfbf" stroke-opacity="1" stroke-width="1" stroke-linecap="square" stroke-linejoin="bevel" transform="matrix(1,0,0,1,1438,1110)" font-family="MS Shell Dlg 2" font-size="9.75" font-weight="400" font-style="normal" > <path vector-effect="none" fill-rule="evenodd" d="M0,1.7 L42.5,1.7 L42.5,0 L50,10 L42.5,20 L42.5,18.3 L0,18.3 L0,1.7"/> </g> <g fill="none" stroke="#000000" stroke-opacity="1" stroke-width="1" stroke-linecap="square" stroke-linejoin="bevel" transform="matrix(1,0,0,1,1447.63,1108)" font-family="MS Shell Dlg 2" font-size="9.75" font-weight="400" font-style="normal" > <text fill="#000000" fill-opacity="1" stroke="none" xml:space="preserve" x="4" y="17" font-family="Arial" font-size="10pt" font-weight="700" font-style="italic" >S1</text> </g> <g fill="#ffff33" fill-opacity="1" stroke="#bfbfbf" stroke-opacity="1" stroke-width="1" stroke-linecap="square" stroke-linejoin="bevel" transform="matrix(1,0,0,1,1489,1110)" font-family="MS Shell Dlg 2" font-size="9.75" font-weight="400" font-style="normal" > <rect x="0" y="0" width="50" height="20"/> </g> <g fill="none" stroke="#000000" stroke-opacity="1" stroke-width="1" stroke-linecap="square" stroke-linejoin="bevel" transform="matrix(1,0,0,1,1496,1108)" font-family="MS Shell Dlg 2" font-size="9.75" font-weight="400" font-style="normal" > <text fill="#000000" fill-opacity="1" stroke="none" xml:space="preserve" x="4" y="17" font-family="Arial" font-size="10pt" font-weight="700" font-style="italic" >nad1</text> </g> <g fill="#ff9999" fill-opacity="1" stroke="#bfbfbf" stroke-opacity="1" stroke-width="1" stroke-linecap="square" stroke-linejoin="bevel" transform="matrix(1,0,0,1,1540,1110)" font-family="MS Shell Dlg 2" font-size="9.75" font-weight="400" font-style="normal" > <path vector-effect="none" fill-rule="evenodd" d="M0,1.7 L42.5,1.7 L42.5,0 L50,10 L42.5,20 L42.5,18.3 L0,18.3 L0,1.7"/> </g> <g fill="none" stroke="#000000" stroke-opacity="1" stroke-width="1" stroke-linecap="square" stroke-linejoin="bevel" transform="matrix(1,0,0,1,1553.13,1108)" font-family="MS Shell Dlg 2" font-size="9.75" font-weight="400" font-style="normal" > <text fill="#000000" fill-opacity="1" stroke="none" xml:space="preserve" x="4" y="17" font-family="Arial" font-size="10pt" font-weight="700" font-style="italic" >G</text> </g> <g fill="#99ffff" fill-opacity="1" stroke="#bfbfbf" stroke-opacity="1" stroke-width="1" stroke-linecap="square" stroke-linejoin="bevel" transform="matrix(1,0,0,1,1591,1110)" font-family="MS Shell Dlg 2" font-size="9.75" font-weight="400" font-style="normal" > <path vector-effect="none" fill-rule="evenodd" d="M0,5 C0,2.23858 2.23858,0 5,0 L45,0 C47.7614,0 50,2.23858 50,5 L50,15 C50,17.7614 47.7614,20 45,20 L5,20 C2.23858,20 0,17.7614 0,15 L0,5"/> </g> <g fill="none" stroke="#000000" stroke-opacity="1" stroke-width="1" stroke-linecap="square" stroke-linejoin="bevel" transform="matrix(1,0,0,1,1599.5,1108)" font-family="MS Shell Dlg 2" font-size="9.75" font-weight="400" font-style="normal" > <text fill="#000000" fill-opacity="1" stroke="none" xml:space="preserve" x="4" y="17" font-family="Arial" font-size="10pt" font-weight="700" font-style="italic" >rrnS</text> </g> <g fill="#ff9999" fill-opacity="1" stroke="#bfbfbf" stroke-opacity="1" stroke-width="1" stroke-linecap="square" stroke-linejoin="bevel" transform="matrix(1,0,0,1,1642,1110)" font-family="MS Shell Dlg

2" font-size="9.75" font-weight="400" font-style="normal" > <path vector-effect="none" fill-rule="evenodd" d="M0,1.7 L42.5,1.7 L42.5,0 L50,10 L42.5,20 L42.5,18.3 L0,18.3 L0,1.7"/> </g> <g fill="none" stroke="#000000" stroke-opacity="1" stroke-width="1" stroke-linecap="square" stroke-linejoin="bevel" transform="matrix(1,0,0,1,1654.63,1108)" font-family="MS Shell Dlg 2" font-size="9.75" font-weight="400" font-style="normal" > <text fill="#000000" fill-opacity="1" stroke="none" xml:space="preserve" x="4" y="17" font-family="Arial" font-size="10pt" font-weight="700" font-style="italic" >Q</text> </g> <g fill="#ffff33" fill-opacity="1" stroke="#bfbfbf" stroke-opacity="1" stroke-width="1" stroke-linecap="square" stroke-linejoin="bevel" transform="matrix(1,0,0,1,1693,1110)" font-family="MS Shell Dlg 2" font-size="9.75" font-weight="400" font-style="normal" > <rect x="0" y="0" width="50" height="20"/> </g> <g fill="none" stroke="#000000" stroke-opacity="1" stroke-width="1" stroke-linecap="square" stroke-linejoin="bevel" transform="matrix(1,0,0,1,1701,1108)" font-family="MS Shell Dlg 2" font-size="9.75" font-weight="400" font-style="normal" > <text fill="#000000" fill-opacity="1" stroke="none" xml:space="preserve" x="4" y="17" font-family="Arial" font-size="10pt" font-weight="700" font-style="italic" >cox2</text> </g> <g fill="#ff9999" fill-opacity="1" stroke="#bfbfbf" stroke-opacity="1" stroke-width="1" stroke-linecap="square" stroke-linejoin="bevel" transform="matrix(1,0,0,1,1744,1110)" font-family="MS Shell Dlg 2" font-size="9.75" font-weight="400" font-style="normal" > <path vector-effect="none" fill-rule="evenodd" d="M0,1.7 L42.5,1.7 L42.5,0 L50,10 L42.5,20 L42.5,18.3 L0,18.3 L0,1.7"/> </g> <g fill="none" stroke="#000000" stroke-opacity="1" stroke-width="1" stroke-linecap="square" stroke-linejoin="bevel" transform="matrix(1,0,0,1,1757.13,1108)" font-family="MS Shell Dlg 2" font-size="9.75" font-weight="400" font-style="normal" > <text fill="#000000" fill-opacity="1" stroke="none" xml:space="preserve" x="4" y="17" font-family="Arial" font-size="10pt" font-weight="700" font-style="italic" >T</text> </g> <g fill="#ffff33" fill-opacity="1" stroke="#bfbfbf" stroke-opacity="1" stroke-width="1" stroke-linecap="square" stroke-linejoin="bevel" transform="matrix(1,0,0,1,1795,1110)" font-family="MS Shell Dlg 2" font-size="9.75" font-weight="400" font-style="normal" > <rect x="0" y="0" width="50" height="20"/> </g> <g fill="none" stroke="#000000" stroke-opacity="1" stroke-width="1" stroke-linecap="square" stroke-linejoin="bevel" transform="matrix(1,0,0,1,1803.5,1108)" font-family="MS Shell Dlg 2" font-size="9.75" font-weight="400" font-style="normal" > <text fill="#000000" fill-opacity="1" stroke="none" xml:space="preserve" x="4" y="17" font-family="Arial" font-size="10pt" font-weight="700" font-style="italic" >atp8</text> </g> <g fill="#ffff33" fill-opacity="1" stroke="#bfbfbf" stroke-opacity="1" stroke-width="1" stroke-linecap="square" stroke-linejoin="bevel" transform="matrix(1,0,0,1,1846,1110)" font-family="MS Shell Dlg 2" font-size="9.75" font-weight="400" font-style="normal" > <rect x="0" y="0" width="50" height="20"/> </g> <g fill="none" stroke="#000000" stroke-opacity="1" stroke-width="1" stroke-linecap="square" stroke-linejoin="bevel" transform="matrix(1,0,0,1,1855.5,1108)" font-family="MS Shell Dlg 2" font-size="9.75" font-weight="400" font-style="normal" > <text fill="#000000" fill-opacity="1" stroke="none" xml:space="preserve" x="4" y="17" font-family="Arial" font-size="10pt" font-weight="700" font-style="italic" >cytb</text> </g> <g fill="#ff9999" fill-opacity="1" stroke="#bfbfbf" stroke-opacity="1" stroke-width="1" stroke-linecap="square" stroke-linejoin="bevel" transform="matrix(1,0,0,1,1897,1110)" font-family="MS Shell Dlg 2" font-size="9.75" font-weight="400" font-style="normal" > <path vector-effect="none" fill-rule="evenodd" d="M0,1.7 L42.5,1.7 L42.5,0 L50,10 L42.5,20 L42.5,18.3 L0,18.3 L0,1.7"/> </g> <g fill="none" stroke="#000000" stroke-opacity="1" stroke-width="1" stroke-linecap="square" stroke-linejoin="bevel" transform="matrix(1,0,0,1,1910.13,1108)" font-family="MS Shell Dlg 2" font-size="9.75" font-weight="400" font-style="normal" > <text fill="#000000" fill-opacity="1" stroke="none" xml:space="preserve" x="4" y="17" font-family="Arial" font-size="10pt" font-weight="700" font-style="italic" >H</text> </g> <g fill="none" stroke="#000000" stroke-opacity="1" stroke-width="1" stroke-linecap="square" stroke-linejoin="bevel" transform="matrix(1,0,0,1,1948,1110)" font-family="MS Shell Dlg 2" font-size="9.75" font-weight="400" font-style="normal" > <text fill="#000000" fill-opacity="1" stroke="none" xml:space="preserve" x="4" y="17" font-family="Arial" font-size="10pt" font-weight="700" font-style="italic" >(+)</text> </g> <g fill="none" stroke="#000000" stroke-opacity="1" stroke-width="1" stroke-linecap="square" stroke-linejoin="bevel" transform="matrix(1,0,0,1,10,1150)" font-family="MS Shell Dlg 2" font-size="9.75" font-weight="400" font-style="normal" > <text fill="#000000" fill-opacity="1" stroke="none" xml:space="preserve" x="4" y="17" font-family="Arial" font-size="10pt" font-weight="700" font-style="italic" >Perna_perna_OK576481</text> </g> <g fill="#ffff33" fill-opacity="1" stroke="#bfbfbf" stroke-opacity="1" stroke-width="1" stroke-linecap="square" stroke-linejoin="bevel" transform="matrix(1,0,0,1,10,1180)" font-family="MS Shell Dlg 2" font-size="9.75" font-weight="400" font-style="normal" > <rect x="0" y="0" width="50" height="20"/> </g> <g fill="none" stroke="#000000" stroke-opacity="1" stroke-width="1" stroke-linecap="square" stroke-linejoin="bevel" transform="matrix(1,0,0,1,18,1178)" font-family="MS Shell Dlg 2" font-size="9.75" font-weight="400" font-style="normal" > <text fill="#000000" fill-opacity="1" stroke="none" xml:space="preserve" x="4" y="17" font-family="Arial" font-size="10pt" font-weight="700" font-style="italic" >cox1</text> </g> <g fill="#ffff33"

fill-opacity="1" stroke="#bfbfbf" stroke-opacity="1" stroke-width="1" stroke-linecap="square" stroke-linejoin="bevel" transform="matrix(1,0,0,1,61,1180)" font-family="MS Shell Dlg 2" font-size="9.75" font-weight="400" font-style="normal" > <rect x="0" y="0" width="50" height="20"/> </g> <g fill="none" stroke="#000000" stroke-opacity="1" stroke-width="1" stroke-linecap="square" stroke-linejoin="bevel" transform="matrix(1,0,0,1,69.5,1178)" font-family="MS Shell Dlg 2" font-size="9.75" font-weight="400" font-style="normal" > <text fill="#000000" fill-opacity="1" stroke="none" xml:space="preserve" x="4" y="17" font-family="Arial" font-size="10pt" font-weight="700" font-style="italic" >atp6</text> </g> <g fill="#ffff33" fill-opacity="1" stroke="#bfbfbf" stroke-opacity="1" stroke-width="1" stroke-linecap="square" stroke-linejoin="bevel" transform="matrix(1,0,0,1,112,1180)" font-family="MS Shell Dlg 2" font-size="9.75" font-weight="400" font-style="normal" > <rect x="0" y="0" width="50" height="20"/> </g> <g fill="none" stroke="#000000" stroke-opacity="1" stroke-width="1" stroke-linecap="square" stroke-linejoin="bevel" transform="matrix(1,0,0,1,121.5,1178)" font-family="MS Shell Dlg 2" font-size="9.75" font-weight="400" font-style="normal" > <text fill="#000000" fill-opacity="1" stroke="none" xml:space="preserve" x="4" y="17" font-family="Arial" font-size="10pt" font-weight="700" font-style="italic" >cytb</text> </g> <g fill="#ffff33" fill-opacity="1" stroke="#bfbfbf" stroke-opacity="1" stroke-width="1" stroke-linecap="square" stroke-linejoin="bevel" transform="matrix(1,0,0,1,163,1180)" font-family="MS Shell Dlg 2" font-size="9.75" font-weight="400" font-style="normal" > <rect x="0" y="0" width="50" height="20"/> </g> <g fill="none" stroke="#000000" stroke-opacity="1" stroke-width="1" stroke-linecap="square" stroke-linejoin="bevel" transform="matrix(1,0,0,1,171,1178)" font-family="MS Shell Dlg 2" font-size="9.75" font-weight="400" font-style="normal" > <text fill="#000000" fill-opacity="1" stroke="none" xml:space="preserve" x="4" y="17" font-family="Arial" font-size="10pt" font-weight="700" font-style="italic" >cox2</text> </g> <g fill="#ff9999" fill-opacity="1" stroke="#bfbfbf" stroke-opacity="1" stroke-width="1" stroke-linecap="square" stroke-linejoin="bevel" transform="matrix(1,0,0,1,214,1180)" font-family="MS Shell Dlg 2" font-size="9.75" font-weight="400" font-style="normal" > <path vector-effect="none" fill-rule="evenodd" d="M0,1.7 L42.5,1.7 L42.5,0 L50,10 L42.5,20 L42.5,18.3 L0,18.3 L0,1.7"/> </g> <g fill="none" stroke="#000000" stroke-opacity="1" stroke-width="1" stroke-linecap="square" stroke-linejoin="bevel" transform="matrix(1,0,0,1,227.132,1178)" font-family="MS Shell Dlg 2" font-size="9.75" font-weight="400" font-style="normal" > <text fill="#000000" fill-opacity="1" stroke="none" xml:space="preserve" x="4" y="17" font-family="Arial" font-size="10pt" font-weight="700" font-style="italic" >R</text> </g> <g fill="#ff9999" fill-opacity="1" stroke="#bfbfbf" stroke-opacity="1" stroke-width="1" stroke-linecap="square" stroke-linejoin="bevel" transform="matrix(1,0,0,1,265,1180)" font-family="MS Shell Dlg 2" font-size="9.75" font-weight="400" font-style="normal" > <path vector-effect="none" fill-rule="evenodd" d="M0,1.7 L42.5,1.7 L42.5,0 L50,10 L42.5,20 L42.5,18.3 L0,18.3 L0,1.7"/> </g> <g fill="none" stroke="#000000" stroke-opacity="1" stroke-width="1" stroke-linecap="square" stroke-linejoin="bevel" transform="matrix(1,0,0,1,277.132,1178)" font-family="MS Shell Dlg 2" font-size="9.75" font-weight="400" font-style="normal" > <text fill="#000000" fill-opacity="1" stroke="none" xml:space="preserve" x="4" y="17" font-family="Arial" font-size="10pt" font-weight="700" font-style="italic" >M</text> </g> <g fill="#ff9999" fill-opacity="1" stroke="#bfbfbf" stroke-opacity="1" stroke-width="1" stroke-linecap="square" stroke-linejoin="bevel" transform="matrix(1,0,0,1,316,1180)" font-family="MS Shell Dlg 2" font-size="9.75" font-weight="400" font-style="normal" > <path vector-effect="none" fill-rule="evenodd" d="M0,1.7 L42.5,1.7 L42.5,0 L50,10 L42.5,20 L42.5,18.3 L0,18.3 L0,1.7"/> </g> <g fill="none" stroke="#000000" stroke-opacity="1" stroke-width="1" stroke-linecap="square" stroke-linejoin="bevel" transform="matrix(1,0,0,1,329.132,1178)" font-family="MS Shell Dlg 2" font-size="9.75" font-weight="400" font-style="normal" > <text fill="#000000" fill-opacity="1" stroke="none" xml:space="preserve" x="4" y="17" font-family="Arial" font-size="10pt" font-weight="700" font-style="italic" >T</text> </g> <g fill="#ff9999" fill-opacity="1" stroke="#bfbfbf" stroke-opacity="1" stroke-width="1" stroke-linecap="square" stroke-linejoin="bevel" transform="matrix(1,0,0,1,367,1180)" font-family="MS Shell Dlg 2" font-size="9.75" font-weight="400" font-style="normal" > <path vector-effect="none" fill-rule="evenodd" d="M0,1.7 L42.5,1.7 L42.5,0 L50,10 L42.5,20 L42.5,18.3 L0,18.3 L0,1.7"/> </g> <g fill="none" stroke="#000000" stroke-opacity="1" stroke-width="1" stroke-linecap="square" stroke-linejoin="bevel" transform="matrix(1,0,0,1,380.632,1178)" font-family="MS Shell Dlg 2" font-size="9.75" font-weight="400" font-style="normal" > <text fill="#000000" fill-opacity="1" stroke="none" xml:space="preserve" x="4" y="17" font-family="Arial" font-size="10pt" font-weight="700" font-style="italic" >E</text> </g> <g fill="#ff9999" fill-opacity="1" stroke="#bfbfbf" stroke-opacity="1" stroke-width="1" stroke-linecap="square" stroke-linejoin="bevel" transform="matrix(1,0,0,1,418,1180)" font-family="MS Shell Dlg 2" font-size="9.75" font-weight="400" font-style="normal" > <path vector-effect="none" fill-rule="evenodd" d="M0,1.7 L42.5,1.7 L42.5,0 L50,10 L42.5,20 L42.5,18.3 L0,18.3 L0,1.7"/> </g> <g fill="none" stroke="#000000" stroke-opacity="1" stroke-width="1" stroke-linecap="square" stroke-linejoin="bevel" transform="matrix(1,0,0,1,430.632,1178)" font-family="MS Shell Dlg 2" font-size="9.75" font-weight="400" font-style="normal" > <text fill="#000000" fill-opacity="1"

stroke="none" xml:space="preserve" x="4" y="17" font-family="Arial" font-size="10pt" font-weight="700" font-style="italic" >Q</text> </g> <g fill="#ff9999" fill-opacity="1" stroke="#bfbfbf" stroke-opacity="1" stroke-width="1" stroke-linecap="square" stroke-linejoin="bevel" transform="matrix(1,0,0,1,469,1180)" font-family="MS Shell Dlg 2" font-size="9.75" font-weight="400" font-style="normal" > <path vector-effect="none" fill-rule="evenodd" d="M0,1.7 L42.5,1.7 L42.5,0 L50,10 L42.5,20 L42.5,18.3 L0,18.3 L0,1.7"/> </g> <g fill="none" stroke="#000000" stroke-opacity="1" stroke-width="1" stroke-linecap="square" stroke-linejoin="bevel" transform="matrix(1,0,0,1,478.632,1178)" font-family="MS Shell Dlg 2" font-size="9.75" font-weight="400" font-style="normal" > <text fill="#000000" fill-opacity="1" stroke="none" xml:space="preserve" x="4" y="17" font-family="Arial" font-size="10pt" font-weight="700" font-style="italic" >S2</text> </g> <g fill="#ff9999" fill-opacity="1" stroke="#bfbfbf" stroke-opacity="1" stroke-width="1" stroke-linecap="square" stroke-linejoin="bevel" transform="matrix(1,0,0,1,520,1180)" font-family="MS Shell Dlg 2" font-size="9.75" font-weight="400" font-style="normal" > <path vector-effect="none" fill-rule="evenodd" d="M0,1.7 L42.5,1.7 L42.5,0 L50,10 L42.5,20 L42.5,18.3 L0,18.3 L0,1.7"/> </g> <g fill="none" stroke="#000000" stroke-opacity="1" stroke-width="1" stroke-linecap="square" stroke-linejoin="bevel" transform="matrix(1,0,0,1,533.133,1178)" font-family="MS Shell Dlg 2" font-size="9.75" font-weight="400" font-style="normal" > <text fill="#000000" fill-opacity="1" stroke="none" xml:space="preserve" x="4" y="17" font-family="Arial" font-size="10pt" font-weight="700" font-style="italic" >D</text> </g> <g fill="#ffff33" fill-opacity="1" stroke="#bfbfbf" stroke-opacity="1" stroke-width="1" stroke-linecap="square" stroke-linejoin="bevel" transform="matrix(1,0,0,1,571,1180)" font-family="MS Shell Dlg 2" font-size="9.75" font-weight="400" font-style="normal" > <rect x="0" y="0" width="50" height="20"/> </g> <g fill="none" stroke="#000000" stroke-opacity="1" stroke-width="1" stroke-linecap="square" stroke-linejoin="bevel" transform="matrix(1,0,0,1,578,1178)" font-family="MS Shell Dlg 2" font-size="9.75" font-weight="400" font-style="normal" > <text fill="#000000" fill-opacity="1" stroke="none" xml:space="preserve" x="4" y="17" font-family="Arial" font-size="10pt" font-weight="700" font-style="italic" >nad1</text> </g> <g fill="#ff9999" fill-opacity="1" stroke="#bfbfbf" stroke-opacity="1" stroke-width="1" stroke-linecap="square" stroke-linejoin="bevel" transform="matrix(1,0,0,1,622,1180)" font-family="MS Shell Dlg 2" font-size="9.75" font-weight="400" font-style="normal" > <path vector-effect="none" fill-rule="evenodd" d="M0,1.7 L42.5,1.7 L42.5,0 L50,10 L42.5,20 L42.5,18.3 L0,18.3 L0,1.7"/> </g> <g fill="none" stroke="#000000" stroke-opacity="1" stroke-width="1" stroke-linecap="square" stroke-linejoin="bevel" transform="matrix(1,0,0,1,635.633,1178)" font-family="MS Shell Dlg 2" font-size="9.75" font-weight="400" font-style="normal" > <text fill="#000000" fill-opacity="1" stroke="none" xml:space="preserve" x="4" y="17" font-family="Arial" font-size="10pt" font-weight="700" font-style="italic" >P</text> </g> <g fill="#ffff33" fill-opacity="1" stroke="#bfbfbf" stroke-opacity="1" stroke-width="1" stroke-linecap="square" stroke-linejoin="bevel" transform="matrix(1,0,0,1,673,1180)" font-family="MS Shell Dlg 2" font-size="9.75" font-weight="400" font-style="normal" > <rect x="0" y="0" width="50" height="20"/> </g> <g fill="none" stroke="#000000" stroke-opacity="1" stroke-width="1" stroke-linecap="square" stroke-linejoin="bevel" transform="matrix(1,0,0,1,677,1178)" font-family="MS Shell Dlg 2" font-size="9.75" font-weight="400" font-style="normal" > <text fill="#000000" fill-opacity="1" stroke="none" xml:space="preserve" x="4" y="17" font-family="Arial" font-size="10pt" font-weight="700" font-style="italic" >nad4L</text> </g> <g fill="#ff9999" fill-opacity="1" stroke="#bfbfbf" stroke-opacity="1" stroke-width="1" stroke-linecap="square" stroke-linejoin="bevel" transform="matrix(1,0,0,1,724,1180)" font-family="MS Shell Dlg 2" font-size="9.75" font-weight="400" font-style="normal" > <path vector-effect="none" fill-rule="evenodd" d="M0,1.7 L42.5,1.7 L42.5,0 L50,10 L42.5,20 L42.5,18.3 L0,18.3 L0,1.7"/> </g> <g fill="none" stroke="#000000" stroke-opacity="1" stroke-width="1" stroke-linecap="square" stroke-linejoin="bevel" transform="matrix(1,0,0,1,737.633,1178)" font-family="MS Shell Dlg 2" font-size="9.75" font-weight="400" font-style="normal" > <text fill="#000000" fill-opacity="1" stroke="none" xml:space="preserve" x="4" y="17" font-family="Arial" font-size="10pt" font-weight="700" font-style="italic" >Y</text> </g> <g fill="#ffff33" fill-opacity="1" stroke="#bfbfbf" stroke-opacity="1" stroke-width="1" stroke-linecap="square" stroke-linejoin="bevel" transform="matrix(1,0,0,1,775,1180)" font-family="MS Shell Dlg 2" font-size="9.75" font-weight="400" font-style="normal" > <rect x="0" y="0" width="50" height="20"/> </g> <g fill="none" stroke="#000000" stroke-opacity="1" stroke-width="1" stroke-linecap="square" stroke-linejoin="bevel" transform="matrix(1,0,0,1,782,1178)" font-family="MS Shell Dlg 2" font-size="9.75" font-weight="400" font-style="normal" > <text fill="#000000" fill-opacity="1" stroke="none" xml:space="preserve" x="4" y="17" font-family="Arial" font-size="10pt" font-weight="700" font-style="italic" >nad3</text> </g> <g fill="#ff9999" fill-opacity="1" stroke="#bfbfbf" stroke-opacity="1" stroke-width="1" stroke-linecap="square" stroke-linejoin="bevel" transform="matrix(1,0,0,1,826,1180)" font-family="MS Shell Dlg 2" font-size="9.75" font-weight="400" font-style="normal" > <path vector-effect="none" fill-rule="evenodd" d="M0,1.7 L42.5,1.7 L42.5,0 L50,10 L42.5,20 L42.5,18.3 L0,18.3 L0,1.7"/> </g> <g fill="none" stroke="#000000" stroke-opacity="1"

stroke-width="1" stroke-linecap="square" stroke-linejoin="bevel" transform="matrix(1,0,0,1,836.633,1178)" font-family="MS Shell Dlg 2" font-size="9.75" font-weight="400" font-style="normal" > <text fill="#000000" fill-opacity="1" stroke="none" xml:space="preserve" x="4" y="17" font-family="Arial" font-size="10pt" font-weight="700" font-style="italic" >L1</text> </g> <g fill="#ff9999" fill-opacity="1" stroke="#bfbfbf" stroke-opacity="1" stroke-width="1" stroke-linecap="square" stroke-linejoin="bevel" transform="matrix(1,0,0,1,877,1180)" font-family="MS Shell Dlg 2" font-size="9.75" font-weight="400" font-style="normal" > <path vector-effect="none" fill-rule="evenodd" d="M0,1.7 L42.5,1.7 L42.5,0 L50,10 L42.5,20 L42.5,18.3 L0,18.3 L0,1.7"/> </g> <g fill="none" stroke="#000000" stroke-opacity="1" stroke-width="1" stroke-linecap="square" stroke-linejoin="bevel" transform="matrix(1,0,0,1,890.133,1178)" font-family="MS Shell Dlg 2" font-size="9.75" font-weight="400" font-style="normal" > <text fill="#000000" fill-opacity="1" stroke="none" xml:space="preserve" x="4" y="17" font-family="Arial" font-size="10pt" font-weight="700" font-style="italic" >N</text> </g> <g fill="#ffff33" fill-opacity="1" stroke="#bfbfbf" stroke-opacity="1" stroke-width="1" stroke-linecap="square" stroke-linejoin="bevel" transform="matrix(1,0,0,1,928,1180)" font-family="MS Shell Dlg 2" font-size="9.75" font-weight="400" font-style="normal" > <rect x="0" y="0" width="50" height="20"/> </g> <g fill="none" stroke="#000000" stroke-opacity="1" stroke-width="1" stroke-linecap="square" stroke-linejoin="bevel" transform="matrix(1,0,0,1,935,1178)" font-family="MS Shell Dlg 2" font-size="9.75" font-weight="400" font-style="normal" > <text fill="#000000" fill-opacity="1" stroke="none" xml:space="preserve" x="4" y="17" font-family="Arial" font-size="10pt" font-weight="700" font-style="italic" >nad6</text> </g> <g fill="#ff9999" fill-opacity="1" stroke="#bfbfbf" stroke-opacity="1" stroke-width="1" stroke-linecap="square" stroke-linejoin="bevel" transform="matrix(1,0,0,1,979,1180)" font-family="MS Shell Dlg 2" font-size="9.75" font-weight="400" font-style="normal" > <path vector-effect="none" fill-rule="evenodd" d="M0,1.7 L42.5,1.7 L42.5,0 L50,10 L42.5,20 L42.5,18.3 L0,18.3 L0,1.7"/> </g> <g fill="none" stroke="#000000" stroke-opacity="1" stroke-width="1" stroke-linecap="square" stroke-linejoin="bevel" transform="matrix(1,0,0,1,992.133,1178)" font-family="MS Shell Dlg 2" font-size="9.75" font-weight="400" font-style="normal" > <text fill="#000000" fill-opacity="1" stroke="none" xml:space="preserve" x="4" y="17" font-family="Arial" font-size="10pt" font-weight="700" font-style="italic" >C</text> </g> <g fill="#ff9999" fill-opacity="1" stroke="#bfbfbf" stroke-opacity="1" stroke-width="1" stroke-linecap="square" stroke-linejoin="bevel" transform="matrix(1,0,0,1,1030,1180)" font-family="MS Shell Dlg 2" font-size="9.75" font-weight="400" font-style="normal" > <path vector-effect="none" fill-rule="evenodd" d="M0,1.7 L42.5,1.7 L42.5,0 L50,10 L42.5,20 L42.5,18.3 L0,18.3 L0,1.7"/> </g> <g fill="none" stroke="#000000" stroke-opacity="1" stroke-width="1" stroke-linecap="square" stroke-linejoin="bevel" transform="matrix(1,0,0,1,1041.13,1178)" font-family="MS Shell Dlg 2" font-size="9.75" font-weight="400" font-style="normal" > <text fill="#000000" fill-opacity="1" stroke="none" xml:space="preserve" x="4" y="17" font-family="Arial" font-size="10pt" font-weight="700" font-style="italic" >W</text> </g> <g fill="#ff9999" fill-opacity="1" stroke="#bfbfbf" stroke-opacity="1" stroke-width="1" stroke-linecap="square" stroke-linejoin="bevel" transform="matrix(1,0,0,1,1081,1180)" font-family="MS Shell Dlg 2" font-size="9.75" font-weight="400" font-style="normal" > <path vector-effect="none" fill-rule="evenodd" d="M0,1.7 L42.5,1.7 L42.5,0 L50,10 L42.5,20 L42.5,18.3 L0,18.3 L0,1.7"/> </g> <g fill="none" stroke="#000000" stroke-opacity="1" stroke-width="1" stroke-linecap="square" stroke-linejoin="bevel" transform="matrix(1,0,0,1,1094.63,1178)" font-family="MS Shell Dlg 2" font-size="9.75" font-weight="400" font-style="normal" > <text fill="#000000" fill-opacity="1" stroke="none" xml:space="preserve" x="4" y="17" font-family="Arial" font-size="10pt" font-weight="700" font-style="italic" >K</text> </g> <g fill="#ffff33" fill-opacity="1" stroke="#bfbfbf" stroke-opacity="1" stroke-width="1" stroke-linecap="square" stroke-linejoin="bevel" transform="matrix(1,0,0,1,1132,1180)" font-family="MS Shell Dlg 2" font-size="9.75" font-weight="400" font-style="normal" > <rect x="0" y="0" width="50" height="20"/> </g> <g fill="none" stroke="#000000" stroke-opacity="1" stroke-width="1" stroke-linecap="square" stroke-linejoin="bevel" transform="matrix(1,0,0,1,1139,1178)" font-family="MS Shell Dlg 2" font-size="9.75" font-weight="400" font-style="normal" > <text fill="#000000" fill-opacity="1" stroke="none" xml:space="preserve" x="4" y="17" font-family="Arial" font-size="10pt" font-weight="700" font-style="italic" >nad5</text> </g> <g fill="#ff9999" fill-opacity="1" stroke="#bfbfbf" stroke-opacity="1" stroke-width="1" stroke-linecap="square" stroke-linejoin="bevel" transform="matrix(1,0,0,1,1183,1180)" font-family="MS Shell Dlg 2" font-size="9.75" font-weight="400" font-style="normal" > <path vector-effect="none" fill-rule="evenodd" d="M0,1.7 L42.5,1.7 L42.5,0 L50,10 L42.5,20 L42.5,18.3 L0,18.3 L0,1.7"/> </g> <g fill="none" stroke="#000000" stroke-opacity="1" stroke-width="1" stroke-linecap="square" stroke-linejoin="bevel" transform="matrix(1,0,0,1,1192.63,1178)" font-family="MS Shell Dlg 2" font-size="9.75" font-weight="400" font-style="normal" > <text fill="#000000" fill-opacity="1" stroke="none" xml:space="preserve" x="4" y="17" font-family="Arial" font-size="10pt" font-weight="700" font-style="italic" >S1</text> </g> <g fill="#ff9999" fill-opacity="1" stroke="#bfbfbf" stroke-opacity="1" stroke-width="1" stroke-linecap="square" stroke-linejoin="bevel" transform="matrix(1,0,0,1,1234,1180)" font-family="MS Shell Dlg 2" font-size="9.75" font-

weight="400" font-style="normal" > <path vector-effect="none" fill-rule="evenodd" d="M0,1.7 L42.5,1.7 L42.5,0 L50,10 L42.5,20 L42.5,18.3 L0,18.3 L0,1.7"/> </g> <g fill="none" stroke="#000000" stroke-opacity="1" stroke-width="1" stroke-linecap="square" stroke-linejoin="bevel" transform="matrix(1,0,0,1,1247.13,1178)" font-family="MS Shell Dlg 2" font-size="9.75" font-weight="400" font-style="normal" > <text fill="#000000" fill-opacity="1" stroke="none" xml:space="preserve" x="4" y="17" font-family="Arial" font-size="10pt" font-weight="700" font-style="italic" >A</text> </g> <g fill="#ff9999" fill-opacity="1" stroke="#bfbfbf" stroke-opacity="1" stroke-width="1" stroke-linecap="square" stroke-linejoin="bevel" transform="matrix(1,0,0,1,1285,1180)" font-family="MS Shell Dlg 2" font-size="9.75" font-weight="400" font-style="normal" > <path vector-effect="none" fill-rule="evenodd" d="M0,1.7 L42.5,1.7 L42.5,0 L50,10 L42.5,20 L42.5,18.3 L0,18.3 L0,1.7"/> </g> <g fill="none" stroke="#000000" stroke-opacity="1" stroke-width="1" stroke-linecap="square" stroke-linejoin="bevel" transform="matrix(1,0,0,1,1295.63,1178)" font-family="MS Shell Dlg 2" font-size="9.75" font-weight="400" font-style="normal" > <text fill="#000000" fill-opacity="1" stroke="none" xml:space="preserve" x="4" y="17" font-family="Arial" font-size="10pt" font-weight="700" font-style="italic" >L2</text> </g> <g fill="#99ffff" fill-opacity="1" stroke="#bfbfbf" stroke-opacity="1" stroke-width="1" stroke-linecap="square" stroke-linejoin="bevel" transform="matrix(1,0,0,1,1336,1180)" font-family="MS Shell Dlg 2" font-size="9.75" font-weight="400" font-style="normal" > <path vector-effect="none" fill-rule="evenodd" d="M0,5 C0,2.23858 2.23858,0 5,0 L45,0 C47.7614,0 50,2.23858 50,5 L50,15 C50,17.7614 47.7614,20 45,20 L5,20 C2.23858,20 0,17.7614 0,15 L0,5"/> </g> <g fill="none" stroke="#000000" stroke-opacity="1" stroke-width="1" stroke-linecap="square" stroke-linejoin="bevel" transform="matrix(1,0,0,1,1344.5,1178)" font-family="MS Shell Dlg 2" font-size="9.75" font-weight="400" font-style="normal" > <text fill="#000000" fill-opacity="1" stroke="none" xml:space="preserve" x="4" y="17" font-family="Arial" font-size="10pt" font-weight="700" font-style="italic" >rrnS</text> </g> <g fill="#ff9999" fill-opacity="1" stroke="#bfbfbf" stroke-opacity="1" stroke-width="1" stroke-linecap="square" stroke-linejoin="bevel" transform="matrix(1,0,0,1,1387,1180)" font-family="MS Shell Dlg 2" font-size="9.75" font-weight="400" font-style="normal" > <path vector-effect="none" fill-rule="evenodd" d="M0,1.7 L42.5,1.7 L42.5,0 L50,10 L42.5,20 L42.5,18.3 L0,18.3 L0,1.7"/> </g> <g fill="none" stroke="#000000" stroke-opacity="1" stroke-width="1" stroke-linecap="square" stroke-linejoin="bevel" transform="matrix(1,0,0,1,1402.13,1178)" font-family="MS Shell Dlg 2" font-size="9.75" font-weight="400" font-style="normal" > <text fill="#000000" fill-opacity="1" stroke="none" xml:space="preserve" x="4" y="17" font-family="Arial" font-size="10pt" font-weight="700" font-style="italic" >I</text> </g> <g fill="#ff9999" fill-opacity="1" stroke="#bfbfbf" stroke-opacity="1" stroke-width="1" stroke-linecap="square" stroke-linejoin="bevel" transform="matrix(1,0,0,1,1438,1180)" font-family="MS Shell Dlg 2" font-size="9.75" font-weight="400" font-style="normal" > <path vector-effect="none" fill-rule="evenodd" d="M0,1.7 L42.5,1.7 L42.5,0 L50,10 L42.5,20 L42.5,18.3 L0,18.3 L0,1.7"/> </g> <g fill="none" stroke="#000000" stroke-opacity="1" stroke-width="1" stroke-linecap="square" stroke-linejoin="bevel" transform="matrix(1,0,0,1,1451.13,1178)" font-family="MS Shell Dlg 2" font-size="9.75" font-weight="400" font-style="normal" > <text fill="#000000" fill-opacity="1" stroke="none" xml:space="preserve" x="4" y="17" font-family="Arial" font-size="10pt" font-weight="700" font-style="italic" >G</text> </g> <g fill="#ff9999" fill-opacity="1" stroke="#bfbfbf" stroke-opacity="1" stroke-width="1" stroke-linecap="square" stroke-linejoin="bevel" transform="matrix(1,0,0,1,1489,1180)" font-family="MS Shell Dlg 2" font-size="9.75" font-weight="400" font-style="normal" > <path vector-effect="none" fill-rule="evenodd" d="M0,1.7 L42.5,1.7 L42.5,0 L50,10 L42.5,20 L42.5,18.3 L0,18.3 L0,1.7"/> </g> <g fill="none" stroke="#000000" stroke-opacity="1" stroke-width="1" stroke-linecap="square" stroke-linejoin="bevel" transform="matrix(1,0,0,1,1502.13,1178)" font-family="MS Shell Dlg 2" font-size="9.75" font-weight="400" font-style="normal" > <text fill="#000000" fill-opacity="1" stroke="none" xml:space="preserve" x="4" y="17" font-family="Arial" font-size="10pt" font-weight="700" font-style="italic" >V</text> </g> <g fill="#99ffff" fill-opacity="1" stroke="#bfbfbf" stroke-opacity="1" stroke-width="1" stroke-linecap="square" stroke-linejoin="bevel" transform="matrix(1,0,0,1,1540,1180)" font-family="MS Shell Dlg 2" font-size="9.75" font-weight="400" font-style="normal" > <path vector-effect="none" fill-rule="evenodd" d="M0,5 C0,2.23858 2.23858,0 5,0 L45,0 C47.7614,0 50,2.23858 50,5 L50,15 C50,17.7614 47.7614,20 45,20 L5,20 C2.23858,20 0,17.7614 0,15 L0,5"/> </g> <g fill="none" stroke="#000000" stroke-opacity="1" stroke-width="1" stroke-linecap="square" stroke-linejoin="bevel" transform="matrix(1,0,0,1,1549.5,1178)" font-family="MS Shell Dlg 2" font-size="9.75" font-weight="400" font-style="normal" > <text fill="#000000" fill-opacity="1" stroke="none" xml:space="preserve" x="4" y="17" font-family="Arial" font-size="10pt" font-weight="700" font-style="italic" >rrnL</text> </g> <g fill="#ff9999" fill-opacity="1" stroke="#bfbfbf" stroke-opacity="1" stroke-width="1" stroke-linecap="square" stroke-linejoin="bevel" transform="matrix(1,0,0,1,1591,1180)" font-family="MS Shell Dlg 2" font-size="9.75" font-weight="400" font-style="normal" > <path vector-effect="none" fill-rule="evenodd" d="M0,1.7 L42.5,1.7 L42.5,0 L50,10 L42.5,20 L42.5,18.3 L0,18.3 L0,1.7"/> </g> <g fill="none" stroke="#000000" stroke-

opacity="1" stroke-width="1" stroke-linecap="square" stroke-linejoin="bevel" transform="matrix(1,0,0,1,1603.13,1178)" font-family="MS Shell Dlg 2" font-size="9.75" font-weight="400" font-style="normal" > <text fill="#000000" fill-opacity="1" stroke="none" xml:space="preserve" x="4" y="17" font-family="Arial" font-size="10pt" font-weight="700" font-style="italic" >M</text> </g> <g fill="#ff9999" fill-opacity="1" stroke="#bfbfbf" stroke-opacity="1" stroke-width="1" stroke-linecap="square" stroke-linejoin="bevel" transform="matrix(1,0,0,1,1642,1180)" font-family="MS Shell Dlg 2" font-size="9.75" font-weight="400" font-style="normal" > <path vector-effect="none" fill-rule="evenodd" d="M0,1.7 L42.5,1.7 L42.5,0 L50,10 L42.5,20 L42.5,18.3 L0,18.3 L0,1.7"/> </g> <g fill="none" stroke="#000000" stroke-opacity="1" stroke-width="1" stroke-linecap="square" stroke-linejoin="bevel" transform="matrix(1,0,0,1,1655.63,1178)" font-family="MS Shell Dlg 2" font-size="9.75" font-weight="400" font-style="normal" > <text fill="#000000" fill-opacity="1" stroke="none" xml:space="preserve" x="4" y="17" font-family="Arial" font-size="10pt" font-weight="700" font-style="italic" >F</text> </g> <g fill="#ff9999" fill-opacity="1" stroke="#bfbfbf" stroke-opacity="1" stroke-width="1" stroke-linecap="square" stroke-linejoin="bevel" transform="matrix(1,0,0,1,1693,1180)" font-family="MS Shell Dlg 2" font-size="9.75" font-weight="400" font-style="normal" > <path vector-effect="none" fill-rule="evenodd" d="M0,1.7 L42.5,1.7 L42.5,0 L50,10 L42.5,20 L42.5,18.3 L0,18.3 L0,1.7"/> </g> <g fill="none" stroke="#000000" stroke-opacity="1" stroke-width="1" stroke-linecap="square" stroke-linejoin="bevel" transform="matrix(1,0,0,1,1706.13,1178)" font-family="MS Shell Dlg 2" font-size="9.75" font-weight="400" font-style="normal" > <text fill="#000000" fill-opacity="1" stroke="none" xml:space="preserve" x="4" y="17" font-family="Arial" font-size="10pt" font-weight="700" font-style="italic" >H</text> </g> <g fill="#ffff33" fill-opacity="1" stroke="#bfbfbf" stroke-opacity="1" stroke-width="1" stroke-linecap="square" stroke-linejoin="bevel" transform="matrix(1,0,0,1,1744,1180)" font-family="MS Shell Dlg 2" font-size="9.75" font-weight="400" font-style="normal" > <rect x="0" y="0" width="50" height="20"/> </g> <g fill="none" stroke="#000000" stroke-opacity="1" stroke-width="1" stroke-linecap="square" stroke-linejoin="bevel" transform="matrix(1,0,0,1,1752,1178)" font-family="MS Shell Dlg 2" font-size="9.75" font-weight="400" font-style="normal" > <text fill="#000000" fill-opacity="1" stroke="none" xml:space="preserve" x="4" y="17" font-family="Arial" font-size="10pt" font-weight="700" font-style="italic" >cox3</text> </g> <g fill="#ffff33" fill-opacity="1" stroke="#bfbfbf" stroke-opacity="1" stroke-width="1" stroke-linecap="square" stroke-linejoin="bevel" transform="matrix(1,0,0,1,1795,1180)" font-family="MS Shell Dlg 2" font-size="9.75" font-weight="400" font-style="normal" > <rect x="0" y="0" width="50" height="20"/> </g> <g fill="none" stroke="#000000" stroke-opacity="1" stroke-width="1" stroke-linecap="square" stroke-linejoin="bevel" transform="matrix(1,0,0,1,1802,1178)" font-family="MS Shell Dlg 2" font-size="9.75" font-weight="400" font-style="normal" > <text fill="#000000" fill-opacity="1" stroke="none" xml:space="preserve" x="4" y="17" font-family="Arial" font-size="10pt" font-weight="700" font-style="italic" >nad2</text> </g> <g fill="#ffff33" fill-opacity="1" stroke="#bfbfbf" stroke-opacity="1" stroke-width="1" stroke-linecap="square" stroke-linejoin="bevel" transform="matrix(1,0,0,1,1846,1180)" font-family="MS Shell Dlg 2" font-size="9.75" font-weight="400" font-style="normal" > <rect x="0" y="0" width="50" height="20"/> </g> <g fill="none" stroke="#000000" stroke-opacity="1" stroke-width="1" stroke-linecap="square" stroke-linejoin="bevel" transform="matrix(1,0,0,1,1853,1178)" font-family="MS Shell Dlg 2" font-size="9.75" font-weight="400" font-style="normal" > <text fill="#000000" fill-opacity="1" stroke="none" xml:space="preserve" x="4" y="17" font-family="Arial" font-size="10pt" font-weight="700" font-style="italic" >nad4</text> </g> <g fill="#ffff33" fill-opacity="1" stroke="#bfbfbf" stroke-opacity="1" stroke-width="1" stroke-linecap="square" stroke-linejoin="bevel" transform="matrix(1,0,0,1,1897,1180)" font-family="MS Shell Dlg 2" font-size="9.75" font-weight="400" font-style="normal" > <rect x="0" y="0" width="50" height="20"/> </g> <g fill="none" stroke="#000000" stroke-opacity="1" stroke-width="1" stroke-linecap="square" stroke-linejoin="bevel" transform="matrix(1,0,0,1,1905.5,1178)" font-family="MS Shell Dlg 2" font-size="9.75" font-weight="400" font-style="normal" > <text fill="#000000" fill-opacity="1" stroke="none" xml:space="preserve" x="4" y="17" font-family="Arial" font-size="10pt" font-weight="700" font-style="italic" >atp8</text> </g> <g fill="none" stroke="#000000" stroke-opacity="1" stroke-width="1" stroke-linecap="square" stroke-linejoin="bevel" transform="matrix(1,0,0,1,1948,1180)" font-family="MS Shell Dlg 2" font-size="9.75" font-weight="400" font-style="normal" > <text fill="#000000" fill-opacity="1" stroke="none" xml:space="preserve" x="4" y="17" font-family="Arial" font-size="10pt" font-weight="700" font-style="italic" >(+)</text> </g> <g fill="none" stroke="#000000" stroke-opacity="1" stroke-width="1" stroke-linecap="square" stroke-linejoin="bevel" transform="matrix(1,0,0,1,10,1220)" font-family="MS Shell Dlg 2" font-size="9.75" font-weight="400" font-style="normal" > <text fill="#000000" fill-opacity="1" stroke="none" xml:space="preserve" x="4" y="17" font-family="Arial" font-size="10pt" font-weight="700" font-style="italic" >Perna_viridis_JQ970425</text> </g> <g fill="#ffff33" fill-opacity="1" stroke="#bfbfbf" stroke-opacity="1" stroke-width="1" stroke-linecap="square" stroke-linejoin="bevel" transform="matrix(1,0,0,1,10,1250)" font-family="MS Shell Dlg 2" font-size="9.75" font-weight="400" font-style="normal" > <rect x="0" y="0" width="50" height="20"/> </g> <g fill="none"

stroke="#000000" stroke-opacity="1" stroke-width="1" stroke-linecap="square" stroke-linejoin="bevel" transform="matrix(1,0,0,1,18,1248)" font-family="MS Shell Dlg 2" font-size="9.75" font-weight="400" font-style="normal" > <text fill="#000000" fill-opacity="1" stroke="none" xml:space="preserve" x="4" y="17" font-family="Arial" font-size="10pt" font-weight="700" font-style="italic" >cox1</text> </g> <g fill="#ffff33" fill-opacity="1" stroke="#bfbfbf" stroke-opacity="1" stroke-width="1" stroke-linecap="square" stroke-linejoin="bevel" transform="matrix(1,0,0,1,61,1250)" font-family="MS Shell Dlg 2" font-size="9.75" font-weight="400" font-style="normal" > <rect x="0" y="0" width="50" height="20"/> </g> <g fill="none" stroke="#000000" stroke-opacity="1" stroke-width="1" stroke-linecap="square" stroke-linejoin="bevel" transform="matrix(1,0,0,1,68,1248)" font-family="MS Shell Dlg 2" font-size="9.75" font-weight="400" font-style="normal" > <text fill="#000000" fill-opacity="1" stroke="none" xml:space="preserve" x="4" y="17" font-family="Arial" font-size="10pt" font-weight="700" font-style="italic" >nad4</text> </g> <g fill="#ffff33" fill-opacity="1" stroke="#bfbfbf" stroke-opacity="1" stroke-width="1" stroke-linecap="square" stroke-linejoin="bevel" transform="matrix(1,0,0,1,112,1250)" font-family="MS Shell Dlg 2" font-size="9.75" font-weight="400" font-style="normal" > <rect x="0" y="0" width="50" height="20"/> </g> <g fill="none" stroke="#000000" stroke-opacity="1" stroke-width="1" stroke-linecap="square" stroke-linejoin="bevel" transform="matrix(1,0,0,1,120.5,1248)" font-family="MS Shell Dlg 2" font-size="9.75" font-weight="400" font-style="normal" > <text fill="#000000" fill-opacity="1" stroke="none" xml:space="preserve" x="4" y="17" font-family="Arial" font-size="10pt" font-weight="700" font-style="italic" >atp6</text> </g> <g fill="#ff9999" fill-opacity="1" stroke="#bfbfbf" stroke-opacity="1" stroke-width="1" stroke-linecap="square" stroke-linejoin="bevel" transform="matrix(1,0,0,1,163,1250)" font-family="MS Shell Dlg 2" font-size="9.75" font-weight="400" font-style="normal" > <path vector-effect="none" fill-rule="evenodd" d="M0,1.7 L42.5,1.7 L42.5,0 L50,10 L42.5,20 L42.5,18.3 L0,18.3 L0,1.7"/> </g> <g fill="none" stroke="#000000" stroke-opacity="1" stroke-width="1" stroke-linecap="square" stroke-linejoin="bevel" transform="matrix(1,0,0,1,172.632,1248)" font-family="MS Shell Dlg 2" font-size="9.75" font-weight="400" font-style="normal" > <text fill="#000000" fill-opacity="1" stroke="none" xml:space="preserve" x="4" y="17" font-family="Arial" font-size="10pt" font-weight="700" font-style="italic" >S1</text> </g> <g fill="#ffff33" fill-opacity="1" stroke="#bfbfbf" stroke-opacity="1" stroke-width="1" stroke-linecap="square" stroke-linejoin="bevel" transform="matrix(1,0,0,1,214,1250)" font-family="MS Shell Dlg 2" font-size="9.75" font-weight="400" font-style="normal" > <rect x="0" y="0" width="50" height="20"/> </g> <g fill="none" stroke="#000000" stroke-opacity="1" stroke-width="1" stroke-linecap="square" stroke-linejoin="bevel" transform="matrix(1,0,0,1,223.5,1248)" font-family="MS Shell Dlg 2" font-size="9.75" font-weight="400" font-style="normal" > <text fill="#000000" fill-opacity="1" stroke="none" xml:space="preserve" x="4" y="17" font-family="Arial" font-size="10pt" font-weight="700" font-style="italic" >cytb</text> </g> <g fill="#ff9999" fill-opacity="1" stroke="#bfbfbf" stroke-opacity="1" stroke-width="1" stroke-linecap="square" stroke-linejoin="bevel" transform="matrix(1,0,0,1,265,1250)" font-family="MS Shell Dlg 2" font-size="9.75" font-weight="400" font-style="normal" > <path vector-effect="none" fill-rule="evenodd" d="M0,1.7 L42.5,1.7 L42.5,0 L50,10 L42.5,20 L42.5,18.3 L0,18.3 L0,1.7"/> </g> <g fill="none" stroke="#000000" stroke-opacity="1" stroke-width="1" stroke-linecap="square" stroke-linejoin="bevel" transform="matrix(1,0,0,1,278.132,1248)" font-family="MS Shell Dlg 2" font-size="9.75" font-weight="400" font-style="normal" > <text fill="#000000" fill-opacity="1" stroke="none" xml:space="preserve" x="4" y="17" font-family="Arial" font-size="10pt" font-weight="700" font-style="italic" >A</text> </g> <g fill="#ffff33" fill-opacity="1" stroke="#bfbfbf" stroke-opacity="1" stroke-width="1" stroke-linecap="square" stroke-linejoin="bevel" transform="matrix(1,0,0,1,316,1250)" font-family="MS Shell Dlg 2" font-size="9.75" font-weight="400" font-style="normal" > <rect x="0" y="0" width="50" height="20"/> </g> <g fill="none" stroke="#000000" stroke-opacity="1" stroke-width="1" stroke-linecap="square" stroke-linejoin="bevel" transform="matrix(1,0,0,1,324,1248)" font-family="MS Shell Dlg 2" font-size="9.75" font-weight="400" font-style="normal" > <text fill="#000000" fill-opacity="1" stroke="none" xml:space="preserve" x="4" y="17" font-family="Arial" font-size="10pt" font-weight="700" font-style="italic" >cox2</text> </g> <g fill="#ff9999" fill-opacity="1" stroke="#bfbfbf" stroke-opacity="1" stroke-width="1" stroke-linecap="square" stroke-linejoin="bevel" transform="matrix(1,0,0,1,367,1250)" font-family="MS Shell Dlg 2" font-size="9.75" font-weight="400" font-style="normal" > <path vector-effect="none" fill-rule="evenodd" d="M0,1.7 L42.5,1.7 L42.5,0 L50,10 L42.5,20 L42.5,18.3 L0,18.3 L0,1.7"/> </g> <g fill="none" stroke="#000000" stroke-opacity="1" stroke-width="1" stroke-linecap="square" stroke-linejoin="bevel" transform="matrix(1,0,0,1,380.132,1248)" font-family="MS Shell Dlg 2" font-size="9.75" font-weight="400" font-style="normal" > <text fill="#000000" fill-opacity="1" stroke="none" xml:space="preserve" x="4" y="17" font-family="Arial" font-size="10pt" font-weight="700" font-style="italic" >R</text> </g> <g fill="#ff9999" fill-opacity="1" stroke="#bfbfbf" stroke-opacity="1" stroke-width="1" stroke-linecap="square" stroke-linejoin="bevel" transform="matrix(1,0,0,1,418,1250)" font-family="MS Shell Dlg 2" font-size="9.75" font-weight="400" font-

style="normal" > <path vector-effect="none" fill-rule="evenodd" d="M0,1.7 L42.5,1.7 L42.5,0 L50,10 L42.5,20 L42.5,18.3 L0,18.3 L0,1.7"/> </g> <g fill="none" stroke="#000000" stroke-opacity="1" stroke-width="1" stroke-linecap="square" stroke-linejoin="bevel" transform="matrix(1,0,0,1,430.132,1248)" font-family="MS Shell Dlg 2" font-size="9.75" font-weight="400" font-style="normal" > <text fill="#000000" fill-opacity="1" stroke="none" xml:space="preserve" x="4" y="17" font-family="Arial" font-size="10pt" font-weight="700" font-style="italic" >M</text> </g> <g fill="#ff9999" fill-opacity="1" stroke="#bfbfbf" stroke-opacity="1" stroke-width="1" stroke-linecap="square" stroke-linejoin="bevel" transform="matrix(1,0,0,1,469,1250)" font-family="MS Shell Dlg 2" font-size="9.75" font-weight="400" font-style="normal" > <path vector-effect="none" fill-rule="evenodd" d="M0,1.7 L42.5,1.7 L42.5,0 L50,10 L42.5,20 L42.5,18.3 L0,18.3 L0,1.7"/> </g> <g fill="none" stroke="#000000" stroke-opacity="1" stroke-width="1" stroke-linecap="square" stroke-linejoin="bevel" transform="matrix(1,0,0,1,482.132,1248)" font-family="MS Shell Dlg 2" font-size="9.75" font-weight="400" font-style="normal" > <text fill="#000000" fill-opacity="1" stroke="none" xml:space="preserve" x="4" y="17" font-family="Arial" font-size="10pt" font-weight="700" font-style="italic" >D</text> </g> <g fill="#ffff33" fill-opacity="1" stroke="#bfbfbf" stroke-opacity="1" stroke-width="1" stroke-linecap="square" stroke-linejoin="bevel" transform="matrix(1,0,0,1,520,1250)" font-family="MS Shell Dlg 2" font-size="9.75" font-weight="400" font-style="normal" > <rect x="0" y="0" width="50" height="20"/> </g> <g fill="none" stroke="#000000" stroke-opacity="1" stroke-width="1" stroke-linecap="square" stroke-linejoin="bevel" transform="matrix(1,0,0,1,527,1248)" font-family="MS Shell Dlg 2" font-size="9.75" font-weight="400" font-style="normal" > <text fill="#000000" fill-opacity="1" stroke="none" xml:space="preserve" x="4" y="17" font-family="Arial" font-size="10pt" font-weight="700" font-style="italic" >nad1</text> </g> <g fill="#ffff33" fill-opacity="1" stroke="#bfbfbf" stroke-opacity="1" stroke-width="1" stroke-linecap="square" stroke-linejoin="bevel" transform="matrix(1,0,0,1,571,1250)" font-family="MS Shell Dlg 2" font-size="9.75" font-weight="400" font-style="normal" > <rect x="0" y="0" width="50" height="20"/> </g> <g fill="none" stroke="#000000" stroke-opacity="1" stroke-width="1" stroke-linecap="square" stroke-linejoin="bevel" transform="matrix(1,0,0,1,575,1248)" font-family="MS Shell Dlg 2" font-size="9.75" font-weight="400" font-style="normal" > <text fill="#000000" fill-opacity="1" stroke="none" xml:space="preserve" x="4" y="17" font-family="Arial" font-size="10pt" font-weight="700" font-style="italic" >nad4L</text> </g> <g fill="#ff9999" fill-opacity="1" stroke="#bfbfbf" stroke-opacity="1" stroke-width="1" stroke-linecap="square" stroke-linejoin="bevel" transform="matrix(1,0,0,1,622,1250)" font-family="MS Shell Dlg 2" font-size="9.75" font-weight="400" font-style="normal" > <path vector-effect="none" fill-rule="evenodd" d="M0,1.7 L42.5,1.7 L42.5,0 L50,10 L42.5,20 L42.5,18.3 L0,18.3 L0,1.7"/> </g> <g fill="none" stroke="#000000" stroke-opacity="1" stroke-width="1" stroke-linecap="square" stroke-linejoin="bevel" transform="matrix(1,0,0,1,635.633,1248)" font-family="MS Shell Dlg 2" font-size="9.75" font-weight="400" font-style="normal" > <text fill="#000000" fill-opacity="1" stroke="none" xml:space="preserve" x="4" y="17" font-family="Arial" font-size="10pt" font-weight="700" font-style="italic" >Y</text> </g> <g fill="#ffff33" fill-opacity="1" stroke="#bfbfbf" stroke-opacity="1" stroke-width="1" stroke-linecap="square" stroke-linejoin="bevel" transform="matrix(1,0,0,1,673,1250)" font-family="MS Shell Dlg 2" font-size="9.75" font-weight="400" font-style="normal" > <rect x="0" y="0" width="50" height="20"/> </g> <g fill="none" stroke="#000000" stroke-opacity="1" stroke-width="1" stroke-linecap="square" stroke-linejoin="bevel" transform="matrix(1,0,0,1,680,1248)" font-family="MS Shell Dlg 2" font-size="9.75" font-weight="400" font-style="normal" > <text fill="#000000" fill-opacity="1" stroke="none" xml:space="preserve" x="4" y="17" font-family="Arial" font-size="10pt" font-weight="700" font-style="italic" >nad3</text> </g> <g fill="#ff9999" fill-opacity="1" stroke="#bfbfbf" stroke-opacity="1" stroke-width="1" stroke-linecap="square" stroke-linejoin="bevel" transform="matrix(1,0,0,1,724,1250)" font-family="MS Shell Dlg 2" font-size="9.75" font-weight="400" font-style="normal" > <path vector-effect="none" fill-rule="evenodd" d="M0,1.7 L42.5,1.7 L42.5,0 L50,10 L42.5,20 L42.5,18.3 L0,18.3 L0,1.7"/> </g> <g fill="none" stroke="#000000" stroke-opacity="1" stroke-width="1" stroke-linecap="square" stroke-linejoin="bevel" transform="matrix(1,0,0,1,737.133,1248)" font-family="MS Shell Dlg 2" font-size="9.75" font-weight="400" font-style="normal" > <text fill="#000000" fill-opacity="1" stroke="none" xml:space="preserve" x="4" y="17" font-family="Arial" font-size="10pt" font-weight="700" font-style="italic" >T</text> </g> <g fill="#ffff33" fill-opacity="1" stroke="#bfbfbf" stroke-opacity="1" stroke-width="1" stroke-linecap="square" stroke-linejoin="bevel" transform="matrix(1,0,0,1,775,1250)" font-family="MS Shell Dlg 2" font-size="9.75" font-weight="400" font-style="normal" > <rect x="0" y="0" width="50" height="20"/> </g> <g fill="none" stroke="#000000" stroke-opacity="1" stroke-width="1" stroke-linecap="square" stroke-linejoin="bevel" transform="matrix(1,0,0,1,782,1248)" font-family="MS Shell Dlg 2" font-size="9.75" font-weight="400" font-style="normal" > <text fill="#000000" fill-opacity="1" stroke="none" xml:space="preserve" x="4" y="17" font-family="Arial" font-size="10pt" font-weight="700" font-style="italic" >nad2</text> </g> <g fill="#ff9999" fill-opacity="1" stroke="#bfbfbf" stroke-opacity="1" stroke-width="1" stroke-linecap="square"

stroke-linejoin="bevel" transform="matrix(1,0,0,1,826,1250)" font-family="MS Shell Dlg 2" font-size="9.75" font-
weight="400" font-style="normal" > <path vector-effect="none" fill-rule="evenodd" d="M0,1.7 L42.5,1.7 L42.5,0
L50,10 L42.5,20 L42.5,18.3 L0,18.3 L0,1.7"/> </g> <g fill="none" stroke="#000000" stroke-opacity="1"
stroke-width="1" stroke-linecap="square" stroke-linejoin="bevel" transform="matrix(1,0,0,1,839.633,1248)" font-
family="MS Shell Dlg 2" font-size="9.75" font-weight="400" font-style="normal" > <text fill="#000000" fill-
opacity="1" stroke="none" xml:space="preserve" x="4" y="17" font-family="Arial" font-size="10pt" font-
weight="700" font-style="italic" >E</text> </g> <g fill="#ff9999" fill-opacity="1" stroke="#bfbfbf"
stroke-opacity="1" stroke-width="1" stroke-linecap="square" stroke-linejoin="bevel"
transform="matrix(1,0,0,1,877,1250)" font-family="MS Shell Dlg 2" font-size="9.75" font-weight="400" font-
style="normal" > <path vector-effect="none" fill-rule="evenodd" d="M0,1.7 L42.5,1.7 L42.5,0 L50,10 L42.5,20
L42.5,18.3 L0,18.3 L0,1.7"/> </g> <g fill="none" stroke="#000000" stroke-opacity="1" stroke-width="1"
stroke-linecap="square" stroke-linejoin="bevel" transform="matrix(1,0,0,1,889.633,1248)" font-family="MS Shell
Dlg 2" font-size="9.75" font-weight="400" font-style="normal" > <text fill="#000000" fill-opacity="1"
stroke="none" xml:space="preserve" x="4" y="17" font-family="Arial" font-size="10pt" font-weight="700" font-
style="italic" >Q</text> </g> <g fill="#ff9999" fill-opacity="1" stroke="#bfbfbf" stroke-opacity="1"
stroke-width="1" stroke-linecap="square" stroke-linejoin="bevel" transform="matrix(1,0,0,1,928,1250)" font-
family="MS Shell Dlg 2" font-size="9.75" font-weight="400" font-style="normal" > <path vector-effect="none"
fill-rule="evenodd" d="M0,1.7 L42.5,1.7 L42.5,0 L50,10 L42.5,20 L42.5,18.3 L0,18.3 L0,1.7"/> </g> <g
fill="none" stroke="#000000" stroke-opacity="1" stroke-width="1" stroke-linecap="square" stroke-linejoin="bevel"
transform="matrix(1,0,0,1,937.633,1248)" font-family="MS Shell Dlg 2" font-size="9.75" font-weight="400" font-
style="normal" > <text fill="#000000" fill-opacity="1" stroke="none" xml:space="preserve" x="4" y="17" font-
family="Arial" font-size="10pt" font-weight="700" font-style="italic" >S2</text> </g> <g fill="#ff9999"
fill-opacity="1" stroke="#bfbfbf" stroke-opacity="1" stroke-width="1" stroke-linecap="square" stroke-
linejoin="bevel" transform="matrix(1,0,0,1,979,1250)" font-family="MS Shell Dlg 2" font-size="9.75" font-
weight="400" font-style="normal" > <path vector-effect="none" fill-rule="evenodd" d="M0,1.7 L42.5,1.7 L42.5,0
L50,10 L42.5,20 L42.5,18.3 L0,18.3 L0,1.7"/> </g> <g fill="none" stroke="#000000" stroke-opacity="1"
stroke-width="1" stroke-linecap="square" stroke-linejoin="bevel" transform="matrix(1,0,0,1,992.633,1248)" font-
family="MS Shell Dlg 2" font-size="9.75" font-weight="400" font-style="normal" > <text fill="#000000" fill-
opacity="1" stroke="none" xml:space="preserve" x="4" y="17" font-family="Arial" font-size="10pt" font-
weight="700" font-style="italic" >P</text> </g> <g fill="#ff9999" fill-opacity="1" stroke="#bfbfbf"
stroke-opacity="1" stroke-width="1" stroke-linecap="square" stroke-linejoin="bevel"
transform="matrix(1,0,0,1,1030,1250)" font-family="MS Shell Dlg 2" font-size="9.75" font-weight="400" font-
style="normal" > <path vector-effect="none" fill-rule="evenodd" d="M0,1.7 L42.5,1.7 L42.5,0 L50,10 L42.5,20
L42.5,18.3 L0,18.3 L0,1.7"/> </g> <g fill="none" stroke="#000000" stroke-opacity="1" stroke-width="1"
stroke-linecap="square" stroke-linejoin="bevel" transform="matrix(1,0,0,1,1040.63,1248)" font-family="MS Shell
Dlg 2" font-size="9.75" font-weight="400" font-style="normal" > <text fill="#000000" fill-opacity="1"
stroke="none" xml:space="preserve" x="4" y="17" font-family="Arial" font-size="10pt" font-weight="700" font-
style="italic" >L1</text> </g> <g fill="#ff9999" fill-opacity="1" stroke="#bfbfbf" stroke-opacity="1"
stroke-width="1" stroke-linecap="square" stroke-linejoin="bevel" transform="matrix(1,0,0,1,1081,1250)" font-
family="MS Shell Dlg 2" font-size="9.75" font-weight="400" font-style="normal" > <path vector-effect="none"
fill-rule="evenodd" d="M0,1.7 L42.5,1.7 L42.5,0 L50,10 L42.5,20 L42.5,18.3 L0,18.3 L0,1.7"/> </g> <g
fill="none" stroke="#000000" stroke-opacity="1" stroke-width="1" stroke-linecap="square" stroke-linejoin="bevel"
transform="matrix(1,0,0,1,1094.13,1248)" font-family="MS Shell Dlg 2" font-size="9.75" font-weight="400" font-
style="normal" > <text fill="#000000" fill-opacity="1" stroke="none" xml:space="preserve" x="4" y="17" font-
family="Arial" font-size="10pt" font-weight="700" font-style="italic" >N</text> </g> <g fill="#ffff33"
fill-opacity="1" stroke="#bfbfbf" stroke-opacity="1" stroke-width="1" stroke-linecap="square" stroke-
linejoin="bevel" transform="matrix(1,0,0,1,1132,1250)" font-family="MS Shell Dlg 2" font-size="9.75" font-
weight="400" font-style="normal" > <rect x="0" y="0" width="50" height="20"/> </g> <g fill="none"
stroke="#000000" stroke-opacity="1" stroke-width="1" stroke-linecap="square" stroke-linejoin="bevel"
transform="matrix(1,0,0,1,1139,1248)" font-family="MS Shell Dlg 2" font-size="9.75" font-weight="400" font-
style="normal" > <text fill="#000000" fill-opacity="1" stroke="none" xml:space="preserve" x="4" y="17" font-
family="Arial" font-size="10pt" font-weight="700" font-style="italic" >nad6</text> </g> <g
fill="#ff9999" fill-opacity="1" stroke="#bfbfbf" stroke-opacity="1" stroke-width="1" stroke-linecap="square"
stroke-linejoin="bevel" transform="matrix(1,0,0,1,1183,1250)" font-family="MS Shell Dlg 2" font-size="9.75"
font-weight="400" font-style="normal" > <path vector-effect="none" fill-rule="evenodd" d="M0,1.7 L42.5,1.7
L42.5,0 L50,10 L42.5,20 L42.5,18.3 L0,18.3 L0,1.7"/> </g> <g fill="none" stroke="#000000" stroke-
opacity="1" stroke-width="1" stroke-linecap="square" stroke-linejoin="bevel"
transform="matrix(1,0,0,1,1196.13,1248)" font-family="MS Shell Dlg 2" font-size="9.75" font-weight="400" font-

style="normal" > <text fill="#000000" fill-opacity="1" stroke="none" xml:space="preserve" x="4" y="17" font-family="Arial" font-size="10pt" font-weight="700" font-style="italic" >C</text> </g> <g fill="#ff9999" fill-opacity="1" stroke="#bfbfbf" stroke-opacity="1" stroke-width="1" stroke-linecap="square" stroke-linejoin="bevel" transform="matrix(1,0,0,1,1234,1250)" font-family="MS Shell Dlg 2" font-size="9.75" font-weight="400" font-style="normal" > <path vector-effect="none" fill-rule="evenodd" d="M0,1.7 L42.5,1.7 L42.5,0 L50,10 L42.5,20 L42.5,18.3 L0,18.3 L0,1.7"/> </g> <g fill="none" stroke="#000000" stroke-opacity="1" stroke-width="1" stroke-linecap="square" stroke-linejoin="bevel" transform="matrix(1,0,0,1,1245.13,1248)" font-family="MS Shell Dlg 2" font-size="9.75" font-weight="400" font-style="normal" > <text fill="#000000" fill-opacity="1" stroke="none" xml:space="preserve" x="4" y="17" font-family="Arial" font-size="10pt" font-weight="700" font-style="italic" >W</text> </g> <g fill="#ff9999" fill-opacity="1" stroke="#bfbfbf" stroke-opacity="1" stroke-width="1" stroke-linecap="square" stroke-linejoin="bevel" transform="matrix(1,0,0,1,1285,1250)" font-family="MS Shell Dlg 2" font-size="9.75" font-weight="400" font-style="normal" > <path vector-effect="none" fill-rule="evenodd" d="M0,1.7 L42.5,1.7 L42.5,0 L50,10 L42.5,20 L42.5,18.3 L0,18.3 L0,1.7"/> </g> <g fill="none" stroke="#000000" stroke-opacity="1" stroke-width="1" stroke-linecap="square" stroke-linejoin="bevel" transform="matrix(1,0,0,1,1298.63,1248)" font-family="MS Shell Dlg 2" font-size="9.75" font-weight="400" font-style="normal" > <text fill="#000000" fill-opacity="1" stroke="none" xml:space="preserve" x="4" y="17" font-family="Arial" font-size="10pt" font-weight="700" font-style="italic" >K</text> </g> <g fill="#ffff33" fill-opacity="1" stroke="#bfbfbf" stroke-opacity="1" stroke-width="1" stroke-linecap="square" stroke-linejoin="bevel" transform="matrix(1,0,0,1,1336,1250)" font-family="MS Shell Dlg 2" font-size="9.75" font-weight="400" font-style="normal" > <rect x="0" y="0" width="50" height="20"/> </g> <g fill="none" stroke="#000000" stroke-opacity="1" stroke-width="1" stroke-linecap="square" stroke-linejoin="bevel" transform="matrix(1,0,0,1,1343,1248)" font-family="MS Shell Dlg 2" font-size="9.75" font-weight="400" font-style="normal" > <text fill="#000000" fill-opacity="1" stroke="none" xml:space="preserve" x="4" y="17" font-family="Arial" font-size="10pt" font-weight="700" font-style="italic" >nad5</text> </g> <g fill="#ff9999" fill-opacity="1" stroke="#bfbfbf" stroke-opacity="1" stroke-width="1" stroke-linecap="square" stroke-linejoin="bevel" transform="matrix(1,0,0,1,1387,1250)" font-family="MS Shell Dlg 2" font-size="9.75" font-weight="400" font-style="normal" > <path vector-effect="none" fill-rule="evenodd" d="M0,1.7 L42.5,1.7 L42.5,0 L50,10 L42.5,20 L42.5,18.3 L0,18.3 L0,1.7"/> </g> <g fill="none" stroke="#000000" stroke-opacity="1" stroke-width="1" stroke-linecap="square" stroke-linejoin="bevel" transform="matrix(1,0,0,1,1397.63,1248)" font-family="MS Shell Dlg 2" font-size="9.75" font-weight="400" font-style="normal" > <text fill="#000000" fill-opacity="1" stroke="none" xml:space="preserve" x="4" y="17" font-family="Arial" font-size="10pt" font-weight="700" font-style="italic" >L1</text> </g> <g fill="#99ffff" fill-opacity="1" stroke="#bfbfbf" stroke-opacity="1" stroke-width="1" stroke-linecap="square" stroke-linejoin="bevel" transform="matrix(1,0,0,1,1438,1250)" font-family="MS Shell Dlg 2" font-size="9.75" font-weight="400" font-style="normal" > <path vector-effect="none" fill-rule="evenodd" d="M0,5 C0,2.23858 2.23858,0 5,0 L45,0 C47.7614,0 50,2.23858 50,5 L50,15 C50,17.7614 47.7614,20 45,20 L5,20 C2.23858,20 0,17.7614 0,15 L0,5"/> </g> <g fill="none" stroke="#000000" stroke-opacity="1" stroke-width="1" stroke-linecap="square" stroke-linejoin="bevel" transform="matrix(1,0,0,1,1446.5,1248)" font-family="MS Shell Dlg 2" font-size="9.75" font-weight="400" font-style="normal" > <text fill="#000000" fill-opacity="1" stroke="none" xml:space="preserve" x="4" y="17" font-family="Arial" font-size="10pt" font-weight="700" font-style="italic" >rrnS</text> </g> <g fill="#ff9999" fill-opacity="1" stroke="#bfbfbf" stroke-opacity="1" stroke-width="1" stroke-linecap="square" stroke-linejoin="bevel" transform="matrix(1,0,0,1,1489,1250)" font-family="MS Shell Dlg 2" font-size="9.75" font-weight="400" font-style="normal" > <path vector-effect="none" fill-rule="evenodd" d="M0,1.7 L42.5,1.7 L42.5,0 L50,10 L42.5,20 L42.5,18.3 L0,18.3 L0,1.7"/> </g> <g fill="none" stroke="#000000" stroke-opacity="1" stroke-width="1" stroke-linecap="square" stroke-linejoin="bevel" transform="matrix(1,0,0,1,1504.13,1248)" font-family="MS Shell Dlg 2" font-size="9.75" font-weight="400" font-style="normal" > <text fill="#000000" fill-opacity="1" stroke="none" xml:space="preserve" x="4" y="17" font-family="Arial" font-size="10pt" font-weight="700" font-style="italic" >I</text> </g> <g fill="#ff9999" fill-opacity="1" stroke="#bfbfbf" stroke-opacity="1" stroke-width="1" stroke-linecap="square" stroke-linejoin="bevel" transform="matrix(1,0,0,1,1540,1250)" font-family="MS Shell Dlg 2" font-size="9.75" font-weight="400" font-style="normal" > <path vector-effect="none" fill-rule="evenodd" d="M0,1.7 L42.5,1.7 L42.5,0 L50,10 L42.5,20 L42.5,18.3 L0,18.3 L0,1.7"/> </g> <g fill="none" stroke="#000000" stroke-opacity="1" stroke-width="1" stroke-linecap="square" stroke-linejoin="bevel" transform="matrix(1,0,0,1,1553.13,1248)" font-family="MS Shell Dlg 2" font-size="9.75" font-weight="400" font-style="normal" > <text fill="#000000" fill-opacity="1" stroke="none" xml:space="preserve" x="4" y="17" font-family="Arial" font-size="10pt" font-weight="700" font-style="italic" >G</text> </g> <g fill="#ff9999" fill-opacity="1" stroke="#bfbfbf" stroke-opacity="1" stroke-width="1" stroke-linecap="square" stroke-linejoin="bevel" transform="matrix(1,0,0,1,1591,1250)" font-family="MS Shell Dlg 2" font-size="9.75" font-weight="400" font-

style="normal" > <path vector-effect="none" fill-rule="evenodd" d="M0,1.7 L42.5,1.7 L42.5,0 L50,10 L42.5,20 L42.5,18.3 L0,18.3 L0,1.7"/> </g>   <g fill="none" stroke="#000000" stroke-opacity="1" stroke-width="1" stroke-linecap="square" stroke-linejoin="bevel" transform="matrix(1,0,0,1,1604.13,1248)" font-family="MS Shell Dlg 2" font-size="9.75" font-weight="400" font-style="normal" > <text fill="#000000" fill-opacity="1" stroke="none" xml:space="preserve" x="4" y="17" font-family="Arial" font-size="10pt" font-weight="700" font-style="italic"   >V</text> </g>   <g fill="#99ffff" fill-opacity="1" stroke="#bfbfbf" stroke-opacity="1" stroke-width="1" stroke-linecap="square" stroke-linejoin="bevel" transform="matrix(1,0,0,1,1642,1250)" font-family="MS Shell Dlg 2" font-size="9.75" font-weight="400" font-style="normal" > <path vector-effect="none" fill-rule="evenodd" d="M0,5 C0,2.23858 2.23858,0 5,0 L45,0 C47.7614,0 50,2.23858 50,5 L50,15 C50,17.7614 47.7614,20 45,20 L5,20 C2.23858,20 0,17.7614 0,15 L0,5"/> </g>   <g fill="none" stroke="#000000" stroke-opacity="1" stroke-width="1" stroke-linecap="square" stroke-linejoin="bevel" transform="matrix(1,0,0,1,1651.5,1248)" font-family="MS Shell Dlg 2" font-size="9.75" font-weight="400" font-style="normal" > <text fill="#000000" fill-opacity="1" stroke="none" xml:space="preserve" x="4" y="17" font-family="Arial" font-size="10pt" font-weight="700" font-style="italic"   >rrnL</text> </g>   <g fill="#ff9999" fill-opacity="1" stroke="#bfbfbf" stroke-opacity="1" stroke-width="1" stroke-linecap="square" stroke-linejoin="bevel" transform="matrix(1,0,0,1,1693,1250)" font-family="MS Shell Dlg 2" font-size="9.75" font-weight="400" font-style="normal" > <path vector-effect="none" fill-rule="evenodd" d="M0,1.7 L42.5,1.7 L42.5,0 L50,10 L42.5,20 L42.5,18.3 L0,18.3 L0,1.7"/> </g>   <g fill="none" stroke="#000000" stroke-opacity="1" stroke-width="1" stroke-linecap="square" stroke-linejoin="bevel" transform="matrix(1,0,0,1,1705.13,1248)" font-family="MS Shell Dlg 2" font-size="9.75" font-weight="400" font-style="normal" > <text fill="#000000" fill-opacity="1" stroke="none" xml:space="preserve" x="4" y="17" font-family="Arial" font-size="10pt" font-weight="700" font-style="italic"   >M</text> </g>   <g fill="#ff9999" fill-opacity="1" stroke="#bfbfbf" stroke-opacity="1" stroke-width="1" stroke-linecap="square" stroke-linejoin="bevel" transform="matrix(1,0,0,1,1744,1250)" font-family="MS Shell Dlg 2" font-size="9.75" font-weight="400" font-style="normal" > <path vector-effect="none" fill-rule="evenodd" d="M0,1.7 L42.5,1.7 L42.5,0 L50,10 L42.5,20 L42.5,18.3 L0,18.3 L0,1.7"/> </g>   <g fill="none" stroke="#000000" stroke-opacity="1" stroke-width="1" stroke-linecap="square" stroke-linejoin="bevel" transform="matrix(1,0,0,1,1757.63,1248)" font-family="MS Shell Dlg 2" font-size="9.75" font-weight="400" font-style="normal" > <text fill="#000000" fill-opacity="1" stroke="none" xml:space="preserve" x="4" y="17" font-family="Arial" font-size="10pt" font-weight="700" font-style="italic"   >F</text> </g>   <g fill="#ff9999" fill-opacity="1" stroke="#bfbfbf" stroke-opacity="1" stroke-width="1" stroke-linecap="square" stroke-linejoin="bevel" transform="matrix(1,0,0,1,1795,1250)" font-family="MS Shell Dlg 2" font-size="9.75" font-weight="400" font-style="normal" > <path vector-effect="none" fill-rule="evenodd" d="M0,1.7 L42.5,1.7 L42.5,0 L50,10 L42.5,20 L42.5,18.3 L0,18.3 L0,1.7"/> </g>   <g fill="none" stroke="#000000" stroke-opacity="1" stroke-width="1" stroke-linecap="square" stroke-linejoin="bevel" transform="matrix(1,0,0,1,1808.13,1248)" font-family="MS Shell Dlg 2" font-size="9.75" font-weight="400" font-style="normal" > <text fill="#000000" fill-opacity="1" stroke="none" xml:space="preserve" x="4" y="17" font-family="Arial" font-size="10pt" font-weight="700" font-style="italic"   >H</text> </g>   <g fill="#ffff33" fill-opacity="1" stroke="#bfbfbf" stroke-opacity="1" stroke-width="1" stroke-linecap="square" stroke-linejoin="bevel" transform="matrix(1,0,0,1,1846,1250)" font-family="MS Shell Dlg 2" font-size="9.75" font-weight="400" font-style="normal" > <rect x="0" y="0" width="50" height="20"/> </g>   <g fill="none" stroke="#000000" stroke-opacity="1" stroke-width="1" stroke-linecap="square" stroke-linejoin="bevel" transform="matrix(1,0,0,1,1854,1248)" font-family="MS Shell Dlg 2" font-size="9.75" font-weight="400" font-style="normal" > <text fill="#000000" fill-opacity="1" stroke="none" xml:space="preserve" x="4" y="17" font-family="Arial" font-size="10pt" font-weight="700" font-style="italic" >cox3</text> </g>   <g fill="#ffff33" fill-opacity="1" stroke="#bfbfbf" stroke-opacity="1" stroke-width="1" stroke-linecap="square" stroke-linejoin="bevel" transform="matrix(1,0,0,1,1897,1250)" font-family="MS Shell Dlg 2" font-size="9.75" font-weight="400" font-style="normal" > <rect x="0" y="0" width="50" height="20"/> </g> <g fill="none" stroke="#000000" stroke-opacity="1" stroke-width="1" stroke-linecap="square" stroke-linejoin="bevel" transform="matrix(1,0,0,1,1905.5,1248)" font-family="MS Shell Dlg 2" font-size="9.75" font-weight="400" font-style="normal" > <text fill="#000000" fill-opacity="1" stroke="none" xml:space="preserve" x="4" y="17" font-family="Arial" font-size="10pt" font-weight="700" font-style="italic"   >atp8</text> </g> <g fill="none" stroke="#000000" stroke-opacity="1" stroke-width="1" stroke-linecap="square" stroke-linejoin="bevel" transform="matrix(1,0,0,1,1948,1250)" font-family="MS Shell Dlg 2" font-size="9.75" font-weight="400" font-style="normal" > <text fill="#000000" fill-opacity="1" stroke="none" xml:space="preserve" x="4" y="17" font-family="Arial" font-size="10pt" font-weight="700" font-style="italic"   >(+)</text> </g> <g fill="none" stroke="#000000" stroke-opacity="1" stroke-width="1" stroke-linecap="square" stroke-linejoin="bevel" transform="matrix(1,0,0,1,10,1290)" font-family="MS Shell Dlg 2" font-size="9.75" font-weight="400" font-style="normal" > <text fill="#000000" fill-opacity="1" stroke="none" xml:space="preserve"

x="4" y="17" font-family="Arial" font-size="10pt" font-weight="700" font-style="italic" >Perna_canaliculus_MK775558</text> </g> <g fill="#ffff33" fill-opacity="1" stroke="#bfbfbf" stroke-opacity="1" stroke-width="1" stroke-linecap="square" stroke-linejoin="bevel" transform="matrix(1,0,0,1,10,1320)" font-family="MS Shell Dlg 2" font-size="9.75" font-weight="400" font-style="normal" > <rect x="0" y="0" width="50" height="20"/> </g> <g fill="none" stroke="#000000" stroke-opacity="1" stroke-width="1" stroke-linecap="square" stroke-linejoin="bevel" transform="matrix(1,0,0,1,18,1318)" font-family="MS Shell Dlg 2" font-size="9.75" font-weight="400" font-style="normal" > <text fill="#000000" fill-opacity="1" stroke="none" xml:space="preserve" x="4" y="17" font-family="Arial" font-size="10pt" font-weight="700" font-style="italic" >cox1</text> </g> <g fill="#ffff33" fill-opacity="1" stroke="#bfbfbf" stroke-opacity="1" stroke-width="1" stroke-linecap="square" stroke-linejoin="bevel" transform="matrix(1,0,0,1,61,1320)" font-family="MS Shell Dlg 2" font-size="9.75" font-weight="400" font-style="normal" > <rect x="0" y="0" width="50" height="20"/> </g> <g fill="none" stroke="#000000" stroke-opacity="1" stroke-width="1" stroke-linecap="square" stroke-linejoin="bevel" transform="matrix(1,0,0,1,69.5,1318)" font-family="MS Shell Dlg 2" font-size="9.75" font-weight="400" font-style="normal" > <text fill="#000000" fill-opacity="1" stroke="none" xml:space="preserve" x="4" y="17" font-family="Arial" font-size="10pt" font-weight="700" font-style="italic" >atp6</text> </g> <g fill="#ffff33" fill-opacity="1" stroke="#bfbfbf" stroke-opacity="1" stroke-width="1" stroke-linecap="square" stroke-linejoin="bevel" transform="matrix(1,0,0,1,112,1320)" font-family="MS Shell Dlg 2" font-size="9.75" font-weight="400" font-style="normal" > <rect x="0" y="0" width="50" height="20"/> </g> <g fill="none" stroke="#000000" stroke-opacity="1" stroke-width="1" stroke-linecap="square" stroke-linejoin="bevel" transform="matrix(1,0,0,1,121.5,1318)" font-family="MS Shell Dlg 2" font-size="9.75" font-weight="400" font-style="normal" > <text fill="#000000" fill-opacity="1" stroke="none" xml:space="preserve" x="4" y="17" font-family="Arial" font-size="10pt" font-weight="700" font-style="italic" >cytb</text> </g> <g fill="#ffff33" fill-opacity="1" stroke="#bfbfbf" stroke-opacity="1" stroke-width="1" stroke-linecap="square" stroke-linejoin="bevel" transform="matrix(1,0,0,1,163,1320)" font-family="MS Shell Dlg 2" font-size="9.75" font-weight="400" font-style="normal" > <rect x="0" y="0" width="50" height="20"/> </g> <g fill="none" stroke="#000000" stroke-opacity="1" stroke-width="1" stroke-linecap="square" stroke-linejoin="bevel" transform="matrix(1,0,0,1,171,1318)" font-family="MS Shell Dlg 2" font-size="9.75" font-weight="400" font-style="normal" > <text fill="#000000" fill-opacity="1" stroke="none" xml:space="preserve" x="4" y="17" font-family="Arial" font-size="10pt" font-weight="700" font-style="italic" >cox2</text> </g> <g fill="#ff9999" fill-opacity="1" stroke="#bfbfbf" stroke-opacity="1" stroke-width="1" stroke-linecap="square" stroke-linejoin="bevel" transform="matrix(1,0,0,1,214,1320)" font-family="MS Shell Dlg 2" font-size="9.75" font-weight="400" font-style="normal" > <path vector-effect="none" fill-rule="evenodd" d="M0,1.7 L42.5,1.7 L42.5,0 L50,10 L42.5,20 L42.5,18.3 L0,18.3 L0,1.7"/> </g> <g fill="none" stroke="#000000" stroke-opacity="1" stroke-width="1" stroke-linecap="square" stroke-linejoin="bevel" transform="matrix(1,0,0,1,227.132,1318)" font-family="MS Shell Dlg 2" font-size="9.75" font-weight="400" font-style="normal" > <text fill="#000000" fill-opacity="1" stroke="none" xml:space="preserve" x="4" y="17" font-family="Arial" font-size="10pt" font-weight="700" font-style="italic" >R</text> </g> <g fill="#ff9999" fill-opacity="1" stroke="#bfbfbf" stroke-opacity="1" stroke-width="1" stroke-linecap="square" stroke-linejoin="bevel" transform="matrix(1,0,0,1,265,1320)" font-family="MS Shell Dlg 2" font-size="9.75" font-weight="400" font-style="normal" > <path vector-effect="none" fill-rule="evenodd" d="M0,1.7 L42.5,1.7 L42.5,0 L50,10 L42.5,20 L42.5,18.3 L0,18.3 L0,1.7"/> </g> <g fill="none" stroke="#000000" stroke-opacity="1" stroke-width="1" stroke-linecap="square" stroke-linejoin="bevel" transform="matrix(1,0,0,1,277.132,1318)" font-family="MS Shell Dlg 2" font-size="9.75" font-weight="400" font-style="normal" > <text fill="#000000" fill-opacity="1" stroke="none" xml:space="preserve" x="4" y="17" font-family="Arial" font-size="10pt" font-weight="700" font-style="italic" >M</text> </g> <g fill="#ff9999" fill-opacity="1" stroke="#bfbfbf" stroke-opacity="1" stroke-width="1" stroke-linecap="square" stroke-linejoin="bevel" transform="matrix(1,0,0,1,316,1320)" font-family="MS Shell Dlg 2" font-size="9.75" font-weight="400" font-style="normal" > <path vector-effect="none" fill-rule="evenodd" d="M0,1.7 L42.5,1.7 L42.5,0 L50,10 L42.5,20 L42.5,18.3 L0,18.3 L0,1.7"/> </g> <g fill="none" stroke="#000000" stroke-opacity="1" stroke-width="1" stroke-linecap="square" stroke-linejoin="bevel" transform="matrix(1,0,0,1,329.132,1318)" font-family="MS Shell Dlg 2" font-size="9.75" font-weight="400" font-style="normal" > <text fill="#000000" fill-opacity="1" stroke="none" xml:space="preserve" x="4" y="17" font-family="Arial" font-size="10pt" font-weight="700" font-style="italic" >T</text> </g> <g fill="#ff9999" fill-opacity="1" stroke="#bfbfbf" stroke-opacity="1" stroke-width="1" stroke-linecap="square" stroke-linejoin="bevel" transform="matrix(1,0,0,1,367,1320)" font-family="MS Shell Dlg 2" font-size="9.75" font-weight="400" font-style="normal" > <path vector-effect="none" fill-rule="evenodd" d="M0,1.7 L42.5,1.7 L42.5,0 L50,10 L42.5,20 L42.5,18.3 L0,18.3 L0,1.7"/> </g> <g fill="none" stroke="#000000" stroke-opacity="1" stroke-width="1" stroke-linecap="square" stroke-linejoin="bevel" transform="matrix(1,0,0,1,380.632,1318)" font-family="MS Shell Dlg 2" font-size="9.75" font-weight="400" font-style="normal" > <text fill="#000000" fill-

opacity="1" stroke="none" xml:space="preserve" x="4" y="17" font-family="Arial" font-size="10pt" font-weight="700" font-style="italic" >E</text> </g> <g fill="#ff9999" fill-opacity="1" stroke="#bfbfbf" stroke-opacity="1" stroke-width="1" stroke-linecap="square" stroke-linejoin="bevel" transform="matrix(1,0,0,1,418,1320)" font-family="MS Shell Dlg 2" font-size="9.75" font-weight="400" font-style="normal" > <path vector-effect="none" fill-rule="evenodd" d="M0,1.7 L42.5,1.7 L42.5,0 L50,10 L42.5,20 L42.5,18.3 L0,18.3 L0,1.7"/> </g> <g fill="none" stroke="#000000" stroke-opacity="1" stroke-width="1" stroke-linecap="square" stroke-linejoin="bevel" transform="matrix(1,0,0,1,430.632,1318)" font-family="MS Shell Dlg 2" font-size="9.75" font-weight="400" font-style="normal" > <text fill="#000000" fill-opacity="1" stroke="none" xml:space="preserve" x="4" y="17" font-family="Arial" font-size="10pt" font-weight="700" font-style="italic" >Q</text> </g> <g fill="#ff9999" fill-opacity="1" stroke="#bfbfbf" stroke-opacity="1" stroke-width="1" stroke-linecap="square" stroke-linejoin="bevel" transform="matrix(1,0,0,1,469,1320)" font-family="MS Shell Dlg 2" font-size="9.75" font-weight="400" font-style="normal" > <path vector-effect="none" fill-rule="evenodd" d="M0,1.7 L42.5,1.7 L42.5,0 L50,10 L42.5,20 L42.5,18.3 L0,18.3 L0,1.7"/> </g> <g fill="none" stroke="#000000" stroke-opacity="1" stroke-width="1" stroke-linecap="square" stroke-linejoin="bevel" transform="matrix(1,0,0,1,482.132,1318)" font-family="MS Shell Dlg 2" font-size="9.75" font-weight="400" font-style="normal" > <text fill="#000000" fill-opacity="1" stroke="none" xml:space="preserve" x="4" y="17" font-family="Arial" font-size="10pt" font-weight="700" font-style="italic" >S</text> </g> <g fill="#ff9999" fill-opacity="1" stroke="#bfbfbf" stroke-opacity="1" stroke-width="1" stroke-linecap="square" stroke-linejoin="bevel" transform="matrix(1,0,0,1,520,1320)" font-family="MS Shell Dlg 2" font-size="9.75" font-weight="400" font-style="normal" > <path vector-effect="none" fill-rule="evenodd" d="M0,1.7 L42.5,1.7 L42.5,0 L50,10 L42.5,20 L42.5,18.3 L0,18.3 L0,1.7"/> </g> <g fill="none" stroke="#000000" stroke-opacity="1" stroke-width="1" stroke-linecap="square" stroke-linejoin="bevel" transform="matrix(1,0,0,1,533.133,1318)" font-family="MS Shell Dlg 2" font-size="9.75" font-weight="400" font-style="normal" > <text fill="#000000" fill-opacity="1" stroke="none" xml:space="preserve" x="4" y="17" font-family="Arial" font-size="10pt" font-weight="700" font-style="italic" >D</text> </g> <g fill="#ffff33" fill-opacity="1" stroke="#bfbfbf" stroke-opacity="1" stroke-width="1" stroke-linecap="square" stroke-linejoin="bevel" transform="matrix(1,0,0,1,571,1320)" font-family="MS Shell Dlg 2" font-size="9.75" font-weight="400" font-style="normal" > <rect x="0" y="0" width="50" height="20"/> </g> <g fill="none" stroke="#000000" stroke-opacity="1" stroke-width="1" stroke-linecap="square" stroke-linejoin="bevel" transform="matrix(1,0,0,1,578,1318)" font-family="MS Shell Dlg 2" font-size="9.75" font-weight="400" font-style="normal" > <text fill="#000000" fill-opacity="1" stroke="none" xml:space="preserve" x="4" y="17" font-family="Arial" font-size="10pt" font-weight="700" font-style="italic" >nad1</text> </g> <g fill="#ff9999" fill-opacity="1" stroke="#bfbfbf" stroke-opacity="1" stroke-width="1" stroke-linecap="square" stroke-linejoin="bevel" transform="matrix(1,0,0,1,622,1320)" font-family="MS Shell Dlg 2" font-size="9.75" font-weight="400" font-style="normal" > <path vector-effect="none" fill-rule="evenodd" d="M0,1.7 L42.5,1.7 L42.5,0 L50,10 L42.5,20 L42.5,18.3 L0,18.3 L0,1.7"/> </g> <g fill="none" stroke="#000000" stroke-opacity="1" stroke-width="1" stroke-linecap="square" stroke-linejoin="bevel" transform="matrix(1,0,0,1,635.633,1318)" font-family="MS Shell Dlg 2" font-size="9.75" font-weight="400" font-style="normal" > <text fill="#000000" fill-opacity="1" stroke="none" xml:space="preserve" x="4" y="17" font-family="Arial" font-size="10pt" font-weight="700" font-style="italic" >P</text> </g> <g fill="#ffff33" fill-opacity="1" stroke="#bfbfbf" stroke-opacity="1" stroke-width="1" stroke-linecap="square" stroke-linejoin="bevel" transform="matrix(1,0,0,1,673,1320)" font-family="MS Shell Dlg 2" font-size="9.75" font-weight="400" font-style="normal" > <rect x="0" y="0" width="50" height="20"/> </g> <g fill="none" stroke="#000000" stroke-opacity="1" stroke-width="1" stroke-linecap="square" stroke-linejoin="bevel" transform="matrix(1,0,0,1,677,1318)" font-family="MS Shell Dlg 2" font-size="9.75" font-weight="400" font-style="normal" > <text fill="#000000" fill-opacity="1" stroke="none" xml:space="preserve" x="4" y="17" font-family="Arial" font-size="10pt" font-weight="700" font-style="italic" >nad4L</text> </g> <g fill="#ff9999" fill-opacity="1" stroke="#bfbfbf" stroke-opacity="1" stroke-width="1" stroke-linecap="square" stroke-linejoin="bevel" transform="matrix(1,0,0,1,724,1320)" font-family="MS Shell Dlg 2" font-size="9.75" font-weight="400" font-style="normal" > <path vector-effect="none" fill-rule="evenodd" d="M0,1.7 L42.5,1.7 L42.5,0 L50,10 L42.5,20 L42.5,18.3 L0,18.3 L0,1.7"/> </g> <g fill="none" stroke="#000000" stroke-opacity="1" stroke-width="1" stroke-linecap="square" stroke-linejoin="bevel" transform="matrix(1,0,0,1,737.633,1318)" font-family="MS Shell Dlg 2" font-size="9.75" font-weight="400" font-style="normal" > <text fill="#000000" fill-opacity="1" stroke="none" xml:space="preserve" x="4" y="17" font-family="Arial" font-size="10pt" font-weight="700" font-style="italic" >Y</text> </g> <g fill="#ffff33" fill-opacity="1" stroke="#bfbfbf" stroke-opacity="1" stroke-width="1" stroke-linecap="square" stroke-linejoin="bevel" transform="matrix(1,0,0,1,775,1320)" font-family="MS Shell Dlg 2" font-size="9.75" font-weight="400" font-style="normal" > <rect x="0" y="0" width="50" height="20"/> </g> <g fill="none" stroke="#000000" stroke-

opacity="1" stroke-width="1" stroke-linecap="square" stroke-linejoin="bevel" transform="matrix(1,0,0,1,782,1318)" font-family="MS Shell Dlg 2" font-size="9.75" font-weight="400" font-style="normal" > <text fill="#000000" fill-opacity="1" stroke="none" xml:space="preserve" x="4" y="17" font-family="Arial" font-size="10pt" font-weight="700" font-style="italic" >nad3</text> </g> <g fill="#ff9999" fill-opacity="1" stroke="#bfbfbf" stroke-opacity="1" stroke-width="1" stroke-linecap="square" stroke-linejoin="bevel" transform="matrix(1,0,0,1,826,1320)" font-family="MS Shell Dlg 2" font-size="9.75" font-weight="400" font-style="normal" > <path vector-effect="none" fill-rule="evenodd" d="M0,1.7 L42.5,1.7 L42.5,0 L50,10 L42.5,20 L42.5,18.3 L0,18.3 L0,1.7"/> </g> <g fill="none" stroke="#000000" stroke-opacity="1" stroke-width="1" stroke-linecap="square" stroke-linejoin="bevel" transform="matrix(1,0,0,1,840.133,1318)" font-family="MS Shell Dlg 2" font-size="9.75" font-weight="400" font-style="normal" > <text fill="#000000" fill-opacity="1" stroke="none" xml:space="preserve" x="4" y="17" font-family="Arial" font-size="10pt" font-weight="700" font-style="italic" >L</text> </g> <g fill="#ff9999" fill-opacity="1" stroke="#bfbfbf" stroke-opacity="1" stroke-width="1" stroke-linecap="square" stroke-linejoin="bevel" transform="matrix(1,0,0,1,877,1320)" font-family="MS Shell Dlg 2" font-size="9.75" font-weight="400" font-style="normal" > <path vector-effect="none" fill-rule="evenodd" d="M0,1.7 L42.5,1.7 L42.5,0 L50,10 L42.5,20 L42.5,18.3 L0,18.3 L0,1.7"/> </g> <g fill="none" stroke="#000000" stroke-opacity="1" stroke-width="1" stroke-linecap="square" stroke-linejoin="bevel" transform="matrix(1,0,0,1,890.133,1318)" font-family="MS Shell Dlg 2" font-size="9.75" font-weight="400" font-style="normal" > <text fill="#000000" fill-opacity="1" stroke="none" xml:space="preserve" x="4" y="17" font-family="Arial" font-size="10pt" font-weight="700" font-style="italic" >N</text> </g> <g fill="#ffff33" fill-opacity="1" stroke="#bfbfbf" stroke-opacity="1" stroke-width="1" stroke-linecap="square" stroke-linejoin="bevel" transform="matrix(1,0,0,1,928,1320)" font-family="MS Shell Dlg 2" font-size="9.75" font-weight="400" font-style="normal" > <rect x="0" y="0" width="50" height="20"/> </g> <g fill="none" stroke="#000000" stroke-opacity="1" stroke-width="1" stroke-linecap="square" stroke-linejoin="bevel" transform="matrix(1,0,0,1,935,1318)" font-family="MS Shell Dlg 2" font-size="9.75" font-weight="400" font-style="normal" > <text fill="#000000" fill-opacity="1" stroke="none" xml:space="preserve" x="4" y="17" font-family="Arial" font-size="10pt" font-weight="700" font-style="italic" >nad6</text> </g> <g fill="#ff9999" fill-opacity="1" stroke="#bfbfbf" stroke-opacity="1" stroke-width="1" stroke-linecap="square" stroke-linejoin="bevel" transform="matrix(1,0,0,1,979,1320)" font-family="MS Shell Dlg 2" font-size="9.75" font-weight="400" font-style="normal" > <path vector-effect="none" fill-rule="evenodd" d="M0,1.7 L42.5,1.7 L42.5,0 L50,10 L42.5,20 L42.5,18.3 L0,18.3 L0,1.7"/> </g> <g fill="none" stroke="#000000" stroke-opacity="1" stroke-width="1" stroke-linecap="square" stroke-linejoin="bevel" transform="matrix(1,0,0,1,992.133,1318)" font-family="MS Shell Dlg 2" font-size="9.75" font-weight="400" font-style="normal" > <text fill="#000000" fill-opacity="1" stroke="none" xml:space="preserve" x="4" y="17" font-family="Arial" font-size="10pt" font-weight="700" font-style="italic" >C</text> </g> <g fill="#ff9999" fill-opacity="1" stroke="#bfbfbf" stroke-opacity="1" stroke-width="1" stroke-linecap="square" stroke-linejoin="bevel" transform="matrix(1,0,0,1,1030,1320)" font-family="MS Shell Dlg 2" font-size="9.75" font-weight="400" font-style="normal" > <path vector-effect="none" fill-rule="evenodd" d="M0,1.7 L42.5,1.7 L42.5,0 L50,10 L42.5,20 L42.5,18.3 L0,18.3 L0,1.7"/> </g> <g fill="none" stroke="#000000" stroke-opacity="1" stroke-width="1" stroke-linecap="square" stroke-linejoin="bevel" transform="matrix(1,0,0,1,1041.13,1318)" font-family="MS Shell Dlg 2" font-size="9.75" font-weight="400" font-style="normal" > <text fill="#000000" fill-opacity="1" stroke="none" xml:space="preserve" x="4" y="17" font-family="Arial" font-size="10pt" font-weight="700" font-style="italic" >W</text> </g> <g fill="#ff9999" fill-opacity="1" stroke="#bfbfbf" stroke-opacity="1" stroke-width="1" stroke-linecap="square" stroke-linejoin="bevel" transform="matrix(1,0,0,1,1081,1320)" font-family="MS Shell Dlg 2" font-size="9.75" font-weight="400" font-style="normal" > <path vector-effect="none" fill-rule="evenodd" d="M0,1.7 L42.5,1.7 L42.5,0 L50,10 L42.5,20 L42.5,18.3 L0,18.3 L0,1.7"/> </g> <g fill="none" stroke="#000000" stroke-opacity="1" stroke-width="1" stroke-linecap="square" stroke-linejoin="bevel" transform="matrix(1,0,0,1,1094.63,1318)" font-family="MS Shell Dlg 2" font-size="9.75" font-weight="400" font-style="normal" > <text fill="#000000" fill-opacity="1" stroke="none" xml:space="preserve" x="4" y="17" font-family="Arial" font-size="10pt" font-weight="700" font-style="italic" >K</text> </g> <g fill="#ffff33" fill-opacity="1" stroke="#bfbfbf" stroke-opacity="1" stroke-width="1" stroke-linecap="square" stroke-linejoin="bevel" transform="matrix(1,0,0,1,1132,1320)" font-family="MS Shell Dlg 2" font-size="9.75" font-weight="400" font-style="normal" > <rect x="0" y="0" width="50" height="20"/> </g> <g fill="none" stroke="#000000" stroke-opacity="1" stroke-width="1" stroke-linecap="square" stroke-linejoin="bevel" transform="matrix(1,0,0,1,1139,1318)" font-family="MS Shell Dlg 2" font-size="9.75" font-weight="400" font-style="normal" > <text fill="#000000" fill-opacity="1" stroke="none" xml:space="preserve" x="4" y="17" font-family="Arial" font-size="10pt" font-weight="700" font-style="italic" >nad5</text> </g> <g fill="#ff9999" fill-opacity="1" stroke="#bfbfbf" stroke-opacity="1" stroke-width="1" stroke-linecap="square" stroke-linejoin="bevel" transform="matrix(1,0,0,1,1183,1320)" font-family="MS Shell Dlg

2" font-size="9.75" font-weight="400" font-style="normal" > <path vector-effect="none" fill-rule="evenodd" d="M0,1.7 L42.5,1.7 L42.5,0 L50,10 L42.5,20 L42.5,18.3 L0,18.3 L0,1.7"/> </g> <g fill="none" stroke="#000000" stroke-opacity="1" stroke-width="1" stroke-linecap="square" stroke-linejoin="bevel" transform="matrix(1,0,0,1,1196.13,1318)" font-family="MS Shell Dlg 2" font-size="9.75" font-weight="400" font-style="normal" > <text fill="#000000" fill-opacity="1" stroke="none" xml:space="preserve" x="4" y="17" font-family="Arial" font-size="10pt" font-weight="700" font-style="italic" >S</text> </g> <g fill="#ff9999" fill-opacity="1" stroke="#bfbfbf" stroke-opacity="1" stroke-width="1" stroke-linecap="square" stroke-linejoin="bevel" transform="matrix(1,0,0,1,1234,1320)" font-family="MS Shell Dlg 2" font-size="9.75" font-weight="400" font-style="normal" > <path vector-effect="none" fill-rule="evenodd" d="M0,1.7 L42.5,1.7 L42.5,0 L50,10 L42.5,20 L42.5,18.3 L0,18.3 L0,1.7"/> </g> <g fill="none" stroke="#000000" stroke-opacity="1" stroke-width="1" stroke-linecap="square" stroke-linejoin="bevel" transform="matrix(1,0,0,1,1247.13,1318)" font-family="MS Shell Dlg 2" font-size="9.75" font-weight="400" font-style="normal" > <text fill="#000000" fill-opacity="1" stroke="none" xml:space="preserve" x="4" y="17" font-family="Arial" font-size="10pt" font-weight="700" font-style="italic" >A</text> </g> <g fill="#ff9999" fill-opacity="1" stroke="#bfbfbf" stroke-opacity="1" stroke-width="1" stroke-linecap="square" stroke-linejoin="bevel" transform="matrix(1,0,0,1,1285,1320)" font-family="MS Shell Dlg 2" font-size="9.75" font-weight="400" font-style="normal" > <path vector-effect="none" fill-rule="evenodd" d="M0,1.7 L42.5,1.7 L42.5,0 L50,10 L42.5,20 L42.5,18.3 L0,18.3 L0,1.7"/> </g> <g fill="none" stroke="#000000" stroke-opacity="1" stroke-width="1" stroke-linecap="square" stroke-linejoin="bevel" transform="matrix(1,0,0,1,1299.13,1318)" font-family="MS Shell Dlg 2" font-size="9.75" font-weight="400" font-style="normal" > <text fill="#000000" fill-opacity="1" stroke="none" xml:space="preserve" x="4" y="17" font-family="Arial" font-size="10pt" font-weight="700" font-style="italic" >L</text> </g> <g fill="#99ffff" fill-opacity="1" stroke="#bfbfbf" stroke-opacity="1" stroke-width="1" stroke-linecap="square" stroke-linejoin="bevel" transform="matrix(1,0,0,1,1336,1320)" font-family="MS Shell Dlg 2" font-size="9.75" font-weight="400" font-style="normal" > <path vector-effect="none" fill-rule="evenodd" d="M0,5 C0,2.23858 2.23858,0 5,0 L45,0 C47.7614,0 50,2.23858 50,5 L50,15 C50,17.7614 47.7614,20 45,20 L5,20 C2.23858,20 0,17.7614 0,15 L0,5"/> </g> <g fill="none" stroke="#000000" stroke-opacity="1" stroke-width="1" stroke-linecap="square" stroke-linejoin="bevel" transform="matrix(1,0,0,1,1344.5,1318)" font-family="MS Shell Dlg 2" font-size="9.75" font-weight="400" font-style="normal" > <text fill="#000000" fill-opacity="1" stroke="none" xml:space="preserve" x="4" y="17" font-family="Arial" font-size="10pt" font-weight="700" font-style="italic" >rrnS</text> </g> <g fill="#ff9999" fill-opacity="1" stroke="#bfbfbf" stroke-opacity="1" stroke-width="1" stroke-linecap="square" stroke-linejoin="bevel" transform="matrix(1,0,0,1,1387,1320)" font-family="MS Shell Dlg 2" font-size="9.75" font-weight="400" font-style="normal" > <path vector-effect="none" fill-rule="evenodd" d="M0,1.7 L42.5,1.7 L42.5,0 L50,10 L42.5,20 L42.5,18.3 L0,18.3 L0,1.7"/> </g> <g fill="none" stroke="#000000" stroke-opacity="1" stroke-width="1" stroke-linecap="square" stroke-linejoin="bevel" transform="matrix(1,0,0,1,1402.13,1318)" font-family="MS Shell Dlg 2" font-size="9.75" font-weight="400" font-style="normal" > <text fill="#000000" fill-opacity="1" stroke="none" xml:space="preserve" x="4" y="17" font-family="Arial" font-size="10pt" font-weight="700" font-style="italic" >I</text> </g> <g fill="#ff9999" fill-opacity="1" stroke="#bfbfbf" stroke-opacity="1" stroke-width="1" stroke-linecap="square" stroke-linejoin="bevel" transform="matrix(1,0,0,1,1438,1320)" font-family="MS Shell Dlg 2" font-size="9.75" font-weight="400" font-style="normal" > <path vector-effect="none" fill-rule="evenodd" d="M0,1.7 L42.5,1.7 L42.5,0 L50,10 L42.5,20 L42.5,18.3 L0,18.3 L0,1.7"/> </g> <g fill="none" stroke="#000000" stroke-opacity="1" stroke-width="1" stroke-linecap="square" stroke-linejoin="bevel" transform="matrix(1,0,0,1,1451.13,1318)" font-family="MS Shell Dlg 2" font-size="9.75" font-weight="400" font-style="normal" > <text fill="#000000" fill-opacity="1" stroke="none" xml:space="preserve" x="4" y="17" font-family="Arial" font-size="10pt" font-weight="700" font-style="italic" >G</text> </g> <g fill="#ff9999" fill-opacity="1" stroke="#bfbfbf" stroke-opacity="1" stroke-width="1" stroke-linecap="square" stroke-linejoin="bevel" transform="matrix(1,0,0,1,1489,1320)" font-family="MS Shell Dlg 2" font-size="9.75" font-weight="400" font-style="normal" > <path vector-effect="none" fill-rule="evenodd" d="M0,1.7 L42.5,1.7 L42.5,0 L50,10 L42.5,20 L42.5,18.3 L0,18.3 L0,1.7"/> </g> <g fill="none" stroke="#000000" stroke-opacity="1" stroke-width="1" stroke-linecap="square" stroke-linejoin="bevel" transform="matrix(1,0,0,1,1502.13,1318)" font-family="MS Shell Dlg 2" font-size="9.75" font-weight="400" font-style="normal" > <text fill="#000000" fill-opacity="1" stroke="none" xml:space="preserve" x="4" y="17" font-family="Arial" font-size="10pt" font-weight="700" font-style="italic" >V</text> </g> <g fill="#99ffff" fill-opacity="1" stroke="#bfbfbf" stroke-opacity="1" stroke-width="1" stroke-linecap="square" stroke-linejoin="bevel" transform="matrix(1,0,0,1,1540,1320)" font-family="MS Shell Dlg 2" font-size="9.75" font-weight="400" font-style="normal" > <path vector-effect="none" fill-rule="evenodd" d="M0,5 C0,2.23858 2.23858,0 5,0 L45,0 C47.7614,0 50,2.23858 50,5 L50,15 C50,17.7614 47.7614,20 45,20 L5,20 C2.23858,20 0,17.7614 0,15 L0,5"/> </g> <g fill="none" stroke="#000000" stroke-

opacity="1" stroke-width="1" stroke-linecap="square" stroke-linejoin="bevel"
transform="matrix(1,0,0,1,1549.5,1318)" font-family="MS Shell Dlg 2" font-size="9.75" font-weight="400" font-
style="normal" > <text fill="#000000" fill-opacity="1" stroke="none" xml:space="preserve" x="4" y="17" font-
family="Arial" font-size="10pt" font-weight="700" font-style="italic" >rrnL</text> </g> <g
fill="#ff9999" fill-opacity="1" stroke="#bfbfbf" stroke-opacity="1" stroke-width="1" stroke-linecap="square"
stroke-linejoin="bevel" transform="matrix(1,0,0,1,1591,1320)" font-family="MS Shell Dlg 2" font-size="9.75"
font-weight="400" font-style="normal" > <path vector-effect="none" fill-rule="evenodd" d="M0,1.7 L42.5,1.7
L42.5,0 L50,10 L42.5,20 L42.5,18.3 L0,18.3 L0,1.7"/> </g> <g fill="none" stroke="#000000" stroke-
opacity="1" stroke-width="1" stroke-linecap="square" stroke-linejoin="bevel"
transform="matrix(1,0,0,1,1603.13,1318)" font-family="MS Shell Dlg 2" font-size="9.75" font-weight="400" font-
style="normal" > <text fill="#000000" fill-opacity="1" stroke="none" xml:space="preserve" x="4" y="17" font-
family="Arial" font-size="10pt" font-weight="700" font-style="italic" >M</text> </g> <g fill="#ff9999"
fill-opacity="1" stroke="#bfbfbf" stroke-opacity="1" stroke-width="1" stroke-linecap="square" stroke-
linejoin="bevel" transform="matrix(1,0,0,1,1642,1320)" font-family="MS Shell Dlg 2" font-size="9.75" font-
weight="400" font-style="normal" > <path vector-effect="none" fill-rule="evenodd" d="M0,1.7 L42.5,1.7 L42.5,0
L50,10 L42.5,20 L42.5,18.3 L0,18.3 L0,1.7"/> </g> <g fill="none" stroke="#000000" stroke-opacity="1"
stroke-width="1" stroke-linecap="square" stroke-linejoin="bevel" transform="matrix(1,0,0,1,1655.63,1318)" font-
family="MS Shell Dlg 2" font-size="9.75" font-weight="400" font-style="normal" > <text fill="#000000" fill-
opacity="1" stroke="none" xml:space="preserve" x="4" y="17" font-family="Arial" font-size="10pt" font-
weight="700" font-style="italic" >F</text> </g> <g fill="#ff9999" fill-opacity="1" stroke="#bfbfbf"
stroke-opacity="1" stroke-width="1" stroke-linecap="square" stroke-linejoin="bevel"
transform="matrix(1,0,0,1,1693,1320)" font-family="MS Shell Dlg 2" font-size="9.75" font-weight="400" font-
style="normal" > <path vector-effect="none" fill-rule="evenodd" d="M0,1.7 L42.5,1.7 L42.5,0 L50,10 L42.5,20
L42.5,18.3 L0,18.3 L0,1.7"/> </g> <g fill="none" stroke="#000000" stroke-opacity="1" stroke-width="1"
stroke-linecap="square" stroke-linejoin="bevel" transform="matrix(1,0,0,1,1706.13,1318)" font-family="MS Shell
Dlg 2" font-size="9.75" font-weight="400" font-style="normal" > <text fill="#000000" fill-opacity="1"
stroke="none" xml:space="preserve" x="4" y="17" font-family="Arial" font-size="10pt" font-weight="700" font-
style="italic" >H</text> </g> <g fill="#ffff33" fill-opacity="1" stroke="#bfbfbf" stroke-opacity="1"
stroke-width="1" stroke-linecap="square" stroke-linejoin="bevel" transform="matrix(1,0,0,1,1744,1320)" font-
family="MS Shell Dlg 2" font-size="9.75" font-weight="400" font-style="normal" > <rect x="0" y="0" width="50"
height="20"/> </g> <g fill="none" stroke="#000000" stroke-opacity="1" stroke-width="1" stroke-
linecap="square" stroke-linejoin="bevel" transform="matrix(1,0,0,1,1752,1318)" font-family="MS Shell Dlg 2"
font-size="9.75" font-weight="400" font-style="normal" > <text fill="#000000" fill-opacity="1" stroke="none"
xml:space="preserve" x="4" y="17" font-family="Arial" font-size="10pt" font-weight="700" font-style="italic"
>cox3</text> </g> <g fill="#ffff33" fill-opacity="1" stroke="#bfbfbf" stroke-opacity="1" stroke-width="1"
stroke-linecap="square" stroke-linejoin="bevel" transform="matrix(1,0,0,1,1795,1320)" font-family="MS Shell Dlg
2" font-size="9.75" font-weight="400" font-style="normal" > <rect x="0" y="0" width="50" height="20"/> </g>
<g fill="none" stroke="#000000" stroke-opacity="1" stroke-width="1" stroke-linecap="square" stroke-
linejoin="bevel" transform="matrix(1,0,0,1,1802,1318)" font-family="MS Shell Dlg 2" font-size="9.75" font-
weight="400" font-style="normal" > <text fill="#000000" fill-opacity="1" stroke="none" xml:space="preserve"
x="4" y="17" font-family="Arial" font-size="10pt" font-weight="700" font-style="italic" >nad2</text> </g>
<g fill="#ffff33" fill-opacity="1" stroke="#bfbfbf" stroke-opacity="1" stroke-width="1" stroke-linecap="square"
stroke-linejoin="bevel" transform="matrix(1,0,0,1,1846,1320)" font-family="MS Shell Dlg 2" font-size="9.75"
font-weight="400" font-style="normal" > <rect x="0" y="0" width="50" height="20"/> </g> <g fill="none"
stroke="#000000" stroke-opacity="1" stroke-width="1" stroke-linecap="square" stroke-linejoin="bevel"
transform="matrix(1,0,0,1,1853,1318)" font-family="MS Shell Dlg 2" font-size="9.75" font-weight="400" font-
style="normal" > <text fill="#000000" fill-opacity="1" stroke="none" xml:space="preserve" x="4" y="17" font-
family="Arial" font-size="10pt" font-weight="700" font-style="italic" >nad4</text> </g> <g fill="#ffff33"
fill-opacity="1" stroke="#bfbfbf" stroke-opacity="1" stroke-width="1" stroke-linecap="square" stroke-
linejoin="bevel" transform="matrix(1,0,0,1,1897,1320)" font-family="MS Shell Dlg 2" font-size="9.75" font-
weight="400" font-style="normal" > <rect x="0" y="0" width="50" height="20"/> </g> <g fill="none"
stroke="#000000" stroke-opacity="1" stroke-width="1" stroke-linecap="square" stroke-linejoin="bevel"
transform="matrix(1,0,0,1,1905.5,1318)" font-family="MS Shell Dlg 2" font-size="9.75" font-weight="400" font-
style="normal" > <text fill="#000000" fill-opacity="1" stroke="none" xml:space="preserve" x="4" y="17" font-
family="Arial" font-size="10pt" font-weight="700" font-style="italic" >atp8</text> </g> <g fill="none"
stroke="#000000" stroke-opacity="1" stroke-width="1" stroke-linecap="square" stroke-linejoin="bevel"
transform="matrix(1,0,0,1,1948,1320)" font-family="MS Shell Dlg 2" font-size="9.75" font-weight="400" font-
style="normal" > <text fill="#000000" fill-opacity="1" stroke="none" xml:space="preserve" x="4" y="17" font-

family="Arial" font-size="10pt" font-weight="700" font-style="italic" >(+)</text> </g> <g fill="none"
stroke="#000000" stroke-opacity="1" stroke-width="1" stroke-linecap="square" stroke-linejoin="bevel"
transform="matrix(1,0,0,1,10,1360)" font-family="MS Shell Dlg 2" font-size="9.75" font-weight="400" font-
style="normal" > <text fill="#000000" fill-opacity="1" stroke="none" xml:space="preserve" x="4" y="17" font-
family="Arial" font-size="10pt" font-weight="700" font-style="italic" >Perna_viridis_MW727515</text> </g>
<g fill="#ffff33" fill-opacity="1" stroke="#bfbfbf" stroke-opacity="1" stroke-width="1" stroke-linecap="square"
stroke-linejoin="bevel" transform="matrix(1,0,0,1,10,1390)" font-family="MS Shell Dlg 2" font-size="9.75" font-
weight="400" font-style="normal" > <rect x="0" y="0" width="50" height="20"/> </g> <g fill="none"
stroke="#000000" stroke-opacity="1" stroke-width="1" stroke-linecap="square" stroke-linejoin="bevel"
transform="matrix(1,0,0,1,18,1388)" font-family="MS Shell Dlg 2" font-size="9.75" font-weight="400" font-
style="normal" > <text fill="#000000" fill-opacity="1" stroke="none" xml:space="preserve" x="4" y="17" font-
family="Arial" font-size="10pt" font-weight="700" font-style="italic" >cox1</text> </g> <g fill="#ffff33"
fill-opacity="1" stroke="#bfbfbf" stroke-opacity="1" stroke-width="1" stroke-linecap="square" stroke-
linejoin="bevel" transform="matrix(1,0,0,1,61,1390)" font-family="MS Shell Dlg 2" font-size="9.75" font-
weight="400" font-style="normal" > <rect x="0" y="0" width="50" height="20"/> </g> <g fill="none"
stroke="#000000" stroke-opacity="1" stroke-width="1" stroke-linecap="square" stroke-linejoin="bevel"
transform="matrix(1,0,0,1,68,1388)" font-family="MS Shell Dlg 2" font-size="9.75" font-weight="400" font-
style="normal" > <text fill="#000000" fill-opacity="1" stroke="none" xml:space="preserve" x="4" y="17" font-
family="Arial" font-size="10pt" font-weight="700" font-style="italic" >nad4</text> </g> <g fill="#ffff33"
fill-opacity="1" stroke="#bfbfbf" stroke-opacity="1" stroke-width="1" stroke-linecap="square" stroke-
linejoin="bevel" transform="matrix(1,0,0,1,112,1390)" font-family="MS Shell Dlg 2" font-size="9.75" font-
weight="400" font-style="normal" > <rect x="0" y="0" width="50" height="20"/> </g> <g fill="none"
stroke="#000000" stroke-opacity="1" stroke-width="1" stroke-linecap="square" stroke-linejoin="bevel"
transform="matrix(1,0,0,1,120.5,1388)" font-family="MS Shell Dlg 2" font-size="9.75" font-weight="400" font-
style="normal" > <text fill="#000000" fill-opacity="1" stroke="none" xml:space="preserve" x="4" y="17" font-
family="Arial" font-size="10pt" font-weight="700" font-style="italic" >atp6</text> </g> <g
fill="#ff9999" fill-opacity="1" stroke="#bfbfbf" stroke-opacity="1" stroke-width="1" stroke-linecap="square"
stroke-linejoin="bevel" transform="matrix(1,0,0,1,163,1390)" font-family="MS Shell Dlg 2" font-size="9.75" font-
weight="400" font-style="normal" > <path vector-effect="none" fill-rule="evenodd" d="M0,1.7 L42.5,1.7 L42.5,0
L50,10 L42.5,20 L42.5,18.3 L0,18.3 L0,1.7"/> </g> <g fill="none" stroke="#000000" stroke-opacity="1"
stroke-width="1" stroke-linecap="square" stroke-linejoin="bevel" transform="matrix(1,0,0,1,176.132,1388)" font-
family="MS Shell Dlg 2" font-size="9.75" font-weight="400" font-style="normal" > <text fill="#000000" fill-
opacity="1" stroke="none" xml:space="preserve" x="4" y="17" font-family="Arial" font-size="10pt" font-
weight="700" font-style="italic" >S</text> </g> <g fill="#ffff33" fill-opacity="1" stroke="#bfbfbf"
stroke-opacity="1" stroke-width="1" stroke-linecap="square" stroke-linejoin="bevel"
transform="matrix(1,0,0,1,214,1390)" font-family="MS Shell Dlg 2" font-size="9.75" font-weight="400" font-
style="normal" > <rect x="0" y="0" width="50" height="20"/> </g> <g fill="none" stroke="#000000" stroke-
opacity="1" stroke-width="1" stroke-linecap="square" stroke-linejoin="bevel"
transform="matrix(1,0,0,1,223.5,1388)" font-family="MS Shell Dlg 2" font-size="9.75" font-weight="400" font-
style="normal" > <text fill="#000000" fill-opacity="1" stroke="none" xml:space="preserve" x="4" y="17" font-
family="Arial" font-size="10pt" font-weight="700" font-style="italic" >cytb</text> </g> <g
fill="#ff9999" fill-opacity="1" stroke="#bfbfbf" stroke-opacity="1" stroke-width="1" stroke-linecap="square"
stroke-linejoin="bevel" transform="matrix(1,0,0,1,265,1390)" font-family="MS Shell Dlg 2" font-size="9.75" font-
weight="400" font-style="normal" > <path vector-effect="none" fill-rule="evenodd" d="M0,1.7 L42.5,1.7 L42.5,0
L50,10 L42.5,20 L42.5,18.3 L0,18.3 L0,1.7"/> </g> <g fill="none" stroke="#000000" stroke-opacity="1"
stroke-width="1" stroke-linecap="square" stroke-linejoin="bevel" transform="matrix(1,0,0,1,278.132,1388)" font-
family="MS Shell Dlg 2" font-size="9.75" font-weight="400" font-style="normal" > <text fill="#000000" fill-
opacity="1" stroke="none" xml:space="preserve" x="4" y="17" font-family="Arial" font-size="10pt" font-
weight="700" font-style="italic" >A</text> </g> <g fill="#ffff33" fill-opacity="1" stroke="#bfbfbf"
stroke-opacity="1" stroke-width="1" stroke-linecap="square" stroke-linejoin="bevel"
transform="matrix(1,0,0,1,316,1390)" font-family="MS Shell Dlg 2" font-size="9.75" font-weight="400" font-
style="normal" > <rect x="0" y="0" width="50" height="20"/> </g> <g fill="none" stroke="#000000" stroke-
opacity="1" stroke-width="1" stroke-linecap="square" stroke-linejoin="bevel"
transform="matrix(1,0,0,1,324,1388)" font-family="MS Shell Dlg 2" font-size="9.75" font-weight="400" font-
style="normal" > <text fill="#000000" fill-opacity="1" stroke="none" xml:space="preserve" x="4" y="17" font-
family="Arial" font-size="10pt" font-weight="700" font-style="italic" >cox2</text> </g> <g
fill="#ff9999" fill-opacity="1" stroke="#bfbfbf" stroke-opacity="1" stroke-width="1" stroke-linecap="square"
stroke-linejoin="bevel" transform="matrix(1,0,0,1,367,1390)" font-family="MS Shell Dlg 2" font-size="9.75" font-

weight="400" font-style="normal" > <path vector-effect="none" fill-rule="evenodd" d="M0,1.7 L42.5,1.7 L42.5,0 L50,10 L42.5,20 L42.5,18.3 L0,18.3 L0,1.7"/> </g> <g fill="none" stroke="#000000" stroke-opacity="1" stroke-width="1" stroke-linecap="square" stroke-linejoin="bevel" transform="matrix(1,0,0,1,380.132,1388)" font-family="MS Shell Dlg 2" font-size="9.75" font-weight="400" font-style="normal" > <text fill="#000000" fill-opacity="1" stroke="none" xml:space="preserve" x="4" y="17" font-family="Arial" font-size="10pt" font-weight="700" font-style="italic" >R</text> </g> <g fill="#ff9999" fill-opacity="1" stroke="#bfbfbf" stroke-opacity="1" stroke-width="1" stroke-linecap="square" stroke-linejoin="bevel" transform="matrix(1,0,0,1,418,1390)" font-family="MS Shell Dlg 2" font-size="9.75" font-weight="400" font-style="normal" > <path vector-effect="none" fill-rule="evenodd" d="M0,1.7 L42.5,1.7 L42.5,0 L50,10 L42.5,20 L42.5,18.3 L0,18.3 L0,1.7"/> </g> <g fill="none" stroke="#000000" stroke-opacity="1" stroke-width="1" stroke-linecap="square" stroke-linejoin="bevel" transform="matrix(1,0,0,1,430.132,1388)" font-family="MS Shell Dlg 2" font-size="9.75" font-weight="400" font-style="normal" > <text fill="#000000" fill-opacity="1" stroke="none" xml:space="preserve" x="4" y="17" font-family="Arial" font-size="10pt" font-weight="700" font-style="italic" >M</text> </g> <g fill="#ff9999" fill-opacity="1" stroke="#bfbfbf" stroke-opacity="1" stroke-width="1" stroke-linecap="square" stroke-linejoin="bevel" transform="matrix(1,0,0,1,469,1390)" font-family="MS Shell Dlg 2" font-size="9.75" font-weight="400" font-style="normal" > <path vector-effect="none" fill-rule="evenodd" d="M0,1.7 L42.5,1.7 L42.5,0 L50,10 L42.5,20 L42.5,18.3 L0,18.3 L0,1.7"/> </g> <g fill="none" stroke="#000000" stroke-opacity="1" stroke-width="1" stroke-linecap="square" stroke-linejoin="bevel" transform="matrix(1,0,0,1,482.132,1388)" font-family="MS Shell Dlg 2" font-size="9.75" font-weight="400" font-style="normal" > <text fill="#000000" fill-opacity="1" stroke="none" xml:space="preserve" x="4" y="17" font-family="Arial" font-size="10pt" font-weight="700" font-style="italic" >D</text> </g> <g fill="#ffff33" fill-opacity="1" stroke="#bfbfbf" stroke-opacity="1" stroke-width="1" stroke-linecap="square" stroke-linejoin="bevel" transform="matrix(1,0,0,1,520,1390)" font-family="MS Shell Dlg 2" font-size="9.75" font-weight="400" font-style="normal" > <rect x="0" y="0" width="50" height="20"/> </g> <g fill="none" stroke="#000000" stroke-opacity="1" stroke-width="1" stroke-linecap="square" stroke-linejoin="bevel" transform="matrix(1,0,0,1,527,1388)" font-family="MS Shell Dlg 2" font-size="9.75" font-weight="400" font-style="normal" > <text fill="#000000" fill-opacity="1" stroke="none" xml:space="preserve" x="4" y="17" font-family="Arial" font-size="10pt" font-weight="700" font-style="italic" >nad1</text> </g> <g fill="#ffff33" fill-opacity="1" stroke="#bfbfbf" stroke-opacity="1" stroke-width="1" stroke-linecap="square" stroke-linejoin="bevel" transform="matrix(1,0,0,1,571,1390)" font-family="MS Shell Dlg 2" font-size="9.75" font-weight="400" font-style="normal" > <rect x="0" y="0" width="50" height="20"/> </g> <g fill="none" stroke="#000000" stroke-opacity="1" stroke-width="1" stroke-linecap="square" stroke-linejoin="bevel" transform="matrix(1,0,0,1,575,1388)" font-family="MS Shell Dlg 2" font-size="9.75" font-weight="400" font-style="normal" > <text fill="#000000" fill-opacity="1" stroke="none" xml:space="preserve" x="4" y="17" font-family="Arial" font-size="10pt" font-weight="700" font-style="italic" >nad4L</text> </g> <g fill="#ff9999" fill-opacity="1" stroke="#bfbfbf" stroke-opacity="1" stroke-width="1" stroke-linecap="square" stroke-linejoin="bevel" transform="matrix(1,0,0,1,622,1390)" font-family="MS Shell Dlg 2" font-size="9.75" font-weight="400" font-style="normal" > <path vector-effect="none" fill-rule="evenodd" d="M0,1.7 L42.5,1.7 L42.5,0 L50,10 L42.5,20 L42.5,18.3 L0,18.3 L0,1.7"/> </g> <g fill="none" stroke="#000000" stroke-opacity="1" stroke-width="1" stroke-linecap="square" stroke-linejoin="bevel" transform="matrix(1,0,0,1,635.633,1388)" font-family="MS Shell Dlg 2" font-size="9.75" font-weight="400" font-style="normal" > <text fill="#000000" fill-opacity="1" stroke="none" xml:space="preserve" x="4" y="17" font-family="Arial" font-size="10pt" font-weight="700" font-style="italic" >Y</text> </g> <g fill="#ffff33" fill-opacity="1" stroke="#bfbfbf" stroke-opacity="1" stroke-width="1" stroke-linecap="square" stroke-linejoin="bevel" transform="matrix(1,0,0,1,673,1390)" font-family="MS Shell Dlg 2" font-size="9.75" font-weight="400" font-style="normal" > <rect x="0" y="0" width="50" height="20"/> </g> <g fill="none" stroke="#000000" stroke-opacity="1" stroke-width="1" stroke-linecap="square" stroke-linejoin="bevel" transform="matrix(1,0,0,1,680,1388)" font-family="MS Shell Dlg 2" font-size="9.75" font-weight="400" font-style="normal" > <text fill="#000000" fill-opacity="1" stroke="none" xml:space="preserve" x="4" y="17" font-family="Arial" font-size="10pt" font-weight="700" font-style="italic" >nad3</text> </g> <g fill="#ff9999" fill-opacity="1" stroke="#bfbfbf" stroke-opacity="1" stroke-width="1" stroke-linecap="square" stroke-linejoin="bevel" transform="matrix(1,0,0,1,724,1390)" font-family="MS Shell Dlg 2" font-size="9.75" font-weight="400" font-style="normal" > <path vector-effect="none" fill-rule="evenodd" d="M0,1.7 L42.5,1.7 L42.5,0 L50,10 L42.5,20 L42.5,18.3 L0,18.3 L0,1.7"/> </g> <g fill="none" stroke="#000000" stroke-opacity="1" stroke-width="1" stroke-linecap="square" stroke-linejoin="bevel" transform="matrix(1,0,0,1,737.133,1388)" font-family="MS Shell Dlg 2" font-size="9.75" font-weight="400" font-style="normal" > <text fill="#000000" fill-opacity="1" stroke="none" xml:space="preserve" x="4" y="17" font-family="Arial" font-size="10pt" font-weight="700" font-style="italic" >T</text> </g> <g fill="#ffff33" fill-opacity="1" stroke="#bfbfbf"

stroke-opacity="1" stroke-width="1" stroke-linecap="square" stroke-linejoin="bevel" transform="matrix(1,0,0,1,775,1390)" font-family="MS Shell Dlg 2" font-size="9.75" font-weight="400" font-style="normal" > <rect x="0" y="0" width="50" height="20"/> </g> <g fill="none" stroke="#000000" stroke-opacity="1" stroke-width="1" stroke-linecap="square" stroke-linejoin="bevel" transform="matrix(1,0,0,1,782,1388)" font-family="MS Shell Dlg 2" font-size="9.75" font-weight="400" font-style="normal" > <text fill="#000000" fill-opacity="1" stroke="none" xml:space="preserve" x="4" y="17" font-family="Arial" font-size="10pt" font-weight="700" font-style="italic" >nad2</text> </g> <g fill="#ff9999" fill-opacity="1" stroke="#bfbfbf" stroke-opacity="1" stroke-width="1" stroke-linecap="square" stroke-linejoin="bevel" transform="matrix(1,0,0,1,826,1390)" font-family="MS Shell Dlg 2" font-size="9.75" font-weight="400" font-style="normal" > <path vector-effect="none" fill-rule="evenodd" d="M0,1.7 L42.5,1.7 L42.5,0 L50,10 L42.5,20 L42.5,18.3 L0,18.3 L0,1.7"/> </g> <g fill="none" stroke="#000000" stroke-opacity="1" stroke-width="1" stroke-linecap="square" stroke-linejoin="bevel" transform="matrix(1,0,0,1,839.633,1388)" font-family="MS Shell Dlg 2" font-size="9.75" font-weight="400" font-style="normal" > <text fill="#000000" fill-opacity="1" stroke="none" xml:space="preserve" x="4" y="17" font-family="Arial" font-size="10pt" font-weight="700" font-style="italic" >E</text> </g> <g fill="#ff9999" fill-opacity="1" stroke="#bfbfbf" stroke-opacity="1" stroke-width="1" stroke-linecap="square" stroke-linejoin="bevel" transform="matrix(1,0,0,1,877,1390)" font-family="MS Shell Dlg 2" font-size="9.75" font-weight="400" font-style="normal" > <path vector-effect="none" fill-rule="evenodd" d="M0,1.7 L42.5,1.7 L42.5,0 L50,10 L42.5,20 L42.5,18.3 L0,18.3 L0,1.7"/> </g> <g fill="none" stroke="#000000" stroke-opacity="1" stroke-width="1" stroke-linecap="square" stroke-linejoin="bevel" transform="matrix(1,0,0,1,889.633,1388)" font-family="MS Shell Dlg 2" font-size="9.75" font-weight="400" font-style="normal" > <text fill="#000000" fill-opacity="1" stroke="none" xml:space="preserve" x="4" y="17" font-family="Arial" font-size="10pt" font-weight="700" font-style="italic" >Q</text> </g> <g fill="#ff9999" fill-opacity="1" stroke="#bfbfbf" stroke-opacity="1" stroke-width="1" stroke-linecap="square" stroke-linejoin="bevel" transform="matrix(1,0,0,1,928,1390)" font-family="MS Shell Dlg 2" font-size="9.75" font-weight="400" font-style="normal" > <path vector-effect="none" fill-rule="evenodd" d="M0,1.7 L42.5,1.7 L42.5,0 L50,10 L42.5,20 L42.5,18.3 L0,18.3 L0,1.7"/> </g> <g fill="none" stroke="#000000" stroke-opacity="1" stroke-width="1" stroke-linecap="square" stroke-linejoin="bevel" transform="matrix(1,0,0,1,941.133,1388)" font-family="MS Shell Dlg 2" font-size="9.75" font-weight="400" font-style="normal" > <text fill="#000000" fill-opacity="1" stroke="none" xml:space="preserve" x="4" y="17" font-family="Arial" font-size="10pt" font-weight="700" font-style="italic" >S</text> </g> <g fill="#ff9999" fill-opacity="1" stroke="#bfbfbf" stroke-opacity="1" stroke-width="1" stroke-linecap="square" stroke-linejoin="bevel" transform="matrix(1,0,0,1,979,1390)" font-family="MS Shell Dlg 2" font-size="9.75" font-weight="400" font-style="normal" > <path vector-effect="none" fill-rule="evenodd" d="M0,1.7 L42.5,1.7 L42.5,0 L50,10 L42.5,20 L42.5,18.3 L0,18.3 L0,1.7"/> </g> <g fill="none" stroke="#000000" stroke-opacity="1" stroke-width="1" stroke-linecap="square" stroke-linejoin="bevel" transform="matrix(1,0,0,1,992.633,1388)" font-family="MS Shell Dlg 2" font-size="9.75" font-weight="400" font-style="normal" > <text fill="#000000" fill-opacity="1" stroke="none" xml:space="preserve" x="4" y="17" font-family="Arial" font-size="10pt" font-weight="700" font-style="italic" >P</text> </g> <g fill="#ff9999" fill-opacity="1" stroke="#bfbfbf" stroke-opacity="1" stroke-width="1" stroke-linecap="square" stroke-linejoin="bevel" transform="matrix(1,0,0,1,1030,1390)" font-family="MS Shell Dlg 2" font-size="9.75" font-weight="400" font-style="normal" > <path vector-effect="none" fill-rule="evenodd" d="M0,1.7 L42.5,1.7 L42.5,0 L50,10 L42.5,20 L42.5,18.3 L0,18.3 L0,1.7"/> </g> <g fill="none" stroke="#000000" stroke-opacity="1" stroke-width="1" stroke-linecap="square" stroke-linejoin="bevel" transform="matrix(1,0,0,1,1044.13,1388)" font-family="MS Shell Dlg 2" font-size="9.75" font-weight="400" font-style="normal" > <text fill="#000000" fill-opacity="1" stroke="none" xml:space="preserve" x="4" y="17" font-family="Arial" font-size="10pt" font-weight="700" font-style="italic" >L</text> </g> <g fill="#ff9999" fill-opacity="1" stroke="#bfbfbf" stroke-opacity="1" stroke-width="1" stroke-linecap="square" stroke-linejoin="bevel" transform="matrix(1,0,0,1,1081,1390)" font-family="MS Shell Dlg 2" font-size="9.75" font-weight="400" font-style="normal" > <path vector-effect="none" fill-rule="evenodd" d="M0,1.7 L42.5,1.7 L42.5,0 L50,10 L42.5,20 L42.5,18.3 L0,18.3 L0,1.7"/> </g> <g fill="none" stroke="#000000" stroke-opacity="1" stroke-width="1" stroke-linecap="square" stroke-linejoin="bevel" transform="matrix(1,0,0,1,1094.13,1388)" font-family="MS Shell Dlg 2" font-size="9.75" font-weight="400" font-style="normal" > <text fill="#000000" fill-opacity="1" stroke="none" xml:space="preserve" x="4" y="17" font-family="Arial" font-size="10pt" font-weight="700" font-style="italic" >N</text> </g> <g fill="#ffff33" fill-opacity="1" stroke="#bfbfbf" stroke-opacity="1" stroke-width="1" stroke-linecap="square" stroke-linejoin="bevel" transform="matrix(1,0,0,1,1132,1390)" font-family="MS Shell Dlg 2" font-size="9.75" font-weight="400" font-style="normal" > <rect x="0" y="0" width="50" height="20"/> </g> <g fill="none" stroke="#000000" stroke-opacity="1" stroke-width="1" stroke-linecap="square" stroke-linejoin="bevel" transform="matrix(1,0,0,1,1139,1388)" font-family="MS Shell Dlg 2" font-size="9.75" font-weight="400" font-

style="normal" > <text fill="#000000" fill-opacity="1" stroke="none" xml:space="preserve" x="4" y="17" font-family="Arial" font-size="10pt" font-weight="700" font-style="italic" >nad6</text> </g> <g fill="#ff9999" fill-opacity="1" stroke="#bfbfbf" stroke-opacity="1" stroke-width="1" stroke-linecap="square" stroke-linejoin="bevel" transform="matrix(1,0,0,1,1183,1390)" font-family="MS Shell Dlg 2" font-size="9.75" font-weight="400" font-style="normal" > <path vector-effect="none" fill-rule="evenodd" d="M0,1.7 L42.5,1.7 L42.5,0 L50,10 L42.5,20 L42.5,18.3 L0,18.3 L0,1.7"/> </g> <g fill="none" stroke="#000000" stroke-opacity="1" stroke-width="1" stroke-linecap="square" stroke-linejoin="bevel" transform="matrix(1,0,0,1,1196.13,1388)" font-family="MS Shell Dlg 2" font-size="9.75" font-weight="400" font-style="normal" > <text fill="#000000" fill-opacity="1" stroke="none" xml:space="preserve" x="4" y="17" font-family="Arial" font-size="10pt" font-weight="700" font-style="italic" >C</text> </g> <g fill="#ff9999" fill-opacity="1" stroke="#bfbfbf" stroke-opacity="1" stroke-width="1" stroke-linecap="square" stroke-linejoin="bevel" transform="matrix(1,0,0,1,1234,1390)" font-family="MS Shell Dlg 2" font-size="9.75" font-weight="400" font-style="normal" > <path vector-effect="none" fill-rule="evenodd" d="M0,1.7 L42.5,1.7 L42.5,0 L50,10 L42.5,20 L42.5,18.3 L0,18.3 L0,1.7"/> </g> <g fill="none" stroke="#000000" stroke-opacity="1" stroke-width="1" stroke-linecap="square" stroke-linejoin="bevel" transform="matrix(1,0,0,1,1245.13,1388)" font-family="MS Shell Dlg 2" font-size="9.75" font-weight="400" font-style="normal" > <text fill="#000000" fill-opacity="1" stroke="none" xml:space="preserve" x="4" y="17" font-family="Arial" font-size="10pt" font-weight="700" font-style="italic" >W</text> </g> <g fill="#ff9999" fill-opacity="1" stroke="#bfbfbf" stroke-opacity="1" stroke-width="1" stroke-linecap="square" stroke-linejoin="bevel" transform="matrix(1,0,0,1,1285,1390)" font-family="MS Shell Dlg 2" font-size="9.75" font-weight="400" font-style="normal" > <path vector-effect="none" fill-rule="evenodd" d="M0,1.7 L42.5,1.7 L42.5,0 L50,10 L42.5,20 L42.5,18.3 L0,18.3 L0,1.7"/> </g> <g fill="none" stroke="#000000" stroke-opacity="1" stroke-width="1" stroke-linecap="square" stroke-linejoin="bevel" transform="matrix(1,0,0,1,1298.63,1388)" font-family="MS Shell Dlg 2" font-size="9.75" font-weight="400" font-style="normal" > <text fill="#000000" fill-opacity="1" stroke="none" xml:space="preserve" x="4" y="17" font-family="Arial" font-size="10pt" font-weight="700" font-style="italic" >K</text> </g> <g fill="#ffff33" fill-opacity="1" stroke="#bfbfbf" stroke-opacity="1" stroke-width="1" stroke-linecap="square" stroke-linejoin="bevel" transform="matrix(1,0,0,1,1336,1390)" font-family="MS Shell Dlg 2" font-size="9.75" font-weight="400" font-style="normal" > <rect x="0" y="0" width="50" height="20"/> </g> <g fill="none" stroke="#000000" stroke-opacity="1" stroke-width="1" stroke-linecap="square" stroke-linejoin="bevel" transform="matrix(1,0,0,1,1343,1388)" font-family="MS Shell Dlg 2" font-size="9.75" font-weight="400" font-style="normal" > <text fill="#000000" fill-opacity="1" stroke="none" xml:space="preserve" x="4" y="17" font-family="Arial" font-size="10pt" font-weight="700" font-style="italic" >nad5</text> </g> <g fill="#ff9999" fill-opacity="1" stroke="#bfbfbf" stroke-opacity="1" stroke-width="1" stroke-linecap="square" stroke-linejoin="bevel" transform="matrix(1,0,0,1,1387,1390)" font-family="MS Shell Dlg 2" font-size="9.75" font-weight="400" font-style="normal" > <path vector-effect="none" fill-rule="evenodd" d="M0,1.7 L42.5,1.7 L42.5,0 L50,10 L42.5,20 L42.5,18.3 L0,18.3 L0,1.7"/> </g> <g fill="none" stroke="#000000" stroke-opacity="1" stroke-width="1" stroke-linecap="square" stroke-linejoin="bevel" transform="matrix(1,0,0,1,1401.13,1388)" font-family="MS Shell Dlg 2" font-size="9.75" font-weight="400" font-style="normal" > <text fill="#000000" fill-opacity="1" stroke="none" xml:space="preserve" x="4" y="17" font-family="Arial" font-size="10pt" font-weight="700" font-style="italic" >L</text> </g> <g fill="#99ffff" fill-opacity="1" stroke="#bfbfbf" stroke-opacity="1" stroke-width="1" stroke-linecap="square" stroke-linejoin="bevel" transform="matrix(1,0,0,1,1438,1390)" font-family="MS Shell Dlg 2" font-size="9.75" font-weight="400" font-style="normal" > <path vector-effect="none" fill-rule="evenodd" d="M0,5 C0,2.23858 2.23858,0 5,0 L45,0 C47.7614,0 50,2.23858 50,5 L50,15 C50,17.7614 47.7614,20 45,20 L5,20 C2.23858,20 0,17.7614 0,15 L0,5"/> </g> <g fill="none" stroke="#000000" stroke-opacity="1" stroke-width="1" stroke-linecap="square" stroke-linejoin="bevel" transform="matrix(1,0,0,1,1446.5,1388)" font-family="MS Shell Dlg 2" font-size="9.75" font-weight="400" font-style="normal" > <text fill="#000000" fill-opacity="1" stroke="none" xml:space="preserve" x="4" y="17" font-family="Arial" font-size="10pt" font-weight="700" font-style="italic" >rrnS</text> </g> <g fill="#ff9999" fill-opacity="1" stroke="#bfbfbf" stroke-opacity="1" stroke-width="1" stroke-linecap="square" stroke-linejoin="bevel" transform="matrix(1,0,0,1,1489,1390)" font-family="MS Shell Dlg 2" font-size="9.75" font-weight="400" font-style="normal" > <path vector-effect="none" fill-rule="evenodd" d="M0,1.7 L42.5,1.7 L42.5,0 L50,10 L42.5,20 L42.5,18.3 L0,18.3 L0,1.7"/> </g> <g fill="none" stroke="#000000" stroke-opacity="1" stroke-width="1" stroke-linecap="square" stroke-linejoin="bevel" transform="matrix(1,0,0,1,1504.13,1388)" font-family="MS Shell Dlg 2" font-size="9.75" font-weight="400" font-style="normal" > <text fill="#000000" fill-opacity="1" stroke="none" xml:space="preserve" x="4" y="17" font-family="Arial" font-size="10pt" font-weight="700" font-style="italic" >I</text> </g> <g fill="#ff9999" fill-opacity="1" stroke="#bfbfbf" stroke-opacity="1" stroke-width="1" stroke-linecap="square" stroke-linejoin="bevel" transform="matrix(1,0,0,1,1540,1390)" font-family="MS Shell Dlg 2" font-size="9.75" font-

weight="400" font-style="normal" > <path vector-effect="none" fill-rule="evenodd" d="M0,1.7 L42.5,1.7 L42.5,0 L50,10 L42.5,20 L42.5,18.3 L0,18.3 L0,1.7"/> </g> <g fill="none" stroke="#000000" stroke-opacity="1" stroke-width="1" stroke-linecap="square" stroke-linejoin="bevel" transform="matrix(1,0,0,1,1553.13,1388)" font-family="MS Shell Dlg 2" font-size="9.75" font-weight="400" font-style="normal" > <text fill="#000000" fill-opacity="1" stroke="none" xml:space="preserve" x="4" y="17" font-family="Arial" font-size="10pt" font-weight="700" font-style="italic" >G</text> </g> <g fill="#ff9999" fill-opacity="1" stroke="#bfbfbf" stroke-opacity="1" stroke-width="1" stroke-linecap="square" stroke-linejoin="bevel" transform="matrix(1,0,0,1,1591,1390)" font-family="MS Shell Dlg 2" font-size="9.75" font-weight="400" font-style="normal" > <path vector-effect="none" fill-rule="evenodd" d="M0,1.7 L42.5,1.7 L42.5,0 L50,10 L42.5,20 L42.5,18.3 L0,18.3 L0,1.7"/> </g> <g fill="none" stroke="#000000" stroke-opacity="1" stroke-width="1" stroke-linecap="square" stroke-linejoin="bevel" transform="matrix(1,0,0,1,1604.13,1388)" font-family="MS Shell Dlg 2" font-size="9.75" font-weight="400" font-style="normal" > <text fill="#000000" fill-opacity="1" stroke="none" xml:space="preserve" x="4" y="17" font-family="Arial" font-size="10pt" font-weight="700" font-style="italic" >V</text> </g> <g fill="#99ffff" fill-opacity="1" stroke="#bfbfbf" stroke-opacity="1" stroke-width="1" stroke-linecap="square" stroke-linejoin="bevel" transform="matrix(1,0,0,1,1642,1390)" font-family="MS Shell Dlg 2" font-size="9.75" font-weight="400" font-style="normal" > <path vector-effect="none" fill-rule="evenodd" d="M0,5 C0,2.23858 2.23858,0 5,0 L45,0 C47.7614,0 50,2.23858 50,5 L50,15 C50,17.7614 47.7614,20 45,20 L5,20 C2.23858,20 0,17.7614 0,15 L0,5"/> </g> <g fill="none" stroke="#000000" stroke-opacity="1" stroke-width="1" stroke-linecap="square" stroke-linejoin="bevel" transform="matrix(1,0,0,1,1651.5,1388)" font-family="MS Shell Dlg 2" font-size="9.75" font-weight="400" font-style="normal" > <text fill="#000000" fill-opacity="1" stroke="none" xml:space="preserve" x="4" y="17" font-family="Arial" font-size="10pt" font-weight="700" font-style="italic" >rrnL</text> </g> <g fill="#ff9999" fill-opacity="1" stroke="#bfbfbf" stroke-opacity="1" stroke-width="1" stroke-linecap="square" stroke-linejoin="bevel" transform="matrix(1,0,0,1,1693,1390)" font-family="MS Shell Dlg 2" font-size="9.75" font-weight="400" font-style="normal" > <path vector-effect="none" fill-rule="evenodd" d="M0,1.7 L42.5,1.7 L42.5,0 L50,10 L42.5,20 L42.5,18.3 L0,18.3 L0,1.7"/> </g> <g fill="none" stroke="#000000" stroke-opacity="1" stroke-width="1" stroke-linecap="square" stroke-linejoin="bevel" transform="matrix(1,0,0,1,1705.13,1388)" font-family="MS Shell Dlg 2" font-size="9.75" font-weight="400" font-style="normal" > <text fill="#000000" fill-opacity="1" stroke="none" xml:space="preserve" x="4" y="17" font-family="Arial" font-size="10pt" font-weight="700" font-style="italic" >M</text> </g> <g fill="#ff9999" fill-opacity="1" stroke="#bfbfbf" stroke-opacity="1" stroke-width="1" stroke-linecap="square" stroke-linejoin="bevel" transform="matrix(1,0,0,1,1744,1390)" font-family="MS Shell Dlg 2" font-size="9.75" font-weight="400" font-style="normal" > <path vector-effect="none" fill-rule="evenodd" d="M0,1.7 L42.5,1.7 L42.5,0 L50,10 L42.5,20 L42.5,18.3 L0,18.3 L0,1.7"/> </g> <g fill="none" stroke="#000000" stroke-opacity="1" stroke-width="1" stroke-linecap="square" stroke-linejoin="bevel" transform="matrix(1,0,0,1,1757.63,1388)" font-family="MS Shell Dlg 2" font-size="9.75" font-weight="400" font-style="normal" > <text fill="#000000" fill-opacity="1" stroke="none" xml:space="preserve" x="4" y="17" font-family="Arial" font-size="10pt" font-weight="700" font-style="italic" >F</text> </g> <g fill="#ff9999" fill-opacity="1" stroke="#bfbfbf" stroke-opacity="1" stroke-width="1" stroke-linecap="square" stroke-linejoin="bevel" transform="matrix(1,0,0,1,1795,1390)" font-family="MS Shell Dlg 2" font-size="9.75" font-weight="400" font-style="normal" > <path vector-effect="none" fill-rule="evenodd" d="M0,1.7 L42.5,1.7 L42.5,0 L50,10 L42.5,20 L42.5,18.3 L0,18.3 L0,1.7"/> </g> <g fill="none" stroke="#000000" stroke-opacity="1" stroke-width="1" stroke-linecap="square" stroke-linejoin="bevel" transform="matrix(1,0,0,1,1808.13,1388)" font-family="MS Shell Dlg 2" font-size="9.75" font-weight="400" font-style="normal" > <text fill="#000000" fill-opacity="1" stroke="none" xml:space="preserve" x="4" y="17" font-family="Arial" font-size="10pt" font-weight="700" font-style="italic" >H</text> </g> <g fill="#ffff33" fill-opacity="1" stroke="#bfbfbf" stroke-opacity="1" stroke-width="1" stroke-linecap="square" stroke-linejoin="bevel" transform="matrix(1,0,0,1,1846,1390)" font-family="MS Shell Dlg 2" font-size="9.75" font-weight="400" font-style="normal" > <rect x="0" y="0" width="50" height="20"/> </g> <g fill="none" stroke="#000000" stroke-opacity="1" stroke-width="1" stroke-linecap="square" stroke-linejoin="bevel" transform="matrix(1,0,0,1,1854,1388)" font-family="MS Shell Dlg 2" font-size="9.75" font-weight="400" font-style="normal" > <text fill="#000000" fill-opacity="1" stroke="none" xml:space="preserve" x="4" y="17" font-family="Arial" font-size="10pt" font-weight="700" font-style="italic" >cox3</text> </g> <g fill="#ffff33" fill-opacity="1" stroke="#bfbfbf" stroke-opacity="1" stroke-width="1" stroke-linecap="square" stroke-linejoin="bevel" transform="matrix(1,0,0,1,1897,1390)" font-family="MS Shell Dlg 2" font-size="9.75" font-weight="400" font-style="normal" > <rect x="0" y="0" width="50" height="20"/> </g> <g fill="none" stroke="#000000" stroke-opacity="1" stroke-width="1" stroke-linecap="square" stroke-linejoin="bevel" transform="matrix(1,0,0,1,1905.5,1388)" font-family="MS Shell Dlg 2" font-size="9.75" font-weight="400" font-style="normal" > <text fill="#000000" fill-opacity="1" stroke="none" xml:space="preserve"

x="4" y="17" font-family="Arial" font-size="10pt" font-weight="700" font-style="italic" >atp8</text> </g>
<g fill="none" stroke="#000000" stroke-opacity="1" stroke-width="1" stroke-linecap="square" stroke-linejoin="bevel" transform="matrix(1,0,0,1,1948,1390)" font-family="MS Shell Dlg 2" font-size="9.75" font-weight="400" font-style="normal" > <text fill="#000000" fill-opacity="1" stroke="none" xml:space="preserve" x="4" y="17" font-family="Arial" font-size="10pt" font-weight="700" font-style="italic" >(+)</text> </g>
<g fill="none" stroke="#000000" stroke-opacity="1" stroke-width="1" stroke-linecap="square" stroke-linejoin="bevel" transform="matrix(1,0,0,1,10,1430)" font-family="MS Shell Dlg 2" font-size="9.75" font-weight="400" font-style="normal" > <text fill="#000000" fill-opacity="1" stroke="none" xml:space="preserve" x="4" y="17" font-family="Arial" font-size="10pt" font-weight="700" font-style="italic" >Bathymodiolus_childressi_NC_059707</text> </g> <g fill="#ffff33" fill-opacity="1" stroke="#bfbfbf" stroke-opacity="1" stroke-width="1" stroke-linecap="square" stroke-linejoin="bevel" transform="matrix(1,0,0,1,10,1460)" font-family="MS Shell Dlg 2" font-size="9.75" font-weight="400" font-style="normal" > <rect x="0" y="0" width="50" height="20"/> </g> <g fill="none" stroke="#000000" stroke-opacity="1" stroke-width="1" stroke-linecap="square" stroke-linejoin="bevel" transform="matrix(1,0,0,1,18,1458)" font-family="MS Shell Dlg 2" font-size="9.75" font-weight="400" font-style="normal" > <text fill="#000000" fill-opacity="1" stroke="none" xml:space="preserve" x="4" y="17" font-family="Arial" font-size="10pt" font-weight="700" font-style="italic" >cox1</text> </g> <g fill="#ff9999" fill-opacity="1" stroke="#bfbfbf" stroke-opacity="1" stroke-width="1" stroke-linecap="square" stroke-linejoin="bevel" transform="matrix(1,0,0,1,61,1460)" font-family="MS Shell Dlg 2" font-size="9.75" font-weight="400" font-style="normal" > <path vector-effect="none" fill-rule="evenodd" d="M0,1.7 L42.5,1.7 L42.5,0 L50,10 L42.5,20 L42.5,18.3 L0,18.3 L0,1.7"/> </g> <g fill="none" stroke="#000000" stroke-opacity="1" stroke-width="1" stroke-linecap="square" stroke-linejoin="bevel" transform="matrix(1,0,0,1,74.6325,1458)" font-family="MS Shell Dlg 2" font-size="9.75" font-weight="400" font-style="normal" > <text fill="#000000" fill-opacity="1" stroke="none" xml:space="preserve" x="4" y="17" font-family="Arial" font-size="10pt" font-weight="700" font-style="italic" >P</text> </g> <g fill="#ff9999" fill-opacity="1" stroke="#bfbfbf" stroke-opacity="1" stroke-width="1" stroke-linecap="square" stroke-linejoin="bevel" transform="matrix(1,0,0,1,112,1460)" font-family="MS Shell Dlg 2" font-size="9.75" font-weight="400" font-style="normal" > <path vector-effect="none" fill-rule="evenodd" d="M0,1.7 L42.5,1.7 L42.5,0 L50,10 L42.5,20 L42.5,18.3 L0,18.3 L0,1.7"/> </g> <g fill="none" stroke="#000000" stroke-opacity="1" stroke-width="1" stroke-linecap="square" stroke-linejoin="bevel" transform="matrix(1,0,0,1,125.632,1458)" font-family="MS Shell Dlg 2" font-size="9.75" font-weight="400" font-style="normal" > <text fill="#000000" fill-opacity="1" stroke="none" xml:space="preserve" x="4" y="17" font-family="Arial" font-size="10pt" font-weight="700" font-style="italic" >E</text> </g> <g fill="#ff9999" fill-opacity="1" stroke="#bfbfbf" stroke-opacity="1" stroke-width="1" stroke-linecap="square" stroke-linejoin="bevel" transform="matrix(1,0,0,1,163,1460)" font-family="MS Shell Dlg 2" font-size="9.75" font-weight="400" font-style="normal" > <path vector-effect="none" fill-rule="evenodd" d="M0,1.7 L42.5,1.7 L42.5,0 L50,10 L42.5,20 L42.5,18.3 L0,18.3 L0,1.7"/> </g> <g fill="none" stroke="#000000" stroke-opacity="1" stroke-width="1" stroke-linecap="square" stroke-linejoin="bevel" transform="matrix(1,0,0,1,176.132,1458)" font-family="MS Shell Dlg 2" font-size="9.75" font-weight="400" font-style="normal" > <text fill="#000000" fill-opacity="1" stroke="none" xml:space="preserve" x="4" y="17" font-family="Arial" font-size="10pt" font-weight="700" font-style="italic" >C</text> </g> <g fill="#ffff33" fill-opacity="1" stroke="#bfbfbf" stroke-opacity="1" stroke-width="1" stroke-linecap="square" stroke-linejoin="bevel" transform="matrix(1,0,0,1,214,1460)" font-family="MS Shell Dlg 2" font-size="9.75" font-weight="400" font-style="normal" > <rect x="0" y="0" width="50" height="20"/> </g> <g fill="none" stroke="#000000" stroke-opacity="1" stroke-width="1" stroke-linecap="square" stroke-linejoin="bevel" transform="matrix(1,0,0,1,221,1458)" font-family="MS Shell Dlg 2" font-size="9.75" font-weight="400" font-style="normal" > <text fill="#000000" fill-opacity="1" stroke="none" xml:space="preserve" x="4" y="17" font-family="Arial" font-size="10pt" font-weight="700" font-style="italic" >nad3</text> </g> <g fill="#ff9999" fill-opacity="1" stroke="#bfbfbf" stroke-opacity="1" stroke-width="1" stroke-linecap="square" stroke-linejoin="bevel" transform="matrix(1,0,0,1,265,1460)" font-family="MS Shell Dlg 2" font-size="9.75" font-weight="400" font-style="normal" > <path vector-effect="none" fill-rule="evenodd" d="M0,1.7 L42.5,1.7 L42.5,0 L50,10 L42.5,20 L42.5,18.3 L0,18.3 L0,1.7"/> </g> <g fill="none" stroke="#000000" stroke-opacity="1" stroke-width="1" stroke-linecap="square" stroke-linejoin="bevel" transform="matrix(1,0,0,1,279.132,1458)" font-family="MS Shell Dlg 2" font-size="9.75" font-weight="400" font-style="normal" > <text fill="#000000" fill-opacity="1" stroke="none" xml:space="preserve" x="4" y="17" font-family="Arial" font-size="10pt" font-weight="700" font-style="italic" >L</text> </g> <g fill="#ff9999" fill-opacity="1" stroke="#bfbfbf" stroke-opacity="1" stroke-width="1" stroke-linecap="square" stroke-linejoin="bevel" transform="matrix(1,0,0,1,316,1460)" font-family="MS Shell Dlg 2" font-size="9.75" font-weight="400" font-style="normal" > <path vector-effect="none" fill-rule="evenodd" d="M0,1.7 L42.5,1.7 L42.5,0 L50,10 L42.5,20

L42.5,18.3 L0,18.3 L0,1.7"/> </g> <g fill="none" stroke="#000000" stroke-opacity="1" stroke-width="1" stroke-linecap="square" stroke-linejoin="bevel" transform="matrix(1,0,0,1,329.132,1458)" font-family="MS Shell Dlg 2" font-size="9.75" font-weight="400" font-style="normal" > <text fill="#000000" fill-opacity="1" stroke="none" xml:space="preserve" x="4" y="17" font-family="Arial" font-size="10pt" font-weight="700" font-style="italic" >S</text> </g> <g fill="#ffff33" fill-opacity="1" stroke="#bfbfbf" stroke-opacity="1" stroke-width="1" stroke-linecap="square" stroke-linejoin="bevel" transform="matrix(1,0,0,1,367,1460)" font-family="MS Shell Dlg 2" font-size="9.75" font-weight="400" font-style="normal" > <rect x="0" y="0" width="50" height="20"/> </g> <g fill="none" stroke="#000000" stroke-opacity="1" stroke-width="1" stroke-linecap="square" stroke-linejoin="bevel" transform="matrix(1,0,0,1,375.5,1458)" font-family="MS Shell Dlg 2" font-size="9.75" font-weight="400" font-style="normal" > <text fill="#000000" fill-opacity="1" stroke="none" xml:space="preserve" x="4" y="17" font-family="Arial" font-size="10pt" font-weight="700" font-style="italic" >atp8</text> </g> <g fill="#ff9999" fill-opacity="1" stroke="#bfbfbf" stroke-opacity="1" stroke-width="1" stroke-linecap="square" stroke-linejoin="bevel" transform="matrix(1,0,0,1,418,1460)" font-family="MS Shell Dlg 2" font-size="9.75" font-weight="400" font-style="normal" > <path vector-effect="none" fill-rule="evenodd" d="M0,1.7 L42.5,1.7 L42.5,0 L50,10 L42.5,20 L42.5,18.3 L0,18.3 L0,1.7"/> </g> <g fill="none" stroke="#000000" stroke-opacity="1" stroke-width="1" stroke-linecap="square" stroke-linejoin="bevel" transform="matrix(1,0,0,1,432.132,1458)" font-family="MS Shell Dlg 2" font-size="9.75" font-weight="400" font-style="normal" > <text fill="#000000" fill-opacity="1" stroke="none" xml:space="preserve" x="4" y="17" font-family="Arial" font-size="10pt" font-weight="700" font-style="italic" >L</text> </g> <g fill="#ff9999" fill-opacity="1" stroke="#bfbfbf" stroke-opacity="1" stroke-width="1" stroke-linecap="square" stroke-linejoin="bevel" transform="matrix(1,0,0,1,469,1460)" font-family="MS Shell Dlg 2" font-size="9.75" font-weight="400" font-style="normal" > <path vector-effect="none" fill-rule="evenodd" d="M0,1.7 L42.5,1.7 L42.5,0 L50,10 L42.5,20 L42.5,18.3 L0,18.3 L0,1.7"/> </g> <g fill="none" stroke="#000000" stroke-opacity="1" stroke-width="1" stroke-linecap="square" stroke-linejoin="bevel" transform="matrix(1,0,0,1,482.632,1458)" font-family="MS Shell Dlg 2" font-size="9.75" font-weight="400" font-style="normal" > <text fill="#000000" fill-opacity="1" stroke="none" xml:space="preserve" x="4" y="17" font-family="Arial" font-size="10pt" font-weight="700" font-style="italic" >K</text> </g> <g fill="#99ffff" fill-opacity="1" stroke="#bfbfbf" stroke-opacity="1" stroke-width="1" stroke-linecap="square" stroke-linejoin="bevel" transform="matrix(1,0,0,1,520,1460)" font-family="MS Shell Dlg 2" font-size="9.75" font-weight="400" font-style="normal" > <path vector-effect="none" fill-rule="evenodd" d="M0,5 C0,2.23858 2.23858,0 5,0 L45,0 C47.7614,0 50,2.23858 50,5 L50,15 C50,17.7614 47.7614,20 45,20 L5,20 C2.23858,20 0,17.7614 0,15 L0,5"/> </g> <g fill="none" stroke="#000000" stroke-opacity="1" stroke-width="1" stroke-linecap="square" stroke-linejoin="bevel" transform="matrix(1,0,0,1,528.5,1458)" font-family="MS Shell Dlg 2" font-size="9.75" font-weight="400" font-style="normal" > <text fill="#000000" fill-opacity="1" stroke="none" xml:space="preserve" x="4" y="17" font-family="Arial" font-size="10pt" font-weight="700" font-style="italic" >rrnS</text> </g> <g fill="#ff9999" fill-opacity="1" stroke="#bfbfbf" stroke-opacity="1" stroke-width="1" stroke-linecap="square" stroke-linejoin="bevel" transform="matrix(1,0,0,1,571,1460)" font-family="MS Shell Dlg 2" font-size="9.75" font-weight="400" font-style="normal" > <path vector-effect="none" fill-rule="evenodd" d="M0,1.7 L42.5,1.7 L42.5,0 L50,10 L42.5,20 L42.5,18.3 L0,18.3 L0,1.7"/> </g> <g fill="none" stroke="#000000" stroke-opacity="1" stroke-width="1" stroke-linecap="square" stroke-linejoin="bevel" transform="matrix(1,0,0,1,583.633,1458)" font-family="MS Shell Dlg 2" font-size="9.75" font-weight="400" font-style="normal" > <text fill="#000000" fill-opacity="1" stroke="none" xml:space="preserve" x="4" y="17" font-family="Arial" font-size="10pt" font-weight="700" font-style="italic" >Q</text> </g> <g fill="#ff9999" fill-opacity="1" stroke="#bfbfbf" stroke-opacity="1" stroke-width="1" stroke-linecap="square" stroke-linejoin="bevel" transform="matrix(1,0,0,1,622,1460)" font-family="MS Shell Dlg 2" font-size="9.75" font-weight="400" font-style="normal" > <path vector-effect="none" fill-rule="evenodd" d="M0,1.7 L42.5,1.7 L42.5,0 L50,10 L42.5,20 L42.5,18.3 L0,18.3 L0,1.7"/> </g> <g fill="none" stroke="#000000" stroke-opacity="1" stroke-width="1" stroke-linecap="square" stroke-linejoin="bevel" transform="matrix(1,0,0,1,635.633,1458)" font-family="MS Shell Dlg 2" font-size="9.75" font-weight="400" font-style="normal" > <text fill="#000000" fill-opacity="1" stroke="none" xml:space="preserve" x="4" y="17" font-family="Arial" font-size="10pt" font-weight="700" font-style="italic" >Y</text> </g> <g fill="#ffff33" fill-opacity="1" stroke="#bfbfbf" stroke-opacity="1" stroke-width="1" stroke-linecap="square" stroke-linejoin="bevel" transform="matrix(1,0,0,1,673,1460)" font-family="MS Shell Dlg 2" font-size="9.75" font-weight="400" font-style="normal" > <rect x="0" y="0" width="50" height="20"/> </g> <g fill="none" stroke="#000000" stroke-opacity="1" stroke-width="1" stroke-linecap="square" stroke-linejoin="bevel" transform="matrix(1,0,0,1,681.5,1458)" font-family="MS Shell Dlg 2" font-size="9.75" font-weight="400" font-style="normal" > <text fill="#000000" fill-opacity="1" stroke="none" xml:space="preserve" x="4" y="17" font-family="Arial" font-size="10pt" font-weight="700" font-style="italic" >atp6</text> </g> <g fill="#ff9999" fill-opacity="1" stroke="#bfbfbf" stroke-opacity="1" stroke-width="1"

stroke-linecap="square" stroke-linejoin="bevel" transform="matrix(1,0,0,1,724,1460)" font-family="MS Shell Dlg 2" font-size="9.75" font-weight="400" font-style="normal" > <path vector-effect="none" fill-rule="evenodd" d="M0,1.7 L42.5,1.7 L42.5,0 L50,10 L42.5,20 L42.5,18.3 L0,18.3 L0,1.7"/> </g> <g fill="none" stroke="#000000" stroke-opacity="1" stroke-width="1" stroke-linecap="square" stroke-linejoin="bevel" transform="matrix(1,0,0,1,737.133,1458)" font-family="MS Shell Dlg 2" font-size="9.75" font-weight="400" font-style="normal" > <text fill="#000000" fill-opacity="1" stroke="none" xml:space="preserve" x="4" y="17" font-family="Arial" font-size="10pt" font-weight="700" font-style="italic" >R</text> </g> <g fill="#ff9999" fill-opacity="1" stroke="#bfbfbf" stroke-opacity="1" stroke-width="1" stroke-linecap="square" stroke-linejoin="bevel" transform="matrix(1,0,0,1,775,1460)" font-family="MS Shell Dlg 2" font-size="9.75" font-weight="400" font-style="normal" > <path vector-effect="none" fill-rule="evenodd" d="M0,1.7 L42.5,1.7 L42.5,0 L50,10 L42.5,20 L42.5,18.3 L0,18.3 L0,1.7"/> </g> <g fill="none" stroke="#000000" stroke-opacity="1" stroke-width="1" stroke-linecap="square" stroke-linejoin="bevel" transform="matrix(1,0,0,1,787.133,1458)" font-family="MS Shell Dlg 2" font-size="9.75" font-weight="400" font-style="normal" > <text fill="#000000" fill-opacity="1" stroke="none" xml:space="preserve" x="4" y="17" font-family="Arial" font-size="10pt" font-weight="700" font-style="italic" >M</text> </g> <g fill="#ff9999" fill-opacity="1" stroke="#bfbfbf" stroke-opacity="1" stroke-width="1" stroke-linecap="square" stroke-linejoin="bevel" transform="matrix(1,0,0,1,826,1460)" font-family="MS Shell Dlg 2" font-size="9.75" font-weight="400" font-style="normal" > <path vector-effect="none" fill-rule="evenodd" d="M0,1.7 L42.5,1.7 L42.5,0 L50,10 L42.5,20 L42.5,18.3 L0,18.3 L0,1.7"/> </g> <g fill="none" stroke="#000000" stroke-opacity="1" stroke-width="1" stroke-linecap="square" stroke-linejoin="bevel" transform="matrix(1,0,0,1,839.133,1458)" font-family="MS Shell Dlg 2" font-size="9.75" font-weight="400" font-style="normal" > <text fill="#000000" fill-opacity="1" stroke="none" xml:space="preserve" x="4" y="17" font-family="Arial" font-size="10pt" font-weight="700" font-style="italic" >V</text> </g> <g fill="#ffff33" fill-opacity="1" stroke="#bfbfbf" stroke-opacity="1" stroke-width="1" stroke-linecap="square" stroke-linejoin="bevel" transform="matrix(1,0,0,1,877,1460)" font-family="MS Shell Dlg 2" font-size="9.75" font-weight="400" font-style="normal" > <rect x="0" y="0" width="50" height="20"/> </g> <g fill="none" stroke="#000000" stroke-opacity="1" stroke-width="1" stroke-linecap="square" stroke-linejoin="bevel" transform="matrix(1,0,0,1,884,1458)" font-family="MS Shell Dlg 2" font-size="9.75" font-weight="400" font-style="normal" > <text fill="#000000" fill-opacity="1" stroke="none" xml:space="preserve" x="4" y="17" font-family="Arial" font-size="10pt" font-weight="700" font-style="italic" >nad4</text> </g> <g fill="#ff9999" fill-opacity="1" stroke="#bfbfbf" stroke-opacity="1" stroke-width="1" stroke-linecap="square" stroke-linejoin="bevel" transform="matrix(1,0,0,1,928,1460)" font-family="MS Shell Dlg 2" font-size="9.75" font-weight="400" font-style="normal" > <path vector-effect="none" fill-rule="evenodd" d="M0,1.7 L42.5,1.7 L42.5,0 L50,10 L42.5,20 L42.5,18.3 L0,18.3 L0,1.7"/> </g> <g fill="none" stroke="#000000" stroke-opacity="1" stroke-width="1" stroke-linecap="square" stroke-linejoin="bevel" transform="matrix(1,0,0,1,941.133,1458)" font-family="MS Shell Dlg 2" font-size="9.75" font-weight="400" font-style="normal" > <text fill="#000000" fill-opacity="1" stroke="none" xml:space="preserve" x="4" y="17" font-family="Arial" font-size="10pt" font-weight="700" font-style="italic" >N</text> </g> <g fill="#ffff33" fill-opacity="1" stroke="#bfbfbf" stroke-opacity="1" stroke-width="1" stroke-linecap="square" stroke-linejoin="bevel" transform="matrix(1,0,0,1,979,1460)" font-family="MS Shell Dlg 2" font-size="9.75" font-weight="400" font-style="normal" > <rect x="0" y="0" width="50" height="20"/> </g> <g fill="none" stroke="#000000" stroke-opacity="1" stroke-width="1" stroke-linecap="square" stroke-linejoin="bevel" transform="matrix(1,0,0,1,987,1458)" font-family="MS Shell Dlg 2" font-size="9.75" font-weight="400" font-style="normal" > <text fill="#000000" fill-opacity="1" stroke="none" xml:space="preserve" x="4" y="17" font-family="Arial" font-size="10pt" font-weight="700" font-style="italic" >cox3</text> </g> <g fill="#ff9999" fill-opacity="1" stroke="#bfbfbf" stroke-opacity="1" stroke-width="1" stroke-linecap="square" stroke-linejoin="bevel" transform="matrix(1,0,0,1,1030,1460)" font-family="MS Shell Dlg 2" font-size="9.75" font-weight="400" font-style="normal" > <path vector-effect="none" fill-rule="evenodd" d="M0,1.7 L42.5,1.7 L42.5,0 L50,10 L42.5,20 L42.5,18.3 L0,18.3 L0,1.7"/> </g> <g fill="none" stroke="#000000" stroke-opacity="1" stroke-width="1" stroke-linecap="square" stroke-linejoin="bevel" transform="matrix(1,0,0,1,1043.63,1458)" font-family="MS Shell Dlg 2" font-size="9.75" font-weight="400" font-style="normal" > <text fill="#000000" fill-opacity="1" stroke="none" xml:space="preserve" x="4" y="17" font-family="Arial" font-size="10pt" font-weight="700" font-style="italic" >F</text> </g> <g fill="#ff9999" fill-opacity="1" stroke="#bfbfbf" stroke-opacity="1" stroke-width="1" stroke-linecap="square" stroke-linejoin="bevel" transform="matrix(1,0,0,1,1081,1460)" font-family="MS Shell Dlg 2" font-size="9.75" font-weight="400" font-style="normal" > <path vector-effect="none" fill-rule="evenodd" d="M0,1.7 L42.5,1.7 L42.5,0 L50,10 L42.5,20 L42.5,18.3 L0,18.3 L0,1.7"/> </g> <g fill="none" stroke="#000000" stroke-opacity="1" stroke-width="1" stroke-linecap="square" stroke-linejoin="bevel" transform="matrix(1,0,0,1,1094.13,1458)" font-family="MS Shell Dlg 2" font-size="9.75" font-weight="400" font-style="normal" > <text fill="#000000" fill-

opacity="1" stroke="none" xml:space="preserve" x="4" y="17" font-family="Arial" font-size="10pt" font-weight="700" font-style="italic" >S</text> </g> <g fill="#99ffff" fill-opacity="1" stroke="#bfbfbf" stroke-opacity="1" stroke-width="1" stroke-linecap="square" stroke-linejoin="bevel" transform="matrix(1,0,0,1,1132,1460)" font-family="MS Shell Dlg 2" font-size="9.75" font-weight="400" font-style="normal" > <path vector-effect="none" fill-rule="evenodd" d="M0,5 C0,2.23858 2.23858,0 5,0 L45,0 C47.7614,0 50,2.23858 50,5 L50,15 C50,17.7614 47.7614,20 45,20 L5,20 C2.23858,20 0,17.7614 0,15 L0,5"/> </g> <g fill="none" stroke="#000000" stroke-opacity="1" stroke-width="1" stroke-linecap="square" stroke-linejoin="bevel" transform="matrix(1,0,0,1,1141.5,1458)" font-family="MS Shell Dlg 2" font-size="9.75" font-weight="400" font-style="normal" > <text fill="#000000" fill-opacity="1" stroke="none" xml:space="preserve" x="4" y="17" font-family="Arial" font-size="10pt" font-weight="700" font-style="italic" >rrnL</text> </g> <g fill="#ff9999" fill-opacity="1" stroke="#bfbfbf" stroke-opacity="1" stroke-width="1" stroke-linecap="square" stroke-linejoin="bevel" transform="matrix(1,0,0,1,1183,1460)" font-family="MS Shell Dlg 2" font-size="9.75" font-weight="400" font-style="normal" > <path vector-effect="none" fill-rule="evenodd" d="M0,1.7 L42.5,1.7 L42.5,0 L50,10 L42.5,20 L42.5,18.3 L0,18.3 L0,1.7"/> </g> <g fill="none" stroke="#000000" stroke-opacity="1" stroke-width="1" stroke-linecap="square" stroke-linejoin="bevel" transform="matrix(1,0,0,1,1196.13,1458)" font-family="MS Shell Dlg 2" font-size="9.75" font-weight="400" font-style="normal" > <text fill="#000000" fill-opacity="1" stroke="none" xml:space="preserve" x="4" y="17" font-family="Arial" font-size="10pt" font-weight="700" font-style="italic" >S</text> </g> <g fill="#ff9999" fill-opacity="1" stroke="#bfbfbf" stroke-opacity="1" stroke-width="1" stroke-linecap="square" stroke-linejoin="bevel" transform="matrix(1,0,0,1,1234,1460)" font-family="MS Shell Dlg 2" font-size="9.75" font-weight="400" font-style="normal" > <path vector-effect="none" fill-rule="evenodd" d="M0,1.7 L42.5,1.7 L42.5,0 L50,10 L42.5,20 L42.5,18.3 L0,18.3 L0,1.7"/> </g> <g fill="none" stroke="#000000" stroke-opacity="1" stroke-width="1" stroke-linecap="square" stroke-linejoin="bevel" transform="matrix(1,0,0,1,1247.13,1458)" font-family="MS Shell Dlg 2" font-size="9.75" font-weight="400" font-style="normal" > <text fill="#000000" fill-opacity="1" stroke="none" xml:space="preserve" x="4" y="17" font-family="Arial" font-size="10pt" font-weight="700" font-style="italic" >D</text> </g> <g fill="#ffff33" fill-opacity="1" stroke="#bfbfbf" stroke-opacity="1" stroke-width="1" stroke-linecap="square" stroke-linejoin="bevel" transform="matrix(1,0,0,1,1285,1460)" font-family="MS Shell Dlg 2" font-size="9.75" font-weight="400" font-style="normal" > <rect x="0" y="0" width="50" height="20"/> </g> <g fill="none" stroke="#000000" stroke-opacity="1" stroke-width="1" stroke-linecap="square" stroke-linejoin="bevel" transform="matrix(1,0,0,1,1292,1458)" font-family="MS Shell Dlg 2" font-size="9.75" font-weight="400" font-style="normal" > <text fill="#000000" fill-opacity="1" stroke="none" xml:space="preserve" x="4" y="17" font-family="Arial" font-size="10pt" font-weight="700" font-style="italic" >nad6</text> </g> <g fill="#ff9999" fill-opacity="1" stroke="#bfbfbf" stroke-opacity="1" stroke-width="1" stroke-linecap="square" stroke-linejoin="bevel" transform="matrix(1,0,0,1,1336,1460)" font-family="MS Shell Dlg 2" font-size="9.75" font-weight="400" font-style="normal" > <path vector-effect="none" fill-rule="evenodd" d="M0,1.7 L42.5,1.7 L42.5,0 L50,10 L42.5,20 L42.5,18.3 L0,18.3 L0,1.7"/> </g> <g fill="none" stroke="#000000" stroke-opacity="1" stroke-width="1" stroke-linecap="square" stroke-linejoin="bevel" transform="matrix(1,0,0,1,1351.13,1458)" font-family="MS Shell Dlg 2" font-size="9.75" font-weight="400" font-style="normal" > <text fill="#000000" fill-opacity="1" stroke="none" xml:space="preserve" x="4" y="17" font-family="Arial" font-size="10pt" font-weight="700" font-style="italic" >I</text> </g> <g fill="#ffff33" fill-opacity="1" stroke="#bfbfbf" stroke-opacity="1" stroke-width="1" stroke-linecap="square" stroke-linejoin="bevel" transform="matrix(1,0,0,1,1387,1460)" font-family="MS Shell Dlg 2" font-size="9.75" font-weight="400" font-style="normal" > <rect x="0" y="0" width="50" height="20"/> </g> <g fill="none" stroke="#000000" stroke-opacity="1" stroke-width="1" stroke-linecap="square" stroke-linejoin="bevel" transform="matrix(1,0,0,1,1394,1458)" font-family="MS Shell Dlg 2" font-size="9.75" font-weight="400" font-style="normal" > <text fill="#000000" fill-opacity="1" stroke="none" xml:space="preserve" x="4" y="17" font-family="Arial" font-size="10pt" font-weight="700" font-style="italic" >nad2</text> </g> <g fill="#ff9999" fill-opacity="1" stroke="#bfbfbf" stroke-opacity="1" stroke-width="1" stroke-linecap="square" stroke-linejoin="bevel" transform="matrix(1,0,0,1,1438,1460)" font-family="MS Shell Dlg 2" font-size="9.75" font-weight="400" font-style="normal" > <path vector-effect="none" fill-rule="evenodd" d="M0,1.7 L42.5,1.7 L42.5,0 L50,10 L42.5,20 L42.5,18.3 L0,18.3 L0,1.7"/> </g> <g fill="none" stroke="#000000" stroke-opacity="1" stroke-width="1" stroke-linecap="square" stroke-linejoin="bevel" transform="matrix(1,0,0,1,1449.13,1458)" font-family="MS Shell Dlg 2" font-size="9.75" font-weight="400" font-style="normal" > <text fill="#000000" fill-opacity="1" stroke="none" xml:space="preserve" x="4" y="17" font-family="Arial" font-size="10pt" font-weight="700" font-style="italic" >W</text> </g> <g fill="#ff9999" fill-opacity="1" stroke="#bfbfbf" stroke-opacity="1" stroke-width="1" stroke-linecap="square" stroke-linejoin="bevel" transform="matrix(1,0,0,1,1489,1460)" font-family="MS Shell Dlg 2" font-size="9.75" font-

weight="400" font-style="normal" > <path vector-effect="none" fill-rule="evenodd" d="M0,1.7 L42.5,1.7 L42.5,0 L50,10 L42.5,20 L42.5,18.3 L0,18.3 L0,1.7"/> </g> <g fill="none" stroke="#000000" stroke-opacity="1" stroke-width="1" stroke-linecap="square" stroke-linejoin="bevel" transform="matrix(1,0,0,1,1502.13,1458)" font-family="MS Shell Dlg 2" font-size="9.75" font-weight="400" font-style="normal" > <text fill="#000000" fill-opacity="1" stroke="none" xml:space="preserve" x="4" y="17" font-family="Arial" font-size="10pt" font-weight="700" font-style="italic" >G</text> </g> <g fill="#ff9999" fill-opacity="1" stroke="#bfbfbf" stroke-opacity="1" stroke-width="1" stroke-linecap="square" stroke-linejoin="bevel" transform="matrix(1,0,0,1,1540,1480)" font-family="MS Shell Dlg 2" font-size="9.75" font-weight="400" font-style="normal" > <path vector-effect="none" fill-rule="evenodd" d="M0,10 L7.5,0 L7.5,1.7 L50,1.7 L50,18.3 L7.5,18.3 L7.5,20 L0,10"/> </g> <g fill="none" stroke="#000000" stroke-opacity="1" stroke-width="1" stroke-linecap="square" stroke-linejoin="bevel" transform="matrix(1,0,0,1,1560.87,1478)" font-family="MS Shell Dlg 2" font-size="9.75" font-weight="400" font-style="normal" > <text fill="#000000" fill-opacity="1" stroke="none" xml:space="preserve" x="4" y="17" font-family="Arial" font-size="10pt" font-weight="700" font-style="italic" >T</text> </g> <g fill="#ff9999" fill-opacity="1" stroke="#bfbfbf" stroke-opacity="1" stroke-width="1" stroke-linecap="square" stroke-linejoin="bevel" transform="matrix(1,0,0,1,1591,1460)" font-family="MS Shell Dlg 2" font-size="9.75" font-weight="400" font-style="normal" > <path vector-effect="none" fill-rule="evenodd" d="M0,1.7 L42.5,1.7 L42.5,0 L50,10 L42.5,20 L42.5,18.3 L0,18.3 L0,1.7"/> </g> <g fill="none" stroke="#000000" stroke-opacity="1" stroke-width="1" stroke-linecap="square" stroke-linejoin="bevel" transform="matrix(1,0,0,1,1604.13,1458)" font-family="MS Shell Dlg 2" font-size="9.75" font-weight="400" font-style="normal" > <text fill="#000000" fill-opacity="1" stroke="none" xml:space="preserve" x="4" y="17" font-family="Arial" font-size="10pt" font-weight="700" font-style="italic" >G</text> </g> <g fill="#ff9999" fill-opacity="1" stroke="#bfbfbf" stroke-opacity="1" stroke-width="1" stroke-linecap="square" stroke-linejoin="bevel" transform="matrix(1,0,0,1,1642,1480)" font-family="MS Shell Dlg 2" font-size="9.75" font-weight="400" font-style="normal" > <path vector-effect="none" fill-rule="evenodd" d="M0,10 L7.5,0 L7.5,1.7 L50,1.7 L50,18.3 L7.5,18.3 L7.5,20 L0,10"/> </g> <g fill="none" stroke="#000000" stroke-opacity="1" stroke-width="1" stroke-linecap="square" stroke-linejoin="bevel" transform="matrix(1,0,0,1,1662.87,1478)" font-family="MS Shell Dlg 2" font-size="9.75" font-weight="400" font-style="normal" > <text fill="#000000" fill-opacity="1" stroke="none" xml:space="preserve" x="4" y="17" font-family="Arial" font-size="10pt" font-weight="700" font-style="italic" >T</text> </g> <g fill="#ff9999" fill-opacity="1" stroke="#bfbfbf" stroke-opacity="1" stroke-width="1" stroke-linecap="square" stroke-linejoin="bevel" transform="matrix(1,0,0,1,1693,1460)" font-family="MS Shell Dlg 2" font-size="9.75" font-weight="400" font-style="normal" > <path vector-effect="none" fill-rule="evenodd" d="M0,1.7 L42.5,1.7 L42.5,0 L50,10 L42.5,20 L42.5,18.3 L0,18.3 L0,1.7"/> </g> <g fill="none" stroke="#000000" stroke-opacity="1" stroke-width="1" stroke-linecap="square" stroke-linejoin="bevel" transform="matrix(1,0,0,1,1706.13,1458)" font-family="MS Shell Dlg 2" font-size="9.75" font-weight="400" font-style="normal" > <text fill="#000000" fill-opacity="1" stroke="none" xml:space="preserve" x="4" y="17" font-family="Arial" font-size="10pt" font-weight="700" font-style="italic" >G</text> </g> <g fill="#ff9999" fill-opacity="1" stroke="#bfbfbf" stroke-opacity="1" stroke-width="1" stroke-linecap="square" stroke-linejoin="bevel" transform="matrix(1,0,0,1,1744,1480)" font-family="MS Shell Dlg 2" font-size="9.75" font-weight="400" font-style="normal" > <path vector-effect="none" fill-rule="evenodd" d="M0,10 L7.5,0 L7.5,1.7 L50,1.7 L50,18.3 L7.5,18.3 L7.5,20 L0,10"/> </g> <g fill="none" stroke="#000000" stroke-opacity="1" stroke-width="1" stroke-linecap="square" stroke-linejoin="bevel" transform="matrix(1,0,0,1,1764.87,1478)" font-family="MS Shell Dlg 2" font-size="9.75" font-weight="400" font-style="normal" > <text fill="#000000" fill-opacity="1" stroke="none" xml:space="preserve" x="4" y="17" font-family="Arial" font-size="10pt" font-weight="700" font-style="italic" >T</text> </g> <g fill="#ffff33" fill-opacity="1" stroke="#bfbfbf" stroke-opacity="1" stroke-width="1" stroke-linecap="square" stroke-linejoin="bevel" transform="matrix(1,0,0,1,1795,1460)" font-family="MS Shell Dlg 2" font-size="9.75" font-weight="400" font-style="normal" > <rect x="0" y="0" width="50" height="20"/> </g> <g fill="none" stroke="#000000" stroke-opacity="1" stroke-width="1" stroke-linecap="square" stroke-linejoin="bevel" transform="matrix(1,0,0,1,1804.5,1458)" font-family="MS Shell Dlg 2" font-size="9.75" font-weight="400" font-style="normal" > <text fill="#000000" fill-opacity="1" stroke="none" xml:space="preserve" x="4" y="17" font-family="Arial" font-size="10pt" font-weight="700" font-style="italic" >cytb</text> </g> <g fill="#ffff33" fill-opacity="1" stroke="#bfbfbf" stroke-opacity="1" stroke-width="1" stroke-linecap="square" stroke-linejoin="bevel" transform="matrix(1,0,0,1,1846,1460)" font-family="MS Shell Dlg 2" font-size="9.75" font-weight="400" font-style="normal" > <rect x="0" y="0" width="50" height="20"/> </g> <g fill="none" stroke="#000000" stroke-opacity="1" stroke-width="1" stroke-linecap="square" stroke-linejoin="bevel" transform="matrix(1,0,0,1,1850,1458)" font-family="MS Shell Dlg 2" font-size="9.75" font-weight="400" font-style="normal" > <text fill="#000000" fill-opacity="1" stroke="none" xml:space="preserve" x="4" y="17" font-family="Arial" font-size="10pt" font-weight="700" font-style="italic" >nad4L</text> </g> <g

fill="#ff9999" fill-opacity="1" stroke="#bfbfbf" stroke-opacity="1" stroke-width="1" stroke-linecap="square" stroke-linejoin="bevel" transform="matrix(1,0,0,1,1897,1460)" font-family="MS Shell Dlg 2" font-size="9.75" font-weight="400" font-style="normal" > <path vector-effect="none" fill-rule="evenodd" d="M0,1.7 L42.5,1.7 L42.5,0 L50,10 L42.5,20 L42.5,18.3 L0,18.3 L0,1.7"/> </g> <g fill="none" stroke="#000000" stroke-opacity="1" stroke-width="1" stroke-linecap="square" stroke-linejoin="bevel" transform="matrix(1,0,0,1,1910.13,1458)" font-family="MS Shell Dlg 2" font-size="9.75" font-weight="400" font-style="normal" > <text fill="#000000" fill-opacity="1" stroke="none" xml:space="preserve" x="4" y="17" font-family="Arial" font-size="10pt" font-weight="700" font-style="italic" >A</text> </g> <g fill="#ff9999" fill-opacity="1" stroke="#bfbfbf" stroke-opacity="1" stroke-width="1" stroke-linecap="square" stroke-linejoin="bevel" transform="matrix(1,0,0,1,1948,1460)" font-family="MS Shell Dlg 2" font-size="9.75" font-weight="400" font-style="normal" > <path vector-effect="none" fill-rule="evenodd" d="M0,1.7 L42.5,1.7 L42.5,0 L50,10 L42.5,20 L42.5,18.3 L0,18.3 L0,1.7"/> </g> <g fill="none" stroke="#000000" stroke-opacity="1" stroke-width="1" stroke-linecap="square" stroke-linejoin="bevel" transform="matrix(1,0,0,1,1961.13,1458)" font-family="MS Shell Dlg 2" font-size="9.75" font-weight="400" font-style="normal" > <text fill="#000000" fill-opacity="1" stroke="none" xml:space="preserve" x="4" y="17" font-family="Arial" font-size="10pt" font-weight="700" font-style="italic" >H</text> </g> <g fill="#ffff33" fill-opacity="1" stroke="#bfbfbf" stroke-opacity="1" stroke-width="1" stroke-linecap="square" stroke-linejoin="bevel" transform="matrix(1,0,0,1,1999,1460)" font-family="MS Shell Dlg 2" font-size="9.75" font-weight="400" font-style="normal" > <rect x="0" y="0" width="50" height="20"/> </g> <g fill="none" stroke="#000000" stroke-opacity="1" stroke-width="1" stroke-linecap="square" stroke-linejoin="bevel" transform="matrix(1,0,0,1,2006,1458)" font-family="MS Shell Dlg 2" font-size="9.75" font-weight="400" font-style="normal" > <text fill="#000000" fill-opacity="1" stroke="none" xml:space="preserve" x="4" y="17" font-family="Arial" font-size="10pt" font-weight="700" font-style="italic" >nad5</text> </g> <g fill="#ffff33" fill-opacity="1" stroke="#bfbfbf" stroke-opacity="1" stroke-width="1" stroke-linecap="square" stroke-linejoin="bevel" transform="matrix(1,0,0,1,2050,1460)" font-family="MS Shell Dlg 2" font-size="9.75" font-weight="400" font-style="normal" > <rect x="0" y="0" width="50" height="20"/> </g> <g fill="none" stroke="#000000" stroke-opacity="1" stroke-width="1" stroke-linecap="square" stroke-linejoin="bevel" transform="matrix(1,0,0,1,2058,1458)" font-family="MS Shell Dlg 2" font-size="9.75" font-weight="400" font-style="normal" > <text fill="#000000" fill-opacity="1" stroke="none" xml:space="preserve" x="4" y="17" font-family="Arial" font-size="10pt" font-weight="700" font-style="italic" >cox2</text> </g> <g fill="#ffff33" fill-opacity="1" stroke="#bfbfbf" stroke-opacity="1" stroke-width="1" stroke-linecap="square" stroke-linejoin="bevel" transform="matrix(1,0,0,1,2101,1460)" font-family="MS Shell Dlg 2" font-size="9.75" font-weight="400" font-style="normal" > <rect x="0" y="0" width="50" height="20"/> </g> <g fill="none" stroke="#000000" stroke-opacity="1" stroke-width="1" stroke-linecap="square" stroke-linejoin="bevel" transform="matrix(1,0,0,1,2108,1458)" font-family="MS Shell Dlg 2" font-size="9.75" font-weight="400" font-style="normal" > <text fill="#000000" fill-opacity="1" stroke="none" xml:space="preserve" x="4" y="17" font-family="Arial" font-size="10pt" font-weight="700" font-style="italic" >nad1</text> </g> <g fill="none" stroke="#000000" stroke-opacity="1" stroke-width="1" stroke-linecap="square" stroke-linejoin="bevel" transform="matrix(1,0,0,1,2152,1460)" font-family="MS Shell Dlg 2" font-size="9.75" font-weight="400" font-style="normal" > <text fill="#000000" fill-opacity="1" stroke="none" xml:space="preserve" x="4" y="17" font-family="Arial" font-size="10pt" font-weight="700" font-style="italic" >(+)</text> </g> <g fill="none" stroke="#000000" stroke-opacity="1" stroke-width="1" stroke-linecap="square" stroke-linejoin="bevel" transform="matrix(1,0,0,1,2152,1480)" font-family="MS Shell Dlg 2" font-size="9.75" font-weight="400" font-style="normal" > <text fill="#000000" fill-opacity="1" stroke="none" xml:space="preserve" x="4" y="17" font-family="Arial" font-size="10pt" font-weight="700" font-style="italic" >(-)</text> </g> <g fill="none" stroke="#000000" stroke-opacity="1" stroke-width="1" stroke-linecap="square" stroke-linejoin="bevel" transform="matrix(1,0,0,1,10,1520)" font-family="MS Shell Dlg 2" font-size="9.75" font-weight="400" font-style="normal" > <text fill="#000000" fill-opacity="1" stroke="none" xml:space="preserve" x="4" y="17" font-family="Arial" font-size="10pt" font-weight="700" font-style="italic" >Bathymodiolus_japonicus_AP014560</text> </g> <g fill="#ffff33" fill-opacity="1" stroke="#bfbfbf" stroke-opacity="1" stroke-width="1" stroke-linecap="square" stroke-linejoin="bevel" transform="matrix(1,0,0,1,10,1550)" font-family="MS Shell Dlg 2" font-size="9.75" font-weight="400" font-style="normal" > <rect x="0" y="0" width="50" height="20"/> </g> <g fill="none" stroke="#000000" stroke-opacity="1" stroke-width="1" stroke-linecap="square" stroke-linejoin="bevel" transform="matrix(1,0,0,1,18,1548)" font-family="MS Shell Dlg 2" font-size="9.75" font-weight="400" font-style="normal" > <text fill="#000000" fill-opacity="1" stroke="none" xml:space="preserve" x="4" y="17" font-family="Arial" font-size="10pt" font-weight="700" font-style="italic" >cox1</text> </g> <g fill="#ff9999" fill-opacity="1" stroke="#bfbfbf" stroke-opacity="1" stroke-width="1" stroke-linecap="square" stroke-linejoin="bevel"

transform="matrix(1,0,0,1,61,1550)" font-family="MS Shell Dlg 2" font-size="9.75" font-weight="400" font-style="normal" > <path vector-effect="none" fill-rule="evenodd" d="M0,1.7 L42.5,1.7 L42.5,0 L50,10 L42.5,20 L42.5,18.3 L0,18.3 L0,1.7"/> </g> <g fill="none" stroke="#000000" stroke-opacity="1" stroke-width="1" stroke-linecap="square" stroke-linejoin="bevel" transform="matrix(1,0,0,1,74.6325,1548)" font-family="MS Shell Dlg 2" font-size="9.75" font-weight="400" font-style="normal" > <text fill="#000000" fill-opacity="1" stroke="none" xml:space="preserve" x="4" y="17" font-family="Arial" font-size="10pt" font-weight="700" font-style="italic" >P</text> </g> <g fill="#ff9999" fill-opacity="1" stroke="#bfbfbf" stroke-opacity="1" stroke-width="1" stroke-linecap="square" stroke-linejoin="bevel" transform="matrix(1,0,0,1,112,1550)" font-family="MS Shell Dlg 2" font-size="9.75" font-weight="400" font-style="normal" > <path vector-effect="none" fill-rule="evenodd" d="M0,1.7 L42.5,1.7 L42.5,0 L50,10 L42.5,20 L42.5,18.3 L0,18.3 L0,1.7"/> </g> <g fill="none" stroke="#000000" stroke-opacity="1" stroke-width="1" stroke-linecap="square" stroke-linejoin="bevel" transform="matrix(1,0,0,1,125.632,1548)" font-family="MS Shell Dlg 2" font-size="9.75" font-weight="400" font-style="normal" > <text fill="#000000" fill-opacity="1" stroke="none" xml:space="preserve" x="4" y="17" font-family="Arial" font-size="10pt" font-weight="700" font-style="italic" >E</text> </g> <g fill="#ff9999" fill-opacity="1" stroke="#bfbfbf" stroke-opacity="1" stroke-width="1" stroke-linecap="square" stroke-linejoin="bevel" transform="matrix(1,0,0,1,163,1550)" font-family="MS Shell Dlg 2" font-size="9.75" font-weight="400" font-style="normal" > <path vector-effect="none" fill-rule="evenodd" d="M0,1.7 L42.5,1.7 L42.5,0 L50,10 L42.5,20 L42.5,18.3 L0,18.3 L0,1.7"/> </g> <g fill="none" stroke="#000000" stroke-opacity="1" stroke-width="1" stroke-linecap="square" stroke-linejoin="bevel" transform="matrix(1,0,0,1,176.132,1548)" font-family="MS Shell Dlg 2" font-size="9.75" font-weight="400" font-style="normal" > <text fill="#000000" fill-opacity="1" stroke="none" xml:space="preserve" x="4" y="17" font-family="Arial" font-size="10pt" font-weight="700" font-style="italic" >C</text> </g> <g fill="#ffff33" fill-opacity="1" stroke="#bfbfbf" stroke-opacity="1" stroke-width="1" stroke-linecap="square" stroke-linejoin="bevel" transform="matrix(1,0,0,1,214,1550)" font-family="MS Shell Dlg 2" font-size="9.75" font-weight="400" font-style="normal" > <rect x="0" y="0" width="50" height="20"/> </g> <g fill="none" stroke="#000000" stroke-opacity="1" stroke-width="1" stroke-linecap="square" stroke-linejoin="bevel" transform="matrix(1,0,0,1,221,1548)" font-family="MS Shell Dlg 2" font-size="9.75" font-weight="400" font-style="normal" > <text fill="#000000" fill-opacity="1" stroke="none" xml:space="preserve" x="4" y="17" font-family="Arial" font-size="10pt" font-weight="700" font-style="italic" >nad3</text> </g> <g fill="#ff9999" fill-opacity="1" stroke="#bfbfbf" stroke-opacity="1" stroke-width="1" stroke-linecap="square" stroke-linejoin="bevel" transform="matrix(1,0,0,1,265,1550)" font-family="MS Shell Dlg 2" font-size="9.75" font-weight="400" font-style="normal" > <path vector-effect="none" fill-rule="evenodd" d="M0,1.7 L42.5,1.7 L42.5,0 L50,10 L42.5,20 L42.5,18.3 L0,18.3 L0,1.7"/> </g> <g fill="none" stroke="#000000" stroke-opacity="1" stroke-width="1" stroke-linecap="square" stroke-linejoin="bevel" transform="matrix(1,0,0,1,275.632,1548)" font-family="MS Shell Dlg 2" font-size="9.75" font-weight="400" font-style="normal" > <text fill="#000000" fill-opacity="1" stroke="none" xml:space="preserve" x="4" y="17" font-family="Arial" font-size="10pt" font-weight="700" font-style="italic" >L2</text> </g> <g fill="#ff9999" fill-opacity="1" stroke="#bfbfbf" stroke-opacity="1" stroke-width="1" stroke-linecap="square" stroke-linejoin="bevel" transform="matrix(1,0,0,1,316,1550)" font-family="MS Shell Dlg 2" font-size="9.75" font-weight="400" font-style="normal" > <path vector-effect="none" fill-rule="evenodd" d="M0,1.7 L42.5,1.7 L42.5,0 L50,10 L42.5,20 L42.5,18.3 L0,18.3 L0,1.7"/> </g> <g fill="none" stroke="#000000" stroke-opacity="1" stroke-width="1" stroke-linecap="square" stroke-linejoin="bevel" transform="matrix(1,0,0,1,327.132,1548)" font-family="MS Shell Dlg 2" font-size="9.75" font-weight="400" font-style="normal" > <text fill="#000000" fill-opacity="1" stroke="none" xml:space="preserve" x="4" y="17" font-family="Arial" font-size="10pt" font-weight="700" font-style="italic" >W</text> </g> <g fill="#ffff33" fill-opacity="1" stroke="#bfbfbf" stroke-opacity="1" stroke-width="1" stroke-linecap="square" stroke-linejoin="bevel" transform="matrix(1,0,0,1,367,1550)" font-family="MS Shell Dlg 2" font-size="9.75" font-weight="400" font-style="normal" > <rect x="0" y="0" width="50" height="20"/> </g> <g fill="none" stroke="#000000" stroke-opacity="1" stroke-width="1" stroke-linecap="square" stroke-linejoin="bevel" transform="matrix(1,0,0,1,375.5,1548)" font-family="MS Shell Dlg 2" font-size="9.75" font-weight="400" font-style="normal" > <text fill="#000000" fill-opacity="1" stroke="none" xml:space="preserve" x="4" y="17" font-family="Arial" font-size="10pt" font-weight="700" font-style="italic" >atp8</text> </g> <g fill="#ff9999" fill-opacity="1" stroke="#bfbfbf" stroke-opacity="1" stroke-width="1" stroke-linecap="square" stroke-linejoin="bevel" transform="matrix(1,0,0,1,418,1550)" font-family="MS Shell Dlg 2" font-size="9.75" font-weight="400" font-style="normal" > <path vector-effect="none" fill-rule="evenodd" d="M0,1.7 L42.5,1.7 L42.5,0 L50,10 L42.5,20 L42.5,18.3 L0,18.3 L0,1.7"/> </g> <g fill="none" stroke="#000000" stroke-opacity="1" stroke-width="1" stroke-linecap="square" stroke-linejoin="bevel" transform="matrix(1,0,0,1,428.632,1548)" font-family="MS Shell Dlg 2" font-size="9.75" font-weight="400" font-style="normal" > <text fill="#000000" fill-opacity="1" stroke="none" xml:space="preserve" x="4" y="17" font-

family="Arial" font-size="10pt" font-weight="700" font-style="italic" >L1</text> </g> <g fill="#ff9999" fill-opacity="1" stroke="#bfbfbf" stroke-opacity="1" stroke-width="1" stroke-linecap="square" stroke-linejoin="bevel" transform="matrix(1,0,0,1,469,1550)" font-family="MS Shell Dlg 2" font-size="9.75" font-weight="400" font-style="normal" > <path vector-effect="none" fill-rule="evenodd" d="M0,1.7 L42.5,1.7 L42.5,0 L50,10 L42.5,20 L42.5,18.3 L0,18.3 L0,1.7"/> </g> <g fill="none" stroke="#000000" stroke-opacity="1" stroke-width="1" stroke-linecap="square" stroke-linejoin="bevel" transform="matrix(1,0,0,1,482.632,1548)" font-family="MS Shell Dlg 2" font-size="9.75" font-weight="400" font-style="normal" > <text fill="#000000" fill-opacity="1" stroke="none" xml:space="preserve" x="4" y="17" font-family="Arial" font-size="10pt" font-weight="700" font-style="italic" >K</text> </g> <g fill="#99ffff" fill-opacity="1" stroke="#bfbfbf" stroke-opacity="1" stroke-width="1" stroke-linecap="square" stroke-linejoin="bevel" transform="matrix(1,0,0,1,520,1550)" font-family="MS Shell Dlg 2" font-size="9.75" font-weight="400" font-style="normal" > <path vector-effect="none" fill-rule="evenodd" d="M0,5 C0,2.23858 2.23858,0 5,0 L45,0 C47.7614,0 50,2.23858 50,5 L50,15 C50,17.7614 47.7614,20 45,20 L5,20 C2.23858,20 0,17.7614 0,15 L0,5"/> </g> <g fill="none" stroke="#000000" stroke-opacity="1" stroke-width="1" stroke-linecap="square" stroke-linejoin="bevel" transform="matrix(1,0,0,1,528.5,1548)" font-family="MS Shell Dlg 2" font-size="9.75" font-weight="400" font-style="normal" > <text fill="#000000" fill-opacity="1" stroke="none" xml:space="preserve" x="4" y="17" font-family="Arial" font-size="10pt" font-weight="700" font-style="italic" >rrnS</text> </g> <g fill="#ff9999" fill-opacity="1" stroke="#bfbfbf" stroke-opacity="1" stroke-width="1" stroke-linecap="square" stroke-linejoin="bevel" transform="matrix(1,0,0,1,571,1550)" font-family="MS Shell Dlg 2" font-size="9.75" font-weight="400" font-style="normal" > <path vector-effect="none" fill-rule="evenodd" d="M0,1.7 L42.5,1.7 L42.5,0 L50,10 L42.5,20 L42.5,18.3 L0,18.3 L0,1.7"/> </g> <g fill="none" stroke="#000000" stroke-opacity="1" stroke-width="1" stroke-linecap="square" stroke-linejoin="bevel" transform="matrix(1,0,0,1,583.633,1548)" font-family="MS Shell Dlg 2" font-size="9.75" font-weight="400" font-style="normal" > <text fill="#000000" fill-opacity="1" stroke="none" xml:space="preserve" x="4" y="17" font-family="Arial" font-size="10pt" font-weight="700" font-style="italic" >Q</text> </g> <g fill="#ff9999" fill-opacity="1" stroke="#bfbfbf" stroke-opacity="1" stroke-width="1" stroke-linecap="square" stroke-linejoin="bevel" transform="matrix(1,0,0,1,622,1550)" font-family="MS Shell Dlg 2" font-size="9.75" font-weight="400" font-style="normal" > <path vector-effect="none" fill-rule="evenodd" d="M0,1.7 L42.5,1.7 L42.5,0 L50,10 L42.5,20 L42.5,18.3 L0,18.3 L0,1.7"/> </g> <g fill="none" stroke="#000000" stroke-opacity="1" stroke-width="1" stroke-linecap="square" stroke-linejoin="bevel" transform="matrix(1,0,0,1,635.633,1548)" font-family="MS Shell Dlg 2" font-size="9.75" font-weight="400" font-style="normal" > <text fill="#000000" fill-opacity="1" stroke="none" xml:space="preserve" x="4" y="17" font-family="Arial" font-size="10pt" font-weight="700" font-style="italic" >Y</text> </g> <g fill="#ffff33" fill-opacity="1" stroke="#bfbfbf" stroke-opacity="1" stroke-width="1" stroke-linecap="square" stroke-linejoin="bevel" transform="matrix(1,0,0,1,673,1550)" font-family="MS Shell Dlg 2" font-size="9.75" font-weight="400" font-style="normal" > <rect x="0" y="0" width="50" height="20"/> </g> <g fill="none" stroke="#000000" stroke-opacity="1" stroke-width="1" stroke-linecap="square" stroke-linejoin="bevel" transform="matrix(1,0,0,1,681.5,1548)" font-family="MS Shell Dlg 2" font-size="9.75" font-weight="400" font-style="normal" > <text fill="#000000" fill-opacity="1" stroke="none" xml:space="preserve" x="4" y="17" font-family="Arial" font-size="10pt" font-weight="700" font-style="italic" >atp6</text> </g> <g fill="#ff9999" fill-opacity="1" stroke="#bfbfbf" stroke-opacity="1" stroke-width="1" stroke-linecap="square" stroke-linejoin="bevel" transform="matrix(1,0,0,1,724,1550)" font-family="MS Shell Dlg 2" font-size="9.75" font-weight="400" font-style="normal" > <path vector-effect="none" fill-rule="evenodd" d="M0,1.7 L42.5,1.7 L42.5,0 L50,10 L42.5,20 L42.5,18.3 L0,18.3 L0,1.7"/> </g> <g fill="none" stroke="#000000" stroke-opacity="1" stroke-width="1" stroke-linecap="square" stroke-linejoin="bevel" transform="matrix(1,0,0,1,737.133,1548)" font-family="MS Shell Dlg 2" font-size="9.75" font-weight="400" font-style="normal" > <text fill="#000000" fill-opacity="1" stroke="none" xml:space="preserve" x="4" y="17" font-family="Arial" font-size="10pt" font-weight="700" font-style="italic" >R</text> </g> <g fill="#ff9999" fill-opacity="1" stroke="#bfbfbf" stroke-opacity="1" stroke-width="1" stroke-linecap="square" stroke-linejoin="bevel" transform="matrix(1,0,0,1,775,1550)" font-family="MS Shell Dlg 2" font-size="9.75" font-weight="400" font-style="normal" > <path vector-effect="none" fill-rule="evenodd" d="M0,1.7 L42.5,1.7 L42.5,0 L50,10 L42.5,20 L42.5,18.3 L0,18.3 L0,1.7"/> </g> <g fill="none" stroke="#000000" stroke-opacity="1" stroke-width="1" stroke-linecap="square" stroke-linejoin="bevel" transform="matrix(1,0,0,1,787.133,1548)" font-family="MS Shell Dlg 2" font-size="9.75" font-weight="400" font-style="normal" > <text fill="#000000" fill-opacity="1" stroke="none" xml:space="preserve" x="4" y="17" font-family="Arial" font-size="10pt" font-weight="700" font-style="italic" >M</text> </g> <g fill="#ff9999" fill-opacity="1" stroke="#bfbfbf" stroke-opacity="1" stroke-width="1" stroke-linecap="square" stroke-linejoin="bevel" transform="matrix(1,0,0,1,826,1550)" font-family="MS Shell Dlg 2" font-size="9.75" font-weight="400" font-style="normal" > <path vector-effect="none" fill-rule="evenodd" d="M0,1.7 L42.5,1.7 L42.5,0 L50,10 L42.5,20

L42.5,18.3 L0,18.3 L0,1.7"/> </g> <g fill="none" stroke="#000000" stroke-opacity="1" stroke-width="1" stroke-linecap="square" stroke-linejoin="bevel" transform="matrix(1,0,0,1,839.133,1548)" font-family="MS Shell Dlg 2" font-size="9.75" font-weight="400" font-style="normal" > <text fill="#000000" fill-opacity="1" stroke="none" xml:space="preserve" x="4" y="17" font-family="Arial" font-size="10pt" font-weight="700" font-style="italic" >V</text> </g> <g fill="#ffff33" fill-opacity="1" stroke="#bfbfbf" stroke-opacity="1" stroke-width="1" stroke-linecap="square" stroke-linejoin="bevel" transform="matrix(1,0,0,1,877,1550)" font-family="MS Shell Dlg 2" font-size="9.75" font-weight="400" font-style="normal" > <rect x="0" y="0" width="50" height="20"/> </g> <g fill="none" stroke="#000000" stroke-opacity="1" stroke-width="1" stroke-linecap="square" stroke-linejoin="bevel" transform="matrix(1,0,0,1,884,1548)" font-family="MS Shell Dlg 2" font-size="9.75" font-weight="400" font-style="normal" > <text fill="#000000" fill-opacity="1" stroke="none" xml:space="preserve" x="4" y="17" font-family="Arial" font-size="10pt" font-weight="700" font-style="italic" >nad4</text> </g> <g fill="#ff9999" fill-opacity="1" stroke="#bfbfbf" stroke-opacity="1" stroke-width="1" stroke-linecap="square" stroke-linejoin="bevel" transform="matrix(1,0,0,1,928,1550)" font-family="MS Shell Dlg 2" font-size="9.75" font-weight="400" font-style="normal" > <path vector-effect="none" fill-rule="evenodd" d="M0,1.7 L42.5,1.7 L42.5,0 L50,10 L42.5,20 L42.5,18.3 L0,18.3 L0,1.7"/> </g> <g fill="none" stroke="#000000" stroke-opacity="1" stroke-width="1" stroke-linecap="square" stroke-linejoin="bevel" transform="matrix(1,0,0,1,941.133,1548)" font-family="MS Shell Dlg 2" font-size="9.75" font-weight="400" font-style="normal" > <text fill="#000000" fill-opacity="1" stroke="none" xml:space="preserve" x="4" y="17" font-family="Arial" font-size="10pt" font-weight="700" font-style="italic" >N</text> </g> <g fill="#ffff33" fill-opacity="1" stroke="#bfbfbf" stroke-opacity="1" stroke-width="1" stroke-linecap="square" stroke-linejoin="bevel" transform="matrix(1,0,0,1,979,1550)" font-family="MS Shell Dlg 2" font-size="9.75" font-weight="400" font-style="normal" > <rect x="0" y="0" width="50" height="20"/> </g> <g fill="none" stroke="#000000" stroke-opacity="1" stroke-width="1" stroke-linecap="square" stroke-linejoin="bevel" transform="matrix(1,0,0,1,987,1548)" font-family="MS Shell Dlg 2" font-size="9.75" font-weight="400" font-style="normal" > <text fill="#000000" fill-opacity="1" stroke="none" xml:space="preserve" x="4" y="17" font-family="Arial" font-size="10pt" font-weight="700" font-style="italic" >cox3</text> </g> <g fill="#ff9999" fill-opacity="1" stroke="#bfbfbf" stroke-opacity="1" stroke-width="1" stroke-linecap="square" stroke-linejoin="bevel" transform="matrix(1,0,0,1,1030,1550)" font-family="MS Shell Dlg 2" font-size="9.75" font-weight="400" font-style="normal" > <path vector-effect="none" fill-rule="evenodd" d="M0,1.7 L42.5,1.7 L42.5,0 L50,10 L42.5,20 L42.5,18.3 L0,18.3 L0,1.7"/> </g> <g fill="none" stroke="#000000" stroke-opacity="1" stroke-width="1" stroke-linecap="square" stroke-linejoin="bevel" transform="matrix(1,0,0,1,1043.63,1548)" font-family="MS Shell Dlg 2" font-size="9.75" font-weight="400" font-style="normal" > <text fill="#000000" fill-opacity="1" stroke="none" xml:space="preserve" x="4" y="17" font-family="Arial" font-size="10pt" font-weight="700" font-style="italic" >F</text> </g> <g fill="#ff9999" fill-opacity="1" stroke="#bfbfbf" stroke-opacity="1" stroke-width="1" stroke-linecap="square" stroke-linejoin="bevel" transform="matrix(1,0,0,1,1081,1550)" font-family="MS Shell Dlg 2" font-size="9.75" font-weight="400" font-style="normal" > <path vector-effect="none" fill-rule="evenodd" d="M0,1.7 L42.5,1.7 L42.5,0 L50,10 L42.5,20 L42.5,18.3 L0,18.3 L0,1.7"/> </g> <g fill="none" stroke="#000000" stroke-opacity="1" stroke-width="1" stroke-linecap="square" stroke-linejoin="bevel" transform="matrix(1,0,0,1,1090.63,1548)" font-family="MS Shell Dlg 2" font-size="9.75" font-weight="400" font-style="normal" > <text fill="#000000" fill-opacity="1" stroke="none" xml:space="preserve" x="4" y="17" font-family="Arial" font-size="10pt" font-weight="700" font-style="italic" >S1</text> </g> <g fill="#99ffff" fill-opacity="1" stroke="#bfbfbf" stroke-opacity="1" stroke-width="1" stroke-linecap="square" stroke-linejoin="bevel" transform="matrix(1,0,0,1,1132,1550)" font-family="MS Shell Dlg 2" font-size="9.75" font-weight="400" font-style="normal" > <path vector-effect="none" fill-rule="evenodd" d="M0,5 C0,2.23858 2.23858,0 5,0 L45,0 C47.7614,0 50,2.23858 50,5 L50,15 C50,17.7614 47.7614,20 45,20 L5,20 C2.23858,20 0,17.7614 0,15 L0,5"/> </g> <g fill="none" stroke="#000000" stroke-opacity="1" stroke-width="1" stroke-linecap="square" stroke-linejoin="bevel" transform="matrix(1,0,0,1,1141.5,1548)" font-family="MS Shell Dlg 2" font-size="9.75" font-weight="400" font-style="normal" > <text fill="#000000" fill-opacity="1" stroke="none" xml:space="preserve" x="4" y="17" font-family="Arial" font-size="10pt" font-weight="700" font-style="italic" >rrnL</text> </g> <g fill="#ff9999" fill-opacity="1" stroke="#bfbfbf" stroke-opacity="1" stroke-width="1" stroke-linecap="square" stroke-linejoin="bevel" transform="matrix(1,0,0,1,1183,1550)" font-family="MS Shell Dlg 2" font-size="9.75" font-weight="400" font-style="normal" > <path vector-effect="none" fill-rule="evenodd" d="M0,1.7 L42.5,1.7 L42.5,0 L50,10 L42.5,20 L42.5,18.3 L0,18.3 L0,1.7"/> </g> <g fill="none" stroke="#000000" stroke-opacity="1" stroke-width="1" stroke-linecap="square" stroke-linejoin="bevel" transform="matrix(1,0,0,1,1192.63,1548)" font-family="MS Shell Dlg 2" font-size="9.75" font-weight="400" font-style="normal" > <text fill="#000000" fill-opacity="1" stroke="none" xml:space="preserve" x="4" y="17" font-family="Arial" font-size="10pt" font-weight="700" font-style="italic" >S2</text> </g> <g fill="#ff9999"

fill-opacity="1" stroke="#bfbfbf" stroke-opacity="1" stroke-width="1" stroke-linecap="square" stroke-linejoin="bevel" transform="matrix(1,0,0,1,1234,1550)" font-family="MS Shell Dlg 2" font-size="9.75" font-weight="400" font-style="normal" > <path vector-effect="none" fill-rule="evenodd" d="M0,1.7 L42.5,1.7 L42.5,0 L50,10 L42.5,20 L42.5,18.3 L0,18.3 L0,1.7"/> </g> <g fill="none" stroke="#000000" stroke-opacity="1" stroke-width="1" stroke-linecap="square" stroke-linejoin="bevel" transform="matrix(1,0,0,1,1247.13,1548)" font-family="MS Shell Dlg 2" font-size="9.75" font-weight="400" font-style="normal" > <text fill="#000000" fill-opacity="1" stroke="none" xml:space="preserve" x="4" y="17" font-family="Arial" font-size="10pt" font-weight="700" font-style="italic" >D</text> </g> <g fill="#ffff33" fill-opacity="1" stroke="#bfbfbf" stroke-opacity="1" stroke-width="1" stroke-linecap="square" stroke-linejoin="bevel" transform="matrix(1,0,0,1,1285,1550)" font-family="MS Shell Dlg 2" font-size="9.75" font-weight="400" font-style="normal" > <rect x="0" y="0" width="50" height="20"/> </g> <g fill="none" stroke="#000000" stroke-opacity="1" stroke-width="1" stroke-linecap="square" stroke-linejoin="bevel" transform="matrix(1,0,0,1,1292,1548)" font-family="MS Shell Dlg 2" font-size="9.75" font-weight="400" font-style="normal" > <text fill="#000000" fill-opacity="1" stroke="none" xml:space="preserve" x="4" y="17" font-family="Arial" font-size="10pt" font-weight="700" font-style="italic" >nad6</text> </g> <g fill="#ff9999" fill-opacity="1" stroke="#bfbfbf" stroke-opacity="1" stroke-width="1" stroke-linecap="square" stroke-linejoin="bevel" transform="matrix(1,0,0,1,1336,1550)" font-family="MS Shell Dlg 2" font-size="9.75" font-weight="400" font-style="normal" > <path vector-effect="none" fill-rule="evenodd" d="M0,1.7 L42.5,1.7 L42.5,0 L50,10 L42.5,20 L42.5,18.3 L0,18.3 L0,1.7"/> </g> <g fill="none" stroke="#000000" stroke-opacity="1" stroke-width="1" stroke-linecap="square" stroke-linejoin="bevel" transform="matrix(1,0,0,1,1351.13,1548)" font-family="MS Shell Dlg 2" font-size="9.75" font-weight="400" font-style="normal" > <text fill="#000000" fill-opacity="1" stroke="none" xml:space="preserve" x="4" y="17" font-family="Arial" font-size="10pt" font-weight="700" font-style="italic" >I</text> </g> <g fill="#ffff33" fill-opacity="1" stroke="#bfbfbf" stroke-opacity="1" stroke-width="1" stroke-linecap="square" stroke-linejoin="bevel" transform="matrix(1,0,0,1,1387,1550)" font-family="MS Shell Dlg 2" font-size="9.75" font-weight="400" font-style="normal" > <rect x="0" y="0" width="50" height="20"/> </g> <g fill="none" stroke="#000000" stroke-opacity="1" stroke-width="1" stroke-linecap="square" stroke-linejoin="bevel" transform="matrix(1,0,0,1,1394,1548)" font-family="MS Shell Dlg 2" font-size="9.75" font-weight="400" font-style="normal" > <text fill="#000000" fill-opacity="1" stroke="none" xml:space="preserve" x="4" y="17" font-family="Arial" font-size="10pt" font-weight="700" font-style="italic" >nad2</text> </g> <g fill="#ff9999" fill-opacity="1" stroke="#bfbfbf" stroke-opacity="1" stroke-width="1" stroke-linecap="square" stroke-linejoin="bevel" transform="matrix(1,0,0,1,1438,1550)" font-family="MS Shell Dlg 2" font-size="9.75" font-weight="400" font-style="normal" > <path vector-effect="none" fill-rule="evenodd" d="M0,1.7 L42.5,1.7 L42.5,0 L50,10 L42.5,20 L42.5,18.3 L0,18.3 L0,1.7"/> </g> <g fill="none" stroke="#000000" stroke-opacity="1" stroke-width="1" stroke-linecap="square" stroke-linejoin="bevel" transform="matrix(1,0,0,1,1449.13,1548)" font-family="MS Shell Dlg 2" font-size="9.75" font-weight="400" font-style="normal" > <text fill="#000000" fill-opacity="1" stroke="none" xml:space="preserve" x="4" y="17" font-family="Arial" font-size="10pt" font-weight="700" font-style="italic" >W</text> </g> <g fill="#ff9999" fill-opacity="1" stroke="#bfbfbf" stroke-opacity="1" stroke-width="1" stroke-linecap="square" stroke-linejoin="bevel" transform="matrix(1,0,0,1,1489,1550)" font-family="MS Shell Dlg 2" font-size="9.75" font-weight="400" font-style="normal" > <path vector-effect="none" fill-rule="evenodd" d="M0,1.7 L42.5,1.7 L42.5,0 L50,10 L42.5,20 L42.5,18.3 L0,18.3 L0,1.7"/> </g> <g fill="none" stroke="#000000" stroke-opacity="1" stroke-width="1" stroke-linecap="square" stroke-linejoin="bevel" transform="matrix(1,0,0,1,1502.13,1548)" font-family="MS Shell Dlg 2" font-size="9.75" font-weight="400" font-style="normal" > <text fill="#000000" fill-opacity="1" stroke="none" xml:space="preserve" x="4" y="17" font-family="Arial" font-size="10pt" font-weight="700" font-style="italic" >G</text> </g> <g fill="#ff9999" fill-opacity="1" stroke="#bfbfbf" stroke-opacity="1" stroke-width="1" stroke-linecap="square" stroke-linejoin="bevel" transform="matrix(1,0,0,1,1540,1570)" font-family="MS Shell Dlg 2" font-size="9.75" font-weight="400" font-style="normal" > <path vector-effect="none" fill-rule="evenodd" d="M0,10 L7.5,0 L7.5,1.7 L50,1.7 L50,18.3 L7.5,18.3 L7.5,20 L0,10"/> </g> <g fill="none" stroke="#000000" stroke-opacity="1" stroke-width="1" stroke-linecap="square" stroke-linejoin="bevel" transform="matrix(1,0,0,1,1560.87,1568)" font-family="MS Shell Dlg 2" font-size="9.75" font-weight="400" font-style="normal" > <text fill="#000000" fill-opacity="1" stroke="none" xml:space="preserve" x="4" y="17" font-family="Arial" font-size="10pt" font-weight="700" font-style="italic" >T</text> </g> <g fill="#ffff33" fill-opacity="1" stroke="#bfbfbf" stroke-opacity="1" stroke-width="1" stroke-linecap="square" stroke-linejoin="bevel" transform="matrix(1,0,0,1,1591,1550)" font-family="MS Shell Dlg 2" font-size="9.75" font-weight="400" font-style="normal" > <rect x="0" y="0" width="50" height="20"/> </g> <g fill="none" stroke="#000000" stroke-opacity="1" stroke-width="1" stroke-linecap="square" stroke-linejoin="bevel" transform="matrix(1,0,0,1,1600.5,1548)" font-family="MS Shell Dlg 2" font-size="9.75" font-

weight="400" font-style="normal" > <text fill="#000000" fill-opacity="1" stroke="none" xml:space="preserve" x="4" y="17" font-family="Arial" font-size="10pt" font-weight="700" font-style="italic" >cytb</text> </g> <g fill="#ffff33" fill-opacity="1" stroke="#bfbfbf" stroke-opacity="1" stroke-width="1" stroke-linecap="square" stroke-linejoin="bevel" transform="matrix(1,0,0,1,1642,1550)" font-family="MS Shell Dlg 2" font-size="9.75" font-weight="400" font-style="normal" > <rect x="0" y="0" width="50" height="20"/> </g> <g fill="none" stroke="#000000" stroke-opacity="1" stroke-width="1" stroke-linecap="square" stroke-linejoin="bevel" transform="matrix(1,0,0,1,1646,1548)" font-family="MS Shell Dlg 2" font-size="9.75" font-weight="400" font-style="normal" > <text fill="#000000" fill-opacity="1" stroke="none" xml:space="preserve" x="4" y="17" font-family="Arial" font-size="10pt" font-weight="700" font-style="italic" >nad4L</text> </g> <g fill="#ff9999" fill-opacity="1" stroke="#bfbfbf" stroke-opacity="1" stroke-width="1" stroke-linecap="square" stroke-linejoin="bevel" transform="matrix(1,0,0,1,1693,1550)" font-family="MS Shell Dlg 2" font-size="9.75" font-weight="400" font-style="normal" > <path vector-effect="none" fill-rule="evenodd" d="M0,1.7 L42.5,1.7 L42.5,0 L50,10 L42.5,20 L42.5,18.3 L0,18.3 L0,1.7"/> </g> <g fill="none" stroke="#000000" stroke-opacity="1" stroke-width="1" stroke-linecap="square" stroke-linejoin="bevel" transform="matrix(1,0,0,1,1706.13,1548)" font-family="MS Shell Dlg 2" font-size="9.75" font-weight="400" font-style="normal" > <text fill="#000000" fill-opacity="1" stroke="none" xml:space="preserve" x="4" y="17" font-family="Arial" font-size="10pt" font-weight="700" font-style="italic" >A</text> </g> <g fill="#ff9999" fill-opacity="1" stroke="#bfbfbf" stroke-opacity="1" stroke-width="1" stroke-linecap="square" stroke-linejoin="bevel" transform="matrix(1,0,0,1,1744,1550)" font-family="MS Shell Dlg 2" font-size="9.75" font-weight="400" font-style="normal" > <path vector-effect="none" fill-rule="evenodd" d="M0,1.7 L42.5,1.7 L42.5,0 L50,10 L42.5,20 L42.5,18.3 L0,18.3 L0,1.7"/> </g> <g fill="none" stroke="#000000" stroke-opacity="1" stroke-width="1" stroke-linecap="square" stroke-linejoin="bevel" transform="matrix(1,0,0,1,1757.13,1548)" font-family="MS Shell Dlg 2" font-size="9.75" font-weight="400" font-style="normal" > <text fill="#000000" fill-opacity="1" stroke="none" xml:space="preserve" x="4" y="17" font-family="Arial" font-size="10pt" font-weight="700" font-style="italic" >H</text> </g> <g fill="#ffff33" fill-opacity="1" stroke="#bfbfbf" stroke-opacity="1" stroke-width="1" stroke-linecap="square" stroke-linejoin="bevel" transform="matrix(1,0,0,1,1795,1550)" font-family="MS Shell Dlg 2" font-size="9.75" font-weight="400" font-style="normal" > <rect x="0" y="0" width="50" height="20"/> </g> <g fill="none" stroke="#000000" stroke-opacity="1" stroke-width="1" stroke-linecap="square" stroke-linejoin="bevel" transform="matrix(1,0,0,1,1802,1548)" font-family="MS Shell Dlg 2" font-size="9.75" font-weight="400" font-style="normal" > <text fill="#000000" fill-opacity="1" stroke="none" xml:space="preserve" x="4" y="17" font-family="Arial" font-size="10pt" font-weight="700" font-style="italic" >nad5</text> </g> <g fill="#ffff33" fill-opacity="1" stroke="#bfbfbf" stroke-opacity="1" stroke-width="1" stroke-linecap="square" stroke-linejoin="bevel" transform="matrix(1,0,0,1,1846,1550)" font-family="MS Shell Dlg 2" font-size="9.75" font-weight="400" font-style="normal" > <rect x="0" y="0" width="50" height="20"/> </g> <g fill="none" stroke="#000000" stroke-opacity="1" stroke-width="1" stroke-linecap="square" stroke-linejoin="bevel" transform="matrix(1,0,0,1,1854,1548)" font-family="MS Shell Dlg 2" font-size="9.75" font-weight="400" font-style="normal" > <text fill="#000000" fill-opacity="1" stroke="none" xml:space="preserve" x="4" y="17" font-family="Arial" font-size="10pt" font-weight="700" font-style="italic" >cox2</text> </g> <g fill="#ffff33" fill-opacity="1" stroke="#bfbfbf" stroke-opacity="1" stroke-width="1" stroke-linecap="square" stroke-linejoin="bevel" transform="matrix(1,0,0,1,1897,1550)" font-family="MS Shell Dlg 2" font-size="9.75" font-weight="400" font-style="normal" > <rect x="0" y="0" width="50" height="20"/> </g> <g fill="none" stroke="#000000" stroke-opacity="1" stroke-width="1" stroke-linecap="square" stroke-linejoin="bevel" transform="matrix(1,0,0,1,1904,1548)" font-family="MS Shell Dlg 2" font-size="9.75" font-weight="400" font-style="normal" > <text fill="#000000" fill-opacity="1" stroke="none" xml:space="preserve" x="4" y="17" font-family="Arial" font-size="10pt" font-weight="700" font-style="italic" >nad1</text> </g> <g fill="none" stroke="#000000" stroke-opacity="1" stroke-width="1" stroke-linecap="square" stroke-linejoin="bevel" transform="matrix(1,0,0,1,1948,1550)" font-family="MS Shell Dlg 2" font-size="9.75" font-weight="400" font-style="normal" > <text fill="#000000" fill-opacity="1" stroke="none" xml:space="preserve" x="4" y="17" font-family="Arial" font-size="10pt" font-weight="700" font-style="italic" >(+)</text> </g> <g fill="none" stroke="#000000" stroke-opacity="1" stroke-width="1" stroke-linecap="square" stroke-linejoin="bevel" transform="matrix(1,0,0,1,1948,1570)" font-family="MS Shell Dlg 2" font-size="9.75" font-weight="400" font-style="normal" > <text fill="#000000" fill-opacity="1" stroke="none" xml:space="preserve" x="4" y="17" font-family="Arial" font-size="10pt" font-weight="700" font-style="italic" >(-)</text> </g> <g fill="none" stroke="#000000" stroke-opacity="1" stroke-width="1" stroke-linecap="square" stroke-linejoin="bevel" transform="matrix(1,0,0,1,10,1610)" font-family="MS Shell Dlg 2" font-size="9.75" font-weight="400" font-style="normal" > <text fill="#000000" fill-opacity="1" stroke="none" xml:space="preserve" x="4" y="17" font-family="Arial" font-size="10pt" font-weight="700" font-style="italic"

>Bathymodiolus_securiformis_NC_039552</text> </g> <g fill="#ffff33" fill-opacity="1" stroke="#bfbfbf" stroke-opacity="1" stroke-width="1" stroke-linecap="square" stroke-linejoin="bevel" transform="matrix(1,0,0,1,10,1640)" font-family="MS Shell Dlg 2" font-size="9.75" font-weight="400" font-style="normal" > <rect x="0" y="0" width="50" height="20"/> </g> <g fill="none" stroke="#000000" stroke-opacity="1" stroke-width="1" stroke-linecap="square" stroke-linejoin="bevel" transform="matrix(1,0,0,1,18,1638)" font-family="MS Shell Dlg 2" font-size="9.75" font-weight="400" font-style="normal" > <text fill="#000000" fill-opacity="1" stroke="none" xml:space="preserve" x="4" y="17" font-family="Arial" font-size="10pt" font-weight="700" font-style="italic" >cox1</text> </g> <g fill="#ff9999" fill-opacity="1" stroke="#bfbfbf" stroke-opacity="1" stroke-width="1" stroke-linecap="square" stroke-linejoin="bevel" transform="matrix(1,0,0,1,61,1640)" font-family="MS Shell Dlg 2" font-size="9.75" font-weight="400" font-style="normal" > <path vector-effect="none" fill-rule="evenodd" d="M0,1.7 L42.5,1.7 L42.5,0 L50,10 L42.5,20 L42.5,18.3 L0,18.3 L0,1.7"/> </g> <g fill="none" stroke="#000000" stroke-opacity="1" stroke-width="1" stroke-linecap="square" stroke-linejoin="bevel" transform="matrix(1,0,0,1,74.6325,1638)" font-family="MS Shell Dlg 2" font-size="9.75" font-weight="400" font-style="normal" > <text fill="#000000" fill-opacity="1" stroke="none" xml:space="preserve" x="4" y="17" font-family="Arial" font-size="10pt" font-weight="700" font-style="italic" >P</text> </g> <g fill="#ff9999" fill-opacity="1" stroke="#bfbfbf" stroke-opacity="1" stroke-width="1" stroke-linecap="square" stroke-linejoin="bevel" transform="matrix(1,0,0,1,112,1640)" font-family="MS Shell Dlg 2" font-size="9.75" font-weight="400" font-style="normal" > <path vector-effect="none" fill-rule="evenodd" d="M0,1.7 L42.5,1.7 L42.5,0 L50,10 L42.5,20 L42.5,18.3 L0,18.3 L0,1.7"/> </g> <g fill="none" stroke="#000000" stroke-opacity="1" stroke-width="1" stroke-linecap="square" stroke-linejoin="bevel" transform="matrix(1,0,0,1,125.632,1638)" font-family="MS Shell Dlg 2" font-size="9.75" font-weight="400" font-style="normal" > <text fill="#000000" fill-opacity="1" stroke="none" xml:space="preserve" x="4" y="17" font-family="Arial" font-size="10pt" font-weight="700" font-style="italic" >E</text> </g> <g fill="#ff9999" fill-opacity="1" stroke="#bfbfbf" stroke-opacity="1" stroke-width="1" stroke-linecap="square" stroke-linejoin="bevel" transform="matrix(1,0,0,1,163,1640)" font-family="MS Shell Dlg 2" font-size="9.75" font-weight="400" font-style="normal" > <path vector-effect="none" fill-rule="evenodd" d="M0,1.7 L42.5,1.7 L42.5,0 L50,10 L42.5,20 L42.5,18.3 L0,18.3 L0,1.7"/> </g> <g fill="none" stroke="#000000" stroke-opacity="1" stroke-width="1" stroke-linecap="square" stroke-linejoin="bevel" transform="matrix(1,0,0,1,176.132,1638)" font-family="MS Shell Dlg 2" font-size="9.75" font-weight="400" font-style="normal" > <text fill="#000000" fill-opacity="1" stroke="none" xml:space="preserve" x="4" y="17" font-family="Arial" font-size="10pt" font-weight="700" font-style="italic" >C</text> </g> <g fill="#ffff33" fill-opacity="1" stroke="#bfbfbf" stroke-opacity="1" stroke-width="1" stroke-linecap="square" stroke-linejoin="bevel" transform="matrix(1,0,0,1,214,1640)" font-family="MS Shell Dlg 2" font-size="9.75" font-weight="400" font-style="normal" > <rect x="0" y="0" width="50" height="20"/> </g> <g fill="none" stroke="#000000" stroke-opacity="1" stroke-width="1" stroke-linecap="square" stroke-linejoin="bevel" transform="matrix(1,0,0,1,221,1638)" font-family="MS Shell Dlg 2" font-size="9.75" font-weight="400" font-style="normal" > <text fill="#000000" fill-opacity="1" stroke="none" xml:space="preserve" x="4" y="17" font-family="Arial" font-size="10pt" font-weight="700" font-style="italic" >nad3</text> </g> <g fill="#ff9999" fill-opacity="1" stroke="#bfbfbf" stroke-opacity="1" stroke-width="1" stroke-linecap="square" stroke-linejoin="bevel" transform="matrix(1,0,0,1,265,1640)" font-family="MS Shell Dlg 2" font-size="9.75" font-weight="400" font-style="normal" > <path vector-effect="none" fill-rule="evenodd" d="M0,1.7 L42.5,1.7 L42.5,0 L50,10 L42.5,20 L42.5,18.3 L0,18.3 L0,1.7"/> </g> <g fill="none" stroke="#000000" stroke-opacity="1" stroke-width="1" stroke-linecap="square" stroke-linejoin="bevel" transform="matrix(1,0,0,1,275.632,1638)" font-family="MS Shell Dlg 2" font-size="9.75" font-weight="400" font-style="normal" > <text fill="#000000" fill-opacity="1" stroke="none" xml:space="preserve" x="4" y="17" font-family="Arial" font-size="10pt" font-weight="700" font-style="italic" >L2</text> </g> <g fill="#ff9999" fill-opacity="1" stroke="#bfbfbf" stroke-opacity="1" stroke-width="1" stroke-linecap="square" stroke-linejoin="bevel" transform="matrix(1,0,0,1,316,1640)" font-family="MS Shell Dlg 2" font-size="9.75" font-weight="400" font-style="normal" > <path vector-effect="none" fill-rule="evenodd" d="M0,1.7 L42.5,1.7 L42.5,0 L50,10 L42.5,20 L42.5,18.3 L0,18.3 L0,1.7"/> </g> <g fill="none" stroke="#000000" stroke-opacity="1" stroke-width="1" stroke-linecap="square" stroke-linejoin="bevel" transform="matrix(1,0,0,1,327.132,1638)" font-family="MS Shell Dlg 2" font-size="9.75" font-weight="400" font-style="normal" > <text fill="#000000" fill-opacity="1" stroke="none" xml:space="preserve" x="4" y="17" font-family="Arial" font-size="10pt" font-weight="700" font-style="italic" >W</text> </g> <g fill="#ffff33" fill-opacity="1" stroke="#bfbfbf" stroke-opacity="1" stroke-width="1" stroke-linecap="square" stroke-linejoin="bevel" transform="matrix(1,0,0,1,367,1640)" font-family="MS Shell Dlg 2" font-size="9.75" font-weight="400" font-style="normal" > <rect x="0" y="0" width="50" height="20"/> </g> <g fill="none" stroke="#000000" stroke-opacity="1" stroke-width="1" stroke-linecap="square" stroke-linejoin="bevel" transform="matrix(1,0,0,1,375.5,1638)" font-family="MS Shell Dlg 2"

font-size="9.75" font-weight="400" font-style="normal" > <text fill="#000000" fill-opacity="1" stroke="none" xml:space="preserve" x="4" y="17" font-family="Arial" font-size="10pt" font-weight="700" font-style="italic" >atp8</text> </g> <g fill="#ff9999" fill-opacity="1" stroke="#bfbfbf" stroke-opacity="1" stroke-width="1" stroke-linecap="square" stroke-linejoin="bevel" transform="matrix(1,0,0,1,418,1640)" font-family="MS Shell Dlg 2" font-size="9.75" font-weight="400" font-style="normal" > <path vector-effect="none" fill-rule="evenodd" d="M0,1.7 L42.5,1.7 L42.5,0 L50,10 L42.5,20 L42.5,18.3 L0,18.3 L0,1.7"/> </g> <g fill="none" stroke="#000000" stroke-opacity="1" stroke-width="1" stroke-linecap="square" stroke-linejoin="bevel" transform="matrix(1,0,0,1,428.632,1638)" font-family="MS Shell Dlg 2" font-size="9.75" font-weight="400" font-style="normal" > <text fill="#000000" fill-opacity="1" stroke="none" xml:space="preserve" x="4" y="17" font-family="Arial" font-size="10pt" font-weight="700" font-style="italic" >L1</text> </g> <g fill="#ff9999" fill-opacity="1" stroke="#bfbfbf" stroke-opacity="1" stroke-width="1" stroke-linecap="square" stroke-linejoin="bevel" transform="matrix(1,0,0,1,469,1640)" font-family="MS Shell Dlg 2" font-size="9.75" font-weight="400" font-style="normal" > <path vector-effect="none" fill-rule="evenodd" d="M0,1.7 L42.5,1.7 L42.5,0 L50,10 L42.5,20 L42.5,18.3 L0,18.3 L0,1.7"/> </g> <g fill="none" stroke="#000000" stroke-opacity="1" stroke-width="1" stroke-linecap="square" stroke-linejoin="bevel" transform="matrix(1,0,0,1,482.632,1638)" font-family="MS Shell Dlg 2" font-size="9.75" font-weight="400" font-style="normal" > <text fill="#000000" fill-opacity="1" stroke="none" xml:space="preserve" x="4" y="17" font-family="Arial" font-size="10pt" font-weight="700" font-style="italic" >K</text> </g> <g fill="#99ffff" fill-opacity="1" stroke="#bfbfbf" stroke-opacity="1" stroke-width="1" stroke-linecap="square" stroke-linejoin="bevel" transform="matrix(1,0,0,1,520,1640)" font-family="MS Shell Dlg 2" font-size="9.75" font-weight="400" font-style="normal" > <path vector-effect="none" fill-rule="evenodd" d="M0,5 C0,2.23858 2.23858,0 5,0 L45,0 C47.7614,0 50,2.23858 50,5 L50,15 C50,17.7614 47.7614,20 45,20 L5,20 C2.23858,20 0,17.7614 0,15 L0,5"/> </g> <g fill="none" stroke="#000000" stroke-opacity="1" stroke-width="1" stroke-linecap="square" stroke-linejoin="bevel" transform="matrix(1,0,0,1,528.5,1638)" font-family="MS Shell Dlg 2" font-size="9.75" font-weight="400" font-style="normal" > <text fill="#000000" fill-opacity="1" stroke="none" xml:space="preserve" x="4" y="17" font-family="Arial" font-size="10pt" font-weight="700" font-style="italic" >rrnS</text> </g> <g fill="#ff9999" fill-opacity="1" stroke="#bfbfbf" stroke-opacity="1" stroke-width="1" stroke-linecap="square" stroke-linejoin="bevel" transform="matrix(1,0,0,1,571,1640)" font-family="MS Shell Dlg 2" font-size="9.75" font-weight="400" font-style="normal" > <path vector-effect="none" fill-rule="evenodd" d="M0,1.7 L42.5,1.7 L42.5,0 L50,10 L42.5,20 L42.5,18.3 L0,18.3 L0,1.7"/> </g> <g fill="none" stroke="#000000" stroke-opacity="1" stroke-width="1" stroke-linecap="square" stroke-linejoin="bevel" transform="matrix(1,0,0,1,583.633,1638)" font-family="MS Shell Dlg 2" font-size="9.75" font-weight="400" font-style="normal" > <text fill="#000000" fill-opacity="1" stroke="none" xml:space="preserve" x="4" y="17" font-family="Arial" font-size="10pt" font-weight="700" font-style="italic" >Q</text> </g> <g fill="#ff9999" fill-opacity="1" stroke="#bfbfbf" stroke-opacity="1" stroke-width="1" stroke-linecap="square" stroke-linejoin="bevel" transform="matrix(1,0,0,1,622,1640)" font-family="MS Shell Dlg 2" font-size="9.75" font-weight="400" font-style="normal" > <path vector-effect="none" fill-rule="evenodd" d="M0,1.7 L42.5,1.7 L42.5,0 L50,10 L42.5,20 L42.5,18.3 L0,18.3 L0,1.7"/> </g> <g fill="none" stroke="#000000" stroke-opacity="1" stroke-width="1" stroke-linecap="square" stroke-linejoin="bevel" transform="matrix(1,0,0,1,635.633,1638)" font-family="MS Shell Dlg 2" font-size="9.75" font-weight="400" font-style="normal" > <text fill="#000000" fill-opacity="1" stroke="none" xml:space="preserve" x="4" y="17" font-family="Arial" font-size="10pt" font-weight="700" font-style="italic" >Y</text> </g> <g fill="#ffff33" fill-opacity="1" stroke="#bfbfbf" stroke-opacity="1" stroke-width="1" stroke-linecap="square" stroke-linejoin="bevel" transform="matrix(1,0,0,1,673,1640)" font-family="MS Shell Dlg 2" font-size="9.75" font-weight="400" font-style="normal" > <rect x="0" y="0" width="50" height="20"/> </g> <g fill="none" stroke="#000000" stroke-opacity="1" stroke-width="1" stroke-linecap="square" stroke-linejoin="bevel" transform="matrix(1,0,0,1,681.5,1638)" font-family="MS Shell Dlg 2" font-size="9.75" font-weight="400" font-style="normal" > <text fill="#000000" fill-opacity="1" stroke="none" xml:space="preserve" x="4" y="17" font-family="Arial" font-size="10pt" font-weight="700" font-style="italic" >atp6</text> </g> <g fill="#ff9999" fill-opacity="1" stroke="#bfbfbf" stroke-opacity="1" stroke-width="1" stroke-linecap="square" stroke-linejoin="bevel" transform="matrix(1,0,0,1,724,1640)" font-family="MS Shell Dlg 2" font-size="9.75" font-weight="400" font-style="normal" > <path vector-effect="none" fill-rule="evenodd" d="M0,1.7 L42.5,1.7 L42.5,0 L50,10 L42.5,20 L42.5,18.3 L0,18.3 L0,1.7"/> </g> <g fill="none" stroke="#000000" stroke-opacity="1" stroke-width="1" stroke-linecap="square" stroke-linejoin="bevel" transform="matrix(1,0,0,1,737.133,1638)" font-family="MS Shell Dlg 2" font-size="9.75" font-weight="400" font-style="normal" > <text fill="#000000" fill-opacity="1" stroke="none" xml:space="preserve" x="4" y="17" font-family="Arial" font-size="10pt" font-weight="700" font-style="italic" >R</text> </g> <g fill="#ff9999" fill-opacity="1" stroke="#bfbfbf" stroke-opacity="1" stroke-width="1" stroke-linecap="square" stroke-linejoin="bevel" transform="matrix(1,0,0,1,775,1640)" font-family="MS Shell Dlg 2" font-size="9.75" font-

weight="400" font-style="normal" > <path vector-effect="none" fill-rule="evenodd" d="M0,1.7 L42.5,1.7 L42.5,0
L50,10 L42.5,20 L42.5,18.3 L0,18.3 L0,1.7"/> </g> <g fill="none" stroke="#000000" stroke-opacity="1"
stroke-width="1" stroke-linecap="square" stroke-linejoin="bevel" transform="matrix(1,0,0,1,787.133,1638)" font-
family="MS Shell Dlg 2" font-size="9.75" font-weight="400" font-style="normal" > <text fill="#000000" fill-
opacity="1" stroke="none" xml:space="preserve" x="4" y="17" font-family="Arial" font-size="10pt" font-
weight="700" font-style="italic" >M</text> </g> <g fill="#ff9999" fill-opacity="1" stroke="#bfbfbf"
stroke-opacity="1" stroke-width="1" stroke-linecap="square" stroke-linejoin="bevel"
transform="matrix(1,0,0,1,826,1640)" font-family="MS Shell Dlg 2" font-size="9.75" font-weight="400" font-
style="normal" > <path vector-effect="none" fill-rule="evenodd" d="M0,1.7 L42.5,1.7 L42.5,0 L50,10 L42.5,20
L42.5,18.3 L0,18.3 L0,1.7"/> </g> <g fill="none" stroke="#000000" stroke-opacity="1" stroke-width="1"
stroke-linecap="square" stroke-linejoin="bevel" transform="matrix(1,0,0,1,839.133,1638)" font-family="MS Shell
Dlg 2" font-size="9.75" font-weight="400" font-style="normal" > <text fill="#000000" fill-opacity="1"
stroke="none" xml:space="preserve" x="4" y="17" font-family="Arial" font-size="10pt" font-weight="700" font-
style="italic" >V</text> </g> <g fill="#ffff33" fill-opacity="1" stroke="#bfbfbf" stroke-opacity="1"
stroke-width="1" stroke-linecap="square" stroke-linejoin="bevel" transform="matrix(1,0,0,1,877,1640)" font-
family="MS Shell Dlg 2" font-size="9.75" font-weight="400" font-style="normal" > <rect x="0" y="0" width="50"
height="20"/> </g> <g fill="none" stroke="#000000" stroke-opacity="1" stroke-width="1" stroke-
linecap="square" stroke-linejoin="bevel" transform="matrix(1,0,0,1,884,1638)" font-family="MS Shell Dlg 2" font-
size="9.75" font-weight="400" font-style="normal" > <text fill="#000000" fill-opacity="1" stroke="none"
xml:space="preserve" x="4" y="17" font-family="Arial" font-size="10pt" font-weight="700" font-style="italic"
>nad4</text> </g> <g fill="#ff9999" fill-opacity="1" stroke="#bfbfbf" stroke-opacity="1" stroke-width="1"
stroke-linecap="square" stroke-linejoin="bevel" transform="matrix(1,0,0,1,928,1640)" font-family="MS Shell Dlg
2" font-size="9.75" font-weight="400" font-style="normal" > <path vector-effect="none" fill-rule="evenodd"
d="M0,1.7 L42.5,1.7 L42.5,0 L50,10 L42.5,20 L42.5,18.3 L0,18.3 L0,1.7"/> </g> <g fill="none"
stroke="#000000" stroke-opacity="1" stroke-width="1" stroke-linecap="square" stroke-linejoin="bevel"
transform="matrix(1,0,0,1,941.133,1638)" font-family="MS Shell Dlg 2" font-size="9.75" font-weight="400" font-
style="normal" > <text fill="#000000" fill-opacity="1" stroke="none" xml:space="preserve" x="4" y="17" font-
family="Arial" font-size="10pt" font-weight="700" font-style="italic" >N</text> </g> <g fill="#ffff33"
fill-opacity="1" stroke="#bfbfbf" stroke-opacity="1" stroke-width="1" stroke-linecap="square" stroke-
linejoin="bevel" transform="matrix(1,0,0,1,979,1640)" font-family="MS Shell Dlg 2" font-size="9.75" font-
weight="400" font-style="normal" > <rect x="0" y="0" width="50" height="20"/> </g> <g fill="none"
stroke="#000000" stroke-opacity="1" stroke-width="1" stroke-linecap="square" stroke-linejoin="bevel"
transform="matrix(1,0,0,1,987,1638)" font-family="MS Shell Dlg 2" font-size="9.75" font-weight="400" font-
style="normal" > <text fill="#000000" fill-opacity="1" stroke="none" xml:space="preserve" x="4" y="17" font-
family="Arial" font-size="10pt" font-weight="700" font-style="italic" >cox3</text> </g> <g
fill="#ff9999" fill-opacity="1" stroke="#bfbfbf" stroke-opacity="1" stroke-width="1" stroke-linecap="square"
stroke-linejoin="bevel" transform="matrix(1,0,0,1,1030,1640)" font-family="MS Shell Dlg 2" font-size="9.75"
font-weight="400" font-style="normal" > <path vector-effect="none" fill-rule="evenodd" d="M0,1.7 L42.5,1.7
L42.5,0 L50,10 L42.5,20 L42.5,18.3 L0,18.3 L0,1.7"/> </g> <g fill="none" stroke="#000000" stroke-
opacity="1" stroke-width="1" stroke-linecap="square" stroke-linejoin="bevel"
transform="matrix(1,0,0,1,1043.63,1638)" font-family="MS Shell Dlg 2" font-size="9.75" font-weight="400" font-
style="normal" > <text fill="#000000" fill-opacity="1" stroke="none" xml:space="preserve" x="4" y="17" font-
family="Arial" font-size="10pt" font-weight="700" font-style="italic" >F</text> </g> <g fill="#ff9999"
fill-opacity="1" stroke="#bfbfbf" stroke-opacity="1" stroke-width="1" stroke-linecap="square" stroke-
linejoin="bevel" transform="matrix(1,0,0,1,1081,1640)" font-family="MS Shell Dlg 2" font-size="9.75" font-
weight="400" font-style="normal" > <path vector-effect="none" fill-rule="evenodd" d="M0,1.7 L42.5,1.7 L42.5,0
L50,10 L42.5,20 L42.5,18.3 L0,18.3 L0,1.7"/> </g> <g fill="none" stroke="#000000" stroke-opacity="1"
stroke-width="1" stroke-linecap="square" stroke-linejoin="bevel" transform="matrix(1,0,0,1,1090.63,1638)" font-
family="MS Shell Dlg 2" font-size="9.75" font-weight="400" font-style="normal" > <text fill="#000000" fill-
opacity="1" stroke="none" xml:space="preserve" x="4" y="17" font-family="Arial" font-size="10pt" font-
weight="700" font-style="italic" >S1</text> </g> <g fill="#99ffff" fill-opacity="1" stroke="#bfbfbf"
stroke-opacity="1" stroke-width="1" stroke-linecap="square" stroke-linejoin="bevel"
transform="matrix(1,0,0,1,1132,1640)" font-family="MS Shell Dlg 2" font-size="9.75" font-weight="400" font-
style="normal" > <path vector-effect="none" fill-rule="evenodd" d="M0,5 C0,2.23858 2.23858,0 5,0 L45,0
C47.7614,0 50,2.23858 50,5 L50,15 C50,17.7614 47.7614,20 45,20 L5,20 C2.23858,20 0,17.7614 0,15 L0,5"/>
</g> <g fill="none" stroke="#000000" stroke-opacity="1" stroke-width="1" stroke-linecap="square" stroke-
linejoin="bevel" transform="matrix(1,0,0,1,1141.5,1638)" font-family="MS Shell Dlg 2" font-size="9.75" font-
weight="400" font-style="normal" > <text fill="#000000" fill-opacity="1" stroke="none" xml:space="preserve"

x="4" y="17" font-family="Arial" font-size="10pt" font-weight="700" font-style="italic" >rrnL</text> </g> <g fill="#ff9999" fill-opacity="1" stroke="#bfbfbf" stroke-opacity="1" stroke-width="1" stroke-linecap="square" stroke-linejoin="bevel" transform="matrix(1,0,0,1,1183,1640)" font-family="MS Shell Dlg 2" font-size="9.75" font-weight="400" font-style="normal" > <path vector-effect="none" fill-rule="evenodd" d="M0,1.7 L42.5,1.7 L42.5,0 L50,10 L42.5,20 L42.5,18.3 L0,18.3 L0,1.7"/> </g> <g fill="none" stroke="#000000" stroke-opacity="1" stroke-width="1" stroke-linecap="square" stroke-linejoin="bevel" transform="matrix(1,0,0,1,1192.63,1638)" font-family="MS Shell Dlg 2" font-size="9.75" font-weight="400" font-style="normal" > <text fill="#000000" fill-opacity="1" stroke="none" xml:space="preserve" x="4" y="17" font-family="Arial" font-size="10pt" font-weight="700" font-style="italic" >S2</text> </g> <g fill="#ff9999" fill-opacity="1" stroke="#bfbfbf" stroke-opacity="1" stroke-width="1" stroke-linecap="square" stroke-linejoin="bevel" transform="matrix(1,0,0,1,1234,1640)" font-family="MS Shell Dlg 2" font-size="9.75" font-weight="400" font-style="normal" > <path vector-effect="none" fill-rule="evenodd" d="M0,1.7 L42.5,1.7 L42.5,0 L50,10 L42.5,20 L42.5,18.3 L0,18.3 L0,1.7"/> </g> <g fill="none" stroke="#000000" stroke-opacity="1" stroke-width="1" stroke-linecap="square" stroke-linejoin="bevel" transform="matrix(1,0,0,1,1247.13,1638)" font-family="MS Shell Dlg 2" font-size="9.75" font-weight="400" font-style="normal" > <text fill="#000000" fill-opacity="1" stroke="none" xml:space="preserve" x="4" y="17" font-family="Arial" font-size="10pt" font-weight="700" font-style="italic" >D</text> </g> <g fill="#ffff33" fill-opacity="1" stroke="#bfbfbf" stroke-opacity="1" stroke-width="1" stroke-linecap="square" stroke-linejoin="bevel" transform="matrix(1,0,0,1,1285,1640)" font-family="MS Shell Dlg 2" font-size="9.75" font-weight="400" font-style="normal" > <rect x="0" y="0" width="50" height="20"/> </g> <g fill="none" stroke="#000000" stroke-opacity="1" stroke-width="1" stroke-linecap="square" stroke-linejoin="bevel" transform="matrix(1,0,0,1,1292,1638)" font-family="MS Shell Dlg 2" font-size="9.75" font-weight="400" font-style="normal" > <text fill="#000000" fill-opacity="1" stroke="none" xml:space="preserve" x="4" y="17" font-family="Arial" font-size="10pt" font-weight="700" font-style="italic" >nad6</text> </g> <g fill="#ff9999" fill-opacity="1" stroke="#bfbfbf" stroke-opacity="1" stroke-width="1" stroke-linecap="square" stroke-linejoin="bevel" transform="matrix(1,0,0,1,1336,1640)" font-family="MS Shell Dlg 2" font-size="9.75" font-weight="400" font-style="normal" > <path vector-effect="none" fill-rule="evenodd" d="M0,1.7 L42.5,1.7 L42.5,0 L50,10 L42.5,20 L42.5,18.3 L0,18.3 L0,1.7"/> </g> <g fill="none" stroke="#000000" stroke-opacity="1" stroke-width="1" stroke-linecap="square" stroke-linejoin="bevel" transform="matrix(1,0,0,1,1351.13,1638)" font-family="MS Shell Dlg 2" font-size="9.75" font-weight="400" font-style="normal" > <text fill="#000000" fill-opacity="1" stroke="none" xml:space="preserve" x="4" y="17" font-family="Arial" font-size="10pt" font-weight="700" font-style="italic" >I</text> </g> <g fill="#ffff33" fill-opacity="1" stroke="#bfbfbf" stroke-opacity="1" stroke-width="1" stroke-linecap="square" stroke-linejoin="bevel" transform="matrix(1,0,0,1,1387,1640)" font-family="MS Shell Dlg 2" font-size="9.75" font-weight="400" font-style="normal" > <rect x="0" y="0" width="50" height="20"/> </g> <g fill="none" stroke="#000000" stroke-opacity="1" stroke-width="1" stroke-linecap="square" stroke-linejoin="bevel" transform="matrix(1,0,0,1,1394,1638)" font-family="MS Shell Dlg 2" font-size="9.75" font-weight="400" font-style="normal" > <text fill="#000000" fill-opacity="1" stroke="none" xml:space="preserve" x="4" y="17" font-family="Arial" font-size="10pt" font-weight="700" font-style="italic" >nad2</text> </g> <g fill="#ff9999" fill-opacity="1" stroke="#bfbfbf" stroke-opacity="1" stroke-width="1" stroke-linecap="square" stroke-linejoin="bevel" transform="matrix(1,0,0,1,1438,1640)" font-family="MS Shell Dlg 2" font-size="9.75" font-weight="400" font-style="normal" > <path vector-effect="none" fill-rule="evenodd" d="M0,1.7 L42.5,1.7 L42.5,0 L50,10 L42.5,20 L42.5,18.3 L0,18.3 L0,1.7"/> </g> <g fill="none" stroke="#000000" stroke-opacity="1" stroke-width="1" stroke-linecap="square" stroke-linejoin="bevel" transform="matrix(1,0,0,1,1449.13,1638)" font-family="MS Shell Dlg 2" font-size="9.75" font-weight="400" font-style="normal" > <text fill="#000000" fill-opacity="1" stroke="none" xml:space="preserve" x="4" y="17" font-family="Arial" font-size="10pt" font-weight="700" font-style="italic" >W</text> </g> <g fill="#ff9999" fill-opacity="1" stroke="#bfbfbf" stroke-opacity="1" stroke-width="1" stroke-linecap="square" stroke-linejoin="bevel" transform="matrix(1,0,0,1,1489,1640)" font-family="MS Shell Dlg 2" font-size="9.75" font-weight="400" font-style="normal" > <path vector-effect="none" fill-rule="evenodd" d="M0,1.7 L42.5,1.7 L42.5,0 L50,10 L42.5,20 L42.5,18.3 L0,18.3 L0,1.7"/> </g> <g fill="none" stroke="#000000" stroke-opacity="1" stroke-width="1" stroke-linecap="square" stroke-linejoin="bevel" transform="matrix(1,0,0,1,1502.13,1638)" font-family="MS Shell Dlg 2" font-size="9.75" font-weight="400" font-style="normal" > <text fill="#000000" fill-opacity="1" stroke="none" xml:space="preserve" x="4" y="17" font-family="Arial" font-size="10pt" font-weight="700" font-style="italic" >G</text> </g> <g fill="#ff9999" fill-opacity="1" stroke="#bfbfbf" stroke-opacity="1" stroke-width="1" stroke-linecap="square" stroke-linejoin="bevel" transform="matrix(1,0,0,1,1540,1640)" font-family="MS Shell Dlg 2" font-size="9.75" font-weight="400" font-style="normal" > <path vector-effect="none" fill-rule="evenodd" d="M0,1.7 L42.5,1.7 L42.5,0 L50,10 L42.5,20

L42.5,18.3 L0,18.3 L0,1.7"/> </g> <g fill="none" stroke="#000000" stroke-opacity="1" stroke-width="1" stroke-linecap="square" stroke-linejoin="bevel" transform="matrix(1,0,0,1,1553.13,1638)" font-family="MS Shell Dlg 2" font-size="9.75" font-weight="400" font-style="normal" > <text fill="#000000" fill-opacity="1" stroke="none" xml:space="preserve" x="4" y="17" font-family="Arial" font-size="10pt" font-weight="700" font-style="italic" >G</text> </g> <g fill="#ff9999" fill-opacity="1" stroke="#bfbfbf" stroke-opacity="1" stroke-width="1" stroke-linecap="square" stroke-linejoin="bevel" transform="matrix(1,0,0,1,1591,1660)" font-family="MS Shell Dlg 2" font-size="9.75" font-weight="400" font-style="normal" > <path vector-effect="none" fill-rule="evenodd" d="M0,10 L7.5,0 L7.5,1.7 L50,1.7 L50,18.3 L7.5,18.3 L7.5,20 L0,10"/> </g> <g fill="none" stroke="#000000" stroke-opacity="1" stroke-width="1" stroke-linecap="square" stroke-linejoin="bevel" transform="matrix(1,0,0,1,1611.87,1658)" font-family="MS Shell Dlg 2" font-size="9.75" font-weight="400" font-style="normal" > <text fill="#000000" fill-opacity="1" stroke="none" xml:space="preserve" x="4" y="17" font-family="Arial" font-size="10pt" font-weight="700" font-style="italic" >T</text> </g> <g fill="#ffff33" fill-opacity="1" stroke="#bfbfbf" stroke-opacity="1" stroke-width="1" stroke-linecap="square" stroke-linejoin="bevel" transform="matrix(1,0,0,1,1642,1640)" font-family="MS Shell Dlg 2" font-size="9.75" font-weight="400" font-style="normal" > <rect x="0" y="0" width="50" height="20"/> </g> <g fill="none" stroke="#000000" stroke-opacity="1" stroke-width="1" stroke-linecap="square" stroke-linejoin="bevel" transform="matrix(1,0,0,1,1651.5,1638)" font-family="MS Shell Dlg 2" font-size="9.75" font-weight="400" font-style="normal" > <text fill="#000000" fill-opacity="1" stroke="none" xml:space="preserve" x="4" y="17" font-family="Arial" font-size="10pt" font-weight="700" font-style="italic" >cytb</text> </g> <g fill="#ffff33" fill-opacity="1" stroke="#bfbfbf" stroke-opacity="1" stroke-width="1" stroke-linecap="square" stroke-linejoin="bevel" transform="matrix(1,0,0,1,1693,1640)" font-family="MS Shell Dlg 2" font-size="9.75" font-weight="400" font-style="normal" > <rect x="0" y="0" width="50" height="20"/> </g> <g fill="none" stroke="#000000" stroke-opacity="1" stroke-width="1" stroke-linecap="square" stroke-linejoin="bevel" transform="matrix(1,0,0,1,1697,1638)" font-family="MS Shell Dlg 2" font-size="9.75" font-weight="400" font-style="normal" > <text fill="#000000" fill-opacity="1" stroke="none" xml:space="preserve" x="4" y="17" font-family="Arial" font-size="10pt" font-weight="700" font-style="italic" >nad4L</text> </g> <g fill="#ff9999" fill-opacity="1" stroke="#bfbfbf" stroke-opacity="1" stroke-width="1" stroke-linecap="square" stroke-linejoin="bevel" transform="matrix(1,0,0,1,1744,1640)" font-family="MS Shell Dlg 2" font-size="9.75" font-weight="400" font-style="normal" > <path vector-effect="none" fill-rule="evenodd" d="M0,1.7 L42.5,1.7 L42.5,0 L50,10 L42.5,20 L42.5,18.3 L0,18.3 L0,1.7"/> </g> <g fill="none" stroke="#000000" stroke-opacity="1" stroke-width="1" stroke-linecap="square" stroke-linejoin="bevel" transform="matrix(1,0,0,1,1757.13,1638)" font-family="MS Shell Dlg 2" font-size="9.75" font-weight="400" font-style="normal" > <text fill="#000000" fill-opacity="1" stroke="none" xml:space="preserve" x="4" y="17" font-family="Arial" font-size="10pt" font-weight="700" font-style="italic" >A</text> </g> <g fill="#ff9999" fill-opacity="1" stroke="#bfbfbf" stroke-opacity="1" stroke-width="1" stroke-linecap="square" stroke-linejoin="bevel" transform="matrix(1,0,0,1,1795,1640)" font-family="MS Shell Dlg 2" font-size="9.75" font-weight="400" font-style="normal" > <path vector-effect="none" fill-rule="evenodd" d="M0,1.7 L42.5,1.7 L42.5,0 L50,10 L42.5,20 L42.5,18.3 L0,18.3 L0,1.7"/> </g> <g fill="none" stroke="#000000" stroke-opacity="1" stroke-width="1" stroke-linecap="square" stroke-linejoin="bevel" transform="matrix(1,0,0,1,1808.13,1638)" font-family="MS Shell Dlg 2" font-size="9.75" font-weight="400" font-style="normal" > <text fill="#000000" fill-opacity="1" stroke="none" xml:space="preserve" x="4" y="17" font-family="Arial" font-size="10pt" font-weight="700" font-style="italic" >H</text> </g> <g fill="#ffff33" fill-opacity="1" stroke="#bfbfbf" stroke-opacity="1" stroke-width="1" stroke-linecap="square" stroke-linejoin="bevel" transform="matrix(1,0,0,1,1846,1640)" font-family="MS Shell Dlg 2" font-size="9.75" font-weight="400" font-style="normal" > <rect x="0" y="0" width="50" height="20"/> </g> <g fill="none" stroke="#000000" stroke-opacity="1" stroke-width="1" stroke-linecap="square" stroke-linejoin="bevel" transform="matrix(1,0,0,1,1853,1638)" font-family="MS Shell Dlg 2" font-size="9.75" font-weight="400" font-style="normal" > <text fill="#000000" fill-opacity="1" stroke="none" xml:space="preserve" x="4" y="17" font-family="Arial" font-size="10pt" font-weight="700" font-style="italic" >nad5</text> </g> <g fill="#ffff33" fill-opacity="1" stroke="#bfbfbf" stroke-opacity="1" stroke-width="1" stroke-linecap="square" stroke-linejoin="bevel" transform="matrix(1,0,0,1,1897,1640)" font-family="MS Shell Dlg 2" font-size="9.75" font-weight="400" font-style="normal" > <rect x="0" y="0" width="50" height="20"/> </g> <g fill="none" stroke="#000000" stroke-opacity="1" stroke-width="1" stroke-linecap="square" stroke-linejoin="bevel" transform="matrix(1,0,0,1,1905,1638)" font-family="MS Shell Dlg 2" font-size="9.75" font-weight="400" font-style="normal" > <text fill="#000000" fill-opacity="1" stroke="none" xml:space="preserve" x="4" y="17" font-family="Arial" font-size="10pt" font-weight="700" font-style="italic" >cox2</text> </g> <g fill="#ffff33" fill-opacity="1" stroke="#bfbfbf" stroke-opacity="1" stroke-width="1" stroke-linecap="square" stroke-linejoin="bevel" transform="matrix(1,0,0,1,1948,1640)" font-family="MS Shell Dlg 2" font-size="9.75" font-

weight="400" font-style="normal" > <rect x="0" y="0" width="50" height="20"/> </g> <g fill="none" stroke="#000000" stroke-opacity="1" stroke-width="1" stroke-linecap="square" stroke-linejoin="bevel" transform="matrix(1,0,0,1,1955,1638)" font-family="MS Shell Dlg 2" font-size="9.75" font-weight="400" font-style="normal" > <text fill="#000000" fill-opacity="1" stroke="none" xml:space="preserve" x="4" y="17" font-family="Arial" font-size="10pt" font-weight="700" font-style="italic" >nad1</text> </g> <g fill="none" stroke="#000000" stroke-opacity="1" stroke-width="1" stroke-linecap="square" stroke-linejoin="bevel" transform="matrix(1,0,0,1,1999,1640)" font-family="MS Shell Dlg 2" font-size="9.75" font-weight="400" font-style="normal" > <text fill="#000000" fill-opacity="1" stroke="none" xml:space="preserve" x="4" y="17" font-family="Arial" font-size="10pt" font-weight="700" font-style="italic" >(+)</text> </g> <g fill="none" stroke="#000000" stroke-opacity="1" stroke-width="1" stroke-linecap="square" stroke-linejoin="bevel" transform="matrix(1,0,0,1,1999,1660)" font-family="MS Shell Dlg 2" font-size="9.75" font-weight="400" font-style="normal" > <text fill="#000000" fill-opacity="1" stroke="none" xml:space="preserve" x="4" y="17" font-family="Arial" font-size="10pt" font-weight="700" font-style="italic" >(-)</text> </g> <g fill="none" stroke="#000000" stroke-opacity="1" stroke-width="1" stroke-linecap="square" stroke-linejoin="bevel" transform="matrix(1,0,0,1,10,1700)" font-family="MS Shell Dlg 2" font-size="9.75" font-weight="400" font-style="normal" > <text fill="#000000" fill-opacity="1" stroke="none" xml:space="preserve" x="4" y="17" font-family="Arial" font-size="10pt" font-weight="700" font-style="italic" >Modiolus_modiolus_KX821782</text> </g> <g fill="#ffff33" fill-opacity="1" stroke="#bfbfbf" stroke-opacity="1" stroke-width="1" stroke-linecap="square" stroke-linejoin="bevel" transform="matrix(1,0,0,1,10,1730)" font-family="MS Shell Dlg 2" font-size="9.75" font-weight="400" font-style="normal" > <rect x="0" y="0" width="50" height="20"/> </g> <g fill="none" stroke="#000000" stroke-opacity="1" stroke-width="1" stroke-linecap="square" stroke-linejoin="bevel" transform="matrix(1,0,0,1,18,1728)" font-family="MS Shell Dlg 2" font-size="9.75" font-weight="400" font-style="normal" > <text fill="#000000" fill-opacity="1" stroke="none" xml:space="preserve" x="4" y="17" font-family="Arial" font-size="10pt" font-weight="700" font-style="italic" >cox1</text> </g> <g fill="#ffff33" fill-opacity="1" stroke="#bfbfbf" stroke-opacity="1" stroke-width="1" stroke-linecap="square" stroke-linejoin="bevel" transform="matrix(1,0,0,1,61,1730)" font-family="MS Shell Dlg 2" font-size="9.75" font-weight="400" font-style="normal" > <rect x="0" y="0" width="50" height="20"/> </g> <g fill="none" stroke="#000000" stroke-opacity="1" stroke-width="1" stroke-linecap="square" stroke-linejoin="bevel" transform="matrix(1,0,0,1,68,1728)" font-family="MS Shell Dlg 2" font-size="9.75" font-weight="400" font-style="normal" > <text fill="#000000" fill-opacity="1" stroke="none" xml:space="preserve" x="4" y="17" font-family="Arial" font-size="10pt" font-weight="700" font-style="italic" >nad3</text> </g> <g fill="#ff9999" fill-opacity="1" stroke="#bfbfbf" stroke-opacity="1" stroke-width="1" stroke-linecap="square" stroke-linejoin="bevel" transform="matrix(1,0,0,1,112,1730)" font-family="MS Shell Dlg 2" font-size="9.75" font-weight="400" font-style="normal" > <path vector-effect="none" fill-rule="evenodd" d="M0,1.7 L42.5,1.7 L42.5,0 L50,10 L42.5,20 L42.5,18.3 L0,18.3 L0,1.7"/> </g> <g fill="none" stroke="#000000" stroke-opacity="1" stroke-width="1" stroke-linecap="square" stroke-linejoin="bevel" transform="matrix(1,0,0,1,125.132,1728)" font-family="MS Shell Dlg 2" font-size="9.75" font-weight="400" font-style="normal" > <text fill="#000000" fill-opacity="1" stroke="none" xml:space="preserve" x="4" y="17" font-family="Arial" font-size="10pt" font-weight="700" font-style="italic" >C</text> </g> <g fill="#ff9999" fill-opacity="1" stroke="#bfbfbf" stroke-opacity="1" stroke-width="1" stroke-linecap="square" stroke-linejoin="bevel" transform="matrix(1,0,0,1,163,1730)" font-family="MS Shell Dlg 2" font-size="9.75" font-weight="400" font-style="normal" > <path vector-effect="none" fill-rule="evenodd" d="M0,1.7 L42.5,1.7 L42.5,0 L50,10 L42.5,20 L42.5,18.3 L0,18.3 L0,1.7"/> </g> <g fill="none" stroke="#000000" stroke-opacity="1" stroke-width="1" stroke-linecap="square" stroke-linejoin="bevel" transform="matrix(1,0,0,1,177.132,1728)" font-family="MS Shell Dlg 2" font-size="9.75" font-weight="400" font-style="normal" > <text fill="#000000" fill-opacity="1" stroke="none" xml:space="preserve" x="4" y="17" font-family="Arial" font-size="10pt" font-weight="700" font-style="italic" >L</text> </g> <g fill="#ff9999" fill-opacity="1" stroke="#bfbfbf" stroke-opacity="1" stroke-width="1" stroke-linecap="square" stroke-linejoin="bevel" transform="matrix(1,0,0,1,214,1730)" font-family="MS Shell Dlg 2" font-size="9.75" font-weight="400" font-style="normal" > <path vector-effect="none" fill-rule="evenodd" d="M0,1.7 L42.5,1.7 L42.5,0 L50,10 L42.5,20 L42.5,18.3 L0,18.3 L0,1.7"/> </g> <g fill="none" stroke="#000000" stroke-opacity="1" stroke-width="1" stroke-linecap="square" stroke-linejoin="bevel" transform="matrix(1,0,0,1,227.632,1728)" font-family="MS Shell Dlg 2" font-size="9.75" font-weight="400" font-style="normal" > <text fill="#000000" fill-opacity="1" stroke="none" xml:space="preserve" x="4" y="17" font-family="Arial" font-size="10pt" font-weight="700" font-style="italic" >P</text> </g> <g fill="#ff9999" fill-opacity="1" stroke="#bfbfbf" stroke-opacity="1" stroke-width="1" stroke-linecap="square" stroke-linejoin="bevel" transform="matrix(1,0,0,1,265,1730)" font-family="MS Shell Dlg 2" font-size="9.75" font-weight="400" font-style="normal" > <path vector-effect="none" fill-rule="evenodd" d="M0,1.7 L42.5,1.7 L42.5,0 L50,10 L42.5,20 L42.5,18.3 L0,18.3 L0,1.7"/> </g> <g fill="none" stroke="#000000" stroke-opacity="1"

stroke-width="1" stroke-linecap="square" stroke-linejoin="bevel" transform="matrix(1,0,0,1,278.632,1728)" font-family="MS Shell Dlg 2" font-size="9.75" font-weight="400" font-style="normal" > <text fill="#000000" fill-opacity="1" stroke="none" xml:space="preserve" x="4" y="17" font-family="Arial" font-size="10pt" font-weight="700" font-style="italic" >E</text> </g> <g fill="#ff9999" fill-opacity="1" stroke="#bfbfbf" stroke-opacity="1" stroke-width="1" stroke-linecap="square" stroke-linejoin="bevel" transform="matrix(1,0,0,1,316,1730)" font-family="MS Shell Dlg 2" font-size="9.75" font-weight="400" font-style="normal" > <path vector-effect="none" fill-rule="evenodd" d="M0,1.7 L42.5,1.7 L42.5,0 L50,10 L42.5,20 L42.5,18.3 L0,18.3 L0,1.7"/> </g> <g fill="none" stroke="#000000" stroke-opacity="1" stroke-width="1" stroke-linecap="square" stroke-linejoin="bevel" transform="matrix(1,0,0,1,330.132,1728)" font-family="MS Shell Dlg 2" font-size="9.75" font-weight="400" font-style="normal" > <text fill="#000000" fill-opacity="1" stroke="none" xml:space="preserve" x="4" y="17" font-family="Arial" font-size="10pt" font-weight="700" font-style="italic" >L</text> </g> <g fill="#ff9999" fill-opacity="1" stroke="#bfbfbf" stroke-opacity="1" stroke-width="1" stroke-linecap="square" stroke-linejoin="bevel" transform="matrix(1,0,0,1,367,1730)" font-family="MS Shell Dlg 2" font-size="9.75" font-weight="400" font-style="normal" > <path vector-effect="none" fill-rule="evenodd" d="M0,1.7 L42.5,1.7 L42.5,0 L50,10 L42.5,20 L42.5,18.3 L0,18.3 L0,1.7"/> </g> <g fill="none" stroke="#000000" stroke-opacity="1" stroke-width="1" stroke-linecap="square" stroke-linejoin="bevel" transform="matrix(1,0,0,1,380.132,1728)" font-family="MS Shell Dlg 2" font-size="9.75" font-weight="400" font-style="normal" > <text fill="#000000" fill-opacity="1" stroke="none" xml:space="preserve" x="4" y="17" font-family="Arial" font-size="10pt" font-weight="700" font-style="italic" >R</text> </g> <g fill="#ff9999" fill-opacity="1" stroke="#bfbfbf" stroke-opacity="1" stroke-width="1" stroke-linecap="square" stroke-linejoin="bevel" transform="matrix(1,0,0,1,418,1730)" font-family="MS Shell Dlg 2" font-size="9.75" font-weight="400" font-style="normal" > <path vector-effect="none" fill-rule="evenodd" d="M0,1.7 L42.5,1.7 L42.5,0 L50,10 L42.5,20 L42.5,18.3 L0,18.3 L0,1.7"/> </g> <g fill="none" stroke="#000000" stroke-opacity="1" stroke-width="1" stroke-linecap="square" stroke-linejoin="bevel" transform="matrix(1,0,0,1,431.132,1728)" font-family="MS Shell Dlg 2" font-size="9.75" font-weight="400" font-style="normal" > <text fill="#000000" fill-opacity="1" stroke="none" xml:space="preserve" x="4" y="17" font-family="Arial" font-size="10pt" font-weight="700" font-style="italic" >S</text> </g> <g fill="#ff9999" fill-opacity="1" stroke="#bfbfbf" stroke-opacity="1" stroke-width="1" stroke-linecap="square" stroke-linejoin="bevel" transform="matrix(1,0,0,1,469,1730)" font-family="MS Shell Dlg 2" font-size="9.75" font-weight="400" font-style="normal" > <path vector-effect="none" fill-rule="evenodd" d="M0,1.7 L42.5,1.7 L42.5,0 L50,10 L42.5,20 L42.5,18.3 L0,18.3 L0,1.7"/> </g> <g fill="none" stroke="#000000" stroke-opacity="1" stroke-width="1" stroke-linecap="square" stroke-linejoin="bevel" transform="matrix(1,0,0,1,481.132,1728)" font-family="MS Shell Dlg 2" font-size="9.75" font-weight="400" font-style="normal" > <text fill="#000000" fill-opacity="1" stroke="none" xml:space="preserve" x="4" y="17" font-family="Arial" font-size="10pt" font-weight="700" font-style="italic" >M</text> </g> <g fill="#ff9999" fill-opacity="1" stroke="#bfbfbf" stroke-opacity="1" stroke-width="1" stroke-linecap="square" stroke-linejoin="bevel" transform="matrix(1,0,0,1,520,1730)" font-family="MS Shell Dlg 2" font-size="9.75" font-weight="400" font-style="normal" > <path vector-effect="none" fill-rule="evenodd" d="M0,1.7 L42.5,1.7 L42.5,0 L50,10 L42.5,20 L42.5,18.3 L0,18.3 L0,1.7"/> </g> <g fill="none" stroke="#000000" stroke-opacity="1" stroke-width="1" stroke-linecap="square" stroke-linejoin="bevel" transform="matrix(1,0,0,1,533.633,1728)" font-family="MS Shell Dlg 2" font-size="9.75" font-weight="400" font-style="normal" > <text fill="#000000" fill-opacity="1" stroke="none" xml:space="preserve" x="4" y="17" font-family="Arial" font-size="10pt" font-weight="700" font-style="italic" >K</text> </g> <g fill="#99ffff" fill-opacity="1" stroke="#bfbfbf" stroke-opacity="1" stroke-width="1" stroke-linecap="square" stroke-linejoin="bevel" transform="matrix(1,0,0,1,571,1730)" font-family="MS Shell Dlg 2" font-size="9.75" font-weight="400" font-style="normal" > <path vector-effect="none" fill-rule="evenodd" d="M0,5 C0,2.23858 2.23858,0 5,0 L45,0 C47.7614,0 50,2.23858 50,5 L50,15 C50,17.7614 47.7614,20 45,20 L5,20 C2.23858,20 0,17.7614 0,15 L0,5"/> </g> <g fill="none" stroke="#000000" stroke-opacity="1" stroke-width="1" stroke-linecap="square" stroke-linejoin="bevel" transform="matrix(1,0,0,1,579.5,1728)" font-family="MS Shell Dlg 2" font-size="9.75" font-weight="400" font-style="normal" > <text fill="#000000" fill-opacity="1" stroke="none" xml:space="preserve" x="4" y="17" font-family="Arial" font-size="10pt" font-weight="700" font-style="italic" >rrnS</text> </g> <g fill="#ff9999" fill-opacity="1" stroke="#bfbfbf" stroke-opacity="1" stroke-width="1" stroke-linecap="square" stroke-linejoin="bevel" transform="matrix(1,0,0,1,622,1730)" font-family="MS Shell Dlg 2" font-size="9.75" font-weight="400" font-style="normal" > <path vector-effect="none" fill-rule="evenodd" d="M0,1.7 L42.5,1.7 L42.5,0 L50,10 L42.5,20 L42.5,18.3 L0,18.3 L0,1.7"/> </g> <g fill="none" stroke="#000000" stroke-opacity="1" stroke-width="1" stroke-linecap="square" stroke-linejoin="bevel" transform="matrix(1,0,0,1,634.633,1728)" font-family="MS Shell Dlg 2" font-size="9.75" font-weight="400" font-style="normal" > <text fill="#000000" fill-opacity="1" stroke="none" xml:space="preserve" x="4" y="17" font-family="Arial" font-size="10pt" font-weight="700" font-style="italic" >Q</text> </g> <g fill="#ffff33"

fill-opacity="1" stroke="#bfbfbf" stroke-opacity="1" stroke-width="1" stroke-linecap="square" stroke-linejoin="bevel" transform="matrix(1,0,0,1,673,1730)" font-family="MS Shell Dlg 2" font-size="9.75" font-weight="400" font-style="normal" > <rect x="0" y="0" width="50" height="20"/> </g> <g fill="none" stroke="#000000" stroke-opacity="1" stroke-width="1" stroke-linecap="square" stroke-linejoin="bevel" transform="matrix(1,0,0,1,681.5,1728)" font-family="MS Shell Dlg 2" font-size="9.75" font-weight="400" font-style="normal" > <text fill="#000000" fill-opacity="1" stroke="none" xml:space="preserve" x="4" y="17" font-family="Arial" font-size="10pt" font-weight="700" font-style="italic" >atp6</text> </g> <g fill="#ff9999" fill-opacity="1" stroke="#bfbfbf" stroke-opacity="1" stroke-width="1" stroke-linecap="square" stroke-linejoin="bevel" transform="matrix(1,0,0,1,724,1730)" font-family="MS Shell Dlg 2" font-size="9.75" font-weight="400" font-style="normal" > <path vector-effect="none" fill-rule="evenodd" d="M0,1.7 L42.5,1.7 L42.5,0 L50,10 L42.5,20 L42.5,18.3 L0,18.3 L0,1.7"/> </g> <g fill="none" stroke="#000000" stroke-opacity="1" stroke-width="1" stroke-linecap="square" stroke-linejoin="bevel" transform="matrix(1,0,0,1,737.633,1728)" font-family="MS Shell Dlg 2" font-size="9.75" font-weight="400" font-style="normal" > <text fill="#000000" fill-opacity="1" stroke="none" xml:space="preserve" x="4" y="17" font-family="Arial" font-size="10pt" font-weight="700" font-style="italic" >Y</text> </g> <g fill="#ff9999" fill-opacity="1" stroke="#bfbfbf" stroke-opacity="1" stroke-width="1" stroke-linecap="square" stroke-linejoin="bevel" transform="matrix(1,0,0,1,775,1730)" font-family="MS Shell Dlg 2" font-size="9.75" font-weight="400" font-style="normal" > <path vector-effect="none" fill-rule="evenodd" d="M0,1.7 L42.5,1.7 L42.5,0 L50,10 L42.5,20 L42.5,18.3 L0,18.3 L0,1.7"/> </g> <g fill="none" stroke="#000000" stroke-opacity="1" stroke-width="1" stroke-linecap="square" stroke-linejoin="bevel" transform="matrix(1,0,0,1,788.133,1728)" font-family="MS Shell Dlg 2" font-size="9.75" font-weight="400" font-style="normal" > <text fill="#000000" fill-opacity="1" stroke="none" xml:space="preserve" x="4" y="17" font-family="Arial" font-size="10pt" font-weight="700" font-style="italic" >V</text> </g> <g fill="#ffff33" fill-opacity="1" stroke="#bfbfbf" stroke-opacity="1" stroke-width="1" stroke-linecap="square" stroke-linejoin="bevel" transform="matrix(1,0,0,1,826,1730)" font-family="MS Shell Dlg 2" font-size="9.75" font-weight="400" font-style="normal" > <rect x="0" y="0" width="50" height="20"/> </g> <g fill="none" stroke="#000000" stroke-opacity="1" stroke-width="1" stroke-linecap="square" stroke-linejoin="bevel" transform="matrix(1,0,0,1,833,1728)" font-family="MS Shell Dlg 2" font-size="9.75" font-weight="400" font-style="normal" > <text fill="#000000" fill-opacity="1" stroke="none" xml:space="preserve" x="4" y="17" font-family="Arial" font-size="10pt" font-weight="700" font-style="italic" >nad4</text> </g> <g fill="#ff9999" fill-opacity="1" stroke="#bfbfbf" stroke-opacity="1" stroke-width="1" stroke-linecap="square" stroke-linejoin="bevel" transform="matrix(1,0,0,1,877,1730)" font-family="MS Shell Dlg 2" font-size="9.75" font-weight="400" font-style="normal" > <path vector-effect="none" fill-rule="evenodd" d="M0,1.7 L42.5,1.7 L42.5,0 L50,10 L42.5,20 L42.5,18.3 L0,18.3 L0,1.7"/> </g> <g fill="none" stroke="#000000" stroke-opacity="1" stroke-width="1" stroke-linecap="square" stroke-linejoin="bevel" transform="matrix(1,0,0,1,890.133,1728)" font-family="MS Shell Dlg 2" font-size="9.75" font-weight="400" font-style="normal" > <text fill="#000000" fill-opacity="1" stroke="none" xml:space="preserve" x="4" y="17" font-family="Arial" font-size="10pt" font-weight="700" font-style="italic" >N</text> </g> <g fill="#ffff33" fill-opacity="1" stroke="#bfbfbf" stroke-opacity="1" stroke-width="1" stroke-linecap="square" stroke-linejoin="bevel" transform="matrix(1,0,0,1,928,1730)" font-family="MS Shell Dlg 2" font-size="9.75" font-weight="400" font-style="normal" > <rect x="0" y="0" width="50" height="20"/> </g> <g fill="none" stroke="#000000" stroke-opacity="1" stroke-width="1" stroke-linecap="square" stroke-linejoin="bevel" transform="matrix(1,0,0,1,936,1728)" font-family="MS Shell Dlg 2" font-size="9.75" font-weight="400" font-style="normal" > <text fill="#000000" fill-opacity="1" stroke="none" xml:space="preserve" x="4" y="17" font-family="Arial" font-size="10pt" font-weight="700" font-style="italic" >cox3</text> </g> <g fill="#ff9999" fill-opacity="1" stroke="#bfbfbf" stroke-opacity="1" stroke-width="1" stroke-linecap="square" stroke-linejoin="bevel" transform="matrix(1,0,0,1,979,1730)" font-family="MS Shell Dlg 2" font-size="9.75" font-weight="400" font-style="normal" > <path vector-effect="none" fill-rule="evenodd" d="M0,1.7 L42.5,1.7 L42.5,0 L50,10 L42.5,20 L42.5,18.3 L0,18.3 L0,1.7"/> </g> <g fill="none" stroke="#000000" stroke-opacity="1" stroke-width="1" stroke-linecap="square" stroke-linejoin="bevel" transform="matrix(1,0,0,1,992.633,1728)" font-family="MS Shell Dlg 2" font-size="9.75" font-weight="400" font-style="normal" > <text fill="#000000" fill-opacity="1" stroke="none" xml:space="preserve" x="4" y="17" font-family="Arial" font-size="10pt" font-weight="700" font-style="italic" >F</text> </g> <g fill="#99ffff" fill-opacity="1" stroke="#bfbfbf" stroke-opacity="1" stroke-width="1" stroke-linecap="square" stroke-linejoin="bevel" transform="matrix(1,0,0,1,1030,1730)" font-family="MS Shell Dlg 2" font-size="9.75" font-weight="400" font-style="normal" > <path vector-effect="none" fill-rule="evenodd" d="M0,5 C0,2.23858 2.23858,0 5,0 L45,0 C47.7614,0 50,2.23858 50,5 L50,15 C50,17.7614 47.7614,20 45,20 L5,20 C2.23858,20 0,17.7614 0,15 L0,5"/> </g> <g fill="none" stroke="#000000" stroke-opacity="1" stroke-width="1" stroke-linecap="square" stroke-linejoin="bevel" transform="matrix(1,0,0,1,1039.5,1728)" font-family="MS Shell Dlg 2" font-size="9.75" font-

weight="400" font-style="normal" > <text fill="#000000" fill-opacity="1" stroke="none" xml:space="preserve" x="4" y="17" font-family="Arial" font-size="10pt" font-weight="700" font-style="italic" >rrnL</text> </g> <g fill="#ff9999" fill-opacity="1" stroke="#bfbfbf" stroke-opacity="1" stroke-width="1" stroke-linecap="square" stroke-linejoin="bevel" transform="matrix(1,0,0,1,1081,1730)" font-family="MS Shell Dlg 2" font-size="9.75" font-weight="400" font-style="normal" > <path vector-effect="none" fill-rule="evenodd" d="M0,1.7 L42.5,1.7 L42.5,0 L50,10 L42.5,20 L42.5,18.3 L0,18.3 L0,1.7"/> </g> <g fill="none" stroke="#000000" stroke-opacity="1" stroke-width="1" stroke-linecap="square" stroke-linejoin="bevel" transform="matrix(1,0,0,1,1094.13,1728)" font-family="MS Shell Dlg 2" font-size="9.75" font-weight="400" font-style="normal" > <text fill="#000000" fill-opacity="1" stroke="none" xml:space="preserve" x="4" y="17" font-family="Arial" font-size="10pt" font-weight="700" font-style="italic" >S</text> </g> <g fill="#ff9999" fill-opacity="1" stroke="#bfbfbf" stroke-opacity="1" stroke-width="1" stroke-linecap="square" stroke-linejoin="bevel" transform="matrix(1,0,0,1,1132,1730)" font-family="MS Shell Dlg 2" font-size="9.75" font-weight="400" font-style="normal" > <path vector-effect="none" fill-rule="evenodd" d="M0,1.7 L42.5,1.7 L42.5,0 L50,10 L42.5,20 L42.5,18.3 L0,18.3 L0,1.7"/> </g> <g fill="none" stroke="#000000" stroke-opacity="1" stroke-width="1" stroke-linecap="square" stroke-linejoin="bevel" transform="matrix(1,0,0,1,1145.13,1728)" font-family="MS Shell Dlg 2" font-size="9.75" font-weight="400" font-style="normal" > <text fill="#000000" fill-opacity="1" stroke="none" xml:space="preserve" x="4" y="17" font-family="Arial" font-size="10pt" font-weight="700" font-style="italic" >D</text> </g> <g fill="#ffff33" fill-opacity="1" stroke="#bfbfbf" stroke-opacity="1" stroke-width="1" stroke-linecap="square" stroke-linejoin="bevel" transform="matrix(1,0,0,1,1183,1730)" font-family="MS Shell Dlg 2" font-size="9.75" font-weight="400" font-style="normal" > <rect x="0" y="0" width="50" height="20"/> </g> <g fill="none" stroke="#000000" stroke-opacity="1" stroke-width="1" stroke-linecap="square" stroke-linejoin="bevel" transform="matrix(1,0,0,1,1190,1728)" font-family="MS Shell Dlg 2" font-size="9.75" font-weight="400" font-style="normal" > <text fill="#000000" fill-opacity="1" stroke="none" xml:space="preserve" x="4" y="17" font-family="Arial" font-size="10pt" font-weight="700" font-style="italic" >nad6</text> </g> <g fill="#ff9999" fill-opacity="1" stroke="#bfbfbf" stroke-opacity="1" stroke-width="1" stroke-linecap="square" stroke-linejoin="bevel" transform="matrix(1,0,0,1,1234,1730)" font-family="MS Shell Dlg 2" font-size="9.75" font-weight="400" font-style="normal" > <path vector-effect="none" fill-rule="evenodd" d="M0,1.7 L42.5,1.7 L42.5,0 L50,10 L42.5,20 L42.5,18.3 L0,18.3 L0,1.7"/> </g> <g fill="none" stroke="#000000" stroke-opacity="1" stroke-width="1" stroke-linecap="square" stroke-linejoin="bevel" transform="matrix(1,0,0,1,1249.13,1728)" font-family="MS Shell Dlg 2" font-size="9.75" font-weight="400" font-style="normal" > <text fill="#000000" fill-opacity="1" stroke="none" xml:space="preserve" x="4" y="17" font-family="Arial" font-size="10pt" font-weight="700" font-style="italic" >I</text> </g> <g fill="#ffff33" fill-opacity="1" stroke="#bfbfbf" stroke-opacity="1" stroke-width="1" stroke-linecap="square" stroke-linejoin="bevel" transform="matrix(1,0,0,1,1285,1730)" font-family="MS Shell Dlg 2" font-size="9.75" font-weight="400" font-style="normal" > <rect x="0" y="0" width="50" height="20"/> </g> <g fill="none" stroke="#000000" stroke-opacity="1" stroke-width="1" stroke-linecap="square" stroke-linejoin="bevel" transform="matrix(1,0,0,1,1292,1728)" font-family="MS Shell Dlg 2" font-size="9.75" font-weight="400" font-style="normal" > <text fill="#000000" fill-opacity="1" stroke="none" xml:space="preserve" x="4" y="17" font-family="Arial" font-size="10pt" font-weight="700" font-style="italic" >nad2</text> </g> <g fill="#ff9999" fill-opacity="1" stroke="#bfbfbf" stroke-opacity="1" stroke-width="1" stroke-linecap="square" stroke-linejoin="bevel" transform="matrix(1,0,0,1,1336,1730)" font-family="MS Shell Dlg 2" font-size="9.75" font-weight="400" font-style="normal" > <path vector-effect="none" fill-rule="evenodd" d="M0,1.7 L42.5,1.7 L42.5,0 L50,10 L42.5,20 L42.5,18.3 L0,18.3 L0,1.7"/> </g> <g fill="none" stroke="#000000" stroke-opacity="1" stroke-width="1" stroke-linecap="square" stroke-linejoin="bevel" transform="matrix(1,0,0,1,1347.13,1728)" font-family="MS Shell Dlg 2" font-size="9.75" font-weight="400" font-style="normal" > <text fill="#000000" fill-opacity="1" stroke="none" xml:space="preserve" x="4" y="17" font-family="Arial" font-size="10pt" font-weight="700" font-style="italic" >W</text> </g> <g fill="#ff9999" fill-opacity="1" stroke="#bfbfbf" stroke-opacity="1" stroke-width="1" stroke-linecap="square" stroke-linejoin="bevel" transform="matrix(1,0,0,1,1387,1730)" font-family="MS Shell Dlg 2" font-size="9.75" font-weight="400" font-style="normal" > <path vector-effect="none" fill-rule="evenodd" d="M0,1.7 L42.5,1.7 L42.5,0 L50,10 L42.5,20 L42.5,18.3 L0,18.3 L0,1.7"/> </g> <g fill="none" stroke="#000000" stroke-opacity="1" stroke-width="1" stroke-linecap="square" stroke-linejoin="bevel" transform="matrix(1,0,0,1,1400.13,1728)" font-family="MS Shell Dlg 2" font-size="9.75" font-weight="400" font-style="normal" > <text fill="#000000" fill-opacity="1" stroke="none" xml:space="preserve" x="4" y="17" font-family="Arial" font-size="10pt" font-weight="700" font-style="italic" >G</text> </g> <g fill="#ff9999" fill-opacity="1" stroke="#bfbfbf" stroke-opacity="1" stroke-width="1" stroke-linecap="square" stroke-linejoin="bevel" transform="matrix(1,0,0,1,1438,1730)" font-family="MS Shell Dlg 2" font-size="9.75" font-weight="400" font-

style="normal" > <path vector-effect="none" fill-rule="evenodd" d="M0,1.7 L42.5,1.7 L42.5,0 L50,10 L42.5,20 L42.5,18.3 L0,18.3 L0,1.7"/> </g> <g fill="none" stroke="#000000" stroke-opacity="1" stroke-width="1" stroke-linecap="square" stroke-linejoin="bevel" transform="matrix(1,0,0,1,1451.13,1728)" font-family="MS Shell Dlg 2" font-size="9.75" font-weight="400" font-style="normal" > <text fill="#000000" fill-opacity="1" stroke="none" xml:space="preserve" x="4" y="17" font-family="Arial" font-size="10pt" font-weight="700" font-style="italic" >T</text> </g> <g fill="#ffff33" fill-opacity="1" stroke="#bfbfbf" stroke-opacity="1" stroke-width="1" stroke-linecap="square" stroke-linejoin="bevel" transform="matrix(1,0,0,1,1489,1730)" font-family="MS Shell Dlg 2" font-size="9.75" font-weight="400" font-style="normal" > <rect x="0" y="0" width="50" height="20"/> </g> <g fill="none" stroke="#000000" stroke-opacity="1" stroke-width="1" stroke-linecap="square" stroke-linejoin="bevel" transform="matrix(1,0,0,1,1498.5,1728)" font-family="MS Shell Dlg 2" font-size="9.75" font-weight="400" font-style="normal" > <text fill="#000000" fill-opacity="1" stroke="none" xml:space="preserve" x="4" y="17" font-family="Arial" font-size="10pt" font-weight="700" font-style="italic" >cytb</text> </g> <g fill="#ffff33" fill-opacity="1" stroke="#bfbfbf" stroke-opacity="1" stroke-width="1" stroke-linecap="square" stroke-linejoin="bevel" transform="matrix(1,0,0,1,1540,1730)" font-family="MS Shell Dlg 2" font-size="9.75" font-weight="400" font-style="normal" > <rect x="0" y="0" width="50" height="20"/> </g> <g fill="none" stroke="#000000" stroke-opacity="1" stroke-width="1" stroke-linecap="square" stroke-linejoin="bevel" transform="matrix(1,0,0,1,1544,1728)" font-family="MS Shell Dlg 2" font-size="9.75" font-weight="400" font-style="normal" > <text fill="#000000" fill-opacity="1" stroke="none" xml:space="preserve" x="4" y="17" font-family="Arial" font-size="10pt" font-weight="700" font-style="italic" >nad4L</text> </g> <g fill="#ff9999" fill-opacity="1" stroke="#bfbfbf" stroke-opacity="1" stroke-width="1" stroke-linecap="square" stroke-linejoin="bevel" transform="matrix(1,0,0,1,1591,1730)" font-family="MS Shell Dlg 2" font-size="9.75" font-weight="400" font-style="normal" > <path vector-effect="none" fill-rule="evenodd" d="M0,1.7 L42.5,1.7 L42.5,0 L50,10 L42.5,20 L42.5,18.3 L0,18.3 L0,1.7"/> </g> <g fill="none" stroke="#000000" stroke-opacity="1" stroke-width="1" stroke-linecap="square" stroke-linejoin="bevel" transform="matrix(1,0,0,1,1604.13,1728)" font-family="MS Shell Dlg 2" font-size="9.75" font-weight="400" font-style="normal" > <text fill="#000000" fill-opacity="1" stroke="none" xml:space="preserve" x="4" y="17" font-family="Arial" font-size="10pt" font-weight="700" font-style="italic" >A</text> </g> <g fill="#ff9999" fill-opacity="1" stroke="#bfbfbf" stroke-opacity="1" stroke-width="1" stroke-linecap="square" stroke-linejoin="bevel" transform="matrix(1,0,0,1,1642,1730)" font-family="MS Shell Dlg 2" font-size="9.75" font-weight="400" font-style="normal" > <path vector-effect="none" fill-rule="evenodd" d="M0,1.7 L42.5,1.7 L42.5,0 L50,10 L42.5,20 L42.5,18.3 L0,18.3 L0,1.7"/> </g> <g fill="none" stroke="#000000" stroke-opacity="1" stroke-width="1" stroke-linecap="square" stroke-linejoin="bevel" transform="matrix(1,0,0,1,1655.13,1728)" font-family="MS Shell Dlg 2" font-size="9.75" font-weight="400" font-style="normal" > <text fill="#000000" fill-opacity="1" stroke="none" xml:space="preserve" x="4" y="17" font-family="Arial" font-size="10pt" font-weight="700" font-style="italic" >H</text> </g> <g fill="#ffff33" fill-opacity="1" stroke="#bfbfbf" stroke-opacity="1" stroke-width="1" stroke-linecap="square" stroke-linejoin="bevel" transform="matrix(1,0,0,1,1693,1730)" font-family="MS Shell Dlg 2" font-size="9.75" font-weight="400" font-style="normal" > <rect x="0" y="0" width="50" height="20"/> </g> <g fill="none" stroke="#000000" stroke-opacity="1" stroke-width="1" stroke-linecap="square" stroke-linejoin="bevel" transform="matrix(1,0,0,1,1700,1728)" font-family="MS Shell Dlg 2" font-size="9.75" font-weight="400" font-style="normal" > <text fill="#000000" fill-opacity="1" stroke="none" xml:space="preserve" x="4" y="17" font-family="Arial" font-size="10pt" font-weight="700" font-style="italic" >nad5</text> </g> <g fill="#ffff33" fill-opacity="1" stroke="#bfbfbf" stroke-opacity="1" stroke-width="1" stroke-linecap="square" stroke-linejoin="bevel" transform="matrix(1,0,0,1,1744,1730)" font-family="MS Shell Dlg 2" font-size="9.75" font-weight="400" font-style="normal" > <rect x="0" y="0" width="50" height="20"/> </g> <g fill="none" stroke="#000000" stroke-opacity="1" stroke-width="1" stroke-linecap="square" stroke-linejoin="bevel" transform="matrix(1,0,0,1,1752,1728)" font-family="MS Shell Dlg 2" font-size="9.75" font-weight="400" font-style="normal" > <text fill="#000000" fill-opacity="1" stroke="none" xml:space="preserve" x="4" y="17" font-family="Arial" font-size="10pt" font-weight="700" font-style="italic" >cox2</text> </g> <g fill="#ffff33" fill-opacity="1" stroke="#bfbfbf" stroke-opacity="1" stroke-width="1" stroke-linecap="square" stroke-linejoin="bevel" transform="matrix(1,0,0,1,1795,1730)" font-family="MS Shell Dlg 2" font-size="9.75" font-weight="400" font-style="normal" > <rect x="0" y="0" width="50" height="20"/> </g> <g fill="none" stroke="#000000" stroke-opacity="1" stroke-width="1" stroke-linecap="square" stroke-linejoin="bevel" transform="matrix(1,0,0,1,1802,1728)" font-family="MS Shell Dlg 2" font-size="9.75" font-weight="400" font-style="normal" > <text fill="#000000" fill-opacity="1" stroke="none" xml:space="preserve" x="4" y="17" font-family="Arial" font-size="10pt" font-weight="700" font-style="italic" >nad1</text> </g> <g fill="#ffff33" fill-opacity="1" stroke="#bfbfbf" stroke-opacity="1" stroke-width="1" stroke-linecap="square" stroke-linejoin="bevel" transform="matrix(1,0,0,1,1846,1730)" font-family="MS Shell Dlg 2" font-size="9.75" font-

weight="400" font-style="normal" > <rect x="0" y="0" width="50" height="20"/> </g> <g fill="none"
stroke="#000000" stroke-opacity="1" stroke-width="1" stroke-linecap="square" stroke-linejoin="bevel"
transform="matrix(1,0,0,1,1854.5,1728)" font-family="MS Shell Dlg 2" font-size="9.75" font-weight="400" font-
style="normal" > <text fill="#000000" fill-opacity="1" stroke="none" xml:space="preserve" x="4" y="17" font-
family="Arial" font-size="10pt" font-weight="700" font-style="italic" >atp8</text> </g> <g fill="none"
stroke="#000000" stroke-opacity="1" stroke-width="1" stroke-linecap="square" stroke-linejoin="bevel"
transform="matrix(1,0,0,1,1897,1730)" font-family="MS Shell Dlg 2" font-size="9.75" font-weight="400" font-
style="normal" > <text fill="#000000" fill-opacity="1" stroke="none" xml:space="preserve" x="4" y="17" font-
family="Arial" font-size="10pt" font-weight="700" font-style="italic" >(+)</text> </g> <g fill="none"
stroke="#000000" stroke-opacity="1" stroke-width="1" stroke-linecap="square" stroke-linejoin="bevel"
transform="matrix(1,0,0,1,10,1770)" font-family="MS Shell Dlg 2" font-size="9.75" font-weight="400" font-
style="normal" > <text fill="#000000" fill-opacity="1" stroke="none" xml:space="preserve" x="4" y="17" font-
family="Arial" font-size="10pt" font-weight="700" font-style="italic" >Modiolus_kurilensis_NC_036486</text>
</g> <g fill="#ffff33" fill-opacity="1" stroke="#bfbfbf" stroke-opacity="1" stroke-width="1" stroke-
linecap="square" stroke-linejoin="bevel" transform="matrix(1,0,0,1,10,1800)" font-family="MS Shell Dlg 2" font-
size="9.75" font-weight="400" font-style="normal" > <rect x="0" y="0" width="50" height="20"/> </g> <g
fill="none" stroke="#000000" stroke-opacity="1" stroke-width="1" stroke-linecap="square" stroke-linejoin="bevel"
transform="matrix(1,0,0,1,18,1798)" font-family="MS Shell Dlg 2" font-size="9.75" font-weight="400" font-
style="normal" > <text fill="#000000" fill-opacity="1" stroke="none" xml:space="preserve" x="4" y="17" font-
family="Arial" font-size="10pt" font-weight="700" font-style="italic" >cox1</text> </g> <g fill="#ffff33"
fill-opacity="1" stroke="#bfbfbf" stroke-opacity="1" stroke-width="1" stroke-linecap="square" stroke-
linejoin="bevel" transform="matrix(1,0,0,1,61,1800)" font-family="MS Shell Dlg 2" font-size="9.75" font-
weight="400" font-style="normal" > <rect x="0" y="0" width="50" height="20"/> </g> <g fill="none"
stroke="#000000" stroke-opacity="1" stroke-width="1" stroke-linecap="square" stroke-linejoin="bevel"
transform="matrix(1,0,0,1,68,1798)" font-family="MS Shell Dlg 2" font-size="9.75" font-weight="400" font-
style="normal" > <text fill="#000000" fill-opacity="1" stroke="none" xml:space="preserve" x="4" y="17" font-
family="Arial" font-size="10pt" font-weight="700" font-style="italic" >nad3</text> </g> <g
fill="#ff9999" fill-opacity="1" stroke="#bfbfbf" stroke-opacity="1" stroke-width="1" stroke-linecap="square"
stroke-linejoin="bevel" transform="matrix(1,0,0,1,112,1800)" font-family="MS Shell Dlg 2" font-size="9.75" font-
weight="400" font-style="normal" > <path vector-effect="none" fill-rule="evenodd" d="M0,1.7 L42.5,1.7 L42.5,0
L50,10 L42.5,20 L42.5,18.3 L0,18.3 L0,1.7"/> </g> <g fill="none" stroke="#000000" stroke-opacity="1"
stroke-width="1" stroke-linecap="square" stroke-linejoin="bevel" transform="matrix(1,0,0,1,125.132,1798)" font-
family="MS Shell Dlg 2" font-size="9.75" font-weight="400" font-style="normal" > <text fill="#000000" fill-
opacity="1" stroke="none" xml:space="preserve" x="4" y="17" font-family="Arial" font-size="10pt" font-
weight="700" font-style="italic" >C</text> </g> <g fill="#ff9999" fill-opacity="1" stroke="#bfbfbf"
stroke-opacity="1" stroke-width="1" stroke-linecap="square" stroke-linejoin="bevel"
transform="matrix(1,0,0,1,163,1800)" font-family="MS Shell Dlg 2" font-size="9.75" font-weight="400" font-
style="normal" > <path vector-effect="none" fill-rule="evenodd" d="M0,1.7 L42.5,1.7 L42.5,0 L50,10 L42.5,20
L42.5,18.3 L0,18.3 L0,1.7"/> </g> <g fill="none" stroke="#000000" stroke-opacity="1" stroke-width="1"
stroke-linecap="square" stroke-linejoin="bevel" transform="matrix(1,0,0,1,173.632,1798)" font-family="MS Shell
Dlg 2" font-size="9.75" font-weight="400" font-style="normal" > <text fill="#000000" fill-opacity="1"
stroke="none" xml:space="preserve" x="4" y="17" font-family="Arial" font-size="10pt" font-weight="700" font-
style="italic" >L1</text> </g> <g fill="#ff9999" fill-opacity="1" stroke="#bfbfbf" stroke-opacity="1"
stroke-width="1" stroke-linecap="square" stroke-linejoin="bevel" transform="matrix(1,0,0,1,214,1800)" font-
family="MS Shell Dlg 2" font-size="9.75" font-weight="400" font-style="normal" > <path vector-effect="none"
fill-rule="evenodd" d="M0,1.7 L42.5,1.7 L42.5,0 L50,10 L42.5,20 L42.5,18.3 L0,18.3 L0,1.7"/> </g> <g
fill="none" stroke="#000000" stroke-opacity="1" stroke-width="1" stroke-linecap="square" stroke-linejoin="bevel"
transform="matrix(1,0,0,1,227.632,1798)" font-family="MS Shell Dlg 2" font-size="9.75" font-weight="400" font-
style="normal" > <text fill="#000000" fill-opacity="1" stroke="none" xml:space="preserve" x="4" y="17" font-
family="Arial" font-size="10pt" font-weight="700" font-style="italic" >P</text> </g> <g fill="#ff9999"
fill-opacity="1" stroke="#bfbfbf" stroke-opacity="1" stroke-width="1" stroke-linecap="square" stroke-
linejoin="bevel" transform="matrix(1,0,0,1,265,1800)" font-family="MS Shell Dlg 2" font-size="9.75" font-
weight="400" font-style="normal" > <path vector-effect="none" fill-rule="evenodd" d="M0,1.7 L42.5,1.7 L42.5,0
L50,10 L42.5,20 L42.5,18.3 L0,18.3 L0,1.7"/> </g> <g fill="none" stroke="#000000" stroke-opacity="1"
stroke-width="1" stroke-linecap="square" stroke-linejoin="bevel" transform="matrix(1,0,0,1,278.632,1798)" font-
family="MS Shell Dlg 2" font-size="9.75" font-weight="400" font-style="normal" > <text fill="#000000" fill-
opacity="1" stroke="none" xml:space="preserve" x="4" y="17" font-family="Arial" font-size="10pt" font-
weight="700" font-style="italic" >E</text> </g> <g fill="#ff9999" fill-opacity="1" stroke="#bfbfbf"

stroke-opacity="1" stroke-width="1" stroke-linecap="square" stroke-linejoin="bevel"
transform="matrix(1,0,0,1,316,1800)" font-family="MS Shell Dlg 2" font-size="9.75" font-weight="400" font-
style="normal" > <path vector-effect="none" fill-rule="evenodd" d="M0,1.7 L42.5,1.7 L42.5,0 L50,10 L42.5,20
L42.5,18.3 L0,18.3 L0,1.7"/> </g> <g fill="none" stroke="#000000" stroke-opacity="1" stroke-width="1"
stroke-linecap="square" stroke-linejoin="bevel" transform="matrix(1,0,0,1,326.632,1798)" font-family="MS Shell
Dlg 2" font-size="9.75" font-weight="400" font-style="normal" > <text fill="#000000" fill-opacity="1"
stroke="none" xml:space="preserve" x="4" y="17" font-family="Arial" font-size="10pt" font-weight="700" font-
style="italic" >L2</text> </g> <g fill="#ff9999" fill-opacity="1" stroke="#bfbfbf" stroke-opacity="1"
stroke-width="1" stroke-linecap="square" stroke-linejoin="bevel" transform="matrix(1,0,0,1,367,1800)" font-
family="MS Shell Dlg 2" font-size="9.75" font-weight="400" font-style="normal" > <path vector-effect="none"
fill-rule="evenodd" d="M0,1.7 L42.5,1.7 L42.5,0 L50,10 L42.5,20 L42.5,18.3 L0,18.3 L0,1.7"/> </g> <g
fill="none" stroke="#000000" stroke-opacity="1" stroke-width="1" stroke-linecap="square" stroke-linejoin="bevel"
transform="matrix(1,0,0,1,380.132,1798)" font-family="MS Shell Dlg 2" font-size="9.75" font-weight="400" font-
style="normal" > <text fill="#000000" fill-opacity="1" stroke="none" xml:space="preserve" x="4" y="17" font-
family="Arial" font-size="10pt" font-weight="700" font-style="italic" >R</text> </g> <g fill="#ff9999"
fill-opacity="1" stroke="#bfbfbf" stroke-opacity="1" stroke-width="1" stroke-linecap="square" stroke-
linejoin="bevel" transform="matrix(1,0,0,1,418,1800)" font-family="MS Shell Dlg 2" font-size="9.75" font-
weight="400" font-style="normal" > <path vector-effect="none" fill-rule="evenodd" d="M0,1.7 L42.5,1.7 L42.5,0
L50,10 L42.5,20 L42.5,18.3 L0,18.3 L0,1.7"/> </g> <g fill="none" stroke="#000000" stroke-opacity="1"
stroke-width="1" stroke-linecap="square" stroke-linejoin="bevel" transform="matrix(1,0,0,1,427.632,1798)" font-
family="MS Shell Dlg 2" font-size="9.75" font-weight="400" font-style="normal" > <text fill="#000000" fill-
opacity="1" stroke="none" xml:space="preserve" x="4" y="17" font-family="Arial" font-size="10pt" font-
weight="700" font-style="italic" >S1</text> </g> <g fill="#ff9999" fill-opacity="1" stroke="#bfbfbf"
stroke-opacity="1" stroke-width="1" stroke-linecap="square" stroke-linejoin="bevel"
transform="matrix(1,0,0,1,469,1800)" font-family="MS Shell Dlg 2" font-size="9.75" font-weight="400" font-
style="normal" > <path vector-effect="none" fill-rule="evenodd" d="M0,1.7 L42.5,1.7 L42.5,0 L50,10 L42.5,20
L42.5,18.3 L0,18.3 L0,1.7"/> </g> <g fill="none" stroke="#000000" stroke-opacity="1" stroke-width="1"
stroke-linecap="square" stroke-linejoin="bevel" transform="matrix(1,0,0,1,481.132,1798)" font-family="MS Shell
Dlg 2" font-size="9.75" font-weight="400" font-style="normal" > <text fill="#000000" fill-opacity="1"
stroke="none" xml:space="preserve" x="4" y="17" font-family="Arial" font-size="10pt" font-weight="700" font-
style="italic" >M</text> </g> <g fill="#ff9999" fill-opacity="1" stroke="#bfbfbf" stroke-opacity="1"
stroke-width="1" stroke-linecap="square" stroke-linejoin="bevel" transform="matrix(1,0,0,1,520,1800)" font-
family="MS Shell Dlg 2" font-size="9.75" font-weight="400" font-style="normal" > <path vector-effect="none"
fill-rule="evenodd" d="M0,1.7 L42.5,1.7 L42.5,0 L50,10 L42.5,20 L42.5,18.3 L0,18.3 L0,1.7"/> </g> <g
fill="none" stroke="#000000" stroke-opacity="1" stroke-width="1" stroke-linecap="square" stroke-linejoin="bevel"
transform="matrix(1,0,0,1,533.633,1798)" font-family="MS Shell Dlg 2" font-size="9.75" font-weight="400" font-
style="normal" > <text fill="#000000" fill-opacity="1" stroke="none" xml:space="preserve" x="4" y="17" font-
family="Arial" font-size="10pt" font-weight="700" font-style="italic" >K</text> </g> <g fill="#99ffff"
fill-opacity="1" stroke="#bfbfbf" stroke-opacity="1" stroke-width="1" stroke-linecap="square" stroke-
linejoin="bevel" transform="matrix(1,0,0,1,571,1800)" font-family="MS Shell Dlg 2" font-size="9.75" font-
weight="400" font-style="normal" > <path vector-effect="none" fill-rule="evenodd" d="M0,5 C0,2.23858
2.23858,0 5,0 L45,0 C47.7614,0 50,2.23858 50,5 L50,15 C50,17.7614 47.7614,20 45,20 L5,20 C2.23858,20
0,17.7614 0,15 L0,5"/> </g> <g fill="none" stroke="#000000" stroke-opacity="1" stroke-width="1" stroke-
linecap="square" stroke-linejoin="bevel" transform="matrix(1,0,0,1,579.5,1798)" font-family="MS Shell Dlg 2"
font-size="9.75" font-weight="400" font-style="normal" > <text fill="#000000" fill-opacity="1" stroke="none"
xml:space="preserve" x="4" y="17" font-family="Arial" font-size="10pt" font-weight="700" font-style="italic"
>rrnS</text> </g> <g fill="#ff9999" fill-opacity="1" stroke="#bfbfbf" stroke-opacity="1" stroke-width="1"
stroke-linecap="square" stroke-linejoin="bevel" transform="matrix(1,0,0,1,622,1800)" font-family="MS Shell Dlg
2" font-size="9.75" font-weight="400" font-style="normal" > <path vector-effect="none" fill-rule="evenodd"
d="M0,1.7 L42.5,1.7 L42.5,0 L50,10 L42.5,20 L42.5,18.3 L0,18.3 L0,1.7"/> </g> <g fill="none"
stroke="#000000" stroke-opacity="1" stroke-width="1" stroke-linecap="square" stroke-linejoin="bevel"
transform="matrix(1,0,0,1,634.633,1798)" font-family="MS Shell Dlg 2" font-size="9.75" font-weight="400" font-
style="normal" > <text fill="#000000" fill-opacity="1" stroke="none" xml:space="preserve" x="4" y="17" font-
family="Arial" font-size="10pt" font-weight="700" font-style="italic" >Q</text> </g> <g fill="#ffff33"
fill-opacity="1" stroke="#bfbfbf" stroke-opacity="1" stroke-width="1" stroke-linecap="square" stroke-
linejoin="bevel" transform="matrix(1,0,0,1,673,1800)" font-family="MS Shell Dlg 2" font-size="9.75" font-
weight="400" font-style="normal" > <rect x="0" y="0" width="50" height="20"/> </g> <g fill="none"
stroke="#000000" stroke-opacity="1" stroke-width="1" stroke-linecap="square" stroke-linejoin="bevel"

transform="matrix(1,0,0,1,681.5,1798)" font-family="MS Shell Dlg 2" font-size="9.75" font-weight="400" font-style="normal" > <text fill="#000000" fill-opacity="1" stroke="none" xml:space="preserve" x="4" y="17" font-family="Arial" font-size="10pt" font-weight="700" font-style="italic" >atp6</text> </g> <g fill="#ff9999" fill-opacity="1" stroke="#bfbfbf" stroke-opacity="1" stroke-width="1" stroke-linecap="square" stroke-linejoin="bevel" transform="matrix(1,0,0,1,724,1800)" font-family="MS Shell Dlg 2" font-size="9.75" font-weight="400" font-style="normal" > <path vector-effect="none" fill-rule="evenodd" d="M0,1.7 L42.5,1.7 L42.5,0 L50,10 L42.5,20 L42.5,18.3 L0,18.3 L0,1.7"/> </g> <g fill="none" stroke="#000000" stroke-opacity="1" stroke-width="1" stroke-linecap="square" stroke-linejoin="bevel" transform="matrix(1,0,0,1,737.633,1798)" font-family="MS Shell Dlg 2" font-size="9.75" font-weight="400" font-style="normal" > <text fill="#000000" fill-opacity="1" stroke="none" xml:space="preserve" x="4" y="17" font-family="Arial" font-size="10pt" font-weight="700" font-style="italic" >Y</text> </g> <g fill="#ff9999" fill-opacity="1" stroke="#bfbfbf" stroke-opacity="1" stroke-width="1" stroke-linecap="square" stroke-linejoin="bevel" transform="matrix(1,0,0,1,775,1800)" font-family="MS Shell Dlg 2" font-size="9.75" font-weight="400" font-style="normal" > <path vector-effect="none" fill-rule="evenodd" d="M0,1.7 L42.5,1.7 L42.5,0 L50,10 L42.5,20 L42.5,18.3 L0,18.3 L0,1.7"/> </g> <g fill="none" stroke="#000000" stroke-opacity="1" stroke-width="1" stroke-linecap="square" stroke-linejoin="bevel" transform="matrix(1,0,0,1,788.133,1798)" font-family="MS Shell Dlg 2" font-size="9.75" font-weight="400" font-style="normal" > <text fill="#000000" fill-opacity="1" stroke="none" xml:space="preserve" x="4" y="17" font-family="Arial" font-size="10pt" font-weight="700" font-style="italic" >V</text> </g> <g fill="#ffff33" fill-opacity="1" stroke="#bfbfbf" stroke-opacity="1" stroke-width="1" stroke-linecap="square" stroke-linejoin="bevel" transform="matrix(1,0,0,1,826,1800)" font-family="MS Shell Dlg 2" font-size="9.75" font-weight="400" font-style="normal" > <rect x="0" y="0" width="50" height="20"/> </g> <g fill="none" stroke="#000000" stroke-opacity="1" stroke-width="1" stroke-linecap="square" stroke-linejoin="bevel" transform="matrix(1,0,0,1,833,1798)" font-family="MS Shell Dlg 2" font-size="9.75" font-weight="400" font-style="normal" > <text fill="#000000" fill-opacity="1" stroke="none" xml:space="preserve" x="4" y="17" font-family="Arial" font-size="10pt" font-weight="700" font-style="italic" >nad4</text> </g> <g fill="#ff9999" fill-opacity="1" stroke="#bfbfbf" stroke-opacity="1" stroke-width="1" stroke-linecap="square" stroke-linejoin="bevel" transform="matrix(1,0,0,1,877,1800)" font-family="MS Shell Dlg 2" font-size="9.75" font-weight="400" font-style="normal" > <path vector-effect="none" fill-rule="evenodd" d="M0,1.7 L42.5,1.7 L42.5,0 L50,10 L42.5,20 L42.5,18.3 L0,18.3 L0,1.7"/> </g> <g fill="none" stroke="#000000" stroke-opacity="1" stroke-width="1" stroke-linecap="square" stroke-linejoin="bevel" transform="matrix(1,0,0,1,890.133,1798)" font-family="MS Shell Dlg 2" font-size="9.75" font-weight="400" font-style="normal" > <text fill="#000000" fill-opacity="1" stroke="none" xml:space="preserve" x="4" y="17" font-family="Arial" font-size="10pt" font-weight="700" font-style="italic" >N</text> </g> <g fill="#ffff33" fill-opacity="1" stroke="#bfbfbf" stroke-opacity="1" stroke-width="1" stroke-linecap="square" stroke-linejoin="bevel" transform="matrix(1,0,0,1,928,1800)" font-family="MS Shell Dlg 2" font-size="9.75" font-weight="400" font-style="normal" > <rect x="0" y="0" width="50" height="20"/> </g> <g fill="none" stroke="#000000" stroke-opacity="1" stroke-width="1" stroke-linecap="square" stroke-linejoin="bevel" transform="matrix(1,0,0,1,936,1798)" font-family="MS Shell Dlg 2" font-size="9.75" font-weight="400" font-style="normal" > <text fill="#000000" fill-opacity="1" stroke="none" xml:space="preserve" x="4" y="17" font-family="Arial" font-size="10pt" font-weight="700" font-style="italic" >cox3</text> </g> <g fill="#ff9999" fill-opacity="1" stroke="#bfbfbf" stroke-opacity="1" stroke-width="1" stroke-linecap="square" stroke-linejoin="bevel" transform="matrix(1,0,0,1,979,1800)" font-family="MS Shell Dlg 2" font-size="9.75" font-weight="400" font-style="normal" > <path vector-effect="none" fill-rule="evenodd" d="M0,1.7 L42.5,1.7 L42.5,0 L50,10 L42.5,20 L42.5,18.3 L0,18.3 L0,1.7"/> </g> <g fill="none" stroke="#000000" stroke-opacity="1" stroke-width="1" stroke-linecap="square" stroke-linejoin="bevel" transform="matrix(1,0,0,1,992.633,1798)" font-family="MS Shell Dlg 2" font-size="9.75" font-weight="400" font-style="normal" > <text fill="#000000" fill-opacity="1" stroke="none" xml:space="preserve" x="4" y="17" font-family="Arial" font-size="10pt" font-weight="700" font-style="italic" >F</text> </g> <g fill="#99ffff" fill-opacity="1" stroke="#bfbfbf" stroke-opacity="1" stroke-width="1" stroke-linecap="square" stroke-linejoin="bevel" transform="matrix(1,0,0,1,1030,1800)" font-family="MS Shell Dlg 2" font-size="9.75" font-weight="400" font-style="normal" > <path vector-effect="none" fill-rule="evenodd" d="M0,5 C0,2.23858 2.23858,0 5,0 L45,0 C47.7614,0 50,2.23858 50,5 L50,15 C50,17.7614 47.7614,20 45,20 L5,20 C2.23858,20 0,17.7614 0,15 L0,5"/> </g> <g fill="none" stroke="#000000" stroke-opacity="1" stroke-width="1" stroke-linecap="square" stroke-linejoin="bevel" transform="matrix(1,0,0,1,1039.5,1798)" font-family="MS Shell Dlg 2" font-size="9.75" font-weight="400" font-style="normal" > <text fill="#000000" fill-opacity="1" stroke="none" xml:space="preserve" x="4" y="17" font-family="Arial" font-size="10pt" font-weight="700" font-style="italic" >rrnL</text> </g> <g fill="#ff9999" fill-opacity="1" stroke="#bfbfbf" stroke-opacity="1" stroke-width="1" stroke-linecap="square" stroke-linejoin="bevel" transform="matrix(1,0,0,1,1081,1800)" font-family="MS Shell Dlg 2" font-size="9.75"

font-weight="400" font-style="normal" > <path vector-effect="none" fill-rule="evenodd" d="M0,1.7 L42.5,1.7 L42.5,0 L50,10 L42.5,20 L42.5,18.3 L0,18.3 L0,1.7"/> </g> <g fill="none" stroke="#000000" stroke-opacity="1" stroke-width="1" stroke-linecap="square" stroke-linejoin="bevel" transform="matrix(1,0,0,1,1094.13,1798)" font-family="MS Shell Dlg 2" font-size="9.75" font-weight="400" font-style="normal" > <text fill="#000000" fill-opacity="1" stroke="none" xml:space="preserve" x="4" y="17" font-family="Arial" font-size="10pt" font-weight="700" font-style="italic" >S</text> </g> <g fill="#ff9999" fill-opacity="1" stroke="#bfbfbf" stroke-opacity="1" stroke-width="1" stroke-linecap="square" stroke-linejoin="bevel" transform="matrix(1,0,0,1,1132,1800)" font-family="MS Shell Dlg 2" font-size="9.75" font-weight="400" font-style="normal" > <path vector-effect="none" fill-rule="evenodd" d="M0,1.7 L42.5,1.7 L42.5,0 L50,10 L42.5,20 L42.5,18.3 L0,18.3 L0,1.7"/> </g> <g fill="none" stroke="#000000" stroke-opacity="1" stroke-width="1" stroke-linecap="square" stroke-linejoin="bevel" transform="matrix(1,0,0,1,1145.13,1798)" font-family="MS Shell Dlg 2" font-size="9.75" font-weight="400" font-style="normal" > <text fill="#000000" fill-opacity="1" stroke="none" xml:space="preserve" x="4" y="17" font-family="Arial" font-size="10pt" font-weight="700" font-style="italic" >D</text> </g> <g fill="#ffff33" fill-opacity="1" stroke="#bfbfbf" stroke-opacity="1" stroke-width="1" stroke-linecap="square" stroke-linejoin="bevel" transform="matrix(1,0,0,1,1183,1800)" font-family="MS Shell Dlg 2" font-size="9.75" font-weight="400" font-style="normal" > <rect x="0" y="0" width="50" height="20"/> </g> <g fill="none" stroke="#000000" stroke-opacity="1" stroke-width="1" stroke-linecap="square" stroke-linejoin="bevel" transform="matrix(1,0,0,1,1190,1798)" font-family="MS Shell Dlg 2" font-size="9.75" font-weight="400" font-style="normal" > <text fill="#000000" fill-opacity="1" stroke="none" xml:space="preserve" x="4" y="17" font-family="Arial" font-size="10pt" font-weight="700" font-style="italic" >nad6</text> </g> <g fill="#ff9999" fill-opacity="1" stroke="#bfbfbf" stroke-opacity="1" stroke-width="1" stroke-linecap="square" stroke-linejoin="bevel" transform="matrix(1,0,0,1,1234,1800)" font-family="MS Shell Dlg 2" font-size="9.75" font-weight="400" font-style="normal" > <path vector-effect="none" fill-rule="evenodd" d="M0,1.7 L42.5,1.7 L42.5,0 L50,10 L42.5,20 L42.5,18.3 L0,18.3 L0,1.7"/> </g> <g fill="none" stroke="#000000" stroke-opacity="1" stroke-width="1" stroke-linecap="square" stroke-linejoin="bevel" transform="matrix(1,0,0,1,1249.13,1798)" font-family="MS Shell Dlg 2" font-size="9.75" font-weight="400" font-style="normal" > <text fill="#000000" fill-opacity="1" stroke="none" xml:space="preserve" x="4" y="17" font-family="Arial" font-size="10pt" font-weight="700" font-style="italic" >I</text> </g> <g fill="#ffff33" fill-opacity="1" stroke="#bfbfbf" stroke-opacity="1" stroke-width="1" stroke-linecap="square" stroke-linejoin="bevel" transform="matrix(1,0,0,1,1285,1800)" font-family="MS Shell Dlg 2" font-size="9.75" font-weight="400" font-style="normal" > <rect x="0" y="0" width="50" height="20"/> </g> <g fill="none" stroke="#000000" stroke-opacity="1" stroke-width="1" stroke-linecap="square" stroke-linejoin="bevel" transform="matrix(1,0,0,1,1292,1798)" font-family="MS Shell Dlg 2" font-size="9.75" font-weight="400" font-style="normal" > <text fill="#000000" fill-opacity="1" stroke="none" xml:space="preserve" x="4" y="17" font-family="Arial" font-size="10pt" font-weight="700" font-style="italic" >nad2</text> </g> <g fill="#ff9999" fill-opacity="1" stroke="#bfbfbf" stroke-opacity="1" stroke-width="1" stroke-linecap="square" stroke-linejoin="bevel" transform="matrix(1,0,0,1,1336,1800)" font-family="MS Shell Dlg 2" font-size="9.75" font-weight="400" font-style="normal" > <path vector-effect="none" fill-rule="evenodd" d="M0,1.7 L42.5,1.7 L42.5,0 L50,10 L42.5,20 L42.5,18.3 L0,18.3 L0,1.7"/> </g> <g fill="none" stroke="#000000" stroke-opacity="1" stroke-width="1" stroke-linecap="square" stroke-linejoin="bevel" transform="matrix(1,0,0,1,1347.13,1798)" font-family="MS Shell Dlg 2" font-size="9.75" font-weight="400" font-style="normal" > <text fill="#000000" fill-opacity="1" stroke="none" xml:space="preserve" x="4" y="17" font-family="Arial" font-size="10pt" font-weight="700" font-style="italic" >W</text> </g> <g fill="#ff9999" fill-opacity="1" stroke="#bfbfbf" stroke-opacity="1" stroke-width="1" stroke-linecap="square" stroke-linejoin="bevel" transform="matrix(1,0,0,1,1387,1800)" font-family="MS Shell Dlg 2" font-size="9.75" font-weight="400" font-style="normal" > <path vector-effect="none" fill-rule="evenodd" d="M0,1.7 L42.5,1.7 L42.5,0 L50,10 L42.5,20 L42.5,18.3 L0,18.3 L0,1.7"/> </g> <g fill="none" stroke="#000000" stroke-opacity="1" stroke-width="1" stroke-linecap="square" stroke-linejoin="bevel" transform="matrix(1,0,0,1,1400.13,1798)" font-family="MS Shell Dlg 2" font-size="9.75" font-weight="400" font-style="normal" > <text fill="#000000" fill-opacity="1" stroke="none" xml:space="preserve" x="4" y="17" font-family="Arial" font-size="10pt" font-weight="700" font-style="italic" >G</text> </g> <g fill="#ff9999" fill-opacity="1" stroke="#bfbfbf" stroke-opacity="1" stroke-width="1" stroke-linecap="square" stroke-linejoin="bevel" transform="matrix(1,0,0,1,1438,1820)" font-family="MS Shell Dlg 2" font-size="9.75" font-weight="400" font-style="normal" > <path vector-effect="none" fill-rule="evenodd" d="M0,10 L7.5,0 L7.5,1.7 L50,1.7 L50,18.3 L7.5,18.3 L7.5,20 L0,10"/> </g> <g fill="none" stroke="#000000" stroke-opacity="1" stroke-width="1" stroke-linecap="square" stroke-linejoin="bevel" transform="matrix(1,0,0,1,1458.87,1818)" font-family="MS Shell Dlg 2" font-size="9.75" font-weight="400" font-style="normal" > <text fill="#000000" fill-opacity="1" stroke="none"

xml:space="preserve" x="4" y="17" font-family="Arial" font-size="10pt" font-weight="700" font-style="italic"
>T</text> </g> <g fill="#ffff33" fill-opacity="1" stroke="#bfbfbf" stroke-opacity="1" stroke-width="1"
stroke-linecap="square" stroke-linejoin="bevel" transform="matrix(1,0,0,1,1489,1800)" font-family="MS Shell Dlg
2" font-size="9.75" font-weight="400" font-style="normal" > <rect x="0" y="0" width="50" height="20"/> </g>
<g fill="none" stroke="#000000" stroke-opacity="1" stroke-width="1" stroke-linecap="square" stroke-
linejoin="bevel" transform="matrix(1,0,0,1,1498.5,1798)" font-family="MS Shell Dlg 2" font-size="9.75" font-
weight="400" font-style="normal" > <text fill="#000000" fill-opacity="1" stroke="none" xml:space="preserve"
x="4" y="17" font-family="Arial" font-size="10pt" font-weight="700" font-style="italic" >cytb</text> </g>
<g fill="#ffff33" fill-opacity="1" stroke="#bfbfbf" stroke-opacity="1" stroke-width="1" stroke-linecap="square"
stroke-linejoin="bevel" transform="matrix(1,0,0,1,1540,1800)" font-family="MS Shell Dlg 2" font-size="9.75"
font-weight="400" font-style="normal" > <rect x="0" y="0" width="50" height="20"/> </g> <g fill="none"
stroke="#000000" stroke-opacity="1" stroke-width="1" stroke-linecap="square" stroke-linejoin="bevel"
transform="matrix(1,0,0,1,1544,1798)" font-family="MS Shell Dlg 2" font-size="9.75" font-weight="400" font-
style="normal" > <text fill="#000000" fill-opacity="1" stroke="none" xml:space="preserve" x="4" y="17" font-
family="Arial" font-size="10pt" font-weight="700" font-style="italic" >nad4L</text> </g> <g
fill="#ff9999" fill-opacity="1" stroke="#bfbfbf" stroke-opacity="1" stroke-width="1" stroke-linecap="square"
stroke-linejoin="bevel" transform="matrix(1,0,0,1,1591,1800)" font-family="MS Shell Dlg 2" font-size="9.75"
font-weight="400" font-style="normal" > <path vector-effect="none" fill-rule="evenodd" d="M0,1.7 L42.5,1.7
L42.5,0 L50,10 L42.5,20 L42.5,18.3 L0,18.3 L0,1.7"/> </g> <g fill="none" stroke="#000000" stroke-
opacity="1" stroke-width="1" stroke-linecap="square" stroke-linejoin="bevel"
transform="matrix(1,0,0,1,1604.13,1798)" font-family="MS Shell Dlg 2" font-size="9.75" font-weight="400" font-
style="normal" > <text fill="#000000" fill-opacity="1" stroke="none" xml:space="preserve" x="4" y="17" font-
family="Arial" font-size="10pt" font-weight="700" font-style="italic" >A</text> </g> <g fill="#ff9999"
fill-opacity="1" stroke="#bfbfbf" stroke-opacity="1" stroke-width="1" stroke-linecap="square" stroke-
linejoin="bevel" transform="matrix(1,0,0,1,1642,1800)" font-family="MS Shell Dlg 2" font-size="9.75" font-
weight="400" font-style="normal" > <path vector-effect="none" fill-rule="evenodd" d="M0,1.7 L42.5,1.7 L42.5,0
L50,10 L42.5,20 L42.5,18.3 L0,18.3 L0,1.7"/> </g> <g fill="none" stroke="#000000" stroke-opacity="1"
stroke-width="1" stroke-linecap="square" stroke-linejoin="bevel" transform="matrix(1,0,0,1,1655.13,1798)" font-
family="MS Shell Dlg 2" font-size="9.75" font-weight="400" font-style="normal" > <text fill="#000000" fill-
opacity="1" stroke="none" xml:space="preserve" x="4" y="17" font-family="Arial" font-size="10pt" font-
weight="700" font-style="italic" >H</text> </g> <g fill="#ffff33" fill-opacity="1" stroke="#bfbfbf"
stroke-opacity="1" stroke-width="1" stroke-linecap="square" stroke-linejoin="bevel"
transform="matrix(1,0,0,1,1693,1800)" font-family="MS Shell Dlg 2" font-size="9.75" font-weight="400" font-
style="normal" > <rect x="0" y="0" width="50" height="20"/> </g> <g fill="none" stroke="#000000" stroke-
opacity="1" stroke-width="1" stroke-linecap="square" stroke-linejoin="bevel"
transform="matrix(1,0,0,1,1700,1798)" font-family="MS Shell Dlg 2" font-size="9.75" font-weight="400" font-
style="normal" > <text fill="#000000" fill-opacity="1" stroke="none" xml:space="preserve" x="4" y="17" font-
family="Arial" font-size="10pt" font-weight="700" font-style="italic" >nad5</text> </g> <g fill="#ffff33"
fill-opacity="1" stroke="#bfbfbf" stroke-opacity="1" stroke-width="1" stroke-linecap="square" stroke-
linejoin="bevel" transform="matrix(1,0,0,1,1744,1800)" font-family="MS Shell Dlg 2" font-size="9.75" font-
weight="400" font-style="normal" > <rect x="0" y="0" width="50" height="20"/> </g> <g fill="none"
stroke="#000000" stroke-opacity="1" stroke-width="1" stroke-linecap="square" stroke-linejoin="bevel"
transform="matrix(1,0,0,1,1752,1798)" font-family="MS Shell Dlg 2" font-size="9.75" font-weight="400" font-
style="normal" > <text fill="#000000" fill-opacity="1" stroke="none" xml:space="preserve" x="4" y="17" font-
family="Arial" font-size="10pt" font-weight="700" font-style="italic" >cox2</text> </g> <g fill="#ffff33"
fill-opacity="1" stroke="#bfbfbf" stroke-opacity="1" stroke-width="1" stroke-linecap="square" stroke-
linejoin="bevel" transform="matrix(1,0,0,1,1795,1800)" font-family="MS Shell Dlg 2" font-size="9.75" font-
weight="400" font-style="normal" > <rect x="0" y="0" width="50" height="20"/> </g> <g fill="none"
stroke="#000000" stroke-opacity="1" stroke-width="1" stroke-linecap="square" stroke-linejoin="bevel"
transform="matrix(1,0,0,1,1802,1798)" font-family="MS Shell Dlg 2" font-size="9.75" font-weight="400" font-
style="normal" > <text fill="#000000" fill-opacity="1" stroke="none" xml:space="preserve" x="4" y="17" font-
family="Arial" font-size="10pt" font-weight="700" font-style="italic" >nad1</text> </g> <g fill="none"
stroke="#000000" stroke-opacity="1" stroke-width="1" stroke-linecap="square" stroke-linejoin="bevel"
transform="matrix(1,0,0,1,1846,1800)" font-family="MS Shell Dlg 2" font-size="9.75" font-weight="400" font-
style="normal" > <text fill="#000000" fill-opacity="1" stroke="none" xml:space="preserve" x="4" y="17" font-
family="Arial" font-size="10pt" font-weight="700" font-style="italic" >(+)</text> </g> <g fill="none"
stroke="#000000" stroke-opacity="1" stroke-width="1" stroke-linecap="square" stroke-linejoin="bevel"
transform="matrix(1,0,0,1,1846,1820)" font-family="MS Shell Dlg 2" font-size="9.75" font-weight="400" font-

style="normal" > <text fill="#000000" fill-opacity="1" stroke="none" xml:space="preserve" x="4" y="17" font-family="Arial" font-size="10pt" font-weight="700" font-style="italic" >(-)</text> </g> <g fill="none" stroke="#000000" stroke-opacity="1" stroke-width="1" stroke-linecap="square" stroke-linejoin="bevel" transform="matrix(1,0,0,1,10,1860)" font-family="MS Shell Dlg 2" font-size="9.75" font-weight="400" font-style="normal" > <text fill="#000000" fill-opacity="1" stroke="none" xml:space="preserve" x="4" y="17" font-family="Arial" font-size="10pt" font-weight="700" font-style="italic" >Perna_canaliculus_MG766134</text> </g> <g fill="#ffff33" fill-opacity="1" stroke="#bfbfbf" stroke-opacity="1" stroke-width="1" stroke-linecap="square" stroke-linejoin="bevel" transform="matrix(1,0,0,1,10,1890)" font-family="MS Shell Dlg 2" font-size="9.75" font-weight="400" font-style="normal" > <rect x="0" y="0" width="50" height="20"/> </g> <g fill="none" stroke="#000000" stroke-opacity="1" stroke-width="1" stroke-linecap="square" stroke-linejoin="bevel" transform="matrix(1,0,0,1,18,1888)" font-family="MS Shell Dlg 2" font-size="9.75" font-weight="400" font-style="normal" > <text fill="#000000" fill-opacity="1" stroke="none" xml:space="preserve" x="4" y="17" font-family="Arial" font-size="10pt" font-weight="700" font-style="italic" >cox1</text> </g> <g fill="#ffff33" fill-opacity="1" stroke="#bfbfbf" stroke-opacity="1" stroke-width="1" stroke-linecap="square" stroke-linejoin="bevel" transform="matrix(1,0,0,1,61,1890)" font-family="MS Shell Dlg 2" font-size="9.75" font-weight="400" font-style="normal" > <rect x="0" y="0" width="50" height="20"/> </g> <g fill="none" stroke="#000000" stroke-opacity="1" stroke-width="1" stroke-linecap="square" stroke-linejoin="bevel" transform="matrix(1,0,0,1,69.5,1888)" font-family="MS Shell Dlg 2" font-size="9.75" font-weight="400" font-style="normal" > <text fill="#000000" fill-opacity="1" stroke="none" xml:space="preserve" x="4" y="17" font-family="Arial" font-size="10pt" font-weight="700" font-style="italic" >atp6</text> </g> <g fill="#ffff33" fill-opacity="1" stroke="#bfbfbf" stroke-opacity="1" stroke-width="1" stroke-linecap="square" stroke-linejoin="bevel" transform="matrix(1,0,0,1,112,1890)" font-family="MS Shell Dlg 2" font-size="9.75" font-weight="400" font-style="normal" > <rect x="0" y="0" width="50" height="20"/> </g> <g fill="none" stroke="#000000" stroke-opacity="1" stroke-width="1" stroke-linecap="square" stroke-linejoin="bevel" transform="matrix(1,0,0,1,121.5,1888)" font-family="MS Shell Dlg 2" font-size="9.75" font-weight="400" font-style="normal" > <text fill="#000000" fill-opacity="1" stroke="none" xml:space="preserve" x="4" y="17" font-family="Arial" font-size="10pt" font-weight="700" font-style="italic" >cytb</text> </g> <g fill="#ffff33" fill-opacity="1" stroke="#bfbfbf" stroke-opacity="1" stroke-width="1" stroke-linecap="square" stroke-linejoin="bevel" transform="matrix(1,0,0,1,163,1890)" font-family="MS Shell Dlg 2" font-size="9.75" font-weight="400" font-style="normal" > <rect x="0" y="0" width="50" height="20"/> </g> <g fill="none" stroke="#000000" stroke-opacity="1" stroke-width="1" stroke-linecap="square" stroke-linejoin="bevel" transform="matrix(1,0,0,1,171,1888)" font-family="MS Shell Dlg 2" font-size="9.75" font-weight="400" font-style="normal" > <text fill="#000000" fill-opacity="1" stroke="none" xml:space="preserve" x="4" y="17" font-family="Arial" font-size="10pt" font-weight="700" font-style="italic" >cox2</text> </g> <g fill="#ff9999" fill-opacity="1" stroke="#bfbfbf" stroke-opacity="1" stroke-width="1" stroke-linecap="square" stroke-linejoin="bevel" transform="matrix(1,0,0,1,214,1890)" font-family="MS Shell Dlg 2" font-size="9.75" font-weight="400" font-style="normal" > <path vector-effect="none" fill-rule="evenodd" d="M0,1.7 L42.5,1.7 L42.5,0 L50,10 L42.5,20 L42.5,18.3 L0,18.3 L0,1.7"/> </g> <g fill="none" stroke="#000000" stroke-opacity="1" stroke-width="1" stroke-linecap="square" stroke-linejoin="bevel" transform="matrix(1,0,0,1,227.132,1888)" font-family="MS Shell Dlg 2" font-size="9.75" font-weight="400" font-style="normal" > <text fill="#000000" fill-opacity="1" stroke="none" xml:space="preserve" x="4" y="17" font-family="Arial" font-size="10pt" font-weight="700" font-style="italic" >R</text> </g> <g fill="#ff9999" fill-opacity="1" stroke="#bfbfbf" stroke-opacity="1" stroke-width="1" stroke-linecap="square" stroke-linejoin="bevel" transform="matrix(1,0,0,1,265,1890)" font-family="MS Shell Dlg 2" font-size="9.75" font-weight="400" font-style="normal" > <path vector-effect="none" fill-rule="evenodd" d="M0,1.7 L42.5,1.7 L42.5,0 L50,10 L42.5,20 L42.5,18.3 L0,18.3 L0,1.7"/> </g> <g fill="none" stroke="#000000" stroke-opacity="1" stroke-width="1" stroke-linecap="square" stroke-linejoin="bevel" transform="matrix(1,0,0,1,277.132,1888)" font-family="MS Shell Dlg 2" font-size="9.75" font-weight="400" font-style="normal" > <text fill="#000000" fill-opacity="1" stroke="none" xml:space="preserve" x="4" y="17" font-family="Arial" font-size="10pt" font-weight="700" font-style="italic" >M</text> </g> <g fill="#ff9999" fill-opacity="1" stroke="#bfbfbf" stroke-opacity="1" stroke-width="1" stroke-linecap="square" stroke-linejoin="bevel" transform="matrix(1,0,0,1,316,1890)" font-family="MS Shell Dlg 2" font-size="9.75" font-weight="400" font-style="normal" > <path vector-effect="none" fill-rule="evenodd" d="M0,1.7 L42.5,1.7 L42.5,0 L50,10 L42.5,20 L42.5,18.3 L0,18.3 L0,1.7"/> </g> <g fill="none" stroke="#000000" stroke-opacity="1" stroke-width="1" stroke-linecap="square" stroke-linejoin="bevel" transform="matrix(1,0,0,1,329.132,1888)" font-family="MS Shell Dlg 2" font-size="9.75" font-weight="400" font-style="normal" > <text fill="#000000" fill-opacity="1" stroke="none" xml:space="preserve" x="4" y="17" font-family="Arial" font-size="10pt" font-weight="700" font-style="italic" >T</text> </g> <g fill="#ff9999" fill-opacity="1" stroke="#bfbfbf" stroke-opacity="1" stroke-width="1" stroke-linecap="square" stroke-

linejoin="bevel" transform="matrix(1,0,0,1,367,1890)" font-family="MS Shell Dlg 2" font-size="9.75" font-weight="400" font-style="normal" > <path vector-effect="none" fill-rule="evenodd" d="M0,1.7 L42.5,1.7 L42.5,0 L50,10 L42.5,20 L42.5,18.3 L0,18.3 L0,1.7"/> </g> <g fill="none" stroke="#000000" stroke-opacity="1" stroke-width="1" stroke-linecap="square" stroke-linejoin="bevel" transform="matrix(1,0,0,1,380.632,1888)" font-family="MS Shell Dlg 2" font-size="9.75" font-weight="400" font-style="normal" > <text fill="#000000" fill-opacity="1" stroke="none" xml:space="preserve" x="4" y="17" font-family="Arial" font-size="10pt" font-weight="700" font-style="italic" >E</text> </g> <g fill="#ff9999" fill-opacity="1" stroke="#bfbfbf" stroke-opacity="1" stroke-width="1" stroke-linecap="square" stroke-linejoin="bevel" transform="matrix(1,0,0,1,418,1890)" font-family="MS Shell Dlg 2" font-size="9.75" font-weight="400" font-style="normal" > <path vector-effect="none" fill-rule="evenodd" d="M0,1.7 L42.5,1.7 L42.5,0 L50,10 L42.5,20 L42.5,18.3 L0,18.3 L0,1.7"/> </g> <g fill="none" stroke="#000000" stroke-opacity="1" stroke-width="1" stroke-linecap="square" stroke-linejoin="bevel" transform="matrix(1,0,0,1,430.632,1888)" font-family="MS Shell Dlg 2" font-size="9.75" font-weight="400" font-style="normal" > <text fill="#000000" fill-opacity="1" stroke="none" xml:space="preserve" x="4" y="17" font-family="Arial" font-size="10pt" font-weight="700" font-style="italic" >Q</text> </g> <g fill="#ff9999" fill-opacity="1" stroke="#bfbfbf" stroke-opacity="1" stroke-width="1" stroke-linecap="square" stroke-linejoin="bevel" transform="matrix(1,0,0,1,469,1890)" font-family="MS Shell Dlg 2" font-size="9.75" font-weight="400" font-style="normal" > <path vector-effect="none" fill-rule="evenodd" d="M0,1.7 L42.5,1.7 L42.5,0 L50,10 L42.5,20 L42.5,18.3 L0,18.3 L0,1.7"/> </g> <g fill="none" stroke="#000000" stroke-opacity="1" stroke-width="1" stroke-linecap="square" stroke-linejoin="bevel" transform="matrix(1,0,0,1,482.132,1888)" font-family="MS Shell Dlg 2" font-size="9.75" font-weight="400" font-style="normal" > <text fill="#000000" fill-opacity="1" stroke="none" xml:space="preserve" x="4" y="17" font-family="Arial" font-size="10pt" font-weight="700" font-style="italic" >S</text> </g> <g fill="#ff9999" fill-opacity="1" stroke="#bfbfbf" stroke-opacity="1" stroke-width="1" stroke-linecap="square" stroke-linejoin="bevel" transform="matrix(1,0,0,1,520,1890)" font-family="MS Shell Dlg 2" font-size="9.75" font-weight="400" font-style="normal" > <path vector-effect="none" fill-rule="evenodd" d="M0,1.7 L42.5,1.7 L42.5,0 L50,10 L42.5,20 L42.5,18.3 L0,18.3 L0,1.7"/> </g> <g fill="none" stroke="#000000" stroke-opacity="1" stroke-width="1" stroke-linecap="square" stroke-linejoin="bevel" transform="matrix(1,0,0,1,533.133,1888)" font-family="MS Shell Dlg 2" font-size="9.75" font-weight="400" font-style="normal" > <text fill="#000000" fill-opacity="1" stroke="none" xml:space="preserve" x="4" y="17" font-family="Arial" font-size="10pt" font-weight="700" font-style="italic" >D</text> </g> <g fill="#ffff33" fill-opacity="1" stroke="#bfbfbf" stroke-opacity="1" stroke-width="1" stroke-linecap="square" stroke-linejoin="bevel" transform="matrix(1,0,0,1,571,1890)" font-family="MS Shell Dlg 2" font-size="9.75" font-weight="400" font-style="normal" > <rect x="0" y="0" width="50" height="20"/> </g> <g fill="none" stroke="#000000" stroke-opacity="1" stroke-width="1" stroke-linecap="square" stroke-linejoin="bevel" transform="matrix(1,0,0,1,578,1888)" font-family="MS Shell Dlg 2" font-size="9.75" font-weight="400" font-style="normal" > <text fill="#000000" fill-opacity="1" stroke="none" xml:space="preserve" x="4" y="17" font-family="Arial" font-size="10pt" font-weight="700" font-style="italic" >nad1</text> </g> <g fill="#ff9999" fill-opacity="1" stroke="#bfbfbf" stroke-opacity="1" stroke-width="1" stroke-linecap="square" stroke-linejoin="bevel" transform="matrix(1,0,0,1,622,1890)" font-family="MS Shell Dlg 2" font-size="9.75" font-weight="400" font-style="normal" > <path vector-effect="none" fill-rule="evenodd" d="M0,1.7 L42.5,1.7 L42.5,0 L50,10 L42.5,20 L42.5,18.3 L0,18.3 L0,1.7"/> </g> <g fill="none" stroke="#000000" stroke-opacity="1" stroke-width="1" stroke-linecap="square" stroke-linejoin="bevel" transform="matrix(1,0,0,1,635.633,1888)" font-family="MS Shell Dlg 2" font-size="9.75" font-weight="400" font-style="normal" > <text fill="#000000" fill-opacity="1" stroke="none" xml:space="preserve" x="4" y="17" font-family="Arial" font-size="10pt" font-weight="700" font-style="italic" >P</text> </g> <g fill="#ff9999" fill-opacity="1" stroke="#bfbfbf" stroke-opacity="1" stroke-width="1" stroke-linecap="square" stroke-linejoin="bevel" transform="matrix(1,0,0,1,673,1890)" font-family="MS Shell Dlg 2" font-size="9.75" font-weight="400" font-style="normal" > <path vector-effect="none" fill-rule="evenodd" d="M0,1.7 L42.5,1.7 L42.5,0 L50,10 L42.5,20 L42.5,18.3 L0,18.3 L0,1.7"/> </g> <g fill="none" stroke="#000000" stroke-opacity="1" stroke-width="1" stroke-linecap="square" stroke-linejoin="bevel" transform="matrix(1,0,0,1,686.133,1888)" font-family="MS Shell Dlg 2" font-size="9.75" font-weight="400" font-style="normal" > <text fill="#000000" fill-opacity="1" stroke="none" xml:space="preserve" x="4" y="17" font-family="Arial" font-size="10pt" font-weight="700" font-style="italic" >S</text> </g> <g fill="#ffff33" fill-opacity="1" stroke="#bfbfbf" stroke-opacity="1" stroke-width="1" stroke-linecap="square" stroke-linejoin="bevel" transform="matrix(1,0,0,1,724,1890)" font-family="MS Shell Dlg 2" font-size="9.75" font-weight="400" font-style="normal" > <rect x="0" y="0" width="50" height="20"/> </g> <g fill="none" stroke="#000000" stroke-opacity="1" stroke-width="1" stroke-linecap="square" stroke-linejoin="bevel" transform="matrix(1,0,0,1,728,1888)" font-family="MS Shell Dlg 2" font-size="9.75" font-weight="400" font-style="normal" > <text fill="#000000" fill-opacity="1" stroke="none"

xml:space="preserve" x="4" y="17" font-family="Arial" font-size="10pt" font-weight="700" font-style="italic" >nad4L</text> </g> <g fill="#ff9999" fill-opacity="1" stroke="#bfbfbf" stroke-opacity="1" stroke-width="1" stroke-linecap="square" stroke-linejoin="bevel" transform="matrix(1,0,0,1,775,1890)" font-family="MS Shell Dlg 2" font-size="9.75" font-weight="400" font-style="normal" > <path vector-effect="none" fill-rule="evenodd" d="M0,1.7 L42.5,1.7 L42.5,0 L50,10 L42.5,20 L42.5,18.3 L0,18.3 L0,1.7"/> </g> <g fill="none" stroke="#000000" stroke-opacity="1" stroke-width="1" stroke-linecap="square" stroke-linejoin="bevel" transform="matrix(1,0,0,1,788.633,1888)" font-family="MS Shell Dlg 2" font-size="9.75" font-weight="400" font-style="normal" > <text fill="#000000" fill-opacity="1" stroke="none" xml:space="preserve" x="4" y="17" font-family="Arial" font-size="10pt" font-weight="700" font-style="italic" >Y</text> </g> <g fill="#ffff33" fill-opacity="1" stroke="#bfbfbf" stroke-opacity="1" stroke-width="1" stroke-linecap="square" stroke-linejoin="bevel" transform="matrix(1,0,0,1,826,1890)" font-family="MS Shell Dlg 2" font-size="9.75" font-weight="400" font-style="normal" > <rect x="0" y="0" width="50" height="20"/> </g> <g fill="none" stroke="#000000" stroke-opacity="1" stroke-width="1" stroke-linecap="square" stroke-linejoin="bevel" transform="matrix(1,0,0,1,833,1888)" font-family="MS Shell Dlg 2" font-size="9.75" font-weight="400" font-style="normal" > <text fill="#000000" fill-opacity="1" stroke="none" xml:space="preserve" x="4" y="17" font-family="Arial" font-size="10pt" font-weight="700" font-style="italic" >nad3</text> </g> <g fill="#ff9999" fill-opacity="1" stroke="#bfbfbf" stroke-opacity="1" stroke-width="1" stroke-linecap="square" stroke-linejoin="bevel" transform="matrix(1,0,0,1,877,1890)" font-family="MS Shell Dlg 2" font-size="9.75" font-weight="400" font-style="normal" > <path vector-effect="none" fill-rule="evenodd" d="M0,1.7 L42.5,1.7 L42.5,0 L50,10 L42.5,20 L42.5,18.3 L0,18.3 L0,1.7"/> </g> <g fill="none" stroke="#000000" stroke-opacity="1" stroke-width="1" stroke-linecap="square" stroke-linejoin="bevel" transform="matrix(1,0,0,1,891.133,1888)" font-family="MS Shell Dlg 2" font-size="9.75" font-weight="400" font-style="normal" > <text fill="#000000" fill-opacity="1" stroke="none" xml:space="preserve" x="4" y="17" font-family="Arial" font-size="10pt" font-weight="700" font-style="italic" >L</text> </g> <g fill="#ff9999" fill-opacity="1" stroke="#bfbfbf" stroke-opacity="1" stroke-width="1" stroke-linecap="square" stroke-linejoin="bevel" transform="matrix(1,0,0,1,928,1890)" font-family="MS Shell Dlg 2" font-size="9.75" font-weight="400" font-style="normal" > <path vector-effect="none" fill-rule="evenodd" d="M0,1.7 L42.5,1.7 L42.5,0 L50,10 L42.5,20 L42.5,18.3 L0,18.3 L0,1.7"/> </g> <g fill="none" stroke="#000000" stroke-opacity="1" stroke-width="1" stroke-linecap="square" stroke-linejoin="bevel" transform="matrix(1,0,0,1,941.133,1888)" font-family="MS Shell Dlg 2" font-size="9.75" font-weight="400" font-style="normal" > <text fill="#000000" fill-opacity="1" stroke="none" xml:space="preserve" x="4" y="17" font-family="Arial" font-size="10pt" font-weight="700" font-style="italic" >N</text> </g> <g fill="#ffff33" fill-opacity="1" stroke="#bfbfbf" stroke-opacity="1" stroke-width="1" stroke-linecap="square" stroke-linejoin="bevel" transform="matrix(1,0,0,1,979,1890)" font-family="MS Shell Dlg 2" font-size="9.75" font-weight="400" font-style="normal" > <rect x="0" y="0" width="50" height="20"/> </g> <g fill="none" stroke="#000000" stroke-opacity="1" stroke-width="1" stroke-linecap="square" stroke-linejoin="bevel" transform="matrix(1,0,0,1,986,1888)" font-family="MS Shell Dlg 2" font-size="9.75" font-weight="400" font-style="normal" > <text fill="#000000" fill-opacity="1" stroke="none" xml:space="preserve" x="4" y="17" font-family="Arial" font-size="10pt" font-weight="700" font-style="italic" >nad6</text> </g> <g fill="#ff9999" fill-opacity="1" stroke="#bfbfbf" stroke-opacity="1" stroke-width="1" stroke-linecap="square" stroke-linejoin="bevel" transform="matrix(1,0,0,1,1030,1890)" font-family="MS Shell Dlg 2" font-size="9.75" font-weight="400" font-style="normal" > <path vector-effect="none" fill-rule="evenodd" d="M0,1.7 L42.5,1.7 L42.5,0 L50,10 L42.5,20 L42.5,18.3 L0,18.3 L0,1.7"/> </g> <g fill="none" stroke="#000000" stroke-opacity="1" stroke-width="1" stroke-linecap="square" stroke-linejoin="bevel" transform="matrix(1,0,0,1,1043.13,1888)" font-family="MS Shell Dlg 2" font-size="9.75" font-weight="400" font-style="normal" > <text fill="#000000" fill-opacity="1" stroke="none" xml:space="preserve" x="4" y="17" font-family="Arial" font-size="10pt" font-weight="700" font-style="italic" >C</text> </g> <g fill="#ff9999" fill-opacity="1" stroke="#bfbfbf" stroke-opacity="1" stroke-width="1" stroke-linecap="square" stroke-linejoin="bevel" transform="matrix(1,0,0,1,1081,1890)" font-family="MS Shell Dlg 2" font-size="9.75" font-weight="400" font-style="normal" > <path vector-effect="none" fill-rule="evenodd" d="M0,1.7 L42.5,1.7 L42.5,0 L50,10 L42.5,20 L42.5,18.3 L0,18.3 L0,1.7"/> </g> <g fill="none" stroke="#000000" stroke-opacity="1" stroke-width="1" stroke-linecap="square" stroke-linejoin="bevel" transform="matrix(1,0,0,1,1092.13,1888)" font-family="MS Shell Dlg 2" font-size="9.75" font-weight="400" font-style="normal" > <text fill="#000000" fill-opacity="1" stroke="none" xml:space="preserve" x="4" y="17" font-family="Arial" font-size="10pt" font-weight="700" font-style="italic" >W</text> </g> <g fill="#ff9999" fill-opacity="1" stroke="#bfbfbf" stroke-opacity="1" stroke-width="1" stroke-linecap="square" stroke-linejoin="bevel" transform="matrix(1,0,0,1,1132,1890)" font-family="MS Shell Dlg 2" font-size="9.75" font-weight="400" font-style="normal" > <path vector-effect="none" fill-rule="evenodd" d="M0,1.7 L42.5,1.7 L42.5,0 L50,10 L42.5,20 L42.5,18.3 L0,18.3 L0,1.7"/> </g> <g fill="none" stroke="#000000" stroke-opacity="1" stroke-width="1"

stroke-linecap="square" stroke-linejoin="bevel" transform="matrix(1,0,0,1,1145.63,1888)" font-family="MS Shell Dlg 2" font-size="9.75" font-weight="400" font-style="normal" > <text fill="#000000" fill-opacity="1" stroke="none" xml:space="preserve" x="4" y="17" font-family="Arial" font-size="10pt" font-weight="700" font-style="italic" >K</text> </g> <g fill="#ffff33" fill-opacity="1" stroke="#bfbfbf" stroke-opacity="1" stroke-width="1" stroke-linecap="square" stroke-linejoin="bevel" transform="matrix(1,0,0,1,1183,1890)" font-family="MS Shell Dlg 2" font-size="9.75" font-weight="400" font-style="normal" > <rect x="0" y="0" width="50" height="20"/> </g> <g fill="none" stroke="#000000" stroke-opacity="1" stroke-width="1" stroke-linecap="square" stroke-linejoin="bevel" transform="matrix(1,0,0,1,1190,1888)" font-family="MS Shell Dlg 2" font-size="9.75" font-weight="400" font-style="normal" > <text fill="#000000" fill-opacity="1" stroke="none" xml:space="preserve" x="4" y="17" font-family="Arial" font-size="10pt" font-weight="700" font-style="italic" >nad5</text> </g> <g fill="#ff9999" fill-opacity="1" stroke="#bfbfbf" stroke-opacity="1" stroke-width="1" stroke-linecap="square" stroke-linejoin="bevel" transform="matrix(1,0,0,1,1234,1890)" font-family="MS Shell Dlg 2" font-size="9.75" font-weight="400" font-style="normal" > <path vector-effect="none" fill-rule="evenodd" d="M0,1.7 L42.5,1.7 L42.5,0 L50,10 L42.5,20 L42.5,18.3 L0,18.3 L0,1.7"/> </g> <g fill="none" stroke="#000000" stroke-opacity="1" stroke-width="1" stroke-linecap="square" stroke-linejoin="bevel" transform="matrix(1,0,0,1,1247.13,1888)" font-family="MS Shell Dlg 2" font-size="9.75" font-weight="400" font-style="normal" > <text fill="#000000" fill-opacity="1" stroke="none" xml:space="preserve" x="4" y="17" font-family="Arial" font-size="10pt" font-weight="700" font-style="italic" >A</text> </g> <g fill="#ff9999" fill-opacity="1" stroke="#bfbfbf" stroke-opacity="1" stroke-width="1" stroke-linecap="square" stroke-linejoin="bevel" transform="matrix(1,0,0,1,1285,1890)" font-family="MS Shell Dlg 2" font-size="9.75" font-weight="400" font-style="normal" > <path vector-effect="none" fill-rule="evenodd" d="M0,1.7 L42.5,1.7 L42.5,0 L50,10 L42.5,20 L42.5,18.3 L0,18.3 L0,1.7"/> </g> <g fill="none" stroke="#000000" stroke-opacity="1" stroke-width="1" stroke-linecap="square" stroke-linejoin="bevel" transform="matrix(1,0,0,1,1299.13,1888)" font-family="MS Shell Dlg 2" font-size="9.75" font-weight="400" font-style="normal" > <text fill="#000000" fill-opacity="1" stroke="none" xml:space="preserve" x="4" y="17" font-family="Arial" font-size="10pt" font-weight="700" font-style="italic" >L</text> </g> <g fill="#99ffff" fill-opacity="1" stroke="#bfbfbf" stroke-opacity="1" stroke-width="1" stroke-linecap="square" stroke-linejoin="bevel" transform="matrix(1,0,0,1,1336,1890)" font-family="MS Shell Dlg 2" font-size="9.75" font-weight="400" font-style="normal" > <path vector-effect="none" fill-rule="evenodd" d="M0,5 C0,2.23858 2.23858,0 5,0 L45,0 C47.7614,0 50,2.23858 50,5 L50,15 C50,17.7614 47.7614,20 45,20 L5,20 C2.23858,20 0,17.7614 0,15 L0,5"/> </g> <g fill="none" stroke="#000000" stroke-opacity="1" stroke-width="1" stroke-linecap="square" stroke-linejoin="bevel" transform="matrix(1,0,0,1,1344.5,1888)" font-family="MS Shell Dlg 2" font-size="9.75" font-weight="400" font-style="normal" > <text fill="#000000" fill-opacity="1" stroke="none" xml:space="preserve" x="4" y="17" font-family="Arial" font-size="10pt" font-weight="700" font-style="italic" >rrnS</text> </g> <g fill="#ff9999" fill-opacity="1" stroke="#bfbfbf" stroke-opacity="1" stroke-width="1" stroke-linecap="square" stroke-linejoin="bevel" transform="matrix(1,0,0,1,1387,1890)" font-family="MS Shell Dlg 2" font-size="9.75" font-weight="400" font-style="normal" > <path vector-effect="none" fill-rule="evenodd" d="M0,1.7 L42.5,1.7 L42.5,0 L50,10 L42.5,20 L42.5,18.3 L0,18.3 L0,1.7"/> </g> <g fill="none" stroke="#000000" stroke-opacity="1" stroke-width="1" stroke-linecap="square" stroke-linejoin="bevel" transform="matrix(1,0,0,1,1402.13,1888)" font-family="MS Shell Dlg 2" font-size="9.75" font-weight="400" font-style="normal" > <text fill="#000000" fill-opacity="1" stroke="none" xml:space="preserve" x="4" y="17" font-family="Arial" font-size="10pt" font-weight="700" font-style="italic" >I</text> </g> <g fill="#ff9999" fill-opacity="1" stroke="#bfbfbf" stroke-opacity="1" stroke-width="1" stroke-linecap="square" stroke-linejoin="bevel" transform="matrix(1,0,0,1,1438,1890)" font-family="MS Shell Dlg 2" font-size="9.75" font-weight="400" font-style="normal" > <path vector-effect="none" fill-rule="evenodd" d="M0,1.7 L42.5,1.7 L42.5,0 L50,10 L42.5,20 L42.5,18.3 L0,18.3 L0,1.7"/> </g> <g fill="none" stroke="#000000" stroke-opacity="1" stroke-width="1" stroke-linecap="square" stroke-linejoin="bevel" transform="matrix(1,0,0,1,1451.13,1888)" font-family="MS Shell Dlg 2" font-size="9.75" font-weight="400" font-style="normal" > <text fill="#000000" fill-opacity="1" stroke="none" xml:space="preserve" x="4" y="17" font-family="Arial" font-size="10pt" font-weight="700" font-style="italic" >G</text> </g> <g fill="#ff9999" fill-opacity="1" stroke="#bfbfbf" stroke-opacity="1" stroke-width="1" stroke-linecap="square" stroke-linejoin="bevel" transform="matrix(1,0,0,1,1489,1890)" font-family="MS Shell Dlg 2" font-size="9.75" font-weight="400" font-style="normal" > <path vector-effect="none" fill-rule="evenodd" d="M0,1.7 L42.5,1.7 L42.5,0 L50,10 L42.5,20 L42.5,18.3 L0,18.3 L0,1.7"/> </g> <g fill="none" stroke="#000000" stroke-opacity="1" stroke-width="1" stroke-linecap="square" stroke-linejoin="bevel" transform="matrix(1,0,0,1,1502.13,1888)" font-family="MS Shell Dlg 2" font-size="9.75" font-weight="400" font-style="normal" > <text fill="#000000" fill-opacity="1" stroke="none" xml:space="preserve" x="4" y="17" font-family="Arial" font-size="10pt" font-weight="700" font-style="italic" >V</text> </g> <g fill="#99ffff" fill-opacity="1" stroke="#bfbfbf" stroke-opacity="1"

stroke-width="1" stroke-linecap="square" stroke-linejoin="bevel" transform="matrix(1,0,0,1,1540,1890)" font-family="MS Shell Dlg 2" font-size="9.75" font-weight="400" font-style="normal" > <path vector-effect="none" fill-rule="evenodd" d="M0,5 C0,2.23858 2.23858,0 5,0 L45,0 C47.7614,0 50,2.23858 50,5 L50,15 C50,17.7614 47.7614,20 45,20 L5,20 C2.23858,20 0,17.7614 0,15 L0,5"/> </g> <g fill="none" stroke="#000000" stroke-opacity="1" stroke-width="1" stroke-linecap="square" stroke-linejoin="bevel" transform="matrix(1,0,0,1,1549.5,1888)" font-family="MS Shell Dlg 2" font-size="9.75" font-weight="400" font-style="normal" > <text fill="#000000" fill-opacity="1" stroke="none" xml:space="preserve" x="4" y="17" font-family="Arial" font-size="10pt" font-weight="700" font-style="italic" >rrnL</text> </g> <g fill="#ff9999" fill-opacity="1" stroke="#bfbfbf" stroke-opacity="1" stroke-width="1" stroke-linecap="square" stroke-linejoin="bevel" transform="matrix(1,0,0,1,1591,1890)" font-family="MS Shell Dlg 2" font-size="9.75" font-weight="400" font-style="normal" > <path vector-effect="none" fill-rule="evenodd" d="M0,1.7 L42.5,1.7 L42.5,0 L50,10 L42.5,20 L42.5,18.3 L0,18.3 L0,1.7"/> </g> <g fill="none" stroke="#000000" stroke-opacity="1" stroke-width="1" stroke-linecap="square" stroke-linejoin="bevel" transform="matrix(1,0,0,1,1603.13,1888)" font-family="MS Shell Dlg 2" font-size="9.75" font-weight="400" font-style="normal" > <text fill="#000000" fill-opacity="1" stroke="none" xml:space="preserve" x="4" y="17" font-family="Arial" font-size="10pt" font-weight="700" font-style="italic" >M</text> </g> <g fill="#ff9999" fill-opacity="1" stroke="#bfbfbf" stroke-opacity="1" stroke-width="1" stroke-linecap="square" stroke-linejoin="bevel" transform="matrix(1,0,0,1,1642,1890)" font-family="MS Shell Dlg 2" font-size="9.75" font-weight="400" font-style="normal" > <path vector-effect="none" fill-rule="evenodd" d="M0,1.7 L42.5,1.7 L42.5,0 L50,10 L42.5,20 L42.5,18.3 L0,18.3 L0,1.7"/> </g> <g fill="none" stroke="#000000" stroke-opacity="1" stroke-width="1" stroke-linecap="square" stroke-linejoin="bevel" transform="matrix(1,0,0,1,1655.63,1888)" font-family="MS Shell Dlg 2" font-size="9.75" font-weight="400" font-style="normal" > <text fill="#000000" fill-opacity="1" stroke="none" xml:space="preserve" x="4" y="17" font-family="Arial" font-size="10pt" font-weight="700" font-style="italic" >F</text> </g> <g fill="#ff9999" fill-opacity="1" stroke="#bfbfbf" stroke-opacity="1" stroke-width="1" stroke-linecap="square" stroke-linejoin="bevel" transform="matrix(1,0,0,1,1693,1890)" font-family="MS Shell Dlg 2" font-size="9.75" font-weight="400" font-style="normal" > <path vector-effect="none" fill-rule="evenodd" d="M0,1.7 L42.5,1.7 L42.5,0 L50,10 L42.5,20 L42.5,18.3 L0,18.3 L0,1.7"/> </g> <g fill="none" stroke="#000000" stroke-opacity="1" stroke-width="1" stroke-linecap="square" stroke-linejoin="bevel" transform="matrix(1,0,0,1,1706.13,1888)" font-family="MS Shell Dlg 2" font-size="9.75" font-weight="400" font-style="normal" > <text fill="#000000" fill-opacity="1" stroke="none" xml:space="preserve" x="4" y="17" font-family="Arial" font-size="10pt" font-weight="700" font-style="italic" >H</text> </g> <g fill="#ffff33" fill-opacity="1" stroke="#bfbfbf" stroke-opacity="1" stroke-width="1" stroke-linecap="square" stroke-linejoin="bevel" transform="matrix(1,0,0,1,1744,1890)" font-family="MS Shell Dlg 2" font-size="9.75" font-weight="400" font-style="normal" > <rect x="0" y="0" width="50" height="20"/> </g> <g fill="none" stroke="#000000" stroke-opacity="1" stroke-width="1" stroke-linecap="square" stroke-linejoin="bevel" transform="matrix(1,0,0,1,1752,1888)" font-family="MS Shell Dlg 2" font-size="9.75" font-weight="400" font-style="normal" > <text fill="#000000" fill-opacity="1" stroke="none" xml:space="preserve" x="4" y="17" font-family="Arial" font-size="10pt" font-weight="700" font-style="italic" >cox3</text> </g> <g fill="#ffff33" fill-opacity="1" stroke="#bfbfbf" stroke-opacity="1" stroke-width="1" stroke-linecap="square" stroke-linejoin="bevel" transform="matrix(1,0,0,1,1795,1890)" font-family="MS Shell Dlg 2" font-size="9.75" font-weight="400" font-style="normal" > <rect x="0" y="0" width="50" height="20"/> </g> <g fill="none" stroke="#000000" stroke-opacity="1" stroke-width="1" stroke-linecap="square" stroke-linejoin="bevel" transform="matrix(1,0,0,1,1802,1888)" font-family="MS Shell Dlg 2" font-size="9.75" font-weight="400" font-style="normal" > <text fill="#000000" fill-opacity="1" stroke="none" xml:space="preserve" x="4" y="17" font-family="Arial" font-size="10pt" font-weight="700" font-style="italic" >nad2</text> </g> <g fill="#ffff33" fill-opacity="1" stroke="#bfbfbf" stroke-opacity="1" stroke-width="1" stroke-linecap="square" stroke-linejoin="bevel" transform="matrix(1,0,0,1,1846,1890)" font-family="MS Shell Dlg 2" font-size="9.75" font-weight="400" font-style="normal" > <rect x="0" y="0" width="50" height="20"/> </g> <g fill="none" stroke="#000000" stroke-opacity="1" stroke-width="1" stroke-linecap="square" stroke-linejoin="bevel" transform="matrix(1,0,0,1,1853,1888)" font-family="MS Shell Dlg 2" font-size="9.75" font-weight="400" font-style="normal" > <text fill="#000000" fill-opacity="1" stroke="none" xml:space="preserve" x="4" y="17" font-family="Arial" font-size="10pt" font-weight="700" font-style="italic" >nad4</text> </g> <g fill="#ffff33" fill-opacity="1" stroke="#bfbfbf" stroke-opacity="1" stroke-width="1" stroke-linecap="square" stroke-linejoin="bevel" transform="matrix(1,0,0,1,1897,1890)" font-family="MS Shell Dlg 2" font-size="9.75" font-weight="400" font-style="normal" > <rect x="0" y="0" width="50" height="20"/> </g> <g fill="none" stroke="#000000" stroke-opacity="1" stroke-width="1" stroke-linecap="square" stroke-linejoin="bevel" transform="matrix(1,0,0,1,1905.5,1888)" font-family="MS Shell Dlg 2" font-size="9.75" font-weight="400" font-style="normal" > <text fill="#000000" fill-opacity="1" stroke="none" xml:space="preserve" x="4" y="17" font-

family="Arial" font-size="10pt" font-weight="700" font-style="italic" >atp8</text> </g> <g fill="none" stroke="#000000" stroke-opacity="1" stroke-width="1" stroke-linecap="square" stroke-linejoin="bevel" transform="matrix(1,0,0,1,1948,1890)" font-family="MS Shell Dlg 2" font-size="9.75" font-weight="400" font-style="normal" > <text fill="#000000" fill-opacity="1" stroke="none" xml:space="preserve" x="4" y="17" font-family="Arial" font-size="10pt" font-weight="700" font-style="italic" >(+)</text> </g> </g> </svg>
