# Supplementary material for: Structure, Evolution, and Mitochondrial Genome Analysis of Mussel Species (Bivalvia, Mytilidae)
Source: Int J Mol Sci. 2024 Jun 24;25(13):6902. doi: 10.3390/ijms25136902 (PMC11241113; doi:10.3390/ijms25136902)

Figure S3. The phylogram built by SP PhyloSiut and its IQ-TREE utility for gene tree reconstruction based on 12 PCG-sequences and a combined of rRNA and tRNA nucleotide sequences of 26 analyzed mussels of the family Mytilidae. Bootstrap support scores for IQ-TREE performed in the three modes, (i) the SH-aLRT branch test in n=5000 replications, (ii) Bayes estimation of the nodes' probability (n=2000) and (iii) the Ultrafast Bootstrap ML in n=5000 replications. They are implemented besides nodes with slash in the order that given in previous sentence. The scale below phylogram shows the branch length. File upload from the folder: 2024_02_17-17_15_47.5000SH-aLRT+BI+UML-GTR+I+FG4-12PCG+rRNA_tRNA-rot26sq-rt.tre. The title of the folder explains briefly the content of the analysis made.

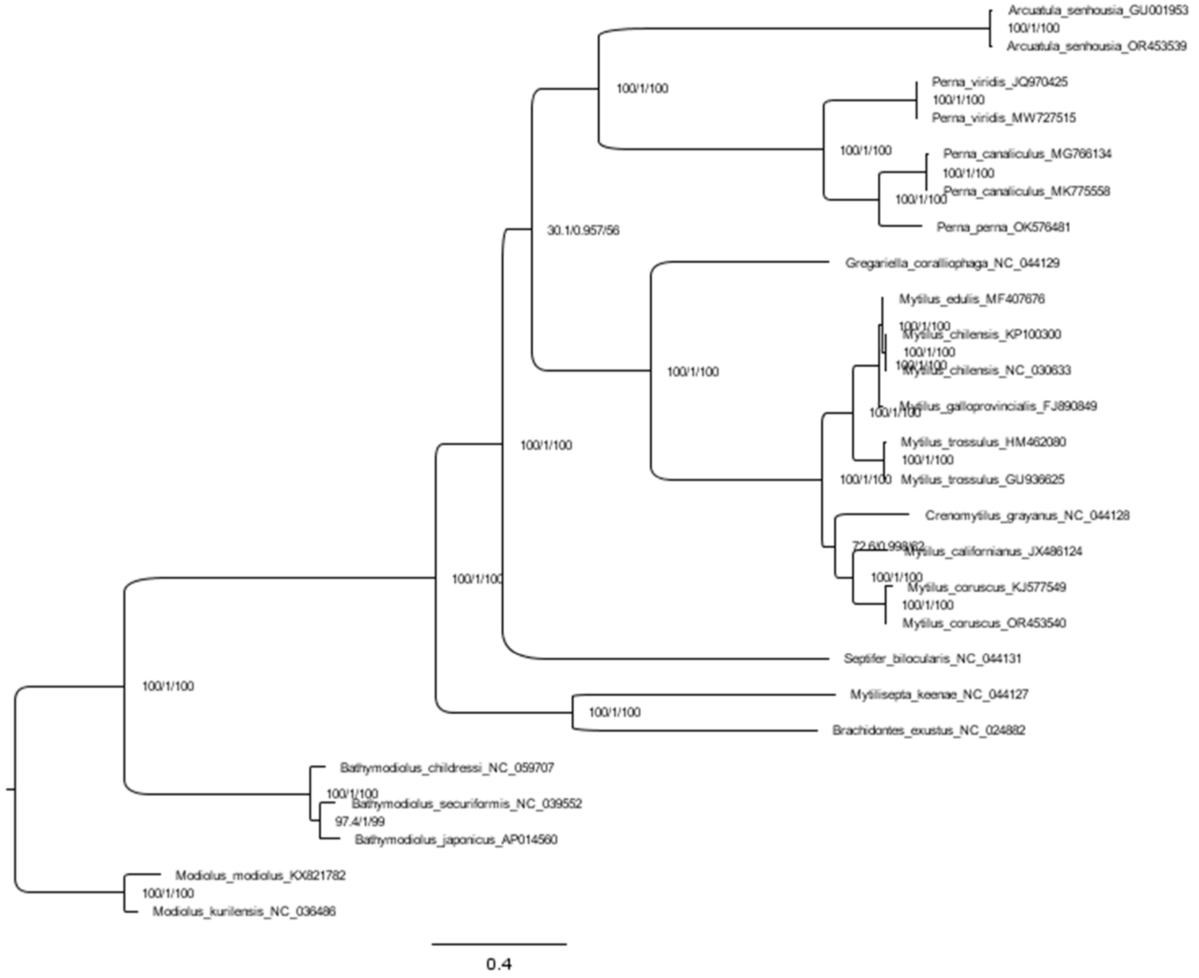

Supplement: Supplementary file 1 [file ijms-25-06902-s001.zip › Figure S3.IQ_partition.txt.treefile-5000SH-aLRT+BI+UML-GTR+I+FG4-12PCG+rRNA_tRNA-rot26sq-rt.tre-png.pdf]
