# Supplementary material for: Structure, Evolution, and Mitochondrial Genome Analysis of Mussel Species (Bivalvia, Mytilidae)
Source: Int J Mol Sci. 2024 Jun 24;25(13):6902. doi: 10.3390/ijms25136902 (PMC11241113; doi:10.3390/ijms25136902)
Supplement: Supplementary file 1 [file ijms-25-06902-s001.zip › Table S1.List of mitogenome squences-v2.pdf]

Table S1. Species list used in the study with the GenBank accession numbers

Ordering No	Species	GenBank Number
1	***Arcuatula senhousia-Ar1***	***OR453538***
2	***Arcuatula senhousia-Ar2***	***OR453539***
3	***Mytilus coruscus-Kart1***	***OR453540***
4	***Mytilus coruscus-Kart3***	***OR453541***
5	*Mytilus californianus F**	*JX486124*
6	*Mytilus coruscus*	*KJ577549*
7	*Mytilus chilensis F*	*KT966847*
8	*Mytilus chilensis*	*KP100300*
9	*Mytilus chilensis*	*NC030633*
10	*Mytilus edulis F*	*MF407676*
11	*Mytilus edulis platensis*	*KP100301*
12	*Mytilus galloprovincialis F*	*FJ890849*
13	*Mytilus galloprovincialis*	*AY497292*
14	*Mytilus galloprovincialis*	*DQ399833*
15	*Mytilus trossulus F*	*GU936625*
16	*Mytilus trossulus F*	*HM462080*
17	*Arcuatula (Musculista) senhousia*	*GU001953*
18	*Bathymodiolus childressi*	*NC059707*
19	*Bathymodiolus japonicus*	*AP014560*
20	*Bathymodiolus securiformis*	*NC039552*
21	*Brachidontes_exustus*	*KM233636*
22	*Coelomactra antiquata*	*JQ423460*
23	*Coelomactra antiquata*	*KC503290*
24	*Coelomactra antiquata*	*KC50329*
25	*Crenomytilus grayanus*	*NC044128*
26	*Gregariella coralliophaga*	*NC044129*
27	*Mactra chinensis*	*KJ754823*
28	*Mactra chinensis*	*NC025510*
29	*Modiolus kurilensis*	*KY242717*
30	*Modiolus modiolus*	*KX821782*
31	*Mytella strigata*	*NC056377*
32	*Mytilisepta keenae*	*NC044127*
33	*Mytilisepta virgata*	*KX094521*
34	*Perna canaliculus*	*MG766134*
35	*Perna canaliculus*	*MK775558*
36	*Perna perna*	*OK576481*
37	*Perna_viridis*	*JQ970425*
38	*Perna viridis*	*MW727515*
39	*Septifer bilocularis*	*MK721549*

Note. Letter F that given beside species name means attribution to female of the mussel observed in the original data source in GenBank.
