# Supplementary material for: Structure, Evolution, and Mitochondrial Genome Analysis of Mussel Species (Bivalvia, Mytilidae)
Source: Int J Mol Sci. 2024 Jun 24;25(13):6902. doi: 10.3390/ijms25136902 (PMC11241113; doi:10.3390/ijms25136902)
Supplement: Supplementary file 1 [file ijms-25-06902-s001.zip › Table S2a.An example file for the set 27 mitogenomes used in BEAST.pdf]

Table S2. An example file for the set 27 mitogenome sequence of the family Mytilidae
(File: Mytilidae27sq-no_pat-pt123ps.xml)

<?xml version="1.0" encoding="UTF-8" standalone="no"?><beast beautitemplate='Standard' beautistatus=" namespace="beast.core:beast.evolution.alignment:beast.evolution.tree.coalescent:beast.core.util:beast.evolution.nuc:beast.evolution.operators:beast.evolution.sitemodel:beast.evolution.substitutionmodel:beast.evolution.likelihood" required="" version="2.6">

<data
id="Mytilidae27sq12PCGs123ps"
spec="Alignment">
<sequence id="seq_Mytilus_californianusF-JX486124" spec="Sequence"
taxon="Mytilus_californianusF-JX486124" totalcount="4"
value="ATGTTAATAGATGTTTTTCTAGGTTTGATGCTCATAGTTACAACTTGATTTGGTTATCTATGTTGT
GGGCGCTTTCGTCTATGGTACCTTTAACAGTGTTGTTTAGGGATGTGAGAATTCGAAGTTTTGTTTTATCC
TTTACTTATTCAATGATTCGCAATGGAAAAGGGCTTAAACTTTCTGGGTTTCCGATGGTAATAAGTGGGT
TGTTTCTAATAATTTTAATATTGAATCTATCTGGGAACTTTCCATTTTTCTTCCCTGTAAGGGGCCAGTTTG
TGTTTGGGTTTTCTTTTGCTTTATCTATTTGAACTTGTTTAGTGCTATCTAGGTTGTTGTGTAGGTTTGAGC
AAAGATTAATAAGGTTAGTGCCAACGGGGCCTTTAATTTGGTACCATTTATAGTAGTGGTTGAATTAATT
AGAGGTATGCTTCGCCCTTTAACTTTGGTTTTACGTTTAACATTAAATCTTGGAGCTGGTAAAGTAATTTT
AACAATATGTAGGAGTGAGTTAGTAGTGGGTTGGTTAATTATAGGGGTAGGAGGGGTCAAAGGCCTCTT
GATAGGTGGAGTCTTTGCCGCTGAAGTTGCAATCGCCTGTATTCAATGCTATATTTTCTGTGTACTTTTAT
GTCTTTATACTGATGACCATAGAAGTTAACGTTGACTTTGGTCAACAAATCATAAAGATATTGGAACCCT
TTATCTATATAGAGGGGTGTGAGGAGGTTTATTTGGGGCAAGATTGAGTTTGATAATTATGCAAGGGCATC
CTGGAGCAGTCTTCTTAAAAGATTGATTCTATAATGTGGTTGTTACAACACATGCTTTAATAATAATTTTCT
TTGCTGTAATACCAATTTTAATCGGGGCTTTTGGTAATTGGCTTATTCCTTTGCTTGTAGGGGGAAAAGAC
ATAATCTATCCACGTATAAATAATTTGAGATATTGACTGTCGCCAAATGCACTATATTTATTAATGCTATCTT
TTAGAACAGATAAAGGAGTAGGGGCAGGATGGACTATTTATCCACCTCTATCTGTATACCCTTATCACAG
AGGGCCTAGGATAGATGTTCTTATTGTGTCTTTACACTTAGCAGGACTTAGCTCTCTAGTGGGGGCAATT
AATTTGCTAGCACAAATAAAAATATACCAGTATTAGAAATAAAAGGGGAACGGGCGGAGCTCTATGTGT
TAAGAATTAGGGTTACTGCAGTTCTTTTAATTATCTCAATTCCAGTGTTAGGAGGGGGTATCACAATAATT
CTGTTTGATCGTAACTTTAACACTACTTTCTTTGACCCTGCAGGGGGGGGTGACCCAGTACTATTTCAGC
ATTTGTTCTGGTTCTTTGGTCATCCTGAAGTATATATTCTTATTTTGCCTGCTTTTGGGGTAATGTCAAAGG
TAATTATGCATTGTTCTGGAAAAGAAGCAGTGTTTGGGCTAATTGGGATGGTTTATGCTATGATTGGAATT
GGAGGTTTAGGTTGTATGGTGTGAGCACATCATATATTTACGGTAGGTCTTAATGTTGATACTCGAGGGTA
CTTTTCTACTGCTACTATAGTAATTGCGGTCCCTACCGGGGTAAAGGTGTTTAGCTGACTAGCAACTATAG
CGGGTAGTAAGTTTAAAATAAAGCCGGCTGCCTTTTGAAGGACAGGATTCTTGTTTTTATTTACTGTAGG
GGGATTAACTGGGGTGATGTTGTCAAGTGCATCAATAGATGTGTCGCTTCATGATACCTATTACGTAGTAG
CCCATTTCCATTATGTACTTAGAATAGGAGCTGTGTTTGGGGTTTTTTGTGGTTTAAACCACTGACTACCC
AATTTGTTGGAGTCTGCTTTAATAAGAAATGAAGGAAAGCCCATTTTATAGCTATATTTTTTGGAGTAAA
TACTACGTTTTTCCCACAACACTTTTTGGGGTTAAGAGGTATGCCACGGCGGTATATAGACTATGCTGATA
TTTATGCTCATTGGCATTGAGTGTCCTCATATGGGTCTGCTGTGTCATTTGGTTCTCTTATATACTTTAAGT
TTTTACTCTGGGAAGCTTTAGTAAGCCAGCGAGGAATGTCTTTTTATGGGAGTCGATATTTTGGTGATATT
GTTCATGAATTAGGAAAAGACCTTTTTCGTTATCATGGCTTTGTAATGATGGTGGCGGTAGCTGTGCTAG
TCTTTGTGATATATATAGGGTGTGTTATTCTCCTTACTAAGTTTCCTATCGTCATTTTTTGAATCGTCAACG
ATTAGAGTTTTGATGGACAATCGTGCCAATACTGCTACTAGTAGGGTTATGGTTCCCGTCAATAATTAATT
TGTATTATATAGAAGAAGTGAAGCGGCCACGTTGAAATTTTAAAGCAATTGGGAAACAATGATATTGATC
TTACGAATGCGACTCCTGTTATACCATTGATTCCTATATAGAAGATCAACAAGAGACTGGTTATCGATTAT
TGGATGTTGATAATCGGATGGTTGCTCCTGCGGATGTACAAATAACTGCTTTTGTCAGTAGTTCAGATGTT
CTTCATTCGTTTGCTCTTCCTAAACTGTTACTTAAAGTGGACGCCATTCCGGGTCGAATTAATCGACTTCC
AATGAAGGCATCCCCAATGCAGTATTTATGGGCAATGTTCTGAAAATTTGTGGGGGTGAATCATAGGTTT
ATGCCTATCGTAATTGAGTTTATCCCTGAAAAATATTTTGTTATGTGATTAGAGGCTTTAAATTAA------------
----
ATGAAACGTAATCCTTACTATGTGCCTGGTCCAAGGCCATGGCCATTTTTTGTTGCTATTTCTGCAAATGG
TATGGCGGTAGGCTTAATTTTATGACTGCATCGCACACCTTTTTTGCTTATGGGCAGATTAGGTTGTATGC
TATTAAGAACTTTTAGTTGATGACGAGATTTAATTCGGGAGGGAGATATTGGGTTTCATACCCGTTTTGTT
ATTAAAAGATTTCGAGATGGGGTAGCGTTATTTATTTTGTCAGAGGTGATATTCTTCTTTTCCTTTTTTTGA
ACCTTTTTCCACAACGCCTTAAGTCCATCCTGTGAGTTGGGAATACGGTGACCCCCTCCAGGAATCCGG
ACGCCAAACCCGTCTTCTACGAGATTGTTTGAGACAGGGTTGCTTATTAGAAGGGGCTTATTTGTGACTC
AAGCCCATAAAAGAATACGGCTAGATTATGATGTGGGTCCGTTTGTTGGATTGGTGGTAACGATTTTGTG
TGGTACTTTGTTTTTCTTAGTGCAGCTACGGGAATACTATTGAAATTCTTATACTATTGCAGACAGGGTAT
ACGGAAGAGTGTTCTATCTGTTAACGGGGTTCCATGGGATGCATGTAGTTGTGGGAACACTCTGGTTAAT

AGTAAGACTGGTGCGTTTGTGGCGGGGAGAATTTTCTAGACAACGGCATTTTGGGTTTGAGGCTTGTAT
TTGGTACTGACATTTTGTAGATGTAGTCTGAGTTCGACTATGGTGCTTAGTGTATGTCTGATTTGGTGGGC
CGTGACGAAAAACTAATAAGCTGGTAAAGATCATAAATGATAGTTTCTACGACTTACCGTGTCCTGTTAA
CTTAAACGCTTGATGAAGGTTTGGCTCTATATTGGGTTTATGCCTTGTAATTCAGCTTTTAAGGGGGCTTT
TACTATCAATTCACTATACGGCTCATGAAGATATAGCTTTTGACTCTGTTGTGCATATTATGCGTAATGTGA
AAAAAGGCTGAATATTGCGAAACATCCATGCTAATGGTTCTTCTATATTTTTTATCTGTATTTACGCTCATA
TTGCTCGTGGGCTTTATTATGGGTCATATTTGGATAAGACAGTTTGGTACTTTGGGGTACATCTTTTTTTAC
TAACGATAGCTGAAGCCTTTCTAGGTTACACGTTACCATGAGGCCAAATATCCTATTGAGGGGCCACTGT
TATCACTAATATACTCAGGGTAATCCCTGTAGTAGGGGAGAGAATACTTCGTTATGTATGAGGTGGTTGGA
CAGTCTGTAATGCAACGTTAAAGCGATTTTATACACTTCATTTTCTTCTTCCGTTTGTAATAGTGGCGGTT
GTATTTCTTCATCTTTTCTTTTTACACGAAAAAGGAAGTAATAATCCTTTGGGGATTGAAAGTGACACTG
TGTGTGTCCCTTTTCACCCTTTTTATACTATTAAGGATTTATTTGGGTACGTTTGCTTTAGGTTTTTTTTTAT
ATATTTAGTATGTGTAGATCCTGAGTTATTGGGTAATCATTTGAACTATTGGCCTGCTAATCCGATAAAAAC
ACCTATTCATGTTCAGCCTGAATGGTACTTTATATTTGCTTATGCAATTCTTCGGTCTATCCCTCACAAGGC
AGGTGGTGTTTATATTATATTTTTATCTATTGTAGTATTATATCTTATTCCTAGTTTGCATAGGGGTAAGTATC
GAAGATTGTGTTTTTACCCATTCAATCAAGTCGTGTTCTGAGTTTTAGTAGGAAGATTTATTAGATTAACG
TGAATTGGGGCTCGCCCAGTTCGTGAGCCGTATATTGTGATAGGCCAGTGTTTCTCAATTATTTACTTCTC
AAGATTACTATTAAACCCCCTTTCATTATGGATGTGGGATAAGCTATTGGACGTGGGGGTAGTGATTGGG
GTTGTACCTTTTGTTGGTGTACTACTTGCTGTAGGATTTTATACGTTACTGGAGCGAAAAATTTTAGCTAT
TATCATAATTCGTAAAGGGCCGTCTAAAGTTAGTTATATAGGGATTTTGCAGCCGTTTAGAGATGCTGGCA
AGTTATTATGTAAGGAATTTATTATGCCTACACGGGCTAACATAGGGCCCTTTATTCTGGCCCCTGCGTTG
ATGTTAACCGTAAGACTTTTAGGGTGACTCTTATACCCTTATAAATCAGCGGAAGTGTTTTATGTTTTTGG
GGTAGTCCTTTTTATAGTGATTACTAGAGTGAGAGTATATGGAGTAATAATGTCTGGGTGGGCTTCGAATT
CTAAGTATTCATTGTTAGGTGCAGTTCGTGCAATAGCACAAAGGATCTCTTATGAGATTCCAATAGGGTTT
ATTTTTTTTTGTGTAGTTCTAGCTTCTGGTGTATTTATGTTTCAGGAAATTAGGGTATCATTTTTTTTCTTCC
CTTTATCTATTATTCTTGTTGTATGAATCCTGTGTATATTAGCAGAAACTAATCGTGCCCCTTTTGATTTTGT
AGAGGGAGAGTCTGAGTTAGTGTCTGGGTATAATGTAGAGTACAGGGGAGGTGGCTTCGCAGTGATGTT
CATTGCGGAGTACTCTAGAATTCTTCTTAGAAGAGTAATAAGGGCTGCAATATTCTTCGGTGGGAATGAG
GCGCTAATTGGGATTTTCATAATGGTTTTTGCAGTCTTTTTTGTAGTTGTACGTGCTTCTCTACCCCGTTTA
CGTTATGATAAGTTAATGAGCCTGTGTTGAACTGTGCTCTTATGTGTTATACTTATGGCTAGAGTATGTGTA
GTAATGTTAATTAGGGTGTAATGGTAAGTTTAGTAATAAGGCCAATAAAGTTAATGAGGATAAGATTGATG
GTTATTGGGACAGTGCTTAGGGTTAGAAGGGAAGAGTTGGTAGGTGTCTGGTTAGGAATGGAGTTAAAT
CTTTATGGTTTCCTTGTAATTATAAATCCGGATGGTCATTACATTCCAGAACCTTGTGTTAAATACTTTGTA
GTGCAAAGAACTGGGTCTATTTTAATGTTAGGAGGCTTCGTGTTGTTGATGCAGTTTGTAGCTGGTGGGC
TAATAATAAGAGTGGTTGGAACGGTGCTAAAGTCTGGAATTTCTCCTTTACACTCTTGGGTTCCTTCAAC
TATTAAAAATAGAAGATGATTGGCAAGAGGATTAATGTTGACTTGACAAAAGGTTGCCCCTCTAATTCTC
TTATCTATAATTATGCCCTTAGGGGGTATGTGAATAGTTATTGGGCTAATAGCAATAATTGGAGCAGTAGG
GGGCTTGAATCAAAATTCAGTGCGAATTATAAGAGCTTATTCATCCTTTGTTCACACGTCTTGGATGCTTT
TAGGGTTGACTTGGTCTACGGTTGTGTTCATAAGATATTTTTTAGTATATAGGATATCAGTAGGGCTATTTT
TCTATGGGTGCTCTCTAATGAACAAGTTAAGAATAATGAGACAATTAAGAGGAGCTGCTAGAGGAATAG
GGCTTATGATACTTATAGGAATACCACCTTTCCTTGGATTCTTGGCTAAAGTGTTGGTGTTTTTAATAAGA
AGTAGGGCTATTATTGTGCTGTGTATTATGGGGTCAGTAATTAGGCTAAAATATTATATTGATTTTTTTTATA
GAATAGTCATAAGAAATAAAAGAGAAGTAAAAGCTATATGAATGTTAGTTACTTCTATAAATTTAATGGG
GGGGGCTCTGATTCTGGTGAGATTTTTGTAAATGGTTATGGGAATAAGGGTTGTCTTTGTATGTATCGTAT
CATTTTTATTTACGGGTTTATTGTTACTAAGAGAAAAGCGAGGCCTTGATCGTGAAAAGTGTAGGCCATA
TGAGTGTGGGTTTGAGCCTATTGGAAGGGCTCGTAGTCCTTTTTCAATTCGGTTTTTCTTAGTAGCTGTTT
TATTTGTAGTGTTTGATGTTGAAGTGGTGCTGTTGATACCCTTTGTCTATATATTTTTCTATGGGAAGAGAT
TATTAGGTATTTTATCCTCAAGAGGGTTTCTATTTATTTTGTTTGTTGGGTTATACCACGAATACCGAGAGG
GATCCTTGGAGTGAGTGGGCTAAATGGTTAAAAGGCTAGCAATCAGGCTTATTGCATTAATTATTATAAA
GAATTTAAACATATCAATTATTGGGCTGAGATTTCTTACAATCATAAGTGTGATGATAGTAAGAACGGTAA
CTGGGAGAGTAGAGTTAAATGGTTTATTCACAACAGATTTTGTGATGGGATTGATGGTAGTATTAACTTTA
TTCGTGGCAGCACTTTCCCACTTAAGAAGGGTGAAAACAGCACGTAAATCAAGATTTAACTTGATAGTT
ATCACAATCAGCCTGATCTTGCTAATAAGTTTTAGAGTGAGAAGGTTTTTTCTCTTTTTTTTTTTTTTTGA
AAGAGTACTGGCGCCCTTGCTGTTATTAATTGTAGGATGGCGGTTACAAGCAGGAGGTTATATGGTGATC
TATACTGTGTTTGGGTCTCTTTTCTTCTTGTGGGGGGTGAGGGCGTTATATATAAGGGGGAGGAGAAGAA
TAAGAGTGAGAAGAATAGTAAAGAAAAGCGCCATGAGATTGTGGTGACTTTATATTCTAGGTTTTTTAAT
CAAATTGCCTATGTACCCTTTTCATTTGTGGCTTCCTAAAGCTCATGTAGAGGCCCCAGTAGCAGGTTCA
ATGTTGTTGGCTGGTGTAGTCTTAAAACTGGGTGGCTATGGTCTTTTACGATTTATAGGGGTTATGCAAAT
AAATCTAAGAAGACTATTTGTGTTGTTATTATTGGTAAATTTGGCTGGAGGATTATATGCCGGGTTGGCTT
GTGTTCGACAGGTAGATCTCAAATGTTTGGTAGCATATTCATCGGTGGCGCATATAAGGCTGGTGTTACTA
GTGTTGAGAAACACTTTAGTGGGAGTAATGGGGGCAATTCTAATTATGGTTGGGCATGGCCTTTGTTCCT
CGGGGTTGTTTAGATATGTTAATGTGGTGTATAAAATAAGGCACTCACGTCTCCTGGTAATAAATAAGGG
AGGGTTATTAATTTGCCCAAGATTGGTATTAATGTGTTTTCTTCTAAGATCTAGAAACATAGCAGCTCCTC

CTAGACTAAATCTATTTGGGGAGATTTTAGTATTTGGGGTAGGGGGCTGAATAAGAGGTGTGTTTCTTTG
TATTTTGGGGTTAATAAGGTTCATTAGGGCCTGTTTTAGGTTATACTTGTATGGAAGGTGTAGGCATGGAA
AAGGGCTAATACATAGAGAATCTTTAAGTTTAATGTGTGATATTATGGTTCTCGGGGTTCATTGAGTACCT
TTAAATTTTCTATTCATGTTTATGCCTTAGCGTATAAGAAAACATCTTCTTTGTCTATTTGTAGGGCTTGAA
ATAATAAGGTTAGGATTGCTGTTTGTGAGACATGTCTTTTTAATAAATCAGTTTTGGTTAATTCTTCTAATT
TTATGTTTAGCTGTTTGTGAGGCTAGGATTTGTTTGGCGCTCTTAGTAATAGTAATGCGACTGTGCGGTAA
TGACTTAATGTCAAGATTAGTAAGAGATtaAAATTAATAAGAGGGAATTTTCCTCTTTTGGTGTTAATTTTT
GGGTATGCATCTATTTCTATTGGCAGTATGAGGAGGTGTTTCTTATTTGAAGTGCCTGTATGAGATAGAAA
TTGTCTATCATTTAGATTTAGAGTTTTAGTTGATAGTGTAAGAATAATTTTTGTTGGTACTGTTTTAGTAAT
CAGAGGCAGAGTAGGAACTTATTGCAAATGGTACATAGCTAGAGAGAGAGATACTATAACCGTTTCATAGG
GTTGGTTTGGTTATTTGTGCTTTCTATAATTTTCATGATTTAGTGCCTAATTTAGTAATGTTACTTATTGGA
TGGGATGGCTTGGGGCTTACTTCATTTTTGTTGGTAGCTTATTATCAAAATAATAAAAGTCTTTCTGCAGC
TATGTTAACAGCTCTAACAAATCGAATTGGGGATGTTCTCGTTTTGGTGAGTATTTCTATCCTCCTAAGTG
AAGGAGGGTGGTTGATTTACAATTATTACCCTATGCAAATATGAAGGTTAGGAATTGTTGTGGTTTTTGC
AGGGATGACCAAGAGGGCCCAGATACCTTTTTGTGCTTGGCTTCCGGCTGCTATAGCTGCTCCAACACC
TGTGTCGTCTCTAGTACATTCTTCTACTTTAGTAACAGCAGGGGTTTACTTGGTGCTTCGGTCATTTTATG
TAGTTAGGGCCAATGCAACCCAAAGGTTGATAGTCTTGAGGTTATTCACTCTGGTATTAGCTGGTTCAAG
CGCAGTGTTTGCCTTTGATTTAAAAAAGGTGATTGCACTTTCTACTCTTAGTCAATTAAGATTAATAATGT
TTTCTATCTCTATTCTTCTCCCACTTGTGGCTTTTTTTCATTTGGTAACCCATGCAGTGTTTAAGGCCCTTC
TTTTTTTAGGGGCTGGCGGCGTGATTCATAGCAACCAAAGAATTCAGGATATTCGGGGTTTAAGAAGATT
GTGGCAAAGGCTGCCTGTGAGGATAGGAGCTATAAGTGTGGCTATTGTATCATTAAGGGGGGCGCCGTT
CATAAGAGGGTTTTACTCTAAAGATTTAATTATTGAAATGATGGACAGAAGGACTTATGGATATATTTTAG
AGCTATTAGGGTTAATTTTTACTTCTTTCTATAGAGCTCGAGTATTTAGGGTAATGTTAGGTTCAAATTATT
TTAATTGTAGAACTCTTCGTGTACCCGAGCACCTAAATATGCAAATCCCATTCTTAAGGCTGTATGTAGGG
GCTATTTTCCTAGGGGTAAGATTAGGGAGAAAAATAGAAAAATTTAGATTTGTAGTGCTTCTTGAGAGAT
ATGAAAGCCTTAGGATTTTTCTGATTCCTTTTGGTCTAATGTGATGGGGAGTACTAACTAAGTTGGGGTTT
AACCCAGCTAAATTAAGATTCTTTTTAAGAATGTGATTTGTGGAGCTTACTCACCCTGGAAAGACCTTGT
TTTTCAAAGGTTCACAAATAGTCTACCAAACTTTGGATCAGGGATGGTTAGAACTGCTAGGCCCACAAG
CTGGGTTGGGGCAAATTAGTCAATTAAATGAAAGCTATTTCATGATTGCCTGAGTAATAGCTGCAGGCAT
AAGATTGATTGCTTTTATATAAATGAGAGTCATAATTATATGTGTAAGTTTGATGTTTATGTTTGTGTTAATT
GCTAAACAACCAATTTCCTTAGGGCTGGTGTTATTAATAGGTTCAATAATTTCATGTGTGGAGATTGCACT
AGAGATCAGAAGATTACTTGGATTTTTGTTGTTTCTGACTTACGTCAGAGGCGTAATAGTTCTATTTTTGT
ATGTGTTAAGAATTTACCCTAATGAGCGATTTAACTTAGAGTTTATTGGGATTGTTATAAGGTGTATTCTAA
CAGGTCTGATTATTACAATAAATTACGAGAATGGGTCTTTGTTTCTTAGGTTTATGGCTGAGATAAGACTC
TATATTCTAATAGCTGGTGTATTATTATTTGTTATACTGGTAGTATCTTACTTGTGTATAAAGACTATAGTAC
CTCTCCGTAGAGTA"/>
<sequence id="seq_Mytilus_coruscusKart1" spec="Sequence" taxon="Mytilus_coruscusKart1"
totalcount="4"
value="atgttaATAGATGTTTTTTCTAGTTTTGATGCTCATAGTTACAATTTGATTTGGCTATCAATGTTGTGGG
TTCTTTCTTCTATGGTACCACTAACTGTATTATTTAGGGACGTGAGTATTCGTAGTTTAGTGTTATCGTTTA
CTTATTCAATGATTCGTAACGGAAAAGGGCTCAAACTTTCCGGGTTTCCATTAGTGATAAGTGGGCTATT
TCTTATAATTTTAATATTAAATTTGTCGGGAAACTTTCCGTTTTTTTTCCCTGTAAGGGGTCAGTTTGTGTT
TGGGTTTTCTTTTGCGCTATCTGTGTGAACTTGTTTAGTATTATCTAGTTTATTGTGTAGGTTTGAACAGA
GATTAATAAGGCTGGTCCCAACTGGGCCACTGATTCTGGTGCCTTTTATAGTAGCGGTTGAATTAATCAG
TGGTATGCTACGCCCTTTGACTTTGGTGTTGCGTTTAACACTGAATCTTGGAGCTGGAAAAGTGATTTTG
ACGATATGTAGGAGAGAGCTAGTAGTAGGGTGATTAATTATAGGAGTAGGGGGAGTTAAAGGGCTTTTA
ATAGGAGGAGTTTTTGCTGCTGAGGTAGCAATTGCCTGTATTCAATGTTATATCTTCTGTGTACTTCTGTG
TCTTTATACTGAGGATCATAGAAGTTAACGATGACTTTGATCAACAAACCATAAGGATATTGGAACTCTTT
ATTTATATAGAGGGGTATGAGGAGGCTTATTTGGGGCAAGACTAAGTCTGATAATTATGCAGGGGCACCC
TGGAGCAGTTTTTTTAAAAGACTGATTTTATAATGTAGTAGTAACAACGCATGCTCTGATAATGATTTTCT
TTGCTGTGATACCCATTTTAATTGGGGCCTTTGGTAACTGGCTTATTCCTTTGCTTGTTGGGGGAAAAGA
CATAATCTATCCACGCATAAACAATCTTAGATATTGACTGTCGCCGAATGCTCTATATTTATTGATGTTATCT
TTTAGGACAGATAAAGGAGTGGGGGCAGGGTGGACGATTTATCCCCCTCTATCTGTGTACCCCTATCATA
GAGGGCCCAGAATGGATGTTCTTATTGTGTCTCTTCATCTGGCAGGACTCAGGTCTTTAGTGGGAGCTAT
TAATTTTGCTAGCACAAATAAGAACATACCAGTATTAGAAATAAAAGGGGAACGGGCTGAGCTTTATGTG
CTAAGAATCAGAGTTACTGCGGTTCTTTTAATTATTTCGATTCCGGTGTTAGGAGGGGGGATCACAATAA
TTTTGTTTGATCGTAATTTTAACACTACTTTCTTTGATCCTGCAGGAGGAGGTGACCCGGTGCTATTTCAG
CATCTATTCTGATTTTTTGGGCACCCTGAAGTATACATTCTTATTTTGCCTGCTTTTGGGGTAATATCTAAG
GTTATTATACATTGCTCTGGAAAAGAGGCGGTGTTTGGGTTAATTGGGATAGTTTATGCTATAATTGGAAT
TGGAGGACTAGGTTGTATGGTGTGAGCACATCATATATTTACGGTAGGGCTTAATGTGGATACTCGGGGG
TACTTCTCAACTGCTACTATAGTAATTGCAGTTCCTACAGGAGTAAAAGTGTTTAGTTGACTAGCAACTAT
GGCAGGAAGAAAGTTCAAAATAAAACCAGCTGCCTTCTGAAGAACAGGTTTTCTGTTTTTATTTACTGT
AGGGGGGTTAACAGGGGTAATATTGTCGAGAGCTTCTATAGATGTGTCACTCCATGATACTTACTATGTG

GTTGCGCATTTTCATTATGTGCTTAGTATGGGGGCTGTGTTTGGAGTTTTCTGTGGTTTGAACCACTGATT
ACCCAATTTTGTTGGAGTTTGTTTTAATAAAAAGTGAAGAAAAGCCCATTTTATAGCTATATTCTTTGGGG
TAAATACCACGTTTTTTCCTCAACACTTTTTAGGGCTAAGAGGTATACCACGACGGTATATAGACTACGCT
GATATTTATGCTCACTGACATTGGGTGTCTTCTTATGGGTCTGCTGTTTCATTTGGGTCTCTTATATATTTTA
AGTTTCTACTTTGGGAGGCTCTAGTAAGCCAGCGAGGAATGTCTTTTTATGGGAGTCGATATTTTGGTGA
TATTGTCCATGAATTAGGAAAAGACCTTTTTCGTTACCATGGCTTTGTAATGATGGTGGCGGTAGCCGTG
TTAGTTTTTGTTATATACATAGGATGCGTGATCCTTCTTACTAAGTTTTCTTATCGCCATTTTTTAAATCGTC
AACGGTTAGAGTTTTGGTGAACAATCGTACCCATATTTTTACTAGTGGGGCTATGGTTTCCGTCAATAATT
AACTTGTACTATATAGAAGAAGTCAAGCGACCACGTTGGAATTTTAAGGCAATTGGAAAGCAGTGATAT
TGGTCCTATGAGTGTGACACTTGTTACACAATCGACTCGTACATAGAGGATCAGCAAGAAACTGGTTATC
GGTTGTTAGATGTGGATAATCGGATGGTTGCTCCTGCGGACGTACAAATAACTGCTTTTGTAAGAAGCTC
AGACGTTCTCCATTCGTTCGCTCTTCCTAAGCTTCTACTAAAAGTGGATGCTATTCCAGGCCGAATTAATC
GCCTGCCGATAAAGGCGTCTCAATGTAGGATTATTTATGGGCAGTGTTCTGAAATCTGCGGGGTAAACCA
TAGGTTTATACCTATCGTAATTGAGTTTATTCCTGAAAAATATTTCGTGATATGATTGGAAGCTCTAAATtaa-

----

ATGAAACGTAATCCTTATTATGTGCCTGGTCCCAGGCCGTGGCCATTCTTTGTGGCTATTTCTGCTAATGG
TATGGCGGTAGGGTTAATCTTATGATTGCATCGGACTCCTTTTCTTCTTATGAGTAGCCTGAGTTGTATGTT
ATTGAGAACCTTTAGTTGATGGCGAGATTTAATTCGCGAAGGAGATATTGGTTTTCATACTCGTTTTGTAA
TTAAGAGGTTTCGAGATGGAGTGGCATTGTTTATCCTGTCGGAGGTAATATTTTTTTTCTCCTTTTTCTGA
ACTTTTTTCCATAACGCCTTGAGCCCTTCTTGTGAACTTGGGATACGATGGCCTCCTCCTGGAATTCGGA
CTCCTAACCCATCATCTACAAGTCTGTTTGAGACAGGTTTGTTAATCAGAAGAGGTCTGTTTGTAACTCA
AGCTCATAAAAGAATGCGGCTAGACTATGATGTCGGTCCCTTTATTGGGTTGGTAGTTACAATCTTATGTG
GTACTCTGTTCTTCTTAGTTCAATTACGGGAATACTATTGGAATTCCTATACTATTGCAGATAGGGTGTACG
GAAGGGTTTTCTATTTGTTAACTGGATTTCATGGGATACATGTGGTTGTGGGGACGCTCTGGCTGATAGT
CAGATTGGTTCGGCTATGACGTGGGGAATTCTCTAGACAACGACATTTTGGGTTCGAGGCTTGTATCTGG
TATTGACATTTTGTGGATGTAGTTTGAGTTGCGTTGTGATGTTTAGTGTATGTATGATTTGGAGGACCGTG
ACGAAAAACTAATAAGCTGGTAAAAATCATAAATGATAGATTCTATGATTTACCGTGTCCTGTGAACTTA
AATGCTTGATGGAGATTTGGCTCCATATTAGGTTTATGTCTTGTAATCCAACTTTTAAGTGGTCTTTTGTTG
TCGATTCATTATACGGCCCACGAAGATATAGCATTTGACTCTGTTGTGCATATTATGCGCAATGTTAAAAA
AGGTTGAATGTTGCGAAATATCCATGCTAATGGGTCTTCTATATTTTTTATCTGTATTTATGCCCACATTGG
TCGTGGTTTGTACTACGGCTCATATTTGGACAAAACAGTTTGGTATTTTGGGGTTCATCTTTTGTTATTAA
CTATGGCTGAGGCCTTTTTAGGTTACACACTACCTTGAGGTCAGATATCATATTGAGGTGCCACTGTGATT
ACTAATATACTTAGGGTGATTCCTGTAGTAGGAGAAAGAATACTTCGTTATGTGTGAGGTGGTTGGACAG
TCTGTAATGCAACGCTAAAGCGGTTTTATACCCTTCATTTTCTTCTTCCATTTGTTATAGTGGCGGTTGTAT
TTCTTCACCTTTTTTTTCCTGCATGAAAAAGGGAGGAATAACCCTTTGGGGATTGAAAGTGATACTGTGTG
TGTTCCTTTTCATCCTTTTTACACTGTGAAAGATTTATTTGGCTATATTTGTTTTAGGTTTTTTTTTATGTAC
TTAGTATGTGTAGACCCTGAGTTATTAGGAAATCACTTAAATTATTGGCCTGCTAACCCGATAAAAACCCC
TATTCATGTCCAGCCAGAGTGATATTTTATATTTGCTTACGCTATTCTCCGTTCAATTCCTCATAAAGCAGG
GGGTGTATATGTTATATTTTTGTCTATTGTGGTCCTGTATCTGATTCCCAGGTTGCACAGAGGTAAATATCG
AAGATTGTGTTTTTACCCCTTTAACCAAGTCGTGTTTTGAGTTCTAGTGGGGAGCTTTATTAGCTTGACG
TGAATTGGGGCTCGTCCAGTTCGTGAGCCTTATATTGTGATAGGCCAATGTTTTTCGATTATTTATTTTTCG
AGGCTTCTATTGAACCCCCTTTCATTATGGATGTGGGATAAATTGCTAGATTTGGGAGTGGTAATTGGGAT
CGTGCCTTTTGTTGGGGTACTTCTTGCTGTAGGGTTTTATACCTTATTGGAGCGCAAAATTTTAGCTATTAT
TATAATCCGCAAAGGACCATCTAAGGTTAGTTATATGGGGATCTTGCAGCCATTTAGTGACGCAGGCAAG
TTGTTATGCAAAGAATTTATTATGCCCACACGGGCTAACATGGGGCCTTTTATTTTGGCCCCAGCGTTAAT
ATTAACCGTAAGGCTTCTGGGATGGCTTTTATACCCTTACAAATCGGCGGAGGTATTTTATGTTTTTGGCG
TGATTCTTTTTATGGTAATTACTAGGGTAAGAGTATATGGGGTGATAATATCTGGGTGAGCTTCAAACTCA
AAATATTCACTGTTGGGTGCGGTCCGTGCAATGGCGCAAAGAATCTCTTACGAGATTCCAATAGGATTTA
TCTTTTTCTGTGTAGTGTTAGTCTCTGGTGTGTTCATATTCCAAGAGATTAGGGCGTCATTTTTTTTCTCCC
CATTATCTGTTATTCTTATCGTGTGAATTTTATGTATATTAGCAGAAACTAACCGTGCTCCTTTTGATTTCGT
GGAAGGGGAGTCTGAGTTAGTTTCTGGGTATAATGTAGAGTACAGAGGAGGAGGTTTTGCAGTGATGTT
TATTGCTGAATATTCTAGAATTCTTCTTAGCAGAGTAATAAGGGCTGCAATATTCTTTGGTGGGAACGAGG
CGCTAATTGGTTTTTTTATGATAGTTTTTGCGGTTTTTTTTGTAATTGTGCGTGCTTCTTTACCGCGTTTGC
GTTATGACAAACTGATAAGCTTGTGCTGAACTGTGCTCCTATGTGTCATGCTTATGGTTAGTGTATGTGTG
GTGACCATAATCAGAGTATAATGGTAAGTTTTGTAATAAGGCCTATAAAATTAATAAGAATAAGGATAATG
GTTATTGGGACAGTGCTTAGGGTTAGAAGAGAAGAATTAGTGGGTGTATGGTTGGGGATAGAATTAAATT
TGTATGGATTCCTTGTGGTTATGAACCCAGATGGGCACTATATTCCAGAGCCTTGTGTTAAATACTTTGTA
GTGCAAAGAACTGGGTCTATTTTAATATTAGGAGGTTTTGTGCTATTGATGCAATTTGTAGTCAGGGGTTT
AATAATGAGAGTTGCTGGAACTGTACTAAAATCTGGTATTTCTCCGCTGCATTCTTGGGTGCCTTCAACT
ATTAAAAATAGAAGGTGGTTAGCGAGAGGGTTGATGTTAACTTGACAAAAGGTAGCCCCTCTGGTTTTA
TTGTCAATAATTATGCCCTTGAGAGGTATGTGGTTAGTTATCGGTCTAATAGCGATAATTGGGGCATTGGG
GGGTTTAAACCAAAATTCGGTGCGAGTTATAAGTGCTTATTCATCCTTTGTTCATACCTCTTGGATGCTCT
TAGGTCTTACCTGATCAAGAGTTGTGTTCGTAAGATATTTTTTAGTATATAGAATGTCAGTAGGGTTGTTT

TTTTATGGATGTTCCCTAATAAATAAGCTAAGAATAAGGAGACAACTAAGAGGGGCTGCAAGAGGAATA
GGGCTTTTAATGCTTATGGGAATGCCCCCCTTCCTTGGGTTTTTAGCTAAGGTGCTAGTGTTTCTGATAAC
AAGAAGGTCAGTAATTGTGTTGTGCATTGTAGGCTCAGTTATTAGGCTAAAATACTATATTGATTTCTTTT
ATAGAATAGTTATAAAAAGTAAGAGAGGAGTGAAAAGTATGTGAATGTTAGTTATTATTATAAATTTAATA
GGGGGAGTAGTAATTTTAGTAAGATTTCTATAAATGGTTATGGTAATGAGTGTTGTTTTTGTGTGTATTGT
ATCACTTCTATTTACGGGATTGTTGTTACTGAGTGAAAAGCGCGGTCTTGATCGTGAAAAATGTAGGCCA
TACGAGTGCGGGTTTGAGCCTATTGGAAGGGCCCGCAGCCCTTTCTCGATTCGGTTTTTCTCGTAGCTG
TATTATTTGTAGTGTTTGACGTTGAGGTAGTTTTATTAATACCTTTTGCTTATATGTTTCTTTATGGTAAGAG
GTTAATGGGAATCTTATCTTCAAGGGGGTTTTTGTTTATTTTGTTTGTGGGGTTGTACCATGAGTATCGAG
AAGGTTCTTTGGAGTGAgtgggataaATGGTTAAAAGGCTAGCAATCAGGCTTATTGCATTAATTATTATAAAA
GACCTAAATATATCGATTATTGGACTGAGACTTTTAACAATTACAAGCTTGATGACAACGAGAACAGCGA
TTAGAAGGGTGGAGATGGGCGGCTTATACACGACAGATTTTGTGATGGGGTTGATGGTAACATTAACCTT
GTTTGTCGCAGCACTTTCTCACCTAAGAAGGGTAAAAACAGCGCGCAAATCAAGGTTTAATTTAATAGT
CGTGAGAATCACTCTGATTTTACTGATAAGATTTAGGGTAAGAAGTTTCTTTCTTTTTTTTTTTTTTTTCG
AGAGAGTGTTAGCACCTCTGCTATTGCTAATTGTGGGATGACGATTACAAGCAGGGGGTTATATGGTTAT
TTATACTGTGTTTGGTTCGCTCTTCTTTCTATGAGGCGTAAGGGCATTATATGTAAGAGGAAGGAGAAGA
ATAAGGGTAGGAAATATAACAAAGAAAAGTGCTATAAGACTGTGGTGACTGTATATTCTGGGCTTCTTAA
TTAAATTGCCCATATACCCTTTCCATTTATGACTTCCTAAGGCTCATGTAGAAGCTCCGGTAGCAGGTTCG
ATGCTGTTAGCTGGTGTAGTTCTAAAACTAGGTGGCTATGGATTACTCCGGTTCATGAGAGTTATGCAGA
TAAATTTGAGAAGAGTATTTATTTTGCTGTTGCTTGTAAATTTGGCTGGAGGACTTTATGCCGGGTTGGTA
TGTGTACGACAGGTGGACCTAAAATGTTTAGTAGCGTATTCATCTGTGGCTCATATGAGTTTGGTGTTGTT
AGTGTTGAGAAACACTCCAGTAGGGGTAATAGGGGCTGTCATCATTATGATCGGTCATGGTCTTTGTTCT
TCTGGTTTATTCAGGTACGTTAACGTGGCGTATAAAATAAGCCACTCACGTCTTCTGGTAATAAATAAAG
GAGGGTTATTAATTTGTCCGAGACTAGTTTTAATGTGTTTTCTTCTAAGTTCTAGAAATATGGCGGCTCCC
CCTAGATTAAATTTATTTGGGGAAATCCTAGTATTTGGGGTTGGAGGTTGAATAAGGGGCCTATTTCTCTG
TATCCTGGGGTTAATAAGGTTTATTAGAGCCTGTTTTAGCTTATACCTGTATGGAAGGTGTAATCATGGAA
AAGGAATAATACATAGAGAGTCCTTAAGCTTTATGTGTGATATTATAATTTTAGCAGTTCATTGGGTGCCT
TTAAATTTCTTGTTTATATTTATGCCTTAGCGTATGAGCAAACACCTTCTTTGCTTGTTTGTGGGGCTTGA
AATAATAAGTTTAGGGTTGCTATTTGTAAGACATGTTTTTCTGATAAACCAGTTTTGGCTGATTCTTTTAAT
TCTGTGCTTAGCTGTTTGTGAGGCTAGAATTTGTTTAGCACTTTTAGTGATAGTAATACGGTTATGCGGCA
ATGATTTAATGTCAAGATTAATAAGAGATGGAAACTAATAAGGAGAAATTTACCTCTTTTGGTATTAATTT
GTGGGTATTTGTCAATTTTCACTGGCAGTATCAGAAGAGCTTATTTGCTTGAAGTGCCTGTGTGGGATAG
ACACTGCTTGTCATTTAGGTTTAGAATTCTGGCGGATAACGTAAGAATAATCTTTGTTGGAACTGTTTTAG
TAATTAGAGGCAGGGTAGCAACTTACTGTAAGTGGTACATAGCTAGGGAAACATATTATAACCGTTTTAT
GGGACTGGTATGGCTCTTTGTGCTTTCCATAGTTTTTATGATTTTAGTGCCTAACTTAGTAATGTTGCTTAT
TGGATGGGATGGCTTAGGGCTTACGTCATTTTTGTTAGTAGCCTACTATCAAAACAACAAAAGTCTATCG
GCAGCCATGTTGACGGCTTTAACAAATCGGATCGGAGATGTTCTCGTTCTGGTCAGCATTTCTATCCTTT
TAAATGAGGGTGGATGATTAGTTTACAGCTATTACCCTGTGAGAATGTGGAGGTTAAGAGTTGTCGTGGT
TCTTGCAGGAATAACTAAAAGAGCGCAGATGCCTTTTTGTGCTTGGCTTCCAGCTGCTATGGCTGCTCCA
ACACCTGTGTCATCTTTGGTGCATTCTTCTACTTTAGTAACAGCAGGGGTTTATTTAGTACTTCGCTCATT
CTATGTGGTTAGGGCTAACGCAACTCAGATATTGATAGTCTTAAGACTGTTTACCTTAGTGTTAGCTGGTT
CAAGCGCAGTGTTTGCGTTTGATTTAAAGAAAGTGATTGCATTATCCACTTTAAGCCAATTGAGCCTAAT
AATGTTTTCTATTTCTATTCTTCTTCCGTTTGTGGCTTTTTTCCATTTGGTAACTCACGCGGTGTTTAAAGC
CTTGCTCTTCTTGGGGGCGGGGGGTGTTATCCATAGAAACCAGAGGATTCAAGATATTCGAGGTTTAAG
AAGTTTATGGCAAAGACTGCCTGTGAGAATAAGAGCAATAAGTGTGGCTATTGTGTCATTAAGTGGGGC
CCCGTTTATAAGAGGATTTTATTCTAAAGACTTAATTATTGAGATAATAGACAGAAGAACTTATGGGTATA
TATTAGAATTATTAGGCCTAATTTTTACCTCCTTTTACAGAGCTCGGGTGTTTAGGGTAATATTAGGGTTGA
ACTATATTAATTGTAGAACTCTTCGTCTATTTGAGCACCTAAACATGCAGGTTCCTTTTTTGAGACTATATG
TAGGTGCTATTTTTTTAGGGTTAAGACTTGGTAGGACAATAGAGAAGTTTGGGTATGTAGTGGTACTTGA
AAGGTATGAGAGGCTTAGAATCTTTTTAATTCCTTTTGGCTTAATATGATGGGGAGTACTAACTAAACTAG
GGTTTAACCCGACTAAATTGAGGTTTTTCTTGAGGATGTGGTTTGTGGAGCTTACTCATCCTGGAAAGAC
TTTATTTTTTAAAAGTTCTCTAATAATGTATCAGACATTAGATCAAGGATGGCTAGAATTATTAGGTCCGC
AGGCTAAGTTAGGACAAGTTAGCCAGTTAAACGAGAATTATTTTACGGTTGCTTGAGTAACAATTGCAG
GTTTGAGGTTAGTTGTTTTTATGTAAATGAGGGTTATAGTAATGTGTGTGGGTTTGATGTTTGTGTTCGTG
TTAATTGCCAAACAACCAATTTCCTTAGGGTTGGTGTTGTTGATAGGTTCTATAATCTCATGCGTAGAAAT
TGCGTTGGAAATCAGAAGGTTATTAGGGTTTTTGTTGTTTCTGACTTATGTTAGAGGTGTGATAGTACTAT
TCTTATATGTGCTAAGAATTTATCCTAATGAACGGTTTAATTTGGAGTTTATTGTAATTGTAATAAGATGTA
TTGTGACAAGGCTGGTTATTACAATGAATTATGAGAATGGGTCATTATTTCTTAGGTTTATAGCTGAAGGG
AGACTCTATATTCTAATAGGGGGCGTATTGCTGTTTGTAATGTTAGTGGTGTCTTATTTGTGTATGAAAACT
ATGGTACCACTTCGTAGGGTA"/>
<sequence id="seq_Mytilus_coruscusKJ577549" spec="Sequence"
taxon="Mytilus_coruscusKJ577549" totalcount="4"
value="ATGTTAATAGATGTTTTTTCTAGTTTTGATGCTCATAGTTACAATTTGATTTGGCTATCAATGTTGTG

GGTTCTTTCTTCTATGGTACCACTAACTGTATTATTTAGGGACGTGAGTATTCGTAGTTTAGTGTTATCGTT
TACTTATTCAATGATTCGTAACGGAAAAGGGCTCAAACTTTCCGGGTTTCCATTAGTGATAAGTGGGCTA
TTTCTTATAATTTTAATATTAAATTTGTCGGGAAACTTTCCGTTTTTTTTCCCTGTAAGGGGTCAGTTTGTG
TTTGGGTTTTCTTTTGCGCTATCTGTGTGAACTTGTTTAGTATTATCTAGTTTATTGTGTAGGTTTGAACAG
AGATTAATAAGGCTGGTCCCAACTGGGCCACTGATTCTGGTGCCTTTTATAGTAGTGGTTGAATTAATCA
GTGGTATGCTACGCCCTTTGACTTTGGTGTTGCGTTTAACACTGAATCTTGGAGCTGGAAAAGTGATTTT
GACGATATGTAGGAGAGAGCTAGTAGTAGGGTGATTAATTATAGGAGTAGGGGGAGTTAAAGGGCTTTT
AATAGGAGGAGTTTTTGCTGCTGAGGTAGCAATTGCCTGTATTCAATGTTATATCTTCTGTGTACTTCTGT
GTCTTTATACTGAGGATCATAGAAGTTAACGATGACTTTGATCAACAAACCATAAGGATATTGGAACTCT
TTATTTATATAGAGGGGTGTGAGGAGGTTTATTGGGGCAAGACTAAGTCTGATAATTATGCAGGGGCAC
CCTGGAGCAGTTTTTTAAAAGACTGATTTTATAATGTAGTAGTAACAACGCATGCTCTGATAATAATTTT
CTTTGCTGTGATACCCATTTTAATTGGGGCCTTCGGTAACTGGCTTATTCCTTTGCTTGTTGGGGGAAAA
GACATAATCTATCCACGTATAAACAATCTTAGATATTGACTGTCGCCGAATGCTCTATATTTATTGATGTTAT
CTTTTAGGACAGATAAAGGAGTGGGGGCAGGGTGGACGATTTATCCCCCTCTATCTGTGTACCCCTATCA
TAGAGGGCCCAGAATGGATGTTCTTATTGTGTCTCTTCATCTGGCAGGACTCAGGTCTTTAGTGGGAGCT
ATTAATTTGCTAGCACAAATAAGAACATACCAGTATTAGAAATAAAAGGGGAACGGGCTGAGCTTTATG
TGCTAAGAATCAGAGTTACTGCGGTTCTTTTAATTATTTCGATTCCGGTGTTAGGAGGGGGGATCACAAT
AATTTTGTTTGATCGTAATTTTAACACTACTTTCTTTGATCCTGCAGGAGGAGGTGACCCGGTGCTATTTC
AGCATCTATTCTGATTTTTTGGGCACCCTGAAGTATACATTCTTATTTTGCCTGCTTTTGGGGTAATATCAA
AGGTTATTATACATTGCTCTGGAAAAGAGGCGGTGTTTGGGTTAATTGGGATAGTTTATGCTATAATTGGG
ATTGGAGGACTAGGTTGTATGGTGTGAGCACATCATATATTTACGGTAGGGCTTAATGTGGATACTCGGG
GGTACTTCTCAACTGCTACTATAGTAATTGCGGTTCCTACAGGAGTAAAAGTGTTTAGTTGACTAGCAAC
TATGGCAGGAAGAAAGTTCAAAATAAAACCAGCTGCCTTCTGAAGAACAGGTTTTCTGTTTTTATTTACT
GTAGGGGGGTTAACAGGGGTAATATTGTCGAGAGCTTCTATAGATGTGTCACTCCATGATACTTACTATGT
GGTTGCGCATTTTCATTATGTGCTTAGTATGGGGGCTGTGTTTGGGGTTTTCTGTGGTTTGAACCACTGAT
TACCTAATTTTGTTGGAGTTTGTTTTAATAAAAAGTGAAGAAAAGCTCATTTTATAGCTATATTCTTTGGG
GTAAATACCACGTTTTTTCCTCAACACTTTTTAGGGCTAAGGGGTATACCACGACGGTATATGGACTACG
CTGATATTTATGCTCACTGACATTGGGTGTCTTCTTATGGGTCTGCTGTTTCATTTGGGTCTCTTATATATTT
TAAGTTTCTACTTTGGGAGGCTTTAGTAAGCCAGCGAGGAATGTCTTTTTATGGGAGTCGATATTTTGGT
GATATTGTCCATGAATTAGGAAAAGACCTTTTTCGTTACCATGGCTTTGTAATGATGGTGGCGGTAGCCG
TGTTAGTTTTTGTTATATACATAGGATGCGTGATTCTTCTTACTAAGTTTCTTATCGTCATTTTTTAAATCG
TCAACGGTTAGAGTTTTGGTGAACAATCGTACCCATATTTTTACTAGTGGGGCTATGGTTTCCGTCAATAA
TTAACTTGTACTATATAGAAGAAGTCAAGCGACCACGTTGGAATTTTAAGGCAATTGGAAAGCAGTGAT
ATTGGTCCTATGAGTGTGACACTTGTTACACAATCGACTCGTACATAGAGGATCAGCAAGAAACTGGTTA
TCGATTGTTAGATGTGGATAATCGGATGGTTGCTCCTGCGGATGTACAAATAACTGCTTTTGTAAGGAGC
TCAGACGTTCTACATTCGTTCGCTCTTCCTAAGCTTCTACTAAAAGTGGACGCTATTCCGGGTCGAATTA
ATCGCCTGCCGATAAAGGCGTCTCAATGTAGGATTATTTATGGGCAGTGTTCTGAAATCTGCGGGGTAAA
CCATAGGTTTATACCTATCGTAATTGAGTTTATTCCTGAAAAATATTTCGTGATATGATTGGAAGCTCTAAA
TTAA------------------------
ATGAAACGTAATCCTTATTATGTGCCTGGTCCCAGGCCGTGGCCATTCTTTGTGGCTATTTCTGCTAATGG
TATGGCGGTAGGGTTAATCTTATGATTGCATCGGACTCCTTTTCTTCTTATGAGTAGCCTGAGTTGTATGTT
ATTGAGAACCTTTAGCTGATGGCGAGATTTAATTCGCGAAGGAGATATTGGTTTTCACACTCGTTTTGTA
ATTAAGAGGTTTCGAGATGGAGTGGCATTGTTTATCTTGTCGGAGGTAATATTTTTTTTCTCCTTTTTCTG
AACTTTTTTCCATAACGCCTTGAGCCCTTCTTGTGAACTTGGGATACGATGGCCCCCTCCTGGAATTCGG
ACTCCTAACCCATCATCTACAAGTCTGTTTGAGACAGGTTTGTTAATCAGAAGAGGTCTGTTTGTAACTC
AAGCTCATAAAAGAATGCGGCTAGACTATGATGTCGGCCCCTTTATTGGGTTGGTAGTTACAATCTTATGC
GGTACTCTGTTCTTCTTAGTTCAACTACGGGAATACTATTGAAATTCTTATACTATTGCAGATAGGGTGTA
CGGAAGGGTTTTCTATTTGCTAACTGGATTTCATGGGATACATGTGGTTGTGGGGACGCTCTGGCTGATA
GTCAGATTGGTTCGGCTATGGCGTGGGGAATTCTCTAGACAACGACATTTTGGGTTCGAGGCTTGTATCT
GGTATTGACATTTTGTGGATGTAGTTTGAGTTGCGTTGTGATGTTTAGTGTATGTATGATTTGGAGGACCG
TGACGAAAAACTAATAAGCTGGTAAAAATCATAAATGACAGATTCTATGATTTACCGTGTCCTGTGAACT
TAAATGCTTGATGAAGATTTGGCTCCATATTAGGTTTATGTCTTGTAATCCAACTTTTAAGTGGTCTTTTGT
TGTCGATTCATTATACGGCCCACGAAGATATAGCATTTGACTCTGTTGTGCATATTATGCGCAATGTTAAA
AAAGGCTGAATGTTGCGGAATATCCATGCTAATGGGTCTTCTATATTTTTTATCTGTATTTATGCCCACATT
GGTCGTGGTTTGTACTACGGCTCATATTTGGACAAAACAGTTTGGTATTTTGGGGTTCATCTTTTGTTATT
AACTATGGCTGAGGCCTTTTTAGGTTACACACTACCTTGAGGCCAGATATCATATTGAGGTGCCACTGTG
ATTACTAATATACTTAGGGTGATTCCTGTAGTAGGAGAAAGAATACTTCGTTATGTGTGAGGTGGTTGGA
CAGTCTGTAATGCAACGCTAAAGCGGTTTTATACCCTTCATTTTCTTCTTCCATTTGTAATAGTGGCGGTT
GTATTTCTTCACCTTTTTTTCCTGCATGAAAAAGGGAGGAATAACCCTTTGGGGATTGAAAGTGATACTG
TGTGTGTTCCTTTTCATCCTTTTTACACTGTGAAAGATTTATTTGGCTATATTTGTTTTAGGTTTTTTTTTAT
GTACTTAGTATGTGTAGACCCTGAGTTATTAGGAAATCACTTAAATTATTGGCCTGCTAACCCGATAAAAA
CCCCTATCCATGTTCAGCCAGAGTGATATTTTATATTTGCTTACGCTATTCTCCGTTCAATTCCTCATAAAG
CAGGGGGTGTATATGTTATATTTTTATCTATTGTGGTCCTGTATCTGATTCCCAGGTTGCACAGAGGTAAAT

ATCGAAGATTGTGTTTTTACCCCTTTAACCAAGTCGTGTTTTGAGTTCTAGTGGGGAGCTTTATCAGCTT
GACGTGAATTGGGGCTCGTCCAGTTCGTGAGCCTTATATTGTGATGGGCCAATGTTTTTCGATTATTTATT
TTTCGAGGCTTCTATTGAACCCCCTTTCATTATGGATGTGGGATAAATTGCTAGATTTGGGAGTGGTAATT
GGGATCGTGCCTTTTGTTGGGGTACTTCTTGCTGTAGGGTTTTATACCTTATTGGAGCGCAAAATTTTAGC
TATTATTATAATCCGTAAAGGACCATCTAAGGTTAGTTATATGGGGATCTTGCAGCCATTTAGTGACGCAG
GCAAGTTGTTATGCAAAGAATTTATTATGCCCACACGGGCTAATATGGGGCCTTTTATTTTGGCCCCAGCG
TTAATATTAACCGTAAGGCTTCTGGGATGGCTTTTATACCCTTACAAATCGGCGGAGGTATTTTATGTTTTT
GGCGTGATTCTTTTTATGGTAATTACTAGGGTAAGAGTATATGGGGTGATAATATCTGGGTGAGCTTCAAA
CTCAAAATATTCACTCTTAGGTGCGGTCCGTGCAATGGCGCAAAGAATCTCTTACGAGATTCCAATAGGA
TTTATCTTTTTCTGTGTAGTGTTAGTCTCTGGTGTGTTCATATTCCAAGAGATTAGGACGTCATTTTTTTTC
TTCCCATTATCTGTTATTCTTATTGTGTGAATTTTATGTATATTAGCAGAAACTAACCGTGCTCCTTTTGATT
TTGTGGAAGGGGAGTCTGAGTTAGTTTCTGGGTATAATGTGGAGTACAGAGGAGGAGGTTTTGCAGTGA
TGTTTATTGCTGAATATTCTAGAATTCTCCTTAGCAGAGTAATAAGGGCTGCAATATTCTTTGGTGGGAAC
GAGGCGCTAATTGGTTTTTTTATGATAGTTTTTGCGGTTTTTTTTGTAATTGTGCGTGCTTCTTTACCGCGT
TTACGTTATGACAAACTGATAAGCTTGTGCTGAACTGTGCTCCTATGTGTCATGCTTATGGTTAGTGTATG
TGTGGTGACCATAATCAGAGTATAATGGTAAGTTTTGTAATAAGGCCTATAAAAGTAATAAGAATAAGGA
TAATGGTTATTGGGACAGTGCTTAGGGTTAGAAGAGAAGAATTAGTGGGTGTATGGTTGGGGATAGAATT
AAATTTGTATGGATTCCTTGTGGTTATGAACCCAGATGGGCACTATATTCCAGAGCCTTGTGTTAAATACT
TTGTAGTGCAAAGAACTGGATCTATTTTAATATTAGGAGGTTTTGTGCTATTGATGCAATTTGTAGTCAGG
GGTTTAATAATGAGAGTTATTGGAACTGTACTAAAATCTGGTATTTCTCCGCTGCATTCTTGGGTGCCTTC
AACTATCAAAAATAGAAGGTGGTTAGCGAGAGGGTTGATGTTAACTTGGCAAAAGGTAGCCCCTCTGGT
TTTATTGTCAATAATTATGCCCCTGAGAGGTATGTGGTTAGTTATCGGTCTAATAGCGATAATTGGGGCATT
GGGGGGTTTAAACCAAAATTCGGTGCGAGTTATAAGCGCTTATTCATCTTTTGTTCATACCTCTTGGATGC
TCTTAGGTCTTACCTGATCAAGAGTTGTGTTCGTAAGATATTTTTTAGTATATAGAATGTCAGTAGGGYTG
TTTTTTTATGGATGTTCCCTAATAAATAAGCTAAGAATAAGGAGACAACTAAGAGGGGCTGCAAGAGGA
ATAGGGCTTTTAATGCTTATGGGAATGCCCCCCTTCCTTGGGTTTTTAGCTAAGGTGCTAGTGTTTCTGAT
AACAAGAAGGTCAGTAATTGTGTTGTGCATTGTAGGCTCAGTTATTAGGCTAAAATATTATATTGATTTCT
TTTATAGAATAGTTATAAAAAGTAAGAGAGGGGTGAAAAGCATGTGAATGTTAGTTATTATTATAAATTTA
ATAGGGGGAGTAGTAATTTTAGTAAGATTTCTATAAATGGTTATGGTAATGAGTGTTGTTTTTGTGTGTATT
GTATCACTTCTATTTACGGGATTGTTGTTATTGAGCGAAAAGCGCGGTCTTGATCGTGAAAAGTGTAGGC
CATACGAGTGTGGGTTTGAGCCTATTGGAAGGGCCCGCAGCCCTTTCTCGATTCGGTTCTTTCTCGTAGC
TGTATTATTTGTAGTGTTTGACGTTGAGGTAGTTTTATTAATACCTTTTGCTTATATGTTTCTTTATGGTAAG
AGGTTAATAGGAATCTTATCTTCAAGGGGGTTTTTGTTTATTTTGTTTGTGGGGTTATACCATGAGTATCG
AGAAGGTTCTTTGGAGTGAGTGGGATAAATGGTTAAAAGGCTAGCAATCAGGCTTATTGCATTAATTATT
ATAAAAGACCTAAATATATCGATTATTGGACTGAGACTTTTAACAATTACAAGCTTGATGACAACGAGAA
CAGCGATTAGAAGGGTGGAGATGGGCGGCTTATACACGACAGATTTTGTGATGGGGTTGATGGTAACAT
TAACCTTGTTTGTCGCAGCACTTTCTCACCTAAGAAGGGTAAAAACAGCGCGCAAATCAAGGTTTAATT
TAATAGTCGTGAGAATCACTCTGATTTTACTGATAAGATTTAGGGTAAGAAGTTTCTTTCTTTTTTTTTTTT
TTCGAGAGAGTgtgATTAGCACCTCTGCTATTGCTAATTGTGGGATGGCGATTACAAGCAGGGGGtatatggttat
ttatactTTATATGGTTATTTATACTGTGTTTGGGCTCTTCTTTCTATGAGGCGTAAGGGCAAGGAGAAGAATA
AGTGTAGGAAATATAGTAAAGAAAAGCGCTATAAGACTGTGGTGACTGTATATTCTGGGCTTCTTAATAA
ACTGCCCATATACCCTTTCCATTTATGACTTCCTAAGGCTCATGTAGAAGCTCCGGTAGCAGGTTCGATAC
TGTTAGCTGGTGTAGTTCTAAAACTAGGTGGCTATGGATTATTACGGTTCATGAGAGTTATGCAGATAAAT
TTGAGAAGAGTATTCATCTTGCTGTTGCTTGTAAATCTGGCTGGAGGACTTTATGCCGGGTTGGTATGTG
TACGACAGGTGGACTTAAAATGTTTAGTAGCGTATTCATCTGTGGCTCATATGAGTTTGGTGTTGTTAGTG
TTGAGAAACACTCCAGTAGGGGTAATAGGGGCTGTTATCATTATGATTGGTCATGGCCTTTGTTCTTCTG
GTTTATTCAGATATGTTAACGTGGCCTATAAAATAAGCCACTCACGTCTTCTGGTAATAAACAAAGGAGG
GTTATTAATTTGTCCGAGACTAGTTTTAATGTGCTTTCTTCTAAGTTCTAGAAATATGGCGGCTCCCCCTA
GATTAAATTTATTTGGGGAAATCCTAGTATTTGGGGTTGGAGGTTGAATAAGGGGCCTATTTCTCTGTATC
CTGGGGTTAATAAGGTTTATTAGAGCCTGTTTTAGCTTATACCTGTATGGAAGGTGTAATCATGGAAAAG
GAATAATACATAGAGAGTCCTTAAGCTTCATGTGTGATATTATAATTTTAGCAGTTCATTGGGTGCCTTTA
AATTTCTTGTTTATATTTATGCCTTAGCGTATGAGCAAACACCTTCTTTGCTTGTTTGTGGGGCTTGAAAT
AATAAGTCTAGGGTTGCTGTTTGTAAGACATGTTTTTCTGATAAACCAGTTTTGGCTGATTCTTTTAATTC
TGTGCTTAGCTGTTTGTGAGGCTAGAATTTGTTTAGCACTTTTAGTGATAGTAATACGGTTATGCGGCAAT
GATTTAATGTCAAGATTAATAAGAGATGGAAACTAATAAGGAGAAATTTACCTCTTTTGGTATTAATTTGT
GGGTATTTGTCAATTTTCACTGGCAGTATCAGAAGAGCTTATTTGCTTGAAGTGCCTGTGTGGGATAGAC
ACTGCTTGTCATTTAGGTTTAGAATTCTGGCGGATAACGTAAGAATAATCTTTGTTGGGACTGTTTTAGTA
ATTAGAGGCAGGGTAGCAACTTACTGTAAGTGGTACATAGCTAGGGAAACATATTATAACCGTTTTATGG
GACTGGTATGGCTCTTTGTGCTTTCCATAATTTTTATGATTTTAGTGCCTAATTTAGTAATGTTGCTTATTG
GATGGGATGGCTTAGGGCTTACGTCATTTTTGTTAGTAGCCTACTATCAAAACAACAAAAGTCTATCGGC
AGCCATGTTGACGGCTTTAACAAATCGGATCGGAGATGTTCTCGTTCTGGTCAGCATTTCTATCCTTTTAA
ATGAGGGTGGATGATTAGTTTACAGCTATTATCCTGTGAGAATGTGGAGGTTAAGAGTCGTCGTGGTTCT
TGCAGGAATAACTAAAAGAGCGCAGATGCCTTTTTGTGCTTGGCTTCCAGCTGCTATGGCTGCTCCAAC

ACCTGTGTCGTCTTTGGTGCATTCTTCTACTTTAGTAACAGCAGGGGTTTATTTAGTGCTTCGCTCATTCT
ATGTGGTTAGGGCTAACGCAACTCAGATATTGATAATCTTAAGACTGTTTACCTTAGTGTTAGCTGGTTCA
AGCGCAGTGTTTGCGTTTGATTTAAAGAAAGTGATTGCATTATCCACTTTAAGCCAATTGAGCCTAATAA
TGTTTTCTATTTCTATTCTTCTTCCGTTTGTGGCTTTTTTCCATTTGGTAACTCACGCGGTGTTTAAAGCCT
TGCTCTTCTTGGGGGCGGGGGGTGTTATCCATAGAAACCAGAGGATTCAAGATATCCGAGGTTTAAGAA
GTTTATGGCAAAGACTGCCTGTGAGAATAAGAGCAATAAGTGTGGCTATTGTGTCCTTAAGTGGGGCCC
CGTTTATAAGAGGATTTTATTCTAAAGACTTAATTATTGAGATAATAGACAGAAGAACTTATGGGTATATAT
TAGAATTATTAGGCCTAATTTTTACCTCCTTTTACAGAGCTCGGGTGTTTAGGGTAATATTAGGGTCAAAC
TATATTAATTGTAGGACTCTTCGGCTATTTGAGCACCTAAACATGCAGGTTCCTTTTTTGAGACTATATGTA
GGTGCTATTTTTTTAGGGTTAAGACTTGGTAGGACAATAGAGAAGTTTGGGTATGTAGTGGTACTTGAAA
GGTATGAGAGGCTTAGAATCTTTTTAATTCCTTTTGGCTTAATATGATGGGGAGTACTAACTAAACTAGGG
TTTAACCCGACTAAATTGAGGTTTTTCTTGAGGATGTGGCTTGTGGAGCTTACTCATCCTGGAAAGACTT
TATTTTTTAAAAGCTCTCTAATAATGTATCAGACATTAGATCAAGGATGGCTAGAATTATTAGGTCCGCAG
GCTAAGTTAGGACAAGTTAGCCAGTTAAACGAGAATTATTTTACGGTTGCTTGAGTAACAATTGCAGGTT
TGAGGTTAGTTGTTTTTATGTAAATGAGGGTTATAGTAATGTGTGTGGGTTTGATGTTTGTGTTCGTGTTA
ATTGCCAAACAACCAATTTCCTTAGGGTTGGTGTTGTTGATAGGTTCTATAATCTCATGTGTAGAAATTGC
GTTGGAAATCAGAAGGTTATTAGGGTTTTTGTTGTTTCTGACTTATGTTAGAGGTGTAATAGTACTATTCT
TATATGTGCTAAGAATTTATCCTAATGAACGGTTTAATTTGGAGTTTATTGTAATTGTAATAAGATGTATTG
TGACAAGGCTGGTTATCACAATGAATTATGAGAATGGGTCATTATTTCTTAGGTTTATAGCTGAAGGGAG
ACTCTATATTCTAATAGCGGGCGTATTGCTGTTTGTAATGTTAGTGGTGTCTTATTTGTGTATGAAAACTAT
GGTGCCACTTCGTAGGGTA"/>
<sequence id="seq_Mytilus_chilensisKP100300" spec="Sequence"
taxon="Mytilus_chilensisKP100300" totalcount="4"
value="ATGTTAATAGATGTATTTTCTAGATTTGATGCTCACAGCTACAACTTAATTTGGTTATCTATGTTGT
GGTTACTTTCGTCTATAGTACCAATAACTGTGCTATTTAGAGACGTAAGCACTCGAAGCTTGGTACTGTC
TTTTACTTATTCTATAATCCGGAATGGGAAAGGTTTAAAGTTATCTGGGTTTCCTTTGGTGATAAGCGGCT
TGTTTATAATAATTTTAATGTTGAATTTGTCTGGGAACTTCCCCTTTTTTTTCCCTGTAAGAGGTCAGTTTG
TATTTGGATTTTCTTTTGCTTTATCTATTTGGACCTGTTTAGTACTATCTAGCTTACTGTGCAGTTTTGAGC
AGGGTTTGATGAGGCTTGTCCCGACAGGTCCGTTAATCCTTGTGCCTTTTATAGTAGTAGTTGAGCTAAT
TAGTGGGATACTTCGCCCCTTAACTCTAGTCTTGCGGCTAACGTTGAATTTAGGGGCTGGTAAAGTAATC
TTAACTATATGTAGAAGGGAGTTGGTAGTCGGTTGGTTAATTACAGGAGTAGGGGGGATTAAAGGTTTGC
TAATAGGTGGCGTCTTTGCTGCTGAAGTTGCAATTGCTTGTATTCAGTGTTACATTTTCTGTGTGTTATTG
TGTCTCTATACTGAGGACCATAGGAGTTAGCGATGGTTGTGGTCAACAAATCATAAAGATATTGGTACTC
TTTATCTATATAGTGGGGTCTGAGGAGGCTTGTTTGGGGCGAGGTTAAGGCTAATAATCATACAAGGGCA
TCCGGGAGCAGTATTTTTAAAAGATTGGTTTTATAATGTGGTTGTTACAACACACGCCTTAATAATAATTT
TCTTTGCTGTAATACCGATTCTAATCGGAGCTTTTGGTAATTGGCTGATTCCTTTATTAGTAGGTGGTAAA
GATATAATTTATCCGCGGATAAATAATTTGAGTTATTGGTTATCTCCTAATGCGCTATATTTACTTATGCTAT
CTTTTAGAACGGATAAAGGAGTGGGTGCTGGATGGACTATTTACCCGCCATTGTCTGTATACCCTTATCAT
AGCGGGCCGAGGATAGATGTTCTTATTGTGTCCTTGCATTTAGCTGGGTTAAGTTCTTTGGTGGGTGCTAT
TAATTTTGCTAGTACCAACAAAAACATACCAGTTCTAGAGATAAAGGGAGAACGAGCTGAGCTTTATGT
CCTAAGGATCAGAGTTACTGCCGTATTGTTAATTATTTCTATCCCGGTTTTAGGAGGGGGCATTACAATAA
TTTTGTTTGATCGGAATTTTAACACAACATTTTTTGATCCAGCAGGAGGGGGTGATCCCGTTTTGTTTCA
ACATTTGTTTTGATTTTTTGGTCACCCTGAGGTGTACATTCTTATTCTACCTGCTTTTGGTGTGATATCAAA
AGTAATTATGCATTGTTCCGGAAAGGAGGCAGTTTTCGGGTTAATTGGGATGGTGTATGCAATAATTGGA
ATTGGAGGGCTAGGTTGTATGGTGTGGGCTCACCATATATTTACAGTAGGGCTTAATGTTGATACTCGAG
GTTATTTTTCTACTGCAACTATAGTAATCGCAGTCCCTACAGGGGTAAAAGTATTCAGGTGGTTAGCAAC
TATAGCAGGGAGAAAATTTAAGATAAAGCCTGCTGCTTACTGAAGTACGGGGTTCTTGTTTCTATTCACC
GTAGGAGGCTTAACAGGGGTGTTATTATCAAGGGCTTCTATGGACGTATCGCTTCATGATACTTATTATGT
GGTGGCTCATTTCCACTATGTGCTAAGTATAGGAGCGGTGTTCGGAGTATTTTGTGGCTTGAATCATTGAT
TGCCTAACTTTGTTGGAGTATGCTTTAATAAGAAATGGAGAAAGGCCCACTTTATAGCAATGTTTTTTGG
TGTAAATACCACTTTCTTTCCTCAACACTTTCTAGGGCTGAGGGGTATGCCTCGACGGTACATAGATTATG
CTGACATTTATGCTCATTGGCATTGGGTGTCTTCTTATGGGTCTGCAGTGTCTTTTGGCTCTTTGATATATT
TTAAGTTCCTACTCTGAGAAGCTTTAGTGAGTCAACGAGGGATGTCTTTTTATGGGAGTCGATATTTTGG
TGATATTGTCCATGAATTGGGGAAGGACCTATTTCGGTATCACGGATTTGTGATGATAGTAGCAGTGGCT
GTATTAGTTTTTGTTATGTATATAGGGTGCGTGATCCTGTTTACTAAATTTTCTTACCGTCATTTCTTAAATC
GCCAACGATTGGAGTTTTGATGAACTATTGTGCCGATGTTGATGTTAGTAGGGCTGTGGTTTCCTTCGAT
GATTAATTTGTATTATATGGAAGAAGTAAAGCGTCCACGTTGAAATTTCAAAGCAATTGGAAAGCAATGA
TACTGATCTTATGAGTGCGATACCTGTTATACAATTGACTCATACATAGAAGACCAACAGGAGACAGGGT
ACCGCTTGCTGGATGTTGACAATCGGATGGTTGCCCCTGCAGATGTACAAATAACTGCTTTTGTAAGGAG
TTCTGACGTGCTACATTCGTTTGCGCTTCCTAAATTATTAATCAAAGTAGATGCTATTCCGGGCCGAATCA
ATCGGCTTCCGATAAAAGCATCTCAGTGTAGTATTATTTATGGCCAGTGCTCTGAAATTTGTGGAGTAAA
CCACAGGTTTATACCAATTGTGATTGAATTTATTCCTGAGAAATATTTGTTATATGGTTGGAAGCTCTTAA
TTAG------------------------

ATGAATCGTAATCCTTACTATGTACCAGGTCCAAGTCCCTGGCCATTTTTTGTGGCTATTTCGGCTAACGG
AATAGCGGTAGGGTTAATTTTGTGACTGCATCGAACCCCCTTTTTATTAATAGGTAGTCTGGTTTGCATAT
TATTGAGAACTTTTAGATGGTGACGCGATTTAATTCGTGAAGGAGACATTGGTTTTCACACTCGTTTTGT
AATCAAAAGATTTCGAGATTGCGTTGCCTTGTTTATTCTGTCTGAAGTGATATTTTTCTTTACTTTTTTCTG
GACTTTTTTCCATAATGCTTTAAGGCCTTCGTGTGAGCTAGGAATACGGTGGCCTCCTCCTGGAATCCGT
ACGCCAAACCCCTCATCTACTAGTCTGTTTGAGACAGGTCTTCTAATTAGAAGAGGGCTGTTTGTAACTC
AAGCCCATAAGAGGATGCGCTTGGATTACGACGTAGGGCCATTCATCGGCTTAGTGGTGACAATCGTATG
CGGGACTGTGTTTTTCTTGGTACAACTGCGGGAATATTATTGAAACTCCTATACTATTGCAGATAGGGTTT
ATGGTAGGGTTTTTTACTTATTAACTGGCTTCCACGGGATACATGTTGTCGTAGGGACTATTTGGCTAATG
GTAAGGTTAGTTCGACTATGACGAGGGGAGTTTTCTAGCCAACGACACTTTGGGTTTGAGGCTTGCATT
TGGTACTGACATTTTGTAGATGTGGTATGAGTGGCATTGTGGTGCTTAGTGTATGTGTGGTTTGGAGGAC
CGTGACGAAGTACAAATAAACTGGTAAAGATTATGAATGATAGATTCTATGATTTGCCTTGCCCTGTAAA
CTTAAACGCCTGGTGAAGGTTTGGCTCTATACTAGGCTTGTGTCTGGTTATCCAACTTTTGAGGGGTCTT
TTATTATCAGCCCACTACACTGCTCATGAAGACATGGCATTTGACTCTGTAGTCCACATTATGCGTAATGT
GGAAAAAGGGTGAATATTGCGCAATATCCATGCAAATGGGTCTTCTATGTTCTTTATCTGCATTTATGCTC
ACATTGCTCGTGGGTTGTACTATGGGTCTTATTTGGATAAGACCGTGTGGTACTTTGGGGTACACCTGTT
CTTGTTGACTATGGCTGAGGCTTTTCTCGGCTATACTCTGCCTTGGGGGCAGATATCGTACTGGGGGGCT
ACTGTAATCACTAATATACTTAGAGTAAGCCCTGTGGTAGGTGAAAGGATACTTCGCTATGTATGAGGGG
GGTGGACTGTGTGTAATGCAACGTTAAAGCGGTTTTACACACTTCACTTCCTTTTACCGTTTGTAATAGT
GGCGGTTGTTTTTTTGCACTTATTTTTTTTACATGAGAAGGGTAGTAATAACCCTTTAGGAATTGAAAGG
GGTACTATATGTGTGCCTTTCCATCCTTTCTATACTATTAAAGACCTCTTTGGTTATGTTTGCTTTAGGTTCT
TTTTTATGTATTTAGTGTGCGTAGACCCTGAGCTTCTAGGGAATCATCTAAACTATTGGCCTGCTAACCCT
ATAAAAACGCCTATTCACGTTCAGCCTGAATGGTACTTTATATTTGCCTATGCAATTCTGCGTTCAATTCC
GCATAAAGCAGGGGGAGTATATGTTATGTTCCTTTCAATTGTAGTGCTGTACTTAATTCCTACGCTTCATA
CAGGTAAGTACGGAAGTTTATGCTTCTACCCATTGAATCAAGTAGTGTTTTGGGTGTTGGTTGGAAGGTT
TATTAGCCTAACATGAATTGGTGCTCGTCCAGTGCGTGAACCTTATATCATTTTGGGGCAGTGCCTTTCAG
TTATTTATTTCTCTAGTTTATTGTTAAACCCCCTTTCTTTATGGGTGTGAGATAAATTACTTGAAGTGGCAG
TAATTGTAAGTATTATTCCTTTCGTAGGTGTCCTTCTTGCCGTAGGGTTTTACACCTTATTGGAGCGTAAG
ATCCTGGCCATCATCATGATCCGAAAAGGCCCGTCCAAGGTAAGTTACATGGGGATCTTGCAGCCTTTTA
GTGATGCAGGTAAGCTATTATGTAAAGAGTTTATCGTGCCCACGCGTGCTAATGTAGGGCCATTTATTCTG
GCTCCTGCACTAATACTAGCTATTAGCTTATTAGGTTGGCTTTTATACCCTTATAAGTCAGCTGAAGTGTTT
TATGTTTTTGGGGTGATTTTGTTTATAGTTATTACGAGAGTTAGCGTTTATGGGGTAATAATGTCTGGATGG
GCATCTAACTCCAAATACTCCTTACTAGGTGCAGTCCGTGCGATGGCGCAAAGAATTTCTTATGAAATCC
CCATAGGGTTTATTTTTTTTTGTGTAGTGTTGTGTTCAGGCGTGTTTATGTTTCAAGAAATTAGAGTGTTA
TTTTTTTTCTTTCCTTTGTGCGTAGTTATACTTGTTTGAATGTTGTGTATACTAGCTGAGACTAATCGGGCA
CCGTTTGACTTTGTGGAAGGAGAGTCGGAGTTAGTGTCGGGTTATAACGTGGAGTACAGAGGAGGGGG
TTTTGCAGTTATATTTATTGCCGAGTACTCTAGAATTCTCCTTAGAAGAGTAATAAGGGCGGCGATATTCT
TCGGAGGAAATGAAGCGTTAGTTGGGTTTTTCATAATGGTTTTCGCAATTTTCTTTGTAGTTGTTCGTGCT
TCTTTACCACGCTTACGTTATGACAAGCTAATAAACTTATGTTGAACTGTCCTTTTATGTGTTATACTTACG
GCTAGTGTGTGTGTAGTAGTCTTAGTGGGGGTGTAATGGTAAGTTTTGTGGTGAGACCTATAAAATTAGT
GAGTTTAGGGGTAATATTAATTGGAACAATCCTCAGGGTCAGAAGAGAGGAGCTAGTAGGGGTGTGGTT
AGGTTTGGAGTTAAATCTTTATGGTTTTCTTGTGATTATAAACCCAGATGGGCATTATAGCCCTGAGCCTT
GCGTAAAATACTTTGTTGTACAAAGAACGGGGTCAATTTTGATACTAGTGGGGTTTGTAAGCTTAATACA
GCATGTAGTGAGGGGGTTAGTGATGAGCACAGCGGGTACGGTGCTAAAGTCTGGCGTTTTTCCTCTACA
CTCGTGGGTGCCCTCCATTATTAAAAACAGGAGATGGTTAGCAAGGGGGTTAATACTAACCTGGCAAAA
AGTGGCCCCCCTTGTTTTTTTATCAATAATCTTACCTTCTAAAAGGCTTTGAGTAGTGATTGTATCGATAG
CTGGGATTGGGGCAGTTGGAGGGCTTAACCAAAACTCAGTGCGGGTAATAAGAGCCTACTCTTCATTTG
TTCATACATCTTGAATGTTGTTAGGGCTTACATGATCAAGGGTAGTTTTCGTAGGGTACTTTGCAGTCTAT
AGGTTATCGGTAGGGCTGTTTTTCTACGGGTGCTCGTTAATAAACAAGATAAGAATAGGTAGCCAGCTAA
GTAGGGCTGCTAGCGGTATGGGGTTGTTAATACTTATAGGGATGCCTCCGTTTCTTGGTTTCTTAGCAAA
AGTATTGGTATTTTTGATGAGGGGAAGGCCCGTAATTGTGGCATGCATCATAGGATCAGTCATTAGATTAA
AATTTATATTGATTTCTTTTACAGAATGGTAATAAAAAATAAGGTAGAAGTTAAAGCCATTTGGAGCCTG
GTGATTTGCATAAACATTATGGGGGGGGCGCTGATTTTAGTAAGATTTATTTAGATGGTTATGGGGTTAAG
GGTTGTATTCGTTTGTATCGTGTGTTGTCTGTTCACAGGGTTAATAATATTAAGAGAAAAACGGGGGCTG
GACCGAGAGAAATGTAGGCCGTATGAATGTGGGTTCGAGCCTATCGGAAGAGCTCGAAGCCCTTTCTCC
ATTCGATTCTTTTTAGTGGCAGTTTTGTTTGTGGTGTTTGATGTGGAGGTAGTCCTATTGATACCTTTTGC
TTACATATTTTTTTATGGCAAAAGAGTGTTGGGTATTTTATCTTCAAGGGGGTTCCTCCTTATTCTGTTTGT
GGGCCTATATCATGAATACCGTGAAGGTTCTTTGGAGTGAGTGGGTTAAATGGTTAAAAGGTTAGCAATT
AGGCTTATTGCATTAATTATTATAAAAAACCTGAATGTGAGTATTATTGGGTTAAGTGTTTTAACAATTTTG
AGTATAGGTGCCGCAAGAGTTGCGACAGGAGGGGTAGAATTAAATGGGCTATACTCGACGGACTTCGTG
ATGGGTTTGATGATCACATTGACATTATTTGTCGCAATTCTTTCTTATTTAAGTAGAGCTAAAGTCTCCCG
GAAAGCAAGATTTAACTTAATGGTTATCAGAATTAGTCTTATTTAGTGATAAGGTTTAGGGTGAGGAGG
TTTTTTTTTTTTTTCTTTTTTTTCGAAAGTGTGTTAGCGCCTTTGTTACTGTTAATCGTAGGCTGGCGTCTA

CAGGCAGGAGGCTATATAGTGATTTATACTGTATTTGGGTCGCTTTTTTTTCTATGGGGAGTAAGAGAGCT
TTACCTAAGAGGGAGAAGAAGGATGAGAGTGGGAAGATTAGTAAAAAAAAGGGCTATAAGACTGTGAT
GGCTGTACATTTTAGGGTTCCTAATTAAGTTACCAATATACCCGTTCCATTTGTGGCTCCCTAAGGCCCAT
GTAGAGGCCCCCGTGGCAGGTTCAATGCTGCTGGCCGGGGTAGTACTAAAATTAGGAGGATATGGCCTA
CTTCGGTTTATATTAGTAATACAAGTCAGGCTAAGAAGAGTGTTTGTGTTGCTACTAAGGGTAAATTTGG
CAGGGGGCTTCTACGCCGGACTGGCCTGTGTACGGCAAGTAGACCTAAAATGCTTGGTGGCATACTCGT
CCGTAGCTCATATAAGTCTAGTCCTATTAGTGCTAAGCAACACGCTATTAGGGGTGATAGGGGCTATTATT
ATTATGGTTGGCCACGGGTTATGCTCGTCAGGTTTATTTAGGTACGTAAATGCTATTTATAAGATGAGTCA
CTCGCGCTTACTAGTAATAAACAAAGGTGGGTTACTTTTTTGCCCTGTATTAGTGTTGATGTGTTTTCTTC
TAAGATCAAGGAACATGGCAGCCCCACCTAGCCTTAATCTATTCGGGGAAATCCTCGTTTTTGGCGTCGG
NGGGTGAATAAGGGGGGCCTTTTTATTTATCCTGGGGTTGATGAGCTTCATTAGGGCTTGCTTTAGATTAT
ATTTATATGGGAGGTGCTCACACGGAAAAGGGCTTATACATAGGGAGTCGCTGAATTTACTCTGCGATGT
GTTCGTCTTGTTGTCTCATTGGGTGCCGTTAAATTTCTTGTTTATATTCATACCTTAACGTATAAATAAACA
CCTATTGTGTCTGTTTGTAGGGCTTGAGATAATGAGGCTGGGCTTACTTTTTGTAACACATGTTTTCTTAA
TAAATCAGTTTTGACTAATTTTATTAATTCTATGTTTAGCTGTTTGTGAAGCTAGCATTTGTTTGGCCCTGC
TGGTCATAGTGATGCGATTATGCGGCGACGACCTAATATCAAGATTGCTAAGAGATGTATACAATCAAAG
GGTAACCTAGGGTTGCTTATATTAATCTTAGGGTATTTATTCATTTTTACAGGAAGGAGGGGAAGAGCGTA
CTTACTTGAAGTTCCTGTTTGAGAGAGCAATTGTCTGTCTTTTAGTTTTAGATTCTTACTAGATAATGTAA
GGATAATTTTTGTTGGGACAGTTTTGGTGATCAGAGGAAGTGTAGCAACCTATTGTAAGTGGTATATAGC
TGAAGAGCTATACTACAACCGGTTTATGGGGTTGGTGTGGTTATTTGTTTTATCTATAGTGTTTATAATCTT
AGTGCCTAATCTACTAATACTTTTAATTGGATGGGATGGGCTAGGCCTCACTTCTTTTCTATTAGTGGCTT
ACTACCAAAATAACAAAAGACTATCTGCGGCTATGTTGACAGCCTTAACTAATCGAATTGGAGACGTTCT
TGTTCTAATTAGCATTTCTATCTTTCTAAGAGAGGGGGGGTGGTTAATTTATAGGTATCACCCAGTACAAA
TATGGTTGTTAGGTTTTATAGTGGTTTTTGCAGGGATAACTAAAAGAGCACAGATGCCATTTTGCGCATG
ACTGCCAGCTGCCATAGCCGCACCGACACCTGTCTCTTCTTTAGTGCACTCTTCAACACTGGTAACAGC
TGGGGTTTATCTTGTACTTCGTTCGTTCTATATCATCAGTTCTAACGTAACACAAGTTCTAATAATTTTAAG
GTTATTTACCTTGGTACTAGCTGGATCAAGTGCAGTGTTCGCGTTTGATCTAAAAAAAGTAATTGCACTC
TCGACTTTAAGTCAACTAAGGTTGATAATGTTTTCGATTTCAATCCTCCTTCCGTCTGTGGCGTTTTTCCA
TCTGGTGACTCATGCAGTGTTTAAAGCTTTGCTGTTTTTGGGTGCAGGGGGTGTTATTCATAGAAACCAA
AGAATTCAGGATATTCGTGGGTTAAGAAGACTGTGACAAGGGTTGCCGGTAAGAATAGGTGCAATAAGA
GTTGCAATTGTATCTTTAAGAGGGGCTCCTTTTATGAGAGGTTTTTTCTCCAAAGACCTCATGATTGAGAT
GATAGACAGAAGGACTTATGGGTACTTATTAGAGTTACTAGGGTTAATTTTTACTTCTTTTTATAGGGCAC
GAGTATTTAGAGTAATACTCGGGTCCAATTATGTTAATAATAGAACCTTGCGTATTAATGAGCATTTAAATA
TACAAACCCCGTTTCTTAGCCTATATATTGGAGCTATTATTTTAGGGGTGGTGCTGGGTAGAAAAATAGAA
AGATTTGGGTTCGTAGTAGTTCTTGAAAACTATGAAAGACTTAGAGTCTTTTTTATTCCTTTTGGGTTACT
GTGACGAGGAGTCATTAGTAAGCTAGGGTCGAGTCCTGCTAAGTTAAGATTTTTCTTGAGGATGTGGTTT
ATAGAATTAACTCATCCTGGGAAGGTGATCTTTTTTAAAGGGTCAAGAACAGTGGCCCAAAGTTTGGAC
CAAGGTTGACTAGAGCTATTGGGGCCTCAAGCTGGATTAGGTCAATTCAGTTGCTTAAATGAGAACTATT
TTACTGTGGTGTGATTAGTAGCCGGGGGCGTTAGCTTAATTATTTTTATATAAATGAGAGTTATAATTATAT
GTGTAAGTTTGATGTCTGTATTCGTGTTGATTGCTAAGCAACCAATCTCTTTAGGGCTAGTATTATTAAGG
GGGTCTATAATTGCATGTGTGGAAATTGCATTGGAGGTTAGAAGTCTGTTAGGGTTCTTGTTATTTTTAAC
CTATGTTAGGGGCGTGATAGTCTTGTTCTTATATGTCCTTAGTATTTATCCTAATGAGCGCTTTAATTTAGA
GTTTGTTATAATTGTGAGAAGGTGCGCTATGATGGGCCTTGTGGGATTAATGAATTATGAGAGTGGGTCA
TTATTTTTAAGGTTTATGGCTGAAGGAAGGCTATATATCCTAATAGCAGGCGTTTTGCTGTTTGTGATGTTA
GTGGTGTCGTATTTGTGTATAAAAACCATAGTGCCTTTGCGAAGAGTA"/>
<sequence id="seq_Mytilus_chilensisNC030633" spec="Sequence" taxon="Mytilus_chilensisNC030633" totalcount="4" value="ATGTTAATAGATGTATTTTCTAGATTTGATGCTCACAGCTACAACTTAATTTGGTTATCTATGTTGT
GGTTACTTTCGTCTATAGTACCAATAACTGTGCTATTTAGAGACGTAAGCACTCGAAGCTTGGTACTGTC
TTTTACTTATTCTATAATCCGGAATGGGAAAGGTTTAAAGTTATCTGGGTTTCCTTTGGTGATAAGCGGCT
TGTTTATAATAATTTTAATGTTGAATTTGTCTGGGAACTTCCCCTTTTTTTTCCCTGTAAGAGGTCAGTTTG
TATTTGGATTTTCTTTTGCTTTATCTATTTGGACCTGTTTAGTACTATCTAGCTTACTGTGCAGTTTTGAGC
AGGGTTTGATGAGGCTTGTCCCGACAGGTCCGTTAATCCTTGTGCCTTTTATAGTAGTAGTTGAGCTAAT
TAGTGGGATACTTCGCCCCTTAACTCTAGTCTTGCGGCTAACGTTGAATTTAGGGGCTGGTAAAGTAATC
TTAACTATATGTAGAAGGGAGTTGGTAGTCGGTTGGTTAATTACAGGAGTAGGGGGGATTAAAGGTTTGC
TAATAGGTGGCGTCTTTGCTGCTGAAGTTGCAATTGCTTGTATTCAGTGTTACATTTTCTGTGTGTTATTG
TGTCTCTATACTGAGGACCATAGGAGTTAGCGATGGTTGTGGTCAACAAATCATAAAGATATTGGTACTC
TTTATCTATATAGTGGGGTCTGAGGAGGCTTGTTTGGGGCGAGGTTAAGGCTAATAATCATACAAGGGCA
TCCGGGAGCAGTATTTTTAAAAGATTGGTTTTATAATGTGGTTGTTACAACACACGCCTTAATAATAATTT
TCTTTGCTGTAATACCGATTCTAATCGGAGCTTTTGGTAATTGGCTGATTCCTTTATTAGTAGGTGGTAAA
GATATAATTTATCCGCGGATAAATAATTTGAGTTATTGGTTATCTCCTAATGCGCTATATTTACTTATGCTAT
CTTTTAGAACGGATAAAGGAGTGGGTGCTGGATGGACTATTTACCCGCCATTGTCTGTATACCCTTATCAT
AGCGGGCCGAGGATAGATGTTCTTATTGTGTCCTTGCATTTAGCTGGGTTAAGTTCTTTGGTGGGTGCTAT

TAATTTTGCTAGTACCAACAAAAACATACCAGTTCTAGAGATAAAGGGAGAACGAGCTGAGCTTTATGT
CCTAAGGATCAGAGTTACTGCCGTATTGTTAATTATTTCTATCCCGGTTTTAGGAGGGGGCATTACAATAA
TTTTGTTTGATCGGAATTTTAACACAACATTTTTTGATCCAGCAGGAGGGGGTGATCCCGTTTTGTTTCA
ACATTTGTTTTGATTTTTTGGTCACCCTGAGGTGTACATTCTTATTCTACCTGCTTTTGGTGTGATATCAAA
AGTAATTATGCATTGTTCCGGAAAGGAGGCAGTTTTCGGGTTAATTGGGATGGTGTATGCAATAATTGGA
ATTGGAGGGCTAGGTTGTATGGTGTGGGCTCACCATATATTTACAGTAGGGCTTAATGTTGATACTCGAG
GTTATTTTTCTACTGCAACTATAGTAATCGCAGTCCCTACAGGGGTAAAAGTATTCAGGTGGTTAGCAAC
TATAGCAGGGAGAAAATTTAAGATAAAGCCTGCTGCTTACTGAAGTACGGGGTTCTTGTTTCTATTCACC
GTAGGAGGCTTAACAGGGGTGTTATTATCAAGGGCTTCTATGGACGTATCGCTTCATGATACTTATTATGT
GGTGGCTCATTTCCACTATGTGCTAAGTATAGGAGCGGTGTTCGGAGTATTTTGTGGCTTGAATCATTGAT
TGCCTAACTTTGTTGGAGTATGCTTTAATAAGAAATGGAGAAAGGCCCACTTTATAGCAATGTTTTTTGG
TGTAAATACCACTTTCTTTCCTCAACACTTTCTAGGGCTGAGGGGTATGCCTCGACGGTACATAGATTATG
CTGACATTTATGCTCATTGGCATTGGGTGTCTTCTTATGGGTCTGCAGTGTCTTTTGGCTCTTTGATATATT
TTAAGTTCCTACTCTGAGAAGCTTTAGTGAGTCAACGAGGGATGTCTTTTTATGGGAGTCGATATTTTGG
TGATATTGTCCATGAATTGGGGAAGGACCTATTTCGGTATCACGGATTTGTGATGATAGTAGCAGTGGCT
GTATTAGTTTTTGTTATGTATATAGGGTGCGTGATCCTGTTTACTAAATTTTCTTACCGTCATTTCTTAAATC
GCCAACGATTGGAGTTTTGATGAACTATTGTGCCGATGTTGATGTTAGTAGGGCTGTGGTTTCCTTCGAT
GATTAATTTGTATTATATGGAAGAAGTAAAGCGTCCACGTTGAAATTTCAAAGCAATTGGAAAGCAATGA
TACTGATCTTATGAGTGCGATACCTGTTATACAATTGACTCATACATAGAAGACCAACAGGAGACAGGGT
ACCGCTTGCTGGATGTTGACAATCGGATGGTTGCCCCTGCAGATGTACAAATAACTGCTTTTGTAAGGAG
TTCTGACGTGCTACATTCGTTTGCGCTTCCTAAATTATTAATCAAAGTAGATGCTATTCCGGGCCGAATCA
ATCGGCTTCCGATAAAAGCATCTCAGTGTAGTATTATTTATGGCCAGTGCTCTGAAATTTGTGGAGTAAA
CCACAGGTTTATACCAATTGTGATTGAATTTATTCCTGAGAAATATTTTGTTATATGGTTGGAAGCTCTTAA
TTAG-----------------------
ATGAATCGTAATCCTTACTATGTACCAGGTCCAAGTCCCTGGCCATTTTTTGTGGCTATTTCGGCTAACGG
AATAGCGGTAGGGTTAATTTTGTGACTGCATCGAACCCCCTTTTTATTAATAGGTAGTCTGGTTTGCATAT
TATTGAGAACTTTTAGATGGTGACGCGATTTAATTCGTGAAGGAGACATTGGTTTTCACACTCGTTTTGT
AATCAAAAGATTTCGAGATTGCGTTGCCTTGTTTATTCTGTCTGAAGTGATATTTTTCTTTACTTTTTTCTG
GACTTTTTTCCATAATGCTTTAAGGCCTTCGTGTGAGCTAGGAATACGGTGGCCTCCTCCTGGAATCCGT
ACGCCAAACCCCTCATCTACTAGTCTGTTTGAGACAGGTCTTCTAATTAGAAGAGGGCTGTTTGTAACTC
AAGCCCATAAGAGGATGCGCTTGGATTACGACGTAGGGCCATTCATCGGCTTAGTGGTGACAATCGTATG
CGGGACTGTGTTTTTCTTGGTACAACTGCGGGAATATTATTGAAACTCCTATACTATTGCAGATAGGGTTT
ATGGTAGGGTTTTTTACTTATTAACTGGCTTCCACGGGATACATGTTGTCGTAGGGACTATTTGGCTAATG
GTAAGGTTAGTTCGACTATGACGAGGGGAGTTTTCTAGCCAACGACACTTTGGGTTTGAGGCTTGCATT
TGGTACTGACATTTTGTAGATGTGGTATGAGTGGCATTGTGGTGCTTAGTGTATGTGTGGTTTGGAGGAC
CGTGACGAAGTACAAATAAACTGGTAAAGATTATGAATGATAGATTCTATGATTTGCCTTGCCCTGTAAA
CTTAAACGCCTGGTGAAGGTTTGGCTCTATACTAGGCTTGTGTCTGGTTATCCAACTTTTGAGGGGTCTT
TTATTATCAGCCCACTACACTGCTCATGAAGACATGGCATTTGACTCTGTAGTCCACATTATGCGTAATGT
GGAAAAAGGGTGAATATTGCGCAATATCCATGCAAATGGGTCTTCTATGTTCTTTATCTGCATTTATGCTC
ACATTGCTCGTGGGTTGTACTATGGGTCTTATTTGGATAAGACCGTGTGGTACTTTGGGGTACACCTGTT
CTTGTTGACTATGGCTGAGGCTTTTCTCGGCTATACTCTGCCTTGGGGGCAGATATCGTACTGGGGGGCT
ACTGTAATCACTAATATACTTAGAGTAAGCCCTGTGGTAGGTGAAAGGATACTTCGCTATGTATGAGGGG
GGTGGACTGTGTGTAATGCAACGTTAAAGCGGTTTTACACACTTCACTTCCTTTTACCGTTTGTAATAGT
GGCGGTTGTTTTTTTGCACTTATTTTTTTTACATGAGAAGGGTAGTAATAACCCTTTAGGAATTGAAAGG
GGTACTATATGTGTGCCTTTCCATCCTTTCTATACTATTAAAGACCTCTTTGGTTATGTTTGCTTTAGGTTCT
TTTTTATGTATTTAGTGTGCGTAGACCCTGAGCTTCTAGGGAATCATCTAAACTATTGGCCTGCTAACCCT
ATAAAAACGCCTATTCACGTTCAGCCTGAATGGTACTTTATATTTGCCTATGCAATTCTGCGTTCAATTCC
GCATAAAGCAGGGGGAGTATATGTTATGTTCCTTTCAATTGTAGTGCTGTACTTAATTCCTACGCTTCATA
CAGGTAAGTACGGAAGTTTATGCTTCTACCCATTGAATCAAGTAGTGTTTTGGGTGTTGGTTGGAAGGTT
TATTAGCCTAACATGAATTGGTGCTCGTCCAGTGCGTGAACCTTATATCATTTTGGGGCAGTGCCTTTCAG
TTATTTATTTCTCTAGTTTATTGTTAAACCCCCTTTCTTTATGGGTGTGAGATAAATTACTTGAAGTGGCAG
TAATTGTAAGTATTATTCCTTTCGTAGGTGTCCTTCTTGCCGTAGGGTTTTACACCTTATTGGAGCGTAAG
ATCCTGGCCATCATCATGATCCGAAAAGGCCCGTCCAAGGTAAGTTACATGGGGATCTTGCAGCCTTTTA
GTGATGCAGGTAAGCTATTATGTAAAGAGTTTATCGTGCCCACGCGTGCTAATGTAGGGCCATTTATTCTG
GCTCCTGCACTAATACTAGCTATTAGCTTATTAGGTTGGCTTTTATACCCTTATAAGTCAGCTGAAGTGTTT
TATGTTTTTGGGGTGATTTTGTTTATAGTTATTACGAGAGTTAGCGTTTATGGGGTAATAATGTCTGGATGG
GCATCTAACTCCAAATACTCCTTACTAGGTGCAGTCCGTGCGATGGCGCAAAGAATTTCTTATGAAATCC
CCATAGGGTTTATTTTTTTTTGTGTAGTGTTGTGTTCAGGCGTGTTTATGTTTCAAGAAATTAGAGTGTTA
TTTTTTTTCTTTCCTTTGTGCGTAGTTATACTTGTTTGAATGTTGTGTATACTAGCTGAGACTAATCGGGCA
CCGTTTGACTTTGTGGAAGGAGAGTCGGAGTTAGTGTCGGGTTATAACGTGGAGTACAGAGGAGGGGG
TTTTGCAGTTATATTTATTGCCGAGTACTCTAGAATTCTCCTTAGAAGAGTAATAAGGGCGGCGATATTCT
TCGGAGGAAATGAAGCGTTAGTTGGGTTTTTCATAATGGTTTTCGCAATTTTCTTTGTAGTTGTTCGTGCT
TCTTTACCACGCTTACGTTATGACAAGCTAATAAACTTATGTTGAACTGTCCTTTTATGTGTTATACTTACG

GCTAGTGTGTGTGTAGTAGTCTTAGTGGGGGTGTAATGGTAAGTTTTGTGGTGAGACCTATAAAATTAGT
GAGTTTAGGGGTAATATTAATTGGAACAATCCTCAGGGTCAGAAGAGAGGAGCTAGTAGGGGTGTGGTT
AGGTTTGGAGTTAAATCTTTATGGTTTTCTTGTGATTATAAACCCAGATGGGCATTATAGCCCTGAGCCTT
GCGTAAAATACTTTGTTGTACAAAGAACGGGGTCAATTTTGATACTAGTGGGGTTTGTAAGCTTAATACA
GCATGTAGTGAGGGGGTTAGTGATGAGCACAGCGGGTACGGTGCTAAAGTCTGGCGTTTTTCCTCTACA
CTCGTGGGTGCCCTCCATTATTAAAAACAGGAGATGGTTAGCAAGGGGGTTAATACTAACCTGGCAAAA
AGTGGCCCCCCTTGTTTTTTATCAATAATCTTACCTTCTAAAAGGCTTTGAGTAGTGATTGTATCGATAG
CTGGGATTGGGGCAGTTGGAGGGCTTAACCAAAACTCAGTGCGGGTAATAAGAGCCTACTCTTCATTTG
TTCATACATCTTGAATGTTGTTAGGGCTTACATGATCAAGGGTAGTTTTCGTAGGGTACTTTGCAGTCTAT
AGGTTATCGGTAGGGCTGTTTTTCTACGGGTGCTCGTTAATAAACAAGATAAGAATAGGTAGCCAGCTAA
GTAGGGCTGCTAGCGGTATGGGGTTGTTAATACTTATAGGGATGCCTCCGTTTCTTGGTTTCTTAGCAAA
AGTATTGGTATTTTTGATGAGGGGAAGGCCCGTAATTGTGGCATGCATCATAGGATCAGTCATTAGATTAA
AATTTATATTGATTTCTTTTACAGAATGGTAATAAAAAATAAGGTAGAAGTTAAAGCCATTTGGAGCCTG
GTGATTTGCATAAACATTATGGGGGGGGCGCTGATTTTAGTAAGATTTATTTAGATGGTTATGGGGTTAAG
GGTTGTATTCGTTTGTATCGTGTGTTGTCTGTTCACAGGGTTAATAATATTAAGAGAAAAACGGGGGCTG
GACCGAGAGAAATGTAGGCCGTATGAATGTGGGTTCGAGCCTATCGGAAGAGCTCGAAGCCCTTTCTCC
ATTCGATTCTTTTTAGTGGCAGTTTTGTTTGTGGTGTTTGATGTGGAGGTAGTCCTATTGATACCTTTTGC
TTACATATTTTTTTATGGCAAAAGAGTGTTGGGTATTTTATCTTCAAGGGGGGTTCCTCCTTATTCTGTTTGT
GGGCCTATATCATGAATACCGTGAAGGTTCTTTGGAGTGAGTGGGTTAAATGGTTAAAAGGTTAGCAATT
AGGCTTATTGCATTAATTATTATAAAAAACCTGAATGTGAGTATTATTGGGTTAAGTGTTTTAACAATTTTG
AGTATAGGTGCCGCAAGAGTTGCGACAGGAGGGGTAGAATTAAATGGGCTATACTCGACGGACTTCGTG
ATGGGTTTGATGATCACATTGACATTATTTGTCGCAATTCTTTCTTATTTAAGTAGAGCTAAAGTCTCCCG
GAAAGCAAGATTTAACTTAATGGTTATCAGAATTAGTCTTATTTTAGTGATAAGGTTTAGGGTGAGGAGG
TTTTTTTTTTTTTTCTTTTTTTTCGAAAGTGTGTTAGCGCCTTTGTTACTGTTAATCGTAGGCTGGCGTCTA
CAGGCAGGAGGCTATATAGTGATTTATACTGTATTTGGGTCGCTTTTTTTTTCTATGGGGAGTAAGAGAGCT
TTACCTAAGAGGGAGAAGAAGGATGAGAGTGGGAAGATTAGTAAAAAAAAAGGGCTATAAGACTGTGAT
GGCTGTACATTTTAGGGTTCCTAATTAAGTTACCAATATACCCGTTCCATTTGTGGCTCCCTAAGGCCCAT
GTAGAGGCCCCCGTGGCAGGTTCAATGCTGCTGGCCGGGGTAGTACTAAAATTAGGAGGATATGGCCTA
CTTCGGTTTATATTAGTAATACAAGTCAGGCTAAGAAGAGTGTTTGTGTTGCTACTAAGGGTAAATTTGG
CAGGGGGCTTCTACGCCGGACTGGCCTGTGTACGGCAAGTAGACCTAAAATGCTTGGTGGCATACTCGT
CCGTAGCTCATATAAGTCTAGTCCTATTAGTGCTAAGCAACACGCTATTAGGGGTGATAGGGGCTATTATT
ATTATGGTTGGCCACGGGTTATGCTCGTCAGGTTTATTTAGGTACGTAAATGCTATTTATAAGATGAGTCA
CTCGCGCTTACTAGTAATAAACAAAGGTGGGTTACTTTTTTGCCCTGTATTAGTGTTGATGTGTTTTCTTC
TAAGATCAAGGAACATGGCAGCCCCACCTAGCCTTAATCTATTCGGGGAAATCCTCGTTTTTGGCGTCGG
NGGGTGAATAAGGGGGGCCTTTTTATTTATCCTGGGGTTGATGAGCTTCATTAGGGCTTGCTTTAGATTAT
ATTTATATGGGAGGTGCTCACACGGAAAAGGGCTTATACATAGGGAGTCGCTGAATTTACTCTGCGATGT
GTTCGTCTTGTTGTCTCATTGGGTGCCGTTAAATTTCTTGTTTATATTCATACCTTAACGTATAAATAAACA
CCTATTGTGTCTGTTTGTAGGGCTTGAGATAATGAGGCTGGGCTTACTTTTTGTAACACATGTTTTCTTAA
TAAATCAGTTTTGACTAATTTTATTAATTCTATGTTTAGCTGTTTGTGAAGCTAGCATTTGTTTGGCCCTGC
TGGTCATAGTGATGCGATTATGCGGCGACGACCTAATATCAAGATTGCTAAGAGATGTATACAATCAAAG
GGTAACCTAGGGTTGCTTATATTAATCTTAGGGTATTTATTCATTTTTACAGGAAGGAGGGGAAGAGCGTA
CTTACTTGAAGTTCCTGTTTGAGAGAGCAATTGTCTGTCTTTTAGTTTTAGATTCTTACTAGATAATGTAA
GGATAATTTTTGTTGGGACAGTTTTGGTGATCAGAGGAAGTGTAGCAACCTATTGTAAGTGGTATATAGC
TGAAGAGCTATACTACAACCGGTTTATGGGGTTGGTGTGGTTATTTGTTTTATCTATAGTGTTTATAATCTT
AGTGCCTAATCTACTAATACTTTTAATTGGATGGGATGGGCTAGGCCTCACTTCTTTTCTATTAGTGGCTT
ACTACCAAAATAACAAAAGACTATCTGCGGCTATGTTGACAGCCTTAACTAATCGAATTGGAGACGTTCT
TGTTCTAATTAGCATTTCTATCTTTCTAAGAGAGGGGGGGTGGTTAATTTATAGGTATCACCCAGTACAAA
TATGGTTGTTAGGTTTTATAGTGGTTTTTGCAGGGATAACTAAAAGAGCACAGATGCCATTTTGCGCATG
ACTGCCAGCTGCCATAGCCGCACCGACACCTGTCTCTTCTTTAGTGCACTCTTCAACACTGGTAACAGC
TGGGGTTTATCTTGTACTTCGTTCGTTCTATATCATCAGTTCTAACGTAACACAAGTTCTAATAATTTTAAG
GTTATTTACCTTGGTACTAGCTGGATCAAGTGCAGTGTTCGCGTTTGATCTAAAAAAAGTAATTGCACTC
TCGACTTTAAGTCAACTAAGGTTGATAATGTTTTCGATTTCAATCCTCCTTCCGTCTGTGGCGTTTTTCCA
TCTGGTGACTCATGCAGTGTTTAAAGCTTTGCTGTTTTTGGGTGCAGGGGGTGTTATTCATAGAAACCAA
AGAATTCAGGATATTCGTGGGTTAAGAAGACTGTGACAAGGGTTGCCGGTAAGAATAGGTGCAATAAGA
GTTGCAATTGTATCTTTAAGAGGGGCTCCTTTTATGAGAGGTTTTTTCTCCAAAGACCTCATGATTGAGAT
GATAGACAGAAGGACTTATGGGTACTTATTAGAGTTACTAGGGTTAATTTTTACTTCTTTTTATAGGGCAC
GAGTATTTAGAGTAATACTCGGGTCCAATTATGTTAATAATAGAACCTTGCGTATTAATGAGCATTTAAATA
TACAAACCCCGTTTCTTAGCCTATATATTGGAGCTATTATTTTAGGGGTGGTGCTGGGTAGAAAAATAGAA
AGATTTGGGTTCGTAGTAGTTCTTGAAAACTATGAAAGACTTAGAGTCTTTTTTATTCCTTTTGGGTTACT
GTGACGAGGAGTCATTAGTAAGCTAGGGTCGAGTCCTGCTAAGTTAAGATTTTTCTTGAGGATGTGGTTT
ATAGAATTAACTCATCCTGGGAAGGTGATCTTTTTTAAAGGGTCAAGAACAGTGGCCCAAAGTTTGGAC
CAAGGTTGACTAGAGCTATTGGGGCCTCAAGCTGGATTAGGTCAATTCAGTTGCTTAAATGAGAACTATT
TTACTGTGGTGTGATTAGTAGCCGGGGGCGTTAGCTTAATTATTTTTATATAAATGAGAGTTATAATTATAT

GTGTAAGTTTGATGTCTGTATTCGTGTTGATTGCTAAGCAACCAATCTCTTTAGGGCTAGTATTATTAAGG
GGGTCTATAATTGCATGTGTGGAAATTGCATTGGAGGTTAGAAGTCTGTTAGGGTTCTTGTTATTTTTAAC
CTATGTTAGGGGCGTGATAGTCTTGTTCTTATATGTCCTTAGTATTTATCCTAATGAGCGCTTTAATTTAGA
GTTTGTTATAATTGTGAGAAGGTGCGCTATGATGGGCCTTGTGGGATTAATGAATTATGAGAGTGGGTCA
TTATTTTTAAGGTTTATGGCTGAAGGAAGGCTATATATCCTAATAGCAGGCGTTTTGCTGTTTGTGATGTTA
GTGGTGTCGTATTTGTGTATAAAAACCATAGTGCCTTTGCGAAGAGTA"/>
<sequence id="seq_Mytilus_edulisF-MF407676" spec="Sequence" taxon="Mytilus_edulisF-MF407676" totalcount="4"
value="ATGTTAATAGATGTATTTTCTAGATTTGATGCTCACAGCTACAACCTAATTTGGTTATCTATGTTGT
GGCTACTTTCGTCTATAGTACCAATAACTGTGCTATTTAGAGATGTAAGCACTCGAAGCTTGGTACTGTCT
TTTACTTATTCTATAATCCGGAATGGGAAAGGTTTAAAGTTATCTGGGTTTCCTTTGGTGATAAGCGGCTT
GTTTATAATAATTTTAATGTTGAATTTGTCTGGGAACTTCCCTTTTTTTTTTCCTGTAAGAGGTCAGTTTGT
ATTTGGATTTTCTTTTGCTTTATCTATTTGGACCTGTTTAGTACTATCTAGCTTACTGTGCAGTTTTGAGCA
GGGTTTGATGAGGCTTGTTCCGACAGGTCCGTTAATCCTTGTGCCTTTTATAGTAGTAGTTGAGCTAATTA
GTGGGATACTTCGCCCTTTAACTCTAGTCTTGCGGCTAACGTTGAATTTAGGGGCTGGTAAAGTAATCTT
AACTATATGTAGAAGGGAGTTGGTAGTCGGTTGGTTAATTACAGGAGTTGGGGGTATTAAAGGTTTGCTA
ATAGGTGGTGTCTTTGCTGCTGAAGTTGCAATTGCTTGTATTCAGTGTTACATTTTTTGTGTCTTATTGTG
TCTCTATACTGAGGACCATAGGAGTTAGCGATGGTTGTGGTCAACAAATCATAAAGATATTGGGACTCTT
TATCTATATAGTGGGGTCTGAGGAGGCTTGTTCGGGGCAAGGTTAAGTCTGATAATCATACAGGGGCATC
CTGGAGCAGTATTTTTAAAAGACTGGTTTTATAATGTGGTTGTTACAACACACGCCTTAATAATAATTTTC
TTTGCTGTAATACCTATTCTAATCGGAGCTTTTGGTAATTGGCTGATTCCTCTATTAGTAGGTGGTAAAGAT
ATAATTTATCCGCGGATAAATAATTTGAGTTATTGGTTATCTCCTAATGCGCTATATTTACTTATATTATCTTT
TAGAACGGATAAAGGGGTAGGTGCTGGATGGACTATTTACCCGCCATTGTCTGTATACCCTTATCATAGC
GGGCCGAGGATAGATGTTCTTATTGTGTCGTTGCATTTAGCTGGGTTAAGTTCTTTGGTGGGTGCTATTAA
TTTTGCTAGTACCAACAAAAACATACCAGTTTTAGAGATAAAAGGAGAACGAGCTGAGCTTTATGTCCT
AAGGATTAGAGTTACTGCCGTATTGCTAATTATTTCTATTCCGGTTTTAGGAGGGGGTATCACAATAATTC
TGTTTGATCGGAATTTTAACACAACATTTTTTGATCCAGCAGGAGGGGGTGACCCCGTCTTGTTTCAACA
TTTGTTCTGATTTTTTGGGCACCCTGAGGTGTACATTCTTATTCTACCTGCTTTTGGTGTGATATCAAAAG
TAATTATGCATTGTTCCGGGAAGGAGGCAGTTTTCGGGTTAATTGGGATGGTGTATGCAATAATTGGAATT
GGAGGGCTAGGTTGTATGGTGTGGGCTCACCATATATTTACAGTAGGGCTTAATGTTGATACTCGAGGTT
ATTTCTCTACTGCAACTATAGTAATCGCAGTCCCTACAGGGGTAAAAGTATTCAGGTGGTTAGCAACTAT
AGCAGGAAGAAAATTTAAGATAAAGCCTGCTGCTTACTGAAGTACGGGGTTCTTGTTTCTATTCACCGTA
GGAGGCTTAACAGGGGTGTTATTATCAAGGGCTTCTATGGACGTATCGCTTCATGATACTTATTATGTGGT
GGCTCATTTCCACTATGTGCTAAGTATAGGAGCGGTGTTCGGAGTATTTTGTGGCCTGAATCATTGATTGC
CTAACTTTGTTGGAGTATGCTTTAATAAGAAATGGAGAAAAGCCCACTTTATAGCAATGTTTTTTGGTGT
AAATACCACTTTCTTTCCTCAACACTTTCTAGGGCTGAGGGGTATGCCTCGACGGTACATAGATTATGCT
GACATTTATGCTCATTGGCATTGGGTGTCTTCTTATGGGTCTGCAGTGTCTTTTGGCTCTTTGATATATTTT
AAGTTCCTACTCTGAGAAGCTTTAGTGAGTCAACGAGGGATGTCTTTTTATGGGAGTCGATATTTTGGTG
ATATTGTCCATGAATTGGGGAAGGACCTATTTCGGTATCATGGATTTGTGATGATAGTAGCAGTGGCTGTA
TTAGTCTTTGTTATGTATATAGGGTGCGTGATCCTGTTTACTAAATTTCTTACCGTCATTTTTTAAATCGCC
AACGATTGGAGTTTTGATGAACTATTGTGCCGATGTTGATGTTAGTAGGGCTGTGGTTCCCTTCGATGATT
AATTTGTATTATATGGAAGAAGTAAAGCGTCCACGTTGAAATTTCAAAGCAATTGGAAAGCAATGATACT
GATCTTATGAGTGCGATACCTGTTATACAATTGACTCATACATAGAAGACCAACAGGAGACAGGGTACCG
CTTGCTGGATGTTGACAATCGGATGGTTGCTCCTGCAGATGTACAAATAACTGCTTTTGTAAGGAGTTCT
GACGTGCTACATTCGTTTGCGCTTCCTAAATTATTAATCAAAGTAGATGCTATTCCGGGCCGAATCAATCG
GCTTCCGATAAAAGCATCTCAGTGTAGCATTATTTATGGGCAGTGCTCTGAAATTTGTGGAGTAAACCAC
AGGTTTATACCAATTGTGATTGAATTTATTCCTGAGAAATATTTTGTTATATGGTTGGAAGCTCTTAATTAG

ATGAATCGTAATCCTTACTATGTACCAGGCCCAAGTCCGTGGCCATTTTTTGTGGCTATTTCGGCTAACGG
AATAGCGGTAGGGTTAATTTTGTGACTGCATCGAACCCCCTTTTTATTAATAGGTAGTCTGGTTTGCATAT
TATTGAGAACTTTTAGATGGTGACGCGATTTAATTCGTGAAGGAGACATTGGGTTTCACACTCGTTTTGT
AATCAAAAGATTTCGAGATGGCGTTGCCTTGTTTATTCTGTCTGAAGTGATATTTTTCTTCACTTTTTTCT
GGACTTTTTTCCATAATGCTTTAAGGCCCTCGTGTGAGCTAGGAATACGGTGGCCCCCTCCTGGAATTCG
TACGCCAAACCCGTCATCTACTAGTCTGTTTGAGACAGGTCTTCTAATTAGAAGAGGGCTGTTTGTAACT
CAAGCCCATAAGAGGATGCGCTTGGATTACGACGTAGGGCCATTCATCGGCTTAGTGGTGACAATCGTAT
GCGGGACTGTGTTTTTCTTGGTGCAACTGCGGGAATATTATTGAAACTCCTATACTATCGCAGATAGGGT
TTATGGTAGGGTTTTTTACTTACTAACTGGATTCCACGGGATACATGTTGTCGTAGGGACTATTTGACTAA
TGGTAAGGTTAGTTCGACTATGACGCGGGGAGTTTTCTAGTCAACGACATTTCGGGTTTGAGGCTTGTAT
TTGGTACTGACATTTTGTAGATGTGGTATGAGTGGCATTGTGGTGCCTAGTGTATGTGTGGTTTGGAGGA
CCGTGACGAAGTACAAATAAACTGGTAAAGATTATGAATGATAGATTCTATGATTTGCCTTGTCCTGTAA
ACTTAAACGCCTGGTGAAGGTTTGGCTCTATACTAGGCTTGTGTCTGGTTATCCAACTTTTGAGGGGTCT
TTTATTATCAGCCCACTACACTGCTCATGAAGACATGGCATTTGATTCTGTAGTCCACATTATGCGTAATG
TGGAAAAGGGGTGAATATTGCGCAATATCCATGCAAATGGGTCTTCTATGTTCTTTATTTGCATTTATGCT

CACATTGCTCGTGGGTTGTACTATGGGTCTTATTTGGATAAGACCGTGTGGTACTTTGGGGTACACCTGT
TCTTGTTGACTATGGCTGAGGCTTTTCTCGGCTATACTCTGCCTTGGGGGCAGATATCGTACTGGGGGGC
TACTGTGATCACTAATATACTTAGAGTAATCCCTGTGGTAGGTGAAAGGATACTTCGCTACGTATGAGGG
GGGTGGACTGTGTGTAATGCAACGTTAAAGCGGTTTTACACACTTCACTTCCTTTTACCGTTTGTGATAG
TGGCGGTTGTTTTTTTGCACTTATTTTTTTACATGAGAAGGGTAGTAATAACCCTTTAGGAATTGAAAGG
GGCACTATATGTGTGCCTTTTCATCCTTTCTATACTATTAAGGACCTCTTTGGTTATGTTTGCTTTAGGTTC
TTTTTTATGTATTTAGTGTGCGTAGACCCTGAGCTTCTAGGGAATCATCTAAACTACTGGCCTGCTAACCC
TATAAAAACGCCTATTCACGTTCAGCCTGAATGGTACTTTATATTCGCCTATGCAATTCTCCGTTCAATTCC
GCATAAAGCAGGGGGAGTATATGTTATGTTCCTTTCAATCGTAGTGCTATACTTAATTCCTACGCTTCATAC
AGGTAAGTACCGAAGTTTATGCTTCTACCCATTGAATCAAGTAGTGTTTTGGGTGTTGGTTGGAAGGTTT
ATTAGCCTAACATGAATTGGTGCTCGTCCAGTGCGTGAACCTTATATCATTTTGGGGCAGTGTCTTTCAGT
TATTTATTTCTCTAGTTTATTGTTGAACCCCCTTTCTTTATGGGTGTGAGATAAATTACTTGAAGTGGCAGT
AATTGTAAGTATTATCCCTTTCGTAGGTGTCCTTCTTGCTGTAGGGTTTTACACCTTATTGGAGCGTAAGA
TTCTGGCCATCATCATGATCCGAAAAGGCCCGTCCAAGGTAAGTTACATGGGGATCTTGCAGCCTTTTAG
TGATGCAGGTAAGCTATTATGTAAAGAGTTTATCGTGCCCACGCGTGCTAATGTAGGGCCATTTATTCTGG
CTCCTGCACTAATACTAGCTATTAGCTTATTAGGGTGGCTTTTATACCCCTATAAGTCAGCTGAAGTGTTTT
ATGTTTTTGGGGTGATTTTGTTTATAGTTATTACTAGAGTTAGCGTTTATGGGGTAATAATGTCTGGATGGG
CATCTAACTCCAAATACTCCTTACTAGGTGCAGTCCGTGCGATGGCGCAAAGAATTTCTTATGAAATCCC
TATAGGGTTTATTTTTTTTTGTGTAGTGCTGTGTTCAGGCGTGTTTATGTTTCAAGAAATTAGAGTGTTATT
TTTTTTCTTTCCTTTGTGCGTAGTCATACTTGTTTGAATGTTGTGTATACTAGCTGAGACTAATCGGGCAC
CGTTTGACTTTGTGGAAGGAGAGTCGGAGCTAGTGTCGGGTTATAACGTGGAGTACAGAGGAGGGGGT
TTTGCAGTTATATTTATTGCTGAGTACTCTAGAATTCTCCTTAGAAGAGTAATAAGGGCGGCGATATTCTT
CGGAGGAAATGAAGCGTTAGTTGGGTTTTTCATAATGGTTTTCGCAATTTTCTTTGTAGTTGTTCGTGCTT
CTTTACCACGCTTACGTTATGACAAGCTAATAAACTTATGTTGAACTGTCCTTTTATGTGTTATACTTACGG
CTAGTGTGTGTGTAGTAGTCTTAGTGGGGGTGTAATGGTAAGTTTTGTGGTGAGACCTATAAAATTAGTG
AGATTAGGGGTAATATTAATTGGAACAATCCTCAGGGTCAGAAGAGAAGAGCTAGTAGGGGTGTGGTTA
GGTTTGGAGTTAAATCTTTACGGTTTTCTTGTGATTATAAACCCAGATGGGCATTATAGCCCTGAGCCCTG
CGTAAAATACTTTGTTGTACAAAGAACGGGGTCAATTTTGATACTAGTGGGGTTTGTAAGCTTAATACAG
CATGTAGTGAGGGGGTTAGTGATGAGCACAGCGGGTACGGTGCTAAAGTCTGGCGTTTTTCCTCTACAC
TCGTGGGTGCCCTCTATTATTAAAAACAGGAGATGGTTAGCAAGGGGGTTAATACTAACCTGGCAAAAA
GTGGCCCCCCTTGTTTTTTATCAATAATCTTACCTTCTAAAAGGCTTTGAGTAGTGATTGTATCGATAGCT
GGGATTGGGGCAGTGGGAGGTCTCAACCAAAACTCAGTGCGGGTAATAAGAGCCTACTCTTCATTTGTC
CATACATCTTGAATGTTGTTAGGGCTCACATGATCAAGGGTAGTTTTCGTAGGGTACTTTGCAGTCTATAG
GTTATCGGTAGGGCTGTTTTTCTACGGGTGCTCGTTAATAAACAAGATAAGAATAGGTAGCCAGCTAAGT
AGGGCTGCTAGCGGTATGGGGTTGTTAATACTAATAGGGATGCCTCCGTTTCTTGGTTTCTTAGCAAAAG
TATTGGTATTTTTGATGAGGGGAAGGCCTGTAATTGTGGCATGCATCATAGGATCAGTCATTAGATTAAAA
TTTTATATTGATTTCTTTTACAGAATGGTAATAAAAAATAAGGTAGAAGTTAAAGCCATTTGGAGCCTGGT
GATTTGCATAAACATTATGGGGGGGGCGCTGATTTTAGTAAGATTTATTTAGATGGTTATGGGCTTAAGGG
TTGTATTCGTTTGTATCGTGTGTTGTCTGTTTACAGGGTTAATAATATTAAGAGAAAAACGGGGGCTGGA
CCGAGAGAAATGTAGGCCGTATGAATGTGGGTTTGAGCCTATCGGAAGAGCTCGAAGCCCCTTCTCCAT
TCGATTTTTTTAGTGGCAGTTTTGTTTGTGGTGTTCGATGTGGAGGTAGTCCTATTGATACCTTTTGCCT
ACATATTTTTCTATGGCAAAAGAGTGTTGGGTATTTTATCTTCAAGGGGGGTTCCTCCTTATTCTGTTTGTG
GGACTATATCATGAATACCGTGAAGGTTCTTTGGAGTGAGTGGGTTAAATGGTTAAAAGGTTAGCAATTA
GGCTTATTGCATTAATTATTATAAAAAACCTGAATGTGAGTATTATTGGGTTAAGTGTTTTAACAATTTTGA
GTATAGGAGCCGCAAGAGTTGCGACAGGAGGGGTAGAATTAAATGGGCTATACTCGACGGACTTCGTGA
TGGGGTTGATGATCACATTGACATTATTTGTCGCAATTCTTTCTTATTTAAGTAGAGCTAAAGTCTCCCGT
AAAGCAAGATTTAACTTAATGGTTATCAGAATTAGTCTTATTTTAGTGATAAGGTTTAGGGTGAGGAGGT
TTTTTCTTTTTTTTTTTTTTTTCGAAAGTGTGTTAGCGCCTTTGTTACTGTTAATCGTAGGGTGGCGTCTAC
AGGCAGGAGGCTATATAGTGATTTATACTGTATTTGGGTCCCTTTTTTTTCTATGGGGAGTAAGAGAGCTT
TACCTTAGAGGGAGAAGAAGGATGAGAGTGGGAAGATTAGTAAAAAAAAGGGTAATAAGACTGTGATG
GCTATACATTTTAGGGTTTCTAATTAAGTTACCAATATACCCGTTCCATTTGTGGTTACCTAAGGCCCATGT
AGAGGCCCCTGTGGCAGGTTCAATGCTGCTGGCTGGGGTAGTACTAAAATTAGGAGGATACGGCCTACT
TCGGTTTATATTAGTAATACAAGTCAGGCTTAGAAGAGTGTTCGTGTTGCTACTAAGGGTAAACTTGGCA
GGGGGCTTCTACGCCGGACTGGCTTGTGTACGGCAAGTAGACCTAAAATGCTTGGTGGCATACTCGTCC
GTAGCTCACATAAGTCTAGTTCTATTAGTGCTAAGCAACACGCTATTAGGGGTGATAGGGGCTATTATTAT
TATGGTTGGCCACGGGTTATGTTCGTCAGGTTTATTTAGGTACGTAAATGCTATTTATAAGATGAGTCACT
CGCGCTTACTAGTAATAAACAAAGGTGGGTTACTTTTTTGCCCTGCACTAGTGTTGATGTGTTTTCTCCTA
AGATCAAGAAACATAGCAGCCCCACCTAGTCTAAATCTATTCGGGGAAATCCTCGTTTTCGGCGTCGGC
GGGTGAATAAGGGGGGGCCTTTTTATTTATCCTGGGGTTGATGAGCTTTATTAGGGCTTGCTTTAGATTATA
CTTATATGGGAGGTGCTCACACGGAAAAGGGCTTATACATAGGGAGTCGCTGAATTTACTCTGTGATGTG
TTCGTCTTGTTGTCTCATTGGGTGCCGTTAAATTTCTTGTTTATATTTATACCTTAACGTATAAATAAACAC
CTATTGTGTCTGTTTGTAGGGCTTGAAATAATGAGGCTGGGCTTACTATTTGTAACACATGTTTTCTTAAT
AAATCAGTTTTGACTAATTTTATTGATTCTATGTTTAGCTGTTTGTGAAGCTAGCATTTGTTTGGCCCTGCT

GGTCATAGTGATGCGATTATGCGGCGACGACCTAATATCAAGATTGCTAAGAGATGTATACAATCAAAGG
GTAACCTAGGGTTGCTTATATTAATCTTAGGGTATTTATTCATTTTTACAGGAAGGAGGGGAAGAGCGTAC
TTACTTGAAGTTCCTGTTTGAGAGAGCAATTGTCTGTCTTTTAGTTTTAGATTCTTACTAGATAATGTAAG
GATAATTTTTGTTGGGACAGTTTTGGTGATCAGAGGGAGTGTAGCAACCTATTGTAAGTGGTATATAGCT
GAAGAGCTATACTACAACCGGTTTATGGGGTTGGTGTGGTTATTTGTCTTATCTATAGTGTTTATAATCTTA
GTGCCTAATTTAGTAATACTTTTAATTGGATGGGATGGGCTAGGCCTCACTTCTTTTCTATTAGTGGCTTAC
TACCAAAATAACAAAAGACTATCTGCGGCTATGTTGACAGCTTTAACTAATCGAATTGGAGACGTTCTTG
TTCTAATTAGCATTTCTATCTTTCTAAGAGAGGGGGGGTGGTTAATTTATAGGTATCACCCAGTACAAATA
TGGTTATTAGGTTTTATAGTGGTTTTTGCAGGGATAACTAAAAGAGCACAGATGCCTTTTTGCGCATGAC
TGCCAGCTGCCATAGCCGCACCGACACCTGTCTCTTCTTTAGTGCACTCTTCAACACTGGTAACAGCTG
GGGTTTATCTTGTACTTCGTTCGTTCTATATTATCAGTTCTAACGTAACACAAGTTCTAATAATTTTAAGGT
TATTTACTCTGGTACTAGCTGGATCAAGTGCAGTGTTCGCGTTTGATCTAAAAAAAGTAATTGCACTCTC
GACCTTAAGTCAACTAAGGTTGATAATGTTTTCGATTTCAATCCTCCTTCCGTCTGTGGCGTTTTTCCATC
TGGTGACTCATGCAGTGTTTAAAGCTTTGCTGTTTTTGGGTGCAGGGGGTGTTATCCATAGAAACCAAA
GAATTCAGGATATTCGTGGGTTAAGAAGACTGTGACAAGGGTTGCCGGTAAGAATAGGTGCAATAAGAG
TTGCAATTGTATCTTTAAGAGGGGCTCCGTTTATGAGAGGGTTTTTCTCTAAAGACCTCATGATTGAGAT
GATAGGCAGAAGGACTTATGGGTACTTATTAGAGTTATTAGGGTTAATTTTTACTTCTTTTTATAGGGCAC
GAGTATTTAGAGTTATACTCGGGACCAATTATGTTAATAATAGAACCTTGCGTATTAATGAGCATTTAAATA
TACAAACCCCGTTTCTTAGCCTATATATTGGAGCTATTATTTTAGGGGTGGTGCTGGGTAGAAAAATAGAA
AGATTTGGGTTTGTAGTAGTTCTTGAAAACTATGAAAGACTTAGAGTCTTTTTTATTCCTTTTGGGTTACT
GTGACGAGGAGTTATTAGTAAGCTAGGGTCGAGTCCTGCTAAGTTAAGATTTTTTTTGAGGATGTGGTTT
ATAGAATTAACTCATCCTGGGAAGGTGACCTTTTTTAAAGGGTCAAGAACAGTGGCCCAAAGTTTGGAC
CAAGGTTGACTAGAGCTATTGGGGCCTCAAGCTGGATTAGGTCAATTCAGCTGCTTAAATGAGAACTATT
TTACTGTGGTGTGATTAGTAGCCGGGGGCGTTAGCTTAATTATTTTTATATAAATGAGAGTTATAATTATAT
GTGTAAGTTTGATGTCTGTATTCGTGTTGATTGCTAAGCAACCAATCTCTTTAGGGCTAGTACTATTGAGG
GGGTCTATAATTGCATGTGTGGAAATTGCATTGGAGGTTAGAAGTTTGTTAGGGTTTTTGTTATTTTTAAC
TTATGTTAGGGGCGTGATAGTCTTGTTCTTATATGTTCTTAGTATTTATCCTAATGAGCGCTTTAATTTAGA
GTTCGTTATAATTGTGAGAAGGTGTGCTATGATGGGCCTTGTGGGATTAATGAATTATGAGAGTGGGTCA
TTATTTTTAAGGTTTATGGCTGAAGGAAGGCTATATATCCTAATAGCAGGCGTTTTGCTGTTTGTGATGTTA
GTGGTGTCGTATTTGTGCATAAAAACCATAGTGCCTTTGCGAAGAGTA"/>
<sequence id="seq_Mytilus_galloprovincialisF-FJ890" spec="Sequence"
taxon="Mytilus_galloprovincialisF-FJ890" totalcount="4"
value="ATGTTAATAGATGTATTTTCTAGATTTGATGCTCACAGCTACAACTTAATTTGGTTATCTATGTTGT
GGTTGCTTTCGTCTATAGTGCCAATAACTGTGCTATTTAGAGATGTAAGCACTCGAAGCTTGGTACTGTCT
TTTACTTATTCTATAATTCGGAATGGGAAAGGTTTAAAGTTATCTGGGTTTCCTTTGGTGATAAGCGGCTT
GTTTATAATAATTTTGATGTTGAATTTGTCTGGGAACTTCCCCTTTTTTTTCCCTGTAAGAGGTCAGTTTG
TATTTGGATTTTCTTTTGCTTTATCTATTTGGACCTGTTTAGTATTATCTAGCTTACTGTGCAGGTTTGAGC
AGGGTTTGATGAGGCTCGTCCCGACAGGCCCGTTAATCCTTGTGCCTTTTATAGTAGTAGTTGAGCTAAT
TAGCGGTATACTTCGCCCCTTTAACTCTAGTCTTGCGGCTAACGTTGAATTTAGGGGCTGGTAAAGTAATC
TTAACTATATGTAGAAGGGAGTTGGTAGTCGGTTGGTTAATTACAGGAGTTGGGGGTATCAAGGGTTTGC
TAATAGGTGGTGTCTTTGCTGCTGAAGTTGCCATTGCCTGTATTCAGTGTTACATTTTCTGTGTCTTATTGT
GTCTCTATACTGAGGACCATAGGAGTTAGCGATGGTTGTGGTCAACAAATCATAAAGATATTGGCACTCT
TTACCTATATAGTGGGGTCTGAGGAGGTTTGTTCGGGGCAAGTCTAAGCCTGATAATTATACAGGGGCAT
CCTGGAGCAGTATTTTTAAAAGATTGGTTTTATAATGTGGTTGTTACAACACACGCCTTAATAATAATTTT
CTTTGCTGTAATACCTATTCTAATTGGAGCTTTTGGTAATTGGCTTATTCCTCTATTAGTAGGTGGAAAAG
ATATAATCTATCCGCGGATAAATAATTTGAGTTATTGGTTATCTCCTAATGCGCTGTACTTACTTATATTATC
TTTTAGAACGGATAAAGGAGTAGGCGCTGGATGGACTATTTACCCGCCACTGTCTGTATATCCTTATCATA
GCGGGCCGAGGATAGATGTTCTTATTGTGTCTTTGCATTTAGCTGGGTTAAGTTCTTTGGTGGGTGCTATT
AATTTGCTAGTACCAACAAAAACATACCAGTTTTAGAGATAAAAGGAGAACGAGCTGAGCTTTATGTC
CTAAGGATTAGAGTTACTGCCGTATTGCTAATTATTTCTATTCCGGTTTTAGGAGGGGGTATTACAATAATT
CTGTTTGATCGGAATTTTAACACAACATTTTTTGATCCAGCAGGAGGGGGTGACCCTGTCTTGTTTCAAC
ATTTGTTCTGATTTTTTGGGCACCCTGAGGTATACATTCTTATTCTACCTGCTTTTGGTGTGATATCAAAAG
TAATTATGCATTGTTCCGGAAAGGAAGCAGTTTTCGGGTTAATTGGGATGGTGTATGCAATAATTGGAATT
GGAGGACTAGGTTGTATGGTGTGGGCTCACCATATATTTACAGTAGGGCTTAATGTTGATACTCGAGGTT
ATTTCTCTACTGCAACTATAGTAATCGCAGTCCCTACAGGGGTAAAAGTATTCAGGTGGTTAGCAACTAT
AGCAGGAAGAAAGTTTAAGATAAAGCCTGCTGCTTACTGAAGTACGGGGTTCTTGTTTCTATTCACCGT
AGGAGGTTTAACAGGGGTGTTATTATCAAGGGCTTCTATGGACGTATCGCTTCACGATACTTATTATGTGG
TGGCTCATTTTCACTATGTGCTAAGTATAGGAGCGGTGTTCGGAGTATTTTGTGGCCTAAACCATTGATTG
CCTAACTTTGTTGGAGTATGCTTTAATAAGAAATGGAGAAAAGCCCACTTTATAGCAATGTTTTTTGGTG
TAAATACCACTTTCTTCCCTCAACACTTTCTAGGGCTGAGGGGTATACCTCGACGGTACATAGATTATGCT
GACATTTATGCTCATTGGCATTGGGTGTCTTCTTATGGGTCTGCAGTGTCTTTTGGTTCTCTGATATATTTT
AAGTTCTTACTCTGAGAAGCTTTAGTGAGTCAACGAGGGATGTCTTTTTATGGGAGTCGATATTTTGGTG
ATATTGTCCATGAATTGGGGAAGGACCTATTTCGGTATCATGGATTTGTGATGATAGTAGCAGTGGCTGTA

TTAGTCTTTGTTATGTATATAGGGTGCGTGATCCTGTTTACTAAATTTTCTTACCGTCATTTTTTAAATCGCC
AACGATTGGAGTTTTGATGAACTATTGTGCCGATGTTGATGTTAGTAGGGCTGTGGTTCCCTTCGATGATT
AATTTGTATTATATAGAAGAAGTAAAGCGCCCACGTTGAAACTTCAAAGCAATCGGAAAGCAATGATAC
TGATCTTATGAGTGCGATACCTGTTATACAATTGACTCCTACATAGAAGACCAACAGGAGACAGGGTACC
GCTTGCTGGATGTTGACAACCGGATGGTTGCTCCTGCAGATGTACAAATAACTGCTTTTGTAAGGAGTTC
TGACGTGCTACATTCGTTTGCGCTTCCTAAATTATTAATCAAAGTAGATGCTATTCCGGGCCGAATCAATC
GGCTTCCGATAAAAGCATCTCAGTGTAGTATTATTTATGGGCAGTGCTCTGAAATTTGTGGAGTAAACCA
CAGGTTTATACCAATTGTGATTGAATTTATTCCTGAGAAATATTTTGTTATATGGTTGGAAGCTCTTAATTA
G------------------------
ATGAATCGTAATCCTTACTATGTACCAGGCCCAAGTCCGTGGCCATTTTTTGTGGCTATTTCGGCTAATGG
AATAGCGGTAGGGTTAATTTTGTGACTGCATCGAACCCCCTTTGTATTAATAGGTAGTCTGGTTTGCATAT
TATTGAGAACTTTTAGATGGTGACGCGATTTAATTCGTGAAGGAGACATTGGGCTTCACACTCGTTTTGT
GATCAAAAGATTTCGAGATGGCGTTGCCTTGTTTATTCTGTCTGAAGTGATATTTTTCTTCACTTTTTTCT
GGACTTTTTTCCATAATGCTCTAAGGCCTTCGTGTGAGTTAGGAATACGGTGGCCTCCTCCTGGAATTCG
TACACCAAACCCGTCATCTACTAGTCTGTTTGAGACAGGTCTTCTAATTAGAAGAGGGTTGTTTGTAACT
CAAGCCCATAAGAGGATGCGCTTGGATTACGATGTAGGGCCATTCATCGGTTTAGTGGTGACAATCGTAT
GCGGGACCGTGTTTTTTTTGGTACAACTGCGGGAATATTATTGAAACTCCTACACTATTGCAGATAGGGT
TTATGGTAGGGTTTTCTATTTACTAACTGGATTCCATGGGATACATGTTGTCGTAGGGACTATTTGGCTAA
CGGTAAGGTTAGTTCGACTATGACGCGGGGAGTTTTCTAGTCAACGACACTTTGGGTTTGAGGCTTGTAT
TTGGTACTGACATTTTGTAGATGTGGTATGAGTGGCATTGTGGTGCTTAGTGTACGTGTGGTTTGGAGGA
CCGTGACGAAGTACAAATAAACTGGTAAAGATTATGAATGACAGATTCTATGATTTGCCTTGTCCTGTAA
ACTTAAACGCCTGGTGAAGGTTTGGCTCTATACTAGGCTTGTGTCTGGTTATCCAGCTTTTGAGGGGTCT
TTTATTGTCAGCTCACTATACTGCTCATGAAGACATGGCATTTGACTCTGTAGTCCACATTATGCGTAATG
TGGAAAAGGGGTGAATATTACGCAATATCCATGCAAATGGGTCTTCTATGTTCTTTATTTGCATTTATGCT
CACATTGGTCGTGGGCTGTACTATGGGTCTTATTTGGATAAGACCGTGTGGTACTTTGGGGTACACCTGT
TCTTGTTGACTATGGCTGAGGCTTTCCTCGGCTATACTCTGCCTTGGGGACAGATATCGTACTGGGGGGC
TACTGTCATCACTAATATACTTAGAGTAATCCCTGTGGTAGGTGAAAGGATACTTCGCTACGTATGAGGG
GGGTGGACTGTGTGTAATGCAACGTTAAAGCGGTTTTACACACTTCACTTCCTTTTACCGTTTGTAATAG
TGGCGGTTGTTTTTTTGCACTTATTTTTTTTACACGAGAAGGGTAGTAATAACCCTTTAGGAATTGAAAG
GGGTACTATATGTGTGCCTTTTCATCCTTTTTATACTATTAAGGACCTCTTTGGTTATGTTTGCTTTAGGTTC
TTTTTTATGTATTTAGTGTGCGTGGACCCTGAGCTTCTAGGGAACCATCTAAACTACTGGCCTGCTAACCC
TATAAAAACGCCTATTCACGTTCAGCCTGAATGGTATTTTATATTTGCCTATGCAATTCTCCGTTCAATTCC
GCATAAAGCAGGGGGAGTATATGTTATGTTCCTTTCAATCGTAGTGCTATACTTAATTCCTACGCTTCATAC
AGGTAAGTACCGAAGTTTATGCTTCTACCCATTGAATCAAGTAGTGTTTTGGGTGCTGGTTGGAAGGTTT
ATTAGCCTAACATGAATTGGTGCTCGTCCAGTGCGTGAACCTTATATCATTTTGGGGCAGTGTCTTTCAGT
TATTTATTTCTCTAGTTTATTGTTGAACCCCCTTTCTTTATGGGTGTGAGACAAGTTACTTGAAGCGGCAG
TAATTGTAAATATTATCCCTTTTGTAGGTGTCCTTCTTGCTGTGGGGTTTTACACCTTATTGGAGCGTAAG
ATTCTGGCCATCATCATGATCCGAAAAGGCCCGTCCAAGGTAAGTTACATGGGGATCTTGCAGCCTTTTA
GCGATGCAGGTAAGCTATTGTGTAAAGAGTTTATCGTGCCCACGCGTGCTAATGTAGGGCCATTTATTCT
GGCTCCTGCACTAATACTAGCTATTAGCTTATTAGGGTGGCTTTTATACCCCTATAAGTCAGCTGAAGTGT
TTTATGTTTTTGGGGTAATTTTGTTTATAGTTATTACTAGAGTTAGCGTTTATGGGGTAATAATGTCTGGAT
GGGCATCTAACTCCAAATACTCCTTACTAGGTGCAGTCCGTGCGATGGCGCAAAGAATTTCTTATGAAAT
CCCTATAGGGTTTATTTTTTTTTGTGTAGTGCTGTGTTCAGGCGTGTTTATGTTTCAAGAAATTAGAGTGT
TATTTTTTTTCTTTCCTTTGTGCGTAGTTATACTTGTTTGAATGTTGTGTATACTAGCTGAGACTAATCGGG
CACCGTTTGACTTTGTGGAAGGAGAGTCGGAGTTAGTGTCGGGTTACAACGTGGAGTACAGAGGAGGG
GGGTTTGCAGTTATATTTATTGCTGAGTACTCTAGAATTCTCCTTAGAAGAGTAATAAGGGCGGCAATATT
CTTCGGAGGAAATGAAGCGTTAGTTGGGTTTTTCATAATGGTTTTCGCAATTTTCTTTGTAGTTGTTCGTG
CTTCTTTACCACGCTTACGTTATGACAAGCTAATAAACTTATGCTGAACTGTCCTTTTATGTGTTATACTTA
CGGCTAGTGTGTGTGTAGTAGTCCTAGTGGGGGTGTAATGGTAAGTTTTGTGGTGAGACCTATAAAATTA
GTGAGATTAGGGGTAATATTAATTGGAACAATCCTCAGGGTCAGAAGAGAAGAGCTAGTAGGGGTGTGG
TTAGGTTTGGAGCTAAATCTTTATGGTTTTCTTGTGATTATAAACCCAGATGGGCATTATAGCCCTGAGCC
CTGTGTAAAATACTTTGTTGTACAAAGAACGGGGTCAATCTTGATATTAGTGGGGTTTGTAACCTTAATG
CAGCATGTAGTGAGGGGGCTAGTGATGAGCACAGCGGGTACGGTGCTAAAGTCTGGTGTTTTCCCTCTA
CACTCGTGGGTGCCCTCTATTATTAAAAACAGGAGATGGTTAGCAAGGGGGTTAATACTGACCTGGCAA
AAAGTGGCTCCCCTTGTTTTTTTATCAATAATCTTACCTTCTAAAGGGCTTTGAGTAGTCATTGTATCAAT
AGCCGGGATTGGGGCAGTGGGGGGTCTCAACCAGAATTCAGTGCGGGTAATAAGAGCTTACTCCTCATT
TGTGCATACATCTTGAATGTTGTTAGGGCTCACATGATCAAGGGTAGTTTCGTAGGGTATTTTGTAGTCT
ATAGGTTATCGGTAGGGCTGTTTTTCTACGGGTGCTCGTCAATAAACAAGATAAGAATAGGTAGCCAGCT
AAGTAGGGCTGCTAGCGGTATGGGGTTGTTAATACTAATAGGGATGCCCCCGTTTCTTGGGTTCTTAGCA
AAAGTATTGGTATTTTTGATGAGGGGAAGGCCTGTGATTGTGGCATGCATCATAGGATCAGTCATTAGAT
TAAAATTTTATATTGATTTCTTTTACAGAATGGTAATAAAAAATAAGGTAGAAGTTAAAACCATTTGGAGT
CTGGTGATTTGCATAAACATTATGGGGGGGGCGCTGATTTAGTAAGATTTATTTAGATGGTTATGGGCTT
AAGGGTTGTATTCGTTTGTATCGTGTGCTGTCTGTTTACAGGGTTAATAATATTAAGAGAAAAACGGGGA

TTGGACCGAGAGAAATGTAGGCCGTATGAATGTGGGTTTGAGCCTATCGGAAGAGCTCGAAGGCCTTTC
TCCATTCGATTTTTTTTAGTGGCAGTTTTGTTTGTGGTGTTTGATGTGGAGGTAGTCTTATTAATACCTTTT
GCCTACATATTTTTCTATGGCAAAAGAGTGTTGGGTATTTTATCCTCAAGGGGGTTCCTCCTTATTTGTTT
GTGGGATTATATCATGAATACCGTGAAGGTTCTTTGGAGTGAGTCGGTTAAATGGTTAAAAGGTTAGCAA
TTAGGCTTATTGCATTAATTATTATAAAAAACCTGAATGTGAGTATTATTGGGTTAAGTGTTTTAACAATTT
TGAGTATAGGGGCCGCAAGAGTTGCGACAGGAGGGGTAGAATTAAATGGGTTATACTCCACGGATTTCG
TGATGGGGTTGATGATCACATTGACATTATTTGTCGCAATTCTCTCTTATTTAAGTAGAGCTAAAGTCTCC
CGGAAAGCAAGATTTAACTTAATGGTTATCAGAATTAGTCTTATCTTAGTGATAAGGTTCAGGGTGAGGA
GGTTTTTTCTTTTTTTTTTTTTTTTTGAAAGTGTGTTAGCGCCTCTGTTACTGTTAATCGTAGGTTGGCGTC
TACAGGCAGGAGGTTATATAGTGATTTATACTGTATTCGGGTCCCTTTTCTTTCTATGGGGAGTAAGAGAG
CTTTATCTTAGAGGGAGAAGAAGGATGAGAGTGGGAAGATTAGTAAAAAAAAGGGCAATAAGACTGTG
ATGGCTATACATTTTAGGGTTTCTTATTAAGTTACCAATATACCCGTTCCATCTGTGGTTACCTAAGGCCCA
TGTAGAGGCCCCCGTGGCAGGTTCAATGCTGTTGGCTGGGGTAGTACTAAAATTAGGAGGATATGGCCT
ACTTCGGTTTATAGCAGTAATACAAGTTAGGCTCAGAAGAGTGTTCGTGTTGCTACTAAGGGTAAACCTG
GCAGGGGGCTTCTACGCCGGACTGGCCTGTGTACGGCAAGTAGACCTAAAATGCTTGGTGGCATATTCG
TCTGTAGCTCATATAAGTCTAGTTCTATTAGTCCTAAGCAACACGCTATTAGGGGTAATAGGGGCTATCAT
TATTATGGTCGGCCACGGGTTATGTTCGTCAGGCTTATTTAGGTACGTAAATGCTATCTATAAGATGAGGC
ACTCGCGCTTACTAGTAATAAACAAAGGTGGGTTACTTTTTTGCCCTGCATTAGTGTTGATGTGTTTCCTT
CTAAGATCAAGAAATATAGCAGCTCCACCTAGTCTAAATCTGTTTGGGGAAATCCTCGTTTTCGGCGTCG
GTGGGTGAATAAGGGGGGCCTTCTTATTTATCCTGGGGTTGATGAGCTTTATTAGGGCTTGCTTTAGATTA
TACTTATACGGGAGGTGCTCACATGGAAAAGGGCTTATACATAGGGAGTCGCTGAATTTACTCTGCGATG
CGTTTGTCTTGTTGTCTCATTGGGTGCCGCTAAATTTCTTGTTTATATTTATACCTTAACGTATAAATAAAC
ACCTATTGTGTCTGTTTGTAGGGCTTGAGATAATGAGGCTGGGTTTACTATTTGTAACACATGTTTTCTTA
ATAAATCAGTTTTGACTAATTTTATTGATTCTATGTTTAGCTGTTTGTGAAGCTAGTATTTGTTTGGCCTTG
CTGGTCATAGTGATGCGATTATGCGGCGACGACCTAATATCAAGACTGCTAAGAGATGTATACAATCAAA
GGGTAACCTAGGGTTGCTTATATTAATCTTAGGGTATTTATTCATTTTCACAGGAAGGAGGGGAAGAGCG
TATTTACTTGAAGTTCCTGTTTGAGAGAGCAATTGTCTGTCTTTTAGTTTTAGATTCTTACTAGATAATGTA
AGGATAATTTTTGTTGGGACAGTTTTGGTGATCAGAGGGAGTGTAGCAACCTATTGTAAGTGGTATATAG
CTGAAGAAATATACTACAACCGGTTTATGGGGTTGGTGTGGTTATTTGTTTTATCTATAGTGTTCATAATCT
TAGTGCCTAATCTAGTAATACTTTTAATTGGATGGGATGGGCTAGGCCTCACTTCTTTTCTGTTAGTGGCT
TACTACCAAAATAACAAAAGACTATCTGCGGCTATGCTGACAGCTTTAACTAATCGAATTGGAGACGTCC
TTGTTCTAATTAGTATTTCTATCTTTCTAAGAGAGGGGGGGTGGTTAATTTATAGGTATCACCCAGTACAA
ATATGGTTATTAGGTTTTATAGTAGTTTTTGCAGGGATAACTAAAAGAGCACAGATGCCTTTTTGCGCATG
ACTGCCAGCTGCCATAGCCGCACCGACACCTGTCTCTTCTTTAGTGCACTCTTCAACATTGGTAACAGCT
GGGGTTTATTTGGTACTTCGTTCATTTTATATTATCAGTTCTAACGTAACACAAGTTCTAATAATTTTAAGG
TTATTTACTCTGGTACTAGCTGGATCAAGTGCAGTGTTCGCGTTTGATCTAAAAAAAGTAATTGCACTCT
CGACTTTAAGTCAACTAAGGTTGATAATGTTTTCGATTTCAATCCTCCTTCCGTCTGTGGCGTTTTTTCAT
CTGGTGACTCATGCAGTGTTTAAAGCTTTGCTGTTTTTGGGTGCAGGGGGGTGTTATCCATAGAAACCAA
AGAATTCAGGATATTCGTGGGTTAAGAAGGCTGTGACAAGGGTTGCCGGTAAGAATAGGTGCAATAAGA
GTTGCGATTGTATCTTTAAGAGGGGCTCCGTTTATGAGAGGGTTTTTCTCTAAAGACCTCATGATTGAGA
TGATAGACAGAAGAACTTATGGGTACTTATTAGAGTTACTAGGGTTAATTTTTACTTCTTTTTACAGGGCA
CGAGTATTTAGAGTTATACTCGGGTCCAATTATGTTAATAATAGAACCTTGCGTATTAATGAGCATTTAAAT
ATACAAACCCCGTTTCTTAGCCTGTATATTGGAGCTATTATTTTAGGGGTGGTGCTGGGTAGAAAAATGG
AGAGATTCGGGTTTGTAGTAGTTCTTGAAAGCTACGAAAGACTTAGAGTCTTTTTTATTCCTTTTGGGTT
ACTGTGACGAGGAGTTATTAGTAAGCTAGGGTCGAGTCCTGCTAAGTTAAGATTTTTTTTGAGGATGTGG
TTTATAGAATTAACTCATCCTGGGAAGGTGGCCTTTTTTAAAGGGTCAAGAACAGTGGCCCAAAGTTTG
GACCAAGGTTGACTAGAGCTATTGGGGCCTCAAGCTGGATTAGGTCAATTCAGTTGTTTAAATGAGAAC
TATTTTACTGTGGTGTGATTAGTAGCCGGGGGCGTTAGCTTAATTATTTTTATATAAATGAGAGTTATAATT
ATATGTGTAAGTTTGATGTCTGTATTCGTGTTGATTGCTAAGCAGCCAATCTCTTTAGGGCTAGTATTATTG
AGGGGGTCTATAATTGCATGTGTGGAAATTGCATTGGAGGTTAGAAGTTTGTTAGGGTTTTTGTTATTTTT
AACTTATGTTAGGGGTGTGATAGTCCTGTTTTTATATGTTCTTAGGATTTATCCTAATGAGCGCTTTAATTT
AGAGTTTGTTATAATTGTGAGAAGGTGCGCTATGATGGGCTTTGTGGGATTAATAAATTATGAGAGTGGG
TCATTATTTTTAAGGTTTATGGCTGAAGGAAGGCTATATATCTTAATAGCAGGCGTTTTGCTGTTTGTGATG
TTAGTGGTGTCGTACTTGTGTATAAAAACCATAGTGCCTTTGCGAAGAGTA"/>
<sequence id="seq_Mytilus_trossulusF-GU936625" spec="Sequence" taxon="Mytilus_trossulusF-GU936625" totalcount="4"
value="ATGTTAATAGATGTTTTTTCTAGATTTGATGCTCACAGCTATAACTTAATTTGGTTGTCTATGTTATG
GCTGCTGTCTTCTATAGTGCCAATAACCGTGCTATTTAGAGACGTGAGTACGCGGAGTTTGGTGCTATCC
TTTACTTATTCGATGATTCGAAACGGAAAGGGACTAAAGCTATCTGGGTTTCCTCTAGTAATAAGGGGTC
TGTTCATGATAATTCTGATACTAAATCTGTCTGGAAACTTTCCATTCTTTTTCCCTGTAAGAGGGCAGTTT
GTGTTCGGGTTCTCCTTTGCTTTGTCTATTTGAACTTGTTTAGTTTTATCTAGTCTTTTATGCAGATTTGAG
CAGGGGTTGATGAGTCTCGTTCCAACAGGTCCGTTAATCCTTGTGCCTTTTATAGTAGTGGTTGAGCTAA
TTAGTGGCATACTTCACCCTTTAACATTAGTTTTACGTCTGACACTAAATCTGGGAGCTGGTAAAGTAATT

TTAACTATATGCAGGAGAGAGTTAGTAGTTAGCTGGTTACTTACAGGAGTTGGGGGTATTAAAGGGTTAA
TAATGGGCGGTGTTTTTGCCGCTGAAGTTGCAATCGCGTGTATTCAGTGTTATATTTTTTGTGTCTTATTGT
GTCTCTATACGGAGGATCATAGAAGGTAGCGATGGCTGTGATCAACAAATCACAAAGATATTGGAACCCT
TTATCTGTATAGCGGAGTCTGAGGAGGGTTGTTTGGAGCAAGGTTGAGGTTAATGATCATGCAAGGTCAT
CCTGGAGCAGTGTTCTTAAAAGATTGATTCTATAATGTGGTGGTTACAACGCATGCCTTAATAATAATTTT
TTTTGCTGTGATACCTATCTTAATTGGAGCTTTCGGTAATTGGTTGATTCCTCTGCTAGTAGGAGGTAAAG
ATATAATTTACCCGCGAATAAATAACTTAAGTTATTGACTATCTCCTAATGCACTATACTTACTAATACTGTC
CTTTAGAACGGATAAAGGAGTTGGTGCTGGATGAACTATTTACCCCCCTTTATCTGTGTACCCCTATCATA
GGGGCCCTAGGATAGATGTTCTTATTGTGTCACTACATCTAGCTGGGCTTAGCTCTCTAGTGGGGGCTATT
AACTTTGCTAGGACCAATAAAAATATGCCAGTGTTAGAAATGAAAGGAGAACGAGCGGAGCTTTATGTT
TTAAGGATTAGAGTTACTGCAGTTCTTTTAATTATTTCAATTCCGGTTTTAGGAGGGGGTATCACAATAAT
CTTGTTTGACCGAAACTTTAACACAACTTTTTTCGATCCCGCAGGAGGGGGGGACCCCGTTTTGTTCCA
ACATTTGTTTTGATTTTTTGGGCATCCGGAAGTGTATATTCTTATTCTACCTGCCTTTGGTGTGATATCAAA
AGTAATTATGCATTGCTCTGGAAAAGAAGCGGTTTTTGGTCTAATTGGGATAGTATACGCAATAATCGGA
ATTGGAGGGTTAGGGTGTATGGTGTGGGCTCACCACATGTTTACCGTAGGTCTTAATGTTGATACTCGAG
GCTATTTTTCTACTGCAACTATAGTAATCGCTGTTCCTACAGGGGTGAAAGTATTCAGATGATTGGCAACG
ATAGCAGGAAGAAAATTCAAAATAAAGCCTGCCGCCTACTGAAGTACTGGGTTTCTGTTTTTATTCACCG
TGGGAGGGCTAACAGGGGTCTTACTGTCTAGGGCTTCTATGGATGTGTCTCTACACGACACATATTATGT
GGTGGCTCATTTCCATTATGTGCTAAGAATAGGGGCGGTGTTTGGGGTGTTCTGTGGTCTTAACCATTGG
TTGCCAAATTTTGTTGGAGTATGTTTTAATAAGAAATGGAGGAAAGCCCATTTTATAGCAATATTTTTTGG
GGTAAATACTACCTTCTTCCCTCAGCATTTCTTAGGCCTAAGAGGAATGCCTCGACGGTATATAGACTAC
GCTGATATTTATGCTCACTGACATTGGGTGTCTTCTTATGGGTCCGCTGTGTCTTTTGGGTCTCTAATATAT
TTTAAGTTCCTTCTATGAGAGGCTCTAGTAAGCCAGCGAGGGATGTCTTTTTACGGGAGTCGATATTTTG
GTGATATTGTCCATGAACTAGGGAAAGACCTGTTCCGGTACCATGGTTTTGTGATGATAGTAGCAGTGGC
TGTGTTGGTCTTTGTTATGTATATAGGGTGTGTAATCCTTCTTACTAAATTTTCTTATCGCCATTTCTTGAA
CCGTCAACGATTAGAATTTTGATGGACTATTGTGCCAATGTTGATGTTAGTAGGGTTGTGGTTTCCTTCTA
TAATTAACCTATATTATATAGAAGAAGTAAAACGGCCCCGGTGAAACTTTAAGGCGATTGGGAAACAATG
GTACTGATCTTACGAATGTGACACTTGTTATACAATTGATTCTTACATAGAAGACCAGCAGGAGACAGGG
TATCGTTTGTTGGATGTTGATAACCGGATGGTGGCTCCAGCAGATGTGCAAATAACTGCTTTTGTAAGAA
GGTCTGATGTGCTCCATTCGTTTGCACTGCCTAAGTTACTAATTAAAGTAGACGCCATCCCAGGTCGAAT
TAATCGGCTTCCTATAAAAGCTTCCCAGTGTAGAATTATTTACGGGCAGTGTTCTGAAATTTGCGGGGTT
AACCATAGATTTATACCGATTGTGATTGAGTTTATTCCTGAGAAATATTTTGTCATATGGTTGGAAGCTCTT
AACTAA------------------------
ATGAATCGTAATCCTTACTATGTACCAGGTCCAAGTCCGTGGCCCTTTTTTGTGGCTATCTCGGCAAACG
GAATAGCGGTAGGGTTAATTTTGTGACTGCATCGAACTCCCTTTCTATTAATAGGAAGGTTGGGGTGTAT
ACTATTGAGAACTTTTAGATGATGGCGAGACTTAATTCGTGAGGGAGATATTGGGTTTCATACTCGCTTC
GTAATCAAGAGATTTCGTGATGGAGTTGCCCTTTTTATTCTGTCTGAAGTAATGTTCTTCTTTTCTTTTTTT
TGGACTTTCTTCCATAATGCCCTAAGACCCTCGTGTGAACTAGGGATGCGATGACCCCCTCCAGGGATCC
GCACGCCAAACCCGTCGTCGACAAGGCTGTTCGAGACAGGTCTTTTAATTAGGAGGGGGTTATTCGTAA
CTCAAGCCCATAAGAGAATGCGTTTGGATTATGATGTTGGGCCATTTATTGGCCTAGTGGTAACAATTTTA
TGTGGGACTGTGTTCTTCCTAGTGCAACTTCGAGAATACTACTGAAACTCGTACACTATTGCAGATAGGG
TGTATGGAAGAGTGTTTTATTTACTAACTGGGTTTCATGGAATGCACGTAGTCGTGGGGACTTTTTGACT
AATGGTGAGGTTAGTTCGACTATGGCGTGGGGAGTTTTCCAGTCAACGGCACTTTGGTTTTGAGGCTTG
CATTTGGTACTGACACTTCGTAGATGTGGTATGGGTAGCATTATGATGTCTAGTATATGTGTGGTTTGGAG
GACCGTGACGAAGTACTAATAAGCTGGTGAAGATTATGAATGACAGGTTCTATGATTTGCCTTGTCCTGT
AAACTTAAACGCTTGGTGAAGGTTTGGCTCTATACTAGGCTTGTGCCTGATTATCCAACTTCTAAGGGGT
CTTTTATTGTCAACTCATTATACTGCTCATGAAGACATGGCATTCGATTCTGTAGTACATATTATGCGTAAT
GTGGAAAAAGGATGAATGTTGCGTAATATTCATGCAAATGGGTCCTCTATGTTTTTTATCTGTATTTATGC
GCACATTGCTCGTGGGCTGTATTATGGGTCTTATTTAGATAAGACAGTGTGGTATTTTGGGGTGCATTTGT
TTTTGTTAACTATGGCGGAGGCTTTCCTCGGTTACACTTTGCCTTGGGGGCAAATATCATATTGGGGGGC
TACTGTTATTACTAATATACTTAGAGTGATCCCCGTAGTAGGAGAGAGTATGCTCCGCTATGTATGAGGGG
GTTGGACCGTGTGTAATGCAACTCTAAAGCGGTTTTATACTTTACACTTTCTCTTACCGTTTGTGATAGTG
GCGGTTGTTTTTTTACACCTGTTTTTTTTACATGAGAAAGGGAGTAATAACCCTTTGGGTATTGAAAGAG
GTACTATGTGTGTGCCCTTCCACCCCTTCTATACTATCAAAGATCTTTTTGGTTATGTTTGCTTTAGGTTCT
TTTTTATATATTTAGTGTGTGTGGATCCTGAGCTGTTAGGGAATCATTTAAACTATTGGCCTGCTAATCCTA
TAAAAACGCCAATCCATGTTCAGCCTGAGTGGTATTTTATGTTTGCTTATGCAATCCTTCGTTCAATTCCT
CATAAAGCGGGGGGGGTATATGTTATGTTTTTGTCGATTGTAGTATTATACCTAATTCCTAGTCTTCACAGA
GGTAAGTATCGAAGTTTATGTTTTTACCCGTTTAATCAAGTAGTGTTTTGAGTGTTGGTTGGTAGGTTTAT
TAGGTTAACATGGATTGGTGCTCGCCCAGTGCGGGAGCCTTATATCATTTTGGGGCAGTGTCTTTCAGTC
ATTTATTTCTCTAGGTTGTTATTAAACCCCCTTTCTTTGTGGGTGTGGGACAAGCTGCTTGAAGTAGGCGT
GGTTGTTGGTGTTATCCCTTTTGTAGGGGTGCTTCTCGCTGTGGGCTTCTATACTTTGTTGGAACGTAAA
ATTTTGGCTATCATTATAATCCGAAAGGGTCCATCCAAGGTGAGTTATATAGGGATCTTGCAGCCTTTTAG
TGATGCAGGTAAGTTGTTGTGTAAAGAGTTTATTGTGCCTACACGTGCTAACGTAGGGCCCTTCATTTTG

GCTCCTGCACTAATATTAACTATCAGTTTACTTGGATGGCTTTTATACCCGTATAAGTCGGCTGAAGTGTT
TTATGTTTTCGGGGTGATTCTGTTTATAGTTATTACTAGAGTCAGGGTTTACGGGGTAATGATATCCGGATG
GGCTTCTAACTCTAAATACTCTTTGCTAGGTGCAGTTCGTGCGATGGCGCAAAGAATTTCTTATGAGATC
CCTATAGGATTTATCTTCTTTTGTGTGGTGCTGTGCTCGGGTGTGTTTATGTTTCAAGAAATTAGGGTGTC
CTTTTTTTTCTTTCCTTTGTGCGTAGTTATAGTTGTCTGAATGCTGTGTATGCTAGCTGAAACTAATCGGG
CGCCATTTGATTTTGTGGAAGGAGAGTCGGAATTAGTGTCAGGATACAACGTGGAGTACAGCGGAGGG
GGGTTTGCAGTTATATTTATTGCGGAGTACTCTAGTATTCTTCTCAGAAGGGTTATAAGGGCGGCGATATT
TTTCGGGGGAAATGAAGCGTTGATCGGGGTCTTTATGATGGCTTTTGCGGTCTTCTTTGTGGTTATTCGT
GCTTCTTTACCTCGTTTACGTTATGATAAGTTAATGAGTTTGTGTTGGACTGTTCTTCTATGTGTCATACTT
ATGGCTAGTGTGTGTGTAGTAGTTCTAGTTAGGGTGTAATGGTAAGCTTTGTGGTAAGACCTATAAAATT
AGTGAGATTAGGGGTAATATTGATCGGGACAATTCTTAGGGTTAGAAGAGAAGAGATAGTAGGGGTGTG
ACTCGGTCTAGAGCTAAATCTGTATGGATTTCTTGTAATTATAAACCCTGATGGGCACTATAGTCCTGAGC
CCTGTGTAAAATATTTTGTGGTACAAAGAACGGGGTCAATTCTGATACTAGTGGGTTTTGTAACCTTGAT
ACAGCACGTAGTGAGAGGGCTGGTGATAAGGAGGGCGGGTACAGTGCTAAAATCTGGCGTTTTCCCGC
TACATTCGTGGGTCCCTTCAATTATTAAGAACAGCAGATGGTTAGCAAGAGGGTTAATATTAACTTGGCA
AAAAGTAGCCCCCCTTGTCTTTTTATCAATAATTATACCCTCTAAGGGGTTGTGAGTAGTAATTGTATTGA
TAGCTGGAATTGGGGCAGTAGGGGGCCTTAACCAGAACTCAGTACGAGTAATAAGTGTGTACTCGTCGT
TTGTGCATACATCATGAATGCTGTTAGGGCTCACATGGTCAAGAGTAGTCTTTGTAGGGTATTTTGCAGTT
TATTCGCTGTCGGTAGGGCTGTTTTTTTATGGGTGCTCAATAATAAACAAAACAAGAATGGGCGGTCAGA
TTAGTAGAGCCGCGAGGGGTATAGGGTTACTGATACTGATGGGGATGCCTCCTTTCCTTGGCTTTCTAGC
GAAAGTATTGGTGTTTCTAATGAGAGGAAGGGCTGTAATTGTGGCTTGTATTATAGGTTCAGTAATCAGG
CTAAAATTCTACATTGACTTTTTTTATAGGATAGTAATAAAAAAACAAAGCAGAATTCAAGATTATGTGGA
GGATAGTGATCGGGGCTAATCTAGCAGGGGGGGCATTGATCTTGGTGAGATTTATTTAGATGGTTATGGG
GTTAAGAGTTGCGTTTGTCTGCATTGTGTCTTTTTTGTTTACGGGGTTAATGCTACTAAGGGAAAAGCGG
GGCCTAGACCGAGAAAAGTGCAGTCCATATGAGTGTGGATTTGAGCCTATTGGAAGAGCTCGGAGGCC
CTTTTCTATCCGATTCTTTCTAGTAGCAGTTTTGTTCGTCGTGTTTGATGTAGAGGTAGTGCTGTTAATACC
TTTTGCCTACATGTTCTTTTACGGTAAGAGAGTGTTAGGGATTTTGTCCTCAAGGGGTTTCCTTCTTATCT
TGTTTGGGGGTCTCTATCACGAATATCGTGAGGGGTCTTTGGAATGAGTAGGTTAAATGGTTAAAAGGTT
GGCAATTAGACTTATTGCATTGATTATTATAAAAAACCCAAATATGAGTGTCATTGGGTTAAGCGTTCTAA
CTATTCTAAGTATGGGCGCCACAAGAGTCGCGATAGGGGGGGTAGAGTTGAACGGGTTGTACACCACAG
ACTTTGTAATAGGGTTAATGGTTACACTAACTCTATTTGTAGCAATTCTTTCCTACCTAAGGAGGGTTAAG
ATCCACCGGAAAGCAAGATTTAATTTAATAATTATCAGAATCAGCCTAATTTTAGTGATAAGATTCAGGGT
GAGGAGGTTCTTTCTTTTTTTTTTTTTTTTTGAAAGTGTGCTAGCCCCTCTGTTGTTATTAATTGTAGGCT
GACGTTTACAGGCAGGGGGGTACATAGTAATCTATACGGTGTTCGGATCTCTTTTTTTCTTATGGGGGGTA
AGAGAACTCTATATTAGAGGGAGGAGGAGGATAAGTGTGGTAAGGCTAGTAAAAAAAAGGGGAATGAG
ATTGTGATGGCTATACATTCTAGGGTTTCTTATCAAGTTACCAATATATCCATTTCACCTGTGACTACCTAA
GGCTCACGTAGAGGCCCCAGTAGCCGGTTCAATGCTATTGGCCGGGGTGGTACTAAAATTAGGAGGGTA
CGGGCTGCTTCGATTTATAATAGTTATACAAATAAGGCTTAGAAGAGTGTTTGTGCTGCTACTAGTGGTG
AACTTGGCAGGAGGTGTCTATGCAGGATTAGCGTGTGTACGGCAAGTGGACCTAAAATGTTTGGTAGCA
TATTCCTCCGTAGCGCATATGAGGCTTGTGCTATTAGTGCTCAGGAACACGGTATTAGGGGTAGTGGGGG
CCATTATCATTATGATCGGGCATGGGTTGTGTTCATCAGGTTTGTTCAGGTATGTGAATGCTATCTATAAGA
TGAGGCACTCGCGCCTGCTAGTAATAAATAAAGGGGGCTTGTTAGTCTGCCCAAGTCTAGTCTTAATGTG
TTTCCTGTTAAGATCAAGCAACATAGCAGCCCCTCCTAGTTTAAACTTACTTGGGGAAATCCTCGTTTTT
GGCGTGGGAGGGTGAATAAGCGGAGTGTTCCTGCTTATCTTGGGTCTGATAAGCTTTATTAGGGCGTGTT
TTAGATTATACCTATATGGAAGTTGTTGTCACGGGAAGGGGGTGTCACACAGGGAGTCCTTAAACTTGGT
TTGTGATGTTTTTGTTCTGGCGGCTCATTGGATACCGCTGAACTTTATGTTTATGTTTATACCCTAACGTAT
AAACAAACACCTTTTGTGTTTATTTGTAGGGCTTGAAATAATAAGACTAGGCTTGTTGTTTGTAACCCAT
GTGTTTCTAATAAATCAGTTTTGGTTAATTTTACTAATTCTATGTTTAGCTGTTTGCGAAGCCAGAATTTGC
TTGGCCCTTCTGGTTATGGTGATGCGACTATGCGGAGACGATTTGATGTCAAGGTTACTAAGAGATGTAT
ACAATTAAAAAGTAACTTAGGGTTACTTTTACTGATTCTGGGATACTTATTTATTCTTAGAGGAAGGGCCG
GAAAAGCTTATTTATTGGAAGTTCCTGTTTGAGAGAGTAATTGTCTCTCATTTAGCTTCAGAGTTCTACTA
GATAGCGTGAGAATAGTTTTTGTTGGGACGGTTTTGGTAATTAGAGGAAGTGTAGCAACCTACTGCAAG
TGGTATATAGCTGGAGAGCCATACTACAAGCGGTTTATGGGATTAGTATGGTTGTTTGTGCTGTCTATAGT
GTTTATAATCTTAGTTCCTAATTTAGTAATACTTTTAATTGGTTGAGACGGGCTAGGGCTCACCTCATTCCT
ATTAGTGGCTTATTACCAGAACAATAAGAGGCTATCTGCGGCTATGTTGACAGCTTTGACTAATCGAATT
GGGGATGTTCTTGTACTCCTTAGAGTTTCTATTTTTTTAAGAGAAGGGGGGTGGTTAATTTATATATACCA
CCCAGTGCAGACATGGGTTTTAGGGTTTGTGGTAGTTCTTGCAGGTATAACTAAAAGGGCACAAATGCC
GTTTTGCGCATGGCTACCTGCTGCCATGGCGGCACCCACACCGGTCTCCTCTTTGGTGCATTCTTCGACA
TTGGTGACAGCTGGGGTTTATTTGATTCTTCGCTCTTTTTATATTATCAGAGCTAATGTGACTCAAATACTT
ATAGTCTTAAGACTATTTACTCTAATATTAGCGGGGTCAAGGGCTGTGTTTGCGTTTGACCTAAAAAAGG
TAATCGCACTCTCGACTTTGAGGCAGTTAAGGTTAATGATATTCTCGATTTCAATCCTTCTTCCGTCTGTA
GCTTTTTTTCATTTAGTAACCCATGCGGTATTTAAAGCTTTGTTGTTTCTAGGCGCAGGGGGTGTTATTCA
TAGAAACCAAAGAATCCAAGATATCCGGGGGTTAAGAAGCTTGTGGCAAGGATTACCGGTAAGAATGG

GTGCAATAACGGTTGCAATTGTGTCCTTGAGAGGGGCCCCGTTTATAAGAGGGTTTTTCTCTAAAGACCT
GATAATTGAGATGATAGACAGAAGAACTTATGGGTATTTATTAGAGCTAACAGGTTTAATCTTCACTTCTT
TTTATAGGGCACGGGTATTTAGAGTGATACTTGGGTCTAATTACGTTAATAGCAGAAGTTTGCGGATTAAT
GAGCACTTAAATATACAAACTCCTTTTCTTAGCCTGTATATTGGGGCTATTATCTTAGGAGGGGTATTAGG
GAGGAAAATAGAAAGGTTTGGGTTTGTAGTAGTTCTTGAGAAATATGAGAGAGTCAGAGTATTTCTTATT
CCCTTTGGGTTATTGTGATGAGGAGTGCTTAGAAAATTAGGGTCTAAGCCTGCCAAATTAAGATTCTTTT
TGAGAATGTGGCTCATAGAGCTAACCCACCCCGGAAAAATGGCCTTCTTTAAGGGGTCCAGGACGGTAG
CTCAAAGTCTGGATCAAGGTTGACTAGAGCTATTGGGTCCTCAAAAGGGACTAGGTCAATTCAGCTGTT
TGAATGAAAATTATTTTACCGTAGTATGAGTGGTAGCGGGGGGCGTAAGCTTAATTATGTTTATATAAATG
AGAGTTATAGTCATATGTGTAATTTTGATGGCTGTGTTTGTCTTAATTGCCAAGCAACCTATTTCTTTAGG
GCTAGTGCTATTGAGGGGGTCTATAATTGCATGTGTGGAGGTTGCACTGGAGGTTAGGAGGTTGTTAGG
GTTCTTATTGTTTCTAACTTACGTTAGGGGTGTTATAGTCCTGTTCTTGTATGTTTTAAGGATCTACCCCAA
TGAACGTTTTAATCTAGAGTTTATGGTTCTCGTCAGAAGGTGTGCCAGAGCGGTCCTTATGGGTCTTATG
AATTACGAGAGCGGGTCTTTGTTTCTAAGTTTCATGGCTGAAAGAAGCTTATATATCCTAATAGCTGGTGT
GTTACTGTTTGTAATATTAGTGGTGTCGTATTTGTGCATAAAAACCATGGTGCCTTTACGAAGAGTA"/>
<sequence id="seq_Mytilus_trossulusF-HM462080" spec="Sequence"
taxon="Mytilus_trossulusF-HM462080" totalcount="4"
value="ATGTTAATAGATGTTTTTCTAGATTTGATGCTCACAGCTATAACTTAATTTGGTTGTCTATGTTATG
GTTGCTGTCTTCTATAGTGCCAATAACCGTGCTATTTAGAGATGTGAGTACGCGGAGTTTGGTGCTATCCT
TTACTTATTCGATGATTCGAAACGGAAAGGGACTAAAGCTATCTGGGTTTCCTCTAGTAATAAGGGGTCT
GTTCATGATAATTCTGATACTAAATCTGTCTGGAAACTTTCCATTCTTTTTCCCTGTAAGAGGGCAGTTTG
TGTTCGGGTTCTCCTTTGCTTTGTCTATTTGAACTTGTTTAGTTTTATCTAGTCTTTTATGCAGATTTGAGC
AGGGGTTGATGAGTCTCGTTCCAACAGGTCCGTTAATCCTTGTGCCTTTTATAGTAGTGGTTGAGCTAAT
TAGTGGCATACTTCGCCCTTTAACATTAGTTTTACGTCTGACACTAAATCTGGGAGCTGGTAAAGTAATTC
TAACTATATGCAGGAGAGAGTTAGTAGTTAGCTGGTTACTTACAGGAGTTGGGGGTATTAAAGGGTTGAT
AATGGGCGGTGTTTTTGCCGCTGAAGTTGCAATCGCGTGTATTCAGTGTTATATTTTTTGTGTCTTATTGT
GTCTCTATACGGAGGATCATAGAAGGTAGCGATGGCTGTGATCAACAAATCACAAAGATATCGGAACCC
TTTATCTGTATAGCGGAGTCTGAGGAGGGTTGTTTGGAGCAAGGTTGAGGTTAATGATCATGCAAGGTC
ATCCTGGAGCAGTGTTCTTAAAAGATTGATTCTATAATGTGGTGGTTACAACGCATGCCTTAATAATAATT
TTTTTTGCTGTGATACCTATCTTAATTGGAGCTTTCGGTAATTGGTTGATTCCTCTGCTAGTAGGAGGTAA
AGATATAATTTACCCGCGAATAAATAACTTAAGTTATTGACTATCTCCTAATGCACTATATTTACTAATACTG
TCCTTTAGAACGGATAAAGGAGTTGGTGCTGGATGAACTATTTACCCCCCTTTATCTGTGTACCCCTATCA
TAGGGGCCCTAGGATAGATGTTCTTATTGTGTCACTACATCTAGCTGGGCTCAGCTCTCTAGTGGGGGCT
ATTAACTTTGCTAGGACCAATAAAAATATGCCAGTGTTAGAAATGAAAGGAGAACGAGCGGAGCTTTAT
GTTTTAAGGATTAGAGTTACTGCAGTTCTTTTAATTATTTCAATTCCGGTTCTAGGAGGGGGTATCACAAT
AATCTTGTTTGACCGAAACTTTAACACAACTTTTTTTGATCCCGCAGGAGGGGGGGACCCCGTTTTGTT
CCAACATTTGTTTTGATTTTTTGGGCATCCGGAAGTGTATATTCTTATTCTACCTGCCTTTGGTGTGATATC
AAAAGTAATTATGCATTGCTCTGGAAAAGAAGCGGTTTTTGGTCTAATTGGGATAGTATACGCAATAATC
GGAATTGGAGGGTTAGGGTGTATGGTGTGGGCTCACCACATGTTTACCGTAGGTCTTAATGTTGATACTC
GAGGCTATTTTTCTACTGCAACTATAGTAATCGCTGTTCCTACAGGGGTGAAAGTATTCAGATGACTGGC
AACTATAGCAGGAAGAAAATTCAAAATAAAGCCTGCCGCCTACTGAAGTACTGGGTTTCTGTTTTTATTC
ACCGTGGGAGGGCTAACAGGGGTCTTACTGTCTAGGGCTTCTATGGATGTGTCTCTACACGACACATATT
ATGTGGTGGCTCATTTCCATTATGTGCTAAGAATAGGGGGCGGTGTTTGGGGTGTTCTGTGGTCTTAACCA
TTGGTTGCCAAATTTTGTTGGAGTATGTTTTAATAAGAAATGGAGGAAAGCCCATTTTATAGCAATATTTT
TTGGGGTAAATACTACCTTCTTCCCTCAGCATTTCTTAGGCCTAAGAGGAATGCCTCGACGGTATATAGA
CTATGCTGATATTTATGCTCACTGACATTGGGTGTCTTCTTATGGGTCCGCTGTGTCTTTTGGGTCTCTAAT
ATATTTTAAGTTCCTTCTATGAGAGGCTCTAGTAAGCCAGCGAGGGATGTCTTTTTATGGGAGTCGATATT
TTGGTGATATTGTCCATGAACTAGGGAAAGACCTGTTCCGGTACCATGGTTTTGTGATGATAGTAGCAGT
GGCTGTGTTGGTCTTTGTTATGTATATAGGGTGTGTAATCCTTCTTACTAAATTTTCTTATCGCCATTTCTT
GAACCGTCAACGATTAGAATTTTGATGGACTATTGTGCCAATGTTGATGTTAGTAGGGTTGTGGTTTCCT
TCTATAATTAACCTATATTATATAGAAGAAGTAAAACGGCCCCGGTGAAATTTTAAGGCGATTGGGAAAC
AATGGTACTGATCTTACGAATGTGACACTTGTTATACAATTGATTCTTACATAGAAGACCAGCAGGAGAC
AGGGTATCGTTTGTTGGATGTTGATAACCGGATGGTGGCTCCAGCAGATGTGCAAATAACTGCTTTTGTA
AGAAGGTCTGATGTGCTCCATTCGTTTGCACTCCCTAAGTTACTAATTAAAGTAGACGCCATCCCAGGTC
GAATTAATCGGCTTCCTATAAAAGCTTCCCAGTGTAGAATTATTTACGGGCAGTGTTCTGAAATTTGTGG
GGTTAACCATAGATTTATACCGATTGTGATTGAGTTTATTCCTGAGAAATATTTGTCATATGGTTGGAAGC
TCTTAACTAA------------------------
ATGAATCGTAATCCTTACTATGTACCAGGTCCAAGTCCGTGGCCCTTCTTTGTGGCTATCTCGGCAAACG
GAATAGCGGTAGGGTTAATTTTGTGACTGCATCGAACTCCTTTTCTATTAATAGGAAGGTTGGGGTGTATA
CTATTGAGAACTTTTAGATGATGGCGAGACTTAATTCGTGAGGGAGATATTGGGTTTCATACTCGCTTCGT
AATCAAGAGATTTCGTGATGGAGTTGCCCTTTTTATTCTGTCTGAAGTAATGTTCTTCTTTTCTTTTTTTTG
GACTTTCTTCCATAATGCCCTAAGACCCTCGTGTGAACTAGGGATGCGATGACCCCCTCCAGGGATCCGC
ACGCCAAACCCGTCGTCGACAAGGCTGTTCGAGACAGGTCTTTTAATTAGGAGTGGGTTATTTGTAACT

CAAGCCCATAAGAGAATGCGTTTGGATTATGATGTTGGGCCATTTATTGGCTTAGTGGTAACAATTTTATG
TGGGACCGTGTTCTTCCTAGTGCAACTTCGAGAATACTACTGAAACTCGTACACTATTGCAGATAGGGTG
TATGGAAGAGTGTTTATTTACTAACTGGGTTTCATGGAATGCACGTAGTCGTGGGGACTCTTTGACTAA
TGGTGAGGTTAGTCCGACTATGGCGTGGGGAGTTTTCCAGTCAACGGCACTTTGGTTTTGAGGCTTGCA
TTTGGTACTGACACTTCGTAGATGTGGTATGGGTAGCATTATGATGTCTAGTATATGTGTGGTTTGGAGGA
CCGTGACGAAGTACTAATAAGCTGGTGAAGATTATGAATGACAGGTTCTATGATTTGCCTTGTCCTGTAA
ACTTAAACGCTTGGTGAAGGTTTGGCTCTATACTAGGCTTGTGCCTGATTATCCAACTTCTAAGGGGTCT
TTTATTGTCAACTCATTATACTGCTCATGAAGACATGGCATTCGATTCTGTAGTACATATTATGCGTAATGT
GGAAAAAGGATGAATGTTGCGTAATATTCATGCAAATGGGTCCTCTATGTTTTTTATCTGTATTTATGCGC
ACATTGCTCGTGGGCTGTATTATGGGTCTTATTTAGATAAGACAGTGTGGTATTTTGGGGTGCATTTGTTT
TTGTTAACTATGGCGGAGGCTTTCCTCGGTTACACTTTGCCTTGGGGGCAAATATCATATTGGGGGGCTA
CTGTTATTACTAATATACTTAGAGTGATCCCCGTAGTAGGAGAGAGTATGCTCCGCTATGTATGAGGGGGT
TGGACCGTGTGTAATGCAACTCTAAAGCGGTTTTATACCTTACACTTTCTCTTACCGTTTGTGATAGTGGC
GGTTGTTTTTTTACACCTGTTTTTTTACATGAGAAAGGGAGTAATAACCCTTTGGGTATTGAAAGAGGT
ACTATGTGTGTGCCCTTCCACCCCTTCTATACTATTAAAGATCTTTTTGGTTATGTTTGCTTTAGGTTCTTT
TTTATATATTTAGTGTGTGTGGATCCTGAGCTGTTAGGGAATCATTTAAACTATTGGCCTGCTAATCCTATA
AAAACGCCAATCCATGTTCAGCCTGAGTGGTATTTTATGTTTGCTTATGCAATCCTTCGTTCAATTCCTCA
TAAAGCGGGGGGGGTATATGTTATGTTTTTGTCGATTGTAGTATTATACCTAATTCCTAGTCTTCACAGAG
GTAAGTATCGAAGTTTATGTTTTTACCCGTTTAATCAAGTAGTGTTTTGAGTGTTGGTTGGTAGGTTTATT
AGGTTAACATGGATTGGTGCTCGCCCAGTGCGGGAGCCTTATATCATTTTGGGGCAGTGTCTTTCAGTCA
TTTATTTCTCTAGGTTGTTATTAAACCCTCTTTCTTTGTGGGTGTGGGACAAGCTGCTTGAAGTAGGCGT
GGTTGTTGGTGTTATCCCTTTTGTAGGGGTGCTTCTCGCTGTGGGCTTCTATACTTTGTTGGAACGTAAA
ATTTTGGCTATCATTATAATCCGAAAGGGTCCATCCAAGGTGAGTTATATAGGGATCTTGCAGCCTTTTAG
TGATGCAGGTAAGTTGTTATGTAAAGAGTTTATTGTGCCTACACGTGCTAACGTAGGGCCTTTCATTTTG
GCTCCTGCACTAATATTAACTATCAGTTTACTTGGATGGCTTTTATACCCGTATAAGTCGGCTGAAGTGTT
TTATGTTTTCGGGGTGATTCTGTTTATAGTTATTACTAGAGTCAGGGTTTACGGGGTAATAATATCCGGATG
GGCTTCTAACTCTAAATACTCTTTGCTAGGTGCAGTTCGTGCGATGGCGCAAAGAATTTCTTATGAGATC
CCTATAGGATTTATCTTCTTTTGTGTGGTGCTGTGCTCGGGTGTGTTTATGTTTCAAGAAATTAGGGTGTC
CTTTTTTTTCTTTCCTTTGTGCGTAGTTATAGTTGTCTGAATGCTGTGTATGCTAGCTGAAACTAATCGGG
CGCCATTTGATTTTGTGGAAGGAGAGTCGGAATTAGTGTCAGGATACAACGTGGAGTACAGCGGAGGG
GGGTTTGCAGTTATATTTATTGCGGAGTACTCTAGTATTCTTCTCAGAAGGGTTATAAGGGCGGCGATATT
TTTCGGGGGAAATGAAGTGTTGATCGGGGTCTTTATGATGGCTTTTGCGGTCTTCTTTGTGGTTATTCGT
GCTTCTTTACCTCGTTTACGTTATGATAAGTTAATGAGTTTGTGTTGGACTGTTCTTCTATGTGTCATACTT
ATGGCTAGTGTGTGTGTAGTAGTTCTAGTTAGGGTGTAATGGTAAGCTTTGTGGTAAGACCTATAAAATT
AGTGAGATTAGGGGTAATATTGATCGGGACAATTCTTAGGGTTAGAAGAGCAGAGATAGTAGGGGTGTG
ACTCGGTCTAGAGCTAAATCTGTATGGATTTCTTGTAATTATAAACCCTGATGGGCACTATAGTCCTGAGC
CCTGTGTAAAATATTTTGTGGTACAAAGAACGGGGTCAATTCTGATACTAGTGGGTTTTGTAACCTTGAT
ACAGCACGCAGTGAGAGGGCTGGTGATAAGGAGGGCGGGTACAGTGCTAAAATCTGGCGTTTTCCCGC
TACATTCGTGGGTCCCTTCAATTATTAAGAACAGCAGATGGTTAGCAAGAGGGTTAATATTAACTTGGCA
AAAAGTAGCCCCTCTTGTCTTTTTATCAATAATTATACCCTCTAAGGGGTTGTGAGTAGTAATTGTATTGA
TAGCTGGAATTGGGGCAGTAGGGGGCCTTAACCAGAATTCAGTACGAGTAATAAGTGCGTACTCGTCGT
TTGTGCATACATCATGAATGCTGTTAGGGCTCACATGGTCAAGAGTAGTCTTTGTAGGGTATTTTGCAGTT
TATTCGCTGTCGGTAGGGCTGTTTTTTTATGGGTGCTCAATAATAAACAAAACAAGAATGGGCGGTCAGA
TTAGTAGAGCCGCGAGGGGTATAGGGTTACTGATACTGATGGGGATGCCTCCTTTCCTTGGCTTTCTAGC
GAAAGTATTGGTGTTTCTAATGAGAGGAAGGGCTGTAATCGTGGCTTGTATTATAGGTTCAGTAATCAGG
CTAAAATTCTACATTGACTTTTTTTATAGGATAGTAATAAAAAACAAAGCAGAATTCAAGATTATGTGGA
GGATAGTGATCGGGGCTAACCTAGCAGGGGGGGCATTGATCTTGGTGAGATTTATTTAGATGGTTATGGG
GTTAAGAGTTGCGTTTGTCTGCATTGTGTCTTTTTTGTTTACGGGGTTAATGCTACTAAGGGAAAAGCGG
GGCCTAGACCGAGAAAAGTGCAGTCCATATGAGTGTGGATTTGAGCCTATTGGAAGAGCTCGGAGGCC
CTTTTCTATCCGATTCTTTCTAGTAGCAGTTTTGTTCGTCGTGTTTGATGTAGAGGTAGTGCTGTTAATACC
TTTTGCCTACATGTTCTTTTACGGTAAGAGAGTGCTAGGGATTTTGTCCTCAAGGGGTTTCCTTCTTATCT
TGTTTGGGGGTCTCTACCACGAATATCGTGAGGGGTCCTTGGAATGAGTAGGTTAAATGGTTAAAAGGT
TGGCAATTAGACTTATTGCATTGATTATTATAAAAAACCTAAATATGAGTGTCATTGGGTTAAGCGTTCTA
ACTATTCTAAGTATGGGCGCTACAAGAGTCGCGATAGGGGGGGTAGAGTTGAACGGGTTGTACACCACA
GACTTTGTAATAGGGTTAATGGTTACACTAACTCTATTTGTAGCAATTCTTTCCTATCTAAGGAGGGTTAA
GATCCACCGGAAAGCAAGATTTAATTTAATAATTATCAGAATCAGCCTAATTTTAGTGATAAGATTCAGGG
TGAGGAGGTTCTTTCTTTTTTTTTTTTTTTTTGAAAGTGTGCTAGCCCCTCTGTTGTTATTAATTGTAGGCT
GACGTTTACAGGCAGGGGGGTACATAGTAATCTATACGGTGTTCGGATCTCTTTTTTTTCTTATGGGGGGTA
AGAGAACTCTATATTAGAGGGAGGAGGAGGATAAGTGTGGTAAGGCTAGTAAAAAAAAGGGGAATGAG
ATTGTGATGGCTATACATTCTAGGGTTTCTTATCAAGCTACCAATATATCCATTTCACCTGTGACTACCTAA
GGCTCACGTAGAGGCCCCAGTAGCCGGTTCAATGTTATTGGCCGGGGTGGTACTAAAATTAGGAGGGTA
CGGGCTGCTTCGATTTATAATAGTTATACAAATAAGGCTTAGAAGCGTGTTTGTGCTGCTACTAGTGGTG
AACTTGGCAGGAGGTGTCTACGCAGGATTAGCGTGTGTACGGCAAGTGGACCTAAAATGTTTGGTAGCA

TATTCCTCCGTAGCGCATATGAGGCTTGTGCTATTAGTGCTCAGCAACACGGTATTAGGGGTAGTGGGGG
CCATTATCATTATGATCGGGCATGGGTTGTGTTCATCAGGTTTGTTCAGGTATGTGAATGCTATCTATAAGA
TGAGGCACTCGCGTCTGCTAGTAATAAATAAAGGGGGCTTGTTAGTCTGCCCAAGTCTAGTCTTAATGTG
TTTCCTGTTAAGATCAAGCAACATAGCAGCCCCTCCTAGTTTAAACTTATTTGGGGAAATCCTCGTTTTTG
GCGTGGGAGGGTGAATAAGCGGAGTGTTCCTGCTTATCCTGGGTCTGATAAGCTTTATTAGGGCATGTTT
TAGATTATACCTATATGGAAGTTGTTGTCACGGGAAGGGGGTGTCACACAGGGAGTCCTTAAACTTGGTT
TGTGATGTTTTTGTTCTGGCGGCTCATTGGATACCGCTGAACTTTATGTTTATGTTTATACCCTAACGTATA
AACAAACACCTTTTGTGTTTATTTGTAGGGCTTGAAATAATAAGACTAGGCTTGTTGTTTGTAACCCATG
TGTTTCTAATAAATCAGTTTTGGTTAATTTTACTAATTTTATGTTTAGCTGTTTGCGAAGCCAGAATTTGCT
TGGCCCTTCTGGTTATGGTGATGCGACTATGCGGAGACGATTTGATGTCAAGGTTACTAAGAGATGTATA
CAATTAAAGAGTAACTTAGGGTTACTTTTACTGATTCTGGGATACTTATTTATTCTTAGAGGGAGGACCGG
AAAAGCTTATTTATTGGAAGTTCCTGTTTGAGAGAGTAATTGCCTCTCATTTAGCTTCAGAGTTCTACTAG
ATAGCGTAAGAATAGTTTTTGTTGGGACGGTTTTGGTAATTAGAGGAAGTGTAGCAACTTACTGCAAGTG
GTATATAGCTGGAGAGCCATACTACAAGCGGTTTATGGGATTAGTATGGTTGTTTGTGCTGTCTATAGTGT
TTATAATCTTAGTTCCTAATTTAGTAATACTTTTAATTGGTTGAGACGGGCTAGGGCTCACCTCATTCCTAT
TAGTGGCTTATTACCAGAACAATAAGAGGCTATCTGCGGCTATGTTGACAGCTTTGACTAATCGAATTGG
GGATGTTCTTGTACTCCTTAGAGTTTCTATTTTTTTAAGAGAAGGGGGGTGGTTAATTTATATATACCACC
CAGTGCAGACATGGGTTTTAGGGTTTGTGGTAGTTCTTGCAGGTATAACTAAAAGCGCACAAATGCCGT
TTTGCGCATGGCTACCTGCTGCCATGGCGGCACCCACACCGGTCTCCTCTTTGGTGCATTCTTCGACATT
GGTGACAGCTGGGGTTTATTTGATTCTTCGCTCTTTTTATATTATCAGAGCTAATGTGACTCAAATACTTAT
AGTCTTAAGACTATTTACTTTAATATTAGCGGGGTCAAGGGCTGTGTTTGCGTTTGACCTAAAAAAGGTA
ATCGCACTCTCGACTTTGAGGCAGTTAAGGTTAATAATATTCTCGATTTCAATCCTTCTTCCGTCTGTAGC
TTTTTTTCATTTAGTAACCCATGCGGTATTTAAAGCTTTGTTGTTTCTAGGCGCAGGGGGTGTTATTCATA
GAAACCAAAGAATCCAAGATATCCGGGGGTTAAGAAGCTTGTGGCAAGGATTACCGGTAAGAATGGGT
GCAATAACGGTTGCAATTGTGTCCTTGAGAGGGGCCCCGTTTATAAGGGGGTTTTTCTCTAAAGACCTAA
TAATTGAGATGATAGACAGAAGAACTTATGGGTATTTATTAGAGCTAACAGGTTTAATCTTCACTTCTTTT
TATAGGGCACGGGTATTTAGAGTGATACTTGGGTCTAATTACGTTAATAGCAGAAGTTTGCGGATTAATGA
GCACTTAAATATACAAACTCCTTTTCTTAGCCTGTATATTGGGGCTATTATATTAGGAGGGGTATTAGGGA
GGAAAATAGAAAGGTTTGGGTTTGTAGTAGTTCTTGAGAAATATGAGAGAGTCAGAGTATTTCTTATTCC
CTTTGGGTTATTGTGATGAGGAGTGCTTAGAAAATTAGGGTCTAAGCCTGCCAAATTAAGATTCTTTTTG
AGAATGTGGCTCATAGAGCTAACCCACCCCGGAAAAATGGCCTTCTTTAAGGGGTCCAGGACGGTAGCT
CAAAGTCTGGATCAAGGTTGACTAGAGCTATTGGGTCCTCAAAAGGGACTAGGTCAATTCAGCTGTTTG
AATGAAAATTATTTTACCGTAGTATGAGTGGTAGCGGGGGGCGTAAGCTTAATTATGTTTATATAAATGAG
AGTTATAGTCATATGTGTAATTTTGATGGCTGTGTTTGTCTTAATTGCCAAGCAACCTATTTCTTTAGGGCT
AGTGCTATTGAGGGGGTCTATAATTGCATGTGTGGAGGTTGCACTGGAGGTTAGAAGGTTGTTAGGGTT
CTTATTGTTCCTAACTTACGTTAGGGGTGTTATAGTCCTGTTCTTGTATGTTTTAAGGATCTACCCGAATGA
ACGTTTTAATCTAGAGTTTATGGTTCTCGTCAGAAGGTGTGCCAGAGCGGTCCTTATGGGTCTTATGAAT
TACGAGAGCGGGTCTTTGTTTCTAAGTTTCATGGCTGAAAGAAGCTTATATATCCTAATAGCTGGTGTGTT
ACTGTTTGTAATATTAGTGGTGTCGTATTTGTGCATAAAAACCATGGTGCCTTTACGAAGAGTA"/>
<sequence id="seq_Arcuatula_senhousia1" spec="Sequence" taxon="Arcuatula_senhousia1"
totalcount="4"
value="ATGATAACTGATGTGTTTTCTGTTTTTGATGAACATTACATGAGAAGAAGATATGGCTACTATCTAT
GATTTACGTGTGTTGGGATTCCTTTAGTACTATTGTTTTGTGACTTAGTGCTTCGTGATAAACTGTTAGAA
GGCTCTTTCATACTTATGAAGAGCATTAACTGTTTTAAGTTAAGAGGGTTTCCTTTAGGTATTAGGAGGTT
ATTGATATTGATCCTTACTGTTAACGTATTTTCTCTATTTCCCTATGTTTTTAAGGTTAGTGCACATTTTTCA
TTTGGTAGGAGGTTCTCTTTGAGTATATGAAGTGCCATTATTATTTCTAGTGCTTTTTGAAGGTTTGAGCA
AACTTTTGCTATATTGGTACCGTATGATCCGGTGGTAATAGGCCCTTTTATGGTGTTAATTGAGTGTGTTTC
GCATCTTCTTCGTCCTATGTCTTTATTTATTCGATTAGCAATAAACTTAGCTACTGGAAAAGTTATAATAGT
AATAGCTACCTCTTTAGGTTTAAGCCTGCTTATCGTGGGGGAGCTTCTTGTAACATTCTTAGTTTTACTAG
GTAGAATAATGTGTTTTGAAGCTGGAAGGGGTGTTGCTCAGGCATTTATTTTTTGTTTTTTGTTATCATTAT
ATGCATCAGAGCATAGTGAGTAGGCTGGTGCAAGTTTAAGTCTTCTTATCCGAGTTCATTTATCGCATCCT
GGTAATTACTTTATTAAAAGAGAGTCATTTTATAATGTTGTTGTTACAACAGCTCTTATAATCTTTTTTGCT
GTAATGCCTTTGATTGGTGCCTTTGGTAATTGGCTGATTCCTTTGTGTATTGGAGGAGGTGACTTAGTGTT
CCCTCGTCTAAACAATCTGAGGTATTGGTTAGCTCCTAATGCTTTGTATTTATTAATGATTTCTGTTTTTAC
TGAAAAAGGGGCTGGTACAGGGTGGACAATCTATCCTCCTTTATCTTCTGTTGAATTTCATAGAGGTCCT
GCCGTAGACATCTTAATTACTTCATTACACGCAATTGGTCTTAGTTCATTAGTAGGGGCTATTAATTTTGG
GTGTACTAATAAAAACATACCTGTCCCTAAAATGAAAGGGGAAACGTCTGAGCTTTATTTATGAAGGCTA
ACTGTTACGGCTGTTCTTTTGATTATTTCTGTTCCTGTTTTAGCAGGAGGTATTACTATGCTATTGTTTGAC
CGTAATTTTAATAGAACTTTTTTTGACCCTATCGGAGGGGGTGATCCTGTTTTGTTTCAACATTTATTCTG
GTTTTTTGGTCACCCAGAGGTTTATATTTAATTTTACCTGCTTTTGGTATTATATCTAAAGTAGTAATAAAT
CAAAATGGTAAAGAAGCTGTGTTTGGTCAAGTAGGGATGCTTTATGCTATAGTTGGTATTGGGGGCCTAG
GTTGTGTTGTTTGAGCTCACCATATATTTACTGTTGGGATAAATGTAGACTCACGTGCTTACTTCACTAGC
TTGACAATGGTTATTGCAGTACCAACGGGTGTAAAAGTATTTAGGTGAATAGCTACTATAAGTGGTGGTA

AGTTTAGGATATCCCCAAGAGGGTTTTGAAGTTTAGGGTTTTTATTCTTATTTACAGTAGGGGGTCTAACA
GGTGTTATGCTTTCTAGATCTTCTTTAGATGTCTGTTTACATGACACTTATTATGTGACGGCTCACTTCCAT
TATGTGTTGTCAATAGGAGCTGTTTTTGGTATTTTCTGTGGGTTAAATCATTGATTTCCTTTATTTTTTGGG
GTTAATTTGAATAAAAAAATGGTCATTGACACATTTTTTCATTATGTTTGTAGGTGTTAATATAACCTTTTTT
CCGCAACATTTTTTAGGGTTAAGAGGAATACCCCGTCGTTACTGTGATTATGCTGATTGCTACGCTAAGT
GGCACTGGTTTTCTTCTTATGGTGCTATAATTTCGTTTGGCTCTTTAATATACTTCTTGTTTATTGTTTGGG
AAGCAGTTGTCTGCTCTCGTGGGCTCGTTTTTTCTAGAAGAATAGCTAGGGATCTTGAGTGACAAAATC
AGGTATACCCTCCTTATTCGCATACTATGCCGTTGTGAGGTAGAAAAGGTTTTCAAGATTCTTATTATCAA
GTAGGTATAGACATTATAAAGTATCATGGTCTTGTAATAATGGTAATTGTTTTTATTTTAGGTTTAGTAGGT
TACATAGGTGTGTTTTTGCTTTGTCGTTCTTTAAGGTATCGTCATCATTCTCAGTGAGAAAAATTAGAGTA
TGGTTGAACAGTTTTGCCTATTATCCTATTAGCTTTACTGTGAGGCCCTTCTATAAAAAATTTATATCATAT
AGATGATATTAAAACTCCTCAGTGAACTTTTAAAGCAGTTGCCTCTCAGTGGTATTGAAACTATGAACAT
CAGCAGCAGTTCGAGTTTCAGTCTTATATAGCCGCAGATAGGGGAGCTGGTTATCGGTTGTTAGATGTAG
ATTGGCGGATAGTTGTACCTGCTCAATCTCAAGTCACAATGTATGTGGCTAGAACAGATGTTTTACACTC
TTTCTCGTTACCTTCTGTTTTGTTAAAAGTGGATGCTATTCCAGGACGTGTAAATCAACTTCCTTGTGTGT
TTGGCTTGCCAGGTGTTTATTATGGGCAGTGTTCTGAGATTTGTGGTATCAACCACTCTTTTATACCTATT
GTAATAGAAGTAATCCCTGCTAAAGTATTCATTGGTTGGTTAGAAGGTCTGACTAAC------------------------
ATGGCTCGTAATCCTTATTATGTTCCTGGTTTAAGTCCTTGGCCTTTTTTAGTTGCTCTAAATATTGGAAGA
CTTTGTTTAAGTCTTGTAATGTGAATACATCGAAGGGTTTTTATGATTTTATTACCTGTTTTAGGTGTTCTT
TTGTGTTTATTATGTTGGTGGCGTAATTTATTAGATGAAGTAGACTTAGGTTTCCACAATCGTTATGTGGTA
AAAACTTATCGCGATGGAGTAGCAATTTTTATTTTTAGTGAAGCTATAATGTTTTTCAGCCTTTTTTGAGC
TTTTCTCTATTCAAGGGAAAGGCCTTCAGCGAGATTGGATTTATTATGGCCTCCTTTAGGTGTTCGCTGTC
CTAAGCCTTTTAGGACGGCCCTATTCAGAACTGGCCTTTTAATCAGTAGAAGTTTTTATTGTGTTTGGGC
GCATAAAGCTATGTATAGTGATTATAGATGAGCCCCTATTATTGGTGTGGCTTATTCTGTTCTTTGTGGGGC
TGTCTTTTTGCGGTATCAATTTCATGAGTACTACATAAACTCTTTTTCAATAGCCGATAGGGTTTTTGGGA
GGTGTTTTTATATCTTGACAGGGTTTCATGGTTTCCATGTGGTTATTGGCAGTTTGTGATTATTGGTTAGTA
TATTTCGTTTAATTTTGGGGCATTTTAGTCGCAAGCGTCATTTTGGTCTTGTGGCCTGTTTTTGATATTGAC
ACTTTGTTGATGTTGTCTGGGTTTTTGTTTGACTTTTATTTTACTTAGTAATAGGTGGGatttttcgaaaacgtaatgga
ttagctaatgttctgacaggaagggtttatgacttgccttgtCCTATTAATTTAAGATGGTGATGAAGGTTTGGTTCTATATTAGGGC
TTTGTTTGACAATTCAATTGATTAGAGGCATCATTCTCTCTTTCCACTATAGAGCACATGAAGACCTTGCG
TTTGATTCAGTTATTCATATCGTTCGTAATGTAAAAAAAGGGTGGTTTTTACGGAGAGTACACACTAATG
GTGCGTCAATATTTTTTATTTGTCTATATGTACATATTGCTCGAGGCATTTATTATGGTTCTTATTTGGATTCT
GCTGTGTGAAATGTTGGTGTAATCCTTTATTTATTGGTTATAGGGGAAGCCTTTCTTGGTTATAGATTGCCT
TGGGGTCAGATGTCTTATTGGGGTGTAACTGTTATTACTAATATGGTTACGGTTATTCCTTTTGTAGGGAA
GGATATTTTGCAACATGTTTTAGGGGGTTACTCTGTGTGTAACAATACATTGAAGCGATTTTACTCTTTAC
ATTTTGTTATCCCTTTTGTAATTATGGGACTAGCAAGGCTTCATTTACTTCTCTTACATGAAAATGGAAGA
AATAATCCTTTGGGTGTTGAGAGAGATAGAATATTAGTTCCTTTTCATCCTTTTTACACGGTAAAAGATCT
GCTGGGGTTTTGTTGGTTTATGTGAGTTTTTGTTTATTTAGTTTGTGTTAATCCTGAGTTGTTAGGTAACC
CTGTTAATTTTATTCCTGCAGACCCTATAAAAACTCCTATTCATATAAAGCCAGAGTGGTATTTTATATTTG
CTTATGCTATTTTACGTTCTATCCCTCATAAAGCTGCAGGTGTTATTGCTATGTTAGCTTCTATTTTGGTTTT
ATTTCTACTCCCCTTGTTACATACAGGTAATTTTCAGGGGCTCGTTTTTTATCCTATTAACCAAGGGTTGTT
TTGGGTTTTTGTTAGGAGGTTTTTAAGGTTAACGTGAATTGGAAATAGCCCTGTTTGTGAGCCTTATGTA
ACCTTGGGGCGGGTGTACAGAGTTATTTACTTTAGATCTATCTTGTTAATCCCTTATAGCCTTAAGCTATG
GGATAAGCTTGTTTTTGCTGAGTTTGTAGTACTGATTTTACCTTTTGTATGTGTTTTGGTTTCGGTAGCTTT
TTATACTTTATTGGAGCGTAAAGTTTTAGGTTATATTATAAGTCGGAAAGGGCCAAATAAGGTGGGGTTTA
TAGGGGTCATTCAACCTTTTAGCGATGCCGCAAAATTATTTACTAAAGAAATAGTTATGCCTGGGTTTTCA
AACAACATGGTTTTTGTTCTATGCCCTGGCTTGATCTTACTTAATGGGTTGAGATTATGGCTTCTTTATCCT
TTTAGTTATACAGAGGTGGTATTTGTTTGTGGGTTAATTCAGTTTCTAGTGGTCTCAAGAACTAGTGTTTA
TGGGGTGATGCTAGCAGGCTGGTCTTCTAACTCTAAATATGCTTTATTAGGTTCCGTACGTGCTGTTGCGC
AAAGAGTTTCTTATGAAGTGCCTTTAACATTTGTGATAATTATTATTTGTTGCTTGTTAGGAAGAATGCTC
TGTCAAGAAGTGAAAGAATTTTTTAGTTCTATTTTATTTGGTTTAGGCGGAATAATTTGGTTAGTGTGTAT
ATTGGCTGAATCTAATCGTGCTCCTTTTGATTTTGTTGAAGGTGAATCTGAGTTAGTTTCTGGTTTTAATG
TGGAGTATAGAAGAGGAGGCTTTGCGATAGTTTTTATAGCAGAGTATGCAGCAATACTGTTTAATAGTCT
TTTTTTTACCGTTTTATTTGTTGGGGGCAGAGGGGCAGTGGTTATATTGGGGATAACGTTAGTTGTAGTGG
GTTATGTATGAGTTCGTGGGTCTTTTCCTCGTATGCGGTATGATAAAATAATAAAACTGTGTTGACGTTAT
TTAACGGTTTTAGTCCTCTGTTTAGGTGTTTTTTGTCTGTGTGTCCCTTACTTATAATATTGGTAGTTATGT
TTAGTCCTTTAATGTTGTTTTGTTCATGCCTGGTGGTAGGAGGTAGTTTGCTTAGTATCAGAAGAAGAAC
ATGAAGGGGTGTCTGAGTGGGGATAGAGCTAAATCTTTTCTCATTTTTAATTTTGATAAATGGAGGAAGC
TTTTTCGATTTAGAACCTTTGATTAAGTATTTTGTAGTCCAAAGGTTAGGTTCAGTTGTGTTTATTTTTAGT
GTAGTTTATGTCTCTTTTTTTTAGAAACGTGATAATAATCCTTCTAGTATTAGGTCTATTTTTAAAGATTGGG
ATTTTTCCTTTTCATAGATGGGTTCCCAGGGTTGTTGTAAAAAGCCGTTGAATTGTAGGTGGGTTGGTGT
TAACGTGACAAAAACTGGCGCCTTTGGTTtttTTTTTGATGTTAATTAGAAGTAGTGTAGTTTTTATTAGGG
TTTTGTTTATAGTAGTTATTGGCGGCGCTGGCGGTCTAAACCAAAGCAGGGTGCGGGGTATAGCTAGCTA

CTCTTCTTTCGTTCACATATCTTGAATAATGGTAGGCCTTCTCTACTCATTTTTTGTGTTTTTGTTTTATTTT
TTTATTTACTCTATATCTCTCTTTTTATTTTTTCTAGGCTGTAGCAATAGTGGTAAAAGCTCTTTAGGGAGT
CAAAGGTTTAGTTTACTAGGTTTAATTGGGGTTCTTATAATAATAGGGGTTCCTCCTTTTCTGGGCTTTTTA
AGGAAATTATTAGTAATGTTATCTAGGCCTACTTTCGCTTTGGTTGTTTGTCTATTAGGTTCTGTTGTAAGG
CTTAAGTTTTATACTTCTTTCTTTTATAGTATGTTTTTAAACGGTTTGTATGGAATTGAATCTGTAGTGTCG
CTTAGCTTAGTATTAAATGTTTTAGGATTATTTTTAATTGTGGTAGTGCTTTTTTTGGTTTTTTTATGTTGCga
gatggttTTTTTGTTAATGCTTAGATTTAGGCTACTGCCGTTGTTTATACTCTCCTCTAAATCTGATTATAGGCG
TGAAAAGCTCTCTCCTTATGAGTGTGGGTTTGAGCCTGTATTCAGTGCTCGAACTAGGTTTTCTGTTCGG
TTTTTTTTAGTCGCTGTCTTATTTGTGGTGTTTGATGTTGAACTGTCTATCTTAGTAGCAACAATTTTTTCT
ATCAGGCTTATAAAAAACTTAATCAGACTAGTGAGTGTTATTGTTTTTCTAGTTGTTCTTTTTTTAGGGTT
GTTTCATGAGTTTCGTGAGGTTCGTTAAATTGAATTTACTtAaGTGGTATTGTGGTTATTAAGTATATTTCTG
GTGTTAATAGTAGTATTGCAACGCGAGTTAGTGGTTAGGGCACTACTAATAAGGTTTTATTTGGCACTCTA
TCTCTGTGTAGTACCAGATAAAGTGTGGATACTTCATGGTGTTATAGTCTGAGATTTTTTGAGTTTAAGAA
TATCTCTTTTAGGGGTATTTGTAGTGGCAATAGGGGTATTAAGAACAAAGGTGAGCGTGCGGAACAAGC
TATTTCTATTGGTAAATATGTTTTTATGTATAGTTGTAGTAATAGCGTTTAGCGTAAGAAGTTTCTTTCTTTT
TTTTTTTTTTTTCGAGAGCTCTCTGATTCCTTTAATTTTAATAATCGTAGGCTGGCGTTTACAAGCTGGGG
TGTACATACTAATTTATACTGTAGTAGGTTCTCTATTTTTTCTTTTAGGGGTTTGTTTTATATATTTTAATAGA
AGAGACTGTATAATCTTAAGAAGGTCATGTGTTAAAAATATATGGAGGTTTTGATGAGTATTCTTGTTTGG
GTTTTTGGTTAAAATACCTTCTTATCCCTTTCATTTATGACTTCTAAAAGCACATGTGGAGGCACCTGTAG
CTGGTTCTATAGTGTTAGCAGGGGTTATTCTTAAATTTGGGGGGTATGGCATAATCCGATTGTTTAGGGTT
ATTAATGTCTGTTATTACGATAGGTTATGTCTGATTGTAGTCGTAAGACTATTAGGGGGGTTTTACAGAAG
CGTTGTTTGTGTTCGTCAGACTGATTTAAAACGCCTGGTGGCTTACTCCTCTGTTAGACATATAAGAATA
GTAATTCTATGTTTTAGAAATTCTTTTCTAGGGTTAGAAGGGGTTATTTGGCTAATGTTAGGGCATGGCTT
GTGTTCTTCTGGGTTATTCATGTTAGTGTCAGTAGTTTACTCTAGGAGGGAGTCTCGAGTTCTTCTTCTTA
ATAAAGGAGGGTTAGTTAAAGCACCCTGTTTAGCAATAGTTAGATTTCTTCTTTGTGTTAGAAATATGGC
GGCGCCCCCTAGGTTAAACCTGTTGGCAGAGATTTGCTTATATATTTGCATCAGTTCTATGTATCACTATAT
GGTGCTGTTCTTTGCTATAGTTAGGTTTTTTAGGGCTGCCTATAGGCTTTATTTGTATACCAGTTGTTTTCA
TGGTAGCCTGAGAGGAGGTCTATTTAGAGTTCTAGGGCATAGAGATACTATCGTTTTAATATGTCACTGA
ATCCCCTTAAATATGTTGGTTATTATATTATATTAGATACATAAGTACTTTTTATTGAGATTGTTTATTAGTAT
AGAGGTTGTAATCTTAGGCCTTGTTGTTTTATGTGTTGCTGTAAATTTTTTTTATGTAGCTTCATTAAAATT
AGTGCTGTGTCTTGCAGTGTGTGATGCAGGGGTATTTTTGGCGTTAATTGTTTCACTAATACGGCGTGTT
GGAAGAGACAGTGTTAGAAGAATATCTTCTTTTAAtttaaaATATTGAATCATTTTATTTTTTTTGCGCTCATT
ATTGGGTATACTTTTATTTTAGTTGGAAGAGTCTCTAGCGTTTATTTAATTGAGATAGTGTTTTGAGAGTC
AGGAAGAGTAAGGATAGTGTTTAGGGTCTTAGTGGATTATATAAGGCTAGTTTTTATTGGGACTGTAATA
GTAATTGGTGGAAGAGTTTTAATTTACACAAGATGGTACATATCAGATGAGCTTTATTACAAACGATTTGT
TAGTTTAGTGTTACTATTTATTCTTTCAATAGTGTTAATAATCCTTATCCCTAACTTAATTGGGATTTTAATC
GGATGAGATGGTTTGGGTTTAACATCCTTTTTATTGGTGTGTTATTATTGTAACAGAAAATCGCTCGCTGC
TAGACTACTGACGGCCCTTACTAATCGGATTGGCGATGTGCTGATTTAATGAGAATTGGTTTTATAATTG
TGGAGAACTCCTGGTCTTTATATCAATTTACTTTTGGTGAGACAATTTCGTTAGTGGGCTTACTGACTTTT
GCTGCAATAACTAAGAGTGCACAGATGCCTTTTTGTGCTTGACTACCTGCTGCTATAGCGGCTCCAACGC
CTGTTTCTGCATTAGTACATTCTTCTACTTTAGTTACGGCAGGCGTGTATTTACTTATTCGTTCGTTTAAGG
TAATCTCACTTGACTTTATAGAAGTGTTAGAAGTCTTAAGGCTGGTTACTCTTTGTTTGGCTGGGACTAG
TGCGTTAGTGTGTTTGGATCTCAAAAAAGTAGTGGCATTATCTACCTTAAGTCAGCTGAGCGTTATAATA
CTAAGAATTTCCTTGTATGCGCCTGTTCTTGCCTTTTTTCATTTAATTACTCATGCGTTATTTAAGGCACTC
CTTTTTTTATCGGTAGGGTCTGTAATCCACTCTTATGGTAATATCCAAGATATCCGAATAGTTGGAGGCTG
TTGGAGTACATTACCCAAAAGAATAAGGGCTATAGTAATCGCTGTTAGTTCTCTATCGGGGCTACCTTTTT
TGAGTGGCTTCTTCTCTAAAGATCTGATTGTAGATTGCTATAGGAGTAGCAGTCTAATAGTGGTCTTAGTA
GGGGTTGGAATTGCTATAACAAGAGTCTATAGTCTACGAATTTGAAGAAGTTTGTTTAGATTAAATATAG
GCGTGAGTCCTGTTATAAGAAGAGATGAAAAATTAGAACTCACTTTTCCTTATCTGTGTTTAAGGTTAGG
AGCTCTATTTGTGGGCTATGTATTAAGAGGGCAAATTATAGGCGTTTCTCAGTTTAGAGGTTCCAGAAATT
TTGAGATTGTGGTACTAGTGGGCGCATTTTTGGGTTTATTTATATGGGCAAACAGGGGAAGGTTACAATT
TAATCTTAAAAAATTTCCTTTTTTGTTAAGAATATGGTGCTTAGAAGTTAGACAAGTTTTGGCAGGTGTTA
GGCTTTCATTGTCAGAAAAACTGTCATCGAGCTTAGACAATGGGTGGTTAGAGACAATTGGCCCACAAA
GAAACTTAATGAGTGTATCACAAAATAATGAGGGGTTCTTTATGAGTTGCTTAGTGGTGAGTTGTGTTTT
GGTTTTATTCTTTTTTGTAGCGttggagttgttgttagtttcagccgttttgtttttttttATAATTATTTCTTTTAGTTTCCCTTATGCT
TTAGCCATATTTCTTATTATTCTTTCGTTATCTATTTGTTTTTTAGTAAGATGATTTTATAGAAGGTTAATAG
GGTTGCTGATTTTCATAATTTATGTAGGGGGTGTGTTAATCATATTTCTTTACAGCCTTAGAGTGCTACCTA
ATGAAAGATTTTACTCAGACTATGGGTTCTTCTACTTTTTGTGTTGGGTGCTTTTTGTTATGTCTATTTTTA
ATTATGAAATGTCCTTTCATTATATAAGGTTAGTCAGTTTTGGGGGTATATTTGTCTTTATGGCGTTAGTTTT
ATTTTTCCTTATACTGGTAGTTTGTAACTTATGTGATAAAAAGCGTATTCCATTACGGAAGCTT"/>
<sequence id="seq_Musculista_senhousiaGU001953" spec="Sequence"
taxon="Musculista_senhousiaGU001953" totalcount="4"
value="ATGATAACTGATGTGTTTTCTGTTTTTGATGAACATTACATGAGAAGAAGATATGGCTACTATCTAT

GATTTACGTGTGTTGGGATTCCTTTAGTACTCTTGTTTTGTGACTTAGTGCTTCGTGATAAACTGTTAGAA
GGCTCTTTCATACTTATGAAGAGTATTAACTGTTTTAAGTTAAGAGGGTTTCCTTTAGGTATTAGGAGGTT
ATTGATATTGATCCTTACTGTTAACGTATTTTCTCTATTTCCCTATGTTTTTAAGGTTAGTGCACATTTTTCA
TTTGGTAGGAGGTTCTCTTTGAGTATATGAAGTGCCATTATTATTTCTAGTGCTTTTTGAAGGTTTGAGCA
AACTTTTGCTATATTGGTACCGTATGATCCGGTGGTAATAGGCCCTTTTATGGTGTTAATTGAGTGTGTTTC
GCATCTTCTTCGTCCTATGTCTTTATTTATTCGACTAGCAATAAACTTAGCTACTGGAAAAGTTATAATAGT
AATAGCTACCTCTTTAGGTTTAAGCCTGCTTATTGTGGGGGAGCTTCTTGTAACATTCTTAGTTTTACTAG
GTAGAATAATGTGTTTTGAAGCTGGAAGGGGTGTTGCTCAGGCATTTATTTTTTGTTTTTTGTTATCATTAT
ATGCATCAGAGCATAGTGAGTAGGCTGGTGCAAGTTTAAGTCTTCTTATCCGAGTTCATTTATCTCATCCT
GGTAATTACTTTATTAAAAGAGAGTCATTTTATAATGTTGTTGTTACAACAGCTCTTATAATCTTTTTTGCT
GTAATGCCTTTGATTGGTGCTTTTGGTAATTGGCTGATTCCTTTGTGTATTGGAGGAGGTGACTTAGTGTT
CCCTCGTCTAAATAATCTGAGGTATTGGTTAGCTCCTAATGCTTTGTATTTATTAATGATTTCTGTTTTTACT
GAAAAAGGGGCCGGTACAGGGTGGACAATTTATCCTCCTTTATCTTCTGTTGAATTTCATAGAGGTCCTG
CCGTAGACATCTTAATTACTTCATTACACGCAATTGGTCTTAGTTCATTAGTAGGGGCTATTAATTTTGGGT
GTACTAATAAAAACATACCTGTTCCTAAAATGAAAGGGGAAACGTCTGAGCTTTATTTATGAAGGCTAAC
TGTTACGGCTGTTCTTTTGATTATTTCTGTTCCTGTTTTAGCAGGAGGTATTACTATGCTGTTGTTTGACCG
TAATTTTAATAGAACTTTTTTTGACCCTATCGGAGGGGGTGATCCTGTTTTGTTTCAACATTTATTCTGGTT
TTTTGGTCACCCAGAGGTTTATATTTTAATTTTACCTGCTTTTGGTATTATATCTAAAGTAGTAATAAATCA
AAATGGTAAAGAAGCTGTGTTTGGTCAAGTAGGGATGCTTTATGCTATAGTTGGTATTGGGGGGCTAGGT
TGTGTTGTTTGAGCTCACCATATATTTACTGTTGGGATAAATGTAGACTCACGTGCTTACTTCACTAGCTT
GACAATGGTTATTGCAGTACCAACGGGTGTAAAAGTATTTAGGTGAATAGCTACTATAAGTGGTGGTAAG
TTTAGGATATCCCCAAGAGGGGTTTGAAGTCTAGGGTTTTTATTCTTATTTACAGTAGGGGGTCTAACAG
GTGTTATGCTTTCTAGATCTTCTTTAGATGTCTGTTTACATGACACTTATTATGTGACGGCTCACTTTCATT
ATGTGTTGTCAATAGGAGCTGTTTTTGGTATTTTCTGTGGGTTAAATCATTGATTTCCTTTATTTTTTGGGG
TTAATTTGAATAAAAAATGGTCACTGACACATTTTTTCATTATGTTTGTAGGTGTTAATATAACCTTTTTTC
CCCAGCATTTTTTGGGGTTAAGGGGAATACCCCGTCGTTACTGTGATTATGCTGATTGCTACGCTAAGTG
GCACTGGTTTTCTTCTTATGGTGCCATAATTTCGTTTGGTTCTTTAATATACTTCTTGTTTATTGTTTGGGA
AGCAGTTGTCTGCTCTCGTGGGCTCGTTTTTTCTAGAAGAATAGCTAGGGATCTTGAGTGACAAAATCA
GGTATACCCTCCTTATTCTCATACTATGCCGTTGTGAGGTAGAAAAGGTTTTCAAGATTCTTATTATCAAG
TAGGTATAGACATTATAAAGTATCATGGTCTTGTAATAATGGTGATTGTTTTTATTTTAGGTTTAGTAGGTT
ACATAGGTGTGTTTTTGCTTTGTCGTTCTTTAAGATATCGTCATCATTCTCAGTGAGAAAAATTAGAGTAT
GGTTGAACAGTTTTGCCTATTATCCTATTAGCTTTACTGTGAGGCCCTTCTATAAAAAATTTATATCATATA
GATGATATTAAAACTCCTCAGTGAACTTTTAAAGCAGTTGCCTCTCAGTGGTATTGAAACTATGAACATC
AGCAGCAGTTCGAGTTTCAGTCTTATATAGCCGCAGATAGGGGAGCTGGCTATCGGTTGTTAGATGTAGA
TTGGCGGATAGTTGTACCTGCTCAAACTCAAGTCACAATGTATGTGGCTAGAACAGATGTTTTACACTCT
TTCTCGTTACCTTCTGTTTTGTTAAAAGTGGATGCTATTCCAGGACGTGTAAATCAACTTCCTTGTGTGTT
TGGCTTGCCAGGTGTTTATTATGGGCAGTGTTCTGAGATTTGTGGTATCAACCACTCTTTTATACCTATTG
TAATAGAAGTAATCCCTGCTAAAGTATTCATTGGTTGGTTAGAAGGTCTGACTAAC-----------------------
ATGGCTCGTAATCCTTATTATGTTCCTGGTTTAAGTCCTTGGCCTTTTTTAGTTGCTCTAAATATTGGAAGA
CTTTGTTTAAGTCTTGTAATGTGAATACATCGAAGGGTTTTTATGGTTTTATTACCTGTTTTAGGTGTTCTT
TTGTGTTTATTGTGTTGGTGGCGTAATCTATTAGATGAAGTAGATTTAGGTTTTCACAATCGTTATGTGGTA
AAAACTTATCGCGATGGAGTAGCAATTTTTATTTTTAGTGAAGCTATAATGTTTTTCAGCCTTTTTTGAGC
TTTTCTCTATTCAAGGGAAAGGCCTTCAGCGAGATTGGATTTATTATGGCCTCCTTTAGGTGTTCGCTGTC
CTAAGCCTTTTAGGACGGCCCTATTCAGAACTGGCCTTTTAATCAGTAGAAGTTTTTATTGTGTTTGGGC
GCATAAAGCTATGTATAGTGATTATAGATGAGCCCCTATTATTGGTGTGGCTTATTCTGTTCTTTGTGGGGC
TGTCTTTTTGCGGTATCAATTTCATGAGTACTACATAAACTCTTTTTCAATAGCCGATAGGGTTTTTGGGA
GGTGTTTTTATATCTTAACAGGGTTTCATGGTTTCCATGTGGTTATTGGCAGTTTGTGATTATTGGTTAGTA
TATTTCGTTTAATTTTGGGGCATTTTAGTCGCAAGCGTCATTTTGGTCTTGTGGCCTGTTTTTGATATTGAC
ACTTTGTTGATGTTGTCTGGGTTTTTGTTTGACTTTTATTTTACTTAGTAATAGGTGGGATTTTTCGAAAA
CGTAATGGATTAGCTAATGTTCTGACAGGAAGGGTTTATGACTTGCCTTGTCCTATTAATTTAAGATGGTG
ATGAAGGTTTGGTTCTATATTAGGGCTTTGTTTGACAATTCAATTGATTAGAGGCATCATTCTCTCTTTCC
ACTATAGAGCACATGAAGACCTTGCGTTTGACTCAGTTATTCATATCGTTCGTAATGTAAAAAAAGGGTG
GTTTTTACGGAGAGTACACACTAATGGTGCGTCAATATTTTTTATTTGTCTATATGTACATATTGCTCGAGG
TATTTATTACGGTTCTTATTTGGATTCTGCTGTGTGAAATGTTGGTGTAATTCTTTATTTATTGGTTATAGGG
GAAGCCTTTCTTGGTTATAGATTGCCTTGGGGCCAGATGTCTTATTGGGGTGTAACTGTTATTACTAATAT
GGTTACGGTTATTCCTTTTGTGGGGAAAGATATTTTGCAACATGTTTTAGGGGGCTACTCTGTGTGTAAC
AATACATTGAAGCGATTTTACTCTTTACATTTTGTTATCCCTTTTGTAATTATGGGACTAGCAAGGCTTCAT
TTACTTCTCTTACATGAAAATGGAAGAAATAATCCTTTGGGTGTTGAGAGAGATAGAATATTAGTTCCTTT
TCACCCTTTTTATACGGTAAAAGATCTGCTGGGGTTTTGTTGGTTTATGTGAGTTTTTGTTTACTTAGTTT
GTGTTAATCCTGAGTTGTTAGGTAACCCTGTTAATTTTATTCCTGCAGACCCTATAAAAACTCCTATTCAT
ATAAAGCCAGAGTGGTATTTTATATTTGCTTATGCTATTTTACGTTCTATCCCTCATAAAGCTGCAGGTGTT
ATTGCTATGTTAGCTTCTATCTTGGTTTTATTTCTACTCCCCTTGTTACATACAGGTAATTTTCAGGGGCTC
GCTTTTTATCCTATTAACCAAGGGTTGTTTTGGGTTTTTGTTAGGAGGTTTTTAAGGTTAACGTGAATTGG

AAATAGCCCTGTTTGTGAGCCTTATGTAACCTTGGGGCGGGTGTACAGAGTTATTTACTTTAGATCTATCT
TGTTAATCCCTTATAGTCTTAAGCTATGGGATAAGCTTGTTTTTGCTGAGTTTGTAGTACTGATTTTACCTT
TTGTATGTGTTTTGGTTTCGGTAGCTTTTTATACCTTATTGGAGCGTAAAGTTTTAGGTTATATTATAAGTC
GGAAAGGGCCAAATAAGGTGGGGTTTATAGGGGTCATTCAACCTTTTAGCGATGCCGCAAAATTATTTAC
TAAAGAAATAGTTATGCCTGGGTTTTCAAACAACATGGTTTTTGTGCTATGCCCTGGCTTGATCTTACTTA
ATGGGTTGAGATTATGGCTTCTTTATCCTTTTAGTTATACAGAGGTGGTATTTGTTTGTGGGTTAATTCAGT
TTCTAGTGGTCTCAAGAACTAGTGTTTATGGGGTGATGCTAGCAGGCTGGTCTTCTAACTCTAAATATGC
TTTATTAGGTTCCGTACGTGCTGTTGCGCAAAGAGTTTCTTATGAAGTGCCTTTAACATTTGTGATAATTA
TTATTTGTTGCTTGTTAGGAAGAATGCTCTGTCAAGAAGTAAAAGAAGTTTTTAGTTCTATTTTATTTGGT
TTAGGCGGAATAATTTGGTTAGTGTGTATATTGGCTGAATCTAATCGTGCTCCTTTTGATTTTGTTGAAGG
TGAATCTGAGTTAGTTTCTGGTTTTAATGTGGAGTATAGAAGAGGAGGCTTTGCGATAGTTTTTATAGCA
GAGTATGCAGCAATACTGTTTAATAGTCTTTTTTTTACCGTTTTATTTGTTGGGGGCAGAGGGGTAGTGGT
TATATTAGGGATAACGTTAGTTGTAGTGGGTTATGTATGAGTTCGCGGGTCTTTTCCTCGTATGCGGTATG
ATAAAATAATAAAACTGTGTTGACGTTACTTAACGGTTTTAGTCCTCTGTTTAGGTGTTTTTTGTCTGTGT
GTCCCTTACTTATAATATTGGTAGTTATGTTTAGTCCTTTAATGTTGTTTTGTTCATGCCTGGTGGTAGGAG
GTAGTTTGCTTAGTATCAGAAGAAGAACATGAAGGGGTGTCTGAGTGGGGATAGAGTTAAATCTTTTCT
CATTTTTAATTTTGATGAATGGAGGAAGCTTTTTCGATTTAGAACCTTTGATTAAGTATTTTGTAGTCCAA
AGGTTAGGTTCAGTTGTATTTATTTTTAGTGTAGTTTATGTCTCTTTTTTAGAAACGTGAATAATAATCCTT
CTAGTATTAGGTCTATTTTTAAAGATTGGGATTTTTCCTTTTCATAGATGGGTTCCCAGGGTTGTTGTAAA
AAGCCGTTGAATTGTAGGTGGGTTGGTGTTAACGTGACAAAAACTGGCGCCTTTGGTTtttTTTTTGATGT
TAATTAGAAGTAGTGTAGTTTTTATTAGGGTTTTGTTTATAGTAGTTATTGGCGGCGCTGGCGGTCTAAAC
CAAAGCAGGGTGCGGGGTATAGCTAGCTACTCTTCTTTCGTTCACATATCTTGAATAATGGTAGGCCTTCT
CTACTCATTTTTTGTGTTTTTGTTTTATTTTTTTATTTACTCTATATCTCTCTTTTTATTTTTTCTAGGCTGTA
GCAATAGTGGTAAAAGCTCTTTAGGGAGTCAAAGGTTTAGTTTACTAGGTTTAATTGGGGTTCTTATAAT
AATAGGGGTTCCTCCTTTTCTGGGCTTTTTAAGGAAGTTATTAGTAATGTTATCTAGGCCTACTTTCGCTT
TGGTTGTTTGTCTATTAGGTTCTGTTGTAAGGCTTAAGTTTTATACTTCTTTCTTTTATAGTATGTTTTTAAA
CGGTTTGTATGGAATTGAATCTGTAGTGTCGCTTAGCTTAGTATTAAATGTTTTAGGATTATTTTTAATTGT
GGTAGTGCTTTTTCTGAGGTTTTTAGGCGTTGAGATGGTTTTCTTGTTAATGCTTAGATTTAGGTTACTGC
CGTTGTTTATACTTTCTTCCAAATCTGATTATAGGCGTGAAAAACTCTCTCCTTATGAGTGTGGGTTTGAG
CCTGTATTCAGTGCTCGAACTAGATTTTCTGTTCGGTTTTTTTTAGTCGCTGTCTTATTTGTGGTGTTTGAT
GTTGAACTGTCTATCTTAGTAGCAACAATTTTTTCTATCAGGCTTATAAAAAACTTAATCAGACTAGTGAG
TGTTATTGTTTTTCTAGTTGTTCTTTTTTTAGGGTTGTTTCATGAGTTTCGTGAAGGTTCGTTAAATTGAAT
TTACTAAGTGGTATTGTGGTTATTAAGTATATTTCTGGTGTTAATAGTAGTATTGCAACGCGAGTTAGTGGT
TAGGGCACTACTAATAAGGTTTTATTTGGCACTCTATCTCTGTGTAGTACCAGATAAAGTGTGGATACTTC
ATGGTGTTATAGTCTGAGATTTTTTGAGYTTAAGAATATCTCTTTTAGGGGTATTTGTAGTGGCAATAGGG
GTATTAAGAACAAAGGTGAGCGTGCGGAACAAGCTATTTCTATTGGTAAATATGTTTTTATGTATAGTTGT
AGTAATAGCGTTTAGCGTAAGAAGTTTTTTTCTTTTTTTTTTTTTTTTGAGAGCGACTCTGATTCCTTTAAT
TTTAATAATCGTAGGCTGGCGTTTACAAGCTGGGGTGTACATACTAATTTATACTGTAGTAGGTTCTCTATT
TTTTCTTTTAGGGGTTTGTTTTATATATTTTAATAGAAGAGACTGTATAATCTTAAGAAGGTCATGTGTTAA
AAATATGTGGAGGTTTTGATGAGTATTCTTGTTTGGGTTCTTGGTTAAAATACCTTCTTATCCCTTTCATTT
ATGACTTCTAAAAGCACATGTGGAGGCACCTGTAGCTGGTTCTATAGTGTTAGCAGGGGTGATTCTTAAA
TTTGGGGGGTATGGCATAATCCGATTGTTTAGGGTTATTAATGTCTGTTATTACGATAGGTTATGTCTGATT
GTAGTCGTAAGCCTATTAGGGGGGGTTTTACAGAAGCGTTGTTTGTGTTCGTCAGACTGATTTAAAACGCC
TGGTGGCTTACTCCTCTGTTAGACATATAAGAATAGTAATTCTATGTTTTAGAAATTCTTTTCTAGGGTTAG
AAGGGGTTATTTGGCTAATGTTAGGGCATGGCTTGTGTTCTTCTGGGTTATTCATGTTAGTGTCAGTAGTT
TACTCTAGGAGGGAGTCTCGAGTTCTTCTTCTTAATAAAGGAGGGTTGGTTAAAGCACCCTGTTTAGCA
ATAGTTAGATTTCTTCTTTGTGTTAGAAATATGGCGGCGCCCCCTAGGTTAAACCTGTTGGCAGAGATTT
GCTTATATATTTGCATCAGTTCTATATATTACTATATGGTGCTGTTTTTTGCTATAGTTAGGTTTTTTAGGGCT
GCCTATAGGCTTTATTTGTATACCAGTTGTTTTCATGGTAGCCTGAGAGGAGGTCTATTTAGAGTTCTAGG
GCATAGAGATACTATCGTTTTAATATGTCACTGAATCCCCTTAAATATGCTGGTTATTATATTATATTAGATA
CATAAGTACTTTTTATTGAGATTGTTTATTAGTATAGAGGTTGTAATCTTAGGTCTTGTTGTTTTATGTGTT
GCTGTAAAATTTTTTTATGTAGCTTCATTAAAATTAGTGCTGTGTCTTGCAGTGTGTGATGCAGGGGTATT
TTTGGCGTTAATTGTTTCACTAATACGGCGTGTTGGAAGAGACAGTGTTAGAAGAATATCTTCTTTTAAttt
aaaATATTGAATCATTTTATTTTTTTTGCGCTCATTATTGGGTATACTTTTATTTTAGTTGGAAGAGTCTCTAG
CGTTTATTTAATTGAGATAGTGTTTTGAGAGTTAGGAAGAGTAAGGATAGTGTTTAGGGTCTTAGTGGAT
TATATAAGGCTAGTTTTTATTGGGACTGTAATAGTAATTGGTGGAAGAGTTTTAATTTACACCAGATGGTA
CATATCAGATGAGCTTTATTACAAACGATTTGTTAGTTTAGTGTTACTATTTATTCTTTCAATAGTGCTAAT
AATCCTTATCCCTAACTTAATTGGGATTTTAATCGGATGAGATGGTTTGGGTTTAACATCCTTTTTATTGGT
GTGTTATTATTGTAACAGAAAATCGCTCGCTGCTAGACTACTGACGGCCCTTACTAATCGGATTGGCGAT
GTGCTGATTTTAATGAGAATTGGTTTTATAATTGTGGAGAACTCCTGGTCTTTATATCAATTTACTTTTGGT
GAGACAATTTCGTTAGTGGGCTTACTGACTTTTGCTGCAATAACTAAGAGTGCACAGATGCCTTTTTGTG
CTTGACTACCTGCTGCTATAGCGGCTCCAACGCCTGTTTCTGCATTAGTACATTCTTCTACTTTAGTTACG
GCAGGCGTGTATTTACTTATTCGTTCGTTTAAGGTAATCTCACTGGATTTTATAGAGGTGTTAGAAGTCTT

AAGGCTGGTTACTCTTTGTTTGGCTGGGACTAGTGCGTTAGTGTGTTTGGATCTCAAAAAAGTAGTGGC
ATTATCTACTTTAAGTCAGCTGAGCGTTATAATACTAAGAATTTCCTTGTATGCGCCTGTTCTTGCCTTTTT
TCATTTAATTACTCATGCGTTATTTAAGGCACTCCTTTTTTTATCGGTAGGGTCTGTAATCCACTCTTATGG
TAATATCCAAGATATCCGAATAGTTGGAGGCTGTTGGAGTACATTACCCAAAAGAATAAGGGCTATAGTA
ATCGCTGTTAGTTCTCTATCGGGGCTACCTTTTTTGAGTGGCTTCTTCTCTAAAGATCTGATTGTAGATTG
CTATAGGAGTAGCAGTCTAATAGTGGTTTTAGTAGGGGTTGGAATTGCTATAACAAGAGTCTATAGTCTAC
GAATTTGAAGAAGTTTGTTTAGATTAAATATAGGCGTGAGTCCTGTTATAAGAAGAGATGAAAAGTTAGA
ACTCACTTTTCCTTATCTGTGTTTAAGGTTAGGAGCCCTATTTGTGGGCTATGTATTAAGAGGGCAAATTA
TAGGCGTTTCTCAGTTTAGAGGTTCCAGAAATTTTGAGATTGTGGTACTAGTGGGCGCATTTTTGGGTTT
ATTTATATGGGCAAACAGGGGAAGGTTACAATTTAATCTTAAAAAATTTCCTTTTTTGTTAAGAATATGGT
GCTTAGAAGTTAGACAAGTTTTGGCAGGTGTTAGGCTTTCATTGTCAGAAAAACTGTCATCGAGCTTAG
ACAATGGGTGGTTAGAGACAATTGGCCCACAAAGAAACTTAATGAGTGTATCACAGAATAATGAGGGGT
TCTTTATGAGTTGCTTAGTGGTGAGTTGTGTTTTGGTTTTATTCTTCTTTGTAGCGTTGGAGTTGTTGTTA
GTTTCAGCCGTTTTGTTTTTTTTTATAATTATTTCTTTTAGTTTCCCTTATGCTTTAGCCATATTTCTTATTAT
TCTTTCGTTATCTATTTGTTTTTTAGTAAGATGATTTTACAGAAGGTTAATAGGGTTGCTGATTTTCATAAT
TTATGTAGGGGGTGTCTTAATCATATTTCTTTACAGCCTTAGAGTGCTACCTAATGAAAGATTTTACTCAG
ACTATGGGTTCTTCTATTTTTTGTGTTGGGTGCTTTTTGTTATGTCTATTTTTAATTATGAAATGTCCTTTCA
TTATATAAGGTTAGTCAGTTTTGGGGGTATATTTGTCTTTATGGCGTTAGTTTTATTTTTCCTTATACTGGTA
GTTTGTAACTTATGTGATAAAAAGCGTATCCCATTACGGAAGCTT"/>
<sequence id="seq_Bathymodiolus_childressiNC059707" spec="Sequence"
taxon="Bathymodiolus_childressiNC059707" totalcount="4"
value="GTGCTATTTGATTTGCTATCTGGTTTTGACtctttTCTTATAATATAGGATGGTCATCTTTTTTGCGTatc
TTAAGTTTCTTCTTTATTTTGATCTTGGTTAGTACTTTGTTTTTACTTTATGCCAAGGGAGCAGCTGTTAGT
TGGAGTGTGTTTAAAACTTCAAACGGTATGAATTTATCAGGATTCCCACTAGTTGTTTCTTCTCTCTTCAT
TTTTCTAATGAGGGTAAATATAATGGGTTTGGTACCTTTTTCTTTCAGTGTAACGTCCCATTTAAGTTTGG
GCCCAAACTTAGCCTTTTTAATGTGGGGCTCTTTATGAGTCTCAGGCCATCGAGTTAGATCTAAGCAAAA
TTTAACTAGACTGGTGCCAAGGTTTCCAATGTTTTTAGTTCCTTTTTTGCTATTAGTTGAAATTGTCACCA
TTAGTTCTCGCCCGATTACTCTTGGTTTTCGATTAATAATTAATATTATTGCGGGGCATTTAATCTTGTCTAT
GGGAACTAATGTCAATAGGTTTATTTCTTTAGGCCCTTTTTATGGGGCAGTTTACTTTTCTTTATTCGCAAT
AGGGCTGGCGGCGGAATTAGCCGTAGGTTTAATTCAAGCTTTCATTTTTTGCTCTTTATTGAGTCTTTATA
CTAATGACCACGCCAATTAATGATCGGGAATAATTGGTACAAGTTTGAGGATGCTGATTCGTATTGAGCT
AGCTCGTCCTGGTAGCggaTTTTTAGGTGATGACCAGCTTTATAATGTTGTAACTCATGCATTGGTTATAAT
TTTTTTTATAATGCCTTTGATGGTGGGCGGTTTCGGAAATTGACTACTTCCTTTAATGATAGGTTCAATTG
ACATAATTTTTCCTCGTTTAAATAATCTAAGGTTTTGGTTTTTGCCAGCATCTCTTTTTACTCTTTTGTTGT
CTACATTTATTGAGAGAGGCGCTGGGACTGGTTGAACCCTGTATCCTCCGCTATCTTCTTATACTGGGCA
CAGTGGTCCAGCTGTGGACATGTCTTTATTTTCTTTACATTTAGCAGGTGCTTCCTCTATTGGTGGTTCAA
TTAATTTTTTGACAAGGATGAAGAATATGTCTGTTGAAAGAATGCGAGGGGAGCGGATAGTTCTATTTGT
TTGATCCATGGCTGTGACAGCAGTCTTATTATTAGTTTCTTTACCTGTATTAGCAGGAGGAATCACCATGT
TGATTTTTGACCGCCATTTCAATACTTCTTTTTATGACCCTAGAGGTGGTGGAGATCCAGTTTTATACCAG
CATCTGTTTTGGTTTTTTGGGCATCCAGAAGTTTACGTTCTTATCTTACCAGGGTTTGGAATAGTATCTCA
TGTAGTTGCTCATTGTGCAGGAAAGGATGAAGTGTTTGGTGTGTTAGGAATAGTTTATGCTATGGTTTGT
ATCGGTGTTCTGGGGTTTATTGTTTGAGGGCACCATATATTTACTGTAGGAATAGATGTAGACTCTCGAGC
TTATTTTACTTCAGCTACAATAATTATTGCTGTTCCAACTGGAGTCAAAGTGTTTAGTTGACTGGCTACTT
TAAACGGTGGAAATTTATTGCATGAGCCAGCACTTTATTGGGCTGTTGGTTTCATTTTTCTCTTTACTGTT
GGGGGGCTTACTGGTATTATGTTATCAAATTCTTCTCTTGATGTGGCGTTGCATGATACTTATTATGTTACT
GCTCATTTTCATTATGTTCTCTCTATAGGGGCAGTATTTGCTTTATTTTGTGGTTTCTTTCATTGATTTCCTC
TATTCTATGGGTACTGTTATCATGAACGTTGAAGTAAGGCCCACTTTTTTATCATGTTCATCGGTGTAAATA
TTACTTTTTTTCCTCAACATTTTTTAGGTCTAAGCGGAATGCCCCGCCGCTATTCTGATTATCCAGATTGTT
TTATAAAATGGCATGTAGTGTCCTCTTTAGGGTCATGGTTGAGATTTGTTAGTGTTTTATACTTTCTTTTTA
TTGTGTGGGAAGCTTTGGTAAGACAACGAGGTGTTGTGTGCAAAAGAAATCGACCCGGGGCTATTGAA
TGAAGGGAATGGTGTTGTCCTTTGATGCCATTATGAGGTAGATTTAGGTTTCAAGATTGTTATTATCATATT
GGGGAGTATCTTGGTTTGTTTCATGAGGGTGTGATGTGCCTTATGATTTTTATTTTGTCTGTTGTGTTGTAT
GGAGTAGGTTGGGTTTTCACCTCGGGAAGAAGGTTTCGTTATTTACGTGAGGCACAAGCAGTAGAAAC
AGCATGAACTATTATTCCTAGGGTGTGTTTAGTCTGTGTGGCCATCCCTTCTATACATTTATTGTATGTAAT
AGATGAAATTGGTAGCCCTAAGTTCTGTTTTAAAGCAATTGGTCATCAGTGATTTTGGTCTTATGAAATG
GAGGACGTACTAGGATTTGATTCTTTTATGGAGCGGGAAATAGACAGTGGTTATCGATTATTGGATGTAG
ACCAACGGATGGTTGCGCCGGCTAATACCGGAATTCGGTGTATGGTAAGAAGGGCGGATGTTATTCATTC
CTTTGCCCTCCCGGGCTGCATACTAAAAGTAGACGCGATTCCAGGCCGTGTTAACGAAGTTCCTATGACT
GTAAACATGTGCGGGGTTCTTTATGGCCAGTGCTCTGAGATTTGTGGTGCAAACCATAGGTTTATGCCCA
TTGTGATTGAGTTTATTCATCCGCGGGTGTATAATTTATGGGTGGAATCTTTTGACATC-----------------------
ATGCCACGTAATGCTTTTTATTTGGTTGGGCCAAGTCCGTGGCCTGTTTTTACTTCTATAGGAGCTTTTTG
TATGGCAGTAGGTTTTGTTTCTTGGTTCCATAAGCATGGTTACGCCCTTTTATTAGGTGTAGTTGTGTTAAT
ACTTTCTTTAAGACAGTGGTGACGTGATGTTATGCGTGAGGGTGATTTAGGTTTTCACACTTCATATGTT

GTTAAAGGGTTGCGGGACGGATTTATTCTTTTTTTGGTTTCAGAGATTATATTTTTCTTTTCTTTATTTTGA
GCTTTTTTCCATATAAGTTTGGCTCCTGACATTTCAATGGGATGCATGTGACCTCCTAAGGGTTTGGAAA
CCTTAGACCCTATAAAGGTTCCATTATGCGGAACAACGGTGTTAGTTGGTTCTGGTGCATCTCTTATGTAT
TCTCACGCTGCGATTCGTGCAGGTTTAAATACTCATGCAGTTTTAGGGACTTTTTATACTATTGTGCTAGG
CTTGATTTTTACACGATTACAAGGATACGAATACTATTGAGCTAGGTTTACTATTGCTGACAGTGTTTATG
GGAGTTTGTTTTATATTATGACTGGGTTTCATGGCCTTCATGTTATATTCGGGACAGGCTTTTTACTTGTAA
GCTTAGTGCGTTTAATGCGTAATCGGTTCACCCCCCCGCAACCATTTTGGGTTTATGGTGTGTTCTTGATAT
TGACATTTTGTTGATGTTGTTTGAATTGGCTTGTATTTAGTTGTTTATTGCTGGGGTAGTTAACCCTTACGT
AAGCAGGATAGAGTAATAAAAATTTTAAATAATAGGTTGTATGATTTACCCGCGCCTGTCAATTTAAGGG
TCTGATGGAATTTTGGGTCTTTGTTGGGTTTATGCCTGGTTATTCAAATTGTTACAGGTTTTATCTTAAGCC
TCCATTATACGGCCCACACAAATATGTCATTTGATGCGCTTATTCATGTGATTCGGGATGTGAACAATGGT
TGGCTAATTCGTGGTATACATGCTAATGGTGCATCTCTTTTTTTTGTTTGTGCTTACTTTCATATTGGCCGT
GGCTTGTATTATGGGTCTTACAAGTCCCGGATGGTATGAAATGTGGGGGTAATTTTGCTTTTCCTTCTCAT
GGCTACTGCTTTTTTAGGTTATGTTCTTCCTTGAGGGCAGATGTCTTATTGAGGGGCAACTGTTATTACAA
AATTAGTAACTGCCGTGCCTTATGTCGGTGACATAATTTTGTATTGAATTTGGGGTGGTTATACAGTTTGT
AACGCTACTTTGGTGCGATTTTATTCTTTTCATTTTATTCTCCCATTTATTATAATTGTTTTTAGTGTTCTTC
ATTTGTTTTACTTGCATGAAGAAGGGGCTAACAACCCGTTGGGGGTCAGTGCAGATGCGGTTTTGGTGC
GATTTCATCCTTTCTACACATATAAGGATGCAGTAGGTTTCTTCATTTTATTTTTTGGTCTTTTACTATTAGT
TTGTTATTTTCCGGACCTTTTAGGAAATGTAAACAATTGGATTCCCGCGGACTCAATAAAGACCCCCCTT
CAAATTGAACCAGAGTGGTATTTTTTATTTGCTTATTCTATTCTTCGTTCAATTCCCAATAAAGTTGGGGG
GGCAACCGCCTTGGTGGTATCAGTTTTAATTTTATTTCTTATTCCTAGGCTGCACACAGGTCAGTTTCGTT
CCAACAGGTTCTATCCTTTAGGCCAACTTTTCTTTTGGGCTTTAGTTGCAGCTTGAGTGGGCTTAACATG
AGTTGGGGCTTGTCCGGTACAGCACCCCTATGATTCTTTGGGCTGCTGTTTTACTTTCCTTTATTTCTTTT
TTATTGTACTGATTCCTATAAGTCAAGGTGTGTGGGACGTGTTAATTAAAATGCAAGTTATCAGCTTTATT
TTACCAGGGGTTTTTGGTTTATTAGCAGTAGGGTGATTTACATTAGTTGAGCGTAAGGTGTTGGGTTATAT
TATGACTCGTAAAGGCCCCAATAAAGTGGGGTTTTTAGGGCTAATACAGCCAATAAGTGATGGGGCTAA
ACTATTTTCTAAAGAAATGCTTGTGCCAATATATAGGAATTTTGTCCCATTCCTGGTGTGCCCTGTGGTGA
CGTTTTTTATTGCCTTGTTGCTGTGATTGCTATATCCATTTCACTCTTCAGAGGGTGTGTTTACGTGTGGG
GTATTATTCTATCTGGCAAATTCTGGGACTACTGTTTATGGGGTGATGGTGGCAGGATGATCATCAAATTC
AAAATATGCCCTTTTAGGGACCATGCGGGCTATGGCACAAAGTATTTCATACGAAGTGAGAATAGCATTA
GTGCTGTTAAGATGTGTTTTCATGTCGGGGTCAATGCATCTTCAATCCGTGAAGTTGTTGTTTATCATGGG
TGCAATTTTACCTTTTTTTGTGGTGTGATTAATTTCTATGTTAGCAGAAACTAATCGTGCGCCATTTGATTT
TGTAGAGGGCGAATCAGAGCTGGTTTCAGGTTTTAACGTGGAGTATAGTAGTGGGGGTTTTGCCCTTATT
TACATAGCAGAGTACTCTAATATGTTGTTTAACAGCTTGTTCACATGTGTGATATTTTTGGGTACAAGGGA
CGCAATAATGGTTGGACAGGCCTGAATTTTTCTTTTTTTTTTCTTGTGAGCTCGTGGGACTTTTCCTCGTT
TTCGATATGACATACTAATAGGATTGGCTTGAAAGACTTTTTTATGTATTGTTTTAGGGTTGAGTTTATTTA
CTACTATAATAATTTATACTTTataATTAGGCGGTTTGAGGGCCCTTTAAGAGTTTTGGGGGGTTTTACTTTA
ATTTCTGGTTTGATCATAGTAGTCTCAACTGATAGGCACCTAGCTGCATGGGTTGGGATAGAAGTAAATAT
GCTTGGGTTTATGTGCCTTTTGAGTGTTAAATCCGTGTTAAACATGCGTGTCTTGATTAATTATTTTGTTTT
TCAGAGACTTGGCTCGACTATATATTTGTTTGGGTCAAGTGTTATTTTGTTTAATATTGCTTTAGGGTATTT
TTTAATTCATCTGGGACTTCTTTGCAAAGCTGGTCTTTTTCCTTTTTGGGTGTGGGTTCCTTCTGTAGTCA
ACTCTAGGGGCTGATTTGTTAGCTATCTATTATTAGGGATTCAAAAAGTTGGGCCTTTATTTTTGGGTGTG
TGGTCCCCTTGCAGAGAGTTTTTATATTTTGTAATTTTTGCTATAAGGGCTTTGGGCGGTTTAGGCGGGTG
TATCCAGAATTCTTATCGTGATGTATTGGTGTATTCTTCTTTTGTCCATAGTGCGTGAATGCTGGTATGTTT
AATTGAGTCTCAGAATTTATTTCTTTTTTATGTTGGAGCTTATCTTGTACAATTAGGTTTAGTTGTTTACTA
CTTGTGGCGCAGAAATGCAAGCTCTGTAAAGAGAGGGAAATGATCCCTTATAGTAGCAAGGTGTTTAAT
AAGCTTGAGGGGGTTGCCCCCATTAGCCGGGTTTGTGGTGAAGATAACTGTTGTTTTTTGCGTTGACAA
ATTAATTTTGTTATTTACATTAGCTGGTTCTATTACGGCCATATTTTATTATTTGAAAGCTGCAAATAGCTCC
ATTGTTAAATTATTAAATGATGGGGGCGTTTTTCTCTCTTTTACTTTTGGTTTGTATGCTTGAATTTATTTAT
TGGGGTGCTTGCTTTTGTAATAATCTGTATCTATCCTTTGGTTGGTTGTGATGATGGGTGTTTGTtttatgttatcta
taatgCTCATGTTATCTATGACGGGGCAGTGGAGTCGTGAAAAGGTTTCCCCCTATGAGTGCGGGTTTGATC
CGATTCTTAGGGCCCGTAGGAGGTTTTCTTTGCGCTTTTTTCTGCTTGGCGTTTTGTTTTTAGTGTTTGAT
GTGGAAGTGGTTATGGTTGTTCCACTTTTGTTTGTATTGTACGGGGGGGTGAAGGTAGTGGGCCTTGTGT
GTTTAGTAGGGTTCTTACATGTTCTTACTATTGGGTGCCTCTATGAGCGTCGCGACGGGTCGATGGATTG
GATTAGTGAGGTGGTTATTGGTCTTGTTATATGTATACTTTTTTTAGGCTTGCTAGGTAGCTTAGAGTTATC
AATTGCTGGCTTGTCTTTACTAGGTGCAATAgcgATGTTATCTTTATTTTTATGCGCTCAATACTCTGAGTTA
ATTGGATTTTTTAATTTGGACTTTCCAGGGCAGGCCTTAGTAGTTCTTAGAATTTATATTAGATTTTTAATG
GTGTTGGGCAGGGCCGCGGTTTCCCGTTTTAGTGCATTTAATAGACTTATTACTTGTATCGGTGTTTTGTT
AGTTTGTGCTTTTAGTGTGAGGGCCACTTTCTTCTTCTTCGTTTTTTTTGAGGGGGTTTTGTTTCCTACTT
TAATGTTGATTGTAGGATGGCGGTTACAAGCAGTTGTTTATATAGTTATTTATACTGTTATAGGGTCACTCC
CACTTTTGTACGGGTTTGGGAAGTTATACTTTAATTACAGTGATAATTTATTTTTGGAGTACTTTTTGGATA
AAAGAATTTTGAGTTTAGTTGGCTTTATTTGTTAGCGTTTTTAGTTAAGCTTCCAGTTTCCCCTTGCAT
TTATGATTGCCTAAGGCTCATGTAGAGGCTCCAGTTGCCGGTTCTATAATTTTAGCAGGCCTTTTGCTTAA

GTTAGGGGGTTACGGGATTATTCGTTTAAGAGGCCTTCTTGTTATCAAAAGAATTAGAATAGCTATACTGG
TAGTAATAGTTAGGCTTTTTGGAGGGATTCTTACAAGTATGGTTTGTTTACGTCAGACAGATTTTAAATCC
TTAGTGGCTTATTCCTCTGTTGGACATATAAGTTTTGTTTTAGTCTTATTGAGTAATACTTTATGGGGGCTT
ATAGGGGCGATGTTGGTTATAATCGGGCATGGGCTGTGCTCTTCTGGTTTATTTTCTTTGGTTAATGTTTTT
TATCTTAGAAGTCGCTCTCGTTTGTTGGCAATAAATAAGGGATTTTTAATACTTAGCCCTTGTGTCGCTTT
GTTGTGTTTTCTGTTGAGGGTCAGAAATATAGCCAGCCCACCTAGTTTGAACTTATTTGGAGAACTTTTT
ATTTATATAGCGGGTGCTTCTATAAATATACTGTTTTTTCCTTTATTAATGTTGTTGAGATTTTTGAGCGCTT
GCTATAGGCTTTATATTTATATCAGCTCCCAACACGGCAAATGTATAAATAGACTAGACTCACGAATAAGT
ATAATAGACATTTCGGTGTTGACTTTTCATTGAATGCCTTTAAATTTTTTGTTTGTAGTTATTCCATAGGTG
CGCAGAATACATTTGATTACGATTTTCTTAGGGATGGAGTTTAGAGCGCTTGGCCTGTTTATCGTTGGGTC
TTTATCGGCGTACAATAGTCTGTTTATTCTACTACTTGTAATTTGTACTAGGGTGTCTGAAGCAGCTATTAT
GCTGTCTTTGATGGTTATAATGACTCGTTTGTACGGAAATGATCGATGTTTGAGTTTGATGACTGATAATT
TAAAGTTAATGAAGTTTTAGGTTGTATTTTTATGGTTTTAGGATGGGGTGTAATAAGTATAATCCCCTTTAG
CTCTTTAGTTCTTGTTGACTATAGAATTTGATTATCTAGAAATTTGTCGGTGAATTTAACATTGCTGTTAGA
TCAAGTTAGAGGGTTGTTCATAGCTACGATTTTTTTGATTTCGGGGTCTGTTTTAATTTACTGTTCTTGGT
ATATGGCCGATGAAATTTATTTTTTGCGTTTTATTTATCTTGTTGTATTTTTCGTTCTATCTATGATATCCCTA
ATTATAATTCCTAATTTAATTGCTCTTCTGCTGGGATGAGATGGGCTTGGTATTACCTCTTTTTTATTGGTG
GTTTATTACCAAAATAATAAGTCTTTGGGGGCAGGCATAGTCACAGCTTTGACTAATCGAGTTGGAGATG
CAATCCTTTTATGTATCATTGGCGTATTTGCTAGGGAGGGAGGATGAATCCTATTTGAGTTTTTACCTAGA
ATAAGGTATTTTGTCCCTTTTCTTTTAGTTATAGCTGCTGTTACTAAAAGGGCCCAAGTGCCCTATTCGGC
GTGGTTGCCTGCTGCAATAGCTGCCCCCACCCCTGTTTCTGCACTAGTTCATTCCTCTACTTTGGTCACA
GCTGGGGTTTACCTTTTGATTCGTTCTTACCCCCTTCTTTCTCAAAGATCTTTAAGGGTGTTAAAGTGTCT
TAGACTTTTCACTTTACTAATGGCTGGGAGGGTGGCCCTTGTTGAGGTGGATTTAAAAAAGATTGTAGCA
TTATCTACTTTAAGCCAATTGAGAATAATATTATTCGCTATTTCAATTTGTTTACCAAAAGTGGCTTTTTTC
CATCTTATCACTCATGCTATGTTTAAATCTCTTTTGTTTTTAAGTACCGGGGTTGTGATTCACACAAGGTT
AAAATGGCAGGATATTCGCTTTTTAGGGAAAAACTGAGCTCGTTTGCCCGTTAGAATGAGTTGTGTCAC
TGCCGCGAGTCTTTCTTTGTGCGGGATGCCTTTTTTGAGAGGGTTCTATTCAAAAGATTTGATTATTGAAT
TTCAAGGCGGGGATTTGGTTATTTATTTTATACTAATTGTTGGAACTTTATTTACCTCTTGGTACTCCCTTC
GTATATTTAATTTGTTTTTGAGTAGAAATAAGAGTGTTAGTCCTGTGGTTCACGCAGAAGAAGCCAGGAG
TGTTAAATTTGCTTACAGGGGCTTATTGATTAGCTCTGTGATTATAGGGTTTTTATTAGTTAACTTAGTCTC
AGACCTTGATCTCACGGCTATGGTGAGTAATGTGGAAAAGTTTGTTGTAATATTACTAGTGGTGGCTATTA
TCATTGAGGTTTTTGCAGGATGAAAACGGAAAACCAAAGCGGGTTATGGCTATTTGGCTAGTATGTGGC
ATTTTAAACTGTTACAATTTAGCCCTAGTGCGGGTTTAAAGCTAGCTAGTCTTATTACAGTTAGAATGGAG
AAAGGATGGTTAGAAAAAGTTGGGCCCCAAGGAAGAGTTCAAACGTTGGGTTTAATTAATCAGTACCAT
TTTTTATTTGTGTTGCTCTTTAGGTTATTTATTGTAATTATTTTGTTCTTGTAATTGTTAGAAACATTCTTCT
TTTTCATTTCCTGTTTTGTATGCATGTTGAGGATCGGGGAGCCTTTGTTTATGGGTTTGGCTTTACTTGTT
GTATCCCTTATTATTTCAATTTTGTTGGGGTTAGAAATAGGGAGTCTTGTGGGTTATTTCGTTTTTTTAATC
TATATTGGTGGATTAATAGTTTTGTTTGGATATGTGCTTAGAATTTTCCCAAATCAGCGTTTTGGGGCCCCT
GTATTAGTTGTTAAACTATTATTATTTTTATTAGGATTTGTTTCAGTTTCTCATAAAAATAAAAGAGATCAC
TTTTTGAGTTGGGGGGATTTTGGCTCGATATACCTGTTTGTTGCGTTTCTTCTTCTTTTTGTATTGTTGTTA
GTTGTTGCTTTTTGTAAGAAGCGTCGTATACCACTCCGCACGGTG"/>
<sequence id="seq_Bathymodiolus_japonicusAP014560" spec="Sequence"
taxon="Bathymodiolus_japonicusAP014560" totalcount="4"
value="GTGTTGTTAGATTTGTTATCAGGTTTCGACtctttTCTTATAATCTAAGATGATCGTCATTTTTACGTatc
TTAAGTTTTTATTTTATCTTGATCTTGGCCAGCACTTTGTTTTTACCTTTTACCAAGGGGTTAGCTGTTAGT
TGGAGTATGTTCAAAAGTTCAACTGGCATGAATTTATCAGGATTTCCATTAATTGTTTCTTCCCTATTTATT
TTTTTAATAAGAGTGAATATAATGGGTTTGGTGCCTTTCTCTTTTAGTGTGACGTCTCATTTAAGTTTGGG
CCCAGCTTTAGCTTTTTTAATGTGGGGCTCTTTATGAGTCTCAGGACATCGAGTTAGATCTAAGCAAAAT
TTAACTAGGTTGGTGCCAAGGTTTCCAATGTTTTTAGTTCCTTTTTTGTTATTAGTTGAAATTGTTACTATT
AGTTCTCGTCCGATTACGCTTGGCTTTCGATTAATAATTAATATTATTGCGGGGCATTTAATCTTGTCTATG
GGATCTAATGTTAATAGATTCATTTCTTTAGGTCCTTTTTTTGGTGCAGTTTATTTTTCTTTATTTGCAATAG
GATTGGCGGCGGAATTAGCTGTAGGCCTAATTCAAGCTTTCATTTTCTGCTCTTTATTGAGTCTTTATACT
AATGATCATGCCAATTAATGATCGGGAATAATTGGTACAAGTTTGAGTATGTTGATTCGTATTGAATTGGC
TCGTCCTGGGAGTggaTTTTTAGGTGATGATCAGCTTTATAATGTTGTAACTCATGCATTGGTTATAATTTTT
TTCATGATGCCTTTGATGGTGGGTGGTTTTGGAAATTGGCTACTTCCTTTGATAATAGGTTCAATTGACAT
AATCTTTCCTCGTTTAAATAATTTAAGGTTTTGGTTCTTACCAGCTTCTCTCTTTACTCTTTTGTTGTCTAC
TTTTATTGAGAGAGGTGCTGGAACTGGTTGAACTTTGTACCCACCCCTGTCTTCCTACACTGGACACAG
GGGCCCAGCTGTGGATATATCTTTATTTTCTTTACATTTAGCAGGTGCTTCTTCTATTGGTGGTTCAATTAA
TTTTTTAACTAGGATGAAGAATATGTCTGTAGAAAGAATACGAGGGGAGCGGATAGTTTTATTTGTTTGA
TCTATGGCTGTAACAGCAGTTTTACTATTAGTTTCTTTACCTGTATTAGCAGGAGGAATTACTATGCTGATT
TTTGATCGTCATTTCAATACTTCTTTTTACGACCCTAGAGGTGGTGGAGATCCAGTTTTATACCAACATTT
GTTTTGGTTTTTTGGGCACCCAGAAGTCTACGTTCTTATTTTACCAGGATTTGGAATAGTGTCTCATGTAG
TTGCTCATTGTGCAGGAAAGGATGAAGTGTTTGGTGTGCTAGGAATAGTTTATGCTATGGTTTGTATCGG

TGTCCTTGGGTTTATTGTTTGAGGCCATCATATATTCACTGTAGGAATAGATGTAGACTCTCGAGCTTATTT
TACTTCAGCTACAATAATTATTGCTGTGCCAACTGGAGTTAAAGTGTTTAGTTGATTGGCTACTTTAAATG
GAGGAAACTTGTTACATGAGCCTGCACTTTATTGGGCGGTTGGTTTCATCTTCCTTTTTACTGTTGGGGG
TCTTACTGGTATTATGTTATCAAATTCTTCTCTTGATGTGGCGTTGCATGACACTTATTATGTTACTGCTCAT
TTTCATTATGTCCTTTCTATAGGGGCAGTATTTGCCCTATTCTGTGGGTTTTTTCATTGATTTCCTTTATTCT
ATGGGTATTGTTACCATGAACGTTGAAGCAAAGCTCATTTTTTTATCATGTTTATTGGTGTAAATATCACTT
TTTTTCCTCAACATTTTTTAGGCTTAAGAGGAATACCTCGTCGTTATTCTGACTATCCAGATTGTTTTATAA
AATGGCATGTAGTGTCATCTTTAGGTTCATGATTGAGATTTGTTAGTGTTTTATACTTTCTTTTTATTGTGT
GGGAAGCTTTGGTAAGACAACGAGGTGTTGTTTGTAAAAGAAATCGACCTGGGGCTATTGAATGAAGG
GAGTGATGTTGTCCTTTGATGCCATTATGAGGTAGTTTTAGGTTTCAAGATTGTTATTATCATATTGGGGA
GTATCTTGGTTTGTTTCATGAGGGCGTGATGTGTCTTATAGTTTTCATTTTGTCTGTTGTGCTGTATGGAGT
AGGTTGGGTTTTTTCTTCGGGAAGAAGGTTTCGTTATTTACGTGAGGCACAAGCGGTAGAAACAGCATG
AACCATTATTCCTAGGGTGTGTTTAGTTTGTGTGGCTATCCCTTCTATACACTTATTGTATGTAATAGATGA
AATTGGTAACCCTAAATTCTGTTTTAAAGCAATTGGTCATCAATGGTTTTGATCGTATGAGATGGAAGATG
TGCTAGGATTTGATTCTTTTATGGAGCGGGAAATAGATAGGGGTTATCGGTTATTGGATGTAGACCAACG
GATGGTTGCGCCGGCTAATACCGGAATTCGGTGTATAGTAAGAAGGGCAGATGTCATTCATTCCTTTGCC
CTCCCTGGTTGCATATTAAAAGTAGACGCGATTCCAGGCCGTGTTAATGAAGTTCCTATAACTGTAAACA
TGTGTGGAGTTCTTTATGGTCAGTGTTCTGAGATTTGTGGAGCAAATCACAGGTTTATGCCCATTGTAATT
GAATTCATTCATCCGCGAGTGTACAATTTATGGGTAGAGTCTTTTGACATC------------------------
ATGCCACGTAATGCTTTTTATTTGGTCGGGCCAAGCCCGTGGCCTGTTTTTACTTCTATAGGAGCTTTTTG
TATGGCAGTAGGTTTTGTTTCTTGATTTCATAAGCATGGCTACGTTCTTTTATTAGGTGTGGTTGTGTTAAT
TCTTTCTTTAAGTCAATGGTGGCGTGATGTCATGCGTGAAGGTGATTTAGGTTTTCATACTTCATATGTTG
TTAAAGGTTTGCGGGATGGATTTATCCTTTTTTTGGTCTCAGAAATTATATTCTTCTTTTCTTTATTTTGAG
CTTTTTTCCACATAAGTTTAGCCCCTGATATTTCAATGGGGTGTATATGACCTCCCAAAGGTTTGGAGACT
TTAGACCCTATAAAAGTACCATTATGTGGAACTACAGTACTAGTTGGTTCTGGCGCGTCCCTTATGTATTC
TCATGCTGCTATTCGTGCAGGTTTTAATACTCACGCAGTTTTAGGAACTTTTTATACTATTGTGTTAGGTTT
GTTTTTTACACGATTACAAGGATACGAATATTATTGGGCTAGGTTTACTATCGCTGATAGTGCTTATGGGA
GTTTGTTTTATATTATGACCGGGTTTCATGGCCTTCATGTTATGTTTGGAACAGGGTTTTTACTTGTAAGCT
TAGTTCGTTTGATACGTAATCGGTTTACCCCACGCAACCATTTTGGGTTTATGGTGTGTTCTTGATACTGA
CATTTTGTTGATGTCGTTTGAATTGGTTTATATTTAGTTGTTTATTGTTGGGGTAGTTAACCTTTACGCAAA
CAGGATAGAGTAATAAAAATTTTAAATAATAGATTATATGATTTGCCTGCGCCTGTTAATTTAAGGGTTTG
ATGAAATTTTGGGTCTTTGTTAGGCTTATGCTTGGTTATTCAAATTGTTACAGGTTTTATTTTAAGCGTCCA
TTATACGGCCCACACAACTATGTCATTTGATTCGCTTATTCATGTGGTTCGTAACGTAAATAATGGTTGGT
TAATTCGTGGGATACATGCTAATGGTGCGTCTCTTTTCTTTGTTTGTGCTTATTTTCACATTGGTCGTGGTT
TGTATTATGGTTCTTATAAGTCTCGTATGGTGTGAAATGTAGGGGTAATTTTGCTTTTTCTTCTGATAGCTA
CTGCTTTTTTAGGTTATGTCCTCCCTTGAGGGCAGATGTCTTATTGAGGGGCAACTGTTATTACAAAACTA
GTAACTGCTGTGCCTTATGTTGGTAACATAATTTTGTATTGAATTTGGGGTGGTTTCACAGTTTGTAATGC
CACCTTGGTGCGATTTTATTCTTTTCATTTCACTCTTCCATTTGTTATAATTGTTTTTAGTGTTCTTCATTTG
CTTTACTTGCATGAAGAAGGGGCCAATAACCCGTTGGGGGTTAGTGCAGATGTGGCTTTGGTGCGGTTT
CATCCTTTTTACACATATAAGGATGCAGTAGGATTCTTTATTTTGTTTTTTGGTCTTTTATTTTTAGCTTGTT
ATTTTCCAGACCTTTTAGGAAATGTGAATAATTGAATTCCTGCAAATGTAATAAACACTCCCCTTCAAATT
GAGCCTGAGTGATACTTCTTATTTGCTTACTCTATTCTTCGTGCAATCCCCAATAAAACTGGCGGAGTTGC
TGCTTTGGTGGCATCAGTTTTAATTTTATTTCTTATTCCTAGGTTGCACACCGGCCAGTTCCGTTCCAATA
GGTTTTATCCTTTAGGTCAACTTTTCTTTTGGCTCTTTGTTTTAGTTTGAGTAGGTTTAACATGACTTGGG
GCTTGTCCGGTGCAGCACCCTTATGATTCTTTGGGGTACTTTTTTACTTTCCTCTATTTCTTTTTTATTATG
CTGATCCCTATAAGACAAGGCATGTGGGATTGGTTAATTAAAATGCAAGTTATCAGCTTTATTTTACCAGG
AGTTTTTGGTTTATTAGCAGTAGGATGATTTACATTAGTTGAGCGTAAAGTGTTAGGTTACATTATAACTC
GTAAAGGCCCCAATAAAGTGGGACTTTTAGGGTTAATGCAACCAATAAGTGATGGGGCTAAACTATTTTC
TAAAGAACTGGTTGTGCCAATATATAGGAATTTTGTTCCATTTCTGGTGTGTCCTGTGGTGACGTTCTTTA
TCGCCTTGTTGCTATGATTGCTTTACCCATTTCACTCTTCAGAAGGTATATTTATGTGCGGGGTATTATTTT
ATTTGGCAAATTCTGGAGCTAACGTTTATGGGGTAATGGTGGCAGGATGATCATCAAATTCAAAATATGC
CCTTTTAGGGGCCATGCGGGCTATGGCACAAAGTATTTCATACGAAGTGAGAATGGCATTAGTACTTTTA
AGATGTGTTTTTATATCAGGGTCAATACATCTTCAATCCGTGAAGTTGTTGTTTTTTATGGGTGCAATTCTA
CCTTTTTTTGTGGTGTGATTAATTTCTATGTTAGCAGAAACTAATCGTGCACCATTTGATTTTGTAGAGGG
CGAATCAGAGCTGGTGTCAGGTTTTAATGTGGAGTATAGTAGGGGCGGGTTCGCCCTTATTTTCATAGCA
GAATACTCTAATATGTTGTTCAATAGCTTGTTTACATGTGTAATATTTTTGGGCACAAGTGACGCAATAAT
GGTCGGGCAGGCATGAATTTTTCTTTTCTTTTTTTTGTGGGCTCGTGGAACTTTTCCTCGTTTTCGATACG
ATATGTTAATAAGGTTGGCTTGAAAGACTTTTTTATGTATTGTCTTGGGGTTGAGTTTATTTGTTTCTATAA
TTATTTATATTTataattaggcggtttgagggccctttaATAGTTTTGAGGGGTTTTACTCTAACTTTTGGTTTGATTATTA
TAGTTTCAACTGATAGCCACATGACTGCGTGGGTTGGGATAGAAGTAAATATGCTTGGGTTTATATGCCTT
TTGGGAATTAAATCAGTGTTAAATATGCGTGTTTTGATTAATTATTTTGTTTTTCAGAGACTTGGTTCAAC
TATGTATTTGTTCGGTTCAAGCGTTATTTTGTTTAATATTGCTTTAGGGTACTTTTTAATTCATTTAGGACTT
CTTTGCAAAGCTGGCCTTTTCCCTTTTTGGGTATGGGTTCCTTCTGTAGTAAATTCTAGGGGTTGACTTGT

TAGCTATCTATTATTAGGTATTCAAAAAGTTGGGCCTTTACTTTTGGGTGTGTGGTCTCCTTGTAGGGAAT
TTTTATATCTTGTAATTTTTGCCATAAGGGTTTTGGGTGGTTTAGGCGGGTCTATTCAGAACTCTTATCGTG
ATGTATTGGTGTATTCTTCTTTTGTTCATGGTGCGTGAATGTTGGTATGTTTAATTGAGTCTCAGAATTTAT
TCTTTTTTTATATTGGAGCTTATCTTGTACAATTAGGTTTAGTTGTTTACTATCTGTGGTGCAGAAATGCAA
ATTCTATAAAAAGAGGAAAATGGTCTCTTATAGTAGCAAGGTCTCTAATAAGTTTGAGGGGGTTACCTCC
ATTAGCTGGTTTTGTGGTAAAGATAGTTGTTGTTTTTTGCGTTGATAAATTAATTTTGTTATTTACATTAGC
TGGTTCTATTATGGCCATATTTTACTACTTGAAAGCGGCAAATAGCTCTATCGTTAAATTATTAGATGATGA
GGGCTGCTTTCTCTCTTTTACTTTTGGTTTATATGCTTGAATCTATTTATTGGGGTGTCTTTTCTTGTAATAA
TCTGTGTTTGTTTTTTGGCTGGTTGTGATAATGGGTGTTTGTTTTATGTTATCTATAATGctcatgttatctatgacgG
GGCAGTGGAGTCGTGAAAAGGTTTCCCCCTATGAGTGTGGGTTTGATCCAATTCTTAGGGCCCGTAGGA
GGTTTTCTTTGCGGTTTTTCTTACTTGGTGTGTTGTTTTTAGTGTTTGATGTGGAAGTGGTTATGGTTGTT
CCACTTTTGTTTGTATTGTACGGAGGGGCGGAGGTAGTGGGTGTTGTATGTTTAGTGGGGTTTTTACATG
TGCTTACTATTGGGTGTCTTTATGAGCGTCGTGATGGGTCCATGGATTGGGTTAGTGAAGTGATTATTGGT
CTTGTTATATGTATACTTTTTTTAGGTTTGTTGGGTAGATTAGAGTTATCAATTGCTGGATTGTCTTTATTGG
GCGCGATAgcgATGATATCTTTATTTTTGTGCGCTGAGTACTCTGAGTTAACTGGAGTTTTTAATTTGGATT
TCCCAGGGCAGGCTTTAGTAGTCCTTAGAATTTATATTAGATTTTTAATGGTGATGGGCAGGGCTATGGTT
TCTCGTTTTAGTAGGTTTAATAGCCTTATTATTTGTATTGGTGCTTTGTTAATTTGTGCCTTTAGTGTGAGT
GCCACTTTCTTCTTTTTCGTTCTTTTTGAGGGGGTTTTGTTCCCTACTTTACTGTTGGTTGTAGGGTGGCG
ATTGCAAGCAGTTGTTTATATGGTTATTTATACTGTTATAGGATCACTTCCACTTTTGTACGGGTTTGGGAG
TCTATATTTTAATTATAGTGATAATTTATTTTTGGAGTACTTTTTGGACAAAAGAATTTTGAGTTTTAGTTG
GCTTTATCTGTTAGCGTTTTTAGTTAAGCTCCCTGTTTTTCCTCTACATTTATGATTACCTAAGGCTCATGT
AGAGGCTCCAGTTGCTGGTTCTATGATTTTGGCAGGCCTTTTGCTTAAGTTAGGGGGTTACGGGGTTATT
CGTTTAAGAGGCCTTCTTATTATTAAAAGAATCAGAATAACTATATTAGTGGTAATAGTTAGGCTTTTTGG
AGGAATTCTTACAAGTATGGTTTGTTTACGTCAGACAGATTTTAAATCTTTAGTAGCTTATTCTTCTGTTG
GGCACATAAGTTTTGTTTTAGTTTTATTGAGTAATACTTTATGGGGGCTTATAGGGGCCATGCTAGTCATA
ATTGGGCATGGTTTATGTTCCTCTGGCTTATTTTCTTTGGTTAATACTTTCTATCTTAGAAGTCGTTCTCGC
TTGCTGGCAATAAATAAGGGGTATTTAATACTTAGCCCTTGTGTAGCTTTGTTGTGCTTTTTGCTGAGGGT
TAGAAATATAGCTAGCCCACCCAGGTTGAACCTATTTGGAGAACTTTTTATTTATATAGCAGGTGCTTCTA
TAAACATGTTGTTTTTCCCTTTATTAATGTTGTTGAGGTTTTTGAGTGCCTGCTATAGGCTTTATATTTATAT
TAGTTCCCAACATGGTAAATGTATGAATAGATTAGACTCACGTACAAGTATAAGAGATATTTCGGTGTTGG
CTTTTCATTGAATGCCTTTAAACTTTTTGTTTGTAGTTATTCCATAGGTGCGTAGAACACATTTAATTACGA
TCTTTCTAGGGATGGAGTTTAGTGCACTTGGCGTTTTTGTGATTGGATCTTTATCGGCGTACAACAGTTTA
TTTGTTTTATTAGTTGTAATCTGTATTAGGGTATCTGAAGCAGCCATTATGCTGTCTTTGATGGTCATAATA
ACTCGTATGTACGGAAACGATCGATGTCTGAATCTAATAACTGATAATTTAAAGTTAATGAAGTTTTAGGT
TGTATTTTTATGGTTTTAGGGTGGGGTACTATAAGTATAGTTCCCTTTAGCCCTTTAGTTCTTGTTGACTAT
AGAATTTGATTATCTAGAAATTTGTCAGTAAATTTGACATTATTGTTAGATCAAGTTAGGGGGTTATTTATA
GCTACCATTTTTTTGATTTCGGGGTCTGTTTTAGTTTATTGCTCTTGGTATATAGCGGATGAAATTTATTTTT
CGCGTTTCATTTATCTTGTTGTACTTTTTGTTTTATCTATAATATCCTTAATTATAATCCCTAATTTAATTGCT
CTTTTACTGGGATGAGATGGACTTGGTATTACCTCTTTTTTATTGGTGGTTTATTATCAAAATAATAAGTCT
TTGGGAGCAGGCATAGTTACGGCCCTGACTAATCGTGTCGGAGATTCAATCCTCTTATGTATTATTGGTGT
GTTTGCTAGGGAGGGAGGGTGAGTATTATTTGAGTTTTTACCTAGTATAGGGTATTTTGTCCCTTTTCTTT
TAGTAATAGCAGCTATTACTAAAAGAGCTCAAGTGCCTTATTCGGCGTGGTTGCCTGCTGCAATAGCTGC
CCCTACCCCTGTTTCTGCATTAGTTCACTCGTCTACTTTGGTTACAGCTGGGGTTTATCTTTTGATTCGTTC
CTACCCACTTCTTTCTCAAAGATCTTTAAGGGTGCTAAAGTGTCTCAGGCTTTTTACTTTACTAATAGCTG
GGAGGGTGGCTCTTGTTGAGGTGGATTTAAAAAAGATTGTAGCATTATCTACTTTAAGCCAGTTAAGAAT
AATATTATTTGCTATCTCAATTTGTCTACCAAAAGTAGCTTTTTTTCATCTTATTACCCATGCTATATTTAAG
TCTCTTTTGTTTCTAAGTGCAGGGGTTGTGATTCACAGAAGGTTAAAATGACAGGATATTCGTTTTTTAG
GAAAAAATTGAGCGCGGTTGCCAGTCAGGATGAGTTGTGTTACTGCCGCGAGTCTTTCTTTATGTGGCA
TACCCTTTCTGAGGGGGTTTACTCAAAAGACTTGATTATTGAATTTCAAGGTGGGGATTTGGTTGTTTA
CTTTATAGTAGTTGTTGGAACTTTATTTACCTCTTGATATTCCCTCCGTATATTTAATCTGTTTTTGAGTGCA
AATAAGAGTACTAGTCTTGTAACTCACGCGAAAGAAGATAGGAGAGTTAAACTAGCTTACAGGGGTTTA
TTGGTTAGATCTGTGATTATAGGTTTTTTATTAGTTAACTTAGTTTCAGACTTTGATCTCATGGCTATGGTG
AGTAATGTGGAAAAATTTGTTGTAATATCATTGGTGGTTGCTGCCATCATTGAGGTTTCTGCAGGATGAA
AAAGGAATAAAAAAGTAGGTTATGGTTATTTGGCTAGTATATGGCATTTTAAGCTGTTACAATTTAGTCCT
AGTGCAGGCTTAAAGTTAGCTAGTCTTATTACAGTTAGAATGGAGAAAGGGTGATTAGAAAAAATTGGC
CCTCAAGGAAGAGTTCAAACTTTAGGTTTTAGTAACCAATACCATTTTTTAACTGTGTTGCTTTTTAGGC
TATTTCTTGTGATTATTTTGTGCTTGTAGTTGTTAGAAACATTTCTCTTTTTTGTTTCTTGTTTGGTATGCAT
GTTAAGGATCAGAGAGCCTTTGTTTATGGGTTTGTCCCTATTCGTTGTATCTTTTATTATTTCAATTCTATTA
GGGCTGGAAATGGGTAGCCTTGTGGGTTATTTCGTTTTTTTAATTTATATTGGTGGATTAATAGTTTTGTTT
GGATATGTGCTTAGAATTTTTCCGAATCAGCGGTTTGGGGTCCCTGTATTGTTTGTCAAATTATTATCAGT
TCTATTAAGATTCGTTTTGGTTTTTCATAAAAATAAGGGAGACCAGTTTTTAAGTTTGGGGGATTTTGGCT
CGATATACCTGTTTGTTGCGTTTCTTCTTCTTTTTGTATTGTTGTTAGTTGTTGCTTTTTGTAAGAAGCGTC
GCATGCCTCTTCGCATGGTG"/>

<sequence id="seq_Bathymodiolus_securiformisNC0395" spec="Sequence"
taxon="Bathymodiolus_securiformisNC0395" totalcount="4"
value="GTGCTATTAGATTTGTTGTCAGGTTTTGACtcttttTCTTACAATATGAGTTGGTCATCTTTCTTACGTat
cTTAAGCTTTTATCTTATTTTGACTTTGGTTAGTATTTTGTTTTTATCTTTTACTAAGGGTTTAGCTGTTAGT
TGGAGTATGTTTAAAACTTCAACCGGCATGAGTTTATCAGGATTTCCATTAATTGTTTCTTCTTTGTTCATT
TTTTTAATAAGAGTAAATATAATGGGTTTGGTACCTTTTTCTTTTAGTGTGACGTCTCATTTAAGTTTAGGC
CCTGCCCTAGCTTTTTTAATGTGGGGTTCTTTGTGAATTTCAGGCCATCGAGTTAGCTCTAAGCAAAATTT
AACTAGGTTGGTACCAAGGTTTCCTATATTTTTAGTTCCTTTTTTGCTATTAGTTGAAATTGTTACCATTAG
TTCTCGCCCGATTACTCTTGGCTTTCGATTGATAATTAATATCATTGCGGGGCATTTAATTTTATCTATGGG
AACTAATGTTAATAGATTTATTTCTTTAGGCCCTTTTTATGGTGCGGTTTACTTTTCTTTATTTGCAATGGG
ATTAGCGGCGGAATTAGCTGTAGGGCTAATTCAAGCTTTTATTTTTTGCTCTTTATTGAGACTTTACACTA
ATGATCACGCCAATTAATGATCGGGAATAATTGGTACAAGTTTGAGGATGTTGATTCGTATTGAATTAGCT
CGTCCTGGGAGCggaTTTTTAGGAGATGACCAGCTTTATAATGTTGTAACTCATGCATTGGTTATGATTTTT
TTTATAATGCCTTTGATGGTGGGCGGTTTTGGAAATTGGCTACTTCCTTTAATAATAGGTTCAATTGACAT
AATTTTCCCTCGTTTAAATAATTTAAGGTTTTGGTTTTTGCCGGCGTCTCTTTTTACTCTTTTGTTATCTAC
TTTTATTGAGAGAGGCGCTGGAACTGGTTGAACTTTGTACCCCCCTTTATCTTCTTATACTGGTCATAGGG
GCCCAGCCGTGGATATGTCTTTATTTTCTTTACATTTGGCAGGTGCTTCTTCTATTGGTGGTTCAATTAATT
TTCTGACGAGAATGAAGAATATGTCGGTTGAAAGAATGCGAGGGGAGCGGATAGTTTTATTTGTTTGATC
CATGGCCGTAACAGCAGTTTTATTATTAGTTTCTTTACCTGTATTAGCAGGAGGAATTACTATGTTGATTTT
TGATCGTCATTTTAACACTTCTTTTTATGACCCTAGGGGCGGAGGAGACCCAGTTTTATACCAACATTTGT
TTTGGTTTTTTGGGCATCCAGAAGTTTATGTCCTTATTTTACCAGGGTTCGGAATAGTGTCTCATGTGGTT
GCTCATTGTGCGGGAAAGGATGAAGTGTTTGGTGTGTTAGGAATAGTTTATGCTATAGTTTGTATTGGTG
TTTTGGGTTTTATTGTTTGAGGGCACCATATATTTACCGTAGGAATAGATGTAGACTCTCGAGCTTATTTTA
CTTCAGCTACAATAATTATTGCTGTTCCAACTGGAGTTAAAGTGTTTAGTTGATTGGCTACTTTAAATGGT
GGAAATTTATTGCATGAGCCCGCACTTTATTGGGCAGTAGGTTTCATTTTTCTTTTTACTGTGGGAGGCCT
TACTGGTATTATGCTATCAAATTCTTCTCTTGATGTGGCGTTGCATGATACTTATTATGTTACTGCTCACTTT
CATTATGTTCTTTCTATAGGTGCAGTATTTGCCCTATTTTGTGGTTTCTTTCATTGATTTCCTTTATTTTATG
GGTACTGCTACCATGAACGTTGAAGTAAAGCTCATTTTTTTATTATGTTTATTGGTGTAAATATTACTTTCT
TCCCCCAGCATTTTTTAGGTTTAAGTGGGATGCCTCGTCGTTACTCTGATTATCCAGATTGTTTTATAAAAT
GGCATGTAGTGTCATCTTTAGGGTCATGGCTGAGATTTGTCAGTGTTTTATACTTTCTTTTTATTGTGTGG
GAAGCTTTGGTAAGACAACGAGGTGTTGTGTGTAAAAGAAACCGACCCGGGGCTATTGAATGAAGGGA
GTGGTGTTGTCCTTTGATGCCATTATGAGGTAGTTTTAGGTTTCAAGATTGTTATTATCATATTGGGGAGTA
TCTTGGTTTGTTTCATGAGGGTGTGATATGCCTTATAATTTTTATTTTGTCTGTTGTGTTGTATGGAGTAGG
TTGGGTTTTCACCTCGGGAAGAAGGTTTCGTTATTTACGTGAGGCACAAGCGGTAGAAACAGCATGAAC
TATTATTCCTAGGGTGTGTTTAGTTTGTGTGGCTATCCCGTCTATACACTTATTGTATGTAATAGATGAAAT
TGGGAGTCCTAAGTTCTGTTTTAAAGCAATTGGCCATCAGTGATTCTGGTCTTACGAAATGGAAGATGTG
TTGGGATTTGATTCTTTTATGGAGCGGGAGATAGAGAGAGGTTATCGATTATTGGATGTAGACCAACGGA
TGGTTGCGCCGGCTAATACCGGAATTCGATGCATGGTAAGAAGGGCGGATGTTATTCACTCCTTTGCTCT
CCCTGGTTGCATATTAAAAGTCGACGCTATTCCAGGCCGTGTTAATGAAGTTCCTATAACTGTAAACATGT
GTGGAGTTCTTTATGGTCAGTGCTCTGAGATTTGTGGCGCAAACCACAGGTTTATGCCCATTGTGATTGA
ATTTATTCATCCGCGTGTGTATAATTTATGGGTAGAATCTTTTGACATC-----------------------
ATGCCACGTAATGCTTTTTATTTGGTTGGGCCAAGTCCGTGGCCTGTTTTTACTTCTATAGGAGCTTTTTG
TATGGCAGTAGGTTTCGTTTCTTGGTTTCATAAGCATGGTTATGCTCTTTTATTAGGTGTGGTTGTATTAAT
TCTTTCTTTAAGACAATGGTGGCGTGATGTTATGCGTGAGGGTGATTTAGGTTTTCACACTTCATATGTTG
TTAAAGGTTTACGGGATGGATTTATTCTTTTTTTGGTTTCAGAAATTATATTTTTCTTTTCTTTATTTTGAGC
TTTTTTCCACATAAGGTTAGCTCCTGATATTTCAATGGGATGTATGTGACCTCCTAAAGGTTTGGAAACTT
TAGACCCTATAAAAGTTCCATTATGCGGAACAACCGTATTAGTGGGCTCTGGTGCGTCTCTTATGTATTCT
CATGCTGCGATTCGTGCAGGTTTAAATACTCATGCAGTTTTAGGAACTTTTTATACTATTGTGTTAGGCTT
GTTTTTTACACGGTTACAAGGATATGAATACTATTGAGCTAGGTTTACTATTGCTGATAGTGCTTATGGGA
GTTTGTTTTATATTATAACTGGGTTTCATGGGCTTCATGTTATATTTGGGACAGGATTTTTACTTGTCAGCT
TAGTTCGTTTAATGCGTAATCGGTTCACCCCACGCAACCATTTTGGGTTCATGGTGTGTTCTTGATATTGA
CATTTTGTTGATGTTGTTTGGATTGGCTTGTATTTAGTTGTCTATTGCTGGGGTAGTTAACCTTTACGCAA
GCAGGATAGAGTAATAAAAATTTTAAATAATAGATTGTATGATTTACCCGCGCCTGTTAATTTAAGGGTCT
GATGAAATTTTGGGTCTTTGTTAGGTTTATGCTTGGTTATTCAAATCGTTACAGGTTTCATCTTAAGCCTC
CATTATACGGCCCACACAAATATGTCATTTGATGCGGTTATTCATGTGATTCGAAATGTAAATAATGGGTG
GTTAATTCGTGGCATACATGCTAATGGTGCGTCTCTTTTCTTTGTTTGTGCTTATTTCACATTGGTCGTGG
TTTGTATTATGGTTCTTATAAGTCTCGGATGGTGTGAAATGTTGGGGTGATTTTGTTTTTTCTTCTTATAGC
TACTGCTTTCTTGGGTTATGTTCTCCCTTGGGGGCAAATATCTTATTGAGGGGCAACTGTTATTACAAAAT
TAGTGACTGCTGTGCCTTATGTTGGTGATATAATTTTGTATTGAATTTGGGGCGGTTATACAGTTTGTAATG
CTACTTTGGTGCGATTTTATTCCTTTCATTTTATTCTTCCATTTATTATAATTGTTTTTAGTGTTCTTCATTTG
CTTTACTTGCATGAAGAGGGGGCTAACAACCCGTTGGGGGTGAGTGCAGATGTGGCTTTGGTGCGATTT
CATCCTTTTTACACATATAAGGATGCAGTAGGTTTCTTCATTTTATTTTTTGGTCTTTTATTATTAGTTTGCT
ATTTCCTGACCTTTTAGGAAACGCGAACAATTGAATTCCTGCGGATTCAATAAAAACTCCACTTCACAT

TGAACCAGAGTGGTACTTTTTATTTGCTTATTCTATTCTTCGTTCAATTCCTAATAAAGTAGGTGGGGCAG
CTGCCTTGGTGCTATCAATTTTAATTTTATTTCTTGTTCCTAGGTTGCATACAGGTCAGTTTCGTTCTAACA
GGTTTTATCCTTTAGGTCAGCTTTTCTTTTGGATTCTGGTTGCAGTTTGAGTGATTTTAACATGGCTTGGG
GCTTGTTCGGTTCAGTACCCTTATGATTCTTTGGGGTGTTTCTTTACTTTCTTCTATTTCTTTTTTATTATAC
TAGTTCCTTTAAGACAAGGCGTGTGGGATTGGTTAATTAAGATGCAAGTTATCAGCTTTATTCTACCAGG
AGTTTTTGGTTTATTGGCAGTAGGATGATTTACATTAGTCGAGCGCAAAGTGTTAGGTTATATCATGACTC
GTAAAGGCCCTAATAAAGTGGGATTTTTAGGGTTAATGCAACCAATAAGTGATGGGGCTAAACTATTTTC
TAAAGAAATACTAGTACCAATATATAGGAATTTTGTCCCATTTCTGGTGTGTCCTGTGGTGACATTTTTTAT
TGCCTTGGTGCTGTGATTACTATACCCATTTCACTCTTCTGAGGGTGTGTTTATGTGCGGGGTATTATTTTA
TCTGGCAAACTCTGGAGCTAATGTTTATGGCGTAATGGTGGCAGGGTGATCATCAAATTCAAAATATGCC
CTTTTAGGGGCTATGCGGGCTATGGCACAAAGTATTTCATACGAAGTGAGAATAGCATTAGTACTGTTAA
GATGTGTTTTTATGTCGGGGTCAATACATCTTCAATCTGTGAAGTTGTTGTTTATCATGGGTGCAATTTTA
CCTTTTTTTGTGGTGTGGTTAATTTCTATGTTAGCAGAAACTAATCGTGCACCATTTGATTTTGTAGAGGG
CGAATCAGAGCTGGTATCAGGTTTTAACGTGGAGTACAGTAGAGGCGGGTTCGCCCTTATTTACATAGCA
GAATATTCTAATATGTTGTTTAATAGCCTGTTTACATGCGTGATATTTTTGGGTACAAGTGACGCAGTAATA
GTCGGGCAGGCATGAATTTTTCTTTTTTTTTTCTTGTGGGCTCGTGGGACTTTCCCTCGTTTTCGATATGA
TATACTAATAAGGCTGGCCTGAAAGACTTTTTTATGTATTGTTTTGGGGTTGAGTTTATTTATTTCTATAAT
TATTTATATTTTataATTAGACGATTTGAGGGTCCTTTAATAGTTTTGAGGGTTTTTACTTTAACTTTTGGTTT
GATTATTATAATCTCAACCGATAGGCATCTAACTGCATGGGTTGGGATAGAAGTAAATATGCTTGGGTTTA
TGTGCCTTTTGGGAATTAAATCAGTGTTAAATATGCGTGTTTTGATTAATTATTTTGTTTTTCAGAGATTTG
GCTCAACTATATATTTGTTTGGGTCAAGTGTTATTTTATTTAATATTGCTTTAGGATATTTTTTAATTCATTTA
GGACTTCTTTGTAAAGCTGGACTTTTCCCTTTTTGGGTGTGGGTTCCTTCTGTAGTCAACTCTAGGAACT
GATTTGTTAGCTATTTGCTATTAGGAATTCAAAAAGTTGGCCCTTTACTTTTGGGTGTGTGGTCCCCTTGT
AGAGAGTTTTTATATTTTGTAATTTTTGCTATAAGAATTTTGGGTGGTTTAGGTGGGTCTATTCAGAATTCT
TATCGTGACGTATTGGTGTATTCTTCTTTTGTTCACGGTGCTTGAATGTTAGTATGTTTGATTGAATCCCAG
AATCTATTTCTTTTTTATATTGGAGCTTATCTTGTACAATTAGGTTTAGTCATTTACTATTTGTGGCGCAGA
AATGCAAACTCTATAAAGAGGGGGAAATGATCACTTATAGTGGCAAGGTCTTTGATAAGTTTGAGGGGG
CTGCCTCCATTAGCTGGTTTTGTGGTAAAGATAACTGTTGTTTTTTGCGTTGATAAGTTAATTTTGTTATTT
ACATTAGCTGGCTCTATTACGGCTATATTTTATTATTTGAAAGCTGCAAATAGCTCCATTGTTAAATTATTA
GATGATGAGGGCATTTTCCTCTTTTTTACTTTTGGTTTATACGTTTGAATTTATTTGTTGGGGTGTTTCTTT
TTGTAATAATCTGTGTCTGTCTTCTGGTTGGTTGTGATAATGGGTGTTTGTCTTATGTTGTCTATAATGctcat
gttatctatgacgGGGCAGTGGAGCCGTGAAAAGGTTTCGCCTTATGAGTGTGGGTTTGACCCAATCCTTAGGG
CCCGTAGGAGGTTTTCTTTGCGTTTTTTCTTGCTTGGTGTTTTGTTTTTAGTGTTTGATGTAGAAGTGGTT
ATGGTTGTTCCCCTTTTGTTTGTATTGTATGGTGGGGCGGAGGTAGTGGGTGTTGTGTGTTTAGTGGGTT
TTTTACATGTGCTTACTATTGGTTGTCTTTATGAGCGTCGCGACGGGTCGATGGATTGGGTTAGTGAGgtgA
TTATTGGTTCTGTTATGTGTATACTTTTTTTAGGGTTGTTAGGAAGATTGGAGTTATCAATTGCTGGTTTGT
CTTTACTAGGTGCGATAGCGATGataTCTTTATTTCTGTGCGCTGAATACTCTGAATTAATTGGAGTCTTTAA
TTTGGATTTTCCAGGGCAGGCCTTAGTAGTCCTTAGAATTTATATCAGATTTTTAATGGTGATGGGCAGGG
CTGTGGTGTCTCGTTTTAGGAGGTTTAATAGGCTTATTATTTGTATTGGTGTTTTGTTGATTTGTGCTTTTA
GTGTAAGGGCCACTTTCCTGTTCTTTGTCCTTTTTGAGGGAGTTTTATTTCCAACCTTGTTGTTGATTGTA
GGATGGCGGTTACAAGCAGTTGTTTATATAGTTATTTATACTGTTATAGGATCACTACCCCTTTTGTACGGG
TTTGGGAAACTATATTTTAATTATAGTGACAATTTATTTCTGGAGTACTTTTTGGATAAAAGAATTTTGAG
GTTTAGCTGGCTTTACTTATTAGCGTTTTTAGTTAAGCTTCCGGTTTTTCCTTTACATTTATGATTACCTAA
AGCTCATGTAGAGGCCCCAGTCGCTGGTTCTATGATTTTAGCAGGTCTTTTGCTTAAGTTAGGGGGTTAC
GGGGTTATTCGTTTAAGAGGCCTCCTTATTATTAAAAGAATTAGAATAACTATACTAGTAGTAATAGTTAG
GCTTTTTGGAGGGATTCTCACAAGTATGGTTTGTTTACGTCAGACAGATTTTAAATCTTTAGTAGCTTATT
CTTCTGTTGGACATATAAGTTTTGTTTTAGTCCTATTGAGTAATACTTTATGAGGGCTTATAGGAGCGATGT
TAGTCATGATTGGTCATGGATTATGCTCTTCTGGTTTATTTTCTCTGGTTAATACTTTCTATCTTAGAAGCC
GTTCTCGTTTGTTGGCAATAAATAAGGGATACTTAATACTCAGCCCGTGTGTAGCTTTGATGTGCTTTTTG
TTGAGGGTTAGAAATATAGCTAGCCCACCTAGGTTGAACTTATTTGGAGAACTTTTTATTTACATAGCAG
GTGCTTCTATAAATATGTTGTTTTTCCCTTTATTAATGCTGTTGAGGTTTTTGAGTGCTTGCTATAGGCTTT
ATATTTATATTAGTTCCCAACATGGAAAATGTATAAATAGACTAGATTCACGTAGAAGTATGATAGACATTT
CGGTGTTGACTTTTCACTGAATGCCTTTAAACTTTTTGTTTGTAGTTATTCCATAGATGCGTAGAATACAT
TTGATTACGATTTTTCTAGGGATGGAGTTTAGTGCGCTTGGGGTTTTTGTCATTGGGTCTTTATCGGCGTA
CAATAATTTATTTGTTCTACTAGTTGTAATTTGTATTAGGGTGTCTGAAGCAGCCATTATGCTGTCTTTGAT
GGTTATAATGACTCGTATATATGGAAATGATCGATGCCTTAATCTGATAACTGATAATTTAAAGTTAATGAA
GTTTTAGGTTGTATTTTTATGATTTTAGGATGGGGTACAATAAGTATAATCCCTTTCAGCCCTTTAGTTCTT
GTTGACTATAGAATTTGATTGTCTGGAAATTTGTCGGTAAATTTGACACTTCTACTCGATCAAATTAGAGG
ATTATTTATAGCCACCATTTTTTTGATTTCTGGCTCTGTTTTAGTTTATTGCTCCTGGTACATGGCTGATGA
AATTTATTTTTCGCGTTTTATTTATCTTGTTGTACTTTTTGTTTTATCTATAATATCCTTAATTATAATTCCTAA
TTTAATTGCTCTTTTGCTGGGATGAGACGGACTTGGTATTACCTCTTTTTTATTGGTGGTCTATTACCAAA
ATAACAAGTCTTTGGGGGCAGGTATAGTTACGGCCTTGACTAATCGAGTTGGAGATTCAATCCTGTTATG
CATTATTGGTGTGTTTGCTAGGGAGGGGGGATGAGTATTATTTGAATTTTTACCTAGTATGAGGTATTTTG

TCCCTTTTCTTTTAGTAATAGCTGCTATTACTAAAAGAGCCCAAGTGCCTTATTCGGCGTGGTTGCCTGCT
GCAATAGCTGCCCCCACCCCTGTTTCTGCATTAGTCCACTCGTCTACTTTGGTGACAGCTGGGGTTTACC
TTTTGATTCGTTCTTACCCCCTTCTTTCTCAAAGGTCTTTAAGGGTATTAAAGTGTCTCAGGCTTTTTACT
TTACTAATGGCTGGGAGGGTAGCTCTTGTTGAGGTGGATTTAAAAAAGATTGTAGCATTATCTACTTTAA
GCCAGTTGAGAATAATATTATTCGCTATTTCAATTTGTTTACCAAAAGTGGCTTTCTTTCATCTTGTTACTC
ATGCTATATTTAAATCTCTCTTGTTTTTAAGGGCAGGGGTTGTGATTCATAGAAGGTCAAAATGGCAGGAT
ATTCGCTTTTTAGGAAAAAATTGAGCTCGTTTGCCAGTTAGGATAAGTTGTATTACTGCCGCGAGTCTCT
CTTTATGTGGTATGCCCTTTTTGAGAGGGTTTTATTCAAAAGACTTGATTATTGAGTTTCAAGGCGGGGA
CTTAGTCATTTATTTTATACTAGTTGTTGGAACTTTATTTACCTCTTGGTACTCCCTTCGTATATTTAATTTG
TTTTTGAGTACAAATAAGAGTGTTAGCCCTGTAATTTTCGTGGAAGAAGACAGGAGTGTTAAATTTGCTT
ATATGGGCTTATTAGTTAGCTCTGTGGTTATAGGCTTTTTATTAGTTAACTTAGTTTCAGACCTTGATCTCA
TGGCTATGGTGAGTAATGTGGAAAAGTTTGTTGTAATATTATTGGTGGTGGCTGTTATTATTGAGGTTTCT
GCAGAATGAAAAAAACAAAACAAAGTGGGTTATGGTTATTTGGCTAGTATATGGCATTTTAAACTGTTAC
AATTTAGCCCTAGTGTGGGTTTAAAATTAGCTAGTCTTATTACAGTTAGGATGGAGAAAGGGTGATTAGA
AAAAGTTGGCCCTCAAAGAAGAGTTCAAACGTTAGGTTTTAGCAATCAATACCACTTTTTATTGAGACT
TAGGCTGTTTCTTTTGATTATTTTGGGGAGGTACTTGTAATTGTTAGAAACATTTCTCTTCTTTGTTTCTTG
TTTGGTATGTATGTTAAGGATTAGGGAGCCTTTGTTTATGGGATTGGCCTTATTCGTTGTATCTTTTATTATT
TCTGTTTTGTTGGGGTTAGAAATAGGTAGTCTTGTGGGTTATTTTGTTTTTTTAATTTATATTGGTGGATTA
ATAGTTTTGTTTGGGTATGTTCTTAGAATTTTTCCAAATCAGCGATTCGGGGCTCCTGTTTTATTTGTTAAA
TTATTATTAGTTTTACTAAGATTTGTTTTGGTTTTTCATAAAAATAAGAGAGATCAGTTTTTAAGCTTGGG
GGATTTTGGTTCCATATACTTGTTTGTTGCGTTTTTCCTCCTTTTTGTATTGTTGTTAGTTGTTGCTTTTTGT
AAGAAGCGTCGTATACCTCTTCGGATGGTG"/>
<sequence id="seq_Brachidontes_exustusKM233636" spec="Sequence"
taxon="Brachidontes_exustusKM233636" totalcount="4"
value="ATGATATTAGACATTTTCTCTTCTTTTGACATAAACTGTTACAATACTATATCATTAAGATCTGTGTG
AGTTATAAGCTTTTTTTTCTTAATTTTTTGTTTGAATAGCAAGGTTTTTAGGTTAAAAATGGTAATTAGATT
TATATATGGAGTAGTAAAACGAGTTCAGGGAGGGTATTTTTCTGGGTTCTCCTTAAGCGTTTGTTCCTTGT
TTTTTCTAATGGTATGAATAAATATAAATAACATTGTCCCATTTTTTTTTCCTTTAATATGCCATCTTCCCCT
GCCGATGTTTTTTGCTTGTTTTTTTTGGGGTATTTTGGTTGTGTCAAGCTTACTTAATAGTTGGGAACAGG
TTGTTGCAATGGCAGTCCCCCTAGGTCCTTTAATTCTATCTCCTCTAATAGTTTTATTAGAAACAATTGCTT
CTATTGTCCGTCCTATCACTTTAACAATACGGGTGGTGTTTAATTTAGCGGGAGGGCAAGTTATTTTAGGG
CTAATTGCTGAGTTAGATAAAAATTTAGGTTTGTTAGCTATGTTAGTGATTATCCTTGTGGTAGTATTAGGT
ATTGGGTTCGCATTTGAAGTAGGAATCGGGCTTTTGCAATCTTACATCTATTGTATACTGTTGTGCATATAC
AGTGAGGATCATTCTATGTGAATTTGAAGCGGTTTGGCGGGAGTTGGTTATAGAATATTAATTCGTTTGCA
TTTGATACATCCTGGTAATTTCTTGTTGAAATCAGATAGTCTATACAACATTGTGACACATGCTTTAGTTAT
AATTTTTTTCGTAATGCCTTTACTTATTGGTGCTTTTGGTAACTGGCTTATTCCTTTAATAATTGGTGCTATA
GACCTTGCTTTTCCGCGTGTTAATAATTTAGTTTCTGAATTCTGCCTAGTGCCTTTTATTTATTATTATTAT
CAGGTTATGTGGAAGAAGGAGTGGGGACTGGTTGGACTATTTATCCTCCTTTATCTACTGTAGAATACCA
TAGAAGCCCTGCGATGGATCTTGCTATTTTATCACTTCATTTAGCAGGTTCTGGGTCTTTAATAGGCGCTA
TTAATTTCTTAACTTCTAATAAAAATCTCCCTGTTAATAAAATAAAGGGAGAGCGATCTGTCCTGTATGTA
TGAAGAATCACGGTTACAGCCTTTTTGTTGCTGTTATCTTTACCGGTTTTAGCAGGAGCCATCACTATACT
GTTGTTTGATCGTAATTTTAATACCACATTTTATGACCCAATCGGAGGAGGAGATCCTGTGTTATTTATAC
ATCTTTTCTGGTTTTTTGGACACCCAGAAGTGTACATCCTAATTCTGCCGGGGTTTGGTGTAATATCACAC
GTTACTGCGCATTATGCTGGTAAAGAAGCTCCCTTTGGTGCAACAGGTATAATATATGCTATAATCTCTATC
GGAATTATGGGTTTTATTGTTTGAGGGCATCACATATTTACGGTGGGCCTTAATGTAGATACGCGTAACTA
TTTTGGTTCTGCTACCTTAATTATTGCTGTGCCTACCGGAATTAAAGTTTTTAGGTGATTAGCTACTCTGG
CTGGAGGTCGACAAACTTCTAAAACGCCTGTCTTATGAAGTATAGGCTTTATTGTACTATTTACAGTTGG
AGGTTTAACAGGGGTCACTCTAGCATCATCTTCATTAGATGTCTCGTTACACGACACCTATTTTGTTACAG
CACACTTTCATTACGTGCTATCCATAGGTGCTGTGTTTGCTATTTTTTGTGCCTTTACTCATTGGTACCCTA
TGTTTTTTGGTTCTAATTTGCATGGTCGTTGAAGAAAAGGACATTTTTTCTCTATGTTTGTAGCTGTTAAT
TGTACTTTTTTTCCTATGCACTTCTTAGGTTTAAGAGGTATGCCGCGTCGTTATTGCGATTATCCTAATTGT
TACGCTAAATGGCATTGATTAGCTAGTTATGGAGCGGTTATAGGTTATATGTCTTTAATATTCTTCATGTTC
CTATTGTGAGAAAGAGTTGTAAGTAAACGAGGAGTCGTATTTCATTCTGCTCTCTCAACTGAATTGGAGT
GGATAAAACACTTGTTTCCTGTGGTATTGTACGGTTCTAAATATTTTCAAGATGTTATTTATGTGATTGGTG
ATAATCTTCAAAATTTTTTTCAGTATATAATAATGCTTAGTGTATTTATTCTATCTTTTGTTTTAATTATTATAT
ATCGCGTTTGTACAAGTGTCTGGAAATATCGTTATTTTAAACATAGAGAATTGTTAGAATGGGTCTGGAC
TGTGATTCCTATAATCACTTTATTTGTGCTATGGTTTCCTTCTACTATAAACCTTTATGATATAAACCATGGC
GGCGAGCCTAAATGGTCTTTCAAAGCTATCGGACATCAATGGTACTGATCTTACGAGCTTAAGAAGCAAT
TATCTATTGATTCTTATATAGATGTGAGTGCTgagagaGGCTATCGTTTACTTGACGTGGATCAGCGTATAGTT
GTACCTGCGCTGACACAAATTCGTTTATTAGTGTCTAGTGTGGATGTGTTACACTCCTTTGCCATGCCTGC
TTTAATGTTGAAAGTAGATGCAATTCCAGGCCGAATAAATCAACTACCTTTTACGGTAAGACGAACTGGA
GTTTTTTATGGGCAATGTTCAGAGATTTGTGGAGTAAATCACAGCTTCATACCAATTGTTCTAGAATTTAT
TCGTAAGGAAGAGTTTGACCAGTGATTAGTGAAAGTTTGTGAT-----------------------

ATGGTACGCAATCTTTATTATCGAGTTAGCCCTAGTCCATGGCCTTTTATAGTGTCATTTTGTCTTTTAGAT
TCAGCTTTAGGGTTAGTGACGTGAATAAGAGGAGATGATTTAATATTTATTGGTGGTTTTTTATTGCTTTT
GTGTTGTGTAACTCTGTGATGACGGGACCTACTGCGCGAAGGGGACCAAGGATATCACACAAAAAAAG
TGGTTAAAAATTTTCGTGATGGTATAGCTATGTTTATTGTTTCGGAGGTTATATTTTTTTTCTCTTTCTTTTG
AGCCTTTTTTCATAGTAGTCTCAGACCAAATATTGAAGTTAGAGGCACTTGACCTCCTGTTGGGCTTCGT
ATTCCTAATGCTTTTGGGATTCCGATAGTTAACACAGGAATTCTGGTATTAAGAGGAGCTTTTATTAATTAT
TCACTAGGTTCAGTGCGATGTAATTATGACCACGGCGGGATCGCTGGATTGGTCATGGCAATCCTTCTTT
CTTTTATCTTTCTTGCGATTCAATACCATGAGTATCAAGTTAACTCTTTTAGCATAGCTGATGGTATTTATG
GTAGTACTTTTTATATATTAACAGGTTTTCATGGCTTTCATGTATTAGCGGGAACTATGTTTATACTCGTCA
CTCTCGTGCGGATATGGTATGGTCACTTTTTGTCGGATCGATTTTTTGGGTTGCAAGCTTGTGTCTGATAT
TGGCACTTTGTGGACATTGTTTGAATTGGTGTTTGGGTTTTTTTCTATGTTTGAGGCGGAGGTCCTTTTCG
AAAGCGACACTGGTTTTTAAAAATTCTTAGCTATGGACTATATGATTTACCTTGTCCTATCAATCTTAGTG
TATGATGAAGTTTTGGCTCAATATTAGGTTTGTGTTTAACTATTCAAATTGTCACAGGTTTTATGCTTTCAT
TTTATTACGTGCCTCATGCTGATATGGCCTTTGATTCTGTTATTTATATTATACGTAATGTTCATAAAGGGTG
GATAGTACGAAGTATCCATTCTAATGGAGCATCTATGTTTTTTATGTGTATTTACATTCACATCGGTCGTGG
ACTATACTATGGTTCTTATTTAGATAAGGCAGTCTGAAATGTTGGAATTGCTTTATACTTAATGTTAAGCGC
AGAAGCTTTTTTAGGTTATGTTTTACCTTGAGGTCAAATATCTTATTGAGGAGCTGTAGTAATTTCTAGAA
TGTTAACTGCTATTCCTTATGTGGGCCAAACCATAGCTGAGTGGTTGTGAGGTGGTTATGTTGTTAATACT
CGTACTTTAACGCGATTTTATTCGTTTCACTTTATCTTACCTTTTTTAATGGTAGTTGTAGTTCTTTTACACT
TGTTTTATTTGCATGAAAAAGGTAGAAATAATCCTCTAGGTGTAAGTAGGGACAGAATGCTAATTCCTTT
TCATCCTAGATATACAGTTAAAGATATTTTCGGATTTGTTTGTATGCTGTTCGTTCTTATGTTTTTTGTATGT
GTGAAACCAGAAATGTTAGGCAATCCTTTAAACTTTATTCCTGCAGATAGAATAAAAACTCCTGTTCATA
TCCAGCCAGAGTGGTATTTTTTGTTTGCTTATACTATTTTACGGTCTATTCCTCATAAAGCGGGTGGAATT
GTAGCAATGTTGGCTTCTATTCTTGTACTGGCTGTTATACCCTTTGTTCATACAGGAAAGTTTCGTGGGCT
AGCATATTATCCTATTCATCAAATACTTTTTTGGGCTTTTATCTCTGTGTTTTTAGGTATGACTGCTATTGGA
ATGCGTCCAGTAATAGAGCCTATTTATACCGTCGGTCAAATTTTAAGAGTGATTTATTTCGGTCTAATCTTA
TTTATTCCTCTAAGTATATTTATGTGAGATAAGCTAATTTTCGTGGATGTTGTCATAATTGTTTTACCTTTTA
TTTGCGTATTGTTAGCTGTAGGATTTTTTACGTTATTTGAGCGAAAAGTGTTAGCTGCAATTATAATTCGTA
AGGGCCCAAACAAGGTTGGGTATATAGGATTACTTCAACCTTTTAGGGACGCAGGAAAATTGTTTTGTA
AAGAGTTTGTTTATCCTAATCGTGCTAATGTCATACCTTTTTTTTTTGCCCCTTCCTTGATGTTGGGAGTAT
CAATAAGATTGTGAATTTTATATCCTTTAAACTTTGTTGATATAGTATTTGTCTTTGGCGCGCTGCAATTTA
TGGTAACTGCCGGTGTTAGCGTTTTTGGTGTGATAATGGCTGGTTGGGCTTCCAATTCTAAATATGCCTTA
CTAGGTTCAGTCCGAAGAATTGCACAAAGAATTTCGTATGAAATCCCGTTTTGTTTAATTCTTTTTTCTGT
AGCCGTTTTTATCTTAACTTTTATGTTTCAAGAAATTATGTTATTTTTTGTACTGTGTCTTTATTTAAGGGG
TATTAGATTGTGATTGATTTCTATCCTTGCTGAAAACCATCGCGCCCCTTTTGATTTTGTTGAAGGAGAAT
CAGAGCTTGTTTCTGGGTTTAATGTTGAGTATAGTGGCGGTGGCTTTGCTATAATTTTTATATCTGAATAC
AGGAGAATAATATTTAGTAGTATCGTAACAAGTGTTATATTTTTTGGTGGAAGAGAGTTGTTAGTTAGATT
TCTATCTCTTTTTTTCATTTATTTTTTTGTTTGGATTCGTGGGACTTTTCCACGAATGCGCTATGATAAACT
TATGTATTTAGGTTGGACGATTTTTACTATTGTTCCTTTGGTTTATTTGATAGCGATTGTAGTTGTTGGTTAT
GTCtATTTTACAATatgGCTGTAAACCCCATTATGCTTATAAGAATGTTTTTAGTGTTGTTCGGAACTTTGGT
GAGCGTGTTTAGCTCTTCTTGACTGGGGGTTTGACTTGGCATAGAAATTAATCTACTTAATTTTATAGTAC
TCATAAACCCTGACGGTGTTTTTGTTGTTGAACCTGCAGCTAAGTATTTTGTAATTCAATGTGTGGGGTC
TAATTTTATTCTTATAGGTTTTTTATTAAGTGGCGTTTATGCCAATATATCTAATGTTCTTTTAGTAATTGGTT
TAATGTTAAAAAGAGGCGTATGTCCATTTCATGCTTGATTACCTTCAGTGGTCAGTTCTTCTAATTGGTTC
CCCGCTCTATGAATTTTAACGTGGCAGAAATTAGCCCCTTTTGTCTTTATAGGTTGATTTATCAGGAACTC
CATTGTAGCATTTAGAGTCGGAAGTTTAGCACTAGTGGGAGGTATTGGTGGTTTAAACCAGCAAAGGAT
TCGCGGTTTGTTAGCTTATTCTTCATTTGTCCATTCTTCCTGAATAATTTTAGCTCTTATAAAATCATTTTGA
ATTTTTATTCTGTATTGAGTTGTTTACTGTTTTAGCGTTGGAATAGTTTTTTTGAGCGCTGCATCCTATGGA
AAACTTTACTTAAAAAGAAAAGGACGGTTGATTTGAGCTTCATTTGGAGTATTTATATTAATGGGTTTACC
GCCTTTCTTAGGTTTTGCTTGTAAAATTTTGGTATTTCTAAGAATTGATAGTTATATAATTTTCATATGTGTG
GTAGGTTCTCTGATCAGAATGAAATATTATCTAACACTTTCTTATTCTTTTATTCTCGGTCATTGATCTATCA
ATAAAATATCTATTTTTAGCATCTTAATATTGAATTTTATTGGTTTTATAGTAATGAGAGTTTTTTTATTTGTA
atgATGTTGTTTGTATCGGTAGTCTTTGTTTTTATTATTTGTATGTTGTTAAGTGTGTTGGTGTGCTTATCTTT
TTACAAAGTATACGATCGTGAAAAAAGCTCTCCATATGAGTGTGGGTTTGAAAGAATAATATCCGCGCGA
CATCCTTTTGCAGTGCGGTTTTTTTTAGTAGCAGCCATTTTCGTTGTATTTGATGTAGAAATTGCTTTATTA
ATTCCAGTAATCTATGTATTTTTTTATAGTTTTAGGTTAATAACTATGGTTATTGCTTTGGTCTTTGTAGTAC
TATTATTTGTAGGCCTGTTCCACGAACATCGTGAAGGTTCTTTGGATTGAGTGGCTTAAATGTTTTTAGGG
TTATTTTTTAGGTTGATGTATATCCTATTTATTCGCAAGTTTAGAGTGAGCTTGAGCGCTGCGAGATTTCTT
GTTGTAAAATGTATAATGCTGATTAGGCAAGGGTCATCAAGTTTCGAAATAAGGGGGCTTGTGTGATTTG
ATTTTTTAAGTTCTAGCATAGTATGTTTAACCGTATATATTAGTTGTTTAATACTTATTGAAAGTTGTTCAAT
AGCACGTAAAATAGAAATAAAGTTAATTTTAACAGTATTGTGTGGTGTTTTAGTTGGAACTTTTAGATGA
ACTAATCTTTTTTTTTTTTTTTTTTTCTTTGAAAAAGTACTAATTCCAGTCTTATTTTTGATCTTGATCTGAC
GTCTACAGGCTGGTAGTTATATGATTTTGTACACCAGATTGGGTTCTTACCCATTTTTGTTCGCTATTAGTA

TTATGGTGTCTGGCGGTAGAGATATAATGCAGTTAAGCTCTTTTCTAAACCGTGAGTTTTGCTTTTTTTTC
TGGTTCTACATATTAGGGTTCTTGATCAAACTTCCTATGTTTCCTTTTCACTTATGGTTGCCTAAAGCCCAT
GTAGAGGCCCCTGTAGTTGGTTCTATAATTTTAGCTGGAGTACTCCTTAAATTAGGTGGTTACGGTATGCT
TCGTTACCTAAATTTAGTCTTAATAAAAATATGTAGGTTTAGTAGAACTTTATTAGCCATTGGTTTAGTAGG
GGGTTTTTTAACTAGAGTGATGTGCATGCGTCAGGTAGATTTAAAGTCCCTGATCGCATATTCTTCTGTTG
GCCACATGAGCTTGGTTATCTTGATTCTAAGTGACAAGATGGTAGGGTTTTTAGGAGCTGTTCTAATAAT
GTTGGGCCACGGCCTGTGTTCCTCTGGACTATTTCTTTTGGTAAATCTGTTTTACAAGCAAAGTGGTTCC
CGTTCTGTATTATACAATAAGGGAAGTCTTATACTTACACCTAGTTTAGCGTTGTTTTGTTTTATACTAAGT
GCTGGTAATATGGCTGCCCCTCCCTCTTTAAACTTTGCTGGTGAAGCCCTTTTGTTCATGAGAAGTTCTG
TGTTAAGGAGATGATTGCTTGTGGTGGTAGGTCTAATAAGTTTTATAAGAGCTTGTTATTCCTTGTTCTTT
TATGGTGTATGTTGTCATGGGAAAAGTTCGAAAATCCGTGATTCTGGTATTTATATATCTAGTATGTTAAAT
TTGTTTTTTCACTGGTTTCCTTTAAATTTTCTATTCTTATTTTTGTAAtaaTATCAGCGAACACATCTGATTAG
AGTATTTTTAGGAATAGAGTTTATTACTCTTAGTGTTATTGCCATAAGAGCTGTGAGTATGACTAGAATAA
GATGTTTTGTTTTGTTAGTTATGTGTATAGCTGTTTGTGAAGCAAGAGTGGCACTAGCTTTGATTGTAAGA
ATAGTTCGAGTAAGGGGCAGCGACAAAAGTGTAAACTTAATTTTGGATAATTTAAAAATGTTATAATAAA
AAGGTTTATAAGAGCTGTTCTAGGTTATGTATTACTTATTATCAGAACAAGTAATAATCTGACAGTACTCA
CATTCTCTTTATTGAGTTACAATGTCTTTGATATTACTTTTTCTTTAATGTTTGATGAAGTAAGCTTTCTTTT
TGTAGGGGTGGTTTTAATTATTTCGGGAAGAGTTGGGGTTTACTCTTTCTGGTATATAGACAATGAAAAG
TTTTTATTGCGATTTTTGTGGCTAATTTTTCTTTTTGTTGGTTCTATAATTATACTAATTTTAGTACCCAATCT
TGTTTGTCTTATACTTGGTTGAGACGGTTTGGGATTGATTTCTTTCTTATTAGTCTGTTATTATCAAAATAG
TAAAAGTTTCAGGGCTGCAATGGTTACTGCTCTAACTAATCGTGTGGGTGATGTGCTTATTTTAATGTGTA
TCGGAATAGGGAGTTTTTATGGAGATTGGATTTTCTATGGGTATAATTACGGGTTTTGTGTAATTATAGCTA
CTTTGCTTATAATTGCCGGAACTACTAAAAGAGCTCAAGTTCCTTTTTCTGCCTGATTGCCTGCTGCCATG
GCAGCTCCTACTCCCGTATCTTCTTTGGTACACTCTTCTACTCTTGTGACAGCAGGTGTTTATTTGTTAAT
ACGTGGGTTTTGATTGTTAGAGCCCAGAAGATTAACAGTGTTAAAAATTATAAGATTAGTTACTATGGTC
ATAGCTGGAACGGTGGCCATTTTAGAAGTGGACTTCAAAAAAATTATTGCTCTATCTACTTTAAGCCAGT
TAGGTGTAATAATATTTTGTTTAAGTATAAAGATCCCTTATGTATGTTTCTTTCACCTAGTTACTCATGCAA
CCTTTAAAGCTTTACTCTTTGTAAGAGCAGGTTGTGTGATTCACTCTAGCTCTGGTAATCAAGATATGCG
GAGCCTGGGCAAGTGTTGAATGGATTTGCCAATTAGGATAAGGTTTCTAAGAGTAGCAAGAATGTCTTT
GGCAGGAATTCCCTTTATGAGAGGGTTTTATTCTAAAGACTTAATTATTGAAATAACTAATAGTGACTCTT
TTTTATACATTATTATGATAGTGGGAGTCTCCTTAACTTCTTGATATAGATTTCGTGCGCTCTCAGTGGTTG
CAGGAAATAATAAAAGAGTGATAGGTCTTGTTTGAACCAAAGAAAGGTCTTTATTAGTTATTTCTTACTT
CTCTCTTTTTTTTTGTGCTGTTTTTAGGGGTTGAATCATTAACAGAAAATGTAGGCTAGTTTATTTCCCCG
CTTTCATTGACAGTGTGCTATTTTGAGTGATTTGTTTCATTCCTGTTGGGGTAGTATTTTGGTCCTCCGTC
GATTTGTTTAGTGACTCTCCTCCTAAAGTCAAGTTTGTAGCTTCTATGTGAAATCTTAAAATTAGACAATT
TTTGATCCAACCTATGATAAGGTATAGATACAATGTTTGCCGTTCTTTAGATCAAGGATGATTGGAAATAG
TCGGACCTCAGATTTCTTTATCTAACATAAGTCGCACTAACCAACGTTATTTTTTATCTTTGTTAACTTTTT
TCTTTGTTCTATTTTTTTTCGTGGGGGTTTGAATGGTTTTGTTTGTAGTGTTTAGTGTTGTCCTTTTAATAA
TTTTATTGTTTATCATAGAACCATATCCTCTTGGTATAATACTGATTTTTAGTTGTGTAGGGCTATGCTACGG
ATTAAGTTATTATGTGAGATCCTTATTGAGGTTTCTTGTTTTTATGAGATATGTAGGGGGGGTCATGGTTCT
TTTTTTATATGTTGTCAGTATTCATCCTAATCAAAAATTTAATGTAAGCTTCCATTTTTTATGAATTAGCTTA
TTTTTAACTTCTTTTTGATTGTTTGGATGATACTTAAGTAGTCTTAAAAGGTTCCATTTTGTTAACGTAGGT
AATTTGTATTTATTAATAGGGGTTATTCTCTTAGTCAATTTATGTGTAGTGTGTAATATTTGTATAAAAAAA
ACACTACCTCTACGTTCTGTT"/>
<sequence id="seq_Crenomytilus_grayanusNC044128" spec="Sequence"
taxon="Crenomytilus_grayanusNC044128" totalcount="4"
value="ATGTTAATAGATGTTTTTTCTAGGTTTGATGCTCATAGTTATAATTTAATTAGGCTATCTATGTTGTG
ATTGCTTTCGTCTCTTGTTCCTTTAACTGTGATTTTTAGGGATATTAGAGTTCAAGGCTTAGTTCTGTCGTT
TACTTACTCAATAATTCGGAATGGAAAAGGGCTTAAACTTTCTGGGTTTCCGCTAGTAATGAGGGGGTTG
TTTATAATGGTGTTAATGCTAAATTTATCGGGAAATTTTCCGTTTTTTTTCCCTGTAAGGGGTCAATTTGTG
TTTGGGTTTTCTTTTGCTTTGTCTATTTGAACTTGTTTGGTTGTGTCTAGATTGTTATGCAGATTTGAACA
AGGCTTAATAAGGTTGGTGCCGACTGGACCAATGGTTTTGGTTCCATTCATGGTAGTAGTTGAGTTGATT
AGGGGTATACTTCGTCCATTAACACTGGTGCTTCGGTTAACGTTAAACCTTGGGGCTGGTAAGGTAATTT
TAACTATGTGTAGTGGTGAGCTTGTGGTGAGTTGACTAGTTAGTGGAGTTGGGAGAGTTAAAGGTTTATT
AATAGGAGGCGTTTTCGCTGCTGAGGTTGCGATTGCTTGTATTCAATGTTATATTTTTTGTGTGTTGTTGT
GTCTTTACACTGAGGACCATAGAAGGTAACGATGACTGTGGTCTACAAATCACAAAGATATTGGAACAC
TTTATTTGTATAGGGGAGTGTGAGGAGGGTTATTTGGAGCTAGGTTAAGGCTAATAATTATGCAGGGGCA
TCCTGGAGCGGTGTTTTTAAAAGACTGGTTTTATAACGTAGTGGTTACTACACACGCTTTAATAATAATTT
TCTTTGCTGTTATACCGATTCTTATTGGTGCCTTTGGTAATTGGCTAATTCCTTTGCTTGTTGGAGGGAAG
GACATAATCTACCCCCGTATGAACAACTTGAGGTATTGACTTTCCCCTAATGCACTGTATTTACTAATGTT
GTCTTTTAGAACAGATAAAGGAGTGGGGGCTGGTTGGACTATTTATCCTCCGCTGTCAGTGTACCCCTAT
CATAGAGGGCCAAGGATAGACGTACTGATTGTTTCTTTACATTTAGCAGGGTTAAGGTCCTTGGTTGGGG
CTATTAATTTTGCTAGTACAAACAAAAATATACCTGTGTTGGAGATAAAGGGAGAGCGAGCTGAGCTGTA

TGTGTTAAGAATCAGGGTTACTGCAGTATTATTGATTATTTCTATCCCAGTATTGGGAGGTGGTATTACGAT
GATTTTGTTTGATCGTAATTTTAACACTACCTTTTTTGACCCAGCAGGGGGTGGTGACCCGGTGTTGTTT
CAACACTTATTCTGGTTTTTCGGACACCCAGAAGTGTATATCCTCATTCTTCCTGCTTTTGGGGTAATGTC
TAAGGTAATCATACATTGTTCTGGAAAAGAAGCGGTGTTTGGGTTAATCGGAATGGTATATGCTATGATTG
GAATTGGAGGGCTTGGTTGTATAGTGTGGGCTCACCACATGTTTACAGTGGGGCTAAATGTTGATACTCG
AGGGTACTTTTCTACTGCTACTATAGTAATTGCTGTTCCGACAGGAGTAAAAGTATTTAGATGATTAGCAA
CTATAGCAGGAAGTAAGTTCAAAATGAAGCCTGCTGCTTTTTGAAGAACTGGGTTCTTATTTCTATTTAC
TGTAGGAGGATTAACTGGAGTAATACTATCAAGGGCTTCTATGGATGTATCATTACACGATACTTATTATGT
AGTGGCTCATTTTCATTATGTGTTAAGGATAGGGGCTGTGTTTGGAGTGTTTTGTGGGCTCAATCACTGG
CTACCAAATTTTGTTGGGGTGTGCTTTAATAAGAAGTGAAGAAAAGCTCATTTTATGGCTATATTCTTTGG
GGTAAATACTACTTTTTTCCCTCAACACTTTTTAGGGTTAAGCGGGATACCTCGTCGTTACATAGATTACG
CGGATATTTATGCTCACTGGCATTGGGTGTCCTCTTACGGATCTGCTGTATCCTTTGGATCGCTGATGTATT
TTAAGTTTTTACTTTGAGAAGCATTGGTGAGCCAGCGAGGGATGTCTTTTTATGGGAGTCGATATTTTGG
TGATATTGTGCACGAGTTGGGAAAAGATTTATTTCGTTATCATGGTTTTGTGATGATAGTAGCAGTGGCCG
TGTTAGCCTTTGTAATGTACATAGGGTGTGTTATTCTTTTTACTAAGTACTCTTACCGCCACTTTCTAAATC
GTCAACGATTGGAGTTTTGGTGGACAATTGTGCCTATGCTTTTACTGGTAGGGTTGTGGTTTCCTTCAAT
AATTAATTTATATTATATAGAGGAAGTAAAGCGGCCGCGATGAAACTTTAAAGCTATTGGTAAGCAATGAT
ACTGGTCTTATGAATGCGATGCCTGCTATACAATTGATTCTTATATGGAAGATCAGCAAGAAACTGGGTAT
CGTTTATTAGACGTAGATAATCGTATGGTTGCCCCTGCGGATGTCCAGATAACCGCTTTTGTGAGGAGTT
CTGACGTTCTTCACTCTTTTTCATTACCTAAACTGTTGTTAAAGGTTGATGCTATTCCAGGTCGTATTAAT
CGTCTTCCAATAAAAGCGTCGCAATGCAGTATTATTTACGGGCAATGCTCTGAGATTTGTGGAGTAAACC
ATAGTTTTATGCCAATTGTTATTGAGTTCATTCCTGAGAAATATTTCGTTATATGGTTGGAAGCTCTTAATT
AA------------------------
ATGAAACGTAATCCTTATTATGTTCCTGGCCCAAGACCATGGCCATTTTTTGTAGCTATTTCTGCTAATGG
TATGGCGGTGGGGTTAATTTTGTGGCTGCATCGTACACCTTTTTTACTTATAGGGAGATTGGGGTGCATAT
TATTAAGTACCTTTAGTTGATGACGTGATTTGATTCGGGAAGGAGATATGGGGCTCCATACTCGTTTTGTC
ATCAAAAGATTTCGGGATGGGGTGGCTTTGTTTATCTTGTCTGAAGTAATATTCTTTTTTACATTTTTTTGG
ACCTTTTTTCATAACGCTTTGAGTCCTTCTTGTGAGCTGGGAATGCGGTGACCTCCTCCTGGAATCCGGA
CACCAAACCCTTCATCTACGAGGTTGTTTGAGACGGGATTATTAATTAGAAGGGGGTTGTTTGTGACTCA
AGCTCATAAGAGTATGCGCTTGGACTATGATGTGGGGCCTTTTGTTGGACTAGTTGTGACAATTTTGTGT
GGAACGTTGTTCTTTTTAGTTCAATTGCGAGAATATTACTGAAATTCGTATACCATCGCGGATAGCGTATA
CGGAAGAGTGTTTTATCTTTTAACTGGTTTCCATGGGATACATGTGGTTGTGGGGACACTGTGGTTAATG
GTGAGACTGATTCGCTTGTGACGAGGGGATTTTTCTAGTCAACGACATTTTGGGTTTGAGGCTTGTATCT
GATATTGGCATTTTGTAGACGTAGTCTGAGTGGCGCTGTGATGTCTAGTGTATGTGTGATTTGGGGGGCC
GTGACGAAAGACTAATAAGCTGGTAAAGATCATGAATGATAGATTCTACGATCTTCCGTGCCCTGTGAAC
TTAAACGCTTGGTGAAGATTTGGGTCTATACTTGGGCTGTGTTTAGTAATTCAGCTTCTGAGAGGGCTTT
TGCTATCTATTCATTATACGGCTCATGAAGATATGGCATTCGACTCTGTTGTGCACATTATACGCAATGTGA
AGAAAGGGTGAATGTTACGGAATATCCATGCTAATGGGTCTTCTATGTTTTTTATTTGTCTCTATGCCCATA
TTGGCCGAGGGCTTTATTACGGATCGTATTTAGATAAAACGGTTTGATACTTTGGGGTCCACTTATTCTTG
TTGACTATAGCTGAAGCCTTTCTGGGGTATACTCTACCATGGGGGCAGATATCTTACTGAGGGGCTACTG
TGATTACGAATATGCTTAGGGTAATCCCGGTGGTTGGAGAAAGAATACTCCGGTATGTGTGAGGAGGCT
GAACAGTGTGTAACGCTACGCTTAAGCGGTTTTATACCCTTCATTTTTTGCTCCCATTTTTGATAGTTGCG
GTTGTTTTTCTTCATCTTTTTTTTTCTACATGAGAAGGGGAGAAATAATCCGTTGGGGATTGAAAGAGACA
CAATATGCGTCCCTTTCCACCCTTTTTACACTGTTAAAGACTTGTTTGGGTATGTATGCTTTAGATTCTTTT
TTATATACCTGGTTTGCGTTGATCCTGAGCTCTTAGGCAATCATTTAAATTATTGGCCCGCAAACCCTATA
AAGACTCCAATTCATGTTCAGCCAGAATGGTACTTTATATTTGCCTACGCGATTCTTCGGTCTATTCCGCA
TAAGGCGGGGGGAGTATACATTATGTTTTTATCCATTGTAGTGTTGTACTTGGTCCCGAGTCTTCACAGAG
GTAAGTATCGAAGATTGTGTTTTTACCCGTTTAACCAAGTAGTGTTCTGAGTCTTAGTGGGGAGGTTTAT
TAGACTAACGTGAATTGGGGCCCGTCCGGTTCGTGAGCCTTATATTGTGATGGGGCAGTACTTTTCTGTT
ATCTACTTTTCAAGGCTTCTACTGAATCCGCTTTCTCTATGGATGTGGGATAAGTTGTTAGACGTGGGAGT
GGTGGTTGGGATTGTCCCTTTTATTGGAGTACTTCTTGCGGTTGGGTTTTATACGTTGTTGGAGCGTAAA
ATTTTGGCTATTATTATAATTCGTAAAGGGCCCGCTAAAGTCAGGTATATGGGGATTCTGCAGCCATTCAG
TGATGCCGGAAAACTTTTATGCAAAGAGTTTATTACGCCTACGCGCGCTAACGCAAGACCTTTTGTGTTG
TCCCCTGCTTTAATATTGACTGTGAGTTTACTTGGATGGCTACTATACCCTTATAAATCAGCAGAAGTGGC
TTATGTTTTTGGGGTTATCCTTTTTATGGTAATTACAAGAGTCAGCGTGTATGGGGTGATAATGTCTGGAT
GAGCCTCTAACTCAAAATATTCTCTGCTAGGTGCTGTTCGTGCAATGGCCCAAAGTATCTCCTATGAGAT
TCCAATAGGATTTATTTTTTTTTGTGTAGTTTTATGCTCTGGCGTGTTTATGGTCCAAGAAATTAGAGTCG
GGTTCTTCTTTTTTCCGTTGTCTATAATTCTTGTGGTATGGGTTCTATGCATACTGGCAGAAACTAATCGA
GCTCCTTTTGACTTTGTGGAAGGGGAGTCGGAATTAGTGTCCGGCTATAATGTGGAGTATAGCGGGGGC
GGCTTTGCAGTGATATTTATTGCTGAGTACTCCAGAATTTTACTGAGAAGAATAATAAGAGCTGCAATGT
TTTTCGGAGGCAACGAAGCGTGGATGGGTGTTTATATGATGGTTTTTGCGGTTCTTTTTGTAGTGGTTCG
TGCATCTTTACCACGTCTGCGGTACGATAAACTGATAAGATTATGCTGAACAGTGCTTTTGTGTGTTATGC
TTATAGCCAGAGTGTGTGTAGTAATTCTAATCAGCGTATAATGGTAAGTTTTGTAATAAGACCAATAAAAG

TAATGAGAATACTGATAGTTATTTTTGGAACAGGATTAAGGTTGAGAAGAGAAGAGCTAATTGGTGTGT
GACTGGGTATAGAACTAAATTTGTACGGGTTTTTGATTCTTATAAATCCTGACGGCTACTACACACCTGA
GCCCTGTGTAAAATATTTTGTAGTACAAAGCAGGGCATCTATCTTAATGTTAGGGGGTTTTATGTTATTAAT
GCATTTTGTAGTCGGAGGGTTAGTTATAAGGGTAGTGGGCGTTTTATTAAAGTCAGGAGTTTTTCCGCTA
CATTCGTGAGTGCCGTCTACTATTAAAAATAGGAGATGGCTAGCAAGTGGGTTAATATTAACTTGACAAA
AGATTGCGCCTTTGATCTTTTTGTCTGTAATTGCCCCGTTAGGAAGTCTTTGGGTGGTTATCGGGTTAATG
GCTGGTATTGGGGGAGTAGGGGGTTTAAACCAAAACTCTGTCCGTGTGATAAGGGCATACTCGTCATTT
GTCCACACGTCTTGGATATTACTAGGGCTAAGGTGGTCCAGTGTAGTATTTGCAAGGTATTTCGTAGTGT
ATAGAATGTCCGTAGGGATATTTTTTTATGGGTGTTCGCTAATAAATAAAAGAAAAGTGATGAGACAACT
AAGGGGGGCTGCTAGAGGGATAGGTCTCTTAATGCTTATGGGTATACCTCCGTTTCTTGGGTTTGTGGCA
AAGGTGTTGGTTTTTCTCATAACTAGGAGGGGTGTAATTGTAGTGTGTGTTATAGGGTCTGTGGTTAGGC
TAAAATATTATATTGACTTTTTTTATAGAATAGTGATGAAGCATAAGAGAAGGGTTAAGATAATATGGGGT
TTGATAGTAATTGCAAATTTAATAGGTGGGGCGTTGATTGTAATGAGGTTTATCTAAATGGTTATAGGGAC
AAGAGTTTTGTTTGTGTGTATTGTGTCTTTTTTATTTACAGGGCTGTTATTGTTGAGAGAAAAACGGGGG
TTAGACCGTGAGAAATGTAGCCCGTATGAGTGTGGGTTTGAACCCATTGGAAGTGCACGCAGGTCTTTT
TCTATTCGCTTTTTTCTGGTAGCTGTTTTGTTTGTGGTGTTTGATGTTGAAGTGGTGTTGTTGATGCCTTT
TGTGTACATGTTCCTTTATAGTAAGAGGTTACTTGGAATGGTGTCATCAAGAGGCTTTTTATTTATTCTGTT
TATCGGGTTGTACCATGAGTACCGAGAGGGGTCTTTGGAATGAGTGGGATAAATGGTTAAAAGACTGGC
AATTGGGCTTATCGCGTTAATGGTAATGAAAGACCTGAACATATCTATTATTGGATTGAGGGTTTTGACAA
TTATAAGATTGACAATAACAAGGGCAGCCAACGGTAAAATTGAGCTAAATGGGTTATACAGAATTGATTT
CATAATAGGGTTAATAGTCACATTAACTTTATTTATCGCGGTTCTATCTCACTTAAGAAGAATTAAAACGG
TGCGTAAGCCAAGATTCAATCTAATAATTATTGGGATTAGACTAATCCTACTAATGAGTTTCAGGGTAAGT
AGATTCTTCCTTTTTTTTTTTTTTTTTTTGAAAGAGTGTTAGCACCATTACTACTGCTAATTGTAGGTTGAG
GATATCAGCCGGAGCGATTGCAAGCAGGTGGactGTACATGGTGATTTACACTGTTTTTGGATTGTTCTTT
CTATGAGGCGTCAGAACGAGGAGAAGAATGGGTGTGGGGAGACTGGTGAAAAAGAGATATATGGGGCT
GTGGTGGTTATATATCCTAGGATTTTTGATAAATTGCCCATATACCCCTTCCATTTGTGATTGCCTAAGGCT
CATGTAGAGGCTCCTGTAGCCGGGTCAATGCTACTAGCTGGTGTGGTGTTAAAATTAGGGGGGTACGGA
TTATTACGGTTTATGAACTTTATGCAGCTAAACCTAAGAAGGGTGTCTTTGCTGTTACTATTTGTTAATTTA
GTTGGTGGGGCCTATGCGGGGATAGTGTGTGTTCGTCAAGTAGACTTGAAATGTTTGGTAGCGTACTCGT
CTGTGGCTCATATGAGATTGGTGTTACTAGCCCTAAGAAACACGCCAGTTGGAGTAATGGGAGCTATTAT
TATTATAGTAGGGCATGGGCTTTGTTCATCTGGGTTGTTTAGGTATGTGAACGTGGCGTACAAGATAAGA
CACTCTCGGTTGTTAGTAATAAATAAAGGCGGGTTATTAATTTGCCCGAGGCTGGTGCTGGTGTGTTTCT
TACTGAGGTCTAGAAATATGGCTGCTCCACCAAGCTTAAACTTATTCGGGGAAGTTTTGGTTTTTGGGGT
GAGGGCGTGAATAAGGATAGTATTTTTGTGTATTTTAGGGCTGATGAGGTTTATTAGAGCTTGTTTTAGTT
TGTATCTGTACAGAAGTTGTTGCCACGGTAAAGGGTTAGTGTATAGAGAGTCTTTGAGACTACTTTGTGA
CGCAATGGTTTTAGGAGCCCACTGAATACCTCTAAACCTACTGTTTTTATTTATACCTTAGCGTATAAACA
CACACCTTCTTTCTTTGTTTGTTGGTCTTGAAATAATAAGATTAGGGTTGCTTTTTGTTGGACACGTTTTT
TTAATAAATCAATTCTGAGTAATCCTTTTAATCTTATGTTTAGCTGTGTGTGAAGCTAGAATTTGTCTGGCT
TTATTAGTTATAGTGATGCGGTTATGCGGTAATGACTTAATGTCAAGGTTAGTGAGAGATGCAAACTATTG
AGTGAAAGTTTTTTGCTTTTGTTGTTAATCTGTGGGTACGTGTCAATCTTTGCAGGCAGGTTGAGTAAGG
TTTATTTGTTTGAGGTGTGTATTTGAGACAGGCATTGTTTGCCCTTTAGATTTAGAATTTTATTGGATAGA
GTAAGAATAATTTTTATTGGGACAGTTTTAGTGATCAGGGGGAGAGTGGCTACTTACTGTAAATGATACA
TATCAAGAGAGGTTTACTATAACCGTTTTATGGGGCTGGTTTGATTGTTTGTGCTTTCTATAATCTTCATGA
TTTTGATCCCTAATCTAGTAATGCTACTAATCGGGTGAGATGGCTTAGGTCTTACTTCATTTTTATTAGTGG
CTTACTATCAAAATAATAAAAGACTCTCGGCAGCCATATTAACAGCTTTGACTAATCGTGTGGGGGATGT
TTTTGTTTTAGTTAGGGTTTCAATTCTTTTGAATGAGGGGAGATGGCTAATTTATGACTATCACCCTGTGT
GCATATGAGCTCTGGGAGGTGTTGTTGTTCTTGCAGGTATGACTAAAAGAGCCCAAATACCTTTTTGTGC
TTGGCTTCCAGCTGCTATAGCGGCTCCGACACCGGTATCTTCTTTAGTTCACTCTTCTACGCTGGTAACA
GCAGGGGTGTACTTAGTACTTCGTTCGTTTTATGTAATTAAAATGGATGTTATGCAAATATTAATGGTTTTT
AGGTTATTCACTTTGGTATTAGCGGGCTCTAGGGCGGTTTTCGCATTTGATCTAAAAAAAGTGATTGCGC
TTTCTACTTTGAGTCAGCTAAGTTTGATGATGTTTTCTATTTCTATTATGTTGCCTTTTGTTGCTTTTTTTCA
TTTGGTTACACATGCGGTGTTTAAAGCTCTTCTTTTTTTAGGAGCTGGAGGGGTTATTCACAGGAACCAG
AGTACTCAGGATATTCGGAGGTTAAGAAGGTTGTGGCAAAGACTGCCTGTGAGAATAAGAGCAATGAG
GGTAGCAATTGTGTCACTGAGAGGTGCTCCTTTCATAAGAGGATTTTATTCCAAAGACTTGATTATTGAG
ATAATGGGTGACAGAACATATGGGTATGTTTTAGAGTTATTAGGTCTTGTTTTTACGTCTTTTTATAGGGCT
CGTGTGTTCAGGGTTATATTAAGGTCAAATTACGTTAACTGTGGGACTTTACGGTCATCTGAGCACATAA
ATATACAAATTCCATTCTTGAGCTTGTATGTTGGTGCTGTTATTTTAGGTGTCAGACTGGGGAAAAGAATG
GAGAAGTTTGGTTTTGTAGTATTACTTGAAAAGTATGAAAGGCTTGTAATTTTTATAATTCCACTTGGGCT
TATATGATGGAGGGTGTTAACTAAATTAAGCTTAAGGCCGTCTAAACGGGGATTTTTTTTGAGAATATGG
CTTGTTGAGTTGACTCACCCTGCAAAGAGTGTGTTTTTTAAGAGCTCGAAGATGGTGTACGAAACTTTA
GATCAGGGTTGGCTAGAATTGTTAGGTCCGCAAGTCGGTCTAAGTAAAATTAGGCAGTTAAATGAGAAC
TATTTTACTGTTGTTTGAGTTGTGTCTGTAAGACTGAGTTTAATTATCTTTATATAAATGAGGGTTATAATT
TTGTGTATAAGTTTGATTGTTGTTTTTGCTTTAATTGCTAAGCAACCAATTTCTCTTGGACTGGTTTTGTTA

GTTGGGTCTATAGTATCATGCGTAGAGATTGCATTGGAAGTTAGGAGATTGTTGGGGTTCTTACTTTTTTT
GACCTATGTAAGGGGGGTTATAGTGCTGTTTTTATACGTATTGAGTATTTATCCTAATGAACGATTTAATTT
AAGATTTATTGTTATTGTAGTAGGATGCTTTGCAGTAAGATTGATGATGATGGTAAACTATGAGAGCGGGT
TTTTGTTTCTTGGGTTTATGGCTGAAAAGAGCCTCTATATTCTAATAGCTAGAGTGTTATTGTTTGTGATGC
TTGTGGTTTCTTATTTATGCATAAAAACTATAGTTCCACTACGGAAAGTG"/>
<sequence id="seq_Gregariella_coralliophagaNC04412" spec="Sequence"
taxon="Gregariella_coralliophagaNC04412" totalcount="4"
value="ATGATAATAGATGTTTTTTCGAGGTTTGATGGGCATAGATTTAATTTCTTTGTTGGAGCTATACTGT
GGTTATTTAGCACTTTAACTCCGTTTATAGTAATTTTTGGTGATGTGACAGTACGTAACTTTTTGTTGGTTT
TTAGCTTTAGTATATTACGTAATGGTAAAGGTCTTATAATTGCTGGGTTGCCGCTTGGGGTGAGAAGGTTA
TTTTTATTAGTGCTTATATTAAATTTGTCTGGAAATGTTCCTTATTTCTTCCCAGTTAGAGCTCATTTTGTAT
TTGGATTTTCATTTGCATTTTCGTTTTGAACTTGCTTGGTAATTTCAGGTATAGTTTCTAGGTTTGAGCAA
AGATTTATAAGACTAGTCCCTAGAGGACCTTTAATTTTAATTCCGTTTATAGTAGTGGTTGAAATTTTTAG
GGGTATGTTACGCCCATTAACATTAGTTTTACGGTTATCATTGAATTTATCTGCTGGTAAAGTAATTTTATC
TTTACTTGGATCAGGGTTATTATCTTCTTTATTAATTTCAAGCTTTTATAATATTTTTGGTGTAATTATTAGA
GGTATTTTTGTAATAGAAATTATTATTGCTTGTATCCAATGCTATATTTTTTGTGTGTTAATTACACTGTATT
CAGGGGACCACAGGGACTAACGTTGGCTGTACTCTACTAATCATAAAGATATTGGTACACTATATCTTCTT
AGAGGGGTGTGGGCAGGGCTAGTTGGTGCAGGTTTAAGTTTAATTATTATGCAGAGTCATCCTAACAGA
ATTTTTTTGAAAGATTGATTTTATAACGTTGTGGTTACTACTCATGCATTAATGATGATTTTTTTTGCTGTG
ATACCAATCTTAATTGGTGCATTTGGTAATTGACTAATTCCTCTTTTAGTCGGTGGTCAAGATATAATTTAC
CCACGAATAAACAACTTAAGGTACTGGCTATCTCCCAATGCCTTGTATTTATTACTCCTTTCTTTTAGGAC
AGATAAAGGGGTTGGTGCAGGATGAACTATTTATCCTCCTCTATCTGTGTACCCTTATCATAGAGGGCCTA
GAATAGATGTTTTAATTACTTCTCTTCATATGACTGGATTAAGATCTTTAGTTGGAGCTATTAATTTTGCTA
GAACAAATAAGAATATGCCAGTATTAGAAATAAAAGGAGAAAAAGCAGAGTTGTATATTCTTAGAATTTC
TATCACTGCAGTTTTATTGATTATTGCTGTTCCTGTATTAGGTGGTGGAGTTACTATAATTTTGTTTGATCG
TAATTTTAATTCTACTTTTTTTGACCCAGCAGGTGGAGGTGACCCAGTTTTGTTTCAACATATTTTTTGGT
TTTTTGGTCATCCTGAAGTTTATATTTTGATTCTTCCTGCTTTTGGTGTAATATCAAAAGTAATTATACATTG
CGCTGGTAAAGAAGCTGTCTTTGGTTTAATTGGAATAGTGTATGCTATAATTGGTATCGGAGGTTTAGGGT
GTATAGTCTGAGCTCATCATATATTTACTGTAGGGTTAAATGTAGATACTCGTGCTTACTTTTCTACTGCTA
CTATAATTATTGCTGTTCCAACTGGTGTTAAGGTTTTTAGGTGGTTAGCTACTATTGGAGGGAGGCGGTTT
AAATTCAAGCCTTCTGGTTGTTGAAGAATTGGATTTTTATTTTTATTTACTGTAGGAGGTTTAACTGGGGT
TATATTGGCCAGCTCATCTATGGATGTGTCTATACATGATACCTATTATGTTGTAGCTCATTTTCACTATGTT
CTAAGGATAGGGGCTGTATTCGGTATCTTTTGTGGGCTTAATCAATGATTACCTAATTTTGTTGGAGTAAA
CTTAAATAAAAAATGAAGAAAAAGTCATTTTTCTGCTATGTTTTTAGGGGTAAATACAACCTTTTTTCCTC
AGCACTTTTTGGGGCTTAGAGGAATACCCCGTCGTTACAGGGATTATGCTGATGTTTATGCGCGGTGGCA
TTGGGTTTCTTCTTATGGGTCTATTGTGTCTTTTGGTTCACTTATATATTTTAAATTTTTATTGTGAGAAGCT
TTAGTTAGTCAACGAGGAATGTCATTTTATGGAAGGTGATTTTTTGGTGATATTATTCAGCAGCAGGGGC
GAGATATTATTGCTTACCATGGATTTGTTATAATGGTAATAATTGCTGTTTTAGTAATAGTACTTTATATAGG
AACAGTAGTTTTAGTTAGAAAAGCTACTTATCGTTATTTTTTAAATCGTCAACGTCTTGAGTTCTGATGAA
CTTTGATTCCTATGATTTTATTGACTGGGCTGTGGTTCCCTTCAATAAAAAATTTATATGTGGTTGATGAAG
TGAAAACGCCGCGCTGGAATTTTAAAGCTGTTGGTAAACAATGGTACTGGTCATATGAGTGCCACCCAA
CGCGAATTATTGACTCTTATATAATAAGAAAGGGGGAAGTAGGTTATCGTTTGTTGGATGTAGACAATCG
AATAGTTGCTCCTGCAGGGGTTCAAATAACTTGCTATGTTACTAGTTCTGATGTGCTTCATTCTTTTGCAT
TGCCAAAATTGCTTCTTAAAGTAGATGCTATTCCTGGGCGAATCAATCGCTTACCTATAAAGGTATCTCAA
AGATGTGTGTTATACGGCCAATGTTCGGAGATTTGTGGGGTTAATCACAGGTTTATGCCAATTGTTATCGA
GTTTATTCCAGAGAAATATTTTGTTAAATGGATAGATGCTCATTTGtaa-----------------------
ATGAATATAAAACCTTATTATGTTCCTGGGGCAAGACCTTGGCCTTTTTTAGTAGCAGTTGCGTGCAATG
GTATGTGTGTTAGTTTAATCTTATGGCTGCATCGTACTCCATATTGACTAATGGGTAGGTTGTTAAGATTAG
GTTTAAGCTTGGTCAGATGGTGACGTGATTTGCTGCGTGAAGGGGATATAGGGTTACACACACGATTTGT
AGTAAAAAGATTTCGTGATGGTGTTGCTTTTTTTATTTTGTCGGAAGTCATGTTCTTCTTTTCTTTTTTCTG
GTCATTTTTTCATAGGTGTTTAAGTCCTTCTATAGAGCTTGGAGGACGATGGCCTCCGCCAGGGATTCGA
ACACCTAACCCAGTATCTACAGGCTTGTTTAACACTACTCTTTTGATTAGAAGAGGAGTATTTGCTACTTA
TGCCCATAAAAGAATCATTAGAGATTACGATAAAGGCTCTTTTCAGGGGTTAGGATTAACTATTGTGTGT
GGAGTTTTATTTTTTAGAGTACAATTGCGGGAATATTACTGAAACTCCTTCACTATTGCCGATGGGGTGTA
TGGTAGAACTTTTTATATATTAACTGGGTTCCATGGAATGCACGTATTTGTTGGTACTTTGTGATTAATAGT
AAGATTTGGTCGATTATGATGTGGTCACTTTAGAAGACGTCGTCATTTTGGCTTAGAAGCTTGTCTTTGAT
ATTGACATTTTGTTGATGTAGTGTGAGTCTTTGTATGATTGTTTGTATATTTATGATTTGGTGGTCCTTTGC
GTAAGCGTCATGTTTTGTTAAAAATTTTAAATGATAGGTTTTATGATTTGCCATGTCCTGTAAATTTGAATG
TGTGGTGAAGGTTTGGATCAATGTTGGGGTTGTGCCTGGTGATTCAATTTGTTAGAGGGTTACTATTGTC
TGTTCATTATACTGCTCACGAAGATATGGCATTTGATTCTGTTATTCATATTATACGAAATGTAAAAAAGGG
TTGAATGCTGCGAAGAATCCATGCTAACGGTGCGTCAATGTTTTTTATATGTATTTATGTTCACATTGCAC
GTGGAATTTATTATGGATCTTATTTAGATGTACCGGTATGAAATATTGGTGTATTATTGTATTTTATGGTAAT
AGCAGAAGCATTCTTAGGGTATGCGTTGCCTTGAGGGCAAATATCTTATTGGGGTGCTACGGTAGTTACA

AACATGCTTACTGTAATTCCTTTATTTGGGGAGAAACTATGCTATTACATATGAGGTGGGTGAACGGTGTG
CAACGCTACGTTACAGCGGTTTTATACTCTTCATTTTATACTTCCTTTTTTAATAGTATGTGTGGCTGTACT
ACATCTTTTTTATCTTCATGAGAATGGAAGTAACAATCCTTTGGGTATTGAAAGGGACACAATATGTATTC
CATTTCATCCTTTTTATACTGTTAAAGACTTATTTGGTTTTGTCTGTTTTAGATGGGTGTTTATATATCTAGT
ATGTGTTGAGCCTGAGTTATTAGGTAACGTGCACAATTATACTCCTGCGGACTCAATAAAAACGCCTCTT
GATGTTCAGCCTGAGTGATACTTTGTTTTTGCTTACTCTATTTTGCGCTCAATTCCTCATAAGGTGGGCGG
TGTTGTTGCCATAGGGGGCGCTATTGCTGTGTTATTAGTAATCCCTGTTATACATACGGGCGAGTTTCGTA
GTCTGTGTTTTTACCCATTTAATCAAATATTGTTTTGGTGTTTAATTGGTAGTTTTATTGGTTTAACTTGAG
CCGGCTCTCGTCCTGCACGAGAACCTTTTATTAGAATGGGGTTATGATTCTCGTGCTCTTACTATGTGTGT
ATTATCTTAAATCCTTTAAGTATGTGAATATGGGATTATCTACTTAAATTATCAGTGCTCGTAGGAGTAATT
CCTGTAGTAGGAGTGCTGTTGGCAGTAGGGTTTTTTACTCTTCTAGAGCGTAAGTTGTTAGCTATTATCAT
AATTCGAAAAGGTCCAGCAAAAGTTAGTTTTATGGGCATTTTGCAGCCGTTTAGAGATGCGGGCAAACT
ATTTTGCAAAGAATTTGTTGTACCATCTCGCGCAGTAATAGCCCCCTTCATTATGTGTCCAGGGGTGATAT
TACTGATCAGATTAGTGGGGTGGCTGTTGTACCCGTATAAATGTGTAGAAGTAGTTTATTTAGCAGGTATT
ATTCAATTTATTGTTGTTGCTAGTATTAGAGTTTACGGGGTTATAGTTGCTGGATGGGCATCTAACTCTAA
ATACGCTCTTTTGGGGTCAGTTCGTGCAATAGCACAAAGAATCTCGTATGAAATTCCTTTAGGTTTTGCG
GTAATAGCAGTTTTATTTGTAGTCAGTTCTTTTATGCTACAAGAAATTAGAATAAGGTTTATTATACTTTTG
TTTCCTGTAATTTTTATTATTTGAATTTTATGCATATTAGCTGAAACTAATCGCGCGCCGTTTGATTTTGTAG
AGGGGGAGTCTGAATTAGTGTCTGGGTTTAATGTTGAATACAGTGGAGGGGGGCTTTGCAATAATGTTTAT
GTCTGAATACGCAACTATACTTCTAAACAGGTTAATTACAGCAACAGTGTTTTTAGGCGGAAGGGAGCT
ATTTATAAGAGTAGCTATAATACTGTTTGTAGTAGTATTTGTATGAGTACGAGCATCGTTGCCGCGAATAC
GGTATGATAAATTAATAGGTTTGTGTTGGTCTGTTCTATTATGTGTAGCAATAAGCGCGTGTGTGTTTTACT
TTGTTTTAAGAAATTAGtaATGTTTTTTAGTTTATTAAGCCCTATAATGATGTTAAGCGGTTTTGCAGTATTT
TTTGGGGCTATTATTAGAGTCAGAAGTATAAGGTGAGTGGGGTTGTGGGTCGGAATAGAACTAAATTTGT
TTGGTTTTTTGATTTTTATAAATTTTGATGGAGTCAGAGTCCCAGAGCCGTGTGTTAAATATTTTATCGTTC
AAAGAACCGGGTCTATGTTTTTGGTTATAGGATTTTTGGGGGTAGAATTTGCTTCAGTTATGACAATGTTT
TTAATTGTAAGTGGTGCTACTTTAAAGGCAGGGGTGTTTCCTTTCCACTCTTGAGTGCCTTTAGTGGTTA
AGAATAGAAGATGAATGAGCAGAAGGTTAATTTTAACTTGGCAAAAATTAGCCCCTTTAATAGCAATAGC
TATTGTTATATCTAAAATTTTCCTTTCTATCTTAGTTTTTTTCATAGCATTAATTGGAGGTATCGGTGGATTA
AATCAGTTGTCTATTCGCTTGATAAGAGCCTATTCATCTTTTGTTCACACATCTTGGATATTAGCTAGCTTA
TTAAATTCATTAGTTGTATTTGTTTTCTATTTTTTTATTTATATACTATCAGTTTTATGTCTGTTTTGATGTTG
TGCTAAAATTAATAAATATAGGGTTGTAAGGAGTGGTTTTAGCGCTTCTGCTAGAGTTTGTTTAATTATAC
TGAGAGGGGTGCCCCCGTTTTTAGGATTTTTAGGCAAAATTTTAGTATTTTTATCTGTAATAAGTATAGAA
ATTTTTCCATGCATTATTGGGTCAGTTATTAGACTAAAGTTCTATCTTTCTTTTTTTTTATAGAATAGTAATAA
GATATTTAAGTGGAGCTGAAAAAATTATCACCACTTTAGTTGTTATTAATGTTGTCGGGTTAATAGTGGTT
TTTATGATTTTTTAAtaaATGGTATTGTTTTCAATTGTATTATTTATGTTAATTTTATCCACTATTTTCACAGTA
ATATTATTTTTGTCTGAAAAAGGTGGAACAAATCGAGAAAAAAAGAAGCCCTTATGAGTGTGGATTTGAG
ACAATTGGAAGAGCTCGTAGTACATTTTCTGTTCGGTTTTTTTTAGTTGCTGTGTTGTTTGTAGTGTTTGA
TGTGGAGGTAGTGTTAGTTATTCCTTTAATTTATATAGTTTGAACAATAAAAAGAGTTTTAGGCATTTTAG
TTGGTCTCATATTTATTTTTGTCTTGTTTTTAGGGTTATTTCATGAGTACCGAGAAGGGTCTTTAAATTGAG
TTTAAtaaATGACTATATCTTTAATTATTGGCACTATTATTTTGATAATAATCGGTAAAATAGAAGCAGCAATA
GCCGGATTAGTTTTACTAATATTTATTATAGTATACGGAGTAGCAAATGCAGCAGAAAGTGTAGAAATCAT
AGGATGCTTTAATTACGACTATGTAGTCTTAATACTAGTGAGATTAACGATGTTTATTATGGTATTATCGTT
AATAATAAGAACAaaaGTAGAGCGGAAACAAGAGTATAACTTGTTTATATTAAGAATTAATCTATTTTTGAT
TATAAGATTTCTTGTAAGAAGTTTTTTTCTTTTTTTTTTCTTCTTTGAAAGAAGTCTATTCCCACTATTAAT
AGTGATTGTAGGGTGGCGACTACAAGCTGGGGCTTATATAATTATCTATACTGTTTTTGGCTCTCTTTTCTT
TTTATTTGGCATTAGATATCTGTTTATCTTAGGGAGTGATAGTATAATTATAGTTTCATTTGTTAATAAAAGA
TTATTAAGATTGTGGTGGTTATTTATTTTAGGGTTCTTAGTAAAGCTTCCAATATACCCCTTTCACTTATGG
CTTCCTAAAGCTCATGTTGAGGCGCCAGTAGCTGGCTCTATGGTTTTGGCAGGAGTCGTGTTGAAATTAG
GCGGTTATGGGCTGTTACGATTTGTGGGGTTGGTAGGCATAAAGAGCTTTAGATTCTCTAGATTGTTATTG
CTAGTTTGTTTAATAGGAGGATTTTATGCGAGACTTGTTTGTTTGCGACAAGTGGATCTAAAGTGTCTTG
TCGCTTATTCTTCTGTGGCTCATATAAGTTTGGTTCTATTGATCATTAGAAACAGTGATCTAGGGGTAATA
GGTGCCCTTCTTATTATAGTTGGCCATGGTTTATGCTCATCTGGGTTGTTTGGATACGTAAACACTATTTAT
AAGGTTTCTAATTCACGTATGTTAATAATAAATAAAGGAGTGTTAATTATTTCTCCGGTAAGAGCTATGGT
ATGTTTTTTATTGAGGTCTAGTAATATGGCTGCGCCTCCTAGACTAAATTTGGCAGGAGAGGTATTTATTT
ATGGCGTAGCTTCTTGAGTAAGTAAAGTATTATTAATATGTGTCATATTAATTAGTTTTATGAGGGCTTGTT
ATAGTTTGTATTTTTATAGGAGTTGTAGTCATGGAAAGACTAGTGGCTACAGCGTATCAAATTTTTTTGAG
TGTGAGATGGTTGTACTTTTGATACATTGATTGCCTTTAAACTTTTTGTTTTTGTTTATTCCTTAACGAAAA
AATGCCCATTTAATAAGCATTTTTATTGGTATTGAGATAGCTGGATTAGGAGTTATTTTTTTATCTACTTTTA
GATTAAGGCATAATGTTTGACTAATTTTTTTGATTGTATGCTTAGGGATTTGTGAAGCTAGATTATGTTTAG
CCTTGTTAGTAATGGTTATACGTTTGTGTGGAAATGACTTAGTAAAAAACCTTAGATTAATAAGTTAACTT
TAAAAAAAAATTTGATCTTTTTATTTGGTGTTACTGGGGTTTTTTGTTTATTGTTTAGCAGAATACCCAATT
CAATACTATTTCAAGCTAGTGTGTGAAAAAGTAATTGTGTGGATTTAGATTTGAGCTATTATTGGATAGA

GTAAGTGTTTTATTTGTAGGGACTGTACTAATTATTGGGAATAGAGTAATGATTTACATAAAATGATATATA
AGTAATGAGTTATTTTTTTCTCGTTTTAGATGGCTTATTTATTTGTTTTTGATCTCTATAATCTTTATAATTAG
TATTCCTAATTTAATCATACTACTTATCGGATGAGACGGCTTAGGTTTAACATCTTTTCTTCTTGTTGCTTAT
TATCAAAATAATAAAAGATTATCGGCTGCTATACTAACTGCTTTAACTAACCGCATTGGTGATGTGTTAAT
TTTAATAAGTATTGGAATATTCATTAAAGAGATAAACTGAATTATTTATGAGTATTCTCCTGTAGTTTTATC
AGGAATGTGTTTAGCTTTAATCTTAGCTGGGATGACTAAAAGAGCTCAATTTCCATTTTGTGCGTGACTA
CCAGCTGCTATAGCTGCCCCTACTCCTGTTTCATCTTTAGTTCATTCATCTACGTTGGTTACAGCTGGTGT
TTACTTATTATTGCGTAGGTTTGATGTTTTAATTTGTAATAGACTGTGAATGCTAAAATTTTTAAGATTATTA
ACTTTAATTTTAGCTGGATCTAGGGCTTCAGTTACAGTAGATTTTAAAAAAATTATTGCTTTATCTACATTA
AGTCAGCTTAGTGTAATAATATTAGCTGTTTCTATAGGTCTCTCTTATGTAGCGTTTTTTCATTTAGTTACT
CACGCAGTTTTTAAAGCACTTCTATTTTTAAGGGCTGGTAGTGTAATTCATAGATACCGGGGGGTTCAAG
ATATTCGTTTTTTAGGTAAATGTTGACAGGAGTTACCCATTAGAATGAGAGCTATATTAGTCTCAATAATAT
CCTTGGTTGGGGCTCCTTTTTTAAGAGGATTTTATAGTAAACACTTAATCATAGAGATAGAAGACGAATG
TATTTTTATGTATATAATAGAAATAATTGGATTAAGCTATACTTCATACTATAGGAGACGAGTGTTTAGATCT
ATAATCGGCAGAAATAAGTCGGATTTAACAGTTGTCTCTGTAAAAGAGGATATTTTGATTATTATTCCTTT
TTTTATTTTAGGCGCTGGAGCTTTGTTAATTGGAGATATTATAGCTAATAAAAATGAGCTTTTTTTCTTTGC
TGTATTTTCGTCTTCTATTGAAAGGTTGTTAGCTAATATTATTCCATATGGTATAATTATATGAAGAGTAATT
AGTTATTCTGCAGCATTTTATAATAAAATAATGTTTTTTTTAAATATATGACTGGTAGAATTAACTCACCCA
GCTAAAGCAATACTATTTAACTATTCTTTTATAGTTTATCGAGTTTTAGACTATGGATGATTAGAACTAATT
GGGCCCCAATATTCATTTGGAAAGTTTTCTAAGATTAATGAAGGGTACTTTATGGCGTTAAGTCTTTTTGC
AACTATTATAATAATTTTTTTGTTAGTGTAAATGTGAATAATATTAATTTCGTTTGTGTTATTGTTTATTTCTA
GGCTCCTGTTTAATCAGCCCATTTTACTGGGATTTATTTTAATCATAAGGTCTATGCTAGTATGTTTTTTTAT
CGCTGCTGAAATTAGAAGCGTTTTAGGTTTTATAGTTTTTTTAACTTATGTCAGAGGGGTAATAGTTTTAT
TTTTGTATGTGTTAAGAATCTACCCTAACGAAGCTTTCTACATAAAAAATTAGTATTACCTTGGTAGCCTGT
TTGGTGGTTAGACTAGGAACATATTTAAATCGTGATAGAGACTCTTTGTGACTCTCTTTTATATCTAGTGG
GGTGATTTACTTATTTATAGCGTTTATTTTATTGTATGTTATAGTTATTGTTTCTTATTTATGTATAAAAAAAA
TAGCACCGCTTCGGTCCTTA"/>
<sequence id="seq_Modiolus_kurilensisKY242717" spec="Sequence"
taxon="Modiolus_kurilensisKY242717" totalcount="4"
value="GTTATATTAGATTTGCTTTCTAGGTTTGATtctttTCTTTTAGAATGAGGATTTCCTGCGGGTTGTGGG
TTAGTATGGGAATTAGTTTATTTGTGCTTGTTGGTAGAGATAAAGGGCTTAGAGAGATTATTTTTCATAAG
GTCTTATCTATTTTAAGAGGTATAACTGGTCAATTTGTATCGGGGTTTAACATTAGTGTTATTAGTCTGTTT
GTATTTTTAATTATAGTAAATTTGTTGGGATTGGTTCCTTTTTCTTTTAGAGTTAGATCTCAAGTAGGATTA
GGGCCCTCAATAGCATTTTTTATGTGGTTGTGTTTGTGATTATCTGGATTGCGTGTGTCGTGGCGTCAAAC
TTTGTGTACATTAGTGCCTAGATATCCCATATTTTTAATTCCGTTTTTATTGTTGGTAGAGGTGGTAACAAT
TAGTTCTCGTCCTGTTACATTGGGATTGCGACTAATAATCAACTTAACTGCTGGTCATTTAATTATAGGTAT
GTTAACTAATGTAAATACAAATGTTGTTCTTTGTTCTGCCTATGGGTTGCAGTTTTTAGGGCTTATTGCTAT
GAGTCTTAGTGCTGAAATGGTTATCGGGGGGTTGCAAGCYTTTATTTTTTGTACTCTTTTAGCGTTATATA
GAAATGAACATCCTAGGTAATGATCGGGAATAGTGGGGATTGGTTTGAGAATACTGATTCGAATTGAGTT
AGGTCGTCCTGGAAGAggaTTTTTAGGGGATGATCAGCTGTATAATGTTGTCACTCACGCATTGGTTATAA
TTTTTTTTATAATGCCTTTGATGGTAGGGGGGTTTGGAAATTGACTTCTTCCATTAATAATGGGCTCTGTG
GATATAATTTTCCCACGACTTAATAATTTGAGATTTTGGTTTCTTCCTTCTTCATTATTTATGCTATTAAGGT
CTACTTTTATTGAGAGTGGCTCAGGGACTGGATGGACTCTTTATCCTCCTCTYTCTTCATATACTGGACAT
AGGGGCCCAGCTGTTGACATATCCTTATTTTCTTTACATTTGGCAGGTGCTTCTTCTATTGGTGGTTCTATT
AATTTTTTGACTAGTATAAAAAATATGCCGGTGGAGGTGATGCGAGGAGAGCGGATAATGTTGTTTTTGT
GGTCTATGGTGGTAACAGCTGTTTTACTATTAGTTTCTTTGCCAGTTTTAGCTGGTGGAATTACTATGTTG
ATTTTTGATCGTCACTTTAATACCTCTTTTTATGATCCTGTAGGAGGGGGAGACCCAGTTTTGTATCAACA
TTTGTTTTGGTTTTTTGGTCATCCTGAGGTATATGTACTTATTCTTCCTGGGTTCGGAATAGTGTCTCATGT
TGTAGCACATTGTGCTGGTAAAGACGAGGTCTTTGGGGTTTTAGGAATAATTTATGCTATAGTGTGTATTG
GAGTGTTGGGCTTTATTGTCTGGGGTCATCATATGTTTACTGTCGGAATGGATGTTGATTCTCGGGCATAT
TTTACGTCGGCCACAATAATTATTGCTGTGCCTACGGGTGTGAAGGTTTTYAGTTGATTGGCTACACTTA
ATGGGGGAAGCTTATTGATTGAGACTGCTTTATTATGAGCTGTGGGGTTTATTTTTTTATTTACGGTAGGA
GGTCTTACTGGAATTATACTATCAAACTCTTCTCTTGATGTAGCTATACATGACACTTACTATGTAACAGCC
CATTTCCATTATGTGTTATCGATGGGAGCAGTTTTTGCCTTATTTTGTGGGTTTTTTCATTGGTTTCCTTTAT
TCTATGGTTATTGTTTTCATGAGCGGTGAAGAAAGGCTCATTTCTTTATAATGTTTATTGGAGTAAATTTAA
CTTTTTTCCCTCAGCATTTTCTTGGGTTGAGAGGGATGCCACGTCGTTATTCGGATTACCCAGATTGTTTT
ATGAAATGGCATGTAGTGTCATCATGGGGGTCTTTATTAAGGTTTGTGAGGGTTCTGTATTTTCTTTTTATT
GTATGAGAGGGTCTATTGAGTCAGCGTGGTGTTGTGTGTAAGAGTAATCGCCCTGGAGCAATTGAGTGA
AAAATGTGATGCTGTCCTCTTATGCCGTTGTGAGGTAGAAAAGGTTTTCAAGATTCTTATTATCAAGTAG
GTGAATATCTAAGCTTCTTTCATGAAGGAGTTATATGTTTAATTGTTTTTATTTTATCTGTTGTTTTATATGG
TCTTAGTTGAGTGTTTAGAACAAAAAGAAGTTACCGGTTTTTACGTGAGGCGCAAGGTGTAGAGACGG
CATGGACCATCATTCCTAGATTATGTTTAATTGGTGTAGCAGTTCCTTCTATACACCTTCTCTATGTAATAG
ATGAAATTGGAAGCCCTTCCTTTTGTTTTAAGGCGATTGGTCATCAGTGGTACTGAAGGTACGAGATGGT

AGATGTTTTAGGGTTTGATTCTTTTATAAGCCGTAGAGAAGATGACGGCTATCGACTATTAGATGTTGATC
AGCGAATGGTAGCGCCGTCTAACACTGGGATTCGTTGTATGGTTAGTAGGGCTGACGTAATTCATTCTTT
TGCTATCCCTGGGTGTATGTTAAAAGTAGATGCAATTCCAGGGCGTGTTAACGAAATCCCTATGACTGTG
GCTATAAGGGGGGTTTTGTATGGTCAATGCTCAGAGATCTGTGGTGCTAACCACAGCTTTATACCTATCGT
AGTTGAGTTTATTCCTCCAAGGGTATACAACCGATGGATCAAATCAATCGATGAT------------------------
ATTCCACGAAATCCTTATTATTTAGTAGGTTCTAGTCCATGGCCCGTTTTTACCTCTATTGGTGGGTTATGT
TTAGCAGTTGGATTCGTTTCTTGGTTTCATATACACAGGTATAGTCTTTTGTTTGGGTTGTTAGTTTTAGG
GTTTTCTTTAGGTCAATGATGGCGTGATGTTATGCGTGAAGGAGATTTAGGTTATCATACTTCCTTTGTTG
TAAAAGGTTTGCGTGATGGGTTTATTTTGTTTTTAGTGTCTGAGGTGATGTTTTTCTTCTCTTTATTTTGGG
CTTTTTTCCACATAAGGTTAGCTCCTGATGTGTCTGTGGGGTGTGTYTGGCCTCCTAGGGGAGTAGAGAC
TCTTGATCCTTTTAAAGTTCCTTTGTGTGGTACTACTGTTTTAGTTGGGTCAGGAGCTTCTTTAATATATGC
TCATGCTGCTATTCGGGCTGGTTTAAATAAAGATGCTATTTATGGGACGGGGGCTACTATTTTATTAGGTC
TTTTATTTTCTCGTCTTCAAGCATACGAGTATTATTGAGCTAGATTTACTATTGCTGATAGTGCTTATGGTA
GCTTATTCTATATCATAACTGGGTTTCATGGTATGCATGTAATATTTGGGACTGGGTTTTTAATTGTAAGAT
TGGTGCGATTGTTTCGTTATCGATTTACTCCACGAAATCACTATGGGTTTATAGTGTGTTCTTGGTACTGG
CATTTTGTAGACGTAGTTTGGATTGGGTTGTACGTAGTTGTTTATATTTGAGGTAGTTAGCCATATCGAAA
GAGTCATAGGCTACTAAAATTTGTAAATAATAGTTTGTATGATCTACCAGCACCAGTAAATTTAAGGGTGT
GATGGAATTTTGGATCTATATTAGGTTTATGTTTAGTTATTCAAATTGTTAGAGGGTTGGTTTTGTCACTTC
ATTACACAGCTCATGTTGATATAGCGTTTGATGCAGTAATTCATATTGTTCGTGATGTAAATAAGGGTTGG
ATGATTCGTAGTATGCATGCAAATGGGGCATCAATATTCTTTTTATGTATTTATGCTCATATTGGGCGTGGT
ATTTACTATGGGTCTTATAAGTATAGGGAAGTTTGAAATGTGGGGGTAGTTCTGTATTTGTTGGTAATGGC
AACGGCTTTTTTAGGATATGTTCTTCCTTGGGGTCAGATATCCTATTGAGGTGCAACGGTTATTACGAGTT
TATTAACGGCTATTCCTTATGTAGGGGAGATATTAGTTCATTGAGTTTGGGGTGGGTATTCAGTTTCTAATG
CTACACTGGTGCGGTTTTATTCTTTTCATTTTATTTTACCTTTTATTATTGCGGCTTTTAGGGTAGTACATTT
ATTATTTCTTCATGAAAGAGGGTCAAATAATCCATTAGGTGTTTCAAGTAATGATATGCTTATTCGATTTCA
TCCATTTTATACATCTAAAGACTTAGTTGGGTTTTTAGGCTTATTTTTTATTCTTATACTTCTTGTGTGTTAT
TACCCGGAATTATTGGGGAATGTAAATAATTGAATTCCAGCCGATCCAATGAAGACTCCACTTCATATTGA
GCCTGAGTGGTATTTTCTGTTTGCTTACACTATTTTACGGTCTATTCCTAATAAAGCTGGAGGAGCCCTTG
CTTTGGTTGTGTCTGTTTTGGTTTTATTTGTTATTCCTTTGCTTCATACTGGAAAGTTTCGAGGTCTTTCTT
ATTATCCAGTAAGTCAGGTTTTTTTTTGAGTGTTGATTAACGTTTGATTAGGGTTGACTTGATTAGGGACG
TGCTTTCCGGAGTACCCATTTGAGGAGATTGGTCGGGCGTTGACATGCGGGTATTTTATTATTATTATTTTA
ATTCCTTTGACGCAAAGGAGTTGGGATAAGTTAATTGAGATGCAGCTGATTAGATTTATTTTGCCTAGGG
TCTTTGCGCTAGTCGCAGTGGCATGGTACACTCTATTTGAACGAAAGGTGCTTGGATATATTATAAACCGT
AAAGGCCCTAATAAAGTGGGTATATTGGGGTTAATTCAACCATTGGCTGACGGGGTGAAGTTATTTTCTA
AAGAATTTATTCTGCCTACATTCAGTAATATTCTTCCATTCATATTGTGTCCAATTGTTACATTTTTTATTGC
ATTGGTATTCTGATTATTATACCCATTTTCAACAGCAGAGGGTGTGTTTACATGCGGATTATTATTTTATCT
GGCTAATTCTGGTGTGAATGTTTATGGGGTTTTAGTGGCTGGGTGATCATCTAATTCCAAGTACGCATTGT
TGGGATCAATACGTGGGGTAGCACAAAGTGTTTCTTATGAGGTTAGGATGGCCCTTACCCTATTAGGGGG
TGTTTACCTAGTGGGTGTAATGAATTTACAGTCTATAAAGTTGGTGTTTGTAGTTGGAGTAATTATTCCGT
TTGTATGCGTATGATTAATTACAATATTAGCTGAAACTAATCGTGCCCCATTTGACTTTGTAGAGGGTGAG
TCAGAGCTAGTGTCGGGGTTTAATGTGGAGTACAGAAGTGTGGGGTTTGCGCTGATTTATATAGCTGAGT
ACGCTAACATGCTTTTTAATAGCCTTTTTACATGTATTATGTTTCTAGGAGTAAGTGATGCCCTTATAAGTG
TAGAAGCGTGCTTTTTTTTTTTTTTTCTTTATTTGAGTTCGGGGGACTTTACCTCGTTTTCGATATGATATGT
TAATAAGCTTAGCATGAAAGAGGTTTTTAAGTTTAGTATTAAGTGGGGTGTTAGTAATTATTTCTTTAGTT
TGTTTGTTATAAGTGTAAATATTTATAACCCCTTTTACTTACTTAGAGGGTTTTTACTAATTGGGGGTATTT
TAGTAACTTTGAGGACTGATTCTAAAATCAGGGGGTGGTTAGGGATAGAAATTAATGTAATAGGGTTTTT
AGGGGTATTAAGAGTTCGCGGTTTTATAAATATTTCTGTTGGCCTGAAATATTTTATTATTCAGGTGCTTGG
GTCAGGCTTATTTTTAATGGGTGTGTTAGTGTTTTATCACTTAATGTTGCAGAGAGGCTGAATAGTAGAG
ATGGGATTATTTTGTAAGGCAGGTATTTTTCCTTTTCATGGGTGGGTTCCATCGGTGATCAATTCAGGGGA
CTGAATTAGTGGATGATTGGTTATGAGGCTTCAAAAGTTGGCTCCTATTATTGTTTCGGTCTGGAGTAGGT
CCgagCTTTTTATTTATATAGGGTTAGTGGGGCTAAGGGTTGTTGGGTCTTTAGGTGGATTAAATCAAATAT
CTGTTCGTGGAATCTTAGCTTATTCTTCATTTGTGCATGGTGGCTGGATGTTGGTAGCGTTAATTCATTCTA
ATGAACTTTTCTTTTTGTATTTTCTAGGCTATATAGTTCAGCTAAGAGTTGTAGTGGGTATTTGTTATGATT
TAGATGTACAGAAAAGAGCAAGGATAAAAATATCTTTTTTGGGGGGAGTAATATCATTAAGTTTAGGGGG
GCTTCCTCCTATAGGAGGTTTTTTGTTTAAACTTAGAGTGTTTCTTTCGGTGAATTTAGGGGTTTTAGTGG
CCCCGGTGGTGGGGTCTGTCGTATCYCTTCTTTTTTACTTGCGCATAATAAATGGGTTTATGTTGGGGAGA
AACAGGTTAGCTAGCTTGTTCTTAGTGTTTAATGGGGTTTGTTATATTGGATTTGGTATAATTTTTGGCTTA
TTCAGTTAAtaaTGGCTGTGGGGTGCTGTGGTAGTAATAGTGGTTCCTTGTATTTTGCTTTTTTTAGGCTTAT
TGGCTATAGGAGATATAAGTagaagtAGTCGAGAAAAGTCTTCTTGTTATGAGTGTGGGTTTGAGCCGATTC
GAACTGCTCGAAGGAGGTTTTCATTGCGATTTTTTCTTTTAGGTGTTTTGTTTGTTGTTTTTGATGTAGAA
GTTGTAATAATGGTTCCTATCCTATTTGGTGTTAGAGTAGGGGGATCAGCAGTAAGGATTATTTGTTTAAT
TTTATTTATTATTGTGTTAGTGGTAGGATGTTTATATGAGCGGCGTGATGGCTCCATAGATTGGATTAAGGA
GATGGTTTTGAGGGGTGTTGCAGTAGTTTTGTTGTTACTTACTTTAGGCGGTGAGGTAGAGGTGATTTTT

GGTGTGCTAATTTGCTCTGTTCTTtttTTTATACCCTTATTAAGTTGGGAAAGTAGGGTTGAAATGGGGGGT
TTGACTAGGCTAGATTTGGTTGGTGTGGTTATAGTAATTTTATCCTTTTATATCAGTATTTTAATGCTTTTGA
GAAGGATTGGAGTTAAGCGGTTTAATGCATTTAGAAAAGTAATTTTGTGTATTTGTGTGGTTTTAGTGTT
GGCGTTTAGTTTTAGGTCTATATTTTTATTTTATATTTGCTTTGAGAGAGTGTTGATTCCAACTTTGCTTTT
AATCCTGGGTTGACGGGTTCAAGCAGTTAACTATATGCTAGTTTATACTGTAGGTGGTTCTATACCCTTAA
TTTATGGATTAAGAAGTCTTTATTGGAGTGGGACTAGTAGGATAATGTTGCTTGGAAGGTTAGATAAGAG
TGTTATTGCTTTTTCTTGATTGTATGTTCTTGCTTTTTTGGTTAAGCTTCCAGTGTTTCCATTCCACTTGTG
GCTACCTAAAGCCCATGTAGAGGCTCCTGTTTCGGGTTCTATAATTCTTGCAGGTCTATTGCTAAAACTTG
GTGGTTATGGGTTTATCCGTTTGTGTGGGTTTGTGGAGTTTCTTCGTTTAAGGGTTCCAGTAATGGTGTTG
TCTGTAAGGCTATTTGGGGGTGTATTAACGAGGGTAATATGTATGCGTCAAACAGATTTGAAGAGTTTGG
TAGCATATTCATCTATTGGTCATATGAGATTTGTTTTATTAGTGGTTACAAATGTTTCATGAGGAGTTTTAG
GAGGGGTTTTAATTATATTAGGTCATGGTTTGTGCTCATCTGCTTTATTTTCTTTGGTAAATTACATGTACG
GAGTTAGTAGTAGTCGTCTTATTAGCCTAAATAAAGGTTATTTACTAATGTCCCCTTCTTTGTCTTTAGTGT
GCTTTTTGTTGGCGGTTAGAAATATGGCTAGACCTCCTAGATTGAATTTGTTTGGGGAGTTATTAATGTTT
ATAGTAGGAAGTTTATTTAGGGTATTAGTCTTAATTTTACTGGGTTTTATAAGGTTTATGGCTGCATGCTAT
AGATTGTATATTTATGTGGGAACACAACATGGGAAAAGGTGTGGGCTATGGGTAAGAATAAATGGTGTAT
GTAGCGGATTTGTATTATTAGCTCATTGATTTCCTTTAAACTTTTTGTTTTTGtttattcctTAGTCGCGTAGAGA
TCACTTAGTTAGAGTGTTTTTAGGTATAGAGTTTATAGCTTTAGGAGTTTTTTTAATAGCTTTTGTCCCGTC
CATTAACAGYCTTTTTATTCTTCTTGTTTTGTTGTGCATAGCGGTTTCAGAGGCAGCTGTTATACTGTCTT
TTATAGTTCAAGTAACTCGTCTTTATGGGAGTGACCAAGTATCTAGGGTTATAGTAGATAGATTCAGTTTT
CTAAAGTTTTAGGTGTGTTGTTTCTAGCTTTAGGGGCGATGCTTCTTAAGGTAGCAAGTAGTTGTGTAGT
CATCATTTTGGAGTTTAGGCTTTGACTCTCTTATATTTCATCAGTAAGTTTTAGATTGTTCTTTGATTGTAG
TGGGTTATTGTTTATTAGAACAGTTTTACTTATTTCAGGTTCAGTCCTAATTTATTGTAGTTGGTACATAGA
CGATGAGATCTATTATAAACGGTTTATTTTTTTAGTATTACTATTTGTTGGTTCAATAGTTTTGCTTATCTCT
ATCCCCAATTTAATTTGTTTGCTAATTGGGTGAGACGGGCTTGGAATTACTTCTTTTTTGTTAGTTGTGTAT
TATCAAAATAATAAATCTCTTGGAGCAGGGATAGTAACGGCATTAGTAAATCGGATTGGTGATGTGTTGC
TCCTGTTTGTTATTGGTGCTTTGGTTAGTGAAGGAAGGTGATTATTATATGAGGGATGCCTTAGGATAAGG
TACCCAATTCCTATTATTTTAATATTTGGGGCTATTACTAAAAGTGCACAAATACCCTTTTCTGCATGGCTT
CCTGCGGCTATAGCGGCTCCAACGCCTGTGTCTTCTCTTGTTCATTCTTCAACCTTAGTTACTGCAGGTG
TTTATTTGTTATTTCGTTGTAATTTCTTATTATTAAGTAGAGGGATAGAAACATTAAAGGTGTTAAGCTTAG
TGACTTTAGTGATAGCAGGTAGTTCAGCATTGGTAGAAGTGGATTTAAAAAAAGTAATTGCTCTTTCGAC
TCTAAGTCAGTTAAGTATGATGCTTTTTGCTTTGTCTTTAGGGTTAGTAGGGGTGTCTTTTTTTCATCTTGT
GACTCATGCCTCTTTTAAAGCTTTACTTTTCTTGTGTGCGGGTGTGGTTATCCACAGAAACCATAAATGT
CAAGACATTCGGTTTTTAGGTAAAAGTTGGCCATTTCTTCCCATTAGGATATCTTGCTTAGTTGTTGCTAA
TATATCCTTATGTGGCTTACCTTTTTTAAGGGGATTTTATTCTAAAGATATGATTATTGAGCTAGTAAAAGG
CGAGGGATTAATTTACTTTTTGGAGGTCTTGGGTACTTTGTTTACTTCTTGATATTCATTTCGTATGTTGAA
TGTAATGTATGGAATAAATAAGGGTGTTAGACGGGTTAGGTTTAGAAGAGAAAGAAATATTTTAAAATTT
GCTTATTTTAGGTTGTTGGTTAGTTCTGTATTAATTGGTTGGGTGTTAGGGCTTTTAGTAGAGCAGCTGAA
TAGAAGGGTCCATCTATTTAGATTTGATAAAGGCTTAGCTAGAAGCTTCGTTTTTGCTATTGTTAGGTACG
GGCTTTCAATTCTTTACCAAGGGAAAAATGCTGGTATGTGGTTCCTTGCAACAATGTGAAATTTGAAACT
AACCCAGATGCCATCAAAAATTTTGATGTTTTATTCAAATGCTGTTGTTGTAAATTTAGAGAAAGGATGG
TTAGAAAAAGTTGGTCCACAAAAATTGATTAGTAGAATTAGAAGATTAAACCAGGGTTACCAACAGTTA
AAAGTACTTAGTGTGTCTTTTATGGCTTTCTTATTTTTTCTAAGAATGAATTTGATTTTTTTTTGTGGTGTA
GTTATATTTTTATGCGTTTTAAGGGTTTGGGAGTCTTTATATATTGGGTTAATTTTAGGGGTTATGTCTATTT
GTGTTTCTGTTATTCTCGGTGGAAGAATAAGAAGGCTGGCAGGGTATTTTGTTTTTTTAATTTATGTGGGT
GGTTTGATAGTGTTGTTTGGGTATGTTTTAAGCGTTTTTCCTAACCAGTACTTTGCCTTTGGTTTTCTTCC
AGGTAAGTTTTTATTTTTGTCTATGGTTtttATTAGACTTTTTAGGATTAGTGGGGCAAGAAAACTGGGGGG
TTTTTTAATATTTAATAGGATATATTTATTTGTTGGTGGATTTTTGTTATTTATTCTTATTTTAGTTGTTTCGTT
ATGTAAGAAGCGCCACTTACCTCTTCGTGGGGGA"/>
<sequence id="seq_Modiolus_modiolusKX821782" spec="Sequence"
taxon="Modiolus_modiolusKX821782" totalcount="4"
value="GTTATATTAGATTTGCTTTCTAGGTTTGATTCTTTTAGAATGAGTAGAATTattTCTTGCGGATTGTGG
ATCAGCATGGGAATTAGGTTATTTGTGTTGATTGGTAGTGATAAAGGGTTTAGTGAAATTGTTTTACACA
AAATTTTGTCTGTTTTGAGAGGTATAACTGGTCAATTTGTATCGGGATTTAGTATTAGTGTTATTAGTTTGT
TTGTATTTTTAATTATGGTAAATTTGTTGGGACTGGTGCCTTTTTCTTTTAGAGTAAGGTCTCAAGTAGGA
TTAGGTCCTTCAATAGCATTTTTTATGTGGTTGTGTTTGTGGTTATCTGGGTTGCGTGTCTCGTGGCGGCA
GACCTTATGTACTTTGGTGCCTAGATATCCTATGTTCCTGATTCCTTTTTTATTGTTGGTAGAGGTAGTAAC
AATTAGATCTCGCCCTGTAACATTGGGATTACGATTAATAATTAATTTAACTGCTGGGCATTTGATTATAGG
GATGTTGACCAATGTTAATACAAATGTTGTTCTTTGTTCTGCTTATGGATTTCAATTTCTAGGGCTTATTGC
TATGAGCTTAAGTGCTGAGATGGTAATTGGGGGGTTGCAAGCCTTTATTTTTTGTACACTTTTAGGGTTAT
ATAGAAATGAACATCCTAGATAAGGTGTTTGATCGGGAATGGTGGGGATTGGTTTAAGAATGTTAATTCG
AATTGAGTTAGGTCGTCCTGGAAGAttaTTTTTAGGGGACGATCAGCTATATGTCATTACGGCCCATGCTTT
AGTTATAATTTTCATGGTTATGCCTTTAATGGTCGGGGGTTTTGGGAATTGGCTTCTTCCATTAATAATAGG

TTCTGTAGATATAATTTTTCCGCGACTTAACAATTTGAGATTTTGGTTTCTCCCCTCTTCATTATTTATACTG
TTGAGGTCTACTTTTATTGAAAGCGGGTCCGGTACTGGATGGACTTTATACCCTCCTTTGTCTTCATATAC
AGGACATAGTGGCCCAGCTGTTGACATATCTTTATTTTCTTTACATTTGGCAGGTGCTTCTTCTATTGGTG
GATCTATTAACTTTTTAACTAGTATAAAAAATATGCCGGTGGAGGTAATGCGAGGAGAGCGGATAATGTT
GTTCTTGTGGTCTATGGTGGTAACAGCTGTTCTTTTATTGGTGTCTTTGCCCGTGCTGGCTGGCGGTATTA
CTATGCTGATTTTTGATCGTCATTTTAATACTTCTTTTTATGACCCTGTAGGAGGGGGAGATCCAGTGTTG
TACCAACATCTATTTTGGTTTTTTGGTCATCCTGAGGTATATGTACTTATTCTTCCCGGGTTTGGGATAGTA
TCTCACGTTGTAGCTCACTGTGCTGGTAAAGATGAAGTTTTTGGGGTTTTAGGAATAATTTATGCTATAGT
GTGTATTGGGGTTTTAGGATTTATTGTGTGAGGCCATCATATGTTTACTGTCGGAATGGACGTTGACTCCC
GAGCATATTTCACATCGGCTACCATAATTATTGCCGTACCTACTGGTGTAAAGGTTTTTAGTTGATTGGCT
ACCCTTAATGGGGGTAGGTTGCTAATTGAGACTGCTTTGTTGTGAGCTGTTGGGTTTATTTTCTTGTTTAC
GGTAGGAGGCCTTACTGGAATCATGCTTTCCAATTCTTCCCTTGATGTGGCCATACATGATACTTATTATG
TAACAGCTCACTTTCATTACGTACTATCGATGGGAGCAGTTTTTGCTTTGTTTTGTGGATTTTTTCATTGG
TTTCCTCTGTTTTATGGATACTGTTTCCACGAGCGGTGAAGAAAGGCCCATTTTTTTATAATATTTATTGG
AGTAAATTTAACCTTTTTTCCTCAGCATTTCTTGGGATTGAGAGGAATACCACGCCGTTATTCAGACTATC
CAGACTGTTTTATGAAATGGCATGTAGTTTCTTCATGGGGGTCTTTATTAAGTTTTGTAAGGGTTCTGTAT
TTTCTTTTCATTGTATGAGAGGGGTTATTGAGTCAGCGTGGTGTTGTGTGTAAGAGTAACCGTCCTGGAG
CAATTGAGTGAAAAAATGTGGTGTTGTATGCCGTTGTGGGGTAGAAAAGGTTTTCAAGATTCTTATTATCA
AGTTGGTGAATACCTAAGCTTCTTTCATGAAGGAGTTATGTGTTTAATTGTTTTTATTTTATCTGTTGTTTT
ATATGGACTTGGTTGGGTGTTTAGAACAAAAAGAAGCTACCGGTTTTTACGCGAAGCTCAAGGTGTAGA
GACGGCATGAACCATTATTCCTAGTTTATGTTTAATTGGTGTAGCAGTTCCTTCTATGCATCTTCTTTATGT
TATAGATGAAATTGGAAGTCCTTCTTTTTGTTTTAAAGCAATTGGCCACCAGTGATATTGAAGGTACGAG
ATGGTAGATGTATTAGGGTTTGACTCCTTTATGAGTCGGAGAGAAGATGATGGCTATCGATTATTAGATGT
TGATCAGCGAATGGTAGCGCCTTCTAACACTGGGATTCGTTGTATGGTCAGGAGGGCTGATGTAATCCAT
TCTTTTGCTATCCCTGGGTGTATGTTAAAAGTAGATGCAATTCCAGGTCGTGTTAATGAAATCCCTATAAC
TGTGGCCATAAGAGGGGTTTTATATGGTCAATGTTCAGAGATTTGTGGTGCTAATCATAGTTTTATACCTA
TTGTAGTTGAGTTTATCCCTCCAAGGGTATATAATCGATGAATTAAATCTATTGAGGAT-----------------------
ATTCCACGAAATCCTTATTATTTAGTAGGTTCTAGTCCATGGCCTATTTTTACTTCTATTGGTGGGTTGTGT
TTAGCAGTTGGATTTGTTTCTTGGTTTCATGTGCACAGGTATAGTCTTTTGTTTGGATTCTTAGTTTTAGGT
TTTTCTTTAGGTCAATGATGGCGTGATGTTATGCGTGAAGGAGAGTTGGGTTATCATACTTCCTTTGTTAT
AAAAGGTTTGCGTGATGGTTTTATTTTGTTTTTAGTATCTGAGATTATATTTTTCTTTTCTCTCTTTTGGGC
TTTTTTTCATATAAGGTTAGCCCCTGATGTTTCTGTTGGGTGTGTGTGGCCTCCTAGGGGGGTAGAGACC
CTTGACCCATTTAAAGTTCCTTTATGTGGTACTACTGTTTTAGTTGGGTCAGGTGCTTCTTTAATATACGC
CCATGCTGCTATTCGGGCTGGGTTAAACAAAGATGCTATTTATGGGACAGGAGCCACTATTCTATTAGGT
CTTTTATTCTCTCGTCTTCAAGCATATGAGTATTATTGAGCTAGGTTTACTATTGCGGATAGTGCTTATGGC
AGCTTGTTTTATATTATGACTGGGTTTCATGGGATACATGTAATATTTGGGACAGGGTTTTTAATTGTGAGT
TTGGTACGGTTGTTCCGTTATCGATTTACTCCACGCAATCATTATGGGTTTATAGTGTGTTCTTGGTATTGG
CACTTTGTAGACGTAGTTTGGATTGGGCTGTATGTAGTTGTTTATATTTGAGGTAGTTagCCATATCGAAAG
AACCATAGGCTACTGAAATTTGTAAATAATAGTTTGTATGATTTACCAGCACCAGTTAATTTAAGGGTGTG
ATGGAATTTTGGATCTATGTTAGGTTTATGTTTAGTTATTCAAATTGTTAGTGGGTTGGTGTTGTCGTTTCA
TTACACAGCTCATGTTGATATGGCGTTTGATGCAGTGATCCATATTGTTCGTGATGTAAATAAGGGTTGAA
TGATTCGGAGTATACATGCAAATGGGGCTTCAATATTTTTTTTATGCATTTATGCTCATATTGGTCGTGGAA
TCTACTATGGGTCGTATAAGTATAGGGAAGTTTGAAATGTAGGAGTGGTTTTGTACTTGTTGGTAATGGC
AACGGCTTTTTTAGGTTATGTTCTCCCTTGGGGTCAGATGTCTTATTGAGGTGCAACAGTTATTACTAGCT
TATTAACAGCTATCCCTTATGTAGGCGAAATATTAGTACATTGAGTTTGGGGGGGGTATTCGGTTTCTAAT
GCCACATTGGTGCGGTTCTATTCCTTCCATTTTATTTTACCATTTGTTATTGCGGCTTTAAGGGTTGTTCAT
TTGCTATTTCTTCATGAAAGAGGTTCAAACAATCCATTAGGAGTGTCGAGTAATGATATACTTATTCGATT
TCATCCTTTTTATACGTCCAAAGATTTAGTGGGGTTTTTTGGTTTATTTTTTCTTCTTATAGTTCTTGTATGT
TACTACCCAGAGTTGCTGGGGAATGTAAATAACTGAATTCCAGCTGACCCAATGAAGACTCCACTTCATA
TTGAGCCTGAGTGGTATTTTCTGTTCGCTTATACTATTTTACGGTCTATTCCTAATAAAGCTGGGGGGGCT
CTTGCCTTGGTTGTGTCTGTTTTTGTTTTATTTGTTATTCCTTTACTTCATACTGGAAAATTTCGAGGTCTT
TCTTATTATCCAGTAAGTCAAGTTTTTTTCTGAGTGTTGATTAACGTTTGATTAGGGTTGACTTGGTTAGG
GACATGCTTCCCGGAGTACCCATTTGAGGAGATTGGTCGGGCGTTGACTTGTTGATATTTTATCATTATTG
TTTTAATTCCTTTGACGCAAAGAAGTTGAGACAAGTTAATTGAGGCTGAGTTTGTAGTACTGATTTTACC
TTTTGTATGTGTTTTGGTTTCGGTAGCTTTTTATACCTTATTGGAGCGTAAAGTTTTAGGTTATATTATAAG
TCGGAAAGGGCCAAATAAGGTGGGGTTTATAGGGGTCATTCAACCTTTTAGCGATGCCGCAAAATTATTT
ACTAAAGAAATAGTTATGCCTGGGTTTTCAAACAACATGGTTTTTGTGCTATGCCCTGGCTTGATCTTACT
TAATGGGTTGAGATTATGGCTTCTTTATCCTTTTAGTTATACAGAGGTGGTATTTGTTTGTGGGTTAATTCA
GTTTCTAGTGGTCTCAAGAACTAGTGTTTATGGGGTGATGCTAGCAGGCTGGTCTTCTAACTCTAAATAT
GCTTTATTAGGTTCCGTACGTGCTGTTGCGCAAAGAGTTTCTTATGAAGTGCCTTTAACATTTGTGATAAT
TATTATTTGTTGCTTGTTAGGAAGAATGCTCTGTCAAGAAGTAAAAGAAGTTTTTAGTTCTATTTTATTTG
GTTTAGGCGGAATAATTTGGTTAGTGTGTATATTGGCTGAATCTAATCGTGCTCCTTTTGATTTTGTTGAA
GGTGAATCTGAGTTAGTTTCTGGTTTTAATGTGGAGTATAGAAGAGGAGGCTTTGCGATAGTTTTTATAG

CAGAGTATGCAGCAATACTGTTTAATAGTCTTTTTTTTACCGTTTTATTTGTTGGGGGCAGAGGGGTAGTG
GTTATATTAGGGATAACGTTAGTTGTAGTGGGTTATGTATGAGTTCGCGGGTCTTTTCCTCGTATGCGGTAT
GATAAAATAATAAAACTGTGTTGACGTTACTTAACGGTTTTAGTCCTCTGTTTAGGTGTTTTTTGTCTGTG
TGTCCCTTACTTATAataagtATAAATATTTATAATCCCTTTTATTTGCTTAGAGGGCTTTTATTGATTGGAGGA
ATTTTAGTAACTTTAAGTACTGATTCTAAAATTAGGGGCTGGTTAGGAATAGAGATTAATGTAATAGGGTT
TTTAGGGGTGTTAAGGGTTCGGGGTTTTATAAACATTTCTGTTGGATTGAAATATTTTATTATTCAGGTGC
TTGGGTCAGGCTTATTTTTAATGGGTGTGTTAGTATTTTATCATTTGATGTTGCAGAGAGGGTGGATAGTA
GAAATGGGGCTATTTTGTAAAGCAGGTATTTTTCCCCTTCATGGATGGGTTCCATCAGTTATTAATTCAGG
GGATTGAGTTAGTGGGTGGCTGGTTATAAGGGTTCAAAAGTTGGCTCCTATTATTATTTCAGTCTGGAGT
AGGTCTgagTTTTTTATTTATATAGGGTTAGTAGGATTAAGGGTTGTTGGGGCCTTAGGTGGGTTAAATCAA
ATGTCTGTCCGTGGAATTTTAGCCTATTCTTCCTTTGTGCATGGTGGGTGGATACTGGTGGCGTTAACTCA
TTCTAATGAACTTTTCTTTTTATATTTTTTAGGCTATTTAGTTCAGTTAACAGTTGTAGTAGGGATCTGTTAT
GATTTAGATGTAGAGAAAAGAGCAAGGAGAAAAATATCTTTTTTAGGTGGAGTGATATCATTAAGTTTAG
GGGGTCTTCCTCCCATAGGAGGCTTTTTGTTTAAACTGAGGGTGTTTCTTTCGGTGAATTTGAGGGTGTT
AGTGGTCCCAGTAGCGGGGTCAGTCTTGTCGCTTCTCTTTTACTTACGGTTGATAAACGGGTTTATGTTG
GGGAGAAATAGTTTAGTAAGGTTGTTTCTGGTGTTTAATGGGGTCTGTTATATAGGATTTGGGATAATTTT
TGGTTTATTTAGTTAGtaaGGTTTGCTTTGTATTTGGTTTATAGTGGCTGTGAGGTGCTGTTTGTATTTTACT
TTTTTGTTATGGCTGGCTATAGGAGATAGAAGTAGTCGAGAAAAGTCTTCTTGTTATGAGTGCGGGTTTG
AGCCAATTCGAACTGCTCGAAGTAGGTTTTCATTGCGATTTTTTCTTCTAGGGGTTTTGTTTGTTGTTTTT
GATGTAGAAATTGTGATAATGGTTCCTATTTTATTTGGTGTTAGAGTGGGGGGATCAGCATCTAGAGTTGT
TTGTTTAGTTTTGTTTATCATTGTGTTAGTAGTGGGGTGTTTATATGAGCGGCGGGATGGCTCTATAGATTG
AATTAAAGAGATGGTTTTGAGGGGTGTGGCAGTAGTTTTGTTATTATTTACCGTGGGTGGAGAAGTAGA
GGTAGTCTTTGGGGTGTTAATTTGTACTATTCTTTTTATGCCTTTGTTGAGATGAGAAAATagtAGGGTTGA
AATGGGGGGTTTAACTAGATTGGATTTAGTTGGTGTGATCATGGTAATTTTGTCCTTTTATATTAGGGGTC
TGATGCTTTTGAGAAGAGTTGGAGTGAAGCGGTTCAATGCATTTAGAAAAGTAATTTTGTGTATTTGTGT
GGTTTTAGTGTTAGCGTTTAGTTTTAGGTCTATATTTTTATTTTATATTTGTTTTGAAAGGGTGTTAATCCCA
ACTTTGCTCTTAATTTTGGGTTGACGGGTTCAAGCGGTTAATTACATGTTAGTTTATACTGTAGGTGGTTC
TATACCTTTAATTTATGGCTTAAGAAGTCTTTATTGGAGAGGAACGAGTAGAATAACCTTGCTTGGAAGT
TTAGATAAAAGTGTTATCGCTTTCTCCTGGTTATATGTTCTTGCTTTTTTGGTTAAACTTCCGGTGTTTCCG
TTTCATTTGTGATTACCTAAAGCTCATGTAGAGGCTCCTGTTTCGGGTTCTATGATTCTTGCGGGGTTGTT
GTTAAAACTTGGTGGTTATGGGTTTATTCGCTTATGTGGGTTTGTTGAATTTCTTCGTTTGAGGGTCCCAG
TGGTGGTTTTGTCTGTGAGGCTATTTGGGGGTGTATTAACTAGGGTAATGTGTATGCGTCAAACAGACCT
AAAAAGTTTGGTAGCGTATTCATCTATTGGTCATATGAGGTTTGTTTTATTAGTTGTTACAAATGTTTCATG
AGGAGTCTTAGGAGGGGTTTTAATTATATTAGGGCATGGGTTGTGTTCATCTGCTTTGTTCTCTTTGGTAA
ATTACATGTATGGTGTTAGTAGTAGTCGTCTTATTAGATTAAATAAAGGGTATCTACTTATATCTCCTTCTTT
GTCTTTAGTGTGTTTTTTGTTGGCAGTTAGAAATATAGCTAGACCTCCCAGGTTGAATTTGTTTGGGGAG
TTATTAATGTTTATAGTAGGCAGGGTTTTTAGGGTGTTAGTTTTAGTCTTGTTAGGATTTATAAGGTTTATG
GCTGCTTGCTATAGGCTATATATTTATGTGGGAACACAGCATGGTAAAAGGTGCGGTTTATGAGTAAGAA
GAAGCAGTGTATGTAGGGGACTTGTGCTGTTAGCTCATTGGTTTCCTTTGAACCTTTTATTTTTGTTTATT
CCTTagTCACGTAGAGCTCATTTAGTTAGAGTGTTTTTAGGCATAGAGTTTATAGCTTTAGGGACTTTTTTA
ATAGCCTTTGTTCCGTCCATAAATAGGCTTTTTATCCTTCTTGTTTTGTTGTGCATAGCGGTTTCAGAGGC
AGCTGTTATATTGTCTTTTATAGTCCAAGTGACGCGCCTTTATGGGAGAGACCAAGTATCTAGGGTTATGG
TAGATAAATTCAGTTTTCTAAAGTTTTAGGTATAATGTTCTTAGTTTTAGGGGTGATACTCCTTAAGGCGG
CAAGTAGTTGTGTGGTGATTATTTTGGAGTTTAGGCTTTGACTTTCCTATCTTTCATCTGTAAACTTTAGA
CTATTCTTTGATTGTAGTGGGTTATTGTTTATTAGAACTGTTTTACTTATTTCAGGTTCAGTACTAATTTATT
GCAGTTGGTACATAGATGATGAGGTCTACTATAAGCGGTTTATTTTTTTAGTGTTATTGTTTGTTGGCTCA
ATAGTTCTACTTATTTCTATCCCCAATTTAATTTGTTTGTTAATTGGGTGAGATGGTCTAGGAATTACCTCT
TTTTTGTTAGTTGTGTATTATCAAAATAACAAGTCTCTTGGAGCAGGGATAGTGACGGCGTTAGTAAATC
GAATTGGGGATGTGTTGCTTCTTTTTGTGATCGGTGCTTTGGTTAGTGAAGGGAGGTGGTTGTTATATGA
AAGGTGCCTTAGATTAAGGTATTTTGTTCCTATTATTCTAATATTTGGGGCTATTACTAAAAGTGCTCAAAT
GCCTTTTTCTGCATGGCTTCCTGCGGCTATAGCGGCTCCAACTCCTGTGTCTTCTCTTGTACATTCTTCAA
CTTTGGTTACTGCGGGTGTTTATCTGTTATTTCGTTGTAATTTCTTATTATTAAATAGGGGAGTAGAAACAT
TAAAGGTTCTTAGCTTAGTAACTTTGGTAATAGCAGGTAGTTCGGCATTGGTAGAAGTAGATTTAAAAAA
GGTAATTGCTCTTTCGACTTTAAGTCAGTTAAGTATGATACTTTTTGCTTTATCGTTAGGGTTAGTAGGGG
TATCTTTTTTCCATCTTGTGACTCATGCTTCTTTTAAGGCTTTACTTTTCTTGTGTGCGGGTGTAGTTATTC
ACAGTAATCATAAGTGTCAAGATATTCGGTTTTTAGGTAAAAGGTGGCCATTCCTTCCTATTAGGATATCC
TGCTTAGTTGTTGCTAATATGTCTTTATGTGGTGTTCCTTTTTTAAGGGGGTTTTATTCCAAAGATTTAATT
ATTGAGTTAGTAAAAGGTGAGGGGTTAATTTACTTTTTGGAGGTTTTGGGTACTTTGTTTACCTCGTGGT
ATTCGTTTCGTATGTTAAACCTAATGTATGGAATGAACAAAGGGGTTAGACGGGTTAGGTTTAGTAGGGA
AAGGAATATTTTAAAGATTGCCTATTCTAGGTTGTTAGTTAGTTCTGTGTTGATTGGTTGGGTGTTAGGGC
TTTTAGTAGAACAGTTGAATAGAAGGGTTCATTTATTTAGATTTGACAAAAGCTTAGCTAGAACTTTCGT
TTTTGCTGTCGTTAGGTATGGGCTTTCAATTCTTTACCAAAGGAAAGCTGGGGGTATATGATTTCTTGCAA
CAATGTGAAATTTGAAATTAACTCAAATACCATCAAAACTTTTGATGGCTTATTCAAATGCTGTTGTTGTA

AGATTAGAGAAAGGATGGTTAGAAAAAGTTGGGCCGCAAAAATTAATTAGCGGAATTAGAAAACTAAAT
CAGGGTTATCAGCAGTTAAAGGTACTCAGTTTGTCTTTCATGGCTCTTTTGTTTTTATTAAGGAATGAATT
TGGTTTTTTTTGTGGTGTAGTTATATTTTTATGTGTTTTAAGGGTTTGGGAGTCTTTATATATTGGATTAATC
TTAGGGGTTATGTCTATTTGTGTTTCTATCATTTTGGGGAGGAGAATAAGAAGGCTGGCAGGGTATTTTGT
TTTTTTAATTTATGTAGGGGGTTTGATAGTGCTTTTTGGGTATGTTTTAAGAGTTTTTCCTAATCAGTACTT
TGCTTTTGGTTTTCTGCCTGGTAAGTTTTTATTTTTGTTTATGGCTTTTattAGGCTTTTTAGGGTAAGTAGG
GCAAGAAAACTTGGGGGTTTCTTGATGTTTAACAGGATATATTTATTTGTTGGTGGTTTTTTATTATTTGTT
CTTATTTTAGTTGTTTCTTTATGTAAAAAGCGTCATTTACCACTTCGTGGGGGG"/>
<sequence id="seq_Mytilisepta_keenaeNC044127" spec="Sequence"
taxon="Mytilisepta_keenaeNC044127" totalcount="4"
value="ATGGGGATAGATGTGTTTTCTGTTTTTGATGATAATCAGTTAAATACTTTTTGGAGAAATGTTTTGT
GAGGTTGAAGATTGTTTCTTTTTGTTACTTTATTAAGGAAGTTAATTCGGGTTTTTTTACTATTGGTAAG
CTTGTAAGTGATGTAgttggtGTTGGAAGATCAGTGtatTTAAGGGGTTTTTCAGTTGTGATTTTTTCTTTATTT
ATTTTGATTTTATGGTCAAATTTTACAAGTGTAATTCCTTATTTTTATCCTGTTAGGTGCCATATTCCTTATA
TTGCTTCTTTTTCTTTGTATGTCTGAATGAGACTTGTGATTTCTAGGTTGGTTAATAGATATATACAGGTGT
TAGGTAGTTTAGTTCCTTCAGGTCCTATAAGATTGTCTCCTTTGTTAGTGTTGATTGAGATGGTTTCCTCT
CTTATTCGTCCATTAGTTTTAGTAATGCGATTAGTTTTTAATTTGGTAACAGGACAAATTCTTTTAGGTTTA
TTAGGAGAAACTTTTTTGGAGGTTCTTTTAGTTGGGTCTTTTGTTAATTTAGGATTAGTGATAATAGTAAT
AGTTTATTTTTTTGAGCTTTTTGTTTGTCTTTTGCAGAGGTATATTTTTTGTCAATTACTTTATTGTTATAGT
GAGGATCATTCATTGTTTcgatgattgATAAGAACTAACCACAAGGAGATTGGGAGATTATACCTTACTTTTGG
TATTTGGAGAGGTTTAGTTGGTGTTGGGTATAGAATATTAATTTTGAGGATGCATCCTGGTAATTTGCTTC
TTAAAGATAGTTTGTATAATGTGATTGTAACTAGGCATGCTTTAGTAATAATTTTTTTTGCTGTTATGCCTT
TGTTGATTGGTGCTTTTGGAAATTGATTGGTGCCTCTATTTTTAAGTGCTATGGATTTAGTATTTCCTCGTA
TCAATAATTTTAGTTTTTGAATTTTACCTAGTGCTTTATACTTGTTATTGCTTTCTGCTTATGTTGAGGATGG
AGTTGGGACTGGTTGAACTATTTACCCTCCGCTGTCTAGTTATACTTATCATAGTTCACCTGCTGTTGATT
TAGCGATTTTATCTCTTCATTTAGCTGGAAGTGGTTCTTTGATGGGGGCTATTAATTTTTTGGGCTCTAATA
AGAGGTTACCTGTAGATAAAATAAAGGGTGAGCGCTCTGTCTTATATATTTGAAGAATTAGTGTTACTGCT
TTTTTACTGCTTCTATCTTTACCTGTATTGGCTGGTGGAATTACTATATTGCTTTTTGATCGTAATTTTAATA
GAACTTTTTTTGATCCAATAGGTGGTGGTGATCCTGTTTTATTTATACATTTATTTTGATTTTTTGGTCATCC
TGAGGTTTATATTTTAATTCTTCCTGGTTTTGGTGTAATATCGCATGTAACTGCACATTATGCTGGGAAGAC
TTCCCCATTTGGTGTTACTGGTATAATGTACGCAATAATTTCTATTGGTTTAATGGGATTTATTGTTTGGGG
GCATCATATGTTTACAGTAGGGTTGAATGTTGACACGCGAATATATTTTACTTCTGCTACAATGATTATTGC
TGTTCCTACGGGAATTAAGGTCTTTAGATGACTTGCAACTTTAGCTGGAGGCCGTAAGACTTTTAAAACG
CCGGTCTTGTGAAGGATTGGTTTTATTGTTTTATTTACTATTGGTGGTTTAACTGGTTTAATTCTTTCTTCT
TCTTCGTTAGATATTAGATTACATGACACTTATTATGTTACTGCTCATTTTCATTATGTATTGTCAATAGGGG
CTGTGTTTGCTATTTTTTGTGCATTTACTCATTGGTATCCTTTGTTTTATGGGGTTAATTTGCATAAGCGTT
GAAGAAAAGGGCATTTTTTTTCTATGTTTGTTGCGGTTAATATTACTTTTTTCCCGATACATTTTTTAGGGA
TAAGAGGGATGCCACGCCGTTATTGTGATTATCCAGATTGTTATTCTAAATGGCATTGATTGTGTACATATG
GTGCTGTTATAGCTTACATGTCGTTGATGTATTTTATGTTTTTGTTGTGAGAGAGAATGGTGAGAAAACGT
GGGgtgtctATGTATGGGTCTAAAGTATTTCAAGATTGAATTTATGATATTGGGGATGCGTTGAATCTTTTCTA
TCATAATATAATATTGGTTGCTGTTTTTATTGTTTGTCTTGTAGGGTATTATCTTTTTCGGATTAATTTTTGC
AGAACTAGATATCGGGGTTTTAAACATAATAATGTTTTGGAATGAATTTGAACTCTCGTTCCTATGTTAAT
TTTGGCTGTTTTGTGGGTGCCTTCTGTTAGTAATTTGTATTTGATAAATCATATTGGGGAGCCTAAATGGT
CTTTTAAGGCCATTGGTCATCAGTGGTATTGAACTTATGAGTTGTATGAGGAAGTTATTTTAGAGTCTTAT
ATAAATAATATTGATGATGGTAAATATCGTCTTTTAGATGTTGATCAGCGTATAGTTGCTCCTATTAATATAC
AGTTGCGGGTTCTTGTTAGAAGTACGGATGTTTTACATTCTTTTGCTTTACCGTCTTGTATATTGAAAGTG
GATGCAATTCCTGGTCGAATAACACAGACGCCTCTTATAGTAGATAAAAGAGGTGTTGCTTATGGACAAT
GTTCAGAATTGTGTGGTGTGAATCATAGTTTTATACCTATTGTGGTTGAGTTTATTCCTACTAAGACTTTCT
TAGATTGGTTAAAGGTTACTGAGTTA------------------------
ATGGTGCGAAGTCCTTATTATCGAGTGAGACCTAGTCCTTGGCCTCCTTTGGTGGCGTTTTGTTTAGTTA
ATATAGCTCTTGGGTTGGTTAATTGGATATATCGAGTTAATATTTATATTATTTTTGGTGGGTTTTTGTTAGC
TAGATGTTTGAGTTTGTGGTGACGAGATTTATTACGTGAAGGGGATCAAGGATATCATAGAAAATATGTA
ATTAAAACTTTTCGTGATGGTATAGTAATATTTATTGTTTCAGAAGTAATATTCTTTTTTTCATTTTTTTGGG
CTTTTTTTACTAGAAGATTAAGTCCTAATGTTGAGATTGGGGGAAATTGGACTCCTTATGGGATTCGTAGT
CCTAACGCTTTTTCAATTCCTTTGTTAAATACGTGAATTTTAGTTACTAGTGGTATTTCTGTAAATTATTCT
TATAATTCGTTGAAGTGTGATTATGATTATGGTCCTGTTGTTGGGATAGTTTTTACTATCTTTTGTGGGGTT
GTTTTTGTAGGTCTGCAGTATAAGGAGTATTTTAGTAATTCTTTTTGTATTTCTGATGGTATTTATGGTAGT
GTTTTTTACATATTAACTGGTTTTCATGGTGCTCATGTAATTTTTGGAACTGTGTTTTTAATTGTGACTTTT
GGTCGGTTGTGATTTGGCCATTTTTTGTCAAATCGTCATTTTGGGTTTGAGGCATGTGTGTGATATTGACA
TTTTGTTGATGTTATTTGGATTGTTGTTTACATTTTTGTTTATGTTTGAAGTGGTGGGCCTTTTCGTAAGCG
ACATTGGTTGTTGAAAACTGTTAGAGCTAGAATTTATGATTTGCCTTGCCCGATTAATTTAAATGTGTGGT
GGAGTTTTGGTTCTATGTTGGGTTTATGTTTAGTAACACAAATCGTTACTGGTTTTATATTGTCTTTTTATT
ATATTCCTCACGGTGATATGGCTTATGATTCTGTTTTTTACATTATACGTAATGTCCATAAAGGTTGAATGG

TGCGTGGTATTCATGCTAATGGAGCGTCTGTGTTTTTTATGTGTATTTACGTTCACATTGCTCGTGGGTTAT
ATTATGGTTCTTATTTGGATAAAGGTGTTTGGAATGTGGGGGTTATTCTTTATCTTATGTTAATAGGTGAAT
CTTTTTTAGGTTATGTTCTTCCTTGGGGTCAAATATCTTATTGGGGGGCTGTTGTAATTACTAGTATATTAA
CAGCAATCCCTTATGTGGGTAACAGGATTGTAGAGTATGTTTGAGGAGGTTATGTTGTTAATACTCGTAC
ATTGACTCGATTTTATTCTTTTCATTTTATTATTCCTTTTTTAATAGTTGTGATAGTAATACTTCATTTTTTTT
ATCTTCATGATAAGGGTAGAAATAATCCATTAGGAATTAGAAGGGATAGTATGTTGATTCCTTTTCATCCT
TTTTATACTGTAAAAGATATTGTTGGGTTTGTTGCTATGTTTATGGTTTTAATGTATTTTGTTTGTGTTAAAC
CTGAAGCATTGGGTAACCCTTTAAATTATATTATTATTGATCAGTATAAGACTCCTATTCATATTCAACCTG
AATGGTATTTTTTATTTGCTTATACTATTTTGCGTTCTATTCCTCATAAAGTTGGTGGAATTTTAGCTATATT
AGGGTCTATTCTTATTTTGTTTTTAATGCCTTTAATCCATACTGGGCAGTTTCGTAGGCTAAGATTTTATCC
AATACATCAGCTTCTTTTTTGATTTAATGTTAGAGTGTTTATTGGTTTAACTCTTATTGGAATACGTATTGT
TATTGAGCCTTATATTTTTGTGGGACGGGTTTTAACTGGTTGTTATTTTGTTGGGATATTATTATTACCTTTG
AGTTTGTTTTTGTGGGATTTCTTAATTACAATTTGGTTGATTTTGAATGGGTTACCAATTATTTGTGTATTA
TTAGCTATTGGGTTTTTTACTCTTTTTGAGCGAAAGTTATTAGGAAGAATAATACTGCGAAAAGGTCCTA
ATAAAGTAGGATTTATAGGTTTGTTACAGCCTTTTAGTGATGCTGGTAAACTTTTTTGTAAAGAGGTAAAT
GTACCTCGGTTTGCTAATGTGATACCTTTTGTTATTGCTCCTGTTTTTATACTTATGATTTCTATGAGATTAT
GGATTATATATCCTTTTAGAAGTGTTGGTGCTTTGTTTATTTTCGGTGTTTTACAGTTTTTGGCTACGGCTG
GGGTAAGTGTATATGGCGTAATAGTAGCTGGATGATCCTCTAATTCTAAATATTCTTTATTAGGATCAGTAC
GTAGCGTTGCTCAGAGAATTTCTTATGAAATTTCGTTTAGATTAATTATTTTTGTGTTAGTTTTAATGTCTA
TAAGATTTTTTATTCAAGAAATTTCATTTGCTTTTATATTTGTTGCGTATTTGATAGGTTTAGTTTTATGAAT
AGTGTGTATTTTAGCAGAAAATCATCGTGCTCCTTTTGATTTTGTGGAAGGTGAGTCTGAATTAGTTTCT
GGATTTAATGTGGAGTATAGAGGTGGATTATTTGCTATAATTTTTATAGCTGAGTATGGAAGAATATTATTT
TCTAGAATCTTAAGAGCATGTTTGTTTTTTGGTGGTAGAGAGGTTTTTATGAGTATTGTTTTTCTGTTCTTT
GATTATTTTTTTGTTTGAGTTCGTGGAAGGTTTCCGCGGATGCGTTATGATAAGTTAATAAAAAATTGGGTG
AACAGTATTTATAGTTATTCCTTTTATTTTTTCATTGTTAGTTTTTTATATTCTTTGTTTTTATTTAAGAATTG
AAAGCTTAACCCTATGGGGGTTTTAAGGGCTGTGGTTATAGTTTTTAGAAGATTAATAAGGATTTCTAGG
AGAACGTGATTAGGTGTTTGGGCGGGTTTTGAGTTAAATCTTTTAAGTTTTATAGTTTTAATAAATTTAGA
GAGAACACGCGCTATTAGGCCTTGTATTAAGTATTTTATTATTCAGTCTTTGGGGTCTGGATTAATTTTAAT
AGGGTTTTTATGTGGTGAATTTTATTTCAGTGAAAATGAGTTTGTGTTTTTTGGTGGGATTATATTAAAGG
GAGGGATTGCCCCATTTCATTTTTGGGTTCCTTCGGTTGTAAACTCTGCTTCTTGGTTAGCAGGGGGATT
AATCCTTTCATGGCAGAAGTTAGCTCCTTTTTTTTTAGTGGGGTGATTGTTTAGGGATTGGATAATCGTTA
TTAGTGCAGGTTTACTAGCTTTAATTGGTGGAATTGGGGGGTTGAATCAACACTCTGTGCGTGGGTTAAT
ATCATATTCCTCCTTTGTTCATAGATCGTGAATAATAGTAGCTTTGCTTAGCTCATTTTCTTTGTTTATTTTT
TATTGAATAGTTTACAGAATAAGAGTGGTTTTGATGTTTTGGTCTTGTTCTAAGGCACGGAAGCAGTTTT
TGAAAAGGAAAATGCGTGTGTTTTGTAGTTGTTTAAGACTTTTTATGTTAAGAGGATTGCCCCCTTTTTT
AGGGTTTGTTTCTAAACTTTTAGTAATAATATCAGTTAACAGGATTGTGGTTTTTGTTTGTGCAATTGGAT
CAGTGATTAGGTTAAAGTACTATTTATCTGCTCTTAATTCATTTATTTTTGGTGATATAGGTTGGTCTCAGT
CTATTGTAATTTTGAGAGTTTTTCTAAATGTTTTTGGGTTTCTTTTAATTGTGATTGGTTGTTAAcaaagatttAT
GTTAGTAAGGGTGGTATTTATTATTTGTTTAGGTCTTTTTTTGTTAAGCTTATGGTTTTTGTCTATAAAATGT
GGAGAAGACCGTGAGAAGTCTTCTCCATACGAGTGTGGTTTGATGGATTGTTAAGCGCTCGTAGGCCT
TTTTCCTTGCGATTTTTTTTGGTAGCTGTAATATTTGTTGTTTTTGATGTGGAAGTTGTCTTGTTTGTGCCT
ATTGTTTATTCTTTTTGATTTTTTAAAAGTGCTATAACTATACTTATAAGAAGATTGTTTTTGTTAATTTTGT
TTTTAGGGTTATTTCATGAATACCGTGAAGGTTCTTTAGAGTGGGTAGATTAAATGGTTTTAGGGTTTTTG
GTTAGTCTTGTTGGGTTGTTAGTGTTAAAAGATGTAAAAGCAAGATTGGTGGGGTTTGCAGTGATTCTGT
TTTTTTCTATAATCTTACTAGGATCAAGATCAATACATTATGAGTTATTAGGTATTTTTAACCTTGATTTTCT
TGCTAGTAGAATAATTTGCTTAACGTTATATGTGGTTAGGCTTATAGTAATAATAAGATTAAAAGTAAAGC
GAGTTTCGTTGATAAACTTTATAAATTTGAGTATTGGCGTAGTCTTAGTTTGTGTTTTTACTGTAAGGAAT
TTTTTTTTTTTTTTTTTCTTCTTTGAGAGGTCTTTAATTCCTATTGTGTTTATGTTGATTTTTTGGCGTCTTC
AGGCAGTAAATTACATAGTGATTTATATAAGTGCTGGGTCTTTTCCTTTCTTATTTGGTTTAAGGAATTTGG
TGAGTTCAGGGAGTGATAGAATGTCTATATATAATAGGGTTTTTAAGAGTTGTTGTTGGTTTTATTGGTTT
TATTTGATGGGGTTTTTTGTTAAGTTACCTATATTTCCTTTTCATTTATGGTTACCTAAAGCTCATGTTGAG
GCTCCGGTAGTTGGTTCTATAATTCTTGCTGGTGTTTTGTTGAAATTGGGTGGTTATGGGATTATTCGTTTT
TTAGGTTGTATAATAGTGCCTTTATTTAGCGGTTCTTGCGTTTTAGTTTCTGTGGCTTTATTTGGGGGATTT
TTAGCTAGGGTTATATGTTTAATGCAGGTCGATTTAAAGTCTTTAGTGGCTTACTCTTCTGTTGGTCATATG
AGATTAATGGTTTTTGCACTTTGTCAGAGGGAGGTGAGTTTTTATGGTTGTATTATTTTAATAGTGGGTCA
TGGTTTATGTTCTTCAGGCTTATTTTGTTTAGTATATATATTTTATTGTGTTTCTGGCTCTCGATCTGTTATTT
TAAATAAGGGGTTTTTATTTAAGATTCCTGCTTTTGTGTTATGTTGTTTTATTTTAGGTGTTAGGAATATAG
GAGCGCCGCCAAGGCTGGGTTTTGTTGGTGAGGTGTTGTTATTTATAAGTTGTAGTATGGTTTCTTATTGG
TTTGTCTTTGTTTTTTGTTTGATGAGTTTTATAAGTGCATGTTATAGCCTTTATCTTTATGGTGTTTCTTGTC
ATGGAAAGGAAGATAGTTTATTATATTTAAGTATTTCTTTTAAGGATTTGTTTGTGTTGTTTTTGCATATTTT
TCCTTTGGTTAGTTTGTTTTTGTTTTTGTAAtagGTACAAAGGAAACACTTAATTAGGGTGTTTTTAGGGAT
AGAATTTATGGCTTTAAGAGCTATGTTAATTAGGTCTATTGCGGTTTATAGAAGATCAAGTTACGTTTTATT
TACTATATGTATAGCTGTATGTGAGGCAAGTATCGCTTTGGCGCTAATTGTAAGTATAGTGCGTGTTCATG

GAAGAGATCGTGTAATAGCATTGAGTTTGGATAGatgagtgttgtaATGGTTTTGGGGTTTTTAGGGTATTAATT
GGTTATTTTTTGATGGTAAGTTCTCTTTTTCATTCTTGTAGAGTTCTTGAAGTAGTATTGTGTGTTGTAAGT
AGGTGTGAAGTTGGTGCTAGATTTTAGTTGATGAAGTTAGGGTAATTTTAGTGGGGTAGTTTAGTAA
TCTTTGGAAGTGTAAGGATTTATAGTAAATGGTATATGAATGATGAAGTTTTTATCGGCGGTTTATTATTT
TGATTTATTTATTTGTAGGTTCTATGATTATATTAATTTTAGGTCTAATCTAGTTGGTTTGATAGTTGGGTG
GGATGGTTTAGGTTTAGTTTCTTTTCTCTTAGTGTGTTATTATCAGAATTCTTCTAGAATAGGGGCTGCTAT
GTTGACTGTCTTAGTAAACCGTGTTGGGGATGTTTTATTTTAGCCAGTATTGGTTTGATAAGTATATGGG
GTGAGTTTATAGTTTATGATCGTTTTTTGTTTGAAAGTTTAGGAGTTAGGTTTTTATTATTTTGGCTGGTA
TAACTAAGAGGGCGCAAATGCCTTTTTGTTCATGGCTTCCTGCAGCTATAGCAGCTCCTACTCCTGTTTC
ATCTTTAGTTCATTCTTCAACTTTAGTGACTGCTGGTGTTTATTTAATTATTCGTTGTGTAAGGTTGTGTGG
GTTGTTGGGGATGGAATTTTTAAAGTTTATAAGTTTGTTAACGTTGGTTATAGCTGGTATAGCTGGTTTGT
TGGAGAGTGATTTTAAGAAGGTTATTGCTTTATCAACTTTAAGTCAATTAAGTGTAATAATGTTCTCTTTG
AGTTTAGGGCTTTATAGTTTAGCTTTTTTCCATTTAGTAACTCATGCTACTTTTAAGGCTTTGTTATTTTTG
AGGGCGGGAGCTGTGATTCATTCTAACAAAGGGTGTCAAGATTTGCGTTTGTTGGGCGGAATGTGAATA
AATTTACCAGTTAGAAGTGCTGTTATAGTCGTTGCTAGATTTTCATTGTGTGGGGTTCCTTTTATAAGTGG
ATTTATTTCTAAGGATTTAATTATTGAGTTAATGAATGGGATGATTTGATTTTATTTTTTTATGTTATTAGGT
ATTATGTTTACATCTTGGTATTCTGTTCGAATTATGTCTGTTGTTTTTGGGTTAAATAAGTGTGTAGTTAGG
AGTATTAAGATTAGGGAGCCTTTAGATGTAATCTTTTCTTACTTTTGCTTATATTTTGGTGCAGTATTTAGG
GGTTACTTAATGGTGGAAAAGATAGGAATTCTTGTTTATCAAAGATTTGTTGATTCATCTTTTTTCTTTTTT
TTGTTATTTTTACCTTTTGGTggaGGAATTTGGTTTATTTTTTCTACGTTTTTAAAAAAGACTTCTAAAGTTT
GGTTTATTGTTTCAATGTGAAATTATAAGTTAAGTCAACTTCCTAGAGGTAGTTTATTTGTTTGTGGAAAT
TATCTTGTTCGATGTATGGATTTAGGTTGGTTAGAGAAGGTAGGTCCTCAGGAATTACTTGGAAAATTAA
GAAAGTCAAATCAAAAATATTTTTTATTGTTTAGAATAGTTATTGTTATAATTTCTTTAGTGTCTTTGTGTT
ACATGATGATAGGGGTTTTTATAGTAGTAGCTATTGGGATTTGTAGTGTTTCTCTAGAGAGCCGTTTACT
TTAGGATTTTCTCTAATCTCTGTGTGTTTAATAGTGTGTTTGCTTATGGTTGGGGCTAGAAGGTCTTTGTT
AGCTTTTTTAGTTTTTATGAGGTATGTAAGTGGTGTTATAATTTTGTTTCTTTATGTATTAAGTGTTCATCCT
AATCAGAGAATGTCAAGTGGCAAAAAATTTCTTTGTTTTTTTTCTTTTGTTTTAGGGATTTTGGTTTGGTA
TGGATATGGTAGTGGTCTTGAAGGTTTTTGTTTTGTAGGGATATCTAGTCTTTTTTTGTTTATAGGGTTAGT
CTTATTGTTTGTTTTATTTGTAGTGTGTTATTTATGTAAAAAGAAGCGTTTACCTCTTCGTTCTATT"/>
<sequence id="seq_Mytilisepta_virgataKX094521" spec="Sequence"
taxon="Mytilisepta_virgataKX094521" totalcount="4"
value="ATGGTAATAGATGTATTTTCTGTGTTTGACAATGATAATTTTAATTCTTTTTCTTTAAATGTTGTTTG
AAGTTGAAGATTGTTTCTTCTTGTTACTTTTTTAAGAAAGTTAATACGTGTATTTTATACGTTAGGAAGAT
TCGTTTTAGACTTTGTTGGTAAAGTAGAAGGTTTAAAATTAACGGGTTTTGCTGTTTCTATTTTTTCATTG
TTTAGTTTGATTTTATGAATTAATTTGAGGAGTGTTGTTCCTTATTTTTTTCCTGTTAGGTGTCATGTTCCT
TATATTGCTTCATTTTCTTTGTATGTTTGAATAGGTTTGGTTATATCTAGGTTAGTAAATAGGTGAGTGCAG
GTTGTAGGAAGTTTGGTTCCGTCTGGTCCTATGAGATTATCGCCGTTATTAGTATTAACTGAGATTGTTTC
TTCTGCTGTTCGCCCATTGGTTTTGGTGATGCGATTAGTTTTTAATTTGGTGACGGGTCAGATTCTTTTTG
GTTTGTTAGGGGAAGTTTTTTCTGAGGCTCTTTTGATAGGCTCTGTTTTTAACTTGGGTTTGTTGACAGT
AGTTATAGTTTATTTTTTTGAAATTTTTGTTTGTGTTTTGCAAGCTTATATTTTTTGTCAGCTTCTTTGTAGT
TATAGTGAGGATCATTCATCATTTCGATGATTGATAAGTACTAATCATAAGGAAATTGGTAGTTTGTATTTA
ATGTTTGGAATATGGAGAGGTTTGGTTGGTGTAGGGTATAGGATGTTAATTCTAAGGATACATCCTGGGA
ATTTACTTTTAAAAGATAGTTTGTATAATGTGATTGTGACTAGACATGCACTAGTGATAATTTTTTTTGCTG
TTATGCCTTTATTAATTGGTGCTTTTGGTAATTGATTAGTACCTTTGTTTTTAAGTGCCATAGATTTGGTGT
TTCCTCGTATTAACAATTTTAGGTTTTGGATTTTACCTAGTGCTTTGTATTTATTATTGCTTTCTGCTTATGT
TGAAGATGGTGTTGGTACTGGTTGAACTATTTATCCTCCTTTATCTATTTATACTTATCATAGTTCTCCTGCT
GTTGATTTGGCTATTTTATCGTTACATTTAGCTGGAAGCGGGTCTCTGATGGGAGCTATCAATTTTTTAAC
TTCTAATAAAAGATTGCCGGTGGATAAGATAAAGGGTGAGCGTTCTGTTTTATATGTTTGGAGAATTACT
GTGACTGCTTTTTTGTTACTTTTGTCTTTGCCTGTGTTGGCTGGTGGTATTACTATACTGTTGTTTGATCGT
AATTTTAATAGGACTTTTTTTGATCCAATAGGTGGAGGGGATCCTGTTTTATTTATACATTTGTTTTGGTTT
TTTGGTCATCCTGAGGTTTATATTTTAATTCTTCCTGGATTTGGGGTAATGTCACATGTTACTGCTCATTAT
GCTGGAAAATCGTCTCCGTTTGGTGCTGTTGGTATAATGTATGCAATAATTTCTATCGGTTTAATGGGGTT
TATTGTTTGGGGGCATCATATATTTACGGTTGGTTTAAATGTAGATACTCGAATATATTTACTTCTGCTACT
ATAATTATTGCTGTTCCCACTGGTATTAAAGTTTTTAGATGGCTTGCAACGTTGGCTGGAGGTCGAAAGT
CTTCTAGAACTCCTGTTTTGTGAAGAATAGGTTTTATTGTTTTATTTACTATTGGTGGATTAACTGGTTTAA
TTCTTTCATCTTCTTCGTTGGATATTAGGTTGCATGATACTTATTATGTAACTGCTCATTTTCATTATGTTTT
GTCAATGGGGGCGGTATTTGCTATTTTTTGCGCATTTACTCATTGATATCCTTTGTTCTATGGAAATAATTT
ACATGGTCGTTGAAGTAAGGGACACTTTTTTTCTATGTTTGTTGCAGTTAATTTAACTTTTTTTCCAATAC
ATTTTTTAGGATTAAGAGGGATGCCTCGTCGTTATTGTGATTATCCTGATTGTTATTCTAAGTGGCATTGAT
TGTGTAGGTATGGTGCTATTATAGCTTATATGTCTTTGATGTATTTTATGTTTTTATTATGGGAAAGAATGGT
TAGGAAACGTGGTGTGTCTATGTATGGGTCTAAAGTTTTCAAGATTGATTTTATGATGTAGGAGATGCAT
TAAATTCTTTTTATCATAATATAATATTGGTTGCGGTTTTTATTATTTCTCTTGTAGGATATTTCCTTTTCCGT
ATTAATTTTTGTAGTGTGAGATATCGCTCTTTTAAGCATCAAAATGTTTTGGAGTGGGTGTGAACTGTTGT

GCCTATATTAATCTTAGCTGTTTTATGGGTGCCTTCGGTTCGTAATTTATATCTTATAAATCATATTGGAGAG
CCTAAGTGGTCTTTTAAAGCTGTTGGTCATCAGTGGTATTGAACTTATGAGGTTTATCAGGAGGTTGTTC
TTGAGTCATATATAGATAATATTGATGATGGAaaaTATCGACTTTTAGATGTTGATCAGCGTATAGTTGCTCC
TGTGAATTTGCAGTTGCGTGTTCTTGTTAGAAGTGTGGATGTTTTGCATTCATTTTCTTTGCCTTCGTGTA
TATTGAAGGTTGATGCTATTCCTGGGCGTATAACACAAACACCTCTTTTAGTTGATAAGAGAGGAGTAGT
TTATGGTCAGTGTTCGGAATTATGTGGTGTAAATCATAGATTTATGCCTATCGTAGTTGAATTTATTCCTGT
TAGTAGATTCTTGGAGTGGttaaaggttactgagtaa-----------------------
GTGGTGCGAAGTCCTTATTATCGTGTTAGACCTAGTCCTTGACCCCCTTTAGTAGCTTTTTGTTTGGTTAA
TATGGCTATTGGTTTAGTTAGTTGAATGTATCGGGTTAATATTAATATTATTTGGGAGGTTTATTATTGGTT
AGTTGTTTATATTTATGGTGGCGTGATGTGTTGCGCGAAGGGGATCAGGGTTATCATAGGAAATATGTTAT
TAAAACATATCGTGATGGTATAATTATATTTATTGTTTCTGAAGTTATATTTTTTTTTCTTTTTTTTGAGCAT
TTTTTCATAGGAGTTTAAGACCAAATGTTGAAGTTGGGGGTAATTGACCTCCTTGCGGTATTCGTAGTCC
TAATGCTTTTTCGATCCCTTTATTAAATACTTGGGTTTTAGTCACTAAAGGTATCTCGGTAAATTATGCACA
TAATTCATTGAAATGTGATTATGATTATGGGTCTATTATTGGGATAATTTTTACTATTGTTTGTGGAATTTTT
TTTGTGAAATTACAATATATGGAGTATTTTAGCAATTCTTTTTGTATTTCTGATAGTGTATATGGAAAAGTT
TTTTATATATTAACTGGCTTTCATGGTGCTCATGTAATTTTTGGGACTTTATTTCTAATAGTGACTCTTGGTC
GTTTATGATTTGGTCATTTTTTATTAAATCGTCGTTTTGGGTTTGAAGCGTGTGTTTGATATTGACATTTTG
TTGATGTTATTTGAATTGCTGTCTATATTTTTGTTTATGTGTGAGGTGGGGGTCCTTTTCGTAAACGACATT
GATTTTTAAAGGTTGTTAGAGCTAGGGTTTATGATTTACCTTGTCCTATTAATTTGAGTGTATGGTGAAGT
TTTGGTTCTATATTAGGCTTATGTTTGGTTATGCAAATTATTACTGGGTTTATGTTGTCTTTTTATTACATTC
CTCATGCTGATATAGCTTATGATTCCGTTATTTATATTATACGTAATGTTCATAAGGGTTGGATGGTTCGTAG
GATTCATTCAAATGGTGCTTCTGTATTTTTTATATGTATTTATGTTCATATTGCTCGTGGGTTATATTATGGTT
CTTATTTAGATAAAGGTGTTTGAAATGTCGGGGTAGTTTTGTATCTTATGGTGATGGCTGAGTCTTTTTTA
GGTTATGTTCTTCCCTGGGGTCAAATATCTTATTGGGGGGCTGTAGTTATTACTAGAATGTTAACTGCTATT
CCTTATGTAGGTCATAGGATTGTAGAATATGTTTGAGGAGGTTATGTTGTTAATACTCGTACATTAACTCG
GTTTTATTCTTTTCACTTTATTATTCCATTTTTAATGATTGTGGTAATTATACTTCATCTTCTTTATTTACATG
ATAAGGGAAGGAATAATCCGTTGGGAATTAGGAGTGATTCTATATTAATCCCTTTTCATCCTTTTTATACTG
TTAAAGATATTGTTGGGTTTGTTTCGATGTTTATAGTTCTAATATACTTTGTTTGTGTTAAACCTGAGGCAT
TAGGTAATCCGTTGAATTATATTCCTGCTGATAGATATAAAACTCCTGTTCATATTCAACCTGAGTGGTATT
TTTTATTTGCTTATACTATTTTACGTTCTATTCCTCATAAGGCCGGTGGTATTTTAGCTATGTTGGGATCTAT
TTTTTTATTATTTTTGATACCTTTTATTCATACTGGACAGTTTCGGAGATTAAGGTTTTACCCCATTCATCA
GCTTCTTTTTTGGTTTAATGTTAGTGTATTTGGTGGTCTAACTATAATTGGTATACGTCCTGTGTTAGAGCC
TTATTATACTGTTGGACAGTGTTTAACTGTTTGTTATTTTGTTGGGATATTATTGTTACCTTTGACCTTATTT
TTATGAGATTTTCTAATTGCAATGTGATTAATCTTAGTAAGATTACCTTTTATTTGTGTATTGTTAGCTGTAG
GATTTTTTACATTGTTTGAACGGAAGTTGTTAGCAGGAATAATGTTGCGTAAAGGTCCTAATAAAGTTGG
TTTCATGGGTTTGTTACAACCTTTTAGTGACGCAGGAAAGCTTTTCTGTAAAGAGGTGAACATTCCAAG
GTGTTCTAATGTTTTGCCTTTTGTGGTTGCTCCAATATTTATATTAATGATTTCTATGAGTTTATGAATTTTA
TACCCCTTTAAAAGCGTTAGTGTTTTATTTATTTTTGGCATTTTACAGTTTTTAGTTACTGCTGGAGTAGCT
GTATATGGTGTTATAGTAGCTGGATGGTCTTCTAATTCTAAATATTCTTTATTAGGGTCAGTTCGTAGAATT
GCGCAAAGAATTTCTTACGAAATTTCTTTTGGATTAATTGTGTTCGTATTGGTGTTTATGTCAATGAGATT
TTTTATTCAAGAAATCTCGTTTGTTTTTATATTTGTTTTGTATTTAATAGGGTTAATTTTGTGGATAGTTTGT
ATTTTAGCAGAAAATCATCGTGCGCCCTTTGATTTTGTGGAAGGTGAATCTGAGTTGGTGTCTGGCTTTA
ATGTGGAATATAGAGGTGGATTGTTTGCTTTAATTTTTATATCAGAGTATGGAAGAATATTGTTCTCTAGG
ATTTTAAGGGCCTGTTTGTTTTTTGGGGGGAGAGAGATATTTATTAGAGTTATTTTTTTGTTTTTTGATTAT
TTTTTTGTGTGAGTTCGTGGCAGGTTCCCTCGGATACGTTATGATAAATTAATAAAAATGGGGTGAACTAT
TTTTATAGTTATTCCCCTTATGTTTGTATTATTAGTTTTTTATATTCTTTGTTTTTATTTAAAAATTGTAAATTT
AACCCTATAAGAGTTTTAAGAATTGTAGTTATAGTATTTAGAAGATTGGTGAGAGTCTCTAGGAGTACTT
GATTAGGTGTATGAGTAGGTTTTGAATTAAATCTATTAAGTTTTATGGTATTAATAAACTTAGAAAGTAAAT
GGGCTATTAGGCCTTGTACTAAATATTTTATCATTCAATCATTAGGTTCTGGTTTAATTTTGATAACTTTCTT
ATGTGATGAACTTTACTCTGATGAGAATGAGCTTGTATTTTGTGCTGGTGTTATGTTGAAAGGTGGAATT
GCACCTTTTCATTTTTGAGTCCCTTCAATTGTTAATTCTACTACATGACTAGCAGGTGGTTTAATTCTTTCA
TGACAGAAATTAGCTCCTCTTTTTTTAATGGGATGGTTGTTTAGGGATTGAATAATTATTGTCGGTGCTAG
TCTATCAGCTTTAATTGGTGGTGTTGGTGGGTTAAATCAACATTCTATTCGTGGTTTAATAGTATACTCTTC
TTTTGTACACACTTCATGAATAATGTTAGCTTTGATTAGGTCCTTTTCATTATTTGTTTTTTATTGACTTGTT
TATAGAGTAAGGTTAGTTTTAATGTTTTGATCTTGTTTTAAAGTTAGAAAACAATTTCTTAAAAGTAAAAT
GCGAGTATTCCATAGATGTTTGAGTCTTTTAATGTTGAGAGGTCTACCTCCATTCTTAGGTTTTGTATCAA
AGCTTTTAGTAATAATGTCAATTAACAGAGTTGTAGTGTTTGTTTCTGTAATTGGTTCAGTTATTAGGTTA
AAATATTACTTGTCTGCTCTTTACTCTTTTATTTTTGGTGATACAGATTGTTCTCAGTTTATTGTAATTGTAA
GTGTGTTTTTAAATGTTTTAGGGTTTCTGTTAGTAGTAATTGGGTGTTATCAAAGATTTATGTTGATAAGA
ATAGTGTTTATTATTATTTTATGTTTTTTGTTATTAAGATTATGGTTTTTGTCTATAAAAACTCGTGAAGATT
ATCAGAAGTCATCTCCATATGAATGTGGGTTTGATGGGTTATTGAGAGCACGAAGTCCTTTTTCTTTGCG
GTTTTTTTTGGTGGCTGTTATATTTGTAGTGTTTGATGTAAAGGTTGTTTTAGTTGTGCCTATTATTTATTCT
TTTTGATTTTTTAAAACTGTTATGGCTATAGTTACAAGATGCTTATTTTTACTTGTTTTATTTGTTGGTTTGT

ATCACGAATATCGTGAAGGTtctttagagtgggtagattaaATGGTGTTAGGGTTAATATTTAGTGTAATATTTTTATTG
GTGTTAAGAAATGTAAAAGTAAGATTAGTGGGTTTTATAGTAATATTAGTTTTTGCAATAATAATATTAGG
GTCAATATCTGTAAGCTATGAGCTATTAGGTTTATTTAATCTCGACTTTCTTGCGAGTAGAATAATTAGTTT
AACAGTTTATGTGATTAGTCTGATAATCTTTATGAGGTTAAAAGTAAAGCGTATGAGGTTGATAAATTTTA
TTAATCTTAGAATAAGTTTAATTTTGATTTTAGTTTTTACTGTAAGAAATTTCTTTCTTTTTTTTTTTTTTTT
TGAAAGTTCGTTAATTCCTGTTATATTTATAGTAATCTTTTGGCGTCTTCAGGCTGTTAATTATATAATAATT
TATATGAGAGCTGGTTCTTTTCCATTACTTTTTGGTTTAAGTAATTTAGTTTATTGTGGTAGTGATAGAATG
TCCGTTTATAATAGTTTATTCAAAGGTTGTTGTTGATTTTATTGGTTTTATTTGATAGGGTTTTTTGTTAAAT
TGCCTATGTTCCCTTTTCATTTATGGTTGCCTAAGGCTCATGTGGAAGCTCCAGTGGTTGGATCAATAATT
CTTGCTGGTGTTTTATTGAAGTTAGGCGGATATGGTATTATTCGTTTTTTAGGATTAACTATAATTCCTATAT
TAAGGAGTTCTTGTATTTTAATTTCTGTTGCTTTATTTGGGGGTTTTATAGCAGGAATTATATGTTTGATGC
AGGTTGATTTAAAGTCTTTAGTGGCATATTCTTCTGTTGGTCATATAAGTTTGTTAGTGCTTACTCTTAGTC
AAAGTGATGTGAGGTTTTATGGTTCTATTATTTTAATAGTTGGTCATGGATTGTGTTCTTCGGGTTTGTTTT
GTTTAGTATATATATTTTATTGTATTTCTGGTTCTCGTTCAGTGGTTTTAAATAAGGGGTTTTTATTAAAAGT
TCCTGCATTTGTATTGTGTTGTTTTGTATTAAGAATTGGAAATATGAGTGCGCCGCCTAGATTAAATTTTGT
TGGGGAAATTATATTTTTTATAAGTTGTAGAATGGTCTCTTATTGATTTGTTTTAATTTTTTCTTTGATTAGA
TTTGTGGGAGCATGTTACAGTTTATATTTTTATGGTGTTTGTTGTCATGGGAAAGAAAATAGTCTTTTATAT
TTGGGGGTTTCTTTTAAGGATTTATTTGTATTGTTTTTGCATATCTTTCCTTTAAATagtttgtttttgtttttgtaatagGTC
CAAAGACGGCATCTAATTAGTGTGTTTTTAGGAATAAAGTTTATAGCTTTAAGTTTTATGTTGGTTGGATC
TATTTCAGTTTATAGAAACTCAAGGTATGTTTTATTAATTATATGTATAGCTGTGTGTGAGGCAAGGGTTGC
TTTAGCTTTAATTGTTAATATAGTACGTGTTTACAGAAGAGATCGTGTAATAATAttgagtttggatagATGAGTGT
TGTAGGAGTTTTAGGGTTTTTAAGTGTTTTATTAGGGTATGTGATAATAATAAGTTCATTTTTTCATTCTTG
TGTGGTTTTGGAGGTAGTATTATGTAGGATTAATAGGTGTGAAATTGGGGCGAGATTTTTGTTAGATGAA
GTTAGGGTTATTTTTGGAGGGGTGGTGTTAGTTATTTCTGGTAGTGTGGGAATTTATAGAAAATGGTATAT
GAATGATGAAGTTTTTTATTGACGTTTTATGATTCTCATTTATTTATTTGTTGGTTCTATGCTTATACTAATT
TTTAGGTCTAATTTGATTGGATTAATATTGGGATGAGATGGATTAGGTTTAGTTTCTTTTCTTTTGGTATGT
TATTATCAGAATCCATCTAGATTAGGGGCTTCAATATTGACTGTTTTAGTTAATCGTGTAGGGGATGTGTTT
ATTTTAGCAAGAATTGGTTTAATGAGAAGATGAGGAGATTTTATAGTGTATGAACGGTCTATGTTTGAGA
ATTTAGGAGTGAGATTATTTGTTGTTGTAGCTGGTATGACTAAAAGTGCGCAAATACCTTTTTGTTCTTGA
CTTCCCGCAGCTATAGCTGCGCCAACTCCTGTTTCATCTTTAGTTCATTCGTCAACTTTAGTGACTGCAG
GGGTTTATTTAATTATTCGTTGTGTAAGTTTATGTGGATTATGTGGAATGGGGGTTTTAAAATTTATAAGTT
TGTTGACTTTAATTATAGCTGGAATAGCTGGTACACTGGAGAGTGATTTTAAAAAAGTTATTGCTTTATCA
ACTTTAAGTCAATTAGGGGTTATAATGTTTTCTTTAAGCTTGGGGTTTTATACTCTAGCATTTTTTCATTTA
GTAACCCACGCCTCATTTAAAGCGTTGCTGTTCTTGAGAGCAGGTGTGGTTATTCACTCTAATAAAGGGT
GTCAGGATTTGCGTTTGTTAGGTGAAAGGTGGAAAAATTTACCAATTAGAAGTGCAGCTATAGTTGTTGC
TAGATTTTCATTATGTGGAATTCCTTTTATGAGGGGGTTTTATTCTAAGGATTTGGTTATCGAGTTGATAAA
AAGTATGATTTGATTTTATTTTATTATATTATTGGGTGTAAGATTTACATCATGGTATTCTGTTCGTGTTGTG
TCTGTTGTTTTTGGTTTAAATAAATGTGTGATAAGAAGTGTTAGGATAAAGGAGTCGTTTGATGTGATTG
CTTCTTATTGTTGTTTATATTTTGGCGCAGTATTTAGAGGTTATTTAATAGTAATAAAAATAGAAATTTTTTT
TTATCAAAGGTTTATTGATTCTTTTTTTTTTTTTCTGTTACTTTTTATACCATTTGGGGGATTTATTTATTCTA
TGTTTGTGAAGGAAGTTGGGaagactTCTAATATTCATTTTGTTGTTTTGATGTGGAATTTTAAGTTAAGTCA
ATTTCCGGTTAGGTATTTGTTTAATTATGGTAATAACCTTGTTCGTTGTATAGATTTAGGTTGGTTGGAGAA
GGTTGGTCCTCAAGAGTTTCTTGGGAGAATTAGAAAAATAAATCAAAAATATTTTTTATTGTTGTTTGCT
GTTTTTGTTATTTTGATTTTGCTGtctttgtgaaatATGATAATAGGGGTTTATATTGTAATGGCTGTAATGATTTGT
AGTGTTTTCTCTAGCGAACCTTTTACTTTGGGTTTTTCATTAATTTCTTGTTGTTTGATAGTGTGTTTGTTA
ATGGTTAAAGTTAGAAGGTCTTTATTAGCTTTTTTGGTTTTTATAAGTTATGTTAGAGGTATTATGGTTTTA
TTTTTGTATGTTTTAAGTGTTCATCCTAATCAAAGAATATCAGGTGGTAAAATGTTTCTTTTAATTTCTTTG
GTTTTGAGATTTTTATGGTGATATGGTCATGTTGATAATGGTCTTGAGAATTTTTGTTTTGTTGGAATAGTT
AGGCTATATTTGTTTATAGGTGTGGTTTTGTTGTTTGATTTGTTTGTTGTGTGTTATCTTTGTAAAAAAAAA
CGTtaacctcttcgttctatt"/>
<sequence id="seq_Perna_canaliculusMG766134" spec="Sequence"
taxon="Perna_canaliculusMG766134" totalcount="4"
value="ATGTTGATAGATGTTTTTTCCATGTTTGATGATTATAACTTTAATACTTTTAAAAGGTGGTATGTGT
GGGGATATTCTTTGTTCCTTCCTCTTTTTATTATTTCGTCTAGTATGTCGATTTATTCTCTGTTTTTGCAAAA
ATGTATAAAATTAATTAGTAGAGGTAGAGGTATATATTTATCAGGCTTTCCTCTTCTTGTAGCTAGTATATTC
TCTATCATATTAAGCGTTAACGTATCTAATAGTATTCCTTATTTTTTCAGTGTAAGCGCTCATTTTTCATTTG
GATTTACCTTTGCTTCAGTAATTTGGCTATGTTTAATTATGTCTAGTATTTTTACTAGGTTTATCCAAAATAT
AGCTCTTTTGGTTCCAAGAGGGCCAGAGGGGTTAGCTCCATTTATAGTTCTTCTAGAAATTATTACGAAC
TTGCTTCGGCCTATTACTTTGATTATTCGGTTAGCAATAAATATAGCTACTGGTAAAGTCATTATGCTATTA
CTTGGGAACGCTGCCTTGAACTTGGCTTTTCTAAGAACTTGTGGGTTTGTAGTTATTAGGGTTTTAGGGG
CTATTTTTTCTTTGGAAGTAGCAGTAAGTTTTATTCAAAGGTATATTTTTTGTACCCTTTTGTGTCTTTATG
CTGATGAGCATAGAAAATAGCGCTGGTTTATATCAACCAATCATAAAGATATTGGTACTTTGTATTTGATTT
CGGGTATGTGGGCTGGTCTTATAGGAAGAAGTCTAAGGTTAATCATCATTCAATCTCATCCAGGGGGGAG

TTTCTTAAAAGAGAGTCTGTATAATGTAGTAGTAACTACGCATGCCCTTGTTATAATTTTTTTGCTGTTAT
GCCGTTGTTGATCGGGGCGTTTGGAAATTGATTATTACCTTTATGTATTGGAGGGTGTGATCTTATTTTTCC
TCGTTTAAATAACCTTAGGTTTTGGTTAGCTCCAAATGCGTTATATCTTCTTATCTTGTCCTTTATAACGGA
AAAAGGGGCAGGTACTGGTTGGACTATTTATCCTCCACTGTCTTCAGGGCTGTATCATACTGGACCGGCA
GTTGATATTTTAATTACTTCTCTTCATTTGATTGGTCTGAGATCTTTGTTAGGGTCTATTAATTTCGTAAGG
ACTAATAAAAATATACCTACTGTTAAGATAAAGGGAGAAAAATCAGAGTTATACCTTTGAAGTATTACGG
TTACAGGAGTGTTGCTAATTATTTCTGTTCCTGTCCTTGCTGGGGGTATTACTATATTGTTATTTGATCGAA
ATTTTAACACTAGGTTCTTTGATCCTATCGGGGGAGGGGATCCTGTTTTATTTCAGCACGTGTTTTGGTTT
TTTGGACATCCTGAAGTTTATATCTTAATTCTTCCTGCTTTTGGAATTATATCTAAGGTTATTATGCACTATT
CAGGAAAAGATTCTGTGTTTGGTTCAGTGGGAATATTATATGCTATAGTTGGAATTGGGGTAATAGGGTG
TGTTGTTTGGGCACACCATATATTTACGGTTGGTTTAAATGTAGACACTCGAACTTACTTTACTTCTGCTA
CGATAGTGATTGCAGTTCCTACTGGTGTAAAAGTATTTAGTTGAATAGCAACTATAGGTGGAAGTAAAAT
TAAATTTAATTCTTCTGTACTGTGAAGTACTGGGTTTTTATTTTTATTTACTGTAGGAGGGCTAACTGGAAT
TATATTATCAAGGTCTTCTTTAGATGTTACGCTTCATGACACATATTATGTGACAGCTCACTTTCATTATGT
ATTATCTATGGGAGCTGTCTTTGGAATCTTCTGTGGGCTTAATCATTGATTTCCTTTATTCTATGGAGTGAA
TTTCCATAAAAAATGGTCTAAGATTCATTTTTATCTTATGTTTTTAGGGGTAAATTTAACATTTTTCCCACA
GCACTTTTTAGGGTTAAAGGGTATACCACGTCGTTATTGTGATTATCCAGATTGTTATTCTGTCTGACATT
GAGTGTCTTCATATGGAGCACTTATTTCATTTGGATCTTTGTTATATTTTATTTTCATTGTTTGAGAAGCTAT
AGTAAGTCAACGAGGTGTGTCATTATTTGAAGCTTTATTTTTTGGTGATGTATTGCAAGAGTTTGGATCTG
AGATCGTAAAATATTATGGTTATGTGATAATGGTATTATTACTTGTAATCATTGTTGTTTTGTATATAGGAAT
AATAATCATAAATCATAGATATTCTTATCGTAATTTTAAAAATCGTCAAATACTAGAGTATATTTGAACTGT
TATACCTACCTTTTTGTTAGCGATACTTTGGTGTCCATCTATCTTAAACTTATATCGAATAGAAGATCTAAA
AGATCCAGTTTGGAGGTTTAAGGCTGTTGGAAAACAATGATACTGGACTTATGAGAATGGGGAAACAAT
AGTAATTGATTCTTATATGGACCGGGATTCGGGTGTTGGATATCGCCTTCTAGATGTAGATTGACGTTTGG
TGGCTCCTGCTAACGCACAAATTTCGTGTTACGTTACAAGAAGTGATGTAATTCACTCTTTTGCCCTTCC
TGGAGCACTCTTAAAAGCAGACGCTATTCCTGGACGAATTAATGTTCTTCCAATAAAAATTAGTCAGAGC
TGTATTCTTTATGGTCAATGCTCTGAAATCTGTGGTATTAATCATAGGTTCATACCAATTGTTATTGAATTT
GTTCCAGTAGATGTATTCATAAGGTTCTACgctGGGTATAATTAA-----------------------
ATGCATCGAAATCCTTATTATGTGCCTGGACCAAGTCCTTGGCCTTTGGTTGTAGGTGTTTGCTGTAATAG
CTTATGTGTAAGCTTAGTATTATGAATACATCGAATACAAGAGAAACTATTTTTGACTTTTTTAGTTTTAGG
TGTAACTGCTACGTTATGATGAACTGATGTTTTACGTGAGGGAGATATAGGGGTTCAAACTCAACATGTG
TTAAAAAGGTACCGAGATGGTGTAGGTCTTTTCATCTTTTCTGAAATTATGTTTTTCTTTTCATTTTTTTGG
GCTCTTTTTCATAATTGTTTAAGGCCTTCAGGTAATCTTGGGTGTGAATGACCTCCTTTAGGGATCCGGAC
TCTTGACCCAATATCTACAGCATTATTTAATACTTTTCTTTTAATTAGTAGGGGGTCATTTTGTACTTATGTT
CATAACACAATTCGTAAATGTTATGATAGGTGGTGTCTTGTTAATATATTACTTACTATTACTTGCGGGGTT
CTTTTTTTATTAGGACAAGGGAATGAATATTTCTTTTGTTCTTTTAGAATCGCTGACAGGGTATATGGAAG
AACTTTTTATATGTTGACTGGATTCCATGGAGGTCACGTTATAGTGGGAACAACATGGTTAATTGTTAGGT
TCTTTCGGATGTGGCGCGGTCATTTTAGAAAAGATCGGCATTTTGGACTTGAAGCATGTTTGTGGTATTG
ACATTTTGTTGATGTAGTTTGGTTGTTTGTTTGATTAATTGCTTATTTTTGAATAGGTGGACCTTTTCGTAA
GCGCCATTGGCTTCTAAAAATTTAAATAATAGTCTTTATGACCTTCCCTGTCCTGTTAACCTAAACGCTT
TTTGAAGGTTTGGTTCTATACTAGGATTATGTATCGTAATTCAAATAGTTAGAGGTATTCTTTTATCTCTAC
ATTTTATTCCTCATGAGAGAATAGCATTCGATTCAGTGTATCATATTATGCGGGATGTTAATAAAGGCTGAT
TTCTTCGAAACATACATGTTGGGGGATGTTCTATATTTTTCATTTGTCTCTATGTTCACATTGGTCGTGGTA
TTTACTACGGATCTTATATAAGACGTCATGTATGGTTTGTAGGGGTAACTTTATTTCTAATAGTAATAGGCG
AAGCCTTTCTAGGTTATAGGTTACCTTGAGGACAAATATCTTTTTGAGGGGTAACAGTAATTTCTAATCTT
TTTACGGTGATCCCTTATGTATCGCAAAACTTACTATTTACTCTCTGAGGGCATTGAAGTGTAAGTGGGTA
CACCTTGCAACGGTTTTTTATCTTTCATTTTCTACTTCCCTTTGTCATCATTGTGTTTTCAACTCTGCATAT
ATTTTTTTTACATGAAAATGGGAGTAATAATCCTTTAGGTGTAAATAGGGACTCTATATGTGTGCCTTTTC
ACCCTTTTTACACTGTTAAAGATATTTTTGGGTTTGTGTGTTTCGGGTGGGTGTTAATATATTTAGTGTGTG
TAAATCCTGAGCTTGTAGTAAATAAAATTAACTATCATCCAGCCGATCCTTATCATACCCCCCTTCAGGTA
GAGCCAGAGTGGTACTTTTTGTTTGCGTATGCAATGCTTCGATCTATCCCTCACAAACTAGGAGGAGTTT
TAGCTTTGCTAGCATCTGTTTTAGGCTTATATTTATACCCTTTCATTCATACAGGAAAGTTTCGAAGGTTAG
CATTCTATCCTGTTAGTCAAATATTCTTTTGGTGCTTCGTTGTTAATTTCTTGAGCCTTATTTGAGTAGGGC
AAATACCTGTTCGTGAGCCATATATTACTATAGGCCAGGTGTATACAGGAGTCTATTTTTCTACATTAATTC
TGCCCCCTATGTTAACAGGGGTATGGGATAAATTAATTTTTATTTATTTTATTAGTATTATTCTTCCTTTTGT
GTGTGTGTTGGTAAGGGTGGCATTTTATACCTTGCTGGAGCGTAAAGTGCTTGGGTATATTATAATTCGAA
AAGGGCCAAATAAGGTTGGATACGCGGGTATTATACAACCCTTTAGAGATGCTGGGAAGCTTTTTAATAA
GGAGTATGTAAAGCCTGGTTTTGCTAATCTAGTGCCTTTTTTATTATGTCCTGGCATTATTTTGGTAACTAG
AATACTATTATGGCTTCTTTATCCGTATGATTATACATCTGTAATACTAACTTGCGGACTAATACAGTTTTTA
GTAACGTCAGGGATGCACGTGTATGGTGTAATAGTAGCTGGATGGTCTTCTAATTCTAAGTATTCTTTGTT
GGGAGCAGTTCGAGGAGTAGCTCAAAGAATTTCATATGAAGTACCGATAACTTTTGTTATCTTAATTGTT
GCTTATTCATTGAGAAGATTATGGATTCAAGAAGTTAAGGGAATCTTTTCAATAGCGATAGGGTTACTTAG
AAGGGGGGTATGAATCACTTGTATGTTGGCTGAGACCAATCGGGCGCCGTTTGATTTTGTGGAAGGGGA

GTCTGAGCTAGTTTCTGGGTTTAATGTAGAGTATAGTAGAGGAGGATTTGCGATAATTTTTATGGCTGAGT
ATGGGGCTATATTATTCAATAGTGTTTTCTTTGTGACTCTTTTTTTAGGAGGAGGGGAGTTATTAATAAGA
GTAAGAACAATAATTTGGGTTCTATTTTTTATCTGAATTCGTGGAACTGTTCCACGAATTCGATATGATCA
GTTAATAGGGTTGTGTTGGAAAGTTCTCCTAAGGGTAATGTTGAGTATAAGAGGGTTAATTTTAATTATCA
GGTTTTCCTTATGATAGGAGTTGCGTTAAGTCCTCTATTGATTTTTTGTGGGGTACTAGTAATTATAGGTAA
TCTCTTAAGAATTAGAAGTAGTTCTTGGTTAGGGGTGTGGCTAGGAATGGAAATTAACCTTTTTGGGTTT
TTGATTATAATAAATCCTGAAGGTTGTTGTGTTGCTCAGAGGGGAGTAAAGTATTTTGTTACTCAGAGAA
TTGGGTCTATACTATTATTATTTGGGTTTTTTTTAGTTTTTCTATTTAATAGGGTAGGGGCTGTAGTTATCAT
AATAGGATTGTTAATAAAAACAGGAATCTTTCCATTTCATAACTGGGTTCCAGACGTTGTTATAGTTTCAA
GTTGACTAATTGGTTGTTTAATTTTGACTTGGCAAAAGTTAGCCCCATTTTCCTTTTTTTCATTCTTCCCC
GGTTCTTGGTTTCTTATTACTAGGTTGGTTTTTATGAGCTTAGTAGGCTGTCTTGGTGGATTAAATCAGAA
TTCTGTTCGGGGAATAGCAGTTTATTCTTCATTTGTACATAATTCCTGGATAATACTTTCTTTACTTTACTC
ATTTAGGGTATTTTTTGTTTATTACTTAATTTACAGATTGAGAGTGGTGGTATTTTTTTTTAGGTGTTGAAC
TCTTAGAAAAAGTAGAATGATGAGATATTCAATTAGGTGGATAGGGATGCTAAGACTGTTAATATTGTCT
GGGGTACCCCCCTTCATAGGATTTTTTATAAAATTGATTGTGGTATTAAGGTGTCCTATTATATTATTAATCA
TTTGTTTGTTAAGGTCAGTAGTTAGATTGAAGTTTTATATATCGTTTTTTTACAGAATAATTTTAAATAGAA
TTTATTGTGTAAAGTTATTTTTAGCATTTAGTCTAGCTAACTTTATTATAGGAAGAATTTCTATAAGGTACT
TTATTTGGTAGATGATCGAATGTTTAACCCTTGTGTTTCTTATTGCTTTAAGACTAATTTTGATACTAGTAG
CTTTGTTATCTGAAAAATCATTTGAAAGTCGAGAAAAGTCTTCTCCGTATGAATGTGGTTTTGATCCTATT
TTAAGGGCTCGAAGTTCTTTCTCATTACGGTTTTTTCTTCTTGCAGTTTTGTTTGTCGTTTTCGACGTAGA
ATTGTCTTTATTAATACCTGTAGTAACAAGGTTGATAAGAGGTTTTAATTATATTAGTCTGGTTAGATGCTT
TTTATTTTTAGTAATTCTATTTCTAGGCATTTATCATGAGTGACGAGAAGGGTCTTTGAATTGAGTAGTTTA
AGTGGCCGTAAGTTTGATTATTTCTTTGATTATACTCTTTGTTTTAGGTAATAGGACAATTAGGTTAATAGT
TTTATTTTTCTTATTTTTAACTGTTTTAGAGATAGGCACTATCAGGGACGAATTTGTAGCATTAGGCGGGA
GGGTAAGGTACGATTTATTGTCTTATAGGTTAAGTGTTCTAAGAATTTTTATTATATTGCTTAGCCTGATAA
GGAGTAAGAGGGTACAACGAATTAAGTTATTTTATTTTATGAACTTAGCTTTGTTAATAGTCTTAGTTTTA
GCTTTTTGCACATCTAGTTTTTTGGGTTTTTTCTTTTTTTTTTGAATCTGCTTTAATTCCATTGATCCTTATTA
TCATGGGCTGGCGGATGGAGGCAAGGACTTATATATTTATTTATACGGTGTTCGGATCATTGTTCTTCTTAT
TTGGAGTGTGTTTGTTGTTTTATAGGGGGAGGGATAATATGTTATTAGGAAGAAATATTTCTAAAAAAGT
GAGAGGGTTTTGGTGGTTGTTTGTTCTGGGGTTTCTAGTTAAATTGCCGGTTTATCCATTTCATTTATGAT
TACCTAAAGCTCATGTCGAGGCGCCTGTAGCTGGTTCAATAGTATTAGCTGGAGTTGTTCTTAAGTTAGG
GGGCTATGGATTACTTCGACTTATAATAGTGATAAGAGTAAATTTAGGGTTAAAGTCTAATATAATGTTGA
GAATTGGGATGGTAGGTGGTGTATATGCTAGATTAGTCTGTTGCCGCCAGACAGACCTAAAATGTCTAGT
CGCTTACTCTTCTGTTGGGCATATAAGGCTGGTAATGCTATGTCTTAGAAATATTAGAATAGGGGTTAAGG
GAGCTCTATTTATTATAGTAGGTCATGGTCTATGCTCCTCTGGGATGTTTAGGTTGGTGAACGTATTTTACA
ATCATTCAGGGTATCGTAATCTTTACATAAACAAGGGTTTCCTGATGAAAAGTCCAGTTTTATGTTTAGTA
GGATTTCTTCTGTGTTCTAGAAATATAGCAGCCCCTCCCAGTCTAAATTTGTTTGGCGAAGTTTTAATGTT
TGTGTGTTCTTACTTGATTTCATTCTGTTTCTTAGTTTTACTAATGGTAATAACAATTATTAGAGCTGTATAC
AGACTTCACTTATACACAACAACTTGTCATGGAAAATCTTCTGAAGCCAAGGTTAGGTTAGAGACTTATA
GGGCTGTTTTCGTATTATTAGTACATTGAATTCCATTGAATTTCATCTTTTTGTTTATTCCTTAGACTAAAA
AAAATCATTTAATTAGTGTATTTATTGGGATAGAGTTTATAATATTAGGAATTTTATACTGCTGTAGTAGATT
AATATTTTATAATATTAGAATAATTTTTCTAGTAATGTGTTTAGGGGTTTGTGAGGCAAGGGTGTGCTTAGC
TCTTTTAGTAATAATAGTACGACTTTCAGGGAATGATCTGATCTCGAGACTTTCTTTGTACAATTTAATAAT
TTTTCTCAAAAAATGAGGTTGATAGTTATATTAGGCTATGTATTGGTGTTATTAAGGAGGTTCGAAAGTGT
TTATTTTTTTGAATTCATGTTATGGGATTTAAACAGATTAAGGTTCTCTGTAAATTTACTCTTAGATTCATT
GAGGATAATATTTTTAGGTACTATTTTAATTATTTCAGGTAGGGTTTTAATATACTGTTATTGATACATGGAT
GATGAAAAGAACTTTTATCGATTTATATATTTAGTCTATTTATTTGTAGGGTCTATGATGTTTATGATTCTTA
TTCCTAATCTTGTTACCCTTCTTATTGGCTGGGACGGTTTAGGGCTTACTTCTTTTTTGCTTGTTGCACATT
ATCAAAATAATAAATCTTTGTCAGGAAGTTTGTTAACGGCTTTAACTAACCGTATCGGGGACGGTCTGAT
CTTGGCTAGAATTTCATTATGAGCGGTTGAGAGGGGTTGGGTATTGTATAATTTTGAAAGTCAGTTTGTG
AATTTTTTTATTTTAGCCTTAATTTTTGGAGGAATAACAAAAAGAGCTCAAATACCTTTTTGTGCTTGGTT
GCCTGCTGCTATAGCTGCTCCTACTCCAGTTTCTTCTCTAGTTCATTCATCTACACTAGTGACTGCTGGGG
TATATTTATTAATTCGTTCTTATCCAGTAATTAAGGGAAGCTTGATGACTAGTTTAAAGTTTCTTAGTTTGT
TTACATTGGTTTTAGCTAGAAGTGTTGCTATTAACTGTTTTGATATAAAGAAAATCATTGCTTTGTCGACT
TTAAGTCAATTAAGGGTGATAATATTTTCATTGTCTCATGGTCTTGTTTCTATTTCTTTTTTTCATTTGGTAA
TGCATGCCCTATTTAAAGCTTTATTATTTTTAACAGCAGGTGCAGTAATTCATTCTCTCAAAGGGTGTCAA
GATATTCGAAGGATAGGGGGATGCTGGAGAATTTTACCAAGTAGAATAGTGTGTATGTTCATTGCTAATT
GTTCATTGTCAGGGCTTCCATTTATAAGAGGGTTTTATTCTAAAGATATAATTCTGGATATATTTAATAGGA
GAAATTTTTGGTTTATAATTATTATACCGGGGCTTATACTTACTAGATTTTATAGCATGCGAGTTTGAATAG
TTTGCTTCGGAAGGAATAAGGTATCAATTAGTACACTTTGTTTAAAAGAGCCAAAAAGGCTACTTATTCC
TTATTCGCTACTAGCAGTCGGTGCTGTGTTTCTAGGAGGAGTTTTAACTCCTAGTGAGTGTCTGTTTAGTT
ATCGGAGATCTTCGGCGAGAGAAATAGGAACTTTAGGGGTTCTTTTTGTTTTTGGAATTGGACTAATTAG
AGTTTCTAGAAAGTTTGGGAAAGATTTCCGGGGCTTTTCGTTCCTATTTAGAATGTGATTTTTAGAGTTG

ACTCACGGAGTAAAAAAATGTTTTTTTGATGTAAGGGGGACAATTTTGAAAAGATGTGACAAAGGAATA
TTAGAGACTGTAGGTCCTAAAAGATTCTGTATAAAATTAAGTTCTTATAATGAAGATTATTTTTTGCTATTT
TTTTTTTTTATTGGAGCTCTCTCTTTTCTTTTCTTTTGAAATATGCAACTCCTTTTAATTTACTGAACTGTA
ATTAGTTTGTGTGTGGTTTTTATTGGACAGCCTTATCTATTAGGGCTAGTGCTGTTGATGACTTCTATGTTT
GTGTGTATGATTATTGGGGTAAATGTAAGAACATTTTTAGCTTTACTTTTATTCATGAGATATGTGGGGGG
TTTAATGGTTCTTTTCATTTATGTGTTAAGTGTATTTCCTAATGAAAATTTCGAAGTAATGGGCTTTATGAT
TGTGAGTGTGGCAATAATGAGATTTTTAATATTTCCAGTAAAAAGGCAAAGAAAGGGATTATTCtttCACTA
TATAAGGTTTGTGGGGGTTTTTGTTTTTATAGCCCTTTTTCTTTTATTTATTATGATGTGTGTATCGTATCTT
GTAATAAAAAAACGAATGCCATTGCGTGTAAAT"/>
<sequence id="seq_Perna_canaliculusMK775558" spec="Sequence"
taxon="Perna_canaliculusMK775558" totalcount="4"
value="ATGTTGATAGATGTTTTTTCCATGTTTGATGATTATAACTTTAATACTTTTAAAAGGTGGTATGTGT
GGGGATATTCTTTGTTCCTTCCTCTTTTTATTATTTCGTCTAGTATGTCGATTTATTCTCTGTTTTTGCAAAA
ATGTATAAAATTAATTAGTAGAGGTAGAGGTATATATTTATCAGGCTTTCCTCTTCTTGTAGCTAGTATATTC
TCTATTATATTAAGCGTTAACGTATCTAATAGTATTCCTTATTTTTTCAGTGTAAGCGCTCATTTTTCATTTG
GATTTACTTTTGCTTCAGTAATTTGGCTATGTTTAATTATGTCTAGTATTTTTACTAGGTTTATCCAAAATAT
AGCTCTTTTGGTTCCAAGAGGGCCAGAGGGGTTAGCTCCATTTATAGTTCTTCTAGAAATTATTACGAAC
TTGCTTCGGCCTATTACTTTGATTATTCGGTTAGCAATAAATATAGCTACTGGTAAAGTCATTATGCTATTA
CTTGGGAACGCTGCCTTGAACTTGGCTTTTTTAAGAACTTGTGGGTTTGTAGTTATTAGGGTTTTAGGGG
CTATTTTTTCTTTGGAAGTAGCAGTAAGTTTTATTCAAAGGTATATTTTTTGTACCCTTTTGTGTCTTTATG
CTGATGAGCATAGAAAATAGCGCTGGTTTATATCAACCAATCATAAAGATATTGGTACTTTGTATTTGATTT
CGGGTATGTGGGCTGGTCTTATAGGAAGAAGTCTAAGGTTAATCATCATTCAATCTCATCCAGGGGGGAG
TTTCTTAAAAGAGAGTCTGTATAATGTAGTAGTAACTACGCATGCCCTTGTTATAATTTTTTTTGCTGTTAT
GCCGTTGTTGATCGGGGCGTTTGGAAATTGATTATTACCTTTATGTATTGGAGGGTGTGATCTTATTTTTCC
TCGTTTAAATAACCTTAGGTTTTGGTTAGCTCCAAATGCGTTATATCTTCTTATCTTGTCCTTTATAACGGA
AAAAGGGGCAGGTACCGGTTGGACTATCTATCCTCCACTGTCTTCAGGGCTGTATCATACTGGACCGGC
AGTTGATATTTTAATTACTTCTCTTCATTTGATTGGTCTGAGATCTTTGTTAGGGTCTATTAATTTCGTAAG
GACTAATAAAAATATACCTACTGTTAAGATAAAGGGAGAAAAATCAGAGTTATACCTTTGAAGTATTACG
GTTACAGGAGTGTTGCTAATTATTTCTGTTCCTGTCCTTGCTGGGGGTATTACTATATTGTTATTTGATCGA
AATTTTAACACTAGGTTCTTTGATCCTATCGGGGGAGGGGATCCTGTTTTATTTCAGCACGTGTTTTGGTT
TTTTGGACATCCTGAAGTTTATATCTTAATTCTTCCTGCTTTTGGAATTATATCTAAGGTTATTATGCACTAT
TCAGGAAAAGATTCTGTGTTCGGTTCAGTGGGAATATTATATGCTATAGTTGGAATTGGGGTAATAGGGT
GTGTTGTTTGGGCACACCATATATTTACGGTTGGTTTAAATGTAGACACTCGAACTTACTTTACTTCTGCT
ACGATAGTGATTGCAGTTCCTACTGGTGTAAAAGTATTTAGTTGAATAGCAACTATAGGTGGAAGTAAAA
TTAAATTTAATTCTTCTGTACTGTGAAGTACTGGGTTTTTATTTTTATTTACTGTAGGAGGGCTAACTGGA
ATTATATTATCAAGGTCTTCTTTAGATGTTACGCTTCATGACACATATTATGTGACAGCTCACTTTCATTAT
GTATTATCTATGGGAGCTGTCTTTGGAATCTTCTGTGGGCTTAATCACTGATTTCCTTTATTCTATGGAGTG
AATTTCCATAAAAAATGGTCTAAGATTCATTTTTATCTTATGTTTTTAGGGGTAAATTTAACATTTTTCCCA
CAGCACTTTTTAGGGTTAAAGGGTATACCACGTCGTTATTGTGATTATCCAGATTGTTATTCTGTCTGACA
TTGAGTGTCTTCATATGGAGCACTTATTTCATTTGGATCTTTGTTATATTTTATTTTCATTGTTTGAGAAGCT
ATAGTAAGTCAACGAGGTGTGTCATTATTTGAAGCTTTATTTTTTGGTGATGTATTGCAAGAGTTTGGATC
TGAGATCGTAAAATATTATGGTTATGTGATAATGGTATTATTACTTGTAATCATTGTTGTTTTGTATATAGGA
ATAATAATCATAAATCATAGATATTCTTATCGTAATTTTAAAAATCGTCAAATACTAGAGTATATTTGAACTG
TTATACCTACCTTTTTGTTAGCGATACTTTGGTGTCCATCTATCTTAAACTTATATCGAATAGAAGATCTAA
AAGATCCAGTTTGGAGGTTTAAGGCTGTTGGAAAACAATGATACTGGACTTATGAGAATGGGGAAACAA
TAGTAATTGATTCTTATATGGACCGGGATTCGGGTGTTGGATATCGCCTTCTAGATGTAGATTGACGTTTG
GTGGCTCCTGCTAACGCACAAATTTCGTGTTACGTTACAAGAAGTGATGTAATTCACTCTTTTGCCCTTC
CTGGAGCACTCTTAAAAGCAGACGCTATTCCTGGACGAATTAATGTTCTTCCAATAAAAATTAGTCAGAG
CTGTATTCTTTATGGTCAATGCTCTGAAATCTGTGGTATTAATCATAGGTTCATACCAATTGTTATTGAATT
TGTTCCAGTAGATGTATTCATAAGGTTCTACgctGGGTATAATTAA------------------------
ATGCATCGAAATCCTTATTATGTGCCTGGACCAAGTCCTTGGCCTTTGGTTGTAGGTGTTTGCTGTAATAG
CTTATGTGTAAGCTTAGTATTATGAATACATCGAATACAAGAGAAACTATTTTTGACTTTTTTAGTTTTAGG
TGTAACTGCTACGTTATGATGAACTGATGTTTTACGTGAGGGAGATATAGGGGTTCAAACTCAACATGTG
TTAAAAAGGTACCGAGATGGTGTAGGTCTTTTCATCTTTTCTGAAATTATGTTTTTCTTTTCATTTTTTTGG
GCTCTTTTTCATAATTGTTTAAGGCCTTCAGGTAATCTTGGGTGTGAATGACCTCCTTTAGGGATTCGGAC
TCTTGACCCAATATCTACAGCATTATTTAATACTTTTCTTTTAATTAGTAGGGGGTCATTTTGTACTTATGTT
CATAACACAATTCGTAAATGTTATGATAGGTGGTGTCTTGTTAATATATTACTTACTATTACTTGCGGGGTT
CTTTTTTTATTAGGACAAGGGAATGAATATTTCTTTTGTTCTTTTAGAATCGCTGACAGGGTATATGGAAG
AACTTTTTATATGTTGACTGGATTCCATGGAGGTCACGTTATAGTGGGAACAACATGGTTAATTGTTAGGT
TCTTTCGGATGTGGCGCGGTCATTTTAGAAAAGATCGGCATTTTGGACTTGAAGCATGTTTGTGGTATTG
ACATTTTGTTGATGTAGTTTGGTTGTTTGTTTGATTAATTGCTTATTTTTGAATAGGTGGACCTTTTCGTAA
GCGCCATTGGCTTCTAAAAATTTTAAATAATAGTCTTTATGACCTTCCCTGTCCTGTTAACTTAAACGCTT
TTTGAAGGTTTGGTTCTATACTAGGATTATGTATCGTAATTCAAATAGTTAGAGGTATTCTTTTATCTCTAC

ATTTTATTCCTCATGAGAGAATAGCATTCGATTCAGTGTATCATATTATGCGGGATGTTAATAAAGGCTGAT
TTCTTCGAAACATACATGTTGGGGGATGTTCTATATTTTTCATTTGTCTCTATGTTCACATTGGTCGTGGTA
TTTACTACGGATCTTATATAAGACGTCATGTATGGTTTGTAGGGGTAACTTTATTTCTAATAGTAATAGGCG
AAGCCTTTCTAGGTTATAGGTTGCCTTGAGGACAAATATCTTTTTGAGGGGTAACAGTAATTTCTAATCTT
TTTACGGTGATCCCTTATGTATCGCAAAACTTACTATTTACTCTTTGAGGGCATTGAAGTGTAAGTGGGTA
CACCTTGCAACGGTTTTTTATCTTTCATTTTCTACTTCCCTTTGTCATCATTGTGTTTTCAACTCTGCATAT
ATTCTTTTTACATGAAAATGGGAGTAATAATCCTTTAGGTGTAAATAGGGACTCTATATGTGTGCCTTTTC
ACCCTTTTTATACTGTTAAAGATATTTTTGGGTTTGTGTGTTTCGGGTGGGTGTTAATATATTTAGTGTGTG
TAAATCCTGAGCTTGTAGTAAATAAAATTAACTATCATCCAGCCGATCCTTATCATACCCCCCTTCAGGTA
GAGCCAGAGTGGTACTTTTTGTTTGCGTATGCAATGCTTCGATCTATCCCTCACAAACTAGGAGGAGTTT
TAGCTTTGCTAGCATCTGTTTTAGGCTTATATTTATACCCTTTCATTCATACAGGAAAGTTTCGAAGGCTA
GCATTCTATCCTGTTAGTCAAATATTCTTTTGGTGCTTCGTTGTTAATTTCTTGAGCCTTATTTGAGTAGGG
CAAATACCTGTTCGTGAGCCATATATTACTATAGGCCAGGTGTATACAGGAGTCTATTTTTCTACATTAATT
CTGCCCCCTATGTTAACAGGGGTATGAGATAAATTAATTTTTATTTATTTTATTAGTATTATTCTTCCTTTTG
TGTGTGTGTTGGTAAGGGTGGCATTTTATACCTTGCTGGAGCGTAAAGTGCTTGGGTATATTATAATTCGA
AAAGGGCCAAATAAGGTTGGATACGCGGGTATTATACAACCCTTTAGAGATGCTGGGAAGCTTTTTAATA
AGGAGTATGTAAAGCCTGGTTTTGCTAATCTAGTGCCTTTTTTATTATGTCCTGGCATTATTTTGGTAACTA
GAATACTATTATGGCTTCTTTATCCGTATGATTATACATCTGTAATACTAACTTGCGGACTAATACAGTTTTT
AGTAACGTCAGGGATGCACGTGTATGGTGTAATAGTAGCTGGATGGTCTTCTAATTCTAAGTATTCTTTGT
TGGGAGCAGTTCGAGGAGTAGCTCAAAGAATTTCATATGAAGTACCAATAACTTTTGTTATCTTAATTGT
TGCTTATTCATTGAGAAGATTATGGATTCAAGAAGTTAAGGGAATCTTTTCAATAGCGATAGGGTTACTTA
GAAGGGGGGTATGAATCACTTGTATGTTGGCTGAGACTAATCGGGCGCCGTTTGATTTTGTGGAAGGGG
AGTCTGAGCTAGTTTCTGGGTTTAATGTAGAGTATAGTAGAGGAGGATTTGCGATAATTTTTATGGCTGA
GTATGGGGCTATATTATTCAATAGTGTTTTCTTTGTGACTCTTTTTTTAGGAGGAGGGGAGTTATTAATAAG
AGTAAGAACAATAATTTGGGTTCTATTTTTTATCTGAATTCGTGGAACCGTTCCACGAATTCGATATGATC
AGTTAATAGGGTTGTGTTGGAAAGTTCTCCTAAGGGTAATGTTGAGTATAAGAGGGTTAATTTTAATTATC
AGGTTTTCCTTATGATAGGAGTTGCGTTAAGTCCTCTATTGATTTTTTGTGGGGTACTAGTAATTATAGGTA
ATCTCTTAAGAATTAGAAGTAGTTCTTGGTTAGGGGTGTGGCTAGGAATAGAAATTAACCTTTTTGGGTT
TTTGATTATAATAAATCCTGAAGGTTGTTGTGTTGCTCAGAGGGGAGTAAAGTATTTTGTTACTCAGAGA
ATTGGGTCTATACTATTATTATTTGGGTTTTTTTTAGTTTTTCTATTTAATAGGGTAGGGGCTGTAGTTATCA
TAATAGGATTGTTAATAAAAACAGGAATCTTTCCATTTCATAACTGGGTTCCAGACGTTGTTATAGTTTCA
AGTTGACTAATTGGTTGTTTAATTTTGACTTGGCAAAAGTTAGCCCCATTTTCCTTTTTTTCATTCTTCCC
CGGTTCTTGGTTTCTTATTACTAGGTTGGTTTTTATGAGCTTAGTAGGCTGTCTTGGTGGATTAAATCAGA
ATTCTGTTCGGGGAATAGCAGTTTATTCTTCATTTGTACATAATTCCTGGATAATACTTTCTTTACTTTACT
CATTTAGGGTATTTTTTTGTTTATTACTTAATTTACAGATTGAGAGTGATGGTATTTTTTTTTAGGTGTTGAA
CTCTTAGAAAAAGTAGAATGATGAGATATTCAATTAGGTGGATAGGGATGCTAAGACTGTTAATATTGTCT
GGGGTACCCCCCTTCATAGGATTTTTTATAAAATTGATTGTGGTATTAAGGTGTCCTATTATATTATTAATCA
TTTGTTTGTTAAGGTCAGTAGTTAGATTGAAGTTTTATATATCGTTTTTTTACAGAATAATTTTAAATAGAA
TTTATTGTGTAAAGTTATTTTTAGCATTTAGTCTAGCTAACTTTATTATAGGAAGAATTTCTATAAGGTACT
TTATTTGGTAGATGATTGAATGTTTAACCCTTGTGTTTCTTATTGCTTTAAGACTAATTTTGATACTAGTAG
CTTTGTTATCTGAAAAATCATTTGAAAGTCGAGAAAAGTCTTCTCCGTATGAATGTGGTTTTGATCCTATT
TTAAGGGCTCGAAGTTCTTTCTCATTACGGTTTTTTCTTCTTGCAGTTTTGTTTGTCGTTTTCGACGTAGA
ATTGTCTTTATTAATACCTGTAGTAACAAGGTTGATAAGAGGTTTTAATTATATTAGTCTGGTTAGATGCTT
TTTATTTTTAGTAATTCTATTTCTAGGCATTTATCATGAGTGACGAGAAGGGTCTTTGAATTGAGTAGTTTA
AGTGGCCGTAAGTTTGATTATTTCTTTGATTATACTCTTTGTTTTAGGTAATAGGACAATTAGGTTAATAGT
TTTATTTTTCTTATTTTTAACTGTTTTAGAGATAGGCACTATCAGGGACGAATTTGTAGCATTAGGCGGGA
GGGTAAGGTACGATTTATTGTCTTATAGGTTAAGTGTTCTAAGAATTTTTATTATATTGCTTAGCCTAATAA
GGAGTAAGAGGGTACAACGAATTAAGTTATTTTATTTTATGAACTTAGCTTTGTTAATAGTCTTAGTTTTA
GCTTTTTGCACATCTAGTTTTTTGGGTTTTTTCTTTTTTTTTGAATCTGCTTTAATTCCATTGATCCTTATTA
TCATGGGCTGGCGGATGGAGGCAAGGACTTATATATTTATTTATACGGTGTTCGGATCATTGTTCTTCTTAT
TTGGAGTGTGTTTGTTGTTTTATAGGGGGAGGGATAACATGTTATTAGGAAGAAACATTTCTAAAAAAGT
GAGAGGGTTTTGGTGGTTGTTTGTTCTGGGGTTTCTAGTTAAATTGCCGGTTTATCCATTTCATTTATGAT
TACCTAAAGCTCATGTCGAGGCGCCTGTAGCTGGTTCAATAGTATTGGCTGGAGTTGTTCTTAAGTTAGG
GGGCTATGGATTACTTCGACTTATAATAGTGATAAGAGTAAATTTAGGGTTAAAGTCTAATATAATGTTGA
GAATTGGGATGGTAGGTGGTGTATATGCTAGATTAGTCTGTTGTCGCCAGACAGACCTAAAATGTCTAGT
CGCTTACTCTTCTGTTGGGCATATAAGGCTGGTAATGCTATGTCTTAGAAATATTAGAATAGGGGTTAAGG
GAGCTCTATTTATTATAGTAGGTCATGGTCTATGCTCCTCTGGGATGTTTAGGCTGGTGAACGTATTTTAC
AATCATTCAGGGTATCGTAATCTTTACATAAACAAGGGTTTCCTGATGAAAAGCCCAGTTTTATGTTTAGT
AGGATTTCTTCTGTGTTCTAGAAATATAGCAGCCCCTCCCAGTCTAAATTTGTTTGGCGAAGTTTTAATGT
TTGTGTGTTCTTACTTGATTTCATTCTGTTTCTTAGTTTTACTAATGGTAATAACAATTATTAGAGCTGTATA
CAGACTTCACTTATACACAACAACTTGTCATGGAAAATCTTCTGAAGCCAAGGTTAGGTTAGAGACTTAT
AGGGCTGTTTTTGTATTATTAGTACATTGAATTCCATTGAATTTCATCTTTTTGTTTATTCCTTAGACTAAA
AAAAATCATTTAATTAGTGTATTTATTGGGATAGAGTTTATAATATTAGGAATTTTATACTGCTGTAGTAGAT

TAATATTTTATAATATTAGAATAATTTTTCTAGTAATGTGTTTAGGGGTTTGTGAGGCAAGGGTGTGCTTAG
CTCTTTTAGTAATAATAGTACGACTTTCAGGGAATGATCTGATCTCGAGACTTTCTTTGTACAAtttaataatttttc
tcaaaaaATGAGGTTGATAGTTATATTAGGCTATGTATTGGTGTTATTAAGGAGGTTCGAAAGTGTTTATTTTT
TTGAATTCATGTTATGGGATTTAAACAGATTAAGGTTCTCTGTAAATTTACTCTTAGATTCATTGAGGATA
ATATTTTTAGGTACTATTCTAATTATTTCAGGTAGGGTTTTAATATACTGTTATTGATACATGGATGATGAAA
AGAACTTTTATCGATTTATATATTTAGTCTATTTATTTGTAGGGTCTATGATGTTTATGATTCTTATTCCTAAT
CTTGTTACCCTTCTTATTGGCTGGGACGGTTTAGGGCTTACTTCTTTTTTGCTTGTTGCACATTATCAAAA
TAATAAATCTTTGTCAGGAAGTTTGTTAACGGCTTTAACTAACCGTATCGGGGACGGTCTGATCTTGGCT
AGAATTTCACTATGAGCGGTTGAGAGGGGTTGGGTATTGTATAATTTTGAAAGTCAGTTTGTGAATTTTT
TTATTTTAGCCTTAATTTTTGGAGGAATAACAAAAAGAGCTCAAATACCTTTTTGTGCTTGGTTGCCTGCT
GCTATAGCTGCTCCTACTCCAGTTTCTTCTCTAGTTCATTCATCTACACTAGTGACTGCTGGGGTATATTTA
TTAATTCGTTCTTATCCAGTAATTAAGGGGAGCTTGATGACTAGTTTAAAGTTTCTTAGTTTGTTTACATT
GGTTTTAGCTAGAAGTGTTGCTATTAACTGTTTTGATATAAAGAAAATCATTGCTTTGTCGACTTTAAGTC
AATTAAGGGTGATAATATTTTCATTGTCTCATGGTCTTGTTTCTATTTCTTTTTTTCATTTGGTAATGCATGC
CCTATTTAAAGCTTTATTATTTTTAACAGCAGGTGCAGTAATTCATTCTCTCAAAGGGTGTCAAGATATTC
GAAGGATAGGGGGATGCTGGAGAATTTTACCAAGTAGAATAGTGTGTATGTTTATTGCTAATTGTTCATTG
TCAGGGCTTCCATTTATAAGAGGGTTTTATTCTAAAGATATAATTCTGGATATATTTAATAGGAGAAATTTT
TGGTTTATAATTATTATACCGGGGCTTATACTTACTAGATTTTATAGCATGCGAGTTTGAATAGTTTGCTTC
GGAAGGAATAAGGTATCAATTAGTACACTTTGTTTAAAAGAGCCAAAAAGGCTACTTATTCCTTATTCGC
TACTAGCAGTCGGTGCTGTGTTTCTAGGAGGAGTTTTAACTCCTAGTGAGTGTCTGTTTAGTTATCGGAG
ATCTTCGGCGAGAGAAATAGGAACTTTAGGGGTTCTTTTTGTTTTTGGAATTGGACTAATTAGAGTTTCT
AGAAAGTTTGGGAAAGATTTCCGGGGCTTTTCGTTCCTATTTAGAATGTGATTTTTAGAGTTGACTCACG
GAGTAAAAAAATGTTTTTTTGATGTAAGGGGGATAATTTTGAAAAGATGTGACAAAGGAATATTAGAGA
CTGTAGGTCCTAAAAGATTCTGTATAAAATTAAGTTCTTATAATGAAGATTATTTTTTGCTATTTTTTTTTTT
TATTGGAGCTCTTTCTTTTCTTTTCTTTTGAAATATGCAACTCCTTTTAATTTACTGAACTGTAATTAGTTT
GTGTGTGGTTTTTATTGGACAGCCTTATCTATTAGGGCTAGTGCTGTTGATGACTTCTATGTTTGTGTGTAT
GATTATTGGGGTAAATGTAAGAACATTTTTAGCTTTACTTTTATTCATGAGATATGTGGGGGGTTTAATGG
TTCTTTTCATTTATGTGTTAAGTGTATTTCCTAATGAAAATTTCGAAGTAATGGGCTTTATGATTGTGAGTG
TGGCAATAATGAGATTTTTAATATTTCCAGTAAAAAGGCAAAAAAAGGGATTATTTtttCACTATATAAGGT
TTGTGGGGGTTTTTGTTTTTATAGCCCTTTTTCTTTTATTTATTATGATGTGTGTATCGTATCTTGTAATAAA
AAAACGAATGCCATTGCGTGTAAAT"/>
<sequence id="seq_Perna_pernaOK576481" spec="Sequence" taxon="Perna_pernaOK576481"
totalcount="4"
value="ATGTTAATAGATGTTTTTTCCATGTTTGATGATTATAATTTTAATACTTTTAAAAGGTGGTATGTGTG
GGGGTATTCTTTATTATTTCCTCTTTTTATTGTTTCATCTAGTGTGTCAATTTATTTTTCGTTTTTACAGAAG
TGTATGAAGTTAATTAACAGGGGAAGAGGGTTATACTTGTCTGGATTTCCACTATTAGTAGGTAGTTTATT
TTCTATTATACTAAGAGTAAATGTATCTAACAGCATTCCTTATTTTTTTAGGGTAAGAGCACATTTTTCATT
TGGGTTTACCTTTGCTTCGGTGGTGTGATTATGTCTGATTACATCTAGAATTTTTACTAGCTTTATTCAAAA
TATAGCTCTTCTAGTTCCGAGTGGACCGGAAGGTTTAGCACCTTTTATAGTGTTGCTAGAAATTATCACTA
ATTTGCTTCGTCCTATTACGTTGATCATTCGGTTGGCTATAAATATAGCTACTGGTAAAGTAATTATACTCT
TACTTGGCAATGCGGCTTTAAATCTAGCTTTTGCGAGATCTTGTGGGTTTGTGGTGGTTAGAGTGTTGGG
GGCAATTTTTTCTCTGGAAGTGGCGGTAAGGTTTATTCAAAGATACATTTTTTGTACTCTTTTATGTCTTT
ACGCTGACGAGCATAGAAAATAACGATGGTTTATGTCAACCAATCATAAAGATATTGGTACTTTGTATTTG
CTCTCAGGAATGTGGGCAGGCCTTATAGGCAGGAGGTTGAGATTAATCATTATTCAATCTCACCCAGGGG
GAAGGTTTTTGAAAGAAAGATTATATAACGTGGTGGTGACTACCCACGCTCTTGTTATAATCTTTTTTGCT
GTTATACCGTTGCTTATTGGGGCTTTTGGAAATTGATTATTGCCTTTGTGTATTGGAGGGTGTGATTTAATT
TTTCCTCGATTAAATAATTTGAGGTTCTGGTTAGCTCCGAATGCTCTTTATTTATTGATCTTATCTTTTATAA
CAGAAAAAGGGGCTGGTACAGGTTGAACTATTTACCCTCCTCTATCATCTGGTCTATATCATACTGGGCCT
GCAGTTGATATTTTAATTACCTCTCTTCACTTGATTGGATTAAGCTCTTTATTAGGATCTATTAATTTTGTCA
GGACTAATAAGAATATGCCAACAGTTAAGATAAAAGGGGAAAAATCTGAGTTATATCTGTGAAGAATTAC
TGTCACAGGTGTATTGTTAATTATTTCTGTACCTGTTCTTGCTGGAGGGATTACAATACTTTTATTTGATCG
AAATTTCAACACTAGATTCTTTGATCCTATTGGAGGGGGAGATCCTGTTCTATTTCAGCATGTTTTCTGAT
TTTTTGGTCACCCTGAAGTTTATATTTTAATTCTTCCTGCCTTTGGAATTATGTCTAAAGTAATTATACATTA
TTCTGGAAAAGATTCTGTATTTGGTTCAGTGGGAATATTATACGCTATAGTGGGAATCGGAGTAATAGGAT
GTGTTGTTTGGGCGCATCACATGTTTACGGTAGGATTGAATGTGGATACTCGAACTTATTTTACTTCTGCA
ACTATGGTAATTGCAGTTCCTACAGGGGTGAAAGTTTTTAGTTGGATAGCAACAATAGGTGGAAGTAAA
ATTAAATTCACTACATCTGTCCTGTGGAGCACAGGGTTCTTATTTCTGTTTACGGTAGGGGGTCTGACAG
GGATTATGTTATCAAGCTCTTCTTTGGATGTTACTCTTCATGATACGTATTATGTGACAGCTCATTTTCATT
ATGTATTATCTATAGGAGCTGTGTTTGGTATTTTCTGTGGTTTAAATCATTGGTTTCCTCTATTTTATGGGGT
AAATTTTCACAAAAAATGATCTAAGGTTCACTTTTATCTAATATTTCTAGGGGTAAATTTAACTTTTTTCCC
TCAACATTTTTTAGGATTAAAAGGAATGCCTCGTCGCTATTGTGATTATCCAGATTGTTATTCGATATGGC
ATTGAGTGTCTTCATATGGAGCACTGATTTCTTTTGGTTCGTTACTATATTTCATTTTTATTGTTTGGGAAG
CTATAGTAAGCCAACGTGGGGTGTCATTATTTGAGGCCTTATTTTTTGGTGATGTATTACAATCAGAGTTT

GGTTCTGAGATTGTAAAGTATTATGGTTATGTAATAATAGTGCTGTTACTCGTAATTATTGTTGTCTTATATA
TAGGGACTATAATTATAAATCATAGGTATTCCTATCGAAATTTCAAAAATCGGCAGATATTAGAGTATGTGT
GGACTGCTATACCTACTTTTTTGTTAGCGATGCTTTGATGCCCATCTATTTAAATCTTTATCGAATAGAGG
ATTTAAAAGATCCTGTTTGAAGGTTTAAAGCTGTGGGTAAACAATGATATTGGACTTATGAAGTAAATTT
AAAAGACGGAGAAACTATAGTGATTGATTCTTACATAGATCGGGATTCAGGCGTTGAGTTAGAGTCTGG
TTATCGTCTCTTAGATGTCGATTGGCGTTTGGTAGCTCCTGCTGATGCACAAATTTCTTGTTATGTGACAA
GAAGTGATGTCATTCATTCTTTTGCCCTTCCTGGCGCATTGCTAAAAGCCGATGCTATTCCTGGCCGAATT
AATGTTCTCCCTATAAAGATTAGCCAAAGTTGTATTTTGTATGGGCAATGCTCAGAAATTTGCGGGATCA
ATCATAGGTTTATGCCTATTGTTATTGAGTTTGTTCCGGTAGATGTTTTTATAAAATTTTATGGGTACAATTA
AATGCATCGAAATCCTTATTATGTGCCTGGGCCTAGACCTTGGCCTTTAGTTGTGGGCGTATGCTGTAATA
GTTTATGCGTAAGCTTAGTATTATGAATACACCGAATACAAGAAAAACTGTTTTTGACATTTTTGGTTTTA
GGTTTAACTGCTACTTTATGGTGAACTGATGTGCTTCGTGAGGGGGATATGGGGGTTCAAACTCAGTATG
TTTTAAAAAGATACCGAGATGGTATAGGTCTTTTTATTTTTTCCGAAATTATATTTTTCTTTTCCTTTTTTTG
GGCTCTTTTTCATAACTGTCTAAGCCCTTCTGGCAACATTGGGTGTGAGTGGCCTCCTCTAGGAATTCGA
ACCCTTGATCCAATATCAACGGCGTTATTTAATACTTTTTTACTAATTAGAAGCGGTTCATTTTGTACTTAT
GTCCATAATACAATTCGTAAGTGTTATGATAGATGGTGTCTAATTAATATATTACTAACAATCACGTGCGGA
GTTCTTTTTTTACTAGGACAAGGAAATGAATATTTTTTCTGTTCTTTTAGGATCGCTGATAGTGTGTATGG
TAGTACTTTTTATATATTAACAGGGTTCCATGGAGGGCATGTCATAGTGGGAACAACTTGATTAATCGTTA
GATTTTTCCGAATATGACGTGGGCACTTTAGTAAGGATCGTCATTTTGGATTGGAAGCGTGTCTATGATAT
TGGCATTTTGTTGATGTAGTTTGGCTTTTTGTCTGGTTAATTGCATATTTCTGATTAGGAGGACCTTTTCGT
AAGCGTCATTGATTTTTAAAGATTCTAAATAACAGGCTTTATGACCTCCCTTGTCCTGTAAATTTGAATGC
TTTTTGGAGATTTGGATCCATATTAGGTTTGTGCATTGTAATTCAAATGGTTAGGGGAATTTTGTTATCTCT
ACACTTTATTCCGCATGAGAGGATAGCATTCGATTCAGTGTACCATATTATACGGGATGTGAACAAGGGA
TGGTTTCTTCGTAACATGCATGTTGGTGGATGTTCAATATTTTTTATTTGTCTTTACGTTCACATTGGTCGT
GGTATTTACTATGGGTCTTATATAAGACGACATGTGTGGTTTGTGGGAGTAACTCTGTTTTTAATAGTAAT
AGGGGAAGCTTTTCTAGGGTATAGGTTACCTTGGGGTCAAATATCTTTCTGGGGAGTAACAGTAATTTCT
AATTTGTTTACGGTAATTCCTTATGTGTCGCAAAACTTATTATTCACCCTTTGGGGGCATTGAAGAGTGAG
GGGGTATACTTTACAACGTTTCTTCATTTTTCATTTTTTACTTCCATTTGTAATTATTGTGTTTTCTACTCTA
CACATATTTTTTCTTCACGAAAACGGAAGAAATAACCCTTTAGGAGTTAATAGGGATTCTATATGTATTCC
TTTTCATCCTTTTTATACAGTAAAAGACATTTTTGGTTTTGTTTGTTTTGGGTGAGTATTAATATATCTGGT
GTGTGTTAATCCTGAGTTAGTAGTTAATAAAATTAACTATCATCCGGCTGACCCATATCACACTCCTCTTC
AGGTAGAGCCAGAGTGGTATTTTCTATTTGCCTATGCAATGCTTCGATCTATTCCTCATAAGTTAGGGGGA
GTTTTAGCTCTGTTAGCGTCTGTGTTAGGTTTATATTTATATCCTTTCATTCACACAGGAAAATTCCGGAG
CTTAGCATTTTACCCAGTTAGTCAAATACTTTTTTGATGTTTTGTAGTTAATTTCTTGAGACTTATTTGAGT
TGGGCAAATACCCGTTCGTGAGCCTTATATCACAATAGGACAAGTTTATACAGGAGTGTATTTTTCTACGT
TAATTCTTCCTCCATTATTAACAGGATTGTGAGATAAGTTAATTTTCCTGTATTTTGTTAGAATTATTCTTCC
TTTCGTTTGCGTATTGGTAAGAGTAGCTTTTTACACTCTGCTAGAGCGAAAAGTGTTAGGTTATATTATGA
TCCGAAAAGGGCCTAATAAGGTTGGATACGCTGGAATTATACAGCCTTTTAGGGATGCTGGAAACTTTT
TAACAAAGAATACGTAAAGCCTGGCTATGCCAATTTATTGCCTTTTCTTTTGTGTCCGGCTATTATTTTGG
TAACGAGTCTGCTTTTGTGGTTATTATACCCTTATGATTTACCTCTGTAATATTAACTTGTGGTTTGATGC
AGTTTTTAGTTACCTCTGGCATGCATGTGTATGGGGTAATAGTGGCTGGTTGATCTTCTAATTCTAAATATT
CCTTGTTAGGGGCAGTGCGAGGGGTAGCTCAGAGAATTTCATATGAAGTACCGATGACTTTTGTTATTTT
GATTGTAGCGTATTCCTTAAGAAGATTGTGGATTCAAGAAGTTAAGAATATTTTTTCTATAGCTATAGGGC
TATTAAGAAGTGGGGTGTGGTTAACGTGTATGTTAGCTGAGACTAATCCTGCTCCATTCGATTTTGTAGA
AGGGGAGTCTGAGTTGGTTTCTGGTTTTAATGTAGAGTACAGAAGGGGAGGATTCGCAATAATTTTTATG
GCTGAATATGGTGCTATATTATTCCATAGAGTATTTTTTGTAACTCTTTTTTTGGGAGGGGGTGAGTTACTA
GTGAGAGTGAGGACTATAATTTGGGTGCTATTTTTTATCTGAATTCGTGGAACTGTCCCACGAATTCGGT
ATGACCAGTTGATGGGGTTGTGTTGAAAAGTGTTGCTTAGAGTAATACTAAGGATAAGTGCATTAATTTT
AGTTGTAAGATTTTTCTTATGATAGGAGTTGTATTAAGTCCTCTATTGATTTTTTGTGGAATATTAGTAATTT
TAGGGAACTTTCTCAGAATCAGGAGTAGCTCATGATTAGGGGTATGGTTAGGAATAGAGATTAATCTTTT
TGGGTTTTTAATCATGATAAATCCTGAAGGTTGTTGTGTTGCTCAAAGAGGGGTAAAGTACTTTGTAACT
CAAAGAATTGGGTCTATACTTCTTCTTTTCGGGTTTTTTTTGGTGTTTTTATTTAATAGGGTGGGCAGCGT
AATTATTACAACAGGTCTGCTAATGAAAGCCGGCATTTTTCCTTTCCATAATTGGGTTCCAGATGTCGTAA
TAGTTTCAAGGTGGCCTATTGGGTGTCTTATTTAACTTGACAAAAGCTAGCTCCATTTCCTTTTTTTCA
TTTTTCCCAGGTTCATGGCTTTTAATTATTAGGTTAGTTTTTATAAGGATAGTAGGCAGTTTTGGAGGCTTA
AACCAGAATTCTGTTCGGGGGATAGCGGTTTATTCTTCTTTTGTTCATAATTCTTGAATAATACTTTCTCTC
TTTTACTCGTTTAGTATTTTTTTTATTTATTATTTAATTTATAGAATAAGGGTTTTTCTTTTTTTCTTTAGGTG
TTGAACCTTAAGAAAGAGGAGGATGATAAGGTATTCAATCAGCTGAATAGGAATATTAAGACTACTAATG
CTTTCAGGTGTTCCTCCTTTTATAGGATTTTTTATAAAACTTATTGTGGTTTTGAGATGTCCCGTTATGTTA
ATAACTGTTTGTCTTTTAAGGTCAGTGATTAGGTTAAAATTTTATATGTCATTTTTTTATAGAATAATTTTGA
ATACTATGTATTGTGTAAAATTGTTAATGACATTTAGGTTAATGAACTTTATCGTGGGAGGAATTTCTATAA
GGTACTTTATTTGATAGATGATTGAATGTGTAACTCTTGTGTTTCTTATTGGACTGAGTTTGATTTTGATAG
TAATTGTTTTGTTATCTGAAAAGTCTTCTGAAAGCCGTGAAAAGTCATCTCCTTATGAGTGTGGGTTTGA

TCCTATTTTAAGAGCCCGAAGGTCTTTTTCTCTACGATTTTTTCTTCTCGCAGTATTGTTTGTTGTATTTGA
CGTGGAGTTATCTTTATTAATGCCTGTAGTAACAAGGTTAATAAGCGGGTTTAATTATATTAGGTTCATTAG
TTGTTTTTTATTTTTAACTATCTTGTTTTTGGGCATTTTTCATGAGTGGCGGGAAGGGTCTTTAAATTGAG
TGGTTTAAGTGGCCGTAAGTTTAACTATTACTTTGATTATACTCTTTGTCTTAGGTAATAAGGCAATAAGG
TTGATAGTCCTTCTTTTCTTATTTTTAACTGTCTTAGAACTAGGGATGGTTAACGATGAATTTTTAGCTTTA
GGCGGGAGGATAAGATATGACTTTTTATCTTATAGCTTAAGTGCTTTAAGAGTATTTATTGTATTACTAAGG
CTAATGAGTAGTAAAAATGTTCAACGAGTTAAATTATTCTACTTTATGAATTTGGTACTACTAATGGTTTT
GGTCTTAGCTTTTTGCACCTCCAGTTTTTTAGGTTTTTTCTTTTTTTTTGAGTCCGCTTTAATTCCTCTTAT
TCTTATTATTATAGGCTGACGGATAGAGGCAAGGACTTATATATTTATTTACACGGTGTTTGGATCTCTGTT
TTTTTTATTCGGGGTGTCCCTGTTATTATATAGGGGAAGTGATAATATGTTATTGGCGAGTAATGTAAATAA
GAAGATCAGAGGATTTTGATGGTTATTCGTACTAGGATTTTTAATTAAATTGCCTGTTTACCCTTTTCATCT
ATGGTTACCAAAGGCTCACGTGGAGGCTCCCGTGGCAGGTTCAATGGTCTTAGCGGGCGTTGTGTTAAA
ACTGGGGGGTTATGGATTATTACGGTTAATAACCGTGATAAGAGTGAATCTAGGATTAAAGTCCAATGTA
ATATTAAGAGTAGGGATAGTAGGAGGAGTATATGCAAGGTTGGTTTGTTGTCGGCAAACGGACTTAAAAT
GTTTGGTTGCCTACTCCTCCGTAGGGCATATAAGGTTAGTAATGTTATGCTTGAGAAATTTAAGAATAGGA
GTTAAAGGAGCTATTTTCATTATAGTGGGGCATGGGTTGTGTTCCTCTGGAATGTTTAGTTTGGTAAACGT
TTTTTATAGTCATTCAGGATTCCGAAATTTGTACATAAACAAAGGTTTTTTAATTAAAAGTCCAATTTTATG
TCTTGTAAGGTTTCTTTTATGTTCTAGAAATATAGCAGCTCCGCCTAGTTTAAATTTATTTGGAGAAGTTTT
AATATTCGTATGCTCGTACCTTATTTCATTTTGCTTTTTAGTACTACTAATGGTAATAACAATCATTAGCGCT
ATTTATAGGCTTCATTTGTATACGAGAACTTGTCACGGGAAGGTTTCAGAGGCTCAAACTAGATTGGAGA
CTTATAGGTCTATTTTTGTTCTTTTAGCACATTGGATCCCGTTAAATTTCCTGTTTTTATTTATACCTTAGAC
GAAAAAAAATCATTTAATCAGGGTTTTTATTGGGATGGAGTTTATAATGTTGAGAATTCTATATTGTTGTA
GTACATTGGTGTTTTATAACGTTAGAATAATTTTTTTAGTAATGTGTTTAGGGGTCTGTGAAGCAAGTGTT
TGTCTAGCTCTTTTAGTAATGATGGTGCGGCTTTCAGGGAATGATTTAATTTCTAGCCTTTCTCTGTATAAtt
taataatttttctcaaaaaATGCTAGTAATAGCGGTATTGGGGTATTTATTTGTGCTAATAGGGAGTTTTGAGAGTGTT
TATTTTCTAGAGTTCATGTTGTGAGACTCAAATAGATTAAGCTTTTCTGTAAATGTACTTTTAGATTCTTTA
AGTTTAATATTTCTGGGGACTATTTTAATCATTTCGGGGAGGGTATTGATATATTGTTATTGGTATATAGATG
ATGAAATCTATTTTTTTCGTTTCATATATCTTGTTTACTTATTTGTAGGGTCAATAATATTTATAATTCTTATT
CCTAATCTTGTTACTCTTTTGATTGGTTGAGATGGACTGGGGTTAACTTCTTTCCTACTTGTTGCACACTA
TCAGAATAATAAATCTCTATCTGGAAGACTATTAACAGCTTTAACTAATCGAGTTGGAGATGGTTTAATTT
TGTCTAGAATTTCATTGTGGGCAAAGGAAAGAAGCTGGGTAATTTATAATTTTGAGAGTCAATTTGTAAA
CTTTTTTATTTTGAGTTTGGTTTTGGGGGGTATAACTAAAAGAGCACAAATGCCTTTTTGTGCTTGGTTAC
CAGCAGCTATAGCTGCTCCTACTCCAGTATCTTCTTTAGTTCATTCTTCTACACTAGTGACAGCTGGGGTA
TATTTGCTGATCCGTTCTTATCCTGTAATTAAAAAAAGCTTAATAACTAGTTTAAAGTTCTTGAGGCTGTT
TACATTGGTTTTAGCTAGCAGAACAGCAATTTACTGTTTTGACATAAAAAAAATTATTGCTTTGTCAACTT
TAAGTCAACTAAGGGTAATAATATTCTCACTGTCTCATGACTTAGTATCGGTTTCTTTTTTTCATTTAGTAA
TACATGCTTTGTTTAAAGCTCTTTTATTTTTGACAGCTGGTGCAGTTATTCATTCTTTTAAAGGGTGCCAG
GACATTCGAAGAATGGGAGGGTGTTGGAGAGTTCTCCCCTGTAGGATAGTGTGTATATTTATTGCTAATT
GTTCTTTGTCAGGTTTACCATTTTTAAGAGGGTTTTACTCAAAAGATATAATTTTGGATATGTTCAATAGA
AGAAATTTTTGGTTTATGATCATCATACCTGGTCTTATACTCACGAGGTTTTATAGAATGCGGGTTTGAAT
GGTTTGTTTTGGAAGAAATAAAGTTCCAATTAGAACTCTTTGTGTTCAGGAGTCAGTGAAATTACTCATA
CCTTATTTGCTTCTTGCTTTTGGCGCTGTATTTCTAGGAGGAATTTTAACCCCAAATGAGTGTATATTTAGT
TATCGAAGCTCATCAGCAGGAGAAATAAGGTTATTGAGAGTTTTGTTTGGGTTTGGGGTTCTGTTTGCTA
GAATTTTAAGAAATTATGGTAAAAAATACAAGACCTTTTCATTTTTGTTTAGAATATGGTTCTTAGAGTTA
ACTCATGGGGTGAAAAAGGGCTTTTTCAAAATAAGAGAAAATATTTACAGAGATGTGATAAAGGGATA
CTAGAAACTGTTGGGCCTAAGGAATTCTGTATGAAATTAAGGTCTTATAATGAAGATTATTTTTTACTTTC
TTTTTTCTTTATTGGACTTCTTTCTGTGTTCTTTTTTTGTAATATGCAACTTCTTATGATTTATTGGGCCGTC
ATCATAATATGTGTGATTTTTATTAGCCAGCCTTATTTATTAGGTTTAGTACTAATGCTAACTTCTATATTTAT
TTGTATAATTGTTGGAGTAAACGTTAGGAGATTTTTAGGGTTTCTTCTGTTTATAAGATATGTAGGGGGTT
TGCTGGTTCTTTTTGTTTACGTATTAAGGGTTTTTCCAAATGAAGATTTTAAGATAAGAAGGTTTTTACTG
GTAAGAAGAGGTCTAATATCTTTTCTTTTATTTCCAATTCATAGATTAAGAAAATCTCTTTTTtttCATTATAT
AGGGTTTATTGGAGTTTTTGTGTTTATGGCTCTTTTTCTTTTGTTTATCATAATATGTGTGTCTTATCTTGTG
ATAAAAAAACGAATACCGTTGCGTGTGAAT"/>
<sequence id="seq_Perna_viridisJQ970425" spec="Sequence" taxon="Perna_viridisJQ970425"
totalcount="4"
value="ATGTTAATAGATGTTTTTTCTATGTTTGATGATTATAATTTAATACATTTAAAAGGTGGTATGTCTG
GGGATATTCATTGTTTATTCCTTTGTTTATCGTGAGATCATCATTAAGGATTTATTATTCATTTTTACAGAAA
TGTTTGAAATTGATTGGTAGGGGAAGGGGTTTGTATATTTCAGGTTTTCCTCTATTAGTTGTGAGTCTTTT
TTCAATTATACTAAGGGTAAATGTTTCTAATAGAATTCCTTATTTTTTTAGGGTGAGGGCACATTTTTCGTT
TGGCTTTACTTTTGCGTCTGTGATTTGGCTGTGTATTATTACTTCTAGGGTTTTTACAAGGTTTATTCAGAA
TATGGCTTTGCTGGTTCCAAGAGGTCCTGAGGGGTTAGCTCCATTTATGGTGCTTTTAGAGATTATTACAA
ATTTATTGCGTCCAATTACTTTAGTTATTCGATTAGCTATAAATATGGCTACTGGTAAGGTTATTATGTTATT
ATTAGGGAATGCTGCGTTAAATCTTGCATTTGTTGGGGTTGTAGGGTTTATAGTTGTTAGAGTGTTAGGTG

CTATTTTTTCTTTAGAGGTGGCTGTTAGGTTTATTCAGAGATATATTTTTTGTACTCTATTATGTTTGTATGC
TGATGAACATAGGAAATAGcgatggtttATGTCTACTAATCATAAAGATATTGGTACTTTGTATTTACTTTCTGG
GATATGAGCTGGGTTAATGGGGAGAAGGCTTAGGTTAATTATTATTCAGTCTCATCCCGGGGGTAATTTTT
TGAAAGAAAGGTTATATAATGTTGTAGTAACAACTCATGCATTAGTAATAATTTTTTTTGCTGTAATGCCTT
TACTTATTGGTGCTTTTGGGAATTGATTACTTCCATTATGTATTGGTGGTGTTGATTTAATTTTTCCTCGTTT
AAATAATTTGAGATTTTGGTTGGCACCTAATGCTTTGTACTTACTTATTTTGTCGTTTATAACGGAGAAAG
GAGCTGGGACAGGTTGAACTATTTATCCACCTTTATCTTCTGGGTTGTACCATACTGGGCCTGCTGTTGAT
ATTTTGATTACGTCTTTACATTTAATTGGATTGAGTTCTTTATTAGGTTCGATTAATTTTGTGAGGACTAAT
AAGAATATGCCTACAATAAAAATAAAGGGTGAGAAATCTGAGTTGTATTTGTGGAGGATTACCGTAACCG
GTGTTCTTTTAATTATTTCTGTGCCAGTTCTGGCTGGTGGGATTACTATATTGTTGTTTGATCGAAATTTCA
ATACTAGGTTTTTTGATCCTATTGGAGGGGGAGATCCTGTTTTATTTCAGCATGTATTTTGGTTTTTTGGTC
ATCCTGAGGTGTACATTCTTATTCTTCCGGCGTTTGGTGTGATGTCAAAAGTAATTATGCATTATTCTGGT
AAAGATTCTGTTTTTGGTTCGGTTGGGATATTATATGCTATAGTTGGTATTGGTGTTATAGGTTGTGTGGTG
TGGGCCCATCATATATTTACTGTGGGTTTAAATGTAGACACACGAACTTATTTTACATCAGCTACAATAGT
AATCGCTGTTCCTACGGGTGTGAAAGTTTTTAGTTGGATGGCTACAATAGGAGGAAGAAAAATTAAGTT
AACTACATCTGTTTTGTGAAGGACTGGTTTTTTATTTTTGTTTACTGTTGGGGGACTTACGGGAATTATAC
TTTCTAGTTCTTCTTTGGATGTAACATTACATGATACATATTATGTAACAGCACATTTCCATTATGTCTTGTC
GATAGGGGCTGTATTCGGTATTTTTTGTGGGTTGAACCATTGATTTCCTTTGTTTTATGGTGTAAACTTTC
ATAAAAAGTGATCTAAAGTTCATTTTTATTTAATATTTTTAGGAGTGAATTTAACATTCTTTCCCCAGCATT
TCTTAGGATTAAAAGGGATACCGCGTCGTTATTGTGATTATCCAGATTGTTATTCTACATGGCATTGGGTAT
CTTCTTATGGTGCTTTAATTTCTTTTGGGTCTTTGTTATATTTTATTTTTGTTGTATGGGAGGCTATGGTGAG
TCAACGAGGTGTGTCATTGTTTGAGGCTATATTTTTTGGTGATGTATTAGAGGAGTTTGGTTCTGAAATTA
TTAAATATTATGGTTATGTGATAATAGTTTTATTGTTGGTTATTATTGTTGTTTTGTATATGGGAATAATGATT
ATGAATCATAGATATTCTTATCGTAATTTTAAAAATCGACAAATATTGGAGTATGCTTGAACTGCAATACC
AACGTTTCTTTTGGCGTTATTATGATGTCCTTCAATTTTGAATTTGTATCGGATAGATGATTTGAAGGATCC
TGTCTGAAGGTTTAAGGCTGTTGGAAAGCAGTGGTATTGAACTTATGAGAATGGGGATACGATGGTTATT
GATTCTTATATAGATCGTGATTCTGGGGTGGGTTATCGATTACTTGATGTTGATTGGCGTTTAGTGGCTCCT
GCTAATGCGCAGATTTCGTGTTATGTTACAAGGAGGGATGTAATTCATTCTTTTGCGCTTCCGGGTGCGTT
GATAAAAGCGGATGCTATTCCTGGACGGATTAATGTACTTCCTATAAAAATTAGTCAGAGTTGTATTTTAT
ATGGGCAGTGTTCAGAAATTTGTGGGATCAATCATAGATTTATACCTATTGTTATTGAGTTTGTTCCCGTT
GATGTGTTTATGGATTTTTATGGGTATTAGaattaa-----------------------
ATGCATCGAAGTCCTTATTATGTCCCAGGGCCAAGGCCTTGGCCATTAATTGTAGGTGTGTGTTGTAATAG
GTTATGTGTAAGTCTTGTGTTGTGGATACATCGAATACAAGAGAAACTTTTTGTTACATTATTGGTGTTAA
TTATTACTGCGATTCTGTGATGAACAGATGTTTTGCGTGAAGGGGATATGGGTGTGCAGACTCAGTTTGT
AATTAAGAGATACCGAGATGGAGTTGGCCTTTTTATCTTTTCTGAAATTATATTCTTTTTTTCGTTTTTTTG
GGCACTTTTTCATAGTTGTCTAAGTCCTTCTGGAAATCTTGGGTGCGAGTGACCTCCTTTAGGGATTCGG
ACGTTAGATCCGTTATCTACTGCCCTATTCAACACTTTTTTGTTAATTAGAAGCGGGTCATTTTGTACTTAT
GTTCACAACTCAATTCGAACAGGTTATGATTCTTGATGTCTGATCAATATGGTAATTACTATCTCTTGTGG
TGTAATATTTTTAATTGGGCAAGGTCATGAGTATTTTTTTAGTCCTTTTAGAATTGCGGATAGAGTGTACG
GAAGAACTTTTTATATGCTAACTGGGTTCCATGGTGCTCATGTTATGGTAGGAACTACTTGGTTAATTGTT
AGGTTGAGTCGGATGTGATTAGGCCATTTTTCTAAAGACCGTCATTTTGGATTGGAAGCTTGTTTATGATA
TTGACACTTTGTTGATGTGGTGTGGCTAGGGGTATGACTAATTGCTTATTTTTGGATGGGGGGACCTCTT
CGGAAACGTCATTGATTATTGAAAATTTTGAATAATAGGTTATATGATTTACCATGTCCAATTAATTTAAAT
ATATTTTGGAGATTTGGTTCTATGTTAGGTTTGTGTATTGTTATTCAGGTAGTTAGTGGAATTTTATTATCTC
TTCATTTTATCCCTCATGAGGCAATAGCTTTTGATTCTGTCTATCATATTATGCGTGATGTGAATAAGGGTT
GGTTTTTGCGGAATGTTCATGTTGGTGGGTGTTCTATGTTCTTCGTTTGTTTGTATGTTCATATTGGTCGTG
GGATTTATTATGGGTCTTATTTAAGTAAGCACGTTTGGTTAGTGGGTGTGACTCTGTTTTTGTTGGTGATG
GCAGAAGCATTTTTAGGGTATAGATTGCCTTGGGGTCAGATATCGTTTTGAGGTGTAACTGTGATTTCTA
ATTTGTTTACTGTGATCCCGTATATTTCTCAAAATTTGTTGTTTACTATTTGGGGGCATTGAAGCGTTAGG
GGGTATACTTTACAGCGATTTTTTATTTTTCACTTTCTTCTTCCTTTTGTCATTATTGTTTTTTCGACTTTGC
ATTTATTTTTTCTTCATGAGAATGGGAGAAATAATCCTTTGGGTATTAGAAGGGATTCAATATGTATTCCTT
TTCATCCATTTTACACTGTCAAAGATATTTTTGGGTTTGTTTGTTTTGGTTGAGCTTTGATATATTTTGTTT
GTGTAAATCCTGAACTGGTAGTAAATAAAATCAATTATCATCCAGCAGACCCTTATCACACTCCTTTACAG
GTAGAGCCGGAGTGATATTTTTTGTTTGCTTATGCTATGTTACGTTCAATTCCTCATAAGCTTAGTGGGGT
GCTTGCCTTACTTGCTTCAGTAACTGGATTGTATCTATATCCTTTTATTCATACTGGGAAGTTTCGTAGGTT
TGCATTTTATCCCGTAAGACAGATGCTGTTTTGGTGTTTTGTTGTAAATTTTTTGAGGTTAATTTGGGTTG
GGCAAATACCTGTGCGAGAGCCTTTTATTAGAATAGGTCAGGTATATACTGGTATTTATTTTTCGACTTTG
ATTTTTCCTCCTATGTTAACTGGTCTTTGGGATAAGTTGATTTTCCTTTATTTTGTAAGGTTGATTCTTCCG
TTTGTTTGTGTTTTGGTGAGGGTTGCTTTTTATACTCTTGTTGAACGAAAGGTGTTAGGTTATATTATGATT
CGTAAGGGGCCTAATAAGGTTGGGTATAGGGGAATTATGCAGCCTTTTAGGGATGCAGGGAAGCTTTTTA
GAAAAGAGTATGTGATACCGGGATTTGCTAATGTTGTTCCTTTCGTTTTGTGTCCTGCTGTTATTCTTTTT
ACTAGAATGATGTTATGGTTTCTTTATCCTTATAGTTATGTTTCGATAGTGTTTACGTGTGGGATTGTTCAG
TTTTTAGTAACATCTGGTATCCATGTATATGGAGTTATAGTAGCTGGTTGGTCTTCTAATTCAAAGTACTCT

CTTTTAGGGGCTGTTCGGGGGGTAGCACAAAGAATTTCTTATGAAGTTCCTATAACTTTTGTGGTTTTGA
TAGTAGCGTTTTGTATTGGGAGATTATGGTTGCAGGAGGTTAAGATAATATTTTCTATTCTTATAAGATTAT
TGAGGAGAGGGGTTTGAGTAACTTGTATGTTGGCTGAGACCAATCGCGCTCCGTTTGATTTTGTTGAGG
GGGAGTCTGAGTTAGTATCAGGGTTTAATGTGGAGTATAGAAGAGGAGGTTTTGCAATAATTTTTATGGC
GGAGTATGGGGCTATATTATTTAATAGGATTTTTTTTATTACTATTTTTTTAGGCGGTAATGAGTTATTAATG
AGGGTTAGAACAATAGTATGAGTTCTTTTTTTTGTGTGAATTCGGGGAACAGTTCCCCGAATTCGATATG
ATCAGCTTATAGGATTGTGTTGGAAAGTTTTATTAAGGGTAGTTTTGAGAATAACTGGATTTGTAGCTATT
ATCAGATTTTTTTTatgataggaATAAGAGTTAGACCTTTGTTAAGAGCTTGTGGGTTATTAGTTGTATTAGGTA
ATTTAGTAAGGATTATAAGAAATAGATGAATGGGTGTGTGGTTAGGAATAGAACTTAATTTATTTGGCTTT
TTAATTATAATAAATCCTGAAGGGTGTTGTGTTGCTCAGGGAAGAATCAAGTACTTTATTGCACAAAGGA
TTGGTTCTATGTTAATATTGTTTGGGTTTTTTTGTGTGTCTATGTTTAATAGAGTGGGGAGGTTAATTCTTG
TTGGGGGAATTCTTTTAAAAGCGGGGGTTTTTCCTTTTCATAGATGAGTTCCGGACGTTGTTATTGTGTCT
AATTGGTTTATTGGGTGTATAATTTTAACATGGCAAAAGTTAGCTCCATTTTCTTTGTTTTCGTTTTTTCCT
AGTTCTTGGGTCTTGGTAATAAGATTGATTTTTATAAGGTTGGTGGGATGCTTTGGGGGATTAAATCAAC
ATTCAGTTCGTGGAATGGCAGTTTATTCTTCTTTTGTTCATAACTCTTGGCTGATTCTTTCTTTATTTTATTC
CTTTGGTGTTTTTTTTATTTATTATTTGGTGTATAGGTTGAGGGTTTTAATATTTTTTTTGAGGTGTTGGGTA
GTTAGAAAAAGGAGCTTAATAAGGTACTCCGTTAGATGATTGGGAATATTAAGTCTATTAATGTTATCTGG
TGTTCCACCTTTTATGGGGTTTTTTGTTAAATTGATTGTGATGTTAAGATGCCCGTCTATTCTTTTGATTAT
TTGTTTGGGGGGATCTATCGTAAGGCTAAAGTTTTATATTTCTTTTTTCTATAGAATGATTTTGAATTCTTAT
TTTCAATTGGAGTTGGTTTATATTTTTTGTAGGATGAATTTTGTAGTAGGGGTTAGTTCGTTATTGTTTTTT
ATATGGTGTATGATTGAATGTTTTCTTTTGTTTTTTCTTTTTATTTTAAGGATGGTGTTAGTACTAGTATTTT
TATTATCAGAGAAAACATTTGAGAGACGTGAGAAGTCGTCCCCTTATGAGTGTGGGTTTGACCCTATTTT
AAGGGCGCGAAGGTCTTTTTCTCTTCGTTTTTTTCTTTTAGGGGTCTTATTTGTTGTTTTTGATGTTGAGT
TGTCTTTGTTAATACCTGTGGTTCTTAGGATTAGGGTGGGGTTTAGCTATGTGGGGCTTATTAGATGTTTT
ATCTTTCTTTTAGTTTTGTTTTTAGGAATTTTTCATGAGTGACGAGAAGGGTCATTGAACTGGGTTATTTA
GGTGGCAGTAAGTATAATATTAGTATGAATCTTTCTTTGTATTGTTAGAAATAAAATGTTGAGGCTTTTAA
GTTTGGCTTTGTTAATAGTAGGGGTTATAGAGATAGGAGTAATCAATGATGAGGTAATTAGAATGGGGGG
TAGTCTTAGATATGATTTTTTATCCTATAGGTTAAGGGTTTTGAGGATTTTTATTATATTGTTAAGCTTAGTT
AGAAGAAAAAGGGTAATACGAACTTCTTTGTTTTATTTTTTAAATTTAAGATTATTAATAGTGTTAGTTTTT
GCTTTTTGTACTTCTAGTTTTCTAGGGTTTTTCTTTTTTTTTGAGTCTGCTTTGATTCCTTTAATTTTGGTTA
TTATAGGGTGACGGATAGAGGCAAGAACTTATATGTTTATCTATACGGTATTTGGTTCTCTTTTTTTTCTTGT
TTGGGATTTGTCTTCTTTTTTATAGAGGAAGGGATAATATATTATTAGGAAGGAATGTGAATAAAAAGGTT
AGAATGTTTTGGTGGGTTTTTATGATTGGGTTTCTAATTAAGTTGCCTGTATATCCTTTTCATTTATGGTTA
CCTAAGGCTCATGTAGAGGCTCCTGTTGCTGGATCAATGGTTTTGGCTGGGGTAGTATTAAAACTGGGTG
GATATGGGATATTGCGGTTGTTAATAGTAATAAGTGTAAATTTGGGGTTAAAATGTAATCTGATACTATGC
GTAGGGATAGTTGGTGGTTTTTATGCAAGATTGGTATGTTGTCGTCAGACGGATATAAAATGTTTAGTAGC
ATACTCTTCTGTAGGTCATATAAGGTTGGTTACTTTGTGTCTAAGAAACATTAGAATAGGGGTAAAAGGT
GCTTTGTTTATTATAATTGGGCATGGGTTATGTTCTTCTGGGATATTTAGCCTTGTTAATGTTTTTTATAGTC
ATTCTGGTTATCGAAATCTGTATATAAATAAGGGGTTTTTGATAAAAAGTCCAATTCTGTGTTTGGTAGGA
TTTTTATTGTGTTCTAGTAATATAGCTGCTCCGCCGAGGTTAAATTTGTTGGGTGAGGTGCTAATGTTTATT
TGTTCATATGTAATTTCTTTTTGTTTTCTGTTGTTGTTAATAGCAATAACTATAGTTAGTGCTGTTTATAGCT
TGCATTTGTACACATCTACATGTCATGGAAAAGGGTCAGAGAGGATGATGGGATGAGATATTTATAGATC
TGTGTTCGTTTTGTTAGCTCATTGGTTGCCTTTGAATTTTTTGTTTTTGTTTATACCTTagTTAAAAAAAGG
GCATCTTATTAGAATTTTCATTGGAATGGAATTTATAATGTTAGGGGTGTTGTATAGTGCTAGGATCATAAT
GTGTTATAATGTAAGAATAGTATTCTTAATTATGTGTTTAGGGGTATGTGAGGCAAGGGTATGCTTAGCTT
TATTAGTTATGATAGTGCGTTTGTCAGGAAATGATTTAATTAGAAGATTGTCTTTGTATaaTTTAGGAAAGT
TTATCAAAAAATATTATTCATAGTGGTTTTTGGGTATTGCTTTATTTTATTAAGAAGAGTGAAGAGTGTCTA
TTTTATGGAGTTTATGTTGTGGGATTTAGGGGGTATAGGATTGTCTATTAATTTATTGGTTGATAGAATTGG
GGTGATATTCATTGGGACTATCATAATTATTTCTGGTAGAGTATTAATATACTGCTATTGGTATATAGATGAT
GAGAAATATTTTTTCCGTTTTATATACTTGGTTTATTTATTTGTTGGATCAATAATATTTATAATTTTAATTCC
TAATTTAATTACGTTGTTAATTGGGTGGGATGGGTTAGGATTAACTTCTTTTTTGTTAGTTGCACATTATCA
AAATTCTAGGTCTTTGTCAGGGAGCTTGTTAACGGCATTGACGAATCGGATTGGGGATGGGTTAATCTTG
TTAAGAATTTCTTTGTGAGGAAGAGAAAATATGTGAGTTTTGTATAATTTCAGAGGATCTTTTATAAGTGT
CTTTATTTGTGCGCTTATTTTTGGTGGAATAACTAAGAGTGCGCAGATACCTTTTTGTGCTTGGTTACCGG
CTGCTATGGCTGCTCCCACTCCTGTGTCTTCTTTGGTTCATTCTTCCACGTTAGTTACAGCGGGTGTATAT
TTACTTATTCGTGCGTATCCAGTAATAAGAAAGAGAATAATATTAGTGTTAAAGTTACTAAGGTTGTTTAC
TTTGGTGTTAGCTAGAAGCGCAGCAATTTTTTGTTTTGATATAAAGAAAATTATTGCATTATCAACTTTAA
GGCAATTAAGTGTAATAATATTTTCTTTATCTCATGGGTTGGTTTATGCTTCTTTTTTTCATTTAGTAATGCA
TGCATTGTTTAAAGCACTTCTTTTTTTTGTCAGCAGGGGTAGTTATTCATTCTATAAAAAGTTGCCAAGATA
TTCGTAGAATAGGAAATTGCTGAGCAAATATGCCTTTTAGAATAGTTTCTATATTTATTGCGAGGTGTTCA
CTTTCAGGTCTTCCATTTATAAGTGGGTTTTTTTCTAAGGATATAATTGTTGATGTGTATAATAGAAAAAAT
TTTTTCTTTTTTGTTATAATGCCTGGGTTAGGGTTGACTAGAATTTACAGAATACGAGTATGAATAGTAAG
GTTTGGAGAAAACAAAGTTATGTTAGGATGTTTGAGAGTAAAAGAGCCAATAAAACTTCTAATCCCTTAT

TTAAGGCTTGGTTTTGGGGCATTATTTTTGGGTAAAATAATATGTCCAATTACTGAGAGGTTGATTTATTA
CAGGAGTTCATCATTAGGGGAGATATTAGTTTTAAGAATTCTATTTAGGGGAGTATATTTAAGAGTAGCAA
ACTGGGGAAAAGGGCCATCTttcAAAAAGTCTTCTTTTTATTTAGAATATGATACTTAGAGTTAGTACATG
TAATCAAAAAGATATTTTTTGACCTTAGAAAATCTATTTATGAAGTGTGTGATAAAGGAATATTAGAGACA
GTGGGGCCGAAGTGGATGAGAATAAAAATAAGATGTTATAACGAGAATTTCTTTCTTTTTTTTTTTGTGT
TTAGGGGAATAATTTTATAATGAGAATTGTTATTATGGAATTTATTTATTTGCATTTGGTAGTAGTTAGATT
GTGTGTTGTTTTTGTTAGTCAACCTTACTTACTGGGGTTAGTTCTTTTAATTTCTTCAATACTAGTTTGTTT
AATTATTGGGGTTAACATTAGAAGATTTCTTGGATTTTTGTTATTTATAAGATATGTGGGAGGGCTCATAGT
TTTATTTGTTTACGTTCTTAGTGTATTTCCTAATGAAAATTTTAAAACGAAAACGTTTTTTATAAGGTTAAG
GTTTGGAGTTCTGGTAGTGATATTACCTGTAAAGTCGGGTGCTCAAGAACTGCAGTTTCATTTTATGAGG
TTTTCTCATGTTTTTGTTTTTATAGCTTTATTTCTTTTGTTTATTATATTATGTGTTTCTTATTTAGTAATAAA
AAAACGGGTTCCTTTACGATCAATC"/>
<sequence id="seq_Perna_viridisMW727515" spec="Sequence"
taxon="Perna_viridisMW727515" totalcount="4"
value="ATGTTAATAGATGTTTTTCTATGTTTGATGATTATAATTTAATACATTTAAAAGGTGGTATGTCTG
GGGATATTCATTGTTTATTCCTTTGTTTATCGTGAGATCATCATTAAGGATTTATTATTCATTTTTACAGAAA
TGTTTGAAATTGATTGGTAGGGGAAGGGGTTTGTATATTTCAGGTTTTCCTCTATTAGTTGTGAGTCTTTT
TTCAATTATACTAAGGGTAAATGTTTCTAATAGAATTCCTTATTTTTTTAGGGTGAGGGCACATTTTTCGTT
TGGCTTTACTTTTGCGTCTGTGATTTGGCTGTGTATTATTACTTCTAGGGTTTTTACAAGGTTTATTCAGAA
TATGGCTTTGCTGGTTCCAAGAGGTCCTGAGGGGTTAGCTCCATTTATGGTGCTTTTAGAGATTATTACAA
ATTTATTGCGTCCAATTACTTTAGTTATTCGATTAGCTATAAATATGGCTACTGGTAAGGTTATTATGTTATT
ATTAGGGAATGCTGCGTTAAATCTTGCATTTGTTGGGGTTGTAGGGTTTATAGTTGTTAGAGTGTTAGGTG
CTATTTTTTCTTTAGAGGTGGCTGTTAGGTTTATTCAGAGATATATTTTTTGTACTCTATTATGTTTGTATGC
TGATGAACATAGGAAATAGcgatggtttATGTCTACTAATCATAAAGATATTGGTACTTTGTATTTACTTTCTGG
GATATGAGCTGGGTTAATGGGGAGAAGGCTTAGGTTAATTATTATTCAGTCTCATCCCGGGGGTAATTTTT
TGAAAGAAAGGTTATATAATGTTGTAGTAACAACTCATGCATTAGTAATAATTTTTTTTGCTGTAATGCCTT
TACTTATTGGTGCTTTTGGGAATTGATTACTTCCATTATGTATTGGTGGTGTTGATTTAATTTTTCCTCGTTT
AAATAATTTGAGATTTTGGTTGGCACCTAATGCTTTGTACTTACTTATTTTGTCGTTTATAACGGAGAAAG
GAGCTGGGACAGGTTGAACTATTTATCCACCTTTATCTTCTGGGTTGTACCATACTGGGCCTGCTGTTGAT
ATTTTGATTACGTCTTTACATTTAATTGGATTGAGTTCTTTATTAGGTTCGATTAATTTTGTGAGGACTAAT
AAGAATATGCCTACAATAAAAATAAAGGGTGAGAAATCTGAGTTGTATTTGTGGAGGATTACCGTAACCG
GTGTTCTTTTAATTATTTCTGTGCCAGTTCTGGCTGGTGGGATTACTATATTGTTGTTTGATCGAAATTTCA
ATACTAGGTTTTTTGATCCTATTGGAGGGGGAGATCCTGTTTTATTTCAGCATGTATTTTGGTTTTTTGGTC
ATCCTGAGGTGTACATTCTTATTCTTCCGGCGTTTGGTGTGATGTCAAAAGTAATTATGCATTATTCTGGT
AAAGATTCTGTTTTTGGTTCGGTTGGGATATTATATGCTATAGTTGGTATTGGTGTTATAGGTTGTGTGGTG
TGGGCCCATCATATATTTACTGTGGGTTTAAATGTAGACACACGAACTTATTTTACATCAGCTACAATAGT
AATCGCTGTTCCTACGGGTGTGAAAGTTTTTAGTTGGATGGCTACAATAGGAGGAAGAAAAATTAAGTT
AACTACATCTGTTTTGTGAAGGACTGGTTTTTTATTTTTGTTTACTGTTGGGGGACTTACGGGAATTATAC
TTTCTAGTTCTTCTTTGGATGTAACATTACATGATACATATTATGTAACAGCACATTTCCATTATGTCTTGTC
GATAGGGGCTGTATTCGGTATTTTTTGTGGGTTGAACCATTGATTTCCTTTGTTTTATGGTGTAAACTTTC
ATAAAAAGTGATCTAAAGTTCATTTTTATTTAATATTTTTAGGAGTGAATTTAACATTCTTTCCCCAGCATT
TCTTAGGATTAAAAGGGATACCGCGTCGTTATTGTGATTATCCAGATTGTTATTCTACATGGCATTGGGTAT
CTTCTTATGGTGCTTTAATTTCTTTTGGGTCTTTGTTATATTTTATTTTTGTTGTATGGGAGGCTATGGTGAG
TCAACGAGGTGTGTCATTGTTTGAGGCTATATTTTTTGGTGATGTATTAGAGGAGTTTGGTTCTGAAATTA
TTAAATATTATGGTTATGTGATAATAGTTTTATTGTTGGTTATTATTGTTGTTTTGTATATGGGAATAATGATT
ATGAATCATAGATATTCTTATCGTAATTTTAAAAATCGACAAATATTGGAGTATGCTTGAACTGCAATACC
AACGTTTCTTTTGGCGTTATTATGATGTCCTTCAATTTTGAATTTGTATCGGATAGATGATTTGAAGGATCC
TGTCTGAAGGTTTAAGGCTGTTGGAAAGCAGTGGTATTGAACTTATGAGAATGGGGATACGATGGTTATT
GATTCTTATATAGATCGTGATTCTGGGGTGGGTTATCGATTACTTGATGTTGATTGGCGTTTAGTGGCTCCT
GCTAATGCGCAGATTTCGTGTTATGTTACAAGGAGGGATGTAATTCATTCTTTTGCGCTTCCGGGTGCGTT
GATAAAAGCGGATGCTATTCCTGGACGGATTAATGTACTTCCTATAAAAATTAGTCAGAGTTGTATTTTAT
ATGGGCAGTGTTCAGAAATTTGTGGGATCAATCATAGATTTATACCTATTGTTATTGAGTTTGTTCCCGTT
GATGTGTTTATGGATTTTTATGGGTATTAGaattaa-----------------------
ATGCATCGAAGTCCTTATTATGTCCCAGGGCCAAGGCCTTGGCCATTAATTGTAGGTGTGTGTTGTAATAG
GTTATGTGTAAGTCTTGTGTTGTGGATACATCGAATACAAGAGAAACTTTTTGTTACATTATTGGTGTTAA
TTATTACTGCGATTCTGTGATGAACAGATGTTTTGCGTGAAGGGGATATGGGTGTGCAGACTCAGTTTGT
AATTAAGAGATACCGAGATGGAGTTGGCCTTTTTATCTTTTCTGAAATTATATTCTTTTTTTCGTTTTTTTG
GGCACTTTTTCATAGTTGTCTAAGTCCTTCTGGAAATCTTGGGTGCGAGTGACCTCCTTTAGGGATTCGG
ACGTTAGATCCGTTATCTACTGCCCTATTCAACACTTTTTTGTTAATTAGAAGCGGGTCATTTTGTACTTAT
GTTCACAACTCAATTCGAACAGGTTATGATTCTTGATGTCTGATCAATATGGTAATTACTATCTCTTGTGG
TGTAATATTTTTAATTGGGCAAGGTCATGAGTATTTTTTTAGTCCTTTTAGAATTGCGGATAGAGTGTACG
GAAGAACTTTTTATATGCTAACTGGGTTCCATGGTGCTCATGTTATGGTAGGAACTACTTGGTTAATTGTT
AGGTTGAGTCGGATGTGATTAGGCCATTTTTCTAAAGACCGTCATTTTGGATTGGAAGCTTGTTTATGATA

TTGACACTTTGTTGATGTGGTGTGGCTAGGGGTATGACTAATTGCTTATTTTTGGATGGGGGGACCTCTT
CGGAAACGTCATTGATTATTGAAAATTTTGAATAATAGGTTATATGATTTACCATGTCCAATTAATTTAAAT
ATATTTTGGAGATTTGGTTCTATGTTAGGTTTGTGTATTGTTATTCAGGTAGTTAGTGGAATTTTATTATCTC
TTCATTTTATCCCTCATGAGGCAATAGCTTTTGATTCTGTCTATCATATTATGCGTGATGTGAATAAGGGTT
GGTTTTTGCGGAATGTTCATGTTGGTGGGTGTTCTATGTTCTTCGTTTGTTTGTATGTTCATATTGGTCGTG
GGATTTATTATGGGTCTTATTTAAGTAAGCACGTTTGGTTAGTGGGTGTGACTCTGTTTTTGTTGGTGATG
GCAGAAGCATTTTTAGGGTATAGATTGCCTTGGGGTCAGATATCGTTTTGAGGTGTAACTGTGATTTCTA
ATTTGTTTACTGTGATCCCGTATATTTCTCAAAATTTGTTGTTTACTATTTGGGGGCATTGAAGCGTTAGG
GGGTATACTTTACAGCGATTTTTTATTTTTCACTTTCTTCTTCCTTTTGTCATTATTGTTTTTTCGACTTTGC
ATTTATTTTTTCTTCATGAGAATGGGAGAAATAATCCTTTGGGTATTAGAAGGGATTCAATATGTATTCCTT
TTCATCCATTTTACACTGTCAAAGATATTTTTGGGTTTGTTTGTTTTGGTTGAGCTTTGATATATTTTGTTT
GTGTAAATCCTGAACTGGTAGTAAATAAAATCAATTATCATCCAGCAGACCCTTATCACACTCCTTTACAG
GTAGAGCCGGAGTGATATTTTTTGTTTGCTTATGCTATGTTACGTTCAATTCCTCATAAGCTTAGTGGGGT
GCTTGCCTTACTTGCTTCAGTAACTGGATTGTATCTATATCCTTTTATTCATACTGGGAAGTTTCGTAGGTT
TGCATTTTATCCCGTAAGACAGATGCTGTTTTGGTGTTTTGTTGTAAATTTTTTGAGGTTAATTTGGGTTG
GGCAAATACCTGTGCGAGAGCCTTTTATTAGAATAGGTCAGGTATATACTGGTATTTATTTTTCGACTTTG
ATTTTTCCTCCTATGTTAACTGGTCTTTGGGATAAGTTGATTTTCCTTTATTTTGTAAGGTTGATTCTTCCG
TTTGTTTGTGTTTTGGTGAGGGTTGCTTTTTATACTCTTGTTGAACGAAAGGTGTTAGGTTATATTATGATT
CGTAAGGGGCCTAATAAGGTTGGGTATAGGGGAATTATGCAGCCTTTTAGGGATGCAGGGAAGCTTTTTA
GAAAAGAGTATGTGATACCGGGATTTGCTAATGTTGTTCCTTTCGTTTTGTGTCCTGCTGTTATTCTTTTT
ACTAGAATGATGTTATGGTTTCTTTATCCTTATAGTTATGTTTCGATAGTGTTTACGTGTGGGATTGTTCAG
TTTTTAGTAACATCTGGTATCCATGTATATGGAGTTATAGTAGCTGGTTGGTCTTCTAATTCAAAGTACTCT
CTTTTAGGGGCTGTTCGGGGGGTAGCACAAAGAATTTCTTATGAAGTTCCTATAACTTTTGTGGTTTTGA
TAGTAGCGTTTTGTATTGGGAGATTATGGTTGCAGGAGGTTAAGATAATATTTTCTATTCTTATAAGATTAT
TGAGGAGAGGGGTTTGAGTAACTTGTATGTTGGCTGAGACCAATCGCGCTCCGTTTGATTTTGTTGAGG
GGGAGTCTGAGTTAGTATCAGGGTTTAATGTGGAGTATAGAAGAGGAGGTTTTGCAATAATTTTTATGGC
GGAGTATGGGGCTATATTATTTAATAGGATTTTTTTTATTACTATTTTTTTAGGCGGTAATGAGTTATTAATG
AGGGTTAGAACAATAGTATGAGTTCTTTTTTTTGTGTGAATTCGGGGAACTGTTCCCCGAATTCGATATG
ATCAGCTTATAGGATTGTGTTGGAAAGTTTATTAAGGGTAGTTTTGAGAATAACTGGATTTGTAGCTATT
ATCAGATTTTTTTTatgataggaATAAGAGTTAGACCTTTGTTAAGAGCTTGTGGGTTATTAGTTGTATTAGGTA
ATTTAGTAAGGATTATAAGAAATAGATGAATGGGTGTGTGGTTAGGAATAGAACTTAATTTATTTGGCTTT
TTAATTATAATAAATCCTGAAGGGTGTTGTGTTGCTCAGGGAAGAATCAAGTACTTTATTGCACAAAGGA
TTGGTTCTATGTTAATATTGTTTGGGTTTTTTTGTGTGTCTATGTTTAATAGAGTGGGGAGGTTAATTCTTG
TTGGGGGAATTCTTTTAAAAGCGGGGGTTTTTCCTTTTCATAGATGAGTTCCGGACGTTGTTATTGTGTCT
AATTGGTTTATTGGGTGTATAATTTTAACATGGCAAAAGTTAGCTCCATTTTCTTTGTTTTCGTTTTTTCCT
AGTTCTTGGGTCTTGGTAATAAGATTGATTTTTATAAGGTTGGTGGGATGCTTTGGGGGATTAAATCAAC
ATTCAGTTCGTGGAATGGCAGTTTATTCTTCTTTTGTTCATAACTCTTGGCTGATTCTTTCTTTATTTTATTC
CTTTGGTGTTTTTTTTATTTATTATTTGGTGTATAGGTTGAGGGTTTTAATATTTTTTTTGAGGTGTTGGGTA
GTTAGAAAAAGGAGCTTAATAAGGTACTCCGTTAGATGATTGGGAATATTAAGTCTATTAATGTTATCTGG
TGTTCCACCTTTTATGGGGTTTTTTGTTAAATTGATTGTGATGTTAAGATGCCCGTCTATTCTTTTGATTAT
TTGTTTGGGGGGATCTATCGTAAGGCTAAAGTTTTATATTTCTTTTTTCTATAGAATGATTTTGAATTCTTAT
TTTCAATTGGAGTTGGTTTATATTTTTTGTAGGATGAATTTTGTAGTAGGGGTTAGTTCGTTATTGTTTTTT
ATATGGTGTATGATTGAATGTTTTCTTTTGTTTTTTCTTTTTATTTTAAGGATGGTGTTAGTACTAGTATTTT
TATTATCAGAGAAAACATTTGAGAGACGTGAGAAGTCGTCCCCTTATGAGTGTGGGTTTGACCCTATTTT
AAGGGCGCGAAGGTCTTTTTCTCTTCGTTTTTTTCTTTTAGGGGTCTTATTTGTTGTTTTTGATGTTGAGT
TGTCTTTGTTAATACCTGTGGTTCTTAGGATTAGGGTGGGGTTTAGCTATGTGGGGCTTATTAGATGTTTT
ATCTTTCTTTTAGTTTTGTTTTTAGGAATTTTTCATGAGTGACGAGAAGGGTCATTGAACTGGGTTATTTA
GGTGGCAGTAAGTATAATATTAGTATGAATCTTTCTTTGTATTGTTAGAAATAAAATGTTGAGGCTTTTAA
GTTTGGCTTTGTTAATAGTAGGGGTTATAGAGATAGGAGTAATCAATGATGAGGTAATTAGAATGGGGGG
TAGTCTTAGATATGATTTTTTATCCTATAGGTTAAGGGTTTTGAGGATTTTTATTATATTGTTAAGCTTAGTT
AGAAGAAAAAGGGTAATACGAACTTCTTTGTTTTATTTTTTAAATTTAAGATTATTAATAGTGTTAGTTTTT
GCTTTTTGTACTTCTAGTTTTCTAGGGTTTTTCTTTTTTTTTGAGTCTGCTTTGATTCCTTTAATTTTGGTTA
TTATAGGGTGACGGATAGAGGCAAGAACTTATATGTTTATCTATACGGTATTTGGTTCTCTTTTTTTCTTGT
TTGGGATTTGTCTTCTTTTTTATAGAGGAAGGGATAATATATTATTAGGAAGAAATGTAAATAAAAAGGTT
AGAATGTTTTGGTGGGTTTTTATGATTGGGTTTCTAATTAAGTTGCCTGTATATCCTTTTCATTTATGGTTA
CCTAAGGCTCATGTGGAGGCTCCTGTTGCTGGATCAATGGTTTTGGCTGGGGTAGTATTAAAACTGGGTG
GATATGGTATATTGCGGTTGTTAATAGTAATAAGCGTAAATTTGGGGTTAAAATGTAATCTGATATTATGCG
TAGGGATAGTTGGTGGTTTTTATGCAAGATTGGTATGTTGTCGTCAGACGGATATAAAATGTTTAGTAGCA
TACTCTTCTGTAGGCCATATAAGGTTGGTTACTTTATGTCTAAGAAACATTAGGATAGGGGTAAAAGGTG
CTTTGTTTATTATAATTGGGCATGGGTTATGTTCTTCTGGGATATTTAGCCTTGTTAATGTTTTTTATAGTCA
TTCTGGTTATCGAAATCTGTATATAAATAAGGGGTTTTTGATAAAAAGTCCAATTCTGTGTTTGGTAGGAT
TTTTATTGTGTTCTAGTAATATAGCTGCTCCGCCGAGGTTAAATTGTTGGGTGAGGTGCTAATGTTCATT
TGTTCATATGTAATTTCTTTTTGTTTTCTGTTGTTGTTAATGGCAATAACTATAGTTAGTGCTGTTTATAGCT

TGCATTTGTACACATCTACATGTCATGGAAAAGGGTCAGAGAGGATGATGGGATGAGATATTTATAGATC
TGTGTTCGTTTTGTTAGCTCATTGGTTGCCTTTGAATTTTTTGTTTTTGTTTATACCTTagTTAAAAAAAGG
GCATCTTATTAGAATTTTCATTGGAATGGAATTTATAATGTTAGGGGTGTTGTATAGTGCTAGGATCATAAT
GTGTTATAATGTAAGAATAGTATTCTTAATTATGTGTTTAGGGGTATGTGAGGCAAGGGTATGCTTAGCTT
TATTAGTTATGATAGTGCGTTTGTCAGGAAATGATTTAATTAGAAGATTGTCTTTGTATaaTTTAGGAAAGT
TTATCAAAAAATATTATTCATAGTGGTTTTTGGGTATTGCTTTATTTTATTAAGAAGAGTGAAGAGTGTCTA
TTTTATGGAGTTTATGTTGTGGGATTTAGGGGGTATAGGATTGTCTATTAATTTATTGGTTGATAGAATTGG
GGTGATATTCATTGGGACTATCATAATTATTTCTGGTAGAGTATTAATATACTGCTATTGGTATATAGATGAT
GAGAAATATTTTTTCCGTTTTATATACTTGGTTTATTTATTTGTTGGATCAATAATATTTATAATTTTAATTCC
TAATTTAATTACGTTGTTAATTGGGTGGGATGGGTTAGGATTAACTTCTTTTTTGTTAGTTGCACATTATCA
AAATTCTAGGTCTTTGTCAGGGAGCTTGTTAACGGCATTGACGAATCGGATTGGGGATGGGTTAATCTTG
TTAAGAATTTCTTTGTGAGGAAGAGAAAATATGTGAGTTTTGTATAATTTCAGAGGATCTTTTATAAGTGT
CTTTATTTGTGCGCTTATTTTTGGTGGAATAACTAAGAGTGCGCAGATACCTTTTTGTGCTTGGTTACCGG
CTGCTATGGCTGCTCCCACTCCTGTGTCTTCTTTGGTTCATTCTTCCACGTTAGTTACAGCGGGTGTATAT
TTACTTATTCGTGCGTATCCAGTAATAAGAAAGAGAATAATATTAGTGTTAAAGTTACTAAGGTTGTTTAC
TTTGGTGTTAGCTAGAAGCGCAGCAATTTTTTGTTTGATATAAAGAAAATTATTGCATTATCAACTTTAA
GGCAATTAAGGGTAATAATATTTTCTTTATCTCATGGGTTGGTTTATGTTTCTTTTTTTCATTTAGTAATGCA
TGCATTGTTTAAAGCACTTCTTTTTTTGTCAGCAGGGGTAGTTATTCATTCTATAAAAAGTTGCCAAGATA
TTCGTAGAATAGGAAATTGCTGAGCAAATATGCCTTTTAGAATAGTTTCTATATTTATTGCGAGGTGTTCA
CTTTCAGGCCTTCCATTTATAAGTGGGTTTTTTTCTAAGGATATAATTGTTGATGTGTATAATAGAAAAAAT
TTTTTCTTTTTTGTTATAATGCCTGGGTTAGGGTTGACTAGAATTTACAGAATACGAGTATGAATAGTAAG
GTTTGGAGAAAACAAAGTTATGTTAGGATGTTTGAGAGTAAAAGAGCCAATAAAACTTCTAATCCCTTAT
TTAAGGCTTGGTTTTGGGGCATTATTTTTGGGTAAAATAATATGTCCAATCACTGAGAGGTTGATTTATTA
CAGGAGTTCATCATTAGGGGAGATATTAGTTTTAAGAATTCTATTTAGGGGAGTATATTTAAGAGTAGCAA
ACTGGGGAAAAGGGCCATCTttcAAAAAGTCTTCTTTTTTATTTAGAATATGATACTTAGAGTTAGTACATG
TAATCAAAAAGATATTTTTTGACCTTAGAAAATCTATTTATGAAGTGTGTGATAAAGGCATATTAGAGACA
GTGGGGCCGAAGTGGATGAGAATAAAAATAAGATGTTATAACGAGAATTTCTTTCTTTTTTTTTTTGTGT
TTAGGGGAATAATTTTTATAATGAGAATTGTTATTATGGAATTTATTTATTTGCATTTGGTAGTAGTTAGATT
GTGTGTTGTTTTTGTTAGTCAACCTTACTTACTGGGGTTAGTTCTTTTAATTTCTTCAATACTAGTTTGTTT
AATTATTGGGGTTAACATTAGAAGATTTCTTGGATTTTTGTTATTTATAAGATATGTGGGAGGGCTCATAGT
TTTATTTGTTTACGTTCTTAGTGTATTTCCTAATGAAAATTTTAAAACGAAAACGTTTTTTATAAGGTTAAG
GTTTGGAGTTCTGGTAGTGATATTACCTGTAAAGTCGGGCGCTCAAGAACTGCAGTTTCATTTTATGAGG
TTTTCTCATGTTTTTGTTTTTATAGCTTTATTTCTTTTGTTTATTATATTATGTGTTTCTTATTTAGTAATAAA
AAAACGGGTTCCTTTACGATCAATC"/>
<sequence id="seq_Septifer_bilocularisMK721549" spec="Sequence"
taxon="Septifer_bilocularisMK721549" totalcount="4"
value="ATGTTGATAGATGTTTTTCTGGGTTTGACGATCATAATTTTAATATTTTTTTGTAACATTCATTTG
AGCTTTTAGATGTGTTTTTCCCATTTTGGTAATATCATCTATATTAGTTGTTGTGGGTAGTATCATTTCTAAG
TGCTATATAACTCTTGGAAGAGGAAAAGGGTTGCGTTTAACAGGGTTTTCCTTATTAATTTGTTCTTTATT
TTTTATTATTGTTTTGTCTAATCTTTCAGGTTGTATTCCTTATTTTTTTTCTGTTAGTGCTCACTTTGTTTTT
GGGTTTTCTTATGCCATTATTATTTGATTTAGTATTATCATTTCAACTGTTTTCTGTAGTTATGAGCAGACTG
TAAGAATAATAGTTCCTTCTGGTCCCTTAGGTCTTGTACCGTTTGTTGCTATTTTGGAAGTTTTAAGTCAC
ATATTACGTCCTCTTACTTTAATTGTGCGGTTAGCATTAAATATTTCTACTGGTAAAATTATTTTGACTTTAT
TAAGTGAGATGGGTTTTGTTTTGTTTCTTTATAATTTAGTGTGATTTGTTCTTGTTAGTATTATAGGGCTAA
TTACTGCATTAGAATTAGGTGTGAGTTGTATTCAGGCTTATATTTTTTGCATTTTATTATGCCTATATAGTGG
TGATCATAGAGAATAACGTTGGTTTACATCTGTAAACCATAAAGAAATTGGTACTTTATATCTCCTTATTG
GTGTGTGATCGGGGTTAGTAGGAACAGGATTAAGAGTGTTGATTGTTCATTCCCATCCTGGAAGTAAGCT
TATAAAAGAAAGGTTTTATAATGTTGTGGTTACGTCACATGCTCTTATAATAATCTTTTTTGCTGTAATACC
TATTTTAATTGGTGGATTTGCTAATTGATTGCTTCCTTTATGTCTTGGTGCTGCTGATCTTATTTTTCCCCGT
CTTAATAATTTGAGTTATTGACTTGTTCCTAACTCATTATATTTGATAACTTGTTCTATATTTACTGAAAAAG
GTGCTGGTACTGGCTGAACTATTTACCCTCCTTTATCTAGTGTCGCTTATCATAATGGCCCCGCAGTAGAC
ACTGCTATTTTTTCTTTGCATGTTGTGGGTATAGGGTCTCTTGTTGGTGGTTTAAATTTTTTAGTTACTAAT
AAAGATGTTCCTGTTTTTCATATGAAAGGTGAAAAAGCGGAGTTATACTTAGCAAGAATTTCTGTTACAA
GTTTTTTGCTTGTTGCGTCTATTCCTGTTCTTGCTGGTGGTATTACTATGTTGTTATTTGACCGTAATTTTA
ACACTACATTTTTTGATCCTATAGGAGGAGGTGATCCTGTTTTGTTTCAGCATATTTTTTGGTTTTTTGGTC
ATCCGGAAGTCTATATCTTAATTCTTCCTGCTTTTGGTATTATGTCAAAAGTAATTTTACATTTTTCGGGTA
AATTAAAAGTTTTTGGTGCTTATGGTATGTATTATGCGATGGTAGGAATTGGTGGTTTGGGGCTTATAGTAT
GGGCTCATCATATATTTACAGTAGGTTTAAATGTTGATTCTCGTATGTATTATACTTCTGCTACAATAATCAT
TGCTGTTCCCACGGGTGTGAAAGTTTTTAGATGACTTTCTACTATGGCAGGAGGTCGTATTAAATTCTATC
CTCCTGCATGTTGAAGAACGGGGTTTTTATTTTTATTTACTGTCGGTGGTTTGACAGGAATTATACTATCT
AGTTCTTCTTTAGATGTTAGTCTTCATGATACTTATTATGTTACAGCTCACTTTCATTATGTTCTTTCGATGG
GTGCTGTATTTGGGATCTTTTGTGGTATTACTCACTGATTTCCTATATTTTATGGGGTTAGTTTACATCGTA
AATGGTCGAAGATACATTTTTTTGCTATATTTGTAGGGGTCAATTTAACTTTTTTTCCTATACATTTTTTAG

GATTGAGAGGTATGCCGCGCCGGTATTGTGATTATCCAGATTGTTATTCTAAGTGACATTGATTGTGTTCT
TACGGAGCTACTGCTTCTTATATTTCTTTGCTTTTTTTTATGTTTATTTTATGAGAAGCAATAGTAAGCCAG
CGTAGAGTGGTTTTTTATGGGTCTATATATTTTCGTGATTGTATTTATAAAATTGGTCAAGATATTTTAGTGT
ATCATGGTTATGTTATAATGGTAGTATTTCTTGTTCTTATACTAGTGTTGTATGTTGGTACGGTAGTTACATG
TACAGGGTATCGTAGGCGTTTTTTTACTGATCATCAACAATTAGAATGGTGATGAACAGTTATTCCTATGA
TTCTTTTAGCTGCTTTGTGATGTCCTTCAACTTTAAATTTATATCGTATGGATGATATTAAGATTCCACGGT
GAAATTTTAAAGCATTAGGTAAGCAATGATATTGAAGTTACGAGTGTGAAACTGTTTTTATATTTGATTCT
TACATAAAACAAGATAGTGGAACAGGGTACCGTTTACTAGATGTTGATCATCGTATGGTAGCACCAGCAG
GAGTGCAAACAACTGTTTTTGTAACTAGTCCAGATGTTCTTCATTCTTTTAGGTTGTATGGCACTATACTA
AAAGTTGATGCAATTCCTGGTCGTCTTAATCAGCTTCCTTTGTTGGTAAATCGTGTGTGTATTTTGTATGG
TCAGTGTTCTGAAATTTGCGGGGTTAATCATAGATTTATGCCTATTGTCATTGAATTTATTCCTGAGCAATA
TTTTATTAAGTGAATTAATGCTATGGAAGAA-----------------------
ATGAAACGAAGACCTTTTTATGTTCCCGATCCAAGTCCGTGGCCTTTTTTTGTAGCTATTGCTTTAAATAA
TATGGCTATTGGGTTAGTTTTATGGATACATCGTAAAGATTTAATGTTGTTGGGTGGTGCTTTAATTTTATT
AATCAGATCTGTCAGCTGGTGGCGTGATTTGTTACGTGAAGGTGACATGGGTTTTCATACGCGGTTTGTT
ATTAAAGGTTATCGTGACGGTATAGGATTGTTTATTTTTTCCGAAGTAATGTTTTTCTTCTCTTTTTTTTGA
GCTTTTTTTCACAATGCATTAAGTCCTTCTACCGAATTAGGTATGCGTTGACCTCCTCCAGGGATTCGTGC
TCCTCAGCCTTGTTCAATTCCACTTTTCAATACAGCGTTGTTGATCAGTAGCGGTGCTTTTGTTACTTTGG
CTCATAAAAGGGTAATTAGAGAGTATGTTCAGGGGCCTTTGTTAGGTTTATTTATAGCAATTGTCTGTGGT
GTATTATTTTTGTTTGTTCAAGGATTTGAGTATTTTAGTAATTCGTTTACGTTGTCTGATAGGGTTTACGGA
AGTGTTTTTTATGTTTTAACAGGTTTTCATGGGACTCACGTATTAGTAGGTAGTGTTTGGCTTATTGTGAC
TTTTGTACGAACGTGATTAGGTCATTTTCGACAAAGACGTCATTTTGGTATAAGAGCTTGTATCTGATACT
GACATTTTGTAGATGTTGTTTGATTATTTGTCTATTTTATAGTGTATTCTTGATTTGGTGGTCCTTTTCGTAA
ACGTAATAAATTGATAAAAATTATCAATAACAGGCTATATGATCTTCCTTGTCCTATTAACTTGAGAGTATG
ATGAAGATTTGGTTCAATGCTTGGCTTATGTTTAGTTATTCAGATTGTAACTGGTTTTATATTAAGAACTTT
TTATACTGCTGATGAAACTATGTCGTTTGATTCGGTAATTTTTATTATACGTAATGTTAAAAAGGGTTGAAT
GTTTCGTAGCATTCATGCTAATGGTGCTTCAGTATTCTTTATTTGTATTTATATTCATATTGGTCGTGGTTTG
TACTATGGGTCATATCTTTATGTCCACGTATGAAATATTGGAATTATGCTGTATTTGTTACTAATAGCTGAA
GCTTTTTTAGGTTATGTTCTTCCGTGGGGTCAAATGTCTTTTTGGGGTGCTACTGTGATTACAAACCTTAT
GACTGTAATTCCATTTTTTGGCAAAACTATCACTCAATGAATTTGAGGTTATTATACTGTGTCTAACCCAA
CACTAAAGCGTTTTTATTCTTTTCACTTTATTGTTCCTTTTTTAATGGTTGTTATAAGTGCTTTGCACCTTT
TTTATCTTCATGAAACTGGTAGAAATAATCCTTTAGGTATTGAAAGTGATATAATGTGTATTCCTTTTCATC
CTTTTTACACTGTAAAAGATCTTTTTGGATTTGTTTGTCTTGCGTGAGGTTTGATATTTTTAGTATGTGTTA
AACCTGAGATGTTAGGTAATGTTACTAATTATATTCCTGCTAATCCAATAAAAACCCCTAAACATGTAAAA
CCTGAATGATATTTTCTTTTTGCATACGCTATCTTACGTTCAATTCCTAATAAAAGAGGTGGTATTTGTGCA
ATGTTGTTGTCTATTTTGATTTTGTATCTTCTTCCTTTTATTCATACCGGTAAGTTTCGTAGATTGTGTTTTT
ATCCTTTTAATCAAATAATTTTTTGATGTTTTATTTCTATTTTTATTGGACTATCTTATGCTGGTTATAGTCCT
CCGCGTGAACCGTGGTTAACGTGTGCGTGATATTTAACTTTATTATATTTTCCTTTAATTGTTTTGAATCCT
TTGTCTTTATTTGTATGAGATTGGTTAATTAGTACACATTTTTTAATTTTAATTTTGCCATTAGTATGTGCTT
TGCTCGCGGTGGCATTTTATACTTTATTAGAACGTAAATTGTTGGGTTATATTATATTACGTAAAGGACCAA
ATAAAGTTGGTTTTATAGGTATTCTTCAGCCTTTCAGGGACGCAGGTAAACTTTTTTGTAAGGAGGTTGT
AGTGCCGCGTTATGCAAATTTTATTCCATTTATTTTATGTCCTACGTTTGTTTTAGGAATCTCTTTAACTTTA
TGAGTCATATACCCTTTTAAAAATAGTGAATTAATTTTTGTTTGTGGACTTGTTCAGTTTTTAGTTATTTCA
AGCATAAGAGTATATGGAACAATAGTTGCTGGATGATCTTCAAATTCAAAGTATGCTCTTTTGGGGGCTG
TGCGTAGTGTAGCACAAAGTATTTCTTACGAAGTGCCTATGAGTTTTATCATTTTGTGTTTTATTTATGGA
AGTAGGAGTTTTATACTTCAAGAAATTGGCATAATTATGTTTTTTTTTCCTTTTTTCCCATGTTGTGTGTTG
TGAGTTATTTGTATTTTAGCAGAAACTAATCGTGCTCCTTTTGATTTTGTTGAGGGTGAATCAGAATTAGT
TTCAGGGTTTAATGTTGAGTACAGAGGTGGAGCTTTTGCTATAATTTATATGGCTGAATACTCAAGAATAC
TGTTTAACAGAGTAATCAGAAGTGTTTTGTTTTTTGGGAGAAACGAAATTTTTATAAGAATTATATGTATA
TTTTTTGTTGTAGGTTTTGTGTGAGTGCGGGGCACTATACCACGAATACGCTATGATAAGCTTATGAAATT
GTGCTGGACTGTAATTTTGTGCGTGGCAATGTGCGTAGCGTGTGTGGTTATATGTTATAGATTTTTGTAAG
CTTTAGTTTGTTTATTAGACCTATAATAATTTTGAGCATTTGTTTGGTTTTTTTAGGTCCTACTATAAGGTTA
ACAAGTGATAATCTGTACGGTGTATGATTAGGTTTAGAATTAAATTTATTTGCTTTTATTATTATATAATAAATC
CTGAAGGATTTTGGATTGCAGAACCATGCATTAAATACTTTGTAGTTCAAGTTATTGGTTCTAGATTTGTA
TTAATTAGTGCAATAACTTTAAATTTTTATGCTTCTTATAGTTTGTTTTTAAGATTAATTGGTTTTTTTATTA
AAGCTGGTATTTTTTCCTTTTCATTCGTGGGTTCCTTCAGTGGTTAATTCGTCTGATTGGTTAGTTAGAGGT
ATAGTTTTAACTTGGCAAAAAATTGGTTCCTTTTATCTTTGTTGGTTTTATAAGAAACTATTATATTTTATTTG
TTGCTCTTGTAGCCATAGGAATTTTAGGTGGTGTTGGAGGTTTAAATCAACATAGTGTTCGCTCTATAAGT
GCTTATTCTTCTTTTGTTCATTCTTCGTGAATACTTGCTTCTTTTATAAGTTCGTTGATTACTTTTTTGTTTT
ATTTTCTTTTTTATTGTGTGTCTTTGTATATTTTTTTTTACGGGTGTTGTAAAGTAGGCAAGTCTTATGCTA
AGAATAAAACGGTAAGATTTCTGGGATGTTTAGGTATTTTAATGCTTAGAGGAATCCCTCCCATGTCAGG
GTTTTTCCCAAAAGTTGTTGTTTTTTTAAGCGTTGATAGTTTAGTTGTGTTGTGATGTGTAATTAGGTCTT
TTATTAGGTTGAAGTATTATTTGTCTTATTTTTATCTTATATTAATTTCTTCTTTTTATAATTTTAGTTTTCATT

TCTTTTTCATTGTTTTTTTTAACTTAATTAGCTTTGTCTATTTGTTGACTATTATTTAAcaaATGAGCCTTTTT
TTTGTTTCTTTTTTCATTGTCATTCTAATAGCTTTGTTGTGAGCCTTAATGTTTTTATCAGAAAAGTCGTTA
GTTAGACGTGAGAAAAGTTCTCCTTACGAATGTGGTTTTGAAGTTGTGATAAGAGCCCGTGCTCCGTTT
TCTTTGCGTTTTTTTATTTTGGCCGTACTCTTTGTTGTGTTTGATGTAGAAGTAGCATTAATAGTTCCTGTA
GTGTTTAGTATCAGTTTTGCTAAAAGAACTTTTGGATTAGTTAGTAGCGTTTTATTTTTTTTTGTTTTGTTT
ATTGGCTTATTTCATGAGTATCGTGAAGGTTCTCTTGATTGAGTTAGTTAAATTATTACAGGTGCGTGTGT
TTTACTAGTGTTTATTGTTATGTTAAGAGATAGTTTAACTATAGTATTATTGTTATTATGTTTAAGTGTTATG
TGTATTACAAGAGTTTATATGCAAAGTGAAGTTATTGACTACATAGGGATTGTATCATTAGATAGTACAAG
AACAAGATTAATTACATTATCTATTTTTGTAAGTGTGTTGTCGATGCTAAGGTCTGTAAATGTTGTGCGGG
CAAAACTTTTTAGTTTTATTATGAGTATAAGAGTTCTAAGCCTTGTTTTATCATTTAGAGTAGGTAATTTTT
TTTTATTTTTTTTTTTGCTTTGAAAGAACTTTAATGCCTATTTTATTTTTGATTGTTGGATGGCGCCTGCAAG
CAGGCAGATATATAGTTTTATATACGGTTTTTGGTTCTTACTTTTTTCTTTTTGGTATTAGATACTTAGCTTT
TAACGGATCAGGTTACATGTTTTTAACAGATAAATGTGGTAAAATTATTTCTATAGTATGAGTCATTTTTGT
AGTAGGATTTTTAGTTAAGTTACCTGTATATCCATTTCATTTATGGCTTCCCAAAGCTCACGTAGAGGCGC
CTGTTGCGGGGTCTATGCTTTTGGCAGGGGTCCTTCTTAAGCTTGGTGGTTATGGTTTAATTCGATTTTTT
TCCGTTGTTAGCTGTTCTTATAATATATTTCTTGATTTGTTTTAGTTGTAGGTCTTCTTGGTGGTGTGTACT
GTGGTTTAATATGTTTGCGTCAGGTAGATTTAAAATGTTTAGTTGCTTATTCTTCTGTTTCTCATATAAGGT
TAGTACTTCTTATTATGAGTAATACATTTGTTGGAGTGTTAGGAGCTGTTGTTATTATGATTGGCCATGGTT
TATGTTCATCTGGTCTTTTTAGAATAGTTAATCTATACTATTTAAATAGAAAATCTCGTCTGCTTAGAATAA
ATAAAGGTGGATTAATTATTTTTCCTTATCTTAGATTTATGTGTTTTTTGTTAAGTTCATCTAATATGGCGGC
ACCACCTAGTCTCAATCTTCTTGGGGAAATTTTAGTGTTTATTTCTTGTGGTTATGTGTCATTAATTTTTTT
GATTATGATTGGCCTTATTAGATTTTTTAGTGCGTGTTATAGGTTGTATGTTTATTGTTCTTGTAATCATGGA
AAAGTAGGTAATTATCCTACTAGATGATATAACTTTTTAGACTTTTTTGTTCTTTTTAGTCATTGGGTTCCT
TTGAATATACTATTTTTGTTTGTGCCTTAACGTCAGAAAGTTCATCTTATTGGTGTATTTGTGGGTTTAGAA
TTTATAAGTCTTGGTATTTTGTTTGCTGCTGGTTTATTTCTTAATAATGTTGTATGTTTAATTTTTTTGGTGT
TGTGTTTTGCTGTGTGTGAAGCTAGTATTTCGTTAGCTTTGATTGTCATGATGGTGCGTTTATGTGGTAAT
GACTTAGTTAGAAACTTAGTTTGTGATAAGAAATGTGTGTTAAGAAATTGTGGTTAATTTTGTGATGTTT
GGGTGCTTTATTTATTGTAATGTGTGGTAATAAAGATTTAATTGTTATAGAATTTGATTATCTTATTAGAGA
ATCTTTTGTTTTTAGATTTGGCTTAATTTTTGATAGATTATCTATTGGTTTTGTTGGCTGTATTATGTTGATTT
CTGGTAGCGTTTTTGTTTATAGAATTTGATACATAGACAATGAAGTGTTTTTTAAGCGATTTATTTTTTTGG
TTTATTTGTTTGTTTTGTCAATAATATTTATAATTATAACTTGTAACTTAGTTTCTTTGCTTATTGGTTGAGA
TGGGTTAGGACTTACTTCTTTTTTGTTAGTTTGTTATTATCAAAATAATAAATCTTTGTCTGGAGCTATGCT
TACGGCTTTAAGTAATCGTGTAGGTGATGTTTTTATTTTATTTAGTATTTGTTTTTTTATAAATGAGGGAGG
ATGATCTTTATATAGTTATAACAGAATGATATATATATATTTTTTTGTTTAATTATTTTATTTGCAGGTATAACTA
AAAGAGCACAGATGCCTTTTGTAGCCTGGTTGCCTGCTGCTATAGCGGCTCCTACTCCAGTTTCTTCTTT
GGTCCATTCATCAACTCTTGTTACAGCAGGTGTTTATTTAGTTCTTCGTAGTTATGGTTGTTTAGTTCATTC
GAGTTTTTTAATTTTAAAATTTCTTTCTATTATTACTTTAGTTTTAGCTGGTTCTAGTGCTTTGAGAGTTGT
AGATTTAAAAAAAGTAGTTGCTCTTTCTACGTTAAGTCAGTTAAGTGTTATAATATTTAGAATTTCTATTAT
ATTGCCTTTTATTTCTTTTTTTCATTTAGTTACTCATGCCGTATTTAAAGCTTTACTTTTTTTAAGTGCGGGG
TGTTTTATTCATAGACTATTAAATTGTCAGGATTATCGTATAATTGGTTCAGGTTGGAAATTAGTTCCTTTT
ACCAGGGTAGCAATAATTTGTGCTAACATATCTCTTTGTGGAGTTCCCTTTATAAGAGGATTTTTTTCTAA
AGATTTAATTATTGAAGTTATAGGCGGGGATTTTTTTATTTATTTTATAGAGTTGCTTGGGTTTTTTTTTAGC
AGTTGATACAGTATACGTATAATAAATGTTATTTTTGGTGAGAGTTTTTATTTTGTTAAGCATACAAAGTGG
AGTGAACCTTTTGAGTTGAAATTTTCTTATTTTTTTTTGTTATGTGGTGCTGTTATAGTTGGTGCTGTTATA
AAATCTAAAGTAGTTTGTTTTAACGAAATTGCTATAATTATAAATGTAGATTTTGTATTTTTTTTGTTTTTTT
GATTAGGATTAGCTTTTTTTATTAGATTATTTATTGTTTCTCATTTTTACATAAAAAAATGAATTTTTAGGGTC
TGTGTGATGAATAGAATTATCTCAGCCTTTTAGATATGGTGTTATGAACTTATCTGATTCTCTTGTTAAAGT
TCTTGATAAAGGATGATTAGAGTTTTTGGGTCCTCAGTATTTTGGGTCTTTGTTGTCAAAACACAATCAAT
TATTTTTTCTTGTAATAATAGTGATTTGTTGTTGTATAGTTATAAAAATTTACTATAAATGTTAATGTTTTTT
TGTTCTTTTTTCATCTCTGTTGTGTGTTGTGTTTGTTCTGTTCAACCTTTAGTTTTAGGTTTTTTTCTTATTA
TTAATTCTTTACTTGTAAGAGTAGTTGTTGGTGCAGTTACAAGAAGTTTATTAGGTTTTTTGTTTTTTATAA
CCTATGTTGGTGGGGTGATGGTGCTTTTTCTTTACGTTCTTAGTATTTACCCTAATGAAAAGATATTAATAA
GTTGACAAATGTTTGGATTGTTAATTATTTTTTTATTTTTCATTAATATAATAAGATATAGAGAAAAAGAAA
TGGTTTTTCATTATATAAGTGTTGCTGAATTATTTGTTTTTTTAGCAGTTGTTTTACTTTTTTGTATAATAAT
TGTAAGTTACGTATGTATAAAAAAACGTGTTCCATTTCGTAGGATT"/>
</data>
<data
id="Mytilidae27sq12PCGs"
spec="Alignment"
name="alignment">
<sequence id="seq_Arcuatula_senhousia11" spec="Sequence" taxon="Arcuatula_senhousia1"
totalcount="4"
value="ATGATAACTGATGTGTTTTCTGTTTTTGATGAACATTACATGAGAAGAAGATATGGCTACTATCTAT

GATTTACGTGTGTTGGGATTCCTTTAGTACTATTGTTTTGTGACTTAGTGCTTCGTGATAAACTGTTAGAA
GGCTCTTTCATACTTATGAAGAGCATTAACTGTTTTAAGTTAAGAGGGTTTCCTTTAGGTATTAGGAGGTT
ATTGATATTGATCCTTACTGTTAACGTATTTTCTCTATTTCCCTATGTTTTTAAGGTTAGTGCACATTTTTCA
TTTGGTAGGAGGTTCTCTTTGAGTATATGAAGTGCCATTATTATTTCTAGTGCTTTTTGAAGGTTTGAGCA
AACTTTTGCTATATTGGTACCGTATGATCCGGTGGTAATAGGCCCTTTTATGGTGTTAATTGAGTGTGTTTC
GCATCTTCTTCGTCCTATGTCTTTATTTATTCGATTAGCAATAAACTTAGCTACTGGAAAAGTTATAATAGT
AATAGCTACCTCTTTAGGTTTAAGCCTGCTTATCGTGGGGGAGCTTCTTGTAACATTCTTAGTTTTACTAG
GTAGAATAATGTGTTTTGAAGCTGGAAGGGGTGTTGCTCAGGCATTTATTTTTTGTTTTTTGTTATCATTAT
ATGCATCAGAGCATAGTGAGTAGGCTGGTGCAAGTTTAAGTCTTCTTATCCGAGTTCATTTATCGCATCCT
GGTAATTACTTTATTAAAAGAGAGTCATTTTATAATGTTGTTGTTACAACAGCTCTTATAATCTTTTTTGCT
GTAATGCCTTTGATTGGTGCCTTTGGTAATTGGCTGATTCCTTTGTGTATTGGAGGAGGTGACTTAGTGTT
CCCTCGTCTAAACAATCTGAGGTATTGGTTAGCTCCTAATGCTTTGTATTTATTAATGATTTCTGTTTTTAC
TGAAAAAGGGGCTGGTACAGGGTGGACAATCTATCCTCCTTTATCTTCTGTTGAATTTCATAGAGGTCCT
GCCGTAGACATCTTAATTACTTCATTACACGCAATTGGTCTTAGTTCATTAGTAGGGGCTATTAATTTTGG
GTGTACTAATAAAAACATACCTGTCCCTAAAATGAAAGGGGAAACGTCTGAGCTTTATTTATGAAGGCTA
ACTGTTACGGCTGTTCTTTTGATTATTTCTGTTCCTGTTTTAGCAGGAGGTATTACTATGCTATTGTTTGAC
CGTAATTTTAATAGAACTTTTTTTGACCCTATCGGAGGGGGTGATCCTGTTTTGTTTCAACATTTATTCTG
GTTTTTTGGTCACCCAGAGGTTTATATTTTAATTTTACCTGCTTTTGGTATTATATCTAAAGTAGTAATAAAT
CAAAATGGTAAAGAAGCTGTGTTTGGTCAAGTAGGGATGCTTTATGCTATAGTTGGTATTGGGGGCCTAG
GTTGTGTTGTTTGAGCTCACCATATATTTACTGTTGGGATAAATGTAGACTCACGTGCTTACTTCACTAGC
TTGACAATGGTTATTGCAGTACCAACGGGTGTAAAAGTATTTAGGTGAATAGCTACTATAAGTGGTGGTA
AGTTTAGGATATCCCCAAGAGGGTTTTGAAGTTTAGGGTTTTTATTCTTATTTACAGTAGGGGGTCTAACA
GGTGTTATGCTTTCTAGATCTTCTTTAGATGTCTGTTTACATGACACTTATTATGTGACGGCTCACTTCCAT
TATGTGTTGTCAATAGGAGCTGTTTTTGGTATTTTCTGTGGGTTAAATCATTGATTTCCTTTATTTTTTGGG
GTTAATTTGAATAAAAAAATGGTCATTGACACATTTTTTCATTATGTTTGTAGGTGTTAATATAACCTTTTTT
CCGCAACATTTTTTAGGGTTAAGAGGAATACCCCGTCGTTACTGTGATTATGCTGATTGCTACGCTAAGT
GGCACTGGTTTTCTTCTTATGGTGCTATAATTTCGTTTGGCTCTTTAATATACTTCTTGTTTATTGTTTGGG
AAGCAGTTGTCTGCTCTCGTGGGCTCGTTTTTTCTAGAAGAATAGCTAGGGATCTTGAGTGACAAAATC
AGGTATACCCTCCTTATTCGCATACTATGCCGTTGTGAGGTAGAAAAGGTTTTCAAGATTCTTATTATCAA
GTAGGTATAGACATTATAAAGTATCATGGTCTTGTAATAATGGTAATTGTTTTTATTTTAGGTTTAGTAGGT
TACATAGGTGTGTTTTTGCTTTGTCGTTCTTTAAGGTATCGTCATCATTCTCAGTGAGAAAAATTAGAGTA
TGGTTGAACAGTTTTGCCTATTATCCTATTAGCTTTACTGTGAGGCCCTTCTATAAAAAATTTATATCATAT
AGATGATATTAAAACTCCTCAGTGAACTTTTAAAGCAGTTGCCTCTCAGTGGTATTGAAACTATGAACAT
CAGCAGCAGTTCGAGTTTCAGTCTTATATAGCCGCAGATAGGGGAGCTGGTTATCGGTTGTTAGATGTAG
ATTGGCGGATAGTTGTACCTGCTCAATCTCAAGTCACAATGTATGTGGCTAGAACAGATGTTTTACACTC
TTTCTCGTTACCTTCTGTTTTGTTAAAAGTGGATGCTATTCCAGGACGTGTAAATCAACTTCCTTGTGTGT
TTGGCTTGCCAGGTGTTTATTATGGGCAGTGTTCTGAGATTTGTGGTATCAACCACTCTTTTATACCTATT
GTAATAGAAGTAATCCCTGCTAAAGTATTCATTGGTTGGTTAGAAGGTCTGACTAAC------------------------
ATGGCTCGTAATCCTTATTATGTTCCTGGTTTAAGTCCTTGGCCTTTTTTAGTTGCTCTAAATATTGGAAGA
CTTTGTTTAAGTCTTGTAATGTGAATACATCGAAGGGTTTTTATGATTTTATTACCTGTTTTAGGTGTTCTT
TTGTGTTTATTATGTTGGTGGCGTAATTTATTAGATGAAGTAGACTTAGGTTTCCACAATCGTTATGTGGTA
AAAACTTATCGCGATGGAGTAGCAATTTTTATTTTTAGTGAAGCTATAATGTTTTTCAGCCTTTTTTGAGC
TTTTCTCTATTCAAGGGAAAGGCCTTCAGCGAGATTGGATTTATTATGGCCTCCTTTAGGTGTTCGCTGTC
CTAAGCCTTTTAGGACGGCCCTATTCAGAACTGGCCTTTTAATCAGTAGAAGTTTTTATTGTGTTTGGGC
GCATAAAGCTATGTATAGTGATTATAGATGAGCCCCTATTATTGGTGTGGCTTATTCTGTTCTTTGTGGGGC
TGTCTTTTTGCGGTATCAATTTCATGAGTACTACATAAACTCTTTTTCAATAGCCGATAGGGTTTTTGGGA
GGTGTTTTTATATCTTGACAGGGTTTCATGGTTTCCATGTGGTTATTGGCAGTTTGTGATTATTGGTTAGTA
TATTTCGTTTAATTTTGGGGCATTTTAGTCGCAAGCGTCATTTTGGTCTTGTGGCCTGTTTTTGATATTGAC
ACTTTGTTGATGTTGTCTGGGTTTTTGTTTGACTTTTATTTTACTTAGTAATAGGTGGGatttttcgaaaacgtaatgga
ttagctaatgttctgacaggaagggtttatgacttgccttgtCCTATTAATTTAAGATGGTGATGAAGGTTTGGTTCTATATTAGGGC
TTTGTTTGACAATTCAATTGATTAGAGGCATCATTCTCTCTTTCCACTATAGAGCACATGAAGACCTTGCG
TTTGATTCAGTTATTCATATCGTTCGTAATGTAAAAAAAGGGTGGTTTTTACGGAGAGTACACACTAATG
GTGCGTCAATATTTTTTATTTGTCTATATGTACATATTGCTCGAGGCATTTATTATGGTTCTTATTTGGATTCT
GCTGTGTGAAATGTTGGTGTAATCCTTTATTTATTGGTTATAGGGGAAGCCTTTCTTGGTTATAGATTGCCT
TGGGGTCAGATGTCTTATTGGGGTGTAACTGTTATTACTAATATGGTTACGGTTATTCCTTTTGTAGGGAA
GGATATTTTGCAACATGTTTTAGGGGGTTACTCTGTGTGTAACAATACATTGAAGCGATTTTACTCTTTAC
ATTTTGTTATCCCTTTTGTAATTATGGGACTAGCAAGGCTTCATTTACTTCTCTTACATGAAAATGGAAGA
AATAATCCTTTGGGTGTTGAGAGAGATAGAATATTAGTTCCTTTTCATCCTTTTTACACGGTAAAAGATCT
GCTGGGGTTTTGTTGGTTTATGTGAGTTTTTGTTTATTTAGTTTGTGTTAATCCTGAGTTGTTAGGTAACC
CTGTTAATTTTATTCCTGCAGACCCTATAAAAACTCCTATTCATATAAAGCCAGAGTGGTATTTTATATTTG
CTTATGCTATTTTACGTTCTATCCCTCATAAAGCTGCAGGTGTTATTGCTATGTTAGCTTCTATTTTGGTTTT
ATTTCTACTCCCCTTGTTACATACAGGTAATTTTCAGGGGCTCGTTTTTTATCCTATTAACCAAGGGTTGTT
TTGGGTTTTTGTTAGGAGGTTTTTAAGGTTAACGTGAATTGGAAATAGCCCTGTTTGTGAGCCTTATGTA

ACCTTGGGGCGGGTGTACAGAGTTATTTACTTTAGATCTATCTTGTTAATCCCTTATAGCCTTAAGCTATG
GGATAAGCTTGTTTTTGCTGAGTTTGTAGTACTGATTTTACCTTTTGTATGTGTTTTGGTTTCGGTAGCTTT
TTATACTTTATTGGAGCGTAAAGTTTTAGGTTATATTATAAGTCGGAAAGGGCCAAATAAGGTGGGGTTTA
TAGGGGTCATTCAACCTTTTAGCGATGCCGCAAAATTATTTACTAAAGAAATAGTTATGCCTGGGTTTTCA
AACAACATGGTTTTTGTTCTATGCCCTGGCTTGATCTTACTTAATGGGTTGAGATTATGGCTTCTTTATCCT
TTTAGTTATACAGAGGTGGTATTTGTTTGTGGGTTAATTCAGTTTCTAGTGGTCTCAAGAACTAGTGTTTA
TGGGGTGATGCTAGCAGGCTGGTCTTCTAACTCTAAATATGCTTTATTAGGTTCCGTACGTGCTGTTGCGC
AAAGAGTTTCTTATGAAGTGCCTTTAACATTTGTGATAATTATTATTTGTTGCTTGTTAGGAAGAATGCTC
TGTCAAGAAGTGAAAGAATTTTTTAGTTCTATTTTATTTGGTTTAGGCGGAATAATTTGGTTAGTGTGTAT
ATTGGCTGAATCTAATCGTGCTCCTTTTGATTTTGTTGAAGGTGAATCTGAGTTAGTTTCTGGTTTTAATG
TGGAGTATAGAAGAGGAGGCTTTGCGATAGTTTTTATAGCAGAGTATGCAGCAATACTGTTTAATAGTCT
TTTTTTTACCGTTTTATTTGTTGGGGGCAGAGGGGCAGTGGTTATATTGGGGATAACGTTAGTTGTAGTGG
GTTATGTATGAGTTCGTGGGTCTTTTCCTCGTATGCGGTATGATAAAATAATAAAACTGTGTTGACGTTAT
TTAACGGTTTTAGTCCTCTGTTTAGGTGTTTTTTGTCTGTGTGTCCCTTACTTATAATATTGGTAGTTATGT
TTAGTCCTTTAATGTTGTTTTGTTCATGCCTGGTGGTAGGAGGTAGTTTGCTTAGTATCAGAAGAAGAAC
ATGAAGGGGTGTCTGAGTGGGGATAGAGCTAAATCTTTTCTCATTTTTAATTTTGATAAATGGAGGAAGC
TTTTTCGATTTAGAACCTTTGATTAAGTATTTTGTAGTCCAAAGGTTAGGTTCAGTTGTGTTTATTTTTAGT
GTAGTTTATGTCTCTTTTTTTAGAAACGTGATAATAATCCTTCTAGTATTAGGTCTATTTTTAAAGATTGGG
ATTTTTCCTTTTCATAGATGGGTTCCCAGGGTTGTTGTAAAAAGCCGTTGAATTGTAGGTGGGTTGGTGT
TAACGTGACAAAAACTGGCGCCTTTGGTTtttTTTTTGATGTTAATTAGAAGTAGTGTAGTTTTTATTAGGG
TTTTGTTTATAGTAGTTATTGGCGGCGCTGGCGGTCTAAACCAAAGCAGGGTGCGGGGTATAGCTAGCTA
CTCTTCTTTCGTTCACATATCTTGAATAATGGTAGGCCTTCTCTACTCATTTTTTGTGTTTTTGTTTTATTTT
TTTATTTACTCTATATCTCTCTTTTTATTTTTTCTAGGCTGTAGCAATAGTGGTAAAAGCTCTTTAGGGAGT
CAAAGGTTTAGTTTACTAGGTTTAATTGGGGTTCTTATAATAATAGGGGGTTCCTCCTTTTCTGGGCTTTTTA
AGGAAATTATTAGTAATGTTATCTAGGCCTACTTTCGCTTTGGTTGTTTGTCTATTAGGTTCTGTTGTAAGG
CTTAAGTTTTATACTTCTTTCTTTTATAGTATGTTTTTAAACGGTTTGTATGGAATTGAATCTGTAGTGTCG
CTTAGCTTAGTATTAAATGTTTTAGGATTATTTTTAATTGTGGTAGTGCTTTTTTTGGTTTTTTTATGTTGCga
gatggttTTTTTGTTAATGCTTAGATTTAGGCTACTGCCGTTGTTTATACTCTCCTCTAAATCTGATTATAGGCG
TGAAAAGCTCTCTCCTTATGAGTGTGGGTTTGAGCCTGTATTCAGTGCTCGAACTAGGTTTTCTGTTCGG
TTTTTTTTAGTCGCTGTCTTATTTGTGGTGTTTGATGTTGAACTGTCTATCTTAGTAGCAACAATTTTTTCT
ATCAGGCTTATAAAAAACTTAATCAGACTAGTGAGTGTTATTGTTTTTCTAGTTGTTCTTTTTTTAGGGTT
GTTTCATGAGTTTCGTGAGGTTCGTTAAATTGAATTTACTtAaGTGGTATTGTGGTTATTAAGTATATTTCTG
GTGTTAATAGTAGTATTGCAACGCGAGTTAGTGGTTAGGGCACTACTAATAAGGTTTTATTTGGCACTCTA
TCTCTGTGTAGTACCAGATAAAGTGTGGATACTTCATGGTGTTATAGTCTGAGATTTTTTGAGTTTAAGAA
TATCTCTTTTAGGGGTATTTGTAGTGGCAATAGGGGTATTAAGAACAAAGGTGAGCGTGCGGAACAAGC
TATTTCTATTGGTAAATATGTTTTTATGTATAGTTGTAGTAATAGCGTTTAGCGTAAGAAGTTTCTTTCTTTT
TTTTTTTTTTTTCGAGAGCTCTCTGATTCCTTTAATTTTAATAATCGTAGGCTGGCGTTTACAAGCTGGGG
TGTACATACTAATTTATACTGTAGTAGGTTCTCTATTTTTTCTTTTAGGGGTTTGTTTTATATATTTTAATAGA
AGAGACTGTATAATCTTAAGAAGGTCATGTGTTAAAAATATATGGAGGTTTTGATGAGTATTCTTGTTTGG
GTTTTTGGTTAAAATACCTTCTTATCCCTTTCATTTATGACTTCTAAAAGCACATGTGGAGGCACCTGTAG
CTGGTTCTATAGTGTTAGCAGGGGTTATTCTTAAATTTGGGGGGTATGGCATAATCCGATTGTTTAGGGTT
ATTAATGTCTGTTATTACGATAGGTTATGTCTGATTGTAGTCGTAAGACTATTAGGGGGGTTTTACAGAAG
CGTTGTTTGTGTTCGTCAGACTGATTTAAAACGCCTGGTGGCTTACTCCTCTGTTAGACATATAAGAATA
GTAATTCTATGTTTTAGAAATTCTTTTCTAGGGTTAGAAGGGGTTATTTGGCTAATGTTAGGGCATGGCTT
GTGTTCTTCTGGGTTATTCATGTTAGTGTCAGTAGTTTACTCTAGGAGGGAGTCTCGAGTTCTTCTTCTTA
ATAAAGGAGGGTTAGTTAAAGCACCCTGTTTAGCAATAGTTAGATTTCTTCTTTGTGTTAGAAATATGGC
GGCGCCCCCTAGGTTAAACCTGTTGGCAGAGATTTGCTTATATATTTGCATCAGTTCTATGTATCACTATAT
GGTGCTGTTCTTTGCTATAGTTAGGTTTTTTAGGGCTGCCTATAGGCTTTATTTGTATACCAGTTGTTTTCA
TGGTAGCCTGAGAGGAGGTCTATTTAGAGTTCTAGGGCATAGAGATACTATCGTTTTAATATGTCACTGA
ATCCCCTTAAATATGTTGGTTATTATATTATATTAGATACATAAGTACTTTTTATTGAGATTGTTTATTAGTAT
AGAGGTTGTAATCTTAGGCCTTGTTGTTTTATGTGTTGCTGTAAATTTTTTTTATGTAGCTTCATTAAAATT
AGTGCTGTGTCTTGCAGTGTGTGATGCAGGGGTATTTTTGGCGTTAATTGTTTCACTAATACGGCGTGTT
GGAAGAGACAGTGTTAGAAGAATATCTTCTTTTAAtttaaaATATTGAATCATTTTATTTTTTTTGCGCTCATT
ATTGGGTATACTTTTATTTTAGTTGGAAGAGTCTCTAGCGTTTATTTAATTGAGATAGTGTTTTGAGAGTC
AGGAAGAGTAAGGATAGTGTTTAGGGTCTTAGTGGATTATATAAGGCTAGTTTTTATTGGGACTGTAATA
GTAATTGGTGGAAGAGTTTTAATTTACACAAGATGGTACATATCAGATGAGCTTTATTACAAACGATTTGT
TAGTTTAGTGTTACTATTTATTCTTTCAATAGTGTTAATAATCCTTATCCCTAACTTAATTGGGATTTTAATC
GGATGAGATGGTTTGGGTTTAACATCCTTTTTATTGGTGTGTTATTATTGTAACAGAAAATCGCTCGCTGC
TAGACTACTGACGGCCCTTACTAATCGGATTGGCGATGTGCTGATTTTAATGAGAATTGGTTTTATAATTG
TGGAGAACTCCTGGTCTTTATATCAATTTACTTTTGGTGAGACAATTTCGTTAGTGGGCTTACTGACTTTT
GCTGCAATAACTAAGAGTGCACAGATGCCTTTTTGTGCTTGACTACCTGCTGCTATAGCGGCTCCAACGC
CTGTTTCTGCATTAGTACATTCTTCTACTTTAGTTACGGCAGGCGTGTATTTACTTATTCGTTCGTTTAAGG
TAATCTCACTTGACTTTATAGAAGTGTTAGAAGTCTTAAGGCTGGTTACTCTTTGTTTGGCTGGGACTAG

TGCGTTAGTGTGTTTGGATCTCAAAAAAGTAGTGGCATTATCTACCTTAAGTCAGCTGAGCGTTATAATA
CTAAGAATTTCCTTGTATGCGCCTGTTCTTGCCTTTTTTCATTTAATTACTCATGCGTTATTTAAGGCACTC
CTTTTTTTATCGGTAGGGTCTGTAATCCACTCTTATGGTAATATCCAAGATATCCGAATAGTTGGAGGCTG
TTGGAGTACATTACCCAAAAGAATAAGGGCTATAGTAATCGCTGTTAGTTCTCTATCGGGGCTACCTTTTT
TGAGTGGCTTCTTCTCTAAAGATCTGATTGTAGATTGCTATAGGAGTAGCAGTCTAATAGTGGTCTTAGTA
GGGGTTGGAATTGCTATAACAAGAGTCTATAGTCTACGAATTTGAAGAAGTTTGTTTAGATTAAATATAG
GCGTGAGTCCTGTTATAAGAAGAGATGAAAAATTAGAACTCACTTTTCCTTATCTGTGTTTAAGGTTAGG
AGCTCTATTTGTGGGCTATGTATTAAGAGGGCAAATTATAGGCGTTTCTCAGTTTAGAGGTTCCAGAAATT
TTGAGATTGTGGTACTAGTGGGCGCATTTTTGGGTTTATTTATATGGGCAAACAGGGGAAGGTTACAATT
TAATCTTAAAAAATTTCCTTTTTTGTTAAGAATATGGTGCTTAGAAGTTAGACAAGTTTTGGCAGGTGTTA
GGCTTTCATTGTCAGAAAAACTGTCATCGAGCTTAGACAATGGGTGGTTAGAGACAATTGGCCCACAAA
GAAACTTAATGAGTGTATCACAAAATAATGAGGGGTTCTTTATGAGTTGCTTAGTGGTGAGTTGTGTTTT
GGTTTATTCTTTTTTGTAGCGttggagttgttgttagtttcagccgttttgtttttttttATAATTATTTCTTTTAGTTTCCCTTATGCT
TTAGCCATATTTCTTATTATTCTTTCGTTATCTATTTGTTTTTTAGTAAGATGATTTTATAGAAGGTTAATAG
GGTTGCTGATTTTCATAATTTATGTAGGGGGTGTGTTAATCATATTTCTTTACAGCCTTAGAGTGCTACCTA
ATGAAAGATTTTACTCAGACTATGGGTTCTTCTACTTTTTGTGTTGGGTGCTTTTTGTTATGTCTATTTTTA
ATTATGAAATGTCCTTTCATTATATAAGGTTAGTCAGTTTTGGGGGTATATTTGTCTTTATGGCGTTAGTTTT
ATTTTTCCTTATACTGGTAGTTTGTAACTTATGTGATAAAAAGCGTATTCCATTACGGAAGCTT"/>
<sequence id="seq_Bathymodiolus_childressiNC0597071" spec="Sequence"
taxon="Bathymodiolus_childressiNC059707" totalcount="4"
value="GTGCTATTTGATTTGCTATCTGGTTTTGACtctttTCTTATAATATAGGATGGTCATCTTTTTTGCGTatc
TTAAGTTTCTTCTTTATTTTGATCTTGGTTAGTACTTTGTTTTTACTTTATGCCAAGGGAGCAGCTGTTAGT
TGGAGTGTGTTTAAAACTTCAAACGGTATGAATTTATCAGGATTCCCACTAGTTGTTTCTTCTCTCTTCAT
TTTTCTAATGAGGGTAAATATAATGGGTTTGGTACCTTTTTCTTTCAGTGTAACGTCCCATTTAAGTTTGG
GCCCAAACTTAGCCTTTTTAATGTGGGGCTCTTTATGAGTCTCAGGCCATCGAGTTAGATCTAAGCAAAA
TTTAACTAGACTGGTGCCAAGGTTTCCAATGTTTTTAGTTCCTTTTTTGCTATTAGTTGAAATTGTCACCA
TTAGTTCTCGCCCGATTACTCTTGGTTTTCGATTAATAATTAATATTATTGCGGGGCATTTAATCTTGTCTAT
GGGAACTAATGTCAATAGGTTTATTTCTTTAGGCCCTTTTTATGGGGCAGTTTACTTTTCTTTATTCGCAAT
AGGGCTGGCGGCGGAATTAGCCGTAGGTTTAATTCAAGCTTTCATTTTTTGCTCTTTATTGAGTCTTTATA
CTAATGACCACGCCAATTAATGATCGGGAATAATTGGTACAAGTTTGAGGATGCTGATTCGTATTGAGCT
AGCTCGTCCTGGTAGCggaTTTTTAGGTGATGACCAGCTTTATAATGTTGTAACTCATGCATTGGTTATAAT
TTTTTTTATAATGCCTTTGATGGTGGGCGGTTTCGGAAATTGACTACTTCCTTTAATGATAGGTTCAATTG
ACATAATTTTTCCTCGTTTAAATAATCTAAGGTTTTGGTTTTTGCCAGCATCTCTTTTTACTCTTTTGTTGT
CTACATTTATTGAGAGAGGCGCTGGGACTGGTTGAACCCTGTATCCTCCGCTATCTTCTTATACTGGGCA
CAGTGGTCCAGCTGTGGACATGTCTTTATTTTCTTTACATTTAGCAGGTGCTTCCTCTATTGGTGGTTCAA
TTAATTTTTTGACAAGGATGAAGAATATGTCTGTTGAAAGAATGCGAGGGGAGCGGATAGTTCTATTTGT
TTGATCCATGGCTGTGACAGCAGTCTTATTATTAGTTTCTTTACCTGTATTAGCAGGAGGAATCACCATGT
TGATTTTTGACCGCCATTTCAATACTTCTTTTTATGACCCTAGAGGTGGTGGAGATCCAGTTTTATACCAG
CATCTGTTTTGGTTTTTTGGGCATCCAGAAGTTTACGTTCTTATCTTACCAGGGTTTGGAATAGTATCTCA
TGTAGTTGCTCATTGTGCAGGAAAGGATGAAGTGTTTGGTGTGTTAGGAATAGTTTATGCTATGGTTTGT
ATCGGTGTTCTGGGGTTTATTGTTTGAGGGCACCATATATTTACTGTAGGAATAGATGTAGACTCTCGAGC
TTATTTTACTTCAGCTACAATAATTATTGCTGTTCCAACTGGAGTCAAAGTGTTTAGTTGACTGGCTACTT
TAAACGGTGGAAATTTATTGCATGAGCCAGCACTTTATTGGGCTGTTGGTTTCATTTTTCTCTTTACTGTT
GGGGGGCTTACTGGTATTATGTTATCAAATTCTTCTCTTGATGTGGCGTTGCATGATACTTATTATGTTACT
GCTCATTTTCATTATGTTCTCTCTATAGGGGCAGTATTTGCTTTATTTTGTGGTTTCTTTCATTGATTTCCTC
TATTCTATGGGTACTGTTATCATGAACGTTGAAGTAAGGCCCACTTTTTTATCATGTTCATCGGTGTAAATA
TTACTTTTTTTCCTCAACATTTTTTAGGTCTAAGCGGAATGCCCCGCCGCTATTCTGATTATCCAGATTGTT
TTATAAAATGGCATGTAGTGTCCTCTTTAGGGTCATGGTTGAGATTTGTTAGTGTTTTATACTTTCTTTTTA
TTGTGTGGGAAGCTTTGGTAAGACAACGAGGTGTTGTGTGCAAAAGAAATCGACCCGGGGCTATTGAA
TGAAGGGAATGGTGTTGTCCTTTGATGCCATTATGAGGTAGATTTAGGTTTCAAGATTGTTATTATCATATT
GGGGAGTATCTTGGTTTGTTTCATGAGGGTGTGATGTGCCTTATGATTTTTATTTTGTCTGTTGTGTTGTAT
GGAGTAGGTTGGGTTTTCACCTCGGGAAGAAGGTTTCGTTATTTACGTGAGGCACAAGCAGTAGAAAC
AGCATGAACTATTATTCCTAGGGTGTGTTTAGTCTGTGTGGCCATCCCTTCTATACATTTATTGTATGTAAT
AGATGAAATTGGTAGCCCTAAGTTCTGTTTTAAAGCAATTGGTCATCAGTGATTTTGGTCTTATGAAATG
GAGGACGTACTAGGATTTGATTCTTTTATGGAGCGGGAAATAGACAGTGGTTATCGATTATTGGATGTAG
ACCAACGGATGGTTGCGCCGGCTAATACCGGAATTCGGTGTATGGTAAGAAGGGCGGATGTTATTCATTC
CTTTGCCCTCCCGGGCTGCATACTAAAAGTAGACGCGATTCCAGGCCGTGTTAACGAAGTTCCTATGACT
GTAAACATGTGCGGGGTTCTTTATGGCCAGTGCTCTGAGATTTGTGGTGCAAACCATAGGTTTATGCCCA
TTGTGATTGAGTTTATTCATCCGCGGGTGTATAATTTATGGGTGGAATCTTTTGACATC-----------------------
ATGCCACGTAATGCTTTTTATTTGGTTGGGCCAAGTCCGTGGCCTGTTTTTACTTCTATAGGAGCTTTTTG
TATGGCAGTAGGTTTTGTTTCTTGGTTCCATAAGCATGGTTACGCCCCTTTTATTAGGTGTAGTTGTGTTAAT
ACTTTCTTTAAGACAGTGGTGACGTGATGTTATGCGTGAGGGTGATTTAGGTTTTCACACTTCATATGTT
GTTAAAGGGTTGCGGGACGGATTTATTCTTTTTTTGGTTTCAGAGATTATATTTTTCTTTTCTTTATTTTGA

GCTTTTTCCATATAAGTTTGGCTCCTGACATTTCAATGGGATGCATGTGACCTCCTAAGGGTTTGGAAA
CCTTAGACCCTATAAAGGTTCCATTATGCGGAACAACGGTGTTAGTTGGTTCTGGTGCATCTCTTATGTAT
TCTCACGCTGCGATTCGTGCAGGTTTAAATACTCATGCAGTTTTAGGGACTTTTTATACTATTGTGCTAGG
CTTGATTTTTACACGATTACAAGGATACGAATACTATTGAGCTAGGTTTACTATTGCTGACAGTGTTTATG
GGAGTTTGTTTTATATTATGACTGGGTTTCATGGCCTTCATGTTATATTCGGGACAGGCTTTTTACTTGTAA
GCTTAGTGCGTTTAATGCGTAATCGGTTCACCCCCCGCAACCATTTTGGGTTTATGGTGTGTTCTTGATAT
TGACATTTTGTTGATGTTGTTTGAATTGGCTTGTATTTAGTTGTTTATTGCTGGGGTAGTTAACCCTTACGT
AAGCAGGATAGAGTAATAAAAATTTTAAATAATAGGTTGTATGATTTACCCGCGCCTGTCAATTTAAGGG
TCTGATGGAATTTTGGGTCTTTGTTGGGTTTATGCCTGGTTATTCAAATTGTTACAGGTTTTATCTTAAGCC
TCCATTATACGGCCCACACAAATATGTCATTTGATGCGCTTATTCATGTGATTCGGGATGTGAACAATGGT
TGGCTAATTCGTGGTATACATGCTAATGGTGCATCTCTTTTTTTTGTTTGTGCTTACTTTCATATTGGCCGT
GGCTTGTATTATGGGTCTTACAAGTCCCGGATGGTATGAAATGTGGGGGTAATTTTGCTTTTCCTTCTCAT
GGCTACTGCTTTTTTAGGTTATGTTCTTCCTTGAGGGCAGATGTCTTATTGAGGGGCAACTGTTATTACAA
AATTAGTAACTGCCGTGCCTTATGTCGGTGACATAATTTTGTATTGAATTTGGGGTGGTTATACAGTTTGT
AACGCTACTTTGGTGCGATTTTATTCTTTTCATTTTATTCTCCCATTTATTATAATTGTTTTTAGTGTTCTTC
ATTTGTTTTACTTGCATGAAGAAGGGGCTAACAACCCGTTGGGGGTCAGTGCAGATGCGGTTTTGGTGC
GATTTCATCCTTTCTACACATATAAGGATGCAGTAGGTTTCTTCATTTTATTTTTTGGTCTTTTACTATTAGT
TTGTTATTTTCCGGACCTTTTAGGAAATGTAAACAATTGGATTCCCGCGGACTCAATAAAGACCCCCCTT
CAAATTGAACCAGAGTGGTATTTTTTATTTGCTTATTCTATTCTTCGTTCAATTCCCAATAAAGTTGGGGG
GGCAACCGCCTTGGTGGTATCAGTTTTAATTTTATTTCTTATTCCTAGGCTGCACACAGGTCAGTTTCGTT
CCAACAGGTTCTATCCTTTAGGCCAACTTTTCTTTTGGGCTTTAGTTGCAGCTTGAGTGGGCTTAACATG
AGTTGGGGCTTGTCCGGTACAGCACCCCTATGATTCTTTGGGCTGCTGTTTTACTTTCCTTTATTTCTTTT
TTATTGTACTGATTCCTATAAGTCAAGGTGTGTGGGACGTGTTAATTAAAATGCAAGTTATCAGCTTTATT
TTACCAGGGGTTTTTGGTTTATTAGCAGTAGGGTGATTTACATTAGTTGAGCGTAAGGTGTTGGGTTATAT
TATGACTCGTAAAGGCCCCAATAAAGTGGGGTTTTTAGGGCTAATACAGCCAATAAGTGATGGGGCTAA
ACTATTTTCTAAAGAAATGCTTGTGCCAATATATAGGAATTTTGTCCCATTCCTGGTGTGCCCTGTGGTGA
CGTTTTTTATTGCCTTGTTGCTGTGATTGCTATATCCATTTCACTCTTCAGAGGGTGTGTTTACGTGTGGG
GTATTATTCTATCTGGCAAATTCTGGGACTACTGTTTATGGGGTGATGGTGGCAGGATGATCATCAAATTC
AAAATATGCCCTTTTAGGGACCATGCGGGCTATGGCACAAAGTATTTCATACGAAGTGAGAATAGCATTA
GTGCTGTTAAGATGTGTTTTCATGTCGGGGTCAATGCATCTTCAATCCGTGAAGTTGTTGTTTATCATGGG
TGCAATTTTACCTTTTTTTGTGGTGTGATTAATTTCTATGTTAGCAGAAACTAATCGTGCGCCATTTGATTT
TGTAGAGGGCGAATCAGAGCTGGTTTCAGGTTTTAACGTGGAGTATAGTAGTGGGGGTTTTGCCCTTATT
TACATAGCAGAGTACTCTAATATGTTGTTTAACAGCTTGTTCACATGTGTGATATTTTTGGGTACAAGGGA
CGCAATAATGGTTGGACAGGCCTGAATTTTTCTTTTTTTTTTCTTGTGAGCTCGTGGGACTTTTCCTCGTT
TTCGATATGACATACTAATAGGATTGGCTTGAAAGACTTTTTTATGTATTGTTTTAGGGTTGAGTTTATTTA
CTACTATAATAATTTATACTTTataATTAGGCGGTTTGAGGGCCCTTTAAGAGTTTTGGGGGGTTTTACTTTA
ATTTCTGGTTTGATCATAGTAGTCTCAACTGATAGGCACCTAGCTGCATGGGTTGGGATAGAAGTAAATAT
GCTTGGGTTTATGTGCCTTTTGAGTGTTAAATCCGTGTTAAACATGCGTGTCTTGATTAATTATTTTGTTTT
TCAGAGACTTGGCTCGACTATATATTTGTTTGGGTCAAGTGTTATTTTGTTTAATATTGCTTTAGGGTATTT
TTTAATTCATCTGGGACTTCTTTGCAAAGCTGGTCTTTTTCCTTTTTGGGTGTGGGTTCCTTCTGTAGTCA
ACTCTAGGGGCTGATTTGTTAGCTATCTATTATTAGGGATTCAAAAAGTTGGGCCTTTATTTTTGGGTGTG
TGGTCCCCTTGCAGAGAGTTTTTATATTTTGTAATTTTTGCTATAAGGGCTTTGGGCGGTTTAGGCGGGTG
TATCCAGAATTCTTATCGTGATGTATTGGTGTATTCTTCTTTTGTCCATAGTGCGTGAATGCTGGTATGTTT
AATTGAGTCTCAGAATTTATTTCTTTTTTATGTTGGAGCTTATCTTGTACAATTAGGTTTAGTTGTTTACTA
CTTGTGGCGCAGAAATGCAAGCTCTGTAAAGAGAGGGAAATGATCCCTTATAGTAGCAAGGTGTTTAAT
AAGCTTGAGGGGGTTGCCCCCATTAGCCGGGTTTGTGGTGAAGATAACTGTTGTTTTTTGCGTTGACAA
ATTAATTTTGTTATTTACATTAGCTGGTTCTATTACGGCCATATTTTATTATTTGAAAGCTGCAAATAGCTCC
ATTGTTAAATTATTAAATGATGGGGGCGTTTTTCTCTCTTTTACTTTTGGTTTGTATGCTTGAATTTATTTAT
TGGGGTGCTTGCTTTTGTAATAATCTGTATCTATCCTTTGGTTGGTTGTGATGATGGGTGTTTGTtttatgttatcta
taatgCTCATGTTATCTATGACGGGGCAGTGGAGTCGTGAAAAGGTTTCCCCCTATGAGTGCGGGTTTGATC
CGATTCTTAGGGCCCGTAGGAGGTTTTCTTTGCGCTTTTTTCTGCTTGGCGTTTTGTTTTTAGTGTTTGAT
GTGGAAGTGGTTATGGTTGTTCCACTTTTGTTTGTATTGTACGGGGGGGTGAAGGTAGTGGGCCTTGTGT
GTTTAGTAGGGTTCTTACATGTTCTTACTATTGGGTGCCTCTATGAGCGTCGCGACGGGTCGATGGATTG
GATTAGTGAGGTGGTTATTGGTCTTGTTATATGTATACTTTTTTTAGGCTTGCTAGGTAGCTTAGAGTTATC
AATTGCTGGCTTGTCTTTACTAGGTGCAATAgcgATGTTATCTTTATTTTTATGCGCTCAATACTCTGAGTTA
ATTGGATTTTTTAATTTGGACTTTCCAGGGCAGGCCTTAGTAGTTCTTAGAATTTATATTAGATTTTTAATG
GTGTTGGGCAGGGCCGCGGTTTCCCGTTTTAGTGCATTTAATAGACTTATTACTTGTATCGGTGTTTTGTT
AGTTTGTGCTTTTAGTGTGAGGGCCACTTTCTTCTTCTTCGTTTTTTTTGAGGGGGTTTTGTTTCCTACTT
TAATGTTGATTGTAGGATGGCGGTTACAAGCAGTTGTTTATATAGTTATTTATACTGTTATAGGGTCACTCC
CACTTTTGTACGGGTTTGGGAAGTTATACTTTAATTACAGTGATAATTTATTTTTGGAGTACTTTTTGGATA
AAAGAATTTTGAGTTTTAGTTGGCTTTATTTGTTAGCGTTTTTAGTTAAGCTTCCAGTTTTCCCCTTGCAT
TTATGATTGCCTAAGGCTCATGTAGAGGCTCCAGTTGCCGGTTCTATAATTTTAGCAGGCCTTTTGCTTAA
GTTAGGGGGTTACGGGATTATTCGTTTAAGAGGCCTTCTTGTTATCAAAAGAATTAGAATAGCTATACTGG

TAGTAATAGTTAGGCTTTTTGGAGGGATTCTTACAAGTATGGTTTGTTTACGTCAGACAGATTTTAAATCC
TTAGTGGCTTATTCCTCTGTTGGACATATAAGTTTTGTTTTAGTCTTATTGAGTAATACTTTATGGGGGCTT
ATAGGGGCGATGTTGGTTATAATCGGGCATGGGCTGTGCTCTTCTGGTTTATTTTCTTTGGTTAATGTTTTT
TATCTTAGAAGTCGCTCTCGTTTGTTGGCAATAAATAAGGGATTTTTAATACTTAGCCCTTGTGTCGCTTT
GTTGTGTTTTCTGTTGAGGGTCAGAAATATAGCCAGCCCACCTAGTTTGAACTTATTTGGAGAACTTTTT
ATTTATATAGCGGGTGCTTCTATAAATATACTGTTTTTTCCTTTATTAATGTTGTTGAGATTTTTGAGCGCTT
GCTATAGGCTTTATATTTATATCAGCTCCCAACACGGCAAATGTATAAATAGACTAGACTCACGAATAAGT
ATAATAGACATTTCGGTGTTGACTTTTCATTGAATGCCTTTAAATTTTTTGTTTGTAGTTATTCCATAGGTG
CGCAGAATACATTTGATTACGATTTTCTTAGGGATGGAGTTTAGAGCGCTTGGCCTGTTTATCGTTGGGTC
TTTATCGGCGTACAATAGTCTGTTTATTCTACTACTTGTAATTTGTACTAGGGTGTCTGAAGCAGCTATTAT
GCTGTCTTTGATGGTTATAATGACTCGTTTGTACGGAAATGATCGATGTTTGAGTTTGATGACTGATAATT
TAAAGTTAATGAAGTTTTAGGTTGTATTTTTATGGTTTTAGGATGGGGTGTAATAAGTATAATCCCCTTTAG
CTCTTTAGTTCTTGTTGACTATAGAATTTGATTATCTAGAAATTTGTCGGTGAATTTAACATTGCTGTTAGA
TCAAGTTAGAGGGTTGTTCATAGCTACGATTTTTTTGATTTCGGGGTCTGTTTTAATTTACTGTTCTTGGT
ATATGGCCGATGAAATTTATTTTTTGCGTTTTATTTATCTTGTTGTATTTTTCGTTCTATCTATGATATCCCTA
ATTATAATTCCTAATTTAATTGCTCTTCTGCTGGGATGAGATGGGCTTGGTATTACCTCTTTTTTATTGGTG
GTTTATTACCAAAATAATAAGTCTTTGGGGGCAGGCATAGTCACAGCTTTGACTAATCGAGTTGGAGATG
CAATCCTTTTATGTATCATTGGCGTATTTGCTAGGGAGGGAGGATGAATCCTATTTGAGTTTTTACCTAGA
ATAAGGTATTTTGTCCCTTTTCTTTTAGTTATAGCTGCTGTTACTAAAAGGGCCCAAGTGCCCTATTCGGC
GTGGTTGCCTGCTGCAATAGCTGCCCCCACCCCTGTTTCTGCACTAGTTCATTCCTCTACTTTGGTCACA
GCTGGGGTTTACCTTTTGATTCGTTCTTACCCCCTTCTTTCTCAAAGATCTTTAAGGGTGTTAAAGTGTCT
TAGACTTTTCACTTTACTAATGGCTGGGAGGGTGGCCCTTGTTGAGGTGGATTTAAAAAAGATTGTAGCA
TTATCTACTTTAAGCCAATTGAGAATAATATTATTCGCTATTTCAATTTGTTTACCAAAAGTGGCTTTTTTC
CATCTTATCACTCATGCTATGTTTAAATCTCTTTTGTTTTTAAGTACCGGGGTTGTGATTCACACAAGGTT
AAAATGGCAGGATATTCGCTTTTTAGGGAAAAACTGAGCTCGTTTGCCCGTTAGAATGAGTTGTGTCAC
TGCCGCGAGTCTTTCTTTGTGCGGGATGCCTTTTTTGAGAGGGTTCTATTCAAAAGATTTGATTATTGAAT
TTCAAGGCGGGGATTTGGTTATTTATTTTATACTAATTGTTGGAACTTTATTTACCTCTTGGTACTCCCTTC
GTATATTTAATTTGTTTTTGAGTAGAAATAAGAGTGTTAGTCCTGTGGTTCACGCAGAAGAAGCCAGGAG
TGTTAAATTTGCTTACAGGGGCTTATTGATTAGCTCTGTGATTATAGGGTTTTTATTAGTTAACTTAGTCTC
AGACCTTGATCTCACGGCTATGGTGAGTAATGTGGAAAAGTTTGTTGTAATATTACTAGTGGTGGCTATTA
TCATTGAGGTTTTTGCAGGATGAAAACGGAAAACCAAAGCGGGTTATGGCTATTTGGCTAGTATGTGGC
ATTTTAAACTGTTACAATTTAGCCCTAGTGCGGGTTTAAAGCTAGCTAGTCTTATTACAGTTAGAATGGAG
AAAGGATGGTTAGAAAAAGTTGGCCCCAAGGAAGAGTTCAAACGTTGGGTTTAATTAATCAGTACCAT
TTTTTATTTGTGTTGCTCTTTAGGTTATTTATTGTAATTATTTTGTTCTTGTAATTGTTAGAAACATTCTTCT
TTTTCATTTCCTGTTTTGTATGCATGTTGAGGATCGGGGAGCCTTTGTTTATGGGTTTGGCTTTACTTGTT
GTATCCCTTATTATTTCAATTTTGTTGGGGTTAGAAATAGGGAGTCTTGTGGGTTATTTCGTTTTTTTAATC
TATATTGGTGGATTAATAGTTTTGTTTGGATATGTGCTTAGAATTTTCCCAAATCAGCGTTTTGGGGCCCCT
GTATTAGTTGTTAAACTATTATTATTTTTATTAGGATTTGTTTCAGTTTCTCATAAAAATAAAAGAGATCAC
TTTTTGAGTTGGGGGGATTTTGGCTCGATATACCTGTTTGTTGCGTTTCTTCTTCTTTTTGTATTGTTGTTA
GTTGTTGCTTTTTGTAAGAAGCGTCGTATACCACTCCGCACGGTG"/>
<sequence id="seq_Bathymodiolus_japonicusAP0145601" spec="Sequence"
taxon="Bathymodiolus_japonicusAP014560" totalcount="4"
value="GTGTTGTTAGATTTGTTATCAGGTTTCGACtcttttTCTTATAATCTAAGATGATCGTCATTTTTACGTatc
TTAAGTTTTTATTTTATCTTGATCTTGGCCAGCACTTTGTTTTTACCTTTTACCAAGGGGTTAGCTGTTAGT
TGGAGTATGTTCAAAAGTTCAACTGGCATGAATTTATCAGGATTTCCATTAATTGTTTCTTCCCTATTTATT
TTTTTAATAAGAGTGAATATAATGGGTTTGGTGCCTTTCTCTTTTAGTGTGACGTCTCATTTAAGTTTGGG
CCCAGCTTTAGCTTTTTTAATGTGGGGCTCTTTATGAGTCTCAGGACATCGAGTTAGATCTAAGCAAAAT
TTAACTAGGTTGGTGCCAAGGTTTCCAATGTTTTTAGTTCCTTTTTTGTTATTAGTTGAAATTGTTACTATT
AGTTCTCGTCCGATTACGCTTGGCTTTCGATTAATAATTAATATTATTGCGGGGCATTTAATCTTGTCTATG
GGATCTAATGTTAATAGATTCATTTCTTTAGGTCCTTTTTTTGGTGCAGTTTATTTTTCTTTATTTGCAATAG
GATTGGCGGCGGAATTAGCTGTAGGCCTAATTCAAGCTTTCATTTTCTGCTCTTTATTGAGTCTTTATACT
AATGATCATGCCAATTAATGATCGGGAATAATTGGTACAAGTTTGAGTATGTTGATTCGTATTGAATTGGC
TCGTCCTGGGAGTggaTTTTTAGGTGATGATCAGCTTTATAATGTTGTAACTCATGCATTGGTTATAATTTTT
TTCATGATGCCTTTGATGGTGGGTGGTTTTGGAAATTGGCTACTTCCTTTGATAATAGGTTCAATTGACAT
AATCTTTCCTCGTTTAAATAATTTAAGGTTTTGGTTCTTACCAGCTTCTCTCTTTACTCTTTTGTTGTCTAC
TTTTATTGAGAGAGGTGCTGGAACTGGTTGAACTTTGTACCCACCCCTGTCTTCCTACACTGGACACAG
GGGCCCAGCTGTGGATATATCTTTATTTTCTTTACATTTAGCAGGTGCTTCTTCTATTGGTGGTTCAATTAA
TTTTTTAACTAGGATGAAGAATATGTCTGTAGAAAGAATACGAGGGGAGCGGATAGTTTTATTTGTTTGA
TCTATGGCTGTAACAGCAGTTTTACTATTAGTTTCTTTACCTGTATTAGCAGGAGGAATTACTATGCTGATT
TTTGATCGTCATTTCAATACTTCTTTTTACGACCCTAGAGGTGGTGGAGATCCAGTTTTATACCAACATTT
GTTTTGGTTTTTTGGGCACCCAGAAGTCTACGTTCTTATTTACCAGGATTTGGAATAGTGTCTCATGTAG
TTGCTCATTGTGCAGGAAAGGATGAAGTGTTTGGTGTGCTAGGAATAGTTTATGCTATGGTTTGTATCGG
TGTCCTTGGGTTTATTGTTTGAGGCCATCATATATTCACTGTAGGAATAGATGTAGACTCTCGAGCTTATTT

TACTTCAGCTACAATAATTATTGCTGTGCCAACTGGAGTTAAAGTGTTTAGTTGATTGGCTACTTTAAATG
GAGGAAACTTGTTACATGAGCCTGCACTTTATTGGGCGGTTGGTTTCATCTTCCTTTTTACTGTTGGGGG
TCTTACTGGTATTATGTTATCAAATTCTTCTCTTGATGTGGCGTTGCATGACACTTATTATGTTACTGCTCAT
TTTCATTATGTCCTTTCTATAGGGGCAGTATTTGCCCTATTCTGTGGGTTTTTTCATTGATTTCCTTTATTCT
ATGGGTATTGTTACCATGAACGTTGAAGCAAAGCTCATTTTTTTATCATGTTTATTGGTGTAAATATCACTT
TTTTTCCTCAACATTTTTTAGGCTTAAGAGGAATACCTCGTCGTTATTCTGACTATCCAGATTGTTTTATAA
AATGGCATGTAGTGTCATCTTTAGGTTCATGATTGAGATTTGTTAGTGTTTTATACTTTCTTTTTATTGTGT
GGGAAGCTTTGGTAAGACAACGAGGTGTTGTTTGTAAAAGAAATCGACCTGGGGCTATTGAATGAAGG
GAGTGATGTTGTCCTTTGATGCCATTATGAGGTAGTTTTAGGTTTCAAGATTGTTATTATCATATTGGGGA
GTATCTTGGTTTGTTTCATGAGGGCGTGATGTGTCTTATAGTTTTCATTTTGTCTGTTGTGCTGTATGGAGT
AGGTTGGGTTTTTTCTTCGGGAAGAAGGTTTCGTTATTTACGTGAGGCACAAGCGGTAGAAACAGCATG
AACCATTATTCCTAGGGTGTGTTTAGTTTGTGTGGCTATCCCTTCTATACACTTATTGTATGTAATAGATGA
AATTGGTAACCCTAAATTCTGTTTTAAAGCAATTGGTCATCAATGGTTTTGATCGTATGAGATGGAAGATG
TGCTAGGATTTGATTCTTTTATGGAGCGGGAAATAGATAGGGGTTATCGGTTATTGGATGTAGACCAACG
GATGGTTGCGCCGGCTAATACCGGAATTCGGTGTATAGTAAGAAGGGCAGATGTCATTCATTCCTTTGCC
CTCCCTGGTTGCATATTAAAAGTAGACGCGATTCCAGGCCGTGTTAATGAAGTTCCTATAACTGTAAACA
TGTGTGGAGTTCTTTATGGTCAGTGTTCTGAGATTTGTGGAGCAAATCACAGGTTTATGCCCATTGTAATT
GAATTCATTCATCCGCGAGTGTACAATTTATGGGTAGAGTCTTTTGACATC------------------------
ATGCCACGTAATGCTTTTTATTTGGTCGGGCCAAGCCCGTGGCCTGTTTTTACTTCTATAGGAGCTTTTTG
TATGGCAGTAGGTTTTGTTTCTTGATTTCATAAGCATGGCTACGTTCTTTTATTAGGTGTGGTTGTGTTAAT
TCTTTCTTTAAGTCAATGGTGGCGTGATGTCATGCGTGAAGGTGATTTAGGTTTTCATACTTCATATGTTG
TTAAAGGTTTGCGGGATGGATTTATCCTTTTTTTGGTCTCAGAAATTATATTCTTCTTTTCTTTATTTTGAG
CTTTTTTCCACATAAGTTTAGCCCCTGATATTTCAATGGGGTGTATATGACCTCCCAAAGGTTTGGAGACT
TTAGACCCTATAAAAGTACCATTATGTGGAACTACAGTACTAGTTGGTTCTGGCGCGTCCCTTATGTATTC
TCATGCTGCTATTCGTGCAGGTTTTAATACTCACGCAGTTTTAGGAACTTTTTATACTATTGTGTTAGGTTT
GTTTTTTACACGATTACAAGGATACGAATATTATTGGGCTAGGTTTACTATCGCTGATAGTGCTTATGGGA
GTTTGTTTTATATTATGACCGGGTTTCATGGCCTTCATGTTATGTTTGGAACAGGGTTTTTACTTGTAAGCT
TAGTTCGTTTGATACGTAATCGGTTTACCCCACGCAACCATTTTGGGTTTATGGTGTGTTCTTGATACTGA
CATTTTGTTGATGTCGTTTGAATTGGTTTATATTTAGTTGTTTATTGTTGGGGTAGTTAACCTTTACGCAAA
CAGGATAGAGTAATAAAAATTTTAAATAATAGATTATATGATTTGCCTGCGCCTGTTAATTTAAGGGTTTG
ATGAAATTTTGGGTCTTTGTTAGGCTTATGCTTGGTTATTCAAATTGTTACAGGTTTTATTTTAAGCGTCCA
TTATACGGCCCACACAACTATGTCATTTGATTCGCTTATTCATGTGGTTCGTAACGTAAATAATGGTTGGT
TAATTCGTGGGATACATGCTAATGGTGCGTCTCTTTTCTTTGTTTGTGCTTATTTTCACATTGGTCGTGGTT
TGTATTATGGTTCTTATAAGTCTCGTATGGTGTGAAATGTAGGGGTAATTTTGCTTTTTCTTCTGATAGCTA
CTGCTTTTTTAGGTTATGTCCTCCCTTGAGGGCAGATGTCTTATTGAGGGGCAACTGTTATTACAAAACTA
GTAACTGCTGTGCCTTATGTTGGTAACATAATTTTGTATTGAATTTGGGGTGGTTTCACAGTTTGTAATGC
CACCTTGGTGCGATTTTATTCTTTTCATTTCACTCTTCCATTTGTTATAATTGTTTTTAGTGTTCTTCATTTG
CTTTACTTGCATGAAGAAGGGGCCAATAACCCGTTGGGGGTTAGTGCAGATGTGGCTTTGGTGCGGTTT
CATCCTTTTTACACATATAAGGATGCAGTAGGATTCTTTATTTTGTTTTTTGGTCTTTTATTTTTAGCTTGTT
ATTTTCCAGACCTTTTAGGAAATGTGAATAATTGAATTCCTGCAAATGTAATAAACACTCCCCTTCAAATT
GAGCCTGAGTGATACTTCTTATTTGCTTACTCTATTCTTCGTGCAATCCCCAATAAAACTGGCGGAGTTGC
TGCTTTGGTGGCATCAGTTTTAATTTTATTTCTTATTCCTAGGTTGCACACCGGCCAGTTCCGTTCCAATA
GGTTTTATCCTTTAGGTCAACTTTTCTTTTGGCTCTTTGTTTTAGTTTGAGTAGGTTTAACATGACTTGGG
GCTTGTCCGGTGCAGCACCCTTATGATTCTTTGGGGTACTTTTTTACTTTCCTCTATTTCTTTTTTATTATG
CTGATCCCTATAAGACAAGGCATGTGGGATTGGTTAATTAAAATGCAAGTTATCAGCTTTATTTTACCAGG
AGTTTTTGGTTTATTAGCAGTAGGATGATTTACATTAGTTGAGCGTAAAGTGTTAGGTTACATTATAACTC
GTAAAGGCCCCAATAAAGTGGGACTTTTAGGGTTAATGCAACCAATAAGTGATGGGGCTAAACTATTTTC
TAAAGAACTGGTTGTGCCAATATATAGGAATTTTGTTCCATTTCTGGTGTGTCCTGTGGTGACGTTCTTTA
TCGCCTTGTTGCTATGATTGCTTTACCCATTTCACTCTTCAGAAGGTATATTTATGTGCGGGGTATTATTTT
ATTTGGCAAATTCTGGAGCTAACGTTTATGGGGTAATGGTGGCAGGATGATCATCAAATTCAAAATATGC
CCTTTTAGGGGCCATGCGGGCTATGGCACAAAGTATTTCATACGAAGTGAGAATGGCATTAGTACTTTTA
AGATGTGTTTTTATATCAGGGTCAATACATCTTCAATCCGTGAAGTTGTTGTTTTTTATGGGTGCAATTCTA
CCTTTTTTTGTGGTGTGATTAATTTCTATGTTAGCAGAAACTAATCGTGCACCATTTGATTTTGTAGAGGG
CGAATCAGAGCTGGTGTCAGGTTTTAATGTGGAGTATAGTAGGGGCGGGTTCGCCCTTATTTTCATAGCA
GAATACTCTAATATGTTGTTCAATAGCTTGTTTACATGTGTAATATTTTTGGGCACAAGTGACGCAATAAT
GGTCGGGCAGGCATGAATTTTTCTTTTCTTTTTTTTGTGGGCTCGTGGAACTTTTCCTCGTTTTCGATACG
ATATGTTAATAAGGTTGGCTTGAAAGACTTTTTTATGTATTGTCTTGGGGTTGAGTTTATTTGTTTCTATAA
TTATTTATATTTTataattaggcggtttgagggccctttaATAGTTTTGAGGGGTTTTACTCTAACTTTTGGTTTGATTATTA
TAGTTTCAACTGATAGCCACATGACTGCGTGGGTTGGGATAGAAGTAAATATGCTTGGGTTTATATGCCTT
TTGGGAATTAAATCAGTGTTAAATATGCGTGTTTTGATTAATTATTTTGTTTTTCAGAGACTTGGTTCAAC
TATGTATTTGTTCGGTTCAAGCGTTATTTTGTTTAATATTGCTTTAGGGTACTTTTTAATTCATTTAGGACTT
CTTTGCAAAGCTGGCCTTTTCCCTTTTTGGGTATGGGTTCCTTCTGTAGTAAATTCTAGGGGTTGACTTGT
TAGCTATCTATTATTAGGTATTCAAAAAGTTGGGCCTTTACTTTTGGGTGTGTGGTCTCCTTGTAGGGAAT

TTTTATATCTTGTAATTTTTGCCATAAGGGTTTTGGGTGGTTTAGGCGGGTCTATTCAGAACTCTTATCGTG
ATGTATTGGTGTATTCTTCTTTTGTTCATGGTGCGTGAATGTTGGTATGTTTAATTGAGTCTCAGAATTTAT
TCTTTTTTTATATTGGAGCTTATCTTGTACAATTAGGTTTAGTTGTTTACTATCTGTGGTGCAGAAATGCAA
ATTCTATAAAAAGAGGAAAATGGTCTCTTATAGTAGCAAGGTCTCTAATAAGTTTGAGGGGGTTACCTCC
ATTAGCTGGTTTTGTGGTAAAGATAGTTGTTGTTTTTTGCGTTGATAAATTAATTTTGTTATTTACATTAGC
TGGTTCTATTATGGCCATATTTTACTACTTGAAAGCGGCAAATAGCTCTATCGTTAAATTATTAGATGATGA
GGGCTGCTTTCTCTCTTTTACTTTTGGTTTATATGCTTGAATCTATTTATTGGGGTGTCTTTTCTTGTAATAA
TCTGTGTTTGTTTTTTGGCTGGTTGTGATAATGGGTGTTTGTTTTATGTTATCTATAATGctcatgttatctatgacgG
GGCAGTGGAGTCGTGAAAAGGTTTCCCCCTATGAGTGTGGGTTTGATCCAATTCTTAGGGCCCGTAGGA
GGTTTTCTTTGCGGTTTTTCTTACTTGGTGTGTTGTTTTTAGTGTTTGATGTGGAAGTGGTTATGGTTGTT
CCACTTTTGTTTGTATTGTACGGAGGGGCGGAGGTAGTGGGTGTTGTATGTTTAGTGGGGTTTTTACATG
TGCTTACTATTGGGTGTCTTTATGAGCGTCGTGATGGGTCCATGGATTGGGTTAGTGAAGTGATTATTGGT
CTTGTTATATGTATACTTTTTTTAGGTTTGTTGGGTAGATTAGAGTTATCAATTGCTGGATTGTCTTTATTGG
GCGCGATAgcgATGATATCTTTATTTTTGTGCGCTGAGTACTCTGAGTTAACTGGAGTTTTTAATTTGGATT
TCCCAGGGCAGGCTTTAGTAGTCCTTAGAATTTATATTAGATTTTTAATGGTGATGGGCAGGGCTATGGTT
TCTCGTTTTAGTAGGTTTAATAGCCTTATTATTTGTATTGGTGCTTTGTTAATTTGTGCCTTTAGTGTGAGT
GCCACTTTCTTCTTTTTCGTTCTTTTTGAGGGGGTTTTGTTCCCTACTTTACTGTTGGTTGTAGGGTGGCG
ATTGCAAGCAGTTGTTTATATGGTTATTTATACTGTTATAGGATCACTTCCACTTTTGTACGGGTTTGGGAG
TCTATATTTTAATTATAGTGATAATTTATTTTTGGAGTACTTTTTGGACAAAAGAATTTTGAGTTTTAGTTG
GCTTTATCTGTTAGCGTTTTTAGTTAAGCTCCCTGTTTTTCCTCTACATTTATGATTACCTAAGGCTCATGT
AGAGGCTCCAGTTGCTGGTTCTATGATTTTGGCAGGCCTTTTGCTTAAGTTAGGGGGTTACGGGGTTATT
CGTTTAAGAGGCCTTCTTATTATTAAAAGAATCAGAATAACTATATTAGTGGTAATAGTTAGGCTTTTTGG
AGGAATTCTTACAAGTATGGTTTGTTTACGTCAGACAGATTTAAATCTTTAGTAGCTTATTCTTCTGTTG
GGCACATAAGTTTTGTTTTAGTTTTATTGAGTAATACTTTATGGGGGCTTATAGGGGCCATGCTAGTCATA
ATTGGGCATGGTTTATGTTCCTCTGGCTTATTTTCTTTGGTTAATACTTTCTATCTTAGAAGTCGTTCTCGC
TTGCTGGCAATAAATAAGGGGTATTTAATACTTAGCCCTTGTGTAGCTTTGTTGTGCTTTTTGCTGAGGGT
TAGAAATATAGCTAGCCCACCCAGGTTGAACCTATTTGGAGAACTTTTTATTTATATAGCAGGTGCTTCTA
TAAACATGTTGTTTTTCCCTTTATTAATGTTGTTGAGGTTTTTGAGTGCCTGCTATAGGCTTTATATTTATAT
TAGTTCCCAACATGGTAAATGTATGAATAGATTAGACTCACGTACAAGTATAAGAGATATTTCGGTGTTGG
CTTTTCATTGAATGCCTTTAAACTTTTTGTTTGTAGTTATTCCATAGGTGCGTAGAACACATTTAATTACGA
TCTTTCTAGGGATGGAGTTTAGTGCACTTGGCGTTTTTGTGATTGGATCTTTATCGGCGTACAACAGTTTA
TTTGTTTTATTAGTTGTAATCTGTATTAGGGTATCTGAAGCAGCCATTATGCTGTCTTTGATGGTCATAATA
ACTCGTATGTACGGAAACGATCGATGTCTGAATCTAATAACTGATAATTTAAAGTTAATGAAGTTTTAGGT
TGTATTTTTATGGTTTTAGGGTGGGGTACTATAAGTATAGTTCCCTTTAGCCCTTTAGTTCTTGTTGACTAT
AGAATTTGATTATCTAGAAATTTGTCAGTAAATTTGACATTATTGTTAGATCAAGTTAGGGGGTTATTTATA
GCTACCATTTTTTTGATTTCGGGGTCTGTTTTAGTTTATTGCTCTTGGTATATAGCGGATGAAATTTATTTTT
CGCGTTTCATTTATCTTGTTGTACTTTTTGTTTTATCTATAATATCCTTAATTATAATCCCTAATTTAATTGCT
CTTTTACTGGGATGAGATGGACTTGGTATTACCTCTTTTTTATTGGTGGTTTATTATCAAAATAATAAGTCT
TTGGGAGCAGGCATAGTTACGGCCCTGACTAATCGTGTCGGAGATTCAATCCTCTTATGTATTATTGGTGT
GTTTGCTAGGGAGGGAGGGTGAGTATTATTTGAGTTTTTACCTAGTATAGGGTATTTTGTCCCTTTTCTTT
TAGTAATAGCAGCTATTACTAAAAGAGCTCAAGTGCCTTATTCGGCGTGGTTGCCTGCTGCAATAGCTGC
CCCTACCCCTGTTTCTGCATTAGTTCACTCGTCTACTTTGGTTACAGCTGGGGTTTATCTTTTGATTCGTTC
CTACCCACTTCTTTCTCAAAGATCTTTAAGGGTGCTAAAGTGTCTCAGGCTTTTTACTTTACTAATAGCTG
GGAGGGTGGCTCTTGTTGAGGTGGATTTAAAAAAGATTGTAGCATTATCTACTTTAAGCCAGTTAAGAAT
AATATTATTTGCTATCTCAATTTGTCTACCAAAAGTAGCTTTTTTTCATCTTATTACCCATGCTATATTTAAG
TCTCTTTTGTTTCTAAGTGCAGGGGTTGTGATTCACAGAAGGTTAAAATGACAGGATATTCGTTTTTTAG
GAAAAAATTGAGCGCGGTTGCCAGTCAGGATGAGTTGTGTTACTGCCGCGAGTCTTTCTTTATGTGGCA
TACCCTTTCTGAGGGGGTTTTACTCAAAAGACTTGATTATTGAATTTCAAGGTGGGGATTTGGTTGTTTA
CTTTATAGTAGTTGTTGGAACTTTATTTACCTCTTGATATTCCCTCCGTATATTTAATCTGTTTTTGAGTGCA
AATAAGAGTACTAGTCTTGTAACTCACGCGAAAGAAGATAGGAGAGTTAAACTAGCTTACAGGGGTTTA
TTGGTTAGATCTGTGATTATAGGTTTTTTATTAGTTAACTTAGTTTCAGACTTTGATCTCATGGCTATGGTG
AGTAATGTGGAAAAAATTTGTTGTAATATCATTGGTGGTTGCTGCCATCATTGAGGTTTCTGCAGGATGAA
AAAGGAATAAAAAAGTAGGTTATGGTTATTTGGCTAGTATATGGCATTTTAAGCTGTTACAATTTAGTCCT
AGTGCAGGCTTAAAGTTAGCTAGTCTTATTACAGTTAGAATGGAGAAAGGGTGATTAGAAAAAATTGGC
CCTCAAGGAAGAGTTCAAACTTTAGGTTTTAGTAACCAATACCATTTTTTAACTGTGTTGCTTTTTAGGC
TATTTCTTGTGATTATTTTGTGCTTGTAGTTGTTAGAAACATTTCTCTTTTTTGTTTCTTGTTTGGTATGCAT
GTTAAGGATCAGAGAGCCTTTGTTTATGGGTTTGTCCCTATTCGTTGTATCTTTTATTATTTCAATTCTATTA
GGGCTGGAAATGGGTAGCCTTGTGGGTTATTTCGTTTTTTTAATTTATATTGGTGGATTAATAGTTTTGTTT
GGATATGTGCTTAGAATTTTTCCGAATCAGCGGTTTGGGGTCCCTGTATTGTTTGTCAAATTATTATCAGT
TCTATTAAGATTCGTTTTGGTTTTTCATAAAAATAAGGGAGACCAGTTTTTAAGTTTGGGGGATTTTGGCT
CGATATACCTGTTTGTTGCGTTTCTTCTTCTTTTTGTATTGTTGTTAGTTGTTGCTTTTTGTAAGAAGCGTC
GCATGCCTCTTCGCATGGTG"/>

<sequence id="seq_Bathymodiolus_securiformisNC03951" spec="Sequence" taxon="Bathymodiolus_securiformisNC0395" totalcount="4" value="GTGCTATTAGATTTGTTGTCAGGTTTTGACtcttttTCTTACAATATGAGTTGGTCATCTTTCTTACGTat
cTTAAGCTTTTATCTTATTTTGACTTTGGTTAGTATTTTGTTTTTATCTTTTACTAAGGGTTTAGCTGTTAGT
TGGAGTATGTTTAAAACTTCAACCGGCATGAGTTTATCAGGATTTCCATTAATTGTTTCTTCTTTGTTCATT
TTTTTAATAAGAGTAAATATAATGGGTTTGGTACCTTTTTCTTTTAGTGTGACGTCTCATTTAAGTTTAGGC
CCTGCCCTAGCTTTTTTAATGTGGGGTTCTTTGTGAATTTCAGGCCATCGAGTTAGCTCTAAGCAAAATTT
AACTAGGTTGGTACCAAGGTTTCCTATATTTTTAGTTCCTTTTTTGCTATTAGTTGAAATTGTTACCATTAG
TTCTCGCCCGATTACTCTTGGCTTTCGATTGATAATTAATATCATTGCGGGGCATTTAATTTTATCTATGGG
AACTAATGTTAATAGATTTATTTCTTTAGGCCCTTTTTATGGTGCGGTTTACTTTTCTTTATTTGCAATGGG
ATTAGCGGCGGAATTAGCTGTAGGGCTAATTCAAGCTTTTATTTTTTGCTCTTTATTGAGACTTTACACTA
ATGATCACGCCAATTAATGATCGGGAATAATTGGTACAAGTTTGAGGATGTTGATTCGTATTGAATTAGCT
CGTCCTGGGAGCggaTTTTTAGGAGATGACCAGCTTTATAATGTTGTAACTCATGCATTGGTTATGATTTTT
TTTATAATGCCTTTGATGGTGGGCGGTTTTGGAAATTGGCTACTTCCTTTAATAATAGGTTCAATTGACAT
AATTTTCCCTCGTTTAAATAATTTAAGGTTTTGGTTTTTGCCGGCGTCTCTTTTTACTCTTTTGTTATCTAC
TTTTATTGAGAGAGGCGCTGGAACTGGTTGAACTTTGTACCCCCCTTTATCTTCTTATACTGGTCATAGGG
GCCCAGCCGTGGATATGTCTTTATTTTCTTTACATTTGGCAGGTGCTTCTTCTATTGGTGGTTCAATTAATT
TTCTGACGAGAATGAAGAATATGTCGGTTGAAAGAATGCGAGGGGAGCGGATAGTTTTATTTGTTTGATC
CATGGCCGTAACAGCAGTTTTATTATTAGTTTCTTTACCTGTATTAGCAGGAGGAATTACTATGTTGATTTT
TGATCGTCATTTTAACACTTCTTTTTATGACCCTAGGGGCGGAGGAGACCCAGTTTTATACCAACATTTGT
TTTGGTTTTTTGGGCATCCAGAAGTTTATGTCCTTATTTTACCAGGGTTCGGAATAGTGTCTCATGTGGTT
GCTCATTGTGCGGGAAAGGATGAAGTGTTTGGTGTGTTAGGAATAGTTTATGCTATAGTTTGTATTGGTG
TTTTGGGTTTTATTGTTTGAGGGCACCATATATTTACCGTAGGAATAGATGTAGACTCTCGAGCTTATTTTA
CTTCAGCTACAATAATTATTGCTGTTCCAACTGGAGTTAAAGTGTTTAGTTGATTGGCTACTTTAAATGGT
GGAAATTTATTGCATGAGCCCGCACTTTATTGGGCAGTAGGTTTCATTTTTCTTTTTACTGTGGGAGGCCT
TACTGGTATTATGCTATCAAATTCTTCTCTTGATGTGGCGTTGCATGATACTTATTATGTTACTGCTCACTTT
CATTATGTTCTTTCTATAGGTGCAGTATTTGCCCTATTTTGTGGTTTCTTTCATTGATTTCCTTTATTTTATG
GGTACTGCTACCATGAACGTTGAAGTAAAGCTCATTTTTTTATTATGTTTATTGGTGTAAATATTACTTTCT
TCCCCCAGCATTTTTTAGGTTTAAGTGGGATGCCTCGTCGTTACTCTGATTATCCAGATTGTTTTATAAAAT
GGCATGTAGTGTCATCTTTAGGGTCATGGCTGAGATTTGTCAGTGTTTTATACTTTCTTTTTATTGTGTGG
GAAGCTTTGGTAAGACAACGAGGTGTTGTGTGTAAAAGAAACCGACCCGGGGCTATTGAATGAAGGGA
GTGGTGTTGTCCTTTGATGCCATTATGAGGTAGTTTTAGGTTTCAAGATTGTTATTATCATATTGGGGAGTA
TCTTGGTTTGTTTCATGAGGGTGTGATATGCCTTATAATTTTTATTTTGTCTGTTGTGTTGTATGGAGTAGG
TTGGGTTTTCACCTCGGGAAGAAGGTTTCGTTATTTACGTGAGGCACAAGCGGTAGAAACAGCATGAAC
TATTATTCCTAGGGTGTGTTTAGTTTGTGTGGCTATCCCGTCTATACACTTATTGTATGTAATAGATGAAAT
TGGGAGTCCTAAGTTCTGTTTTAAAGCAATTGGCCATCAGTGATTCTGGTCTTACGAAATGGAAGATGTG
TTGGGATTTGATTCTTTTATGGAGCGGGAGATAGAGAGAGGTTATCGATTATTGGATGTAGACCAACGGA
TGGTTGCGCCGGCTAATACCGGAATTCGATGCATGGTAAGAAGGGCGGATGTTATTCACTCCTTTGCTCT
CCCTGGTTGCATATTAAAAGTCGACGCTATTCCAGGCCGTGTTAATGAAGTTCCTATAACTGTAAACATGT
GTGGAGTTCTTTATGGTCAGTGCTCTGAGATTTGTGGCGCAAACCACAGGTTTATGCCCATTGTGATTGA
ATTTATTCATCCGCGTGTGTATAATTTATGGGTAGAATCTTTTGACATC-----------------------
ATGCCACGTAATGCTTTTTATTTGGTTGGGCCAAGTCCGTGGCCTGTTTTTACTTCTATAGGAGCTTTTTG
TATGGCAGTAGGTTTCGTTTCTTGGTTTCATAAGCATGGTTATGCTCTTTTATTAGGTGTGGTTGTATTAAT
TCTTTCTTTAAGACAATGGTGGCGTGATGTTATGCGTGAGGGTGATTTAGGTTTTCACACTTCATATGTTG
TTAAAGGTTTACGGGATGGATTTATTCTTTTTTTGGTTTCAGAAATTATATTTTTCTTTTCTTTATTTTGAGC
TTTTTTCCACATAAGGTTAGCTCCTGATATTTCAATGGGATGTATGTGACCTCCTAAAGGTTTGGAAACTT
TAGACCCTATAAAAGTTCCATTATGCGGAACAACCGTATTAGTGGGCTCTGGTGCGTCTCTTATGTATTCT
CATGCTGCGATTCGTGCAGGTTTAAATACTCATGCAGTTTTAGGAACTTTTTATACTATTGTGTTAGGCTT
GTTTTTTACACGGTTACAAGGATATGAATACTATTGAGCTAGGTTTACTATTGCTGATAGTGCTTATGGGA
GTTTGTTTTATATTATAACTGGGTTTCATGGGCTTCATGTTATATTTGGGACAGGATTTTTACTTGTCAGCT
TAGTTCGTTTAATGCGTAATCGGTTCACCCCACGCAACCATTTTGGGTTCATGGTGTGTTCTTGATATTGA
CATTTTGTTGATGTTGTTTGGATTGGCTTGTATTTAGTTGTCTATTGCTGGGGTAGTTAACCTTTACGCAA
GCAGGATAGAGTAATAAAAATTTTAAATAATAGATTGTATGATTTACCCGCGCCTGTTAATTTAAGGGTCT
GATGAAATTTTGGGTCTTTGTTAGGTTTATGCTTGGTTATTCAAATCGTTACAGGTTTCATCTTAAGCCTC
CATTATACGGCCCACACAAATATGTCATTTGATGCGGTTATTCATGTGATTCGAAATGTAAATAATGGGTG
GTTAATTCGTGGCATACATGCTAATGGTGCGTCTCTTTTCTTTGTTTGTGCTTATTTTCACATTGGTCGTGG
TTTGTATTATGGTTCTTATAAGTCTCGGATGGTGTGAAATGTTGGGGTGATTTTGTTTTTTCTTCTTATAGC
TACTGCTTTCTTGGGTTATGTTCTCCCTTGGGGGCAAATATCTTATTGAGGGGCAACTGTTATTACAAAAT
TAGTGACTGCTGTGCCTTATGTTGGTGATATAATTTTGTATTGAATTTGGGGCGGTTATACAGTTTGTAATG
CTACTTTGGTGCGATTTTATTCCTTTCATTTTATTCTTCCATTTATTATAATTGTTTTTAGTGTTCTTCATTTG
CTTTACTTGCATGAAGAGGGGGCTAACAACCCGTTGGGGGTGAGTGCAGATGTGGCTTTGGTGCGATTT
CATCCTTTTTACACATATAAGGATGCAGTAGGTTTCTTCATTTTATTTTTTGGTCTTTTATTATTAGTTTGCT
ATTTTCCTGACCTTTTAGGAAACGCGAACAATTGAATTCCTGCGGATTCAATAAAAACTCCACTTCACAT

TGAACCAGAGTGGTACTTTTTATTTGCTTATTCTATTCTTCGTTCAATTCCTAATAAAGTAGGTGGGGCAG
CTGCCTTGGTGCTATCAATTTTAATTTTATTTCTTGTTCCTAGGTTGCATACAGGTCAGTTTCGTTCTAACA
GGTTTATCCTTTAGGTCAGCTTTTCTTTTGGATTCTGGTTGCAGTTTGAGTGATTTTAACATGGCTTGGG
GCTTGTTCGGTTCAGTACCCTTATGATTCTTTGGGGTGTTTCTTTACTTTCTTCTATTTCTTTTTTATTATAC
TAGTTCCTTTAAGACAAGGCGTGTGGGATTGGTTAATTAAGATGCAAGTTATCAGCTTTATTCTACCAGG
AGTTTTTGGTTTATTGGCAGTAGGATGATTTACATTAGTCGAGCGCAAAGTGTTAGGTTATATCATGACTC
GTAAAGGCCCTAATAAAGTGGGATTTTTAGGGTTAATGCAACCAATAAGTGATGGGGCTAAACTATTTTC
TAAAGAAATACTAGTACCAATATATAGGAATTTTGTCCCATTTCTGGTGTGTCCTGTGGTGACATTTTTTAT
TGCCTTGGTGCTGTGATTACTATACCCATTTCACTCTTCTGAGGGTGTGTTTATGTGCGGGGTATTATTTTA
TCTGGCAAACTCTGGAGCTAATGTTTATGGCGTAATGGTGGCAGGGTGATCATCAAATTCAAAATATGCC
CTTTTAGGGGCTATGCGGGCTATGGCACAAAGTATTTCATACGAAGTGAGAATAGCATTAGTACTGTTAA
GATGTGTTTTTATGTCGGGGTCAATACATCTTCAATCTGTGAAGTTGTTGTTTATCATGGGTGCAATTTTA
CCTTTTTTTGTGGTGTGGTTAATTTCTATGTTAGCAGAAACTAATCGTGCACCATTTGATTTTGTAGAGGG
CGAATCAGAGCTGGTATCAGGTTTTAACGTGGAGTACAGTAGAGGCGGGTTCGCCCTTATTTACATAGCA
GAATATTCTAATATGTTGTTTAATAGCCTGTTTACATGCGTGATATTTTTGGGTACAAGTGACGCAGTAATA
GTCGGGCAGGCATGAATTTTTCTTTTTTTTTTCTTGTGGGCTCGTGGGACTTTCCCTCGTTTTCGATATGA
TATACTAATAAGGCTGGCCTGAAAGACTTTTTTATGTATTGTTTTGGGGTTGAGTTTATTTATTTCTATAAT
TATTTATATTTTataATTAGACGATTTGAGGGTCCTTTAATAGTTTTGAGGGTTTTTACTTTAACTTTTGGTTT
GATTATTATAATCTCAACCGATAGGCATCTAACTGCATGGGTTGGGATAGAAGTAAATATGCTTGGGTTTA
TGTGCCTTTTGGGAATTAAATCAGTGTTAAATATGCGTGTTTTGATTAATTATTTTGTTTTTCAGAGATTTG
GCTCAACTATATATTTGTTTGGGTCAAGTGTTATTTTATTTAATATTGCTTTAGGATATTTTTTAATTCATTTA
GGACTTCTTTGTAAAGCTGGACTTTTCCCTTTTTGGGTGTGGGTTCCTTCTGTAGTCAACTCTAGGAACT
GATTTGTTAGCTATTTGCTATTAGGAATTCAAAAAGTTGGCCCTTTACTTTTGGGTGTGTGGTCCCCTTGT
AGAGAGTTTTTATATTTTGTAATTTTTGCTATAAGAATTTTGGGTGGTTTAGGTGGGTCTATTCAGAATTCT
TATCGTGACGTATTGGTGTATTCTTCTTTTGTTCACGGTGCTTGAATGTTAGTATGTTTGATTGAATCCCAG
AATCTATTTCTTTTTTATATTGGAGCTTATCTTGTACAATTAGGTTTAGTCATTTACTATTTGTGGCGCAGA
AATGCAAACTCTATAAAGAGGGGGAAATGATCACTTATAGTGGCAAGGTCTTTGATAAGTTTGAGGGGG
CTGCCTCCATTAGCTGGTTTTGTGGTAAAGATAACTGTTGTTTTTTGCGTTGATAAGTTAATTTTGTTATTT
ACATTAGCTGGCTCTATTACGGCTATATTTTATTATTTGAAAGCTGCAAATAGCTCCATTGTTAAATTATTA
GATGATGAGGGCATTTTCCTCTTTTTTACTTTTGGTTTATACGTTTGAATTTATTTGTTGGGGTGTTTCTTT
TTGTAATAATCTGTGTCTGTCTTCTGGTTGGTTGTGATAATGGGTGTTTGTCTTATGTTGTCTATAATGctcat
gttatctatgacgGGGCAGTGGAGCCGTGAAAAGGTTTCGCCTTATGAGTGTGGGTTTGACCCAATCCTTAGGG
CCCGTAGGAGGTTTTCTTTGCGTTTTTTCTTGCTTGGTGTTTTGTTTTTAGTGTTTGATGTAGAAGTGGTT
ATGGTTGTTCCCCTTTTGTTTGTATTGTATGGTGGGGCGGAGGTAGTGGGTGTTGTGTGTTTAGTGGGTT
TTTTACATGTGCTTACTATTGGTTGTCTTTATGAGCGTCGCGACGGGTCGATGGATTGGGTTAGTGAGgtgA
TTATTGGTTCTGTTATGTGTATACTTTTTTTAGGGTTGTTAGGAAGATTGGAGTTATCAATTGCTGGTTTGT
CTTTACTAGGTGCGATAGCGATGataTCTTTATTTCTGTGCGCTGAATACTCTGAATTAATTGGAGTCTTTAA
TTTGGATTTTCCAGGGCAGGCCTTAGTAGTCCTTAGAATTTATATCAGATTTTTAATGGTGATGGGCAGGG
CTGTGGTGTCTCGTTTTAGGAGGTTTAATAGGCTTATTATTTGTATTGGTGTTTTGTTGATTTGTGCTTTTA
GTGTAAGGGCCACTTTCCTGTTCTTTGTCCTTTTTGAGGGAGTTTTATTTCCAACCTTGTTGTTGATTGTA
GGATGGCGGTTACAAGCAGTTGTTTATATAGTTATTTATACTGTTATAGGATCACTACCCCTTTTGTACGGG
TTTGGGAAACTATATTTTAATTATAGTGACAATTTATTTCTGGAGTACTTTTTGGATAAAAGAATTTTGAG
GTTTAGCTGGCTTTACTTATTAGCGTTTTTAGTTAAGCTTCCGGTTTTTCCTTTACATTTATGATTACCTAA
AGCTCATGTAGAGGCCCCAGTCGCTGGTTCTATGATTTTAGCAGGTCTTTTGCTTAAGTTAGGGGGTTAC
GGGGTTATTCGTTTAAGAGGCCTCCTTATTATTAAAAGAATTAGAATAACTATACTAGTAGTAATAGTTAG
GCTTTTTGGAGGGATTCTCACAAGTATGGTTTGTTTACGTCAGACAGATTTTAAATCTTTAGTAGCTTATT
CTTCTGTTGGACATATAAGTTTTGTTTTAGTCCTATTGAGTAATACTTTATGAGGGCTTATAGGAGCGATGT
TAGTCATGATTGGTCATGGATTATGCTCTTCTGGTTTATTTTCTCTGGTTAATACTTTCTATCTTAGAAGCC
GTTCTCGTTTGTTGGCAATAAATAAGGGATACTTAATACTCAGCCCGTGTGTAGCTTTGATGTGCTTTTTG
TTGAGGGTTAGAAATATAGCTAGCCCACCTAGGTTGAACTTATTTGGAGAACTTTTTATTTACATAGCAG
GTGCTTCTATAAATATGTTGTTTTTCCCTTTATTAATGCTGTTGAGGTTTTTGAGTGCTTGCTATAGGCTTT
ATATTTATATTAGTTCCCAACATGGAAAATGTATAAATAGACTAGATTCACGTAGAAGTATGATAGACATTT
CGGTGTTGACTTTTCACTGAATGCCTTTAAACTTTTTGTTTGTAGTTATTCCATAGATGCGTAGAATACAT
TTGATTACGATTTTTCTAGGGATGGAGTTTAGTGCGCTTGGGGTTTTTGTCATTGGGTCTTTATCGGCGTA
CAATAATTTATTTGTTCTACTAGTTGTAATTTGTATTAGGGTGTCTGAAGCAGCCATTATGCTGTCTTTGAT
GGTTATAATGACTCGTATATATGGAAATGATCGATGCCTTAATCTGATAACTGATAATTTAAAGTTAATGAA
GTTTTAGGTTGTATTTTTATGATTTTAGGATGGGGTACAATAAGTATAATCCCTTTCAGCCCTTTAGTTCTT
GTTGACTATAGAATTTGATTGTCTGGAAATTTGTCGGTAAATTTGACACTTCTACTCGATCAAATTAGAGG
ATTATTTATAGCCACCATTTTTTTGATTTCTGGCTCTGTTTTAGTTTATTGCTCCTGGTACATGGCTGATGA
AATTTATTTTTCGCGTTTTATTTATCTTGTTGTACTTTTTGTTTTATCTATAATATCCTTAATTATAATTCCTAA
TTTAATTGCTCTTTTGCTGGGATGAGACGGACTTGGTATTACCTCTTTTTTATTGGTGGTCTATTACCAAA
ATAACAAGTCTTTGGGGGCAGGTATAGTTACGGCCTTGACTAATCGAGTTGGAGATTCAATCCTGTTATG
CATTATTGGTGTGTTTGCTAGGGAGGGGGGATGAGTATTATTTGAATTTTTACCTAGTATGAGGTATTTTG

TCCCTTTTCTTTTAGTAATAGCTGCTATTACTAAAAGAGCCCAAGTGCCTTATTCGGCGTGGTTGCCTGCT
GCAATAGCTGCCCCCACCCCTGTTTCTGCATTAGTCCACTCGTCTACTTTGGTGACAGCTGGGGTTTACC
TTTTGATTCGTTCTTACCCCCTTCTTTCTCAAAGGTCTTTAAGGGTATTAAAGTGTCTCAGGCTTTTTACT
TTACTAATGGCTGGGAGGGTAGCTCTTGTTGAGGTGGATTTAAAAAAGATTGTAGCATTATCTACTTTAA
GCCAGTTGAGAATAATATTATTCGCTATTTCAATTTGTTTACCAAAAGTGGCTTTCTTTCATCTTGTTACTC
ATGCTATATTTAAATCTCTCTTGTTTTTAAGGGCAGGGGTTGTGATTCATAGAAGGTCAAAATGGCAGGAT
ATTCGCTTTTTAGGAAAAAATTGAGCTCGTTTGCCAGTTAGGATAAGTTGTATTACTGCCGCGAGTCTCT
CTTTATGTGGTATGCCCTTTTTGAGAGGGTTTTATTCAAAAGACTTGATTATTGAGTTTCAAGGCGGGGA
CTTAGTCATTTATTTTATACTAGTTGTTGGAACTTTATTTACCTCTTGGTACTCCCTTCGTATATTTAATTTG
TTTTTGAGTACAAATAAGAGTGTTAGCCCTGTAATTTTCGTGGAAGAAGACAGGAGTGTTAAATTTGCTT
ATATGGGCTTATTAGTTAGCTCTGTGGTTATAGGCTTTTTATTAGTTAACTTAGTTTCAGACCTTGATCTCA
TGGCTATGGTGAGTAATGTGGAAAAGTTTGTTGTAATATTATTGGTGGTGGCTGTTATTATTGAGGTTTCT
GCAGAATGAAAAAAACAAAACAAAGTGGGTTATGGTTATTTGGCTAGTATATGGCATTTTAAACTGTTAC
AATTTAGCCCTAGTGTGGGTTTAAAATTAGCTAGTCTTATTACAGTTAGGATGGAGAAAGGGTGATTAGA
AAAAGTTGGCCCTCAAAGAAGAGTTCAAACGTTAGGTTTTAGCAATCAATACCACTTTTTATTGAGACT
TAGGCTGTTTCTTTTGATTATTTTGGGGAGGTACTTGTAATTGTTAGAAACATTTCTCTTCTTTGTTTCTTG
TTTGGTATGTATGTTAAGGATTAGGGAGCCTTTGTTTATGGGATTGGCCTTATTCGTTGTATCTTTTATTATT
TCTGTTTTGTTGGGGTTAGAAATAGGTAGTCTTGTGGGTTATTTTGTTTTTTTAATTTATATTGGTGGATTA
ATAGTTTTGTTTGGGTATGTTCTTAGAATTTTTCCAAATCAGCGATTCGGGGCTCCTGTTTTATTTGTTAAA
TTATTATTAGTTTTACTAAGATTTGTTTTGGTTTTTCATAAAAATAAGAGAGATCAGTTTTTAAGCTTGGG
GGATTTTGGTTCCATATACTTGTTTGTTGCGTTTTTCCTCCTTTTTGTATTGTTGTTAGTTGTTGCTTTTTGT
AAGAAGCGTCGTATACCTCTTCGGATGGTG"/>
<sequence id="seq_Brachidontes_exustusKM2336361" spec="Sequence"
taxon="Brachidontes_exustusKM233636" totalcount="4"
value="ATGATATTAGACATTTTCTCTTCTTTTGACATAAACTGTTACAATACTATATCATTAAGATCTGTGTG
AGTTATAAGCTTTTTTTTTCTTAATTTTTTGTTTGAATAGCAAGGTTTTTAGGTTAAAAATGGTAATTAGATT
TATATATGGAGTAGTAAAACGAGTTCAGGGAGGGTATTTTTCTGGGTTCTCCTTAAGCGTTTGTTCCTTGT
TTTTTCTAATGGTATGAATAAATATAAATAACATTGTCCCATTTTTTTTTCCTTTAATATGCCATCTTCCCCT
GCCGATGTTTTTTGCTTGTTTTTTTTGGGGTATTTTGGTTGTGTCAAGCTTACTTAATAGTTGGGAACAGG
TTGTTGCAATGGCAGTCCCCCTAGGTCCTTTAATTCTATCTCCTCTAATAGTTTTATTAGAAACAATTGCTT
CTATTGTCCGTCCTATCACTTTAACAATACGGGTGGTGTTTAATTTAGCGGGAGGGCAAGTTATTTTAGGG
CTAATTGCTGAGTTAGATAAAAAATTTAGGTTTGTTAGCTATGTTAGTGATTATCCTTGTGGTAGTATTAGGT
ATTGGGTTCGCATTTGAAGTAGGAATCGGGCTTTTGCAATCTTACATCTATTGTATACTGTTGTGCATATAC
AGTGAGGATCATTCTATGTGAATTTGAAGCGGTTTGGCGGGAGTTGGTTATAGAATATTAATTCGTTTGCA
TTTGATACATCCTGGTAATTTCTTGTTGAAATCAGATAGTCTATACAACATTGTGACACATGCTTTAGTTAT
AATTTTTTTCGTAATGCCTTTACTTATTGGTGCTTTTGGTAACTGGCTTATTCCTTTAATAATTGGTGCTATA
GACCTTGCTTTTCCGCGTGTTAATAATTTTAGTTTCTGAATTCTGCCTAGTGCCTTTTATTTATTATTATTAT
CAGGTTATGTGGAAGAAGGAGTGGGGACTGGTTGGACTATTTATCCTCCTTTATCTACTGTAGAATACCA
TAGAAGCCCTGCGATGGATCTTGCTATTTTATCACTTCATTTAGCAGGTTCTGGGTCTTTAATAGGCGCTA
TTAATTTCTTAACTTCTAATAAAAATCTCCCTGTTAATAAAATAAAGGGAGAGCGATCTGTCCTGTATGTA
TGAAGAATCACGGTTACAGCCTTTTTGTTGCTGTTATCTTTACCGGTTTTAGCAGGAGCCATCACTATACT
GTTGTTTGATCGTAATTTTAATACCACATTTTATGACCCAATCGGAGGAGGAGATCCTGTGTTATTTATAC
ATCTTTTCTGGTTTTTTGGACACCCAGAAGTGTACATCCTAATTCTGCCGGGGTTTGGTGTAATATCACAC
GTTACTGCGCATTATGCTGGTAAAGAAGCTCCCTTTGGTGCAACAGGTATAATATATGCTATAATCTCTATC
GGAATTATGGGTTTTATTGTTTGAGGGCATCACATATTTACGGTGGGCCTTAATGTAGATACGCGTAACTA
TTTTGGTTCTGCTACCTTAATTATTGCTGTGCCTACCGGAATTAAAGTTTTTAGGTGATTAGCTACTCTGG
CTGGAGGTCGACAAACTTCTAAAACGCCTGTCTTATGAAGTATAGGCTTTATTGTACTATTTACAGTTGG
AGGTTTAACAGGGGTCACTCTAGCATCATCTTCATTAGATGTCTCGTTACACGACACCTATTTTGTTACAG
CACACTTTCATTACGTGCTATCCATAGGTGCTGTGTTTGCTATTTTTTGTGCCTTTACTCATTGGTACCCTA
TGTTTTTTGGTTCTAATTTGCATGGTCGTTGAAGAAAAGGACATTTTTTCTCTATGTTTGTAGCTGTTAAT
TGTACTTTTTTTCCTATGCACTTCTTAGGTTTAAGAGGTATGCCGCGTCGTTATTGCGATTATCCTAATTGT
TACGCTAAATGGCATTGATTAGCTAGTTATGGAGCGGTTATAGGTTATATGTCTTTAATATTCTTCATGTTC
CTATTGTGAGAAAGAGTTGTAAGTAAACGAGGAGTCGTATTTCATTCTGCTCTCTCAACTGAATTGGAGT
GGATAAAACACTTGTTTCCTGTGGTATTGTACGGTTCTAAATATTTTCAAGATGTTATTTATGTGATTGGTG
ATAATCTTCAAAATTTTTTTCAGTATATAATAATGCTTAGTGTATTTATTCTATCTTTTGTTTTAATTATTATAT
ATCGCGTTTGTACAAGTGTCTGGAAATATCGTTATTTTAAACATAGAGAATTGTTAGAATGGGTCTGGAC
TGTGATTCCTATAATCACTTTATTTGTGCTATGGTTTCCTTCTACTATAAACCTTTATGATATAAACCATGGC
GGCGAGCCTAAATGGTCTTTCAAAGCTATCGGACATCAATGGTACTGATCTTACGAGCTTAAGAAGCAAT
TATCTATTGATTCTTATATAGATGTGAGTGCTgagagaGGCTATCGTTTACTTGACGTGGATCAGCGTATAGTT
GTACCTGCGCTGACACAAATTCGTTTATTAGTGTCTAGTGTGGATGTGTTACACTCCTTTGCCATGCCTGC
TTTAATGTTGAAAGTAGATGCAATTCCAGGCCGAATAAATCAACTACCTTTTACGGTAAGACGAACTGGA
GTTTTTTATGGGCAATGTTCAGAGATTTGTGGAGTAAATCACAGCTTCATACCAATTGTTCTAGAATTTAT
TCGTAAGGAAGAGTTTGACCAGTGATTAGTGAAAGTTTGTGAT-----------------------

ATGGTACGCAATCTTTATTATCGAGTTAGCCCTAGTCCATGGCCTTTTATAGTGTCATTTTGTCTTTTAGAT
TCAGCTTTAGGGTTAGTGACGTGAATAAGAGGAGATGATTTAATATTTATTGGTGGTTTTTTATTGCTTTT
GTGTTGTGTAACTCTGTGATGACGGGACCTACTGCGCGAAGGGGACCAAGGATATCACACAAAAAAAG
TGGTTAAAAATTTTCGTGATGGTATAGCTATGTTTATTGTTTCGGAGGTTATATTTTTTTTCTCTTTCTTTTG
AGCCTTTTTTCATAGTAGTCTCAGACCAAATATTGAAGTTAGAGGCACTTGACCTCCTGTTGGGCTTCGT
ATTCCTAATGCTTTTGGGATTCCGATAGTTAACACAGGAATTCTGGTATTAAGAGGAGCTTTTATTAATTAT
TCACTAGGTTCAGTGCGATGTAATTATGACCACGGCGGGATCGCTGGATTGGTCATGGCAATCCTTCTTT
CTTTTATCTTTCTTGCGATTCAATACCATGAGTATCAAGTTAACTCTTTTAGCATAGCTGATGGTATTTATG
GTAGTACTTTTTATATATTAACAGGTTTTCATGGCTTTCATGTATTAGCGGGAACTATGTTTATACTCGTCA
CTCTCGTGCGGATATGGTATGGTCACTTTTTGTCGGATCGATTTTTTGGGTTGCAAGCTTGTGTCTGATAT
TGGCACTTTGTGGACATTGTTTGAATTGGTGTTTGGGTTTTTTTCTATGTTTGAGGCGGAGGTCCTTTTCG
AAAGCGACACTGGTTTTTAAAAATTCTTAGCTATGGACTATATGATTTACCTTGTCCTATCAATCTTAGTG
TATGATGAAGTTTTGGCTCAATATTAGGTTTGTGTTTAACTATTCAAATTGTCACAGGTTTTATGCTTTCAT
TTTATTACGTGCCTCATGCTGATATGGCCTTTGATTCTGTTATTTATATTATACGTAATGTTCATAAAGGGTG
GATAGTACGAAGTATCCATTCTAATGGAGCATCTATGTTTTTTATGTGTATTTACATTCACATCGGTCGTGG
ACTATACTATGGTTCTTATTTAGATAAGGCAGTCTGAAATGTTGGAATTGCTTTATACTTAATGTTAAGCGC
AGAAGCTTTTTTAGGTTATGTTTTACCTTGAGGTCAAATATCTTATTGAGGAGCTGTAGTAATTTCTAGAA
TGTTAACTGCTATTCCTTATGTGGGCCAAACCATAGCTGAGTGGTTGTGAGGTGGTTATGTTGTTAATACT
CGTACTTTAACGCGATTTTATTCGTTTCACTTTATCTTACCTTTTTTAATGGTAGTTGTAGTTCTTTTACACT
TGTTTTATTTGCATGAAAAAGGTAGAAATAATCCTCTAGGTGTAAGTAGGGACAGAATGCTAATTCCTTT
TCATCCTAGATATACAGTTAAAGATATTTTCGGATTTGTTTGTATGCTGTTCGTTCTTATGTTTTTTGTATGT
GTGAAACCAGAAATGTTAGGCAATCCTTTAAACTTTATTCCTGCAGATAGAATAAAAACTCCTGTTCATA
TCCAGCCAGAGTGGTATTTTTTGTTTGCTTATACTATTTTACGGTCTATTCCTCATAAAGCGGGTGGAATT
GTAGCAATGTTGGCTTCTATTCTTGTACTGGCTGTTATACCCTTTGTTCATACAGGAAAGTTTCGTGGGCT
AGCATATTATCCTATTCATCAAATACTTTTTTGGGCTTTTATCTCTGTGTTTTTAGGTATGACTGCTATTGGA
ATGCGTCCAGTAATAGAGCCTATTTATACCGTCGGTCAAATTTTAAGAGTGATTTATTTCGGTCTAATCTTA
TTTATTCCTCTAAGTATATTTATGTGAGATAAGCTAATTTTCGTGGATGTTGTCATAATTGTTTTACCTTTTA
TTTGCGTATTGTTAGCTGTAGGATTTTTTACGTTATTTGAGCGAAAAGTGTTAGCTGCAATTATAATTCGTA
AGGGCCCAAACAAGGTTGGGTATATAGGATTACTTCAACCTTTTAGGGACGCAGGAAAATTGTTTTGTA
AAGAGTTTGTTTATCCTAATCGTGCTAATGTCATACCTTTTTTTTTTGCCCCTTCCTTGATGTTGGGAGTAT
CAATAAGATTGTGAATTTTATATCCTTTAAACTTTGTTGATATAGTATTTGTCTTTGGCGCGCTGCAATTTA
TGGTAACTGCCGGTGTTAGCGTTTTTGGTGTGATAATGGCTGGTTGGGCTTCCAATTCTAAATATGCCTTA
CTAGGTTCAGTCCGAAGAATTGCACAAAGAATTTCGTATGAAATCCCGTTTTGTTTAATTCTTTTTTCTGT
AGCCGTTTTTATCTTAACTTTTATGTTTCAAGAAATTATGTTATTTTTTGTACTGTGTCTTTATTTAAGGGG
TATTAGATTGTGATTGATTTCTATCCTTGCTGAAAACCATCGCGCCCCTTTTGATTTTGTTGAAGGAGAAT
CAGAGCTTGTTTCTGGGTTTAATGTTGAGTATAGTGGCGGTGGCTTTGCTATAATTTTTATATCTGAATAC
AGGAGAATAATATTTAGTAGTATCGTAACAAGTGTTATATTTTTTGGTGGAAGAGAGTTGTTAGTTAGATT
TCTATCTCTTTTTTTCATTTATTTTTTTGTTTGGATTCGTGGGACTTTTCCACGAATGCGCTATGATAAACT
TATGTATTTAGGTTGGACGATTTTTACTATTGTTCCTTTGGTTTATTTGATAGCGATTGTAGTTGTTGGTTAT
GTCtATTTTACAATatgGCTGTAAACCCCATTATGCTTATAAGAATGTTTTTAGTGTTGTTCGGAACTTTGGT
GAGCGTGTTTAGCTCTTCTTGACTGGGGGTTTGACTTGGCATAGAAATTAATCTACTTAATTTTATAGTAC
TCATAAACCCTGACGGTGTTTTTGTTGTTGAACCTGCAGCTAAGTATTTTGTAATTCAATGTGTGGGGTC
TAATTTTATTCTTATAGGTTTTTTATTAAGTGGCGTTTATGCCAATATATCTAATGTTCTTTTAGTAATTGGTT
TAATGTTAAAAAGAGGCGTATGTCCATTTCATGCTTGATTACCTTCAGTGGTCAGTTCTTCTAATTGGTTC
CCCGCTCTATGAATTTTAACGTGGCAGAAATTAGCCCCTTTTGTCTTTATAGGTTGATTTATCAGGAACTC
CATTGTAGCATTTAGAGTCGGAAGTTTAGCACTAGTGGGAGGTATTGGTGGTTTAAACCAGCAAAGGAT
TCGCGGTTTGTTAGCTTATTCTTCATTTGTCCATTCTTCCTGAATAATTTTAGCTCTTATAAAATCATTTTGA
ATTTTTATTCTGTATTGAGTTGTTTACTGTTTTAGCGTTGGAATAGTTTTTTTGAGCGCTGCATCCTATGGA
AAACTTTACTTAAAAAGAAAAGGACGGTTGATTTGAGCTTCATTTGGAGTATTTATATTAATGGGTTTACC
GCCTTTCTTAGGTTTTGCTTGTAAAATTTTGGTATTTCTAAGAATTGATAGTTATATAATTTTCATATGTGTG
GTAGGTTCTCTGATCAGAATGAAATATTATCTAACACTTTCTTATTCTTTTATTCTCGGTCATTGATCTATCA
ATAAAATATCTATTTTTAGCATCTTAATATTGAATTTTATTGGTTTTATAGTAATGAGAGTTTTTTTATTTGTA
atgATGTTGTTTGTATCGGTAGTCTTTGTTTTTATTATTTGTATGTTGTTAAGTGTGTTGGTGTGCTTATCTTT
TTACAAAGTATACGATCGTGAAAAAAGCTCTCCATATGAGTGTGGGTTTGAAAGAATAATATCCGCGCGA
CATCCTTTTGCAGTGCGGTTTTTTTTAGTAGCAGCCATTTTCGTTGTATTTGATGTAGAAATTGCTTTATTA
ATTCCAGTAATCTATGTATTTTTTTATAGTTTTAGGTTAATAACTATGGTTATTGCTTTGGTCTTTGTAGTAC
TATTATTTGTAGGCCTGTTCCACGAACATCGTGAAGGTTCTTTGGATTGAGTGGCTTAAATGTTTTTAGGG
TTATTTTTTAGGTTGATGTATATCCTATTTATTCGCAAGTTTAGAGTGAGCTTGAGCGCTGCGAGATTTCTT
GTTGTAAAATGTATAATGCTGATTAGGCAAGGGTCATCAAGTTTCGAAATAAGGGGGCTTGTGTGATTTG
ATTTTTTAAGTTCTAGCATAGTATGTTTAACCGTATATATTAGTTGTTTAATACTTATTGAAAGTTGTTCAAT
AGCACGTAAAATAGAAATAAAGTTAATTTTAACAGTATTGTGTGGTGTTTTAGTTGGAACTTTTAGATGA
ACTAATCTTTTTTTTTTTTTTTTTTTCTTTGAAAAAGTACTAATTCCAGTCTTATTTTTGATCTTGATCTGAC
GTCTACAGGCTGGTAGTTATATGATTTTGTACACCAGATTGGGTTCTTACCCATTTTTGTTCGCTATTAGTA

TTATGGTGTCTGGCGGTAGAGATATAATGCAGTTAAGCTCTTTTCTAAACCGTGAGTTTTGCTTTTTTTTC
TGGTTCTACATATTAGGGTTCTTGATCAAACTTCCTATGTTTCCTTTTCACTTATGGTTGCCTAAAGCCCAT
GTAGAGGCCCCTGTAGTTGGTTCTATAATTTAGCTGGAGTACTCCTTAAATTAGGTGGTTACGGTATGCT
TCGTTACCTAAATTTAGTCTTAATAAAAATATGTAGGTTTAGTAGAACTTTATTAGCCATTGGTTTAGTAGG
GGGTTTTTTAACTAGAGTGATGTGCATGCGTCAGGTAGATTTAAAGTCCCTGATCGCATATTCTTCTGTTG
GCCACATGAGCTTGGTTATCTTGATTCTAAGTGACAAGATGGTAGGGTTTTTAGGAGCTGTTCTAATAAT
GTTGGGCCACGGCCTGTGTTCCTCTGGACTATTTCTTTTGGTAAATCTGTTTTACAAGCAAAGTGGTTCC
CGTTCTGTATTATACAATAAGGGAAGTCTTATACTTACACCTAGTTTAGCGTTGTTTTGTTTTATACTAAGT
GCTGGTAATATGGCTGCCCCTCCCTCTTTAAACTTTGCTGGTGAAGCCCTTTTGTTCATGAGAAGTTCTG
TGTTAAGGAGATGATTGCTTGTGGTGGTAGGTCTAATAAGTTTTATAAGAGCTTGTTATTCCTTGTTCTTT
TATGGTGTATGTTGTCATGGGAAAAGTTCGAAAATCCGTGATTCTGGTATTTATATATCTAGTATGTTAAAT
TTGTTTTTTCACTGGTTTCCTTTAAATTTTCTATTCTTATTTTTGTAAtaaTATCAGCGAACACATCTGATTAG
AGTATTTTTAGGAATAGAGTTTATTACTCTTAGTGTTATTGCCATAAGAGCTGTGAGTATGACTAGAATAA
GATGTTTTGTTTTGTTAGTTATGTGTATAGCTGTTTGTGAAGCAAGAGTGGCACTAGCTTTGATTGTAAGA
ATAGTTCGAGTAAGGGGCAGCGACAAAAGTGTAAACTTAATTTTGGATAATTTAAAAATGTTATAATAAA
AAGGTTTATAAGAGCTGTTCTAGGTTATGTATTACTTATTATCAGAACAAGTAATAATCTGACAGTACTCA
CATTCTCTTTATTGAGTTACAATGTCTTTGATATTACTTTTTCTTTAATGTTTGATGAAGTAAGCTTTCTTTT
TGTAGGGGTGGTTTTAATTATTTCGGGAAGAGTTGGGGTTTACTCTTTCTGGTATATAGACAATGAAAAG
TTTTTATTGCGATTTTTGTGGCTAATTTTTCTTTTTGTTGGTTCTATAATTATACTAATTTTAGTACCCAATCT
TGTTTGTCTTATACTTGGTTGAGACGGTTTGGGATTGATTTCTTTCTTATTAGTCTGTTATTATCAAAATAG
TAAAAGTTTCAGGGCTGCAATGGTTACTGCTCTAACTAATCGTGTGGGTGATGTGCTTATTTTAATGTGTA
TCGGAATAGGGAGTTTTTATGGAGATTGGATTTTCTATGGGTATAATTACGGGTTTTGTGTAATTATAGCTA
CTTTGCTTATAATTGCCGGAACTACTAAAAGAGCTCAAGTTCCTTTTTCTGCCTGATTGCCTGCTGCCATG
GCAGCTCCTACTCCCGTATCTTCTTTGGTACACTCTTCTACTCTTGTGACAGCAGGTGTTTATTTGTTAAT
ACGTGGGTTTTGATTGTTAGAGCCCAGAAGATTAACAGTGTTAAAAATTATAAGATTAGTTACTATGGTC
ATAGCTGGAACGGTGGCCATTTTAGAAGTGGACTTCAAAAAAATTATTGCTCTATCTACTTTAAGCCAGT
TAGGTGTAATAATATTTTGTTTAAGTATAAAGATCCCTTATGTATGTTTCTTTCACCTAGTTACTCATGCAA
CCTTTAAAGCTTTACTCTTTGTAAGAGCAGGTTGTGTGATTCACTCTAGCTCTGGTAATCAAGATATGCG
GAGCCTGGGCAAGTGTTGAATGGATTTGCCAATTAGGATAAGGTTTCTAAGAGTAGCAAGAATGTCTTT
GGCAGGAATTCCCTTTATGAGAGGGTTTATTCTAAAGACTTAATTATTGAAATAACTAATAGTGACTCTT
TTTTATACATTATTATGATAGTGGGAGTCTCCTTAACTTCTTGATATAGATTTCGTGCGCTCTCAGTGGTTG
CAGGAAATAATAAAAGAGTGATAGGTCTTGTTTGAACCAAAGAAAGGTCTTTATTAGTTATTTCTTACTT
CTCTCTTTTTTTTTGTGCTGTTTTTAGGGGTTGAATCATTAACAGAAAATGTAGGCTAGTTTATTTCCCCG
CTTTCATTGACAGTGTGCTATTTTGAGTGATTTGTTTCATTCCTGTTGGGGTAGTATTTTGGTCCTCCGTC
GATTTGTTTAGTGACTCTCCTCCTAAAGTCAAGTTTGTAGCTTCTATGTGAAATCTTAAAATTAGACAATT
TTTGATCCAACCTATGATAAGGTATAGATACAATGTTTGCCGTTCTTTAGATCAAGGATGATTGGAAATAG
TCGGACCTCAGATTTCTTTATCTAACATAAGTCGCACTAACCAACGTTATTTTTTATCTTTGTTAACTTTTT
TCTTTGTTCTATTTTTTTTTCGTGGGGGTTTGAATGGTTTTGTTTGTAGTGTTTAGTGTTGTCCTTTTAATAA
TTTTATTGTTTATCATAGAACCATATCCTCTTGGTATAATACTGATTTTTAGTTGTGTAGGGCTATGCTACGG
ATTAAGTTATTATGTGAGATCCTTATTGAGGTTTCTTGTTTTTATGAGATATGTAGGGGGGGTCATGGTTCT
TTTTTTATATGTTGTCAGTATTCATCCTAATCAAAAATTTAATGTAAGCTTCCATTTTTTATGAATTAGCTTA
TTTTTAACTTCTTTTTGATTGTTTGGATGATACTTAAGTAGTCTTAAAAGGTTCCATTTTGTTAACGTAGGT
AATTTGTATTTATTAATAGGGGTTATTCTCTTAGTCAATTTATGTGTAGTGTGTAATATTTGTATAAAAAAA
ACACTACCTCTACGTTCTGTT"/>
<sequence id="seq_Crenomytilus_grayanusNC0441281" spec="Sequence"
taxon="Crenomytilus_grayanusNC044128" totalcount="4"
value="ATGTTAATAGATGTTTTTTCTAGGTTTGATGCTCATAGTTATAATTTAATTAGGCTATCTATGTTGTG
ATTGCTTTCGTCTCTTGTTCCTTTAACTGTGATTTTTAGGGATATTAGAGTTCAAGGCTTAGTTCTGTCGTT
TACTTACTCAATAATTCGGAATGGAAAAGGGCTTAAACTTTCTGGGTTTCCGCTAGTAATGAGGGGGGTTG
TTTATAATGGTGTTAATGCTAAATTTATCGGGAAATTTTCCGTTTTTTTTCCCTGTAAGGGGTCAATTTGTG
TTTGGGTTTTCTTTTGCTTTGTCTATTTGAACTTGTTTGGTTGTGTCTAGATTGTTATGCAGATTTGAACA
AGGCTTAATAAGGTTGGTGCCGACTGGACCAATGGTTTTGGTTCCATTCATGGTAGTAGTTGAGTTGATT
AGGGGTATACTTCGTCCATTAACACTGGTGCTTCGGTTAACGTTAAACCTTGGGGCTGGTAAGGTAATTT
TAACTATGTGTAGTGGTGAGCTTGTGGTGAGTTGACTAGTTAGTGGAGTTGGGAGAGTTAAAGGTTTATT
AATAGGAGGCGTTTTCGCTGCTGAGGTTGCGATTGCTTGTATTCAATGTTATATTTTTTGTGTGTTGTTGT
GTCTTTACACTGAGGACCATAGAAGGTAACGATGACTGTGGTCTACAAATCACAAAGATATTGGAACAC
TTTATTTGTATAGGGGAGTGTGAGGAGGGTTATTTGGAGCTAGGTTAAGGCTAATAATTATGCAGGGGCA
TCCTGGAGCGGTGTTTTTAAAAGACTGGTTTTATAACGTAGTGGTTACTACACACGCTTTAATAATAATTT
TCTTTGCTGTTATACCGATTCTTATTGGTGCCTTTGGTAATTGGCTAATTCCTTTGCTTGTTGGAGGGAAG
GACATAATCTACCCCCGTATGAACAACTTGAGGTATTGACTTTCCCCTAATGCACTGTATTTACTAATGTT
GTCTTTTAGAACAGATAAAGGAGTGGGGGCTGGTTGGACTATTTATCCTCCGCTGTCAGTGTACCCCTAT
CATAGAGGGCCAAGGATAGACGTACTGATTGTTTCTTTACATTTAGCAGGGTTAAGGTCCTTGGTTGGGG
CTATTAATTTTGCTAGTACAAACAAAAATATACCTGTGTTGGAGATAAAGGGAGAGCGAGCTGAGCTGTA

TGTGTTAAGAATCAGGGTTACTGCAGTATTATTGATTATTTCTATCCCAGTATTGGGAGGTGGTATTACGAT
GATTTTGTTTGATCGTAATTTTAACACTACCTTTTTTGACCCAGCAGGGGGTGGTGACCCGGTGTTGTTT
CAACACTTATTCTGGTTTTTCGGACACCCAGAAGTGTATATCCTCATTCTTCCTGCTTTTGGGGTAATGTC
TAAGGTAATCATACATTGTTCTGGAAAAGAAGCGGTGTTTGGGTTAATCGGAATGGTATATGCTATGATTG
GAATTGGAGGGCTTGGTTGTATAGTGTGGGCTCACCACATGTTTACAGTGGGGCTAAATGTTGATACTCG
AGGGTACTTTTCTACTGCTACTATAGTAATTGCTGTTCCGACAGGAGTAAAAGTATTTAGATGATTAGCAA
CTATAGCAGGAAGTAAGTTCAAAATGAAGCCTGCTGCTTTTTGAAGAACTGGGTTCTTATTTCTATTTAC
TGTAGGAGGATTAACTGGAGTAATACTATCAAGGGCTTCTATGGATGTATCATTACACGATACTTATTATGT
AGTGGCTCATTTTCATTATGTGTTAAGGATAGGGGCTGTGTTTGGAGTGTTTTGTGGGCTCAATCACTGG
CTACCAAATTTTGTTGGGGTGTGCTTTAATAAGAAGTGAAGAAAAGCTCATTTTATGGCTATATTCTTTGG
GGTAAATACTACTTTTTTCCCTCAACACTTTTTAGGGTTAAGCGGGATACCTCGTCGTTACATAGATTACG
CGGATATTTATGCTCACTGGCATTGGGTGTCCTCTTACGGATCTGCTGTATCCTTTGGATCGCTGATGTATT
TTAAGTTTTTACTTTGAGAAGCATTGGTGAGCCAGCGAGGGATGTCTTTTTATGGGAGTCGATATTTTGG
TGATATTGTGCACGAGTTGGGAAAAGATTTATTTCGTTATCATGGTTTTGTGATGATAGTAGCAGTGGCCG
TGTTAGCCTTTGTAATGTACATAGGGTGTGTTATTCTTTTTACTAAGTACTCTTACCGCCACTTTCTAAATC
GTCAACGATTGGAGTTTTGGTGGACAATTGTGCCTATGCTTTTACTGGTAGGGTTGTGGTTTCCTTCAAT
AATTAATTTATATTATATAGAGGAAGTAAAGCGGCCGCGATGAAACTTTAAAGCTATTGGTAAGCAATGAT
ACTGGTCTTATGAATGCGATGCCTGCTATACAATTGATTCTTATATGGAAGATCAGCAAGAAACTGGGTAT
CGTTTATTAGACGTAGATAATCGTATGGTTGCCCCTGCGGATGTCCAGATAACCGCTTTTGTGAGGAGTT
CTGACGTTCTTCACTCTTTTTCATTACCTAAACTGTTGTTAAAGGTTGATGCTATTCCAGGTCGTATTAAT
CGTCTTCCAATAAAAGCGTCGCAATGCAGTATTATTTACGGGCAATGCTCTGAGATTTGTGGAGTAAACC
ATAGTTTTATGCCAATTGTTATTGAGTTCATTCCTGAGAAATATTTCGTTATATGGTTGGAAGCTCTTAATT
AA-----------------------
ATGAAACGTAATCCTTATTATGTTCCTGGCCCAAGACCATGGCCATTTTTTGTAGCTATTTCTGCTAATGG
TATGGCGGTGGGGTTAATTTTGTGGCTGCATCGTACACCTTTTTTACTTATAGGGAGATTGGGGTGCATAT
TATTAAGTACCTTTAGTTGATGACGTGATTTGATTCGGGAAGGAGATATGGGGCTCCATACTCGTTTTGTC
ATCAAAAGATTTCGGGATGGGGTGGCTTTGTTTATCTTGTCTGAAGTAATATTCTTTTTTACATTTTTTTGG
ACCTTTTTTCATAACGCTTTGAGTCCTTCTTGTGAGCTGGGAATGCGGTGACCTCCTCCTGGAATCCGGA
CACCAAACCCTTCATCTACGAGGTTGTTTGAGACGGGATTATTAATTAGAAGGGGGTTGTTTGTGACTCA
AGCTCATAAGAGTATGCGCTTGGACTATGATGTGGGGCCTTTTGTTGGACTAGTTGTGACAATTTTGTGT
GGAACGTTGTTCTTTTTAGTTCAATTGCGAGAATATTACTGAAATTCGTATACCATCGCGGATAGCGTATA
CGGAAGAGTGTTTTATCTTTTAACTGGTTTCCATGGGATACATGTGGTTGTGGGGACACTGTGGTTAATG
GTGAGACTGATTCGCTTGTGACGAGGGGATTTTTCTAGTCAACGACATTTTGGGTTTGAGGCTTGTATCT
GATATTGGCATTTTGTAGACGTAGTCTGAGTGGCGCTGTGATGTCTAGTGTATGTGTGATTTGGGGGGCC
GTGACGAAAGACTAATAAGCTGGTAAAGATCATGAATGATAGATTCTACGATCTTCCGTGCCCTGTGAAC
TTAAACGCTTGGTGAAGATTTGGGTCTATACTTGGGCTGTGTTTAGTAATTCAGCTTCTGAGAGGGCTTT
TGCTATCTATTCATTATACGGCTCATGAAGATATGGCATTCGACTCTGTTGTGCACATTATACGCAATGTGA
AGAAAGGGTGAATGTTACGGAATATCCATGCTAATGGGTCTTCTATGTTTTTTATTTGTCTCTATGCCCATA
TTGGCCGAGGGCTTTATTACGGATCGTATTTAGATAAAACGGTTTGATACTTTGGGGTCCACTTATTCTTG
TTGACTATAGCTGAAGCCTTTCTGGGGTATACTCTACCATGGGGGCAGATATCTTACTGAGGGGCTACTG
TGATTACGAATATGCTTAGGGTAATCCCGGTGGTTGGAGAAAGAATACTCCGGTATGTGTGAGGAGGCT
GAACAGTGTGTAACGCTACGCTTAAGCGGTTTTATACCCTTCATTTTTTGCTCCCATTTTTGATAGTTGCG
GTTGTTTTTCTTCATCTTTTTTTTCTACATGAGAAGGGGAGAAATAATCCGTTGGGGATTGAAAGAGACA
CAATATGCGTCCCTTTCCACCCTTTTTACACTGTTAAAGACTTGTTTGGGTATGTATGCTTTAGATTCTTTT
TTATATACCTGGTTTGCGTTGATCCTGAGCTCTTAGGCAATCATTTAAATTATTGGCCCGCAAACCCTATA
AAGACTCCAATTCATGTTCAGCCAGAATGGTACTTTATATTTGCCTACGCGATTCTTCGGTCTATTCCGCA
TAAGGCGGGGGGAGTATACATTATGTTTTTATCCATTGTAGTGTTGTACTTGGTCCCGAGTCTTCACAGAG
GTAAGTATCGAAGATTGTGTTTTTACCCGTTTAACCAAGTAGTGTTCTGAGTCTTAGTGGGGAGGTTTAT
TAGACTAACGTGAATTGGGGCCCGTCCGGTTCGTGAGCCTTATATTGTGATGGGGCAGTACTTTTCTGTT
ATCTACTTTTCAAGGCTTCTACTGAATCCGCTTTCTCTATGGATGTGGGATAAGTTGTTAGACGTGGGAGT
GGTGGTTGGGATTGTCCCTTTTATTGGAGTACTTCTTGCGGTTGGGTTTTATACGTTGTTGGAGCGTAAA
ATTTTGGCTATTATTATAATTCGTAAAGGGCCCGCTAAAGTCAGGTATATGGGGATTCTGCAGCCATTCAG
TGATGCCGGAAAACTTTTATGCAAAGAGTTTATTACGCCTACGCGCGCTAACGCAAGACCTTTTGTGTTG
TCCCCTGCTTTAATATTGACTGTGAGTTTACTTGGATGGCTACTATACCCTTATAAATCAGCAGAAGTGGC
TTATGTTTTTGGGGTTATCCTTTTTATGGTAATTACAAGAGTCAGCGTGTATGGGGTGATAATGTCTGGAT
GAGCCTCTAACTCAAAATATTCTCTGCTAGGTGCTGTTCGTGCAATGGCCCAAAGTATCTCCTATGAGAT
TCCAATAGGATTTATTTTTTTTTGTGTAGTTTTATGCTCTGGCGTGTTTATGGTCCAAGAAATTAGAGTCG
GGTTCTTCTTTTTTCCGTTGTCTATAATTCTTGTGGTATGGGTTCTATGCATACTGGCAGAAACTAATCGA
GCTCCTTTTGACTTTGTGGAAGGGGAGTCGGAATTAGTGTCCGGCTATAATGTGGAGTATAGCGGGGGC
GGCTTTGCAGTGATATTTATTGCTGAGTACTCCAGAATTTTACTGAGAAGAATAATAAGAGCTGCAATGT
TTTTCGGAGGCAACGAAGCGTGGATGGGTGTTTATATGATGGTTTTTGCGGTTCTTTTTGTAGTGGTTCG
TGCATCTTTACCACGTCTGCGGTACGATAAACTGATAAGATTATGCTGAACAGTGCTTTTGTGTGTTATGC
TTATAGCCAGAGTGTGTGTAGTAATTCTAATCAGCGTATAATGGTAAGTTTTGTAATAAGACCAATAAAAG

TAATGAGAATACTGATAGTTATTTTGGAACAGGATTAAGGTTGAGAAGAGAAGAGCTAATTGGTGTGT
GACTGGGTATAGAACTAAATTTGTACGGGTTTTTGATTCTTATAAATCCTGACGGCTACTACACACCTGA
GCCCTGTGTAAAATATTTTGTAGTACAAAGCAGGGCATCTATCTTAATGTTAGGGGGTTTTATGTTATTAAT
GCATTTTGTAGTCGGAGGGTTAGTTATAAGGGTAGTGGGCGTTTTATTAAAGTCAGGAGTTTTTCCGCTA
CATTCGTGAGTGCCGTCTACTATTAAAAATAGGAGATGGCTAGCAAGTGGGTTAATATTAACTTGACAAA
AGATTGCGCCTTTGATCTTTTTGTCTGTAATTGCCCCGTTAGGAAGTCTTTGGGTGGTTATCGGGTTAATG
GCTGGTATTGGGGGAGTAGGGGGTTTAAACCAAAACTCTGTCCGTGTGATAAGGGCATACTCGTCATTT
GTCCACACGTCTTGGATATTACTAGGGCTAAGGTGGTCCAGTGTAGTATTTGCAAGGTATTTCGTAGTGT
ATAGAATGTCCGTAGGGATATTTTTTTATGGGTGTTCGCTAATAAATAAAAGAAAAGTGATGAGACAACT
AAGGGGGGCTGCTAGAGGGATAGGTCTCTTAATGCTTATGGGTATACCTCCGTTTCTTGGGTTTGTGGCA
AAGGTGTTGGTTTTTCTCATAACTAGGAGGGGTGTAATTGTAGTGTGTGTTATAGGGTCTGTGGTTAGGC
TAAAATATTATATTGACTTTTTTTATAGAATAGTGATGAAGCATAAGAGAAGGGTTAAGATAATATGGGGT
TTGATAGTAATTGCAAATTTAATAGGTGGGGCGTTGATTGTAATGAGGTTTATCTAAATGGTTATAGGGAC
AAGAGTTTTGTTTGTGTGTATTGTGTCTTTTTTATTTACAGGGCTGTTATTGTTGAGAGAAAAACGGGGG
TTAGACCGTGAGAAATGTAGCCCGTATGAGTGTGGGTTTGAACCCATTGGAAGTGCACGCAGGTCTTTT
TCTATTCGCTTTTTTCTGGTAGCTGTTTTGTTTGTGGTGTTTGATGTTGAAGTGGTGTTGTTGATGCCTTT
TGTGTACATGTTCCTTTATAGTAAGAGGTTACTTGGAATGGTGTCATCAAGAGGCTTTTTATTTATTCTGTT
TATCGGGTTGTACCATGAGTACCGAGAGGGGTCTTTGGAATGAGTGGGATAAATGGTTAAAAGACTGGC
AATTGGGCTTATCGCGTTAATGGTAATGAAAGACCTGAACATATCTATTATTGGATTGAGGGTTTTGACAA
TTATAAGATTGACAATAACAAGGGCAGCCAACGGTAAAATTGAGCTAAATGGGTTATACAGAATTGATTT
CATAATAGGGTTAATAGTCACATTAACTTTATTTATCGCGGTTCTATCTCACTTAAGAAGAATTAAAACGG
TGCGTAAGCCAAGATTCAATCTAATAATTATTGGGATTAGACTAATCCTACTAATGAGTTTCAGGGTAAGT
AGATTCTTCCTTTTTTTTTTTTTTTTTTGAAAGAGTGTTAGCACCATTACTACTGCTAATTGTAGGTTGAG
GATATCAGCCGGAGCGATTGCAAGCAGGTGGactGTACATGGTGATTTACACTGTTTTTGGATTGTTCTTT
CTATGAGGCGTCAGAACGAGGAGAAGAATGGGTGTGGGGAGACTGGTGAAAAAGAGATATATGGGGCT
GTGGTGGTTATATATCCTAGGATTTTTGATAAATTGCCCATATACCCCTTCCATTTGTGATTGCCTAAGGCT
CATGTAGAGGCTCCTGTAGCCGGGTCAATGCTACTAGCTGGTGTGGTGTTAAAATTAGGGGGGTACGGA
TTATTACGGTTTATGAACTTTATGCAGCTAAACCTAAGAAGGGTGTCTTTGCTGTTACTATTTGTTAATTTA
GTTGGTGGGGCCTATGCGGGGATAGTGTGTGTTCGTCAAGTAGACTTGAAATGTTTGGTAGCGTACTCGT
CTGTGGCTCATATGAGATTGGTGTTACTAGCCCTAAGAAACACGCCAGTTGGAGTAATGGGAGCTATTAT
TATTATAGTAGGGCATGGGCTTTGTTCATCTGGGTTGTTTAGGTATGTGAACGTGGCGTACAAGATAAGA
CACTCTCGGTTGTTAGTAATAAATAAAGGCGGGTTATTAATTTGCCCGAGGCTGGTGCTGGTGTGTTTCT
TACTGAGGTCTAGAAATATGGCTGCTCCACCAAGCTTAAACTTATTCGGGGAAGTTTTGGTTTTTGGGGT
GAGGGCGTGAATAAGGATAGTATTTTTGTGTATTTTAGGGCTGATGAGGTTTATTAGAGCTTGTTTTAGTT
TGTATCTGTACAGAAGTTGTTGCCACGGTAAAGGGTTAGTGTATAGAGAGTCTTTGAGACTACTTTGTGA
CGCAATGGTTTTAGGAGCCCACTGAATACCTCTAAACCTACTGTTTTTATTTATACCTTAGCGTATAAACA
CACACCTTCTTTCTTTGTTTGTTGGTCTTGAAATAATAAGATTAGGGTTGCTTTTTGTTGGACACGTTTTT
TTAATAAATCAATTCTGAGTAATCCTTTTAATCTTATGTTTAGCTGTGTGTGAAGCTAGAATTTGTCTGGCT
TTATTAGTTATAGTGATGCGGTTATGCGGTAATGACTTAATGTCAAGGTTAGTGAGAGATGCAAACTATTG
AGTGAAAGTTTTTTGCTTTTGTTGTTAATCTGTGGGTACGTGTCAATCTTTGCAGGCAGGTTGAGTAAGG
TTTATTTGTTTGAGGTGTGTATTTGAGACAGGCATTGTTTGCCCTTTAGATTTAGAATTTTATTGGATAGA
GTAAGAATAATTTTTATTGGGACAGTTTTAGTGATCAGGGGGAGAGTGGCTACTTACTGTAAATGATACA
TATCAAGAGAGGTTTACTATAACCGTTTTATGGGGCTGGTTTGATTGTTTGTGCTTTCTATAATCTTCATGA
TTTTGATCCCTAATCTAGTAATGCTACTAATCGGGTGAGATGGCTTAGGTCTTACTTCATTTTTATTAGTGG
CTTACTATCAAAATAATAAAAGACTCTCGGCAGCCATATTAACAGCTTTGACTAATCGTGTGGGGGATGT
TTTTGTTTTAGTTAGGGTTTCAATTCTTTTGAATGAGGGGAGATGGCTAATTTATGACTATCACCCTGTGT
GCATATGAGCTCTGGGAGGTGTTGTTGTTCTTGCAGGTATGACTAAAAGAGCCCAAATACCTTTTTGTGC
TTGGCTTCCAGCTGCTATAGCGGCTCCGACACCGGTATCTTCTTTAGTTCACTCTTCTACGCTGGTAACA
GCAGGGGTGTACTTAGTACTTCGTTCGTTTTATGTAATTAAAATGGATGTTATGCAAATATTAATGGTTTTT
AGGTTATTCACTTTGGTATTAGCGGGCTCTAGGGCGGTTTTCGCATTTGATCTAAAAAAAGTGATTGCGC
TTTCTACTTTGAGTCAGCTAAGTTTGATGATGTTTTCTATTTCTATTATGTTGCCTTTTGTTGCTTTTTTTCA
TTTGGTTACACATGCGGTGTTTAAAGCTCTTCTTTTTTTAGGAGCTGGAGGGGTTATTCACAGGAACCAG
AGTACTCAGGATATTCGGAGGTTAAGAAGGTTGTGGCAAAGACTGCCTGTGAGAATAAGAGCAATGAG
GGTAGCAATTGTGTCACTGAGAGGTGCTCCTTTCATAAGAGGATTTTATTCCAAAGACTTGATTATTGAG
ATAATGGGTGACAGAACATATGGGTATGTTTTAGAGTTATTAGGTCTTGTTTTTACGTCTTTTTATAGGGCT
CGTGTGTTCAGGGTTATATTAAGGTCAAATTACGTTAACTGTGGGACTTTACGGTCATCTGAGCACATAA
ATATACAAATTCCATTCTTGAGCTTGTATGTTGGTGCTGTTATTTTAGGTGTCAGACTGGGGAAAAGAATG
GAGAAGTTTGGTTTTGTAGTATTACTTGAAAAGTATGAAAGGCTTGTAATTTTTATAATTCCACTTGGGCT
TATATGATGGAGGGTGTTAACTAAATTAAGCTTAAGGCCGTCTAAACGGGGATTTTTTTTGAGAATATGG
CTTGTTGAGTTGACTCACCCTGCAAAGAGTGTGTTTTTTAAGAGCTCGAAGATGGTGTACGAAACTTTA
GATCAGGGTTGGCTAGAATTGTTAGGTCCGCAAGTCGGTCTAAGTAAAATTAGGCAGTTAAATGAGAAC
TATTTTACTGTTGTTTGAGTTGTGTCTGTAAGACTGAGTTTAATTATCTTTATATAAATGAGGGTTATAATT
TTGTGTATAAGTTTGATTGTTGTTTTTGCTTTAATTGCTAAGCAACCAATTTCTCTTGGACTGGTTTTGTTA

GTTGGGTCTATAGTATCATGCGTAGAGATTGCATTGGAAGTTAGGAGATTGTTGGGGTTCTTACTTTTTTT
GACCTATGTAAGGGGGGTTATAGTGCTGTTTTTATACGTATTGAGTATTTATCCTAATGAACGATTTAATTT
AAGATTTATTGTTATTGTAGTAGGATGCTTTGCAGTAAGATTGATGATGATGGTAAACTATGAGAGCGGGT
TTTTGTTTCTTGGGTTTATGGCTGAAAAGAGCCTCTATATTCTAATAGCTAGAGTGTTATTGTTTGTGATGC
TTGTGGTTTCTTATTTATGCATAAAAACTATAGTTCCACTACGGAAAGTG"/>
<sequence id="seq_Gregariella_coralliophagaNC044121" spec="Sequence"
taxon="Gregariella_coralliophagaNC04412" totalcount="4"
value="ATGATAATAGATGTTTTTTCGAGGTTTGATGGGCATAGATTTAATTTCTTTGTTGGAGCTATACTGT
GGTTATTTAGCACTTTAACTCCGTTTATAGTAATTTTTGGTGATGTGACAGTACGTAACTTTTTGTTGGTTT
TTAGCTTTAGTATATTACGTAATGGTAAAGGTCTTATAATTGCTGGGTTGCCGCTTGGGGTGAGAAGGTTA
TTTTTATTAGTGCTTATATTAAATTTGTCTGGAAATGTTCCTTATTTCTTCCCAGTTAGAGCTCATTTTGTAT
TTGGATTTTCATTTGCATTTTCGTTTTGAACTTGCTTGGTAATTTCAGGTATAGTTTCTAGGTTTGAGCAA
AGATTTATAAGACTAGTCCCTAGAGGACCTTTAATTTTAATTCCGTTTATAGTAGTGGTTGAAATTTTTAG
GGGTATGTTACGCCCATTAACATTAGTTTTACGGTTATCATTGAATTTATCTGCTGGTAAAGTAATTTTATC
TTTACTTGGATCAGGGTTATTATCTTCTTTATTAATTTCAAGCTTTTATAATATTTTTGGTGTAATTATTAGA
GGTATTTTTGTAATAGAAATTATTATTGCTTGTATCCAATGCTATATTTTTTGTGTGTTAATTACACTGTATT
CAGGGGACCACAGGGACTAACGTTGGCTGTACTCTACTAATCATAAAGATATTGGTACACTATATCTTCTT
AGAGGGGTGTGGGCAGGGCTAGTTGGTGCAGGTTTAAGTTTAATTATTATGCAGAGTCATCCTAACAGA
ATTTTTTTGAAAGATTGATTTTATAACGTTGTGGTTACTACTCATGCATTAATGATGATTTTTTTTGCTGTG
ATACCAATCTTAATTGGTGCATTTGGTAATTGACTAATTCCTCTTTTAGTCGGTGGTCAAGATATAATTTAC
CCACGAATAAACAACTTAAGGTACTGGCTATCTCCCAATGCCTTGTATTTATTACTCCTTTCTTTTAGGAC
AGATAAAGGGGTTGGTGCAGGATGAACTATTTATCCTCCTCTATCTGTGTACCCTTATCATAGAGGGCCTA
GAATAGATGTTTTAATTACTTCTCTTCATATGACTGGATTAAGATCTTTAGTTGGAGCTATTAATTTTGCTA
GAACAAATAAGAATATGCCAGTATTAGAAATAAAAGGAGAAAAAGCAGAGTTGTATATTCTTAGAATTTC
TATCACTGCAGTTTTATTGATTATTGCTGTTCCTGTATTAGGTGGTGGAGTTACTATAATTTTGTTTGATCG
TAATTTTAATTCTACTTTTTTTGACCCAGCAGGTGGAGGTGACCCAGTTTTGTTTCAACATATTTTTTGGT
TTTTTGGTCATCCTGAAGTTTATATTTTGATTCTTCCTGCTTTTGGTGTAATATCAAAAGTAATTATACATTG
CGCTGGTAAAGAAGCTGTCTTTGGTTTAATTGGAATAGTGTATGCTATAATTGGTATCGGAGGTTTAGGGT
GTATAGTCTGAGCTCATCATATATTTACTGTAGGGTTAAATGTAGATACTCGTGCTTACTTTTCTACTGCTA
CTATAATTATTGCTGTTCCAACTGGTGTTAAGGTTTTTAGGTGGTTAGCTACTATTGGAGGGAGGCGGTTT
AAATTCAAGCCTTCTGGTTGTTGAAGAATTGGATTTTTATTTTTATTTACTGTAGGAGGTTTAACTGGGGT
TATATTGGCCAGCTCATCTATGGATGTGTCTATACATGATACCTATTATGTTGTAGCTCATTTTCACTATGTT
CTAAGGATAGGGGCTGTATTCGGTATCTTTTGTGGGCTTAATCAATGATTACCTAATTTTGTTGGAGTAAA
CTTAAATAAAAAATGAAGAAAAAGTCATTTTTCTGCTATGTTTTTAGGGGTAAATACAACCTTTTTTCCTC
AGCACTTTTTGGGGCTTAGAGGAATACCCCGTCGTTACAGGGATTATGCTGATGTTTATGCGCGGTGGCA
TTGGGTTTCTTCTTATGGGTCTATTGTGTCTTTTGGTTCACTTATATATTTTAAATTTTTATTGTGAGAAGCT
TTAGTTAGTCAACGAGGAATGTCATTTTATGGAAGGTGATTTTTTGGTGATATTATTCAGCAGCAGGGGC
GAGATATTATTGCTTACCATGGATTTGTTATAATGGTAATAATTGCTGTTTTAGTAATAGTACTTTATATAGG
AACAGTAGTTTTAGTTAGAAAAGCTACTTATCGTTATTTTTTAAATCGTCAACGTCTTGAGTTCTGATGAA
CTTTGATTCCTATGATTTTATTGACTGGGCTGTGGTTCCCTTCAATAAAAAATTTATATGTGGTTGATGAAG
TGAAAACGCCGCGCTGGAATTTTAAAGCTGTTGGTAAACAATGGTACTGGTCATATGAGTGCCACCCAA
CGCGAATTATTGACTCTTATATAATAAGAAAGGGGGAAGTAGGTTATCGTTTGTTGGATGTAGACAATCG
AATAGTTGCTCCTGCAGGGGGTTCAAATAACTTGCTATGTTACTAGTTCTGATGTGCTTCATTCTTTTGCAT
TGCCAAAATTGCTTCTTAAAGTAGATGCTATTCCTGGGCGAATCAATCGCTTACCTATAAAGGTATCTCAA
AGATGTGTGTTATACGGCCAATGTTCGGAGATTTGTGGGGTTAATCACAGGTTTATGCCAATTGTTATCGA
GTTTATTCCAGAGAAATATTTTGTTAAATGGATAGATGCTCATTTGtaa-----------------------
ATGAATATAAAACCTTATTATGTTCCTGGGGCAAGACCTTGGCCTTTTTTAGTAGCAGTTGCGTGCAATG
GTATGTGTGTTAGTTTAATCTTATGGCTGCATCGTACTCCATATTGACTAATGGGTAGGTTGTTAAGATTAG
GTTTAAGCTTGGTCAGATGGTGACGTGATTTGCTGCGTGAAGGGGATATAGGGTTACACACACGATTTGT
AGTAAAAAGATTTCGTGATGGTGTTGCTTTTTTTATTTTGTCGGAAGTCATGTTCTTCTTTTCTTTTTTCTG
GTCATTTTTTCATAGGTGTTTAAGTCCTTCTATAGAGCTTGGAGGACGATGGCCTCCGCCAGGGATTCGA
ACACCTAACCCAGTATCTACAGGCTTGTTTAACACTACTCTTTTGATTAGAAGAGGAGTATTTGCTACTTA
TGCCCATAAAAGAATCATTAGAGATTACGATAAAGGCTCTTTTCAGGGGTTAGGATTAACTATTGTGTGT
GGAGTTTTATTTTTTAGAGTACAATTGCGGGAATATTACTGAAACTCCTTCACTATTGCCGATGGGGTGTA
TGGTAGAACTTTTTATATATTAACTGGGTTCCATGGAATGCACGTATTTGTTGGTACTTTGTGATTAATAGT
AAGATTTGGTCGATTATGATGTGGTCACTTTAGAAGACGTCGTCATTTTGGCTTAGAAGCTTGTCTTTGAT
ATTGACATTTTGTTGATGTAGTGTGAGTCTTTGTATGATTGTTTGTATATTTATGATTTGGTGGTCCTTTGC
GTAAGCGTCATGTTTTGTTAAAAATTTTAAATGATAGGTTTTATGATTTGCCATGTCCTGTAAATTTGAATG
TGTGGTGAAGGTTTGGATCAATGTTGGGGTTGTGCCTGGTGATTCAATTTGTTAGAGGGTTACTATTGTC
TGTTCATTATACTGCTCACGAAGATATGGCATTTGATTCTGTTATTCATATTATACGAAATGTAAAAAAGGG
TTGAATGCTGCGAAGAATCCATGCTAACGGTGCGTCAATGTTTTTATATGTATTTATGTTCACATTGCAC
GTGGAATTTATTATGGATCTTATTTAGATGTACCGGTATGAAATATTGGTGTATTATTGTATTTTATGGTAAT
AGCAGAAGCATTCTTAGGGTATGCGTTGCCTTGAGGGCAAATATCTTATTGGGGTGCTACGGTAGTTACA

AACATGCTTACTGTAATTCCTTTATTTGGGGAGAAACTATGCTATTACATATGAGGTGGGTGAACGGTGTG
CAACGCTACGTTACAGCGGTTTTATACTCTTCATTTTATACTTCCTTTTTTAATAGTATGTGTGGCTGTACT
ACATCTTTTTTATCTTCATGAGAATGGAAGTAACAATCCTTTGGGTATTGAAAGGGACACAATATGTATTC
CATTTCATCCTTTTTATACTGTTAAAGACTTATTTGGTTTTGTCTGTTTTAGATGGGTGTTTATATATCTAGT
ATGTGTTGAGCCTGAGTTATTAGGTAACGTGCACAATTATACTCCTGCGGACTCAATAAAAACGCCTCTT
GATGTTCAGCCTGAGTGATACTTTGTTTTTGCTTACTCTATTTTGCGCTCAATTCCTCATAAGGTGGGCGG
TGTTGTTGCCATAGGGGGCGCTATTGCTGTGTTATTAGTAATCCCTGTTATACATACGGGCGAGTTTCGTA
GTCTGTGTTTTTACCCATTTAATCAAATATTGTTTTGGTGTTTAATTGGTAGTTTTATTGGTTTAACTTGAG
CCGGCTCTCGTCCTGCACGAGAACCTTTTATTAGAATGGGGTTATGATTCTCGTGCTCTTACTATGTGTGT
ATTATCTTAAATCCTTTAAGTATGTGAATATGGGATTATCTACTTAAATTATCAGTGCTCGTAGGAGTAATT
CCTGTAGTAGGAGTGCTGTTGGCAGTAGGGTTTTTTACTCTTCTAGAGCGTAAGTTGTTAGCTATTATCAT
AATTCGAAAAGGTCCAGCAAAAGTTAGTTTTATGGGCATTTTGCAGCCGTTTAGAGATGCGGGCAAACT
ATTTTGCAAAGAATTTGTTGTACCATCTCGCGCAGTAATAGCCCCCTTCATTATGTGTCCAGGGGTGATAT
TACTGATCAGATTAGTGGGGTGGCTGTTGTACCCGTATAAATGTGTAGAAGTAGTTTATTTAGCAGGTATT
ATTCAATTTATTGTTGTTGCTAGTATTAGAGTTTACGGGGTTATAGTTGCTGGATGGGCATCTAACTCTAA
ATACGCTCTTTTGGGGTCAGTTCGTGCAATAGCACAAAGAATCTCGTATGAAATTCCTTTAGGTTTTGCG
GTAATAGCAGTTTTATTTGTAGTCAGTTCTTTTATGCTACAAGAAATTAGAATAAGGTTTATTATACTTTTG
TTTCCTGTAATTTTTATTATTTGAATTTTATGCATATTAGCTGAAACTAATCGCGCGCCGTTTGATTTTGTAG
AGGGGGAGTCTGAATTAGTGTCTGGGTTTAATGTTGAATACAGTGGAGGGGGCTTTGCAATAATGTTTAT
GTCTGAATACGCAACTATACTTCTAAACAGGTTAATTACAGCAACAGTGTTTTTAGGCGGAAGGGAGCT
ATTTATAAGAGTAGCTATAATACTGTTTGTAGTAGTATTTGTATGAGTACGAGCATCGTTGCCGCGAATAC
GGTATGATAAATTAATAGGTTTGTGTTGGTCTGTTCTATTATGTGTAGCAATAAGCGCGTGTGTGTTTTACT
TTGTTTTAAGAAATTAGtaATGTTTTTTAGTTTATTAAGCCCTATAATGATGTTAAGCGGTTTTGCAGTATTT
TTTGGGGCTATTATTAGAGTCAGAAGTATAAGGTGAGTGGGGTTGTGGGTCGGAATAGAACTAAATTTGT
TTGGTTTTTTGATTTTTATAAATTTTGATGGAGTCAGAGTCCCAGAGCCGTGTGTTAAATATTTTATCGTTC
AAAGAACCGGGTCTATGTTTTTGGTTATAGGATTTTTGGGGGTAGAATTTGCTTCAGTTATGACAATGTTT
TTAATTGTAAGTGGTGCTACTTTAAAGGCAGGGGTGTTTCCTTTCCACTCTTGAGTGCCTTTAGTGGTTA
AGAATAGAAGATGAATGAGCAGAAGGTTAATTTTAACTTGGCAAAAATTAGCCCCTTTAATAGCAATAGC
TATTGTTATATCTAAAATTTTCCTTTCTATCTTAGTTTTTTTCATAGCATTAATTGGAGGTATCGGTGGATTA
AATCAGTTGTCTATTCGCTTGATAAGAGCCTATTCATCTTTTGTTCACACATCTTGGATATTAGCTAGCTTA
TTAAATTCATTAGTTGTATTTGTTTTCTATTTTTTTATTTATATACTATCAGTTTTATGTCTGTTTTGATGTTG
TGCTAAAATTAATAAATATAGGGTTGTAAGGAGTGGTTTTAGCGCTTCTGCTAGAGTTTGTTTAATTATAC
TGAGAGGGGTGCCCCCGTTTTTAGGATTTTTAGGCAAAATTTTAGTATTTTTATCTGTAATAAGTATAGAA
ATTTTTCCATGCATTATTGGGTCAGTTATTAGACTAAAGTTCTATCTTTCTTTTTTTTATAGAATAGTAATAA
GATATTTAAGTGGAGCTGAAAAAATTATCACCACTTTAGTTGTTATTAATGTTGTCGGGTTAATAGTGGTT
TTTATGATTTTTTAAtaaATGGTATTGTTTTCAATTGTATTATTTATGTTAATTTTATCCACTATTTTCACAGTA
ATATTATTTTTGTCTGAAAAAGGTGGAACAAATCGAGAAAAAAGAAGCCCTTATGAGTGTGGATTTGAG
ACAATTGGAAGAGCTCGTAGTACATTTTCTGTTCGGTTTTTTTTAGTTGCTGTGTTGTTTGTAGTGTTTGA
TGTGGAGGTAGTGTTAGTTATTCCTTTAATTTATATAGTTTGAACAATAAAAAGAGTTTTAGGCATTTTAG
TTGGTCTCATATTTATTTTTGTCTTGTTTTTAGGGTTATTTCATGAGTACCGAGAAGGGTCTTTAAATTGAG
TTTAAtaaATGACTATATCTTTAATTATTGGCACTATTATTTTGATAATAATCGGTAAAATAGAAGCAGCAATA
GCCGGATTAGTTTTACTAATATTTATTATAGTATACGGAGTAGCAAATGCAGCAGAAAGTGTAGAAATCAT
AGGATGCTTTAATTACGACTATGTAGTCTTAATACTAGTGAGATTAACGATGTTTATTATGGTATTATCGTT
AATAATAAGAACAaaaGTAGAGCGGAAACAAGAGTATAACTTGTTTATATTAAGAATTAATCTATTTTTGAT
TATAAGATTTCTTGTAAGAAGTTTTTTTCTTTTTTTTTTCTTCTTTGAAAGAAGTCTATTCCCACTATTAAT
AGTGATTGTAGGGTGGCGACTACAAGCTGGGGCTTATATAATTATCTATACTGTTTTTGGCTCTCTTTTCTT
TTTATTTGGCATTAGATATCTGTTTATCTTAGGGAGTGATAGTATAATTATAGTTTCATTTGTTAATAAAAGA
TTATTAAGATTGTGGTGGTTATTTATTTTAGGGTTCTTAGTAAAGCTTCCAATATACCCCTTTCACTTATGG
CTTCCTAAAGCTCATGTTGAGGCGCCAGTAGCTGGCTCTATGGTTTTGGCAGGAGTCGTGTTGAAATTAG
GCGGTTATGGGCTGTTACGATTTGTGGGGTTGGTAGGCATAAAGAGCTTTAGATTCTCTAGATTGTTATTG
CTAGTTTGTTTAATAGGAGGATTTTATGCGAGACTTGTTTGTTTGCGACAAGTGGATCTAAAGTGTCTTG
TCGCTTATTCTTCTGTGGCTCATATAAGTTTGGTTCTATTGATCATTAGAAACAGTGATCTAGGGGTAATA
GGTGCCCTTCTTATTATAGTTGGCCATGGTTTATGCTCATCTGGGTTGTTTGGATACGTAAACACTATTTAT
AAGGTTTCTAATTCACGTATGTTAATAATAAATAAAGGAGTGTTAATTATTTCTCCGGTAAGAGCTATGGT
ATGTTTTTTATTGAGGTCTAGTAATATGGCTGCGCCTCCTAGACTAAATTTGGCAGGAGAGGTATTTATTT
ATGGCGTAGCTTCTTGAGTAAGTAAAGTATTATTAATATGTGTCATATTAATTAGTTTTATGAGGGCTTGTT
ATAGTTTGTATTTTTATAGGAGTTGTAGTCATGGAAAGACTAGTGGCTACAGCGTATCAAATTTTTTTGAG
TGTGAGATGGTTGTACTTTTGATACATTGATTGCCTTTAAACTTTTTGTTTTTGTTTATTCCTTAACGAAAA
AATGCCCATTTAATAAGCATTTTTATTGGTATTGAGATAGCTGGATTAGGAGTTATTTTTTTATCTACTTTTA
GATTAAGGCATAATGTTTGACTAATTTTTTTGATTGTATGCTTAGGGATTTGTGAAGCTAGATTATGTTTAG
CCTTGTTAGTAATGGTTATACGTTTGTGTGGAAATGACTTAGTAAAAAACCTTAGATTAATAAGTTAACTT
TAAAAAAAAATTTGATCTTTTTATTTGGTGTTACTGGGGTTTTTTGTTTATTGTTTAGCAGAATACCCAATT
CAATACTATTTCAAGCTAGTGTGTGAAAAAGTAATTGTGTGGATTTTAGATTTGAGCTATTATTGGATAGA

GTAAGTGTTTTATTTGTAGGGACTGTACTAATTATTGGGAATAGAGTAATGATTTACATAAAATGATATATA
AGTAATGAGTTATTTTTTTCTCGTTTTAGATGGCTTATTTATTTGTTTTTGATCTCTATAATCTTTATAATTAG
TATTCCTAATTTAATCATACTACTTATCGGATGAGACGGCTTAGGTTTAACATCTTTTCTTCTTGTTGCTTAT
TATCAAAATAATAAAAGATTATCGGCTGCTATACTAACTGCTTTAACTAACCGCATTGGTGATGTGTTAAT
TTTAATAAGTATTGGAATATTCATTAAAGAGATAAACTGAATTATTTATGAGTATTCTCCTGTAGTTTTATC
AGGAATGTGTTTAGCTTTAATCTTAGCTGGGATGACTAAAAGAGCTCAATTTCCATTTTGTGCGTGACTA
CCAGCTGCTATAGCTGCCCCTACTCCTGTTTCATCTTTAGTTCATTCATCTACGTTGGTTACAGCTGGTGT
TTACTTATTATTGCGTAGGTTTGATGTTTTAATTTGTAATAGACTGTGAATGCTAAAATTTTTAAGATTATTA
ACTTTAATTTTAGCTGGATCTAGGGCTTCAGTTACAGTAGATTTTAAAAAAATTATTGCTTTATCTACATTA
AGTCAGCTTAGTGTAATAATATTAGCTGTTTCTATAGGTCTCTCTTATGTAGCGTTTTTTCATTTAGTTACT
CACGCAGTTTTTAAAGCACTTCTATTTTTAAGGGCTGGTAGTGTAATTCATAGATACCGGGGGGTTCAAG
ATATTCGTTTTTTAGGTAAATGTTGACAGGAGTTACCCATTAGAATGAGAGCTATATTAGTCTCAATAATAT
CCTTGGTTGGGGCTCCTTTTTTAAGAGGATTTTATAGTAAACACTTAATCATAGAGATAGAAGACGAATG
TATTTTTATGTATATAATAGAAATAATTGGATTAAGCTATACTTCATACTATAGGAGACGAGTGTTTAGATCT
ATAATCGGCAGAAATAAGTCGGATTTAACAGTTGTCTCTGTAAAAGAGGATATTTTGATTATTATTCCTTT
TTTTATTTTAGGCGCTGGAGCTTTGTTAATTGGAGATATTATAGCTAATAAAAATGAGCTTTTTTTCTTTGC
TGTATTTTCGTCTTCTATTGAAAGGTTGTTAGCTAATATTATTCCATATGGTATAATTATATGAAGAGTAATT
AGTTATTCTGCAGCATTTTATAATAAAATAATGTTTTTTTTAAATATATGACTGGTAGAATTAACTCACCCA
GCTAAAGCAATACTATTTAACTATTCTTTTATAGTTTATCGAGTTTTAGACTATGGATGATTAGAACTAATT
GGGCCCCAATATTCATTTGGAAAGTTTTCTAAGATTAATGAAGGGTACTTTATGGCGTTAAGTCTTTTTGC
AACTATTATAATAATTTTTTTGTTAGTGTAAATGTGAATAATATTAATTTCGTTTGTGTTATTGTTTATTTCTA
GGCTCCTGTTTAATCAGCCCATTTTACTGGGATTTATTTTAATCATAAGGTCTATGCTAGTATGTTTTTTTAT
CGCTGCTGAAATTAGAAGCGTTTTAGGTTTTATAGTTTTTTTAACTTATGTCAGAGGGGTAATAGTTTTAT
TTTTGTATGTGTTAAGAATCTACCCTAACGAAGCTTTCTACATAAAAAATTAGTATTACCTTGGTAGCCTGT
TTGGTGGTTAGACTAGGAACATATTTAAATCGTGATAGAGACTCTTTGTGACTCTCTTTTATATCTAGTGG
GGTGATTTACTTATTTATAGCGTTTATTTTATTGTATGTTATAGTTATTGTTTCTTATTTATGTATAAAAAAAA
TAGCACCGCTTCGGTCCTTA"/>
<sequence id="seq_Modiolus_kurilensisKY2427171" spec="Sequence"
taxon="Modiolus_kurilensisKY242717" totalcount="4"
value="GTTATATTAGATTTGCTTTCTAGGTTTGATtctttTCTTTTAGAATGAGGATTTCCTGCGGGTTGTGGG
TTAGTATGGGAATTAGTTTATTTGTGCTTGTTGGTAGAGATAAAGGGCTTAGAGAGATTATTTTTCATAAG
GTCTTATCTATTTTAAGAGGTATAACTGGTCAATTTGTATCGGGGTTTAACATTAGTGTTATTAGTCTGTTT
GTATTTTTAATTATAGTAAATTTGTTGGGATTGGTTCCTTTTTCTTTTAGAGTTAGATCTCAAGTAGGATTA
GGGCCCTCAATAGCATTTTTTATGTGGTTGTGTTTGTGATTATCTGGATTGCGTGTGTCGTGGCGTCAAAC
TTTGTGTACATTAGTGCCTAGATATCCCATATTTTTAATTCCGTTTTTATTGTTGGTAGAGGTGGTAACAAT
TAGTTCTCGTCCTGTTACATTGGGATTGCGACTAATAATCAACTTAACTGCTGGTCATTTAATTATAGGTAT
GTTAACTAATGTAAATACAAATGTTGTTCTTTGTTCTGCCTATGGGTTGCAGTTTTTAGGGCTTATTGCTAT
GAGTCTTAGTGCTGAAATGGTTATCGGGGGGTTGCAAGCYTTTATTTTTTGTACTCTTTTAGCGTTATATA
GAAATGAACATCCTAGGTAATGATCGGGAATAGTGGGGATTGGTTTGAGAATACTGATTCGAATTGAGTT
AGGTCGTCCTGGAAGAggaTTTTTAGGGGATGATCAGCTGTATAATGTTGTCACTCACGCATTGGTTATAA
TTTTTTTTATAATGCCTTTGATGGTAGGGGGGTTTGGAAATTGACTTCTTCCATTAATAATGGGCTCTGTG
GATATAATTTTCCCACGACTTAATAATTTGAGATTTTGGTTTCTTCCTTCTTCATTATTTATGCTATTAAGGT
CTACTTTTATTGAGAGTGGCTCAGGGACTGGATGGACTCTTTATCCTCCTCTYTCTTCATATACTGGACAT
AGGGGCCCAGCTGTTGACATATCCTTATTTTCTTTACATTTGGCAGGTGCTTCTTCTATTGGTGGTTCTATT
AATTTTTTGACTAGTATAAAAAATATGCCGGTGGAGGTGATGCGAGGAGAGCGGATAATGTTGTTTTTGT
GGTCTATGGTGGTAACAGCTGTTTTACTATTAGTTTCTTTGCCAGTTTTAGCTGGTGGAATTACTATGTTG
ATTTTTGATCGTCACTTTAATACCTCTTTTTATGATCCTGTAGGAGGGGGAGACCCAGTTTTGTATCAACA
TTTGTTTTGGTTTTTTGGTCATCCTGAGGTATATGTACTTATTCTTCCTGGGTTCGGAATAGTGTCTCATGT
TGTAGCACATTGTGCTGGTAAAGACGAGGTCTTTGGGGTTTTAGGAATAATTTATGCTATAGTGTGTATTG
GAGTGTTGGGCTTTATTGTCTGGGGTCATCATATGTTTACTGTCGGAATGGATGTTGATTCTCGGGCATAT
TTTACGTCGGCCACAATAATTATTGCTGTGCCTACGGGTGTGAAGGTTTTYAGTTGATTGGCTACACTTA
ATGGGGGAAGCTTATTGATTGAGACTGCTTTATTATGAGCTGTGGGGTTTATTTTTTTATTTACGGTAGGA
GGTCTTACTGGAATTATACTATCAAACTCTTCTCTTGATGTAGCTATACATGACACTTACTATGTAACAGCC
CATTTCCATTATGTGTTATCGATGGGAGCAGTTTTTGCCTTATTTTGTGGGTTTTTTCATTGGTTTCCTTTAT
TCTATGGTTATTGTTTTCATGAGCGGTGAAGAAAGGCTCATTTCTTTATAATGTTTATTGGAGTAAATTTAA
CTTTTTTCCCTCAGCATTTTCTTGGGTTGAGAGGGATGCCACGTCGTTATTCGGATTACCCAGATTGTTTT
ATGAAATGGCATGTAGTGTCATCATGGGGGTCTTTATTAAGGTTTGTGAGGGTTCTGTATTTTCTTTTTATT
GTATGAGAGGGTCTATTGAGTCAGCGTGGTGTTGTGTGTAAGAGTAATCGCCCTGGAGCAATTGAGTGA
AAAATGTGATGCTGTCCTCTTATGCCGTTGTGAGGTAGAAAAGGTTTTCAAGATTCTTATTATCAAGTAG
GTGAATATCTAAGCTTCTTTCATGAAGGAGTTATATGTTTAATTGTTTTTATTTTATCTGTTGTTTTATATGG
TCTTAGTTGAGTGTTTAGAACAAAAAGAAGTTACCGGTTTTTACGTGAGGCGCAAGGTGTAGAGACGG
CATGGACCATCATTCCTAGATTATGTTTAATTGGTGTAGCAGTTCCTTCTATACACCTTCTCTATGTAATAG
ATGAAATTGGAAGCCCTTCCTTTTGTTTTAAGGCGATTGGTCATCAGTGGTACTGAAGGTACGAGATGGT

AGATGTTTTAGGGTTTGATTCTTTTATAAGCCGTAGAGAAGATGACGGCTATCGACTATTAGATGTTGATC
AGCGAATGGTAGCGCCGTCTAACACTGGGATTCGTTGTATGGTTAGTAGGGCTGACGTAATTCATTCTTT
TGCTATCCCTGGGTGTATGTTAAAAGTAGATGCAATTCCAGGGCGTGTTAACGAAATCCCTATGACTGTG
GCTATAAGGGGGGTTTTGTATGGTCAATGCTCAGAGATCTGTGGTGCTAACCACAGCTTTATACCTATCGT
AGTTGAGTTTATTCCTCCAAGGGTATACAACCGATGGATCAAATCAATCGATGAT------------------------
ATTCCACGAAATCCTTATTATTTAGTAGGTTCTAGTCCATGGCCCGTTTTTACCTCTATTGGTGGGTTATGT
TTAGCAGTTGGATTCGTTTCTTGGTTTCATATACACAGGTATAGTCTTTTGTTTGGGTTGTTAGTTTTAGG
GTTTTCTTTAGGTCAATGATGGCGTGATGTTATGCGTGAAGGAGATTTAGGTTATCATACTTCCTTTGTTG
TAAAAGGTTTGCGTGATGGGTTTATTTTGTTTTTAGTGTCTGAGGTGATGTTTTTCTTCTCTTTATTTTGGG
CTTTTTTCCACATAAGGTTAGCTCCTGATGTGTCTGTGGGGTGTGTYTGGCCTCCTAGGGGAGTAGAGAC
TCTTGATCCTTTTAAAGTTCCTTTGTGTGGTACTACTGTTTTAGTTGGGTCAGGAGCTTCTTTAATATATGC
TCATGCTGCTATTCGGGCTGGTTTAAATAAAGATGCTATTTATGGGACGGGGGCTACTATTTTATTAGGTC
TTTTATTTTCTCGTCTTCAAGCATACGAGTATTATTGAGCTAGATTTACTATTGCTGATAGTGCTTATGGTA
GCTTATTCTATATCATAACTGGGTTTCATGGTATGCATGTAATATTTGGGACTGGGTTTTTAATTGTAAGAT
TGGTGCGATTGTTTCGTTATCGATTTACTCCACGAAATCACTATGGGTTTATAGTGTGTTCTTGGTACTGG
CATTTTGTAGACGTAGTTTGGATTGGGTTGTACGTAGTTGTTTATATTTGAGGTAGTTAGCCATATCGAAA
GAGTCATAGGCTACTAAAATTTGTAAATAATAGTTTGTATGATCTACCAGCACCAGTAAATTTAAGGGTGT
GATGGAATTTTGGATCTATATTAGGTTTATGTTTAGTTATTCAAATTGTTAGAGGGTTGGTTTTGTCACTTC
ATTACACAGCTCATGTTGATATAGCGTTTGATGCAGTAATTCATATTGTTCGTGATGTAAATAAGGGTTGG
ATGATTCGTAGTATGCATGCAAATGGGGCATCAATATTCTTTTTATGTATTTATGCTCATATTGGGCGTGGT
ATTTACTATGGGTCTTATAAGTATAGGGAAGTTTGAAATGTGGGGGTAGTTCTGTATTTGTTGGTAATGGC
AACGGCTTTTTTAGGATATGTTCTTCCTTGGGGTCAGATATCCTATTGAGGTGCAACGGTTATTACGAGTT
TATTAACGGCTATTCCTTATGTAGGGGAGATATTAGTTCATTGAGTTTGGGGTGGGTATTCAGTTTCTAATG
CTACACTGGTGCGGTTTTATTCTTTTCATTTTATTTTACCTTTTATTATTGCGGCTTTTAGGGTAGTACATTT
ATTATTTCTTCATGAAAGAGGGTCAAATAATCCATTAGGTGTTTCAAGTAATGATATGCTTATTCGATTTCA
TCCATTTTATACATCTAAAGACTTAGTTGGGTTTTTAGGCTTATTTTTTATTCTTATACTTCTTGTGTGTTAT
TACCCGGAATTATTGGGGAATGTAAATAATTGAATTCCAGCCGATCCAATGAAGACTCCACTTCATATTGA
GCCTGAGTGGTATTTTCTGTTTGCTTACACTATTTTACGGTCTATTCCTAATAAAGCTGGAGGAGCCCTTG
CTTTGGTTGTGTCTGTTTTGGTTTTATTTGTTATTCCTTTGCTTCATACTGGAAAGTTTCGAGGTCTTTCTT
ATTATCCAGTAAGTCAGGTTTTTTTTTGAGTGTTGATTAACGTTTGATTAGGGTTGACTTGATTAGGGACG
TGCTTTCCGGAGTACCCATTTGAGGAGATTGGTCGGGCGTTGACATGCGGGTATTTTATTATTATTATTTA
ATTCCTTTGACGCAAAGGAGTTGGGATAAGTTAATTGAGATGCAGCTGATTAGATTTATTTTGCCTAGGG
TCTTTGCGCTAGTCGCAGTGGCATGGTACACTCTATTTGAACGAAAGGTGCTTGGATATATTATAAACCGT
AAAGGCCCTAATAAAGTGGGTATATTGGGGTTAATTCAACCATTGGCTGACGGGGTGAAGTTATTTTCTA
AAGAATTTATTCTGCCTACATTCAGTAATATTCTTCCATTCATATTGTGTCCAATTGTTACATTTTTTATTGC
ATTGGTATTCTGATTATTATACCCATTTTCAACAGCAGAGGGTGTGTTTACATGCGGATTATTATTTTATCT
GGCTAATTCTGGTGTGAATGTTTATGGGGTTTTAGTGGCTGGGTGATCATCTAATTCCAAGTACGCATTGT
TGGGATCAATACGTGGGGTAGCACAAAGTGTTTCTTATGAGGTTAGGATGGCCCTTACCCTATTAGGGGG
TGTTTACCTAGTGGGTGTAATGAATTTACAGTCTATAAAGTTGGTGTTTGTAGTTGGAGTAATTATTCCGT
TTGTATGCGTATGATTAATTACAATATTAGCTGAAACTAATCGTGCCCCATTTGACTTTGTAGAGGGTGAG
TCAGAGCTAGTGTCGGGGTTTAATGTGGAGTACAGAAGTGTGGGGTTTGCGCTGATTTATATAGCTGAGT
ACGCTAACATGCTTTTTAATAGCCTTTTTACATGTATTATGTTTCTAGGAGTAAGTGATGCCCTTATAAGTG
TAGAAGCGTGCTTTTTTTTTTTTTTTCTTTATTTGAGTTCGGGGGACTTTACCTCGTTTTCGATATGATATGT
TAATAAGCTTAGCATGAAAGAGGTTTTTAAGTTTAGTATTAAGTGGGGTGTTAGTAATTATTTCTTTAGTT
TGTTTGTTATAAGTGTAAATATTTATAACCCCTTTTACTTACTTAGAGGGTTTTTACTAATTGGGGGTATTT
TAGTAACTTTGAGGACTGATTCTAAAATCAGGGGGTGGTTAGGGATAGAAATTAATGTAATAGGGTTTTT
AGGGGTATTAAGAGTTCGCGGTTTTATAAATATTTCTGTTGGCCTGAAATATTTATTATTCAGGTGCTTGG
GTCAGGCTTATTTTTAATGGGTGTGTTAGTGTTTTATCACTTAATGTTGCAGAGAGGCTGAATAGTAGAG
ATGGGATTATTTTGTAAGGCAGGTATTTTTCCTTTTCATGGGTGGGTTCCATCGGTGATCAATTCAGGGGA
CTGAATTAGTGGATGATTGGTTATGAGGCTTCAAAAGTTGGCTCCTATTATTGTTTCGGTCTGGAGTAGGT
CCgagCTTTTTATTTATATAGGGTTAGTGGGGCTAAGGGTTGTTGGGTCTTTAGGTGGATTAAATCAAATAT
CTGTTCGTGGAATCTTAGCTTATTCTTCATTTGTGCATGGTGGCTGGATGTTGGTAGCGTTAATTCATTCTA
ATGAACTTTTCTTTTTGTATTTTCTAGGCTATATAGTTCAGCTAAGAGTTGTAGTGGGTATTTGTTATGATT
TAGATGTACAGAAAAGAGCAAGGATAAAAATATCTTTTTTGGGGGGAGTAATATCATTAAGTTTAGGGGG
GCTTCCTCCTATAGGAGGTTTTTTGTTTAAACTTAGAGTGTTTCTTTCGGTGAATTTAGGGGTTTTAGTGG
CCCCGGTGGTGGGGTCTGTCGTATCYCTTCTTTTTTACTTGCGCATAATAAATGGGTTTATGTTGGGGAGA
AACAGGTTAGCTAGCTTGTTCTTAGTGTTTAATGGGGTTTGTTATATTGGATTTGGTATAATTTTTGGCTTA
TTCAGTTAAtaaTGGCTGTGGGGTGCTGTGGTAGTAATAGTGGTTCCTTGTATTTTGCTTTTTTTAGGCTTAT
TGGCTATAGGAGATATAAGTagaagtAGTCGAGAAAAGTCTTCTTGTTATGAGTGTGGGTTTGAGCCGATTC
GAACTGCTCGAAGGAGGTTTTCATTGCGATTTTTTCTTTTAGGTGTTTTGTTTGTTGTTTTTGATGTAGAA
GTTGTAATAATGGTTCCTATCCTATTTGGTGTTAGAGTAGGGGGATCAGCAGTAAGGATTATTTGTTTAAT
TTTATTTATTATTGTGTTAGTGGTAGGATGTTTATATGAGCGGCGTGATGGCTCCATAGATTGGATTAAGGA
GATGGTTTTGAGGGGTGTTGCAGTAGTTTTGTTGTTACTTACTTTAGGCGGTGAGGTAGAGGTGATTTTT

GGTGTGCTAATTTGCTCTGTTCTTtttTTTATACCCTTATTAAGTTGGGAAAGTAGGGTTGAAATGGGGGGT
TTGACTAGGCTAGATTTGGTTGGTGTGGTTATAGTAATTTTATCCTTTTATATCAGTATTTTAATGCTTTTGA
GAAGGATTGGAGTTAAGCGGTTTAATGCATTTAGAAAAGTAATTTTGTGTATTTGTGTGGTTTTAGTGTT
GGCGTTTAGTTTTAGGTCTATATTTTTATTTTATATTTGCTTTGAGAGAGTGTTGATTCCAACTTTGCTTTT
AATCCTGGGTTGACGGGTTCAAGCAGTTAACTATATGCTAGTTTATACTGTAGGTGGTTCTATACCCTTAA
TTTATGGATTAAGAAGTCTTTATTGGAGTGGGACTAGTAGGATAATGTTGCTTGGAAGGTTAGATAAGAG
TGTTATTGCTTTTTCTTGATTGTATGTTCTTGCTTTTTTGGTTAAGCTTCCAGTGTTTCCATTCCACTTGTG
GCTACCTAAAGCCCATGTAGAGGCTCCTGTTTCGGGTTCTATAATTCTTGCAGGTCTATTGCTAAAACTTG
GTGGTTATGGGTTTATCCGTTTGTGTGGGTTTGTGGAGTTTCTTCGTTTAAGGGTTCCAGTAATGGTGTTG
TCTGTAAGGCTATTTGGGGGTGTATTAACGAGGGTAATATGTATGCGTCAAACAGATTTGAAGAGTTTGG
TAGCATATTCATCTATTGGTCATATGAGATTTGTTTTATTAGTGGTTACAAATGTTTCATGAGGAGTTTTAG
GAGGGGTTTTAATTATATTAGGTCATGGTTTGTGCTCATCTGCTTTATTTTCTTTGGTAAATTACATGTACG
GAGTTAGTAGTAGTCGTCTTATTAGCCTAAATAAAGGTTATTTACTAATGTCCCCTTCTTTGTCTTTAGTGT
GCTTTTTGTTGGCGGTTAGAAATATGGCTAGACCTCCTAGATTGAATTTGTTTGGGGAGTTATTAATGTTT
ATAGTAGGAAGTTTATTTAGGGTATTAGTCTTAATTTTACTGGGTTTTATAAGGTTTATGGCTGCATGCTAT
AGATTGTATATTTATGTGGGAACACAACATGGGAAAAGGTGTGGGCTATGGGTAAGAATAAATGGTGTAT
GTAGCGGATTTGTATTATTAGCTCATTGATTTCCTTTAAACTTTTTGTTTTTGtttattcctTAGTCGCGTAGAGA
TCACTTAGTTAGAGTGTTTTTAGGTATAGAGTTTATAGCTTTAGGAGTTTTTTTAATAGCTTTTGTCCCGTC
CATTAACAGYCTTTTTATTCTTCTTGTTTTGTTGTGCATAGCGGTTTCAGAGGCAGCTGTTATACTGTCTT
TTATAGTTCAAGTAACTCGTCTTTATGGGAGTGACCAAGTATCTAGGGTTATAGTAGATAGATTCAGTTTT
CTAAAGTTTTAGGTGTGTTGTTTCTAGCTTTAGGGGCGATGCTTCTTAAGGTAGCAAGTAGTTGTGTAGT
CATCATTTTGGAGTTTAGGCTTTGACTCTCTTATATTTCATCAGTAAGTTTTAGATTGTTCTTTGATTGTAG
TGGGTTATTGTTTATTAGAACAGTTTTACTTATTTCAGGTTCAGTCCTAATTTATTGTAGTTGGTACATAGA
CGATGAGATCTATTATAAACGGTTTATTTTTTTAGTATTACTATTTGTTGGTTCAATAGTTTTGCTTATCTCT
ATCCCCAATTTAATTTGTTTGCTAATTGGGTGAGACGGGCTTGGAATTACTTCTTTTTTGTTAGTTGTGTAT
TATCAAAATAATAAATCTCTTGGAGCAGGGATAGTAACGGCATTAGTAAATCGGATTGGTGATGTGTTGC
TCCTGTTTGTTATTGGTGCTTTGGTTAGTGAAGGAAGGTGATTATTATATGAGGGATGCCTTAGGATAAGG
TACCCAATTCCTATTATTTTAATATTTGGGGCTATTACTAAAAGTGCACAAATACCCTTTTCTGCATGGCTT
CCTGCGGCTATAGCGGCTCCAACGCCTGTGTCTTCTCTTGTTCATTCTTCAACCTTAGTTACTGCAGGTG
TTTATTTGTTATTTCGTTGTAATTTCTTATTATTAAGTAGAGGGATAGAAACATTAAAGGTGTTAAGCTTAG
TGACTTTAGTGATAGCAGGTAGTTCAGCATTGGTAGAAGTGGATTTAAAAAAAGTAATTGCTCTTTCGAC
TCTAAGTCAGTTAAGTATGATGCTTTTTGCTTTGTCTTTAGGGTTAGTAGGGGTGTCTTTTTTTCATCTTGT
GACTCATGCCTCTTTTAAAGCTTTACTTTTCTTGTGTGCGGGTGTGGTTATCCACAGAAACCATAAATGT
CAAGACATTCGGTTTTTAGGTAAAAGTTGGCCATTTCTTCCCATTAGGATATCTTGCTTAGTTGTTGCTAA
TATATCCTTATGTGGCTTACCTTTTTTAAGGGGATTTTATTCTAAAGATATGATTATTGAGCTAGTAAAAGG
CGAGGGATTAATTTACTTTTTGGAGGTCTTGGGTACTTTGTTTACTTCTTGATATTCATTTCGTATGTTGAA
TGTAATGTATGGAATAAATAAGGGTGTTAGACGGGTTAGGTTTAGAAGAGAAAGAAATATTTTAAAATTT
GCTTATTTTAGGTTGTTGGTTAGTTCTGTATTAATTGGTTGGGTGTTAGGGCTTTTAGTAGAGCAGCTGAA
TAGAAGGGTCCATCTATTTAGATTTGATAAAGGCTTAGCTAGAAGCTTCGTTTTTGCTATTGTTAGGTACG
GGCTTTCAATTCTTTACCAAGGGAAAAATGCTGGTATGTGGTTCCTTGCAACAATGTGAAATTTGAAACT
AACCCAGATGCCATCAAAAATTTTGATGTTTTATTCAAATGCTGTTGTTGTAAATTTAGAGAAAGGATGG
TTAGAAAAAGTTGGTCCACAAAAATTGATTAGTAGAATTAGAAGATTAAACCAGGGTTACCAACAGTTA
AAAGTACTTAGTGTGTCTTTTATGGCTTTCTTATTTTTTCTAAGAATGAATTTGATTTTTTTTTGTGGTGTA
GTTATATTTTTATGCGTTTTAAGGGTTTGGGAGTCTTTATATATTGGGTTAATTTTAGGGGTTATGTCTATTT
GTGTTTCTGTTATTCTCGGTGGAAGAATAAGAAGGCTGGCAGGGTATTTTGTTTTTTTAATTTATGTGGGT
GGTTTGATAGTGTTGTTTGGGTATGTTTTAAGCGTTTTTCCTAACCAGTACTTTGCCTTTGGTTTTCTTCC
AGGTAAGTTTTTATTTTTGTCTATGGTTtttATTAGACTTTTTAGGATTAGTGGGGCAAGAAAACTGGGGGG
TTTTTTAATATTTAATAGGATATATTTATTTGTTGGTGGATTTTTGTTATTTATTCTTATTTTAGTTGTTTCGTT
ATGTAAGAAGCGCCACTTACCTCTTCGTGGGGGA"/>
<sequence id="seq_Modiolus_modiolusKX8217821" spec="Sequence"
taxon="Modiolus_modiolusKX821782" totalcount="4"
value="GTTATATTAGATTTGCTTTCTAGGTTTGATTCTTTTAGAATGAGTAGAATTattTCTTGCGGATTGTGG
ATCAGCATGGGAATTAGGTTATTTGTGTTGATTGGTAGTGATAAAGGGTTTAGTGAAATTGTTTTACACA
AAATTTTGTCTGTTTTGAGAGGTATAACTGGTCAATTTGTATCGGGATTTAGTATTAGTGTTATTAGTTTGT
TTGTATTTTTAATTATGGTAAATTTGTTGGGACTGGTGCCTTTTTCTTTTAGAGTAAGGTCTCAAGTAGGA
TTAGGTCCTTCAATAGCATTTTTTATGTGGTTGTGTTTGTGGTTATCTGGGTTGCGTGTCTCGTGGCGGCA
GACCTTATGTACTTTGGTGCCTAGATATCCTATGTTCCTGATTCCTTTTTTATTGTTGGTAGAGGTAGTAAC
AATTAGATCTCGCCCTGTAACATTGGGATTACGATTAATAATTAATTTAACTGCTGGGCATTTGATTATAGG
GATGTTGACCAATGTTAATACAAATGTTGTTCTTTGTTCTGCTTATGGATTTCAATTTCTAGGGCTTATTGC
TATGAGCTTAAGTGCTGAGATGGTAATTGGGGGGTTGCAAGCCTTTATTTTTTGTACACTTTTAGGGTTAT
ATAGAAATGAACATCCTAGATAAGGTGTTTGATCGGGAATGGTGGGGATTGGTTTAAGAATGTTAATTCG
AATTGAGTTAGGTCGTCCTGGAAGAttaTTTTTAGGGGACGATCAGCTATATGTCATTACGGCCCATGCTTT
AGTTATAATTTTCATGGTTATGCCTTTAATGGTCGGGGGTTTTGGGAATTGGCTTCTTCCATTAATAATAGG

TTCTGTAGATATAATTTTTCCGCGACTTAACAATTTGAGATTTTGGTTTCTCCCCTCTTCATTATTTATACTG
TTGAGGTCTACTTTTATTGAAAGCGGGTCCGGTACTGGATGGACTTTATACCCTCCTTTGTCTTCATATAC
AGGACATAGTGGCCCAGCTGTTGACATATCTTTATTTTCTTTACATTTGGCAGGTGCTTCTTCTATTGGTG
GATCTATTAACTTTTTAACTAGTATAAAAAATATGCCGGTGGAGGTAATGCGAGGAGAGCGGATAATGTT
GTTCTTGTGGTCTATGGTGGTAACAGCTGTTCTTTTATTGGTGTCTTTGCCCGTGCTGGCTGGCGGTATTA
CTATGCTGATTTTTGATCGTCATTTTAATACTTCTTTTTATGACCCTGTAGGAGGGGGAGATCCAGTGTTG
TACCAACATCTATTTTGGTTTTTTGGTCATCCTGAGGTATATGTACTTATTCTTCCCGGGTTTGGGATAGTA
TCTCACGTTGTAGCTCACTGTGCTGGTAAAGATGAAGTTTTTGGGGTTTTAGGAATAATTTATGCTATAGT
GTGTATTGGGGTTTTAGGATTTATTGTGTGAGGCCATCATATGTTTACTGTCGGAATGGACGTTGACTCCC
GAGCATATTTCACATCGGCTACCATAATTATTGCCGTACCTACTGGTGTAAAGGTTTTTAGTTGATTGGCT
ACCCTTAATGGGGGTAGGTTGCTAATTGAGACTGCTTTGTTGTGAGCTGTTGGGTTTATTTTCTTGTTTAC
GGTAGGAGGCCTTACTGGAATCATGCTTTCCAATTCTTCCCTTGATGTGGCCATACATGATACTTATTATG
TAACAGCTCACTTTCATTACGTACTATCGATGGGAGCAGTTTTTGCTTTGTTTTGTGGATTTTTTCATTGG
TTTCCTCTGTTTTATGGATACTGTTTCCACGAGCGGTGAAGAAAGGCCCATTTTTTTATAATATTTATTGG
AGTAAATTTAACCTTTTTTCCTCAGCATTTCTTGGGATTGAGAGGAATACCACGCCGTTATTCAGACTATC
CAGACTGTTTTATGAAATGGCATGTAGTTTCTTCATGGGGGTCTTTATTAAGTTTTGTAAGGGTTCTGTAT
TTTCTTTTCATTGTATGAGAGGGGTTATTGAGTCAGCGTGGTGTTGTGTGTAAGAGTAACCGTCCTGGAG
CAATTGAGTGAAAAATGTGGTGTTGTATGCCGTTGTGGGGTAGAAAAGGTTTTCAAGATTCTTATTATCA
AGTTGGTGAATACCTAAGCTTCTTTCATGAAGGAGTTATGTGTTTAATTGTTTTTATTTTATCTGTTGTTTT
ATATGGACTTGGTTGGGTGTTTAGAACAAAAAGAAGCTACCGGTTTTTACGCGAAGCTCAAGGTGTAGA
GACGGCATGAACCATTATTCCTAGTTTATGTTTAATTGGTGTAGCAGTTCCTTCTATGCATCTTCTTTATGT
TATAGATGAAATTGGAAGTCCTTCTTTTTGTTTTAAAGCAATTGGCCACCAGTGATATTGAAGGTACGAG
ATGGTAGATGTATTAGGGTTTGACTCCTTTATGAGTCGGAGAGAAGATGATGGCTATCGATTATTAGATGT
TGATCAGCGAATGGTAGCGCCTTCTAACACTGGGATTCGTTGTATGGTCAGGAGGGCTGATGTAATCCAT
TCTTTTGCTATCCCTGGGTGTATGTTAAAAGTAGATGCAATTCCAGGTCGTGTTAATGAAATCCCTATAAC
TGTGGCCATAAGAGGGGTTTTATATGGTCAATGTTCAGAGATTTGTGGTGCTAATCATAGTTTTATACCTA
TTGTAGTTGAGTTTATCCCTCCAAGGGTATATAATCGATGAATTAAATCTATTGAGGAT------------------------
ATTCCACGAAATCCTTATTATTTAGTAGGTTCTAGTCCATGGCCTATTTTTACTTCTATTGGTGGGTTGTGT
TTAGCAGTTGGATTTGTTTCTTGGTTTCATGTGCACAGGTATAGTCTTTTGTTTGGATTCTTAGTTTTAGGT
TTTTCTTTAGGTCAATGATGGCGTGATGTTATGCGTGAAGGAGAGTTGGGTTATCATACTTCCTTTGTTAT
AAAAGGTTTGCGTGATGGTTTTATTTTGTTTTTAGTATCTGAGATTATATTTTTCTTTTCTCTCTTTTGGGC
TTTTTTTCATATAAGGTTAGCCCCTGATGTTTCTGTTGGGTGTGTGTGGCCTCCTAGGGGGGTAGAGACC
CTTGACCCATTTAAAGTTCCTTTATGTGGTACTACTGTTTTAGTTGGGTCAGGTGCTTCTTTAATATACGC
CCATGCTGCTATTCGGGCTGGGTTAAACAAAGATGCTATTTATGGGACAGGAGCCACTATTCTATTAGGT
CTTTTATTCTCTCGTCTTCAAGCATATGAGTATTATTGAGCTAGGTTTACTATTGCGGATAGTGCTTATGGC
AGCTTGTTTTATATTATGACTGGGTTTCATGGGATACATGTAATATTTGGGACAGGGTTTTTAATTGTGAGT
TTGGTACGGTTGTTCCGTTATCGATTTACTCCACGCAATCATTATGGGTTTATAGTGTGTTCTTGGTATTGG
CACTTTGTAGACGTAGTTTGGATTGGGCTGTATGTAGTTGTTTATATTTGAGGTAGTTagCCATATCGAAAG
AACCATAGGCTACTGAAATTTGTAAATAATAGTTTGTATGATTTACCAGCACCAGTTAATTTAAGGGTGTG
ATGGAATTTTGGATCTATGTTAGGTTTATGTTTAGTTATTCAAATTGTTAGTGGGTTGGTGTTGTCGTTTCA
TTACACAGCTCATGTTGATATGGCGTTTGATGCAGTGATCCATATTGTTCGTGATGTAAATAAGGGTTGAA
TGATTCGGAGTATACATGCAAATGGGGCTTCAATATTTTTTTTATGCATTTATGCTCATATTGGTCGTGGAA
TCTACTATGGGTCGTATAAGTATAGGGAAGTTTGAAATGTAGGAGTGGTTTTGTACTTGTTGGTAATGGC
AACGGCTTTTTTAGGTTATGTTCTCCCTTGGGGTCAGATGTCTTATTGAGGTGCAACAGTTATTACTAGCT
TATTAACAGCTATCCCTTATGTAGGCGAAATATTAGTACATTGAGTTTGGGGGGGGTATTCGGTTTCTAAT
GCCACATTGGTGCGGTTCTATTCCTTCCATTTTATTTTACCATTTGTTATTGCGGCTTTAAGGGTTGTTCAT
TTGCTATTTCTTCATGAAAGAGGTTCAAACAATCCATTAGGAGTGTCGAGTAATGATATACTTATTCGATT
TCATCCTTTTTATACGTCCAAAGATTTAGTGGGGTTTTTTGGTTTATTTTTTCTTCTTATAGTTCTTGTATGT
TACTACCCAGAGTTGCTGGGGAATGTAAATAACTGAATTCCAGCTGACCCAATGAAGACTCCACTTCATA
TTGAGCCTGAGTGGTATTTTCTGTTCGCTTATACTATTTTACGGTCTATTCCTAATAAAGCTGGGGGGGCT
CTTGCCTTGGTTGTGTCTGTTTTTGTTTTATTTGTTATTCCTTTACTTCATACTGGAAAATTTCGAGGTCTT
TCTTATTATCCAGTAAGTCAAGTTTTTTTCTGAGTGTTGATTAACGTTTGATTAGGGTTGACTTGGTTAGG
GACATGCTTCCCGGAGTACCCATTTGAGGAGATTGGTCGGGCGTTGACTTGTTGATATTTTATCATTATTG
TTTTAATTCCTTTGACGCAAAGAAGTTGAGACAAGTTAATTGAGGCTGAGTTTGTAGTACTGATTTTACC
TTTTGTATGTGTTTTGGTTTCGGTAGCTTTTTATACCTTATTGGAGCGTAAAGTTTTAGGTTATATTATAAG
TCGGAAAGGGCCAAATAAGGTGGGGTTTATAGGGGTCATTCAACCTTTTAGCGATGCCGCAAAATTATTT
ACTAAAGAAATAGTTATGCCTGGGTTTTCAAACAACATGGTTTTTGTGCTATGCCCTGGCTTGATCTTACT
TAATGGGTTGAGATTATGGCTTCTTTATCCTTTTAGTTATACAGAGGTGGTATTTGTTTGTGGGTTAATTCA
GTTTCTAGTGGTCTCAAGAACTAGTGTTTATGGGGTGATGCTAGCAGGCTGGTCTTCTAACTCTAAATAT
GCTTTATTAGGTTCCGTACGTGCTGTTGCGCAAAGAGTTTCTTATGAAGTGCCTTTAACATTTGTGATAAT
TATTATTTGTTGCTTGTTAGGAAGAATGCTCTGTCAAGAAGTAAAAGAAGTTTTTAGTTCTATTTTATTTG
GTTTAGGCGGAATAATTTGGTTAGTGTGTATATTGGCTGAATCTAATCGTGCTCCTTTTGATTTTGTTGAA
GGTGAATCTGAGTTAGTTTCTGGTTTTAATGTGGAGTATAGAAGAGGAGGCTTTGCGATAGTTTTTATAG

CAGAGTATGCAGCAATACTGTTTAATAGTCTTTTTTTTACCGTTTTATTTGTTGGGGGCAGAGGGGTAGTG
GTTATATTAGGGATAACGTTAGTTGTAGTGGGTTATGTATGAGTTCGCGGGTCTTTTCCTCGTATGCGGTAT
GATAAAATAATAAAACTGTGTTGACGTTACTTAACGGTTTTAGTCCTCTGTTTAGGTGTTTTTTGTCTGTG
TGTCCCTTACTTATAataagtATAAATATTTATAATCCCTTTTATTTGCTTAGAGGGCTTTTATTGATTGGAGGA
ATTTTAGTAACTTTAAGTACTGATTCTAAAATTAGGGGCTGGTTAGGAATAGAGATTAATGTAATAGGGTT
TTTAGGGGTGTTAAGGGTTCGGGGTTTTATAAACATTTCTGTTGGATTGAAATATTTTATTATTCAGGTGC
TTGGGTCAGGCTTATTTTTAATGGGTGTGTTAGTATTTTATCATTTGATGTTGCAGAGAGGGTGGATAGTA
GAAATGGGGCTATTTTGTAAAGCAGGTATTTTTCCCCTTCATGGATGGGTTCCATCAGTTATTAATTCAGG
GGATTGAGTTAGTGGGTGGCTGGTTATAAGGGTTCAAAAGTTGGCTCCTATTATTATTTCAGTCTGGAGT
AGGTCTgagTTTTTTATTTATATAGGGTTAGTAGGATTAAGGGTTGTTGGGGCCTTAGGTGGGTTAAATCAA
ATGTCTGTCCGTGGAATTTTAGCCTATTCTTCCTTTGTGCATGGTGGGTGGATACTGGTGGCGTTAACTCA
TTCTAATGAACTTTTCTTTTTATATTTTTTAGGCTATTTAGTTCAGTTAACAGTTGTAGTAGGGATCTGTTAT
GATTTAGATGTAGAGAAAAGAGCAAGGAGAAAAATATCTTTTTTAGGTGGAGTGATATCATTAAGTTTAG
GGGGTCTTCCTCCCATAGGAGGCTTTTGTTTAAACTGAGGGTGTTTCTTTCGGTGAATTTGAGGGTGTT
AGTGGTCCCAGTAGCGGGGTCAGTCTTGTCGCTTCTCTTTTACTTACGGTTGATAAACGGGTTTATGTTG
GGGAGAAATAGTTTAGTAAGGTTGTTTCTGGTGTTTAATGGGGTCTGTTATATAGGATTTGGGATAATTTT
TGGTTTATTTAGTTAGtaaGGTTTGCTTTGTATTTGGTTTATAGTGGCTGTGAGGTGCTGTTTGTATTTTACT
TTTTTGTTATGGCTGGCTATAGGAGATAGAAGTAGTCGAGAAAAGTCTTCTTGTTATGAGTGCGGGTTTG
AGCCAATTCGAACTGCTCGAAGTAGGTTTTCATTGCGATTTTTTCTTCTAGGGGTTTTGTTTGTTGTTTTT
GATGTAGAAATTGTGATAATGGTTCCTATTTTATTTGGTGTTAGAGTGGGGGGATCAGCATCTAGAGTTGT
TTGTTTAGTTTTGTTTATCATTGTGTTAGTAGTGGGGTGTTTATATGAGCGGCGGGATGGCTCTATAGATTG
AATTAAAGAGATGGTTTTGAGGGGTGTGGCAGTAGTTTTGTTATTATTTACCGTGGGTGGAGAAGTAGA
GGTAGTCTTTGGGGTGTTAATTTGTACTATTCTTTTTATGCCTTTGTTGAGATGAGAAAATagtAGGGTTGA
AATGGGGGGTTTAACTAGATTGGATTTAGTTGGTGTGATCATGGTAATTTTGTCCTTTTATATTAGGGGTC
TGATGCTTTTGAGAAGAGTTGGAGTGAAGCGGTTCAATGCATTTAGAAAAGTAATTTTGTGTATTTGTGT
GGTTTTAGTGTTAGCGTTTAGTTTTAGGTCTATATTTTTATTTTATATTTGTTTTGAAAGGGTGTTAATCCCA
ACTTTGCTCTTAATTTTGGGTTGACGGGTTCAAGCGGTTAATTACATGTTAGTTTATACTGTAGGTGGTTC
TATACCTTTAATTTATGGCTTAAGAAGTCTTTATTGGAGAGGAACGAGTAGAATAACCTTGCTTGGAAGT
TTAGATAAAAGTGTTATCGCTTTCTCCTGGTTATATGTTCTTGCTTTTTTGGTTAAACTTCCGGTGTTTCCG
TTTCATTTGTGATTACCTAAAGCTCATGTAGAGGCTCCTGTTTCGGGTTCTATGATTCTTGCGGGGTTGTT
GTTAAAACTTGGTGGTTATGGGTTTATTCGCTTATGTGGGTTTGTTGAATTTCTTCGTTTGAGGGTCCCAG
TGGTGGTTTTGTCTGTGAGGCTATTTGGGGGTGTATTAACTAGGGTAATGTGTATGCGTCAAACAGACCT
AAAAAGTTTGGTAGCGTATTCATCTATTGGTCATATGAGGTTTGTTTTATTAGTTGTTACAAATGTTTCATG
AGGAGTCTTAGGAGGGGTTTTAATTATATTAGGGCATGGGTTGTGTTCATCTGCTTTGTTCTCTTTGGTAA
ATTACATGTATGGTGTTAGTAGTAGTCGTCTTATTAGATTAAATAAAGGGTATCTACTTATATCTCCTTCTTT
GTCTTTAGTGTGTTTTTTGTTGGCAGTTAGAAATATAGCTAGACCTCCCAGGTTGAATTTGTTTGGGGAG
TTATTAATGTTTATAGTAGGCAGGGTTTTTAGGGTGTTAGTTTTAGTCTTGTTAGGATTTATAAGGTTTATG
GCTGCTTGCTATAGGCTATATATTTATGTGGGAACACAGCATGGTAAAAGGTGCGGTTTATGAGTAAGAA
GAAGCAGTGTATGTAGGGGACTTGTGCTGTTAGCTCATTGGTTTCCTTTGAACCTTTTATTTTTGTTTATT
CCTTagtTCACGTAGAGCTCATTTAGTTAGAGTGTTTTTAGGCATAGAGTTTATAGCTTTAGGGACTTTTTTA
ATAGCCTTTGTTCCGTCCATAAATAGGCTTTTTATCCTTCTTGTTTTGTTGTGCATAGCGGTTTCAGAGGC
AGCTGTTATATTGTCTTTTATAGTCCAAGTGACGCGCCTTTATGGGAGAGACCAAGTATCTAGGGTTATGG
TAGATAAATTCAGTTTTCTAAAGTTTTAGGTATAATGTTCTTAGTTTTAGGGGTGATACTCCTTAAGGCGG
CAAGTAGTTGTGTGGTGATTATTTTGGAGTTTAGGCTTTGACTTTCCTATCTTTCATCTGTAAACTTTAGA
CTATTCTTTGATTGTAGTGGGTTATTGTTTATTAGAACTGTTTTACTTATTTCAGGTTCAGTACTAATTTATT
GCAGTTGGTACATAGATGATGAGGTCTACTATAAGCGGTTTATTTTTTTAGTGTTATTGTTTGTTGGCTCA
ATAGTTCTACTTATTTCTATCCCCAATTTAATTTGTTTGTTAATTGGGTGAGATGGTCTAGGAATTACCTCT
TTTTTGTTAGTTGTGTATTATCAAAATAACAAGTCTCTTGGAGCAGGGATAGTGACGGCGTTAGTAAATC
GAATTGGGGATGTGTTGCTTCTTTTTGTGATCGGTGCTTTGGTTAGTGAAGGGAGGTGGTTGTTATATGA
AAGGTGCCTTAGATTAAGGTATTTTGTTCCTATTATTCTAATATTTGGGGCTATTACTAAAAGTGCTCAAAT
GCCTTTTTCTGCATGGCTTCCTGCGGCTATAGCGGCTCCAACTCCTGTGTCTTCTCTTGTACATTCTTCAA
CTTTGGTTACTGCGGGTGTTTATCTGTTATTTCGTTGTAATTTCTTATTATTAAATAGGGGAGTAGAAACAT
TAAAGGTTCTTAGCTTAGTAACTTTGGTAATAGCAGGTAGTTCGGCATTGGTAGAAGTAGATTTAAAAAA
GGTAATTGCTCTTTCGACTTTAAGTCAGTTAAGTATGATACTTTTTGCTTTATCGTTAGGGTTAGTAGGGG
TATCTTTTTTCCATCTTGTGACTCATGCTTCTTTTAAGGCTTTACTTTTCTTGTGTGCGGGTGTAGTTATTC
ACAGTAATCATAAGTGTCAAGATATTCGGTTTTTAGGTAAAAGGTGGCCATTCCTTCCTATTAGGATATCC
TGCTTAGTTGTTGCTAATATGTCTTTATGTGGTGTTCCTTTTTTAAGGGGGTTTATTCCAAAGATTTAATT
ATTGAGTTAGTAAAAGGTGAGGGGTTAATTTACTTTTTGGAGGTTTTGGGTACTTTGTTTACCTCGTGGT
ATTCGTTTCGTATGTTAAACCTAATGTATGGAATGAACAAAGGGGTTAGACGGGTTAGGTTTAGTAGGGA
AAGGAATATTTTAAAGATTGCCTATTCTAGGTTGTTAGTTAGTTCTGTGTTGATTGGTTGGGTGTTAGGGC
TTTTAGTAGAACAGTTGAATAGAAGGGTTCATTTATTTAGATTTGACAAAAGCTTAGCTAGAACTTTCGT
TTTTGCTGTCGTTAGGTATGGGCTTTCAATTCTTTACCAAAGGAAAGCTGGGGGTATATGATTTCTTGCAA
CAATGTGAAATTTGAAATTAACTCAAATACCATCAAAACTTTTGATGGCTTATTCAAATGCTGTTGTTGTA

AGATTAGAGAAAGGATGGTTAGAAAAAGTTGGGCCGCAAAAATTAATTAGCGGAATTAGAAAACTAAAT
CAGGGTTATCAGCAGTTAAAGGTACTCAGTTTGTCTTTCATGGCTCTTTTGTTTTTATTAAGGAATGAATT
TGGTTTTTTTTGTGGTGTAGTTATATTTTTATGTGTTTTAAGGGTTTGGGAGTCTTTATATATTGGATTAATC
TTAGGGGTTATGTCTATTTGTGTTTCTATCATTTTGGGGAGGAGAATAAGAAGGCTGGCAGGGTATTTTGT
TTTTTTAATTTATGTAGGGGGTTTGATAGTGCTTTTTGGGTATGTTTTAAGAGTTTTTCCTAATCAGTACTT
TGCTTTTGGTTTTCTGCCTGGTAAGTTTTTATTTTTGTTTATGGCTTTTattAGGCTTTTTAGGGTAAGTAGG
GCAAGAAAACTTGGGGGTTTCTTGATGTTTAACAGGATATATTTATTTGTTGGTGGTTTTTTATTATTTGTT
CTTATTTTAGTTGTTTCTTTATGTAAAAAGCGTCATTTACCACTTCGTGGGGGG"/>
<sequence id="seq_Musculista_senhousiaGU0019531" spec="Sequence"
taxon="Musculista_senhousiaGU001953" totalcount="4"
value="ATGATAACTGATGTGTTTTCTGTTTTTGATGAACATTACATGAGAAGAAGATATGGCTACTATCTAT
GATTTACGTGTGTTGGGATTCCTTTAGTACTCTTGTTTTGTGACTTAGTGCTTCGTGATAAACTGTTAGAA
GGCTCTTTCATACTTATGAAGAGTATTAACTGTTTTAAGTTAAGAGGGTTTCCTTTAGGTATTAGGAGGTT
ATTGATATTGATCCTTACTGTTAACGTATTTTCTCTATTTCCCTATGTTTTTAAGGTTAGTGCACATTTTTCA
TTTGGTAGGAGGTTCTCTTTGAGTATATGAAGTGCCATTATTATTTCTAGTGCTTTTTGAAGGTTTGAGCA
AACTTTTGCTATATTGGTACCGTATGATCCGGTGGTAATAGGCCCTTTTATGGTGTTAATTGAGTGTGTTTC
GCATCTTCTTCGTCCTATGTCTTTATTTATTCGACTAGCAATAAACTTAGCTACTGGAAAAGTTATAATAGT
AATAGCTACCTCTTTAGGTTTAAGCCTGCTTATTGTGGGGGAGCTTCTTGTAACATTCTTAGTTTTACTAG
GTAGAATAATGTGTTTTGAAGCTGGAAGGGGTGTTGCTCAGGCATTTATTTTTTGTTTTTTGTTATCATTAT
ATGCATCAGAGCATAGTGAGTAGGCTGGTGCAAGTTTAAGTCTTCTTATCCGAGTTCATTTATCTCATCCT
GGTAATTACTTTATTAAAAGAGAGTCATTTTATAATGTTGTTGTTACAACAGCTCTTATAATCTTTTTTGCT
GTAATGCCTTTGATTGGTGCTTTTGGTAATTGGCTGATTCCTTTGTGTATTGGAGGAGGTGACTTAGTGTT
CCCTCGTCTAAATAATCTGAGGTATTGGTTAGCTCCTAATGCTTTGTATTTATTAATGATTTCTGTTTTTACT
GAAAAAGGGGCCGGTACAGGGTGGACAATTTATCCTCCTTTATCTTCTGTTGAATTTCATAGAGGTCCTG
CCGTAGACATCTTAATTACTTCATTACACGCAATTGGTCTTAGTTCATTAGTAGGGGCTATTAATTTTGGGT
GTACTAATAAAAACATACCTGTTCCTAAAATGAAAGGGGAAACGTCTGAGCTTTATTTATGAAGGCTAAC
TGTTACGGCTGTTCTTTTGATTATTTCTGTTCCTGTTTTAGCAGGAGGTATTACTATGCTGTTGTTTGACCG
TAATTTTAATAGAACTTTTTTTGACCCTATCGGAGGGGGTGATCCTGTTTTGTTTCAACATTTATTCTGGTT
TTTTGGTCACCCAGAGGTTTATATTTAATTTTACCTGCTTTTGGTATTATATCTAAAGTAGTAATAAATCA
AAATGGTAAAGAAGCTGTGTTTGGTCAAGTAGGGATGCTTTATGCTATAGTTGGTATTGGGGGGCTAGGT
TGTGTTGTTTGAGCTCACCATATATTTACTGTTGGGATAAATGTAGACTCACGTGCTTACTTCACTAGCTT
GACAATGGTTATTGCAGTACCAACGGGTGTAAAAGTATTTAGGTGAATAGCTACTATAAGTGGTGGTAAG
TTTAGGATATCCCCAAGAGGGGGTTTGAAGTCTAGGGTTTTTATTCTTATTTACAGTAGGGGGTCTAACAG
GTGTTATGCTTTCTAGATCTTCTTTAGATGTCTGTTTACATGACACTTATTATGTGACGGCTCACTTTCATT
ATGTGTTGTCAATAGGAGCTGTTTTTGGTATTTTCTGTGGGTTAAATCATTGATTTCCTTTATTTTTTGGGG
TTAATTTGAATAAAAAAATGGTCACTGACACATTTTTTCATTATGTTTGTAGGTGTTAATATAACCTTTTTTC
CCCAGCATTTTTTGGGGTTAAGGGGAATACCCCGTCGTTACTGTGATTATGCTGATTGCTACGCTAAGTG
GCACTGGTTTTCTTCTTATGGTGCCATAATTTCGTTTGGTTCTTTAATATACTTCTTGTTTATTGTTTGGGA
AGCAGTTGTCTGCTCTCGTGGGCTCGTTTTTTCTAGAAGAATAGCTAGGGATCTTGAGTGACAAAATCA
GGTATACCCTCCTTATTCTCATACTATGCCGTTGTGAGGTAGAAAAGGTTTTCAAGATTCTTATTATCAAG
TAGGTATAGACATTATAAAGTATCATGGTCTTGTAATAATGGTGATTGTTTTTATTTTAGGTTTAGTAGGTT
ACATAGGTGTGTTTTTGCTTTGTCGTTCTTTAAGATATCGTCATCATTCTCAGTGAGAAAAATTAGAGTAT
GGTTGAACAGTTTTGCCTATTATCCTATTAGCTTTACTGTGAGGCCCTTCTATAAAAAATTTATATCATATA
GATGATATTAAAACTCCTCAGTGAACTTTTAAAGCAGTTGCCTCTCAGTGGTATTGAAACTATGAACATC
AGCAGCAGTTCGAGTTTCAGTCTTATATAGCCGCAGATAGGGGAGCTGGCTATCGGTTGTTAGATGTAGA
TTGGCGGATAGTTGTACCTGCTCAAACTCAAGTCACAATGTATGTGGCTAGAACAGATGTTTTACACTCT
TTCTCGTTACCTTCTGTTTTGTTAAAAGTGGATGCTATTCCAGGACGTGTAAATCAACTTCCTTGTGTGTT
TGGCTTGCCAGGTGTTTATTATGGGCAGTGTTCTGAGATTTGTGGTATCAACCACTCTTTTATACCTATTG
TAATAGAAGTAATCCCTGCTAAAGTATTCATTGGTTGGTTAGAAGGTCTGACTAAC-----------------------
ATGGCTCGTAATCCTTATTATGTTCCTGGTTTAAGTCCTTGGCCTTTTTTAGTTGCTCTAAATATTGGAAGA
CTTTGTTTAAGTCTTGTAATGTGAATACATCGAAGGGTTTTTATGGTTTTATTACCTGTTTTAGGTGTTCTT
TTGTGTTTATTGTGTTGGTGGCGTAATCTATTAGATGAAGTAGATTTAGGTTTTCACAATCGTTATGTGGTA
AAAACTTATCGCGATGGAGTAGCAATTTTTATTTTTAGTGAAGCTATAATGTTTTTCAGCCTTTTTTGAGC
TTTTCTCTATTCAAGGGAAAGGCCTTCAGCGAGATTGGATTTATTATGGCCTCCTTTAGGTGTTCGCTGTC
CTAAGCCTTTTAGGACGGCCCTATTCAGAACTGGCCTTTTAATCAGTAGAAGTTTTTATTGTGTTTGGGC
GCATAAAGCTATGTATAGTGATTATAGATGAGCCCCTATTATTGGTGTGGCTTATTCTGTTCTTTGTGGGGC
TGTCTTTTTGCGGTATCAATTTCATGAGTACTACATAAACTCTTTTTCAATAGCCGATAGGGTTTTTGGGA
GGTGTTTTTATATCTTAACAGGGTTTCATGGTTTCCATGTGGTTATTGGCAGTTTGTGATTATTGGTTAGTA
TATTTCGTTTAATTTTGGGGCATTTTAGTCGCAAGCGTCATTTTGGTCTTGTGGCCTGTTTTTGATATTGAC
ACTTTGTTGATGTTGTCTGGGTTTTTGTTTGACTTTTATTTTACTTAGTAATAGGTGGGATTTTTCGAAAA
CGTAATGGATTAGCTAATGTTCTGACAGGAAGGGTTTATGACTTGCCTTGTCCTATTAATTTAAGATGGTG
ATGAAGGTTTGGTTCTATATTAGGGCTTTGTTTGACAATTCAATTGATTAGAGGCATCATTCTCTCTTTCC
ACTATAGAGCACATGAAGACCTTGCGTTTGACTCAGTTATTCATATCGTTCGTAATGTAAAAAAAGGGTG

GTTTTTACGGAGAGTACACACTAATGGTGCGTCAATATTTTTTATTTGTCTATATGTACATATTGCTCGAGG
TATTTATTACGGTTCTTATTTGGATTCTGCTGTGTGAAATGTTGGTGTAATTCTTTATTTATTGGTTATAGGG
GAAGCCTTTCTTGGTTATAGATTGCCTTGGGGCCAGATGTCTTATTGGGGTGTAACTGTTATTACTAATAT
GGTTACGGTTATTCCTTTTGTGGGGAAAGATATTTTGCAACATGTTTTAGGGGGCTACTCTGTGTGTAAC
AATACATTGAAGCGATTTTACTCTTTACATTTTGTTATCCCTTTTGTAATTATGGGACTAGCAAGGCTTCAT
TTACTTCTCTTACATGAAAATGGAAGAAATAATCCTTTGGGTGTTGAGAGAGATAGAATATTAGTTCCTTT
TCACCCTTTTTATACGGTAAAAGATCTGCTGGGGTTTTGTTGGTTTATGTGAGTTTTTGTTTACTTAGTTT
GTGTTAATCCTGAGTTGTTAGGTAACCCTGTTAATTTTATTCCTGCAGACCCTATAAAAACTCCTATTCAT
ATAAAGCCAGAGTGGTATTTTATATTTGCTTATGCTATTTTACGTTCTATCCCTCATAAAGCTGCAGGTGTT
ATTGCTATGTTAGCTTCTATCTTGGTTTTATTTCTACTCCCCTTGTTACATACAGGTAATTTTCAGGGGCTC
GCTTTTTATCCTATTAACCAAGGGTTGTTTTGGGTTTTTGTTAGGAGGTTTTTAAGGTTAACGTGAATTGG
AAATAGCCCTGTTTGTGAGCCTTATGTAACCTTGGGGCGGGTGTACAGAGTTATTTACTTTAGATCTATCT
TGTTAATCCCTTATAGTCTTAAGCTATGGGATAAGCTTGTTTTTGCTGAGTTTGTAGTACTGATTTTACCTT
TTGTATGTGTTTTGGTTTCGGTAGCTTTTTATACCTTATTGGAGCGTAAAGTTTTAGGTTATATTATAAGTC
GGAAAGGGCCAAATAAGGTGGGGTTTATAGGGGTCATTCAACCTTTTAGCGATGCCGCAAAATTATTTAC
TAAAGAAATAGTTATGCCTGGGTTTTCAAACAACATGGTTTTTGTGCTATGCCCTGGCTTGATCTTACTTA
ATGGGTTGAGATTATGGCTTCTTTATCCTTTTAGTTATACAGAGGTGGTATTTGTTTGTGGGTTAATTCAGT
TTCTAGTGGTCTCAAGAACTAGTGTTTATGGGGTGATGCTAGCAGGCTGGTCTTCTAACTCTAAATATGC
TTTATTAGGTTCCGTACGTGCTGTTGCGCAAAGAGTTTCTTATGAAGTGCCTTTAACATTTGTGATAATTA
TTATTTGTTGCTTGTTAGGAAGAATGCTCTGTCAAGAAGTAAAAGAAGTTTTAGTTCTATTTTATTTGGT
TTAGGCGGAATAATTTGGTTAGTGTGTATATTGGCTGAATCTAATCGTGCTCCTTTTGATTTTGTTGAAGG
TGAATCTGAGTTAGTTTCTGGTTTTAATGTGGAGTATAGAAGAGGAGGCTTTGCGATAGTTTTTATAGCA
GAGTATGCAGCAATACTGTTTAATAGTCTTTTTTTTACCGTTTTATTTGTTGGGGGCAGAGGGGTAGTGGT
TATATTAGGGATAACGTTAGTTGTAGTGGGTTATGTATGAGTTCGCGGGTCTTTTCCTCGTATGCGGTATG
ATAAAATAATAAAACTGTGTTGACGTTACTTAACGGTTTTAGTCCTCTGTTTAGGTGTTTTTTGTCTGTGT
GTCCCTTACTTATAATATTGGTAGTTATGTTTAGTCCTTTAATGTTGTTTTGTTCATGCCTGGTGGTAGGAG
GTAGTTTGCTTAGTATCAGAAGAAGAACATGAAGGGGTGTCTGAGTGGGGATAGAGTTAAATCTTTTCT
CATTTTTAATTTTGATGAATGGAGGAAGCTTTTTCGATTTAGAACCTTTGATTAAGTATTTTGTAGTCCAA
AGGTTAGGTTCAGTTGTATTTATTTTTAGTGTAGTTTATGTCTCTTTTTTAGAAACGTGAATAATAATCCTT
CTAGTATTAGGTCTATTTTTAAAGATTGGGATTTTTCCTTTTCATAGATGGGTTCCCAGGGTTGTTGTAAA
AAGCCGTTGAATTGTAGGTGGGTTGGTGTTAACGTGACAAAAACTGGCGCCTTTGGTTtttTTTTTGATGT
TAATTAGAAGTAGTGTAGTTTTTATTAGGGTTTTGTTTATAGTAGTTATTGGCGGCGCTGGCGGTCTAAAC
CAAAGCAGGGTGCGGGGTATAGCTAGCTACTCTTCTTTCGTTCACATATCTTGAATAATGGTAGGCCTTCT
CTACTCATTTTTTGTGTTTTTGTTTTATTTTTTTATTTACTCTATATCTCTCTTTTTATTTTTTCTAGGCTGTA
GCAATAGTGGTAAAAGCTCTTTAGGGAGTCAAAGGTTTAGTTTACTAGGTTTAATTGGGGTTCTTATAAT
AATAGGGGTTCCTCCTTTTCTGGGCTTTTTAAGGAAGTTATTAGTAATGTTATCTAGGCCTACTTTCGCTT
TGGTTGTTTGTCTATTAGGTTCTGTTGTAAGGCTTAAGTTTTATACTTCTTTCTTTTATAGTATGTTTTTAAA
CGGTTTGTATGGAATTGAATCTGTAGTGTCGCTTAGCTTAGTATTAAATGTTTTAGGATTATTTTTAATTGT
GGTAGTGCTTTTTCTGAGGTTTTTAGGCGTTGAGATGGTTTCTTGTTAATGCTTAGATTTAGGTTACTGC
CGTTGTTTATACTTTCTTCCAAATCTGATTATAGGCGTGAAAAACTCTCTCCTTATGAGTGTGGGTTTGAG
CCTGTATTCAGTGCTCGAACTAGATTTCTGTTCGGTTTTTTTTAGTCGCTGTCTTATTTGTGGTGTTTGAT
GTTGAACTGTCTATCTTAGTAGCAACAATTTTTTCTATCAGGCTTATAAAAAAACTTAATCAGACTAGTGAG
TGTTATTGTTTTTCTAGTTGTTCTTTTTTTAGGGTTGTTTCATGAGTTTCGTGAAGGTTCGTTAAATTGAAT
TTACTAAGTGGTATTGTGGTTATTAAGTATATTTCTGGTGTTAATAGTAGTATTGCAACGCGAGTTAGTGGT
TAGGGCACTACTAATAAGGTTTTATTTGGCACTCTATCTCTGTGTAGTACCAGATAAAGTGTGGATACTTC
ATGGTGTTATAGTCTGAGATTTTTTGAGYTTAAGAATATCTCTTTTAGGGGTATTTGTAGTGGCAATAGGG
GTATTAAGAACAAAGGTGAGCGTGCGGAACAAGCTATTTCTATTGGTAAATATGTTTTTATGTATAGTTGT
AGTAATAGCGTTTAGCGTAAGAAGTTTTTTTCTTTTTTTTTTTTTTTTGAGAGCGACTCTGATTCCTTTAAT
TTTAATAATCGTAGGCTGGCGTTTACAAGCTGGGGTGTACATACTAATTTATACTGTAGTAGGTTCTCTATT
TTTTCTTTTAGGGGTTTGTTTTATATATTTTAATAGAAGAGACTGTATAATCTTAAGAAGGTCATGTGTTAA
AAATATGTGGAGGTTTTGATGAGTATTCTTGTTTGGGTTCTTGGTTAAAATACCTTCTTATCCCTTTCATTT
ATGACTTCTAAAAGCACATGTGGAGGCACCTGTAGCTGGTTCTATAGTGTTAGCAGGGGTGATTCTTAAA
TTTGGGGGGTATGGCATAATCCGATTGTTTAGGGTTATTAATGTCTGTTATTACGATAGGTTATGTCTGATT
GTAGTCGTAAGCCTATTAGGGGGGTTTTACAGAAGCGTTGTTTGTGTTCGTCAGACTGATTTAAAACGCC
TGGTGGCTTACTCCTCTGTTAGACATATAAGAATAGTAATTCTATGTTTTAGAAATTCTTTTCTAGGGTTAG
AAGGGGTTATTTGGCTAATGTTAGGGCATGGCTTGTGTTCTTCTGGGTTATTCATGTTAGTGTCAGTAGTT
TACTCTAGGAGGGAGTCTCGAGTTCTTCTTCTTAATAAAGGAGGGTTGGTTAAAGCACCCTGTTTAGCA
ATAGTTAGATTTCTTCTTTGTGTTAGAAATATGGCGGCGCCCCCTAGGTTAAACCTGTTGGCAGAGATTT
GCTTATATATTTGCATCAGTTCTATATATTACTATATGGTGCTGTTTTTTGCTATAGTTAGGTTTTTTAGGGCT
GCCTATAGGCTTTATTTGTATACCAGTTGTTTTCATGGTAGCCTGAGAGGAGGTCTATTTAGAGTTCTAGG
GCATAGAGATACTATCGTTTTAATATGTCACTGAATCCCCTTAAATATGCTGGTTATTATATTATATTAGATA
CATAAGTACTTTTTATTGAGATTGTTATTAGTATAGAGGTTGTAATCTTAGGTCTTGTTGTTTTATGTGTT
GCTGTAAAATTTTTTTATGTAGCTTCATTAAAATTAGTGCTGTGTCTTGCAGTGTGTGATGCAGGGGTATT

TTTGGCGTTAATTGTTTCACTAATACGGCGTGTTGGAAGAGACAGTGTTAGAAGAATATCTTCTTTTAAttt
aaaATATTGAATCATTTTATTTTTTTTGCGCTCATTATTGGGTATACTTTTATTTTAGTTGGAAGAGTCTCTAG
CGTTTATTTAATTGAGATAGTGTTTTGAGAGTTAGGAAGAGTAAGGATAGTGTTTAGGGTCTTAGTGGAT
TATATAAGGCTAGTTTTTATTGGGACTGTAATAGTAATTGGTGGAAGAGTTTTAATTTACACCAGATGGTA
CATATCAGATGAGCTTTATTACAAACGATTTGTTAGTTTAGTGTTACTATTTATTCTTTCAATAGTGCTAAT
AATCCTTATCCCTAACTTAATTGGGATTTTAATCGGATGAGATGGTTTGGGTTTAACATCCTTTTTATTGGT
GTGTTATTATTGTAACAGAAAATCGCTCGCTGCTAGACTACTGACGGCCCTTACTAATCGGATTGGCGAT
GTGCTGATTTTAATGAGAATTGGTTTTATAATTGTGGAGAACTCCTGGTCTTTATATCAATTTACTTTTGGT
GAGACAATTTCGTTAGTGGGCTTACTGACTTTTGCTGCAATAACTAAGAGTGCACAGATGCCTTTTTGTG
CTTGACTACCTGCTGCTATAGCGGCTCCAACGCCTGTTTCTGCATTAGTACATTCTTCTACTTTAGTTACG
GCAGGCGTGTATTTACTTATTCGTTCGTTTAAGGTAATCTCACTGGATTTTATAGAGGTGTTAGAAGTCTT
AAGGCTGGTTACTCTTTGTTTGGCTGGGACTAGTGCGTTAGTGTGTTTGGATCTCAAAAAAGTAGTGGC
ATTATCTACTTTAAGTCAGCTGAGCGTTATAATACTAAGAATTTCCTTGTATGCGCCTGTTCTTGCCTTTTT
TCATTTAATTACTCATGCGTTATTTAAGGCACTCCTTTTTTTATCGGTAGGGTCTGTAATCCACTCTTATGG
TAATATCCAAGATATCCGAATAGTTGGAGGCTGTTGGAGTACATTACCCAAAAGAATAAGGGCTATAGTA
ATCGCTGTTAGTTCTCTATCGGGGCTACCTTTTTTGAGTGGCTTCTTCTCTAAAGATCTGATTGTAGATTG
CTATAGGAGTAGCAGTCTAATAGTGGTTTTAGTAGGGGTTGGAATTGCTATAACAAGAGTCTATAGTCTAC
GAATTTGAAGAAGTTTGTTTAGATTAAATATAGGCGTGAGTCCTGTTATAAGAAGAGATGAAAAGTTAGA
ACTCACTTTTCCTTATCTGTGTTTAAGGTTAGGAGCCCTATTTGTGGGCTATGTATTAAGAGGGCAAATTA
TAGGCGTTTCTCAGTTTAGAGGTTCCAGAAATTTTGAGATTGTGGTACTAGTGGGCGCATTTTTGGGTTT
ATTTATATGGGCAAACAGGGGAAGGTTACAATTTAATCTTAAAAAATTTCCTTTTTTGTTAAGAATATGGT
GCTTAGAAGTTAGACAAGTTTTGGCAGGTGTTAGGCTTTCATTGTCAGAAAAACTGTCATCGAGCTTAG
ACAATGGGTGGTTAGAGACAATTGGCCCACAAAGAAACTTAATGAGTGTATCACAGAATAATGAGGGGT
TCTTTATGAGTTGCTTAGTGGTGAGTTGTGTTTTGGTTTTATTCTTCTTTGTAGCGTTGGAGTTGTTGTTA
GTTTCAGCCGTTTTGTTTTTTTTTATAATTATTTCTTTTAGTTTCCCTTATGCTTTAGCCATATTTCTTATTAT
TCTTTCGTTATCTATTTGTTTTTTAGTAAGATGATTTTACAGAAGGTTAATAGGGTTGCTGATTTTCATAAT
TTATGTAGGGGGTGTCTTAATCATATTTCTTTACAGCCTTAGAGTGCTACCTAATGAAAGATTTTACTCAG
ACTATGGGTTCTTCTATTTTTTGTGTTGGGTGCTTTTTGTTATGTCTATTTTTAATTATGAAATGTCCTTTCA
TTATATAAGGTTAGTCAGTTTTGGGGGTATATTTGTCTTTATGGCGTTAGTTTTATTTTTCCTTATACTGGTA
GTTTGTAACTTATGTGATAAAAAGCGTATCCCATTACGGAAGCTT"/>
<sequence id="seq_Mytilisepta_keenaeNC0441271" spec="Sequence"
taxon="Mytilisepta_keenaeNC044127" totalcount="4"
value="ATGGGGATAGATGTGTTTTCTGTTTTTGATGATAATCAGTTAAATACTTTTTGGAGAAATGTTTTGT
GAGGTTGAAGATTGTTTCTTTTTGTTACTTTATTAAGGAAGTTAATTCGGGTTTTTTTTACTATTGGTAAG
CTTGTAAGTGATGTAgttggtGTTGGAAGATCAGTGtatTTAAGGGGTTTTTCAGTTGTGATTTTTTCTTTATTT
ATTTTGATTTTATGGTCAAATTTTACAAGTGTAATTCCTTATTTTTATCCTGTTAGGTGCCATATTCCTTATA
TTGCTTCTTTTTCTTTGTATGTCTGAATGAGACTTGTGATTTCTAGGTTGGTTAATAGATATATACAGGTGT
TAGGTAGTTTAGTTCCTTCAGGTCCTATAAGATTGTCTCCTTTGTTAGTGTTGATTGAGATGGTTTCCTCT
CTTATTCGTCCATTAGTTTTAGTAATGCGATTAGTTTTTAATTTGGTAACAGGACAAATTCTTTTAGGTTTA
TTAGGAGAAACTTTTTTGGAGGTTCTTTTAGTTGGGTCTTTTGTTAATTTAGGATTAGTGATAATAGTAAT
AGTTTATTTTTTTGAGCTTTTTGTTTGTCTTTTGCAGAGGTATATTTTTTGTCAATTACTTTATTGTTATAGT
GAGGATCATTCATTGTTTcgatgattgATAAGAACTAACCACAAGGAGATTGGGAGATTATACCTTACTTTTGG
TATTTGGAGAGGTTTAGTTGGTGTTGGGTATAGAATATTAATTTTGAGGATGCATCCTGGTAATTTGCTTC
TTAAAGATAGTTTGTATAATGTGATTGTAACTAGGCATGCTTTAGTAATAATTTTTTTTGCTGTTATGCCTT
TGTTGATTGGTGCTTTTGGAAATTGATTGGTGCCTCTATTTTTAAGTGCTATGGATTTAGTATTTCCTCGTA
TCAATAATTTTAGTTTTTGAATTTTACCTAGTGCTTTATACTTGTTATTGCTTTCTGCTTATGTTGAGGATGG
AGTTGGGACTGGTTGAACTATTTACCCTCCGCTGTCTAGTTATACTTATCATAGTTCACCTGCTGTTGATT
TAGCGATTTTATCTCTTCATTTAGCTGGAAGTGGTTCTTTGATGGGGGCTATTAATTTTTTGGGCTCTAATA
AGAGGTTACCTGTAGATAAAATAAAGGGTGAGCGCTCTGTCTTATATATTTGAAGAATTAGTGTTACTGCT
TTTTTACTGCTTCTATCTTTACCTGTATTGGCTGGTGGAATTACTATATTGCTTTTTGATCGTAATTTTAATA
GAACTTTTTTTGATCCAATAGGTGGTGGTGATCCTGTTTTATTTATACATTTATTTTGATTTTTTGGTCATCC
TGAGGTTTATATTTTAATTCTTCCTGGTTTTGGTGTAATATCGCATGTAACTGCACATTATGCTGGGAAGAC
TTCCCCATTTGGTGTTACTGGTATAATGTACGCAATAATTTCTATTGGTTTAATGGGATTTATTGTTTGGGG
GCATCATATGTTTACAGTAGGGTTGAATGTTGACACGCGAATATATTTACTTCTGCTACAATGATTATTGC
TGTTCCTACGGGAATTAAGGTCTTTAGATGACTTGCAACTTTAGCTGGAGGCCGTAAGACTTTTAAAACG
CCGGTCTTGTGAAGGATTGGTTTTATTGTTTTATTTACTATTGGTGGTTTAACTGGTTTAATTCTTTCTTCT
TCTTCGTTAGATATTAGATTACATGACACTTATTATGTTACTGCTCATTTTCATTATGTATTGTCAATAGGGG
CTGTGTTTGCTATTTTTTGTGCATTTACTCATTGGTATCCTTTGTTTTATGGGGTTAATTTGCATAAGCGTT
GAAGAAAAGGGCATTTTTTTTCTATGTTTGTTGCGGTTAATATTACTTTTTTCCCGATACATTTTTTAGGGA
TAAGAGGGATGCCACGCCGTTATTGTGATTATCCAGATTGTTATTCTAAATGGCATTGATTGTGTACATATG
GTGCTGTTATAGCTTACATGTCGTTGATGTATTTTATGTTTTTGTTGTGAGAGAGAATGGTGAGAAAACGT
GGGgtgtctATGTATGGGTCTAAAGTATTTCAAGATTGAATTTATGATATTGGGGATGCGTTGAATCTTTTCTA
TCATAATATAATATTGGTTGCTGTTTTTATTGTTTGTCTTGTAGGGTATTATCTTTTTCGGATTAATTTTTGC

AGAACTAGATATCGGGGTTTTAAACATAATAATGTTTTGGAATGAATTTGAACTCTCGTTCCTATGTTAAT
TTTGGCTGTTTTGTGGGTGCCTTCTGTTAGTAATTTGTATTTGATAAATCATATTGGGGAGCCTAAATGGT
CTTTTAAGGCCATTGGTCATCAGTGGTATTGAACTTATGAGTTGTATGAGGAAGTTATTTTAGAGTCTTAT
ATAAATAATATTGATGATGGTAAATATCGTCTTTTAGATGTTGATCAGCGTATAGTTGCTCCTATTAATATAC
AGTTGCGGGTTCTTGTTAGAAGTACGGATGTTTTACATTCTTTTGCTTTACCGTCTTGTATATTGAAAGTG
GATGCAATTCCTGGTCGAATAACACAGACGCCTCTTATAGTAGATAAAAGAGGTGTTGCTTATGGACAAT
GTTCAGAATTGTGTGGTGTGAATCATAGTTTTATACCTATTGTGGTTGAGTTTATTCCTACTAAGACTTTCT
TAGATTGGTTAAAGGTTACTGAGTTA-----------------------
ATGGTGCGAAGTCCTTATTATCGAGTGAGACCTAGTCCTTGGCCTCCTTTGGTGGCGTTTTGTTTAGTTA
ATATAGCTCTTGGGTTGGTTAATTGGATATATCGAGTTAATATTTATATTATTTTTGGTGGGTTTTTGTTAGC
TAGATGTTTGAGTTTGTGGTGACGAGATTTATTACGTGAAGGGGATCAAGGATATCATAGAAAATATGTA
ATTAAAACTTTTCGTGATGGTATAGTAATATTTATTGTTTCAGAAGTAATATTCTTTTTTTCATTTTTTTGGG
CTTTTTTTACTAGAAGATTAAGTCCTAATGTTGAGATTGGGGGAAATTGGACTCCTTATGGGATTCGTAGT
CCTAACGCTTTTTCAATTCCTTTGTTAAATACGTGAATTTTAGTTACTAGTGGTATTTCTGTAAATTATTCT
TATAATTCGTTGAAGTGTGATTATGATTATGGTCCTGTTGTTGGGATAGTTTTTACTATCTTTTGTGGGGTT
GTTTTTGTAGGTCTGCAGTATAAGGAGTATTTTAGTAATTCTTTTTGTATTTCTGATGGTATTTATGGTAGT
GTTTTTTACATATTAACTGGTTTTCATGGTGCTCATGTAATTTTTGGAACTGTGTTTTTAATTGTGACTTTT
GGTCGGTTGTGATTTGGCCATTTTTTGTCAAATCGTCATTTTGGGTTTGAGGCATGTGTGTGATATTGACA
TTTTGTTGATGTTATTTGGATTGTTGTTTACATTTTTGTTTATGTTTGAAGTGGTGGGCCTTTTCGTAAGCG
ACATTGGTTGTTGAAAACTGTTAGAGCTAGAATTTATGATTTGCCTTGCCCGATTAATTTAAATGTGTGGT
GGAGTTTTGGTTCTATGTTGGGTTTATGTTTAGTAACACAAATCGTTACTGGTTTTATATTGTCTTTTTATT
ATATTCCTCACGGTGATATGGCTTATGATTCTGTTTTTTACATTATACGTAATGTCCATAAAGGTTGAATGG
TGCGTGGTATTCATGCTAATGGAGCGTCTGTGTTTTTTATGTGTATTTACGTTCACATTGCTCGTGGGTTAT
ATTATGGTTCTTATTTGGATAAAGGTGTTTGGAATGTGGGGGTTATTCTTTATCTTATGTTAATAGGTGAAT
CTTTTTTAGGTTATGTTCTTCCTTGGGGTCAAATATCTTATTGGGGGGCTGTTGTAATTACTAGTATATTAA
CAGCAATCCCTTATGTGGGTAACAGGATTGTAGAGTATGTTTGAGGAGGTTATGTTGTTAATACTCGTAC
ATTGACTCGATTTTATTCTTTTCATTTTATTATTCCTTTTTTAATAGTTGTGATAGTAATACTTCATTTTTTTT
ATCTTCATGATAAGGGTAGAAATAATCCATTAGGAATTAGAAGGGATAGTATGTTGATTCCTTTTCATCCT
TTTTATACTGTAAAAGATATTGTTGGGTTTGTTGCTATGTTTATGGTTTTAATGTATTTTGTTTGTGTTAAAC
CTGAAGCATTGGGTAACCCTTTAAATTATATTATTATTGATCAGTATAAGACTCCTATTCATATTCAACCTG
AATGGTATTTTTTATTTGCTTATACTATTTTGCGTTCTATTCCTCATAAAGTTGGTGGAATTTTAGCTATATT
AGGGTCTATTCTTATTTTGTTTTTAATGCCTTTAATCCATACTGGGCAGTTTCGTAGGCTAAGATTTTATCC
AATACATCAGCTTCTTTTTTGATTTAATGTTAGAGTGTTTATTGGTTTAACTCTTATTGGAATACGTATTGT
TATTGAGCCTTATATTTTTGTGGGACGGGTTTTAACTGGTTGTTATTTTGTTGGGATATTATTATTACCTTTG
AGTTTGTTTTTGTGGGATTTCTTAATTACAATTTGGTTGATTTTGAATGGGTTACCAATTATTTGTGTATTA
TTAGCTATTGGGTTTTTTACTCTTTTTGAGCGAAAGTTATTAGGAAGAATAATACTGCGAAAAGGTCCTA
ATAAAGTAGGATTTATAGGTTTGTTACAGCCTTTTAGTGATGCTGGTAAACTTTTTTGTAAAGAGGTAAAT
GTACCTCGGTTTGCTAATGTGATACCTTTTGTTATTGCTCCTGTTTTTATACTTATGATTTCTATGAGATTAT
GGATTATATATCCTTTTAGAAGTGTTGGTGCTTTGTTTATTTTCGGTGTTTTACAGTTTTTGGCTACGGCTG
GGGTAAGTGTATATGGCGTAATAGTAGCTGGATGATCCTCTAATTCTAAATATTCTTTATTAGGATCAGTAC
GTAGCGTTGCTCAGAGAATTTCTTATGAAATTTCGTTTAGATTAATTATTTTTGTGTTAGTTTTAATGTCTA
TAAGATTTTTTATTCAAGAAATTTCATTTGCTTTTATATTTGTTGCGTATTTGATAGGTTTAGTTTTATGAAT
AGTGTGTATTTTAGCAGAAAATCATCGTGCTCCTTTTGATTTTGTGGAAGGTGAGTCTGAATTAGTTTCT
GGATTTAATGTGGAGTATAGAGGTGGATTATTTGCTATAATTTTTATAGCTGAGTATGGAAGAATATTATTT
TCTAGAATCTTAAGAGCATGTTTGTTTTTTGGTGGTAGAGAGGTTTTTATGAGTATTGTTTTTCTGTTCTTT
GATTATTTTTTTGTTTGAGTTCGTGGAAGGTTTCCGCGGATGCGTTATGATAAGTTAATAAAAATTGGGTG
AACAGTATTTATAGTTATTCCTTTTATTTTTTCATTGTTAGTTTTTTATATTCTTTGTTTTTATTTAAGAATTG
AAAGCTTAACCCTATGGGGGTTTTAAGGGCTGTGGTTATAGTTTTTAGAAGATTAATAAGGATTTCTAGG
AGAACGTGATTAGGTGTTTGGGCGGGTTTTGAGTTAAATCTTTTAAGTTTTATAGTTTTAATAAATTTAGA
GAGAACACGCGCTATTAGGCCTTGTATTAAGTATTTTATTATTCAGTCTTTGGGGTCTGGATTAATTTTAAT
AGGGTTTTTATGTGGTGAATTTTATTTCAGTGAAAATGAGTTTGTGTTTTTTGGTGGGATTATATTAAAGG
GAGGGATTGCCCCATTTCATTTTTGGGTTCCTTCGGTTGTAAACTCTGCTTCTTGGTTAGCAGGGGGATT
AATCCTTTCATGGCAGAAGTTAGCTCCTTTTTTTTTAGTGGGGTGATTGTTTAGGGATTGGATAATCGTTA
TTAGTGCAGGTTTACTAGCTTTAATTGGTGGAATTGGGGGGTTGAATCAACACTCTGTGCGTGGGTTAAT
ATCATATTCCTCCTTTGTTCATAGATCGTGAATAATAGTAGCTTTGCTTAGCTCATTTTCTTTGTTTATTTTT
TATTGAATAGTTTACAGAATAAGAGTGGTTTTGATGTTTTGGTCTTGTTCTAAGGCACGGAAGCAGTTTT
TGAAAAGGAAAATGCGTGTGTTTTGTAGTTGTTTAAGACTTTTTATGTTAAGAGGATTGCCCCCTTTTTT
AGGGTTTGTTTCTAAACTTTTAGTAATAATATCAGTTAACAGGATTGTGGTTTTTGTTTGTGCAATTGGAT
CAGTGATTAGGTTAAAGTACTATTTATCTGCTCTTAATTCATTTATTTTTGGTGATATAGGTTGGTCTCAGT
CTATTGTAATTTTGAGAGTTTTTCTAAATGTTTTTGGGTTTCTTTTAATTGTGATTGGTTGTTAAcaaagatttAT
GTTAGTAAGGGTGGTATTTATTATTTGTTTAGGTCTTTTTTTGTTAAGCTTATGGTTTTTGTCTATAAAATGT
GGAGAAGACCGTGAGAAGTCTTCTCCATACGAGTGTGGTTTTGATGGATTGTTAAGCGCTCGTAGGCCT
TTTTCCTTGCGATTTTTTTTGGTAGCTGTAATATTTGTTGTTTTTGATGTGGAAGTTGTCTTGTTTGTGCCT

ATTGTTTATTCTTTTTGATTTTTTAAAAGTGCTATAACTATACTTATAAGAAGATTGTTTTTGTTAATTTTGT
TTTTAGGGTTATTTCATGAATACCGTGAAGGTTCTTTAGAGTGGGTAGATTAAATGGTTTTAGGGTTTTTG
GTTAGTCTTGTTGGGTTGTTAGTGTTAAAAGATGTAAAAGCAAGATTGGTGGGGTTTGCAGTGATTCTGT
TTTTTTCTATAATCTTACTAGGATCAAGATCAATACATTATGAGTTATTAGGTATTTTTAACCTTGATTTTCT
TGCTAGTAGAATAATTTGCTTAACGTTATATGTGGTTAGGCTTATAGTAATAATAAGATTAAAAGTAAAGC
GAGTTTCGTTGATAAACTTTATAAATTTGAGTATTGGCGTAGTCTTAGTTTGTGTTTTTACTGTAAGGAAT
TTTTTTTTTTTTTTTTTCTTCTTTGAGAGGTCTTTAATTCCTATTGTGTTTATGTTGATTTTTTGGCGTCTTC
AGGCAGTAAATTACATAGTGATTTATATAAGTGCTGGGTCTTTTCCTTTCTTATTTGGTTTAAGGAATTTGG
TGAGTTCAGGGAGTGATAGAATGTCTATATATAATAGGGTTTTTAAGAGTTGTTGTTGGTTTTATTGGTTT
TATTTGATGGGGTTTTTTGTTAAGTTACCTATATTTCCTTTTCATTTATGGTTACCTAAAGCTCATGTTGAG
GCTCCGGTAGTTGGTTCTATAATTCTTGCTGGTGTTTTGTTGAAATTGGGTGGTTATGGGATTATTCGTTTT
TTAGGTTGTATAATAGTGCCTTTATTTAGCGGTTCTTGCGTTTTAGTTTCTGTGGCTTTATTTGGGGGATTT
TTAGCTAGGGTTATATGTTTAATGCAGGTCGATTTAAAGTCTTTAGTGGCTTACTCTTCTGTTGGTCATATG
AGATTAATGGTTTTTGCACTTTGTCAGAGGGAGGTGAGTTTTTATGGTTGTATTATTTTAATAGTGGGTCA
TGGTTTATGTTCTTCAGGCTTATTTTGTTTAGTATATATATTTTATTGTGTTTCTGGCTCTCGATCTGTTATTT
TAAATAAGGGGTTTTTATTTAAGATTCCTGCTTTTGTGTTATGTTGTTTTATTTTAGGTGTTAGGAATATAG
GAGCGCCGCCAAGGCTGGGTTTTGTTGGTGAGGTGTTGTTATTTATAAGTTGTAGTATGGTTTCTTATTGG
TTTGTCTTTGTTTTTTGTTTGATGAGTTTTATAAGTGCATGTTATAGCCTTTATCTTTATGGTGTTTCTTGTC
ATGGAAAGGAAGATAGTTTATTATATTTAAGTATTTCTTTTAAGGATTTGTTTGTGTTGTTTTTGCATATTTT
TCCTTTGGTTAGTTTGTTTTTGTTTTTGTAAtagGTACAAAGGAAACACTTAATTAGGGTGTTTTTAGGGAT
AGAATTTATGGCTTTAAGAGCTATGTTAATTAGGTCTATTGCGGTTTATAGAAGATCAAGTTACGTTTTATT
TACTATATGTATAGCTGTATGTGAGGCAAGTATCGCTTTGGCGCTAATTGTAAGTATAGTGCGTGTTCATG
GAAGAGATCGTGTAATAGCATTGAGTTTGGATAGatgagtgttgtaATGGTTTTGGGGTTTTTTAGGGTATTAATT
GGTTATTTTTTGATGGTAAGTTCTCTTTTTCATTCTTGTAGAGTTCTTGAAGTAGTATTGTGTGTTGTAAGT
AGGTGTGAAGTTGGTGCTAGATTTTTAGTTGATGAAGTTAGGGTAATTTTTAGTGGGGTAGTTTTAGTAA
TCTTTGGAAGTGTAAGGATTTATAGTAAATGGTATATGAATGATGAAGTTTTTTATCGGCGGTTTATTATTT
TGATTTATTTATTTGTAGGTTCTATGATTATATTAATTTTTAGGTCTAATCTAGTTGGTTTGATAGTTGGGTG
GGATGGTTTAGGTTTAGTTTCTTTTCTCTTAGTGTGTTATTATCAGAATTCTTCTAGAATAGGGGCTGCTAT
GTTGACTGTCTTAGTAAACCGTGTTGGGGATGTTTTTATTTTAGCCAGTATTGGTTTGATAAGTATATGGG
GTGAGTTTATAGTTTATGATCGTTTTTTGTTTGAAAGTTTAGGAGTTAGGTTTTTTATTATTTTGGCTGGTA
TAACTAAGAGGGCGCAAATGCCTTTTTGTTCATGGCTTCCTGCAGCTATAGCAGCTCCTACTCCTGTTTC
ATCTTTAGTTCATTCTTCAACTTTAGTGACTGCTGGTGTTTATTTAATTATTCGTTGTGTAAGGTTGTGTGG
GTTGTTGGGGATGGAATTTTTAAAGTTTATAAGTTTGTTAACGTTGGTTATAGCTGGTATAGCTGGTTTGT
TGGAGAGTGATTTTAAGAAGGTTATTGCTTTATCAACTTTAAGTCAATTAAGTGTAATAATGTTCTCTTTG
AGTTTAGGGCTTTATAGTTTAGCTTTTTTCCATTTAGTAACTCATGCTACTTTTAAGGCTTTGTTATTTTTG
AGGGCGGGAGCTGTGATTCATTCTAACAAAGGGTGTCAAGATTTGCGTTTGTTGGGCGGAATGTGAATA
AATTTACCAGTTAGAAGTGCTGTTATAGTCGTTGCTAGATTTTCATTGTGTGGGGTTCCTTTTATAAGTGG
ATTTATTTCTAAGGATTTAATTATTGAGTTAATGAATGGGATGATTTGATTTTATTTTTTTATGTTATTAGGT
ATTATGTTTACATCTTGGTATTCTGTTCGAATTATGTCTGTTGTTTTTGGGTTAAATAAGTGTGTAGTTAGG
AGTATTAAGATTAGGGAGCCTTTAGATGTAATCTTTTCTTACTTTTGCTTATATTTTGGTGCAGTATTTAGG
GGTTACTTAATGGTGGAAAAGATAGGAATTCTTGTTTATCAAAGATTTGTTGATTCATCTTTTTTCTTTTTT
TTGTTATTTTTACCTTTTGGTggaGGAATTTGGTTTATTTTTTCTACGTTTTTAAAAAAGACTTCTAAAGTTT
GGTTTATTGTTTCAATGTGAAATTATAAGTTAAGTCAACTTCCTAGAGGTAGTTTATTTGTTTGTGGAAAT
TATCTTGTTCGATGTATGGATTTAGGTTGGTTAGAGAAGGTAGGTCCTCAGGAATTACTTGGAAAATTAA
GAAAGTCAAATCAAAAATATTTTTTATTGTTTAGAATAGTTATTGTTATAATTTCTTTAGTGTCTTTGTGTT
ACATGATGATAGGGGTTTTTATAGTAGTAGCTATTGGGATTTGTAGTGTTTTCTCTAGAGAGCCGTTTACT
TTAGGATTTTCTCTAATCTCTGTGTGTTTAATAGTGTGTTTGCTTATGGTTGGGGCTAGAAGGTCTTTGTT
AGCTTTTTTAGTTTTTATGAGGTATGTAAGTGGTGTTATAATTTTGTTTCTTTATGTATTAAGTGTTCATCCT
AATCAGAGAATGTCAAGTGGCAAAAAATTTCTTTGTTTTTTTTCTTTTGTTTTAGGGATTTTGGTTTGGTA
TGGATATGGTAGTGGTCTTGAAGGTTTTTGTTTTGTAGGGATATCTAGTCTTTTTTTGTTTATAGGGTTAGT
CTTATTGTTTGTTTTATTTGTAGTGTGTTATTTATGTAAAAAGAAGCGTTTACCTCTTCGTTCTATT"/>
<sequence id="seq_Mytilisepta_virgataKX0945211" spec="Sequence"
taxon="Mytilisepta_virgataKX094521" totalcount="4"
value="ATGGTAATAGATGTATTTTCTGTGTTTGACAATGATAATTTTAATTCTTTTTCTTTAAATGTTGTTTG
AAGTTGAAGATTGTTTCTTCTTGTTACTTTTTTAAGAAAGTTAATACGTGTATTTTATACGTTAGGAAGAT
TCGTTTTAGACTTTGTTGGTAAAGTAGAAGGTTTAAAATTAACGGGTTTTGCTGTTTCTATTTTTTCATTG
TTTAGTTTGATTTTATGAATTAATTTGAGGAGTGTTGTTCCTTATTTTTTTCCTGTTAGGTGTCATGTTCCT
TATATTGCTTCATTTTCTTTGTATGTTTGAATAGGTTTGGTTATATCTAGGTTAGTAAATAGGTGAGTGCAG
GTTGTAGGAAGTTTGGTTCCGTCTGGTCCTATGAGATTATCGCCGTTATTAGTATTAACTGAGATTGTTTC
TTCTGCTGTTCGCCCATTGGTTTTGGTGATGCGATTAGTTTTTAATTTGGTGACGGGTCAGATTCTTTTTG
GTTTGTTAGGGGAAGTTTTTTCTGAGGCTCTTTTGATAGGCTCTGTTTTTAACTTGGGTTTGTTGACAGT
AGTTATAGTTTATTTTTTTGAAATTTTTGTTTGTGTTTTGCAAGCTTATATTTTTTGTCAGCTTCTTTGTAGT
TATAGTGAGGATCATTCATCATTTCGATGATTGATAAGTACTAATCATAAGGAAATTGGTAGTTTGTATTTA

ATGTTTGGAATATGGAGAGGTTTGGTTGGTGTAGGGTATAGGATGTTAATTCTAAGGATACATCCTGGGA
ATTTACTTTTAAAAGATAGTTTGTATAATGTGATTGTGACTAGACATGCACTAGTGATAATTTTTTTGCTG
TTATGCCTTTATTAATTGGTGCTTTTGGTAATTGATTAGTACCTTTGTTTTTAAGTGCCATAGATTTGGTGT
TTCCTCGTATTAACAATTTTAGGTTTTGGATTTTACCTAGTGCTTTGTATTTATTATTGCTTTCTGCTTATGT
TGAAGATGGTGTTGGTACTGGTTGAACTATTTATCCTCCTTTATCTATTTATACTTATCATAGTTCTCCTGCT
GTTGATTTGGCTATTTTATCGTTACATTTAGCTGGAAGCGGGTCTCTGATGGGAGCTATCAATTTTTTAAC
TTCTAATAAAAGATTGCCGGTGGATAAGATAAAGGGTGAGCGTTCTGTTTTATATGTTTGGAGAATTACT
GTGACTGCTTTTTTGTTACTTTTGTCTTTGCCTGTGTTGGCTGGTGGTATTACTATACTGTTGTTTGATCGT
AATTTTAATAGGACTTTTTTTGATCCAATAGGTGGAGGGGATCCTGTTTTATTTATACATTTGTTTTGGTTT
TTTGGTCATCCTGAGGTTTATATTTTAATTCTTCCTGGATTTGGGGTAATGTCACATGTTACTGCTCATTAT
GCTGGAAAATCGTCTCCGTTTGGTGCTGTTGGTATAATGTATGCAATAATTTCTATCGGTTTAATGGGGTT
TATTGTTTGGGGGCATCATATATTTACGGTTGGTTTAAATGTAGATACTCGAATATATTTTACTTCTGCTACT
ATAATTATTGCTGTTCCCACTGGTATTAAAGTTTTTAGATGGCTTGCAACGTTGGCTGGAGGTCGAAAGT
CTTCTAGAACTCCTGTTTTGTGAAGAATAGGTTTTATTGTTTTATTTACTATTGGTGGATTAACTGGTTTAA
TTCTTTCATCTTCTTCGTTGGATATTAGGTTGCATGATACTTATTATGTAACTGCTCATTTTCATTATGTTTT
GTCAATGGGGGCGGTATTTGCTATTTTTTGCGCATTTACTCATTGATATCCTTTGTTCTATGGAAATAATTT
ACATGGTCGTTGAAGTAAGGGACACTTTTTTTCTATGTTTGTTGCAGTTAATTTAACTTTTTTTCCAATAC
ATTTTTTAGGATTAAGAGGGATGCCTCGTCGTTATTGTGATTATCCTGATTGTTATTCTAAGTGGCATTGAT
TGTGTAGGTATGGTGCTATTATAGCTTATATGTCTTTGATGTATTTTATGTTTTTATTATGGGAAAGAATGGT
TAGGAAACGTGGTGTGTCTATGTATGGGTCTAAAGTTTTTCAAGATTGATTTTATGATGTAGGAGATGCAT
TAAATTCTTTTTATCATAATATAATATTGGTTGCGGTTTTTATTATTTCTCTTGTAGGATATTTCCTTTTCCGT
ATTAATTTTTGTAGTGTGAGATATCGCTCTTTTAAGCATCAAAATGTTTTGGAGTGGGTGTGAACTGTTGT
GCCTATATTAATCTTAGCTGTTTTATGGGTGCCTTCGGTTCGTAATTTATATCTTATAAATCATATTGGAGAG
CCTAAGTGGTCTTTTAAAGCTGTTGGTCATCAGTGGTATTGAACTTATGAGGTTTATCAGGAGGTTGTTC
TTGAGTCATATATAGATAATATTGATGATGGAaaaTATCGACTTTTAGATGTTGATCAGCGTATAGTTGCTCC
TGTGAATTTGCAGTTGCGTGTTCTTGTTAGAAGTGTGGATGTTTTGCATTCATTTTCTTTGCCTTCGTGTA
TATTGAAGGTTGATGCTATTCCTGGGCGTATAACACAAACACCTCTTTTAGTTGATAAGAGAGGAGTAGT
TTATGGTCAGTGTTCGGAATTATGTGGTGTAAATCATAGATTTATGCCTATCGTAGTTGAATTTATTCCTGT
TAGTAGATTCTTGGAGTGGttaaaggttactgagtaa-----------------------
GTGGTGCGAAGTCCTTATTATCGTGTTAGACCTAGTCCTTGACCCCCTTTAGTAGCTTTTTGTTTGGTTAA
TATGGCTATTGGTTTAGTTAGTTGAATGTATCGGGTTAATATTAATATTATTTTGGGAGGTTTATTATTGGTT
AGTTGTTTATATTTATGGTGGCGTGATGTGTTGCGCGAAGGGGATCAGGGTTATCATAGGAAATATGTTAT
TAAAACATATCGTGATGGTATAATTATATTTATTGTTTCTGAAGTTATATTTTTTTTTTCTTTTTTTTGAGCAT
TTTTTCATAGGAGTTTAAGACCAAATGTTGAAGTTGGGGGTAATTGACCTCCTTGCGGTATTCGTAGTCC
TAATGCTTTTTCGATCCCTTTATTAAATACTTGGGTTTTAGTCACTAAAGGTATCTCGGTAAATTATGCACA
TAATTCATTGAAATGTGATTATGATTATGGGTCTATTATTGGGATAATTTTTACTATTGTTTGTGGAATTTTT
TTTGTGAAATTACAATATATGGAGTATTTTAGCAATTCTTTTTGTATTTCTGATAGTGTATATGGAAAAGTT
TTTTATATATTAACTGGCTTTCATGGTGCTCATGTAATTTTTGGGACTTTATTTCTAATAGTGACTCTTGGTC
GTTTATGATTTGGTCATTTTTTATTAAATCGTCGTTTTGGGTTTGAAGCGTGTGTTTGATATTGACATTTTG
TTGATGTTATTTGAATTGCTGTCTATATTTTTGTTTATGTGTGAGGTGGGGGTCCTTTTCGTAAACGACATT
GATTTTTAAAGGTTGTTAGAGCTAGGGTTTATGATTTACCTTGTCCTATTAATTTGAGTGTATGGTGAAGT
TTTGGTTCTATATTAGGCTTATGTTTGGTTATGCAAATTATTACTGGGTTTATGTTGTCTTTTTATTACATTC
CTCATGCTGATATAGCTTATGATTCCGTTATTTATATTATACGTAATGTTCATAAGGGTTGGATGGTTCGTAG
GATTCATTCAAATGGTGCTTCTGTATTTTTTATATGTATTTATGTTCATATTGCTCGTGGGTTATATTATGGTT
CTTATTTAGATAAAGGTGTTTGAAATGTCGGGGTAGTTTTGTATCTTATGGTGATGGCTGAGTCTTTTTTA
GGTTATGTTCTTCCCTGGGGTCAAATATCTTATTGGGGGGCTGTAGTTATTACTAGAATGTTAACTGCTATT
CCTTATGTAGGTCATAGGATTGTAGAATATGTTTGAGGAGGTTATGTTGTTAATACTCGTACATTAACTCG
GTTTTATTCTTTTCACTTTATTATTCCATTTTTAATGATTGTGGTAATTATACTTCATCTTCTTTATTTACATG
ATAAGGGAAGGAATAATCCGTTGGGAATTAGGAGTGATTCTATATTAATCCCTTTTCATCCTTTTTATACTG
TTAAAGATATTGTTGGGTTTGTTTCGATGTTTATAGTTCTAATATACTTTGTTTGTGTTAAACCTGAGGCAT
TAGGTAATCCGTTGAATTATATTCCTGCTGATAGATATAAAACTCCTGTTCATATTCAACCTGAGTGGTATT
TTTTATTTGCTTATACTATTTTACGTTCTATTCCTCATAAGGCCGGTGGTATTTTAGCTATGTTGGGATCTAT
TTTTTTATTATTTTTGATACCTTTTATTCATACTGGACAGTTTCGGAGATTAAGGTTTTACCCCATTCATCA
GCTTCTTTTTTGGTTTAATGTTAGTGTATTTGGTGGTCTAACTATAATTGGTATACGTCCTGTGTTAGAGCC
TTATTATACTGTTGGACAGTGTTTAACTGTTTGTTATTTTGTTGGGATATTATTGTTACCTTTGACCTTATTT
TTATGAGATTTTCTAATTGCAATGTGATTAATCTTAGTAAGATTACCTTTTATTTGTGTATTGTTAGCTGTAG
GATTTTTTACATTGTTTGAACGGAAGTTGTTAGCAGGAATAATGTTGCGTAAAGGTCCTAATAAAGTTGG
TTTCATGGGTTTGTTACAACCTTTTAGTGACGCAGGAAAGCTTTTCTGTAAAGAGGTGAACATTCCAAG
GTGTTCTAATGTTTTGCCTTTTGTGGTTGCTCCAATATTTATATTAATGATTTCTATGAGTTTATGAATTTTA
TACCCCTTTAAAAGCGTTAGTGTTTTATTTATTTTTGGCATTTTACAGTTTTTAGTTACTGCTGGAGTAGCT
GTATATGGTGTTATAGTAGCTGGATGGTCTTCTAATTCTAAATATTCTTTATTAGGGTCAGTTCGTAGAATT
GCGCAAAGAATTTCTTACGAAATTTCTTTTGGATTAATTGTGTTCGTATTGGTGTTTATGTCAATGAGATT
TTTTATTCAAGAAATCTCGTTTGTTTTTATATTTGTTTTGTATTTAATAGGGTTAATTTTGTGGATAGTTTGT

ATTTTAGCAGAAAATCATCGTGCGCCCTTTGATTTTGTGGAAGGTGAATCTGAGTTGGTGTCTGGCTTTA
ATGTGGAATATAGAGGTGGATTGTTTGCTTTAATTTTTATATCAGAGTATGGAAGAATATTGTTCTCTAGG
ATTTTAAGGGCCTGTTTGTTTTTTGGGGGGAGAGAGATATTTATTAGAGTTATTTTTTTGTTTTTTGATTAT
TTTTTTGTGTGAGTTCGTGGCAGGTTCCCTCGGATACGTTATGATAAATTAATAAAAATGGGGTGAACTAT
TTTTATAGTTATTCCCCTTATGTTTGTATTATTAGTTTTTTATATTCTTTGTTTTTATTTAAAAATTGTAAATTT
AACCCTATAAGAGTTTTAAGAATTGTAGTTATAGTATTTAGAAGATTGGTGAGAGTCTCTAGGAGTACTT
GATTAGGTGTATGAGTAGGTTTTGAATTAAATCTATTAAGTTTTATGGTATTAATAAACTTAGAAAGTAAAT
GGGCTATTAGGCCTTGTACTAAATATTTTATCATTCAATCATTAGGTTCTGGTTTAATTTTGATAACTTTCTT
ATGTGATGAACTTTACTCTGATGAGAATGAGCTTGTATTTTGTGCTGGTGTTATGTTGAAAGGTGGAATT
GCACCTTTTCATTTTTGAGTCCCTTCAATTGTTAATTCTACTACATGACTAGCAGGTGGTTTAATTCTTTCA
TGACAGAAATTAGCTCCTCTTTTTTTAATGGGATGGTTGTTTAGGGATTGAATAATTATTGTCGGTGCTAG
TCTATCAGCTTTAATTGGTGGTGTTGGTGGGTTAAATCAACATTCTATTCGTGGTTTAATAGTATACTCTTC
TTTTGTACACACTTCATGAATAATGTTAGCTTTGATTAGGTCCTTTTCATTATTTGTTTTTTATTGACTTGTT
TATAGAGTAAGGTTAGTTTTAATGTTTTGATCTTGTTTTAAAGTTAGAAAACAATTTCTTAAAAGTAAAAT
GCGAGTATTCCATAGATGTTTGAGTCTTTTAATGTTGAGAGGTCTACCTCCATTCTTAGGTTTTGTATCAA
AGCTTTTAGTAATAATGTCAATTAACAGAGTTGTAGTGTTTGTTTCTGTAATTGGTTCAGTTATTAGGTTA
AAATATTACTTGTCTGCTCTTTACTCTTTTATTTTTGGTGATACAGATTGTTCTCAGTTTATTGTAATTGTAA
GTGTGTTTTTAAATGTTTTAGGGTTTCTGTTAGTAGTAATTGGGTGTTATCAAAGATTTATGTTGATAAGA
ATAGTGTTTATTATTATTTTATGTTTTTTGTTATTAAGATTATGGTTTTTGTCTATAAAAACTCGTGAAGATT
ATCAGAAGTCATCTCCATATGAATGTGGGTTTGATGGGTTATTGAGAGCACGAAGTCCTTTTTCTTTGCG
GTTTTTTTTGGTGGCTGTTATATTTGTAGTGTTTGATGTAAAGGTTGTTTTAGTTGTGCCTATTATTTATTCT
TTTTGATTTTTTAAAACTGTTATGGCTATAGTTACAAGATGCTTATTTTTACTTGTTTTATTTGTTGGTTTGT
ATCACGAATATCGTGAAGGTtctttagagtgggtagattaaATGGTGTTAGGGTTAATATTTAGTGTAATATTTTTATTG
GTGTTAAGAAATGTAAAAGTAAGATTAGTGGGTTTTATAGTAATATTAGTTTTTGCAATAATAATATTAGG
GTCAATATCTGTAAGCTATGAGCTATTAGGTTTATTTAATCTCGACTTTCTTGCGAGTAGAATAATTAGTTT
AACAGTTTATGTGATTAGTCTGATAATCTTTATGAGGTTAAAAGTAAAGCGTATGAGGTTGATAAATTTTA
TTAATCTTAGAATAAGTTTAATTTTGATTTTAGTTTTTACTGTAAGAAATTTCTTTCTTTTTTTTTTTTTTTTT
TGAAAGTTCGTTAATTCCTGTTATATTTATAGTAATCTTTTGGCGTCTTCAGGCTGTTAATTATATAATAATT
TATATGAGAGCTGGTTCTTTTCCATTACTTTTTGGTTTAAGTAATTTAGTTTATTGTGGTAGTGATAGAATG
TCCGTTTATAATAGTTTATTCAAAGGTTGTTGTTGATTTTATTGGTTTTATTTGATAGGGTTTTTTGTTAAAT
TGCCTATGTTCCCTTTTCATTTATGGTTGCCTAAGGCTCATGTGGAAGCTCCAGTGGTTGGATCAATAATT
CTTGCTGGTGTTTTATTGAAGTTAGGCGGATATGGTATTATTCGTTTTTTAGGATTAACTATAATTCCTATAT
TAAGGAGTTCTTGTATTTTAATTTCTGTTGCTTTATTTGGGGGTTTTATAGCAGGAATTATATGTTTGATGC
AGGTTGATTTAAAGTCTTTAGTGGCATATTCTTCTGTTGGTCATATAAGTTTGTTAGTGCTTACTCTTAGTC
AAAGTGATGTGAGGTTTTATGGTTCTATTATTTTAATAGTTGGTCATGGATTGTGTTCTTCGGGTTTGTTTT
GTTTAGTATATATATTTTATTGTATTTCTGGTTCTCGTTCAGTGGTTTTAAATAAGGGGTTTTTATTAAAAGT
TCCTGCATTTGTATTGTGTTGTTTTGTATTAAGAATTGGAAATATGAGTGCGCCGCCTAGATTAAATTTTGT
TGGGGAAATTATATTTTTTATAAGTTGTAGAATGGTCTCTTATTGATTTGTTTTAATTTTTTCTTTGATTAGA
TTTGTGGGAGCATGTTACAGTTTATATTTTTATGGTGTTTGTTGTCATGGGAAAGAAAATAGTCTTTTATAT
TTGGGGGTTTCTTTTAAGGATTTATTTGTATTGTTTTTGCATATCTTTCCTTTAAATagtttgtttttgtttttgtaatagGTC
CAAAGACGGCATCTAATTAGTGTGTTTTTAGGAATAAAGTTTATAGCTTTAAGTTTTATGTTGGTTGGATC
TATTTCAGTTTATAGAAACTCAAGGTATGTTTTATTAATTATATGTATAGCTGTGTGTGAGGCAAGGGTTGC
TTTAGCTTTAATTGTTAATATAGTACGTGTTTACAGAAGAGATCGTGTAATAATAttgagtttggatagATGAGTGT
TGTAGGAGTTTTAGGGTTTTTAAGTGTTTTATTAGGGTATGTGATAATAATAAGTTCATTTTTTCATTCTTG
TGTGGTTTTGGAGGTAGTATTATGTAGGATTAATAGGTGTGAAATTGGGGCGAGATTTTTGTTAGATGAA
GTTAGGGTTATTTTTGGAGGGGTGGTGTTAGTTATTTCTGGTAGTGTGGGAATTTATAGAAAATGGTATAT
GAATGATGAAGTTTTTTATTGACGTTTTATGATTCTCATTTATTTATTTGTTGGTTCTATGCTTATACTAATT
TTTAGGTCTAATTTGATTGGATTAATATTGGGATGAGATGGATTAGGTTTAGTTTCTTTTCTTTTGGTATGT
TATTATCAGAATCCATCTAGATTAGGGGCTTCAATATTGACTGTTTTAGTTAATCGTGTAGGGGATGTGTTT
ATTTTAGCAAGAATTGGTTTAATGAGAAGATGAGGAGATTTTATAGTGTATGAACGGTCTATGTTTGAGA
ATTTAGGAGTGAGATTATTTGTTGTTGTAGCTGGTATGACTAAAAGTGCGCAAATACCTTTTTGTTCTTGA
CTTCCCGCAGCTATAGCTGCGCCAACTCCTGTTTCATCTTTAGTTCATTCGTCAACTTTAGTGACTGCAG
GGGTTTATTTAATTATTCGTTGTGTAAGTTTATGTGGATTATGTGGAATGGGGGTTTTAAAATTTATAAGTT
TGTTGACTTTAATTATAGCTGGAATAGCTGGTACACTGGAGAGTGATTTTAAAAAAGTTATTGCTTTATCA
ACTTTAAGTCAATTAGGGGTTATAATGTTTTCTTTAAGCTTGGGGTTTTATACTCTAGCATTTTTTCATTTA
GTAACCCACGCCTCATTTAAAGCGTTGCTGTTCTTGAGAGCAGGTGTGGTTATTCACTCTAATAAAGGGT
GTCAGGATTTGCGTTTGTTAGGTGAAAGGTGGAAAAATTTACCAATTAGAAGTGCAGCTATAGTTGTTGC
TAGATTTTCATTATGTGGAATTCCTTTTATGAGGGGGTTTTATTCTAAGGATTTGGTTATCGAGTTGATAAA
AAGTATGATTTGATTTTATTTTATTATATTATTGGGTGTAAGATTTACATCATGGTATTCTGTTCGTGTTGTG
TCTGTTGTTTTTGGTTTAAATAAATGTGTGATAAGAAGTGTTAGGATAAAGGAGTCGTTTGATGTGATTG
CTTCTTATTGTTGTTTATATTTGGCGCAGTATTTAGAGGTTATTTAATAGTAATAAAAATAGAAATTTTTTT
TTATCAAAGGTTTATTGATTCTTTTTTTTTTTTTCTGTTACTTTTTATACCATTTGGGGGATTTATTTATTCTA
TGTTTGTGAAGGAAGTTGGGaagactTCTAATATTCATTTTGTTGTTTTGATGTGGAATTTTAAGTTAAGTCA

ATTTCCGGTTAGGTATTTGTTTAATTATGGTAATAACCTTGTTCGTTGTATAGATTTAGGTTGGTTGGAGAA
GGTTGGTCCTCAAGAGTTTCTTGGGAGAATTAGAAAAATAAATCAAAAATATTTTTATTGTTGTTTGCT
GTTTTTGTTATTTTGATTTTGCTGtctttgtgaaatATGATAATAGGGGTTTATATTGTAATGGCTGTAATGATTTGT
AGTGTTTTCTCTAGCGAACCTTTTACTTTGGGTTTTTCATTAATTTCTTGTTGTTTGATAGTGTGTTTGTTA
ATGGTTAAAGTTAGAAGGTCTTTATTAGCTTTTTTGGTTTTTATAAGTTATGTTAGAGGTATTATGGTTTTA
TTTTTGTATGTTTTAAGTGTTCATCCTAATCAAAGAATATCAGGTGGTAAAATGTTTCTTTTAATTTCTTTG
GTTTTGAGATTTTTATGGTGATATGGTCATGTTGATAATGGTCTTGAGAATTTTTGTTTTGTTGGAATAGTT
AGGCTATATTTGTTTATAGGTGTGGTTTTGTTGTTTGATTTGTTTGTTGTGTGTTATCTTTGTAAAAAAAAA
CGTtaacctcttcgttctatt"/>
<sequence id="seq_Mytilus_californianusF-JX4861241" spec="Sequence"
taxon="Mytilus_californianusF-JX486124" totalcount="4"
value="ATGTTAATAGATGTTTTTTCTAGGTTTGATGCTCATAGTTACAACTTGATTTGGTTATCTATGTTGT
GGGCGCTTTCGTCTATGGTACCTTTAACAGTGTTGTTTAGGGATGTGAGAATTCGAAGTTTTGTTTTATCC
TTTACTTATTCAATGATTCGCAATGGAAAAGGGCTTAAACTTTCTGGGTTTCCGATGGTAATAAGTGGGT
TGTTTCTAATAATTTTAATATTGAATCTATCTGGGAACTTTCCATTTTTCTTCCCTGTAAGGGGCCAGTTTG
TGTTTGGGTTTTCTTTTGCTTTATCTATTTGAACTTGTTTAGTGCTATCTAGGTTGTTGTGTAGGTTTGAGC
AAAGATTAATAAGGTTAGTGCCAACGGGGCCTTTAATTTTGGTACCATTTATAGTAGTGGTTGAATTAATT
AGAGGTATGCTTCGCCCTTTAACTTTGGTTTTACGTTTAACATTAAATCTTGGAGCTGGTAAAGTAATTTT
AACAATATGTAGGAGTGAGTTAGTAGTGGGTTGGTTAATTATAGGGGTAGGAGGGGTCAAAGGCCTCTT
GATAGGTGGAGTCTTTGCCGCTGAAGTTGCAATCGCCTGTATTCAATGCTATATTTCTGTGTACTTTTAT
GTCTTTATACTGATGACCATAGAAGTTAACGTTGACTTTGGTCAACAAATCATAAAGATATTGGAACCCT
TTATCTATATAGAGGGGTGTGAGGAGGTTTATTTGGGGCAAGATTGAGTTTGATAATTATGCAAGGGCATC
CTGGAGCAGTCTTCTTAAAAGATTGATTCTATAATGTGGTTGTTACAACACATGCTTTAATAATAATTTTCT
TTGCTGTAATACCAATTTTAATCGGGGCTTTTGGTAATTGGCTTATTCCTTTGCTTGTAGGGGGAAAAGAC
ATAATCTATCCACGTATAAATAATTTGAGATATTGACTGTCGCCAAATGCACTATATTTATTAATGCTATCTT
TTAGAACAGATAAAGGAGTAGGGGCAGGATGGACTATTTATCCACCTCTATCTGTATACCCTTATCACAG
AGGGCCTAGGATAGATGTTCTTATTGTGTCTTTACACTTAGCAGGACTTAGCTCTCTAGTGGGGGCAATT
AATTTGCTAGCACAAATAAAAATATACCAGTATTAGAAATAAAAGGGGAACGGGCGGAGCTCTATGTGT
TAAGAATTAGGGTTACTGCAGTTCTTTTAATTATCTCAATTCCAGTGTTAGGAGGGGGTATCACAATAATT
CTGTTTGATCGTAACTTTAACACTACTTTCTTTGACCCTGCAGGGGGGGGTGACCCAGTACTATTTCAGC
ATTTGTTCTGGTTCTTTGGTCATCCTGAAGTATATATTCTTATTTTGCCTGCTTTTGGGGTAATGTCAAAGG
TAATTATGCATTGTTCTGGAAAAGAAGCAGTGTTTGGGCTAATTGGGATGGTTTATGCTATGATTGGAATT
GGAGGTTTAGGTTGTATGGTGTGAGCACATCATATATTTACGGTAGGTCTTAATGTTGATACTCGAGGGTA
CTTTTCTACTGCTACTATAGTAATTGCGGTCCCTACCGGGGTAAAGGTGTTTAGCTGACTAGCAACTATAG
CGGGTAGTAAGTTTAAAATAAAGCCGGCTGCCTTTTGAAGGACAGGATTCTTGTTTTTATTTACTGTAGG
GGGATTAACTGGGGTGATGTTGTCAAGTGCATCAATAGATGTGTCGCTTCATGATACCTATTACGTAGTAG
CCCATTTCCATTATGTACTTAGAATAGGAGCTGTGTTTGGGGTTTTTTGTGGTTTAAACCACTGACTACCC
AATTTTGTTGGAGTCTGCTTTAATAAGAAATGAAGGAAAGCCCATTTTATAGCTATATTTTTTGGAGTAAA
TACTACGTTTTTCCCACAACACTTTTTGGGGTTAAGAGGTATGCCACGGCGGTATATAGACTATGCTGATA
TTTATGCTCATTGGCATTGAGTGTCCTCATATGGGTCTGCTGTGTCATTTGGTTCTCTTATATACTTTAAGT
TTTTACTCTGGGAAGCTTTAGTAAGCCAGCGAGGAATGTCTTTTTATGGGAGTCGATATTTTGGTGATATT
GTTCATGAATTAGGAAAAGACCTTTTTCGTTATCATGGCTTTGTAATGATGGTGGCGGTAGCTGTGCTAG
TCTTTGTGATATATATAGGGTGTGTTATTCTCCTTACTAAGTTTTCCTATCGTCATTTTTTGAATCGTCAACG
ATTAGAGTTTTGATGGACAATCGTGCCAATACTGCTACTAGTAGGGTTATGGTTCCCGTCAATAATTAATT
TGTATTATATAGAAGAAGTGAAGCGGCCACGTTGAAATTTTAAAGCAATTGGGAAACAATGATATTGATC
TTACGAATGCGACTCCTGTTATACCATTGATTCCTATATAGAAGATCAACAAGAGACTGGTTATCGATTAT
TGGATGTTGATAATCGGATGGTTGCTCCTGCGGATGTACAAATAACTGCTTTTGTCAGTAGTTCAGATGTT
CTTCATTCGTTTGCTCTTCCTAAACTGTTACTTAAAGTGGACGCCATTCCGGGTCGAATTAATCGACTTCC
AATGAAGGCATCCCCAATGCAGTATTTATGGGCAATGTTCTGAAAATTTGTGGGGGTGAATCATAGGTTT
ATGCCTATCGTAATTGAGTTTATCCCTGAAAAATATTTTGTTATGTGATTAGAGGCTTTAAATTAA------------
----
ATGAAACGTAATCCTTACTATGTGCCTGGTCCAAGGCCATGGCCATTTTTTGTTGCTATTTCTGCAAATGG
TATGGCGGTAGGCTTAATTTTATGACTGCATCGCACACCTTTTTTGCTTATGGGCAGATTAGGTTGTATGC
TATTAAGAACTTTTAGTTGATGACGAGATTTAATTCGGGAGGGAGATATTGGGTTTCATACCCGTTTTGTT
ATTAAAAGATTTCGAGATGGGGTAGCGTTATTTATTTTGTCAGAGGTGATATTCTTCTTTTCCTTTTTTTGA
ACCTTTTTCCACAACGCCTTAAGTCCATCCTGTGAGTTGGGAATACGGTGACCCCCTCCAGGAATCCGG
ACGCCAAACCCGTCTTCTACGAGATTGTTTGAGACAGGGTTGCTTATTAGAAGGGGCTTATTTGTGACTC
AAGCCCATAAAAGAATACGGCTAGATTATGATGTGGGTCCGTTTGTTGGATTGGTGGTAACGATTTTGTG
TGGTACTTTGTTTTTCTTAGTGCAGCTACGGGAATACTATTGAAATTCTTATACTATTGCAGACAGGGTAT
ACGGAAGAGTGTTCTATCTGTTAACGGGGTTCCATGGGATGCATGTAGTTGTGGGAACACTCTGGTTAAT
AGTAAGACTGGTGCGTTTGTGGCGGGGAGAATTTTCTAGACAACGGCATTTTGGGTTTGAGGCTTGTAT
TTGGTACTGACATTTTGTAGATGTAGTCTGAGTTCGACTATGGTGCTTAGTGTATGTCTGATTTGGTGGGC
CGTGACGAAAAACTAATAAGCTGGTAAAGATCATAAATGATAGTTTCTACGACTTACCGTGTCCTGTTAA

CTTAAACGCTTGATGAAGGTTTGGCTCTATATTGGGTTTATGCCTTGTAATTCAGCTTTTAAGGGGGCTTT
TACTATCAATTCACTATACGGCTCATGAAGATATAGCTTTTGACTCTGTTGTGCATATTATGCGTAATGTGA
AAAAAGGCTGAATATTGCGAAACATCCATGCTAATGGTTCTTCTATATTTTTTATCTGTATTTACGCTCATA
TTGCTCGTGGGCTTTATTATGGGTCATATTTGGATAAGACAGTTTGGTACTTTGGGGTACATCTTTTTTTAC
TAACGATAGCTGAAGCCTTTCTAGGTTACACGTTACCATGAGGCCAAATATCCTATTGAGGGGCCACTGT
TATCACTAATATACTCAGGGTAATCCCTGTAGTAGGGGAGAGAATACTTCGTTATGTATGAGGTGGTTGGA
CAGTCTGTAATGCAACGTTAAAGCGATTTTATACACTTCATTTTCTTCTTCCGTTTGTAATAGTGGCGGTT
GTATTTCTTCATCTTTTCTTTTTACACGAAAAAGGAAGTAATAATCCTTTGGGGATTGAAAGTGACACTG
TGTGTGTCCCTTTTCACCCTTTTTATACTATTAAGGATTTATTTGGGTACGTTTGCTTTAGGTTTTTTTTTAT
ATATTTAGTATGTGTAGATCCTGAGTTATTGGGTAATCATTTGAACTATTGGCCTGCTAATCCGATAAAAAC
ACCTATTCATGTTCAGCCTGAATGGTACTTTATATTTGCTTATGCAATTCTTCGGTCTATCCCTCACAAGGC
AGGTGGTGTTTATATTATATTTTTATCTATTGTAGTATTATATCTTATTCCTAGTTTGCATAGGGGTAAGTATC
GAAGATTGTGTTTTTACCCATTCAATCAAGTCGTGTTCTGAGTTTTAGTAGGAAGATTTATTAGATTAACG
TGAATTGGGGCTCGCCCAGTTCGTGAGCCGTATATTGTGATAGGCCAGTGTTTCTCAATTATTTACTTCTC
AAGATTACTATTAAACCCCCTTTCATTATGGATGTGGGATAAGCTATTGGACGTGGGGGTAGTGATTGGG
GTTGTACCTTTTGTTGGTGTACTACTTGCTGTAGGATTTTATACGTTACTGGAGCGAAAAATTTTAGCTAT
TATCATAATTCGTAAAGGGCCGTCTAAAGTTAGTTATATAGGGATTTTGCAGCCGTTTAGAGATGCTGGCA
AGTTATTATGTAAGGAATTTATTATGCCTACACGGGCTAACATAGGGCCCTTTATTCTGGCCCCTGCGTTG
ATGTTAACCGTAAGACTTTTAGGGTGACTCTTATACCCTTATAAATCAGCGGAAGTGTTTTATGTTTTTGG
GGTAGTCCTTTTTATAGTGATTACTAGAGTGAGAGTATATGGAGTAATAATGTCTGGGTGGGCTTCGAATT
CTAAGTATTCATTGTTAGGTGCAGTTCGTGCAATAGCACAAAGGATCTCTTATGAGATTCCAATAGGGTTT
ATTTTTTTTTGTGTAGTTCTAGCTTCTGGTGTATTTATGTTTCAGGAAATTAGGGTATCATTTTTTTTCTTCC
CTTTATCTATTATTCTTGTTGTATGAATCCTGTGTATATTAGCAGAAACTAATCGTGCCCCTTTTGATTTTGT
AGAGGGAGAGTCTGAGTTAGTGTCTGGGTATAATGTAGAGTACAGGGGAGGTGGCTTCGCAGTGATGTT
CATTGCGGAGTACTCTAGAATTCTTCTTAGAAGAGTAATAAGGGCTGCAATATTCTTCGGTGGGAATGAG
GCGCTAATTGGGATTTTCATAATGGTTTTTGCAGTCTTTTTTGTAGTTGTACGTGCTTCTCTACCCCGTTTA
CGTTATGATAAGTTAATGAGCCTGTGTTGAACTGTGCTCTTATGTGTTATACTTATGGCTAGAGTATGTGTA
GTAATGTTAATTAGGGTGTAATGGTAAGTTTAGTAATAAGGCCAATAAAGTTAATGAGGATAAGATTGATG
GTTATTGGGACAGTGCTTAGGGTTAGAAGGGAAGAGTTGGTAGGTGTCTGGTTAGGAATGGAGTTAAAT
CTTTATGGTTTCCTTGTAATTATAAATCCGGATGGTCATTACATTCCAGAACCTTGTGTTAAATACTTTGTA
GTGCAAAGAACTGGGTCTATTTTAATGTTAGGAGGCTTCGTGTTGTTGATGCAGTTTGTAGCTGGTGGGC
TAATAATAAGAGTGGTTGGAACGGTGCTAAAGTCTGGAATTTCTCCTTTACACTCTTGGGTTCCTTCAAC
TATTAAAAATAGAAGATGATTGGCAAGAGGATTAATGTTGACTTGACAAAAGGTTGCCCCTCTAATTCTC
TTATCTATAATTATGCCCTTAGGGGGTATGTGAATAGTTATTGGGCTAATAGCAATAATTGGAGCAGTAGG
GGGCTTGAATCAAAATTCAGTGCGAATTATAAGAGCTTATTCATCCTTTGTTCACACGTCTTGGATGCTTT
TAGGGTTGACTTGGTCTACGGTTGTGTTCATAAGATATTTTTTAGTATATAGGATATCAGTAGGGCTATTTT
TCTATGGGTGCTCTCTAATGAACAAGTTAAGAATAATGAGACAATTAAGAGGAGCTGCTAGAGGAATAG
GGCTTATGATACTTATAGGAATACCACCTTTCCTTGGATTCTTGGCTAAAGTGTTGGTGTTTTTAATAAGA
AGTAGGGCTATTATTGTGCTGTGTATTATGGGGTCAGTAATTAGGCTAAAATATTATATTGATTTTTTTATA
GAATAGTCATAAGAAATAAAAGAGAAGTAAAAGCTATATGAATGTTAGTTACTTCTATAAATTTAATGGG
GGGGGCTCTGATTCTGGTGAGATTTTTGTAAATGGTTATGGGAATAAGGGTTGTCTTTGTATGTATCGTAT
CATTTTTATTTACGGGTTTATTGTTACTAAGAGAAAAGCGAGGCCTTGATCGTGAAAAGTGTAGGCCATA
TGAGTGTGGGTTTGAGCCTATTGGAAGGGCTCGTAGTCCTTTTTCAATTCGGTTTTTCTTAGTAGCTGTTT
TATTTGTAGTGTTTGATGTTGAAGTGGTGCTGTTGATACCCTTTGTCTATATATTTTTCTATGGGAAGAGAT
TATTAGGTATTTTATCCTCAAGAGGGTTTCTATTTATTTTGTTTGTTGGGTTATACCACGAATACCGAGAGG
GATCCTTGGAGTGAGTGGGCTAAATGGTTAAAAGGCTAGCAATCAGGCTTATTGCATTAATTATTATAAA
GAATTTAAACATATCAATTATTGGGCTGAGATTTCTTACAATCATAAGTGTGATGATAGTAAGAACGGTAA
CTGGGAGAGTAGAGTTAAATGGTTTATTCACAACAGATTTTGTGATGGGATTGATGGTAGTATTAACTTTA
TTCGTGGCAGCACTTTCCCACTTAAGAAGGGTGAAAACAGCACGTAAATCAAGATTTAACTTGATAGTT
ATCACAATCAGCCTGATCTTGCTAATAAGTTTTAGAGTGAGAAGGTTTTTTCTCTTTTTTTTTTTTTTTTGA
AAGAGTACTGGCGCCCTTGCTGTTATTAATTGTAGGATGGCGGTTACAAGCAGGAGGTTATATGGTGATC
TATACTGTGTTTGGGTCTCTTTTCTTCTTGTGGGGGGTGAGGGCGTTATATATAAGGGGGAGGAGAAGAA
TAAGAGTGAGAAGAATAGTAAAGAAAAGCGCCATGAGATTGTGGTGACTTTATATTCTAGGTTTTTTAAT
CAAATTGCCTATGTACCCTTTTCATTTGTGGCTTCCTAAAGCTCATGTAGAGGCCCCAGTAGCAGGTTCA
ATGTTGTTGGCTGGTGTAGTCTTAAAACTGGGTGGCTATGGTCTTTTACGATTTATAGGGGTTATGCAAAT
AAATCTAAGAAGACTATTTGTGTTGTTATTATTGGTAAATTTGGCTGGAGGATTATATGCCGGGTTGGCTT
GTGTTCGACAGGTAGATCTCAAATGTTTGGTAGCATATTCATCGGTGGCGCATATAAGGCTGGTGTTACTA
GTGTTGAGAAACACTTTAGTGGGAGTAATGGGGGCAATTCTAATTATGGTTGGGCATGGCCTTTGTTCCT
CGGGGTTGTTTAGATATGTTAATGTGGTGTATAAAATAAGGCACTCACGTCTCCTGGTAATAAATAAGGG
AGGGTTATTAATTTGCCCAAGATTGGTATTAATGTGTTTTCTTCTAAGATCTAGAAACATAGCAGCTCCTC
CTAGACTAAATCTATTTGGGGAGATTTAGTATTTGGGGTAGGGGGCTGAATAAGAGGTGTGTTTCTTTG
TATTTTGGGGTTAATAAGGTTCATTAGGGCCTGTTTAGGTTATACTTGTATGGAAGGTGTAGGCATGGAA
AAGGGCTAATACATAGAGAATCTTTAAGTTTAATGTGTGATATTATGGTTCTCGGGGTTCATTGAGTACCT

TTAAATTTTCTATTCATGTTTATGCCTTAGCGTATAAGAAAACATCTTCTTTGTCTATTTGTAGGGCTTGAA
ATAATAAGGTTAGGATTGCTGTTTGTGAGACATGTCTTTTTAATAAATCAGTTTTGGTTAATTCTTCTAATT
TTATGTTTAGCTGTTTGTGAGGCTAGGATTTGTTTGGCGCTCTTAGTAATAGTAATGCGACTGTGCGGTAA
TGACTTAATGTCAAGATTAGTAAGAGATtaAAATTAATAAGAGGGAATTTTCCTCTTTTGGTGTTAATTTTT
GGGTATGCATCTATTTCTATTGGCAGTATGAGGAGGTGTTTCTTATTTGAAGTGCCTGTATGAGATAGAAA
TTGTCTATCATTTAGATTTAGAGTTTAGTTGATAGTGTAAGAATAATTTTTGTTGGTACTGTTTTAGTAAT
CAGAGGCAGAGTAGGAACTTATTGCAAATGGTACATAGCTAGAGAGAGATACTATAACCGTTTCATAGG
GTTGGTTTGGTTATTTGTGCTTTCTATAATTTTCATGATTTTAGTGCCTAATTTAGTAATGTTACTTATTGGA
TGGGATGGCTTGGGGCTTACTTCATTTTTGTTGGTAGCTTATTATCAAAATAATAAAAGTCTTTCTGCAGC
TATGTTAACAGCTCTAACAAATCGAATTGGGGATGTTCTCGTTTTGGTGAGTATTTCTATCCTCCTAAGTG
AAGGAGGGTGGTTGATTTACAATTATTACCCTATGCAAATATGAAGGTTAGGAATTGTTGTGGTTTTTGC
AGGGATGACCAAGAGGGCCCAGATACCTTTTTGTGCTTGGCTTCCGGCTGCTATAGCTGCTCCAACACC
TGTGTCGTCTCTAGTACATTCTTCTACTTTAGTAACAGCAGGGGTTTACTTGGTGCTTCGGTCATTTTATG
TAGTTAGGGCCAATGCAACCCAAAGGTTGATAGTCTTGAGGTTATTCACTCTGGTATTAGCTGGTTCAAG
CGCAGTGTTTGCCTTTGATTTAAAAAAGGTGATTGCACTTTCTACTCTTAGTCAATTAAGATTAATAATGT
TTTCTATCTCTATTCTTCTCCCACTTGTGGCTTTTTTTCATTTGGTAACCCATGCAGTGTTTAAGGCCCTTC
TTTTTTTAGGGGCTGGCGGCGTGATTCATAGCAACCAAAGAATTCAGGATATTCGGGGTTTAAGAAGATT
GTGGCAAAGGCTGCCTGTGAGGATAGGAGCTATAAGTGTGGCTATTGTATCATTAAGGGGGGCGCCGTT
CATAAGAGGGTTTTACTCTAAAGATTTAATTATTGAAATGATGGACAGAAGGACTTATGGATATATTTTAG
AGCTATTAGGGTTAATTTTTACTTCTTTCTATAGAGCTCGAGTATTTAGGGTAATGTTAGGTTCAAATTATT
TTAATTGTAGAACTCTTCGTGTACCCGAGCACCTAAATATGCAAATCCCATTCTTAAGGCTGTATGTAGGG
GCTATTTTCCTAGGGGTAAGATTAGGGAGAAAAATAGAAAAATTTAGATTTGTAGTGCTTCTTGAGAGAT
ATGAAAGCCTTAGGATTTTTCTGATTCCTTTTGGTCTAATGTGATGGGGAGTACTAACTAAGTTGGGGTTT
AACCCAGCTAAATTAAGATTCTTTTTAAGAATGTGATTTGTGGAGCTTACTCACCCTGGAAAGACCTTGT
TTTTCAAAGGTTCACAAATAGTCTACCAAACTTTGGATCAGGGATGGTTAGAACTGCTAGGCCCACAAG
CTGGGTTGGGGCAAATTAGTCAATTAAATGAAAGCTATTTCATGATTGCCTGAGTAATAGCTGCAGGCAT
AAGATTGATTGCTTTTATATAAATGAGAGTCATAATTATATGTGTAAGTTTGATGTTTATGTTTGTGTTAATT
GCTAAACAACCAATTTCCTTAGGGCTGGTGTTATTAATAGGTTCAATAATTTCATGTGTGGAGATTGCACT
AGAGATCAGAAGATTACTTGGATTTTTGTTGTTTCTGACTTACGTCAGAGGCGTAATAGTTCTATTTTTGT
ATGTGTTAAGAATTTACCCTAATGAGCGATTTAACTTAGAGTTTATTGGGATTGTTATAAGGTGTATTCTAA
CAGGTCTGATTATTACAATAAATTACGAGAATGGGTCTTTGTTTCTTAGGTTTATGGCTGAGATAAGACTC
TATATTCTAATAGCTGGTGTATTATTATTTGTTATACTGGTAGTATCTTACTTGTGTATAAAGACTATAGTAC
CTCTCCGTAGAGTA"/>
<sequence id="seq_Mytilus_chilensisKP1003001" spec="Sequence"
taxon="Mytilus_chilensisKP100300" totalcount="4"
value="ATGTTAATAGATGTATTTTCTAGATTTGATGCTCACAGCTACAACTTAATTTGGTTATCTATGTTGT
GGTTACTTTCGTCTATAGTACCAATAACTGTGCTATTTAGAGACGTAAGCACTCGAAGCTTGGTACTGTC
TTTTACTTATTCTATAATCCGGAATGGGAAAGGTTTAAAGTTATCTGGGTTTCCTTTGGTGATAAGCGGCT
TGTTTATAATAATTTTAATGTTGAATTTGTCTGGGAACTTCCCCTTTTTTTTCCCTGTAAGAGGTCAGTTTG
TATTTGGATTTTCTTTTGCTTTATCTATTTGGACCTGTTTAGTACTATCTAGCTTACTGTGCAGTTTTGAGC
AGGGTTTGATGAGGCTTGTCCCGACAGGTCCGTTAATCCTTGTGCCTTTTATAGTAGTAGTTGAGCTAAT
TAGTGGGATACTTCGCCCCTTAACTCTAGTCTTGCGGCTAACGTTGAATTTAGGGGCTGGTAAAGTAATC
TTAACTATATGTAGAAGGGAGTTGGTAGTCGGTTGGTTAATTACAGGAGTAGGGGGGATTAAAGGTTTGC
TAATAGGTGGCGTCTTTGCTGCTGAAGTTGCAATTGCTTGTATTCAGTGTTACATTTTCTGTGTGTTATTG
TGTCTCTATACTGAGGACCATAGGAGTTAGCGATGGTTGTGGTCAACAAATCATAAAGATATTGGTACTC
TTTATCTATATAGTGGGGTCTGAGGAGGCTTGTTTGGGGCGAGGTTAAGGCTAATAATCATACAAGGGCA
TCCGGGAGCAGTATTTTTAAAAGATTGGTTTTATAATGTGGTTGTTACAACACACGCCTTAATAATAATTT
TCTTTGCTGTAATACCGATTCTAATCGGAGCTTTTGGTAATTGGCTGATTCCTTTATTAGTAGGTGGTAAA
GATATAATTTATCCGCGGATAAATAATTTGAGTTATTGGTTATCTCCTAATGCGCTATATTTACTTATGCTAT
CTTTTAGAACGGATAAAGGAGTGGGTGCTGGATGGACTATTTACCCGCCATTGTCTGTATACCCTTATCAT
AGCGGGCCGAGGATAGATGTTCTTATTGTGTCCTTGCATTTAGCTGGGTTAAGTTCTTTGGTGGGTGCTAT
TAATTTTGCTAGTACCAACAAAAACATACCAGTTCTAGAGATAAAGGGAGAACGAGCTGAGCTTTATGT
CCTAAGGATCAGAGTTACTGCCGTATTGTTAATTATTTCTATCCCGGTTTTAGGAGGGGGCATTACAATAA
TTTTGTTTGATCGGAATTTTAACACAACATTTTTTGATCCAGCAGGAGGGGGTGATCCCGTTTTGTTTCA
ACATTTGTTTTGATTTTTTGGTCACCCTGAGGTGTACATTCTTATTCTACCTGCTTTTGGTGTGATATCAAA
AGTAATTATGCATTGTTCCGGAAAGGAGGCAGTTTTCGGGTTAATTGGGATGGTGTATGCAATAATTGGA
ATTGGAGGGCTAGGTTGTATGGTGTGGGCTCACCATATATTTACAGTAGGGCTTAATGTTGATACTCGAG
GTTATTTTTCTACTGCAACTATAGTAATCGCAGTCCCTACAGGGGTAAAAGTATTCAGGTGGTTAGCAAC
TATAGCAGGGAGAAAATTTAAGATAAAGCCTGCTGCTTACTGAAGTACGGGGTTCTTGTTTCTATTCACC
GTAGGAGGCTTAACAGGGGTGTTATTATCAAGGGCTTCTATGGACGTATCGCTTCATGATACTTATTATGT
GGTGGCTCATTTCCACTATGTGCTAAGTATAGGAGCGGTGTTCGGAGTATTTTGTGGCTTGAATCATTGAT
TGCCTAACTTTGTTGGAGTATGCTTTAATAAGAAATGGAGAAAGGCCCACTTTATAGCAATGTTTTTTGG
TGTAAATACCACTTTCTTTCCTCAACACTTTCTAGGGCTGAGGGGTATGCCTCGACGGTACATAGATTATG

CTGACATTTATGCTCATTGGCATTGGGTGTCTTCTTATGGGTCTGCAGTGTCTTTTGGCTCTTTGATATATT
TTAAGTTCCTACTCTGAGAAGCTTTAGTGAGTCAACGAGGGATGTCTTTTTATGGGAGTCGATATTTTGG
TGATATTGTCCATGAATTGGGGAAGGACCTATTTCGGTATCACGGATTTGTGATGATAGTAGCAGTGGCT
GTATTAGTTTTTGTTATGTATATAGGGTGCGTGATCCTGTTTACTAAATTTTCTTACCGTCATTTCTTAAATC
GCCAACGATTGGAGTTTTGATGAACTATTGTGCCGATGTTGATGTTAGTAGGGCTGTGGTTTCCTTCGAT
GATTAATTTGTATTATATGGAAGAAGTAAAGCGTCCACGTTGAAATTTCAAAGCAATTGGAAAGCAATGA
TACTGATCTTATGAGTGCGATACCTGTTATACAATTGACTCATACATAGAAGACCAACAGGAGACAGGGT
ACCGCTTGCTGGATGTTGACAATCGGATGGTTGCCCCTGCAGATGTACAAATAACTGCTTTTGTAAGGAG
TTCTGACGTGCTACATTCGTTTGCGCTTCCTAAATTATTAATCAAAGTAGATGCTATTCCGGGCCGAATCA
ATCGGCTTCCGATAAAAGCATCTCAGTGTAGTATTATTTATGGCCAGTGCTCTGAAATTTGTGGAGTAAA
CCACAGGTTTATACCAATTGTGATTGAATTTATTCCTGAGAAATATTTTGTTATATGGTTGGAAGCTCTTAA
TTAG------------------------
ATGAATCGTAATCCTTACTATGTACCAGGTCCAAGTCCCTGGCCATTTTTTGTGGCTATTTCGGCTAACGG
AATAGCGGTAGGGTTAATTTTGTGACTGCATCGAACCCCCTTTTTATTAATAGGTAGTCTGGTTTGCATAT
TATTGAGAACTTTTAGATGGTGACGCGATTTAATTCGTGAAGGAGACATTGGTTTTCACACTCGTTTTGT
AATCAAAAGATTTCGAGATTGCGTTGCCTTGTTTATTCTGTCTGAAGTGATATTTTTCTTTACTTTTTTCTG
GACTTTTTTCCATAATGCTTTAAGGCCTTCGTGTGAGCTAGGAATACGGTGGCCTCCTCCTGGAATCCGT
ACGCCAAACCCCTCATCTACTAGTCTGTTTGAGACAGGTCTTCTAATTAGAAGAGGGCTGTTTGTAACTC
AAGCCCATAAGAGGATGCGCTTGGATTACGACGTAGGGCCATTCATCGGCTTAGTGGTGACAATCGTATG
CGGGACTGTGTTTTTCTTGGTACAACTGCGGGAATATTATTGAAACTCCTATACTATTGCAGATAGGGTTT
ATGGTAGGGTTTTTTACTTATTAACTGGCTTCCACGGGATACATGTTGTCGTAGGGACTATTTGGCTAATG
GTAAGGTTAGTTCGACTATGACGAGGGGAGTTTTCTAGCCAACGACACTTTGGGTTTGAGGCTTGCATT
TGGTACTGACATTTTGTAGATGTGGTATGAGTGGCATTGTGGTGCTTAGTGTATGTGTGGTTTGGAGGAC
CGTGACGAAGTACAAATAAACTGGTAAAGATTATGAATGATAGATTCTATGATTTGCCTTGCCCTGTAAA
CTTAAACGCCTGGTGAAGGTTTGGCTCTATACTAGGCTTGTGTCTGGTTATCCAACTTTTGAGGGGTCTT
TTATTATCAGCCCACTACACTGCTCATGAAGACATGGCATTTGACTCTGTAGTCCACATTATGCGTAATGT
GGAAAAAGGGTGAATATTGCGCAATATCCATGCAAATGGGTCTTCTATGTTCTTTATCTGCATTTATGCTC
ACATTGCTCGTGGGTTGTACTATGGGTCTTATTTGGATAAGACCGTGTGGTACTTTGGGGTACACCTGTT
CTTGTTGACTATGGCTGAGGCTTTTCTCGGCTATACTCTGCCTTGGGGGCAGATATCGTACTGGGGGGCT
ACTGTAATCACTAATATACTTAGAGTAAGCCCTGTGGTAGGTGAAAGGATACTTCGCTATGTATGAGGGG
GGTGGACTGTGTGTAATGCAACGTTAAAGCGGTTTTACACACTTCACTTCCTTTTACCGTTTGTAATAGT
GGCGGTTGTTTTTTTGCACTTATTTTTTTTACATGAGAAGGGTAGTAATAACCCTTTAGGAATTGAAAGG
GGTACTATATGTGTGCCTTTCCATCCTTTCTATACTATTAAAGACCTCTTTGGTTATGTTTGCTTTAGGTTCT
TTTTTATGTATTTAGTGTGCGTAGACCCTGAGCTTCTAGGGAATCATCTAAACTATTGGCCTGCTAACCCT
ATAAAAACGCCTATTCACGTTCAGCCTGAATGGTACTTTATATTTGCCTATGCAATTCTGCGTTCAATTCC
GCATAAAGCAGGGGGAGTATATGTTATGTTCCTTTCAATTGTAGTGCTGTACTTAATTCCTACGCTTCATA
CAGGTAAGTACGGAAGTTTATGCTTCTACCCATTGAATCAAGTAGTGTTTTGGGTGTTGGTTGGAAGGTT
TATTAGCCTAACATGAATTGGTGCTCGTCCAGTGCGTGAACCTTATATCATTTTGGGGCAGTGCCTTTCAG
TTATTTATTTCTCTAGTTTATTGTTAAACCCCCTTTCTTTATGGGTGTGAGATAAATTACTTGAAGTGGCAG
TAATTGTAAGTATTATTCCTTTCGTAGGTGTCCTTCTTGCCGTAGGGTTTTACACCTTATTGGAGCGTAAG
ATCCTGGCCATCATCATGATCCGAAAAGGCCCGTCCAAGGTAAGTTACATGGGGATCTTGCAGCCTTTTA
GTGATGCAGGTAAGCTATTATGTAAAGAGTTTATCGTGCCCACGCGTGCTAATGTAGGGCCATTTATTCTG
GCTCCTGCACTAATACTAGCTATTAGCTTATTAGGTTGGCTTTTATACCCTTATAAGTCAGCTGAAGTGTTT
TATGTTTTTGGGGTGATTTTGTTTATAGTTATTACGAGAGTTAGCGTTTATGGGGTAATAATGTCTGGATGG
GCATCTAACTCCAAATACTCCTTACTAGGTGCAGTCCGTGCGATGGCGCAAAGAATTTCTTATGAAATCC
CCATAGGGTTTATTTTTTTTTGTGTAGTGTTGTGTTCAGGCGTGTTTATGTTTCAAGAAATTAGAGTGTTA
TTTTTTTTCTTTCCTTTGTGCGTAGTTATACTTGTTTGAATGTTGTGTATACTAGCTGAGACTAATCGGGCA
CCGTTTGACTTTGTGGAAGGAGAGTCGGAGTTAGTGTCGGGTTATAACGTGGAGTACAGAGGAGGGGG
TTTTGCAGTTATATTTATTGCCGAGTACTCTAGAATTCTCCTTAGAAGAGTAATAAGGGCGGCGATATTCT
TCGGAGGAAATGAAGCGTTAGTTGGGTTTTTCATAATGGTTTTCGCAATTTTCTTTGTAGTTGTTCGTGCT
TCTTTACCACGCTTACGTTATGACAAGCTAATAAACTTATGTTGAACTGTCCTTTTATGTGTTATACTTACG
GCTAGTGTGTGTGTAGTAGTCTTAGTGGGGGTGTAATGGTAAGTTTTGTGGTGAGACCTATAAAATTAGT
GAGTTTAGGGGTAATATTAATTGGAACAATCCTCAGGGTCAGAAGAGAGGAGCTAGTAGGGGTGTGGTT
AGGTTTGGAGTTAAATCTTTATGGTTTTCTTGTGATTATAAACCCAGATGGGCATTATAGCCCTGAGCCTT
GCGTAAAATACTTTGTTGTACAAAGAACGGGGTCAATTTTGATACTAGTGGGGTTTGTAAGCTTAATACA
GCATGTAGTGAGGGGGTTAGTGATGAGCACAGCGGGTACGGTGCTAAAGTCTGGCGTTTTTCCTCTACA
CTCGTGGGTGCCCTCCATTATTAAAAACAGGAGATGGTTAGCAAGGGGGTTAATACTAACCTGGCAAAA
AGTGGCCCCCCTTGTTTTTTTATCAATAATCTTACCTTCTAAAAGGCTTTGAGTAGTGATTGTATCGATAG
CTGGGATTGGGGCAGTTGGAGGGCTTAACCAAAACTCAGTGCGGGTAATAAGAGCCTACTCTTCATTTG
TTCATACATCTTGAATGTTGTTAGGGCTTACATGATCAAGGGTAGTTTTCGTAGGGTACTTTGCAGTCTAT
AGGTTATCGGTAGGGCTGTTTTTCTACGGGTGCTCGTTAATAAACAAGATAAGAATAGGTAGCCAGCTAA
GTAGGGCTGCTAGCGGTATGGGGTTGTTAATACTTATAGGGATGCCTCCGTTTCTTGGTTTCTTAGCAAA
AGTATTGGTATTTTTGATGAGGGGAAGGCCCGTAATTGTGGCATGCATCATAGGATCAGTCATTAGATTAA

AATTTATATTGATTTCTTTTACAGAATGGTAATAAAAAATAAGGTAGAAGTTAAAGCCATTTGGAGCCTG
GTGATTTGCATAAACATTATGGGGGGGGCGCTGATTTTAGTAAGATTTATTTAGATGGTTATGGGGTTAAG
GGTTGTATTCGTTTGTATCGTGTGTTGTCTGTTCACAGGGTTAATAATATTAAGAGAAAAACGGGGGCTG
GACCGAGAGAAATGTAGGCCGTATGAATGTGGGTTCGAGCCTATCGGAAGAGCTCGAAGCCCTTTCTCC
ATTCGATTCTTTTTAGTGGCAGTTTTGTTTGTGGTGTTTGATGTGGAGGTAGTCCTATTGATACCTTTTGC
TTACATATTTTTTTATGGCAAAAGAGTGTTGGGTATTTTATCTTCAAGGGGGTTCCTCCTTATTCTGTTTGT
GGGCCTATATCATGAATACCGTGAAGGTTCTTTGGAGTGAGTGGGTTAAATGGTTAAAAGGTTAGCAATT
AGGCTTATTGCATTAATTATTATAAAAAACCTGAATGTGAGTATTATTGGGTTAAGTGTTTTAACAATTTTG
AGTATAGGTGCCGCAAGAGTTGCGACAGGAGGGGTAGAATTAAATGGGCTATACTCGACGGACTTCGTG
ATGGGTTTGATGATCACATTGACATTATTTGTCGCAATTCTTTCTTATTTAAGTAGAGCTAAAGTCTCCCG
GAAAGCAAGATTTAACTTAATGGTTATCAGAATTAGTCTTATTTTAGTGATAAGGTTTAGGGTGAGGAGG
TTTTTTTTTTTTTTCTTTTTTTTCGAAAGTGTGTTAGCGCCTTTGTTACTGTTAATCGTAGGCTGGCGTCTA
CAGGCAGGAGGCTATATAGTGATTTATACTGTATTTGGGTCGCTTTTTTTTCTATGGGGAGTAAGAGAGCT
TTACCTAAGAGGGAGAAGAAGGATGAGAGTGGGAAGATTAGTAAAAAAAAGGGCTATAAGACTGTGAT
GGCTGTACATTTTAGGGTTCCTAATTAAGTTACCAATATACCCGTTCCATTTGTGGCTCCCTAAGGCCCAT
GTAGAGGCCCCCGTGGCAGGTTCAATGCTGCTGGCCGGGGTAGTACTAAAATTAGGAGGATATGGCCTA
CTTCGGTTTATATTAGTAATACAAGTCAGGCTAAGAAGAGTGTTTGTGTTGCTACTAAGGGTAAATTTGG
CAGGGGGCTTCTACGCCGGACTGGCCTGTGTACGGCAAGTAGACCTAAAATGCTTGGTGGCATACTCGT
CCGTAGCTCATATAAGTCTAGTCCTATTAGTGCTAAGCAACACGCTATTAGGGGTGATAGGGGCTATTATT
ATTATGGTTGGCCACGGGTTATGCTCGTCAGGTTTATTTAGGTACGTAAATGCTATTTATAAGATGAGTCA
CTCGCGCTTACTAGTAATAAACAAAGGTGGGTTACTTTTTTGCCCTGTATTAGTGTTGATGTGTTTTCTTC
TAAGATCAAGGAACATGGCAGCCCCACCTAGCCTTAATCTATTCGGGGAAATCCTCGTTTTTGGCGTCGG
NGGGTGAATAAGGGGGGCCTTTTTATTTATCCTGGGGTTGATGAGCTTCATTAGGGCTTGCTTTAGATTAT
ATTTATATGGGAGGTGCTCACACGGAAAAGGGCTTATACATAGGGAGTCGCTGAATTTACTCTGCGATGT
GTTCGTCTTGTTGTCTCATTGGGTGCCGTTAAATTTCTTGTTTATATTCATACCTTAACGTATAAATAAACA
CCTATTGTGTCTGTTTGTAGGGCTTGAGATAATGAGGCTGGGCTTACTTTTTGTAACACATGTTTTCTTAA
TAAATCAGTTTTGACTAATTTTATTAATTCTATGTTTAGCTGTTTGTGAAGCTAGCATTTGTTTGGCCCTGC
TGGTCATAGTGATGCGATTATGCGGCGACGACCTAATATCAAGATTGCTAAGAGATGTATACAATCAAAG
GGTAACCTAGGGTTGCTTATATTAATCTTAGGGTATTTATTCATTTTTACAGGAAGGAGGGGAAGAGCGTA
CTTACTTGAAGTTCCTGTTTGAGAGAGCAATTGTCTGTCTTTTAGTTTTAGATTCTTACTAGATAATGTAA
GGATAATTTTTGTTGGGACAGTTTTGGTGATCAGAGGAAGTGTAGCAACCTATTGTAAGTGGTATATAGC
TGAAGAGCTATACTACAACCGGTTTATGGGGTTGGTGTGGTTATTTGTTTTATCTATAGTGTTTATAATCTT
AGTGCCTAATCTACTAATACTTTTAATTGGATGGGATGGGCTAGGCCTCACTTCTTTTCTATTAGTGGCTT
ACTACCAAAATAACAAAAGACTATCTGCGGCTATGTTGACAGCCTTAACTAATCGAATTGGAGACGTTCT
TGTTCTAATTAGCATTTCTATCTTTCTAAGAGAGGGGGGGTGGTTAATTTATAGGTATCACCCAGTACAAA
TATGGTTGTTAGGTTTTATAGTGGTTTTTGCAGGGATAACTAAAAGAGCACAGATGCCATTTTGCGCATG
ACTGCCAGCTGCCATAGCCGCACCGACACCTGTCTCTTCTTTAGTGCACTCTTCAACACTGGTAACAGC
TGGGGTTTATCTTGTACTTCGTTCGTTCTATATCATCAGTTCTAACGTAACACAAGTTCTAATAATTTTAAG
GTTATTTACCTTGGTACTAGCTGGATCAAGTGCAGTGTTCGCGTTTGATCTAAAAAAAGTAATTGCACTC
TCGACTTTAAGTCAACTAAGGTTGATAATGTTTTCGATTTCAATCCTCCTTCCGTCTGTGGCGTTTTTCCA
TCTGGTGACTCATGCAGTGTTTAAAGCTTTGCTGTTTTTGGGTGCAGGGGGTGTTATTCATAGAAACCAA
AGAATTCAGGATATTCGTGGGTTAAGAAGACTGTGACAAGGGTTGCCGGTAAGAATAGGTGCAATAAGA
GTTGCAATTGTATCTTTAAGAGGGGCTCCTTTTATGAGAGGTTTTTTCTCCAAAGACCTCATGATTGAGAT
GATAGACAGAAGGACTTATGGGTACTTATTAGAGTTACTAGGGTTAATTTTTACTTCTTTTTATAGGGCAC
GAGTATTTAGAGTAATACTCGGGTCCAATTATGTTAATAATAGAACCTTGCGTATTAATGAGCATTTAAATA
TACAAACCCCGTTTCTTAGCCTATATATTGGAGCTATTATTTTAGGGGTGGTGCTGGGTAGAAAAATAGAA
AGATTTGGGTTCGTAGTAGTTCTTGAAAACTATGAAAGACTTAGAGTCTTTTTTATTCCTTTTGGGTTACT
GTGACGAGGAGTCATTAGTAAGCTAGGGTCGAGTCCTGCTAAGTTAAGATTTTTCTTGAGGATGTGGTTT
ATAGAATTAACTCATCCTGGGAAGGTGATCTTTTTTAAAGGGTCAAGAACAGTGGCCCAAAGTTTGGAC
CAAGGTTGACTAGAGCTATTGGGGCCTCAAGCTGGATTAGGTCAATTCAGTTGCTTAAATGAGAACTATT
TTACTGTGGTGTGATTAGTAGCCGGGGGCGTTAGCTTAATTATTTTTATATAAATGAGAGTTATAATTATAT
GTGTAAGTTTGATGTCTGTATTCGTGTTGATTGCTAAGCAACCAATCTCTTTAGGGCTAGTATTATTAAGG
GGGTCTATAATTGCATGTGTGGAAATTGCATTGGAGGTTAGAAGTCTGTTAGGGTTCTTGTTATTTTTAAC
CTATGTTAGGGGCGTGATAGTCTTGTTCTTATATGTCCTTAGTATTTATCCTAATGAGCGCTTTAATTTAGA
GTTTGTTATAATTGTGAGAAGGTGCGCTATGATGGGCCTTGTGGGATTAATGAATTATGAGAGTGGGTCA
TTATTTTTAAGGTTTATGGCTGAAGGAAGGCTATATATCCTAATAGCAGGCGTTTTGCTGTTTGTGATGTTA
GTGGTGTCGTATTTGTGTATAAAAACCATAGTGCCTTTGCGAAGAGTA"/>
<sequence id="seq_Mytilus_chilensisNC0306331" spec="Sequence"
taxon="Mytilus_chilensisNC030633" totalcount="4"
value="ATGTTAATAGATGTATTTTCTAGATTTGATGCTCACAGCTACAACTTAATTTGGTTATCTATGTTGT
GGTTACTTTCGTCTATAGTACCAATAACTGTGCTATTTAGAGACGTAAGCACTCGAAGCTTGGTACTGTC
TTTTACTTATTCTATAATCCGGAATGGGAAAGGTTTAAAGTTATCTGGGTTTCCTTTGGTGATAAGCGGCT
TGTTTATAATAATTTTAATGTTGAATTTGTCTGGGAACTTCCCCTTTTTTTTCCCTGTAAGAGGTCAGTTTG

TATTTGGATTTTCTTTTGCTTTATCTATTTGGACCTGTTTAGTACTATCTAGCTTACTGTGCAGTTTTGAGC
AGGGTTTGATGAGGCTTGTCCCGACAGGTCCGTTAATCCTTGTGCCTTTTATAGTAGTAGTTGAGCTAAT
TAGTGGGATACTTCGCCCCTTAACTCTAGTCTTGCGGCTAACGTTGAATTTAGGGGCTGGTAAAGTAATC
TTAACTATATGTAGAAGGGAGTTGGTAGTCGGTTGGTTAATTACAGGAGTAGGGGGGATTAAAGGTTTGC
TAATAGGTGGCGTCTTTGCTGCTGAAGTTGCAATTGCTTGTATTCAGTGTTACATTTTCTGTGTGTTATTG
TGTCTCTATACTGAGGACCATAGGAGTTAGCGATGGTTGTGGTCAACAAATCATAAAGATATTGGTACTC
TTTATCTATATAGTGGGGTCTGAGGAGGCTTGTTTGGGGCGAGGTTAAGGCTAATAATCATACAAGGGCA
TCCGGGAGCAGTATTTTTAAAAGATTGGTTTTATAATGTGGTTGTTACAACACACGCCTTAATAATAATTT
TCTTTGCTGTAATACCGATTCTAATCGGAGCTTTTGGTAATTGGCTGATTCCTTTATTAGTAGGTGGTAAA
GATATAATTTATCCGCGGATAAATAATTTGAGTTATTGGTTATCTCCTAATGCGCTATATTTACTTATGCTAT
CTTTTAGAACGGATAAAGGAGTGGGTGCTGGATGGACTATTTACCCGCCATTGTCTGTATACCCTTATCAT
AGCGGGCCGAGGATAGATGTTCTTATTGTGTCCTTGCATTTAGCTGGGTTAAGTTCTTTGGTGGGTGCTAT
TAATTTTGCTAGTACCAACAAAAACATACCAGTTCTAGAGATAAAGGGAGAACGAGCTGAGCTTTATGT
CCTAAGGATCAGAGTTACTGCCGTATTGTTAATTATTTCTATCCCGGTTTTAGGAGGGGGCATTACAATAA
TTTTGTTTGATCGGAATTTTAACACAACATTTTTTGATCCAGCAGGAGGGGGTGATCCCGTTTTGTTTCA
ACATTTGTTTTGATTTTTTGGTCACCCTGAGGTGTACATTCTTATTCTACCTGCTTTTGGTGTGATATCAAA
AGTAATTATGCATTGTTCCGGAAAGGAGGCAGTTTTCGGGTTAATTGGGATGGTGTATGCAATAATTGGA
ATTGGAGGGCTAGGTTGTATGGTGTGGGCTCACCATATATTTACAGTAGGGCTTAATGTTGATACTCGAG
GTTATTTTTCTACTGCAACTATAGTAATCGCAGTCCCTACAGGGGTAAAAGTATTCAGGTGGTTAGCAAC
TATAGCAGGGAGAAAATTTAAGATAAAGCCTGCTGCTTACTGAAGTACGGGGTTCTTGTTTCTATTCACC
GTAGGAGGCTTAACAGGGGTGTTATTATCAAGGGCTTCTATGGACGTATCGCTTCATGATACTTATTATGT
GGTGGCTCATTTCCACTATGTGCTAAGTATAGGAGCGGTGTTCGGAGTATTTTGTGGCTTGAATCATTGAT
TGCCTAACTTTGTTGGAGTATGCTTTAATAAGAAATGGAGAAAGGCCCACTTTATAGCAATGTTTTTTGG
TGTAAATACCACTTTCTTTCCTCAACACTTTCTAGGGCTGAGGGGTATGCCTCGACGGTACATAGATTATG
CTGACATTTATGCTCATTGGCATTGGGTGTCTTCTTATGGGTCTGCAGTGTCTTTTGGCTCTTTGATATATT
TTAAGTTCCTACTCTGAGAAGCTTTAGTGAGTCAACGAGGGATGTCTTTTTATGGGAGTCGATATTTTGG
TGATATTGTCCATGAATTGGGGAAGGACCTATTTCGGTATCACGGATTTGTGATGATAGTAGCAGTGGCT
GTATTAGTTTTTGTTATGTATATAGGGTGCGTGATCCTGTTTACTAAATTTTCTTACCGTCATTTCTTAAATC
GCCAACGATTGGAGTTTTGATGAACTATTGTGCCGATGTTGATGTTAGTAGGGCTGTGGTTTCCTTCGAT
GATTAATTTGTATTATATGGAAGAAGTAAAGCGTCCACGTTGAAATTTCAAAGCAATTGGAAAGCAATGA
TACTGATCTTATGAGTGCGATACCTGTTATACAATTGACTCATACATAGAAGACCAACAGGAGACAGGGT
ACCGCTTGCTGGATGTTGACAATCGGATGGTTGCCCCTGCAGATGTACAAATAACTGCTTTTGTAAGGAG
TTCTGACGTGCTACATTCGTTTGCGCTTCCTAAATTATTAATCAAAGTAGATGCTATTCCGGGCCGAATCA
ATCGGCTTCCGATAAAAGCATCTCAGTGTAGTATTATTTATGGCCAGTGCTCTGAAATTTGTGGAGTAAA
CCACAGGTTTATACCAATTGTGATTGAATTTATTCCTGAGAAATATTTTGTTATATGGTTGGAAGCTCTTAA
TTAG------------------------
ATGAATCGTAATCCTTACTATGTACCAGGTCCAAGTCCCTGGCCATTTTTTGTGGCTATTTCGGCTAACGG
AATAGCGGTAGGGTTAATTTTGTGACTGCATCGAACCCCCTTTTTATTAATAGGTAGTCTGGTTTGCATAT
TATTGAGAACTTTTAGATGGTGACGCGATTTAATTCGTGAAGGAGACATTGGTTTTCACACTCGTTTTGT
AATCAAAAGATTTCGAGATTGCGTTGCCTTGTTTATTCTGTCTGAAGTGATATTTTTCTTTACTTTTTTCTG
GACTTTTTTCCATAATGCTTTAAGGCCTTCGTGTGAGCTAGGAATACGGTGGCCTCCTCCTGGAATCCGT
ACGCCAAACCCCTCATCTACTAGTCTGTTTGAGACAGGTCTTCTAATTAGAAGAGGGCTGTTTGTAACTC
AAGCCCATAAGAGGATGCGCTTGGATTACGACGTAGGGCCATTCATCGGCTTAGTGGTGACAATCGTATG
CGGGACTGTGTTTTTCTTGGTACAACTGCGGGAATATTATTGAAACTCCTATACTATTGCAGATAGGGTTT
ATGGTAGGGTTTTTTACTTATTAACTGGCTTCCACGGGATACATGTTGTCGTAGGGACTATTTGGCTAATG
GTAAGGTTAGTTCGACTATGACGAGGGGAGTTTTCTAGCCAACGACACTTTGGGTTTGAGGCTTGCATT
TGGTACTGACATTTTGTAGATGTGGTATGAGTGGCATTGTGGTGCTTAGTGTATGTGTGGTTTGGAGGAC
CGTGACGAAGTACAAATAAACTGGTAAAGATTATGAATGATAGATTCTATGATTTGCCTTGCCCTGTAAA
CTTAAACGCCTGGTGAAGGTTTGGCTCTATACTAGGCTTGTGTCTGGTTATCCAACTTTTGAGGGGTCTT
TTATTATCAGCCCACTACACTGCTCATGAAGACATGGCATTTGACTCTGTAGTCCACATTATGCGTAATGT
GGAAAAAGGGTGAATATTGCGCAATATCCATGCAAATGGGTCTTCTATGTTCTTTATCTGCATTTATGCTC
ACATTGCTCGTGGGTTGTACTATGGGTCTTATTTGGATAAGACCGTGTGGTACTTTGGGGTACACCTGTT
CTTGTTGACTATGGCTGAGGCTTTTCTCGGCTATACTCTGCCTTGGGGGCAGATATCGTACTGGGGGGCT
ACTGTAATCACTAATATACTTAGAGTAAGCCCTGTGGTAGGTGAAAGGATACTTCGCTATGTATGAGGGG
GGTGGACTGTGTGTAATGCAACGTTAAAGCGGTTTTACACACTTCACTTCCTTTTACCGTTTGTAATAGT
GGCGGTTGTTTTTTTGCACTTATTTTTTTTACATGAGAAGGGTAGTAATAACCCTTTAGGAATTGAAAGG
GGTACTATATGTGTGCCTTTCCATCCTTTCTATACTATTAAAGACCTCTTTGGTTATGTTTGCTTTAGGTTCT
TTTTTATGTATTTAGTGTGCGTAGACCCTGAGCTTCTAGGGAATCATCTAAACTATTGGCCTGCTAACCCT
ATAAAAACGCCTATTCACGTTCAGCCTGAATGGTACTTTATATTTGCCTATGCAATTCTGCGTTCAATTCC
GCATAAAGCAGGGGGAGTATATGTTATGTTCCTTTCAATTGTAGTGCTGTACTTAATTCCTACGCTTCATA
CAGGTAAGTACGGAAGTTTATGCTTCTACCCATTGAATCAAGTAGTGTTTTGGGTGTTGGTTGGAAGGTT
TATTAGCCTAACATGAATTGGTGCTCGTCCAGTGCGTGAACCTTATATCATTTTGGGGCAGTGCCTTTCAG
TTATTTATTTCTCTAGTTTATTGTTAAACCCCCTTTCTTTATGGGTGTGAGATAAATTACTTGAAGTGGCAG

TAATTGTAAGTATTATTCCTTTCGTAGGTGTCCTTCTTGCCGTAGGGTTTTACACCTTATTGGAGCGTAAG
ATCCTGGCCATCATCATGATCCGAAAAGGCCCGTCCAAGGTAAGTTACATGGGGATCTTGCAGCCTTTTA
GTGATGCAGGTAAGCTATTATGTAAAGAGTTTATCGTGCCCACGCGTGCTAATGTAGGGCCATTTATTCTG
GCTCCTGCACTAATACTAGCTATTAGCTTATTAGGTTGGCTTTTATACCCTTATAAGTCAGCTGAAGTGTTT
TATGTTTTTGGGGTGATTTTGTTTATAGTTATTACGAGAGTTAGCGTTTATGGGGTAATAATGTCTGGATGG
GCATCTAACTCCAAATACTCCTTACTAGGTGCAGTCCGTGCGATGGCGCAAAGAATTTCTTATGAAATCC
CCATAGGGTTTATTTTTTTTGTGTAGTGTTGTGTTCAGGCGTGTTTATGTTTCAAGAAATTAGAGTGTTA
TTTTTTTTCTTTCCTTTGTGCGTAGTTATACTTGTTTGAATGTTGTGTATACTAGCTGAGACTAATCGGGCA
CCGTTTGACTTTGTGGAAGGAGAGTCGGAGTTAGTGTCGGGTTATAACGTGGAGTACAGAGGAGGGGG
TTTTGCAGTTATATTTATTGCCGAGTACTCTAGAATTCTCCTTAGAAGAGTAATAAGGGCGGCGATATTCT
TCGGAGGAAATGAAGCGTTAGTTGGGTTTTTCATAATGGTTTTCGCAATTTTCTTTGTAGTTGTTCGTGCT
TCTTTACCACGCTTACGTTATGACAAGCTAATAAACTTATGTTGAACTGTCCTTTTATGTGTTATACTTACG
GCTAGTGTGTGTGTAGTAGTCTTAGTGGGGGTGTAATGGTAAGTTTTGTGGTGAGACCTATAAAATTAGT
GAGTTTAGGGGTAATATTAATTGGAACAATCCTCAGGGTCAGAAGAGAGGAGCTAGTAGGGGTGTGGTT
AGGTTTGGAGTTAAATCTTTATGGTTTCTTGTGATTATAAACCCAGATGGGCATTATAGCCCTGAGCCTT
GCGTAAAATACTTTGTTGTACAAAGAACGGGGTCAATTTTGATACTAGTGGGGTTTGTAAGCTTAATACA
GCATGTAGTGAGGGGGTTAGTGATGAGCACAGCGGGTACGGTGCTAAAGTCTGGCGTTTTTCCTCTACA
CTCGTGGGTGCCCTCCATTATTAAAAACAGGAGATGGTTAGCAAGGGGGGTTAATACTAACCTGGCAAAA
AGTGGCCCCCCTTGTTTTTTTATCAATAATCTTACCTTCTAAAAGGCTTTGAGTAGTGATTGTATCGATAG
CTGGGATTGGGGCAGTTGGAGGGCTTAACCAAAACTCAGTGCGGGTAATAAGAGCCTACTCTTCATTTG
TTCATACATCTTGAATGTTGTTAGGGCTTACATGATCAAGGGTAGTTTTCGTAGGGTACTTTGCAGTCTAT
AGGTTATCGGTAGGGCTGTTTTTCTACGGGTGCTCGTTAATAAACAAGATAAGAATAGGTAGCCAGCTAA
GTAGGGCTGCTAGCGGTATGGGGTTGTTAATACTTATAGGGATGCCTCCGTTTCTTGGTTTCTTAGCAAA
AGTATTGGTATTTTTGATGAGGGGAAGGCCCGTAATTGTGGCATGCATCATAGGATCAGTCATTAGATTAA
AATTTTATATTGATTTCTTTTACAGAATGGTAATAAAAAATAAGGTAGAAGTTAAAGCCATTTGGAGCCTG
GTGATTTGCATAAACATTATGGGGGGGGCGCTGATTTTAGTAAGATTTATTTAGATGGTTATGGGGTTAAG
GGTTGTATTCGTTTGTATCGTGTGTTGTCTGTTCACAGGGTTAATAATATTAAGAGAAAAACGGGGGCTG
GACCGAGAGAAATGTAGGCCGTATGAATGTGGGTTCGAGCCTATCGGAAGAGCTCGAAGCCCTTTCTCC
ATTCGATTCTTTTTAGTGGCAGTTTTGTTTGTGGTGTTTGATGTGGAGGTAGTCCTATTGATACCTTTTGC
TTACATATTTTTTTATGGCAAAAGAGTGTTGGGTATTTTATCTTCAAGGGGGTTCCTCCTTATTCTGTTTGT
GGGCCTATATCATGAATACCGTGAAGGTTCTTTGGAGTGAGTGGGTTAAATGGTTAAAAGGTTAGCAATT
AGGCTTATTGCATTAATTATTATAAAAAACCTGAATGTGAGTATTATTGGGTTAAGTGTTTTAACAATTTTG
AGTATAGGTGCCGCAAGAGTTGCGACAGGAGGGGTAGAATTAAATGGGCTATACTCGACGGACTTCGTG
ATGGGTTTGATGATCACATTGACATTATTTGTCGCAATTCTTTCTTATTTAAGTAGAGCTAAAGTCTCCCG
GAAAGCAAGATTTAACTTAATGGTTATCAGAATTAGTCTTATTTTAGTGATAAGGTTTAGGGTGAGGAGG
TTTTTTTTTTTTTTCTTTTTTTTCGAAAGTGTGTTAGCGCCTTTGTTACTGTTAATCGTAGGCTGGCGTCTA
CAGGCAGGAGGCTATATAGTGATTTATACTGTATTTGGGTCGCTTTTTTTTCTATGGGGAGTAAGAGAGCT
TTACCTAAGAGGGAGAAGAAGGATGAGAGTGGGAAGATTAGTAAAAAAAAGGGCTATAAGACTGTGAT
GGCTGTACATTTTAGGGTTCCTAATTAAGTTACCAATATACCCGTTCCATTTGTGGCTCCCTAAGGCCCAT
GTAGAGGCCCCCGTGGCAGGTTCAATGCTGCTGGCCGGGGTAGTACTAAAATTAGGAGGATATGGCCTA
CTTCGGTTTATATTAGTAATACAAGTCAGGCTAAGAAGAGTGTTTGTGTTGCTACTAAGGGTAAATTTGG
CAGGGGGCTTCTACGCCGGACTGGCCTGTGTACGGCAAGTAGACCTAAAATGCTTGGTGGCATACTCGT
CCGTAGCTCATATAAGTCTAGTCCTATTAGTGCTAAGCAACACGCTATTAGGGGTGATAGGGGCTATTATT
ATTATGGTTGGCCACGGGTTATGCTCGTCAGGTTTATTTAGGTACGTAAATGCTATTTATAAGATGAGTCA
CTCGCGCTTACTAGTAATAAACAAAGGTGGGTTACTTTTTTGCCCTGTATTAGTGTTGATGTGTTTTCTTC
TAAGATCAAGGAACATGGCAGCCCCACCTAGCCTTAATCTATTCGGGGAAATCCTCGTTTTTGGCGTCGG
NGGGTGAATAAGGGGGGCCTTTTTATTTATCCTGGGGTTGATGAGCTTCATTAGGGCTTGCTTTAGATTAT
ATTTATATGGGAGGTGCTCACACGGAAAAGGGCTTATACATAGGGAGTCGCTGAATTTACTCTGCGATGT
GTTCGTCTTGTTGTCTCATTGGGTGCCGTTAAATTTCTTGTTTATATTCATACCTTAACGTATAAATAAACA
CCTATTGTGTCTGTTTGTAGGGCTTGAGATAATGAGGCTGGGCTTACTTTTTGTAACACATGTTTTCTTAA
TAAATCAGTTTTGACTAATTTTATTAATTCTATGTTTAGCTGTTTGTGAAGCTAGCATTTGTTTGGCCCTGC
TGGTCATAGTGATGCGATTATGCGGCGACGACCTAATATCAAGATTGCTAAGAGATGTATACAATCAAAG
GGTAACCTAGGGTTGCTTATATTAATCTTAGGGTATTTATTCATTTTTACAGGAAGGAGGGGAAGAGCGTA
CTTACTTGAAGTTCCTGTTTGAGAGAGCAATTGTCTGTCTTTTAGTTTTAGATTCTTACTAGATAATGTAA
GGATAATTTTTGTTGGGACAGTTTTGGTGATCAGAGGAAGTGTAGCAACCTATTGTAAGTGGTATATAGC
TGAAGAGCTATACTACAACCGGTTTATGGGGTTGGTGTGGTTATTTGTTTTATCTATAGTGTTTATAATCTT
AGTGCCTAATCTACTAATACTTTTAATTGGATGGGATGGGCTAGGCCTCACTTCTTTTCTATTAGTGGCTT
ACTACCAAAATAACAAAAGACTATCTGCGGCTATGTTGACAGCCTTAACTAATCGAATTGGAGACGTTCT
TGTTCTAATTAGCATTTCTATCTTTCTAAGAGAGGGGGGGGTGGTTAATTTATAGGTATCACCCAGTACAAA
TATGGTTGTTAGGTTTTATAGTGGTTTTTGCAGGGATAACTAAAAGAGCACAGATGCCATTTTGCGCATG
ACTGCCAGCTGCCATAGCCGCACCGACACCTGTCTCTTCTTTAGTGCACTCTTCAACACTGGTAACAGC
TGGGGTTTATCTTGTACTTCGTTCGTTCTATATCATCAGTTCTAACGTAACACAAGTTCTAATAATTTTAAG
GTTATTTACCTTGGTACTAGCTGGATCAAGTGCAGTGTTCGCGTTTGATCTAAAAAAAGTAATTGCACTC

TCGACTTTAAGTCAACTAAGGTTGATAATGTTTTCGATTTCAATCCTCCTTCCGTCTGTGGCGTTTTTCCA
TCTGGTGACTCATGCAGTGTTTAAAGCTTTGCTGTTTTTGGGTGCAGGGGGTGTTATTCATAGAAACCAA
AGAATTCAGGATATTCGTGGGTTAAGAAGACTGTGACAAGGGTTGCCGGTAAGAATAGGTGCAATAAGA
GTTGCAATTGTATCTTTAAGAGGGGCTCCTTTTATGAGAGGTTTTTTCTCCAAAGACCTCATGATTGAGAT
GATAGACAGAAGGACTTATGGGTACTTATTAGAGTTACTAGGGTTAATTTTTACTTCTTTTTATAGGGCAC
GAGTATTTAGAGTAATACTCGGGTCCAATTATGTTAATAATAGAACCTTGCGTATTAATGAGCATTTAAATA
TACAAACCCCGTTTCTTAGCCTATATATTGGAGCTATTATTTTAGGGGTGGTGCTGGGTAGAAAAATAGAA
AGATTTGGGTTCGTAGTAGTTCTTGAAAACTATGAAAGACTTAGAGTCTTTTTTATTCCTTTTGGGTTACT
GTGACGAGGAGTCATTAGTAAGCTAGGGTCGAGTCCTGCTAAGTTAAGATTTTTCTTGAGGATGTGGTTT
ATAGAATTAACTCATCCTGGGAAGGTGATCTTTTTTAAAGGGTCAAGAACAGTGGCCCAAAGTTTGGAC
CAAGGTTGACTAGAGCTATTGGGGCCTCAAGCTGGATTAGGTCAATTCAGTTGCTTAAATGAGAACTATT
TTACTGTGGTGTGATTAGTAGCCGGGGGCGTTAGCTTAATTATTTTTATATAAATGAGAGTTATAATTATAT
GTGTAAGTTTGATGTCTGTATTCGTGTTGATTGCTAAGCAACCAATCTCTTTAGGGCTAGTATTATTAAGG
GGGTCTATAATTGCATGTGTGGAAATTGCATTGGAGGTTAGAAGTCTGTTAGGGTTCTTGTTATTTTTAAC
CTATGTTAGGGGCGTGATAGTCTTGTTCTTATATGTCCTTAGTATTTATCCTAATGAGCGCTTTAATTTAGA
GTTTGTTATAATTGTGAGAAGGTGCGCTATGATGGGCCTTGTGGGATTAATGAATTATGAGAGTGGGTCA
TTATTTTTAAGGTTTATGGCTGAAGGAAGGCTATATATCCTAATAGCAGGCGTTTTGCTGTTTGTGATGTTA
GTGGTGTCGTATTTGTGTATAAAAACCATAGTGCCTTTGCGAAGAGTA"/>
<sequence id="seq_Mytilus_coruscusKJ5775491" spec="Sequence"
taxon="Mytilus_coruscusKJ577549" totalcount="4"
value="ATGTTAATAGATGTTTTTCTAGTTTTGATGCTCATAGTTACAATTTGATTTGGCTATCAATGTTGTG
GGTTCTTTCTTCTATGGTACCACTAACTGTATTATTTAGGGACGTGAGTATTCGTAGTTTAGTGTTATCGTT
TACTTATTCAATGATTCGTAACGGAAAAGGGCTCAAACTTTCCGGGTTTCCATTAGTGATAAGTGGGCTA
TTTCTTATAATTTTAATATTAAATTTGTCGGGAAACTTTCCGTTTTTTTTCCCTGTAAGGGGTCAGTTTGTG
TTTGGGTTTTCTTTTGCGCTATCTGTGTGAACTTGTTTAGTATTATCTAGTTTATTGTGTAGGTTTGAACAG
AGATTAATAAGGCTGGTCCCAACTGGGCCACTGATTCTGGTGCCTTTTATAGTAGTGGTTGAATTAATCA
GTGGTATGCTACGCCCTTTGACTTTGGTGTTGCGTTTAACACTGAATCTTGGAGCTGGAAAAGTGATTTT
GACGATATGTAGGAGAGAGCTAGTAGTAGGGTGATTAATTATAGGAGTAGGGGGAGTTAAAGGGCTTTT
AATAGGAGGAGTTTTTGCTGCTGAGGTAGCAATTGCCTGTATTCAATGTTATATCTTCTGTGTACTTCTGT
GTCTTTATACTGAGGATCATAGAAGTTAACGATGACTTTGATCAACAAACCATAAGGATATTGGAACTCT
TTATTTATATAGAGGGGTGTGAGGAGGTTTATTTGGGGCAAGACTAAGTCTGATAATTATGCAGGGGCAC
CCTGGAGCAGTTTTTTTAAAAGACTGATTTTATAATGTAGTAGTAACAACGCATGCTCTGATAATAATTTT
CTTTGCTGTGATACCCATTTTAATTGGGGCCTTCGGTAACTGGCTTATTCCTTTGCTTGTTGGGGGAAAA
GACATAATCTATCCACGTATAAACAATCTTAGATATTGACTGTCGCCGAATGCTCTATATTTATTGATGTTAT
CTTTTAGGACAGATAAAGGAGTGGGGGCAGGGTGGACGATTTATCCCCCTCTATCTGTGTACCCCTATCA
TAGAGGGCCCAGAATGGATGTTCTTATTGTGTCTCTTCATCTGGCAGGACTCAGGTCTTTAGTGGGAGCT
ATTAATTTTGCTAGCACAAATAAGAACATACCAGTATTAGAAATAAAAGGGGAACGGGCTGAGCTTTATG
TGCTAAGAATCAGAGTTACTGCGGTTCTTTTAATTATTTCGATTCCGGTGTTAGGAGGGGGGATCACAAT
AATTTTGTTTGATCGTAATTTTAACACTACTTTCTTTGATCCTGCAGGAGGAGGTGACCCGGTGCTATTTC
AGCATCTATTCTGATTTTTTGGGCACCCTGAAGTATACATTCTTATTTTGCCTGCTTTTGGGGTAATATCAA
AGGTTATTATACATTGCTCTGGAAAAGAGGCGGTGTTTGGGTTAATTGGGATAGTTTATGCTATAATTGGG
ATTGGAGGACTAGGTTGTATGGTGTGAGCACATCATATATTTACGGTAGGGCTTAATGTGGATACTCGGG
GGTACTTCTCAACTGCTACTATAGTAATTGCGGTTCCTACAGGAGTAAAAGTGTTTAGTTGACTAGCAAC
TATGGCAGGAAGAAAGTTCAAAATAAAACCAGCTGCCTTCTGAAGAACAGGTTTTCTGTTTTTATTTACT
GTAGGGGGGTTAACAGGGGTAATATTGTCGAGAGCTTCTATAGATGTGTCACTCCATGATACTTACTATGT
GGTTGCGCATTTTCATTATGTGCTTAGTATGGGGGCTGTGTTTGGGGTTTTCTGTGGTTTGAACCACTGAT
TACCTAATTTTGTTGGAGTTTGTTTTAATAAAAAGTGAAGAAAAGCTCATTTTATAGCTATATTCTTTGGG
GTAAATACCACGTTTTTTCCTCAACACTTTTTAGGGCTAAGGGGTATACCACGACGGTATATGGACTACG
CTGATATTTATGCTCACTGACATTGGGTGTCTTCTTATGGGTCTGCTGTTTCATTTGGGTCTCTTATATATTT
TAAGTTTCTACTTTGGGAGGCTTTAGTAAGCCAGCGAGGAATGTCTTTTTATGGGAGTCGATATTTTGGT
GATATTGTCCATGAATTAGGAAAAGACCTTTTTCGTTACCATGGCTTTGTAATGATGGTGGCGGTAGCCG
TGTTAGTTTTTGTTATATACATAGGATGCGTGATTCTTCTTACTAAGTTTTCTTATCGTCATTTTTTAAATCG
TCAACGGTTAGAGTTTTGGTGAACAATCGTACCCATATTTTTACTAGTGGGGCTATGGTTTCCGTCAATAA
TTAACTTGTACTATATAGAAGAAGTCAAGCGACCACGTTGGAATTTTAAGGCAATTGGAAAGCAGTGAT
ATTGGTCCTATGAGTGTGACACTTGTTACACAATCGACTCGTACATAGAGGATCAGCAAGAAACTGGTTA
TCGATTGTTAGATGTGGATAATCGGATGGTTGCTCCTGCGGATGTACAAATAACTGCTTTTGTAAGGAGC
TCAGACGTTCTACATTCGTTCGCTCTTCCTAAGCTTCTACTAAAAGTGGACGCTATTCCGGGTCGAATTA
ATCGCCTGCCGATAAAGGCGTCTCAATGTAGGATTATTTATGGGCAGTGTTCTGAAATCTGCGGGGTAAA
CCATAGGTTTATACCTATCGTAATTGAGTTTATTCCTGAAAAATATTTCGTGATATGATTGGAAGCTCTAAA
TTAA------------------------
ATGAAACGTAATCCTTATTATGTGCCTGGTCCCAGGCCGTGGCCATTCTTTGTGGCTATTTCTGCTAATGG
TATGGCGGTAGGGTTAATCTTATGATTGCATCGGACTCCTTTTCTTCTTATGAGTAGCCTGAGTTGTATGTT
ATTGAGAACCTTTAGCTGATGGCGAGATTTAATTCGCGAAGGAGATATTGGTTTTCACACTCGTTTTGTA

ATTAAGAGGTTTCGAGATGGAGTGGCATTGTTTATCTTGTCGGAGGTAATATTTTTTTTCTCCTTTTTCTG
AACTTTTTTCCATAACGCCTTGAGCCCTTCTTGTGAACTTGGGATACGATGGCCCCCTCCTGGAATTCGG
ACTCCTAACCCATCATCTACAAGTCTGTTTGAGACAGGTTTGTTAATCAGAAGAGGTCTGTTTGTAACTC
AAGCTCATAAAAGAATGCGGCTAGACTATGATGTCGGCCCCTTTATTGGGTTGGTAGTTACAATCTTATGC
GGTACTCTGTTCTTCTTAGTTCAACTACGGGAATACTATTGAAATTCTTATACTATTGCAGATAGGGTGTA
CGGAAGGGTTTTCTATTTGCTAACTGGATTTCATGGGATACATGTGGTTGTGGGGACGCTCTGGCTGATA
GTCAGATTGGTTCGGCTATGGCGTGGGGAATTCTCTAGACAACGACATTTTGGGTTCGAGGCTTGTATCT
GGTATTGACATTTTGTGGATGTAGTTTGAGTTGCGTTGTGATGTTTAGTGTATGTATGATTTGGAGGACCG
TGACGAAAAACTAATAAGCTGGTAAAAATCATAAATGACAGATTCTATGATTTACCGTGTCCTGTGAACT
TAAATGCTTGATGAAGATTTGGCTCCATATTAGGTTTATGTCTTGTAATCCAACTTTTAAGTGGTCTTTTGT
TGTCGATTCATTATACGGCCCACGAAGATATAGCATTTGACTCTGTTGTGCATATTATGCGCAATGTTAAA
AAAGGCTGAATGTTGCGGAATATCCATGCTAATGGGTCTTCTATATTTTTTATCTGTATTTATGCCCACATT
GGTCGTGGTTTGTACTACGGCTCATATTTGGACAAAACAGTTTGGTATTTTGGGGTTCATCTTTTGTTATT
AACTATGGCTGAGGCCTTTTTAGGTTACACACTACCTTGAGGCCAGATATCATATTGAGGTGCCACTGTG
ATTACTAATATACTTAGGGTGATTCCTGTAGTAGGAGAAAGAATACTTCGTTATGTGTGAGGTGGTTGGA
CAGTCTGTAATGCAACGCTAAAGCGGTTTTATACCCTTCATTTTCTTCTTCCATTTGTAATAGTGGCGGTT
GTATTTCTTCACCTTTTTTTCCTGCATGAAAAAGGGAGGAATAACCCCTTTGGGGATTGAAAGTGATACTG
TGTGTGTTCCTTTTCATCCTTTTTACACTGTGAAAGATTTATTTGGCTATATTTGTTTTAGGTTTTTTTTTAT
GTACTTAGTATGTGTAGACCCTGAGTTATTAGGAAATCACTTAAATTATTGGCCTGCTAACCCGATAAAAA
CCCCTATCCATGTTCAGCCAGAGTGATATTTTATATTTGCTTACGCTATTCTCCGTTCAATTCCTCATAAAG
CAGGGGGTGTATATGTTATATTTTTATCTATTGTGGTCCTGTATCTGATTCCCAGGTTGCACAGAGGTAAAT
ATCGAAGATTGTGTTTTTACCCCTTTAACCAAGTCGTGTTTTGAGTTCTAGTGGGGAGCTTTATCAGCTT
GACGTGAATTGGGGCTCGTCCAGTTCGTGAGCCTTATATTGTGATGGGCCAATGTTTTTCGATTATTTATT
TTTCGAGGCTTCTATTGAACCCCCCTTTCATTATGGATGTGGGATAAATTGCTAGATTTGGGAGTGGTAATT
GGGATCGTGCCTTTTGTTGGGGTACTTCTTGCTGTAGGGTTTTATACCTTATTGGAGCGCAAAATTTTAGC
TATTATTATAATCCGTAAAGGACCATCTAAGGTTAGTTATATGGGGATCTTGCAGCCATTTAGTGACGCAG
GCAAGTTGTTATGCAAAGAATTTATTATGCCCACACGGGCTAATATGGGGCCTTTTATTTTGGCCCCAGCG
TTAATATTAACCGTAAGGCTTCTGGGATGGCTTTTATACCCTTACAAATCGGCGGAGGTATTTTATGTTTTT
GGCGTGATTCTTTTTATGGTAATTACTAGGGTAAGAGTATATGGGGTGATAATATCTGGGTGAGCTTCAAA
CTCAAAATATTCACTCTTAGGTGCGGTCCGTGCAATGGCGCAAAGAATCTCTTACGAGATTCCAATAGGA
TTTATCTTTTTCTGTGTAGTGTTAGTCTCTGGTGTGTTCATATTCCAAGAGATTAGGACGTCATTTTTTTTC
TTCCCATTATCTGTTATTCTTATTGTGTGAATTTTATGTATATTAGCAGAAACTAACCGTGCTCCTTTTGATT
TTGTGGAAGGGGAGTCTGAGTTAGTTTCTGGGTATAATGTGGAGTACAGAGGAGGAGGTTTTGCAGTGA
TGTTTATTGCTGAATATTCTAGAATTCTCCTTAGCAGAGTAATAAGGGCTGCAATATTCTTTGGTGGGAAC
GAGGCGCTAATTGGTTTTTTTATGATAGTTTTTGCGGTTTTTTTTTGTAATTGTGCGTGCTTCTTTACCGCGT
TTACGTTATGACAAACTGATAAGCTTGTGCTGAACTGTGCTCCTATGTGTCATGCTTATGGTTAGTGTATG
TGTGGTGACCATAATCAGAGTATAATGGTAAGTTTTGTAATAAGGCCTATAAAAGTAATAAGAATAAGGA
TAATGGTTATTGGGACAGTGCTTAGGGTTAGAAGAGAAGAATTAGTGGGTGTATGGTTGGGGATAGAATT
AAATTTGTATGGATTCCTTGTGGTTATGAACCCAGATGGGCACTATATTCCAGAGCCTTGTGTTAAATACT
TTGTAGTGCAAAGAACTGGATCTATTTTAATATTAGGAGGTTTTGTGCTATTGATGCAATTTGTAGTCAGG
GGTTTAATAATGAGAGTTATTGGAACTGTACTAAAATCTGGTATTTCTCCGCTGCATTCTTGGGTGCCTTC
AACTATCAAAAATAGAAGGTGGTTAGCGAGAGGGTTGATGTTAACTTGGCAAAAGGTAGCCCCTCTGGT
TTTATTGTCAATAATTATGCCCCTGAGAGGTATGTGGTTAGTTATCGGTCTAATAGCGATAATTGGGGCATT
GGGGGGTTTAAACCAAAATTCGGTGCGAGTTATAAGCGCTTATTCATCTTTTGTTCATACCTCTTGGATGC
TCTTAGGTCTTACCTGATCAAGAGTTGTGTTCGTAAGATATTTTTTAGTATATAGAATGTCAGTAGGGYTG
TTTTTTTATGGATGTTCCCTAATAAATAAGCTAAGAATAAGGAGACAACTAAGAGGGGCTGCAAGAGGA
ATAGGGCTTTTAATGCTTATGGGAATGCCCCCCTTCCTTGGGTTTTAGCTAAGGTGCTAGTGTTTCTGAT
AACAAGAAGGTCAGTAATTGTGTTGTGCATTGTAGGCTCAGTTATTAGGCTAAAATATTATATTGATTTCT
TTTATAGAATAGTTATAAAAAGTAAGAGAGGGGTGAAAAGCATGTGAATGTTAGTTATTATTATAAATTTA
ATAGGGGGAGTAGTAATTTTAGTAAGATTTCTATAAATGGTTATGGTAATGAGTGTTGTTTTTGTGTGTATT
GTATCACTTCTATTTACGGGATTGTTGTTATTGAGCGAAAAGCGCGGTCTTGATCGTGAAAAGTGTAGGC
CATACGAGTGTGGGTTTGAGCCTATTGGAAGGGCCCGCAGCCCTTTCTCGATTCGGTTCTTTCTCGTAGC
TGTATTATTTGTAGTGTTTGACGTTGAGGTAGTTTTATTAATACCTTTTGCTTATATGTTTCTTTATGGTAAG
AGGTTAATAGGAATCTTATCTTCAAGGGGGGTTTTTGTTTATTTTGTTTGTGGGGTTATACCATGAGTATCG
AGAAGGTTCTTTGGAGTGAGTGGGATAAATGGTTAAAAGGCTAGCAATCAGGCTTATTGCATTAATTATT
ATAAAAGACCTAAATATATCGATTATTGGACTGAGACTTTTAACAATTACAAGCTTGATGACAACGAGAA
CAGCGATTAGAAGGGTGGAGATGGGCGGCTTATACACGACAGATTTTGTGATGGGGTTGATGGTAACAT
TAACCTTGTTTGTCGCAGCACTTTCTCACCTAAGAAGGGTAAAAACAGCGCGCAAATCAAGGTTTAATT
TAATAGTCGTGAGAATCACTCTGATTTTACTGATAAGATTTAGGGTAAGAAGTTTCTTTCTTTTTTTTTTTT
TTCGAGAGAGTgtgATTAGCACCTCTGCTATTGCTAATTGTGGGATGGCGATTACAAGCAGGGGGtatatggttat
ttatactTTATATGGTTATTTATACTGTGTTTGGGCTCTTCTTTCTATGAGGCGTAAGGGCAAGGAGAAGAATA
AGTGTAGGAAATATAGTAAAGAAAAGCGCTATAAGACTGTGGTGACTGTATATTCTGGGCTTCTTAATAA
ACTGCCCATATACCCTTTCCATTTATGACTTCCTAAGGCTCATGTAGAAGCTCCGGTAGCAGGTTCGATAC

TGTTAGCTGGTGTAGTTCTAAAACTAGGTGGCTATGGATTATTACGGTTCATGAGAGTTATGCAGATAAAT
TTGAGAAGAGTATTCATCTTGCTGTTGCTTGTAAATCTGGCTGGAGGACTTTATGCCGGGTTGGTATGTG
TACGACAGGTGGACTTAAAATGTTTAGTAGCGTATTCATCTGTGGCTCATATGAGTTTGGTGTTGTTAGTG
TTGAGAAACACTCCAGTAGGGGTAATAGGGGCTGTTATCATTATGATTGGTCATGGCCTTTGTTCTTCTG
GTTTATTCAGATATGTTAACGTGGCCTATAAAATAAGCCACTCACGTCTTCTGGTAATAAACAAAGGAGG
GTTATTAATTTGTCCGAGACTAGTTTTAATGTGCTTTCTTCTAAGTTCTAGAAATATGGCGGCTCCCCCTA
GATTAAATTTATTTGGGGAAATCCTAGTATTTGGGGTTGGAGGTTGAATAAGGGGCCTATTTCTCTGTATC
CTGGGGTTAATAAGGTTTATTAGAGCCTGTTTTAGCTTATACCTGTATGGAAGGTGTAATCATGGAAAAG
GAATAATACATAGAGAGTCCTTAAGCTTCATGTGTGATATTATAATTTTAGCAGTTCATTGGGTGCCTTTA
AATTTCTTGTTTATATTTATGCCTTAGCGTATGAGCAAACACCTTCTTTGCTTGTTTGTGGGGCTTGAAAT
AATAAGTCTAGGGTTGCTGTTTGTAAGACATGTTTTTCTGATAAACCAGTTTTGGCTGATTCTTTTAATTC
TGTGCTTAGCTGTTTGTGAGGCTAGAATTTGTTTAGCACTTTTAGTGATAGTAATACGGTTATGCGGCAAT
GATTTAATGTCAAGATTAATAAGAGATGGAAACTAATAAGGAGAAATTTACCTCTTTTGGTATTAATTTGT
GGGTATTTGTCAATTTTCACTGGCAGTATCAGAAGAGCTTATTTGCTTGAAGTGCCTGTGTGGGATAGAC
ACTGCTTGTCATTTAGGTTTAGAATTCTGGCGGATAACGTAAGAATAATCTTTGTTGGGACTGTTTTAGTA
ATTAGAGGCAGGGTAGCAACTTACTGTAAGTGGTACATAGCTAGGGAAACATATTATAACCGTTTTATGG
GACTGGTATGGCTCTTTGTGCTTTCCATAATTTTTATGATTTAGTGCCTAATTTAGTAATGTTGCTTATTG
GATGGGATGGCTTAGGGCTTACGTCATTTTTGTTAGTAGCCTACTATCAAAACAACAAAAGTCTATCGGC
AGCCATGTTGACGGCTTTAACAAATCGGATCGGAGATGTTCTCGTTCTGGTCAGCATTTCTATCCTTTTAA
ATGAGGGTGGATGATTAGTTTACAGCTATTATCCTGTGAGAATGTGGAGGTTAAGAGTCGTCGTGGTTCT
TGCAGGAATAACTAAAAGAGCGCAGATGCCTTTTTGTGCTTGGCTTCCAGCTGCTATGGCTGCTCCAAC
ACCTGTGTCGTCTTTGGTGCATTCTTCTACTTTAGTAACAGCAGGGGTTTATTTAGTGCTTCGCTCATTCT
ATGTGGTTAGGGCTAACGCAACTCAGATATTGATAATCTTAAGACTGTTTACCTTAGTGTTAGCTGGTTCA
AGCGCAGTGTTTGCGTTTGATTTAAAGAAAGTGATTGCATTATCCACTTTAAGCCAATTGAGCCTAATAA
TGTTTTCTATTTCTATTCTTCTTCCGTTTGTGGCTTTTTTCCATTTGGTAACTCACGCGGTGTTTAAAGCCT
TGCTCTTCTTGGGGGCGGGGGGTGTTATCCATAGAAACCAGAGGATTCAAGATATCCGAGGTTTAAGAA
GTTTATGGCAAAGACTGCCTGTGAGAATAAGAGCAATAAGTGTGGCTATTGTGTCCTTAAGTGGGGCCC
CGTTTATAAGAGGATTTATTCTAAAGACTTAATTATTGAGATAATAGACAGAAGAACTTATGGGTATATAT
TAGAATTATTAGGCCTAATTTTTACCTCCTTTTACAGAGCTCGGGTGTTTAGGGTAATATTAGGGTCAAAC
TATATTAATTGTAGGACTCTTCGGCTATTTGAGCACCTAAACATGCAGGTTCCTTTTTTGAGACTATATGTA
GGTGCTATTTTTTTAGGGTTAAGACTTGGTAGGACAATAGAGAAGTTTGGGTATGTAGTGGTACTTGAAA
GGTATGAGAGGCTTAGAATCTTTTTAATTCCTTTTGGCTTAATATGATGGGGAGTACTAACTAAACTAGGG
TTTAACCCGACTAAATTGAGGTTTTTCTTGAGGATGTGGCTTGTGGAGCTTACTCATCCTGGAAAGACTT
TATTTTTTAAAAGCTCTCTAATAATGTATCAGACATTAGATCAAGGATGGCTAGAATTATTAGGTCCGCAG
GCTAAGTTAGGACAAGTTAGCCAGTTAAACGAGAATTATTTTACGGTTGCTTGAGTAACAATTGCAGGTT
TGAGGTTAGTTGTTTTTATGTAAATGAGGGTTATAGTAATGTGTGTGGGTTTGATGTTTGTGTTCGTGTTA
ATTGCCAAACAACCAATTTCCTTAGGGTTGGTGTTGTTGATAGGTTCTATAATCTCATGTGTAGAAATTGC
GTTGGAAATCAGAAGGTTATTAGGGTTTTTGTTGTTTCTGACTTATGTTAGAGGTGTAATAGTACTATTCT
TATATGTGCTAAGAATTTATCCTAATGAACGGTTTAATTTGGAGTTTATTGTAATTGTAATAAGATGTATTG
TGACAAGGCTGGTTATCACAATGAATTATGAGAATGGGTCATTATTTCTTAGGTTTATAGCTGAAGGGAG
ACTCTATATTCTAATAGCGGGCGTATTGCTGTTTGTAATGTTAGTGGTGTCTTATTTGTGTATGAAAACTAT
GGTGCCACTTCGTAGGGTA"/>
<sequence id="seq_Mytilus_coruscusKart11" spec="Sequence" taxon="Mytilus_coruscusKart1" totalcount="4"
value="atgttaATAGATGTTTTTCTAGTTTTGATGCTCATAGTTACAATTTGATTTGGCTATCAATGTTGTGGG
TTCTTTCTTCTATGGTACCACTAACTGTATTATTTAGGGACGTGAGTATTCGTAGTTTAGTGTTATCGTTTA
CTTATTCAATGATTCGTAACGGAAAAGGGCTCAAACTTTCCGGGTTTCCATTAGTGATAAGTGGGCTATT
TCTTATAATTTTAATATTAAATTTGTCGGGAAACTTTCCGTTTTTTTCCCTGTAAGGGGTCAGTTTGTGTT
TGGGTTTTCTTTTGCGCTATCTGTGTGAACTTGTTTAGTATTATCTAGTTTATTGTGTAGGTTTGAACAGA
GATTAATAAGGCTGGTCCCAACTGGGCCACTGATTCTGGTGCCTTTTATAGTAGCGGTTGAATTAATCAG
TGGTATGCTACGCCCTTTGACTTTGGTGTTGCGTTTAACACTGAATCTTGGAGCTGGAAAAGTGATTTTG
ACGATATGTAGGAGAGAGCTAGTAGTAGGGTGATTAATTATAGGAGTAGGGGGAGTTAAAGGGCTTTTA
ATAGGAGGAGTTTTTGCTGCTGAGGTAGCAATTGCCTGTATTCAATGTTATATCTTCTGTGTACTTCTGTG
TCTTTATACTGAGGATCATAGAAGTTAACGATGACTTTGATCAACAAACCATAAGGATATTGGAACTCTTT
ATTTATATAGAGGGGTATGAGGAGGCTTATTTGGGGCAAGACTAAGTCTGATAATTATGCAGGGGCACCC
TGGAGCAGTTTTTTTAAAAGACTGATTTTATAATGTAGTAGTAACAACGCATGCTCTGATAATGATTTTCT
TTGCTGTGATACCCATTTTAATTGGGGCCTTTGGTAACTGGCTTATTCCTTTGCTTGTTGGGGGAAAAGA
CATAATCTATCCACGCATAAACAATCTTAGATATTGACTGTCGCCGAATGCTCTATATTTATTGATGTTATCT
TTTAGGACAGATAAAGGAGTGGGGGCAGGGTGGACGATTTATCCCCCTCTATCTGTGTACCCCTATCATA
GAGGGCCCAGAATGGATGTTCTTATTGTGTCTCTTCATCTGGCAGGACTCAGGTCTTTAGTGGGAGCTAT
TAATTTTGCTAGCACAAATAAGAACATACCAGTATTAGAAATAAAAGGGGAACGGCTGAGCTTTATGTG
CTAAGAATCAGAGTTACTGCGGTTCTTTTAATTATTTCGATTCCGGTGTTAGGAGGGGGGATCACAATAA
TTTTGTTTGATCGTAATTTTAACACTACTTTCTTTGATCCTGCAGGAGGAGGTGACCCGGTGCTATTTCAG

CATCTATTCTGATTTTTTGGGCACCCTGAAGTATACATTCTTATTTTGCCTGCTTTTGGGGTAATATCTAAG
GTTATTATACATTGCTCTGGAAAAGAGGCGGTGTTTGGGTTAATTGGGATAGTTTATGCTATAATTGGAAT
TGGAGGACTAGGTTGTATGGTGTGAGCACATCATATATTTACGGTAGGGCTTAATGTGGATACTCGGGGG
TACTTCTCAACTGCTACTATAGTAATTGCAGTTCCTACAGGAGTAAAAGTGTTTAGTTGACTAGCAACTAT
GGCAGGAAGAAAGTTCAAAATAAAACCAGCTGCCTTCTGAAGAACAGGTTTTCTGTTTTTATTTACTGT
AGGGGGGTTAACAGGGGTAATATTGTCGAGAGCTTCTATAGATGTGTCACTCCATGATACTTACTATGTG
GTTGCGCATTTTCATTATGTGCTTAGTATGGGGGCTGTGTTTGGAGTTTTCTGTGGTTTGAACCACTGATT
ACCCAATTTTGTTGGAGTTTGTTTTAATAAAAAGTGAAGAAAAGCCCATTTTATAGCTATATTCTTTGGGG
TAAATACCACGTTTTTTCCTCAACACTTTTTAGGGCTAAGAGGTATACCACGACGGTATATAGACTACGCT
GATATTTATGCTCACTGACATTGGGTGTCTTCTTATGGGTCTGCTGTTTCATTTGGGTCTCTTATATATTTTA
AGTTTCTACTTTGGGAGGCTCTAGTAAGCCAGCGAGGAATGTCTTTTTATGGGAGTCGATATTTTGGTGA
TATTGTCCATGAATTAGGAAAAGACCTTTTTCGTTACCATGGCTTTGTAATGATGGTGGCGGTAGCCGTG
TTAGTTTTTGTTATATACATAGGATGCGTGATCCTTCTTACTAAGTTTTCTTATCGCCATTTTTTAAATCGTC
AACGGTTAGAGTTTTGGTGAACAATCGTACCCATATTTTTACTAGTGGGGCTATGGTTTCCGTCAATAATT
AACTTGTACTATATAGAAGAAGTCAAGCGACCACGTTGGAATTTTAAGGCAATTGGAAAGCAGTGATAT
TGGTCCTATGAGTGTGACACTTGTTACACAATCGACTCGTACATAGAGGATCAGCAAGAAACTGGTTATC
GGTTGTTAGATGTGGATAATCGGATGGTTGCTCCTGCGGACGTACAAATAACTGCTTTTGTAAGAAGCTC
AGACGTTCTCCATTCGTTCGCTCTTCCTAAGCTTCTACTAAAAGTGGATGCTATTCCAGGCCGAATTAATC
GCCTGCCGATAAAGGCGTCTCAATGTAGGATTATTTATGGGCAGTGTTCTGAAATCTGCGGGGTAAACCA
TAGGTTTATACCTATCGTAATTGAGTTTATTCCTGAAAAATATTTCGTGATATGATTGGAAGCTCTAAATtaa-
----

ATGAAACGTAATCCTTATTATGTGCCTGGTCCCAGGCCGTGGCCATTCTTTGTGGCTATTTCTGCTAATGG
TATGGCGGTAGGGTTAATCTTATGATTGCATCGGACTCCTTTTCTTCTTATGAGTAGCCTGAGTTGTATGTT
ATTGAGAACCTTTAGTTGATGGCGAGATTTAATTCGCGAAGGAGATATTGGTTTTCATACTCGTTTTGTAA
TTAAGAGGTTTCGAGATGGAGTGGCATTGTTTATCCTGTCGGAGGTAATATTTTTTTTCTCCTTTTTCTGA
ACTTTTTTCCATAACGCCTTGAGCCCTTCTTGTGAACTTGGGATACGATGGCCTCCTCCTGGAATTCGGA
CTCCTAACCCATCATCTACAAGTCTGTTTGAGACAGGTTTGTTAATCAGAAGAGGTCTGTTTGTAACTCA
AGCTCATAAAAGAATGCGGCTAGACTATGATGTCGGTCCCTTTATTGGGTTGGTAGTTACAATCTTATGTG
GTACTCTGTTCTTCTTAGTTCAATTACGGGAATACTATTGGAATTCCTATACTATTGCAGATAGGGTGTACG
GAAGGGTTTTCTATTTGTTAACTGGATTTCATGGGATACATGTGGTTGTGGGGACGCTCTGGCTGATAGT
CAGATTGGTTCGGCTATGACGTGGGGAATTCTCTAGACAACGACATTTTGGGTTCGAGGCTTGTATCTGG
TATTGACATTTTGTGGATGTAGTTTGAGTTGCGTTGTGATGTTTAGTGTATGTATGATTTGGAGGACCGTG
ACGAAAAACTAATAAGCTGGTAAAAATCATAAATGATAGATTCTATGATTTACCGTGTCCTGTGAACTTA
AATGCTTGATGGAGATTTGGCTCCATATTAGGTTTATGTCTTGTAATCCAACTTTTAAGTGGTCTTTTGTTG
TCGATTCATTATACGGCCCACGAAGATATAGCATTTGACTCTGTTGTGCATATTATGCGCAATGTTAAAAA
AGGTTGAATGTTGCGAAATATCCATGCTAATGGGTCTTCTATATTTTTTATCTGTATTTATGCCCACATTGG
TCGTGGTTTGTACTACGGCTCATATTTGGACAAAACAGTTTGGTATTTTGGGGTTCATCTTTTGTTATTAA
CTATGGCTGAGGCCTTTTTAGGTTACACACTACCTTGAGGTCAGATATCATATTGAGGTGCCACTGTGATT
ACTAATATACTTAGGGTGATTCCTGTAGTAGGAGAAAGAATACTTCGTTATGTGTGAGGTGGTTGGACAG
TCTGTAATGCAACGCTAAAGCGGTTTTATACCCTTCATTTTCTTCTTCCATTTGTTATAGTGGCGGTTGTAT
TTCTTCACCTTTTTTTCCTGCATGAAAAAGGGAGGAATAACCCTTTGGGGATTGAAAGTGATACTGTGTG
TGTTCCTTTTCATCCTTTTTACACTGTGAAAGATTTATTTGGCTATATTTGTTTTAGGTTTTTTTTTATGTAC
TTAGTATGTGTAGACCCTGAGTTATTAGGAAATCACTTAAATTATTGGCCTGCTAACCCGATAAAAACCCC
TATTCATGTCCAGCCAGAGTGATATTTTATATTTGCTTACGCTATTCTCCGTTCAATTCCTCATAAAGCAGG
GGGTGTATATGTTATATTTTTGTCTATTGTGGTCCTGTATCTGATTCCCAGGTTGCACAGAGGTAAATATCG
AAGATTGTGTTTTTACCCCTTTAACCAAGTCGTGTTTTGAGTTCTAGTGGGGAGCTTTATTAGCTTGACG
TGAATTGGGGCTCGTCCAGTTCGTGAGCCTTATATTGTGATAGGCCAATGTTTTTCGATTATTTATTTTTCG
AGGCTTCTATTGAACCCCCTTTCATTATGGATGTGGGATAAATTGCTAGATTTGGGAGTGGTAATTGGGAT
CGTGCCTTTTGTTGGGGTACTTCTTGCTGTAGGGTTTTATACCTTATTGGAGCGCAAAATTTTAGCTATTAT
TATAATCCGCAAAGGACCATCTAAGGTTAGTTATATGGGGATCTTGCAGCCATTTAGTGACGCAGGCAAG
TTGTTATGCAAAGAATTTATTATGCCCACACGGGCTAACATGGGGCCTTTTATTTTGGCCCCAGCGTTAAT
ATTAACCGTAAGGCTTCTGGGATGGCTTTTATACCCTTACAAATCGGCGGAGGTATTTTATGTTTTTGGCG
TGATTCTTTTTATGGTAATTACTAGGGTAAGAGTATATGGGGTGATAATATCTGGGTGAGCTTCAAACTCA
AAATATTCACTGTTGGGTGCGGTCCGTGCAATGGCGCAAAGAATCTCTTACGAGATTCCAATAGGATTTA
TCTTTTTCTGTGTAGTGTTAGTCTCTGGTGTGTTCATATTCCAAGAGATTAGGGCGTCATTTTTTTTCTCCC
CATTATCTGTTATTCTTATCGTGTGAATTTTATGTATATTAGCAGAAACTAACCGTGCTCCTTTTGATTTCGT
GGAAGGGGAGTCTGAGTTAGTTTCTGGGTATAATGTAGAGTACAGAGGAGGAGGTTTTGCAGTGATGTT
TATTGCTGAATATTCTAGAATTCTTCTTAGCAGAGTAATAAGGGCTGCAATATTCTTTGGTGGGAACGAGG
CGCTAATTGGTTTTTTTATGATAGTTTTTGCGGTTTTTTTTGTAATTGTGCGTGCTTCTTTACCGCGTTTGC
GTTATGACAAACTGATAAGCTTGTGCTGAACTGTGCTCCTATGTGTCATGCTTATGGTTAGTGTATGTGTG
GTGACCATAATCAGAGTATAATGGTAAGTTTTGTAATAAGGCCTATAAAATTAATAAGAATAAGGATAATG
GTTATTGGGACAGTGCTTAGGGTTAGAAGAGAAGAATTAGTGGGTGTATGGTTGGGGATAGAATTAAATT
TGTATGGATTCCTTGTGGTTATGAACCCAGATGGGCACTATATTCCAGAGCCTTGTGTTAAATACTTTGTA

GTGCAAAGAACTGGGTCTATTTTAATATTAGGAGGTTTTGTGCTATTGATGCAATTTGTAGTCAGGGGTTT
AATAATGAGAGTTGCTGGAACTGTACTAAAATCTGGTATTTCTCCGCTGCATTCTTGGGTGCCTTCAACT
ATTAAAAATAGAAGGTGGTTAGCGAGAGGGTTGATGTTAACTTGACAAAAGGTAGCCCCTCTGGTTTTA
TTGTCAATAATTATGCCCTTGAGAGGTATGTGGTTAGTTATCGGTCTAATAGCGATAATTGGGGCATTGGG
GGGTTTAAACCAAAATTCGGTGCGAGTTATAAGTGCTTATTCATCCTTTGTTCATACCTCTTGGATGCTCT
TAGGTCTTACCTGATCAAGAGTTGTGTTCGTAAGATATTTTTTAGTATATAGAATGTCAGTAGGGTTGTTT
TTTTATGGATGTTCCCTAATAAATAAGCTAAGAATAAGGAGACAACTAAGAGGGGCTGCAAGAGGAATA
GGGCTTTTAATGCTTATGGGAATGCCCCCCTTCCTTGGGTTTTTAGCTAAGGTGCTAGTGTTTCTGATAAC
AAGAAGGTCAGTAATTGTGTTGTGCATTGTAGGCTCAGTTATTAGGCTAAAATACTATATTGATTTCTTTT
ATAGAATAGTTATAAAAAGTAAGAGAGGAGTGAAAAGTATGTGAATGTTAGTTATTATTATAAATTTAATA
GGGGGAGTAGTAATTTTAGTAAGATTTCTATAAATGGTTATGGTAATGAGTGTTGTTTTTGTGTGTATTGT
ATCACTTCTATTTACGGGATTGTTGTTACTGAGTGAAAAGCGCGGTCTTGATCGTGAAAAATGTAGGCCA
TACGAGTGCGGGTTTGAGCCTATTGGAAGGGCCCGCAGCCCTTTCTCGATTCGGTTTTTTCTCGTAGCTG
TATTATTTGTAGTGTTTGACGTTGAGGTAGTTTTATTAATACCTTTTGCTTATATGTTTCTTTATGGTAAGAG
GTTAATGGGAATCTTATCTTCAAGGGGGTTTTTGTTTATTTTGTTTGTGGGGTTGTACCATGAGTATCGAG
AAGGTTCTTTGGAGTGAgtgggataaATGGTTAAAAGGCTAGCAATCAGGCTTATTGCATTAATTATTATAAAA
GACCTAAATATATCGATTATTGGACTGAGACTTTTAACAATTACAAGCTTGATGACAACGAGAACAGCGA
TTAGAAGGGTGGAGATGGGCGGCTTATACACGACAGATTTTGTGATGGGGTTGATGGTAACATTAACCTT
GTTTGTCGCAGCACTTTCTCACCTAAGAAGGGTAAAAACAGCGCGCAAATCAAGGTTTAATTTAATAGT
CGTGAGAATCACTCTGATTTTACTGATAAGATTTAGGGTAAGAAGTTTCTTTCTTTTTTTTTTTTTTTTCG
AGAGAGTGTTAGCACCTCTGCTATTGCTAATTGTGGGATGACGATTACAAGCAGGGGGTTATATGGTTAT
TTATACTGTGTTTGGTTCGCTCTTCTTTCTATGAGGCGTAAGGGCATTATATGTAAGAGGAAGGAGAAGA
ATAAGGGTAGGAAATATAACAAAGAAAAGTGCTATAAGACTGTGGTGACTGTATATTCTGGGCTTCTTAA
TTAAATTGCCCATATACCCTTTCCATTTATGACTTCCTAAGGCTCATGTAGAAGCTCCGGTAGCAGGTTCG
ATGCTGTTAGCTGGTGTAGTTCTAAAACTAGGTGGCTATGGATTACTCCGGTTCATGAGAGTTATGCAGA
TAAATTTGAGAAGAGTATTTATTTTGCTGTTGCTTGTAAATTTGGCTGGAGGACTTTATGCCGGGTTGGTA
TGTGTACGACAGGTGGACCTAAAATGTTTAGTAGCGTATTCATCTGTGGCTCATATGAGTTTGGTGTTGTT
AGTGTTGAGAAACACTCCAGTAGGGGTAATAGGGGCTGTCATCATTATGATCGGTCATGGTCTTTGTTCT
TCTGGTTTATTCAGGTACGTTAACGTGGCGTATAAAATAAGCCACTCACGTCTTCTGGTAATAAATAAAG
GAGGGTTATTAATTTGTCCGAGACTAGTTTTAATGTGTTTTCTTCTAAGTTCTAGAAATATGGCGGCTCCC
CCTAGATTAAATTTATTTGGGGAAATCCTAGTATTTGGGGTTGGAGGTTGAATAAGGGGCCTATTTCTCTG
TATCCTGGGGTTAATAAGGTTTATTAGAGCCTGTTTTAGCTTATACCTGTATGGAAGGTGTAATCATGGAA
AAGGAATAATACATAGAGAGTCCTTAAGCTTTATGTGTGATATTATAATTTTAGCAGTTCATTGGGTGCCT
TTAAATTTCTTGTTTATATTTATGCCTTAGCGTATGAGCAAACACCTTCTTTGCTTGTTTGTGGGGCTTGA
AATAATAAGTTTAGGGTTGCTATTTGTAAGACATGTTTTTCTGATAAACCAGTTTTGGCTGATTCTTTTAAT
TCTGTGCTTAGCTGTTTGTGAGGCTAGAATTTGTTTAGCACTTTTAGTGATAGTAATACGGTTATGCGGCA
ATGATTTAATGTCAAGATTAATAAGAGATGGAAACTAATAAGGAGAAATTTACCTCTTTTGGTATTAATTT
GTGGGTATTTGTCAATTTTCACTGGCAGTATCAGAAGAGCTTATTTGCTTGAAGTGCCTGTGTGGGATAG
ACACTGCTTGTCATTTAGGTTTAGAATTCTGGCGGATAACGTAAGAATAATCTTTGTTGGAACTGTTTTAG
TAATTAGAGGCAGGGTAGCAACTTACTGTAAGTGGTACATAGCTAGGGAAACATATTATAACCGTTTTAT
GGGACTGGTATGGCTCTTTGTGCTTTCCATAGTTTTTATGATTTTAGTGCCTAACTTAGTAATGTTGCTTAT
TGGATGGGATGGCTTAGGGCTTACGTCATTTTTGTTAGTAGCCTACTATCAAAACAACAAAAGTCTATCG
GCAGCCATGTTGACGGCTTTAACAAATCGGATCGGAGATGTTCTCGTTCTGGTCAGCATTTCTATCCTTT
TAAATGAGGGTGGATGATTAGTTTACAGCTATTACCCTGTGAGAATGTGGAGGTTAAGAGTTGTCGTGGT
TCTTGCAGGAATAACTAAAAGAGCGCAGATGCCTTTTTGTGCTTGGCTTCCAGCTGCTATGGCTGCTCCA
ACACCTGTGTCATCTTTGGTGCATTCTTCTACTTTAGTAACAGCAGGGGTTTATTTAGTACTTCGCTCATT
CTATGTGGTTAGGGCTAACGCAACTCAGATATTGATAGTCTTAAGACTGTTTACCTTAGTGTTAGCTGGTT
CAAGCGCAGTGTTTGCGTTTGATTTAAAGAAAGTGATTGCATTATCCACTTTAAGCCAATTGAGCCTAAT
AATGTTTTCTATTTCTATTCTTCTTCCGTTTGTGGCTTTTTTCCATTTGGTAACTCACGCGGTGTTTAAAGC
CTTGCTCTTCTTGGGGGCGGGGGGTGTTATCCATAGAAACCAGAGGATTCAAGATATTCGAGGTTTAAG
AAGTTTATGGCAAAGACTGCCTGTGAGAATAAGAGCAATAAGTGTGGCTATTGTGTCATTAAGTGGGGC
CCCGTTTATAAGAGGATTTTATTCTAAAGACTTAATTATTGAGATAATAGACAGAAGAACTTATGGGTATA
TATTAGAATTATTAGGCCTAATTTTTACCTCCTTTTACAGAGCTCGGGTGTTTAGGGTAATATTAGGGTTGA
ACTATATTAATTGTAGAACTCTTCGTCTATTTGAGCACCTAAACATGCAGGTTCCTTTTTTGAGACTATATG
TAGGTGCTATTTTTTTAGGGTTAAGACTTGGTAGGACAATAGAGAAGTTTGGGTATGTAGTGGTACTTGA
AAGGTATGAGAGGCTTAGAATCTTTTTAATTCCTTTTGGCTTAATATGATGGGGAGTACTAACTAAACTAG
GGTTTAACCCGACTAAATTGAGGTTTTTCTTGAGGATGTGGTTTGTGGAGCTTACTCATCCTGGAAAGAC
TTTATTTTTTAAAAGTTCTCTAATAATGTATCAGACATTAGATCAAGGATGGCTAGAATTATTAGGTCCGC
AGGCTAAGTTAGGACAAGTTAGCCAGTTAAACGAGAATTATTTTACGGTTGCTTGAGTAACAATTGCAG
GTTTGAGGTTAGTTGTTTTTATGTAAATGAGGGTTATAGTAATGTGTGTGGGTTTGATGTTTGTGTTCGTG
TTAATTGCCAAACAACCAATTTCCTTAGGGTTGGTGTTGTTGATAGGTTCTATAATCTCATGCGTAGAAAT
TGCGTTGGAAATCAGAAGGTTATTAGGGTTTTTGTTGTTTCTGACTTATGTTAGAGGTGTGATAGTACTAT
TCTTATATGTGCTAAGAATTTATCCTAATGAACGGTTTAATTTGGAGTTTATTGTAATTGTAATAAGATGTA

TTGTGACAAGGCTGGTTATTACAATGAATTATGAGAATGGGTCATTATTTCTTAGGTTTATAGCTGAAGGG
AGACTCTATATTCTAATAGGGGGCGTATTGCTGTTTGTAATGTTAGTGGTGTCTTATTTGTGTATGAAAACT
ATGGTACCACTTCGTAGGGTA"/>
<sequence id="seq_Mytilus_edulisF-MF4076761" spec="Sequence" taxon="Mytilus_edulisF-MF407676" totalcount="4"
value="ATGTTAATAGATGTATTTCTAGATTTGATGCTCACAGCTACAACCTAATTTGGTTATCTATGTTGT
GGCTACTTTCGTCTATAGTACCAATAACTGTGCTATTTAGAGATGTAAGCACTCGAAGCTTGGTACTGTCT
TTTACTTATTCTATAATCCGGAATGGGAAAGGTTTAAAGTTATCTGGGTTTCCTTTGGTGATAAGCGGCTT
GTTTATAATAATTTTAATGTTGAATTTGTCTGGGAACTTCCCTTTTTTTTTTCCTGTAAGAGGTCAGTTTGT
ATTTGGATTTTCTTTTGCTTTATCTATTTGGACCTGTTTAGTACTATCTAGCTTACTGTGCAGTTTTGAGCA
GGGTTTGATGAGGCTTGTTCCGACAGGTCCGTTAATCCTTGTGCCTTTTATAGTAGTAGTTGAGCTAATTA
GTGGGATACTTCGCCCTTTAACTCTAGTCTTGCGGCTAACGTTGAATTTAGGGGCTGGTAAAGTAATCTT
AACTATATGTAGAAGGGAGTTGGTAGTCGGTTGGTTAATTACAGGAGTTGGGGGTATTAAAGGTTTGCTA
ATAGGTGGTGTCTTTGCTGCTGAAGTTGCAATTGCTTGTATTCAGTGTTACATTTTTTGTGTCTTATTGTG
TCTCTATACTGAGGACCATAGGAGTTAGCGATGGTTGTGGTCAACAAATCATAAAGATATTGGGACTCTT
TATCTATATAGTGGGGTCTGAGGAGGCTTGTTCGGGGCAAGGTTAAGTCTGATAATCATACAGGGGCATC
CTGGAGCAGTATTTTTAAAAGACTGGTTTTATAATGTGGTTGTTACAACACACGCCTTAATAATAATTTTC
TTTGCTGTAATACCTATTCTAATCGGAGCTTTTGGTAATTGGCTGATTCCTCTATTAGTAGGTGGTAAAGAT
ATAATTTATCCGCGGATAAATAATTTGAGTTATTGGTTATCTCCTAATGCGCTATATTTACTTATATTATCTTT
TAGAACGGATAAAGGGGTAGGTGCTGGATGGACTATTTACCCGCCATTGTCTGTATACCCTTATCATAGC
GGGCCGAGGATAGATGTTCTTATTGTGTCGTTGCATTTAGCTGGGTTAAGTTCTTTGGTGGGTGCTATTAA
TTTTGCTAGTACCAACAAAAACATACCAGTTTTAGAGATAAAAGGAGAACGAGCTGAGCTTTATGTCCT
AAGGATTAGAGTTACTGCCGTATTGCTAATTATTTCTATTCCGGTTTTAGGAGGGGGTATCACAATAATTC
TGTTTGATCGGAATTTTAACACAACATTTTTTGATCCAGCAGGAGGGGGTGACCCCGTCTTGTTTCAACA
TTTGTTCTGATTTTTTGGGCACCCTGAGGTGTACATTCTTATTCTACCTGCTTTTGGTGTGATATCAAAAG
TAATTATGCATTGTTCCGGGAAGGAGGCAGTTTTCGGGTTAATTGGGATGGTGTATGCAATAATTGGAATT
GGAGGGCTAGGTTGTATGGTGTGGGCTCACCATATATTTACAGTAGGGCTTAATGTTGATACTCGAGGTT
ATTTCTCTACTGCAACTATAGTAATCGCAGTCCCTACAGGGGTAAAAGTATTCAGGTGGTTAGCAACTAT
AGCAGGAAGAAAATTTAAGATAAAGCCTGCTGCTTACTGAAGTACGGGGTTCTTGTTTCTATTCACCGTA
GGAGGCTTAACAGGGGTGTTATTATCAAGGGCTTCTATGGACGTATCGCTTCATGATACTTATTATGTGGT
GGCTCATTTCCACTATGTGCTAAGTATAGGAGCGGTGTTCGGAGTATTTTGTGGCCTGAATCATTGATTGC
CTAACTTTGTTGGAGTATGCTTTAATAAGAAATGGAGAAAAGCCCACTTTATAGCAATGTTTTTTGGTGT
AAATACCACTTTCTTTCCTCAACACTTTCTAGGGCTGAGGGGTATGCCTCGACGGTACATAGATTATGCT
GACATTTATGCTCATTGGCATTGGGTGTCTTCTTATGGGTCTGCAGTGTCTTTTGGCTCTTTGATATATTTT
AAGTTCCTACTCTGAGAAGCTTTAGTGAGTCAACGAGGGATGTCTTTTTATGGGAGTCGATATTTTGGTG
ATATTGTCCATGAATTGGGGAAGGACCTATTTCGGTATCATGGATTTGTGATGATAGTAGCAGTGGCTGTA
TTAGTCTTTGTTATGTATATAGGGTGCGTGATCCTGTTTACTAAATTTTCTTACCGTCATTTTTTAAATCGCC
AACGATTGGAGTTTTGATGAACTATTGTGCCGATGTTGATGTTAGTAGGGCTGTGGTTCCCTTCGATGATT
AATTTGTATTATATGGAAGAAGTAAAGCGTCCACGTTGAAATTTCAAAGCAATTGGAAAGCAATGATACT
GATCTTATGAGTGCGATACCTGTTATACAATTGACTCATACATAGAAGACCAACAGGAGACAGGGTACCG
CTTGCTGGATGTTGACAATCGGATGGTTGCTCCTGCAGATGTACAAATAACTGCTTTTGTAAGGAGTTCT
GACGTGCTACATTCGTTTGCGCTTCCTAAATTATTAATCAAAGTAGATGCTATTCCGGGCCGAATCAATCG
GCTTCCGATAAAAGCATCTCAGTGTAGCATTATTTATGGGCAGTGCTCTGAAATTTGTGGAGTAAACCAC
AGGTTTATACCAATTGTGATTGAATTTATTCCTGAGAAATATTTTGTTATATGGTTGGAAGCTCTTAATTAG

ATGAATCGTAATCCTTACTATGTACCAGGCCCAAGTCCGTGGCCATTTTTTGTGGCTATTTCGGCTAACGG
AATAGCGGTAGGGTTAATTTTGTGACTGCATCGAACCCCCTTTTTATTAATAGGTAGTCTGGTTTGCATAT
TATTGAGAACTTTTAGATGGTGACGCGATTTAATTCGTGAAGGAGACATTGGGTTTCACACTCGTTTTGT
AATCAAAAGATTTCGAGATGGCGTTGCCTTGTTTATTCTGTCTGAAGTGATATTTTTCTTCACTTTTTTCT
GGACTTTTTTCCATAATGCTTTAAGGCCCTCGTGTGAGCTAGGAATACGGTGGCCCCCTCCTGGAATTCG
TACGCCAAACCCGTCATCTACTAGTCTGTTTGAGACAGGTCTTCTAATTAGAAGAGGGCTGTTTGTAACT
CAAGCCCATAAGAGGATGCGCTTGGATTACGACGTAGGGCCATTCATCGGCTTAGTGGTGACAATCGTAT
GCGGGACTGTGTTTTTCTTGGTGCAACTGCGGGAATATTATTGAAACTCCTATACTATCGCAGATAGGGT
TTATGGTAGGGTTTTTTACTTACTAACTGGATTCCACGGGATACATGTTGTCGTAGGGACTATTTGACTAA
TGGTAAGGTTAGTTCGACTATGACGCGGGGAGTTTTCTAGTCAACGACATTTCGGGTTTGAGGCTTGTAT
TTGGTACTGACATTTTGTAGATGTGGTATGAGTGGCATTGTGGTGCCTAGTGTATGTGTGGTTTGGAGGA
CCGTGACGAAGTACAAATAAACTGGTAAAGATTATGAATGATAGATTCTATGATTTGCCTTGTCCTGTAA
ACTTAAACGCCTGGTGAAGGTTTGGCTCTATACTAGGCTTGTGTCTGGTTATCCAACTTTTGAGGGGTCT
TTTATTATCAGCCCACTACACTGCTCATGAAGACATGGCATTTGATTCTGTAGTCCACATTATGCGTAATG
TGGAAAAGGGGTGAATATTGCGCAATATCCATGCAAATGGGTCTTCTATGTTCTTTATTTGCATTTATGCT
CACATTGCTCGTGGGTTGTACTATGGGTCTTATTTGGATAAGACCGTGTGGTACTTTGGGGTACACCTGT
TCTTGTTGACTATGGCTGAGGCTTTTCTCGGCTATACTCTGCCTTGGGGGCAGATATCGTACTGGGGGGC
TACTGTGATCACTAATATACTTAGAGTAATCCCTGTGGTAGGTGAAAGGATACTTCGCTACGTATGAGGG

GGGTGGACTGTGTGTAATGCAACGTTAAAGCGGTTTTACACACTTCACTTCCTTTTACCGTTTGTGATAG
TGGCGGTTGTTTTTTTGCACTTATTTTTTTTACATGAGAAGGGTAGTAATAACCCTTTAGGAATTGAAAGG
GGCACTATATGTGTGCCTTTTCATCCTTTCTATACTATTAAGGACCTCTTTGGTTATGTTTGCTTTAGGTTC
TTTTTTATGTATTTAGTGTGCGTAGACCCTGAGCTTCTAGGGAATCATCTAAACTACTGGCCTGCTAACCC
TATAAAAACGCCTATTCACGTTCAGCCTGAATGGTACTTTATATTCGCCTATGCAATTCTCCGTTCAATTCC
GCATAAAGCAGGGGGAGTATATGTTATGTTCCTTTCAATCGTAGTGCTATACTTAATTCCTACGCTTCATAC
AGGTAAGTACCGAAGTTTATGCTTCTACCCATTGAATCAAGTAGTGTTTTGGGTGTTGGTTGGAAGGTTT
ATTAGCCTAACATGAATTGGTGCTCGTCCAGTGCGTGAACCTTATATCATTTTGGGGCAGTGTCTTTCAGT
TATTTATTTCTCTAGTTTATTGTTGAACCCCCTTTCTTTATGGGTGTGAGATAAATTACTTGAAGTGGCAGT
AATTGTAAGTATTATCCCTTTCGTAGGTGTCCTTCTTGCTGTAGGGTTTTACACCTTATTGGAGCGTAAGA
TTCTGGCCATCATCATGATCCGAAAAGGCCCGTCCAAGGTAAGTTACATGGGGATCTTGCAGCCTTTTAG
TGATGCAGGTAAGCTATTATGTAAAGAGTTTATCGTGCCCACGCGTGCTAATGTAGGGCCATTTATTCTGG
CTCCTGCACTAATACTAGCTATTAGCTTATTAGGGTGGCTTTTATACCCCTATAAGTCAGCTGAAGTGTTTT
ATGTTTTTGGGGTGATTTTGTTTATAGTTATTACTAGAGTTAGCGTTTATGGGGTAATAATGTCTGGATGGG
CATCTAACTCCAAATACTCCTTACTAGGTGCAGTCCGTGCGATGGCGCAAAGAATTTCTTATGAAATCCC
TATAGGGTTTATTTTTTTTTGTGTAGTGCTGTGTTCAGGCGTGTTTATGTTTCAAGAAATTAGAGTGTTATT
TTTTTTCTTTCCTTTGTGCGTAGTCATACTTGTTTGAATGTTGTGTATACTAGCTGAGACTAATCGGGCAC
CGTTTGACTTTGTGGAAGGAGAGTCGGAGCTAGTGTCGGGTTATAACGTGGAGTACAGAGGAGGGGGT
TTTGCAGTTATATTTATTGCTGAGTACTCTAGAATTCTCCTTAGAAGAGTAATAAGGGCGGCGATATTCTT
CGGAGGAAATGAAGCGTTAGTTGGGTTTTTCATAATGGTTTTCGCAATTTTCTTTGTAGTTGTTCGTGCTT
CTTTACCACGCTTACGTTATGACAAGCTAATAAACTTATGTTGAACTGTCCTTTTATGTGTTATACTTACGG
CTAGTGTGTGTGTAGTAGTCTTAGTGGGGGTGTAATGGTAAGTTTTGTGGTGAGACCTATAAAATTAGTG
AGATTAGGGGTAATATTAATTGGAACAATCCTCAGGGTCAGAAGAGAAGAGCTAGTAGGGGTGTGGTTA
GGTTTGGAGTTAAATCTTTACGGTTTTCTTGTGATTATAAACCCAGATGGGCATTATAGCCCTGAGCCCTG
CGTAAAATACTTTGTTGTACAAAGAACGGGGTCAATTTTGATACTAGTGGGGTTTGTAAGCTTAATACAG
CATGTAGTGAGGGGGGTTAGTGATGAGCACAGCGGGTACGGTGCTAAAGTCTGGCGTTTTTCCTCTACAC
TCGTGGGTGCCCTCTATTATTAAAAACAGGAGATGGTTAGCAAGGGGGGTTAATACTAACCTGGCAAAAA
GTGGCCCCCCTTGTTTTTTTATCAATAATCTTACCTTCTAAAAGGCTTTGAGTAGTGATTGTATCGATAGCT
GGGATTGGGGCAGTGGGAGGTCTCAACCAAAACTCAGTGCGGGTAATAAGAGCCTACTCTTCATTTGTC
CATACATCTTGAATGTTGTTAGGGCTCACATGATCAAGGGTAGTTTTCGTAGGGTACTTTGCAGTCTATAG
GTTATCGGTAGGGCTGTTTTTCTACGGGTGCTCGTTAATAAACAAGATAAGAATAGGTAGCCAGCTAAGT
AGGGCTGCTAGCGGTATGGGGTTGTTAATACTAATAGGGATGCCTCCGTTTCTTGGTTTCTTAGCAAAAG
TATTGGTATTTTTGATGAGGGGAAGGCCTGTAATTGTGGCATGCATCATAGGATCAGTCATTAGATTAAAA
TTTTATATTGATTTCTTTTACAGAATGGTAATAAAAAAATAAGGTAGAAGTTAAAGCCATTTGGAGCCTGGT
GATTTGCATAAACATTATGGGGGGGGCGCTGATTTTAGTAAGATTTATTTAGATGGTTATGGGCTTAAGGG
TTGTATTCGTTTGTATCGTGTGTTGTCTGTTTACAGGGTTAATAATATTAAGAGAAAAACGGGGGCTGGA
CCGAGAGAAATGTAGGCCGTATGAATGTGGGTTTGAGCCTATCGGAAGAGCTCGAAGCCCCTTCTCCAT
TCGATTTTTTTTAGTGGCAGTTTTGTTTGTGGTGTTCGATGTGGAGGTAGTCCTATTGATACCTTTTGCCT
ACATATTTTTCTATGGCAAAAGAGTGTTGGGTATTTTATCTTCAAGGGGGTTCCTCCTTATTCTGTTTGTG
GGACTATATCATGAATACCGTGAAGGTTCTTTGGAGTGAGTGGGTTAAATGGTTAAAAGGTTAGCAATTA
GGCTTATTGCATTAATTATTATAAAAAACCTGAATGTGAGTATTATTGGGTTAAGTGTTTTAACAATTTTGA
GTATAGGAGCCGCAAGAGTTGCGACAGGAGGGGTAGAATTAAATGGGCTATACTCGACGGACTTCGTGA
TGGGGTTGATGATCACATTGACATTATTTGTCGCAATTCTTTCTTATTTAAGTAGAGCTAAAGTCTCCCGT
AAAGCAAGATTTAACTTAATGGTTATCAGAATTAGTCTTATTTTAGTGATAAGGTTTAGGGTGAGGAGGT
TTTTTCTTTTTTTTTTTTTTTTCGAAAGTGTGTTAGCGCCTTTGTTACTGTTAATCGTAGGGTGGCGTCTAC
AGGCAGGAGGCTATATAGTGATTTATACTGTATTTGGGTCCCTTTTTTTTTCTATGGGGAGTAAGAGAGCTT
TACCTTAGAGGGAGAAGAAGGATGAGAGTGGGAAGATTAGTAAAAAAAAAGGGTAATAAGACTGTGATG
GCTATACATTTTAGGGTTTCTAATTAAGTTACCAATATACCCGTTCCATTTGTGGTTACCTAAGGCCCATGT
AGAGGCCCCTGTGGCAGGTTCAATGCTGCTGGCTGGGGTAGTACTAAAATTAGGAGGATACGGCCTACT
TCGGTTTATATTAGTAATACAAGTCAGGCTTAGAAGAGTGTTCGTGTTGCTACTAAGGGTAAACTTGGCA
GGGGGCTTCTACGCCGGACTGGCTTGTGTACGGCAAGTAGACCTAAAATGCTTGGTGGCATACTCGTCC
GTAGCTCACATAAGTCTAGTTCTATTAGTGCTAAGCAACACGCTATTAGGGGTGATAGGGGCTATTATTAT
TATGGTTGGCCACGGGTTATGTTCGTCAGGTTTATTTAGGTACGTAAATGCTATTTATAAGATGAGTCACT
CGCGCTTACTAGTAATAAACAAAGGTGGGTTACTTTTTTGCCCTGCACTAGTGTTGATGTGTTTTCTCCTA
AGATCAAGAAACATAGCAGCCCCACCTAGTCTAAATCTATTCGGGGAAATCCTCGTTTTCGGCGTCGGC
GGGTGAATAAGGGGGGCCTTTTTATTTATCCTGGGGTTGATGAGCTTTATTAGGGCTTGCTTTAGATTATA
CTTATATGGGAGGTGCTCACACGGAAAAGGGCTTATACATAGGGAGTCGCTGAATTTACTCTGTGATGTG
TTCGTCTTGTTGTCTCATTGGGTGCCGTTAAATTTCTTGTTTATATTTATACCTTAACGTATAAATAAACAC
CTATTGTGTCTGTTTGTAGGGCTTGAAATAATGAGGCTGGGCTTACTATTTGTAACACATGTTTTCTTAAT
AAATCAGTTTTGACTAATTTTATTGATTCTATGTTTAGCTGTTTGTGAAGCTAGCATTTGTTTGGCCCTGCT
GGTCATAGTGATGCGATTATGCGGCGACGACCTAATATCAAGATTGCTAAGAGATGTATACAATCAAAGG
GTAACCTAGGGTTGCTTATATTAATCTTAGGGTATTTATTCATTTTTACAGGAAGGAGGGGAAGAGCGTAC
TTACTTGAAGTTCCTGTTTGAGAGAGCAATTGTCTGTCTTTTAGTTTTAGATTCTTACTAGATAATGTAAG

GATAATTTTTGTTGGGACAGTTTTGGTGATCAGAGGGAGTGTAGCAACCTATTGTAAGTGGTATATAGCT
GAAGAGCTATACTACAACCGGTTTATGGGGTTGGTGTGGTTATTTGTCTTATCTATAGTGTTTATAATCTTA
GTGCCTAATTTAGTAATACTTTTAATTGGATGGGATGGGCTAGGCCTCACTTCTTTTCTATTAGTGGCTTAC
TACCAAAATAACAAAAGACTATCTGCGGCTATGTTGACAGCTTTAACTAATCGAATTGGAGACGTTCTTG
TTCTAATTAGCATTTCTATCTTTCTAAGAGAGGGGGGGTGGTTAATTTATAGGTATCACCCAGTACAAATA
TGGTTATTAGGTTTTATAGTGGTTTTTGCAGGGATAACTAAAAGAGCACAGATGCCTTTTTGCGCATGAC
TGCCAGCTGCCATAGCCGCACCGACACCTGTCTCTTCTTTAGTGCACTCTTCAACACTGGTAACAGCTG
GGGTTTATCTTGTACTTCGTTCGTTCTATATTATCAGTTCTAACGTAACACAAGTTCTAATAATTTTAAGGT
TATTTACTCTGGTACTAGCTGGATCAAGTGCAGTGTTCGCGTTTGATCTAAAAAAAGTAATTGCACTCTC
GACCTTAAGTCAACTAAGGTTGATAATGTTTTCGATTTCAATCCTCCTTCCGTCTGTGGCGTTTTTCCATC
TGGTGACTCATGCAGTGTTTAAAGCTTTGCTGTTTTTGGGTGCAGGGGGTGTTATCCATAGAAACCAAA
GAATTCAGGATATTCGTGGGTTAAGAAGACTGTGACAAGGGTTGCCGGTAAGAATAGGTGCAATAAGAG
TTGCAATTGTATCTTTAAGAGGGGCTCCGTTTATGAGAGGGTTTTCTCTAAAGACCTCATGATTGAGAT
GATAGGCAGAAGGACTTATGGGTACTTATTAGAGTTATTAGGGTTAATTTTTACTTCTTTTTATAGGGCAC
GAGTATTTAGAGTTATACTCGGGACCAATTATGTTAATAATAGAACCTTGCGTATTAATGAGCATTTAAATA
TACAAACCCCGTTTCTTAGCCTATATATTGGAGCTATTATTTTAGGGGTGGTGCTGGGTAGAAAAATAGAA
AGATTTGGGTTTGTAGTAGTTCTTGAAAACTATGAAAGACTTAGAGTCTTTTTTATTCCTTTTGGGTTACT
GTGACGAGGAGTTATTAGTAAGCTAGGGTCGAGTCCTGCTAAGTTAAGATTTTTTTTGAGGATGTGGTTT
ATAGAATTAACTCATCCTGGGAAGGTGACCTTTTTTAAAGGGTCAAGAACAGTGGCCCAAAGTTTGGAC
CAAGGTTGACTAGAGCTATTGGGGCCTCAAGCTGGATTAGGTCAATTCAGCTGCTTAAATGAGAACTATT
TTACTGTGGTGTGATTAGTAGCCGGGGGCGTTAGCTTAATTATTTTATATAAATGAGAGTTATAATTATAT
GTGTAAGTTTGATGTCTGTATTCGTGTTGATTGCTAAGCAACCAATCTCTTTAGGGCTAGTACTATTGAGG
GGGTCTATAATTGCATGTGTGGAAATTGCATTGGAGGTTAGAAGTTTGTTAGGGTTTTGTTATTTTTAAC
TTATGTTAGGGGCGTGATAGTCTTGTTCTTATATGTTCTTAGTATTTATCCTAATGAGCGCTTTAATTTAGA
GTTCGTTATAATTGTGAGAAGGTGTGCTATGATGGGCCTTGTGGGATTAATGAATTATGAGAGTGGGTCA
TTATTTTTAAGGTTTATGGCTGAAGGAAGGCTATATATCCTAATAGCAGGCGTTTTGCTGTTTGTGATGTTA
GTGGTGTCGTATTTGTGCATAAAAACCATAGTGCCTTTGCGAAGAGTA"/>
<sequence id="seq_Mytilus_galloprovincialisF-FJ8901" spec="Sequence"
taxon="Mytilus_galloprovincialisF-FJ890" totalcount="4"
value="ATGTTAATAGATGTATTTTCTAGATTTGATGCTCACAGCTACAACTTAATTTGGTTATCTATGTTGT
GGTTGCTTTCGTCTATAGTGCCAATAACTGTGCTATTTAGAGATGTAAGCACTCGAAGCTTGGTACTGTCT
TTTACTTATTCTATAATTCGGAATGGGAAAGGTTTAAAGTTATCTGGGTTTCCTTTGGTGATAAGCGGCTT
GTTTATAATAATTTTGATGTTGAATTTGTCTGGGAACTTCCCCTTTTTTTTCCCTGTAAGAGGTCAGTTTG
TATTTGGATTTTCTTTTGCTTTATCTATTTGGACCTGTTTAGTATTATCTAGCTTACTGTGCAGGTTTGAGC
AGGGTTTGATGAGGCTCGTCCCGACAGGCCCGTTAATCCTTGTGCCTTTTATAGTAGTAGTTGAGCTAAT
TAGCGGTATACTTCGCCCTTTAACTCTAGTCTTGCGGCTAACGTTGAATTTAGGGGCTGGTAAAGTAATC
TTAACTATATGTAGAAGGGAGTTGGTAGTCGGTTGGTTAATTACAGGAGTTGGGGGTATCAAGGGTTTGC
TAATAGGTGGTGTCTTTGCTGCTGAAGTTGCCATTGCCTGTATTCAGTGTTACATTTTCTGTGTCTTATTGT
GTCTCTATACTGAGGACCATAGGAGTTAGCGATGGTTGTGGTCAACAAATCATAAAGATATTGGCACTCT
TTACCTATATAGTGGGGTCTGAGGAGGTTTGTTCGGGGCAAGTCTAAGCCTGATAATTATACAGGGGCAT
CCTGGAGCAGTATTTTTAAAAGATTGGTTTTATAATGTGGTTGTTACAACACACGCCTTAATAATAATTTT
CTTTGCTGTAATACCTATTCTAATTGGAGCTTTTGGTAATTGGCTTATTCCTCTATTAGTAGGTGGAAAAG
ATATAATCTATCCGCGGATAAATAATTTGAGTTATTGGTTATCTCCTAATGCGCTGTACTTACTTATATTATC
TTTTAGAACGGATAAAGGAGTAGGCGCTGGATGGACTATTTACCCGCCACTGTCTGTATATCCTTATCATA
GCGGGCCGAGGATAGATGTTCTTATTGTGTCTTTGCATTTAGCTGGGTTAAGTTCTTTGGTGGGTGCTATT
AATTTGCTAGTACCAACAAAAACATACCAGTTTTAGAGATAAAAGGAGAACGAGCTGAGCTTTATGTC
CTAAGGATTAGAGTTACTGCCGTATTGCTAATTATTTCTATTCCGGTTTTAGGAGGGGGTATTACAATAATT
CTGTTTGATCGGAATTTTAACACAACATTTTTTGATCCAGCAGGAGGGGGTGACCCTGTCTTGTTTCAAC
ATTTGTTCTGATTTTTTGGGCACCCTGAGGTATACATTCTTATTCTACCTGCTTTTGGTGTGATATCAAAAG
TAATTATGCATTGTTCCGGAAAGGAAGCAGTTTTCGGGTTAATTGGGATGGTGTATGCAATAATTGGAATT
GGAGGACTAGGTTGTATGGTGTGGGCTCACCATATATTTACAGTAGGGCTTAATGTTGATACTCGAGGTT
ATTTCTCTACTGCAACTATAGTAATCGCAGTCCCTACAGGGGTAAAAGTATTCAGGTGGTTAGCAACTAT
AGCAGGAAGAAAGTTTAAGATAAAGCCTGCTGCTTACTGAAGTACGGGGTTCTTGTTTCTATTCACCGT
AGGAGGTTTAACAGGGGTGTTATTATCAAGGGCTTCTATGGACGTATCGCTTCACGATACTTATTATGTGG
TGGCTCATTTTCACTATGTGCTAAGTATAGGAGCGGTGTTCGGAGTATTTTGTGGCCTAAACCATTGATTG
CCTAACTTTGTTGGAGTATGCTTTAATAAGAAATGGAGAAAAGCCCACTTTATAGCAATGTTTTTTGGTG
TAAATACCACTTTCTTCCCTCAACACTTTCTAGGGCTGAGGGGTATACCTCGACGGTACATAGATTATGCT
GACATTTATGCTCATTGGCATTGGGTGTCTTCTTATGGGTCTGCAGTGTCTTTTGGTTCTCTGATATATTTT
AAGTTCTTACTCTGAGAAGCTTTAGTGAGTCAACGAGGGATGTCTTTTTATGGGAGTCGATATTTTGGTG
ATATTGTCCATGAATTGGGGAAGGACCTATTTCGGTATCATGGATTTGTGATGATAGTAGCAGTGGCTGTA
TTAGTCTTTGTTATGTATATAGGGTGCGTGATCCTGTTTACTAAATTTTCTTACCGTCATTTTTTAAATCGCC
AACGATTGGAGTTTTGATGAACTATTGTGCCGATGTTGATGTTAGTAGGGCTGTGGTTCCCTTCGATGATT
AATTTGTATTATATAGAAGAAGTAAAGCGCCCACGTTGAAACTTCAAAGCAATCGGAAAGCAATGATAC

TGATCTTATGAGTGCGATACCTGTTATACAATTGACTCCTACATAGAAGACCAACAGGAGACAGGGTACC
GCTTGCTGGATGTTGACAACCGGATGGTTGCTCCTGCAGATGTACAAATAACTGCTTTTGTAAGGAGTTC
TGACGTGCTACATTCGTTTGCGCTTCCTAAATTATTAATCAAAGTAGATGCTATTCCGGGCCGAATCAATC
GGCTTCCGATAAAAGCATCTCAGTGTAGTATTATTTATGGGCAGTGCTCTGAAATTTGTGGAGTAAACCA
CAGGTTTATACCAATTGTGATTGAATTTATTCCTGAGAAATATTTTGTTATATGGTTGGAAGCTCTTAATTA
G------------------------
ATGAATCGTAATCCTTACTATGTACCAGGCCCAAGTCCGTGGCCATTTTTTGTGGCTATTTCGGCTAATGG
AATAGCGGTAGGGTTAATTTTGTGACTGCATCGAACCCCCTTTGTATTAATAGGTAGTCTGGTTTGCATAT
TATTGAGAACTTTTAGATGGTGACGCGATTTAATTCGTGAAGGAGACATTGGGCTTCACACTCGTTTTGT
GATCAAAAGATTTCGAGATGGCGTTGCCTTGTTTATTCTGTCTGAAGTGATATTTTTCTTCACTTTTTTCT
GGACTTTTTTCCATAATGCTCTAAGGCCTTCGTGTGAGTTAGGAATACGGTGGCCTCCTCCTGGAATTCG
TACACCAAACCCGTCATCTACTAGTCTGTTTGAGACAGGTCTTCTAATTAGAAGAGGGTTGTTTGTAACT
CAAGCCCATAAGAGGATGCGCTTGGATTACGATGTAGGGCCATTCATCGGTTTAGTGGTGACAATCGTAT
GCGGGACCGTGTTTTTTTTGGTACAACTGCGGGAATATTATTGAAACTCCTACACTATTGCAGATAGGGT
TTATGGTAGGGTTTTCTATTTACTAACTGGATTCCATGGGATACATGTTGTCGTAGGGACTATTTGGCTAA
CGGTAAGGTTAGTTCGACTATGACGCGGGGAGTTTTCTAGTCAACGACACTTTGGGTTTGAGGCTTGTAT
TTGGTACTGACATTTTGTAGATGTGGTATGAGTGGCATTGTGGTGCTTAGTGTACGTGTGGTTTGGAGGA
CCGTGACGAAGTACAAATAAACTGGTAAAGATTATGAATGACAGATTCTATGATTTGCCTTGTCCTGTAA
ACTTAAACGCCTGGTGAAGGTTTGGCTCTATACTAGGCTTGTGTCTGGTTATCCAGCTTTTGAGGGGTCT
TTTATTGTCAGCTCACTATACTGCTCATGAAGACATGGCATTTGACTCTGTAGTCCACATTATGCGTAATG
TGGAAAAGGGGTGAATATTACGCAATATCCATGCAAATGGGTCTTCTATGTTCTTTATTTGCATTTATGCT
CACATTGGTCGTGGGCTGTACTATGGGTCTTATTTGGATAAGACCGTGTGGTACTTTGGGGTACACCTGT
TCTTGTTGACTATGGCTGAGGCTTTCCTCGGCTATACTCTGCCTTGGGGACAGATATCGTACTGGGGGGC
TACTGTCATCACTAATATACTTAGAGTAATCCCTGTGGTAGGTGAAAGGATACTTCGCTACGTATGAGGG
GGGTGGACTGTGTGTAATGCAACGTTAAAGCGGTTTTACACACTTCACTTCCTTTTACCGTTTGTAATAG
TGGCGGTTGTTTTTTTGCACTTATTTTTTTTACACGAGAAGGGTAGTAATAACCCTTTAGGAATTGAAAG
GGGTACTATATGTGTGCCTTTTCATCCTTTTTATACTATTAAGGACCTCTTTGGTTATGTTTGCTTTAGGTTC
TTTTTTATGTATTTAGTGTGCGTGGACCCTGAGCTTCTAGGGAACCATCTAAACTACTGGCCTGCTAACCC
TATAAAAACGCCTATTCACGTTCAGCCTGAATGGTATTTTATATTTGCCTATGCAATTCTCCGTTCAATTCC
GCATAAAGCAGGGGGAGTATATGTTATGTTCCTTTCAATCGTAGTGCTATACTTAATTCCTACGCTTCATAC
AGGTAAGTACCGAAGTTTATGCTTCTACCCATTGAATCAAGTAGTGTTTTGGGTGCTGGTTGGAAGGTTT
ATTAGCCTAACATGAATTGGTGCTCGTCCAGTGCGTGAACCTTATATCATTTTGGGGCAGTGTCTTTCAGT
TATTTATTTCTCTAGTTTATTGTTGAACCCCCCTTTCTTTATGGGTGTGAGACAAGTTACTTGAAGCGGCAG
TAATTGTAAATATTATCCCTTTTGTAGGTGTCCTTCTTGCTGTGGGGTTTTACACCTTATTGGAGCGTAAG
ATTCTGGCCATCATCATGATCCGAAAAGGCCCGTCCAAGGTAAGTTACATGGGGATCTTGCAGCCTTTTA
GCGATGCAGGTAAGCTATTGTGTAAAGAGTTTATCGTGCCCACGCGTGCTAATGTAGGGCCATTTATTCT
GGCTCCTGCACTAATACTAGCTATTAGCTTATTAGGGTGGCTTTTATACCCCTATAAGTCAGCTGAAGTGT
TTTATGTTTTTGGGGTAATTTTGTTTATAGTTATTACTAGAGTTAGCGTTTATGGGGTAATAATGTCTGGAT
GGGCATCTAACTCCAAATACTCCTTACTAGGTGCAGTCCGTGCGATGGCGCAAAGAATTTCTTATGAAAT
CCCTATAGGGTTTATTTTTTTTTGTGTAGTGCTGTGTTCAGGCGTGTTTATGTTTCAAGAAATTAGAGTGT
TATTTTTTTTCTTTCCTTTGTGCGTAGTTATACTTGTTTGAATGTTGTGTATACTAGCTGAGACTAATCGGG
CACCGTTTGACTTTGTGGAAGGAGAGTCGGAGTTAGTGTCGGGTTACAACGTGGAGTACAGAGGAGGG
GGGTTTGCAGTTATATTTATTGCTGAGTACTCTAGAATTCTCCTTAGAAGAGTAATAAGGGCGGCAATATT
CTTCGGAGGAAATGAAGCGTTAGTTGGGTTTTTCATAATGGTTTTCGCAATTTTCTTTGTAGTTGTTCGTG
CTTCTTTACCACGCTTACGTTATGACAAGCTAATAAACTTATGCTGAACTGTCCTTTTATGTGTTATACTTA
CGGCTAGTGTGTGTGTAGTAGTCCTAGTGGGGGTGTAATGGTAAGTTTTGTGGTGAGACCTATAAAATTA
GTGAGATTAGGGGTAATATTAATTGGAACAATCCTCAGGGTCAGAAGAGAAGAGCTAGTAGGGGTGTGG
TTAGGTTTGGAGCTAAATCTTTATGGTTTTCTTGTGATTATAAACCCAGATGGGCATTATAGCCCTGAGCC
CTGTGTAAAATACTTTGTTGTACAAAGAACGGGGTCAATCTTGATATTAGTGGGGTTTGTAACCTTAATG
CAGCATGTAGTGAGGGGGCTAGTGATGAGCACAGCGGGTACGGTGCTAAAGTCTGGTGTTTTCCCTCTA
CACTCGTGGGTGCCCTCTATTATTAAAAACAGGAGATGGTTAGCAAGGGGGGTTAATACTGACCTGGCAA
AAAGTGGCTCCCCTTGTTTTTTTATCAATAATCTTACCTTCTAAAGGGCTTTGAGTAGTCATTGTATCAAT
AGCCGGGATTGGGGCAGTGGGGGGTCTCAACCAGAATTCAGTGCGGGTAATAAGAGCTTACTCCTCATT
TGTGCATACATCTTGAATGTTGTTAGGGCTCACATGATCAAGGGTAGTTTTCGTAGGGTATTTTGTAGTCT
ATAGGTTATCGGTAGGGCTGTTTTTCTACGGGTGCTCGTCAATAAACAAGATAAGAATAGGTAGCCAGCT
AAGTAGGGCTGCTAGCGGTATGGGGTTGTTAATACTAATAGGGATGCCCCCGTTTCTTGGGTTCTTAGCA
AAAGTATTGGTATTTTTGATGAGGGGAAGGCCTGTGATTGTGGCATGCATCATAGGATCAGTCATTAGAT
TAAAATTTTATATTGATTTCTTTTACAGAATGGTAATAAAAAATAAGGTAGAAGTTAAAACCATTTGGAGT
CTGGTGATTTGCATAAACATTATGGGGGGGGCGCTGATTTTAGTAAGATTTATTTAGATGGTTATGGGCTT
AAGGGTTGTATTCGTTTGTATCGTGTGCTGTCTGTTTACAGGGTTAATAATATTAAGAGAAAAACGGGGA
TTGGACCGAGAGAAATGTAGGCCGTATGAATGTGGGTTTGAGCCTATCGGAAGAGCTCGAAGGCCTTTC
TCCATTCGATTTTTTTAGTGGCAGTTTTGTTTGTGGTGTTTGATGTGGAGGTAGTCTTATTAATACCTTTT
GCCTACATATTTTTCTATGGCAAAAGAGTGTTGGGTATTTTATCCTCAAGGGGGTTCCTCCTTATTTTGTTT

GTGGGATTATATCATGAATACCGTGAAGGTTCTTTGGAGTGAGTCGGTTAAATGGTTAAAAGGTTAGCAA
TTAGGCTTATTGCATTAATTATTATAAAAAACCTGAATGTGAGTATTATTGGGTTAAGTGTTTTAACAATTT
TGAGTATAGGGGCCGCAAGAGTTGCGACAGGAGGGGTAGAATTAAATGGGTTATACTCCACGGATTTCG
TGATGGGGTTGATGATCACATTGACATTATTTGTCGCAATTCTCTCTTATTTAAGTAGAGCTAAAGTCTCC
CGGAAAGCAAGATTTAACTTAATGGTTATCAGAATTAGTCTTATCTTAGTGATAAGGTTCAGGGTGAGGA
GGTTTTTTCTTTTTTTTTTTTTTTTTGAAAGTGTGTTAGCGCCTCTGTTACTGTTAATCGTAGGTTGGCGTC
TACAGGCAGGAGGTTATATAGTGATTTATACTGTATTCGGGTCCCTTTTCTTTCTATGGGGAGTAAGAGAG
CTTTATCTTAGAGGGAGAAGAAGGATGAGAGTGGGAAGATTAGTAAAAAAAAGGGCAATAAGACTGTG
ATGGCTATACATTTTAGGGTTTCTTATTAAGTTACCAATATACCCGTTCCATCTGTGGTTACCTAAGGCCCA
TGTAGAGGCCCCCGTGGCAGGTTCAATGCTGTTGGCTGGGGTAGTACTAAAATTAGGAGGATATGGCCT
ACTTCGGTTTATAGCAGTAATACAAGTTAGGCTCAGAAGAGTGTTCGTGTTGCTACTAAGGGTAAACCTG
GCAGGGGGCTTCTACGCCGGACTGGCCTGTGTACGGCAAGTAGACCTAAAATGCTTGGTGGCATATTCG
TCTGTAGCTCATATAAGTCTAGTTCTATTAGTCCTAAGCAACACGCTATTAGGGGTAATAGGGGCTATCAT
TATTATGGTCGGCCACGGGTTATGTTCGTCAGGCTTATTTAGGTACGTAAATGCTATCTATAAGATGAGGC
ACTCGCGCTTACTAGTAATAAACAAAGGTGGGTTACTTTTTTGCCCTGCATTAGTGTTGATGTGTTTCCTT
CTAAGATCAAGAAATATAGCAGCTCCACCTAGTCTAAATCTGTTTGGGGAAATCCTCGTTTTCGGCGTCG
GTGGGTGAATAAGGGGGGCCTTCTTATTTATCCTGGGGTTGATGAGCTTTATTAGGGCTTGCTTTAGATTA
TACTTATACGGGAGGTGCTCACATGGAAAAGGGCTTATACATAGGGAGTCGCTGAATTTACTCTGCGATG
CGTTTGTCTTGTTGTCTCATTGGGTGCCGCTAAATTTCTTGTTTATATTTATACCTTAACGTATAAATAAAC
ACCTATTGTGTCTGTTTGTAGGGCTTGAGATAATGAGGCTGGGTTTACTATTTGTAACACATGTTTTCTTA
ATAAATCAGTTTTGACTAATTTTATTGATTCTATGTTTAGCTGTTTGTGAAGCTAGTATTTGTTTGGCCTTG
CTGGTCATAGTGATGCGATTATGCGGCGACGACCTAATATCAAGACTGCTAAGAGATGTATACAATCAAA
GGGTAACCTAGGGTTGCTTATATTAATCTTAGGGTATTTATTCATTTTCACAGGAAGGAGGGGAAGAGCG
TATTTACTTGAAGTTCCTGTTTGAGAGAGCAATTGTCTGTCTTTTAGTTTTAGATTCTTACTAGATAATGTA
AGGATAATTTTTGTTGGGACAGTTTTGGTGATCAGAGGGAGTGTAGCAACCTATTGTAAGTGGTATATAG
CTGAAGAAATATACTACAACCGGTTTATGGGGTTGGTGTGGTTATTTGTTTTATCTATAGTGTTCATAATCT
TAGTGCCTAATCTAGTAATACTTTTAATTGGATGGGATGGGCTAGGCCTCACTTCTTTTCTGTTAGTGGCT
TACTACCAAAATAACAAAAGACTATCTGCGGCTATGCTGACAGCTTTAACTAATCGAATTGGAGACGTCC
TTGTTCTAATTAGTATTTCTATCTTTCTAAGAGAGGGGGGGTGGTTAATTTATAGGTATCACCCAGTACAA
ATATGGTTATTAGGTTTTATAGTAGTTTTTGCAGGGATAACTAAAAGAGCACAGATGCCTTTTTGCGCATG
ACTGCCAGCTGCCATAGCCGCACCGACACCTGTCTCTTCTTTAGTGCACTCTTCAACATTGGTAACAGCT
GGGGTTTATTTGGTACTTCGTTCATTTTATATTATCAGTTCTAACGTAACACAAGTTCTAATAATTTTAAGG
TTATTTACTCTGGTACTAGCTGGATCAAGTGCAGTGTTCGCGTTTGATCTAAAAAAAGTAATTGCACTCT
CGACTTTAAGTCAACTAAGGTTGATAATGTTTTCGATTTCAATCCTCCTTCCGTCTGTGGCGTTTTTTCAT
CTGGTGACTCATGCAGTGTTTAAAGCTTTGCTGTTTTTGGGTGCAGGGGGGTGTTATCCATAGAAACCAA
AGAATTCAGGATATTCGTGGGTTAAGAAGGCTGTGACAAGGGTTGCCGGTAAGAATAGGTGCAATAAGA
GTTGCGATTGTATCTTTAAGAGGGGCTCCGTTTATGAGAGGGTTTTCTCTAAAGACCTCATGATTGAGA
TGATAGACAGAAGAACTTATGGGTACTTATTAGAGTTACTAGGGTTAATTTTTACTTCTTTTTACAGGGCA
CGAGTATTTAGAGTTATACTCGGGTCCAATTATGTTAATAATAGAACCTTGCGTATTAATGAGCATTTAAAT
ATACAAACCCCGTTTCTTAGCCTGTATATTGGAGCTATTATTTAGGGGTGGTGCTGGGTAGAAAAATGG
AGAGATTCGGGTTTGTAGTAGTTCTTGAAAGCTACGAAAGACTTAGAGTCTTTTTTATTCCTTTTGGGTT
ACTGTGACGAGGAGTTATTAGTAAGCTAGGGTCGAGTCCTGCTAAGTTAAGATTTTTTTTGAGGATGTGG
TTTATAGAATTAACTCATCCTGGGAAGGTGGCCTTTTTTAAAGGGTCAAGAACAGTGGCCCAAAGTTTG
GACCAAGGTTGACTAGAGCTATTGGGGCCTCAAGCTGGATTAGGTCAATTCAGTTGTTTAAATGAGAAC
TATTTTACTGTGGTGTGATTAGTAGCCGGGGGCGTTAGCTTAATTATTTTTATATAAATGAGAGTTATAATT
ATATGTGTAAGTTTGATGTCTGTATTCGTGTTGATTGCTAAGCAGCCAATCTCTTTAGGGCTAGTATTATTG
AGGGGGTCTATAATTGCATGTGTGGAAATTGCATTGGAGGTTAGAAGTTTGTTAGGGTTTTTGTTATTTTT
AACTTATGTTAGGGGTGTGATAGTCCTGTTTTTATATGTTCTTAGGATTTATCCTAATGAGCGCTTTAATTT
AGAGTTTGTTATAATTGTGAGAAGGTGCGCTATGATGGGCTTTGTGGGATTAATAAATTATGAGAGTGGG
TCATTATTTTTAAGGTTTATGGCTGAAGGAAGGCTATATATCTTAATAGCAGGCGTTTTGCTGTTTGTGATG
TTAGTGGTGTCGTACTTGTGTATAAAAACCATAGTGCCTTTGCGAAGAGTA"/>
<sequence id="seq_Mytilus_trossulusF-GU9366251" spec="Sequence" taxon="Mytilus_trossulusF-GU936625" totalcount="4"
value="ATGTTAATAGATGTTTTTCTAGATTTGATGCTCACAGCTATAACTTAATTTGGTTGTCTATGTTATG
GCTGCTGTCTTCTATAGTGCCAATAACCGTGCTATTTAGAGACGTGAGTACGCGGAGTTTGGTGCTATCC
TTTACTTATTCGATGATTCGAAACGGAAAGGGACTAAAGCTATCTGGGTTTCCTCTAGTAATAAGGGGTC
TGTTCATGATAATTCTGATACTAAATCTGTCTGGAAACTTTCCATTCTTTTTCCCTGTAAGAGGGCAGTTT
GTGTTCGGGTTCTCCTTTGCTTTGTCTATTTGAACTTGTTTAGTTTTATCTAGTCTTTTATGCAGATTTGAG
CAGGGGTTGATGAGTCTCGTTCCAACAGGTCCGTTAATCCTTGTGCCTTTTATAGTAGTGGTTGAGCTAA
TTAGTGGCATACTTCACCCTTTAACATTAGTTTTACGTCTGACACTAAATCTGGGAGCTGGTAAAGTAATT
TTAACTATATGCAGGAGAGAGTTAGTAGTTAGCTGGTTACTTACAGGAGTTGGGGGTATTAAAGGGTTAA
TAATGGGCGGTGTTTTTGCCGCTGAAGTTGCAATCGCGTGTATTCAGTGTTATATTTTTTGTGTCTTATTGT
GTCTCTATACGGAGGATCATAGAAGGTAGCGATGGCTGTGATCAACAAATCACAAAGATATTGGAACCCT

TTATCTGTATAGCGGAGTCTGAGGAGGGTTGTTTGGAGCAAGGTTGAGGTTAATGATCATGCAAGGTCAT
CCTGGAGCAGTGTTCTTAAAAGATTGATTCTATAATGTGGTGGTTACAACGCATGCCTTAATAATAATTTT
TTTTGCTGTGATACCTATCTTAATTGGAGCTTTCGGTAATTGGTTGATTCCTCTGCTAGTAGGAGGTAAAG
ATATAATTTACCCGCGAATAAATAACTTAAGTTATTGACTATCTCCTAATGCACTATACTTACTAATACTGTC
CTTTAGAACGGATAAAGGAGTTGGTGCTGGATGAACTATTTACCCCCCTTTATCTGTGTACCCCTATCATA
GGGGCCCTAGGATAGATGTTCTTATTGTGTCACTACATCTAGCTGGGCTTAGCTCTCTAGTGGGGGCTATT
AACTTTGCTAGGACCAATAAAAATATGCCAGTGTTAGAAATGAAAGGAGAACGAGCGGAGCTTTATGTT
TTAAGGATTAGAGTTACTGCAGTTCTTTTAATTATTTCAATTCCGGTTTTAGGAGGGGGTATCACAATAAT
CTTGTTTGACCGAAACTTTAACACAACTTTTTTCGATCCCGCAGGAGGGGGGGACCCCGTTTTGTTCCA
ACATTTGTTTTGATTTTTTGGGCATCCGGAAGTGTATATTCTTATTCTACCTGCCTTTGGTGTGATATCAAA
AGTAATTATGCATTGCTCTGGAAAAGAAGCGGTTTTTGGTCTAATTGGGATAGTATACGCAATAATCGGA
ATTGGAGGGTTAGGGTGTATGGTGTGGGCTCACCACATGTTTACCGTAGGTCTTAATGTTGATACTCGAG
GCTATTTTTCTACTGCAACTATAGTAATCGCTGTTCCTACAGGGGTGAAAGTATTCAGATGATTGGCAACG
ATAGCAGGAAGAAAATTCAAAATAAAGCCTGCCGCCTACTGAAGTACTGGGTTTCTGTTTTTATTCACCG
TGGGAGGGCTAACAGGGGTCTTACTGTCTAGGGCTTCTATGGATGTGTCTCTACACGACACATATTATGT
GGTGGCTCATTTCCATTATGTGCTAAGAATAGGGGCGGTGTTTGGGGTGTTCTGTGGTCTTAACCATTGG
TTGCCAAATTTTGTTGGAGTATGTTTTAATAAGAAATGGAGGAAAGCCCATTTTATAGCAATATTTTTTGG
GGTAAATACTACCTTCTTCCCTCAGCATTTCTTAGGCCTAAGAGGAATGCCTCGACGGTATATAGACTAC
GCTGATATTTATGCTCACTGACATTGGGTGTCTTCTTATGGGTCCGCTGTGTCTTTTGGGTCTCTAATATAT
TTTAAGTTCCTTCTATGAGAGGCTCTAGTAAGCCAGCGAGGGATGTCTTTTTACGGGAGTCGATATTTTG
GTGATATTGTCCATGAACTAGGGAAAGACCTGTTCCGGTACCATGGTTTTGTGATGATAGTAGCAGTGGC
TGTGTTGGTCTTTGTTATGTATATAGGGTGTGTAATCCTTCTTACTAAATTTTCTTATCGCCATTTCTTGAA
CCGTCAACGATTAGAATTTTGATGGACTATTGTGCCAATGTTGATGTTAGTAGGGTTGTGGTTTCCTTCTA
TAATTAACCTATATTATATAGAAGAAGTAAAACGGCCCCGGTGAAACTTTAAGGCGATTGGGAAACAATG
GTACTGATCTTACGAATGTGACACTTGTTATACAATTGATTCTTACATAGAAGACCAGCAGGAGACAGGG
TATCGTTTGTTGGATGTTGATAACCGGATGGTGGCTCCAGCAGATGTGCAAATAACTGCTTTTGTAAGAA
GGTCTGATGTGCTCCATTCGTTTGCACTGCCTAAGTTACTAATTAAAGTAGACGCCATCCCAGGTCGAAT
TAATCGGCTTCCTATAAAAGCTTCCCAGTGTAGAATTATTTACGGGCAGTGTTCTGAAATTTGCGGGGTT
AACCATAGATTTATACCGATTGTGATTGAGTTTATTCCTGAGAAATATTTTGTCATATGGTTGGAAGCTCTT
AACTAA------------------------
ATGAATCGTAATCCTTACTATGTACCAGGTCCAAGTCCGTGGCCCTTTTTTGTGGCTATCTCGGCAAACG
GAATAGCGGTAGGGTTAATTTTGTGACTGCATCGAACTCCCTTTCTATTAATAGGAAGGTTGGGGTGTAT
ACTATTGAGAACTTTTAGATGATGGCGAGACTTAATTCGTGAGGGAGATATTGGGTTTCATACTCGCTTC
GTAATCAAGAGATTTCGTGATGGAGTTGCCCTTTTTATTCTGTCTGAAGTAATGTTCTTCTTTTCTTTTTTT
TGGACTTTCTTCCATAATGCCCTAAGACCCTCGTGTGAACTAGGGATGCGATGACCCCCTCCAGGGATCC
GCACGCCAAACCCGTCGTCGACAAGGCTGTTCGAGACAGGTCTTTTAATTAGGAGGGGGTTATTCGTAA
CTCAAGCCCATAAGAGAATGCGTTTGGATTATGATGTTGGGCCATTTATTGGCCTAGTGGTAACAATTTTA
TGTGGGACTGTGTTCTTCCTAGTGCAACTTCGAGAATACTACTGAAACTCGTACACTATTGCAGATAGGG
TGTATGGAAGAGTGTTTTATTTACTAACTGGGTTTCATGGAATGCACGTAGTCGTGGGGACTTTTTGACT
AATGGTGAGGTTAGTTCGACTATGGCGTGGGGAGTTTTCCAGTCAACGGCACTTTGGTTTTGAGGCTTG
CATTTGGTACTGACACTTCGTAGATGTGGTATGGGTAGCATTATGATGTCTAGTATATGTGTGGTTTGGAG
GACCGTGACGAAGTACTAATAAGCTGGTGAAGATTATGAATGACAGGTTCTATGATTTGCCTTGTCCTGT
AAACTTAAACGCTTGGTGAAGGTTTGGCTCTATACTAGGCTTGTGCCTGATTATCCAACTTCTAAGGGGT
CTTTTATTGTCAACTCATTATACTGCTCATGAAGACATGGCATTCGATTCTGTAGTACATATTATGCGTAAT
GTGGAAAAAGGATGAATGTTGCGTAATATTCATGCAAATGGGTCCTCTATGTTTTTTATCTGTATTTATGC
GCACATTGCTCGTGGGCTGTATTATGGGTCTTATTTAGATAAGACAGTGTGGTATTTTGGGGTGCATTTGT
TTTTGTTAACTATGGCGGAGGCTTTCCTCGGTTACACTTTGCCTTGGGGGCAAATATCATATTGGGGGGC
TACTGTTATTACTAATATACTTAGAGTGATCCCCGTAGTAGGAGAGAGTATGCTCCGCTATGTATGAGGGG
GTTGGACCGTGTGTAATGCAACTCTAAAGCGGTTTTATACTTTACACTTTCTCTTACCGTTTGTGATAGTG
GCGGTTGTTTTTTTACACCTGTTTTTTTTACATGAGAAAGGGAGTAATAACCCTTTGGGTATTGAAAGAG
GTACTATGTGTGTGCCCTTCCACCCCTTCTATACTATCAAAGATCTTTTTGGTTATGTTTGCTTTAGGTTCT
TTTTTATATATTTAGTGTGTGTGGATCCTGAGCTGTTAGGGAATCATTTAAACTATTGGCCTGCTAATCCTA
TAAAAACGCCAATCCATGTTCAGCCTGAGTGGTATTTTATGTTTGCTTATGCAATCCTTCGTTCAATTCCT
CATAAAGCGGGGGGGGTATATGTTATGTTTTTGTCGATTGTAGTATTATACCTAATTCCTAGTCTTCACAGA
GGTAAGTATCGAAGTTTATGTTTTTACCCGTTTAATCAAGTAGTGTTTTGAGTGTTGGTTGGTAGGTTTAT
TAGGTTAACATGGATTGGTGCTCGCCCAGTGCGGGAGCCTTATATCATTTTGGGGCAGTGTCTTTCAGTC
ATTTATTTCTCTAGGTTGTTATTAAACCCCCTTTCTTTGTGGGTGTGGGACAAGCTGCTTGAAGTAGGCGT
GGTTGTTGGTGTTATCCCTTTTGTAGGGGTGCTTCTCGCTGTGGGCTTCTATACTTTGTTGGAACGTAAA
ATTTTGGCTATCATTATAATCCGAAAGGGTCCATCCAAGGTGAGTTATATAGGGATCTTGCAGCCTTTTAG
TGATGCAGGTAAGTTGTTGTGTAAAGAGTTTATTGTGCCTACACGTGCTAACGTAGGGCCCTTCATTTTG
GCTCCTGCACTAATATTAACTATCAGTTTACTTGGATGGCTTTTATACCCGTATAAGTCGGCTGAAGTGTT
TTATGTTTTCGGGGTGATTCTGTTTATAGTTATTACTAGAGTCAGGGTTTACGGGGTAATGATATCCGGATG
GGCTTCTAACTCTAAATACTCTTTGCTAGGTGCAGTTCGTGCGATGGCGCAAAGAATTTCTTATGAGATC

CCTATAGGATTTATCTTCTTTTGTGTGGTGCTGTGCTCGGGTGTGTTTATGTTTCAAGAAATTAGGGTGTC
CTTTTTTTTCTTTCCTTTGTGCGTAGTTATAGTTGTCTGAATGCTGTGTATGCTAGCTGAAACTAATCGGG
CGCCATTTGATTTGTGGAAGGAGAGTCGGAATTAGTGTCAGGATACAACGTGGAGTACAGCGGAGGG
GGGTTTGCAGTTATATTTATTGCGGAGTACTCTAGTATTCTTCTCAGAAGGGTTATAAGGGCGGCGATATT
TTTCGGGGGAAATGAAGCGTTGATCGGGGTCTTTATGATGGCTTTTGCGGTCTTCTTTGTGGTTATTCGT
GCTTCTTTACCTCGTTTACGTTATGATAAGTTAATGAGTTTGTGTTGGACTGTTCTTCTATGTGTCATACTT
ATGGCTAGTGTGTGTGTAGTAGTTCTAGTTAGGGTGTAATGGTAAGCTTTGTGGTAAGACCTATAAAATT
AGTGAGATTAGGGGTAATATTGATCGGGACAATTCTTAGGGTTAGAAGAGAAGAGATAGTAGGGGTGTG
ACTCGGTCTAGAGCTAAATCTGTATGGATTTCTTGTAATTATAAACCCTGATGGGCACTATAGTCCTGAGC
CCTGTGTAAAATATTTTGTGGTACAAAGAACGGGGTCAATTCTGATACTAGTGGGTTTTGTAACCTTGAT
ACAGCACGTAGTGAGAGGGCTGGTGATAAGGAGGGCGGGTACAGTGCTAAAATCTGGCGTTTTCCCGC
TACATTCGTGGGTCCCTTCAATTATTAAGAACAGCAGATGGTTAGCAAGAGGGTTAATATTAACTTGGCA
AAAAGTAGCCCCCCTTGTCTTTTTATCAATAATTATACCCTCTAAGGGGTTGTGAGTAGTAATTGTATTGA
TAGCTGGAATTGGGGCAGTAGGGGGCCTTAACCAGAACTCAGTACGAGTAATAAGTGTGTACTCGTCGT
TTGTGCATACATCATGAATGCTGTTAGGGCTCACATGGTCAAGAGTAGTCTTTGTAGGGTATTTTGCAGTT
TATTCGCTGTCGGTAGGGCTGTTTTTTTATGGGTGCTCAATAATAAACAAAACAAGAATGGGCGGTCAGA
TTAGTAGAGCCGCGAGGGGTATAGGGTTACTGATACTGATGGGGATGCCTCCTTTCCTTGGCTTTCTAGC
GAAAGTATTGGTGTTTCTAATGAGAGGAAGGGCTGTAATTGTGGCTTGTATTATAGGTTCAGTAATCAGG
CTAAAATTCTACATTGACTTTTTTTATAGGATAGTAATAAAAAAACAAAGCAGAATTCAAGATTATGTGGA
GGATAGTGATCGGGGCTAATCTAGCAGGGGGGGCATTGATCTTGGTGAGATTTATTTAGATGGTTATGGG
GTTAAGAGTTGCGTTTGTCTGCATTGTGTCTTTTTTGTTTACGGGGTTAATGCTACTAAGGGAAAAGCGG
GGCCTAGACCGAGAAAAGTGCAGTCCATATGAGTGTGGATTTGAGCCTATTGGAAGAGCTCGGAGGCC
CTTTTCTATCCGATTCTTTCTAGTAGCAGTTTTGTTCGTCGTGTTTGATGTAGAGGTAGTGCTGTTAATACC
TTTTGCCTACATGTTCTTTTACGGTAAGAGAGTGTTAGGGATTTTGTCCTCAAGGGGTTTCCTTCTTATCT
TGTTTGGGGGTCTCTATCACGAATATCGTGAGGGGTCTTTGGAATGAGTAGGTTAAATGGTTAAAAGGTT
GGCAATTAGACTTATTGCATTGATTATTATAAAAAACCCAAATATGAGTGTCATTGGGTTAAGCGTTCTAA
CTATTCTAAGTATGGGCGCCACAAGAGTCGCGATAGGGGGGGTAGAGTTGAACGGGTTGTACACCACAG
ACTTTGTAATAGGGTTAATGGTTACACTAACTCTATTTGTAGCAATTCTTTCCTACCTAAGGAGGGTTAAG
ATCCACCGGAAAGCAAGATTTAATTTAATAATTATCAGAATCAGCCTAATTTTAGTGATAAGATTCAGGGT
GAGGAGGTTCTTTCTTTTTTTTTTTTTTTTTGAAAGTGTGCTAGCCCCTCTGTTGTTATTAATTGTAGGCT
GACGTTTACAGGCAGGGGGGTACATAGTAATCTATACGGTGTTCGGATCTCTTTTTTTCTTATGGGGGGTA
AGAGAACTCTATATTAGAGGGAGGAGGAGGATAAGTGTGGTAAGGCTAGTAAAAAAAAGGGGAATGAG
ATTGTGATGGCTATACATTCTAGGGTTTCTTATCAAGTTACCAATATATCCATTTCACCTGTGACTACCTAA
GGCTCACGTAGAGGCCCCAGTAGCCGGTTCAATGCTATTGGCCGGGGTGGTACTAAAATTAGGAGGGTA
CGGGCTGCTTCGATTTATAATAGTTATACAAATAAGGCTTAGAAGAGTGTTTGTGCTGCTACTAGTGGTG
AACTTGGCAGGAGGTGTCTATGCAGGATTAGCGTGTGTACGGCAAGTGGACCTAAAATGTTTGGTAGCA
TATTCCTCCGTAGCGCATATGAGGCTTGTGCTATTAGTGCTCAGGAACACGGTATTAGGGGTAGTGGGGG
CCATTATCATTATGATCGGGCATGGGTTGTGTTCATCAGGTTTGTTCAGGTATGTGAATGCTATCTATAAGA
TGAGGCACTCGCGCCTGCTAGTAATAAATAAAGGGGGCTTGTTAGTCTGCCCAAGTCTAGTCTTAATGTG
TTTCCTGTTAAGATCAAGCAACATAGCAGCCCCTCCTAGTTTAAACTTACTTGGGGAAATCCTCGTTTTT
GGCGTGGGAGGGTGAATAAGCGGAGTGTTCCTGCTTATCTTGGGTCTGATAAGCTTTATTAGGGCGTGTT
TTAGATTATACCTATATGGAAGTTGTTGTCACGGGAAGGGGGTGTCACACAGGGAGTCCTTAAACTTGGT
TTGTGATGTTTTTGTTCTGGCGGCTCATTGGATACCGCTGAACTTTATGTTTATGTTTATACCCTAACGTAT
AAACAAACACCTTTTGTGTTTATTTGTAGGGCTTGAAATAATAAGACTAGGCTTGTTGTTTGTAACCCAT
GTGTTTCTAATAAATCAGTTTTGGTTAATTTTACTAATTCTATGTTTAGCTGTTTGCGAAGCCAGAATTTGC
TTGGCCCTTCTGGTTATGGTGATGCGACTATGCGGAGACGATTTGATGTCAAGGTTACTAAGAGATGTAT
ACAATTAAAAAGTAACTTAGGGTTACTTTTACTGATTCTGGGATACTTATTTATTCTTAGAGGAAGGGCCG
GAAAAGCTTATTTATTGGAAGTTCCTGTTTGAGAGAGTAATTGTCTCTCATTTAGCTTCAGAGTTCTACTA
GATAGCGTGAGAATAGTTTTTGTTGGGACGGTTTTGGTAATTAGAGGAAGTGTAGCAACCTACTGCAAG
TGGTATATAGCTGGAGAGCCATACTACAAGCGGTTTATGGGATTAGTATGGTTGTTTGTGCTGTCTATAGT
GTTTATAATCTTAGTTCCTAATTTAGTAATACTTTTAATTGGTTGAGACGGGCTAGGGCTCACCTCATTCCT
ATTAGTGGCTTATTACCAGAACAATAAGAGGCTATCTGCGGCTATGTTGACAGCTTTGACTAATCGAATT
GGGGATGTTCTTGTACTCCTTAGAGTTTCTATTTTTTTAAGAGAAGGGGGGTGGTTAATTTATATATACCA
CCCAGTGCAGACATGGGTTTTAGGGTTTGTGGTAGTTCTTGCAGGTATAACTAAAAGGGCACAAATGCC
GTTTTGCGCATGGCTACCTGCTGCCATGGCGGCACCCACACCGGTCTCCTCTTTGGTGCATTCTTCGACA
TTGGTGACAGCTGGGGTTTATTTGATTCTTCGCTCTTTTTATATTATCAGAGCTAATGTGACTCAAATACTT
ATAGTCTTAAGACTATTTACTCTAATATTAGCGGGGTCAAGGGCTGTGTTTGCGTTTGACCTAAAAAAGG
TAATCGCACTCTCGACTTTGAGGCAGTTAAGGTTAATGATATTCTCGATTTCAATCCTTCTTCCGTCTGTA
GCTTTTTTTCATTTAGTAACCCATGCGGTATTTAAAGCTTTGTTGTTTCTAGGCGCAGGGGGTGTTATTCA
TAGAAACCAAAGAATCCAAGATATCCGGGGGTTAAGAAGCTTGTGGCAAGGATTACCGGTAAGAATGG
GTGCAATAACGGTTGCAATTGTGTCCTTGAGAGGGGCCCCGTTTATAAGAGGGTTTTTCTCTAAAGACCT
GATAATTGAGATGATAGACAGAAGAACTTATGGGTATTTATTAGAGCTAACAGGTTTAATCTTCACTTCTT
TTTATAGGGCACGGGTATTTAGAGTGATACTTGGGTCTAATTACGTTAATAGCAGAAGTTTGCGGATTAAT

GAGCACTTAAATATACAAACTCCTTTTCTTAGCCTGTATATTGGGGCTATTATCTTAGGAGGGGTATTAGG
GAGGAAAATAGAAAGGTTTGGGTTTGTAGTAGTTCTTGAGAAATATGAGAGAGTCAGAGTATTTCTTATT
CCCTTTGGGTTATTGTGATGAGGAGTGCTTAGAAAATTAGGGTCTAAGCCTGCCAAATTAAGATTCTTTT
TGAGAATGTGGCTCATAGAGCTAACCCACCCCGGAAAAATGGCCTTCTTTAAGGGGTCCAGGACGGTAG
CTCAAAGTCTGGATCAAGGTTGACTAGAGCTATTGGGTCCTCAAAAGGGACTAGGTCAATTCAGCTGTT
TGAATGAAAATTATTTACCGTAGTATGAGTGGTAGCGGGGGGCGTAAGCTTAATTATGTTTATATAAATG
AGAGTTATAGTCATATGTGTAATTTTGATGGCTGTGTTTGTCTTAATTGCCAAGCAACCTATTTCTTTAGG
GCTAGTGCTATTGAGGGGGTCTATAATTGCATGTGTGGAGGTTGCACTGGAGGTTAGGAGGTTGTTAGG
GTTCTTATTGTTTCTAACTTACGTTAGGGGTGTTATAGTCCTGTTCTTGTATGTTTTAAGGATCTACCCCAA
TGAACGTTTTAATCTAGAGTTTATGGTTCTCGTCAGAAGGTGTGCCAGAGCGGTCCTTATGGGTCTTATG
AATTACGAGAGCGGGTCTTTGTTTCTAAGTTTCATGGCTGAAAGAAGCTTATATATCCTAATAGCTGGTGT
GTTACTGTTTGTAATATTAGTGGTGTCGTATTTGTGCATAAAAACCATGGTGCCTTTACGAAGAGTA"/>
<sequence id="seq_Mytilus_trossulusF-HM4620801" spec="Sequence" taxon="Mytilus_trossulusF-HM462080" totalcount="4"
value="ATGTTAATAGATGTTTTTCTAGATTTGATGCTCACAGCTATAACTTAATTTGGTTGTCTATGTTATG
GTTGCTGTCTTCTATAGTGCCAATAACCGTGCTATTTAGAGATGTGAGTACGCGGAGTTTGGTGCTATCCT
TTACTTATTCGATGATTCGAAACGGAAAGGGACTAAAGCTATCTGGGTTTCCTCTAGTAATAAGGGGTCT
GTTCATGATAATTCTGATACTAAATCTGTCTGGAAACTTTCCATTCTTTTTCCCTGTAAGAGGGCAGTTTG
TGTTCGGGTTCTCCTTTGCTTTGTCTATTTGAACTTGTTTAGTTTTATCTAGTCTTTTATGCAGATTTGAGC
AGGGGTTGATGAGTCTCGTTCCAACAGGTCCGTTAATCCTTGTGCCTTTTATAGTAGTGGTTGAGCTAAT
TAGTGGCATACTTCGCCCTTTAACATTAGTTTTACGTCTGACACTAAATCTGGGAGCTGGTAAAGTAATTC
TAACTATATGCAGGAGAGAGTTAGTAGTTAGCTGGTTACTTACAGGAGTTGGGGGTATTAAAGGGTTGAT
AATGGGCGGTGTTTTTGCCGCTGAAGTTGCAATCGCGTGTATTCAGTGTTATATTTTTTGTGTCTTATTGT
GTCTCTATACGGAGGATCATAGAAGGTAGCGATGGCTGTGATCAACAAATCACAAAGATATCGGAACCC
TTTATCTGTATAGCGGAGTCTGAGGAGGGTTGTTTGGAGCAAGGTTGAGGTTAATGATCATGCAAGGTC
ATCCTGGAGCAGTGTTCTTAAAAGATTGATTCTATAATGTGGTGGTTACAACGCATGCCTTAATAATAATT
TTTTTTGCTGTGATACCTATCTTAATTGGAGCTTTCGGTAATTGGTTGATTCCTCTGCTAGTAGGAGGTAA
AGATATAATTTACCCGCGAATAAATAACTTAAGTTATTGACTATCTCCTAATGCACTATATTTACTAATACTG
TCCTTTAGAACGGATAAAGGAGTTGGTGCTGGATGAACTATTTACCCCCCTTTATCTGTGTACCCCTATCA
TAGGGGCCCTAGGATAGATGTTCTTATTGTGTCACTACATCTAGCTGGGCTCAGCTCTCTAGTGGGGGCT
ATTAACTTTGCTAGGACCAATAAAAATATGCCAGTGTTAGAAATGAAAGGAGAACGAGCGGAGCTTTAT
GTTTTAAGGATTAGAGTTACTGCAGTTCTTTTAATTATTTCAATTCCGGTTCTAGGAGGGGGTATCACAAT
AATCTTGTTTGACCGAAACTTTAACACAACTTTTTTTGATCCCGCAGGAGGGGGGGACCCCGTTTTGTT
CCAACATTTGTTTTGATTTTTTGGGCATCCGGAAGTGTATATTCTTATTCTACCTGCCTTTGGTGTGATATC
AAAAGTAATTATGCATTGCTCTGGAAAAGAAGCGGTTTTTGGTCTAATTGGGATAGTATACGCAATAATC
GGAATTGGAGGGTTAGGGTGTATGGTGTGGGCTCACCACATGTTTACCGTAGGTCTTAATGTTGATACTC
GAGGCTATTTTTCTACTGCAACTATAGTAATCGCTGTTCCTACAGGGGTGAAAGTATTCAGATGACTGGC
AACTATAGCAGGAAGAAAATTCAAAATAAAGCCTGCCGCCTACTGAAGTACTGGGTTTCTGTTTTTATTC
ACCGTGGGAGGGCTAACAGGGGTCTTACTGTCTAGGGCTTCTATGGATGTGTCTCTACACGACACATATT
ATGTGGTGGCTCATTTCCATTATGTGCTAAGAATAGGGGCGGTGTTTGGGGTGTTCTGTGGTCTTAACCA
TTGGTTGCCAAATTTTGTTGGAGTATGTTTTAATAAGAAATGGAGGAAAGCCCATTTTATAGCAATATTTT
TTGGGGTAAATACTACCTTCTTCCCTCAGCATTTCTTAGGCCTAAGAGGAATGCCTCGACGGTATATAGA
CTATGCTGATATTTATGCTCACTGACATTGGGTGTCTTCTTATGGGTCCGCTGTGTCTTTTGGGTCTCTAAT
ATATTTTAAGTTCCTTCTATGAGAGGCTCTAGTAAGCCAGCGAGGGATGTCTTTTTATGGGAGTCGATATT
TTGGTGATATTGTCCATGAACTAGGGAAAGACCTGTTCCGGTACCATGGTTTTGTGATGATAGTAGCAGT
GGCTGTGTTGGTCTTTGTTATGTATATAGGGTGTGTAATCCTTCTTACTAAATTTTCTTATCGCCATTTCTT
GAACCGTCAACGATTAGAATTTTGATGGACTATTGTGCCAATGTTGATGTTAGTAGGGTTGTGGTTTCCT
TCTATAATTAACCTATATTATATAGAAGAAGTAAAACGGCCCCGGTGAAATTTTAAGGCGATTGGGAAAC
AATGGTACTGATCTTACGAATGTGACACTTGTTATACAATTGATTCTTACATAGAAGACCAGCAGGAGAC
AGGGTATCGTTTGTTGGATGTTGATAACCGGATGGTGGCTCCAGCAGATGTGCAAATAACTGCTTTTGTA
AGAAGGTCTGATGTGCTCCATTCGTTTGCACTCCCTAAGTTACTAATTAAAGTAGACGCCATCCCAGGTC
GAATTAATCGGCTTCCTATAAAAGCTTCCCAGTGTAGAATTATTTACGGGCAGTGTTCTGAAATTTGTGG
GGTTAACCATAGATTTATACCGATTGTGATTGAGTTTATTCCTGAGAAATATTTGTCATATGGTTGGAAGC
TCTTAACTAA------------------------
ATGAATCGTAATCCTTACTATGTACCAGGTCCAAGTCCGTGGCCCTTCTTTGTGGCTATCTCGGCAAACG
GAATAGCGGTAGGGTTAATTTTGTGACTGCATCGAACTCCTTTTCTATTAATAGGAAGGTTGGGGTGTATA
CTATTGAGAACTTTTAGATGATGGCGAGACTTAATTCGTGAGGGAGATATTGGGTTTCATACTCGCTTCGT
AATCAAGAGATTTCGTGATGGAGTTGCCCTTTTTATTCTGTCTGAAGTAATGTTCTTCTTTTCTTTTTTTTG
GACTTTCTTCCATAATGCCCTAAGACCCTCGTGTGAACTAGGGATGCGATGACCCCCTCCAGGGATCCGC
ACGCCAAACCCGTCGTCGACAAGGCTGTTCGAGACAGGTCTTTTAATTAGGAGTGGGTTATTTGTAACT
CAAGCCCATAAGAGAATGCGTTTGGATTATGATGTTGGGCCATTTATTGGCTTAGTGGTAACAATTTTATG
TGGGACCGTGTTCTTCCTAGTGCAACTTCGAGAATACTACTGAAACTCGTACACTATTGCAGATAGGGTG
TATGGAAGAGTGTTTTATTTACTAACTGGGTTTCATGGAATGCACGTAGTCGTGGGGACTCTTTGACTAA

TGGTGAGGTTAGTCCGACTATGGCGTGGGGAGTTTTCCAGTCAACGGCACTTTGGTTTTGAGGCTTGCA
TTTGGTACTGACACTTCGTAGATGTGGTATGGGTAGCATTATGATGTCTAGTATATGTGTGGTTTGGAGGA
CCGTGACGAAGTACTAATAAGCTGGTGAAGATTATGAATGACAGGTTCTATGATTTGCCTTGTCCTGTAA
ACTTAAACGCTTGGTGAAGGTTTGGCTCTATACTAGGCTTGTGCCTGATTATCCAACTTCTAAGGGGTCT
TTTATTGTCAACTCATTATACTGCTCATGAAGACATGGCATTCGATTCTGTAGTACATATTATGCGTAATGT
GGAAAAAGGATGAATGTTGCGTAATATTCATGCAAATGGGTCCTCTATGTTTTTATCTGTATTTATGCGC
ACATTGCTCGTGGGCTGTATTATGGGTCTTATTTAGATAAGACAGTGTGGTATTTTGGGGTGCATTTGTTT
TTGTTAACTATGGCGGAGGCTTTCCTCGGTTACACTTTGCCTTGGGGGCAAATATCATATTGGGGGGCTA
CTGTTATTACTAATATACTTAGAGTGATCCCCGTAGTAGGAGAGAGTATGCTCCGCTATGTATGAGGGGGT
TGGACCGTGTGTAATGCAACTCTAAAGCGGTTTTATACCTTACACTTTCTCTTACCGTTTGTGATAGTGGC
GGTTGTTTTTTTACACCTGTTTTTTTACATGAGAAAGGGAGTAATAACCCTTTGGGTATTGAAAGAGGT
ACTATGTGTGTGCCCTTCCACCCCTTCTATACTATTAAAGATCTTTTTGGTTATGTTTGCTTTAGGTTCTTT
TTTATATATTTAGTGTGTGTGGATCCTGAGCTGTTAGGGAATCATTTAAACTATTGGCCTGCTAATCCTATA
AAAACGCCAATCCATGTTCAGCCTGAGTGGTATTTTATGTTTGCTTATGCAATCCTTCGTTCAATTCCTCA
TAAAGCGGGGGGGGTATATGTTATGTTTTTGTCGATTGTAGTATTATACCTAATTCCTAGTCTTCACAGAG
GTAAGTATCGAAGTTTATGTTTTTACCCGTTTAATCAAGTAGTGTTTTGAGTGTTGGTTGGTAGGTTTATT
AGGTTAACATGGATTGGTGCTCGCCCAGTGCGGGAGCCTTATATCATTTTGGGGCAGTGTCTTTCAGTCA
TTTATTTCTCTAGGTTGTTATTAAACCCTCTTTCTTTGTGGGTGTGGGACAAGCTGCTTGAAGTAGGCGT
GGTTGTTGGTGTTATCCCTTTTGTAGGGGTGCTTCTCGCTGTGGGCTTCTATACTTTGTTGGAACGTAAA
ATTTTGGCTATCATTATAATCCGAAAGGGTCCATCCAAGGTGAGTTATATAGGGATCTTGCAGCCTTTTAG
TGATGCAGGTAAGTTGTTATGTAAAGAGTTTATTGTGCCTACACGTGCTAACGTAGGGCCTTTCATTTTG
GCTCCTGCACTAATATTAACTATCAGTTTACTTGGATGGCTTTTATACCCGTATAAGTCGGCTGAAGTGTT
TTATGTTTTCGGGGTGATTCTGTTTATAGTTATTACTAGAGTCAGGGTTTACGGGGTAATAATATCCGGATG
GGCTTCTAACTCTAAATACTCTTTGCTAGGTGCAGTTCGTGCGATGGCGCAAAGAATTTCTTATGAGATC
CCTATAGGATTTATCTTCTTTTGTGTGGTGCTGTGCTCGGGTGTGTTTATGTTTCAAGAAATTAGGGTGTC
CTTTTTTTTCTTTCCTTTGTGCGTAGTTATAGTTGTCTGAATGCTGTGTATGCTAGCTGAAACTAATCGGG
CGCCATTTGATTTTGTGGAAGGAGAGTCGGAATTAGTGTCAGGATACAACGTGGAGTACAGCGGAGGG
GGGTTTGCAGTTATATTTATTGCGGAGTACTCTAGTATTCTTCTCAGAAGGGTTATAAGGGCGGCGATATT
TTTCGGGGGAAATGAAGTGTTGATCGGGGTCTTTATGATGGCTTTTGCGGTCTTCTTTGTGGTTATTCGT
GCTTCTTTACCTCGTTTACGTTATGATAAGTTAATGAGTTTGTGTTGGACTGTTCTTCTATGTGTCATACTT
ATGGCTAGTGTGTGTGTAGTAGTTCTAGTTAGGGTGTAATGGTAAGCTTTGTGGTAAGACCTATAAAATT
AGTGAGATTAGGGGTAATATTGATCGGGACAATTCTTAGGGTTAGAAGAGCAGAGATAGTAGGGGTGTG
ACTCGGTCTAGAGCTAAATCTGTATGGATTTCTTGTAATTATAAACCCTGATGGGCACTATAGTCCTGAGC
CCTGTGTAAAATATTTTGTGGTACAAAGAACGGGGTCAATTCTGATACTAGTGGGTTTTGTAACCTTGAT
ACAGCACGCAGTGAGAGGGCTGGTGATAAGGAGGGCGGGTACAGTGCTAAAATCTGGCGTTTTCCCGC
TACATTCGTGGGTCCCTTCAATTATTAAGAACAGCAGATGGTTAGCAAGAGGGTTAATATTAACTTGGCA
AAAAGTAGCCCCTCTTGTCTTTTTATCAATAATTATACCCTCTAAGGGGTTGTGAGTAGTAATTGTATTGA
TAGCTGGAATTGGGGCAGTAGGGGGCCTTAACCAGAATTCAGTACGAGTAATAAGTGCGTACTCGTCGT
TTGTGCATACATCATGAATGCTGTTAGGGCTCACATGGTCAAGAGTAGTCTTTGTAGGGTATTTTGCAGTT
TATTCGCTGTCGGTAGGGCTGTTTTTTTATGGGTGCTCAATAATAAACAAAACAAGAATGGGCGGTCAGA
TTAGTAGAGCCGCGAGGGGTATAGGGTTACTGATACTGATGGGGATGCCTCCTTTCCTTGGCTTTCTAGC
GAAAGTATTGGTGTTTCTAATGAGAGGAAGGGCTGTAATCGTGGCTTGTATTATAGGTTCAGTAATCAGG
CTAAAATTCTACATTGACTTTTTTTATAGGATAGTAATAAAAAACAAAGCAGAATTCAAGATTATGTGGA
GGATAGTGATCGGGGCTAACCTAGCAGGGGGGGCATTGATCTTGGTGAGATTTATTTAGATGGTTATGGG
GTTAAGAGTTGCGTTTGTCTGCATTGTGTCTTTTTTGTTTACGGGGTTAATGCTACTAAGGGAAAAGCGG
GGCCTAGACCGAGAAAAGTGCAGTCCATATGAGTGTGGATTTGAGCCTATTGGAAGAGCTCGGAGGCC
CTTTTCTATCCGATTCTTTCTAGTAGCAGTTTTGTTCGTCGTGTTTGATGTAGAGGTAGTGCTGTTAATACC
TTTTGCCTACATGTTCTTTTACGGTAAGAGAGTGCTAGGGATTTTGTCCTCAAGGGGTTTCCTTCTTATCT
TGTTTGGGGGTCTCTACCACGAATATCGTGAGGGGTCCTTGGAATGAGTAGGTTAAATGGTTAAAAGGT
TGGCAATTAGACTTATTGCATTGATTATTATAAAAAACCTAAATATGAGTGTCATTGGGTTAAGCGTTCTA
ACTATTCTAAGTATGGGCGCTACAAGAGTCGCGATAGGGGGGGTAGAGTTGAACGGGTTGTACACCACA
GACTTTGTAATAGGGTTAATGGTTACACTAACTCTATTTGTAGCAATTCTTTCCTATCTAAGGAGGGTTAA
GATCCACCGGAAAGCAAGATTTAATTTAATAATTATCAGAATCAGCCTAATTTTAGTGATAAGATTCAGGG
TGAGGAGGTTCTTTCTTTTTTTTTTTTTTTTTGAAAGTGTGCTAGCCCCTCTGTTGTTATTAATTGTAGGCT
GACGTTTACAGGCAGGGGGGTACATAGTAATCTATACGGTGTTCGGATCTCTTTTTTTCTTATGGGGGGTA
AGAGAACTCTATATTAGAGGGAGGAGGAGGATAAGTGTGGTAAGGCTAGTAAAAAAAAGGGGAATGAG
ATTGTGATGGCTATACATTCTAGGGTTTCTTATCAAGCTACCAATATATCCATTTCACCTGTGACTACCTAA
GGCTCACGTAGAGGCCCCAGTAGCCGGTTCAATGTTATTGGCCGGGGTGGTACTAAAATTAGGAGGGTA
CGGGCTGCTTCGATTTATAATAGTTATACAAATAAGGCTTAGAAGCGTGTTTGTGCTGCTACTAGTGGTG
AACTTGGCAGGAGGTGTCTACGCAGGATTAGCGTGTGTACGGCAAGTGGACCTAAAATGTTTGGTAGCA
TATTCCTCCGTAGCGCATATGAGGCTTGTGCTATTAGTGCTCAGCAACACGGTATTAGGGGTAGTGGGGG
CCATTATCATTATGATCGGGCATGGGTTGTGTTCATCAGGTTTGTTCAGGTATGTGAATGCTATCTATAAGA
TGAGGCACTCGCGTCTGCTAGTAATAAATAAAGGGGGCTTGTTAGTCTGCCCAAGTCTAGTCTTAATGTG

TTTCCTGTTAAGATCAAGCAACATAGCAGCCCCTCCTAGTTTAAACTTATTTGGGGAAATCCTCGTTTTTG
GCGTGGGAGGGTGAATAAGCGGAGTGTTCCTGCTTATCCTGGGTCTGATAAGCTTTATTAGGGCATGTTT
TAGATTATACCTATATGGAAGTTGTTGTCACGGGAAGGGGGTGTCACACAGGGAGTCCTTAAACTTGGTT
TGTGATGTTTTTGTTCTGGCGGCTCATTGGATACCGCTGAACTTTATGTTTATGTTTATACCCTAACGTATA
AACAAACACCTTTTGTGTTTATTTGTAGGGCTTGAAATAATAAGACTAGGCTTGTTGTTTGTAACCCATG
TGTTTCTAATAAATCAGTTTTGGTTAATTTTACTAATTTTATGTTTAGCTGTTTGCGAAGCCAGAATTTGCT
TGGCCCTTCTGGTTATGGTGATGCGACTATGCGGAGACGATTTGATGTCAAGGTTACTAAGAGATGTATA
CAATTAAAGAGTAACTTAGGGTTACTTTTACTGATTCTGGGATACTTATTTATTCTTAGAGGGAGGACCGG
AAAAGCTTATTTATTGGAAGTTCCTGTTTGAGAGAGTAATTGCCTCTCATTTAGCTTCAGAGTTCTACTAG
ATAGCGTAAGAATAGTTTTTGTTGGGACGGTTTTGGTAATTAGAGGAAGTGTAGCAACTTACTGCAAGTG
GTATATAGCTGGAGAGCCATACTACAAGCGGTTTATGGGATTAGTATGGTTGTTTGTGCTGTCTATAGTGT
TTATAATCTTAGTTCCTAATTTAGTAATACTTTTAATTGGTTGAGACGGGCTAGGGCTCACCTCATTCCTAT
TAGTGGCTTATTACCAGAACAATAAGAGGCTATCTGCGGCTATGTTGACAGCTTTGACTAATCGAATTGG
GGATGTTCTTGTACTCCTTAGAGTTTCTATTTTTTTAAGAGAAGGGGGGTGGTTAATTTATATATACCACC
CAGTGCAGACATGGGTTTTAGGGTTTGTGGTAGTTCTTGCAGGTATAACTAAAAGCGCACAAATGCCGT
TTTGCGCATGGCTACCTGCTGCCATGGCGGCACCCACACCGGTCTCCTCTTTGGTGCATTCTTCGACATT
GGTGACAGCTGGGGTTTATTTGATTCTTCGCTCTTTTTATATTATCAGAGCTAATGTGACTCAAATACTTAT
AGTCTTAAGACTATTTACTTTAATATTAGCGGGGTCAAGGGCTGTGTTTGCGTTTGACCTAAAAAAGGTA
ATCGCACTCTCGACTTTGAGGCAGTTAAGGTTAATAATATTCTCGATTTCAATCCTTCTTCCGTCTGTAGC
TTTTTTTCATTTAGTAACCCATGCGGTATTTAAAGCTTTGTTGTTTCTAGGCGCAGGGGGTGTTATTCATA
GAAACCAAAGAATCCAAGATATCCGGGGGTTAAGAAGCTTGTGGCAAGGATTACCGGTAAGAATGGGT
GCAATAACGGTTGCAATTGTGTCCTTGAGAGGGGCCCCGTTTATAAGGGGGTTTTTCTCTAAAGACCTAA
TAATTGAGATGATAGACAGAAGAACTTATGGGTATTTATTAGAGCTAACAGGTTTAATCTTCACTTCTTTT
TATAGGGCACGGGTATTTAGAGTGATACTTGGGTCTAATTACGTTAATAGCAGAAGTTTGCGGATTAATGA
GCACTTAAATATACAAACTCCTTTTCTTAGCCTGTATATTGGGGCTATTATATTAGGAGGGGTATTAGGGA
GGAAAATAGAAAGGTTTGGGTTTGTAGTAGTTCTTGAGAAATATGAGAGAGTCAGAGTATTTCTTATTCC
CTTTGGGTTATTGTGATGAGGAGTGCTTAGAAAATTAGGGTCTAAGCCTGCCAAATTAAGATTCTTTTTG
AGAATGTGGCTCATAGAGCTAACCCACCCCGGAAAAATGGCCTTCTTTAAGGGGTCCAGGACGGTAGCT
CAAAGTCTGGATCAAGGTTGACTAGAGCTATTGGGTCCTCAAAAGGGACTAGGTCAATTCAGCTGTTTG
AATGAAAATTATTTACCGTAGTATGAGTGGTAGCGGGGGGCGTAAGCTTAATTATGTTTATATAAATGAG
AGTTATAGTCATATGTGTAATTTTGATGGCTGTGTTTGTCTTAATTGCCAAGCAACCTATTTCTTTAGGGCT
AGTGCTATTGAGGGGGTCTATAATTGCATGTGTGGAGGTTGCACTGGAGGTTAGAAGGTTGTTAGGGTT
CTTATTGTTCCTAACTTACGTTAGGGGTGTTATAGTCCTGTTCTTGTATGTTTTAAGGATCTACCCGAATGA
ACGTTTTAATCTAGAGTTTATGGTTCTCGTCAGAAGGTGTGCCAGAGCGGTCCTTATGGGTCTTATGAAT
TACGAGAGCGGGTCTTTGTTTCTAAGTTTCATGGCTGAAAGAAGCTTATATATCCTAATAGCTGGTGTGTT
ACTGTTTGTAATATTAGTGGTGTCGTATTTGTGCATAAAAACCATGGTGCCTTTACGAAGAGTA"/>
<sequence id="seq_Perna_canaliculusMG7661341" spec="Sequence"
taxon="Perna_canaliculusMG766134" totalcount="4"
value="ATGTTGATAGATGTTTTTTCCATGTTTGATGATTATAACTTTAATACTTTTAAAAGGTGGTATGTGT
GGGGATATTCTTTGTTCCTTCCTCTTTTTATTATTTCGTCTAGTATGTCGATTTATTCTCTGTTTTTGCAAAA
ATGTATAAAATTAATTAGTAGAGGTAGAGGTATATATTTATCAGGCTTTCCTCTTCTTGTAGCTAGTATATTC
TCTATCATATTAAGCGTTAACGTATCTAATAGTATTCCTTATTTTTTCAGTGTAAGCGCTCATTTTTCATTTG
GATTTACCTTTGCTTCAGTAATTTGGCTATGTTTAATTATGTCTAGTATTTTTACTAGGTTTATCCAAAATAT
AGCTCTTTTGGTTCCAAGAGGGCCAGAGGGGTTAGCTCCATTTATAGTTCTTCTAGAAATTATTACGAAC
TTGCTTCGGCCTATTACTTTGATTATTCGGTTAGCAATAAATATAGCTACTGGTAAAGTCATTATGCTATTA
CTTGGGAACGCTGCCTTGAACTTGGCTTTTCTAAGAACTTGTGGGTTTGTAGTTATTAGGGTTTTAGGGG
CTATTTTTTCTTTGGAAGTAGCAGTAAGTTTTATTCAAAGGTATATTTTTTGTACCCTTTTGTGTCTTTATG
CTGATGAGCATAGAAAATAGCGCTGGTTTATATCAACCAATCATAAAGATATTGGTACTTTGTATTTGATTT
CGGGTATGTGGGCTGGTCTTATAGGAAGAAGTCTAAGGTTAATCATCATTCAATCTCATCCAGGGGGGAG
TTTCTTAAAAGAGAGTCTGTATAATGTAGTAGTAACTACGCATGCCCTTGTTATAATTTTTTTTGCTGTTAT
GCCGTTGTTGATCGGGGCGTTTGGAAATTGATTATTACCTTTATGTATTGGAGGGTGTGATCTTATTTTTCC
TCGTTTAAATAACCTTAGGTTTTGGTTAGCTCCAAATGCGTTATATCTTCTTATCTTGTCCTTTATAACGGA
AAAAGGGGCAGGTACTGGTTGGACTATTTATCCTCCACTGTCTTCAGGGCTGTATCATACTGGACCGGCA
GTTGATATTTTAATTACTTCTCTTCATTTGATTGGTCTGAGATCTTTGTTAGGGTCTATTAATTTCGTAAGG
ACTAATAAAAATATACCTACTGTTAAGATAAAGGGAGAAAAATCAGAGTTATACCTTTGAAGTATTACGG
TTACAGGAGTGTTGCTAATTATTTCTGTTCCTGTCCTTGCTGGGGGTATTACTATATTGTTATTTGATCGAA
ATTTTAACACTAGGTTCTTTGATCCTATCGGGGGAGGGGATCCTGTTTTATTTCAGCACGTGTTTTGGTTT
TTTGGACATCCTGAAGTTTATATCTTAATTCTTCCTGCTTTTGGAATTATATCTAAGGTTATTATGCACTATT
CAGGAAAAGATTCTGTGTTTGGTTCAGTGGGAATATTATATGCTATAGTTGGAATTGGGGTAATAGGGTG
TGTTGTTTGGGCACACCATATATTTACGGTTGGTTTAAATGTAGACACTCGAACTTACTTTACTTCTGCTA
CGATAGTGATTGCAGTTCCTACTGGTGTAAAAGTATTTAGTTGAATAGCAACTATAGGTGGAAGTAAAAT
TAAATTTAATTCTTCTGTACTGTGAAGTACTGGGTTTTTATTTTTATTTACTGTAGGAGGGCTAACTGGAAT
TATATTATCAAGGTCTTCTTTAGATGTTACGCTTCATGACACATATTATGTGACAGCTCACTTTCATTATGT

ATTATCTATGGGAGCTGTCTTTGGAATCTTCTGTGGGCTTAATCATTGATTTCCTTTATTCTATGGAGTGAA
TTTCCATAAAAAATGGTCTAAGATTCATTTTTATCTTATGTTTTTAGGGGTAAATTTAACATTTTTCCCACA
GCACTTTTTAGGGTTAAAGGGTATACCACGTCGTTATTGTGATTATCCAGATTGTTATTCTGTCTGACATT
GAGTGTCTTCATATGGAGCACTTATTTCATTTGGATCTTTGTTATATTTTATTTTCATTGTTTGAGAAGCTAT
AGTAAGTCAACGAGGTGTGTCATTATTTGAAGCTTTATTTTTTGGTGATGTATTGCAAGAGTTTGGATCTG
AGATCGTAAAATATTATGGTTATGTGATAATGGTATTATTACTTGTAATCATTGTTGTTTTGTATATAGGAAT
AATAATCATAAATCATAGATATTCTTATCGTAATTTTAAAAATCGTCAAATACTAGAGTATATTTGAACTGT
TATACCTACCTTTTTGTTAGCGATACTTTGGTGTCCATCTATCTTAAACTTATATCGAATAGAAGATCTAAA
AGATCCAGTTTGGAGGTTTAAGGCTGTTGGAAAACAATGATACTGGACTTATGAGAATGGGGAAACAAT
AGTAATTGATTCTTATATGGACCGGGATTCGGGTGTTGGATATCGCCTTCTAGATGTAGATTGACGTTTGG
TGGCTCCTGCTAACGCACAAATTTCGTGTTACGTTACAAGAAGTGATGTAATTCACTCTTTTGCCCTTCC
TGGAGCACTCTTAAAAGCAGACGCTATTCCTGGACGAATTAATGTTCTTCCAATAAAAATTAGTCAGAGC
TGTATTCTTTATGGTCAATGCTCTGAAATCTGTGGTATTAATCATAGGTTCATACCAATTGTTATTGAATTT
GTTCCAGTAGATGTATTCATAAGGTTCTACgctGGGTATAATTAA------------------------
ATGCATCGAAATCCTTATTATGTGCCTGGACCAAGTCCTTGGCCTTTGGTTGTAGGTGTTTGCTGTAATAG
CTTATGTGTAAGCTTAGTATTATGAATACATCGAATACAAGAGAAACTATTTTTGACTTTTTTAGTTTTAGG
TGTAACTGCTACGTTATGATGAACTGATGTTTTACGTGAGGGAGATATAGGGGTTCAAACTCAACATGTG
TTAAAAAGGTACCGAGATGGTGTAGGTCTTTTCATCTTTTCTGAAATTATGTTTTTCTTTTCATTTTTTTGG
GCTCTTTTTCATAATTGTTTAAGGCCTTCAGGTAATCTTGGGTGTGAATGACCTCCTTTAGGGATCCGGAC
TCTTGACCCAATATCTACAGCATTATTTAATACTTTTCTTTTAATTAGTAGGGGGTCATTTTGTACTTATGTT
CATAACACAATTCGTAAATGTTATGATAGGTGGTGTCTTGTTAATATATTACTTACTATTACTTGCGGGGTT
CTTTTTTTATTAGGACAAGGGAATGAATATTTCTTTTGTTCTTTTAGAATCGCTGACAGGGTATATGGAAG
AACTTTTTATATGTTGACTGGATTCCATGGAGGTCACGTTATAGTGGGAACAACATGGTTAATTGTTAGGT
TCTTTCGGATGTGGCGCGGTCATTTTAGAAAAGATCGGCATTTTGGACTTGAAGCATGTTTGTGGTATTG
ACATTTTGTTGATGTAGTTTGGTTGTTTGTTTGATTAATTGCTTATTTTTGAATAGGTGGACCTTTTCGTAA
GCGCCATTGGCTTCTAAAAATTTTAAATAATAGTCTTTATGACCTTCCCTGTCCTGTTAACCTAAACGCTT
TTTGAAGGTTTGGTTCTATACTAGGATTATGTATCGTAATTCAAATAGTTAGAGGTATTCTTTTATCTCTAC
ATTTTATTCCTCATGAGAGAATAGCATTCGATTCAGTGTATCATATTATGCGGGATGTTAATAAAGGCTGAT
TTCTTCGAAACATACATGTTGGGGGATGTTCTATATTTTTCATTTGTCTCTATGTTCACATTGGTCGTGGTA
TTTACTACGGATCTTATATAAGACGTCATGTATGGTTTGTAGGGGTAACTTTATTTCTAATAGTAATAGGCG
AAGCCTTTCTAGGTTATAGGTTACCTTGAGGACAAATATCTTTTTGAGGGGTAACAGTAATTTCTAATCTT
TTTACGGTGATCCCTTATGTATCGCAAAACTTACTATTTACTCTCTGAGGGCATTGAAGTGTAAGTGGGTA
CACCTTGCAACGGTTTTTTATCTTTCATTTTCTACTTCCCTTTGTCATCATTGTGTTTTCAACTCTGCATAT
ATTTTTTTTACATGAAAATGGGAGTAATAATCCTTTAGGTGTAAATAGGGACTCTATATGTGTGCCTTTTC
ACCCTTTTTACACTGTTAAAGATATTTTTGGGTTTGTGTGTTTCGGGTGGGTGTTAATATATTTAGTGTGTG
TAAATCCTGAGCTTGTAGTAAATAAAATTAACTATCATCCAGCCGATCCTTATCATACCCCCCTTCAGGTA
GAGCCAGAGTGGTACTTTTTGTTTGCGTATGCAATGCTTCGATCTATCCCTCACAAACTAGGAGGAGTTT
TAGCTTTGCTAGCATCTGTTTTAGGCTTATATTTATACCCTTTCATTCATACAGGAAAGTTTCGAAGGTTAG
CATTCTATCCTGTTAGTCAAATATTCTTTTGGTGCTTCGTTGTTAATTTCTTGAGCCTTATTTGAGTAGGGC
AAATACCTGTTCGTGAGCCATATATTACTATAGGCCAGGTGTATACAGGAGTCTATTTTTCTACATTAATTC
TGCCCCCTATGTTAACAGGGGTATGGGATAAATTAATTTTTATTTATTTTATTAGTATTATTCTTCCTTTTGT
GTGTGTGTTGGTAAGGGTGGCATTTTATACCTTGCTGGAGCGTAAAGTGCTTGGGTATATTATAATTCGAA
AAGGGCCAAATAAGGTTGGATACGCGGGTATTATACAACCCTTTAGAGATGCTGGGAAGCTTTTTAATAA
GGAGTATGTAAAGCCTGGTTTTGCTAATCTAGTGCCTTTTTTATTATGTCCTGGCATTATTTTGGTAACTAG
AATACTATTATGGCTTCTTTATCCGTATGATTATACATCTGTAATACTAACTTGCGGACTAATACAGTTTTTA
GTAACGTCAGGGATGCACGTGTATGGTGTAATAGTAGCTGGATGGTCTTCTAATTCTAAGTATTCTTTGTT
GGGAGCAGTTCGAGGAGTAGCTCAAAGAATTTCATATGAAGTACCGATAACTTTTGTTATCTTAATTGTT
GCTTATTCATTGAGAAGATTATGGATTCAAGAAGTTAAGGGAATCTTTTCAATAGCGATAGGGTTACTTAG
AAGGGGGGTATGAATCACTTGTATGTTGGCTGAGACCAATCGGGCGCCGTTTGATTTTGTGGAAGGGGA
GTCTGAGCTAGTTTCTGGGTTTAATGTAGAGTATAGTAGAGGAGGATTTGCGATAATTTTTATGGCTGAGT
ATGGGGCTATATTATTCAATAGTGTTTTCTTTGTGACTCTTTTTTTAGGAGGAGGGGAGTTATTAATAAGA
GTAAGAACAATAATTTGGGTTCTATTTTTTTATCTGAATTCGTGGAACTGTTCCACGAATTCGATATGATCA
GTTAATAGGGTTGTGTTGGAAAGTTCTCCTAAGGGTAATGTTGAGTATAAGAGGGTTAATTTTAATTATCA
GGTTTTCCTTATGATAGGAGTTGCGTTAAGTCCTCTATTGATTTTTTGTGGGGTACTAGTAATTATAGGTAA
TCTCTTAAGAATTAGAAGTAGTTCTTGGTTAGGGGTGTGGCTAGGAATGGAAATTAACCTTTTTGGGTTT
TTGATTATAATAAATCCTGAAGGTTGTTGTGTTGCTCAGAGGGGAGTAAAGTATTTTGTTACTCAGAGAA
TTGGGTCTATACTATTATTATTTGGGTTTTTTTTAGTTTTTCTATTTAATAGGGTAGGGGCTGTAGTTATCAT
AATAGGATTGTTAATAAAAACAGGAATCTTTCCATTTCATAACTGGGTTCCAGACGTTGTTATAGTTTCAA
GTTGACTAATTGGTTGTTTAATTTTGACTTGGCAAAAGTTAGCCCCATTTTCCTTTTTTTCATTCTTCCCC
GGTTCTTGGTTTCTTATTACTAGGTTGGTTTTTATGAGCTTAGTAGGCTGTCTTGGTGGATTAAATCAGAA
TTCTGTTCGGGGAATAGCAGTTTATTCTTCATTTGTACATAATTCCTGGATAATACTTTCTTTACTTTACTC
ATTTAGGGTATTTTTTGTTTATTACTTAATTTACAGATTGAGAGTGGTGGTATTTTTTTTTAGGTGTTGAAC
TCTTAGAAAAAGTAGAATGATGAGATATTCAATTAGGTGGATAGGGATGCTAAGACTGTTAATATTGTCT

GGGGTACCCCCCTTCATAGGATTTTTATAAAATTGATTGTGGTATTAAGGTGTCCTATTATATTATTAATCA
TTTGTTTGTTAAGGTCAGTAGTTAGATTGAAGTTTATATATCGTTTTTTACAGAATAATTTAAATAGAA
TTTATTGTGTAAAGTTATTTTTAGCATTTAGTCTAGCTAACTTTATTATAGGAAGAATTTCTATAAGGTACT
TTATTTGGTAGATGATCGAATGTTTAACCCTTGTGTTTCTTATTGCTTTAAGACTAATTTTGATACTAGTAG
CTTTGTTATCTGAAAAATCATTTGAAAGTCGAGAAAAGTCTTCTCCGTATGAATGTGGTTTTGATCCTATT
TTAAGGGCTCGAAGTTCTTTCTCATTACGGTTTTTTCTTCTTGCAGTTTTGTTTGTCGTTTTCGACGTAGA
ATTGTCTTTATTAATACCTGTAGTAACAAGGTTGATAAGAGGTTTTAATTATATTAGTCTGGTTAGATGCTT
TTTATTTTTAGTAATTCTATTTCTAGGCATTTATCATGAGTGACGAGAAGGGTCTTTGAATTGAGTAGTTTA
AGTGGCCGTAAGTTTGATTATTTCTTTGATTATACTCTTTGTTTTAGGTAATAGGACAATTAGGTTAATAGT
TTTATTTTTCTTATTTTTAACTGTTTTAGAGATAGGCACTATCAGGGACGAATTTGTAGCATTAGGCGGGA
GGGTAAGGTACGATTTATTGTCTTATAGGTTAAGTGTTCTAAGAATTTTTATTATATTGCTTAGCCTGATAA
GGAGTAAGAGGGTACAACGAATTAAGTTATTTTATTTTATGAACTTAGCTTTGTTAATAGTCTTAGTTTTA
GCTTTTTGCACATCTAGTTTTTTGGGTTTTTTCTTTTTTTTTGAATCTGCTTTAATTCCATTGATCCTTATTA
TCATGGGCTGGCGGATGGAGGCAAGGACTTATATATTTATTTATACGGTGTTCGGATCATTGTTCTTCTTAT
TTGGAGTGTGTTTGTTGTTTTATAGGGGGAGGGATAATATGTTATTAGGAAGAAATATTTCTAAAAAAGT
GAGAGGGTTTTGGTGGTTGTTTGTTCTGGGGTTTCTAGTTAAATTGCCGGTTTATCCATTTCATTTATGAT
TACCTAAAGCTCATGTCGAGGCGCCTGTAGCTGGTTCAATAGTATTAGCTGGAGTTGTTCTTAAGTTAGG
GGGCTATGGATTACTTCGACTTATAATAGTGATAAGAGTAAATTTAGGGTTAAAGTCTAATATAATGTTGA
GAATTGGGATGGTAGGTGGTGTATATGCTAGATTAGTCTGTTGCCGCCAGACAGACCTAAAATGTCTAGT
CGCTTACTCTTCTGTTGGGCATATAAGGCTGGTAATGCTATGTCTTAGAAATATTAGAATAGGGGTTAAGG
GAGCTCTATTTATTATAGTAGGTCATGGTCTATGCTCCTCTGGGATGTTTAGGTTGGTGAACGTATTTTACA
ATCATTCAGGGTATCGTAATCTTTACATAAACAAGGGTTTCCTGATGAAAAGTCCAGTTTTATGTTTAGTA
GGATTTCTTCTGTGTTCTAGAAATATAGCAGCCCCTCCCAGTCTAAATTTGTTTGGCGAAGTTTTAATGTT
TGTGTGTTCTTACTTGATTTCATTCTGTTTCTTAGTTTTACTAATGGTAATAACAATTATTAGAGCTGTATAC
AGACTTCACTTATACACAACAACTTGTCATGGAAAATCTTCTGAAGCCAAGGTTAGGTTAGAGACTTATA
GGGCTGTTTTCGTATTATTAGTACATTGAATTCCATTGAATTTCATCTTTTTGTTTATTCCTTAGACTAAAA
AAAATCATTTAATTAGTGTATTTATTGGGATAGAGTTTATAATATTAGGAATTTTATACTGCTGTAGTAGATT
AATATTTTATAATATTAGAATAATTTTTCTAGTAATGTGTTTAGGGGTTTGTGAGGCAAGGGTGTGCTTAGC
TCTTTTAGTAATAATAGTACGACTTTCAGGGAATGATCTGATCTCGAGACTTTCTTTGTACAATTTAATAAT
TTTTCTCAAAAAATGAGGTTGATAGTTATATTAGGCTATGTATTGGTGTTATTAAGGAGGTTCGAAAGTGT
TTATTTTTTTGAATTCATGTTATGGGATTTAAACAGATTAAGGTTCTCTGTAAATTTACTCTTAGATTCATT
GAGGATAATATTTTTAGGTACTATTTTAATTATTTCAGGTAGGGTTTTAATATACTGTTATTGATACATGGAT
GATGAAAAGAACTTTTATCGATTTATATATTTAGTCTATTTATTTGTAGGGTCTATGATGTTTATGATTCTTA
TTCCTAATCTTGTTACCCTTCTTATTGGCTGGGACGGTTTAGGGCTTACTTCTTTTTTGCTTGTTGCACATT
ATCAAAATAATAAATCTTTGTCAGGAAGTTTGTTAACGGCTTTAACTAACCGTATCGGGGACGGTCTGAT
CTTGGCTAGAATTTCATTATGAGCGGTTGAGAGGGGTTGGGTATTGTATAATTTTGAAAGTCAGTTTGTG
AATTTTTTTATTTTAGCCTTAATTTTTGGAGGAATAACAAAAAGAGCTCAAATACCTTTTTGTGCTTGGTT
GCCTGCTGCTATAGCTGCTCCTACTCCAGTTTCTTCTCTAGTTCATTCATCTACACTAGTGACTGCTGGGG
TATATTTATTAATTCGTTCTTATCCAGTAATTAAGGGAAGCTTGATGACTAGTTTAAAGTTTCTTAGTTTGT
TTACATTGGTTTTAGCTAGAAGTGTTGCTATTAACTGTTTTGATATAAAGAAAATCATTGCTTTGTCGACT
TTAAGTCAATTAAGGGTGATAATATTTTCATTGTCTCATGGTCTTGTTTCTATTTCTTTTTTTCATTTGGTAA
TGCATGCCCTATTTAAAGCTTTATTATTTTTAACAGCAGGTGCAGTAATTCATTCTCTCAAAGGGTGTCAA
GATATTCGAAGGATAGGGGGATGCTGGAGAATTTTACCAAGTAGAATAGTGTGTATGTTCATTGCTAATT
GTTCATTGTCAGGGCTTCCATTTATAAGAGGGTTTTATTCTAAAGATATAATTCTGGATATATTTAATAGGA
GAAATTTTTGGTTTATAATTATTATACCGGGGCTTATACTTACTAGATTTTATAGCATGCGAGTTTGAATAG
TTTGCTTCGGAAGGAATAAGGTATCAATTAGTACACTTTGTTTAAAAGAGCCAAAAAGGCTACTTATTCC
TTATTCGCTACTAGCAGTCGGTGCTGTGTTTCTAGGAGGAGTTTTAACTCCTAGTGAGTGTCTGTTTAGTT
ATCGGAGATCTTCGGCGAGAGAAATAGGAACTTTAGGGGTTCTTTTTGTTTTTGGAATTGGACTAATTAG
AGTTTCTAGAAAGTTTGGGAAAGATTTCCGGGGCTTTTCGTTCCTATTTAGAATGTGATTTTTAGAGTTG
ACTCACGGAGTAAAAAAATGTTTTTTTGATGTAAGGGGGACAATTTTGAAAAGATGTGACAAAGGAATA
TTAGAGACTGTAGGTCCTAAAAGATTCTGTATAAAATTAAGTTCTTATAATGAAGATTATTTTTTGCTATTT
TTTTTTTTTATTGGAGCTCTCTCTTTTCTTTTCTTTTGAAATATGCAACTCCTTTTAATTTACTGAACTGTA
ATTAGTTTGTGTGTGGTTTTTATTGGACAGCCTTATCTATTAGGGCTAGTGCTGTTGATGACTTCTATGTTT
GTGTGTATGATTATTGGGGTAAATGTAAGAACATTTTTAGCTTTACTTTTATTCATGAGATATGTGGGGGG
TTTAATGGTTCTTTTCATTTATGTGTTAAGTGTATTTCCTAATGAAAATTTCGAAGTAATGGGCTTTATGAT
TGTGAGTGTGGCAATAATGAGATTTTTAATATTTCCAGTAAAAAGGCAAAGAAAGGGATTATTCtttCACTA
TATAAGGTTTGTGGGGGTTTTTGTTTTTATAGCCCTTTTTCTTTTATTTATTATGATGTGTGTATCGTATCTT
GTAATAAAAAAACGAATGCCATTGCGTGTAAAT"/>
<sequence id="seq_Perna_canaliculusMK7755581" spec="Sequence"
taxon="Perna_canaliculusMK775558" totalcount="4"
value="ATGTTGATAGATGTTTTTTCCATGTTTGATGATTATAACTTTAATACTTTTAAAAGGTGGTATGTGT
GGGGATATTCTTTGTTCCTTCCTCTTTTTATTATTTCGTCTAGTATGTCGATTTATTCTCTGTTTTTGCAAAA
ATGTATAAAATTAATTAGTAGAGGTAGAGGTATATATTTATCAGGCTTTCCTCTTCTTGTAGCTAGTATATTC

TCTATTATATTAAGCGTTAACGTATCTAATAGTATTCCTTATTTTTTCAGTGTAAGCGCTCATTTTTCATTTG
GATTTACTTTTGCTTCAGTAATTTGGCTATGTTTAATTATGTCTAGTATTTTTACTAGGTTTATCCAAAATAT
AGCTCTTTTGGTTCCAAGAGGGCCAGAGGGGTTAGCTCCATTTATAGTTCTTCTAGAAATTATTACGAAC
TTGCTTCGGCCTATTACTTTGATTATTCGGTTAGCAATAAATATAGCTACTGGTAAAGTCATTATGCTATTA
CTTGGGAACGCTGCCTTGAACTTGGCTTTTTTAAGAACTTGTGGGTTTGTAGTTATTAGGGTTTTAGGGG
CTATTTTTTCTTTGGAAGTAGCAGTAAGTTTTATTCAAAGGTATATTTTTTGTACCCTTTTGTGTCTTTATG
CTGATGAGCATAGAAAATAGCGCTGGTTTATATCAACCAATCATAAAGATATTGGTACTTTGTATTTGATTT
CGGGTATGTGGGCTGGTCTTATAGGAAGAAGTCTAAGGTTAATCATCATTCAATCTCATCCAGGGGGGAG
TTTCTTAAAAGAGAGTCTGTATAATGTAGTAGTAACTACGCATGCCCTTGTTATAATTTTTTTTGCTGTTAT
GCCGTTGTTGATCGGGGCGTTTGGAAATTGATTATTACCTTTATGTATTGGAGGGTGTGATCTTATTTTTCC
TCGTTTAAATAACCTTAGGTTTTGGTTAGCTCCAAATGCGTTATATCTTCTTATCTTGTCCTTTATAACGGA
AAAAGGGGCAGGTACCGGTTGGACTATCTATCCTCCACTGTCTTCAGGGCTGTATCATACTGGACCGGC
AGTTGATATTTTAATTACTTCTCTTCATTTGATTGGTCTGAGATCTTTGTTAGGGTCTATTAATTTCGTAAG
GACTAATAAAAATATACCTACTGTTAAGATAAAGGGAGAAAAATCAGAGTTATACCTTTGAAGTATTACG
GTTACAGGAGTGTTGCTAATTATTTCTGTTCCTGTCCTTGCTGGGGGTATTACTATATTGTTATTTGATCGA
AATTTTAACACTAGGTTCTTTGATCCTATCGGGGGAGGGGATCCTGTTTTATTTCAGCACGTGTTTTGGTT
TTTTGGACATCCTGAAGTTTATATCTTAATTCTTCCTGCTTTTGGAATTATATCTAAGGTTATTATGCACTAT
TCAGGAAAAGATTCTGTGTTCGGTTCAGTGGGAATATTATATGCTATAGTTGGAATTGGGGTAATAGGGT
GTGTTGTTTGGGCACACCATATATTTACGGTTGGTTTAAATGTAGACACTCGAACTTACTTTACTTCTGCT
ACGATAGTGATTGCAGTTCCTACTGGTGTAAAAGTATTTAGTTGAATAGCAACTATAGGTGGAAGTAAAA
TTAAATTTAATTCTTCTGTACTGTGAAGTACTGGGTTTTTATTTTTATTTACTGTAGGAGGGCTAACTGGA
ATTATATTATCAAGGTCTTCTTTAGATGTTACGCTTCATGACACATATTATGTGACAGCTCACTTTCATTAT
GTATTATCTATGGGAGCTGTCTTTGGAATCTTCTGTGGGCTTAATCACTGATTTCCTTTATTCTATGGAGTG
AATTTCCATAAAAAATGGTCTAAGATTCATTTTTATCTTATGTTTTTAGGGGTAAATTTAACATTTTTCCCA
CAGCACTTTTTAGGGTTAAAGGGTATACCACGTCGTTATTGTGATTATCCAGATTGTTATTCTGTCTGACA
TTGAGTGTCTTCATATGGAGCACTTATTTCATTTGGATCTTTGTTATATTTTATTTTCATTGTTTGAGAAGCT
ATAGTAAGTCAACGAGGTGTGTCATTATTTGAAGCTTTATTTTTTGGTGATGTATTGCAAGAGTTTGGATC
TGAGATCGTAAAATATTATGGTTATGTGATAATGGTATTATTACTTGTAATCATTGTTGTTTTGTATATAGGA
ATAATAATCATAAATCATAGATATTCTTATCGTAATTTTAAAAATCGTCAAATACTAGAGTATATTTGAACTG
TTATACCTACCTTTTTGTTAGCGATACTTTGGTGTCCATCTATCTTAAACTTATATCGAATAGAAGATCTAA
AAGATCCAGTTTGGAGGTTTAAGGCTGTTGGAAAACAATGATACTGGACTTATGAGAATGGGGAAACAA
TAGTAATTGATTCTTATATGGACCGGGATTCGGGTGTTGGATATCGCCTTCTAGATGTAGATTGACGTTTG
GTGGCTCCTGCTAACGCACAAATTTCGTGTTACGTTACAAGAAGTGATGTAATTCACTCTTTTGCCCTTC
CTGGAGCACTCTTAAAAGCAGACGCTATTCCTGGACGAATTAATGTTCTTCCAATAAAAATTAGTCAGAG
CTGTATTCTTTATGGTCAATGCTCTGAAATCTGTGGTATTAATCATAGGTTCATACCAATTGTTATTGAATT
TGTTCCAGTAGATGTATTCATAAGGTTCTACgctGGGTATAATTAA------------------------
ATGCATCGAAATCCTTATTATGTGCCTGGACCAAGTCCTTGGCCTTTGGTTGTAGGTGTTTGCTGTAATAG
CTTATGTGTAAGCTTAGTATTATGAATACATCGAATACAAGAGAAACTATTTTTGACTTTTTTAGTTTTAGG
TGTAACTGCTACGTTATGATGAACTGATGTTTTACGTGAGGGAGATATAGGGGTTCAAACTCAACATGTG
TTAAAAAGGTACCGAGATGGTGTAGGTCTTTTCATCTTTTCTGAAATTATGTTTTTCTTTTCATTTTTTTGG
GCTCTTTTTCATAATTGTTTAAGGCCTTCAGGTAATCTTGGGTGTGAATGACCTCCTTTAGGGATTCGGAC
TCTTGACCCAATATCTACAGCATTATTTAATACTTTTCTTTTAATTAGTAGGGGGTCATTTTGTACTTATGTT
CATAACACAATTCGTAAATGTTATGATAGGTGGTGTCTTGTTAATATATTACTTACTATTACTTGCGGGGTT
CTTTTTTTATTAGGACAAGGGAATGAATATTTCTTTTGTTCTTTTAGAATCGCTGACAGGGTATATGGAAG
AACTTTTTATATGTTGACTGGATTCCATGGAGGTCACGTTATAGTGGGAACAACATGGTTAATTGTTAGGT
TCTTTCGGATGTGGCGCGGTCATTTTAGAAAAGATCGGCATTTTGGACTTGAAGCATGTTTGTGGTATTG
ACATTTTGTTGATGTAGTTTGGTTGTTTGTTTGATTAATTGCTTATTTTTGAATAGGTGGACCTTTTCGTAA
GCGCCATTGGCTTCTAAAAATTTTAAATAATAGTCTTTATGACCTTCCCTGTCCTGTTAACTTAAACGCTT
TTTGAAGGTTTGGTTCTATACTAGGATTATGTATCGTAATTCAAATAGTTAGAGGTATTCTTTTATCTCTAC
ATTTTATTCCTCATGAGAGAATAGCATTCGATTCAGTGTATCATATTATGCGGGATGTTAATAAAGGCTGAT
TTCTTCGAAACATACATGTTGGGGGATGTTCTATATTTTTCATTTGTCTCTATGTTCACATTGGTCGTGGTA
TTTACTACGGATCTTATATAAGACGTCATGTATGGTTTGTAGGGGTAACTTTATTTCTAATAGTAATAGGCG
AAGCCTTTCTAGGTTATAGGTTGCCTTGAGGACAAATATCTTTTTGAGGGGTAACAGTAATTTCTAATCTT
TTTACGGTGATCCCTTATGTATCGCAAAACTTACTATTTACTCTTTGAGGGCATTGAAGTGTAAGTGGGTA
CACCTTGCAACGGTTTTTTATCTTTCATTTTCTACTTCCCTTTGTCATCATTGTGTTTTCAACTCTGCATAT
ATTCTTTTTACATGAAAATGGGAGTAATAATCCTTTAGGTGTAAATAGGGACTCTATATGTGTGCCTTTTC
ACCCTTTTTATACTGTTAAAGATATTTTTGGGTTTGTGTGTTTCGGGTGGGTGTTAATATATTTAGTGTGTG
TAAATCCTGAGCTTGTAGTAAATAAAATTAACTATCATCCAGCCGATCCTTATCATACCCCCCTTCAGGTA
GAGCCAGAGTGGTACTTTTTGTTTGCGTATGCAATGCTTCGATCTATCCCTCACAAACTAGGAGGAGTTT
TAGCTTTGCTAGCATCTGTTTTAGGCTTATATTTATACCCTTTCATTCATACAGGAAAGTTTCGAAGGCTA
GCATTCTATCCTGTTAGTCAAATATTCTTTTGGTGCTTCGTTGTTAATTTCTTGAGCCTTATTTGAGTAGGG
CAAATACCTGTTCGTGAGCCATATATTACTATAGGCCAGGTGTATACAGGAGTCTATTTTTCTACATTAATT
CTGCCCCCTATGTTAACAGGGGTATGAGATAAATTAATTTTTATTTATTTTATTAGTATTATTCTTCCTTTTG

TGTGTGTGTTGGTAAGGGTGGCATTTTATACCTTGCTGGAGCGTAAAGTGCTTGGGTATATTATAATTCGA
AAAGGGCCAAATAAGGTTGGATACGCGGGTATTATACAACCCTTTAGAGATGCTGGGAAGCTTTTTAATA
AGGAGTATGTAAAGCCTGGTTTTGCTAATCTAGTGCCTTTTTTATTATGTCCTGGCATTATTTTGGTAACTA
GAATACTATTATGGCTTCTTTATCCGTATGATTATACATCTGTAATACTAACTTGCGGACTAATACAGTTTTT
AGTAACGTCAGGGATGCACGTGTATGGTGTAATAGTAGCTGGATGGTCTTCTAATTCTAAGTATTCTTTGT
TGGGAGCAGTTCGAGGAGTAGCTCAAAGAATTTCATATGAAGTACCAATAACTTTTGTTATCTTAATTGT
TGCTTATTCATTGAGAAGATTATGGATTCAAGAAGTTAAGGGAATCTTTTCAATAGCGATAGGGTTACTTA
GAAGGGGGGTATGAATCACTTGTATGTTGGCTGAGACTAATCGGGCGCCGTTTGATTTTGTGGAAGGGG
AGTCTGAGCTAGTTTCTGGGTTTAATGTAGAGTATAGTAGAGGAGGATTTGCGATAATTTTTATGGCTGA
GTATGGGGCTATATTATTCAATAGTGTTTTCTTTGTGACTCTTTTTTTAGGAGGAGGGGAGTTATTAATAAG
AGTAAGAACAATAATTTGGGTTCTATTTTTTATCTGAATTCGTGGAACCGTTCCACGAATTCGATATGATC
AGTTAATAGGGTTGTGTTGGAAAGTTCTCCTAAGGGTAATGTTGAGTATAAGAGGGTTAATTTTAATTATC
AGGTTTTCCTTATGATAGGAGTTGCGTTAAGTCCTCTATTGATTTTTTGTGGGGTACTAGTAATTATAGGTA
ATCTCTTAAGAATTAGAAGTAGTTCTTGGTTAGGGGTGTGGCTAGGAATAGAAATTAACCTTTTTGGGTT
TTTGATTATAATAAATCCTGAAGGTTGTTGTGTTGCTCAGAGGGGAGTAAAGTATTTTGTTACTCAGAGA
ATTGGGTCTATACTATTATTATTTGGGTTTTTTTTAGTTTTTCTATTTAATAGGGTAGGGGCTGTAGTTATCA
TAATAGGATTGTTAATAAAAACAGGAATCTTTCCATTTCATAACTGGGTTCCAGACGTTGTTATAGTTTCA
AGTTGACTAATTGGTTGTTTAATTTTGACTTGGCAAAAGTTAGCCCCATTTTCCTTTTTTTCATTCTTCCC
CGGTTCTTGGTTTCTTATTACTAGGTTGGTTTTTATGAGCTTAGTAGGCTGTCTTGGTGGATTAAATCAGA
ATTCTGTTCGGGGAATAGCAGTTTATTCTTCATTTGTACATAATTCCTGGATAATACTTTCTTTACTTTACT
CATTTAGGGTATTTTTTGTTTATTACTTAATTTACAGATTGAGAGTGATGGTATTTTTTTTTAGGTGTTGAA
CTCTTAGAAAAAGTAGAATGATGAGATATTCAATTAGGTGGATAGGGATGCTAAGACTGTTAATATTGTCT
GGGGTACCCCCCTTCATAGGATTTTTTATAAAATTGATTGTGGTATTAAGGTGTCCTATTATATTATTAATCA
TTTGTTTGTTAAGGTCAGTAGTTAGATTGAAGTTTTATATATCGTTTTTTTACAGAATAATTTTAAATAGAA
TTTATTGTGTAAAGTTATTTTTAGCATTTAGTCTAGCTAACTTTATTATAGGAAGAATTTCTATAAGGTACT
TTATTTGGTAGATGATTGAATGTTTAACCCTTGTGTTTCTTATTGCTTTAAGACTAATTTTGATACTAGTAG
CTTTGTTATCTGAAAAATCATTTGAAAGTCGAGAAAAGTCTTCTCCGTATGAATGTGGTTTTGATCCTATT
TTAAGGGCTCGAAGTTCTTTCTCATTACGGTTTTTTCTTCTTGCAGTTTTGTTTGTCGTTTTCGACGTAGA
ATTGTCTTTATTAATACCTGTAGTAACAAGGTTGATAAGAGGTTTTAATTATATTAGTCTGGTTAGATGCTT
TTTATTTTTAGTAATTCTATTTCTAGGCATTTATCATGAGTGACGAGAAGGGTCTTTGAATTGAGTAGTTTA
AGTGGCCGTAAGTTTGATTATTTCTTTGATTATACTCTTTGTTTTAGGTAATAGGACAATTAGGTTAATAGT
TTTATTTTTCTTATTTTTAACTGTTTTAGAGATAGGCACTATCAGGGACGAATTTGTAGCATTAGGCGGGA
GGGTAAGGTACGATTTATTGTCTTATAGGTTAAGTGTTCTAAGAATTTTTATTATATTGCTTAGCCTAATAA
GGAGTAAGAGGGTACAACGAATTAAGTTATTTTATTTTATGAACTTAGCTTTGTTAATAGTCTTAGTTTTA
GCTTTTTGCACATCTAGTTTTTTGGGTTTTTTCTTTTTTTTTGAATCTGCTTTAATTCCATTGATCCTTATTA
TCATGGGCTGGCGGATGGAGGCAAGGACTTATATATTTATTTATACGGTGTTCGGATCATTGTTCTTCTTAT
TTGGAGTGTGTTTGTTGTTTTATAGGGGGAGGGATAACATGTTATTAGGAAGAAACATTTCTAAAAAAGT
GAGAGGGTTTTGGTGGTTGTTTGTTCTGGGGTTTCTAGTTAAATTGCCGGTTTATCCATTTCATTTATGAT
TACCTAAAGCTCATGTCGAGGCGCCTGTAGCTGGTTCAATAGTATTGGCTGGAGTTGTTCTTAAGTTAGG
GGGCTATGGATTACTTCGACTTATAATAGTGATAAGAGTAAATTTAGGGTTAAAGTCTAATATAATGTTGA
GAATTGGGATGGTAGGTGGTGTATATGCTAGATTAGTCTGTTGTCGCCAGACAGACCTAAAATGTCTAGT
CGCTTACTCTTCTGTTGGGCATATAAGGCTGGTAATGCTATGTCTTAGAAATATTAGAATAGGGGTTAAGG
GAGCTCTATTTATTATAGTAGGTCATGGTCTATGCTCCTCTGGGATGTTTAGGCTGGTGAACGTATTTTAC
AATCATTCAGGGTATCGTAATCTTTACATAAACAAGGGTTTCCTGATGAAAAGCCCAGTTTTATGTTTAGT
AGGATTTCTTCTGTGTTCTAGAAATATAGCAGCCCCTCCCAGTCTAAATTTGTTTGGCGAAGTTTTAATGT
TTGTGTGTTCTTACTTGATTTCATTCTGTTTCTTAGTTTTACTAATGGTAATAACAATTATTAGAGCTGTATA
CAGACTTCACTTATACACAACAACTTGTCATGGAAAATCTTCTGAAGCCAAGGTTAGGTTAGAGACTTAT
AGGGCTGTTTTTGTATTATTAGTACATTGAATTCCATTGAATTTCATCTTTTTGTTTATTCCTTAGACTAAA
AAAAATCATTTAATTAGTGTATTTATTGGGATAGAGTTTATAATATTAGGAATTTTATACTGCTGTAGTAGAT
TAATATTTTATAATATTAGAATAATTTTTCTAGTAATGTGTTTAGGGGTTTGTGAGGCAAGGGTGTGCTTAG
CTCTTTTAGTAATAATAGTACGACTTTCAGGGAATGATCTGATCTCGAGACTTTCTTTGTACAAtttaataatttttc
tcaaaaaATGAGGTTGATAGTTATATTAGGCTATGTATTGGTGTTATTAAGGAGGTTCGAAAGTGTTTATTTTT
TTGAATTCATGTTATGGGATTTAAACAGATTAAGGTTCTCTGTAAATTTACTCTTAGATTCATTGAGGATA
ATATTTTTAGGTACTATTCTAATTATTTCAGGTAGGGTTTTAATATACTGTTATTGATACATGGATGATGAAA
AGAACTTTTATCGATTTATATATTTAGTCTATTTATTTGTAGGGTCTATGATGTTTATGATTCTTATTCCTAAT
CTTGTTACCCTTCTTATTGGCTGGGACGGTTTAGGGCTTACTTCTTTTTTGCTTGTTGCACATTATCAAAA
TAATAAATCTTTGTCAGGAAGTTTGTTAACGGCTTTAACTAACCGTATCGGGGACGGTCTGATCTTGGCT
AGAATTTCACTATGAGCGGTTGAGAGGGGTTGGGTATTGTATAATTTTGAAAGTCAGTTTGTGAATTTTT
TTATTTTAGCCTTAATTTTTGGAGGAATAACAAAAAGAGCTCAAATACCTTTTTGTGCTTGGTTGCCTGCT
GCTATAGCTGCTCCTACTCCAGTTTCTTCTCTAGTTCATTCATCTACACTAGTGACTGCTGGGGTATATTTA
TTAATTCGTTCTTATCCAGTAATTAAGGGGAGCTTGATGACTAGTTTAAAGTTTCTTAGTTTGTTTACATT
GGTTTTAGCTAGAAGTGTTGCTATTAACTGTTTTGATATAAAGAAAATCATTGCTTTGTCGACTTTAAGTC
AATTAAGGGTGATAATATTTTCATTGTCTCATGGTCTTGTTTCTATTTCTTTTTTTCATTTGGTAATGCATGC

CCTATTTAAAGCTTTATTATTTTTAACAGCAGGTGCAGTAATTCATTCTCTCAAAGGGTGTCAAGATATTC
GAAGGATAGGGGGATGCTGGAGAATTTTACCAAGTAGAATAGTGTGTATGTTTATTGCTAATTGTTCATTG
TCAGGGCTTCCATTTATAAGAGGGTTTTATTCTAAAGATATAATTCTGGATATATTTAATAGGAGAAATTTT
TGGTTTATAATTATTATACCGGGGCTTATACTTACTAGATTTTATAGCATGCGAGTTTGAATAGTTTGCTTC
GGAAGGAATAAGGTATCAATTAGTACACTTTGTTTAAAAGAGCCAAAAAGGCTACTTATTCCTTATTCGC
TACTAGCAGTCGGTGCTGTGTTTCTAGGAGGAGTTTTAACTCCTAGTGAGTGTCTGTTTAGTTATCGGAG
ATCTTCGGCGAGAGAAATAGGAACTTTAGGGGTTCTTTTTGTTTTTGGAATTGGACTAATTAGAGTTTCT
AGAAAGTTTGGGAAAGATTTCCGGGGCTTTTCGTTCCTATTTAGAATGTGATTTTTAGAGTTGACTCACG
GAGTAAAAAAATGTTTTTTTGATGTAAGGGGGATAATTTTGAAAAGATGTGACAAAGGAATATTAGAGA
CTGTAGGTCCTAAAAGATTCTGTATAAAATTAAGTTCTTATAATGAAGATTATTTTTTGCTATTTTTTTTTTT
TATTGGAGCTCTTTCTTTTCTTTTCTTTTGAAATATGCAACTCCTTTTAATTTACTGAACTGTAATTAGTTT
GTGTGTGGTTTTTATTGGACAGCCTTATCTATTAGGGCTAGTGCTGTTGATGACTTCTATGTTTGTGTGTAT
GATTATTGGGGTAAATGTAAGAACATTTTTAGCTTTACTTTTATTCATGAGATATGTGGGGGGTTTAATGG
TTCTTTTCATTTATGTGTTAAGTGTATTTCCTAATGAAAATTTCGAAGTAATGGGCTTTATGATTGTGAGTG
TGGCAATAATGAGATTTTTAATATTTCCAGTAAAAAGGCAAAAAAAGGGATTATTTtttCACTATATAAGGT
TTGTGGGGGTTTTTGTTTTTATAGCCCTTTTTCTTTTATTTATTATGATGTGTGTATCGTATCTTGTAATAAA
AAAACGAATGCCATTGCGTGTAAAT"/>
<sequence id="seq_Perna_pernaOK5764811" spec="Sequence" taxon="Perna_pernaOK576481"
totalcount="4"
value="ATGTTAATAGATGTTTTTTCCATGTTTGATGATTATAATTTTAATACTTTTAAAAGGTGGTATGTGTG
GGGGTATTCTTTATTATTTCCTCTTTTTATTGTTTCATCTAGTGTGTCAATTTATTTTTCGTTTTTACAGAAG
TGTATGAAGTTAATTAACAGGGGAAGAGGGTTATACTTGTCTGGATTTCCACTATTAGTAGGTAGTTTATT
TTCTATTATACTAAGAGTAAATGTATCTAACAGCATTCCTTATTTTTTTAGGGTAAGAGCACATTTTTCATT
TGGGTTTACCTTTGCTTCGGTGGTGTGATTATGTCTGATTACATCTAGAATTTTTACTAGCTTTATTCAAAA
TATAGCTCTTCTAGTTCCGAGTGGACCGGAAGGTTTAGCACCTTTTATAGTGTTGCTAGAAATTATCACTA
ATTTGCTTCGTCCTATTACGTTGATCATTCGGTTGGCTATAAATATAGCTACTGGTAAAGTAATTATACTCT
TACTTGGCAATGCGGCTTTAAATCTAGCTTTTGCGAGATCTTGTGGGTTTGTGGTGGTTAGAGTGTTGGG
GGCAATTTTTTCTCTGGAAGTGGCGGTAAGGTTTATTCAAAGATACATTTTTTGTACTCTTTTATGTCTTT
ACGCTGACGAGCATAGAAAATAACGATGGTTTATGTCAACCAATCATAAAGATATTGGTACTTTGTATTTG
CTCTCAGGAATGTGGGCAGGCCTTATAGGCAGGAGGTTGAGATTAATCATTATTCAATCTCACCCAGGGG
GAAGGTTTTTGAAAGAAAGATTATATAACGTGGTGGTGACTACCCACGCTCTTGTTATAATCTTTTTTGCT
GTTATACCGTTGCTTATTGGGGCTTTTGGAAATTGATTATTGCCTTTGTGTATTGGAGGGTGTGATTTAATT
TTTCCTCGATTAAATAATTTGAGGTTCTGGTTAGCTCCGAATGCTCTTTATTTATTGATCTTATCTTTTATAA
CAGAAAAAGGGGCTGGTACAGGTTGAACTATTTACCCTCCTCTATCATCTGGTCTATATCATACTGGGCCT
GCAGTTGATATTTTAATTACCTCTCTTCACTTGATTGGATTAAGCTCTTTATTAGGATCTATTAATTTTGTCA
GGACTAATAAGAATATGCCAACAGTTAAGATAAAAGGGGAAAAATCTGAGTTATATCTGTGAAGAATTAC
TGTCACAGGTGTATTGTTAATTATTTCTGTACCTGTTCTTGCTGGAGGGATTACAATACTTTTATTTGATCG
AAATTTCAACACTAGATTCTTTGATCCTATTGGAGGGGGAGATCCTGTTCTATTTCAGCATGTTTTCTGAT
TTTTTGGTCACCCTGAAGTTTATATTTTAATTCTTCCTGCCTTTGGAATTATGTCTAAAGTAATTATACATTA
TTCTGGAAAAGATTCTGTATTTGGTTCAGTGGGAATATTATACGCTATAGTGGGAATCGGAGTAATAGGAT
GTGTTGTTTGGGCGCATCACATGTTTACGGTAGGATTGAATGTGGATACTCGAACTTATTTTACTTCTGCA
ACTATGGTAATTGCAGTTCCTACAGGGGTGAAAGTTTTTAGTTGGATAGCAACAATAGGTGGAAGTAAA
ATTAAATTCACTACATCTGTCCTGTGGAGCACAGGGTTCTTATTTCTGTTTACGGTAGGGGGTCTGACAG
GGATTATGTTATCAAGCTCTTCTTTGGATGTTACTCTTCATGATACGTATTATGTGACAGCTCATTTTCATT
ATGTATTATCTATAGGAGCTGTGTTTGGTATTTTCTGTGGTTTAAATCATTGGTTTCCTCTATTTTATGGGGT
AAATTTTCACAAAAAATGATCTAAGGTTCACTTTTATCTAATATTTCTAGGGGTAAATTTAACTTTTTTCCC
TCAACATTTTTTAGGATTAAAAGGAATGCCTCGTCGCTATTGTGATTATCCAGATTGTTATTCGATATGGC
ATTGAGTGTCTTCATATGGAGCACTGATTTCTTTTGGTTCGTTACTATATTTCATTTTTATTGTTTGGGAAG
CTATAGTAAGCCAACGTGGGGTGTCATTATTTGAGGCCTTATTTTTTGGTGATGTATTACAATCAGAGTTT
GGTTCTGAGATTGTAAAGTATTATGGTTATGTAATAATAGTGCTGTTACTCGTAATTATTGTTGTCTTATATA
TAGGGACTATAATTATAAATCATAGGTATTCCTATCGAAATTTCAAAAATCGGCAGATATTAGAGTATGTGT
GGACTGCTATACCTACTTTTTTGTTAGCGATGCTTTGATGCCCATCTATTTTAAATCTTTATCGAATAGAGG
ATTTAAAAGATCCTGTTTGAAGGTTTAAAGCTGTGGGTAAACAATGATATTGGACTTATGAAGTAAATTT
AAAAGACGGAGAAACTATAGTGATTGATTCTTACATAGATCGGGATTCAGGCGTTGAGTTAGAGTCTGG
TTATCGTCTCTTAGATGTCGATTGGCGTTTGGTAGCTCCTGCTGATGCACAAATTTCTTGTTATGTGACAA
GAAGTGATGTCATTCATTCTTTTGCCCTTCCTGGCGCATTGCTAAAAGCCGATGCTATTCCTGGCCGAATT
AATGTTCTCCCTATAAAGATTAGCCAAAGTTGTATTTTGTATGGGCAATGCTCAGAAATTTGCGGGATCA
ATCATAGGTTTATGCCTATTGTTATTGAGTTTGTTCCGGTAGATGTTTTTATAAAATTTTATGGGTACAATTA
AATGCATCGAAATCCTTATTATGTGCCTGGGCCTAGACCTTGGCCTTTAGTTGTGGGCGTATGCTGTAATA
GTTTATGCGTAAGCTTAGTATTATGAATACACCGAATACAAGAAAAACTGTTTTTGACATTTTTGGTTTTA
GGTTTAACTGCTACTTTATGGTGAACTGATGTGCTTCGTGAGGGGGATATGGGGGTTCAAACTCAGTATG
TTTTAAAAAGATACCGAGATGGTATAGGTCTTTTTATTTTTTCCGAAATTATATTTTTCTTTTCCTTTTTTTG
GGCTCTTTTTCATAACTGTCTAAGCCCTTCTGGCAACATTGGGTGTGAGTGGCCTCCTCTAGGAATTCGA

ACCCTTGATCCAATATCAACGGCGTTATTTAATACTTTTTTACTAATTAGAAGCGGTTCATTTTGTACTTAT
GTCCATAATACAATTCGTAAGTGTTATGATAGATGGTGTCTAATTAATATATTACTAACAATCACGTGCGGA
GTTCTTTTTTTACTAGGACAAGGAAATGAATATTTTTTCTGTTCTTTTAGGATCGCTGATAGTGTGTATGG
TAGTACTTTTTATATATTAACAGGGTTCCATGGAGGGCATGTCATAGTGGGAACAACTTGATTAATCGTTA
GATTTTTCCGAATATGACGTGGGCACTTTAGTAAGGATCGTCATTTTGGATTGGAAGCGTGTCTATGATAT
TGGCATTTTGTTGATGTAGTTTGGCTTTTTGTCTGGTTAATTGCATATTTCTGATTAGGAGGACCTTTTCGT
AAGCGTCATTGATTTTTAAAGATTCTAAATAACAGGCTTTATGACCTCCCTTGTCCTGTAAATTTGAATGC
TTTTTGGAGATTTGGATCCATATTAGGTTTGTGCATTGTAATTCAAATGGTTAGGGGAATTTTGTTATCTCT
ACACTTTATTCCGCATGAGAGGATAGCATTCGATTCAGTGTACCATATTATACGGGATGTGAACAAGGGA
TGGTTTCTTCGTAACATGCATGTTGGTGGATGTTCAATATTTTTTATTTGTCTTTACGTTCACATTGGTCGT
GGTATTTACTATGGGTCTTATATAAGACGACATGTGTGGTTTGTGGGAGTAACTCTGTTTTTAATAGTAAT
AGGGGAAGCTTTTCTAGGGTATAGGTTACCTTGGGGTCAAATATCTTTCTGGGGAGTAACAGTAATTTCT
AATTTGTTTACGGTAATTCCTTATGTGTCGCAAAACTTATTATTCACCCTTTGGGGGCATTGAAGAGTGAG
GGGGTATACTTTACAACGTTTCTTCATTTTTCATTTTTTACTTCCATTTGTAATTATTGTGTTTTCTACTCTA
CACATATTTTTTCTTCACGAAAACGGAAGAAATAACCCTTTAGGAGTTAATAGGGATTCTATATGTATTCC
TTTTCATCCTTTTTATACAGTAAAAGACATTTTTGGTTTTGTTTGTTTTGGGTGAGTATTAATATATCTGGT
GTGTGTTAATCCTGAGTTAGTAGTTAATAAAATTAACTATCATCCGGCTGACCCATATCACACTCCTCTTC
AGGTAGAGCCAGAGTGGTATTTTCTATTTGCCTATGCAATGCTTCGATCTATTCCTCATAAGTTAGGGGGA
GTTTTAGCTCTGTTAGCGTCTGTGTTAGGTTTATATTTATATCCTTTCATTCACACAGGAAAATTCCGGAG
CTTAGCATTTTACCCAGTTAGTCAAATACTTTTTTGATGTTTTGTAGTTAATTTCTTGAGACTTATTTGAGT
TGGGCAAATACCCGTTCGTGAGCCTTATATCACAATAGGACAAGTTTATACAGGAGTGTATTTTTCTACGT
TAATTCTTCCTCCATTATTAACAGGATTGTGAGATAAGTTAATTTTCCTGTATTTTGTTAGAATTATTCTTCC
TTTCGTTTGCGTATTGGTAAGAGTAGCTTTTTACACTCTGCTAGAGCGAAAAGTGTTAGGTTATATTATGA
TCCGAAAAGGGCCTAATAAGGTTGGATACGCTGGAATTATACAGCCTTTTAGGGATGCTGGGAAACTTTT
TAACAAAGAATACGTAAAGCCTGGCTATGCCAATTTATTGCCTTTTCTTTTGTGTCCGGCTATTATTTTGG
TAACGAGTCTGCTTTTGTGGTTATTATACCCTTATGATTTTACCTCTGTAATATTAACTTGTGGTTTGATGC
AGTTTTTAGTTACCTCTGGCATGCATGTGTATGGGGTAATAGTGGCTGGTTGATCTTCTAATTCTAAATATT
CCTTGTTAGGGGCAGTGCGAGGGGTAGCTCAGAGAATTTCATATGAAGTACCGATGACTTTTGTTATTTT
GATTGTAGCGTATTCCTTAAGAAGATTGTGGATTCAAGAAGTTAAGAATATTTTTTCTATAGCTATAGGGC
TATTAAGAAGTGGGGTGTGGTTAACGTGTATGTTAGCTGAGACTAATCCTGCTCCATTCGATTTTGTAGA
AGGGGAGTCTGAGTTGGTTTCTGGTTTTAATGTAGAGTACAGAAGGGGAGGATTCGCAATAATTTTTATG
GCTGAATATGGTGCTATATTATTCCATAGAGTATTTTTTGTAACTCTTTTTTTGGGAGGGGGTGAGTTACTA
GTGAGAGTGAGGACTATAATTTGGGTGCTATTTTTTATCTGAATTCGTGGAACTGTCCCACGAATTCGGT
ATGACCAGTTGATGGGGTTGTGTTGAAAAGTGTTGCTTAGAGTAATACTAAGGATAAGTGCATTAATTTT
AGTTGTAAGATTTTTCTTATGATAGGAGTTGTATTAAGTCCTCTATTGATTTTTTGTGGAATATTAGTAATTT
TAGGGAACTTTCTCAGAATCAGGAGTAGCTCATGATTAGGGGTATGGTTAGGAATAGAGATTAATCTTTT
TGGGTTTTTAATCATGATAAATCCTGAAGGTTGTTGTGTTGCTCAAAGAGGGGTAAAGTACTTTGTAACT
CAAAGAATTGGGTCTATACTTCTTCTTTTCGGGTTTTTTTTGGTGTTTTTATTTAATAGGGTGGGCAGCGT
AATTATTACAACAGGTCTGCTAATGAAAGCCGGCATTTTTCCTTTCCATAATTGGGTTCCAGATGTCGTAA
TAGTTTCAAGGTGGCCTATTGGGTGTCTTATTTTAACTTGACAAAAGCTAGCTCCATTTTCCTTTTTTTCA
TTTTTCCCAGGTTCATGGCTTTTAATTATTAGGTTAGTTTTTATAAGGATAGTAGGCAGTTTTGGAGGCTTA
AACCAGAATTCTGTTCGGGGGATAGCGGTTTATTCTTCTTTTGTTCATAATTCTTGAATAATACTTTCTCTC
TTTTACTCGTTTAGTATTTTTTTTTATTTATTATTTAATTTATAGAATAAGGGTTTTTCTTTTTTTCTTTAGGTG
TTGAACCTTAAGAAAGAGGAGGATGATAAGGTATTCAATCAGCTGAATAGGAATATTAAGACTACTAATG
CTTTCAGGTGTTCCTCCTTTTATAGGATTTTTTATAAAACTTATTGTGGTTTTGAGATGTCCCGTTATGTTA
ATAACTGTTTGTCTTTTAAGGTCAGTGATTAGGTTAAAATTTTATATGTCATTTTTTTATAGAATAATTTTGA
ATACTATGTATTGTGTAAAATTGTTAATGACATTTAGGTTAATGAACTTTATCGTGGGAGGAATTTCTATAA
GGTACTTTATTTGATAGATGATTGAATGTGTAACTCTTGTGTTTCTTATTGGACTGAGTTTGATTTTGATAG
TAATTGTTTTGTTATCTGAAAAGTCTTCTGAAAGCCGTGAAAAGTCATCTCCTTATGAGTGTGGGTTTGA
TCCTATTTTAAGAGCCCGAAGGTCTTTTTCTCTACGATTTTTTCTTCTCGCAGTATTGTTTGTTGTATTTGA
CGTGGAGTTATCTTTATTAATGCCTGTAGTAACAAGGTTAATAAGCGGGTTTAATTATATTAGGTTCATTAG
TTGTTTTTTATTTTTAACTATCTTGTTTTTGGGCATTTTTCATGAGTGGCGGGAAGGGTCTTTAAATTGAG
TGGTTTAAGTGGCCGTAAGTTTAACTATTACTTTGATTATACTCTTTGTCTTAGGTAATAAGGCAATAAGG
TTGATAGTCCTTCTTTTCTTATTTTTAACTGTCTTAGAACTAGGGATGGTTAACGATGAATTTTTAGCTTTA
GGCGGGAGGATAAGATATGACTTTTTATCTTATAGCTTAAGTGCTTTAAGAGTATTTATTGTATTACTAAGG
CTAATGAGTAGTAAAAAATGTTCAACGAGTTAAATTATTCTACTTTATGAATTTGGTACTACTAATGGTTTT
GGTCTTAGCTTTTTGCACCTCCAGTTTTTTAGGTTTTTTCTTTTTTTTTGAGTCCGCTTTAATTCCTCTTAT
TCTTATTATTATAGGCTGACGGATAGAGGCAAGGACTTATATATTTATTTACACGGTGTTTGGATCTCTGTT
TTTTTTATTCGGGGTGTCCCTGTTATTATATAGGGGAAGTGATAATATGTTATTGGCGAGTAATGTAAATAA
GAAGATCAGAGGATTTTGATGGTTATTCGTACTAGGATTTTTAATTAAATTGCCTGTTTACCCTTTTCATCT
ATGGTTACCAAAGGCTCACGTGGAGGCTCCCGTGGCAGGTTCAATGGTCTTAGCGGGCGTTGTGTTAAA
ACTGGGGGGTTATGGATTATTACGGTTAATAACCGTGATAAGAGTGAATCTAGGATTAAAGTCCAATGTA
ATATTAAGAGTAGGGATAGTAGGAGGAGTATATGCAAGGTTGGTTTGTTGTCGGCAAACGGACTTAAAAT

GTTTGGTTGCCTACTCCTCCGTAGGGCATATAAGGTTAGTAATGTTATGCTTGAGAAATTTAAGAATAGGA
GTTAAAGGAGCTATTTTCATTATAGTGGGGCATGGGTTGTGTTCCTCTGGAATGTTTAGTTTGGTAAACGT
TTTTTATAGTCATTCAGGATTCCGAAATTTGTACATAAACAAAGGTTTTTTAATTAAAAGTCCAATTTTATG
TCTTGTAAGGTTTCTTTTATGTTCTAGAAATATAGCAGCTCCGCCTAGTTTAAATTTATTTGGAGAAGTTTT
AATATTCGTATGCTCGTACCTTATTTCATTTTGCTTTTTAGTACTACTAATGGTAATAACAATCATTAGCGCT
ATTTATAGGCTTCATTTGTATACGAGAACTTGTCACGGGAAGGTTTCAGAGGCTCAAACTAGATTGGAGA
CTTATAGGTCTATTTTTGTTCTTTTAGCACATTGGATCCCGTTAAATTTCCTGTTTTTATTTATACCTTAGAC
GAAAAAAAATCATTTAATCAGGGTTTTTATTGGGATGGAGTTTATAATGTTGAGAATTCTATATTGTTGTA
GTACATTGGTGTTTTATAACGTTAGAATAATTTTTTTAGTAATGTGTTTAGGGGTCTGTGAAGCAAGTGTT
TGTCTAGCTCTTTTAGTAATGATGGTGCGGCTTTCAGGGAATGATTTAATTTCTAGCCTTTCTCTGTATAAtt
taataatttttctcaaaaaATGCTAGTAATAGCGGTATTGGGGTATTTATTTGTGCTAATAGGGAGTTTTGAGAGTGTT
TATTTTCTAGAGTTCATGTTGTGAGACTCAAATAGATTAAGCTTTTCTGTAAATGTACTTTTAGATTCTTTA
AGTTTAATATTTCTGGGGACTATTTTAATCATTTCGGGGAGGGTATTGATATATTGTTATTGGTATATAGATG
ATGAAATCTATTTTTTTCGTTTCATATATCTTGTTTACTTATTTGTAGGGTCAATAATATTTATAATTCTTATT
CCTAATCTTGTTACTCTTTTGATTGGTTGAGATGGACTGGGGTTAACTTCTTTCCTACTTGTTGCACACTA
TCAGAATAATAAATCTCTATCTGGAAGACTATTAACAGCTTTAACTAATCGAGTTGGAGATGGTTTAATTT
TGTCTAGAATTTCATTGTGGGCAAAGGAAAGAAGCTGGGTAATTTATAATTTTGAGAGTCAATTTGTAAA
CTTTTTTATTTTGAGTTTGGTTTTGGGGGGTATAACTAAAAGAGCACAAATGCCTTTTTGTGCTTGGTTAC
CAGCAGCTATAGCTGCTCCTACTCCAGTATCTTCTTTAGTTCATTCTTCTACACTAGTGACAGCTGGGGTA
TATTTGCTGATCCGTTCTTATCCTGTAATTAAAAAAAGCTTAATAACTAGTTTAAAGTTCTTGAGGCTGTT
TACATTGGTTTTAGCTAGCAGAACAGCAATTTACTGTTTTGACATAAAAAAAATTATTGCTTTGTCAACTT
TAAGTCAACTAAGGGTAATAATATTCTCACTGTCTCATGACTTAGTATCGGTTTCTTTTTTTCATTTAGTAA
TACATGCTTTGTTTAAAGCTCTTTTATTTTTGACAGCTGGTGCAGTTATTCATTCTTTTAAAGGGTGCCAG
GACATTCGAAGAATGGGAGGGTGTTGGAGAGTTCTCCCCTGTAGGATAGTGTGTATATTTATTGCTAATT
GTTCTTTGTCAGGTTTACCATTTTTAAGAGGGTTTTACTCAAAAGATATAATTTTGGATATGTTCAATAGA
AGAAATTTTTGGTTTATGATCATCATACCTGGTCTTATACTCACGAGGTTTTATAGAATGCGGGTTTGAAT
GGTTTGTTTTGGAAGAAATAAAGTTCCAATTAGAACTCTTTGTGTTCAGGAGTCAGTGAAATTACTCATA
CCTTATTTGCTTCTTGCTTTTGGCGCTGTATTTCTAGGAGGAATTTTAACCCCAAATGAGTGTATATTTAGT
TATCGAAGCTCATCAGCAGGAGAAATAAGGTTATTGAGAGTTTTGTTTGGGTTTGGGGTTCTGTTTGCTA
GAATTTTAAGAAATTATGGTAAAAAATACAAGACCTTTTCATTTTTGTTTAGAATATGGTTCTTAGAGTTA
ACTCATGGGGTGAAAAAGGGCTTTTTCAAAATAAGAGAAAATATTTTACAGAGATGTGATAAAGGGATA
CTAGAAACTGTTGGGCCTAAGGAATTCTGTATGAAATTAAGGTCTTATAATGAAGATTATTTTTTACTTTC
TTTTTTCTTTATTGGACTTCTTTCTGTGTTCTTTTTTTTGTAATATGCAACTTCTTATGATTTATTGGGCCGTC
ATCATAATATGTGTGATTTTTATTAGCCAGCCTTATTTATTAGGTTTAGTACTAATGCTAACTTCTATATTTAT
TTGTATAATTGTTGGAGTAAACGTTAGGAGATTTTTAGGGTTTCTTCTGTTTATAAGATATGTAGGGGGTT
TGCTGGTTCTTTTTGTTTACGTATTAAGGGTTTTTCCAAATGAAGATTTTAAGATAAGAAGGTTTTTACTG
GTAAGAAGAGGTCTAATATCTTTTCTTTTATTTCCAATTCATAGATTAAGAAAATCTCTTTTTtttCATTATAT
AGGGTTTATTGGAGTTTTTGTGTTTATGGCTCTTTTTCTTTTGTTTATCATAATATGTGTGTCTTATCTTGTG
ATAAAAAAACGAATACCGTTGCGTGTGAAT"/>
<sequence id="seq_Perna_viridisJQ9704251" spec="Sequence" taxon="Perna_viridisJQ970425"
totalcount="4"
value="ATGTTAATAGATGTTTTTTCTATGTTTGATGATTATAATTTTAATACATTTAAAAGGTGGTATGTCTG
GGGATATTCATTGTTTATTCCTTTGTTTATCGTGAGATCATCATTAAGGATTTATTATTCATTTTTACAGAAA
TGTTTGAAATTGATTGGTAGGGGAAGGGGTTTGTATATTTCAGGTTTTCCTCTATTAGTTGTGAGTCTTTT
TTCAATTATACTAAGGGTAAATGTTTCTAATAGAATTCCTTATTTTTTTAGGGTGAGGGCACATTTTTCGTT
TGGCTTTACTTTTGCGTCTGTGATTTGGCTGTGTATTATTACTTCTAGGGTTTTTACAAGGTTTATTCAGAA
TATGGCTTTGCTGGTTCCAAGAGGTCCTGAGGGGTTAGCTCCATTTATGGTGCTTTTAGAGATTATTACAA
ATTTATTGCGTCCAATTACTTTAGTTATTCGATTAGCTATAAATATGGCTACTGGTAAGGTTATTATGTTATT
ATTAGGGAATGCTGCGTTAAATCTTGCATTTGTTGGGGTTGTAGGGTTTATAGTTGTTAGAGTGTTAGGTG
CTATTTTTTCTTTAGAGGTGGCTGTTAGGTTTATTCAGAGATATATTTTTTGTACTCTATTATGTTTGTATGC
TGATGAACATAGGAAATAGcgatggtttATGTCTACTAATCATAAAGATATTGGTACTTTGTATTTACTTTCTGG
GATATGAGCTGGGTTAATGGGGAGAAGGCTTAGGTTAATTATTATTCAGTCTCATCCCGGGGGTAATTTTT
TGAAAGAAAGGTTATATAATGTTGTAGTAACAACTCATGCATTAGTAATAATTTTTTTGCTGTAATGCCTT
TACTTATTGGTGCTTTTGGGAATTGATTACTTCCATTATGTATTGGTGGTGTTGATTTAATTTTTCCTCGTTT
AAATAATTTGAGATTTTGGTTGGCACCTAATGCTTTGTACTTACTTATTTTGTCGTTTATAACGGAGAAAG
GAGCTGGGACAGGTTGAACTATTTATCCACCTTTATCTTCTGGGTTGTACCATACTGGGCCTGCTGTTGAT
ATTTTGATTACGTCTTTACATTTAATTGGATTGAGTTCTTTATTAGGTTCGATTAATTTTGTGAGGACTAAT
AAGAATATGCCTACAATAAAAATAAAGGGTGAGAAATCTGAGTTGTATTTGTGGAGGATTACCGTAACCG
GTGTTCTTTTAATTATTTCTGTGCCAGTTCTGGCTGGTGGGATTACTATATTGTTGTTTGATCGAAATTTCA
ATACTAGGTTTTTTGATCCTATTGGAGGGGGAGATCCTGTTTTATTTCAGCATGTATTTTGGTTTTTTGGTC
ATCCTGAGGTGTACATTCTTATTCTTCCGGCGTTTGGTGTGATGTCAAAAGTAATTATGCATTATTCTGGT
AAAGATTCTGTTTTTGGTTCGGTTGGGATATTATATGCTATAGTTGGTATTGGTGTTATAGGTTGTGTGGTG
TGGGCCCATCATATATTTACTGTGGGTTTAAATGTAGACACACGAACTTATTTTACATCAGCTACAATAGT

AATCGCTGTTCCTACGGGTGTGAAAGTTTTTAGTTGGATGGCTACAATAGGAGGAAGAAAAATTAAGTT
AACTACATCTGTTTTGTGAAGGACTGGTTTTTTATTTTTGTTTACTGTTGGGGGACTTACGGGAATTATAC
TTTCTAGTTCTTCTTTGGATGTAACATTACATGATACATATTATGTAACAGCACATTTCCATTATGTCTTGTC
GATAGGGGCTGTATTCGGTATTTTTTGTGGGTTGAACCATTGATTTCCTTTGTTTTATGGTGTAAACTTTC
ATAAAAAGTGATCTAAAGTTCATTTTTATTTAATATTTTTAGGAGTGAATTTAACATTCTTTCCCCAGCATT
TCTTAGGATTAAAAGGGATACCGCGTCGTTATTGTGATTATCCAGATTGTTATTCTACATGGCATTGGGTAT
CTTCTTATGGTGCTTTAATTTCTTTTGGGTCTTTGTTATATTTTATTTTTGTTGTATGGGAGGCTATGGTGAG
TCAACGAGGTGTGTCATTGTTTGAGGCTATATTTTTTGGTGATGTATTAGAGGAGTTTGGTTCTGAAATTA
TTAAATATTATGGTTATGTGATAATAGTTTTATTGTTGGTTATTATTGTTGTTTTGTATATGGGAATAATGATT
ATGAATCATAGATATTCTTATCGTAATTTTAAAAATCGACAAATATTGGAGTATGCTTGAACTGCAATACC
AACGTTTCTTTTGGCGTTATTATGATGTCCTTCAATTTTGAATTTGTATCGGATAGATGATTTGAAGGATCC
TGTCTGAAGGTTTAAGGCTGTTGGAAAGCAGTGGTATTGAACTTATGAGAATGGGGATACGATGGTTATT
GATTCTTATATAGATCGTGATTCTGGGGTGGGTTATCGATTACTTGATGTTGATTGGCGTTTAGTGGCTCCT
GCTAATGCGCAGATTTCGTGTTATGTTACAAGGAGGGATGTAATTCATTCTTTTGCGCTTCCGGGTGCGTT
GATAAAAGCGGATGCTATTCCTGGACGGATTAATGTACTTCCTATAAAAATTAGTCAGAGTTGTATTTTAT
ATGGGCAGTGTTCAGAAATTTGTGGGATCAATCATAGATTTATACCTATTGTTATTGAGTTTGTTCCCGTT
GATGTGTTTATGGATTTTTATGGGTATTAGaattaa------------------------
ATGCATCGAAGTCCTTATTATGTCCCAGGGCCAAGGCCTTGGCCATTAATTGTAGGTGTGTGTTGTAATAG
GTTATGTGTAAGTCTTGTGTTGTGGATACATCGAATACAAGAGAAACTTTTTGTTACATTATTGGTGTTAA
TTATTACTGCGATTCTGTGATGAACAGATGTTTTGCGTGAAGGGGATATGGGTGTGCAGACTCAGTTTGT
AATTAAGAGATACCGAGATGGAGTTGGCCTTTTTATCTTTTCTGAAATTATATTCTTTTTTTCGTTTTTTTG
GGCACTTTTTCATAGTTGTCTAAGTCCTTCTGGAAATCTTGGGTGCGAGTGACCTCCTTTAGGGATTCGG
ACGTTAGATCCGTTATCTACTGCCCTATTCAACACTTTTTTGTTAATTAGAAGCGGGTCATTTTGTACTTAT
GTTCACAACTCAATTCGAACAGGTTATGATTCTTGATGTCTGATCAATATGGTAATTACTATCTCTTGTGG
TGTAATATTTTTAATTGGGCAAGGTCATGAGTATTTTTTTAGTCCTTTTAGAATTGCGGATAGAGTGTACG
GAAGAACTTTTTATATGCTAACTGGGTTCCATGGTGCTCATGTTATGGTAGGAACTACTTGGTTAATTGTT
AGGTTGAGTCGGATGTGATTAGGCCATTTTTCTAAAGACCGTCATTTTGGATTGGAAGCTTGTTTATGATA
TTGACACTTTGTTGATGTGGTGTGGCTAGGGGTATGACTAATTGCTTATTTTTGGATGGGGGGACCTCTT
CGGAAACGTCATTGATTATTGAAAATTTTGAATAATAGGTTATATGATTTACCATGTCCAATTAATTTAAAT
ATATTTTGGAGATTTGGTTCTATGTTAGGTTTGTGTATTGTTATTCAGGTAGTTAGTGGAATTTTATTATCTC
TTCATTTTATCCCTCATGAGGCAATAGCTTTTGATTCTGTCTATCATATTATGCGTGATGTGAATAAGGGTT
GGTTTTTGCGGAATGTTCATGTTGGTGGGTGTTCTATGTTCTTCGTTTGTTTGTATGTTCATATTGGTCGTG
GGATTTATTATGGGTCTTATTTAAGTAAGCACGTTTGGTTAGTGGGTGTGACTCTGTTTTTGTTGGTGATG
GCAGAAGCATTTTTAGGGTATAGATTGCCTTGGGGTCAGATATCGTTTTGAGGTGTAACTGTGATTTCTA
ATTTGTTTACTGTGATCCCGTATATTTCTCAAAATTTGTTGTTTACTATTTGGGGGCATTGAAGCGTTAGG
GGGTATACTTTACAGCGATTTTTTATTTTTCACTTTCTTCTTCCTTTTGTCATTATTGTTTTTTCGACTTTGC
ATTTATTTTTTCTTCATGAGAATGGGAGAAATAATCCTTTGGGTATTAGAAGGGATTCAATATGTATTCCTT
TTCATCCATTTTACACTGTCAAAGATATTTTTGGGTTTGTTTGTTTTGGTTGAGCTTTGATATATTTTGTTT
GTGTAAATCCTGAACTGGTAGTAAATAAAATCAATTATCATCCAGCAGACCCTTATCACACTCCTTTACAG
GTAGAGCCGGAGTGATATTTTTTGTTTGCTTATGCTATGTTACGTTCAATTCCTCATAAGCTTAGTGGGGT
GCTTGCCTTACTTGCTTCAGTAACTGGATTGTATCTATATCCTTTTATTCATACTGGGAAGTTTCGTAGGTT
TGCATTTTATCCCGTAAGACAGATGCTGTTTTGGTGTTTTGTTGTAAATTTTTTGAGGTTAATTTGGGTTG
GGCAAATACCTGTGCGAGAGCCTTTTATTAGAATAGGTCAGGTATATACTGGTATTTATTTTTCGACTTTG
ATTTTTCCTCCTATGTTAACTGGTCTTTGGGATAAGTTGATTTTCCTTTATTTTGTAAGGTTGATTCTTCCG
TTTGTTTGTGTTTTGGTGAGGGTTGCTTTTTATACTCTTGTTGAACGAAAGGTGTTAGGTTATATTATGATT
CGTAAGGGGCCTAATAAGGTTGGGTATAGGGGAATTATGCAGCCTTTTAGGGATGCAGGGAAGCTTTTTA
GAAAAGAGTATGTGATACCGGGATTTGCTAATGTTGTTCCTTTCGTTTTGTGTCCTGCTGTTATTCTTTTT
ACTAGAATGATGTTATGGTTTCTTTATCCTTATAGTTATGTTTCGATAGTGTTTACGTGTGGGATTGTTCAG
TTTTTAGTAACATCTGGTATCCATGTATATGGAGTTATAGTAGCTGGTTGGTCTTCTAATTCAAAGTACTCT
CTTTTAGGGGCTGTTCGGGGGGTAGCACAAAGAATTTCTTATGAAGTTCCTATAACTTTTGTGGTTTTGA
TAGTAGCGTTTTGTATTGGGAGATTATGGTTGCAGGAGGTTAAGATAATATTTTCTATTCTTATAAGATTAT
TGAGGAGAGGGGTTTGAGTAACTTGTATGTTGGCTGAGACCAATCGCGCTCCGTTTGATTTTGTTGAGG
GGGAGTCTGAGTTAGTATCAGGGTTTAATGTGGAGTATAGAAGAGGAGGTTTTGCAATAATTTTTATGGC
GGAGTATGGGGCTATATTATTTAATAGGATTTTTTTTATTACTATTTTTTTAGGCGGTAATGAGTTATTAATG
AGGGTTAGAACAATAGTATGAGTTCTTTTTTTTGTGTGAATTCGGGGAACAGTTCCCCGAATTCGATATG
ATCAGCTTATAGGATTGTGTTGGAAAGTTTTATTAAGGGTAGTTTTGAGAATAACTGGATTTGTAGCTATT
ATCAGATTTTTTTTatgataggaATAAGAGTTAGACCTTTGTTAAGAGCTTGTGGGTTATTAGTTGTATTAGGTA
ATTTAGTAAGGATTATAAGAAATAGATGAATGGGTGTGTGGTTAGGAATAGAACTTAATTTATTTGGCTTT
TTAATTATAATAAATCCTGAAGGGTGTTGTGTTGCTCAGGGAAGAATCAAGTACTTTATTGCACAAAGGA
TTGGTTCTATGTTAATATTGTTTGGGTTTTTTTGTGTGTCTATGTTTAATAGAGTGGGGAGGTTAATTCTTG
TTGGGGGAATTCTTTTAAAAGCGGGGGTTTTTCCTTTTCATAGATGAGTTCCGGACGTTGTTATTGTGTCT
AATTGGTTTATTGGGTGTATAATTTTAACATGGCAAAAGTTAGCTCCATTTTCTTTGTTTTCGTTTTTTCCT
AGTTCTTGGGTCTTGGTAATAAGATTGATTTTTATAAGGTTGGTGGGATGCTTTGGGGGATTAAATCAAC

ATTCAGTTCGTGGAATGGCAGTTTATTCTTCTTTTGTTCATAACTCTTGGCTGATTCTTTCTTTATTTTATTC
CTTTGGTGTTTTTTTATTTATTATTTGGTGTATAGGTTGAGGGTTTTAATATTTTTTTTGAGGTGTTGGGTA
GTTAGAAAAAGGAGCTTAATAAGGTACTCCGTTAGATGATTGGGAATATTAAGTCTATTAATGTTATCTGG
TGTTCCACCTTTTATGGGGTTTTTTGTTAAATTGATTGTGATGTTAAGATGCCCGTCTATTCTTTTGATTAT
TTGTTTGGGGGGATCTATCGTAAGGCTAAAGTTTTATATTTCTTTTTTCTATAGAATGATTTTGAATTCTTAT
TTTCAATTGGAGTTGGTTTATATTTTTTGTAGGATGAATTTTGTAGTAGGGGTTAGTTCGTTATTGTTTTTT
ATATGGTGTATGATTGAATGTTTTCTTTTGTTTTTTCTTTTTATTTTAAGGATGGTGTTAGTACTAGTATTTT
TATTATCAGAGAAAACATTTGAGAGACGTGAGAAGTCGTCCCCTTATGAGTGTGGGTTTGACCCTATTTT
AAGGGCGCGAAGGTCTTTTTCTCTTCGTTTTTTTCTTTTAGGGGTCTTATTTGTTGTTTTTGATGTTGAGT
TGTCTTTGTTAATACCTGTGGTTCTTAGGATTAGGGTGGGGTTTAGCTATGTGGGGCTTATTAGATGTTTT
ATCTTTCTTTTAGTTTTGTTTTTAGGAATTTTTCATGAGTGACGAGAAGGGTCATTGAACTGGGTTATTTA
GGTGGCAGTAAGTATAATATTAGTATGAATCTTTCTTTGTATTGTTAGAAATAAAATGTTGAGGCTTTTAA
GTTTGGCTTTGTTAATAGTAGGGGTTATAGAGATAGGAGTAATCAATGATGAGGTAATTAGAATGGGGGG
TAGTCTTAGATATGATTTTTTATCCTATAGGTTAAGGGTTTTGAGGATTTTTATTATATTGTTAAGCTTAGTT
AGAAGAAAAAGGGTAATACGAACTTCTTTGTTTTATTTTTTAAATTTAAGATTATTAATAGTGTTAGTTTTT
GCTTTTTGTACTTCTAGTTTTCTAGGGTTTTTCTTTTTTTTTGAGTCTGCTTTGATTCCTTTAATTTTGGTTA
TTATAGGGTGACGGATAGAGGCAAGAACTTATATGTTTATCTATACGGTATTTGGTTCTCTTTTTTTTCTTGT
TTGGGATTTGTCTTCTTTTTTTATAGAGGAAGGGATAATATATTATTAGGAAGGAATGTGAATAAAAAGGTT
AGAATGTTTTGGTGGGTTTTTATGATTGGGTTTCTAATTAAGTTGCCTGTATATCCTTTTCATTTATGGTTA
CCTAAGGCTCATGTAGAGGCTCCTGTTGCTGGATCAATGGTTTTGGCTGGGGTAGTATTAAAACTGGGTG
GATATGGGATATTGCGGTTGTTAATAGTAATAAGTGTAAATTTGGGGTTAAAATGTAATCTGATACTATGC
GTAGGGATAGTTGGTGGTTTTTATGCAAGATTGGTATGTTGTCGTCAGACGGATATAAAATGTTTAGTAGC
ATACTCTTCTGTAGGTCATATAAGGTTGGTTACTTTGTGTCTAAGAAACATTAGAATAGGGGTAAAAGGT
GCTTTGTTTATTATAATTGGGCATGGGTTATGTTCTTCTGGGATATTTAGCCTTGTTAATGTTTTTTATAGTC
ATTCTGGTTATCGAAATCTGTATATAAATAAGGGGTTTTTGATAAAAAGTCCAATTCTGTGTTTGGTAGGA
TTTTTATTGTGTTCTAGTAATATAGCTGCTCCGCCGAGGTTAAATTTGTTGGGTGAGGTGCTAATGTTTATT
TGTTCATATGTAATTTCTTTTTGTTTTCTGTTGTTGTTAATAGCAATAACTATAGTTAGTGCTGTTTATAGCT
TGCATTTGTACACATCTACATGTCATGGAAAAGGGTCAGAGAGGATGATGGGATGAGATATTTATAGATC
TGTGTTCGTTTTGTTAGCTCATTGGTTGCCTTTGAATTTTTTGTTTTTGTTTATACCTTagTTAAAAAAAGG
GCATCTTATTAGAATTTTCATTGGAATGGAATTTATAATGTTAGGGGTGTTGTATAGTGCTAGGATCATAAT
GTGTTATAATGTAAGAATAGTATTCTTAATTATGTGTTTAGGGGTATGTGAGGCAAGGGTATGCTTAGCTT
TATTAGTTATGATAGTGCGTTTGTCAGGAAATGATTTAATTAGAAGATTGTCTTTGTATaaTTTAGGAAAGT
TTATCAAAAAATATTATTCATAGTGGTTTTTGGGTATTGCTTTATTTTATTAAGAAGAGTGAAGAGTGTCTA
TTTTATGGAGTTTATGTTGTGGGATTTAGGGGGTATAGGATTGTCTATTAATTTATTGGTTGATAGAATTGG
GGTGATATTCATTGGGACTATCATAATTATTTCTGGTAGAGTATTAATATACTGCTATTGGTATATAGATGAT
GAGAAATATTTTTTCCGTTTTATATACTTGGTTTATTTATTTGTTGGATCAATAATATTTATAATTTTAATTCC
TAATTTAATTACGTTGTTAATTGGGTGGGATGGGTTAGGATTAACTTCTTTTTTGTTAGTTGCACATTATCA
AAATTCTAGGTCTTTGTCAGGGAGCTTGTTAACGGCATTGACGAATCGGATTGGGGATGGGTTAATCTTG
TTAAGAATTTCTTTGTGAGGAAGAGAAAATATGTGAGTTTTGTATAATTTCAGAGGATCTTTTATAAGTGT
CTTTATTTGTGCGCTTATTTTTGGTGGAATAACTAAGAGTGCGCAGATACCTTTTTGTGCTTGGTTACCGG
CTGCTATGGCTGCTCCCACTCCTGTGTCTTCTTTGGTTCATTCTTCCACGTTAGTTACAGCGGGTGTATAT
TTACTTATTCGTGCGTATCCAGTAATAAGAAAGAGAATAATATTAGTGTTAAAGTTACTAAGGTTGTTTAC
TTTGGTGTTAGCTAGAAGCGCAGCAATTTTTTGTTTTGATATAAAGAAAATTATTGCATTATCAACTTTAA
GGCAATTAAGTGTAATAATATTTTCTTTATCTCATGGGTTGGTTTATGCTTCTTTTTTTCATTTAGTAATGCA
TGCATTGTTTAAAGCACTTCTTTTTTTGTCAGCAGGGGTAGTTATTCATTCTATAAAAAGTTGCCAAGATA
TTCGTAGAATAGGAAATTGCTGAGCAAATATGCCTTTTAGAATAGTTTCTATATTTATTGCGAGGTGTTCA
CTTTCAGGTCTTCCATTTATAAGTGGGTTTTTTTCTAAGGATATAATTGTTGATGTGTATAATAGAAAAAAT
TTTTTCTTTTTTGTTATAATGCCTGGGTTAGGGTTGACTAGAATTTACAGAATACGAGTATGAATAGTAAG
GTTTGGAGAAAACAAAGTTATGTTAGGATGTTTGAGAGTAAAAGAGCCAATAAAACTTCTAATCCCTTAT
TTAAGGCTTGGTTTTGGGGCATTATTTTTGGGTAAAATAATATGTCCAATTACTGAGAGGTTGATTTATTA
CAGGAGTTCATCATTAGGGGAGATATTAGTTTTAAGAATTCTATTTAGGGGAGTATATTTAAGAGTAGCAA
ACTGGGGAAAAGGGCCATCTttcAAAAAGTCTTCTTTTTTATTTAGAATATGATACTTAGAGTTAGTACATG
TAATCAAAAAGATATTTTTTGACCTTAGAAAATCTATTTATGAAGTGTGTGATAAAGGAATATTAGAGACA
GTGGGGCCGAAGTGGATGAGAATAAAAATAAGATGTTATAACGAGAATTTCTTTCTTTTTTTTTTTGTGT
TTAGGGGAATAATTTTTATAATGAGAATTGTTATTATGGAATTTATTTATTTGCATTTGGTAGTAGTTAGATT
GTGTGTTGTTTTTGTTAGTCAACCTTACTTACTGGGGTTAGTTCTTTTAATTTCTTCAATACTAGTTTGTTT
AATTATTGGGGTTAACATTAGAAGATTTCTTGGATTTTTGTTATTTATAAGATATGTGGGAGGGCTCATAGT
TTTATTTGTTTACGTTCTTAGTGTATTTCCTAATGAAAATTTTAAAACGAAAACGTTTTTTATAAGGTTAAG
GTTTGGAGTTCTGGTAGTGATATTACCTGTAAAGTCGGGTGCTCAAGAACTGCAGTTTCATTTTATGAGG
TTTTCTCATGTTTTTGTTTTTATAGCTTTATTTCTTTTGTTTATTATATTATGTGTTTCTTATTTAGTAATAAA
AAAACGGGTTCCTTTACGATCAATC"/>
<sequence id="seq_Perna_viridisMW7275151" spec="Sequence" taxon="Perna_viridisMW727515"
totalcount="4"

value="ATGTTAATAGATGTTTTTTCTATGTTTGATGATTATAATTTTAATACATTTAAAAGGTGGTATGTCTG
GGGATATTCATTGTTTATTCCTTTGTTTATCGTGAGATCATCATTAAGGATTTATTATTCATTTTTACAGAAA
TGTTTGAAATTGATTGGTAGGGGAAGGGGTTTGTATATTTCAGGTTTTCCTCTATTAGTTGTGAGTCTTTT
TTCAATTATACTAAGGGTAAATGTTTCTAATAGAATTCCTTATTTTTTTAGGGTGAGGGCACATTTTTCGTT
TGGCTTTACTTTTGCGTCTGTGATTTGGCTGTGTATTATTACTTCTAGGGTTTTTACAAGGTTTATTCAGAA
TATGGCTTTGCTGGTTCCAAGAGGTCCTGAGGGGTTAGCTCCATTTATGGTGCTTTTAGAGATTATTACAA
ATTTATTGCGTCCAATTACTTTAGTTATTCGATTAGCTATAAATATGGCTACTGGTAAGGTTATTATGTTATT
ATTAGGGAATGCTGCGTTAAATCTTGCATTTGTTGGGGTTGTAGGGTTTATAGTTGTTAGAGTGTTAGGTG
CTATTTTTTTCTTTAGAGGTGGCTGTTAGGTTTATTCAGAGATATATTTTTTGTACTCTATTATGTTTGTATGC
TGATGAACATAGGAAATAGcgatggtttATGTCTACTAATCATAAAGATATTGGTACTTTGTATTTACTTTCTGG
GATATGAGCTGGGTTAATGGGGAGAAGGCTTAGGTTAATTATTATTCAGTCTCATCCCGGGGGTAATTTTT
TGAAAGAAAGGTTATATAATGTTGTAGTAACAACTCATGCATTAGTAATAATTTTTTTTGCTGTAATGCCTT
TACTTATTGGTGCTTTTGGGAATTGATTACTTCCATTATGTATTGGTGGTGTTGATTTAATTTTTCCTCGTTT
AAATAATTTGAGATTTTGGTTGGCACCTAATGCTTTGTACTTACTTATTTTGTCGTTTATAACGGAGAAAG
GAGCTGGGACAGGTTGAACTATTTATCCACCTTTATCTTCTGGGTTGTACCATACTGGGCCTGCTGTTGAT
ATTTTGATTACGTCTTTACATTTAATTGGATTGAGTTCTTTATTAGGTTCGATTAATTTTGTGAGGACTAAT
AAGAATATGCCTACAATAAAAATAAAGGGTGAGAAATCTGAGTTGTATTTGTGGAGGATTACCGTAACCG
GTGTTCTTTTAATTATTTCTGTGCCAGTTCTGGCTGGTGGGATTACTATATTGTTGTTTGATCGAAATTTCA
ATACTAGGTTTTTTGATCCTATTGGAGGGGGAGATCCTGTTTTATTTCAGCATGTATTTTGGTTTTTTGGTC
ATCCTGAGGTGTACATTCTTATTCTTCCGGCGTTTGGTGTGATGTCAAAAGTAATTATGCATTATTCTGGT
AAAGATTCTGTTTTTGGTTCGGTTGGGATATTATATGCTATAGTTGGTATTGGTGTTATAGGTTGTGTGGTG
TGGGCCCATCATATATTTACTGTGGGTTTAAATGTAGACACACGAACTTATTTTACATCAGCTACAATAGT
AATCGCTGTTCCTACGGGTGTGAAAGTTTTTAGTTGGATGGCTACAATAGGAGGAAGAAAAATTAAGTT
AACTACATCTGTTTTGTGAAGGACTGGTTTTTTATTTTTGTTTACTGTTGGGGGACTTACGGGAATTATAC
TTTCTAGTTCTTCTTTGGATGTAACATTACATGATACATATTATGTAACAGCACATTTCCATTATGTCTTGTC
GATAGGGGCTGTATTCGGTATTTTTTGTGGGTTGAACCATTGATTTCCTTTGTTTTATGGTGTAAACTTTC
ATAAAAAGTGATCTAAAGTTCATTTTTATTTAATATTTTTAGGAGTGAATTTAACATTCTTTCCCCAGCATT
TCTTAGGATTAAAAGGGATACCGCGTCGTTATTGTGATTATCCAGATTGTTATTCTACATGGCATTGGGTAT
CTTCTTATGGTGCTTTAATTTCTTTTGGGTCTTTGTTATATTTTATTTTTGTTGTATGGGAGGCTATGGTGAG
TCAACGAGGTGTGTCATTGTTTGAGGCTATATTTTTTGGTGATGTATTAGAGGAGTTTGGTTCTGAAATTA
TTAAATATTATGGTTATGTGATAATAGTTTTATTGTTGGTTATTATTGTTGTTTTGTATATGGGAATAATGATT
ATGAATCATAGATATTCTTATCGTAATTTAAAAATCGACAAATATTGGAGTATGCTTGAACTGCAATACC
AACGTTTCTTTTGGCGTTATTATGATGTCCTTCAATTTTGAATTTGTATCGGATAGATGATTTGAAGGATCC
TGTCTGAAGGTTTAAGGCTGTTGGAAAGCAGTGGTATTGAACTTATGAGAATGGGGATACGATGGTTATT
GATTCTTATATAGATCGTGATTCTGGGGTGGGTTATCGATTACTTGATGTTGATTGGCGTTTAGTGGCTCCT
GCTAATGCGCAGATTTCGTGTTATGTTACAAGGAGGGATGTAATTCATTCTTTTGCGCTTCCGGGTGCGTT
GATAAAAGCGGATGCTATTCCTGGACGGATTAATGTACTTCCTATAAAAATTAGTCAGAGTTGTATTTTAT
ATGGGCAGTGTTCAGAAATTTGTGGGATCAATCATAGATTTATACCTATTGTTATTGAGTTTGTTCCCGTT
GATGTGTTTATGGATTTTTATGGGTATTAGaattaa------------------------
ATGCATCGAAGTCCTTATTATGTCCCAGGGCCAAGGCCTTGGCCATTAATTGTAGGTGTGTGTTGTAATAG
GTTATGTGTAAGTCTTGTGTTGTGGATACATCGAATACAAGAGAAACTTTTTGTTACATTATTGGTGTTAA
TTATTACTGCGATTCTGTGATGAACAGATGTTTTGCGTGAAGGGGATATGGGTGTGCAGACTCAGTTTGT
AATTAAGAGATACCGAGATGGAGTTGGCCTTTTTATCTTTTCTGAAATTATATTCTTTTTTTCGTTTTTTTG
GGCACTTTTTCATAGTTGTCTAAGTCCTTCTGGAAATCTTGGGTGCGAGTGACCTCCTTTAGGGATTCGG
ACGTTAGATCCGTTATCTACTGCCCTATTCAACACTTTTTTGTTAATTAGAAGCGGGTCATTTTGTACTTAT
GTTCACAACTCAATTCGAACAGGTTATGATTCTTGATGTCTGATCAATATGGTAATTACTATCTCTTGTGG
TGTAATATTTTTAATTGGGCAAGGTCATGAGTATTTTTTTAGTCCTTTTAGAATTGCGGATAGAGTGTACG
GAAGAACTTTTTATATGCTAACTGGGTTCCATGGTGCTCATGTTATGGTAGGAACTACTTGGTTAATTGTT
AGGTTGAGTCGGATGTGATTAGGCCATTTTTCTAAAGACCGTCATTTTGGATTGGAAGCTTGTTTATGATA
TTGACACTTTGTTGATGTGGTGTGGCTAGGGGTATGACTAATTGCTTATTTTTGGATGGGGGGACCTCTT
CGGAAACGTCATTGATTATTGAAAATTTTGAATAATAGGTTATATGATTTACCATGTCCAATTAATTTAAAT
ATATTTTGGAGATTTGGTTCTATGTTAGGTTTGTGTATTGTTATTCAGGTAGTTAGTGGAATTTTATTATCTC
TTCATTTTATCCCTCATGAGGCAATAGCTTTTGATTCTGTCTATCATATTATGCGTGATGTGAATAAGGGTT
GGTTTTTGCGGAATGTTCATGTTGGTGGGTGTTCTATGTTCTTCGTTTGTTTGTATGTTCATATTGGTCGTG
GGATTTATTATGGGTCTTATTTAAGTAAGCACGTTTGGTTAGTGGGTGTGACTCTGTTTTTGTTGGTGATG
GCAGAAGCATTTTTAGGGTATAGATTGCCTTGGGGTCAGATATCGTTTTGAGGTGTAACTGTGATTTCTA
ATTTGTTTACTGTGATCCCGTATATTTCTCAAAATTTGTTGTTTACTATTTGGGGGCATTGAAGCGTTAGG
GGGTATACTTTACAGCGATTTTTTATTTTTCACTTTCTTCTTCCTTTTGTCATTATTGTTTTTTCGACTTTGC
ATTTATTTTTTCTTCATGAGAATGGGAGAAATAATCCTTTGGGTATTAGAAGGGATTCAATATGTATTCCTT
TTCATCCATTTTACACTGTCAAAGATATTTTTGGGTTTGTTTGTTTTGGTTGAGCTTTGATATATTTTGTTT
GTGTAAATCCTGAACTGGTAGTAAATAAAATCAATTATCATCCAGCAGACCCTTATCACACTCCTTTACAG
GTAGAGCCGGAGTGATATTTTTTGTTTGCTTATGCTATGTTACGTTCAATTCCTCATAAGCTTAGTGGGGT
GCTTGCCTTACTTGCTTCAGTAACTGGATTGTATCTATATCCTTTTATTCATACTGGGAAGTTTCGTAGGTT

TGCATTTTATCCCGTAAGACAGATGCTGTTTTGGTGTTTTGTTGTAAATTTTTTGAGGTTAATTTGGGTTG
GGCAAATACCTGTGCGAGAGCCTTTTATTAGAATAGGTCAGGTATATACTGGTATTTATTTTTCGACTTTG
ATTTTTCCTCCTATGTTAACTGGTCTTTGGGATAAGTTGATTTTCCTTTATTTTGTAAGGTTGATTCTTCCG
TTTGTTTGTGTTTTGGTGAGGGTTGCTTTTTATACTCTTGTTGAACGAAAGGTGTTAGGTTATATTATGATT
CGTAAGGGGCCTAATAAGGTTGGGTATAGGGGAATTATGCAGCCTTTTAGGGATGCAGGGAAGCTTTTTA
GAAAAGAGTATGTGATACCGGGATTTGCTAATGTTGTTCCTTTCGTTTTGTGTCCTGCTGTTATTCTTTTT
ACTAGAATGATGTTATGGTTTCTTTATCCTTATAGTTATGTTTCGATAGTGTTTACGTGTGGGATTGTTCAG
TTTTTAGTAACATCTGGTATCCATGTATATGGAGTTATAGTAGCTGGTTGGTCTTCTAATTCAAAGTACTCT
CTTTTAGGGGCTGTTCGGGGGGTAGCACAAAGAATTTCTTATGAAGTTCCTATAACTTTTGTGGTTTTGA
TAGTAGCGTTTTGTATTGGGAGATTATGGTTGCAGGAGGTTAAGATAATATTTTCTATTCTTATAAGATTAT
TGAGGAGAGGGGTTTGAGTAACTTGTATGTTGGCTGAGACCAATCGCGCTCCGTTTGATTTTGTTGAGG
GGGAGTCTGAGTTAGTATCAGGGTTTAATGTGGAGTATAGAAGAGGAGGTTTTGCAATAATTTTTATGGC
GGAGTATGGGGCTATATTATTTAATAGGATTTTTTTTATTACTATTTTTTTAGGCGGTAATGAGTTATTAATG
AGGGTTAGAACAATAGTATGAGTTCTTTTTTTTGTGTGAATTCGGGGAACTGTTCCCCGAATTCGATATG
ATCAGCTTATAGGATTGTGTTGGAAAGTTTTATTAAGGGTAGTTTTGAGAATAACTGGATTTGTAGCTATT
ATCAGATTTTTTTTatgataggaATAAGAGTTAGACCTTTGTTAAGAGCTTGTGGGTTATTAGTTGTATTAGGTA
ATTTAGTAAGGATTATAAGAAATAGATGAATGGGTGTGTGGTTAGGAATAGAACTTAATTTATTTGGCTTT
TTAATTATAATAAATCCTGAAGGGTGTTGTGTTGCTCAGGGAAGAATCAAGTACTTTATTGCACAAAGGA
TTGGTTCTATGTTAATATTGTTTGGGTTTTTTTGTGTGTCTATGTTTAATAGAGTGGGGAGGTTAATTCTTG
TTGGGGGAATTCTTTTAAAAGCGGGGGTTTTTCCTTTTCATAGATGAGTTCCGGACGTTGTTATTGTGTCT
AATTGGTTTATTGGGTGTATAATTTTAACATGGCAAAAGTTAGCTCCATTTTCTTTGTTTTCGTTTTTTCCT
AGTTCTTGGGTCTTGGTAATAAGATTGATTTTTATAAGGTTGGTGGGATGCTTTGGGGGATTAAATCAAC
ATTCAGTTCGTGGAATGGCAGTTTATTCTTCTTTTGTTCATAACTCTTGGCTGATTCTTTCTTTATTTTATTC
CTTTGGTGTTTTTTTTATTTATTATTTGGTGTATAGGTTGAGGGTTTTAATATTTTTTTTGAGGTGTTGGGTA
GTTAGAAAAAGGAGCTTAATAAGGTACTCCGTTAGATGATTGGGAATATTAAGTCTATTAATGTTATCTGG
TGTTCCACCTTTTATGGGGTTTTTTGTTAAATTGATTGTGATGTTAAGATGCCCGTCTATTCTTTTGATTAT
TTGTTTGGGGGGATCTATCGTAAGGCTAAAGTTTTATATTTCTTTTTTCTATAGAATGATTTTGAATTCTTAT
TTTCAATTGGAGTTGGTTTATATTTTTTGTAGGATGAATTTTGTAGTAGGGGTTAGTTCGTTATTGTTTTTT
ATATGGTGTATGATTGAATGTTTTCTTTTGTTTTTTCTTTTTATTTTAAGGATGGTGTTAGTACTAGTATTTT
TATTATCAGAGAAAACATTTGAGAGACGTGAGAAGTCGTCCCCTTATGAGTGTGGGTTTGACCCTATTTT
AAGGGCGCGAAGGTCTTTTTCTCTTCGTTTTTTTCTTTTAGGGGTCTTATTTGTTGTTTTTGATGTTGAGT
TGTCTTTGTTAATACCTGTGGTTCTTAGGATTAGGGTGGGGTTTAGCTATGTGGGGCTTATTAGATGTTTT
ATCTTTCTTTTAGTTTTGTTTTTAGGAATTTTTCATGAGTGACGAGAAGGGTCATTGAACTGGGTTATTTA
GGTGGCAGTAAGTATAATATTAGTATGAATCTTTCTTTGTATTGTTAGAAATAAAATGTTGAGGCTTTTAA
GTTTGGCTTTGTTAATAGTAGGGGTTATAGAGATAGGAGTAATCAATGATGAGGTAATTAGAATGGGGGG
TAGTCTTAGATATGATTTTTTATCCTATAGGTTAAGGGTTTTGAGGATTTTTATTATATTGTTAAGCTTAGTT
AGAAGAAAAAGGGTAATACGAACTTCTTTGTTTTATTTTTTAAATTTAAGATTATTAATAGTGTTAGTTTTT
GCTTTTTGTACTTCTAGTTTTCTAGGGTTTTTCTTTTTTTTTGAGTCTGCTTTGATTCCTTTAATTTTGGTTA
TTATAGGGTGACGGATAGAGGCAAGAACTTATATGTTTATCTATACGGTATTTGGTTCTCTTTTTTTTCTTGT
TTGGGATTTGTCTTCTTTTTTATAGAGGAAGGGATAATATATTATTAGGAAGAAATGTAAATAAAAAGGTT
AGAATGTTTTGGTGGGTTTTTATGATTGGGTTTCTAATTAAGTTGCCTGTATATCCTTTTCATTTATGGTTA
CCTAAGGCTCATGTGGAGGCTCCTGTTGCTGGATCAATGGTTTTGGCTGGGGTAGTATTAAAACTGGGTG
GATATGGTATATTGCGGTTGTTAATAGTAATAAGCGTAAATTTGGGGTTAAAATGTAATCTGATATTATGCG
TAGGGATAGTTGGTGGTTTTTATGCAAGATTGGTATGTTGTCGTCAGACGGATATAAAATGTTTAGTAGCA
TACTCTTCTGTAGGCCATATAAGGTTGGTTACTTTATGTCTAAGAAACATTAGGATAGGGGTAAAAGGTG
CTTTGTTTATTATAATTGGGCATGGGTTATGTTCTTCTGGGATATTTAGCCTTGTTAATGTTTTTTATAGTCA
TTCTGGTTATCGAAATCTGTATATAAATAAGGGGTTTTTGATAAAAAGTCCAATTCTGTGTTTGGTAGGAT
TTTTATTGTGTTCTAGTAATATAGCTGCTCCGCCGAGGTTAAATTTGTTGGGTGAGGTGCTAATGTTCATT
TGTTCATATGTAATTTCTTTTTGTTTTCTGTTGTTGTTAATGGCAATAACTATAGTTAGTGCTGTTTATAGCT
TGCATTTGTACACATCTACATGTCATGGAAAAGGGTCAGAGAGGATGATGGGATGAGATATTTATAGATC
TGTGTTCGTTTTGTTAGCTCATTGGTTGCCTTTGAATTTTTTGTTTTTGTTTATACCTTagTTAAAAAAAGG
GCATCTTATTAGAATTTTCATTGGAATGGAATTTATAATGTTAGGGGTGTTGTATAGTGCTAGGATCATAAT
GTGTTATAATGTAAGAATAGTATTCTTAATTATGTGTTTAGGGGTATGTGAGGCAAGGGTATGCTTAGCTT
TATTAGTTATGATAGTGCGTTTGTCAGGAAATGATTTAATTAGAAGATTGTCTTTGTATaaTTTAGGAAAGT
TTATCAAAAAATATTATTCATAGTGGTTTTTGGGTATTGCTTTATTTTATTAAGAAGAGTGAAGAGTGTCTA
TTTTATGGAGTTTATGTTGTGGGATTTAGGGGGTATAGGATTGTCTATTAATTTATTGGTTGATAGAATTGG
GGTGATATTCATTGGGACTATCATAATTATTTCTGGTAGAGTATTAATATACTGCTATTGGTATATAGATGAT
GAGAAATATTTTTTCCGTTTTATATACTTGGTTTATTTATTTGTTGGATCAATAATATTTATAATTTTAATTCC
TAATTTAATTACGTTGTTAATTGGGTGGGATGGGTTAGGATTAACTTCTTTTTTGTTAGTTGCACATTATCA
AAATTCTAGGTCTTTGTCAGGGAGCTTGTTAACGGCATTGACGAATCGGATTGGGGATGGGTTAATCTTG
TTAAGAATTTCTTTGTGAGGAAGAGAAAATATGTGAGTTTTGTATAATTTCAGAGGATCTTTTATAAGTGT
CTTTATTTGTGCGCTTATTTTTGGTGGAATAACTAAGAGTGCGCAGATACCTTTTTGTGCTTGGTTACCGG
CTGCTATGGCTGCTCCCACTCCTGTGTCTTCTTTGGTTCATTCTTCCACGTTAGTTACAGCGGGTGTATAT

TTACTTATTCGTGCGTATCCAGTAATAAGAAAGAGAATAATATTAGTGTTAAAGTTACTAAGGTTGTTTAC
TTTGGTGTTAGCTAGAAGCGCAGCAATTTTTTGTTTTGATATAAAGAAAATTATTGCATTATCAACTTTAA
GGCAATTAAGGGTAATAATATTTTCTTTATCTCATGGGTTGGTTTATGTTTCTTTTTTTCATTTAGTAATGCA
TGCATTGTTTAAAGCACTTCTTTTTTTGTCAGCAGGGGTAGTTATTCATTCTATAAAAAGTTGCCAAGATA
TTCGTAGAATAGGAAATTGCTGAGCAAATATGCCTTTTAGAATAGTTTCTATATTTATTGCGAGGTGTTCA
CTTTCAGGCCTTCCATTTATAAGTGGGTTTTTTTCTAAGGATATAATTGTTGATGTGTATAATAGAAAAAAT
TTTTTCTTTTTTGTTATAATGCCTGGGTTAGGGTTGACTAGAATTTACAGAATACGAGTATGAATAGTAAG
GTTTGGAGAAAACAAAGTTATGTTAGGATGTTTGAGAGTAAAAGAGCCAATAAAACTTCTAATCCCTTAT
TTAAGGCTTGGTTTTGGGGCATTATTTTTGGGTAAAATAATATGTCCAATCACTGAGAGGTTGATTTATTA
CAGGAGTTCATCATTAGGGGAGATATTAGTTTTAAGAATTCTATTTAGGGGAGTATATTTAAGAGTAGCAA
ACTGGGGAAAAGGGCCATCTttcAAAAAGTCTTCTTTTTTATTTAGAATATGATACTTAGAGTTAGTACATG
TAATCAAAAAGATATTTTTTGACCTTAGAAAATCTATTTATGAAGTGTGTGATAAAGGCATATTAGAGACA
GTGGGGCCGAAGTGGATGAGAATAAAAATAAGATGTTATAACGAGAATTTCTTTCTTTTTTTTTTTGTGT
TTAGGGGAATAATTTTTATAATGAGAATTGTTATTATGGAATTTATTTATTTGCATTTGGTAGTAGTTAGATT
GTGTGTTGTTTTTGTTAGTCAACCTTACTTACTGGGGTTAGTTCTTTTAATTTCTTCAATACTAGTTTGTTT
AATTATTGGGGTTAACATTAGAAGATTTCTTGGATTTTTGTTATTTATAAGATATGTGGGAGGGCTCATAGT
TTTATTTGTTTACGTTCTTAGTGTATTTCCTAATGAAAATTTTAAAACGAAAACGTTTTTTATAAGGTTAAG
GTTTGGAGTTCTGGTAGTGATATTACCTGTAAAGTCGGGCGCTCAAGAACTGCAGTTTCATTTTATGAGG
TTTTCTCATGTTTTTGTTTTTATAGCTTTATTTCTTTTGTTTATTATATTATGTGTTTCTTATTTAGTAATAAA
AAAACGGGTTCCTTTACGATCAATC"/>
<sequence id="seq_Septifer_bilocularisMK7215491" spec="Sequence"
taxon="Septifer_bilocularisMK721549" totalcount="4"
value="ATGTTGATAGATGTTTTTTCTGGGTTTGACGATCATAATTTTAATATTTTTTTTGTAACATTCATTTG
AGCTTTTAGATGTGTTTTTCCCATTTTGGTAATATCATCTATATTAGTTGTTGTGGGTAGTATCATTTCTAAG
TGCTATATAACTCTTGGAAGAGGAAAAGGGTTGCGTTTAACAGGGTTTTCCTTATTAATTTGTTCTTTATT
TTTTATTATTGTTTTGTCTAATCTTTCAGGTTGTATTCCTTATTTTTTTTTCTGTTAGTGCTCACTTTGTTTTT
GGGTTTTCTTATGCCATTATTATTTGATTTAGTATTATCATTTCAACTGTTTTCTGTAGTTATGAGCAGACTG
TAAGAATAATAGTTCCTTCTGGTCCCTTAGGTCTTGTACCGTTTGTTGCTATTTTGGAAGTTTTAAGTCAC
ATATTACGTCCTCTTACTTTAATTGTGCGGTTAGCATTAAATATTTCTACTGGTAAAATTATTTTGACTTTAT
TAAGTGAGATGGGTTTTGTTTTGTTTCTTTATAATTTAGTGTGATTTGTTCTTGTTAGTATTATAGGGCTAA
TTACTGCATTAGAATTAGGTGTGAGTTGTATTCAGGCTTATATTTTTTGCATTTTATTATGCCTATATAGTGG
TGATCATAGAGAATAACGTTGGTTTACATCTGTAAACCATAAAGAAATTGGTACTTTATATCTCCTTATTG
GTGTGTGATCGGGGTTAGTAGGAACAGGATTAAGAGTGTTGATTGTTCATTCCCATCCTGGAAGTAAGCT
TATAAAAGAAAGGTTTTATAATGTTGTGGTTACGTCACATGCTCTTATAATAATCTTTTTTGCTGTAATACC
TATTTTAATTGGTGGATTTGCTAATTGATTGCTTCCTTTATGTCTTGGTGCTGCTGATCTTATTTTTCCCCGT
CTTAATAATTTGAGTTATTGACTTGTTCCTAACTCATTATATTTGATAACTTGTTCTATATTTACTGAAAAAG
GTGCTGGTACTGGCTGAACTATTTACCCTCCTTTATCTAGTGTCGCTTATCATAATGGCCCCGCAGTAGAC
ACTGCTATTTTTTCTTTGCATGTTGTGGGTATAGGGTCTCTTGTTGGTGGTTTAAATTTTTTAGTTACTAAT
AAAGATGTTCCTGTTTTTCATATGAAAGGTGAAAAAGCGGAGTTATACTTAGCAAGAATTTCTGTTACAA
GTTTTTTGCTTGTTGCGTCTATTCCTGTTCTTGCTGGTGGTATTACTATGTTGTTATTTGACCGTAATTTTA
ACACTACATTTTTTGATCCTATAGGAGGAGGTGATCCTGTTTTGTTTCAGCATATTTTTTGGTTTTTTGGTC
ATCCGGAAGTCTATATCTTAATTCTTCCTGCTTTTGGTATTATGTCAAAAGTAATTTTACATTTTTCGGGTA
AATTAAAAGTTTTTGGTGCTTATGGTATGTATTATGCGATGGTAGGAATTGGTGGTTTGGGGCTTATAGTAT
GGGCTCATCATATATTTACAGTAGGTTTAAATGTTGATTCTCGTATGTATTATACTTCTGCTACAATAATCAT
TGCTGTTCCCACGGGTGTGAAAGTTTTTAGATGACTTTCTACTATGGCAGGAGGTCGTATTAAATTCTATC
CTCCTGCATGTTGAAGAACGGGGTTTTTATTTTTATTTACTGTCGGTGGTTTGACAGGAATTATACTATCT
AGTTCTTCTTTAGATGTTAGTCTTCATGATACTTATTATGTTACAGCTCACTTTCATTATGTTCTTTCGATGG
GTGCTGTATTTGGGATCTTTTGTGGTATTACTCACTGATTTCCTATATTTTATGGGGTTAGTTTACATCGTA
AATGGTCGAAGATACATTTTTTTGCTATATTTGTAGGGGTCAATTTAACTTTTTTTCCTATACATTTTTTAG
GATTGAGAGGTATGCCGCGCCGGTATTGTGATTATCCAGATTGTTATTCTAAGTGACATTGATTGTGTTCT
TACGGAGCTACTGCTTCTTATATTTCTTTGCTTTTTTTTTATGTTTATTTTATGAGAAGCAATAGTAAGCCAG
CGTAGAGTGGTTTTTTATGGGTCTATATATTTTCGTGATTGTATTTATAAAATTGGTCAAGATATTTTAGTGT
ATCATGGTTATGTTATAATGGTAGTATTTCTTGTTCTTATACTAGTGTTGTATGTTGGTACGGTAGTTACATG
TACAGGGTATCGTAGGCGTTTTTTTACTGATCATCAACAATTAGAATGGTGATGAACAGTTATTCCTATGA
TTCTTTTAGCTGCTTTGTGATGTCCTTCAACTTTAAATTTATATCGTATGGATGATATTAAGATTCCACGGT
GAAATTTTAAAGCATTAGGTAAGCAATGATATTGAAGTTACGAGTGTGAAACTGTTTTTATATTTGATTCT
TACATAAAACAAGATAGTGGAACAGGGTACCGTTTACTAGATGTTGATCATCGTATGGTAGCACCAGCAG
GAGTGCAAACAACTGTTTTTGTAACTAGTCCAGATGTTCTTCATTCTTTTAGGTTGTATGGCACTATACTA
AAAGTTGATGCAATTCCTGGTCGTCTTAATCAGCTTCCTTTGTTGGTAAATCGTGTGTGTGTATTTTGTATGG
TCAGTGTTCTGAAATTTGCGGGGTTAATCATAGATTTATGCCTATTGTCATTGAATTTATTCCTGAGCAATA
TTTTATTAAGTGAATTAATGCTATGGAAGAA-----------------------
ATGAAACGAAGACCTTTTTATGTTCCCGATCCAAGTCCGTGGCCTTTTTTTGTAGCTATTGCTTTAAATAA
TATGGCTATTGGGTTAGTTTTATGGATACATCGTAAAGATTTAATGTTGTTGGGTGGTGCTTTAATTTTATT

AATCAGATCTGTCAGCTGGTGGCGTGATTTGTTACGTGAAGGTGACATGGGTTTTCATACGCGGTTTGTT
ATTAAAGGTTATCGTGACGGTATAGGATTGTTTATTTTTTCCGAAGTAATGTTTTTCTTCTCTTTTTTTTGA
GCTTTTTTTCACAATGCATTAAGTCCTTCTACCGAATTAGGTATGCGTTGACCTCCTCCAGGGATTCGTGC
TCCTCAGCCTTGTTCAATTCCACTTTTCAATACAGCGTTGTTGATCAGTAGCGGTGCTTTTGTTACTTTGG
CTCATAAAAGGGTAATTAGAGAGTATGTTCAGGGGCCTTTGTTAGGTTTATTTATAGCAATTGTCTGTGGT
GTATTATTTTTGTTTGTTCAAGGATTTGAGTATTTTAGTAATTCGTTTACGTTGTCTGATAGGGTTTACGGA
AGTGTTTTTTATGTTTTAACAGGTTTTCATGGGACTCACGTATTAGTAGGTAGTGTTTGGCTTATTGTGAC
TTTTGTACGAACGTGATTAGGTCATTTTCGACAAAGACGTCATTTTGGTATAAGAGCTTGTATCTGATACT
GACATTTTGTAGATGTTGTTTGATTATTTGTCTATTTTATAGTGTATTCTTGATTTGGTGGTCCTTTTCGTAA
ACGTAATAAATTGATAAAAATTATCAATAACAGGCTATATGATCTTCCTTGTCCTATTAACTTGAGAGTATG
ATGAAGATTTGGTTCAATGCTTGGCTTATGTTTAGTTATTCAGATTGTAACTGGTTTTATATTAAGAACTTT
TTATACTGCTGATGAAACTATGTCGTTTGATTCGGTAATTTTTATTATACGTAATGTTAAAAAGGGTTGAAT
GTTTCGTAGCATTCATGCTAATGGTGCTTCAGTATTCTTTATTTGTATTTATATTCATATTGGTCGTGGTTTG
TACTATGGGTCATATCTTTATGTCCACGTATGAAATATTGGAATTATGCTGTATTTGTTACTAATAGCTGAA
GCTTTTTTAGGTTATGTTCTTCCGTGGGGTCAAATGTCTTTTTGGGGTGCTACTGTGATTACAAACCTTAT
GACTGTAATTCCATTTTTTGGCAAAACTATCACTCAATGAATTTGAGGTTATTATACTGTGTCTAACCCAA
CACTAAAGCGTTTTTATTCTTTTCACTTTATTGTTCCTTTTTTAATGGTTGTTATAAGTGCTTTGCACCTTT
TTTATCTTCATGAAACTGGTAGAAATAATCCTTTAGGTATTGAAAGTGATATAATGTGTATTCCTTTTCATC
CTTTTTACACTGTAAAAGATCTTTTTGGATTTGTTTGTCTTGCGTGAGGTTTGATATTTTTAGTATGTGTTA
AACCTGAGATGTTAGGTAATGTTACTAATTATATTCCTGCTAATCCAATAAAAACCCCTAAACATGTAAAA
CCTGAATGATATTTTCTTTTTGCATACGCTATCTTACGTTCAATTCCTAATAAAAGAGGTGGTATTTGTGCA
ATGTTGTTGTCTATTTTGATTTTGTATCTTCTTCCTTTTATTCATACCGGTAAGTTTCGTAGATTGTGTTTTT
ATCCTTTTAATCAAATAATTTTTTGATGTTTTATTTCTATTTTTATTGGACTATCTTATGCTGGTTATAGTCCT
CCGCGTGAACCGTGGTTAACGTGTGCGTGATATTTAACTTTATTATATTTTCCTTTAATTGTTTTGAATCCT
TTGTCTTTATTTGTATGAGATTGGTTAATTAGTACACATTTTTTAATTTTAATTTTGCCATTAGTATGTGCTT
TGCTCGCGGTGGCATTTTATACTTTATTAGAACGTAAATTGTTGGGTTATATTATATTACGTAAAGGACCAA
ATAAAGTTGGTTTTATAGGTATTCTTCAGCCTTTCAGGGACGCAGGTAAACTTTTTTGTAAGGAGGTTGT
AGTGCCGCGTTATGCAAATTTTATTCCATTTATTTTATGTCCTACGTTTGTTTTAGGAATCTCTTTAACTTTA
TGAGTCATATACCCTTTTAAAAATAGTGAATTAATTTTTGTTTGTGGACTTGTTCAGTTTTTAGTTATTTCA
AGCATAAGAGTATATGGAACAATAGTTGCTGGATGATCTTCAAATTCAAAGTATGCTCTTTTGGGGGCTG
TGCGTAGTGTAGCACAAAGTATTTCTTACGAAGTGCCTATGAGTTTTATCATTTTGTGTTTTATTTATGGA
AGTAGGAGTTTTATACTTCAAGAAATTGGCATAATTATGTTTTTTTTTCCTTTTTTCCCATGTTGTGTGTTG
TGAGTTATTTGTATTTTAGCAGAAACTAATCGTGCTCCTTTTGATTTTGTTGAGGGTGAATCAGAATTAGT
TTCAGGGTTTAATGTTGAGTACAGAGGTGGAGCTTTTGCTATAATTTATATGGCTGAATACTCAAGAATAC
TGTTTAACAGAGTAATCAGAAGTGTTTTGTTTTTTGGGAGAAACGAAATTTTTATAAGAATTATATGTATA
TTTTTTGTTGTAGGTTTTGTGTGAGTGCGGGGCACTATACCACGAATACGCTATGATAAGCTTATGAAATT
GTGCTGGACTGTAATTTTGTGCGTGGCAATGTGCGTAGCGTGTGTGGTTATATGTTATAGATTTTTGTAAG
CTTTAGTTTGTTTATTAGACCTATAATAATTTTGAGCATTTGTTTGGTTTTTTTAGGTCCTACTATAAGGTTA
ACAAGTGATAATCTGTACGGTGTATGATTAGGTTTAGAATTAAATTTATTTGCTTTTATTATTATAATAAATC
CTGAAGGATTTTGGATTGCAGAACCATGCATTAAATACTTTGTAGTTCAAGTTATTGGTTCTAGATTTGTA
TTAATTAGTGCAATAACTTTAAATTTTTATGCTTCTTATAGTTTGTTTTTAAGATTAATTGGTTTTTTTATTA
AAGCTGGTATTTTTCCTTTTCATTCGTGGGTTCCTTCAGTGGTTAATTCGTCTGATTGGTTAGTTAGAGGT
ATAGTTTTAACTTGGCAAAAATTGGTTCCTTTTATCTTTGTTGGTTTTATAAGAAACTATTATATTTTATTTG
TTGCTCTTGTAGCCATAGGAATTTTAGGTGGTGTTGGAGGTTTAAATCAACATAGTGTTCGCTCTATAAGT
GCTTATTCTTCTTTTGTTCATTCTTCGTGAATACTTGCTTCTTTTATAAGTTCGTTGATTACTTTTTTGTTTT
ATTTTCTTTTTTATTGTGTGTCTTTGTATATTTTTTTTTACGGGTGTTGTAAAGTAGGCAAGTCTTATGCTA
AGAATAAAACGGTAAGATTTCTGGGATGTTTAGGTATTTTAATGCTTAGAGGAATCCCTCCCATGTCAGG
GTTTTTCCCAAAAGTTGTTGTTTTTTTAAGCGTTGATAGTTTAGTTGTGTTGTGATGTGTAATTAGGTCTT
TTATTAGGTTGAAGTATTATTTGTCTTATTTTTATCTTATATTAATTTCTTCTTTTTATAATTTTAGTTTTCATT
TCTTTTTCATTGTTTTTTTTAACTTAATTAGCTTTGTCTATTTGTTGACTATTATTTAAcaaATGAGCCTTTTT
TTTGTTTCTTTTTTCATTGTCATTCTAATAGCTTTGTTGTGAGCCTTAATGTTTTTATCAGAAAAGTCGTTA
GTTAGACGTGAGAAAAGTTCTCCTTACGAATGTGGTTTTGAAGTTGTGATAAGAGCCCGTGCTCCGTTT
TCTTTGCGTTTTTTTATTTTGGCCGTACTCTTTGTTGTGTTTGATGTAGAAGTAGCATTAATAGTTCCTGTA
GTGTTTAGTATCAGTTTTGCTAAAAGAACTTTTGGATTAGTTAGTAGCGTTTTATTTTTTTTTGTTTTGTTT
ATTGGCTTATTTCATGAGTATCGTGAAGGTTCTCTTGATTGAGTTAGTTAAATTATTACAGGTGCGTGTGT
TTTACTAGTGTTTATTGTTATGTTAAGAGATAGTTTAACTATAGTATTATTGTTATTATGTTTAAGTGTTATG
TGTATTACAAGAGTTTATATGCAAAGTGAAGTTATTGACTACATAGGGATTGTATCATTAGATAGTACAAG
AACAAGATTAATTACATTATCTATTTTTGTAAGTGTGTTGTCGATGCTAAGGTCTGTAAATGTTGTGCGGG
CAAAACTTTTTAGTTTTATTATGAGTATAAGAGTTCTAAGCCTTGTTTTATCATTTAGAGTAGGTAATTTTT
TTTTATTTTTTTTTTTGCTTTGAAAGAACTTTAATGCCTATTTTATTTTTGATTGTTGGATGGCGCCTGCAAG
CAGGCAGATATATAGTTTTATATACGGTTTTTGGTTCTTACTTTTTTCTTTTTGGTATTAGATACTTAGCTTT
TAACGGATCAGGTTACATGTTTTTAACAGATAAATGTGGTAAAATTATTTCTATAGTATGAGTCATTTTTGT
AGTAGGATTTTTAGTTAAGTTACCTGTATATCCATTTCATTTATGGCTTCCCAAAGCTCACGTAGAGGCGC

CTGTTGCGGGGTCTATGCTTTTGGCAGGGGTCCTTCTTAAGCTTGGTGGTTATGGTTTAATTCGATTTTTT
TCCGTTGTTAGCTGTTCTTATAATATATTTTCTTGATTTGTTTTAGTTGTAGGTCTTCTTGGTGGTGTGTACT
GTGGTTTAATATGTTTGCGTCAGGTAGATTTAAAATGTTTAGTTGCTTATTCTTCTGTTTCTCATATAAGGT
TAGTACTTCTTATTATGAGTAATACATTTGTTGGAGTGTTAGGAGCTGTTGTTATTATGATTGGCCATGGTT
TATGTTCATCTGGTCTTTTTAGAATAGTTAATCTATACTATTTAAATAGAAAATCTCGTCTGCTTAGAATAA
ATAAAGGTGGATTAATTATTTTTCCTTATCTTAGATTTATGTGTTTTTTGTTAAGTTCATCTAATATGGCGGC
ACCACCTAGTCTCAATCTTCTTGGGGAAATTTTAGTGTTTATTTCTTGTGGTTATGTGTCATTAATTTTTTT
GATTATGATTGGCCTTATTAGATTTTTTAGTGCGTGTTATAGGTTGTATGTTTATTGTTCTTGTAATCATGGA
AAAGTAGGTAATTATCCTACTAGATGATATAACTTTTTAGACTTTTTTGTTCTTTTTAGTCATTGGGTTCCT
TTGAATATACTATTTTTGTTTGTGCCTTAACGTCAGAAAGTTCATCTTATTGGTGTATTTGTGGGTTTAGAA
TTTATAAGTCTTGGTATTTTGTTTGCTGCTGGTTTATTTCTTAATAATGTTGTATGTTTAATTTTTTTGGTGT
TGTGTTTTGCTGTGTGTGAAGCTAGTATTTCGTTAGCTTTGATTGTCATGATGGTGCGTTTATGTGGTAAT
GACTTAGTTAGAAACTTAGTTTGTGATAAGAAATGTGTGTTAAGAAATTGTGGTTAATTTTGTGATGTTT
GGGTGCTTTATTTATTGTAATGTGTGGTAATAAAGATTTAATTGTTATAGAATTTGATTATCTTATTAGAGA
ATCTTTTGTTTTTAGATTTGGCTTAATTTTTGATAGATTATCTATTGGTTTTGTTGGCTGTATTATGTTGATTT
CTGGTAGCGTTTTTGTTTATAGAATTTGATACATAGACAATGAAGTGTTTTTTAAGCGATTTATTTTTTTGG
TTTATTTGTTTGTTTTGTCAATAATATTTATAATTATAACTTGTAACTTAGTTTCTTTGCTTATTGGTTGAGA
TGGGTTAGGACTTACTTCTTTTTTGTTAGTTTGTTATTATCAAAATAATAAATCTTTGTCTGGAGCTATGCT
TACGGCTTTAAGTAATCGTGTAGGTGATGTTTTTATTTTATTTAGTATTTGTTTTTTTATAAATGAGGGAGG
ATGATCTTTATATAGTTATAACAGAATGATATATATATTTTTTTGTTTAATTATTTTATTTGCAGGTATAACTA
AAAGAGCACAGATGCCTTTTGTAGCCTGGTTGCCTGCTGCTATAGCGGCTCCTACTCCAGTTTCTTCTTT
GGTCCATTCATCAACTCTTGTTACAGCAGGTGTTTATTTAGTTCTTCGTAGTTATGGTTGTTTAGTTCATTC
GAGTTTTTTAATTTTAAAATTTCTTTCTATTATTACTTTAGTTTTAGCTGGTTCTAGTGCTTTGAGAGTTGT
AGATTTAAAAAAAGTAGTTGCTCTTTCTACGTTAAGTCAGTTAAGTGTTATAATATTTAGAATTTCTATTAT
ATTGCCTTTTATTTCTTTTTTTCATTTAGTTACTCATGCCGTATTTAAAGCTTTACTTTTTTTAAGTGCGGGG
TGTTTTATTCATAGACTATTAAATTGTCAGGATTATCGTATAATTGGTTCAGGTTGGAAATTAGTTCCTTTT
ACCAGGGTAGCAATAATTTGTGCTAACATATCTCTTTGTGGAGTTCCCTTTATAAGAGGATTTTTTTCTAA
AGATTTAATTATTGAAGTTATAGGCGGGGATTTTTTTATTTATTTTATAGAGTTGCTTGGGTTTTTTTTAGC
AGTTGATACAGTATACGTATAATAAATGTTATTTTTGGTGAGAGTTTTTATTTTGTTAAGCATACAAAGTGG
AGTGAACCTTTTGAGTTGAAATTTTCTTATTTTTTTTTGTTATGTGGTGCTGTTATAGTTGGTGCTGTTATA
AAATCTAAAGTAGTTTGTTTTAACGAAATTGCTATAATTATAAATGTAGATTTTGTATTTTTTTTGTTTTTTT
GATTAGGATTAGCTTTTTTTATTAGATTATTTATTGTTTCTCATTTTTACATAAAAAAATGAATTTTTAGGGTC
TGTGTGATGAATAGAATTATCTCAGCCTTTTAGATATGGTGTTATGAACTTATCTGATTCTCTTGTTAAAGT
TCTTGATAAAGGATGATTAGAGTTTTTGGGTCCTCAGTATTTTGGGTCTTTGTTGTCAAAACACAATCAAT
TATTTTTTCTTGTAATAATAGTGATTTGTTGTTGTATAGTTATAAAAAATTTTACTATAAATGTTAATGTTTTTT
TGTTCTTTTTTCATCTCTGTTGTGTGTTGTGTTTGTTCTGTTCAACCTTTAGTTTTAGGTTTTTTTCTTATTA
TTAATTCTTTACTTGTAAGAGTAGTTGTTGGTGCAGTTACAAGAAGTTTATTAGGTTTTTTGTTTTTTATAA
CCTATGTTGGTGGGGTGATGGTGCTTTTTCTTTACGTTCTTAGTATTTACCCTAATGAAAAGATATTAATAA
GTTGACAAATGTTTGGATTGTTAATTATTTTTTTATTTTTCATTAATATAATAAGATATAGAGAAAAAGAAA
TGGTTTTTCATTATATAAGTGTTGCTGAATTATTTGTTTTTTTAGCAGTTGTTTTACTTTTTTGTATAATAAT
TGTAAGTTACGTATGTATAAAAAAACGTGTTCCATTTCGTAGGATT"/>
</data>

<map name="Uniform" >beast.math.distributions.Uniform</map>
<map name="Exponential" >beast.math.distributions.Exponential</map>
<map name="LogNormal" >beast.math.distributions.LogNormalDistributionModel</map>
<map name="Normal" >beast.math.distributions.Normal</map>
<map name="Beta" >beast.math.distributions.Beta</map>
<map name="Gamma" >beast.math.distributions.Gamma</map>
<map name="LaplaceDistribution" >beast.math.distributions.LaplaceDistribution</map>
<map name="prior" >beast.math.distributions.Prior</map>
<map name="InverseGamma" >beast.math.distributions.InverseGamma</map>
<map name="OneOnX" >beast.math.distributions.OneOnX</map>

<run id="mcmc" spec="MCMC" chainLength="50000000">
<state id="state" spec="State" storeEvery="5000">
<tree id="Tree.t:NADH6_pos1" spec="beast.evolution.tree.Tree" name="stateNode">
<trait id="dateTrait.t:NADH6_pos1" spec="beast.evolution.tree.TraitSet" traitname="date">
Mytilus_californianusF-JX486124=0,
Mytilus_coruscusKart1=0,
Mytilus_coruscusKJ577549=0,
Mytilus_chilensisKP100300=0,
Mytilus_chilensisNC030633=0,
Mytilus_edulisF-MF407676=0,
Mytilus_galloprovincialisF-FJ890=0,
Mytilus_trossulusF-GU936625=0,
Mytilus_trossulusF-HM462080=0,
Arcuatula_senhousia1=0,
Musculista_senhousiaGU001953=0,
Bathymodiolus_childressiNC059707=0,
Bathymodiolus_japonicusAP014560=0,
Bathymodiolus_securiformisNC0395=0,
Brachidontes_exustusKM233636=0,
Crenomytilus_grayanusNC044128=0,
Gregariella_coralliophagaNC04412=0,
Modiolus_kurilensisKY242717=0,
Modiolus_modiolusKX821782=0,
Mytilisepta_keenaeNC044127=0,
Mytilisepta_virgataKX094521=0,
Perna_canaliculusMG766134=0,
Perna_canaliculusMK775558=0,
Perna_pernaOK576481=0,
Perna_viridisJQ970425=0,
Perna_viridisMW727515=0,
Septifer_bilocularisMK721549=0 <taxa id="TaxonSet.NADH6_pos1" spec="TaxonSet">
<alignment id="NADH6_pos1" spec="FilteredAlignment" filter="9959-10381\3">
<data idref="Mytilidae27sq12PCGs123ps"/>
</alignment>
</taxa>
</trait>
<taxonset idref="TaxonSet.NADH6_pos1"/>
</tree>
<tree id="Tree.t:NADH1_pos1" spec="beast.evolution.tree.Tree" name="stateNode">
<trait id="dateTrait.t:NADH1_pos1" spec="beast.evolution.tree.TraitSet" traitname="date">
Mytilus_californianusF-JX486124=0,
Mytilus_coruscusKart1=0,
Mytilus_coruscusKJ577549=0,
Mytilus_chilensisKP100300=0,
Mytilus_chilensisNC030633=0,
Mytilus_edulisF-MF407676=0,
Mytilus_galloprovincialisF-FJ890=0,
Mytilus_trossulusF-GU936625=0,
Mytilus_trossulusF-HM462080=0,
Arcuatula_senhousia1=0,
Musculista_senhousiaGU001953=0,
Bathymodiolus_childressiNC059707=0,
Bathymodiolus_japonicusAP014560=0,
Bathymodiolus_securiformisNC0395=0,
Brachidontes_exustusKM233636=0,
Crenomytilus_grayanusNC044128=0,
Gregariella_coralliophagaNC04412=0,
Modiolus_kurilensisKY242717=0,
Modiolus_modiolusKX821782=0,
Mytilisepta_keenaeNC044127=0,
Mytilisepta_virgataKX094521=0,
Perna_canaliculusMG766134=0,
Perna_canaliculusMK775558=0,

Perna_pernaOK576481=0,
Perna_viridisJQ970425=0,
Perna_viridisMW727515=0,
Septifer_bilocularisMK721549=0 <taxa id="TaxonSet.NADH1_pos1" spec="TaxonSet">
<alignment id="NADH1_pos1" spec="FilteredAlignment" data="@Mytilidae27sq12PCGs123ps" filter="4681-5573\3"/>
</taxa>
</trait>
<taxonset idref="TaxonSet.NADH1_pos1"/>
</tree>
<tree id="Tree.t:COX2_pos1" spec="beast.evolution.tree.Tree" name="stateNode">
<trait id="dateTrait.t:COX2_pos1" spec="beast.evolution.tree.TraitSet" traitname="date">
Mytilus_californianusF-JX486124=0,
Mytilus_coruscusKart1=0,
Mytilus_coruscusKJ577549=0,
Mytilus_chilensisKP100300=0,
Mytilus_chilensisNC030633=0,
Mytilus_edulisF-MF407676=0,
Mytilus_galloprovincialisF-FJ890=0,
Mytilus_trossulusF-GU936625=0,
Mytilus_trossulusF-HM462080=0,
Arcuatula_senhousia1=0,
Musculista_senhousiaGU001953=0,
Bathymodiolus_childressiNC059707=0,
Bathymodiolus_japonicusAP014560=0,
Bathymodiolus_securiformisNC0395=0,
Brachidontes_exustusKM233636=0,
Crenomytilus_grayanusNC044128=0,
Gregariella_coralliophagaNC04412=0,
Modiolus_kurilensisKY242717=0,
Modiolus_modiolusKX821782=0,
Mytilisepta_keenaeNC044127=0,
Mytilisepta_virgataKX094521=0,
Perna_canaliculusMG766134=0,
Perna_canaliculusMK775558=0,
Perna_pernaOK576481=0,
Perna_viridisJQ970425=0,
Perna_viridisMW727515=0,
Septifer_bilocularisMK721549=0 <taxa id="TaxonSet.COX2_pos1" spec="TaxonSet">
<alignment id="COX2_pos1" spec="FilteredAlignment" data="@Mytilidae27sq12PCGs123ps" filter="2089-2781\3"/>
</taxa>
</trait>
<taxonset idref="TaxonSet.COX2_pos1"/>
</tree>
<tree id="Tree.t:NADH1_pos2" spec="beast.evolution.tree.Tree" name="stateNode">
<trait id="dateTrait.t:NADH1_pos2" spec="beast.evolution.tree.TraitSet" traitname="date">
Mytilus_californianusF-JX486124=0,
Mytilus_coruscusKart1=0,
Mytilus_coruscusKJ577549=0,
Mytilus_chilensisKP100300=0,
Mytilus_chilensisNC030633=0,
Mytilus_edulisF-MF407676=0,
Mytilus_galloprovincialisF-FJ890=0,
Mytilus_trossulusF-GU936625=0,
Mytilus_trossulusF-HM462080=0,
Arcuatula_senhousia1=0,
Musculista_senhousiaGU001953=0,
Bathymodiolus_childressiNC059707=0,
Bathymodiolus_japonicusAP014560=0,
Bathymodiolus_securiformisNC0395=0,
Brachidontes_exustusKM233636=0,
Crenomytilus_grayanusNC044128=0,

Gregariella_coralliophagaNC04412=0,
Modiolus_kurilensisKY242717=0,
Modiolus_modiolusKX821782=0,
Mytilisepta_keenaeNC044127=0,
Mytilisepta_virgataKX094521=0,
Perna_canaliculusMG766134=0,
Perna_canaliculusMK775558=0,
Perna_pernaOK576481=0,
Perna_viridisJQ970425=0,
Perna_viridisMW727515=0,
Septifer_bilocularisMK721549=0 <taxa id="TaxonSet.NADH1_pos2" spec="TaxonSet">
<alignment id="NADH1_pos2" spec="FilteredAlignment" data="@Mytilidae27sq12PCGs123ps" filter="4682-5573\3"/>
</taxa>
</trait>
<taxonset idref="TaxonSet.NADH1_pos2"/>
</tree>
<tree id="Tree.t:NADH4_pos1" spec="beast.evolution.tree.Tree" name="stateNode">
<trait id="dateTrait.t:NADH4_pos1" spec="beast.evolution.tree.TraitSet" traitname="date">
Mytilus_californianusF-JX486124=0,
Mytilus_coruscusKart1=0,
Mytilus_coruscusKJ577549=0,
Mytilus_chilensisKP100300=0,
Mytilus_chilensisNC030633=0,
Mytilus_edulisF-MF407676=0,
Mytilus_galloprovincialisF-FJ890=0,
Mytilus_trossulusF-GU936625=0,
Mytilus_trossulusF-HM462080=0,
Arcuatula_senhousia1=0,
Musculista_senhousiaGU001953=0,
Bathymodiolus_childressiNC059707=0,
Bathymodiolus_japonicusAP014560=0,
Bathymodiolus_securiformisNC0395=0,
Brachidontes_exustusKM233636=0,
Crenomytilus_grayanusNC044128=0,
Gregariella_coralliophagaNC04412=0,
Modiolus_kurilensisKY242717=0,
Modiolus_modiolusKX821782=0,
Mytilisepta_keenaeNC044127=0,
Mytilisepta_virgataKX094521=0,
Perna_canaliculusMG766134=0,
Perna_canaliculusMK775558=0,
Perna_pernaOK576481=0,
Perna_viridisJQ970425=0,
Perna_viridisMW727515=0,
Septifer_bilocularisMK721549=0 <taxa id="TaxonSet.NADH4_pos1" spec="TaxonSet">
<alignment id="NADH4_pos1" spec="FilteredAlignment" data="@Mytilidae27sq12PCGs123ps" filter="6852-8126\3"/>
</taxa>
</trait>
<taxonset idref="TaxonSet.NADH4_pos1"/>
</tree>
<tree id="Tree.t:NADH4L_pos3" spec="beast.evolution.tree.Tree" name="stateNode">
<trait id="dateTrait.t:NADH4L_pos3" spec="beast.evolution.tree.TraitSet" traitname="date" value="">
<taxa id="TaxonSet.NADH4L_pos3" spec="TaxonSet">
<alignment id="NADH4L_pos3" spec="FilteredAlignment" data="@Mytilidae27sq12PCGs123ps" filter="8129-8341\3"/>
</taxa>
</trait>
<taxonset idref="TaxonSet.NADH4L_pos3"/>
</tree>
<tree id="Tree.t:NADH4L_pos1" spec="beast.evolution.tree.Tree" name="stateNode">
<trait id="dateTrait.t:NADH4L_pos1" spec="beast.evolution.tree.TraitSet" traitname="date">

Mytilus_californianusF-JX486124=0,
Mytilus_coruscusKart1=0,
Mytilus_coruscusKJ577549=0,
Mytilus_chilensisKP100300=0,
Mytilus_chilensisNC030633=0,
Mytilus_edulisF-MF407676=0,
Mytilus_galloprovincialisF-FJ890=0,
Mytilus_trossulusF-GU936625=0,
Mytilus_trossulusF-HM462080=0,
Arcuatula_senhousia1=0,
Musculista_senhousiaGU001953=0,
Bathymodiolus_childressiNC059707=0,
Bathymodiolus_japonicusAP014560=0,
Bathymodiolus_securiformisNC0395=0,
Brachidontes_exustusKM233636=0,
Crenomytilus_grayanusNC044128=0,
Gregariella_coralliophagaNC04412=0,
Modiolus_kurilensisKY242717=0,
Modiolus_modiolusKX821782=0,
Mytilisepta_keenaeNC044127=0,
Mytilisepta_virgataKX094521=0,
Perna_canaliculusMG766134=0,
Perna_canaliculusMK775558=0,
Perna_pernaOK576481=0,
Perna_viridisJQ970425=0,
Perna_viridisMW727515=0,
Septifer_bilocularisMK721549=0 <taxa id="TaxonSet.NADH4L_pos1" spec="TaxonSet">
<alignment id="NADH4L_pos1" spec="FilteredAlignment" data="@Mytilidae27sq12PCGs123ps" filter="8127-8341\3"/>
</taxa>
</trait>
<taxonset idref="TaxonSet.NADH4L_pos1"/>
</tree>
<tree id="Tree.t:NADH2_pos1" spec="beast.evolution.tree.Tree" name="stateNode">
<trait id="dateTrait.t:NADH2_pos1" spec="beast.evolution.tree.TraitSet" traitname="date">
Mytilus_californianusF-JX486124=0,
Mytilus_coruscusKart1=0,
Mytilus_coruscusKJ577549=0,
Mytilus_chilensisKP100300=0,
Mytilus_chilensisNC030633=0,
Mytilus_edulisF-MF407676=0,
Mytilus_galloprovincialisF-FJ890=0,
Mytilus_trossulusF-GU936625=0,
Mytilus_trossulusF-HM462080=0,
Arcuatula_senhousia1=0,
Musculista_senhousiaGU001953=0,
Bathymodiolus_childressiNC059707=0,
Bathymodiolus_japonicusAP014560=0,
Bathymodiolus_securiformisNC0395=0,
Brachidontes_exustusKM233636=0,
Crenomytilus_grayanusNC044128=0,
Gregariella_coralliophagaNC04412=0,
Modiolus_kurilensisKY242717=0,
Modiolus_modiolusKX821782=0,
Mytilisepta_keenaeNC044127=0,
Mytilisepta_virgataKX094521=0,
Perna_canaliculusMG766134=0,
Perna_canaliculusMK775558=0,
Perna_pernaOK576481=0,
Perna_viridisJQ970425=0,
Perna_viridisMW727515=0,
Septifer_bilocularisMK721549=0 <taxa id="TaxonSet.NADH2_pos1" spec="TaxonSet">

<alignment id="NADH2_pos1" spec="FilteredAlignment" data="@Mytilidae27sq12PCGs123ps" filter="5574-6503\3"/>
</taxa>
</trait>
<taxonset idref="TaxonSet.NADH2_pos1"/>
</tree>
<tree id="Tree.t:NADH3_pos2" spec="beast.evolution.tree.Tree" name="stateNode">
<trait id="dateTrait.t:NADH3_pos2" spec="beast.evolution.tree.TraitSet" traitname="date">
Mytilus_californianusF-JX486124=0,
Mytilus_coruscusKart1=0,
Mytilus_coruscusKJ577549=0,
Mytilus_chilensisKP100300=0,
Mytilus_chilensisNC030633=0,
Mytilus_edulisF-MF407676=0,
Mytilus_galloprovincialisF-FJ890=0,
Mytilus_trossulusF-GU936625=0,
Mytilus_trossulusF-HM462080=0,
Arcuatula_senhousia1=0,
Musculista_senhousiaGU001953=0,
Bathymodiolus_childressiNC059707=0,
Bathymodiolus_japonicusAP014560=0,
Bathymodiolus_securiformisNC0395=0,
Brachidontes_exustusKM233636=0,
Crenomytilus_grayanusNC044128=0,
Gregariella_coralliophagaNC04412=0,
Modiolus_kurilensisKY242717=0,
Modiolus_modiolusKX821782=0,
Mytilisepta_keenaeNC044127=0,
Mytilisepta_virgataKX094521=0,
Perna_canaliculusMG766134=0,
Perna_canaliculusMK775558=0,
Perna_pernaOK576481=0,
Perna_viridisJQ970425=0,
Perna_viridisMW727515=0,
Septifer_bilocularisMK721549=0 <taxa id="TaxonSet.NADH3_pos2" spec="TaxonSet">
<alignment id="NADH3_pos2" spec="FilteredAlignment" data="@Mytilidae27sq12PCGs123ps" filter="6505-6851\3"/>
</taxa>
</trait>
<taxonset idref="TaxonSet.NADH3_pos2"/>
</tree>
<tree id="Tree.t:NADH2_pos3" spec="beast.evolution.tree.Tree" name="stateNode">
<trait id="dateTrait.t:NADH2_pos3" spec="beast.evolution.tree.TraitSet" traitname="date">

Mytilus_californianusF-JX486124=0,
Mytilus_coruscusKart1=0,
Mytilus_coruscusKJ577549=0,
Mytilus_chilensisKP100300=0,
Mytilus_chilensisNC030633=0,
Mytilus_edulisF-MF407676=0,
Mytilus_galloprovincialisF-FJ890=0,
Mytilus_trossulusF-GU936625=0,
Mytilus_trossulusF-HM462080=0,
Arcuatula_senhousia1=0,
Musculista_senhousiaGU001953=0,
Bathymodiolus_childressiNC059707=0,
Bathymodiolus_japonicusAP014560=0,
Bathymodiolus_securiformisNC0395=0,

Brachidontes_exustusKM233636=0,
Crenomytilus_grayanusNC044128=0,
Gregariella_coralliophagaNC04412=0,
Modiolus_kurilensisKY242717=0,
Modiolus_modiolusKX821782=0,
Mytilisepta_keenaeNC044127=0,
Mytilisepta_virgataKX094521=0,
Perna_canaliculusMG766134=0,
Perna_canaliculusMK775558=0,
Perna_pernaOK576481=0,
Perna_viridisJQ970425=0,
Perna_viridisMW727515=0,
Septifer_bilocularisMK721549=0

<taxa id="TaxonSet.NADH2_pos3" spec="TaxonSet">
<alignment id="NADH2_pos3" spec="FilteredAlignment" data="@Mytilidae27sq12PCGs123ps" filter="5576-6503\3"/>
</taxa>
</trait>
<taxonset idref="TaxonSet.NADH2_pos3"/>
</tree>
<tree id="Tree.t:NADH3_pos1" spec="beast.evolution.tree.Tree" name="stateNode">
<trait id="dateTrait.t:NADH3_pos1" spec="beast.evolution.tree.TraitSet" traitname="date">
Mytilus_californianusF-JX486124=0,
Mytilus_coruscusKart1=0,
Mytilus_coruscusKJ577549=0,
Mytilus_chilensisKP100300=0,
Mytilus_chilensisNC030633=0,
Mytilus_edulisF-MF407676=0,
Mytilus_galloprovincialisF-FJ890=0,
Mytilus_trossulusF-GU936625=0,
Mytilus_trossulusF-HM462080=0,
Arcuatula_senhousia1=0,
Musculista_senhousiaGU001953=0,
Bathymodiolus_childressiNC059707=0,
Bathymodiolus_japonicusAP014560=0,
Bathymodiolus_securiformisNC0395=0,
Brachidontes_exustusKM233636=0,
Crenomytilus_grayanusNC044128=0,
Gregariella_coralliophagaNC04412=0,
Modiolus_kurilensisKY242717=0,
Modiolus_modiolusKX821782=0,
Mytilisepta_keenaeNC044127=0,
Mytilisepta_virgataKX094521=0,
Perna_canaliculusMG766134=0,
Perna_canaliculusMK775558=0,
Perna_pernaOK576481=0,
Perna_viridisJQ970425=0,
Perna_viridisMW727515=0,
Septifer_bilocularisMK721549=0 <taxa id="TaxonSet.NADH3_pos1" spec="TaxonSet">
<alignment id="NADH3_pos1" spec="FilteredAlignment" data="@Mytilidae27sq12PCGs123ps" filter="6504-6851\3"/>
</taxa>
</trait>
<taxonset idref="TaxonSet.NADH3_pos1"/>
</tree>
<tree id="Tree.t:NADH2_pos2" spec="beast.evolution.tree.Tree" name="stateNode">
<trait id="dateTrait.t:NADH2_pos2" spec="beast.evolution.tree.TraitSet" traitname="date">
Mytilus_californianusF-JX486124=0,

Mytilus_coruscusKart1=0,
Mytilus_coruscusKJ577549=0,
Mytilus_chilensisKP100300=0,
Mytilus_chilensisNC030633=0,
Mytilus_edulisF-MF407676=0,
Mytilus_galloprovincialisF-FJ890=0,
Mytilus_trossulusF-GU936625=0,
Mytilus_trossulusF-HM462080=0,
Arcuatula_senhousia1=0,
Musculista_senhousiaGU001953=0,
Bathymodiolus_childressiNC059707=0,
Bathymodiolus_japonicusAP014560=0,
Bathymodiolus_securiformisNC0395=0,
Brachidontes_exustusKM233636=0,
Crenomytilus_grayanusNC044128=0,
Gregariella_coralliophagaNC04412=0,
Modiolus_kurilensisKY242717=0,
Modiolus_modiolusKX821782=0,
Mytilisepta_keenaeNC044127=0,
Mytilisepta_virgataKX094521=0,
Perna_canaliculusMG766134=0,
Perna_canaliculusMK775558=0,
Perna_pernaOK576481=0,
Perna_viridisJQ970425=0,
Perna_viridisMW727515=0,
Septifer_bilocularisMK721549=0 <taxa id="TaxonSet.NADH2_pos2" spec="TaxonSet">
<alignment id="NADH2_pos2" spec="FilteredAlignment" data="@Mytilidae27sq12PCGs123ps" filter="5575-6503\3"/>
</taxa>
</trait>
<taxonset idref="TaxonSet.NADH2_pos2"/>
</tree>
<tree id="Tree.t:CYTB_pos2" spec="beast.evolution.tree.Tree" name="stateNode">
<trait id="dateTrait.t:CYTB_pos2" spec="beast.evolution.tree.TraitSet" traitname="date">
Mytilus_californianusF-JX486124=0,
Mytilus_coruscusKart1=0,
Mytilus_coruscusKJ577549=0,
Mytilus_chilensisKP100300=0,
Mytilus_chilensisNC030633=0,
Mytilus_edulisF-MF407676=0,
Mytilus_galloprovincialisF-FJ890=0,
Mytilus_trossulusF-GU936625=0,
Mytilus_trossulusF-HM462080=0,
Arcuatula_senhousia1=0,
Musculista_senhousiaGU001953=0,
Bathymodiolus_childressiNC059707=0,
Bathymodiolus_japonicusAP014560=0,
Bathymodiolus_securiformisNC0395=0,
Brachidontes_exustusKM233636=0,
Crenomytilus_grayanusNC044128=0,
Gregariella_coralliophagaNC04412=0,
Modiolus_kurilensisKY242717=0,
Modiolus_modiolusKX821782=0,
Mytilisepta_keenaeNC044127=0,
Mytilisepta_virgataKX094521=0,
Perna_canaliculusMG766134=0,
Perna_canaliculusMK775558=0,
Perna_pernaOK576481=0,
Perna_viridisJQ970425=0,
Perna_viridisMW727515=0,
Septifer_bilocularisMK721549=0 <taxa id="TaxonSet.CYTB_pos2" spec="TaxonSet">
<alignment id="CYTB_pos2" spec="FilteredAlignment" data="@Mytilidae27sq12PCGs123ps" filter="3557-4680\3"/>

</taxa>
</trait>
<taxonset idref="TaxonSet.CYTB_pos2"/>
</tree>
<tree id="Tree.t:atp6_pos3" spec="beast.evolution.tree.Tree" name="stateNode">
<trait id="dateTrait.t:atp6_pos3" spec="beast.evolution.tree.TraitSet" traitname="date" value="">
<taxa id="TaxonSet.atp6_pos3" spec="TaxonSet">
<alignment id="atp6_pos3" spec="FilteredAlignment" data="@Mytilidae27sq12PCGs123ps" filter="3-
663\3"/>
</taxa>
</trait>
<taxonset idref="TaxonSet.atp6_pos3"/>
</tree>
<tree id="Tree.t:COX1_pos1" spec="beast.evolution.tree.Tree" name="stateNode">
<trait id="dateTrait.t:COX1_pos1" spec="beast.evolution.tree.TraitSet" traitname="date">
Mytilus_californianusF-JX486124=0,
Mytilus_coruscusKart1=0,
Mytilus_coruscusKJ577549=0,
Mytilus_chilensisKP100300=0,
Mytilus_chilensisNC030633=0,
Mytilus_edulisF-MF407676=0,
Mytilus_galloprovincialisF-FJ890=0,
Mytilus_trossulusF-GU936625=0,
Mytilus_trossulusF-HM462080=0,
Arcuatula_senhousia1=0,
Musculista_senhousiaGU001953=0,
Bathymodiolus_childressiNC059707=0,
Bathymodiolus_japonicusAP014560=0,
Bathymodiolus_securiformisNC0395=0,
Brachidontes_exustusKM233636=0,
Crenomytilus_grayanusNC044128=0,
Gregariella_coralliophagaNC04412=0,
Modiolus_kurilensisKY242717=0,
Modiolus_modiolusKX821782=0,
Mytilisepta_keenaeNC044127=0,
Mytilisepta_virgataKX094521=0,
Perna_canaliculusMG766134=0,
Perna_canaliculusMK775558=0,
Perna_pernaOK576481=0,
Perna_viridisJQ970425=0,
Perna_viridisMW727515=0,
Septifer_bilocularisMK721549=0 <taxa id="TaxonSet.COX1_pos1" spec="TaxonSet">
<alignment id="COX1_pos1" spec="FilteredAlignment" data="@Mytilidae27sq12PCGs123ps"
filter="664-2088\3"/>
</taxa>
</trait>
<taxonset idref="TaxonSet.COX1_pos1"/>
</tree>
<tree id="Tree.t:CYTB_pos1" spec="beast.evolution.tree.Tree" name="stateNode">
<trait id="dateTrait.t:CYTB_pos1" spec="beast.evolution.tree.TraitSet" traitname="date">
Mytilus_californianusF-JX486124=0,
Mytilus_coruscusKart1=0,
Mytilus_coruscusKJ577549=0,
Mytilus_chilensisKP100300=0,
Mytilus_chilensisNC030633=0,
Mytilus_edulisF-MF407676=0,
Mytilus_galloprovincialisF-FJ890=0,
Mytilus_trossulusF-GU936625=0,
Mytilus_trossulusF-HM462080=0,
Arcuatula_senhousia1=0,
Musculista_senhousiaGU001953=0,
Bathymodiolus_childressiNC059707=0,
Bathymodiolus_japonicusAP014560=0,

Bathymodiolus_securiformisNC0395=0,
Brachidontes_exustusKM233636=0,
Crenomytilus_grayanusNC044128=0,
Gregariella_coralliophagaNC04412=0,
Modiolus_kurilensisKY242717=0,
Modiolus_modiolusKX821782=0,
Mytilisepta_keenaeNC044127=0,
Mytilisepta_virgataKX094521=0,
Perna_canaliculusMG766134=0,
Perna_canaliculusMK775558=0,
Perna_pernaOK576481=0,
Perna_viridisJQ970425=0,
Perna_viridisMW727515=0,
Septifer_bilocularisMK721549=0 <taxa id="TaxonSet.CYTB_pos1" spec="TaxonSet">
<alignment id="CYTB_pos1" spec="FilteredAlignment" data="@Mytilidae27sq12PCGs123ps" filter="3556-4680\3"/>
</taxa>
</trait>
<taxonset idref="TaxonSet.CYTB_pos1"/>
</tree>
<tree id="Tree.t:NADH4_pos3" spec="beast.evolution.tree.Tree" name="stateNode">
<trait id="dateTrait.t:NADH4_pos3" spec="beast.evolution.tree.TraitSet" traitname="date" value="">
<taxa id="TaxonSet.NADH4_pos3" spec="TaxonSet">
<alignment id="NADH4_pos3" spec="FilteredAlignment" data="@Mytilidae27sq12PCGs123ps" filter="6854-8126\3"/>
</taxa>
</trait>
<taxonset idref="TaxonSet.NADH4_pos3"/>
</tree>
<tree id="Tree.t:COX1_pos3" spec="beast.evolution.tree.Tree" name="stateNode">
<trait id="dateTrait.t:COX1_pos3" spec="beast.evolution.tree.TraitSet" traitname="date" value="">
<taxa id="TaxonSet.COX1_pos3" spec="TaxonSet">
<alignment id="COX1_pos3" spec="FilteredAlignment" data="@Mytilidae27sq12PCGs123ps" filter="666-2088\3"/>
</taxa>
</trait>
<taxonset idref="TaxonSet.COX1_pos3"/>
</tree>
<tree id="Tree.t:COX2_pos2" spec="beast.evolution.tree.Tree" name="stateNode">
<trait id="dateTrait.t:COX2_pos2" spec="beast.evolution.tree.TraitSet" traitname="date">
Mytilus_californianusF-JX486124=0,
Mytilus_coruscusKart1=0,
Mytilus_coruscusKJ577549=0,
Mytilus_chilensisKP100300=0,
Mytilus_chilensisNC030633=0,
Mytilus_edulisF-MF407676=0,
Mytilus_galloprovincialisF-FJ890=0,
Mytilus_trossulusF-GU936625=0,
Mytilus_trossulusF-HM462080=0,
Arcuatula_senhousia1=0,
Musculista_senhousiaGU001953=0,
Bathymodiolus_childressiNC059707=0,
Bathymodiolus_japonicusAP014560=0,
Bathymodiolus_securiformisNC0395=0,
Brachidontes_exustusKM233636=0,
Crenomytilus_grayanusNC044128=0,
Gregariella_coralliophagaNC04412=0,
Modiolus_kurilensisKY242717=0,
Modiolus_modiolusKX821782=0,
Mytilisepta_keenaeNC044127=0,
Mytilisepta_virgataKX094521=0,
Perna_canaliculusMG766134=0,
Perna_canaliculusMK775558=0,

Perna_pernaOK576481=0,
Perna_viridisJQ970425=0,
Perna_viridisMW727515=0,
Septifer_bilocularisMK721549=0 <taxa id="TaxonSet.COX2_pos2" spec="TaxonSet">
<alignment id="COX2_pos2" spec="FilteredAlignment" data="@Mytilidae27sq12PCGs123ps" filter="2090-2781\3"/>
</taxa>
</trait>
<taxonset idref="TaxonSet.COX2_pos2"/>
</tree>
<tree id="Tree.t:CYTB_pos3" spec="beast.evolution.tree.Tree" name="stateNode">
<trait id="dateTrait.t:CYTB_pos3" spec="beast.evolution.tree.TraitSet" traitname="date" value="">
<taxa id="TaxonSet.CYTB_pos3" spec="TaxonSet">
<alignment id="CYTB_pos3" spec="FilteredAlignment" data="@Mytilidae27sq12PCGs123ps" filter="3558-4680\3"/>
</taxa>
</trait>
<taxonset idref="TaxonSet.CYTB_pos3"/>
</tree>
<tree id="Tree.t:NADH5_pos1" spec="beast.evolution.tree.Tree" name="stateNode">
<trait id="dateTrait.t:NADH5_pos1" spec="beast.evolution.tree.TraitSet" traitname="date">
Mytilus_californianusF-JX486124=0,
Mytilus_coruscusKart1=0,
Mytilus_coruscusKJ577549=0,
Mytilus_chilensisKP100300=0,
Mytilus_chilensisNC030633=0,
Mytilus_edulisF-MF407676=0,
Mytilus_galloprovincialisF-FJ890=0,
Mytilus_trossulusF-GU936625=0,
Mytilus_trossulusF-HM462080=0,
Arcuatula_senhousia1=0,
Musculista_senhousiaGU001953=0,
Bathymodiolus_childressiNC059707=0,
Bathymodiolus_japonicusAP014560=0,
Bathymodiolus_securiformisNC0395=0,
Brachidontes_exustusKM233636=0,
Crenomytilus_grayanusNC044128=0,
Gregariella_coralliophagaNC04412=0,
Modiolus_kurilensisKY242717=0,
Modiolus_modiolusKX821782=0,
Mytilisepta_keenaeNC044127=0,
Mytilisepta_virgataKX094521=0,
Perna_canaliculusMG766134=0,
Perna_canaliculusMK775558=0,
Perna_pernaOK576481=0,
Perna_viridisJQ970425=0,
Perna_viridisMW727515=0,
Septifer_bilocularisMK721549=0 <taxa id="TaxonSet.NADH5_pos1" spec="TaxonSet">
<alignment id="NADH5_pos1" spec="FilteredAlignment" data="@Mytilidae27sq12PCGs123ps" filter="8342-9958\3"/>
</taxa>
</trait>
<taxonset idref="TaxonSet.NADH5_pos1"/>
</tree>
<tree id="Tree.t:atp6_pos1" spec="beast.evolution.tree.Tree" name="stateNode">
<trait id="dateTrait.t:atp6_pos1" spec="beast.evolution.tree.TraitSet" traitname="date">

Mytilus_californianusF-JX486124=0,
Mytilus_coruscusKart1=0,
Mytilus_coruscusKJ577549=0,
Mytilus_chilensisKP100300=0,
Mytilus_chilensisNC030633=0,
Mytilus_edulisF-MF407676=0,

Mytilus_galloprovincialisF-FJ890=0,
Mytilus_trossulusF-GU936625=0,
Mytilus_trossulusF-HM462080=0,
Arcuatula_senhousia1=0,
Musculista_senhousiaGU001953=0,
Bathymodiolus_childressiNC059707=0,
Bathymodiolus_japonicusAP014560=0,
Bathymodiolus_securiformisNC0395=0,
Brachidontes_exustusKM233636=0,
Crenomytilus_grayanusNC044128=0,
Gregariella_coralliophagaNC04412=0,
Modiolus_kurilensisKY242717=292.8E6,
Modiolus_modiolusKX821782=292.8E6,
Mytilisepta_keenaeNC044127=0,
Mytilisepta_virgataKX094521=0,
Perna_canaliculusMG766134=0,
Perna_canaliculusMK775558=0,
Perna_pernaOK576481=0,
Perna_viridisJQ970425=0,
Perna_viridisMW727515=0,
Septifer_bilocularisMK721549=0
<taxa id="TaxonSet.atp6_pos1" spec="TaxonSet">
<alignment id="atp6_pos1" spec="FilteredAlignment" data="@Mytilidae27sq12PCGs123ps" filter="1-663\3"/>
</taxa>
</trait>
<taxonset idref="TaxonSet.atp6_pos1"/>
</tree>
<tree id="Tree.t:NADH1_pos3" spec="beast.evolution.tree.Tree" name="stateNode">
<trait id="dateTrait.t:NADH1_pos3" spec="beast.evolution.tree.TraitSet" traitname="date" value="">
<taxa id="TaxonSet.NADH1_pos3" spec="TaxonSet">
<alignment id="NADH1_pos3" spec="FilteredAlignment" data="@Mytilidae27sq12PCGs123ps" filter="4683-5573\3"/>
</taxa>
</trait>
<taxonset idref="TaxonSet.NADH1_pos3"/>
</tree>
<tree id="Tree.t:atp6_pos2" spec="beast.evolution.tree.Tree" name="stateNode">
<trait id="dateTrait.t:atp6_pos2" spec="beast.evolution.tree.TraitSet" traitname="date">

Mytilus_californianusF-JX486124=0,
Mytilus_coruscusKart1=0,
Mytilus_coruscusKJ577549=0,
Mytilus_chilensisKP100300=0,
Mytilus_chilensisNC030633=0,
Mytilus_edulisF-MF407676=0,
Mytilus_galloprovincialisF-FJ890=0,
Mytilus_trossulusF-GU936625=0,
Mytilus_trossulusF-HM462080=0,
Arcuatula_senhousia1=0,
Musculista_senhousiaGU001953=0,
Bathymodiolus_childressiNC059707=0,
Bathymodiolus_japonicusAP014560=0,
Bathymodiolus_securiformisNC0395=0,
Brachidontes_exustusKM233636=0,
Crenomytilus_grayanusNC044128=0,
Gregariella_coralliophagaNC04412=0,
Modiolus_kurilensisKY242717=0,
Modiolus_modiolusKX821782=292.8E6,
Mytilisepta_keenaeNC044127=292.8E6,
Mytilisepta_virgataKX094521=0,
Perna_canaliculusMG766134=0,
Perna_canaliculusMK775558=0,

Perna_pernaOK576481=0,
Perna_viridisJQ970425=0,
Perna_viridisMW727515=0,
Septifer_bilocularisMK721549=0
<taxa id="TaxonSet.atp6_pos2" spec="TaxonSet">
<alignment id="atp6_pos2" spec="FilteredAlignment" data="@Mytilidae27sq12PCGs123ps" filter="2-663\3"/>
</taxa>
</trait>
<taxonset idref="TaxonSet.atp6_pos2"/>
</tree>
<tree id="Tree.t:NADH4_pos2" spec="beast.evolution.tree.Tree" name="stateNode">
<trait id="dateTrait.t:NADH4_pos2" spec="beast.evolution.tree.TraitSet" traitname="date">
Mytilus_californianusF-JX486124=0,
Mytilus_coruscusKart1=0,
Mytilus_coruscusKJ577549=0,
Mytilus_chilensisKP100300=0,
Mytilus_chilensisNC030633=0,
Mytilus_edulisF-MF407676=0,
Mytilus_galloprovincialisF-FJ890=0,
Mytilus_trossulusF-GU936625=0,
Mytilus_trossulusF-HM462080=0,
Arcuatula_senhousia1=0,
Musculista_senhousiaGU001953=0,
Bathymodiolus_childressiNC059707=0,
Bathymodiolus_japonicusAP014560=0,
Bathymodiolus_securiformisNC0395=0,
Brachidontes_exustusKM233636=0,
Crenomytilus_grayanusNC044128=0,
Gregariella_coralliophagaNC04412=0,
Modiolus_kurilensisKY242717=0,
Modiolus_modiolusKX821782=0,
Mytilisepta_keenaeNC044127=0,
Mytilisepta_virgataKX094521=0,
Perna_canaliculusMG766134=0,
Perna_canaliculusMK775558=0,
Perna_pernaOK576481=0,
Perna_viridisJQ970425=0,
Perna_viridisMW727515=0,
Septifer_bilocularisMK721549=0 <taxa id="TaxonSet.NADH4_pos2" spec="TaxonSet">
<alignment id="NADH4_pos2" spec="FilteredAlignment" data="@Mytilidae27sq12PCGs123ps" filter="6853-8126\3"/>
</taxa>
</trait>
<taxonset idref="TaxonSet.NADH4_pos2"/>
</tree>
<tree id="Tree.t:COX3_pos2" spec="beast.evolution.tree.Tree" name="stateNode">
<trait id="dateTrait.t:COX3_pos2" spec="beast.evolution.tree.TraitSet" traitname="date">
Mytilus_californianusF-JX486124=0,
Mytilus_coruscusKart1=0,
Mytilus_coruscusKJ577549=0,
Mytilus_chilensisKP100300=0,
Mytilus_chilensisNC030633=0,
Mytilus_edulisF-MF407676=0,
Mytilus_galloprovincialisF-FJ890=0,
Mytilus_trossulusF-GU936625=0,
Mytilus_trossulusF-HM462080=0,
Arcuatula_senhousia1=0,
Musculista_senhousiaGU001953=0,
Bathymodiolus_childressiNC059707=0,
Bathymodiolus_japonicusAP014560=0,
Bathymodiolus_securiformisNC0395=0,
Brachidontes_exustusKM233636=0,

Crenomytilus_grayanusNC044128=0,
Gregariella_coralliophagaNC04412=0,
Modiolus_kurilensisKY242717=0,
Modiolus_modiolusKX821782=0,
Mytilisepta_keenaeNC044127=0,
Mytilisepta_virgataKX094521=0,
Perna_canaliculusMG766134=0,
Perna_canaliculusMK775558=0,
Perna_pernaOK576481=0,
Perna_viridisJQ970425=0,
Perna_viridisMW727515=0,
Septifer_bilocularisMK721549=0 <taxa id="TaxonSet.COX3_pos2" spec="TaxonSet">
<alignment id="COX3_pos2" spec="FilteredAlignment" data="@Mytilidae27sq12PCGs123ps"
filter="2783-3555\3"/>
</taxa>
</trait>
<taxonset idref="TaxonSet.COX3_pos2"/>
</tree>
<tree id="Tree.t:NADH5_pos3" spec="beast.evolution.tree.Tree" name="stateNode">
<trait id="dateTrait.t:NADH5_pos3" spec="beast.evolution.tree.TraitSet" traitname="date" value="">
<taxa id="TaxonSet.NADH5_pos3" spec="TaxonSet">
<alignment id="NADH5_pos3" spec="FilteredAlignment" data="@Mytilidae27sq12PCGs123ps"
filter="8344-9958\3"/>
</taxa>
</trait>
<taxonset idref="TaxonSet.NADH5_pos3"/>
</tree>
<tree id="Tree.t:NADH6_pos3" spec="beast.evolution.tree.Tree" name="stateNode">
<trait id="dateTrait.t:NADH6_pos3" spec="beast.evolution.tree.TraitSet" traitname="date" value="">
<taxa id="TaxonSet.NADH6_pos3" spec="TaxonSet">
<alignment id="NADH6_pos3" spec="FilteredAlignment" data="@Mytilidae27sq12PCGs123ps"
filter="9961-10381\3"/>
</taxa>
</trait>
<taxonset idref="TaxonSet.NADH6_pos3"/>
</tree>
<tree id="Tree.t:COX3_pos1" spec="beast.evolution.tree.Tree" name="stateNode">
<trait id="dateTrait.t:COX3_pos1" spec="beast.evolution.tree.TraitSet" traitname="date">
Mytilus_californianusF-JX486124=0,
Mytilus_coruscusKart1=0,
Mytilus_coruscusKJ577549=0,
Mytilus_chilensisKP100300=0,
Mytilus_chilensisNC030633=0,
Mytilus_edulisF-MF407676=0,
Mytilus_galloprovincialisF-FJ890=0,
Mytilus_trossulusF-GU936625=0,
Mytilus_trossulusF-HM462080=0,
Arcuatula_senhousia1=0,
Musculista_senhousiaGU001953=0,
Bathymodiolus_childressiNC059707=0,
Bathymodiolus_japonicusAP014560=0,
Bathymodiolus_securiformisNC0395=0,
Brachidontes_exustusKM233636=0,
Crenomytilus_grayanusNC044128=0,
Gregariella_coralliophagaNC04412=0,
Modiolus_kurilensisKY242717=0,
Modiolus_modiolusKX821782=0,
Mytilisepta_keenaeNC044127=0,
Mytilisepta_virgataKX094521=0,
Perna_canaliculusMG766134=0,
Perna_canaliculusMK775558=0,
Perna_pernaOK576481=0,
Perna_viridisJQ970425=0,

Perna_viridisMW727515=0,
Septifer_bilocularisMK721549=0 <taxa id="TaxonSet.COX3_pos1" spec="TaxonSet">
<alignment id="COX3_pos1" spec="FilteredAlignment" data="@Mytilidae27sq12PCGs123ps" filter="2782-3555\3"/>
</taxa>
</trait>
<taxonset idref="TaxonSet.COX3_pos1"/>
</tree>
<tree id="Tree.t:NADH3_pos3" spec="beast.evolution.tree.Tree" name="stateNode">
<trait id="dateTrait.t:NADH3_pos3" spec="beast.evolution.tree.TraitSet" traitname="date" value="">
<taxa id="TaxonSet.NADH3_pos3" spec="TaxonSet">
<alignment id="NADH3_pos3" spec="FilteredAlignment" data="@Mytilidae27sq12PCGs123ps" filter="6506-6851\3"/>
</taxa>
</trait>
<taxonset idref="TaxonSet.NADH3_pos3"/>
</tree>
<tree id="Tree.t:NADH5_pos2" spec="beast.evolution.tree.Tree" name="stateNode">
<trait id="dateTrait.t:NADH5_pos2" spec="beast.evolution.tree.TraitSet" traitname="date">
Mytilus_californianusF-JX486124=0,
Mytilus_coruscusKart1=0,
Mytilus_coruscusKJ577549=0,
Mytilus_chilensisKP100300=0,
Mytilus_chilensisNC030633=0,
Mytilus_edulisF-MF407676=0,
Mytilus_galloprovincialisF-FJ890=0,
Mytilus_trossulusF-GU936625=0,
Mytilus_trossulusF-HM462080=0,
Arcuatula_senhousia1=0,
Musculista_senhousiaGU001953=0,
Bathymodiolus_childressiNC059707=0,
Bathymodiolus_japonicusAP014560=0,
Bathymodiolus_securiformisNC0395=0,
Brachidontes_exustusKM233636=0,
Crenomytilus_grayanusNC044128=0,
Gregariella_coralliophagaNC04412=0,
Modiolus_kurilensisKY242717=0,
Modiolus_modiolusKX821782=0,
Mytilisepta_keenaeNC044127=0,
Mytilisepta_virgataKX094521=0,
Perna_canaliculusMG766134=0,
Perna_canaliculusMK775558=0,
Perna_pernaOK576481=0,
Perna_viridisJQ970425=0,
Perna_viridisMW727515=0,
Septifer_bilocularisMK721549=0 <taxa id="TaxonSet.NADH5_pos2" spec="TaxonSet">
<alignment id="NADH5_pos2" spec="FilteredAlignment" data="@Mytilidae27sq12PCGs123ps" filter="8343-9958\3"/>
</taxa>
</trait>
<taxonset idref="TaxonSet.NADH5_pos2"/>
</tree>
<tree id="Tree.t:NADH6_pos2" spec="beast.evolution.tree.Tree" name="stateNode">
<trait id="dateTrait.t:NADH6_pos2" spec="beast.evolution.tree.TraitSet" traitname="date">
Mytilus_californianusF-JX486124=0,
Mytilus_coruscusKart1=0,
Mytilus_coruscusKJ577549=0,
Mytilus_chilensisKP100300=0,
Mytilus_chilensisNC030633=0,
Mytilus_edulisF-MF407676=0,
Mytilus_galloprovincialisF-FJ890=0,
Mytilus_trossulusF-GU936625=0,
Mytilus_trossulusF-HM462080=0,

Arcuatula_senhousia1=0,
Musculista_senhousiaGU001953=0,
Bathymodiolus_childressiNC059707=0,
Bathymodiolus_japonicusAP014560=0,
Bathymodiolus_securiformisNC0395=0,
Brachidontes_exustusKM233636=0,
Crenomytilus_grayanusNC044128=0,
Gregariella_coralliophagaNC04412=0,
Modiolus_kurilensisKY242717=0,
Modiolus_modiolusKX821782=0,
Mytilisepta_keenaeNC044127=0,
Mytilisepta_virgataKX094521=0,
Perna_canaliculusMG766134=0,
Perna_canaliculusMK775558=0,
Perna_pernaOK576481=0,
Perna_viridisJQ970425=0,
Perna_viridisMW727515=0,
Septifer_bilocularisMK721549=0 <taxa id="TaxonSet.NADH6_pos2" spec="TaxonSet">
<alignment id="NADH6_pos2" spec="FilteredAlignment" data="@Mytilidae27sq12PCGs123ps" filter="9960-10381\3"/>
</taxa>
</trait>
<taxonset idref="TaxonSet.NADH6_pos2"/>
</tree>
<tree id="Tree.t:NADH4L_pos2" spec="beast.evolution.tree.Tree" name="stateNode">
<trait id="dateTrait.t:NADH4L_pos2" spec="beast.evolution.tree.TraitSet" traitname="date">
Mytilus_californianusF-JX486124=0,
Mytilus_coruscusKart1=0,
Mytilus_coruscusKJ577549=0,
Mytilus_chilensisKP100300=0,
Mytilus_chilensisNC030633=0,
Mytilus_edulisF-MF407676=0,
Mytilus_galloprovincialisF-FJ890=0,
Mytilus_trossulusF-GU936625=0,
Mytilus_trossulusF-HM462080=0,
Arcuatula_senhousia1=0,
Musculista_senhousiaGU001953=0,
Bathymodiolus_childressiNC059707=0,
Bathymodiolus_japonicusAP014560=0,
Bathymodiolus_securiformisNC0395=0,
Brachidontes_exustusKM233636=0,
Crenomytilus_grayanusNC044128=0,
Gregariella_coralliophagaNC04412=0,
Modiolus_kurilensisKY242717=0,
Modiolus_modiolusKX821782=0,
Mytilisepta_keenaeNC044127=0,
Mytilisepta_virgataKX094521=0,
Perna_canaliculusMG766134=0,
Perna_canaliculusMK775558=0,
Perna_pernaOK576481=0,
Perna_viridisJQ970425=0,
Perna_viridisMW727515=0,
Septifer_bilocularisMK721549=0 <taxa id="TaxonSet.NADH4L_pos2" spec="TaxonSet">
<alignment id="NADH4L_pos2" spec="FilteredAlignment" data="@Mytilidae27sq12PCGs123ps" filter="8128-8341\3"/>
</taxa>
</trait>
<taxonset idref="TaxonSet.NADH4L_pos2"/>
</tree>
<tree id="Tree.t:COX1_pos2" spec="beast.evolution.tree.Tree" name="stateNode">
<trait id="dateTrait.t:COX1_pos2" spec="beast.evolution.tree.TraitSet" traitname="date">
Mytilus_californianusF-JX486124=0,
Mytilus_coruscusKart1=0,

Mytilus_coruscusKJ577549=0,
Mytilus_chilensisKP100300=0,
Mytilus_chilensisNC030633=0,
Mytilus_edulisF-MF407676=0,
Mytilus_galloprovincialisF-FJ890=0,
Mytilus_trossulusF-GU936625=0,
Mytilus_trossulusF-HM462080=0,
Arcuatula_senhousia1=0,
Musculista_senhousiaGU001953=0,
Bathymodiolus_childressiNC059707=0,
Bathymodiolus_japonicusAP014560=0,
Bathymodiolus_securiformisNC0395=0,
Brachidontes_exustusKM233636=0,
Crenomytilus_grayanusNC044128=0,
Gregariella_coralliophagaNC04412=0,
Modiolus_kurilensisKY242717=0,
Modiolus_modiolusKX821782=0,
Mytilisepta_keenaeNC044127=0,
Mytilisepta_virgataKX094521=0,
Perna_canaliculusMG766134=0,
Perna_canaliculusMK775558=0,
Perna_pernaOK576481=0,
Perna_viridisJQ970425=0,
Perna_viridisMW727515=0,
Septifer_bilocularisMK721549=0 <taxa id="TaxonSet.COX1_pos2" spec="TaxonSet">
<alignment id="COX1_pos2" spec="FilteredAlignment" data="@Mytilidae27sq12PCGs123ps" filter="665-2088\3"/>
</taxa>
</trait>
<taxonset idref="TaxonSet.COX1_pos2"/>
</tree>
<tree id="Tree.t:COX2_pos3" spec="beast.evolution.tree.Tree" name="stateNode">
<trait id="dateTrait.t:COX2_pos3" spec="beast.evolution.tree.TraitSet" traitname="date" value="">
<taxa id="TaxonSet.COX2_pos3" spec="TaxonSet">
<alignment id="COX2_pos3" spec="FilteredAlignment" data="@Mytilidae27sq12PCGs123ps" filter="2091-2781\3"/>
</taxa>
</trait>
<taxonset idref="TaxonSet.COX2_pos3"/>
</tree>
<tree id="Tree.t:COX3_pos3" spec="beast.evolution.tree.Tree" name="stateNode">
<trait id="dateTrait.t:COX3_pos3" spec="beast.evolution.tree.TraitSet" traitname="date" value="">
<taxa id="TaxonSet.COX3_pos3" spec="TaxonSet">
<alignment id="COX3_pos3" spec="FilteredAlignment" data="@Mytilidae27sq12PCGs123ps" filter="2784-3555\3"/>
</taxa>
</trait>
<taxonset idref="TaxonSet.COX3_pos3"/>
</tree>
<tree id="Tree.t:Mytilidae27sq12PCGs" spec="beast.evolution.tree.Tree" name="stateNode">
<trait id="dateTrait.t:Mytilidae27sq12PCGs" spec="beast.evolution.tree.TraitSet" traitname="date">

Arcuatula_senhousia1=0,
Bathymodiolus_childressiNC059707=0,
Bathymodiolus_japonicusAP014560=0,
Bathymodiolus_securiformisNC0395=0,
Brachidontes_exustusKM233636=0,
Crenomytilus_grayanusNC044128=0,
Gregariella_coralliophagaNC04412=0,
Modiolus_kurilensisKY242717=0,
Modiolus_modiolusKX821782=0,
Musculista_senhousiaGU001953=0,

Mytilisepta_keenaeNC044127=0,
Mytilisepta_virgataKX094521=0,
Mytilus_californianusF-JX486124=0,
Mytilus_chilensisKP100300=0,
Mytilus_chilensisNC030633=0,
Mytilus_coruscusKJ577549=0,
Mytilus_coruscusKart1=0,
Mytilus_edulisF-MF407676=0,
Mytilus_galloprovincialisF-FJ890=0,
Mytilus_trossulusF-GU936625=0,
Mytilus_trossulusF-HM462080=0,
Perna_canaliculusMG766134=0,
Perna_canaliculusMK775558=0,
Perna_pernaOK576481=0,
Perna_viridisJQ970425=0,
Perna_viridisMW727515=0,
Septifer_bilocularisMK721549=0

<taxa id="TaxonSet.Mytilidae27sq12PCGs" spec="TaxonSet">
<alignment idref="Mytilidae27sq12PCGs"/>
</taxa>
</trait>
<taxonset idref="TaxonSet.Mytilidae27sq12PCGs"/>
</tree>
<parameter id="clockRate.c:COX2_pos1" spec="parameter.RealParameter" name="stateNode">2.93E-
5</parameter>
<parameter id="clockRate.c:COX3_pos1" spec="parameter.RealParameter" name="stateNode">2.93E-
5</parameter>
<parameter id="clockRate.c:NADH2_pos2" spec="parameter.RealParameter" name="stateNode">2.93E-
5</parameter>
<parameter id="clockRate.c:NADH4_pos3" spec="parameter.RealParameter"
name="stateNode">1.0</parameter>
<parameter id="clockRate.c:NADH4L_pos1" spec="parameter.RealParameter" name="stateNode">2.93E-
5</parameter>
<parameter id="clockRate.c:NADH5_pos2" spec="parameter.RealParameter" name="stateNode">2.93E-
5</parameter>
<parameter id="clockRate.c:NADH6_pos3" spec="parameter.RealParameter"
name="stateNode">1.0</parameter>
<parameter id="clockRate.c:COX1_pos1" spec="parameter.RealParameter" name="stateNode">2.93E-
5</parameter>
<parameter id="clockRate.c:COX1_pos3" spec="parameter.RealParameter"
name="stateNode">1.0</parameter>
<parameter id="clockRate.c:Mytilidae27sq12PCGs" spec="parameter.RealParameter"
name="stateNode">2.93E-5</parameter>
<parameter id="clockRate.c:atp6_pos1" spec="parameter.RealParameter" name="stateNode">2.93E-
5</parameter>
<parameter id="clockRate.c:NADH4_pos2" spec="parameter.RealParameter" name="stateNode">2.93E-
5</parameter>
<parameter id="clockRate.c:NADH1_pos1" spec="parameter.RealParameter" name="stateNode">2.93E-
5</parameter>
<parameter id="clockRate.c:NADH6_pos1" spec="parameter.RealParameter" name="stateNode">2.93E-
5</parameter>
<parameter id="clockRate.c:COX1_pos2" spec="parameter.RealParameter" name="stateNode">2.93E-
5</parameter>
<parameter id="clockRate.c:NADH4L_pos3" spec="parameter.RealParameter"
name="stateNode">1.0</parameter>
<parameter id="clockRate.c:COX2_pos3" spec="parameter.RealParameter"
name="stateNode">1.0</parameter>
<parameter id="clockRate.c:COX3_pos2" spec="parameter.RealParameter" name="stateNode">2.93E-
5</parameter>
<parameter id="clockRate.c:CYTB_pos2" spec="parameter.RealParameter" name="stateNode">2.93E-
5</parameter>

```xml
<parameter id="clockRate.c:atp6_pos2" spec="parameter.RealParameter" name="stateNode">2.93E-5</parameter>
<parameter id="clockRate.c:COX3_pos3" spec="parameter.RealParameter" name="stateNode">1.0</parameter>
<parameter id="clockRate.c:CYTB_pos3" spec="parameter.RealParameter" name="stateNode">1.0</parameter>
<parameter id="clockRate.c:NADH4L_pos2" spec="parameter.RealParameter" name="stateNode">2.93E-5</parameter>
<parameter id="clockRate.c:NADH6_pos2" spec="parameter.RealParameter" name="stateNode">2.93E-5</parameter>
<parameter id="clockRate.c:NADH1_pos2" spec="parameter.RealParameter" name="stateNode">2.93E-5</parameter>
<parameter id="clockRate.c:NADH4_pos1" spec="parameter.RealParameter" name="stateNode">2.93E-5</parameter>
<parameter id="clockRate.c:atp6_pos3" spec="parameter.RealParameter" name="stateNode">1.0</parameter>
<parameter id="clockRate.c:COX2_pos2" spec="parameter.RealParameter" name="stateNode">2.93E-5</parameter>
<parameter id="clockRate.c:NADH2_pos1" spec="parameter.RealParameter" name="stateNode">2.93E-5</parameter>
<parameter id="clockRate.c:NADH5_pos3" spec="parameter.RealParameter" name="stateNode">1.0</parameter>
<parameter id="clockRate.c:NADH5_pos1" spec="parameter.RealParameter" name="stateNode">2.93E-5</parameter>
<parameter id="clockRate.c:NADH3_pos3" spec="parameter.RealParameter" name="stateNode">1.0</parameter>
<parameter id="clockRate.c:NADH2_pos3" spec="parameter.RealParameter" name="stateNode">1.0</parameter>
<parameter id="clockRate.c:NADH3_pos2" spec="parameter.RealParameter" name="stateNode">2.93E-5</parameter>
<parameter id="clockRate.c:NADH3_pos1" spec="parameter.RealParameter" name="stateNode">2.93E-5</parameter>
<parameter id="clockRate.c:CYTB_pos1" spec="parameter.RealParameter" name="stateNode">2.93E-5</parameter>
<parameter id="clockRate.c:NADH1_pos3" spec="parameter.RealParameter" name="stateNode">1.0</parameter>
<parameter id="proportionInvariant.s:atp6_pos1" spec="parameter.RealParameter" lower="0.0" name="stateNode" upper="1.0">0.126</parameter>
<parameter id="gammaShape.s:atp6_pos1" spec="parameter.RealParameter" name="stateNode">1.0</parameter>
<parameter id="rateAC.s:atp6_pos1" spec="parameter.RealParameter" lower="0.0" name="stateNode">1.0</parameter>
<parameter id="rateAG.s:atp6_pos1" spec="parameter.RealParameter" lower="0.0" name="stateNode">1.0</parameter>
<parameter id="rateAT.s:atp6_pos1" spec="parameter.RealParameter" lower="0.0" name="stateNode">1.0</parameter>
<parameter id="rateCG.s:atp6_pos1" spec="parameter.RealParameter" lower="0.0" name="stateNode">1.0</parameter>
<parameter id="rateGT.s:atp6_pos1" spec="parameter.RealParameter" lower="0.0" name="stateNode">1.0</parameter>
<parameter id="rateAC.s:atp6_pos2" spec="parameter.RealParameter" lower="0.0" name="stateNode">1.0</parameter>
<parameter id="rateAG.s:atp6_pos2" spec="parameter.RealParameter" lower="0.0" name="stateNode">1.0</parameter>
<parameter id="rateAT.s:atp6_pos2" spec="parameter.RealParameter" lower="0.0" name="stateNode">1.0</parameter>
<parameter id="rateCG.s:atp6_pos2" spec="parameter.RealParameter" lower="0.0" name="stateNode">1.0</parameter>
<parameter id="rateGT.s:atp6_pos2" spec="parameter.RealParameter" lower="0.0" name="stateNode">1.0</parameter>
<parameter id="proportionInvariant.s:atp6_pos2" spec="parameter.RealParameter" lower="0.0" name="stateNode" upper="1.0">0.126</parameter>
<parameter id="gammaShape.s:atp6_pos2" spec="parameter.RealParameter" name="stateNode">1.0</parameter>
```

```
<parameter id="gammaShape.s:atp6_pos3" spec="parameter.RealParameter" name="stateNode">1.0</parameter>
<parameter id="rateAC.s:atp6_pos3" spec="parameter.RealParameter" lower="0.0" name="stateNode">1.0</parameter>
<parameter id="rateAG.s:atp6_pos3" spec="parameter.RealParameter" lower="0.0" name="stateNode">1.0</parameter>
<parameter id="rateAT.s:atp6_pos3" spec="parameter.RealParameter" lower="0.0" name="stateNode">1.0</parameter>
<parameter id="rateCG.s:atp6_pos3" spec="parameter.RealParameter" lower="0.0" name="stateNode">1.0</parameter>
<parameter id="rateGT.s:atp6_pos3" spec="parameter.RealParameter" lower="0.0" name="stateNode">1.0</parameter>
<parameter id="gammaShape.s:Mytilidae27sq12PCGs" spec="parameter.RealParameter" name="stateNode">1.0</parameter>
<parameter id="gammaShape.s:COX1_pos1" spec="parameter.RealParameter" name="stateNode">1.0</parameter>
<parameter id="rateAC.s:COX1_pos1" spec="parameter.RealParameter" lower="0.0" name="stateNode">1.0</parameter>
<parameter id="rateAG.s:COX1_pos1" spec="parameter.RealParameter" lower="0.0" name="stateNode">1.0</parameter>
<parameter id="rateAT.s:COX1_pos1" spec="parameter.RealParameter" lower="0.0" name="stateNode">1.0</parameter>
<parameter id="rateCG.s:COX1_pos1" spec="parameter.RealParameter" lower="0.0" name="stateNode">1.0</parameter>
<parameter id="rateGT.s:COX1_pos1" spec="parameter.RealParameter" lower="0.0" name="stateNode">1.0</parameter>
<parameter id="gammaShape.s:COX1_pos2" spec="parameter.RealParameter" name="stateNode">1.0</parameter>
<parameter id="gammaShape.s:COX1_pos3" spec="parameter.RealParameter" name="stateNode">1.0</parameter>
<parameter id="rateAC.s:COX1_pos3" spec="parameter.RealParameter" lower="0.0" name="stateNode">1.0</parameter>
<parameter id="rateAG.s:COX1_pos3" spec="parameter.RealParameter" lower="0.0" name="stateNode">1.0</parameter>
<parameter id="rateAT.s:COX1_pos3" spec="parameter.RealParameter" lower="0.0" name="stateNode">1.0</parameter>
<parameter id="rateCG.s:COX1_pos3" spec="parameter.RealParameter" lower="0.0" name="stateNode">1.0</parameter>
<parameter id="rateGT.s:COX1_pos3" spec="parameter.RealParameter" lower="0.0" name="stateNode">1.0</parameter>
<parameter id="gammaShape.s:COX2_pos1" spec="parameter.RealParameter" name="stateNode">1.0</parameter>
<parameter id="rateAC.s:COX2_pos1" spec="parameter.RealParameter" lower="0.0" name="stateNode">1.0</parameter>
<parameter id="rateAG.s:COX2_pos1" spec="parameter.RealParameter" lower="0.0" name="stateNode">1.0</parameter>
<parameter id="rateAT.s:COX2_pos1" spec="parameter.RealParameter" lower="0.0" name="stateNode">1.0</parameter>
<parameter id="rateCG.s:COX2_pos1" spec="parameter.RealParameter" lower="0.0" name="stateNode">1.0</parameter>
<parameter id="rateGT.s:COX2_pos1" spec="parameter.RealParameter" lower="0.0" name="stateNode">1.0</parameter>
<parameter id="gammaShape.s:COX2_pos2" spec="parameter.RealParameter" name="stateNode">1.0</parameter>
<parameter id="rateAC.s:COX2_pos3" spec="parameter.RealParameter" lower="0.0" name="stateNode">1.0</parameter>
<parameter id="rateAG.s:COX2_pos3" spec="parameter.RealParameter" lower="0.0" name="stateNode">1.0</parameter>
<parameter id="rateAT.s:COX2_pos3" spec="parameter.RealParameter" lower="0.0" name="stateNode">1.0</parameter>
<parameter id="rateCG.s:COX2_pos3" spec="parameter.RealParameter" lower="0.0" name="stateNode">1.0</parameter>
```

```xml
<parameter id="rateGT.s:COX2_pos3" spec="parameter.RealParameter" lower="0.0" name="stateNode">1.0</parameter>
<parameter id="gammaShape.s:COX2_pos3" spec="parameter.RealParameter" name="stateNode">1.0</parameter>
<parameter id="gammaShape.s:COX3_pos1" spec="parameter.RealParameter" name="stateNode">1.0</parameter>
<parameter id="rateAC.s:COX3_pos1" spec="parameter.RealParameter" lower="0.0" name="stateNode">1.0</parameter>
<parameter id="rateAG.s:COX3_pos1" spec="parameter.RealParameter" lower="0.0" name="stateNode">1.0</parameter>
<parameter id="rateAT.s:COX3_pos1" spec="parameter.RealParameter" lower="0.0" name="stateNode">1.0</parameter>
<parameter id="rateCG.s:COX3_pos1" spec="parameter.RealParameter" lower="0.0" name="stateNode">1.0</parameter>
<parameter id="rateGT.s:COX3_pos1" spec="parameter.RealParameter" lower="0.0" name="stateNode">1.0</parameter>
<parameter id="gammaShape.s:COX3_pos2" spec="parameter.RealParameter" name="stateNode">1.0</parameter>
<parameter id="rateAC.s:COX3_pos2" spec="parameter.RealParameter" lower="0.0" name="stateNode">1.0</parameter>
<parameter id="rateAG.s:COX3_pos2" spec="parameter.RealParameter" lower="0.0" name="stateNode">1.0</parameter>
<parameter id="rateAT.s:COX3_pos2" spec="parameter.RealParameter" lower="0.0" name="stateNode">1.0</parameter>
<parameter id="rateCG.s:COX3_pos2" spec="parameter.RealParameter" lower="0.0" name="stateNode">1.0</parameter>
<parameter id="rateGT.s:COX3_pos2" spec="parameter.RealParameter" lower="0.0" name="stateNode">1.0</parameter>
<parameter id="gammaShape.s:COX3_pos3" spec="parameter.RealParameter" name="stateNode">1.0</parameter>
<parameter id="rateAC.s:COX3_pos3" spec="parameter.RealParameter" lower="0.0" name="stateNode">1.0</parameter>
<parameter id="rateAG.s:COX3_pos3" spec="parameter.RealParameter" lower="0.0" name="stateNode">1.0</parameter>
<parameter id="rateAT.s:COX3_pos3" spec="parameter.RealParameter" lower="0.0" name="stateNode">1.0</parameter>
<parameter id="rateCG.s:COX3_pos3" spec="parameter.RealParameter" lower="0.0" name="stateNode">1.0</parameter>
<parameter id="rateGT.s:COX3_pos3" spec="parameter.RealParameter" lower="0.0" name="stateNode">1.0</parameter>
<parameter id="gammaShape.s:CYTB_pos1" spec="parameter.RealParameter" name="stateNode">1.0</parameter>
<parameter id="rateAC.s:CYTB_pos1" spec="parameter.RealParameter" lower="0.0" name="stateNode">1.0</parameter>
<parameter id="rateAG.s:CYTB_pos1" spec="parameter.RealParameter" lower="0.0" name="stateNode">1.0</parameter>
<parameter id="rateAT.s:CYTB_pos1" spec="parameter.RealParameter" lower="0.0" name="stateNode">1.0</parameter>
<parameter id="rateCG.s:CYTB_pos1" spec="parameter.RealParameter" lower="0.0" name="stateNode">1.0</parameter>
<parameter id="rateGT.s:CYTB_pos1" spec="parameter.RealParameter" lower="0.0" name="stateNode">1.0</parameter>
<parameter id="gammaShape.s:CYTB_pos2" spec="parameter.RealParameter" name="stateNode">1.0</parameter>
<parameter id="rateAC.s:CYTB_pos2" spec="parameter.RealParameter" lower="0.0" name="stateNode">1.0</parameter>
<parameter id="rateAG.s:CYTB_pos2" spec="parameter.RealParameter" lower="0.0" name="stateNode">1.0</parameter>
<parameter id="rateAT.s:CYTB_pos2" spec="parameter.RealParameter" lower="0.0" name="stateNode">1.0</parameter>
<parameter id="rateCG.s:CYTB_pos2" spec="parameter.RealParameter" lower="0.0" name="stateNode">1.0</parameter>
```

```
        <parameter id="rateGT.s:CYTB_pos2" spec="parameter.RealParameter" lower="0.0"
name="stateNode">1.0</parameter>
        <parameter id="gammaShape.s:CYTB_pos3" spec="parameter.RealParameter"
name="stateNode">1.0</parameter>
        <parameter id="rateAC.s:CYTB_pos3" spec="parameter.RealParameter" lower="0.0"
name="stateNode">1.0</parameter>
        <parameter id="rateAG.s:CYTB_pos3" spec="parameter.RealParameter" lower="0.0"
name="stateNode">1.0</parameter>
        <parameter id="rateAT.s:CYTB_pos3" spec="parameter.RealParameter" lower="0.0"
name="stateNode">1.0</parameter>
        <parameter id="rateCG.s:CYTB_pos3" spec="parameter.RealParameter" lower="0.0"
name="stateNode">1.0</parameter>
        <parameter id="rateGT.s:CYTB_pos3" spec="parameter.RealParameter" lower="0.0"
name="stateNode">1.0</parameter>
        <parameter id="gammaShape.s:NADH1_pos1" spec="parameter.RealParameter"
name="stateNode">1.0</parameter>
        <parameter id="rateAC.s:NADH1_pos1" spec="parameter.RealParameter" lower="0.0"
name="stateNode">1.0</parameter>
        <parameter id="rateAG.s:NADH1_pos1" spec="parameter.RealParameter" lower="0.0"
name="stateNode">1.0</parameter>
        <parameter id="rateAT.s:NADH1_pos1" spec="parameter.RealParameter" lower="0.0"
name="stateNode">1.0</parameter>
        <parameter id="rateCG.s:NADH1_pos1" spec="parameter.RealParameter" lower="0.0"
name="stateNode">1.0</parameter>
        <parameter id="rateGT.s:NADH1_pos1" spec="parameter.RealParameter" lower="0.0"
name="stateNode">1.0</parameter>
        <parameter id="gammaShape.s:NADH1_pos2" spec="parameter.RealParameter"
name="stateNode">1.0</parameter>
        <parameter id="rateAC.s:NADH1_pos2" spec="parameter.RealParameter" lower="0.0"
name="stateNode">1.0</parameter>
        <parameter id="rateAG.s:NADH1_pos2" spec="parameter.RealParameter" lower="0.0"
name="stateNode">1.0</parameter>
        <parameter id="rateAT.s:NADH1_pos2" spec="parameter.RealParameter" lower="0.0"
name="stateNode">1.0</parameter>
        <parameter id="rateCG.s:NADH1_pos2" spec="parameter.RealParameter" lower="0.0"
name="stateNode">1.0</parameter>
        <parameter id="rateGT.s:NADH1_pos2" spec="parameter.RealParameter" lower="0.0"
name="stateNode">1.0</parameter>
        <parameter id="gammaShape.s:NADH1_pos3" spec="parameter.RealParameter"
name="stateNode">1.0</parameter>
        <parameter id="rateAC.s:NADH1_pos3" spec="parameter.RealParameter" lower="0.0"
name="stateNode">1.0</parameter>
        <parameter id="rateAG.s:NADH1_pos3" spec="parameter.RealParameter" lower="0.0"
name="stateNode">1.0</parameter>
        <parameter id="rateAT.s:NADH1_pos3" spec="parameter.RealParameter" lower="0.0"
name="stateNode">1.0</parameter>
        <parameter id="rateCG.s:NADH1_pos3" spec="parameter.RealParameter" lower="0.0"
name="stateNode">1.0</parameter>
        <parameter id="rateGT.s:NADH1_pos3" spec="parameter.RealParameter" lower="0.0"
name="stateNode">1.0</parameter>
        <parameter id="gammaShape.s:NADH2_pos1" spec="parameter.RealParameter"
name="stateNode">1.0</parameter>
        <parameter id="rateAC.s:NADH2_pos1" spec="parameter.RealParameter" lower="0.0"
name="stateNode">1.0</parameter>
        <parameter id="rateAG.s:NADH2_pos1" spec="parameter.RealParameter" lower="0.0"
name="stateNode">1.0</parameter>
        <parameter id="rateAT.s:NADH2_pos1" spec="parameter.RealParameter" lower="0.0"
name="stateNode">1.0</parameter>
        <parameter id="rateCG.s:NADH2_pos1" spec="parameter.RealParameter" lower="0.0"
name="stateNode">1.0</parameter>
        <parameter id="rateGT.s:NADH2_pos1" spec="parameter.RealParameter" lower="0.0"
name="stateNode">1.0</parameter>
```

```
<parameter id="gammaShape.s:NADH2_pos2" spec="parameter.RealParameter" name="stateNode">1.0</parameter>
<parameter id="rateAC.s:NADH2_pos2" spec="parameter.RealParameter" lower="0.0" name="stateNode">1.0</parameter>
<parameter id="rateAG.s:NADH2_pos2" spec="parameter.RealParameter" lower="0.0" name="stateNode">1.0</parameter>
<parameter id="rateAT.s:NADH2_pos2" spec="parameter.RealParameter" lower="0.0" name="stateNode">1.0</parameter>
<parameter id="rateCG.s:NADH2_pos2" spec="parameter.RealParameter" lower="0.0" name="stateNode">1.0</parameter>
<parameter id="rateGT.s:NADH2_pos2" spec="parameter.RealParameter" lower="0.0" name="stateNode">1.0</parameter>
<parameter id="gammaShape.s:NADH2_pos3" spec="parameter.RealParameter" name="stateNode">1.0</parameter>
<parameter id="rateAC.s:NADH2_pos3" spec="parameter.RealParameter" lower="0.0" name="stateNode">1.0</parameter>
<parameter id="rateAG.s:NADH2_pos3" spec="parameter.RealParameter" lower="0.0" name="stateNode">1.0</parameter>
<parameter id="rateAT.s:NADH2_pos3" spec="parameter.RealParameter" lower="0.0" name="stateNode">1.0</parameter>
<parameter id="rateCG.s:NADH2_pos3" spec="parameter.RealParameter" lower="0.0" name="stateNode">1.0</parameter>
<parameter id="rateGT.s:NADH2_pos3" spec="parameter.RealParameter" lower="0.0" name="stateNode">1.0</parameter>
<parameter id="rateAC.s:NADH3_pos1" spec="parameter.RealParameter" lower="0.0" name="stateNode">1.0</parameter>
<parameter id="rateAG.s:NADH3_pos1" spec="parameter.RealParameter" lower="0.0" name="stateNode">1.0</parameter>
<parameter id="rateAT.s:NADH3_pos1" spec="parameter.RealParameter" lower="0.0" name="stateNode">1.0</parameter>
<parameter id="rateCG.s:NADH3_pos1" spec="parameter.RealParameter" lower="0.0" name="stateNode">1.0</parameter>
<parameter id="rateGT.s:NADH3_pos1" spec="parameter.RealParameter" lower="0.0" name="stateNode">1.0</parameter>
<parameter id="gammaShape.s:NADH3_pos1" spec="parameter.RealParameter" name="stateNode">1.0</parameter>
<parameter id="gammaShape.s:NADH3_pos2" spec="parameter.RealParameter" name="stateNode">1.0</parameter>
<parameter id="rateAC.s:NADH3_pos2" spec="parameter.RealParameter" lower="0.0" name="stateNode">1.0</parameter>
<parameter id="rateAG.s:NADH3_pos2" spec="parameter.RealParameter" lower="0.0" name="stateNode">1.0</parameter>
<parameter id="rateAT.s:NADH3_pos2" spec="parameter.RealParameter" lower="0.0" name="stateNode">1.0</parameter>
<parameter id="rateCG.s:NADH3_pos2" spec="parameter.RealParameter" lower="0.0" name="stateNode">1.0</parameter>
<parameter id="rateGT.s:NADH3_pos2" spec="parameter.RealParameter" lower="0.0" name="stateNode">1.0</parameter>
<parameter id="gammaShape.s:NADH3_pos3" spec="parameter.RealParameter" name="stateNode">1.0</parameter>
<parameter id="rateAC.s:NADH3_pos3" spec="parameter.RealParameter" lower="0.0" name="stateNode">1.0</parameter>
<parameter id="rateAG.s:NADH3_pos3" spec="parameter.RealParameter" lower="0.0" name="stateNode">1.0</parameter>
<parameter id="rateAT.s:NADH3_pos3" spec="parameter.RealParameter" lower="0.0" name="stateNode">1.0</parameter>
<parameter id="rateCG.s:NADH3_pos3" spec="parameter.RealParameter" lower="0.0" name="stateNode">1.0</parameter>
<parameter id="rateGT.s:NADH3_pos3" spec="parameter.RealParameter" lower="0.0" name="stateNode">1.0</parameter>
<parameter id="gammaShape.s:NADH4_pos1" spec="parameter.RealParameter" name="stateNode">1.0</parameter>
```

```xml
<parameter id="rateAC.s:NADH4_pos1" spec="parameter.RealParameter" lower="0.0" name="stateNode">1.0</parameter>
<parameter id="rateAG.s:NADH4_pos1" spec="parameter.RealParameter" lower="0.0" name="stateNode">1.0</parameter>
<parameter id="rateAT.s:NADH4_pos1" spec="parameter.RealParameter" lower="0.0" name="stateNode">1.0</parameter>
<parameter id="rateCG.s:NADH4_pos1" spec="parameter.RealParameter" lower="0.0" name="stateNode">1.0</parameter>
<parameter id="rateGT.s:NADH4_pos1" spec="parameter.RealParameter" lower="0.0" name="stateNode">1.0</parameter>
<parameter id="gammaShape.s:NADH4_pos2" spec="parameter.RealParameter" name="stateNode">1.0</parameter>
<parameter id="rateAC.s:NADH4_pos2" spec="parameter.RealParameter" lower="0.0" name="stateNode">1.0</parameter>
<parameter id="rateAG.s:NADH4_pos2" spec="parameter.RealParameter" lower="0.0" name="stateNode">1.0</parameter>
<parameter id="rateAT.s:NADH4_pos2" spec="parameter.RealParameter" lower="0.0" name="stateNode">1.0</parameter>
<parameter id="rateCG.s:NADH4_pos2" spec="parameter.RealParameter" lower="0.0" name="stateNode">1.0</parameter>
<parameter id="rateGT.s:NADH4_pos2" spec="parameter.RealParameter" lower="0.0" name="stateNode">1.0</parameter>
<parameter id="gammaShape.s:NADH4_pos3" spec="parameter.RealParameter" name="stateNode">1.0</parameter>
<parameter id="rateAC.s:NADH4_pos3" spec="parameter.RealParameter" lower="0.0" name="stateNode">1.0</parameter>
<parameter id="rateAG.s:NADH4_pos3" spec="parameter.RealParameter" lower="0.0" name="stateNode">1.0</parameter>
<parameter id="rateAT.s:NADH4_pos3" spec="parameter.RealParameter" lower="0.0" name="stateNode">1.0</parameter>
<parameter id="rateCG.s:NADH4_pos3" spec="parameter.RealParameter" lower="0.0" name="stateNode">1.0</parameter>
<parameter id="rateGT.s:NADH4_pos3" spec="parameter.RealParameter" lower="0.0" name="stateNode">1.0</parameter>
<parameter id="gammaShape.s:NADH4L_pos1" spec="parameter.RealParameter" name="stateNode">1.0</parameter>
<parameter id="rateAC.s:NADH4L_pos1" spec="parameter.RealParameter" lower="0.0" name="stateNode">1.0</parameter>
<parameter id="rateAG.s:NADH4L_pos1" spec="parameter.RealParameter" lower="0.0" name="stateNode">1.0</parameter>
<parameter id="rateAT.s:NADH4L_pos1" spec="parameter.RealParameter" lower="0.0" name="stateNode">1.0</parameter>
<parameter id="rateCG.s:NADH4L_pos1" spec="parameter.RealParameter" lower="0.0" name="stateNode">1.0</parameter>
<parameter id="rateGT.s:NADH4L_pos1" spec="parameter.RealParameter" lower="0.0" name="stateNode">1.0</parameter>
<parameter id="gammaShape.s:NADH4L_pos2" spec="parameter.RealParameter" name="stateNode">1.0</parameter>
<parameter id="rateAC.s:NADH4L_pos2" spec="parameter.RealParameter" lower="0.0" name="stateNode">1.0</parameter>
<parameter id="rateAG.s:NADH4L_pos2" spec="parameter.RealParameter" lower="0.0" name="stateNode">1.0</parameter>
<parameter id="rateAT.s:NADH4L_pos2" spec="parameter.RealParameter" lower="0.0" name="stateNode">1.0</parameter>
<parameter id="rateCG.s:NADH4L_pos2" spec="parameter.RealParameter" lower="0.0" name="stateNode">1.0</parameter>
<parameter id="rateGT.s:NADH4L_pos2" spec="parameter.RealParameter" lower="0.0" name="stateNode">1.0</parameter>
<parameter id="gammaShape.s:NADH4L_pos3" spec="parameter.RealParameter" name="stateNode">1.0</parameter>
<parameter id="rateAC.s:NADH4L_pos3" spec="parameter.RealParameter" lower="0.0" name="stateNode">1.0</parameter>
```

```xml
<parameter id="rateAG.s:NADH4L_pos3" spec="parameter.RealParameter" lower="0.0" name="stateNode">1.0</parameter>
<parameter id="rateAT.s:NADH4L_pos3" spec="parameter.RealParameter" lower="0.0" name="stateNode">1.0</parameter>
<parameter id="rateCG.s:NADH4L_pos3" spec="parameter.RealParameter" lower="0.0" name="stateNode">1.0</parameter>
<parameter id="rateGT.s:NADH4L_pos3" spec="parameter.RealParameter" lower="0.0" name="stateNode">1.0</parameter>
<parameter id="gammaShape.s:NADH5_pos1" spec="parameter.RealParameter" name="stateNode">1.0</parameter>
<parameter id="rateAC.s:NADH5_pos1" spec="parameter.RealParameter" lower="0.0" name="stateNode">1.0</parameter>
<parameter id="rateAG.s:NADH5_pos1" spec="parameter.RealParameter" lower="0.0" name="stateNode">1.0</parameter>
<parameter id="rateAT.s:NADH5_pos1" spec="parameter.RealParameter" lower="0.0" name="stateNode">1.0</parameter>
<parameter id="rateCG.s:NADH5_pos1" spec="parameter.RealParameter" lower="0.0" name="stateNode">1.0</parameter>
<parameter id="rateGT.s:NADH5_pos1" spec="parameter.RealParameter" lower="0.0" name="stateNode">1.0</parameter>
<parameter id="gammaShape.s:NADH5_pos2" spec="parameter.RealParameter" name="stateNode">1.0</parameter>
<parameter id="rateAC.s:NADH5_pos2" spec="parameter.RealParameter" lower="0.0" name="stateNode">1.0</parameter>
<parameter id="rateAG.s:NADH5_pos2" spec="parameter.RealParameter" lower="0.0" name="stateNode">1.0</parameter>
<parameter id="rateAT.s:NADH5_pos2" spec="parameter.RealParameter" lower="0.0" name="stateNode">1.0</parameter>
<parameter id="rateCG.s:NADH5_pos2" spec="parameter.RealParameter" lower="0.0" name="stateNode">1.0</parameter>
<parameter id="rateGT.s:NADH5_pos2" spec="parameter.RealParameter" lower="0.0" name="stateNode">1.0</parameter>
<parameter id="gammaShape.s:NADH5_pos3" spec="parameter.RealParameter" name="stateNode">1.0</parameter>
<parameter id="rateAC.s:NADH5_pos3" spec="parameter.RealParameter" lower="0.0" name="stateNode">1.0</parameter>
<parameter id="rateAG.s:NADH5_pos3" spec="parameter.RealParameter" lower="0.0" name="stateNode">1.0</parameter>
<parameter id="rateAT.s:NADH5_pos3" spec="parameter.RealParameter" lower="0.0" name="stateNode">1.0</parameter>
<parameter id="rateCG.s:NADH5_pos3" spec="parameter.RealParameter" lower="0.0" name="stateNode">1.0</parameter>
<parameter id="rateGT.s:NADH5_pos3" spec="parameter.RealParameter" lower="0.0" name="stateNode">1.0</parameter>
<parameter id="gammaShape.s:NADH6_pos1" spec="parameter.RealParameter" name="stateNode">1.0</parameter>
<parameter id="rateAC.s:NADH6_pos1" spec="parameter.RealParameter" lower="0.0" name="stateNode">1.0</parameter>
<parameter id="rateAG.s:NADH6_pos1" spec="parameter.RealParameter" lower="0.0" name="stateNode">1.0</parameter>
<parameter id="rateAT.s:NADH6_pos1" spec="parameter.RealParameter" lower="0.0" name="stateNode">1.0</parameter>
<parameter id="rateCG.s:NADH6_pos1" spec="parameter.RealParameter" lower="0.0" name="stateNode">1.0</parameter>
<parameter id="rateGT.s:NADH6_pos1" spec="parameter.RealParameter" lower="0.0" name="stateNode">1.0</parameter>
<parameter id="gammaShape.s:NADH6_pos2" spec="parameter.RealParameter" name="stateNode">1.0</parameter>
<parameter id="rateAC.s:NADH6_pos2" spec="parameter.RealParameter" lower="0.0" name="stateNode">1.0</parameter>
<parameter id="rateAG.s:NADH6_pos2" spec="parameter.RealParameter" lower="0.0" name="stateNode">1.0</parameter>
```

```
<parameter id="rateAT.s:NADH6_pos2" spec="parameter.RealParameter" lower="0.0" name="stateNode">1.0</parameter>
<parameter id="rateCG.s:NADH6_pos2" spec="parameter.RealParameter" lower="0.0" name="stateNode">1.0</parameter>
<parameter id="rateGT.s:NADH6_pos2" spec="parameter.RealParameter" lower="0.0" name="stateNode">1.0</parameter>
<parameter id="gammaShape.s:NADH6_pos3" spec="parameter.RealParameter" name="stateNode">1.0</parameter>
<parameter id="rateAC.s:NADH6_pos3" spec="parameter.RealParameter" lower="0.0" name="stateNode">1.0</parameter>
<parameter id="rateAG.s:NADH6_pos3" spec="parameter.RealParameter" lower="0.0" name="stateNode">1.0</parameter>
<parameter id="rateAT.s:NADH6_pos3" spec="parameter.RealParameter" lower="0.0" name="stateNode">1.0</parameter>
<parameter id="rateCG.s:NADH6_pos3" spec="parameter.RealParameter" lower="0.0" name="stateNode">1.0</parameter>
<parameter id="rateGT.s:NADH6_pos3" spec="parameter.RealParameter" lower="0.0" name="stateNode">1.0</parameter>
<parameter id="popSize.t:COX1_pos1" spec="parameter.RealParameter" name="stateNode">2.3</parameter>
<parameter id="popSize.t:COX1_pos2" spec="parameter.RealParameter" name="stateNode">2.3</parameter>
<parameter id="popSize.t:COX1_pos3" spec="parameter.RealParameter" name="stateNode">2.3</parameter>
<parameter id="popSize.t:COX2_pos1" spec="parameter.RealParameter" name="stateNode">2.3</parameter>
<parameter id="popSize.t:COX2_pos2" spec="parameter.RealParameter" name="stateNode">2.3</parameter>
<parameter id="popSize.t:COX2_pos3" spec="parameter.RealParameter" name="stateNode">2.3</parameter>
<parameter id="popSize.t:COX3_pos1" spec="parameter.RealParameter" name="stateNode">0.3</parameter>
<parameter id="popSize.t:COX3_pos2" spec="parameter.RealParameter" name="stateNode">2.3</parameter>
<parameter id="popSize.t:COX3_pos3" spec="parameter.RealParameter" name="stateNode">2.3</parameter>
<parameter id="popSize.t:CYTB_pos1" spec="parameter.RealParameter" name="stateNode">2.3</parameter>
<parameter id="popSize.t:CYTB_pos2" spec="parameter.RealParameter" name="stateNode">2.3</parameter>
<parameter id="popSize.t:CYTB_pos3" spec="parameter.RealParameter" name="stateNode">2.3</parameter>
<parameter id="popSize.t:Mytilidae27sq12PCGs" spec="parameter.RealParameter" name="stateNode">2.3</parameter>
<parameter id="popSize.t:NADH1_pos1" spec="parameter.RealParameter" name="stateNode">2.3</parameter>
<parameter id="popSize.t:NADH1_pos2" spec="parameter.RealParameter" name="stateNode">2.3</parameter>
<parameter id="popSize.t:NADH1_pos3" spec="parameter.RealParameter" name="stateNode">2.3</parameter>
<parameter id="popSize.t:NADH2_pos1" spec="parameter.RealParameter" name="stateNode">2.3</parameter>
<parameter id="popSize.t:NADH2_pos2" spec="parameter.RealParameter" name="stateNode">2.3</parameter>
<parameter id="popSize.t:NADH2_pos3" spec="parameter.RealParameter" name="stateNode">2.3</parameter>
<parameter id="popSize.t:NADH3_pos1" spec="parameter.RealParameter" name="stateNode">2.3</parameter>
<parameter id="popSize.t:NADH3_pos2" spec="parameter.RealParameter" name="stateNode">2.3</parameter>
<parameter id="popSize.t:NADH3_pos3" spec="parameter.RealParameter" name="stateNode">2.3</parameter>
<parameter id="popSize.t:NADH4L_pos1" spec="parameter.RealParameter" name="stateNode">2.3</parameter>
<parameter id="popSize.t:NADH4L_pos2" spec="parameter.RealParameter" name="stateNode">2.3</parameter>
<parameter id="popSize.t:NADH4L_pos3" spec="parameter.RealParameter" name="stateNode">2.3</parameter>
<parameter id="popSize.t:NADH4_pos1" spec="parameter.RealParameter" name="stateNode">2.3</parameter>
<parameter id="popSize.t:NADH4_pos2" spec="parameter.RealParameter" name="stateNode">2.3</parameter>
<parameter id="popSize.t:NADH4_pos3" spec="parameter.RealParameter" name="stateNode">2.3</parameter>
```

```xml
<parameter id="popSize.t:NADH5_pos1" spec="parameter.RealParameter"
name="stateNode">2.3</parameter>
<parameter id="popSize.t:NADH5_pos2" spec="parameter.RealParameter"
name="stateNode">2.3</parameter>
<parameter id="popSize.t:NADH5_pos3" spec="parameter.RealParameter"
name="stateNode">2.3</parameter>
<parameter id="popSize.t:NADH6_pos1" spec="parameter.RealParameter"
name="stateNode">2.3</parameter>
<parameter id="popSize.t:NADH6_pos2" spec="parameter.RealParameter"
name="stateNode">2.3</parameter>
<parameter id="popSize.t:NADH6_pos3" spec="parameter.RealParameter"
name="stateNode">2.3</parameter>
<parameter id="popSize.t:atp6_pos1" spec="parameter.RealParameter" name="stateNode">2.3</parameter>
<parameter id="popSize.t:atp6_pos2" spec="parameter.RealParameter" name="stateNode">2.3</parameter>
<parameter id="popSize.t:atp6_pos3" spec="parameter.RealParameter" name="stateNode">2.3</parameter>
<parameter id="proportionInvariant.s:Mytilidae27sq12PCGs" spec="parameter.RealParameter" lower="0.0"
name="stateNode" upper="1.0">0.126</parameter>
<parameter id="proportionInvariant.s:atp6_pos3" spec="parameter.RealParameter" lower="0.0"
name="stateNode" upper="1.0">0.126</parameter>
<parameter id="proportionInvariant.s:COX1_pos1" spec="parameter.RealParameter" lower="0.0"
name="stateNode" upper="1.0">0.126</parameter>
<parameter id="proportionInvariant.s:COX1_pos2" spec="parameter.RealParameter" lower="0.0"
name="stateNode" upper="1.0">0.126</parameter>
<parameter id="proportionInvariant.s:COX1_pos3" spec="parameter.RealParameter" lower="0.0"
name="stateNode" upper="1.0">0.126</parameter>
<parameter id="proportionInvariant.s:COX2_pos1" spec="parameter.RealParameter" lower="0.0"
name="stateNode" upper="1.0">0.126</parameter>
<parameter id="proportionInvariant.s:COX2_pos2" spec="parameter.RealParameter" lower="0.0"
name="stateNode" upper="1.0">0.126</parameter>
<parameter id="rateAC.s:COX2_pos2" spec="parameter.RealParameter" lower="0.0"
name="stateNode">1.0</parameter>
<parameter id="rateAG.s:COX2_pos2" spec="parameter.RealParameter" lower="0.0"
name="stateNode">1.0</parameter>
<parameter id="rateAT.s:COX2_pos2" spec="parameter.RealParameter" lower="0.0"
name="stateNode">1.0</parameter>
<parameter id="rateCG.s:COX2_pos2" spec="parameter.RealParameter" lower="0.0"
name="stateNode">1.0</parameter>
<parameter id="rateGT.s:COX2_pos2" spec="parameter.RealParameter" lower="0.0"
name="stateNode">1.0</parameter>
<parameter id="proportionInvariant.s:COX2_pos3" spec="parameter.RealParameter" lower="0.0"
name="stateNode" upper="1.0">0.126</parameter>
<parameter id="proportionInvariant.s:COX3_pos1" spec="parameter.RealParameter" lower="0.0"
name="stateNode" upper="1.0">0.126</parameter>
<parameter id="proportionInvariant.s:COX3_pos2" spec="parameter.RealParameter" lower="0.0"
name="stateNode" upper="1.0">0.126</parameter>
<parameter id="proportionInvariant.s:COX3_pos3" spec="parameter.RealParameter" lower="0.0"
name="stateNode" upper="1.0">0.126</parameter>
<parameter id="proportionInvariant.s:CYTB_pos1" spec="parameter.RealParameter" lower="0.0"
name="stateNode" upper="1.0">0.126</parameter>
<parameter id="proportionInvariant.s:CYTB_pos2" spec="parameter.RealParameter" lower="0.0"
name="stateNode" upper="1.0">0.126</parameter>
<parameter id="proportionInvariant.s:CYTB_pos3" spec="parameter.RealParameter" lower="0.0"
name="stateNode" upper="1.0">0.126</parameter>
<parameter id="proportionInvariant.s:NADH1_pos1" spec="parameter.RealParameter" lower="0.0"
name="stateNode" upper="1.0">0.126</parameter>
<parameter id="proportionInvariant.s:NADH1_pos2" spec="parameter.RealParameter" lower="0.0"
name="stateNode" upper="1.0">0.126</parameter>
<parameter id="proportionInvariant.s:NADH1_pos3" spec="parameter.RealParameter" lower="0.0"
name="stateNode" upper="1.0">0.126</parameter>
<parameter id="proportionInvariant.s:NADH2_pos1" spec="parameter.RealParameter" lower="0.0"
name="stateNode" upper="1.0">0.126</parameter>
<parameter id="proportionInvariant.s:NADH2_pos2" spec="parameter.RealParameter" lower="0.0"
name="stateNode" upper="1.0">0.126</parameter>
```

```xml
<parameter id="proportionInvariant.s:NADH2_pos3" spec="parameter.RealParameter" lower="0.0" name="stateNode" upper="1.0">0.126</parameter>
<parameter id="proportionInvariant.s:NADH3_pos1" spec="parameter.RealParameter" lower="0.0" name="stateNode" upper="1.0">0.126</parameter>
<parameter id="proportionInvariant.s:NADH3_pos2" spec="parameter.RealParameter" lower="0.0" name="stateNode" upper="1.0">0.126</parameter>
<parameter id="proportionInvariant.s:NADH3_pos3" spec="parameter.RealParameter" lower="0.0" name="stateNode" upper="1.0">0.126</parameter>
<parameter id="proportionInvariant.s:NADH4_pos1" spec="parameter.RealParameter" lower="0.0" name="stateNode" upper="1.0">0.126</parameter>
<parameter id="proportionInvariant.s:NADH4_pos2" spec="parameter.RealParameter" lower="0.0" name="stateNode" upper="1.0">0.126</parameter>
<parameter id="proportionInvariant.s:NADH4_pos3" spec="parameter.RealParameter" lower="0.0" name="stateNode" upper="1.0">0.126</parameter>
<parameter id="proportionInvariant.s:NADH4L_pos1" spec="parameter.RealParameter" lower="0.0" name="stateNode" upper="1.0">0.126</parameter>
<parameter id="proportionInvariant.s:NADH4L_pos2" spec="parameter.RealParameter" lower="0.0" name="stateNode" upper="1.0">0.126</parameter>
<parameter id="proportionInvariant.s:NADH4L_pos3" spec="parameter.RealParameter" lower="0.0" name="stateNode" upper="1.0">0.126</parameter>
<parameter id="proportionInvariant.s:NADH5_pos1" spec="parameter.RealParameter" lower="0.0" name="stateNode" upper="1.0">0.126</parameter>
<parameter id="proportionInvariant.s:NADH5_pos2" spec="parameter.RealParameter" lower="0.0" name="stateNode" upper="1.0">0.126</parameter>
<parameter id="proportionInvariant.s:NADH5_pos3" spec="parameter.RealParameter" lower="0.0" name="stateNode" upper="1.0">0.126</parameter>
<parameter id="proportionInvariant.s:NADH6_pos1" spec="parameter.RealParameter" lower="0.0" name="stateNode" upper="1.0">0.126</parameter>
<parameter id="proportionInvariant.s:NADH6_pos2" spec="parameter.RealParameter" lower="0.0" name="stateNode" upper="1.0">0.126</parameter>
<parameter id="proportionInvariant.s:NADH6_pos3" spec="parameter.RealParameter" lower="0.0" name="stateNode" upper="1.0">0.126</parameter>
<parameter id="rateAC.s:Mytilidae27sq12PCGs" spec="parameter.RealParameter" lower="0.0" name="stateNode">1.0</parameter>
<parameter id="rateAG.s:Mytilidae27sq12PCGs" spec="parameter.RealParameter" lower="0.0" name="stateNode">1.0</parameter>
<parameter id="rateAT.s:Mytilidae27sq12PCGs" spec="parameter.RealParameter" lower="0.0" name="stateNode">1.0</parameter>
<parameter id="rateCG.s:Mytilidae27sq12PCGs" spec="parameter.RealParameter" lower="0.0" name="stateNode">1.0</parameter>
<parameter id="rateGT.s:Mytilidae27sq12PCGs" spec="parameter.RealParameter" lower="0.0" name="stateNode">1.0</parameter>
<parameter id="freqParameter.s:atp6_pos1" spec="parameter.RealParameter" dimension="4" lower="0.0" name="stateNode" upper="1.0">0.25</parameter>
<parameter id="freqParameter.s:COX1_pos1" spec="parameter.RealParameter" dimension="4" lower="0.0" name="stateNode" upper="1.0">0.25</parameter>
<parameter id="freqParameter.s:COX1_pos3" spec="parameter.RealParameter" dimension="4" lower="0.0" name="stateNode" upper="1.0">0.25</parameter>
<parameter id="freqParameter.s:NADH5_pos3" spec="parameter.RealParameter" dimension="4" lower="0.0" name="stateNode" upper="1.0">0.25</parameter>
<parameter id="freqParameter.s:NADH2_pos3" spec="parameter.RealParameter" dimension="4" lower="0.0" name="stateNode" upper="1.0">0.25</parameter>
<parameter id="freqParameter.s:NADH3_pos3" spec="parameter.RealParameter" dimension="4" lower="0.0" name="stateNode" upper="1.0">0.25</parameter>
<parameter id="freqParameter.s:NADH1_pos1" spec="parameter.RealParameter" dimension="4" lower="0.0" name="stateNode" upper="1.0">0.25</parameter>
<parameter id="freqParameter.s:NADH6_pos1" spec="parameter.RealParameter" dimension="4" lower="0.0" name="stateNode" upper="1.0">0.25</parameter>
<parameter id="freqParameter.s:NADH1_pos2" spec="parameter.RealParameter" dimension="4" lower="0.0" name="stateNode" upper="1.0">0.25</parameter>
<parameter id="freqParameter.s:NADH4L_pos1" spec="parameter.RealParameter" dimension="4" lower="0.0" name="stateNode" upper="1.0">0.25</parameter>
```

```xml
<parameter id="freqParameter.s:CYTB_pos1" spec="parameter.RealParameter" dimension="4" lower="0.0" name="stateNode" upper="1.0">0.25</parameter>
<parameter id="freqParameter.s:atp6_pos2" spec="parameter.RealParameter" dimension="4" lower="0.0" name="stateNode" upper="1.0">0.25</parameter>
<parameter id="freqParameter.s:NADH6_pos3" spec="parameter.RealParameter" dimension="4" lower="0.0" name="stateNode" upper="1.0">0.25</parameter>
<parameter id="freqParameter.s:NADH2_pos2" spec="parameter.RealParameter" dimension="4" lower="0.0" name="stateNode" upper="1.0">0.25</parameter>
<parameter id="freqParameter.s:NADH6_pos2" spec="parameter.RealParameter" dimension="4" lower="0.0" name="stateNode" upper="1.0">0.25</parameter>
<parameter id="freqParameter.s:NADH4L_pos2" spec="parameter.RealParameter" dimension="4" lower="0.0" name="stateNode" upper="1.0">0.25</parameter>
<parameter id="freqParameter.s:NADH4_pos3" spec="parameter.RealParameter" dimension="4" lower="0.0" name="stateNode" upper="1.0">0.25</parameter>
<parameter id="freqParameter.s:NADH5_pos1" spec="parameter.RealParameter" dimension="4" lower="0.0" name="stateNode" upper="1.0">0.25</parameter>
<parameter id="freqParameter.s:NADH5_pos2" spec="parameter.RealParameter" dimension="4" lower="0.0" name="stateNode" upper="1.0">0.25</parameter>
<parameter id="freqParameter.s:NADH3_pos2" spec="parameter.RealParameter" dimension="4" lower="0.0" name="stateNode" upper="1.0">0.25</parameter>
<parameter id="freqParameter.s:Mytilidae27sq12PCGs" spec="parameter.RealParameter" dimension="4" lower="0.0" name="stateNode" upper="1.0">0.25</parameter>
<parameter id="freqParameter.s:COX3_pos1" spec="parameter.RealParameter" dimension="4" lower="0.0" name="stateNode" upper="1.0">0.25</parameter>
<parameter id="freqParameter.s:NADH2_pos1" spec="parameter.RealParameter" dimension="4" lower="0.0" name="stateNode" upper="1.0">0.25</parameter>
<parameter id="freqParameter.s:NADH3_pos1" spec="parameter.RealParameter" dimension="4" lower="0.0" name="stateNode" upper="1.0">0.25</parameter>
<parameter id="freqParameter.s:NADH1_pos3" spec="parameter.RealParameter" dimension="4" lower="0.0" name="stateNode" upper="1.0">0.25</parameter>
<parameter id="freqParameter.s:CYTB_pos2" spec="parameter.RealParameter" dimension="4" lower="0.0" name="stateNode" upper="1.0">0.25</parameter>
<parameter id="freqParameter.s:COX2_pos2" spec="parameter.RealParameter" dimension="4" lower="0.0" name="stateNode" upper="1.0">0.25</parameter>
<parameter id="freqParameter.s:COX2_pos3" spec="parameter.RealParameter" dimension="4" lower="0.0" name="stateNode" upper="1.0">0.25</parameter>
<parameter id="freqParameter.s:COX3_pos2" spec="parameter.RealParameter" dimension="4" lower="0.0" name="stateNode" upper="1.0">0.25</parameter>
<parameter id="freqParameter.s:CYTB_pos3" spec="parameter.RealParameter" dimension="4" lower="0.0" name="stateNode" upper="1.0">0.25</parameter>
<parameter id="freqParameter.s:NADH4_pos2" spec="parameter.RealParameter" dimension="4" lower="0.0" name="stateNode" upper="1.0">0.25</parameter>
<parameter id="freqParameter.s:COX3_pos3" spec="parameter.RealParameter" dimension="4" lower="0.0" name="stateNode" upper="1.0">0.25</parameter>
<parameter id="freqParameter.s:NADH4L_pos3" spec="parameter.RealParameter" dimension="4" lower="0.0" name="stateNode" upper="1.0">0.25</parameter>
<parameter id="freqParameter.s:COX2_pos1" spec="parameter.RealParameter" dimension="4" lower="0.0" name="stateNode" upper="1.0">0.25</parameter>
<parameter id="freqParameter.s:NADH4_pos1" spec="parameter.RealParameter" dimension="4" lower="0.0" name="stateNode" upper="1.0">0.25</parameter>
<parameter id="freqParameter.s:atp6_pos3" spec="parameter.RealParameter" dimension="4" lower="0.0" name="stateNode" upper="1.0">0.25</parameter>
</state>

<init id="RandomTree.t:NADH6_pos1" spec="beast.evolution.tree.RandomTree" estimate="false" initial="@Tree.t:NADH6_pos1" taxa="@NADH6_pos1">
<populationModel id="ConstantPopulation0.t:NADH6_pos1" spec="ConstantPopulation">
<parameter id="randomPopSize.t:NADH6_pos1" spec="parameter.RealParameter" name="popSize">1.0</parameter>
</populationModel>
</init>
```

```xml
<init id="RandomTree.t:NADH1_pos1" spec="beast.evolution.tree.RandomTree" estimate="false" initial="@Tree.t:NADH1_pos1" taxa="@NADH1_pos1">
    <populationModel id="ConstantPopulation0.t:NADH1_pos1" spec="ConstantPopulation">
        <parameter id="randomPopSize.t:NADH1_pos1" spec="parameter.RealParameter" name="popSize">1.0</parameter>
    </populationModel>
</init>

<init id="RandomTree.t:COX2_pos1" spec="beast.evolution.tree.RandomTree" estimate="false" initial="@Tree.t:COX2_pos1" taxa="@COX2_pos1">
    <populationModel id="ConstantPopulation0.t:COX2_pos1" spec="ConstantPopulation">
        <parameter id="randomPopSize.t:COX2_pos1" spec="parameter.RealParameter" name="popSize">1.0</parameter>
    </populationModel>
</init>

<init id="RandomTree.t:NADH1_pos2" spec="beast.evolution.tree.RandomTree" estimate="false" initial="@Tree.t:NADH1_pos2" taxa="@NADH1_pos2">
    <populationModel id="ConstantPopulation0.t:NADH1_pos2" spec="ConstantPopulation">
        <parameter id="randomPopSize.t:NADH1_pos2" spec="parameter.RealParameter" name="popSize">1.0</parameter>
    </populationModel>
</init>

<init id="RandomTree.t:NADH4_pos1" spec="beast.evolution.tree.RandomTree" estimate="false" initial="@Tree.t:NADH4_pos1" taxa="@NADH4_pos1">
    <populationModel id="ConstantPopulation0.t:NADH4_pos1" spec="ConstantPopulation">
        <parameter id="randomPopSize.t:NADH4_pos1" spec="parameter.RealParameter" name="popSize">1.0</parameter>
    </populationModel>
</init>

<init id="RandomTree.t:NADH4L_pos3" spec="beast.evolution.tree.RandomTree" estimate="false" initial="@Tree.t:NADH4L_pos3" taxa="@NADH4L_pos3">
    <populationModel id="ConstantPopulation0.t:NADH4L_pos3" spec="ConstantPopulation">
        <parameter id="randomPopSize.t:NADH4L_pos3" spec="parameter.RealParameter" name="popSize">1.0</parameter>
    </populationModel>
</init>

<init id="RandomTree.t:NADH4L_pos1" spec="beast.evolution.tree.RandomTree" estimate="false" initial="@Tree.t:NADH4L_pos1" taxa="@NADH4L_pos1">
    <populationModel id="ConstantPopulation0.t:NADH4L_pos1" spec="ConstantPopulation">
        <parameter id="randomPopSize.t:NADH4L_pos1" spec="parameter.RealParameter" name="popSize">1.0</parameter>
    </populationModel>
</init>

<init id="RandomTree.t:NADH2_pos1" spec="beast.evolution.tree.RandomTree" estimate="false" initial="@Tree.t:NADH2_pos1" taxa="@NADH2_pos1">
    <populationModel id="ConstantPopulation0.t:NADH2_pos1" spec="ConstantPopulation">
        <parameter id="randomPopSize.t:NADH2_pos1" spec="parameter.RealParameter" name="popSize">1.0</parameter>
    </populationModel>
</init>

<init id="RandomTree.t:NADH3_pos2" spec="beast.evolution.tree.RandomTree" estimate="false" initial="@Tree.t:NADH3_pos2" taxa="@NADH3_pos2">
    <populationModel id="ConstantPopulation0.t:NADH3_pos2" spec="ConstantPopulation">
        <parameter id="randomPopSize.t:NADH3_pos2" spec="parameter.RealParameter" name="popSize">1.0</parameter>
    </populationModel>
</init>
```

```
    <init id="RandomTree.t:NADH2_pos3" spec="beast.evolution.tree.RandomTree" estimate="false"
initial="@Tree.t:NADH2_pos3" taxa="@NADH2_pos3">
        <populationModel id="ConstantPopulation0.t:NADH2_pos3" spec="ConstantPopulation">
            <parameter id="randomPopSize.t:NADH2_pos3" spec="parameter.RealParameter"
name="popSize">1.0</parameter>
        </populationModel>
    </init>

    <init id="RandomTree.t:NADH3_pos1" spec="beast.evolution.tree.RandomTree" estimate="false"
initial="@Tree.t:NADH3_pos1" taxa="@NADH3_pos1">
        <populationModel id="ConstantPopulation0.t:NADH3_pos1" spec="ConstantPopulation">
            <parameter id="randomPopSize.t:NADH3_pos1" spec="parameter.RealParameter"
name="popSize">1.0</parameter>
        </populationModel>
    </init>

    <init id="RandomTree.t:NADH2_pos2" spec="beast.evolution.tree.RandomTree" estimate="false"
initial="@Tree.t:NADH2_pos2" taxa="@NADH2_pos2">
        <populationModel id="ConstantPopulation0.t:NADH2_pos2" spec="ConstantPopulation">
            <parameter id="randomPopSize.t:NADH2_pos2" spec="parameter.RealParameter"
name="popSize">1.0</parameter>
        </populationModel>
    </init>

    <init id="RandomTree.t:CYTB_pos2" spec="beast.evolution.tree.RandomTree" estimate="false"
initial="@Tree.t:CYTB_pos2" taxa="@CYTB_pos2">
        <populationModel id="ConstantPopulation0.t:CYTB_pos2" spec="ConstantPopulation">
            <parameter id="randomPopSize.t:CYTB_pos2" spec="parameter.RealParameter"
name="popSize">1.0</parameter>
        </populationModel>
    </init>

    <init id="RandomTree.t:atp6_pos3" spec="beast.evolution.tree.RandomTree" estimate="false"
initial="@Tree.t:atp6_pos3" taxa="@atp6_pos3">
        <populationModel id="ConstantPopulation0.t:atp6_pos3" spec="ConstantPopulation">
            <parameter id="randomPopSize.t:atp6_pos3" spec="parameter.RealParameter"
name="popSize">1.0</parameter>
        </populationModel>
    </init>

    <init id="RandomTree.t:COX1_pos1" spec="beast.evolution.tree.RandomTree" estimate="false"
initial="@Tree.t:COX1_pos1" taxa="@COX1_pos1">
        <populationModel id="ConstantPopulation0.t:COX1_pos1" spec="ConstantPopulation">
            <parameter id="randomPopSize.t:COX1_pos1" spec="parameter.RealParameter"
name="popSize">1.0</parameter>
        </populationModel>
    </init>

    <init id="RandomTree.t:CYTB_pos1" spec="beast.evolution.tree.RandomTree" estimate="false"
initial="@Tree.t:CYTB_pos1" taxa="@CYTB_pos1">
        <populationModel id="ConstantPopulation0.t:CYTB_pos1" spec="ConstantPopulation">
            <parameter id="randomPopSize.t:CYTB_pos1" spec="parameter.RealParameter"
name="popSize">1.0</parameter>
        </populationModel>
    </init>

    <init id="RandomTree.t:NADH4_pos3" spec="beast.evolution.tree.RandomTree" estimate="false"
initial="@Tree.t:NADH4_pos3" taxa="@NADH4_pos3">
        <populationModel id="ConstantPopulation0.t:NADH4_pos3" spec="ConstantPopulation">
            <parameter id="randomPopSize.t:NADH4_pos3" spec="parameter.RealParameter"
name="popSize">1.0</parameter>
        </populationModel>
```

```
    </init>

    <init id="RandomTree.t:COX1_pos3" spec="beast.evolution.tree.RandomTree" estimate="false"
initial="@Tree.t:COX1_pos3" taxa="@COX1_pos3">
        <populationModel id="ConstantPopulation0.t:COX1_pos3" spec="ConstantPopulation">
            <parameter id="randomPopSize.t:COX1_pos3" spec="parameter.RealParameter"
name="popSize">1.0</parameter>
        </populationModel>
    </init>

    <init id="RandomTree.t:COX2_pos2" spec="beast.evolution.tree.RandomTree" estimate="false"
initial="@Tree.t:COX2_pos2" taxa="@COX2_pos2">
        <populationModel id="ConstantPopulation0.t:COX2_pos2" spec="ConstantPopulation">
            <parameter id="randomPopSize.t:COX2_pos2" spec="parameter.RealParameter"
name="popSize">1.0</parameter>
        </populationModel>
    </init>

    <init id="RandomTree.t:CYTB_pos3" spec="beast.evolution.tree.RandomTree" estimate="false"
initial="@Tree.t:CYTB_pos3" taxa="@CYTB_pos3">
        <populationModel id="ConstantPopulation0.t:CYTB_pos3" spec="ConstantPopulation">
            <parameter id="randomPopSize.t:CYTB_pos3" spec="parameter.RealParameter"
name="popSize">1.0</parameter>
        </populationModel>
    </init>

    <init id="RandomTree.t:NADH5_pos1" spec="beast.evolution.tree.RandomTree" estimate="false"
initial="@Tree.t:NADH5_pos1" taxa="@NADH5_pos1">
        <populationModel id="ConstantPopulation0.t:NADH5_pos1" spec="ConstantPopulation">
            <parameter id="randomPopSize.t:NADH5_pos1" spec="parameter.RealParameter"
name="popSize">1.0</parameter>
        </populationModel>
    </init>

    <init id="RandomTree.t:atp6_pos1" spec="beast.evolution.tree.RandomTree" estimate="false"
initial="@Tree.t:atp6_pos1" taxa="@atp6_pos1">
        <populationModel id="ConstantPopulation0.t:atp6_pos1" spec="ConstantPopulation">
            <parameter id="randomPopSize.t:atp6_pos1" spec="parameter.RealParameter"
name="popSize">1.0</parameter>
        </populationModel>
    </init>

    <init id="RandomTree.t:NADH1_pos3" spec="beast.evolution.tree.RandomTree" estimate="false"
initial="@Tree.t:NADH1_pos3" taxa="@NADH1_pos3">
        <populationModel id="ConstantPopulation0.t:NADH1_pos3" spec="ConstantPopulation">
            <parameter id="randomPopSize.t:NADH1_pos3" spec="parameter.RealParameter"
name="popSize">1.0</parameter>
        </populationModel>
    </init>

    <init id="RandomTree.t:atp6_pos2" spec="beast.evolution.tree.RandomTree" estimate="false"
initial="@Tree.t:atp6_pos2" taxa="@atp6_pos2">
        <populationModel id="ConstantPopulation0.t:atp6_pos2" spec="ConstantPopulation">
            <parameter id="randomPopSize.t:atp6_pos2" spec="parameter.RealParameter"
name="popSize">1.0</parameter>
        </populationModel>
    </init>

    <init id="RandomTree.t:NADH4_pos2" spec="beast.evolution.tree.RandomTree" estimate="false"
initial="@Tree.t:NADH4_pos2" taxa="@NADH4_pos2">
        <populationModel id="ConstantPopulation0.t:NADH4_pos2" spec="ConstantPopulation">
            <parameter id="randomPopSize.t:NADH4_pos2" spec="parameter.RealParameter"
name="popSize">1.0</parameter>
```

```
        </populationModel>
    </init>

    <init id="RandomTree.t:COX3_pos2" spec="beast.evolution.tree.RandomTree" estimate="false"
initial="@Tree.t:COX3_pos2" taxa="@COX3_pos2">
        <populationModel id="ConstantPopulation0.t:COX3_pos2" spec="ConstantPopulation">
            <parameter id="randomPopSize.t:COX3_pos2" spec="parameter.RealParameter"
name="popSize">1.0</parameter>
        </populationModel>
    </init>

    <init id="RandomTree.t:NADH5_pos3" spec="beast.evolution.tree.RandomTree" estimate="false"
initial="@Tree.t:NADH5_pos3" taxa="@NADH5_pos3">
        <populationModel id="ConstantPopulation0.t:NADH5_pos3" spec="ConstantPopulation">
            <parameter id="randomPopSize.t:NADH5_pos3" spec="parameter.RealParameter"
name="popSize">1.0</parameter>
        </populationModel>
    </init>

    <init id="RandomTree.t:NADH6_pos3" spec="beast.evolution.tree.RandomTree" estimate="false"
initial="@Tree.t:NADH6_pos3" taxa="@NADH6_pos3">
        <populationModel id="ConstantPopulation0.t:NADH6_pos3" spec="ConstantPopulation">
            <parameter id="randomPopSize.t:NADH6_pos3" spec="parameter.RealParameter"
name="popSize">1.0</parameter>
        </populationModel>
    </init>

    <init id="RandomTree.t:COX3_pos1" spec="beast.evolution.tree.RandomTree" estimate="false"
initial="@Tree.t:COX3_pos1" taxa="@COX3_pos1">
        <populationModel id="ConstantPopulation0.t:COX3_pos1" spec="ConstantPopulation">
            <parameter id="randomPopSize.t:COX3_pos1" spec="parameter.RealParameter"
name="popSize">1.0</parameter>
        </populationModel>
    </init>

    <init id="RandomTree.t:NADH3_pos3" spec="beast.evolution.tree.RandomTree" estimate="false"
initial="@Tree.t:NADH3_pos3" taxa="@NADH3_pos3">
        <populationModel id="ConstantPopulation0.t:NADH3_pos3" spec="ConstantPopulation">
            <parameter id="randomPopSize.t:NADH3_pos3" spec="parameter.RealParameter"
name="popSize">1.0</parameter>
        </populationModel>
    </init>

    <init id="RandomTree.t:NADH5_pos2" spec="beast.evolution.tree.RandomTree" estimate="false"
initial="@Tree.t:NADH5_pos2" taxa="@NADH5_pos2">
        <populationModel id="ConstantPopulation0.t:NADH5_pos2" spec="ConstantPopulation">
            <parameter id="randomPopSize.t:NADH5_pos2" spec="parameter.RealParameter"
name="popSize">1.0</parameter>
        </populationModel>
    </init>

    <init id="RandomTree.t:NADH6_pos2" spec="beast.evolution.tree.RandomTree" estimate="false"
initial="@Tree.t:NADH6_pos2" taxa="@NADH6_pos2">
        <populationModel id="ConstantPopulation0.t:NADH6_pos2" spec="ConstantPopulation">
            <parameter id="randomPopSize.t:NADH6_pos2" spec="parameter.RealParameter"
name="popSize">1.0</parameter>
        </populationModel>
    </init>

    <init id="RandomTree.t:NADH4L_pos2" spec="beast.evolution.tree.RandomTree" estimate="false"
initial="@Tree.t:NADH4L_pos2" taxa="@NADH4L_pos2">
        <populationModel id="ConstantPopulation0.t:NADH4L_pos2" spec="ConstantPopulation">
```

```
            <parameter id="randomPopSize.t:NADH4L_pos2" spec="parameter.RealParameter"
name="popSize">1.0</parameter>
        </populationModel>
    </init>

    <init id="RandomTree.t:COX1_pos2" spec="beast.evolution.tree.RandomTree" estimate="false"
initial="@Tree.t:COX1_pos2" taxa="@COX1_pos2">
        <populationModel id="ConstantPopulation0.t:COX1_pos2" spec="ConstantPopulation">
            <parameter id="randomPopSize.t:COX1_pos2" spec="parameter.RealParameter"
name="popSize">1.0</parameter>
        </populationModel>
    </init>

    <init id="RandomTree.t:COX2_pos3" spec="beast.evolution.tree.RandomTree" estimate="false"
initial="@Tree.t:COX2_pos3" taxa="@COX2_pos3">
        <populationModel id="ConstantPopulation0.t:COX2_pos3" spec="ConstantPopulation">
            <parameter id="randomPopSize.t:COX2_pos3" spec="parameter.RealParameter"
name="popSize">1.0</parameter>
        </populationModel>
    </init>

    <init id="RandomTree.t:COX3_pos3" spec="beast.evolution.tree.RandomTree" estimate="false"
initial="@Tree.t:COX3_pos3" taxa="@COX3_pos3">
        <populationModel id="ConstantPopulation0.t:COX3_pos3" spec="ConstantPopulation">
            <parameter id="randomPopSize.t:COX3_pos3" spec="parameter.RealParameter"
name="popSize">1.0</parameter>
        </populationModel>
    </init>

    <init id="RandomTree.t:Mytilidae27sq12PCGs" spec="beast.evolution.tree.RandomTree" estimate="false"
initial="@Tree.t:Mytilidae27sq12PCGs" taxa="@Mytilidae27sq12PCGs">
        <populationModel id="ConstantPopulation0.t:Mytilidae27sq12PCGs" spec="ConstantPopulation">
            <parameter id="randomPopSize.t:Mytilidae27sq12PCGs" spec="parameter.RealParameter"
name="popSize">1.0</parameter>
        </populationModel>
    </init>

    <distribution id="posterior" spec="util.CompoundDistribution">
        <distribution id="prior" spec="util.CompoundDistribution">
            <distribution id="CoalescentConstant.t:COX1_pos1" spec="Coalescent">
                <populationModel id="ConstantPopulation.t:COX1_pos1" spec="ConstantPopulation"
popSize="@popSize.t:COX1_pos1"/>
                <treeIntervals id="TreeIntervals.t:COX1_pos1" spec="TreeIntervals" tree="@Tree.t:COX1_pos1"/>
            </distribution>
            <distribution id="CoalescentConstant.t:COX1_pos2" spec="Coalescent">
                <populationModel id="ConstantPopulation.t:COX1_pos2" spec="ConstantPopulation"
popSize="@popSize.t:COX1_pos2"/>
                <treeIntervals id="TreeIntervals.t:COX1_pos2" spec="TreeIntervals" tree="@Tree.t:COX1_pos2"/>
            </distribution>
            <distribution id="CoalescentConstant.t:COX1_pos3" spec="Coalescent">
                <populationModel id="ConstantPopulation.t:COX1_pos3" spec="ConstantPopulation"
popSize="@popSize.t:COX1_pos3"/>
                <treeIntervals id="TreeIntervals.t:COX1_pos3" spec="TreeIntervals" tree="@Tree.t:COX1_pos3"/>
            </distribution>
            <distribution id="CoalescentConstant.t:COX2_pos1" spec="Coalescent">
                <populationModel id="ConstantPopulation.t:COX2_pos1" spec="ConstantPopulation"
popSize="@popSize.t:COX2_pos1"/>
                <treeIntervals id="TreeIntervals.t:COX2_pos1" spec="TreeIntervals" tree="@Tree.t:COX2_pos1"/>
            </distribution>
            <distribution id="CoalescentConstant.t:COX2_pos2" spec="Coalescent">
                <populationModel id="ConstantPopulation.t:COX2_pos2" spec="ConstantPopulation"
popSize="@popSize.t:COX2_pos2"/>
                <treeIntervals id="TreeIntervals.t:COX2_pos2" spec="TreeIntervals" tree="@Tree.t:COX2_pos2"/>
```

```xml
            </distribution>
            <distribution id="CoalescentConstant.t:COX2_pos3" spec="Coalescent">
                <populationModel id="ConstantPopulation.t:COX2_pos3" spec="ConstantPopulation" popSize="@popSize.t:COX2_pos3"/>
                <treeIntervals id="TreeIntervals.t:COX2_pos3" spec="TreeIntervals" tree="@Tree.t:COX2_pos3"/>
            </distribution>
            <distribution id="CoalescentConstant.t:COX3_pos1" spec="Coalescent">
                <populationModel id="ConstantPopulation.t:COX3_pos1" spec="ConstantPopulation" popSize="@popSize.t:COX3_pos1"/>
                <treeIntervals id="TreeIntervals.t:COX3_pos1" spec="TreeIntervals" tree="@Tree.t:COX3_pos1"/>
            </distribution>
            <distribution id="CoalescentConstant.t:COX3_pos2" spec="Coalescent">
                <populationModel id="ConstantPopulation.t:COX3_pos2" spec="ConstantPopulation" popSize="@popSize.t:COX3_pos2"/>
                <treeIntervals id="TreeIntervals.t:COX3_pos2" spec="TreeIntervals" tree="@Tree.t:COX3_pos2"/>
            </distribution>
            <distribution id="CoalescentConstant.t:COX3_pos3" spec="Coalescent">
                <populationModel id="ConstantPopulation.t:COX3_pos3" spec="ConstantPopulation" popSize="@popSize.t:COX3_pos3"/>
                <treeIntervals id="TreeIntervals.t:COX3_pos3" spec="TreeIntervals" tree="@Tree.t:COX3_pos3"/>
            </distribution>
            <distribution id="CoalescentConstant.t:CYTB_pos1" spec="Coalescent">
                <populationModel id="ConstantPopulation.t:CYTB_pos1" spec="ConstantPopulation" popSize="@popSize.t:CYTB_pos1"/>
                <treeIntervals id="TreeIntervals.t:CYTB_pos1" spec="TreeIntervals" tree="@Tree.t:CYTB_pos1"/>
            </distribution>
            <distribution id="CoalescentConstant.t:CYTB_pos2" spec="Coalescent">
                <populationModel id="ConstantPopulation.t:CYTB_pos2" spec="ConstantPopulation" popSize="@popSize.t:CYTB_pos2"/>
                <treeIntervals id="TreeIntervals.t:CYTB_pos2" spec="TreeIntervals" tree="@Tree.t:CYTB_pos2"/>
            </distribution>
            <distribution id="CoalescentConstant.t:CYTB_pos3" spec="Coalescent">
                <populationModel id="ConstantPopulation.t:CYTB_pos3" spec="ConstantPopulation" popSize="@popSize.t:CYTB_pos3"/>
                <treeIntervals id="TreeIntervals.t:CYTB_pos3" spec="TreeIntervals" tree="@Tree.t:CYTB_pos3"/>
            </distribution>
            <distribution id="CoalescentConstant.t:Mytilidae27sq12PCGs" spec="Coalescent">
                <populationModel id="ConstantPopulation.t:Mytilidae27sq12PCGs" spec="ConstantPopulation" popSize="@popSize.t:Mytilidae27sq12PCGs"/>
                <treeIntervals id="TreeIntervals.t:Mytilidae27sq12PCGs" spec="TreeIntervals" tree="@Tree.t:Mytilidae27sq12PCGs"/>
            </distribution>
            <distribution id="CoalescentConstant.t:NADH1_pos1" spec="Coalescent">
                <populationModel id="ConstantPopulation.t:NADH1_pos1" spec="ConstantPopulation" popSize="@popSize.t:NADH1_pos1"/>
                <treeIntervals id="TreeIntervals.t:NADH1_pos1" spec="TreeIntervals" tree="@Tree.t:NADH1_pos1"/>
            </distribution>
            <distribution id="CoalescentConstant.t:NADH1_pos2" spec="Coalescent">
                <populationModel id="ConstantPopulation.t:NADH1_pos2" spec="ConstantPopulation" popSize="@popSize.t:NADH1_pos2"/>
                <treeIntervals id="TreeIntervals.t:NADH1_pos2" spec="TreeIntervals" tree="@Tree.t:NADH1_pos2"/>
            </distribution>
            <distribution id="CoalescentConstant.t:NADH1_pos3" spec="Coalescent">
                <populationModel id="ConstantPopulation.t:NADH1_pos3" spec="ConstantPopulation" popSize="@popSize.t:NADH1_pos3"/>
                <treeIntervals id="TreeIntervals.t:NADH1_pos3" spec="TreeIntervals" tree="@Tree.t:NADH1_pos3"/>
            </distribution>
            <distribution id="CoalescentConstant.t:NADH2_pos1" spec="Coalescent">
                <populationModel id="ConstantPopulation.t:NADH2_pos1" spec="ConstantPopulation" popSize="@popSize.t:NADH2_pos1"/>
                <treeIntervals id="TreeIntervals.t:NADH2_pos1" spec="TreeIntervals" tree="@Tree.t:NADH2_pos1"/>
            </distribution>
            <distribution id="CoalescentConstant.t:NADH2_pos2" spec="Coalescent">
```

```
            <populationModel id="ConstantPopulation.t:NADH2_pos2" spec="ConstantPopulation"
popSize="@popSize.t:NADH2_pos2"/>
            <treeIntervals id="TreeIntervals.t:NADH2_pos2" spec="TreeIntervals" tree="@Tree.t:NADH2_pos2"/>
        </distribution>
        <distribution id="CoalescentConstant.t:NADH2_pos3" spec="Coalescent">
            <populationModel id="ConstantPopulation.t:NADH2_pos3" spec="ConstantPopulation"
popSize="@popSize.t:NADH2_pos3"/>
            <treeIntervals id="TreeIntervals.t:NADH2_pos3" spec="TreeIntervals" tree="@Tree.t:NADH2_pos3"/>
        </distribution>
        <distribution id="CoalescentConstant.t:NADH3_pos1" spec="Coalescent">
            <populationModel id="ConstantPopulation.t:NADH3_pos1" spec="ConstantPopulation"
popSize="@popSize.t:NADH3_pos1"/>
            <treeIntervals id="TreeIntervals.t:NADH3_pos1" spec="TreeIntervals" tree="@Tree.t:NADH3_pos1"/>
        </distribution>
        <distribution id="CoalescentConstant.t:NADH3_pos2" spec="Coalescent">
            <populationModel id="ConstantPopulation.t:NADH3_pos2" spec="ConstantPopulation"
popSize="@popSize.t:NADH3_pos2"/>
            <treeIntervals id="TreeIntervals.t:NADH3_pos2" spec="TreeIntervals" tree="@Tree.t:NADH3_pos2"/>
        </distribution>
        <distribution id="CoalescentConstant.t:NADH3_pos3" spec="Coalescent">
            <populationModel id="ConstantPopulation.t:NADH3_pos3" spec="ConstantPopulation"
popSize="@popSize.t:NADH3_pos3"/>
            <treeIntervals id="TreeIntervals.t:NADH3_pos3" spec="TreeIntervals" tree="@Tree.t:NADH3_pos3"/>
        </distribution>
        <distribution id="CoalescentConstant.t:NADH4L_pos1" spec="Coalescent">
            <populationModel id="ConstantPopulation.t:NADH4L_pos1" spec="ConstantPopulation"
popSize="@popSize.t:NADH4L_pos1"/>
            <treeIntervals id="TreeIntervals.t:NADH4L_pos1" spec="TreeIntervals"
tree="@Tree.t:NADH4L_pos1"/>
        </distribution>
        <distribution id="CoalescentConstant.t:NADH4L_pos2" spec="Coalescent">
            <populationModel id="ConstantPopulation.t:NADH4L_pos2" spec="ConstantPopulation"
popSize="@popSize.t:NADH4L_pos2"/>
            <treeIntervals id="TreeIntervals.t:NADH4L_pos2" spec="TreeIntervals"
tree="@Tree.t:NADH4L_pos2"/>
        </distribution>
        <distribution id="CoalescentConstant.t:NADH4L_pos3" spec="Coalescent">
            <populationModel id="ConstantPopulation.t:NADH4L_pos3" spec="ConstantPopulation"
popSize="@popSize.t:NADH4L_pos3"/>
            <treeIntervals id="TreeIntervals.t:NADH4L_pos3" spec="TreeIntervals"
tree="@Tree.t:NADH4L_pos3"/>
        </distribution>
        <distribution id="CoalescentConstant.t:NADH4_pos1" spec="Coalescent">
            <populationModel id="ConstantPopulation.t:NADH4_pos1" spec="ConstantPopulation"
popSize="@popSize.t:NADH4_pos1"/>
            <treeIntervals id="TreeIntervals.t:NADH4_pos1" spec="TreeIntervals" tree="@Tree.t:NADH4_pos1"/>
        </distribution>
        <distribution id="CoalescentConstant.t:NADH4_pos2" spec="Coalescent">
            <populationModel id="ConstantPopulation.t:NADH4_pos2" spec="ConstantPopulation"
popSize="@popSize.t:NADH4_pos2"/>
            <treeIntervals id="TreeIntervals.t:NADH4_pos2" spec="TreeIntervals" tree="@Tree.t:NADH4_pos2"/>
        </distribution>
        <distribution id="CoalescentConstant.t:NADH4_pos3" spec="Coalescent">
            <populationModel id="ConstantPopulation.t:NADH4_pos3" spec="ConstantPopulation"
popSize="@popSize.t:NADH4_pos3"/>
            <treeIntervals id="TreeIntervals.t:NADH4_pos3" spec="TreeIntervals" tree="@Tree.t:NADH4_pos3"/>
        </distribution>
        <distribution id="CoalescentConstant.t:NADH5_pos1" spec="Coalescent">
            <populationModel id="ConstantPopulation.t:NADH5_pos1" spec="ConstantPopulation"
popSize="@popSize.t:NADH5_pos1"/>
            <treeIntervals id="TreeIntervals.t:NADH5_pos1" spec="TreeIntervals" tree="@Tree.t:NADH5_pos1"/>
        </distribution>
        <distribution id="CoalescentConstant.t:NADH5_pos2" spec="Coalescent">
```

```
<populationModel id="ConstantPopulation.t:NADH5_pos2" spec="ConstantPopulation"
popSize="@popSize.t:NADH5_pos2"/>
<treeIntervals id="TreeIntervals.t:NADH5_pos2" spec="TreeIntervals" tree="@Tree.t:NADH5_pos2"/>
</distribution>
<distribution id="CoalescentConstant.t:NADH5_pos3" spec="Coalescent">
<populationModel id="ConstantPopulation.t:NADH5_pos3" spec="ConstantPopulation"
popSize="@popSize.t:NADH5_pos3"/>
<treeIntervals id="TreeIntervals.t:NADH5_pos3" spec="TreeIntervals" tree="@Tree.t:NADH5_pos3"/>
</distribution>
<distribution id="CoalescentConstant.t:NADH6_pos1" spec="Coalescent">
<populationModel id="ConstantPopulation.t:NADH6_pos1" spec="ConstantPopulation"
popSize="@popSize.t:NADH6_pos1"/>
<treeIntervals id="TreeIntervals.t:NADH6_pos1" spec="TreeIntervals" tree="@Tree.t:NADH6_pos1"/>
</distribution>
<distribution id="CoalescentConstant.t:NADH6_pos2" spec="Coalescent">
<populationModel id="ConstantPopulation.t:NADH6_pos2" spec="ConstantPopulation"
popSize="@popSize.t:NADH6_pos2"/>
<treeIntervals id="TreeIntervals.t:NADH6_pos2" spec="TreeIntervals" tree="@Tree.t:NADH6_pos2"/>
</distribution>
<distribution id="CoalescentConstant.t:NADH6_pos3" spec="Coalescent">
<populationModel id="ConstantPopulation.t:NADH6_pos3" spec="ConstantPopulation"
popSize="@popSize.t:NADH6_pos3"/>
<treeIntervals id="TreeIntervals.t:NADH6_pos3" spec="TreeIntervals" tree="@Tree.t:NADH6_pos3"/>
</distribution>
<distribution id="CoalescentConstant.t:atp6_pos1" spec="Coalescent">
<populationModel id="ConstantPopulation.t:atp6_pos1" spec="ConstantPopulation"
popSize="@popSize.t:atp6_pos1"/>
<treeIntervals id="TreeIntervals.t:atp6_pos1" spec="TreeIntervals" tree="@Tree.t:atp6_pos1"/>
</distribution>
<distribution id="CoalescentConstant.t:atp6_pos2" spec="Coalescent">
<populationModel id="ConstantPopulation.t:atp6_pos2" spec="ConstantPopulation"
popSize="@popSize.t:atp6_pos2"/>
<treeIntervals id="TreeIntervals.t:atp6_pos2" spec="TreeIntervals" tree="@Tree.t:atp6_pos2"/>
</distribution>
<distribution id="CoalescentConstant.t:atp6_pos3" spec="Coalescent">
<populationModel id="ConstantPopulation.t:atp6_pos3" spec="ConstantPopulation"
popSize="@popSize.t:atp6_pos3"/>
<treeIntervals id="TreeIntervals.t:atp6_pos3" spec="TreeIntervals" tree="@Tree.t:atp6_pos3"/>
</distribution>
<prior id="ClockPrior.c:COX1_pos1" name="distribution" x="@clockRate.c:COX1_pos1">
<Normal id="Normal.37" name="distr">
<parameter id="RealParameter.1557" spec="parameter.RealParameter" estimate="false"
name="mean">2.93E-5</parameter>
<parameter id="RealParameter.1558" spec="parameter.RealParameter" estimate="false"
name="sigma">1.0</parameter>
</Normal>
</prior>
<prior id="ClockPrior.c:COX1_pos2" name="distribution" x="@clockRate.c:COX1_pos2">
<Normal id="Normal.38" name="distr">
<parameter id="RealParameter.1559" spec="parameter.RealParameter" estimate="false"
name="mean">2.93E-5</parameter>
<parameter id="RealParameter.1560" spec="parameter.RealParameter" estimate="false"
name="sigma">1.0</parameter>
</Normal>
</prior>
<prior id="ClockPrior.c:COX1_pos3" name="distribution" x="@clockRate.c:COX1_pos3">
<Normal id="Normal.39" name="distr">
<parameter id="RealParameter.1561" spec="parameter.RealParameter" estimate="false"
name="mean">2.93</parameter>
<parameter id="RealParameter.1562" spec="parameter.RealParameter" estimate="false"
name="sigma">1.0</parameter>
</Normal>
</prior>
```

```xml
<prior id="ClockPrior.c:COX2_pos1" name="distribution" x="@clockRate.c:COX2_pos1">
<Normal id="Normal.40" name="distr">
<parameter id="RealParameter.1563" spec="parameter.RealParameter" estimate="false" name="mean">2.93E-5</parameter>
<parameter id="RealParameter.1564" spec="parameter.RealParameter" estimate="false" name="sigma">1.0</parameter>
</Normal>
</prior>
<prior id="ClockPrior.c:COX2_pos2" name="distribution" x="@clockRate.c:COX2_pos2">
<Normal id="Normal.41" name="distr">
<parameter id="RealParameter.1565" spec="parameter.RealParameter" estimate="false" name="mean">2.93E-5</parameter>
<parameter id="RealParameter.1566" spec="parameter.RealParameter" estimate="false" name="sigma">1.0</parameter>
</Normal>
</prior>
<prior id="ClockPrior.c:COX2_pos3" name="distribution" x="@clockRate.c:COX2_pos3">
<Normal id="Normal.42" name="distr">
<parameter id="RealParameter.1567" spec="parameter.RealParameter" estimate="false" name="mean">2.93</parameter>
<parameter id="RealParameter.1568" spec="parameter.RealParameter" estimate="false" name="sigma">1.0</parameter>
</Normal>
</prior>
<prior id="ClockPrior.c:COX3_pos1" name="distribution" x="@clockRate.c:COX3_pos1">
<Normal id="Normal.43" name="distr">
<parameter id="RealParameter.1569" spec="parameter.RealParameter" estimate="false" name="mean">2.93E-5</parameter>
<parameter id="RealParameter.1570" spec="parameter.RealParameter" estimate="false" name="sigma">1.0</parameter>
</Normal>
</prior>
<prior id="ClockPrior.c:COX3_pos2" name="distribution" x="@clockRate.c:COX3_pos2">
<Normal id="Normal.44" name="distr">
<parameter id="RealParameter.1571" spec="parameter.RealParameter" estimate="false" name="mean">0.9</parameter>
<parameter id="RealParameter.1572" spec="parameter.RealParameter" estimate="false" name="sigma">1.0</parameter>
</Normal>
</prior>
<prior id="ClockPrior.c:COX3_pos3" name="distribution" x="@clockRate.c:COX3_pos3">
<Normal id="Normal.45" name="distr">
<parameter id="RealParameter.1573" spec="parameter.RealParameter" estimate="false" name="mean">2.9</parameter>
<parameter id="RealParameter.1574" spec="parameter.RealParameter" estimate="false" name="sigma">1.0</parameter>
</Normal>
</prior>
<prior id="ClockPrior.c:CYTB_pos1" name="distribution" x="@clockRate.c:CYTB_pos1">
<Normal id="Normal.46" name="distr">
<parameter id="RealParameter.1575" spec="parameter.RealParameter" estimate="false" name="mean">2.93E-5</parameter>
<parameter id="RealParameter.1576" spec="parameter.RealParameter" estimate="false" name="sigma">1.0</parameter>
</Normal>
</prior>
<prior id="ClockPrior.c:CYTB_pos2" name="distribution" x="@clockRate.c:CYTB_pos2">
<Normal id="Normal.47" name="distr">
<parameter id="RealParameter.1577" spec="parameter.RealParameter" estimate="false" name="mean">2.93E-5</parameter>
<parameter id="RealParameter.1578" spec="parameter.RealParameter" estimate="false" name="sigma">1.0</parameter>
</Normal>
```

```xml
</prior>
<prior id="ClockPrior.c:CYTB_pos3" name="distribution" x="@clockRate.c:CYTB_pos3">
<Normal id="Normal.48" name="distr">
<parameter id="RealParameter.1579" spec="parameter.RealParameter" estimate="false" name="mean">2.9</parameter>
<parameter id="RealParameter.1580" spec="parameter.RealParameter" estimate="false" name="sigma">1.0</parameter>
</Normal>
</prior>
<prior id="ClockPrior.c:Mytilidae27sq12PCGs" name="distribution" x="@clockRate.c:Mytilidae27sq12PCGs">
<Normal id="Normal.49" name="distr">
<parameter id="RealParameter.1581" spec="parameter.RealParameter" estimate="false" name="mean">2.93E-5</parameter>
<parameter id="RealParameter.1582" spec="parameter.RealParameter" estimate="false" name="sigma">1.0</parameter>
</Normal>
</prior>
<prior id="ClockPrior.c:NADH1_pos1" name="distribution" x="@clockRate.c:NADH1_pos1">
<Normal id="Normal.50" name="distr">
<parameter id="RealParameter.1583" spec="parameter.RealParameter" estimate="false" name="mean">2.93E-5</parameter>
<parameter id="RealParameter.1584" spec="parameter.RealParameter" estimate="false" name="sigma">1.0</parameter>
</Normal>
</prior>
<prior id="ClockPrior.c:NADH1_pos2" name="distribution" x="@clockRate.c:NADH1_pos2">
<Normal id="Normal.51" name="distr">
<parameter id="RealParameter.1585" spec="parameter.RealParameter" estimate="false" name="mean">2.93E-5</parameter>
<parameter id="RealParameter.1586" spec="parameter.RealParameter" estimate="false" name="sigma">1.0</parameter>
</Normal>
</prior>
<prior id="ClockPrior.c:NADH1_pos3" name="distribution" x="@clockRate.c:NADH1_pos3">
<Normal id="Normal.52" name="distr">
<parameter id="RealParameter.1587" spec="parameter.RealParameter" estimate="false" name="mean">2.9</parameter>
<parameter id="RealParameter.1588" spec="parameter.RealParameter" estimate="false" name="sigma">1.0</parameter>
</Normal>
</prior>
<prior id="ClockPrior.c:NADH2_pos1" name="distribution" x="@clockRate.c:NADH2_pos1">
<Normal id="Normal.98" name="distr">
<parameter id="RealParameter.1677" spec="parameter.RealParameter" estimate="false" name="mean">2.93E-5</parameter>
<parameter id="RealParameter.1678" spec="parameter.RealParameter" estimate="false" name="sigma">1.0</parameter>
</Normal>
</prior>
<prior id="ClockPrior.c:NADH2_pos2" name="distribution" x="@clockRate.c:NADH2_pos2">
<Normal id="Normal.53" name="distr">
<parameter id="RealParameter.1589" spec="parameter.RealParameter" estimate="false" name="mean">2.93E-5</parameter>
<parameter id="RealParameter.1590" spec="parameter.RealParameter" estimate="false" name="sigma">1.0</parameter>
</Normal>
</prior>
<prior id="ClockPrior.c:NADH2_pos3" name="distribution" x="@clockRate.c:NADH2_pos3">
<Normal id="Normal.54" name="distr">
<parameter id="RealParameter.1591" spec="parameter.RealParameter" estimate="false" name="mean">2.9</parameter>
```

```xml
<parameter id="RealParameter.1592" spec="parameter.RealParameter" estimate="false" name="sigma">1.0</parameter>
</Normal>
</prior>
<prior id="ClockPrior.c:NADH3_pos1" name="distribution" x="@clockRate.c:NADH3_pos1">
<Normal id="Normal.55" name="distr">
<parameter id="RealParameter.1593" spec="parameter.RealParameter" estimate="false" name="mean">2.93E-5</parameter>
<parameter id="RealParameter.1594" spec="parameter.RealParameter" estimate="false" name="sigma">1.0</parameter>
</Normal>
</prior>
<prior id="ClockPrior.c:NADH3_pos2" name="distribution" x="@clockRate.c:NADH3_pos2">
<Normal id="Normal.56" name="distr">
<parameter id="RealParameter.1595" spec="parameter.RealParameter" estimate="false" name="mean">2.93E-5</parameter>
<parameter id="RealParameter.1596" spec="parameter.RealParameter" estimate="false" name="sigma">1.0</parameter>
</Normal>
</prior>
<prior id="ClockPrior.c:NADH3_pos3" name="distribution" x="@clockRate.c:NADH3_pos3">
<Normal id="Normal.57" name="distr">
<parameter id="RealParameter.1597" spec="parameter.RealParameter" estimate="false" name="mean">2.9</parameter>
<parameter id="RealParameter.1598" spec="parameter.RealParameter" estimate="false" name="sigma">1.0</parameter>
</Normal>
</prior>
<prior id="ClockPrior.c:NADH4L_pos1" name="distribution" x="@clockRate.c:NADH4L_pos1">
<Normal id="Normal.58" name="distr">
<parameter id="RealParameter.1599" spec="parameter.RealParameter" estimate="false" name="mean">2.93E-5</parameter>
<parameter id="RealParameter.1600" spec="parameter.RealParameter" estimate="false" name="sigma">1.0</parameter>
</Normal>
</prior>
<prior id="ClockPrior.c:NADH4L_pos2" name="distribution" x="@clockRate.c:NADH4L_pos2">
<Normal id="Normal.59" name="distr">
<parameter id="RealParameter.1601" spec="parameter.RealParameter" estimate="false" name="mean">2.93E-5</parameter>
<parameter id="RealParameter.1602" spec="parameter.RealParameter" estimate="false" name="sigma">1.0</parameter>
</Normal>
</prior>
<prior id="ClockPrior.c:NADH4L_pos3" name="distribution" x="@clockRate.c:NADH4L_pos3">
<Normal id="Normal.60" name="distr">
<parameter id="RealParameter.1603" spec="parameter.RealParameter" estimate="false" name="mean">2.9</parameter>
<parameter id="RealParameter.1604" spec="parameter.RealParameter" estimate="false" name="sigma">1.0</parameter>
</Normal>
</prior>
<prior id="ClockPrior.c:NADH4_pos1" name="distribution" x="@clockRate.c:NADH4_pos1">
<Normal id="Normal.61" name="distr">
<parameter id="RealParameter.1605" spec="parameter.RealParameter" estimate="false" name="mean">2.93E-5</parameter>
<parameter id="RealParameter.1606" spec="parameter.RealParameter" estimate="false" name="sigma">1.0</parameter>
</Normal>
</prior>
<prior id="ClockPrior.c:NADH4_pos2" name="distribution" x="@clockRate.c:NADH4_pos2">
<Normal id="Normal.62" name="distr">
```

```
                <parameter id="RealParameter.1607" spec="parameter.RealParameter" estimate="false"
name="mean">2.93E-5</parameter>
                <parameter id="RealParameter.1608" spec="parameter.RealParameter" estimate="false"
name="sigma">1.0</parameter>
            </Normal>
        </prior>
        <prior id="ClockPrior.c:NADH4_pos3" name="distribution" x="@clockRate.c:NADH4_pos3">
            <Normal id="Normal.63" name="distr">
                <parameter id="RealParameter.1609" spec="parameter.RealParameter" estimate="false"
name="mean">2.9</parameter>
                <parameter id="RealParameter.1610" spec="parameter.RealParameter" estimate="false"
name="sigma">1.0</parameter>
            </Normal>
        </prior>
        <prior id="ClockPrior.c:NADH5_pos1" name="distribution" x="@clockRate.c:NADH5_pos1">
            <Normal id="Normal.64" name="distr">
                <parameter id="RealParameter.1611" spec="parameter.RealParameter" estimate="false"
name="mean">2.93E-5</parameter>
                <parameter id="RealParameter.1612" spec="parameter.RealParameter" estimate="false"
name="sigma">1.0</parameter>
            </Normal>
        </prior>
        <prior id="ClockPrior.c:NADH5_pos2" name="distribution" x="@clockRate.c:NADH5_pos2">
            <Normal id="Normal.65" name="distr">
                <parameter id="RealParameter.1613" spec="parameter.RealParameter" estimate="false"
name="mean">2.93E-5</parameter>
                <parameter id="RealParameter.1614" spec="parameter.RealParameter" estimate="false"
name="sigma">1.0</parameter>
            </Normal>
        </prior>
        <prior id="ClockPrior.c:NADH5_pos3" name="distribution" x="@clockRate.c:NADH5_pos3">
            <Normal id="Normal.66" name="distr">
                <parameter id="RealParameter.1615" spec="parameter.RealParameter" estimate="false"
name="mean">2.9</parameter>
                <parameter id="RealParameter.1616" spec="parameter.RealParameter" estimate="false"
name="sigma">1.0</parameter>
            </Normal>
        </prior>
        <prior id="ClockPrior.c:NADH6_pos1" name="distribution" x="@clockRate.c:NADH6_pos1">
            <Normal id="Normal.67" name="distr">
                <parameter id="RealParameter.1617" spec="parameter.RealParameter" estimate="false"
name="mean">2.93E-5</parameter>
                <parameter id="RealParameter.1618" spec="parameter.RealParameter" estimate="false"
name="sigma">1.0</parameter>
            </Normal>
        </prior>
        <prior id="ClockPrior.c:NADH6_pos2" name="distribution" x="@clockRate.c:NADH6_pos2">
            <Normal id="Normal.68" name="distr">
                <parameter id="RealParameter.1619" spec="parameter.RealParameter" estimate="false"
name="mean">2.93E-5</parameter>
                <parameter id="RealParameter.1620" spec="parameter.RealParameter" estimate="false"
name="sigma">1.0</parameter>
            </Normal>
        </prior>
        <prior id="ClockPrior.c:NADH6_pos3" name="distribution" x="@clockRate.c:NADH6_pos3">
            <Normal id="Normal.69" name="distr">
                <parameter id="RealParameter.1621" spec="parameter.RealParameter" estimate="false"
name="mean">2.9</parameter>
                <parameter id="RealParameter.1622" spec="parameter.RealParameter" estimate="false"
name="sigma">1.0</parameter>
            </Normal>
        </prior>
        <prior id="ClockPrior.c:atp6_pos1" name="distribution" x="@clockRate.c:atp6_pos1">
```

```xml
<Normal id="Normal.70" name="distr">
<parameter id="RealParameter.1623" spec="parameter.RealParameter" estimate="false" name="mean">2.93E-5</parameter>
<parameter id="RealParameter.1624" spec="parameter.RealParameter" estimate="false" name="sigma">1.0</parameter>
</Normal>
</prior>
<prior id="ClockPrior.c:atp6_pos2" name="distribution" x="@clockRate.c:atp6_pos2">
<Normal id="Normal.71" name="distr">
<parameter id="RealParameter.1625" spec="parameter.RealParameter" estimate="false" name="mean">2.93E-5</parameter>
<parameter id="RealParameter.1626" spec="parameter.RealParameter" estimate="false" name="sigma">1.0</parameter>
</Normal>
</prior>
<prior id="ClockPrior.c:atp6_pos3" name="distribution" x="@clockRate.c:atp6_pos3">
<Normal id="Normal.72" name="distr">
<parameter id="RealParameter.1627" spec="parameter.RealParameter" estimate="false" name="mean">2.9</parameter>
<parameter id="RealParameter.1628" spec="parameter.RealParameter" estimate="false" name="sigma">1.0</parameter>
</Normal>
</prior>
<prior id="FrequenciesPrior.s:COX1_pos1" name="distribution" x="@freqParameter.s:COX1_pos1">
<Uniform id="Uniform.115" name="distr"/>
</prior>
<prior id="FrequenciesPrior.s:COX1_pos3" name="distribution" x="@freqParameter.s:COX1_pos3">
<Uniform id="Uniform.116" name="distr"/>
</prior>
<prior id="FrequenciesPrior.s:COX2_pos1" name="distribution" x="@freqParameter.s:COX2_pos1">
<Uniform id="Uniform.117" name="distr"/>
</prior>
<prior id="FrequenciesPrior.s:COX2_pos2" name="distribution" x="@freqParameter.s:COX2_pos2">
<Uniform id="Uniform.66" name="distr"/>
</prior>
<prior id="FrequenciesPrior.s:COX2_pos3" name="distribution" x="@freqParameter.s:COX2_pos3">
<Uniform id="Uniform.118" name="distr"/>
</prior>
<prior id="FrequenciesPrior.s:COX3_pos1" name="distribution" x="@freqParameter.s:COX3_pos1">
<Uniform id="Uniform.119" name="distr"/>
</prior>
<prior id="FrequenciesPrior.s:COX3_pos2" name="distribution" x="@freqParameter.s:COX3_pos2">
<Uniform id="Uniform.120" name="distr"/>
</prior>
<prior id="FrequenciesPrior.s:COX3_pos3" name="distribution" x="@freqParameter.s:COX3_pos3">
<Uniform id="Uniform.121" name="distr"/>
</prior>
<prior id="FrequenciesPrior.s:CYTB_pos1" name="distribution" x="@freqParameter.s:CYTB_pos1">
<Uniform id="Uniform.122" name="distr"/>
</prior>
<prior id="FrequenciesPrior.s:CYTB_pos2" name="distribution" x="@freqParameter.s:CYTB_pos2">
<Uniform id="Uniform.123" name="distr"/>
</prior>
<prior id="FrequenciesPrior.s:CYTB_pos3" name="distribution" x="@freqParameter.s:CYTB_pos3">
<Uniform id="Uniform.124" name="distr"/>
</prior>
<prior id="FrequenciesPrior.s:Mytilidae27sq12PCGs" name="distribution" x="@freqParameter.s:Mytilidae27sq12PCGs">
<Uniform id="Uniform.361" name="distr"/>
</prior>
<prior id="FrequenciesPrior.s:NADH1_pos1" name="distribution" x="@freqParameter.s:NADH1_pos1">
<Uniform id="Uniform.125" name="distr"/>
</prior>
```

```
<prior id="FrequenciesPrior.s:NADH1_pos2" name="distribution" x="@freqParameter.s:NADH1_pos2">
  <Uniform id="Uniform.126" name="distr"/>
</prior>
<prior id="FrequenciesPrior.s:NADH1_pos3" name="distribution" x="@freqParameter.s:NADH1_pos3">
  <Uniform id="Uniform.127" name="distr"/>
</prior>
<prior id="FrequenciesPrior.s:NADH2_pos1" name="distribution" x="@freqParameter.s:NADH2_pos1">
  <Uniform id="Uniform.128" name="distr"/>
</prior>
<prior id="FrequenciesPrior.s:NADH2_pos2" name="distribution" x="@freqParameter.s:NADH2_pos2">
  <Uniform id="Uniform.129" name="distr"/>
</prior>
<prior id="FrequenciesPrior.s:NADH2_pos3" name="distribution" x="@freqParameter.s:NADH2_pos3">
  <Uniform id="Uniform.130" name="distr"/>
</prior>
<prior id="FrequenciesPrior.s:NADH3_pos1" name="distribution" x="@freqParameter.s:NADH3_pos1">
  <Uniform id="Uniform.131" name="distr"/>
</prior>
<prior id="FrequenciesPrior.s:NADH3_pos2" name="distribution" x="@freqParameter.s:NADH3_pos2">
  <Uniform id="Uniform.132" name="distr"/>
</prior>
<prior id="FrequenciesPrior.s:NADH3_pos3" name="distribution" x="@freqParameter.s:NADH3_pos3">
  <Uniform id="Uniform.133" name="distr"/>
</prior>
<prior id="FrequenciesPrior.s:NADH4L_pos1" name="distribution"
x="@freqParameter.s:NADH4L_pos1">
  <Uniform id="Uniform.137" name="distr"/>
</prior>
<prior id="FrequenciesPrior.s:NADH4L_pos2" name="distribution"
x="@freqParameter.s:NADH4L_pos2">
  <Uniform id="Uniform.138" name="distr"/>
</prior>
<prior id="FrequenciesPrior.s:NADH4L_pos3" name="distribution"
x="@freqParameter.s:NADH4L_pos3">
  <Uniform id="Uniform.139" name="distr"/>
</prior>
<prior id="FrequenciesPrior.s:NADH4_pos1" name="distribution" x="@freqParameter.s:NADH4_pos1">
  <Uniform id="Uniform.134" name="distr"/>
</prior>
<prior id="FrequenciesPrior.s:NADH4_pos2" name="distribution" x="@freqParameter.s:NADH4_pos2">
  <Uniform id="Uniform.135" name="distr"/>
</prior>
<prior id="FrequenciesPrior.s:NADH4_pos3" name="distribution" x="@freqParameter.s:NADH4_pos3">
  <Uniform id="Uniform.136" name="distr"/>
</prior>
<prior id="FrequenciesPrior.s:NADH5_pos1" name="distribution" x="@freqParameter.s:NADH5_pos1">
  <Uniform id="Uniform.140" name="distr"/>
</prior>
<prior id="FrequenciesPrior.s:NADH5_pos2" name="distribution" x="@freqParameter.s:NADH5_pos2">
  <Uniform id="Uniform.141" name="distr"/>
</prior>
<prior id="FrequenciesPrior.s:NADH5_pos3" name="distribution" x="@freqParameter.s:NADH5_pos3">
  <Uniform id="Uniform.142" name="distr"/>
</prior>
<prior id="FrequenciesPrior.s:NADH6_pos1" name="distribution" x="@freqParameter.s:NADH6_pos1">
  <Uniform id="Uniform.143" name="distr"/>
</prior>
<prior id="FrequenciesPrior.s:NADH6_pos2" name="distribution" x="@freqParameter.s:NADH6_pos2">
  <Uniform id="Uniform.144" name="distr"/>
</prior>
<prior id="FrequenciesPrior.s:NADH6_pos3" name="distribution" x="@freqParameter.s:NADH6_pos3">
  <Uniform id="Uniform.145" name="distr"/>
</prior>
```

```
<prior id="FrequenciesPrior.s:atp6_pos1" name="distribution" x="@freqParameter.s:atp6_pos1">
  <Uniform id="Uniform.111" name="distr"/>
</prior>
<prior id="FrequenciesPrior.s:atp6_pos2" name="distribution" x="@freqParameter.s:atp6_pos2">
  <Uniform id="Uniform.112" name="distr"/>
</prior>
<prior id="FrequenciesPrior.s:atp6_pos3" name="distribution" x="@freqParameter.s:atp6_pos3">
  <Uniform id="Uniform.113" name="distr"/>
</prior>
<prior id="GammaShapePrior.s:COX1_pos1" name="distribution" x="@gammaShape.s:COX1_pos1">
  <Exponential id="Exponential.3" name="distr">
    <parameter id="RealParameter.3" spec="parameter.RealParameter" estimate="false"
name="mean">1.0</parameter>
  </Exponential>
</prior>
<prior id="GammaShapePrior.s:COX1_pos2" name="distribution" x="@gammaShape.s:COX1_pos2">
  <Exponential id="Exponential.4" name="distr">
    <parameter id="RealParameter.4" spec="parameter.RealParameter" estimate="false"
name="mean">1.0</parameter>
  </Exponential>
</prior>
<prior id="GammaShapePrior.s:COX1_pos3" name="distribution" x="@gammaShape.s:COX1_pos3">
  <Exponential id="Exponential.5" name="distr">
    <parameter id="RealParameter.5" spec="parameter.RealParameter" estimate="false"
name="mean">1.0</parameter>
  </Exponential>
</prior>
<prior id="GammaShapePrior.s:COX2_pos1" name="distribution" x="@gammaShape.s:COX2_pos1">
  <Exponential id="Exponential.6" name="distr">
    <parameter id="RealParameter.6" spec="parameter.RealParameter" estimate="false"
name="mean">1.0</parameter>
  </Exponential>
</prior>
<prior id="GammaShapePrior.s:COX2_pos2" name="distribution" x="@gammaShape.s:COX2_pos2">
  <Exponential id="Exponential.7" name="distr">
    <parameter id="RealParameter.7" spec="parameter.RealParameter" estimate="false"
name="mean">1.0</parameter>
  </Exponential>
</prior>
<prior id="GammaShapePrior.s:COX2_pos3" name="distribution" x="@gammaShape.s:COX2_pos3">
  <Exponential id="Exponential.8" name="distr">
    <parameter id="RealParameter.8" spec="parameter.RealParameter" estimate="false"
name="mean">1.0</parameter>
  </Exponential>
</prior>
<prior id="GammaShapePrior.s:COX3_pos1" name="distribution" x="@gammaShape.s:COX3_pos1">
  <Exponential id="Exponential.9" name="distr">
    <parameter id="RealParameter.9" spec="parameter.RealParameter" estimate="false"
name="mean">1.0</parameter>
  </Exponential>
</prior>
<prior id="GammaShapePrior.s:COX3_pos2" name="distribution" x="@gammaShape.s:COX3_pos2">
  <Exponential id="Exponential.10" name="distr">
    <parameter id="RealParameter.10" spec="parameter.RealParameter" estimate="false"
name="mean">1.0</parameter>
  </Exponential>
</prior>
<prior id="GammaShapePrior.s:COX3_pos3" name="distribution" x="@gammaShape.s:COX3_pos3">
  <Exponential id="Exponential.11" name="distr">
    <parameter id="RealParameter.11" spec="parameter.RealParameter" estimate="false"
name="mean">1.0</parameter>
  </Exponential>
</prior>
```

```
<prior id="GammaShapePrior.s:CYTB_pos1" name="distribution" x="@gammaShape.s:CYTB_pos1">
    <Exponential id="Exponential.12" name="distr">
        <parameter id="RealParameter.12" spec="parameter.RealParameter" estimate="false"
name="mean">1.0</parameter>
    </Exponential>
</prior>
<prior id="GammaShapePrior.s:CYTB_pos2" name="distribution" x="@gammaShape.s:CYTB_pos2">
    <Exponential id="Exponential.13" name="distr">
        <parameter id="RealParameter.13" spec="parameter.RealParameter" estimate="false"
name="mean">1.0</parameter>
    </Exponential>
</prior>
<prior id="GammaShapePrior.s:CYTB_pos3" name="distribution" x="@gammaShape.s:CYTB_pos3">
    <Exponential id="Exponential.14" name="distr">
        <parameter id="RealParameter.14" spec="parameter.RealParameter" estimate="false"
name="mean">1.0</parameter>
    </Exponential>
</prior>
<prior id="GammaShapePrior.s:Mytilidae27sq12PCGs" name="distribution"
x="@gammaShape.s:Mytilidae27sq12PCGs">
    <Exponential id="Exponential.36" name="distr">
        <parameter id="RealParameter.36" spec="parameter.RealParameter" estimate="false"
name="mean">2.0</parameter>
    </Exponential>
</prior>
<prior id="GammaShapePrior.s:NADH1_pos1" name="distribution" x="@gammaShape.s:NADH1_pos1">
    <Exponential id="Exponential.15" name="distr">
        <parameter id="RealParameter.15" spec="parameter.RealParameter" estimate="false"
name="mean">1.0</parameter>
    </Exponential>
</prior>
<prior id="GammaShapePrior.s:NADH1_pos2" name="distribution" x="@gammaShape.s:NADH1_pos2">
    <Exponential id="Exponential.16" name="distr">
        <parameter id="RealParameter.16" spec="parameter.RealParameter" estimate="false"
name="mean">1.0</parameter>
    </Exponential>
</prior>
<prior id="GammaShapePrior.s:NADH1_pos3" name="distribution" x="@gammaShape.s:NADH1_pos3">
    <Exponential id="Exponential.17" name="distr">
        <parameter id="RealParameter.17" spec="parameter.RealParameter" estimate="false"
name="mean">1.0</parameter>
    </Exponential>
</prior>
<prior id="GammaShapePrior.s:NADH2_pos1" name="distribution" x="@gammaShape.s:NADH2_pos1">
    <Exponential id="Exponential.18" name="distr">
        <parameter id="RealParameter.18" spec="parameter.RealParameter" estimate="false"
name="mean">1.0</parameter>
    </Exponential>
</prior>
<prior id="GammaShapePrior.s:NADH2_pos2" name="distribution" x="@gammaShape.s:NADH2_pos2">
    <Exponential id="Exponential.19" name="distr">
        <parameter id="RealParameter.19" spec="parameter.RealParameter" estimate="false"
name="mean">1.0</parameter>
    </Exponential>
</prior>
<prior id="GammaShapePrior.s:NADH2_pos3" name="distribution" x="@gammaShape.s:NADH2_pos3">
    <Exponential id="Exponential.20" name="distr">
        <parameter id="RealParameter.20" spec="parameter.RealParameter" estimate="false"
name="mean">1.0</parameter>
    </Exponential>
</prior>
<prior id="GammaShapePrior.s:NADH3_pos1" name="distribution" x="@gammaShape.s:NADH3_pos1">
    <Exponential id="Exponential.21" name="distr">
```

<parameter id="RealParameter.21" spec="parameter.RealParameter" estimate="false"
name="mean">1.0</parameter>
</Exponential>
</prior>
<prior id="GammaShapePrior.s:NADH3_pos2" name="distribution" x="@gammaShape.s:NADH3_pos2">
<Exponential id="Exponential.22" name="distr">
<parameter id="RealParameter.22" spec="parameter.RealParameter" estimate="false"
name="mean">1.0</parameter>
</Exponential>
</prior>
<prior id="GammaShapePrior.s:NADH3_pos3" name="distribution" x="@gammaShape.s:NADH3_pos3">
<Exponential id="Exponential.23" name="distr">
<parameter id="RealParameter.23" spec="parameter.RealParameter" estimate="false"
name="mean">1.0</parameter>
</Exponential>
</prior>
<prior id="GammaShapePrior.s:NADH4L_pos1" name="distribution"
x="@gammaShape.s:NADH4L_pos1">
<Exponential id="Exponential.27" name="distr">
<parameter id="RealParameter.27" spec="parameter.RealParameter" estimate="false"
name="mean">1.0</parameter>
</Exponential>
</prior>
<prior id="GammaShapePrior.s:NADH4L_pos2" name="distribution"
x="@gammaShape.s:NADH4L_pos2">
<Exponential id="Exponential.28" name="distr">
<parameter id="RealParameter.28" spec="parameter.RealParameter" estimate="false"
name="mean">1.0</parameter>
</Exponential>
</prior>
<prior id="GammaShapePrior.s:NADH4L_pos3" name="distribution"
x="@gammaShape.s:NADH4L_pos3">
<Exponential id="Exponential.29" name="distr">
<parameter id="RealParameter.29" spec="parameter.RealParameter" estimate="false"
name="mean">1.0</parameter>
</Exponential>
</prior>
<prior id="GammaShapePrior.s:NADH4_pos1" name="distribution" x="@gammaShape.s:NADH4_pos1">
<Exponential id="Exponential.24" name="distr">
<parameter id="RealParameter.24" spec="parameter.RealParameter" estimate="false"
name="mean">1.0</parameter>
</Exponential>
</prior>
<prior id="GammaShapePrior.s:NADH4_pos2" name="distribution" x="@gammaShape.s:NADH4_pos2">
<Exponential id="Exponential.25" name="distr">
<parameter id="RealParameter.25" spec="parameter.RealParameter" estimate="false"
name="mean">1.0</parameter>
</Exponential>
</prior>
<prior id="GammaShapePrior.s:NADH4_pos3" name="distribution" x="@gammaShape.s:NADH4_pos3">
<Exponential id="Exponential.26" name="distr">
<parameter id="RealParameter.26" spec="parameter.RealParameter" estimate="false"
name="mean">1.0</parameter>
</Exponential>
</prior>
<prior id="GammaShapePrior.s:NADH5_pos1" name="distribution" x="@gammaShape.s:NADH5_pos1">
<Exponential id="Exponential.30" name="distr">
<parameter id="RealParameter.30" spec="parameter.RealParameter" estimate="false"
name="mean">1.0</parameter>
</Exponential>
</prior>
<prior id="GammaShapePrior.s:NADH5_pos2" name="distribution" x="@gammaShape.s:NADH5_pos2">
<Exponential id="Exponential.31" name="distr">

```
                <parameter id="RealParameter.31" spec="parameter.RealParameter" estimate="false"
name="mean">1.0</parameter>
            </Exponential>
        </prior>
        <prior id="GammaShapePrior.s:NADH5_pos3" name="distribution" x="@gammaShape.s:NADH5_pos3">
            <Exponential id="Exponential.32" name="distr">
                <parameter id="RealParameter.32" spec="parameter.RealParameter" estimate="false"
name="mean">1.0</parameter>
            </Exponential>
        </prior>
        <prior id="GammaShapePrior.s:NADH6_pos1" name="distribution" x="@gammaShape.s:NADH6_pos1">
            <Exponential id="Exponential.33" name="distr">
                <parameter id="RealParameter.33" spec="parameter.RealParameter" estimate="false"
name="mean">1.0</parameter>
            </Exponential>
        </prior>
        <prior id="GammaShapePrior.s:NADH6_pos2" name="distribution" x="@gammaShape.s:NADH6_pos2">
            <Exponential id="Exponential.34" name="distr">
                <parameter id="RealParameter.34" spec="parameter.RealParameter" estimate="false"
name="mean">1.0</parameter>
            </Exponential>
        </prior>
        <prior id="GammaShapePrior.s:NADH6_pos3" name="distribution" x="@gammaShape.s:NADH6_pos3">
            <Exponential id="Exponential.35" name="distr">
                <parameter id="RealParameter.35" spec="parameter.RealParameter" estimate="false"
name="mean">1.0</parameter>
            </Exponential>
        </prior>
        <prior id="GammaShapePrior.s:atp6_pos1" name="distribution" x="@gammaShape.s:atp6_pos1">
            <Exponential id="Exponential.0" name="distr">
                <parameter id="RealParameter.0" spec="parameter.RealParameter" estimate="false"
name="mean">1.0</parameter>
            </Exponential>
        </prior>
        <prior id="GammaShapePrior.s:atp6_pos2" name="distribution" x="@gammaShape.s:atp6_pos2">
            <Exponential id="Exponential.1" name="distr">
                <parameter id="RealParameter.1" spec="parameter.RealParameter" estimate="false"
name="mean">1.0</parameter>
            </Exponential>
        </prior>
        <prior id="GammaShapePrior.s:atp6_pos3" name="distribution" x="@gammaShape.s:atp6_pos3">
            <Exponential id="Exponential.2" name="distr">
                <parameter id="RealParameter.2" spec="parameter.RealParameter" estimate="false"
name="mean">1.0</parameter>
            </Exponential>
        </prior>
        <prior id="PopSizePrior.t:COX1_pos1" name="distribution" x="@popSize.t:COX1_pos1">
            <Normal id="Normal.73" name="distr">
                <parameter id="RealParameter.1555" spec="parameter.RealParameter" estimate="false"
name="mean">2.3</parameter>
                <parameter id="RealParameter.1556" spec="parameter.RealParameter" estimate="false"
name="sigma">1.0</parameter>
            </Normal>
        </prior>
        <prior id="PopSizePrior.t:COX1_pos2" name="distribution" x="@popSize.t:COX1_pos2">
            <Normal id="Normal.74" name="distr">
                <parameter id="RealParameter.1629" spec="parameter.RealParameter" estimate="false"
name="mean">2.3</parameter>
                <parameter id="RealParameter.1630" spec="parameter.RealParameter" estimate="false"
name="sigma">1.0</parameter>
            </Normal>
        </prior>
        <prior id="PopSizePrior.t:COX1_pos3" name="distribution" x="@popSize.t:COX1_pos3">
```

```xml
<Normal id="Normal.75" name="distr">
<parameter id="RealParameter.1631" spec="parameter.RealParameter" estimate="false" name="mean">2.3</parameter>
<parameter id="RealParameter.1632" spec="parameter.RealParameter" estimate="false" name="sigma">1.0</parameter>
</Normal>
</prior>
<prior id="PopSizePrior.t:COX2_pos1" name="distribution" x="@popSize.t:COX2_pos1">
<Normal id="Normal.76" name="distr">
<parameter id="RealParameter.1633" spec="parameter.RealParameter" estimate="false" name="mean">2.3</parameter>
<parameter id="RealParameter.1634" spec="parameter.RealParameter" estimate="false" name="sigma">1.0</parameter>
</Normal>
</prior>
<prior id="PopSizePrior.t:COX2_pos2" name="distribution" x="@popSize.t:COX2_pos2">
<Normal id="Normal.77" name="distr">
<parameter id="RealParameter.1635" spec="parameter.RealParameter" estimate="false" name="mean">2.3</parameter>
<parameter id="RealParameter.1636" spec="parameter.RealParameter" estimate="false" name="sigma">1.0</parameter>
</Normal>
</prior>
<prior id="PopSizePrior.t:COX2_pos3" name="distribution" x="@popSize.t:COX2_pos3">
<Normal id="Normal.79" name="distr">
<parameter id="RealParameter.1639" spec="parameter.RealParameter" estimate="false" name="mean">2.3</parameter>
<parameter id="RealParameter.1640" spec="parameter.RealParameter" estimate="false" name="sigma">1.0</parameter>
</Normal>
</prior>
<prior id="PopSizePrior.t:COX3_pos1" name="distribution" x="@popSize.t:COX3_pos1">
<Normal id="Normal.80" name="distr">
<parameter id="RealParameter.1641" spec="parameter.RealParameter" estimate="false" name="mean">2.3</parameter>
<parameter id="RealParameter.1642" spec="parameter.RealParameter" estimate="false" name="sigma">1.0</parameter>
</Normal>
</prior>
<prior id="PopSizePrior.t:COX3_pos2" name="distribution" x="@popSize.t:COX3_pos2">
<Normal id="Normal.81" name="distr">
<parameter id="RealParameter.1643" spec="parameter.RealParameter" estimate="false" name="mean">2.3</parameter>
<parameter id="RealParameter.1644" spec="parameter.RealParameter" estimate="false" name="sigma">1.0</parameter>
</Normal>
</prior>
<prior id="PopSizePrior.t:COX3_pos3" name="distribution" x="@popSize.t:COX3_pos3">
<Normal id="Normal.82" name="distr">
<parameter id="RealParameter.1645" spec="parameter.RealParameter" estimate="false" name="mean">2.3</parameter>
<parameter id="RealParameter.1646" spec="parameter.RealParameter" estimate="false" name="sigma">1.0</parameter>
</Normal>
</prior>
<prior id="PopSizePrior.t:CYTB_pos1" name="distribution" x="@popSize.t:CYTB_pos1">
<Normal id="Normal.83" name="distr">
<parameter id="RealParameter.1647" spec="parameter.RealParameter" estimate="false" name="mean">2.3</parameter>
<parameter id="RealParameter.1648" spec="parameter.RealParameter" estimate="false" name="sigma">1.0</parameter>
</Normal>
</prior>
```

```xml
<prior id="PopSizePrior.t:CYTB_pos2" name="distribution" x="@popSize.t:CYTB_pos2">
<Normal id="Normal.84" name="distr">
<parameter id="RealParameter.1649" spec="parameter.RealParameter" estimate="false" name="mean">2.3</parameter>
<parameter id="RealParameter.1650" spec="parameter.RealParameter" estimate="false" name="sigma">1.0</parameter>
</Normal>
</prior>
<prior id="PopSizePrior.t:CYTB_pos3" name="distribution" x="@popSize.t:CYTB_pos3">
<Normal id="Normal.85" name="distr">
<parameter id="RealParameter.1651" spec="parameter.RealParameter" estimate="false" name="mean">2.3</parameter>
<parameter id="RealParameter.1652" spec="parameter.RealParameter" estimate="false" name="sigma">1.0</parameter>
</Normal>
</prior>
<prior id="PopSizePrior.t:Mytilidae27sq12PCGs" name="distribution" x="@popSize.t:Mytilidae27sq12PCGs">
<Normal id="Normal.78" name="distr">
<parameter id="RealParameter.1637" spec="parameter.RealParameter" estimate="false" name="mean">2.3</parameter>
<parameter id="RealParameter.1638" spec="parameter.RealParameter" estimate="false" name="sigma">1.0</parameter>
</Normal>
</prior>
<prior id="PopSizePrior.t:NADH1_pos1" name="distribution" x="@popSize.t:NADH1_pos1">
<Normal id="Normal.86" name="distr">
<parameter id="RealParameter.1653" spec="parameter.RealParameter" estimate="false" name="mean">2.3</parameter>
<parameter id="RealParameter.1654" spec="parameter.RealParameter" estimate="false" name="sigma">1.0</parameter>
</Normal>
</prior>
<prior id="PopSizePrior.t:NADH1_pos2" name="distribution" x="@popSize.t:NADH1_pos2">
<Normal id="Normal.87" name="distr">
<parameter id="RealParameter.1655" spec="parameter.RealParameter" estimate="false" name="mean">2.3</parameter>
<parameter id="RealParameter.1656" spec="parameter.RealParameter" estimate="false" name="sigma">1.0</parameter>
</Normal>
</prior>
<prior id="PopSizePrior.t:NADH1_pos3" name="distribution" x="@popSize.t:NADH1_pos3">
<Normal id="Normal.88" name="distr">
<parameter id="RealParameter.1657" spec="parameter.RealParameter" estimate="false" name="mean">2.3</parameter>
<parameter id="RealParameter.1658" spec="parameter.RealParameter" estimate="false" name="sigma">1.0</parameter>
</Normal>
</prior>
<prior id="PopSizePrior.t:NADH2_pos1" name="distribution" x="@popSize.t:NADH2_pos1">
<Normal id="Normal.89" name="distr">
<parameter id="RealParameter.1659" spec="parameter.RealParameter" estimate="false" name="mean">2.3</parameter>
<parameter id="RealParameter.1660" spec="parameter.RealParameter" estimate="false" name="sigma">1.0</parameter>
</Normal>
</prior>
<prior id="PopSizePrior.t:NADH2_pos2" name="distribution" x="@popSize.t:NADH2_pos2">
<Normal id="Normal.90" name="distr">
<parameter id="RealParameter.1661" spec="parameter.RealParameter" estimate="false" name="mean">2.3</parameter>
<parameter id="RealParameter.1662" spec="parameter.RealParameter" estimate="false" name="sigma">1.0</parameter>
```

```xml
</Normal>
</prior>
<prior id="PopSizePrior.t:NADH2_pos3" name="distribution" x="@popSize.t:NADH2_pos3">
<Normal id="Normal.91" name="distr">
<parameter id="RealParameter.1663" spec="parameter.RealParameter" estimate="false" name="mean">2.3</parameter>
<parameter id="RealParameter.1664" spec="parameter.RealParameter" estimate="false" name="sigma">1.0</parameter>
</Normal>
</prior>
<prior id="PopSizePrior.t:NADH3_pos1" name="distribution" x="@popSize.t:NADH3_pos1">
<Normal id="Normal.92" name="distr">
<parameter id="RealParameter.1665" spec="parameter.RealParameter" estimate="false" name="mean">2.3</parameter>
<parameter id="RealParameter.1666" spec="parameter.RealParameter" estimate="false" name="sigma">1.0</parameter>
</Normal>
</prior>
<prior id="PopSizePrior.t:NADH3_pos2" name="distribution" x="@popSize.t:NADH3_pos2">
<Normal id="Normal.93" name="distr">
<parameter id="RealParameter.1667" spec="parameter.RealParameter" estimate="false" name="mean">2.3</parameter>
<parameter id="RealParameter.1668" spec="parameter.RealParameter" estimate="false" name="sigma">1.0</parameter>
</Normal>
</prior>
<prior id="PopSizePrior.t:NADH3_pos3" name="distribution" x="@popSize.t:NADH3_pos3">
<Normal id="Normal.94" name="distr">
<parameter id="RealParameter.1669" spec="parameter.RealParameter" estimate="false" name="mean">2.3</parameter>
<parameter id="RealParameter.1670" spec="parameter.RealParameter" estimate="false" name="sigma">1.0</parameter>
</Normal>
</prior>
<prior id="PopSizePrior.t:NADH4L_pos1" name="distribution" x="@popSize.t:NADH4L_pos1">
<Normal id="Normal.95" name="distr">
<parameter id="RealParameter.1671" spec="parameter.RealParameter" estimate="false" name="mean">2.3</parameter>
<parameter id="RealParameter.1672" spec="parameter.RealParameter" estimate="false" name="sigma">1.0</parameter>
</Normal>
</prior>
<prior id="PopSizePrior.t:NADH4L_pos2" name="distribution" x="@popSize.t:NADH4L_pos2">
<Normal id="Normal.96" name="distr">
<parameter id="RealParameter.1673" spec="parameter.RealParameter" estimate="false" name="mean">2.3</parameter>
<parameter id="RealParameter.1674" spec="parameter.RealParameter" estimate="false" name="sigma">1.0</parameter>
</Normal>
</prior>
<prior id="PopSizePrior.t:NADH4L_pos3" name="distribution" x="@popSize.t:NADH4L_pos3">
<Normal id="Normal.97" name="distr">
<parameter id="RealParameter.1675" spec="parameter.RealParameter" estimate="false" name="mean">2.3</parameter>
<parameter id="RealParameter.1676" spec="parameter.RealParameter" estimate="false" name="sigma">1.0</parameter>
</Normal>
</prior>
<prior id="PopSizePrior.t:NADH4_pos1" name="distribution" x="@popSize.t:NADH4_pos1">
<OneOnX id="OneOnX.62" name="distr"/>
</prior>
<prior id="PopSizePrior.t:NADH4_pos2" name="distribution" x="@popSize.t:NADH4_pos2">
<OneOnX id="OneOnX.63" name="distr"/>
```

</prior>
<prior id="PopSizePrior.t:NADH4_pos3" name="distribution" x="@popSize.t:NADH4_pos3">
<OneOnX id="OneOnX.64" name="distr"/>
</prior>
<prior id="PopSizePrior.t:NADH5_pos1" name="distribution" x="@popSize.t:NADH5_pos1">
<OneOnX id="OneOnX.65" name="distr"/>
</prior>
<prior id="PopSizePrior.t:NADH5_pos2" name="distribution" x="@popSize.t:NADH5_pos2">
<OneOnX id="OneOnX.66" name="distr"/>
</prior>
<prior id="PopSizePrior.t:NADH5_pos3" name="distribution" x="@popSize.t:NADH5_pos3">
<OneOnX id="OneOnX.67" name="distr"/>
</prior>
<prior id="PopSizePrior.t:NADH6_pos1" name="distribution" x="@popSize.t:NADH6_pos1">
<OneOnX id="OneOnX.68" name="distr"/>
</prior>
<prior id="PopSizePrior.t:NADH6_pos2" name="distribution" x="@popSize.t:NADH6_pos2">
<OneOnX id="OneOnX.69" name="distr"/>
</prior>
<prior id="PopSizePrior.t:NADH6_pos3" name="distribution" x="@popSize.t:NADH6_pos3">
<OneOnX id="OneOnX.70" name="distr"/>
</prior>
<prior id="PopSizePrior.t:atp6_pos1" name="distribution" x="@popSize.t:atp6_pos1">
<OneOnX id="OneOnX.71" name="distr"/>
</prior>
<prior id="PopSizePrior.t:atp6_pos2" name="distribution" x="@popSize.t:atp6_pos2">
<OneOnX id="OneOnX.72" name="distr"/>
</prior>
<prior id="PopSizePrior.t:atp6_pos3" name="distribution" x="@popSize.t:atp6_pos3">
<OneOnX id="OneOnX.73" name="distr"/>
</prior>
<prior id="PropInvariantPrior.s:COX1_pos1" name="distribution"
x="@proportionInvariant.s:COX1_pos1">
<Uniform id="Uniform.28" name="distr"/>
</prior>
<prior id="PropInvariantPrior.s:COX1_pos2" name="distribution"
x="@proportionInvariant.s:COX1_pos2">
<Uniform id="Uniform.37" name="distr"/>
</prior>
<prior id="PropInvariantPrior.s:COX1_pos3" name="distribution"
x="@proportionInvariant.s:COX1_pos3">
<Uniform id="Uniform.47" name="distr"/>
</prior>
<prior id="PropInvariantPrior.s:COX2_pos1" name="distribution"
x="@proportionInvariant.s:COX2_pos1">
<Uniform id="Uniform.56" name="distr"/>
</prior>
<prior id="PropInvariantPrior.s:COX2_pos2" name="distribution"
x="@proportionInvariant.s:COX2_pos2">
<Uniform id="Uniform.65" name="distr"/>
</prior>
<prior id="PropInvariantPrior.s:COX2_pos3" name="distribution"
x="@proportionInvariant.s:COX2_pos3">
<Uniform id="Uniform.75" name="distr"/>
</prior>
<prior id="PropInvariantPrior.s:COX3_pos1" name="distribution"
x="@proportionInvariant.s:COX3_pos1">
<Uniform id="Uniform.84" name="distr"/>
</prior>
<prior id="PropInvariantPrior.s:COX3_pos2" name="distribution"
x="@proportionInvariant.s:COX3_pos2">
<Uniform id="Uniform.93" name="distr"/>
</prior>

```
<prior id="PropInvariantPrior.s:COX3_pos3" name="distribution"
x="@proportionInvariant.s:COX3_pos3">
    <Uniform id="Uniform.102" name="distr"/>
</prior>
<prior id="PropInvariantPrior.s:CYTB_pos1" name="distribution"
x="@proportionInvariant.s:CYTB_pos1">
    <Uniform id="Uniform.114" name="distr"/>
</prior>
<prior id="PropInvariantPrior.s:CYTB_pos2" name="distribution"
x="@proportionInvariant.s:CYTB_pos2">
    <Uniform id="Uniform.154" name="distr"/>
</prior>
<prior id="PropInvariantPrior.s:CYTB_pos3" name="distribution"
x="@proportionInvariant.s:CYTB_pos3">
    <Uniform id="Uniform.163" name="distr"/>
</prior>
<prior id="PropInvariantPrior.s:Mytilidae27sq12PCGs" name="distribution"
x="@proportionInvariant.s:Mytilidae27sq12PCGs">
    <Uniform id="Uniform.362" name="distr"/>
</prior>
<prior id="PropInvariantPrior.s:NADH1_pos1" name="distribution"
x="@proportionInvariant.s:NADH1_pos1">
    <Uniform id="Uniform.172" name="distr"/>
</prior>
<prior id="PropInvariantPrior.s:NADH1_pos2" name="distribution"
x="@proportionInvariant.s:NADH1_pos2">
    <Uniform id="Uniform.181" name="distr"/>
</prior>
<prior id="PropInvariantPrior.s:NADH1_pos3" name="distribution"
x="@proportionInvariant.s:NADH1_pos3">
    <Uniform id="Uniform.190" name="distr"/>
</prior>
<prior id="PropInvariantPrior.s:NADH2_pos1" name="distribution"
x="@proportionInvariant.s:NADH2_pos1">
    <Uniform id="Uniform.199" name="distr"/>
</prior>
<prior id="PropInvariantPrior.s:NADH2_pos2" name="distribution"
x="@proportionInvariant.s:NADH2_pos2">
    <Uniform id="Uniform.208" name="distr"/>
</prior>
<prior id="PropInvariantPrior.s:NADH2_pos3" name="distribution"
x="@proportionInvariant.s:NADH2_pos3">
    <Uniform id="Uniform.217" name="distr"/>
</prior>
<prior id="PropInvariantPrior.s:NADH3_pos1" name="distribution"
x="@proportionInvariant.s:NADH3_pos1">
    <Uniform id="Uniform.226" name="distr"/>
</prior>
<prior id="PropInvariantPrior.s:NADH3_pos2" name="distribution"
x="@proportionInvariant.s:NADH3_pos2">
    <Uniform id="Uniform.235" name="distr"/>
</prior>
<prior id="PropInvariantPrior.s:NADH3_pos3" name="distribution"
x="@proportionInvariant.s:NADH3_pos3">
    <Uniform id="Uniform.244" name="distr"/>
</prior>
<prior id="PropInvariantPrior.s:NADH4L_pos1" name="distribution"
x="@proportionInvariant.s:NADH4L_pos1">
    <Uniform id="Uniform.280" name="distr"/>
</prior>
<prior id="PropInvariantPrior.s:NADH4L_pos2" name="distribution"
x="@proportionInvariant.s:NADH4L_pos2">
    <Uniform id="Uniform.289" name="distr"/>
```

```
            </prior>
            <prior id="PropInvariantPrior.s:NADH4L_pos3" name="distribution"
x="@proportionInvariant.s:NADH4L_pos3">
                <Uniform id="Uniform.298" name="distr"/>
            </prior>
            <prior id="PropInvariantPrior.s:NADH4_pos1" name="distribution"
x="@proportionInvariant.s:NADH4_pos1">
                <Uniform id="Uniform.253" name="distr"/>
            </prior>
            <prior id="PropInvariantPrior.s:NADH4_pos2" name="distribution"
x="@proportionInvariant.s:NADH4_pos2">
                <Uniform id="Uniform.262" name="distr"/>
            </prior>
            <prior id="PropInvariantPrior.s:NADH4_pos3" name="distribution"
x="@proportionInvariant.s:NADH4_pos3">
                <Uniform id="Uniform.271" name="distr"/>
            </prior>
            <prior id="PropInvariantPrior.s:NADH5_pos1" name="distribution"
x="@proportionInvariant.s:NADH5_pos1">
                <Uniform id="Uniform.307" name="distr"/>
            </prior>
            <prior id="PropInvariantPrior.s:NADH5_pos2" name="distribution"
x="@proportionInvariant.s:NADH5_pos2">
                <Uniform id="Uniform.316" name="distr"/>
            </prior>
            <prior id="PropInvariantPrior.s:NADH5_pos3" name="distribution"
x="@proportionInvariant.s:NADH5_pos3">
                <Uniform id="Uniform.325" name="distr"/>
            </prior>
            <prior id="PropInvariantPrior.s:NADH6_pos1" name="distribution"
x="@proportionInvariant.s:NADH6_pos1">
                <Uniform id="Uniform.334" name="distr"/>
            </prior>
            <prior id="PropInvariantPrior.s:NADH6_pos2" name="distribution"
x="@proportionInvariant.s:NADH6_pos2">
                <Uniform id="Uniform.343" name="distr"/>
            </prior>
            <prior id="PropInvariantPrior.s:NADH6_pos3" name="distribution"
x="@proportionInvariant.s:NADH6_pos3">
                <Uniform id="Uniform.352" name="distr"/>
            </prior>
            <prior id="PropInvariantPrior.s:atp6_pos1" name="distribution" x="@proportionInvariant.s:atp6_pos1">
                <Uniform id="Uniform.2" name="distr"/>
            </prior>
            <prior id="PropInvariantPrior.s:atp6_pos2" name="distribution" x="@proportionInvariant.s:atp6_pos2">
                <Uniform id="Uniform.5" name="distr"/>
            </prior>
            <prior id="PropInvariantPrior.s:atp6_pos3" name="distribution" x="@proportionInvariant.s:atp6_pos3">
                <Uniform id="Uniform.19" name="distr"/>
            </prior>
            <prior id="RateACPrior.s:COX1_pos1" name="distribution" x="@rateAC.s:COX1_pos1">
                <Gamma id="Gamma.24" name="distr">
                    <parameter id="RealParameter.85" spec="parameter.RealParameter" estimate="false"
name="alpha">0.05</parameter>
                    <parameter id="RealParameter.86" spec="parameter.RealParameter" estimate="false"
name="beta">10.0</parameter>
                </Gamma>
            </prior>
            <prior id="RateACPrior.s:COX1_pos3" name="distribution" x="@rateAC.s:COX1_pos3">
                <Gamma id="Gamma.30" name="distr">
                    <parameter id="RealParameter.97" spec="parameter.RealParameter" estimate="false"
name="alpha">0.05</parameter>
```

<parameter id="RealParameter.98" spec="parameter.RealParameter" estimate="false"
name="beta">10.0</parameter>
</Gamma>
</prior>
<prior id="RateACPrior.s:COX2_pos1" name="distribution" x="@rateAC.s:COX2_pos1">
<Gamma id="Gamma.36" name="distr">
<parameter id="RealParameter.109" spec="parameter.RealParameter" estimate="false"
name="alpha">0.05</parameter>
<parameter id="RealParameter.110" spec="parameter.RealParameter" estimate="false"
name="beta">10.0</parameter>
</Gamma>
</prior>
<prior id="RateACPrior.s:COX2_pos2" name="distribution" x="@rateAC.s:COX2_pos2">
<Gamma id="Gamma.130" name="distr">
<parameter id="RealParameter.611" spec="parameter.RealParameter" estimate="false"
name="alpha">0.05</parameter>
<parameter id="RealParameter.612" spec="parameter.RealParameter" estimate="false"
name="beta">10.0</parameter>
</Gamma>
</prior>
<prior id="RateACPrior.s:COX2_pos3" name="distribution" x="@rateAC.s:COX2_pos3">
<Gamma id="Gamma.42" name="distr">
<parameter id="RealParameter.121" spec="parameter.RealParameter" estimate="false"
name="alpha">0.05</parameter>
<parameter id="RealParameter.122" spec="parameter.RealParameter" estimate="false"
name="beta">10.0</parameter>
</Gamma>
</prior>
<prior id="RateACPrior.s:COX3_pos1" name="distribution" x="@rateAC.s:COX3_pos1">
<Gamma id="Gamma.48" name="distr">
<parameter id="RealParameter.133" spec="parameter.RealParameter" estimate="false"
name="alpha">0.05</parameter>
<parameter id="RealParameter.134" spec="parameter.RealParameter" estimate="false"
name="beta">10.0</parameter>
</Gamma>
</prior>
<prior id="RateACPrior.s:COX3_pos2" name="distribution" x="@rateAC.s:COX3_pos2">
<Gamma id="Gamma.54" name="distr">
<parameter id="RealParameter.145" spec="parameter.RealParameter" estimate="false"
name="alpha">0.05</parameter>
<parameter id="RealParameter.146" spec="parameter.RealParameter" estimate="false"
name="beta">10.0</parameter>
</Gamma>
</prior>
<prior id="RateACPrior.s:COX3_pos3" name="distribution" x="@rateAC.s:COX3_pos3">
<Gamma id="Gamma.60" name="distr">
<parameter id="RealParameter.157" spec="parameter.RealParameter" estimate="false"
name="alpha">0.05</parameter>
<parameter id="RealParameter.158" spec="parameter.RealParameter" estimate="false"
name="beta">10.0</parameter>
</Gamma>
</prior>
<prior id="RateACPrior.s:CYTB_pos1" name="distribution" x="@rateAC.s:CYTB_pos1">
<Gamma id="Gamma.66" name="distr">
<parameter id="RealParameter.169" spec="parameter.RealParameter" estimate="false"
name="alpha">0.05</parameter>
<parameter id="RealParameter.170" spec="parameter.RealParameter" estimate="false"
name="beta">10.0</parameter>
</Gamma>
</prior>
<prior id="RateACPrior.s:CYTB_pos2" name="distribution" x="@rateAC.s:CYTB_pos2">
<Gamma id="Gamma.72" name="distr">

```xml
<parameter id="RealParameter.181" spec="parameter.RealParameter" estimate="false" name="alpha">0.05</parameter>
<parameter id="RealParameter.182" spec="parameter.RealParameter" estimate="false" name="beta">10.0</parameter>
</Gamma>
</prior>
<prior id="RateACPrior.s:CYTB_pos3" name="distribution" x="@rateAC.s:CYTB_pos3">
<Gamma id="Gamma.78" name="distr">
<parameter id="RealParameter.193" spec="parameter.RealParameter" estimate="false" name="alpha">0.05</parameter>
<parameter id="RealParameter.194" spec="parameter.RealParameter" estimate="false" name="beta">10.0</parameter>
</Gamma>
</prior>
<prior id="RateACPrior.s:Mytilidae27sq12PCGs" name="distribution" x="@rateAC.s:Mytilidae27sq12PCGs">
<Gamma id="Gamma.288" name="distr">
<parameter id="RealParameter.1520" spec="parameter.RealParameter" estimate="false" name="alpha">0.05</parameter>
<parameter id="RealParameter.1521" spec="parameter.RealParameter" estimate="false" name="beta">10.0</parameter>
</Gamma>
</prior>
<prior id="RateACPrior.s:NADH1_pos1" name="distribution" x="@rateAC.s:NADH1_pos1">
<Gamma id="Gamma.84" name="distr">
<parameter id="RealParameter.205" spec="parameter.RealParameter" estimate="false" name="alpha">0.05</parameter>
<parameter id="RealParameter.206" spec="parameter.RealParameter" estimate="false" name="beta">10.0</parameter>
</Gamma>
</prior>
<prior id="RateACPrior.s:NADH1_pos2" name="distribution" x="@rateAC.s:NADH1_pos2">
<Gamma id="Gamma.90" name="distr">
<parameter id="RealParameter.217" spec="parameter.RealParameter" estimate="false" name="alpha">0.05</parameter>
<parameter id="RealParameter.218" spec="parameter.RealParameter" estimate="false" name="beta">10.0</parameter>
</Gamma>
</prior>
<prior id="RateACPrior.s:NADH1_pos3" name="distribution" x="@rateAC.s:NADH1_pos3">
<Gamma id="Gamma.96" name="distr">
<parameter id="RealParameter.229" spec="parameter.RealParameter" estimate="false" name="alpha">0.05</parameter>
<parameter id="RealParameter.230" spec="parameter.RealParameter" estimate="false" name="beta">10.0</parameter>
</Gamma>
</prior>
<prior id="RateACPrior.s:NADH2_pos1" name="distribution" x="@rateAC.s:NADH2_pos1">
<Gamma id="Gamma.102" name="distr">
<parameter id="RealParameter.241" spec="parameter.RealParameter" estimate="false" name="alpha">0.05</parameter>
<parameter id="RealParameter.242" spec="parameter.RealParameter" estimate="false" name="beta">10.0</parameter>
</Gamma>
</prior>
<prior id="RateACPrior.s:NADH2_pos2" name="distribution" x="@rateAC.s:NADH2_pos2">
<Gamma id="Gamma.108" name="distr">
<parameter id="RealParameter.253" spec="parameter.RealParameter" estimate="false" name="alpha">0.05</parameter>
<parameter id="RealParameter.254" spec="parameter.RealParameter" estimate="false" name="beta">10.0</parameter>
</Gamma>
</prior>
```

```
<prior id="RateACPrior.s:NADH2_pos3" name="distribution" x="@rateAC.s:NADH2_pos3">
    <Gamma id="Gamma.114" name="distr">
        <parameter id="RealParameter.265" spec="parameter.RealParameter" estimate="false"
name="alpha">0.05</parameter>
        <parameter id="RealParameter.266" spec="parameter.RealParameter" estimate="false"
name="beta">10.0</parameter>
    </Gamma>
</prior>
<prior id="RateACPrior.s:NADH3_pos1" name="distribution" x="@rateAC.s:NADH3_pos1">
    <Gamma id="Gamma.120" name="distr">
        <parameter id="RealParameter.277" spec="parameter.RealParameter" estimate="false"
name="alpha">0.05</parameter>
        <parameter id="RealParameter.278" spec="parameter.RealParameter" estimate="false"
name="beta">10.0</parameter>
    </Gamma>
</prior>
<prior id="RateACPrior.s:NADH3_pos2" name="distribution" x="@rateAC.s:NADH3_pos2">
    <Gamma id="Gamma.126" name="distr">
        <parameter id="RealParameter.289" spec="parameter.RealParameter" estimate="false"
name="alpha">0.05</parameter>
        <parameter id="RealParameter.290" spec="parameter.RealParameter" estimate="false"
name="beta">10.0</parameter>
    </Gamma>
</prior>
<prior id="RateACPrior.s:NADH3_pos3" name="distribution" x="@rateAC.s:NADH3_pos3">
    <Gamma id="Gamma.132" name="distr">
        <parameter id="RealParameter.301" spec="parameter.RealParameter" estimate="false"
name="alpha">0.05</parameter>
        <parameter id="RealParameter.302" spec="parameter.RealParameter" estimate="false"
name="beta">10.0</parameter>
    </Gamma>
</prior>
<prior id="RateACPrior.s:NADH4L_pos1" name="distribution" x="@rateAC.s:NADH4L_pos1">
    <Gamma id="Gamma.156" name="distr">
        <parameter id="RealParameter.349" spec="parameter.RealParameter" estimate="false"
name="alpha">0.05</parameter>
        <parameter id="RealParameter.350" spec="parameter.RealParameter" estimate="false"
name="beta">10.0</parameter>
    </Gamma>
</prior>
<prior id="RateACPrior.s:NADH4L_pos2" name="distribution" x="@rateAC.s:NADH4L_pos2">
    <Gamma id="Gamma.162" name="distr">
        <parameter id="RealParameter.361" spec="parameter.RealParameter" estimate="false"
name="alpha">0.05</parameter>
        <parameter id="RealParameter.362" spec="parameter.RealParameter" estimate="false"
name="beta">10.0</parameter>
    </Gamma>
</prior>
<prior id="RateACPrior.s:NADH4L_pos3" name="distribution" x="@rateAC.s:NADH4L_pos3">
    <Gamma id="Gamma.168" name="distr">
        <parameter id="RealParameter.373" spec="parameter.RealParameter" estimate="false"
name="alpha">0.05</parameter>
        <parameter id="RealParameter.374" spec="parameter.RealParameter" estimate="false"
name="beta">10.0</parameter>
    </Gamma>
</prior>
<prior id="RateACPrior.s:NADH4_pos1" name="distribution" x="@rateAC.s:NADH4_pos1">
    <Gamma id="Gamma.138" name="distr">
        <parameter id="RealParameter.313" spec="parameter.RealParameter" estimate="false"
name="alpha">0.05</parameter>
        <parameter id="RealParameter.314" spec="parameter.RealParameter" estimate="false"
name="beta">10.0</parameter>
    </Gamma>
```

```xml
</prior>
<prior id="RateACPrior.s:NADH4_pos2" name="distribution" x="@rateAC.s:NADH4_pos2">
<Gamma id="Gamma.144" name="distr">
<parameter id="RealParameter.325" spec="parameter.RealParameter" estimate="false" name="alpha">0.05</parameter>
<parameter id="RealParameter.326" spec="parameter.RealParameter" estimate="false" name="beta">10.0</parameter>
</Gamma>
</prior>
<prior id="RateACPrior.s:NADH4_pos3" name="distribution" x="@rateAC.s:NADH4_pos3">
<Gamma id="Gamma.150" name="distr">
<parameter id="RealParameter.337" spec="parameter.RealParameter" estimate="false" name="alpha">0.05</parameter>
<parameter id="RealParameter.338" spec="parameter.RealParameter" estimate="false" name="beta">10.0</parameter>
</Gamma>
</prior>
<prior id="RateACPrior.s:NADH5_pos1" name="distribution" x="@rateAC.s:NADH5_pos1">
<Gamma id="Gamma.174" name="distr">
<parameter id="RealParameter.385" spec="parameter.RealParameter" estimate="false" name="alpha">0.05</parameter>
<parameter id="RealParameter.386" spec="parameter.RealParameter" estimate="false" name="beta">10.0</parameter>
</Gamma>
</prior>
<prior id="RateACPrior.s:NADH5_pos2" name="distribution" x="@rateAC.s:NADH5_pos2">
<Gamma id="Gamma.180" name="distr">
<parameter id="RealParameter.397" spec="parameter.RealParameter" estimate="false" name="alpha">0.05</parameter>
<parameter id="RealParameter.398" spec="parameter.RealParameter" estimate="false" name="beta">10.0</parameter>
</Gamma>
</prior>
<prior id="RateACPrior.s:NADH5_pos3" name="distribution" x="@rateAC.s:NADH5_pos3">
<Gamma id="Gamma.186" name="distr">
<parameter id="RealParameter.409" spec="parameter.RealParameter" estimate="false" name="alpha">0.05</parameter>
<parameter id="RealParameter.410" spec="parameter.RealParameter" estimate="false" name="beta">10.0</parameter>
</Gamma>
</prior>
<prior id="RateACPrior.s:NADH6_pos1" name="distribution" x="@rateAC.s:NADH6_pos1">
<Gamma id="Gamma.192" name="distr">
<parameter id="RealParameter.421" spec="parameter.RealParameter" estimate="false" name="alpha">0.05</parameter>
<parameter id="RealParameter.422" spec="parameter.RealParameter" estimate="false" name="beta">10.0</parameter>
</Gamma>
</prior>
<prior id="RateACPrior.s:NADH6_pos2" name="distribution" x="@rateAC.s:NADH6_pos2">
<Gamma id="Gamma.198" name="distr">
<parameter id="RealParameter.433" spec="parameter.RealParameter" estimate="false" name="alpha">0.05</parameter>
<parameter id="RealParameter.434" spec="parameter.RealParameter" estimate="false" name="beta">10.0</parameter>
</Gamma>
</prior>
<prior id="RateACPrior.s:NADH6_pos3" name="distribution" x="@rateAC.s:NADH6_pos3">
<Gamma id="Gamma.204" name="distr">
<parameter id="RealParameter.445" spec="parameter.RealParameter" estimate="false" name="alpha">0.05</parameter>
<parameter id="RealParameter.446" spec="parameter.RealParameter" estimate="false" name="beta">10.0</parameter>
```

```
            </Gamma>
        </prior>
        <prior id="RateACPrior.s:atp6_pos1" name="distribution" x="@rateAC.s:atp6_pos1">
            <Gamma id="Gamma.0" name="distr">
                <parameter id="RealParameter.37" spec="parameter.RealParameter" estimate="false"
name="alpha">0.05</parameter>
                <parameter id="RealParameter.38" spec="parameter.RealParameter" estimate="false"
name="beta">10.0</parameter>
            </Gamma>
        </prior>
        <prior id="RateACPrior.s:atp6_pos2" name="distribution" x="@rateAC.s:atp6_pos2">
            <Gamma id="Gamma.6" name="distr">
                <parameter id="RealParameter.49" spec="parameter.RealParameter" estimate="false"
name="alpha">0.05</parameter>
                <parameter id="RealParameter.50" spec="parameter.RealParameter" estimate="false"
name="beta">10.0</parameter>
            </Gamma>
        </prior>
        <prior id="RateACPrior.s:atp6_pos3" name="distribution" x="@rateAC.s:atp6_pos3">
            <Gamma id="Gamma.12" name="distr">
                <parameter id="RealParameter.61" spec="parameter.RealParameter" estimate="false"
name="alpha">0.05</parameter>
                <parameter id="RealParameter.62" spec="parameter.RealParameter" estimate="false"
name="beta">10.0</parameter>
            </Gamma>
        </prior>
        <prior id="RateAGPrior.s:COX1_pos1" name="distribution" x="@rateAG.s:COX1_pos1">
            <Gamma id="Gamma.25" name="distr">
                <parameter id="RealParameter.87" spec="parameter.RealParameter" estimate="false"
name="alpha">0.05</parameter>
                <parameter id="RealParameter.88" spec="parameter.RealParameter" estimate="false"
name="beta">20.0</parameter>
            </Gamma>
        </prior>
        <prior id="RateAGPrior.s:COX1_pos3" name="distribution" x="@rateAG.s:COX1_pos3">
            <Gamma id="Gamma.31" name="distr">
                <parameter id="RealParameter.99" spec="parameter.RealParameter" estimate="false"
name="alpha">0.05</parameter>
                <parameter id="RealParameter.100" spec="parameter.RealParameter" estimate="false"
name="beta">20.0</parameter>
            </Gamma>
        </prior>
        <prior id="RateAGPrior.s:COX2_pos1" name="distribution" x="@rateAG.s:COX2_pos1">
            <Gamma id="Gamma.37" name="distr">
                <parameter id="RealParameter.111" spec="parameter.RealParameter" estimate="false"
name="alpha">0.05</parameter>
                <parameter id="RealParameter.112" spec="parameter.RealParameter" estimate="false"
name="beta">20.0</parameter>
            </Gamma>
        </prior>
        <prior id="RateAGPrior.s:COX2_pos2" name="distribution" x="@rateAG.s:COX2_pos2">
            <Gamma id="Gamma.136" name="distr">
                <parameter id="RealParameter.613" spec="parameter.RealParameter" estimate="false"
name="alpha">0.05</parameter>
                <parameter id="RealParameter.614" spec="parameter.RealParameter" estimate="false"
name="beta">20.0</parameter>
            </Gamma>
        </prior>
        <prior id="RateAGPrior.s:COX2_pos3" name="distribution" x="@rateAG.s:COX2_pos3">
            <Gamma id="Gamma.43" name="distr">
                <parameter id="RealParameter.123" spec="parameter.RealParameter" estimate="false"
name="alpha">0.05</parameter>
```

```xml
<parameter id="RealParameter.124" spec="parameter.RealParameter" estimate="false" name="beta">20.0</parameter>
</Gamma>
</prior>
<prior id="RateAGPrior.s:COX3_pos1" name="distribution" x="@rateAG.s:COX3_pos1">
<Gamma id="Gamma.49" name="distr">
<parameter id="RealParameter.135" spec="parameter.RealParameter" estimate="false" name="alpha">0.05</parameter>
<parameter id="RealParameter.136" spec="parameter.RealParameter" estimate="false" name="beta">20.0</parameter>
</Gamma>
</prior>
<prior id="RateAGPrior.s:COX3_pos2" name="distribution" x="@rateAG.s:COX3_pos2">
<Gamma id="Gamma.55" name="distr">
<parameter id="RealParameter.147" spec="parameter.RealParameter" estimate="false" name="alpha">0.05</parameter>
<parameter id="RealParameter.148" spec="parameter.RealParameter" estimate="false" name="beta">20.0</parameter>
</Gamma>
</prior>
<prior id="RateAGPrior.s:COX3_pos3" name="distribution" x="@rateAG.s:COX3_pos3">
<Gamma id="Gamma.61" name="distr">
<parameter id="RealParameter.159" spec="parameter.RealParameter" estimate="false" name="alpha">0.05</parameter>
<parameter id="RealParameter.160" spec="parameter.RealParameter" estimate="false" name="beta">20.0</parameter>
</Gamma>
</prior>
<prior id="RateAGPrior.s:CYTB_pos1" name="distribution" x="@rateAG.s:CYTB_pos1">
<Gamma id="Gamma.67" name="distr">
<parameter id="RealParameter.171" spec="parameter.RealParameter" estimate="false" name="alpha">0.05</parameter>
<parameter id="RealParameter.172" spec="parameter.RealParameter" estimate="false" name="beta">20.0</parameter>
</Gamma>
</prior>
<prior id="RateAGPrior.s:CYTB_pos2" name="distribution" x="@rateAG.s:CYTB_pos2">
<Gamma id="Gamma.73" name="distr">
<parameter id="RealParameter.183" spec="parameter.RealParameter" estimate="false" name="alpha">0.05</parameter>
<parameter id="RealParameter.184" spec="parameter.RealParameter" estimate="false" name="beta">20.0</parameter>
</Gamma>
</prior>
<prior id="RateAGPrior.s:CYTB_pos3" name="distribution" x="@rateAG.s:CYTB_pos3">
<Gamma id="Gamma.79" name="distr">
<parameter id="RealParameter.195" spec="parameter.RealParameter" estimate="false" name="alpha">0.05</parameter>
<parameter id="RealParameter.196" spec="parameter.RealParameter" estimate="false" name="beta">20.0</parameter>
</Gamma>
</prior>
<prior id="RateAGPrior.s:Mytilidae27sq12PCGs" name="distribution" x="@rateAG.s:Mytilidae27sq12PCGs">
<Gamma id="Gamma.289" name="distr">
<parameter id="RealParameter.1522" spec="parameter.RealParameter" estimate="false" name="alpha">0.05</parameter>
<parameter id="RealParameter.1523" spec="parameter.RealParameter" estimate="false" name="beta">20.0</parameter>
</Gamma>
</prior>
<prior id="RateAGPrior.s:NADH1_pos1" name="distribution" x="@rateAG.s:NADH1_pos1">
<Gamma id="Gamma.85" name="distr">
```

```xml
<parameter id="RealParameter.207" spec="parameter.RealParameter" estimate="false" name="alpha">0.05</parameter>
<parameter id="RealParameter.208" spec="parameter.RealParameter" estimate="false" name="beta">20.0</parameter>
</Gamma>
</prior>
<prior id="RateAGPrior.s:NADH1_pos2" name="distribution" x="@rateAG.s:NADH1_pos2">
<Gamma id="Gamma.91" name="distr">
<parameter id="RealParameter.219" spec="parameter.RealParameter" estimate="false" name="alpha">0.05</parameter>
<parameter id="RealParameter.220" spec="parameter.RealParameter" estimate="false" name="beta">20.0</parameter>
</Gamma>
</prior>
<prior id="RateAGPrior.s:NADH1_pos3" name="distribution" x="@rateAG.s:NADH1_pos3">
<Gamma id="Gamma.97" name="distr">
<parameter id="RealParameter.231" spec="parameter.RealParameter" estimate="false" name="alpha">0.05</parameter>
<parameter id="RealParameter.232" spec="parameter.RealParameter" estimate="false" name="beta">20.0</parameter>
</Gamma>
</prior>
<prior id="RateAGPrior.s:NADH2_pos1" name="distribution" x="@rateAG.s:NADH2_pos1">
<Gamma id="Gamma.103" name="distr">
<parameter id="RealParameter.243" spec="parameter.RealParameter" estimate="false" name="alpha">0.05</parameter>
<parameter id="RealParameter.244" spec="parameter.RealParameter" estimate="false" name="beta">20.0</parameter>
</Gamma>
</prior>
<prior id="RateAGPrior.s:NADH2_pos2" name="distribution" x="@rateAG.s:NADH2_pos2">
<Gamma id="Gamma.109" name="distr">
<parameter id="RealParameter.255" spec="parameter.RealParameter" estimate="false" name="alpha">0.05</parameter>
<parameter id="RealParameter.256" spec="parameter.RealParameter" estimate="false" name="beta">20.0</parameter>
</Gamma>
</prior>
<prior id="RateAGPrior.s:NADH2_pos3" name="distribution" x="@rateAG.s:NADH2_pos3">
<Gamma id="Gamma.115" name="distr">
<parameter id="RealParameter.267" spec="parameter.RealParameter" estimate="false" name="alpha">0.05</parameter>
<parameter id="RealParameter.268" spec="parameter.RealParameter" estimate="false" name="beta">20.0</parameter>
</Gamma>
</prior>
<prior id="RateAGPrior.s:NADH3_pos1" name="distribution" x="@rateAG.s:NADH3_pos1">
<Gamma id="Gamma.121" name="distr">
<parameter id="RealParameter.279" spec="parameter.RealParameter" estimate="false" name="alpha">0.05</parameter>
<parameter id="RealParameter.280" spec="parameter.RealParameter" estimate="false" name="beta">20.0</parameter>
</Gamma>
</prior>
<prior id="RateAGPrior.s:NADH3_pos2" name="distribution" x="@rateAG.s:NADH3_pos2">
<Gamma id="Gamma.127" name="distr">
<parameter id="RealParameter.291" spec="parameter.RealParameter" estimate="false" name="alpha">0.05</parameter>
<parameter id="RealParameter.292" spec="parameter.RealParameter" estimate="false" name="beta">20.0</parameter>
</Gamma>
</prior>
<prior id="RateAGPrior.s:NADH3_pos3" name="distribution" x="@rateAG.s:NADH3_pos3">
```

```xml
<Gamma id="Gamma.133" name="distr">
<parameter id="RealParameter.303" spec="parameter.RealParameter" estimate="false" name="alpha">0.05</parameter>
<parameter id="RealParameter.304" spec="parameter.RealParameter" estimate="false" name="beta">20.0</parameter>
</Gamma>
</prior>
<prior id="RateAGPrior.s:NADH4L_pos1" name="distribution" x="@rateAG.s:NADH4L_pos1">
<Gamma id="Gamma.157" name="distr">
<parameter id="RealParameter.351" spec="parameter.RealParameter" estimate="false" name="alpha">0.05</parameter>
<parameter id="RealParameter.352" spec="parameter.RealParameter" estimate="false" name="beta">20.0</parameter>
</Gamma>
</prior>
<prior id="RateAGPrior.s:NADH4L_pos2" name="distribution" x="@rateAG.s:NADH4L_pos2">
<Gamma id="Gamma.163" name="distr">
<parameter id="RealParameter.363" spec="parameter.RealParameter" estimate="false" name="alpha">0.05</parameter>
<parameter id="RealParameter.364" spec="parameter.RealParameter" estimate="false" name="beta">20.0</parameter>
</Gamma>
</prior>
<prior id="RateAGPrior.s:NADH4L_pos3" name="distribution" x="@rateAG.s:NADH4L_pos3">
<Gamma id="Gamma.169" name="distr">
<parameter id="RealParameter.375" spec="parameter.RealParameter" estimate="false" name="alpha">0.05</parameter>
<parameter id="RealParameter.376" spec="parameter.RealParameter" estimate="false" name="beta">20.0</parameter>
</Gamma>
</prior>
<prior id="RateAGPrior.s:NADH4_pos1" name="distribution" x="@rateAG.s:NADH4_pos1">
<Gamma id="Gamma.139" name="distr">
<parameter id="RealParameter.315" spec="parameter.RealParameter" estimate="false" name="alpha">0.05</parameter>
<parameter id="RealParameter.316" spec="parameter.RealParameter" estimate="false" name="beta">20.0</parameter>
</Gamma>
</prior>
<prior id="RateAGPrior.s:NADH4_pos2" name="distribution" x="@rateAG.s:NADH4_pos2">
<Gamma id="Gamma.145" name="distr">
<parameter id="RealParameter.327" spec="parameter.RealParameter" estimate="false" name="alpha">0.05</parameter>
<parameter id="RealParameter.328" spec="parameter.RealParameter" estimate="false" name="beta">20.0</parameter>
</Gamma>
</prior>
<prior id="RateAGPrior.s:NADH4_pos3" name="distribution" x="@rateAG.s:NADH4_pos3">
<Gamma id="Gamma.151" name="distr">
<parameter id="RealParameter.339" spec="parameter.RealParameter" estimate="false" name="alpha">0.05</parameter>
<parameter id="RealParameter.340" spec="parameter.RealParameter" estimate="false" name="beta">20.0</parameter>
</Gamma>
</prior>
<prior id="RateAGPrior.s:NADH5_pos1" name="distribution" x="@rateAG.s:NADH5_pos1">
<Gamma id="Gamma.175" name="distr">
<parameter id="RealParameter.387" spec="parameter.RealParameter" estimate="false" name="alpha">0.05</parameter>
<parameter id="RealParameter.388" spec="parameter.RealParameter" estimate="false" name="beta">20.0</parameter>
</Gamma>
</prior>
```

<prior id="RateAGPrior.s:NADH5_pos2" name="distribution" x="@rateAG.s:NADH5_pos2">
<Gamma id="Gamma.181" name="distr">
<parameter id="RealParameter.399" spec="parameter.RealParameter" estimate="false"
name="alpha">0.05</parameter>
<parameter id="RealParameter.400" spec="parameter.RealParameter" estimate="false"
name="beta">20.0</parameter>
</Gamma>
</prior>
<prior id="RateAGPrior.s:NADH5_pos3" name="distribution" x="@rateAG.s:NADH5_pos3">
<Gamma id="Gamma.187" name="distr">
<parameter id="RealParameter.411" spec="parameter.RealParameter" estimate="false"
name="alpha">0.05</parameter>
<parameter id="RealParameter.412" spec="parameter.RealParameter" estimate="false"
name="beta">20.0</parameter>
</Gamma>
</prior>
<prior id="RateAGPrior.s:NADH6_pos1" name="distribution" x="@rateAG.s:NADH6_pos1">
<Gamma id="Gamma.193" name="distr">
<parameter id="RealParameter.423" spec="parameter.RealParameter" estimate="false"
name="alpha">0.05</parameter>
<parameter id="RealParameter.424" spec="parameter.RealParameter" estimate="false"
name="beta">20.0</parameter>
</Gamma>
</prior>
<prior id="RateAGPrior.s:NADH6_pos2" name="distribution" x="@rateAG.s:NADH6_pos2">
<Gamma id="Gamma.199" name="distr">
<parameter id="RealParameter.435" spec="parameter.RealParameter" estimate="false"
name="alpha">0.05</parameter>
<parameter id="RealParameter.436" spec="parameter.RealParameter" estimate="false"
name="beta">20.0</parameter>
</Gamma>
</prior>
<prior id="RateAGPrior.s:NADH6_pos3" name="distribution" x="@rateAG.s:NADH6_pos3">
<Gamma id="Gamma.205" name="distr">
<parameter id="RealParameter.447" spec="parameter.RealParameter" estimate="false"
name="alpha">0.05</parameter>
<parameter id="RealParameter.448" spec="parameter.RealParameter" estimate="false"
name="beta">20.0</parameter>
</Gamma>
</prior>
<prior id="RateAGPrior.s:atp6_pos1" name="distribution" x="@rateAG.s:atp6_pos1">
<Gamma id="Gamma.1" name="distr">
<parameter id="RealParameter.39" spec="parameter.RealParameter" estimate="false"
name="alpha">0.05</parameter>
<parameter id="RealParameter.40" spec="parameter.RealParameter" estimate="false"
name="beta">20.0</parameter>
</Gamma>
</prior>
<prior id="RateAGPrior.s:atp6_pos2" name="distribution" x="@rateAG.s:atp6_pos2">
<Gamma id="Gamma.7" name="distr">
<parameter id="RealParameter.51" spec="parameter.RealParameter" estimate="false"
name="alpha">0.05</parameter>
<parameter id="RealParameter.52" spec="parameter.RealParameter" estimate="false"
name="beta">20.0</parameter>
</Gamma>
</prior>
<prior id="RateAGPrior.s:atp6_pos3" name="distribution" x="@rateAG.s:atp6_pos3">
<Gamma id="Gamma.13" name="distr">
<parameter id="RealParameter.63" spec="parameter.RealParameter" estimate="false"
name="alpha">0.05</parameter>
<parameter id="RealParameter.64" spec="parameter.RealParameter" estimate="false"
name="beta">20.0</parameter>
</Gamma>

</prior>
<prior id="RateATPrior.s:COX1_pos1" name="distribution" x="@rateAT.s:COX1_pos1">
<Gamma id="Gamma.26" name="distr">
<parameter id="RealParameter.89" spec="parameter.RealParameter" estimate="false"
name="alpha">0.05</parameter>
<parameter id="RealParameter.90" spec="parameter.RealParameter" estimate="false"
name="beta">10.0</parameter>
</Gamma>
</prior>
<prior id="RateATPrior.s:COX1_pos3" name="distribution" x="@rateAT.s:COX1_pos3">
<Gamma id="Gamma.32" name="distr">
<parameter id="RealParameter.101" spec="parameter.RealParameter" estimate="false"
name="alpha">0.05</parameter>
<parameter id="RealParameter.102" spec="parameter.RealParameter" estimate="false"
name="beta">10.0</parameter>
</Gamma>
</prior>
<prior id="RateATPrior.s:COX2_pos1" name="distribution" x="@rateAT.s:COX2_pos1">
<Gamma id="Gamma.38" name="distr">
<parameter id="RealParameter.113" spec="parameter.RealParameter" estimate="false"
name="alpha">0.05</parameter>
<parameter id="RealParameter.114" spec="parameter.RealParameter" estimate="false"
name="beta">10.0</parameter>
</Gamma>
</prior>
<prior id="RateATPrior.s:COX2_pos2" name="distribution" x="@rateAT.s:COX2_pos2">
<Gamma id="Gamma.142" name="distr">
<parameter id="RealParameter.615" spec="parameter.RealParameter" estimate="false"
name="alpha">0.05</parameter>
<parameter id="RealParameter.616" spec="parameter.RealParameter" estimate="false"
name="beta">10.0</parameter>
</Gamma>
</prior>
<prior id="RateATPrior.s:COX2_pos3" name="distribution" x="@rateAT.s:COX2_pos3">
<Gamma id="Gamma.44" name="distr">
<parameter id="RealParameter.125" spec="parameter.RealParameter" estimate="false"
name="alpha">0.05</parameter>
<parameter id="RealParameter.126" spec="parameter.RealParameter" estimate="false"
name="beta">10.0</parameter>
</Gamma>
</prior>
<prior id="RateATPrior.s:COX3_pos1" name="distribution" x="@rateAT.s:COX3_pos1">
<Gamma id="Gamma.50" name="distr">
<parameter id="RealParameter.137" spec="parameter.RealParameter" estimate="false"
name="alpha">0.05</parameter>
<parameter id="RealParameter.138" spec="parameter.RealParameter" estimate="false"
name="beta">10.0</parameter>
</Gamma>
</prior>
<prior id="RateATPrior.s:COX3_pos2" name="distribution" x="@rateAT.s:COX3_pos2">
<Gamma id="Gamma.56" name="distr">
<parameter id="RealParameter.149" spec="parameter.RealParameter" estimate="false"
name="alpha">0.05</parameter>
<parameter id="RealParameter.150" spec="parameter.RealParameter" estimate="false"
name="beta">10.0</parameter>
</Gamma>
</prior>
<prior id="RateATPrior.s:COX3_pos3" name="distribution" x="@rateAT.s:COX3_pos3">
<Gamma id="Gamma.62" name="distr">
<parameter id="RealParameter.161" spec="parameter.RealParameter" estimate="false"
name="alpha">0.05</parameter>
<parameter id="RealParameter.162" spec="parameter.RealParameter" estimate="false"
name="beta">10.0</parameter>

</Gamma>
</prior>
<prior id="RateATPrior.s:CYTB_pos1" name="distribution" x="@rateAT.s:CYTB_pos1">
<Gamma id="Gamma.68" name="distr">
<parameter id="RealParameter.173" spec="parameter.RealParameter" estimate="false" name="alpha">0.05</parameter>
<parameter id="RealParameter.174" spec="parameter.RealParameter" estimate="false" name="beta">10.0</parameter>
</Gamma>
</prior>
<prior id="RateATPrior.s:CYTB_pos2" name="distribution" x="@rateAT.s:CYTB_pos2">
<Gamma id="Gamma.74" name="distr">
<parameter id="RealParameter.185" spec="parameter.RealParameter" estimate="false" name="alpha">0.05</parameter>
<parameter id="RealParameter.186" spec="parameter.RealParameter" estimate="false" name="beta">10.0</parameter>
</Gamma>
</prior>
<prior id="RateATPrior.s:CYTB_pos3" name="distribution" x="@rateAT.s:CYTB_pos3">
<Gamma id="Gamma.80" name="distr">
<parameter id="RealParameter.197" spec="parameter.RealParameter" estimate="false" name="alpha">0.05</parameter>
<parameter id="RealParameter.198" spec="parameter.RealParameter" estimate="false" name="beta">10.0</parameter>
</Gamma>
</prior>
<prior id="RateATPrior.s:Mytilidae27sq12PCGs" name="distribution" x="@rateAT.s:Mytilidae27sq12PCGs">
<Gamma id="Gamma.290" name="distr">
<parameter id="RealParameter.1524" spec="parameter.RealParameter" estimate="false" name="alpha">0.05</parameter>
<parameter id="RealParameter.1525" spec="parameter.RealParameter" estimate="false" name="beta">10.0</parameter>
</Gamma>
</prior>
<prior id="RateATPrior.s:NADH1_pos1" name="distribution" x="@rateAT.s:NADH1_pos1">
<Gamma id="Gamma.86" name="distr">
<parameter id="RealParameter.209" spec="parameter.RealParameter" estimate="false" name="alpha">0.05</parameter>
<parameter id="RealParameter.210" spec="parameter.RealParameter" estimate="false" name="beta">10.0</parameter>
</Gamma>
</prior>
<prior id="RateATPrior.s:NADH1_pos2" name="distribution" x="@rateAT.s:NADH1_pos2">
<Gamma id="Gamma.92" name="distr">
<parameter id="RealParameter.221" spec="parameter.RealParameter" estimate="false" name="alpha">0.05</parameter>
<parameter id="RealParameter.222" spec="parameter.RealParameter" estimate="false" name="beta">10.0</parameter>
</Gamma>
</prior>
<prior id="RateATPrior.s:NADH1_pos3" name="distribution" x="@rateAT.s:NADH1_pos3">
<Gamma id="Gamma.98" name="distr">
<parameter id="RealParameter.233" spec="parameter.RealParameter" estimate="false" name="alpha">0.05</parameter>
<parameter id="RealParameter.234" spec="parameter.RealParameter" estimate="false" name="beta">10.0</parameter>
</Gamma>
</prior>
<prior id="RateATPrior.s:NADH2_pos1" name="distribution" x="@rateAT.s:NADH2_pos1">
<Gamma id="Gamma.104" name="distr">
<parameter id="RealParameter.245" spec="parameter.RealParameter" estimate="false" name="alpha">0.05</parameter>

<parameter id="RealParameter.246" spec="parameter.RealParameter" estimate="false"
name="beta">10.0</parameter>
</Gamma>
</prior>
<prior id="RateATPrior.s:NADH2_pos2" name="distribution" x="@rateAT.s:NADH2_pos2">
<Gamma id="Gamma.110" name="distr">
<parameter id="RealParameter.257" spec="parameter.RealParameter" estimate="false"
name="alpha">0.05</parameter>
<parameter id="RealParameter.258" spec="parameter.RealParameter" estimate="false"
name="beta">10.0</parameter>
</Gamma>
</prior>
<prior id="RateATPrior.s:NADH2_pos3" name="distribution" x="@rateAT.s:NADH2_pos3">
<Gamma id="Gamma.116" name="distr">
<parameter id="RealParameter.269" spec="parameter.RealParameter" estimate="false"
name="alpha">0.05</parameter>
<parameter id="RealParameter.270" spec="parameter.RealParameter" estimate="false"
name="beta">10.0</parameter>
</Gamma>
</prior>
<prior id="RateATPrior.s:NADH3_pos1" name="distribution" x="@rateAT.s:NADH3_pos1">
<Gamma id="Gamma.122" name="distr">
<parameter id="RealParameter.281" spec="parameter.RealParameter" estimate="false"
name="alpha">0.05</parameter>
<parameter id="RealParameter.282" spec="parameter.RealParameter" estimate="false"
name="beta">10.0</parameter>
</Gamma>
</prior>
<prior id="RateATPrior.s:NADH3_pos2" name="distribution" x="@rateAT.s:NADH3_pos2">
<Gamma id="Gamma.128" name="distr">
<parameter id="RealParameter.293" spec="parameter.RealParameter" estimate="false"
name="alpha">0.05</parameter>
<parameter id="RealParameter.294" spec="parameter.RealParameter" estimate="false"
name="beta">10.0</parameter>
</Gamma>
</prior>
<prior id="RateATPrior.s:NADH3_pos3" name="distribution" x="@rateAT.s:NADH3_pos3">
<Gamma id="Gamma.134" name="distr">
<parameter id="RealParameter.305" spec="parameter.RealParameter" estimate="false"
name="alpha">0.05</parameter>
<parameter id="RealParameter.306" spec="parameter.RealParameter" estimate="false"
name="beta">10.0</parameter>
</Gamma>
</prior>
<prior id="RateATPrior.s:NADH4L_pos1" name="distribution" x="@rateAT.s:NADH4L_pos1">
<Gamma id="Gamma.158" name="distr">
<parameter id="RealParameter.353" spec="parameter.RealParameter" estimate="false"
name="alpha">0.05</parameter>
<parameter id="RealParameter.354" spec="parameter.RealParameter" estimate="false"
name="beta">10.0</parameter>
</Gamma>
</prior>
<prior id="RateATPrior.s:NADH4L_pos2" name="distribution" x="@rateAT.s:NADH4L_pos2">
<Gamma id="Gamma.164" name="distr">
<parameter id="RealParameter.365" spec="parameter.RealParameter" estimate="false"
name="alpha">0.05</parameter>
<parameter id="RealParameter.366" spec="parameter.RealParameter" estimate="false"
name="beta">10.0</parameter>
</Gamma>
</prior>
<prior id="RateATPrior.s:NADH4L_pos3" name="distribution" x="@rateAT.s:NADH4L_pos3">
<Gamma id="Gamma.170" name="distr">

```
            <parameter id="RealParameter.377" spec="parameter.RealParameter" estimate="false"
name="alpha">0.05</parameter>
            <parameter id="RealParameter.378" spec="parameter.RealParameter" estimate="false"
name="beta">10.0</parameter>
         </Gamma>
      </prior>
      <prior id="RateATPrior.s:NADH4_pos1" name="distribution" x="@rateAT.s:NADH4_pos1">
         <Gamma id="Gamma.140" name="distr">
            <parameter id="RealParameter.317" spec="parameter.RealParameter" estimate="false"
name="alpha">0.05</parameter>
            <parameter id="RealParameter.318" spec="parameter.RealParameter" estimate="false"
name="beta">10.0</parameter>
         </Gamma>
      </prior>
      <prior id="RateATPrior.s:NADH4_pos2" name="distribution" x="@rateAT.s:NADH4_pos2">
         <Gamma id="Gamma.146" name="distr">
            <parameter id="RealParameter.329" spec="parameter.RealParameter" estimate="false"
name="alpha">0.05</parameter>
            <parameter id="RealParameter.330" spec="parameter.RealParameter" estimate="false"
name="beta">10.0</parameter>
         </Gamma>
      </prior>
      <prior id="RateATPrior.s:NADH4_pos3" name="distribution" x="@rateAT.s:NADH4_pos3">
         <Gamma id="Gamma.152" name="distr">
            <parameter id="RealParameter.341" spec="parameter.RealParameter" estimate="false"
name="alpha">0.05</parameter>
            <parameter id="RealParameter.342" spec="parameter.RealParameter" estimate="false"
name="beta">10.0</parameter>
         </Gamma>
      </prior>
      <prior id="RateATPrior.s:NADH5_pos1" name="distribution" x="@rateAT.s:NADH5_pos1">
         <Gamma id="Gamma.176" name="distr">
            <parameter id="RealParameter.389" spec="parameter.RealParameter" estimate="false"
name="alpha">0.05</parameter>
            <parameter id="RealParameter.390" spec="parameter.RealParameter" estimate="false"
name="beta">10.0</parameter>
         </Gamma>
      </prior>
      <prior id="RateATPrior.s:NADH5_pos2" name="distribution" x="@rateAT.s:NADH5_pos2">
         <Gamma id="Gamma.182" name="distr">
            <parameter id="RealParameter.401" spec="parameter.RealParameter" estimate="false"
name="alpha">0.05</parameter>
            <parameter id="RealParameter.402" spec="parameter.RealParameter" estimate="false"
name="beta">10.0</parameter>
         </Gamma>
      </prior>
      <prior id="RateATPrior.s:NADH5_pos3" name="distribution" x="@rateAT.s:NADH5_pos3">
         <Gamma id="Gamma.188" name="distr">
            <parameter id="RealParameter.413" spec="parameter.RealParameter" estimate="false"
name="alpha">0.05</parameter>
            <parameter id="RealParameter.414" spec="parameter.RealParameter" estimate="false"
name="beta">10.0</parameter>
         </Gamma>
      </prior>
      <prior id="RateATPrior.s:NADH6_pos1" name="distribution" x="@rateAT.s:NADH6_pos1">
         <Gamma id="Gamma.194" name="distr">
            <parameter id="RealParameter.425" spec="parameter.RealParameter" estimate="false"
name="alpha">0.05</parameter>
            <parameter id="RealParameter.426" spec="parameter.RealParameter" estimate="false"
name="beta">10.0</parameter>
         </Gamma>
      </prior>
      <prior id="RateATPrior.s:NADH6_pos2" name="distribution" x="@rateAT.s:NADH6_pos2">
```

```xml
<Gamma id="Gamma.200" name="distr">
<parameter id="RealParameter.437" spec="parameter.RealParameter" estimate="false" name="alpha">0.05</parameter>
<parameter id="RealParameter.438" spec="parameter.RealParameter" estimate="false" name="beta">10.0</parameter>
</Gamma>
</prior>
<prior id="RateATPrior.s:NADH6_pos3" name="distribution" x="@rateAT.s:NADH6_pos3">
<Gamma id="Gamma.206" name="distr">
<parameter id="RealParameter.449" spec="parameter.RealParameter" estimate="false" name="alpha">0.05</parameter>
<parameter id="RealParameter.450" spec="parameter.RealParameter" estimate="false" name="beta">10.0</parameter>
</Gamma>
</prior>
<prior id="RateATPrior.s:atp6_pos1" name="distribution" x="@rateAT.s:atp6_pos1">
<Gamma id="Gamma.2" name="distr">
<parameter id="RealParameter.41" spec="parameter.RealParameter" estimate="false" name="alpha">0.05</parameter>
<parameter id="RealParameter.42" spec="parameter.RealParameter" estimate="false" name="beta">10.0</parameter>
</Gamma>
</prior>
<prior id="RateATPrior.s:atp6_pos2" name="distribution" x="@rateAT.s:atp6_pos2">
<Gamma id="Gamma.8" name="distr">
<parameter id="RealParameter.53" spec="parameter.RealParameter" estimate="false" name="alpha">0.05</parameter>
<parameter id="RealParameter.54" spec="parameter.RealParameter" estimate="false" name="beta">10.0</parameter>
</Gamma>
</prior>
<prior id="RateATPrior.s:atp6_pos3" name="distribution" x="@rateAT.s:atp6_pos3">
<Gamma id="Gamma.14" name="distr">
<parameter id="RealParameter.65" spec="parameter.RealParameter" estimate="false" name="alpha">0.05</parameter>
<parameter id="RealParameter.66" spec="parameter.RealParameter" estimate="false" name="beta">10.0</parameter>
</Gamma>
</prior>
<prior id="RateCGPrior.s:COX1_pos1" name="distribution" x="@rateCG.s:COX1_pos1">
<Gamma id="Gamma.27" name="distr">
<parameter id="RealParameter.91" spec="parameter.RealParameter" estimate="false" name="alpha">0.05</parameter>
<parameter id="RealParameter.92" spec="parameter.RealParameter" estimate="false" name="beta">10.0</parameter>
</Gamma>
</prior>
<prior id="RateCGPrior.s:COX1_pos3" name="distribution" x="@rateCG.s:COX1_pos3">
<Gamma id="Gamma.33" name="distr">
<parameter id="RealParameter.103" spec="parameter.RealParameter" estimate="false" name="alpha">0.05</parameter>
<parameter id="RealParameter.104" spec="parameter.RealParameter" estimate="false" name="beta">10.0</parameter>
</Gamma>
</prior>
<prior id="RateCGPrior.s:COX2_pos1" name="distribution" x="@rateCG.s:COX2_pos1">
<Gamma id="Gamma.39" name="distr">
<parameter id="RealParameter.115" spec="parameter.RealParameter" estimate="false" name="alpha">0.05</parameter>
<parameter id="RealParameter.116" spec="parameter.RealParameter" estimate="false" name="beta">10.0</parameter>
</Gamma>
</prior>
```

<prior id="RateCGPrior.s:COX2_pos2" name="distribution" x="@rateCG.s:COX2_pos2">
<Gamma id="Gamma.148" name="distr">
<parameter id="RealParameter.617" spec="parameter.RealParameter" estimate="false"
name="alpha">0.05</parameter>
<parameter id="RealParameter.618" spec="parameter.RealParameter" estimate="false"
name="beta">10.0</parameter>
</Gamma>
</prior>
<prior id="RateCGPrior.s:COX2_pos3" name="distribution" x="@rateCG.s:COX2_pos3">
<Gamma id="Gamma.45" name="distr">
<parameter id="RealParameter.127" spec="parameter.RealParameter" estimate="false"
name="alpha">0.05</parameter>
<parameter id="RealParameter.128" spec="parameter.RealParameter" estimate="false"
name="beta">10.0</parameter>
</Gamma>
</prior>
<prior id="RateCGPrior.s:COX3_pos1" name="distribution" x="@rateCG.s:COX3_pos1">
<Gamma id="Gamma.51" name="distr">
<parameter id="RealParameter.139" spec="parameter.RealParameter" estimate="false"
name="alpha">0.05</parameter>
<parameter id="RealParameter.140" spec="parameter.RealParameter" estimate="false"
name="beta">10.0</parameter>
</Gamma>
</prior>
<prior id="RateCGPrior.s:COX3_pos2" name="distribution" x="@rateCG.s:COX3_pos2">
<Gamma id="Gamma.57" name="distr">
<parameter id="RealParameter.151" spec="parameter.RealParameter" estimate="false"
name="alpha">0.05</parameter>
<parameter id="RealParameter.152" spec="parameter.RealParameter" estimate="false"
name="beta">10.0</parameter>
</Gamma>
</prior>
<prior id="RateCGPrior.s:COX3_pos3" name="distribution" x="@rateCG.s:COX3_pos3">
<Gamma id="Gamma.63" name="distr">
<parameter id="RealParameter.163" spec="parameter.RealParameter" estimate="false"
name="alpha">0.05</parameter>
<parameter id="RealParameter.164" spec="parameter.RealParameter" estimate="false"
name="beta">10.0</parameter>
</Gamma>
</prior>
<prior id="RateCGPrior.s:CYTB_pos1" name="distribution" x="@rateCG.s:CYTB_pos1">
<Gamma id="Gamma.69" name="distr">
<parameter id="RealParameter.175" spec="parameter.RealParameter" estimate="false"
name="alpha">0.05</parameter>
<parameter id="RealParameter.176" spec="parameter.RealParameter" estimate="false"
name="beta">10.0</parameter>
</Gamma>
</prior>
<prior id="RateCGPrior.s:CYTB_pos2" name="distribution" x="@rateCG.s:CYTB_pos2">
<Gamma id="Gamma.75" name="distr">
<parameter id="RealParameter.187" spec="parameter.RealParameter" estimate="false"
name="alpha">0.05</parameter>
<parameter id="RealParameter.188" spec="parameter.RealParameter" estimate="false"
name="beta">10.0</parameter>
</Gamma>
</prior>
<prior id="RateCGPrior.s:CYTB_pos3" name="distribution" x="@rateCG.s:CYTB_pos3">
<Gamma id="Gamma.81" name="distr">
<parameter id="RealParameter.199" spec="parameter.RealParameter" estimate="false"
name="alpha">0.05</parameter>
<parameter id="RealParameter.200" spec="parameter.RealParameter" estimate="false"
name="beta">10.0</parameter>
</Gamma>

</prior>
<prior id="RateCGPrior.s:Mytilidae27sq12PCGs" name="distribution"
x="@rateCG.s:Mytilidae27sq12PCGs">
<Gamma id="Gamma.291" name="distr">
<parameter id="RealParameter.1526" spec="parameter.RealParameter" estimate="false"
name="alpha">0.05</parameter>
<parameter id="RealParameter.1527" spec="parameter.RealParameter" estimate="false"
name="beta">10.0</parameter>
</Gamma>
</prior>
<prior id="RateCGPrior.s:NADH1_pos1" name="distribution" x="@rateCG.s:NADH1_pos1">
<Gamma id="Gamma.87" name="distr">
<parameter id="RealParameter.211" spec="parameter.RealParameter" estimate="false"
name="alpha">0.05</parameter>
<parameter id="RealParameter.212" spec="parameter.RealParameter" estimate="false"
name="beta">10.0</parameter>
</Gamma>
</prior>
<prior id="RateCGPrior.s:NADH1_pos2" name="distribution" x="@rateCG.s:NADH1_pos2">
<Gamma id="Gamma.93" name="distr">
<parameter id="RealParameter.223" spec="parameter.RealParameter" estimate="false"
name="alpha">0.05</parameter>
<parameter id="RealParameter.224" spec="parameter.RealParameter" estimate="false"
name="beta">10.0</parameter>
</Gamma>
</prior>
<prior id="RateCGPrior.s:NADH1_pos3" name="distribution" x="@rateCG.s:NADH1_pos3">
<Gamma id="Gamma.99" name="distr">
<parameter id="RealParameter.235" spec="parameter.RealParameter" estimate="false"
name="alpha">0.05</parameter>
<parameter id="RealParameter.236" spec="parameter.RealParameter" estimate="false"
name="beta">10.0</parameter>
</Gamma>
</prior>
<prior id="RateCGPrior.s:NADH2_pos1" name="distribution" x="@rateCG.s:NADH2_pos1">
<Gamma id="Gamma.105" name="distr">
<parameter id="RealParameter.247" spec="parameter.RealParameter" estimate="false"
name="alpha">0.05</parameter>
<parameter id="RealParameter.248" spec="parameter.RealParameter" estimate="false"
name="beta">10.0</parameter>
</Gamma>
</prior>
<prior id="RateCGPrior.s:NADH2_pos2" name="distribution" x="@rateCG.s:NADH2_pos2">
<Gamma id="Gamma.111" name="distr">
<parameter id="RealParameter.259" spec="parameter.RealParameter" estimate="false"
name="alpha">0.05</parameter>
<parameter id="RealParameter.260" spec="parameter.RealParameter" estimate="false"
name="beta">10.0</parameter>
</Gamma>
</prior>
<prior id="RateCGPrior.s:NADH2_pos3" name="distribution" x="@rateCG.s:NADH2_pos3">
<Gamma id="Gamma.117" name="distr">
<parameter id="RealParameter.271" spec="parameter.RealParameter" estimate="false"
name="alpha">0.05</parameter>
<parameter id="RealParameter.272" spec="parameter.RealParameter" estimate="false"
name="beta">10.0</parameter>
</Gamma>
</prior>
<prior id="RateCGPrior.s:NADH3_pos1" name="distribution" x="@rateCG.s:NADH3_pos1">
<Gamma id="Gamma.123" name="distr">
<parameter id="RealParameter.283" spec="parameter.RealParameter" estimate="false"
name="alpha">0.05</parameter>

```
<parameter id="RealParameter.284" spec="parameter.RealParameter" estimate="false"
name="beta">10.0</parameter>
</Gamma>
</prior>
<prior id="RateCGPrior.s:NADH3_pos2" name="distribution" x="@rateCG.s:NADH3_pos2">
<Gamma id="Gamma.129" name="distr">
<parameter id="RealParameter.295" spec="parameter.RealParameter" estimate="false"
name="alpha">0.05</parameter>
<parameter id="RealParameter.296" spec="parameter.RealParameter" estimate="false"
name="beta">10.0</parameter>
</Gamma>
</prior>
<prior id="RateCGPrior.s:NADH3_pos3" name="distribution" x="@rateCG.s:NADH3_pos3">
<Gamma id="Gamma.135" name="distr">
<parameter id="RealParameter.307" spec="parameter.RealParameter" estimate="false"
name="alpha">0.05</parameter>
<parameter id="RealParameter.308" spec="parameter.RealParameter" estimate="false"
name="beta">10.0</parameter>
</Gamma>
</prior>
<prior id="RateCGPrior.s:NADH4L_pos1" name="distribution" x="@rateCG.s:NADH4L_pos1">
<Gamma id="Gamma.159" name="distr">
<parameter id="RealParameter.355" spec="parameter.RealParameter" estimate="false"
name="alpha">0.05</parameter>
<parameter id="RealParameter.356" spec="parameter.RealParameter" estimate="false"
name="beta">10.0</parameter>
</Gamma>
</prior>
<prior id="RateCGPrior.s:NADH4L_pos2" name="distribution" x="@rateCG.s:NADH4L_pos2">
<Gamma id="Gamma.165" name="distr">
<parameter id="RealParameter.367" spec="parameter.RealParameter" estimate="false"
name="alpha">0.05</parameter>
<parameter id="RealParameter.368" spec="parameter.RealParameter" estimate="false"
name="beta">10.0</parameter>
</Gamma>
</prior>
<prior id="RateCGPrior.s:NADH4L_pos3" name="distribution" x="@rateCG.s:NADH4L_pos3">
<Gamma id="Gamma.171" name="distr">
<parameter id="RealParameter.379" spec="parameter.RealParameter" estimate="false"
name="alpha">0.05</parameter>
<parameter id="RealParameter.380" spec="parameter.RealParameter" estimate="false"
name="beta">10.0</parameter>
</Gamma>
</prior>
<prior id="RateCGPrior.s:NADH4_pos1" name="distribution" x="@rateCG.s:NADH4_pos1">
<Gamma id="Gamma.141" name="distr">
<parameter id="RealParameter.319" spec="parameter.RealParameter" estimate="false"
name="alpha">0.05</parameter>
<parameter id="RealParameter.320" spec="parameter.RealParameter" estimate="false"
name="beta">10.0</parameter>
</Gamma>
</prior>
<prior id="RateCGPrior.s:NADH4_pos2" name="distribution" x="@rateCG.s:NADH4_pos2">
<Gamma id="Gamma.147" name="distr">
<parameter id="RealParameter.331" spec="parameter.RealParameter" estimate="false"
name="alpha">0.05</parameter>
<parameter id="RealParameter.332" spec="parameter.RealParameter" estimate="false"
name="beta">10.0</parameter>
</Gamma>
</prior>
<prior id="RateCGPrior.s:NADH4_pos3" name="distribution" x="@rateCG.s:NADH4_pos3">
<Gamma id="Gamma.153" name="distr">
```

<parameter id="RealParameter.343" spec="parameter.RealParameter" estimate="false"
name="alpha">0.05</parameter>
<parameter id="RealParameter.344" spec="parameter.RealParameter" estimate="false"
name="beta">10.0</parameter>
</Gamma>
</prior>
<prior id="RateCGPrior.s:NADH5_pos1" name="distribution" x="@rateCG.s:NADH5_pos1">
<Gamma id="Gamma.177" name="distr">
<parameter id="RealParameter.391" spec="parameter.RealParameter" estimate="false"
name="alpha">0.05</parameter>
<parameter id="RealParameter.392" spec="parameter.RealParameter" estimate="false"
name="beta">10.0</parameter>
</Gamma>
</prior>
<prior id="RateCGPrior.s:NADH5_pos2" name="distribution" x="@rateCG.s:NADH5_pos2">
<Gamma id="Gamma.183" name="distr">
<parameter id="RealParameter.403" spec="parameter.RealParameter" estimate="false"
name="alpha">0.05</parameter>
<parameter id="RealParameter.404" spec="parameter.RealParameter" estimate="false"
name="beta">10.0</parameter>
</Gamma>
</prior>
<prior id="RateCGPrior.s:NADH5_pos3" name="distribution" x="@rateCG.s:NADH5_pos3">
<Gamma id="Gamma.189" name="distr">
<parameter id="RealParameter.415" spec="parameter.RealParameter" estimate="false"
name="alpha">0.05</parameter>
<parameter id="RealParameter.416" spec="parameter.RealParameter" estimate="false"
name="beta">10.0</parameter>
</Gamma>
</prior>
<prior id="RateCGPrior.s:NADH6_pos1" name="distribution" x="@rateCG.s:NADH6_pos1">
<Gamma id="Gamma.195" name="distr">
<parameter id="RealParameter.427" spec="parameter.RealParameter" estimate="false"
name="alpha">0.05</parameter>
<parameter id="RealParameter.428" spec="parameter.RealParameter" estimate="false"
name="beta">10.0</parameter>
</Gamma>
</prior>
<prior id="RateCGPrior.s:NADH6_pos2" name="distribution" x="@rateCG.s:NADH6_pos2">
<Gamma id="Gamma.201" name="distr">
<parameter id="RealParameter.439" spec="parameter.RealParameter" estimate="false"
name="alpha">0.05</parameter>
<parameter id="RealParameter.440" spec="parameter.RealParameter" estimate="false"
name="beta">10.0</parameter>
</Gamma>
</prior>
<prior id="RateCGPrior.s:NADH6_pos3" name="distribution" x="@rateCG.s:NADH6_pos3">
<Gamma id="Gamma.207" name="distr">
<parameter id="RealParameter.451" spec="parameter.RealParameter" estimate="false"
name="alpha">0.05</parameter>
<parameter id="RealParameter.452" spec="parameter.RealParameter" estimate="false"
name="beta">10.0</parameter>
</Gamma>
</prior>
<prior id="RateCGPrior.s:atp6_pos1" name="distribution" x="@rateCG.s:atp6_pos1">
<Gamma id="Gamma.3" name="distr">
<parameter id="RealParameter.43" spec="parameter.RealParameter" estimate="false"
name="alpha">0.05</parameter>
<parameter id="RealParameter.44" spec="parameter.RealParameter" estimate="false"
name="beta">10.0</parameter>
</Gamma>
</prior>
<prior id="RateCGPrior.s:atp6_pos2" name="distribution" x="@rateCG.s:atp6_pos2">

```xml
<Gamma id="Gamma.9" name="distr">
<parameter id="RealParameter.55" spec="parameter.RealParameter" estimate="false" name="alpha">0.05</parameter>
<parameter id="RealParameter.56" spec="parameter.RealParameter" estimate="false" name="beta">10.0</parameter>
</Gamma>
</prior>
<prior id="RateCGPrior.s:atp6_pos3" name="distribution" x="@rateCG.s:atp6_pos3">
<Gamma id="Gamma.15" name="distr">
<parameter id="RealParameter.67" spec="parameter.RealParameter" estimate="false" name="alpha">0.05</parameter>
<parameter id="RealParameter.68" spec="parameter.RealParameter" estimate="false" name="beta">10.0</parameter>
</Gamma>
</prior>
<prior id="RateGTPrior.s:COX1_pos1" name="distribution" x="@rateGT.s:COX1_pos1">
<Gamma id="Gamma.29" name="distr">
<parameter id="RealParameter.95" spec="parameter.RealParameter" estimate="false" name="alpha">0.05</parameter>
<parameter id="RealParameter.96" spec="parameter.RealParameter" estimate="false" name="beta">10.0</parameter>
</Gamma>
</prior>
<prior id="RateGTPrior.s:COX1_pos3" name="distribution" x="@rateGT.s:COX1_pos3">
<Gamma id="Gamma.35" name="distr">
<parameter id="RealParameter.107" spec="parameter.RealParameter" estimate="false" name="alpha">0.05</parameter>
<parameter id="RealParameter.108" spec="parameter.RealParameter" estimate="false" name="beta">10.0</parameter>
</Gamma>
</prior>
<prior id="RateGTPrior.s:COX2_pos1" name="distribution" x="@rateGT.s:COX2_pos1">
<Gamma id="Gamma.41" name="distr">
<parameter id="RealParameter.119" spec="parameter.RealParameter" estimate="false" name="alpha">0.05</parameter>
<parameter id="RealParameter.120" spec="parameter.RealParameter" estimate="false" name="beta">10.0</parameter>
</Gamma>
</prior>
<prior id="RateGTPrior.s:COX2_pos2" name="distribution" x="@rateGT.s:COX2_pos2">
<Gamma id="Gamma.160" name="distr">
<parameter id="RealParameter.621" spec="parameter.RealParameter" estimate="false" name="alpha">0.05</parameter>
<parameter id="RealParameter.622" spec="parameter.RealParameter" estimate="false" name="beta">10.0</parameter>
</Gamma>
</prior>
<prior id="RateGTPrior.s:COX2_pos3" name="distribution" x="@rateGT.s:COX2_pos3">
<Gamma id="Gamma.47" name="distr">
<parameter id="RealParameter.131" spec="parameter.RealParameter" estimate="false" name="alpha">0.05</parameter>
<parameter id="RealParameter.132" spec="parameter.RealParameter" estimate="false" name="beta">10.0</parameter>
</Gamma>
</prior>
<prior id="RateGTPrior.s:COX3_pos1" name="distribution" x="@rateGT.s:COX3_pos1">
<Gamma id="Gamma.53" name="distr">
<parameter id="RealParameter.143" spec="parameter.RealParameter" estimate="false" name="alpha">0.05</parameter>
<parameter id="RealParameter.144" spec="parameter.RealParameter" estimate="false" name="beta">10.0</parameter>
</Gamma>
</prior>
```

```
<prior id="RateGTPrior.s:COX3_pos2" name="distribution" x="@rateGT.s:COX3_pos2">
  <Gamma id="Gamma.59" name="distr">
    <parameter id="RealParameter.155" spec="parameter.RealParameter" estimate="false"
name="alpha">0.05</parameter>
    <parameter id="RealParameter.156" spec="parameter.RealParameter" estimate="false"
name="beta">10.0</parameter>
  </Gamma>
</prior>
<prior id="RateGTPrior.s:COX3_pos3" name="distribution" x="@rateGT.s:COX3_pos3">
  <Gamma id="Gamma.65" name="distr">
    <parameter id="RealParameter.167" spec="parameter.RealParameter" estimate="false"
name="alpha">0.05</parameter>
    <parameter id="RealParameter.168" spec="parameter.RealParameter" estimate="false"
name="beta">10.0</parameter>
  </Gamma>
</prior>
<prior id="RateGTPrior.s:CYTB_pos1" name="distribution" x="@rateGT.s:CYTB_pos1">
  <Gamma id="Gamma.71" name="distr">
    <parameter id="RealParameter.179" spec="parameter.RealParameter" estimate="false"
name="alpha">0.05</parameter>
    <parameter id="RealParameter.180" spec="parameter.RealParameter" estimate="false"
name="beta">10.0</parameter>
  </Gamma>
</prior>
<prior id="RateGTPrior.s:CYTB_pos2" name="distribution" x="@rateGT.s:CYTB_pos2">
  <Gamma id="Gamma.77" name="distr">
    <parameter id="RealParameter.191" spec="parameter.RealParameter" estimate="false"
name="alpha">0.05</parameter>
    <parameter id="RealParameter.192" spec="parameter.RealParameter" estimate="false"
name="beta">10.0</parameter>
  </Gamma>
</prior>
<prior id="RateGTPrior.s:CYTB_pos3" name="distribution" x="@rateGT.s:CYTB_pos3">
  <Gamma id="Gamma.83" name="distr">
    <parameter id="RealParameter.203" spec="parameter.RealParameter" estimate="false"
name="alpha">0.05</parameter>
    <parameter id="RealParameter.204" spec="parameter.RealParameter" estimate="false"
name="beta">10.0</parameter>
  </Gamma>
</prior>
<prior id="RateGTPrior.s:Mytilidae27sq12PCGs" name="distribution"
x="@rateGT.s:Mytilidae27sq12PCGs">
  <Gamma id="Gamma.293" name="distr">
    <parameter id="RealParameter.1530" spec="parameter.RealParameter" estimate="false"
name="alpha">0.05</parameter>
    <parameter id="RealParameter.1531" spec="parameter.RealParameter" estimate="false"
name="beta">10.0</parameter>
  </Gamma>
</prior>
<prior id="RateGTPrior.s:NADH1_pos1" name="distribution" x="@rateGT.s:NADH1_pos1">
  <Gamma id="Gamma.89" name="distr">
    <parameter id="RealParameter.215" spec="parameter.RealParameter" estimate="false"
name="alpha">0.05</parameter>
    <parameter id="RealParameter.216" spec="parameter.RealParameter" estimate="false"
name="beta">10.0</parameter>
  </Gamma>
</prior>
<prior id="RateGTPrior.s:NADH1_pos2" name="distribution" x="@rateGT.s:NADH1_pos2">
  <Gamma id="Gamma.95" name="distr">
    <parameter id="RealParameter.227" spec="parameter.RealParameter" estimate="false"
name="alpha">0.05</parameter>
    <parameter id="RealParameter.228" spec="parameter.RealParameter" estimate="false"
name="beta">10.0</parameter>
```

</Gamma>
</prior>
<prior id="RateGTPrior.s:NADH1_pos3" name="distribution" x="@rateGT.s:NADH1_pos3">
<Gamma id="Gamma.101" name="distr">
<parameter id="RealParameter.239" spec="parameter.RealParameter" estimate="false"
name="alpha">0.05</parameter>
<parameter id="RealParameter.240" spec="parameter.RealParameter" estimate="false"
name="beta">10.0</parameter>
</Gamma>
</prior>
<prior id="RateGTPrior.s:NADH2_pos1" name="distribution" x="@rateGT.s:NADH2_pos1">
<Gamma id="Gamma.107" name="distr">
<parameter id="RealParameter.251" spec="parameter.RealParameter" estimate="false"
name="alpha">0.05</parameter>
<parameter id="RealParameter.252" spec="parameter.RealParameter" estimate="false"
name="beta">10.0</parameter>
</Gamma>
</prior>
<prior id="RateGTPrior.s:NADH2_pos2" name="distribution" x="@rateGT.s:NADH2_pos2">
<Gamma id="Gamma.113" name="distr">
<parameter id="RealParameter.263" spec="parameter.RealParameter" estimate="false"
name="alpha">0.05</parameter>
<parameter id="RealParameter.264" spec="parameter.RealParameter" estimate="false"
name="beta">10.0</parameter>
</Gamma>
</prior>
<prior id="RateGTPrior.s:NADH2_pos3" name="distribution" x="@rateGT.s:NADH2_pos3">
<Gamma id="Gamma.119" name="distr">
<parameter id="RealParameter.275" spec="parameter.RealParameter" estimate="false"
name="alpha">0.05</parameter>
<parameter id="RealParameter.276" spec="parameter.RealParameter" estimate="false"
name="beta">10.0</parameter>
</Gamma>
</prior>
<prior id="RateGTPrior.s:NADH3_pos1" name="distribution" x="@rateGT.s:NADH3_pos1">
<Gamma id="Gamma.125" name="distr">
<parameter id="RealParameter.287" spec="parameter.RealParameter" estimate="false"
name="alpha">0.05</parameter>
<parameter id="RealParameter.288" spec="parameter.RealParameter" estimate="false"
name="beta">10.0</parameter>
</Gamma>
</prior>
<prior id="RateGTPrior.s:NADH3_pos2" name="distribution" x="@rateGT.s:NADH3_pos2">
<Gamma id="Gamma.131" name="distr">
<parameter id="RealParameter.299" spec="parameter.RealParameter" estimate="false"
name="alpha">0.05</parameter>
<parameter id="RealParameter.300" spec="parameter.RealParameter" estimate="false"
name="beta">10.0</parameter>
</Gamma>
</prior>
<prior id="RateGTPrior.s:NADH3_pos3" name="distribution" x="@rateGT.s:NADH3_pos3">
<Gamma id="Gamma.137" name="distr">
<parameter id="RealParameter.311" spec="parameter.RealParameter" estimate="false"
name="alpha">0.05</parameter>
<parameter id="RealParameter.312" spec="parameter.RealParameter" estimate="false"
name="beta">10.0</parameter>
</Gamma>
</prior>
<prior id="RateGTPrior.s:NADH4L_pos1" name="distribution" x="@rateGT.s:NADH4L_pos1">
<Gamma id="Gamma.161" name="distr">
<parameter id="RealParameter.359" spec="parameter.RealParameter" estimate="false"
name="alpha">0.05</parameter>

```
        <parameter id="RealParameter.360" spec="parameter.RealParameter" estimate="false"
name="beta">10.0</parameter>
      </Gamma>
    </prior>
    <prior id="RateGTPrior.s:NADH4L_pos2" name="distribution" x="@rateGT.s:NADH4L_pos2">
      <Gamma id="Gamma.167" name="distr">
        <parameter id="RealParameter.371" spec="parameter.RealParameter" estimate="false"
name="alpha">0.05</parameter>
        <parameter id="RealParameter.372" spec="parameter.RealParameter" estimate="false"
name="beta">10.0</parameter>
      </Gamma>
    </prior>
    <prior id="RateGTPrior.s:NADH4L_pos3" name="distribution" x="@rateGT.s:NADH4L_pos3">
      <Gamma id="Gamma.173" name="distr">
        <parameter id="RealParameter.383" spec="parameter.RealParameter" estimate="false"
name="alpha">0.05</parameter>
        <parameter id="RealParameter.384" spec="parameter.RealParameter" estimate="false"
name="beta">10.0</parameter>
      </Gamma>
    </prior>
    <prior id="RateGTPrior.s:NADH4_pos1" name="distribution" x="@rateGT.s:NADH4_pos1">
      <Gamma id="Gamma.143" name="distr">
        <parameter id="RealParameter.323" spec="parameter.RealParameter" estimate="false"
name="alpha">0.05</parameter>
        <parameter id="RealParameter.324" spec="parameter.RealParameter" estimate="false"
name="beta">10.0</parameter>
      </Gamma>
    </prior>
    <prior id="RateGTPrior.s:NADH4_pos2" name="distribution" x="@rateGT.s:NADH4_pos2">
      <Gamma id="Gamma.149" name="distr">
        <parameter id="RealParameter.335" spec="parameter.RealParameter" estimate="false"
name="alpha">0.05</parameter>
        <parameter id="RealParameter.336" spec="parameter.RealParameter" estimate="false"
name="beta">10.0</parameter>
      </Gamma>
    </prior>
    <prior id="RateGTPrior.s:NADH4_pos3" name="distribution" x="@rateGT.s:NADH4_pos3">
      <Gamma id="Gamma.155" name="distr">
        <parameter id="RealParameter.347" spec="parameter.RealParameter" estimate="false"
name="alpha">0.05</parameter>
        <parameter id="RealParameter.348" spec="parameter.RealParameter" estimate="false"
name="beta">10.0</parameter>
      </Gamma>
    </prior>
    <prior id="RateGTPrior.s:NADH5_pos1" name="distribution" x="@rateGT.s:NADH5_pos1">
      <Gamma id="Gamma.179" name="distr">
        <parameter id="RealParameter.395" spec="parameter.RealParameter" estimate="false"
name="alpha">0.05</parameter>
        <parameter id="RealParameter.396" spec="parameter.RealParameter" estimate="false"
name="beta">10.0</parameter>
      </Gamma>
    </prior>
    <prior id="RateGTPrior.s:NADH5_pos2" name="distribution" x="@rateGT.s:NADH5_pos2">
      <Gamma id="Gamma.185" name="distr">
        <parameter id="RealParameter.407" spec="parameter.RealParameter" estimate="false"
name="alpha">0.05</parameter>
        <parameter id="RealParameter.408" spec="parameter.RealParameter" estimate="false"
name="beta">10.0</parameter>
      </Gamma>
    </prior>
    <prior id="RateGTPrior.s:NADH5_pos3" name="distribution" x="@rateGT.s:NADH5_pos3">
      <Gamma id="Gamma.191" name="distr">
```

```
                <parameter id="RealParameter.419" spec="parameter.RealParameter" estimate="false"
name="alpha">0.05</parameter>
                <parameter id="RealParameter.420" spec="parameter.RealParameter" estimate="false"
name="beta">10.0</parameter>
            </Gamma>
        </prior>
        <prior id="RateGTPrior.s:NADH6_pos1" name="distribution" x="@rateGT.s:NADH6_pos1">
            <Gamma id="Gamma.197" name="distr">
                <parameter id="RealParameter.431" spec="parameter.RealParameter" estimate="false"
name="alpha">0.05</parameter>
                <parameter id="RealParameter.432" spec="parameter.RealParameter" estimate="false"
name="beta">10.0</parameter>
            </Gamma>
        </prior>
        <prior id="RateGTPrior.s:NADH6_pos2" name="distribution" x="@rateGT.s:NADH6_pos2">
            <Gamma id="Gamma.203" name="distr">
                <parameter id="RealParameter.443" spec="parameter.RealParameter" estimate="false"
name="alpha">0.05</parameter>
                <parameter id="RealParameter.444" spec="parameter.RealParameter" estimate="false"
name="beta">10.0</parameter>
            </Gamma>
        </prior>
        <prior id="RateGTPrior.s:NADH6_pos3" name="distribution" x="@rateGT.s:NADH6_pos3">
            <Gamma id="Gamma.209" name="distr">
                <parameter id="RealParameter.455" spec="parameter.RealParameter" estimate="false"
name="alpha">0.05</parameter>
                <parameter id="RealParameter.456" spec="parameter.RealParameter" estimate="false"
name="beta">10.0</parameter>
            </Gamma>
        </prior>
        <prior id="RateGTPrior.s:atp6_pos1" name="distribution" x="@rateGT.s:atp6_pos1">
            <Gamma id="Gamma.5" name="distr">
                <parameter id="RealParameter.47" spec="parameter.RealParameter" estimate="false"
name="alpha">0.05</parameter>
                <parameter id="RealParameter.48" spec="parameter.RealParameter" estimate="false"
name="beta">10.0</parameter>
            </Gamma>
        </prior>
        <prior id="RateGTPrior.s:atp6_pos2" name="distribution" x="@rateGT.s:atp6_pos2">
            <Gamma id="Gamma.11" name="distr">
                <parameter id="RealParameter.59" spec="parameter.RealParameter" estimate="false"
name="alpha">0.05</parameter>
                <parameter id="RealParameter.60" spec="parameter.RealParameter" estimate="false"
name="beta">10.0</parameter>
            </Gamma>
        </prior>
        <prior id="RateGTPrior.s:atp6_pos3" name="distribution" x="@rateGT.s:atp6_pos3">
            <Gamma id="Gamma.17" name="distr">
                <parameter id="RealParameter.71" spec="parameter.RealParameter" estimate="false"
name="alpha">0.05</parameter>
                <parameter id="RealParameter.72" spec="parameter.RealParameter" estimate="false"
name="beta">10.0</parameter>
            </Gamma>
        </prior>
    </distribution>
    <distribution id="likelihood" spec="util.CompoundDistribution" useThreads="true">
        <distribution id="treeLikelihood.atp6_pos1" spec="ThreadedTreeLikelihood" data="@atp6_pos1"
tree="@Tree.t:atp6_pos1">
            <siteModel id="SiteModel.s:atp6_pos1" spec="SiteModel" gammaCategoryCount="15"
proportionInvariant="@proportionInvariant.s:atp6_pos1" shape="@gammaShape.s:atp6_pos1">
                <parameter id="mutationRate.s:atp6_pos1" spec="parameter.RealParameter" estimate="false"
name="mutationRate">2.93E-5</parameter>
```

```
                <substModel id="gtr.s:atp6_pos1" spec="GTR" rateAC="@rateAC.s:atp6_pos1"
rateAG="@rateAG.s:atp6_pos1" rateAT="@rateAT.s:atp6_pos1" rateCG="@rateCG.s:atp6_pos1"
rateGT="@rateGT.s:atp6_pos1">
                    <parameter id="rateCT.s:atp6_pos1" spec="parameter.RealParameter" estimate="false" lower="0.0"
name="rateCT">1.0</parameter>
                    <frequencies id="estimatedFreqs.s:atp6_pos1" spec="Frequencies"
frequencies="@freqParameter.s:atp6_pos1"/>
                </substModel>
            </siteModel>
            <branchRateModel id="StrictClock.c:atp6_pos1"
spec="beast.evolution.branchratemodel.StrictClockModel" clock.rate="@clockRate.c:atp6_pos1"/>
        </distribution>
        <distribution id="treeLikelihood.atp6_pos2" spec="ThreadedTreeLikelihood" data="@atp6_pos2"
tree="@Tree.t:atp6_pos2">
            <siteModel id="SiteModel.s:atp6_pos2" spec="SiteModel" gammaCategoryCount="15"
proportionInvariant="@proportionInvariant.s:atp6_pos2" shape="@gammaShape.s:atp6_pos2">
                <parameter id="mutationRate.s:atp6_pos2" spec="parameter.RealParameter" estimate="false"
name="mutationRate">2.93E-5</parameter>
                <substModel id="gtr.s:atp6_pos2" spec="GTR" rateAC="@rateAC.s:atp6_pos2"
rateAG="@rateAG.s:atp6_pos2" rateAT="@rateAT.s:atp6_pos2" rateCG="@rateCG.s:atp6_pos2"
rateGT="@rateGT.s:atp6_pos2">
                    <parameter id="rateCT.s:atp6_pos2" spec="parameter.RealParameter" estimate="false" lower="0.0"
name="rateCT">1.0</parameter>
                    <frequencies id="estimatedFreqs.s:atp6_pos2" spec="Frequencies"
frequencies="@freqParameter.s:atp6_pos2"/>
                </substModel>
            </siteModel>
            <branchRateModel id="StrictClock.c:atp6_pos2"
spec="beast.evolution.branchratemodel.StrictClockModel" clock.rate="@clockRate.c:atp6_pos2"/>
        </distribution>
        <distribution id="treeLikelihood.atp6_pos3" spec="ThreadedTreeLikelihood" data="@atp6_pos3"
tree="@Tree.t:atp6_pos3">
            <siteModel id="SiteModel.s:atp6_pos3" spec="SiteModel" gammaCategoryCount="15"
proportionInvariant="@proportionInvariant.s:atp6_pos3" shape="@gammaShape.s:atp6_pos3">
                <parameter id="mutationRate.s:atp6_pos3" spec="parameter.RealParameter" estimate="false"
name="mutationRate">1.0</parameter>
                <substModel id="gtr.s:atp6_pos3" spec="GTR" rateAC="@rateAC.s:atp6_pos3"
rateAG="@rateAG.s:atp6_pos3" rateAT="@rateAT.s:atp6_pos3" rateCG="@rateCG.s:atp6_pos3"
rateGT="@rateGT.s:atp6_pos3">
                    <parameter id="rateCT.s:atp6_pos3" spec="parameter.RealParameter" estimate="false" lower="0.0"
name="rateCT">1.0</parameter>
                    <frequencies id="estimatedFreqs.s:atp6_pos3" spec="Frequencies"
frequencies="@freqParameter.s:atp6_pos3"/>
                </substModel>
            </siteModel>
            <branchRateModel id="StrictClock.c:atp6_pos3"
spec="beast.evolution.branchratemodel.StrictClockModel" clock.rate="@clockRate.c:atp6_pos3"/>
        </distribution>
        <distribution id="treeLikelihood.COX1_pos1" spec="ThreadedTreeLikelihood" data="@COX1_pos1"
tree="@Tree.t:COX1_pos1">
            <siteModel id="SiteModel.s:COX1_pos1" spec="SiteModel" gammaCategoryCount="15"
proportionInvariant="@proportionInvariant.s:COX1_pos1" shape="@gammaShape.s:COX1_pos1">
                <parameter id="mutationRate.s:COX1_pos1" spec="parameter.RealParameter" estimate="false"
name="mutationRate">2.93E-5</parameter>
                <substModel id="gtr.s:COX1_pos1" spec="GTR" rateAC="@rateAC.s:COX1_pos1"
rateAG="@rateAG.s:COX1_pos1" rateAT="@rateAT.s:COX1_pos1" rateCG="@rateCG.s:COX1_pos1"
rateGT="@rateGT.s:COX1_pos1">
                    <parameter id="rateCT.s:COX1_pos1" spec="parameter.RealParameter" estimate="false"
lower="0.0" name="rateCT">1.0</parameter>
                    <frequencies id="estimatedFreqs.s:COX1_pos1" spec="Frequencies"
frequencies="@freqParameter.s:COX1_pos1"/>
                </substModel>
            </siteModel>
```

```xml
<branchRateModel id="StrictClock.c:COX1_pos1" spec="beast.evolution.branchratemodel.StrictClockModel" clock.rate="@clockRate.c:COX1_pos1"/>
</distribution>
<distribution id="treeLikelihood.COX1_pos2" spec="ThreadedTreeLikelihood" data="@COX1_pos2" tree="@Tree.t:COX1_pos2">
<siteModel id="SiteModel.s:COX1_pos2" spec="SiteModel" gammaCategoryCount="15" proportionInvariant="@proportionInvariant.s:COX1_pos2" shape="@gammaShape.s:COX1_pos2">
<parameter id="mutationRate.s:COX1_pos2" spec="parameter.RealParameter" estimate="false" name="mutationRate">2.93E-5</parameter>
<substModel id="JC69.s:COX1_pos2" spec="JukesCantor"/>
</siteModel>
<branchRateModel id="StrictClock.c:COX1_pos2" spec="beast.evolution.branchratemodel.StrictClockModel" clock.rate="@clockRate.c:COX1_pos2"/>
</distribution>
<distribution id="treeLikelihood.COX1_pos3" spec="ThreadedTreeLikelihood" data="@COX1_pos3" tree="@Tree.t:COX1_pos3">
<siteModel id="SiteModel.s:COX1_pos3" spec="SiteModel" gammaCategoryCount="15" proportionInvariant="@proportionInvariant.s:COX1_pos3" shape="@gammaShape.s:COX1_pos3">
<parameter id="mutationRate.s:COX1_pos3" spec="parameter.RealParameter" estimate="false" name="mutationRate">1.0</parameter>
<substModel id="gtr.s:COX1_pos3" spec="GTR" rateAC="@rateAC.s:COX1_pos3" rateAG="@rateAG.s:COX1_pos3" rateAT="@rateAT.s:COX1_pos3" rateCG="@rateCG.s:COX1_pos3" rateGT="@rateGT.s:COX1_pos3">
<parameter id="rateCT.s:COX1_pos3" spec="parameter.RealParameter" estimate="false" lower="0.0" name="rateCT">1.0</parameter>
<frequencies id="estimatedFreqs.s:COX1_pos3" spec="Frequencies" frequencies="@freqParameter.s:COX1_pos3"/>
</substModel>
</siteModel>
<branchRateModel id="StrictClock.c:COX1_pos3" spec="beast.evolution.branchratemodel.StrictClockModel" clock.rate="@clockRate.c:COX1_pos3"/>
</distribution>
<distribution id="treeLikelihood.COX2_pos1" spec="ThreadedTreeLikelihood" data="@COX2_pos1" tree="@Tree.t:COX2_pos1">
<siteModel id="SiteModel.s:COX2_pos1" spec="SiteModel" gammaCategoryCount="15" proportionInvariant="@proportionInvariant.s:COX2_pos1" shape="@gammaShape.s:COX2_pos1">
<parameter id="mutationRate.s:COX2_pos1" spec="parameter.RealParameter" estimate="false" name="mutationRate">2.93E-5</parameter>
<substModel id="gtr.s:COX2_pos1" spec="GTR" rateAC="@rateAC.s:COX2_pos1" rateAG="@rateAG.s:COX2_pos1" rateAT="@rateAT.s:COX2_pos1" rateCG="@rateCG.s:COX2_pos1" rateGT="@rateGT.s:COX2_pos1">
<parameter id="rateCT.s:COX2_pos1" spec="parameter.RealParameter" estimate="false" lower="0.0" name="rateCT">1.0</parameter>
<frequencies id="estimatedFreqs.s:COX2_pos1" spec="Frequencies" frequencies="@freqParameter.s:COX2_pos1"/>
</substModel>
</siteModel>
<branchRateModel id="StrictClock.c:COX2_pos1" spec="beast.evolution.branchratemodel.StrictClockModel" clock.rate="@clockRate.c:COX2_pos1"/>
</distribution>
<distribution id="treeLikelihood.COX2_pos2" spec="ThreadedTreeLikelihood" data="@COX2_pos2" tree="@Tree.t:COX2_pos2">
<siteModel id="SiteModel.s:COX2_pos2" spec="SiteModel" gammaCategoryCount="15" proportionInvariant="@proportionInvariant.s:COX2_pos2" shape="@gammaShape.s:COX2_pos2">
<parameter id="mutationRate.s:COX2_pos2" spec="parameter.RealParameter" estimate="false" name="mutationRate">2.93E-5</parameter>
<substModel id="gtr.s:COX2_pos2" spec="GTR" rateAC="@rateAC.s:COX2_pos2" rateAG="@rateAG.s:COX2_pos2" rateAT="@rateAT.s:COX2_pos2" rateCG="@rateCG.s:COX2_pos2" rateGT="@rateGT.s:COX2_pos2">
<parameter id="rateCT.s:COX2_pos2" spec="parameter.RealParameter" estimate="false" lower="0.0" name="rateCT">1.0</parameter>
<frequencies id="estimatedFreqs.s:COX2_pos2" spec="Frequencies" frequencies="@freqParameter.s:COX2_pos2"/>
```

```
</substModel>
</siteModel>
<branchRateModel id="StrictClock.c:COX2_pos2"
spec="beast.evolution.branchratemodel.StrictClockModel" clock.rate="@clockRate.c:COX2_pos2"/>
</distribution>
<distribution id="treeLikelihood.COX2_pos3" spec="ThreadedTreeLikelihood" data="@COX2_pos3"
tree="@Tree.t:COX2_pos3">
<siteModel id="SiteModel.s:COX2_pos3" spec="SiteModel" gammaCategoryCount="15"
proportionInvariant="@proportionInvariant.s:COX2_pos3" shape="@gammaShape.s:COX2_pos3">
<parameter id="mutationRate.s:COX2_pos3" spec="parameter.RealParameter" estimate="false"
name="mutationRate">1.0</parameter>
<substModel id="gtr.s:COX2_pos3" spec="GTR" rateAC="@rateAC.s:COX2_pos3"
rateAG="@rateAG.s:COX2_pos3" rateAT="@rateAT.s:COX2_pos3" rateCG="@rateCG.s:COX2_pos3"
rateGT="@rateGT.s:COX2_pos3">
<parameter id="rateCT.s:COX2_pos3" spec="parameter.RealParameter" estimate="false"
lower="0.0" name="rateCT">1.0</parameter>
<frequencies id="estimatedFreqs.s:COX2_pos3" spec="Frequencies"
frequencies="@freqParameter.s:COX2_pos3"/>
</substModel>
</siteModel>
<branchRateModel id="StrictClock.c:COX2_pos3"
spec="beast.evolution.branchratemodel.StrictClockModel" clock.rate="@clockRate.c:COX2_pos3"/>
</distribution>
<distribution id="treeLikelihood.COX3_pos1" spec="ThreadedTreeLikelihood" data="@COX3_pos1"
tree="@Tree.t:COX3_pos1">
<siteModel id="SiteModel.s:COX3_pos1" spec="SiteModel" gammaCategoryCount="15"
proportionInvariant="@proportionInvariant.s:COX3_pos1" shape="@gammaShape.s:COX3_pos1">
<parameter id="mutationRate.s:COX3_pos1" spec="parameter.RealParameter" estimate="false"
name="mutationRate">2.93E-5</parameter>
<substModel id="gtr.s:COX3_pos1" spec="GTR" rateAC="@rateAC.s:COX3_pos1"
rateAG="@rateAG.s:COX3_pos1" rateAT="@rateAT.s:COX3_pos1" rateCG="@rateCG.s:COX3_pos1"
rateGT="@rateGT.s:COX3_pos1">
<parameter id="rateCT.s:COX3_pos1" spec="parameter.RealParameter" estimate="false"
lower="0.0" name="rateCT">1.0</parameter>
<frequencies id="estimatedFreqs.s:COX3_pos1" spec="Frequencies"
frequencies="@freqParameter.s:COX3_pos1"/>
</substModel>
</siteModel>
<branchRateModel id="StrictClock.c:COX3_pos1"
spec="beast.evolution.branchratemodel.StrictClockModel" clock.rate="@clockRate.c:COX3_pos1"/>
</distribution>
<distribution id="treeLikelihood.COX3_pos2" spec="ThreadedTreeLikelihood" data="@COX3_pos2"
tree="@Tree.t:COX3_pos2">
<siteModel id="SiteModel.s:COX3_pos2" spec="SiteModel" gammaCategoryCount="15"
proportionInvariant="@proportionInvariant.s:COX3_pos2" shape="@gammaShape.s:COX3_pos2">
<parameter id="mutationRate.s:COX3_pos2" spec="parameter.RealParameter" estimate="false"
name="mutationRate">2.93E-5</parameter>
<substModel id="gtr.s:COX3_pos2" spec="GTR" rateAC="@rateAC.s:COX3_pos2"
rateAG="@rateAG.s:COX3_pos2" rateAT="@rateAT.s:COX3_pos2" rateCG="@rateCG.s:COX3_pos2"
rateGT="@rateGT.s:COX3_pos2">
<parameter id="rateCT.s:COX3_pos2" spec="parameter.RealParameter" estimate="false"
lower="0.0" name="rateCT">1.0</parameter>
<frequencies id="estimatedFreqs.s:COX3_pos2" spec="Frequencies"
frequencies="@freqParameter.s:COX3_pos2"/>
</substModel>
</siteModel>
<branchRateModel id="StrictClock.c:COX3_pos2"
spec="beast.evolution.branchratemodel.StrictClockModel" clock.rate="@clockRate.c:COX3_pos2"/>
</distribution>
<distribution id="treeLikelihood.COX3_pos3" spec="ThreadedTreeLikelihood" data="@COX3_pos3"
tree="@Tree.t:COX3_pos3">
<siteModel id="SiteModel.s:COX3_pos3" spec="SiteModel" gammaCategoryCount="15"
proportionInvariant="@proportionInvariant.s:COX3_pos3" shape="@gammaShape.s:COX3_pos3">
```

```xml
<parameter id="mutationRate.s:COX3_pos3" spec="parameter.RealParameter" estimate="false" name="mutationRate">1.0</parameter>
<substModel id="gtr.s:COX3_pos3" spec="GTR" rateAC="@rateAC.s:COX3_pos3" rateAG="@rateAG.s:COX3_pos3" rateAT="@rateAT.s:COX3_pos3" rateCG="@rateCG.s:COX3_pos3" rateGT="@rateGT.s:COX3_pos3">
<parameter id="rateCT.s:COX3_pos3" spec="parameter.RealParameter" estimate="false" lower="0.0" name="rateCT">1.0</parameter>
<frequencies id="estimatedFreqs.s:COX3_pos3" spec="Frequencies" frequencies="@freqParameter.s:COX3_pos3"/>
</substModel>
</siteModel>
<branchRateModel id="StrictClock.c:COX3_pos3" spec="beast.evolution.branchratemodel.StrictClockModel" clock.rate="@clockRate.c:COX3_pos3"/>
</distribution>
<distribution id="treeLikelihood.CYTB_pos1" spec="ThreadedTreeLikelihood" data="@CYTB_pos1" tree="@Tree.t:CYTB_pos1">
<siteModel id="SiteModel.s:CYTB_pos1" spec="SiteModel" gammaCategoryCount="15" proportionInvariant="@proportionInvariant.s:CYTB_pos1" shape="@gammaShape.s:CYTB_pos1">
<parameter id="mutationRate.s:CYTB_pos1" spec="parameter.RealParameter" estimate="false" name="mutationRate">2.93E-5</parameter>
<substModel id="gtr.s:CYTB_pos1" spec="GTR" rateAC="@rateAC.s:CYTB_pos1" rateAG="@rateAG.s:CYTB_pos1" rateAT="@rateAT.s:CYTB_pos1" rateCG="@rateCG.s:CYTB_pos1" rateGT="@rateGT.s:CYTB_pos1">
<parameter id="rateCT.s:CYTB_pos1" spec="parameter.RealParameter" estimate="false" lower="0.0" name="rateCT">1.0</parameter>
<frequencies id="estimatedFreqs.s:CYTB_pos1" spec="Frequencies" frequencies="@freqParameter.s:CYTB_pos1"/>
</substModel>
</siteModel>
<branchRateModel id="StrictClock.c:CYTB_pos1" spec="beast.evolution.branchratemodel.StrictClockModel" clock.rate="@clockRate.c:CYTB_pos1"/>
</distribution>
<distribution id="treeLikelihood.CYTB_pos2" spec="ThreadedTreeLikelihood" data="@CYTB_pos2" tree="@Tree.t:CYTB_pos2">
<siteModel id="SiteModel.s:CYTB_pos2" spec="SiteModel" gammaCategoryCount="15" proportionInvariant="@proportionInvariant.s:CYTB_pos2" shape="@gammaShape.s:CYTB_pos2">
<parameter id="mutationRate.s:CYTB_pos2" spec="parameter.RealParameter" estimate="false" name="mutationRate">2.93E-5</parameter>
<substModel id="gtr.s:CYTB_pos2" spec="GTR" rateAC="@rateAC.s:CYTB_pos2" rateAG="@rateAG.s:CYTB_pos2" rateAT="@rateAT.s:CYTB_pos2" rateCG="@rateCG.s:CYTB_pos2" rateGT="@rateGT.s:CYTB_pos2">
<parameter id="rateCT.s:CYTB_pos2" spec="parameter.RealParameter" estimate="false" lower="0.0" name="rateCT">1.0</parameter>
<frequencies id="estimatedFreqs.s:CYTB_pos2" spec="Frequencies" frequencies="@freqParameter.s:CYTB_pos2"/>
</substModel>
</siteModel>
<branchRateModel id="StrictClock.c:CYTB_pos2" spec="beast.evolution.branchratemodel.StrictClockModel" clock.rate="@clockRate.c:CYTB_pos2"/>
</distribution>
<distribution id="treeLikelihood.CYTB_pos3" spec="ThreadedTreeLikelihood" data="@CYTB_pos3" tree="@Tree.t:CYTB_pos3">
<siteModel id="SiteModel.s:CYTB_pos3" spec="SiteModel" gammaCategoryCount="15" proportionInvariant="@proportionInvariant.s:CYTB_pos3" shape="@gammaShape.s:CYTB_pos3">
<parameter id="mutationRate.s:CYTB_pos3" spec="parameter.RealParameter" estimate="false" name="mutationRate">1.0</parameter>
<substModel id="gtr.s:CYTB_pos3" spec="GTR" rateAC="@rateAC.s:CYTB_pos3" rateAG="@rateAG.s:CYTB_pos3" rateAT="@rateAT.s:CYTB_pos3" rateCG="@rateCG.s:CYTB_pos3" rateGT="@rateGT.s:CYTB_pos3">
<parameter id="rateCT.s:CYTB_pos3" spec="parameter.RealParameter" estimate="false" lower="0.0" name="rateCT">1.0</parameter>
<frequencies id="estimatedFreqs.s:CYTB_pos3" spec="Frequencies" frequencies="@freqParameter.s:CYTB_pos3"/>
```

```
                </substModel>
            </siteModel>
            <branchRateModel id="StrictClock.c:CYTB_pos3"
spec="beast.evolution.branchratemodel.StrictClockModel" clock.rate="@clockRate.c:CYTB_pos3"/>
        </distribution>
        <distribution id="treeLikelihood.NADH1_pos1" spec="ThreadedTreeLikelihood" data="@NADH1_pos1"
tree="@Tree.t:NADH1_pos1">
            <siteModel id="SiteModel.s:NADH1_pos1" spec="SiteModel" gammaCategoryCount="15"
proportionInvariant="@proportionInvariant.s:NADH1_pos1" shape="@gammaShape.s:NADH1_pos1">
                <parameter id="mutationRate.s:NADH1_pos1" spec="parameter.RealParameter" estimate="false"
name="mutationRate">2.93E-5</parameter>
                <substModel id="gtr.s:NADH1_pos1" spec="GTR" rateAC="@rateAC.s:NADH1_pos1"
rateAG="@rateAG.s:NADH1_pos1" rateAT="@rateAT.s:NADH1_pos1" rateCG="@rateCG.s:NADH1_pos1"
rateGT="@rateGT.s:NADH1_pos1">
                    <parameter id="rateCT.s:NADH1_pos1" spec="parameter.RealParameter" estimate="false"
lower="0.0" name="rateCT">1.0</parameter>
                    <frequencies id="estimatedFreqs.s:NADH1_pos1" spec="Frequencies"
frequencies="@freqParameter.s:NADH1_pos1"/>
                </substModel>
            </siteModel>
            <branchRateModel id="StrictClock.c:NADH1_pos1"
spec="beast.evolution.branchratemodel.StrictClockModel" clock.rate="@clockRate.c:NADH1_pos1"/>
        </distribution>
        <distribution id="treeLikelihood.NADH1_pos2" spec="ThreadedTreeLikelihood" data="@NADH1_pos2"
tree="@Tree.t:NADH1_pos2">
            <siteModel id="SiteModel.s:NADH1_pos2" spec="SiteModel" gammaCategoryCount="15"
proportionInvariant="@proportionInvariant.s:NADH1_pos2" shape="@gammaShape.s:NADH1_pos2">
                <parameter id="mutationRate.s:NADH1_pos2" spec="parameter.RealParameter" estimate="false"
name="mutationRate">2.93E-5</parameter>
                <substModel id="gtr.s:NADH1_pos2" spec="GTR" rateAC="@rateAC.s:NADH1_pos2"
rateAG="@rateAG.s:NADH1_pos2" rateAT="@rateAT.s:NADH1_pos2" rateCG="@rateCG.s:NADH1_pos2"
rateGT="@rateGT.s:NADH1_pos2">
                    <parameter id="rateCT.s:NADH1_pos2" spec="parameter.RealParameter" estimate="false"
lower="0.0" name="rateCT">1.0</parameter>
                    <frequencies id="estimatedFreqs.s:NADH1_pos2" spec="Frequencies"
frequencies="@freqParameter.s:NADH1_pos2"/>
                </substModel>
            </siteModel>
            <branchRateModel id="StrictClock.c:NADH1_pos2"
spec="beast.evolution.branchratemodel.StrictClockModel" clock.rate="@clockRate.c:NADH1_pos2"/>
        </distribution>
        <distribution id="treeLikelihood.NADH1_pos3" spec="ThreadedTreeLikelihood" data="@NADH1_pos3"
tree="@Tree.t:NADH1_pos3">
            <siteModel id="SiteModel.s:NADH1_pos3" spec="SiteModel" gammaCategoryCount="15"
proportionInvariant="@proportionInvariant.s:NADH1_pos3" shape="@gammaShape.s:NADH1_pos3">
                <parameter id="mutationRate.s:NADH1_pos3" spec="parameter.RealParameter" estimate="false"
name="mutationRate">1.0</parameter>
                <substModel id="gtr.s:NADH1_pos3" spec="GTR" rateAC="@rateAC.s:NADH1_pos3"
rateAG="@rateAG.s:NADH1_pos3" rateAT="@rateAT.s:NADH1_pos3" rateCG="@rateCG.s:NADH1_pos3"
rateGT="@rateGT.s:NADH1_pos3">
                    <parameter id="rateCT.s:NADH1_pos3" spec="parameter.RealParameter" estimate="false"
lower="0.0" name="rateCT">1.0</parameter>
                    <frequencies id="estimatedFreqs.s:NADH1_pos3" spec="Frequencies"
frequencies="@freqParameter.s:NADH1_pos3"/>
                </substModel>
            </siteModel>
            <branchRateModel id="StrictClock.c:NADH1_pos3"
spec="beast.evolution.branchratemodel.StrictClockModel" clock.rate="@clockRate.c:NADH1_pos3"/>
        </distribution>
        <distribution id="treeLikelihood.NADH2_pos1" spec="ThreadedTreeLikelihood" data="@NADH2_pos1"
tree="@Tree.t:NADH2_pos1">
            <siteModel id="SiteModel.s:NADH2_pos1" spec="SiteModel" gammaCategoryCount="15"
proportionInvariant="@proportionInvariant.s:NADH2_pos1" shape="@gammaShape.s:NADH2_pos1">
```

```
<parameter id="mutationRate.s:NADH2_pos1" spec="parameter.RealParameter" estimate="false"
name="mutationRate">2.93E-5</parameter>
<substModel id="gtr.s:NADH2_pos1" spec="GTR" rateAC="@rateAC.s:NADH2_pos1"
rateAG="@rateAG.s:NADH2_pos1" rateAT="@rateAT.s:NADH2_pos1" rateCG="@rateCG.s:NADH2_pos1"
rateGT="@rateGT.s:NADH2_pos1">
<parameter id="rateCT.s:NADH2_pos1" spec="parameter.RealParameter" estimate="false"
lower="0.0" name="rateCT">1.0</parameter>
<frequencies id="estimatedFreqs.s:NADH2_pos1" spec="Frequencies"
frequencies="@freqParameter.s:NADH2_pos1"/>
</substModel>
</siteModel>
<branchRateModel id="StrictClock.c:NADH2_pos1"
spec="beast.evolution.branchratemodel.StrictClockModel" clock.rate="@clockRate.c:NADH2_pos1"/>
</distribution>
<distribution id="treeLikelihood.NADH2_pos2" spec="ThreadedTreeLikelihood" data="@NADH2_pos2"
tree="@Tree.t:NADH2_pos2">
<siteModel id="SiteModel.s:NADH2_pos2" spec="SiteModel" gammaCategoryCount="15"
proportionInvariant="@proportionInvariant.s:NADH2_pos2" shape="@gammaShape.s:NADH2_pos2">
<parameter id="mutationRate.s:NADH2_pos2" spec="parameter.RealParameter" estimate="false"
name="mutationRate">2.93E-5</parameter>
<substModel id="gtr.s:NADH2_pos2" spec="GTR" rateAC="@rateAC.s:NADH2_pos2"
rateAG="@rateAG.s:NADH2_pos2" rateAT="@rateAT.s:NADH2_pos2" rateCG="@rateCG.s:NADH2_pos2"
rateGT="@rateGT.s:NADH2_pos2">
<parameter id="rateCT.s:NADH2_pos2" spec="parameter.RealParameter" estimate="false"
lower="0.0" name="rateCT">1.0</parameter>
<frequencies id="estimatedFreqs.s:NADH2_pos2" spec="Frequencies"
frequencies="@freqParameter.s:NADH2_pos2"/>
</substModel>
</siteModel>
<branchRateModel id="StrictClock.c:NADH2_pos2"
spec="beast.evolution.branchratemodel.StrictClockModel" clock.rate="@clockRate.c:NADH2_pos2"/>
</distribution>
<distribution id="treeLikelihood.NADH2_pos3" spec="ThreadedTreeLikelihood" data="@NADH2_pos3"
tree="@Tree.t:NADH2_pos3">
<siteModel id="SiteModel.s:NADH2_pos3" spec="SiteModel" gammaCategoryCount="15"
proportionInvariant="@proportionInvariant.s:NADH2_pos3" shape="@gammaShape.s:NADH2_pos3">
<parameter id="mutationRate.s:NADH2_pos3" spec="parameter.RealParameter" estimate="false"
name="mutationRate">1.0</parameter>
<substModel id="gtr.s:NADH2_pos3" spec="GTR" rateAC="@rateAC.s:NADH2_pos3"
rateAG="@rateAG.s:NADH2_pos3" rateAT="@rateAT.s:NADH2_pos3" rateCG="@rateCG.s:NADH2_pos3"
rateGT="@rateGT.s:NADH2_pos3">
<parameter id="rateCT.s:NADH2_pos3" spec="parameter.RealParameter" estimate="false"
lower="0.0" name="rateCT">1.0</parameter>
<frequencies id="estimatedFreqs.s:NADH2_pos3" spec="Frequencies"
frequencies="@freqParameter.s:NADH2_pos3"/>
</substModel>
</siteModel>
<branchRateModel id="StrictClock.c:NADH2_pos3"
spec="beast.evolution.branchratemodel.StrictClockModel" clock.rate="@clockRate.c:NADH2_pos3"/>
</distribution>
<distribution id="treeLikelihood.NADH3_pos1" spec="ThreadedTreeLikelihood" data="@NADH3_pos1"
tree="@Tree.t:NADH3_pos1">
<siteModel id="SiteModel.s:NADH3_pos1" spec="SiteModel" gammaCategoryCount="15"
proportionInvariant="@proportionInvariant.s:NADH3_pos1" shape="@gammaShape.s:NADH3_pos1">
<parameter id="mutationRate.s:NADH3_pos1" spec="parameter.RealParameter" estimate="false"
name="mutationRate">2.93E-5</parameter>
<substModel id="gtr.s:NADH3_pos1" spec="GTR" rateAC="@rateAC.s:NADH3_pos1"
rateAG="@rateAG.s:NADH3_pos1" rateAT="@rateAT.s:NADH3_pos1" rateCG="@rateCG.s:NADH3_pos1"
rateGT="@rateGT.s:NADH3_pos1">
<parameter id="rateCT.s:NADH3_pos1" spec="parameter.RealParameter" estimate="false"
lower="0.0" name="rateCT">1.0</parameter>
<frequencies id="estimatedFreqs.s:NADH3_pos1" spec="Frequencies"
frequencies="@freqParameter.s:NADH3_pos1"/>
```

```
            </substModel>
          </siteModel>
          <branchRateModel id="StrictClock.c:NADH3_pos1"
spec="beast.evolution.branchratemodel.StrictClockModel" clock.rate="@clockRate.c:NADH3_pos1"/>
        </distribution>
        <distribution id="treeLikelihood.NADH3_pos2" spec="ThreadedTreeLikelihood" data="@NADH3_pos2"
tree="@Tree.t:NADH3_pos2">
          <siteModel id="SiteModel.s:NADH3_pos2" spec="SiteModel" gammaCategoryCount="15"
proportionInvariant="@proportionInvariant.s:NADH3_pos2" shape="@gammaShape.s:NADH3_pos2">
            <parameter id="mutationRate.s:NADH3_pos2" spec="parameter.RealParameter" estimate="false"
name="mutationRate">2.93E-5</parameter>
            <substModel id="gtr.s:NADH3_pos2" spec="GTR" rateAC="@rateAC.s:NADH3_pos2"
rateAG="@rateAG.s:NADH3_pos2" rateAT="@rateAT.s:NADH3_pos2" rateCG="@rateCG.s:NADH3_pos2"
rateGT="@rateGT.s:NADH3_pos2">
              <parameter id="rateCT.s:NADH3_pos2" spec="parameter.RealParameter" estimate="false"
lower="0.0" name="rateCT">1.0</parameter>
              <frequencies id="estimatedFreqs.s:NADH3_pos2" spec="Frequencies"
frequencies="@freqParameter.s:NADH3_pos2"/>
            </substModel>
          </siteModel>
          <branchRateModel id="StrictClock.c:NADH3_pos2"
spec="beast.evolution.branchratemodel.StrictClockModel" clock.rate="@clockRate.c:NADH3_pos2"/>
        </distribution>
        <distribution id="treeLikelihood.NADH3_pos3" spec="ThreadedTreeLikelihood" data="@NADH3_pos3"
tree="@Tree.t:NADH3_pos3">
          <siteModel id="SiteModel.s:NADH3_pos3" spec="SiteModel" gammaCategoryCount="15"
proportionInvariant="@proportionInvariant.s:NADH3_pos3" shape="@gammaShape.s:NADH3_pos3">
            <parameter id="mutationRate.s:NADH3_pos3" spec="parameter.RealParameter" estimate="false"
name="mutationRate">1.0</parameter>
            <substModel id="gtr.s:NADH3_pos3" spec="GTR" rateAC="@rateAC.s:NADH3_pos3"
rateAG="@rateAG.s:NADH3_pos3" rateAT="@rateAT.s:NADH3_pos3" rateCG="@rateCG.s:NADH3_pos3"
rateGT="@rateGT.s:NADH3_pos3">
              <parameter id="rateCT.s:NADH3_pos3" spec="parameter.RealParameter" estimate="false"
lower="0.0" name="rateCT">1.0</parameter>
              <frequencies id="estimatedFreqs.s:NADH3_pos3" spec="Frequencies"
frequencies="@freqParameter.s:NADH3_pos3"/>
            </substModel>
          </siteModel>
          <branchRateModel id="StrictClock.c:NADH3_pos3"
spec="beast.evolution.branchratemodel.StrictClockModel" clock.rate="@clockRate.c:NADH3_pos3"/>
        </distribution>
        <distribution id="treeLikelihood.NADH4_pos1" spec="ThreadedTreeLikelihood" data="@NADH4_pos1"
tree="@Tree.t:NADH4_pos1">
          <siteModel id="SiteModel.s:NADH4_pos1" spec="SiteModel" gammaCategoryCount="15"
proportionInvariant="@proportionInvariant.s:NADH4_pos1" shape="@gammaShape.s:NADH4_pos1">
            <parameter id="mutationRate.s:NADH4_pos1" spec="parameter.RealParameter" estimate="false"
name="mutationRate">2.93E-5</parameter>
            <substModel id="gtr.s:NADH4_pos1" spec="GTR" rateAC="@rateAC.s:NADH4_pos1"
rateAG="@rateAG.s:NADH4_pos1" rateAT="@rateAT.s:NADH4_pos1" rateCG="@rateCG.s:NADH4_pos1"
rateGT="@rateGT.s:NADH4_pos1">
              <parameter id="rateCT.s:NADH4_pos1" spec="parameter.RealParameter" estimate="false"
lower="0.0" name="rateCT">1.0</parameter>
              <frequencies id="estimatedFreqs.s:NADH4_pos1" spec="Frequencies"
frequencies="@freqParameter.s:NADH4_pos1"/>
            </substModel>
          </siteModel>
          <branchRateModel id="StrictClock.c:NADH4_pos1"
spec="beast.evolution.branchratemodel.StrictClockModel" clock.rate="@clockRate.c:NADH4_pos1"/>
        </distribution>
        <distribution id="treeLikelihood.NADH4_pos2" spec="ThreadedTreeLikelihood" data="@NADH4_pos2"
tree="@Tree.t:NADH4_pos2">
          <siteModel id="SiteModel.s:NADH4_pos2" spec="SiteModel" gammaCategoryCount="15"
proportionInvariant="@proportionInvariant.s:NADH4_pos2" shape="@gammaShape.s:NADH4_pos2">
```

```xml
<parameter id="mutationRate.s:NADH4_pos2" spec="parameter.RealParameter" estimate="false" name="mutationRate">2.93E-5</parameter>
<substModel id="gtr.s:NADH4_pos2" spec="GTR" rateAC="@rateAC.s:NADH4_pos2" rateAG="@rateAG.s:NADH4_pos2" rateAT="@rateAT.s:NADH4_pos2" rateCG="@rateCG.s:NADH4_pos2" rateGT="@rateGT.s:NADH4_pos2">
<parameter id="rateCT.s:NADH4_pos2" spec="parameter.RealParameter" estimate="false" lower="0.0" name="rateCT">1.0</parameter>
<frequencies id="estimatedFreqs.s:NADH4_pos2" spec="Frequencies" frequencies="@freqParameter.s:NADH4_pos2"/>
</substModel>
</siteModel>
<branchRateModel id="StrictClock.c:NADH4_pos2" spec="beast.evolution.branchratemodel.StrictClockModel" clock.rate="@clockRate.c:NADH4_pos2"/>
</distribution>
<distribution id="treeLikelihood.NADH4_pos3" spec="ThreadedTreeLikelihood" data="@NADH4_pos3" tree="@Tree.t:NADH4_pos3">
<siteModel id="SiteModel.s:NADH4_pos3" spec="SiteModel" gammaCategoryCount="15" proportionInvariant="@proportionInvariant.s:NADH4_pos3" shape="@gammaShape.s:NADH4_pos3">
<parameter id="mutationRate.s:NADH4_pos3" spec="parameter.RealParameter" estimate="false" name="mutationRate">1.0</parameter>
<substModel id="gtr.s:NADH4_pos3" spec="GTR" rateAC="@rateAC.s:NADH4_pos3" rateAG="@rateAG.s:NADH4_pos3" rateAT="@rateAT.s:NADH4_pos3" rateCG="@rateCG.s:NADH4_pos3" rateGT="@rateGT.s:NADH4_pos3">
<parameter id="rateCT.s:NADH4_pos3" spec="parameter.RealParameter" estimate="false" lower="0.0" name="rateCT">1.0</parameter>
<frequencies id="estimatedFreqs.s:NADH4_pos3" spec="Frequencies" frequencies="@freqParameter.s:NADH4_pos3"/>
</substModel>
</siteModel>
<branchRateModel id="StrictClock.c:NADH4_pos3" spec="beast.evolution.branchratemodel.StrictClockModel" clock.rate="@clockRate.c:NADH4_pos3"/>
</distribution>
<distribution id="treeLikelihood.NADH4L_pos1" spec="ThreadedTreeLikelihood" data="@NADH4L_pos1" tree="@Tree.t:NADH4L_pos1">
<siteModel id="SiteModel.s:NADH4L_pos1" spec="SiteModel" gammaCategoryCount="15" proportionInvariant="@proportionInvariant.s:NADH4L_pos1" shape="@gammaShape.s:NADH4L_pos1">
<parameter id="mutationRate.s:NADH4L_pos1" spec="parameter.RealParameter" estimate="false" name="mutationRate">2.93E-5</parameter>
<substModel id="gtr.s:NADH4L_pos1" spec="GTR" rateAC="@rateAC.s:NADH4L_pos1" rateAG="@rateAG.s:NADH4L_pos1" rateAT="@rateAT.s:NADH4L_pos1" rateCG="@rateCG.s:NADH4L_pos1" rateGT="@rateGT.s:NADH4L_pos1">
<parameter id="rateCT.s:NADH4L_pos1" spec="parameter.RealParameter" estimate="false" lower="0.0" name="rateCT">1.0</parameter>
<frequencies id="estimatedFreqs.s:NADH4L_pos1" spec="Frequencies" frequencies="@freqParameter.s:NADH4L_pos1"/>
</substModel>
</siteModel>
<branchRateModel id="StrictClock.c:NADH4L_pos1" spec="beast.evolution.branchratemodel.StrictClockModel" clock.rate="@clockRate.c:NADH4L_pos1"/>
</distribution>
<distribution id="treeLikelihood.NADH4L_pos2" spec="ThreadedTreeLikelihood" data="@NADH4L_pos2" tree="@Tree.t:NADH4L_pos2">
<siteModel id="SiteModel.s:NADH4L_pos2" spec="SiteModel" gammaCategoryCount="15" proportionInvariant="@proportionInvariant.s:NADH4L_pos2" shape="@gammaShape.s:NADH4L_pos2">
<parameter id="mutationRate.s:NADH4L_pos2" spec="parameter.RealParameter" estimate="false" name="mutationRate">2.93E-5</parameter>
<substModel id="gtr.s:NADH4L_pos2" spec="GTR" rateAC="@rateAC.s:NADH4L_pos2" rateAG="@rateAG.s:NADH4L_pos2" rateAT="@rateAT.s:NADH4L_pos2" rateCG="@rateCG.s:NADH4L_pos2" rateGT="@rateGT.s:NADH4L_pos2">
<parameter id="rateCT.s:NADH4L_pos2" spec="parameter.RealParameter" estimate="false" lower="0.0" name="rateCT">1.0</parameter>
<frequencies id="estimatedFreqs.s:NADH4L_pos2" spec="Frequencies" frequencies="@freqParameter.s:NADH4L_pos2"/>
```

```
</substModel>
</siteModel>
<branchRateModel id="StrictClock.c:NADH4L_pos2"
spec="beast.evolution.branchratemodel.StrictClockModel" clock.rate="@clockRate.c:NADH4L_pos2"/>
</distribution>
<distribution id="treeLikelihood.NADH4L_pos3" spec="ThreadedTreeLikelihood"
data="@NADH4L_pos3" tree="@Tree.t:NADH4L_pos3">
<siteModel id="SiteModel.s:NADH4L_pos3" spec="SiteModel" gammaCategoryCount="15"
proportionInvariant="@proportionInvariant.s:NADH4L_pos3" shape="@gammaShape.s:NADH4L_pos3">
<parameter id="mutationRate.s:NADH4L_pos3" spec="parameter.RealParameter" estimate="false"
name="mutationRate">1.0</parameter>
<substModel id="gtr.s:NADH4L_pos3" spec="GTR" rateAC="@rateAC.s:NADH4L_pos3"
rateAG="@rateAG.s:NADH4L_pos3" rateAT="@rateAT.s:NADH4L_pos3" rateCG="@rateCG.s:NADH4L_pos3"
rateGT="@rateGT.s:NADH4L_pos3">
<parameter id="rateCT.s:NADH4L_pos3" spec="parameter.RealParameter" estimate="false"
lower="0.0" name="rateCT">1.0</parameter>
<frequencies id="estimatedFreqs.s:NADH4L_pos3" spec="Frequencies"
frequencies="@freqParameter.s:NADH4L_pos3"/>
</substModel>
</siteModel>
<branchRateModel id="StrictClock.c:NADH4L_pos3"
spec="beast.evolution.branchratemodel.StrictClockModel" clock.rate="@clockRate.c:NADH4L_pos3"/>
</distribution>
<distribution id="treeLikelihood.NADH5_pos1" spec="ThreadedTreeLikelihood" data="@NADH5_pos1"
tree="@Tree.t:NADH5_pos1">
<siteModel id="SiteModel.s:NADH5_pos1" spec="SiteModel" gammaCategoryCount="15"
proportionInvariant="@proportionInvariant.s:NADH5_pos1" shape="@gammaShape.s:NADH5_pos1">
<parameter id="mutationRate.s:NADH5_pos1" spec="parameter.RealParameter" estimate="false"
name="mutationRate">2.93E-5</parameter>
<substModel id="gtr.s:NADH5_pos1" spec="GTR" rateAC="@rateAC.s:NADH5_pos1"
rateAG="@rateAG.s:NADH5_pos1" rateAT="@rateAT.s:NADH5_pos1" rateCG="@rateCG.s:NADH5_pos1"
rateGT="@rateGT.s:NADH5_pos1">
<parameter id="rateCT.s:NADH5_pos1" spec="parameter.RealParameter" estimate="false"
lower="0.0" name="rateCT">1.0</parameter>
<frequencies id="estimatedFreqs.s:NADH5_pos1" spec="Frequencies"
frequencies="@freqParameter.s:NADH5_pos1"/>
</substModel>
</siteModel>
<branchRateModel id="StrictClock.c:NADH5_pos1"
spec="beast.evolution.branchratemodel.StrictClockModel" clock.rate="@clockRate.c:NADH5_pos1"/>
</distribution>
<distribution id="treeLikelihood.NADH5_pos2" spec="ThreadedTreeLikelihood" data="@NADH5_pos2"
tree="@Tree.t:NADH5_pos2">
<siteModel id="SiteModel.s:NADH5_pos2" spec="SiteModel" gammaCategoryCount="15"
proportionInvariant="@proportionInvariant.s:NADH5_pos2" shape="@gammaShape.s:NADH5_pos2">
<parameter id="mutationRate.s:NADH5_pos2" spec="parameter.RealParameter" estimate="false"
name="mutationRate">2.93E-5</parameter>
<substModel id="gtr.s:NADH5_pos2" spec="GTR" rateAC="@rateAC.s:NADH5_pos2"
rateAG="@rateAG.s:NADH5_pos2" rateAT="@rateAT.s:NADH5_pos2" rateCG="@rateCG.s:NADH5_pos2"
rateGT="@rateGT.s:NADH5_pos2">
<parameter id="rateCT.s:NADH5_pos2" spec="parameter.RealParameter" estimate="false"
lower="0.0" name="rateCT">1.0</parameter>
<frequencies id="estimatedFreqs.s:NADH5_pos2" spec="Frequencies"
frequencies="@freqParameter.s:NADH5_pos2"/>
</substModel>
</siteModel>
<branchRateModel id="StrictClock.c:NADH5_pos2"
spec="beast.evolution.branchratemodel.StrictClockModel" clock.rate="@clockRate.c:NADH5_pos2"/>
</distribution>
<distribution id="treeLikelihood.NADH5_pos3" spec="ThreadedTreeLikelihood" data="@NADH5_pos3"
tree="@Tree.t:NADH5_pos3">
<siteModel id="SiteModel.s:NADH5_pos3" spec="SiteModel" gammaCategoryCount="15"
proportionInvariant="@proportionInvariant.s:NADH5_pos3" shape="@gammaShape.s:NADH5_pos3">
```

```
<parameter id="mutationRate.s:NADH5_pos3" spec="parameter.RealParameter" estimate="false"
name="mutationRate">1.0</parameter>
<substModel id="gtr.s:NADH5_pos3" spec="GTR" rateAC="@rateAC.s:NADH5_pos3"
rateAG="@rateAG.s:NADH5_pos3" rateAT="@rateAT.s:NADH5_pos3" rateCG="@rateCG.s:NADH5_pos3"
rateGT="@rateGT.s:NADH5_pos3">
<parameter id="rateCT.s:NADH5_pos3" spec="parameter.RealParameter" estimate="false"
lower="0.0" name="rateCT">1.0</parameter>
<frequencies id="estimatedFreqs.s:NADH5_pos3" spec="Frequencies"
frequencies="@freqParameter.s:NADH5_pos3"/>
</substModel>
</siteModel>
<branchRateModel id="StrictClock.c:NADH5_pos3"
spec="beast.evolution.branchratemodel.StrictClockModel" clock.rate="@clockRate.c:NADH5_pos3"/>
</distribution>
<distribution id="treeLikelihood.NADH6_pos1" spec="ThreadedTreeLikelihood" data="@NADH6_pos1"
tree="@Tree.t:NADH6_pos1">
<siteModel id="SiteModel.s:NADH6_pos1" spec="SiteModel" gammaCategoryCount="15"
proportionInvariant="@proportionInvariant.s:NADH6_pos1" shape="@gammaShape.s:NADH6_pos1">
<parameter id="mutationRate.s:NADH6_pos1" spec="parameter.RealParameter" estimate="false"
name="mutationRate">2.93E-5</parameter>
<substModel id="gtr.s:NADH6_pos1" spec="GTR" rateAC="@rateAC.s:NADH6_pos1"
rateAG="@rateAG.s:NADH6_pos1" rateAT="@rateAT.s:NADH6_pos1" rateCG="@rateCG.s:NADH6_pos1"
rateGT="@rateGT.s:NADH6_pos1">
<parameter id="rateCT.s:NADH6_pos1" spec="parameter.RealParameter" estimate="false"
lower="0.0" name="rateCT">1.0</parameter>
<frequencies id="estimatedFreqs.s:NADH6_pos1" spec="Frequencies"
frequencies="@freqParameter.s:NADH6_pos1"/>
</substModel>
</siteModel>
<branchRateModel id="StrictClock.c:NADH6_pos1"
spec="beast.evolution.branchratemodel.StrictClockModel" clock.rate="@clockRate.c:NADH6_pos1"/>
</distribution>
<distribution id="treeLikelihood.NADH6_pos2" spec="ThreadedTreeLikelihood" data="@NADH6_pos2"
tree="@Tree.t:NADH6_pos2">
<siteModel id="SiteModel.s:NADH6_pos2" spec="SiteModel" gammaCategoryCount="15"
proportionInvariant="@proportionInvariant.s:NADH6_pos2" shape="@gammaShape.s:NADH6_pos2">
<parameter id="mutationRate.s:NADH6_pos2" spec="parameter.RealParameter" estimate="false"
name="mutationRate">2.93E-5</parameter>
<substModel id="gtr.s:NADH6_pos2" spec="GTR" rateAC="@rateAC.s:NADH6_pos2"
rateAG="@rateAG.s:NADH6_pos2" rateAT="@rateAT.s:NADH6_pos2" rateCG="@rateCG.s:NADH6_pos2"
rateGT="@rateGT.s:NADH6_pos2">
<parameter id="rateCT.s:NADH6_pos2" spec="parameter.RealParameter" estimate="false"
lower="0.0" name="rateCT">1.0</parameter>
<frequencies id="estimatedFreqs.s:NADH6_pos2" spec="Frequencies"
frequencies="@freqParameter.s:NADH6_pos2"/>
</substModel>
</siteModel>
<branchRateModel id="StrictClock.c:NADH6_pos2"
spec="beast.evolution.branchratemodel.StrictClockModel" clock.rate="@clockRate.c:NADH6_pos2"/>
</distribution>
<distribution id="treeLikelihood.NADH6_pos3" spec="ThreadedTreeLikelihood" data="@NADH6_pos3"
tree="@Tree.t:NADH6_pos3">
<siteModel id="SiteModel.s:NADH6_pos3" spec="SiteModel" gammaCategoryCount="15"
proportionInvariant="@proportionInvariant.s:NADH6_pos3" shape="@gammaShape.s:NADH6_pos3">
<parameter id="mutationRate.s:NADH6_pos3" spec="parameter.RealParameter" estimate="false"
name="mutationRate">1.0</parameter>
<substModel id="gtr.s:NADH6_pos3" spec="GTR" rateAC="@rateAC.s:NADH6_pos3"
rateAG="@rateAG.s:NADH6_pos3" rateAT="@rateAT.s:NADH6_pos3" rateCG="@rateCG.s:NADH6_pos3"
rateGT="@rateGT.s:NADH6_pos3">
<parameter id="rateCT.s:NADH6_pos3" spec="parameter.RealParameter" estimate="false"
lower="0.0" name="rateCT">1.0</parameter>
<frequencies id="estimatedFreqs.s:NADH6_pos3" spec="Frequencies"
frequencies="@freqParameter.s:NADH6_pos3"/>
```

```
                </substModel>
            </siteModel>
            <branchRateModel id="StrictClock.c:NADH6_pos3"
spec="beast.evolution.branchratemodel.StrictClockModel" clock.rate="@clockRate.c:NADH6_pos3"/>
        </distribution>
        <distribution id="treeLikelihood.Mytilidae27sq12PCGs" spec="ThreadedTreeLikelihood"
data="@Mytilidae27sq12PCGs" tree="@Tree.t:Mytilidae27sq12PCGs">
            <siteModel id="SiteModel.s:Mytilidae27sq12PCGs" spec="SiteModel" gammaCategoryCount="15"
proportionInvariant="@proportionInvariant.s:Mytilidae27sq12PCGs"
shape="@gammaShape.s:Mytilidae27sq12PCGs">
                <parameter id="mutationRate.s:Mytilidae27sq12PCGs" spec="parameter.RealParameter"
estimate="false" name="mutationRate">2.93E-5</parameter>
                <substModel id="gtr.s:Mytilidae27sq12PCGs" spec="GTR"
rateAC="@rateAC.s:Mytilidae27sq12PCGs" rateAG="@rateAG.s:Mytilidae27sq12PCGs"
rateAT="@rateAT.s:Mytilidae27sq12PCGs" rateCG="@rateCG.s:Mytilidae27sq12PCGs"
rateGT="@rateGT.s:Mytilidae27sq12PCGs">
                    <parameter id="rateCT.s:Mytilidae27sq12PCGs" spec="parameter.RealParameter" estimate="false"
lower="0.0" name="rateCT">1.0</parameter>
                    <frequencies id="estimatedFreqs.s:Mytilidae27sq12PCGs" spec="Frequencies"
frequencies="@freqParameter.s:Mytilidae27sq12PCGs"/>
                </substModel>
            </siteModel>
            <branchRateModel id="StrictClock.c:Mytilidae27sq12PCGs"
spec="beast.evolution.branchratemodel.StrictClockModel" clock.rate="@clockRate.c:Mytilidae27sq12PCGs"/>
        </distribution>
    </distribution>
</distribution>

<operator id="StrictClockRateScaler.c:COX2_pos1" spec="ScaleOperator"
parameter="@clockRate.c:COX2_pos1" weight="3.0"/>

<operator id="strictClockUpDownOperator.c:COX2_pos1" spec="UpDownOperator" scaleFactor="0.75"
weight="3.0">
    <up idref="clockRate.c:COX2_pos1"/>
    <down idref="Tree.t:COX2_pos1"/>
</operator>

<operator id="StrictClockRateScaler.c:COX3_pos1" spec="ScaleOperator"
parameter="@clockRate.c:COX3_pos1" weight="3.0"/>

<operator id="strictClockUpDownOperator.c:COX3_pos1" spec="UpDownOperator" scaleFactor="0.75"
weight="3.0">
    <up idref="clockRate.c:COX3_pos1"/>
    <down idref="Tree.t:COX3_pos1"/>
</operator>

<operator id="StrictClockRateScaler.c:NADH2_pos2" spec="ScaleOperator"
parameter="@clockRate.c:NADH2_pos2" weight="3.0"/>

<operator id="strictClockUpDownOperator.c:NADH2_pos2" spec="UpDownOperator" scaleFactor="0.75"
weight="3.0">
    <up idref="clockRate.c:NADH2_pos2"/>
    <down idref="Tree.t:NADH2_pos2"/>
</operator>

<operator id="StrictClockRateScaler.c:NADH4_pos3" spec="ScaleOperator"
parameter="@clockRate.c:NADH4_pos3" weight="3.0"/>

<operator id="strictClockUpDownOperator.c:NADH4_pos3" spec="UpDownOperator" scaleFactor="0.75"
weight="3.0">
    <up idref="clockRate.c:NADH4_pos3"/>
    <down idref="Tree.t:NADH4_pos3"/>
</operator>
```

```
    <operator id="StrictClockRateScaler.c:NADH4L_pos1" spec="ScaleOperator"
parameter="@clockRate.c:NADH4L_pos1" weight="3.0"/>

    <operator id="strictClockUpDownOperator.c:NADH4L_pos1" spec="UpDownOperator" scaleFactor="0.75"
weight="3.0">
        <up idref="clockRate.c:NADH4L_pos1"/>
        <down idref="Tree.t:NADH4L_pos1"/>
    </operator>

    <operator id="StrictClockRateScaler.c:NADH5_pos2" spec="ScaleOperator"
parameter="@clockRate.c:NADH5_pos2" weight="3.0"/>

    <operator id="strictClockUpDownOperator.c:NADH5_pos2" spec="UpDownOperator" scaleFactor="0.75"
weight="3.0">
        <up idref="clockRate.c:NADH5_pos2"/>
        <down idref="Tree.t:NADH5_pos2"/>
    </operator>

    <operator id="StrictClockRateScaler.c:NADH6_pos3" spec="ScaleOperator"
parameter="@clockRate.c:NADH6_pos3" weight="3.0"/>

    <operator id="strictClockUpDownOperator.c:NADH6_pos3" spec="UpDownOperator" scaleFactor="0.75"
weight="3.0">
        <up idref="clockRate.c:NADH6_pos3"/>
        <down idref="Tree.t:NADH6_pos3"/>
    </operator>

    <operator id="StrictClockRateScaler.c:COX1_pos1" spec="ScaleOperator"
parameter="@clockRate.c:COX1_pos1" weight="3.0"/>

    <operator id="strictClockUpDownOperator.c:COX1_pos1" spec="UpDownOperator" scaleFactor="0.75"
weight="3.0">
        <up idref="clockRate.c:COX1_pos1"/>
        <down idref="Tree.t:COX1_pos1"/>
    </operator>

    <operator id="StrictClockRateScaler.c:COX1_pos3" spec="ScaleOperator"
parameter="@clockRate.c:COX1_pos3" weight="3.0"/>

    <operator id="strictClockUpDownOperator.c:COX1_pos3" spec="UpDownOperator" scaleFactor="0.75"
weight="3.0">
        <up idref="clockRate.c:COX1_pos3"/>
        <down idref="Tree.t:COX1_pos3"/>
    </operator>

    <operator id="StrictClockRateScaler.c:Mytilidae27sq12PCGs" spec="ScaleOperator"
parameter="@clockRate.c:Mytilidae27sq12PCGs" weight="3.0"/>

    <operator id="strictClockUpDownOperator.c:Mytilidae27sq12PCGs" spec="UpDownOperator"
scaleFactor="0.75" weight="3.0">
        <up idref="clockRate.c:Mytilidae27sq12PCGs"/>
        <down idref="Tree.t:Mytilidae27sq12PCGs"/>
    </operator>

    <operator id="StrictClockRateScaler.c:atp6_pos1" spec="ScaleOperator" parameter="@clockRate.c:atp6_pos1"
weight="3.0"/>

    <operator id="strictClockUpDownOperator.c:atp6_pos1" spec="UpDownOperator" scaleFactor="0.75"
weight="3.0">
        <up idref="clockRate.c:atp6_pos1"/>
        <down idref="Tree.t:atp6_pos1"/>
    </operator>
```

```
    <operator id="StrictClockRateScaler.c:NADH4_pos2" spec="ScaleOperator"
parameter="@clockRate.c:NADH4_pos2" weight="3.0"/>

    <operator id="strictClockUpDownOperator.c:NADH4_pos2" spec="UpDownOperator" scaleFactor="0.75"
weight="3.0">
        <up idref="clockRate.c:NADH4_pos2"/>
        <down idref="Tree.t:NADH4_pos2"/>
    </operator>

    <operator id="StrictClockRateScaler.c:NADH1_pos1" spec="ScaleOperator"
parameter="@clockRate.c:NADH1_pos1" weight="3.0"/>

    <operator id="strictClockUpDownOperator.c:NADH1_pos1" spec="UpDownOperator" scaleFactor="0.75"
weight="3.0">
        <up idref="clockRate.c:NADH1_pos1"/>
        <down idref="Tree.t:NADH1_pos1"/>
    </operator>

    <operator id="StrictClockRateScaler.c:NADH6_pos1" spec="ScaleOperator"
parameter="@clockRate.c:NADH6_pos1" weight="3.0"/>

    <operator id="strictClockUpDownOperator.c:NADH6_pos1" spec="UpDownOperator" scaleFactor="0.75"
weight="3.0">
        <up idref="clockRate.c:NADH6_pos1"/>
        <down idref="Tree.t:NADH6_pos1"/>
    </operator>

    <operator id="StrictClockRateScaler.c:COX1_pos2" spec="ScaleOperator"
parameter="@clockRate.c:COX1_pos2" weight="3.0"/>

    <operator id="strictClockUpDownOperator.c:COX1_pos2" spec="UpDownOperator" scaleFactor="0.75"
weight="3.0">
        <up idref="clockRate.c:COX1_pos2"/>
        <down idref="Tree.t:COX1_pos2"/>
    </operator>

    <operator id="StrictClockRateScaler.c:NADH4L_pos3" spec="ScaleOperator"
parameter="@clockRate.c:NADH4L_pos3" weight="3.0"/>

    <operator id="strictClockUpDownOperator.c:NADH4L_pos3" spec="UpDownOperator" scaleFactor="0.75"
weight="3.0">
        <up idref="clockRate.c:NADH4L_pos3"/>
        <down idref="Tree.t:NADH4L_pos3"/>
    </operator>

    <operator id="StrictClockRateScaler.c:COX2_pos3" spec="ScaleOperator"
parameter="@clockRate.c:COX2_pos3" weight="3.0"/>

    <operator id="strictClockUpDownOperator.c:COX2_pos3" spec="UpDownOperator" scaleFactor="0.75"
weight="3.0">
        <up idref="clockRate.c:COX2_pos3"/>
        <down idref="Tree.t:COX2_pos3"/>
    </operator>

    <operator id="StrictClockRateScaler.c:COX3_pos2" spec="ScaleOperator"
parameter="@clockRate.c:COX3_pos2" weight="3.0"/>

    <operator id="strictClockUpDownOperator.c:COX3_pos2" spec="UpDownOperator" scaleFactor="0.75"
weight="3.0">
        <up idref="clockRate.c:COX3_pos2"/>
        <down idref="Tree.t:COX3_pos2"/>
    </operator>
```

```
    <operator id="StrictClockRateScaler.c:CYTB_pos2" spec="ScaleOperator"
parameter="@clockRate.c:CYTB_pos2" weight="3.0"/>

    <operator id="strictClockUpDownOperator.c:CYTB_pos2" spec="UpDownOperator" scaleFactor="0.75"
weight="3.0">
        <up idref="clockRate.c:CYTB_pos2"/>
        <down idref="Tree.t:CYTB_pos2"/>
    </operator>

    <operator id="StrictClockRateScaler.c:atp6_pos2" spec="ScaleOperator" parameter="@clockRate.c:atp6_pos2"
weight="3.0"/>

    <operator id="strictClockUpDownOperator.c:atp6_pos2" spec="UpDownOperator" scaleFactor="0.75"
weight="3.0">
        <up idref="clockRate.c:atp6_pos2"/>
        <down idref="Tree.t:atp6_pos2"/>
    </operator>

    <operator id="StrictClockRateScaler.c:COX3_pos3" spec="ScaleOperator"
parameter="@clockRate.c:COX3_pos3" weight="3.0"/>

    <operator id="strictClockUpDownOperator.c:COX3_pos3" spec="UpDownOperator" scaleFactor="0.75"
weight="3.0">
        <up idref="clockRate.c:COX3_pos3"/>
        <down idref="Tree.t:COX3_pos3"/>
    </operator>

    <operator id="StrictClockRateScaler.c:CYTB_pos3" spec="ScaleOperator"
parameter="@clockRate.c:CYTB_pos3" weight="3.0"/>

    <operator id="strictClockUpDownOperator.c:CYTB_pos3" spec="UpDownOperator" scaleFactor="0.75"
weight="3.0">
        <up idref="clockRate.c:CYTB_pos3"/>
        <down idref="Tree.t:CYTB_pos3"/>
    </operator>

    <operator id="StrictClockRateScaler.c:NADH4L_pos2" spec="ScaleOperator"
parameter="@clockRate.c:NADH4L_pos2" weight="3.0"/>

    <operator id="strictClockUpDownOperator.c:NADH4L_pos2" spec="UpDownOperator" scaleFactor="0.75"
weight="3.0">
        <up idref="clockRate.c:NADH4L_pos2"/>
        <down idref="Tree.t:NADH4L_pos2"/>
    </operator>

    <operator id="StrictClockRateScaler.c:NADH6_pos2" spec="ScaleOperator"
parameter="@clockRate.c:NADH6_pos2" weight="3.0"/>

    <operator id="strictClockUpDownOperator.c:NADH6_pos2" spec="UpDownOperator" scaleFactor="0.75"
weight="3.0">
        <up idref="clockRate.c:NADH6_pos2"/>
        <down idref="Tree.t:NADH6_pos2"/>
    </operator>

    <operator id="StrictClockRateScaler.c:NADH1_pos2" spec="ScaleOperator"
parameter="@clockRate.c:NADH1_pos2" weight="3.0"/>

    <operator id="strictClockUpDownOperator.c:NADH1_pos2" spec="UpDownOperator" scaleFactor="0.75"
weight="3.0">
        <up idref="clockRate.c:NADH1_pos2"/>
        <down idref="Tree.t:NADH1_pos2"/>
    </operator>
```

<operator id="StrictClockRateScaler.c:NADH4_pos1" spec="ScaleOperator"
parameter="@clockRate.c:NADH4_pos1" weight="3.0"/>

<operator id="strictClockUpDownOperator.c:NADH4_pos1" spec="UpDownOperator" scaleFactor="0.75"
weight="3.0">
<up idref="clockRate.c:NADH4_pos1"/>
<down idref="Tree.t:NADH4_pos1"/>
</operator>

<operator id="StrictClockRateScaler.c:atp6_pos3" spec="ScaleOperator" parameter="@clockRate.c:atp6_pos3"
weight="3.0"/>

<operator id="strictClockUpDownOperator.c:atp6_pos3" spec="UpDownOperator" scaleFactor="0.75"
weight="3.0">
<up idref="clockRate.c:atp6_pos3"/>
<down idref="Tree.t:atp6_pos3"/>
</operator>

<operator id="StrictClockRateScaler.c:COX2_pos2" spec="ScaleOperator"
parameter="@clockRate.c:COX2_pos2" weight="3.0"/>

<operator id="strictClockUpDownOperator.c:COX2_pos2" spec="UpDownOperator" scaleFactor="0.75"
weight="3.0">
<up idref="clockRate.c:COX2_pos2"/>
<down idref="Tree.t:COX2_pos2"/>
</operator>

<operator id="StrictClockRateScaler.c:NADH2_pos1" spec="ScaleOperator"
parameter="@clockRate.c:NADH2_pos1" weight="3.0"/>

<operator id="strictClockUpDownOperator.c:NADH2_pos1" spec="UpDownOperator" scaleFactor="0.75"
weight="3.0">
<up idref="clockRate.c:NADH2_pos1"/>
<down idref="Tree.t:NADH2_pos1"/>
</operator>

<operator id="StrictClockRateScaler.c:NADH5_pos3" spec="ScaleOperator"
parameter="@clockRate.c:NADH5_pos3" weight="3.0"/>

<operator id="strictClockUpDownOperator.c:NADH5_pos3" spec="UpDownOperator" scaleFactor="0.75"
weight="3.0">
<up idref="clockRate.c:NADH5_pos3"/>
<down idref="Tree.t:NADH5_pos3"/>
</operator>

<operator id="StrictClockRateScaler.c:NADH5_pos1" spec="ScaleOperator"
parameter="@clockRate.c:NADH5_pos1" weight="3.0"/>

<operator id="strictClockUpDownOperator.c:NADH5_pos1" spec="UpDownOperator" scaleFactor="0.75"
weight="3.0">
<up idref="clockRate.c:NADH5_pos1"/>
<down idref="Tree.t:NADH5_pos1"/>
</operator>

<operator id="StrictClockRateScaler.c:NADH3_pos3" spec="ScaleOperator"
parameter="@clockRate.c:NADH3_pos3" weight="3.0"/>

<operator id="strictClockUpDownOperator.c:NADH3_pos3" spec="UpDownOperator" scaleFactor="0.75"
weight="3.0">
<up idref="clockRate.c:NADH3_pos3"/>
<down idref="Tree.t:NADH3_pos3"/>
</operator>

```
    <operator id="StrictClockRateScaler.c:NADH2_pos3" spec="ScaleOperator"
parameter="@clockRate.c:NADH2_pos3" weight="3.0"/>

    <operator id="strictClockUpDownOperator.c:NADH2_pos3" spec="UpDownOperator" scaleFactor="0.75"
weight="3.0">
        <up idref="clockRate.c:NADH2_pos3"/>
        <down idref="Tree.t:NADH2_pos3"/>
    </operator>

    <operator id="StrictClockRateScaler.c:NADH3_pos2" spec="ScaleOperator"
parameter="@clockRate.c:NADH3_pos2" weight="3.0"/>

    <operator id="strictClockUpDownOperator.c:NADH3_pos2" spec="UpDownOperator" scaleFactor="0.75"
weight="3.0">
        <up idref="clockRate.c:NADH3_pos2"/>
        <down idref="Tree.t:NADH3_pos2"/>
    </operator>

    <operator id="StrictClockRateScaler.c:NADH3_pos1" spec="ScaleOperator"
parameter="@clockRate.c:NADH3_pos1" weight="3.0"/>

    <operator id="strictClockUpDownOperator.c:NADH3_pos1" spec="UpDownOperator" scaleFactor="0.75"
weight="3.0">
        <up idref="clockRate.c:NADH3_pos1"/>
        <down idref="Tree.t:NADH3_pos1"/>
    </operator>

    <operator id="StrictClockRateScaler.c:CYTB_pos1" spec="ScaleOperator"
parameter="@clockRate.c:CYTB_pos1" weight="3.0"/>

    <operator id="strictClockUpDownOperator.c:CYTB_pos1" spec="UpDownOperator" scaleFactor="0.75"
weight="3.0">
        <up idref="clockRate.c:CYTB_pos1"/>
        <down idref="Tree.t:CYTB_pos1"/>
    </operator>

    <operator id="StrictClockRateScaler.c:NADH1_pos3" spec="ScaleOperator"
parameter="@clockRate.c:NADH1_pos3" weight="3.0"/>

    <operator id="strictClockUpDownOperator.c:NADH1_pos3" spec="UpDownOperator" scaleFactor="0.75"
weight="3.0">
        <up idref="clockRate.c:NADH1_pos3"/>
        <down idref="Tree.t:NADH1_pos3"/>
    </operator>

    <operator id="proportionInvariantScaler.s:atp6_pos1" spec="ScaleOperator"
parameter="@proportionInvariant.s:atp6_pos1" scaleFactor="0.5" weight="0.1"/>

    <operator id="gammaShapeScaler.s:atp6_pos1" spec="ScaleOperator" parameter="@gammaShape.s:atp6_pos1"
scaleFactor="0.5" weight="0.1"/>

    <operator id="RateACScaler.s:atp6_pos1" spec="ScaleOperator" parameter="@rateAC.s:atp6_pos1"
scaleFactor="0.5" weight="0.1"/>

    <operator id="RateAGScaler.s:atp6_pos1" spec="ScaleOperator" parameter="@rateAG.s:atp6_pos1"
scaleFactor="0.5" weight="0.1"/>

    <operator id="RateATScaler.s:atp6_pos1" spec="ScaleOperator" parameter="@rateAT.s:atp6_pos1"
scaleFactor="0.5" weight="0.1"/>

    <operator id="RateCGScaler.s:atp6_pos1" spec="ScaleOperator" parameter="@rateCG.s:atp6_pos1"
scaleFactor="0.5" weight="0.1"/>
```

<operator id="RateGTScaler.s:atp6_pos1" spec="ScaleOperator" parameter="@rateGT.s:atp6_pos1" scaleFactor="0.5" weight="0.1"/>

<operator id="RateACScaler.s:atp6_pos2" spec="ScaleOperator" parameter="@rateAC.s:atp6_pos2" scaleFactor="0.5" weight="0.1"/>

<operator id="RateAGScaler.s:atp6_pos2" spec="ScaleOperator" parameter="@rateAG.s:atp6_pos2" scaleFactor="0.5" weight="0.1"/>

<operator id="RateATScaler.s:atp6_pos2" spec="ScaleOperator" parameter="@rateAT.s:atp6_pos2" scaleFactor="0.5" weight="0.1"/>

<operator id="RateCGScaler.s:atp6_pos2" spec="ScaleOperator" parameter="@rateCG.s:atp6_pos2" scaleFactor="0.5" weight="0.1"/>

<operator id="RateGTScaler.s:atp6_pos2" spec="ScaleOperator" parameter="@rateGT.s:atp6_pos2" scaleFactor="0.5" weight="0.1"/>

<operator id="proportionInvariantScaler.s:atp6_pos2" spec="ScaleOperator" parameter="@proportionInvariant.s:atp6_pos2" scaleFactor="0.5" weight="0.1"/>

<operator id="gammaShapeScaler.s:atp6_pos2" spec="ScaleOperator" parameter="@gammaShape.s:atp6_pos2" scaleFactor="0.5" weight="0.1"/>

<operator id="gammaShapeScaler.s:atp6_pos3" spec="ScaleOperator" parameter="@gammaShape.s:atp6_pos3" scaleFactor="0.5" weight="0.1"/>

<operator id="RateACScaler.s:atp6_pos3" spec="ScaleOperator" parameter="@rateAC.s:atp6_pos3" scaleFactor="0.5" weight="0.1"/>

<operator id="RateAGScaler.s:atp6_pos3" spec="ScaleOperator" parameter="@rateAG.s:atp6_pos3" scaleFactor="0.5" weight="0.1"/>

<operator id="RateATScaler.s:atp6_pos3" spec="ScaleOperator" parameter="@rateAT.s:atp6_pos3" scaleFactor="0.5" weight="0.1"/>

<operator id="RateCGScaler.s:atp6_pos3" spec="ScaleOperator" parameter="@rateCG.s:atp6_pos3" scaleFactor="0.5" weight="0.1"/>

<operator id="RateGTScaler.s:atp6_pos3" spec="ScaleOperator" parameter="@rateGT.s:atp6_pos3" scaleFactor="0.5" weight="0.1"/>

<operator id="gammaShapeScaler.s:Mytilidae27sq12PCGs" spec="ScaleOperator" parameter="@gammaShape.s:Mytilidae27sq12PCGs" scaleFactor="0.5" weight="0.1"/>

<operator id="gammaShapeScaler.s:COX1_pos1" spec="ScaleOperator" parameter="@gammaShape.s:COX1_pos1" scaleFactor="0.5" weight="0.1"/>

<operator id="RateACScaler.s:COX1_pos1" spec="ScaleOperator" parameter="@rateAC.s:COX1_pos1" scaleFactor="0.5" weight="0.1"/>

<operator id="RateAGScaler.s:COX1_pos1" spec="ScaleOperator" parameter="@rateAG.s:COX1_pos1" scaleFactor="0.5" weight="0.1"/>

<operator id="RateATScaler.s:COX1_pos1" spec="ScaleOperator" parameter="@rateAT.s:COX1_pos1" scaleFactor="0.5" weight="0.1"/>

<operator id="RateCGScaler.s:COX1_pos1" spec="ScaleOperator" parameter="@rateCG.s:COX1_pos1" scaleFactor="0.5" weight="0.1"/>

<operator id="RateGTScaler.s:COX1_pos1" spec="ScaleOperator" parameter="@rateGT.s:COX1_pos1" scaleFactor="0.5" weight="0.1"/>

<operator id="gammaShapeScaler.s:COX1_pos2" spec="ScaleOperator" parameter="@gammaShape.s:COX1_pos2" scaleFactor="0.5" weight="0.1"/>

<operator id="gammaShapeScaler.s:COX1_pos3" spec="ScaleOperator" parameter="@gammaShape.s:COX1_pos3" scaleFactor="0.5" weight="0.1"/>

<operator id="RateACScaler.s:COX1_pos3" spec="ScaleOperator" parameter="@rateAC.s:COX1_pos3" scaleFactor="0.5" weight="0.1"/>

<operator id="RateAGScaler.s:COX1_pos3" spec="ScaleOperator" parameter="@rateAG.s:COX1_pos3" scaleFactor="0.5" weight="0.1"/>

<operator id="RateATScaler.s:COX1_pos3" spec="ScaleOperator" parameter="@rateAT.s:COX1_pos3" scaleFactor="0.5" weight="0.1"/>

<operator id="RateCGScaler.s:COX1_pos3" spec="ScaleOperator" parameter="@rateCG.s:COX1_pos3" scaleFactor="0.5" weight="0.1"/>

<operator id="RateGTScaler.s:COX1_pos3" spec="ScaleOperator" parameter="@rateGT.s:COX1_pos3" scaleFactor="0.5" weight="0.1"/>

<operator id="gammaShapeScaler.s:COX2_pos1" spec="ScaleOperator" parameter="@gammaShape.s:COX2_pos1" scaleFactor="0.5" weight="0.1"/>

<operator id="RateACScaler.s:COX2_pos1" spec="ScaleOperator" parameter="@rateAC.s:COX2_pos1" scaleFactor="0.5" weight="0.1"/>

<operator id="RateAGScaler.s:COX2_pos1" spec="ScaleOperator" parameter="@rateAG.s:COX2_pos1" scaleFactor="0.5" weight="0.1"/>

<operator id="RateATScaler.s:COX2_pos1" spec="ScaleOperator" parameter="@rateAT.s:COX2_pos1" scaleFactor="0.5" weight="0.1"/>

<operator id="RateCGScaler.s:COX2_pos1" spec="ScaleOperator" parameter="@rateCG.s:COX2_pos1" scaleFactor="0.5" weight="0.1"/>

<operator id="RateGTScaler.s:COX2_pos1" spec="ScaleOperator" parameter="@rateGT.s:COX2_pos1" scaleFactor="0.5" weight="0.1"/>

<operator id="gammaShapeScaler.s:COX2_pos2" spec="ScaleOperator" parameter="@gammaShape.s:COX2_pos2" scaleFactor="0.5" weight="0.1"/>

<operator id="RateACScaler.s:COX2_pos3" spec="ScaleOperator" parameter="@rateAC.s:COX2_pos3" scaleFactor="0.5" weight="0.1"/>

<operator id="RateAGScaler.s:COX2_pos3" spec="ScaleOperator" parameter="@rateAG.s:COX2_pos3" scaleFactor="0.5" weight="0.1"/>

<operator id="RateATScaler.s:COX2_pos3" spec="ScaleOperator" parameter="@rateAT.s:COX2_pos3" scaleFactor="0.5" weight="0.1"/>

<operator id="RateCGScaler.s:COX2_pos3" spec="ScaleOperator" parameter="@rateCG.s:COX2_pos3" scaleFactor="0.5" weight="0.1"/>

<operator id="RateGTScaler.s:COX2_pos3" spec="ScaleOperator" parameter="@rateGT.s:COX2_pos3" scaleFactor="0.5" weight="0.1"/>

<operator id="gammaShapeScaler.s:COX2_pos3" spec="ScaleOperator" parameter="@gammaShape.s:COX2_pos3" scaleFactor="0.5" weight="0.1"/>

<operator id="gammaShapeScaler.s:COX3_pos1" spec="ScaleOperator" parameter="@gammaShape.s:COX3_pos1" scaleFactor="0.5" weight="0.1"/>

<operator id="RateACScaler.s:COX3_pos1" spec="ScaleOperator" parameter="@rateAC.s:COX3_pos1" scaleFactor="0.5" weight="0.1"/>

<operator id="RateAGScaler.s:COX3_pos1" spec="ScaleOperator" parameter="@rateAG.s:COX3_pos1" scaleFactor="0.5" weight="0.1"/>

<operator id="RateATScaler.s:COX3_pos1" spec="ScaleOperator" parameter="@rateAT.s:COX3_pos1" scaleFactor="0.5" weight="0.1"/>

<operator id="RateCGScaler.s:COX3_pos1" spec="ScaleOperator" parameter="@rateCG.s:COX3_pos1" scaleFactor="0.5" weight="0.1"/>

<operator id="RateGTScaler.s:COX3_pos1" spec="ScaleOperator" parameter="@rateGT.s:COX3_pos1" scaleFactor="0.5" weight="0.1"/>

<operator id="gammaShapeScaler.s:COX3_pos2" spec="ScaleOperator" parameter="@gammaShape.s:COX3_pos2" scaleFactor="0.5" weight="0.1"/>

<operator id="RateACScaler.s:COX3_pos2" spec="ScaleOperator" parameter="@rateAC.s:COX3_pos2" scaleFactor="0.5" weight="0.1"/>

<operator id="RateAGScaler.s:COX3_pos2" spec="ScaleOperator" parameter="@rateAG.s:COX3_pos2" scaleFactor="0.5" weight="0.1"/>

<operator id="RateATScaler.s:COX3_pos2" spec="ScaleOperator" parameter="@rateAT.s:COX3_pos2" scaleFactor="0.5" weight="0.1"/>

<operator id="RateCGScaler.s:COX3_pos2" spec="ScaleOperator" parameter="@rateCG.s:COX3_pos2" scaleFactor="0.5" weight="0.1"/>

<operator id="RateGTScaler.s:COX3_pos2" spec="ScaleOperator" parameter="@rateGT.s:COX3_pos2" scaleFactor="0.5" weight="0.1"/>

<operator id="gammaShapeScaler.s:COX3_pos3" spec="ScaleOperator" parameter="@gammaShape.s:COX3_pos3" scaleFactor="0.5" weight="0.1"/>

<operator id="RateACScaler.s:COX3_pos3" spec="ScaleOperator" parameter="@rateAC.s:COX3_pos3" scaleFactor="0.5" weight="0.1"/>

<operator id="RateAGScaler.s:COX3_pos3" spec="ScaleOperator" parameter="@rateAG.s:COX3_pos3" scaleFactor="0.5" weight="0.1"/>

<operator id="RateATScaler.s:COX3_pos3" spec="ScaleOperator" parameter="@rateAT.s:COX3_pos3" scaleFactor="0.5" weight="0.1"/>

<operator id="RateCGScaler.s:COX3_pos3" spec="ScaleOperator" parameter="@rateCG.s:COX3_pos3" scaleFactor="0.5" weight="0.1"/>

<operator id="RateGTScaler.s:COX3_pos3" spec="ScaleOperator" parameter="@rateGT.s:COX3_pos3" scaleFactor="0.5" weight="0.1"/>

<operator id="gammaShapeScaler.s:CYTB_pos1" spec="ScaleOperator" parameter="@gammaShape.s:CYTB_pos1" scaleFactor="0.5" weight="0.1"/>

<operator id="RateACScaler.s:CYTB_pos1" spec="ScaleOperator" parameter="@rateAC.s:CYTB_pos1" scaleFactor="0.5" weight="0.1"/>

<operator id="RateAGScaler.s:CYTB_pos1" spec="ScaleOperator" parameter="@rateAG.s:CYTB_pos1" scaleFactor="0.5" weight="0.1"/>

<operator id="RateATScaler.s:CYTB_pos1" spec="ScaleOperator" parameter="@rateAT.s:CYTB_pos1" scaleFactor="0.5" weight="0.1"/>

<operator id="RateCGScaler.s:CYTB_pos1" spec="ScaleOperator" parameter="@rateCG.s:CYTB_pos1" scaleFactor="0.5" weight="0.1"/>

<operator id="RateGTScaler.s:CYTB_pos1" spec="ScaleOperator" parameter="@rateGT.s:CYTB_pos1" scaleFactor="0.5" weight="0.1"/>

<operator id="gammaShapeScaler.s:CYTB_pos2" spec="ScaleOperator" parameter="@gammaShape.s:CYTB_pos2" scaleFactor="0.5" weight="0.1"/>

<operator id="RateACScaler.s:CYTB_pos2" spec="ScaleOperator" parameter="@rateAC.s:CYTB_pos2" scaleFactor="0.5" weight="0.1"/>

<operator id="RateAGScaler.s:CYTB_pos2" spec="ScaleOperator" parameter="@rateAG.s:CYTB_pos2" scaleFactor="0.5" weight="0.1"/>

<operator id="RateATScaler.s:CYTB_pos2" spec="ScaleOperator" parameter="@rateAT.s:CYTB_pos2" scaleFactor="0.5" weight="0.1"/>

<operator id="RateCGScaler.s:CYTB_pos2" spec="ScaleOperator" parameter="@rateCG.s:CYTB_pos2" scaleFactor="0.5" weight="0.1"/>

<operator id="RateGTScaler.s:CYTB_pos2" spec="ScaleOperator" parameter="@rateGT.s:CYTB_pos2" scaleFactor="0.5" weight="0.1"/>

<operator id="gammaShapeScaler.s:CYTB_pos3" spec="ScaleOperator" parameter="@gammaShape.s:CYTB_pos3" scaleFactor="0.5" weight="0.1"/>

<operator id="RateACScaler.s:CYTB_pos3" spec="ScaleOperator" parameter="@rateAC.s:CYTB_pos3" scaleFactor="0.5" weight="0.1"/>

<operator id="RateAGScaler.s:CYTB_pos3" spec="ScaleOperator" parameter="@rateAG.s:CYTB_pos3" scaleFactor="0.5" weight="0.1"/>

<operator id="RateATScaler.s:CYTB_pos3" spec="ScaleOperator" parameter="@rateAT.s:CYTB_pos3" scaleFactor="0.5" weight="0.1"/>

<operator id="RateCGScaler.s:CYTB_pos3" spec="ScaleOperator" parameter="@rateCG.s:CYTB_pos3" scaleFactor="0.5" weight="0.1"/>

<operator id="RateGTScaler.s:CYTB_pos3" spec="ScaleOperator" parameter="@rateGT.s:CYTB_pos3" scaleFactor="0.5" weight="0.1"/>

<operator id="gammaShapeScaler.s:NADH1_pos1" spec="ScaleOperator" parameter="@gammaShape.s:NADH1_pos1" scaleFactor="0.5" weight="0.1"/>

<operator id="RateACScaler.s:NADH1_pos1" spec="ScaleOperator" parameter="@rateAC.s:NADH1_pos1" scaleFactor="0.5" weight="0.1"/>

<operator id="RateAGScaler.s:NADH1_pos1" spec="ScaleOperator" parameter="@rateAG.s:NADH1_pos1" scaleFactor="0.5" weight="0.1"/>

<operator id="RateATScaler.s:NADH1_pos1" spec="ScaleOperator" parameter="@rateAT.s:NADH1_pos1" scaleFactor="0.5" weight="0.1"/>

<operator id="RateCGScaler.s:NADH1_pos1" spec="ScaleOperator" parameter="@rateCG.s:NADH1_pos1" scaleFactor="0.5" weight="0.1"/>

<operator id="RateGTScaler.s:NADH1_pos1" spec="ScaleOperator" parameter="@rateGT.s:NADH1_pos1" scaleFactor="0.5" weight="0.1"/>

<operator id="gammaShapeScaler.s:NADH1_pos2" spec="ScaleOperator" parameter="@gammaShape.s:NADH1_pos2" scaleFactor="0.5" weight="0.1"/>

<operator id="RateACScaler.s:NADH1_pos2" spec="ScaleOperator" parameter="@rateAC.s:NADH1_pos2" scaleFactor="0.5" weight="0.1"/>

<operator id="RateAGScaler.s:NADH1_pos2" spec="ScaleOperator" parameter="@rateAG.s:NADH1_pos2" scaleFactor="0.5" weight="0.1"/>

<operator id="RateATScaler.s:NADH1_pos2" spec="ScaleOperator" parameter="@rateAT.s:NADH1_pos2" scaleFactor="0.5" weight="0.1"/>

<operator id="RateCGScaler.s:NADH1_pos2" spec="ScaleOperator" parameter="@rateCG.s:NADH1_pos2" scaleFactor="0.5" weight="0.1"/>

<operator id="RateGTScaler.s:NADH1_pos2" spec="ScaleOperator" parameter="@rateGT.s:NADH1_pos2" scaleFactor="0.5" weight="0.1"/>

<operator id="gammaShapeScaler.s:NADH1_pos3" spec="ScaleOperator" parameter="@gammaShape.s:NADH1_pos3" scaleFactor="0.5" weight="0.1"/>

<operator id="RateACScaler.s:NADH1_pos3" spec="ScaleOperator" parameter="@rateAC.s:NADH1_pos3" scaleFactor="0.5" weight="0.1"/>

<operator id="RateAGScaler.s:NADH1_pos3" spec="ScaleOperator" parameter="@rateAG.s:NADH1_pos3" scaleFactor="0.5" weight="0.1"/>

<operator id="RateATScaler.s:NADH1_pos3" spec="ScaleOperator" parameter="@rateAT.s:NADH1_pos3" scaleFactor="0.5" weight="0.1"/>

<operator id="RateCGScaler.s:NADH1_pos3" spec="ScaleOperator" parameter="@rateCG.s:NADH1_pos3" scaleFactor="0.5" weight="0.1"/>

<operator id="RateGTScaler.s:NADH1_pos3" spec="ScaleOperator" parameter="@rateGT.s:NADH1_pos3" scaleFactor="0.5" weight="0.1"/>

<operator id="gammaShapeScaler.s:NADH2_pos1" spec="ScaleOperator" parameter="@gammaShape.s:NADH2_pos1" scaleFactor="0.5" weight="0.1"/>

<operator id="RateACScaler.s:NADH2_pos1" spec="ScaleOperator" parameter="@rateAC.s:NADH2_pos1" scaleFactor="0.5" weight="0.1"/>

<operator id="RateAGScaler.s:NADH2_pos1" spec="ScaleOperator" parameter="@rateAG.s:NADH2_pos1" scaleFactor="0.5" weight="0.1"/>

<operator id="RateATScaler.s:NADH2_pos1" spec="ScaleOperator" parameter="@rateAT.s:NADH2_pos1" scaleFactor="0.5" weight="0.1"/>

<operator id="RateCGScaler.s:NADH2_pos1" spec="ScaleOperator" parameter="@rateCG.s:NADH2_pos1" scaleFactor="0.5" weight="0.1"/>

<operator id="RateGTScaler.s:NADH2_pos1" spec="ScaleOperator" parameter="@rateGT.s:NADH2_pos1" scaleFactor="0.5" weight="0.1"/>

<operator id="gammaShapeScaler.s:NADH2_pos2" spec="ScaleOperator" parameter="@gammaShape.s:NADH2_pos2" scaleFactor="0.5" weight="0.1"/>

<operator id="RateACScaler.s:NADH2_pos2" spec="ScaleOperator" parameter="@rateAC.s:NADH2_pos2" scaleFactor="0.5" weight="0.1"/>

<operator id="RateAGScaler.s:NADH2_pos2" spec="ScaleOperator" parameter="@rateAG.s:NADH2_pos2" scaleFactor="0.5" weight="0.1"/>

<operator id="RateATScaler.s:NADH2_pos2" spec="ScaleOperator" parameter="@rateAT.s:NADH2_pos2" scaleFactor="0.5" weight="0.1"/>

<operator id="RateCGScaler.s:NADH2_pos2" spec="ScaleOperator" parameter="@rateCG.s:NADH2_pos2" scaleFactor="0.5" weight="0.1"/>

<operator id="RateGTScaler.s:NADH2_pos2" spec="ScaleOperator" parameter="@rateGT.s:NADH2_pos2" scaleFactor="0.5" weight="0.1"/>

<operator id="gammaShapeScaler.s:NADH2_pos3" spec="ScaleOperator" parameter="@gammaShape.s:NADH2_pos3" scaleFactor="0.5" weight="0.1"/>

<operator id="RateACScaler.s:NADH2_pos3" spec="ScaleOperator" parameter="@rateAC.s:NADH2_pos3" scaleFactor="0.5" weight="0.1"/>

<operator id="RateAGScaler.s:NADH2_pos3" spec="ScaleOperator" parameter="@rateAG.s:NADH2_pos3" scaleFactor="0.5" weight="0.1"/>

<operator id="RateATScaler.s:NADH2_pos3" spec="ScaleOperator" parameter="@rateAT.s:NADH2_pos3" scaleFactor="0.5" weight="0.1"/>

<operator id="RateCGScaler.s:NADH2_pos3" spec="ScaleOperator" parameter="@rateCG.s:NADH2_pos3" scaleFactor="0.5" weight="0.1"/>

<operator id="RateGTScaler.s:NADH2_pos3" spec="ScaleOperator" parameter="@rateGT.s:NADH2_pos3" scaleFactor="0.5" weight="0.1"/>

<operator id="RateACScaler.s:NADH3_pos1" spec="ScaleOperator" parameter="@rateAC.s:NADH3_pos1" scaleFactor="0.5" weight="0.1"/>

<operator id="RateAGScaler.s:NADH3_pos1" spec="ScaleOperator" parameter="@rateAG.s:NADH3_pos1" scaleFactor="0.5" weight="0.1"/>

<operator id="RateATScaler.s:NADH3_pos1" spec="ScaleOperator" parameter="@rateAT.s:NADH3_pos1" scaleFactor="0.5" weight="0.1"/>

<operator id="RateCGScaler.s:NADH3_pos1" spec="ScaleOperator" parameter="@rateCG.s:NADH3_pos1" scaleFactor="0.5" weight="0.1"/>

<operator id="RateGTScaler.s:NADH3_pos1" spec="ScaleOperator" parameter="@rateGT.s:NADH3_pos1" scaleFactor="0.5" weight="0.1"/>

<operator id="gammaShapeScaler.s:NADH3_pos1" spec="ScaleOperator" parameter="@gammaShape.s:NADH3_pos1" scaleFactor="0.5" weight="0.1"/>

<operator id="gammaShapeScaler.s:NADH3_pos2" spec="ScaleOperator" parameter="@gammaShape.s:NADH3_pos2" scaleFactor="0.5" weight="0.1"/>

<operator id="RateACScaler.s:NADH3_pos2" spec="ScaleOperator" parameter="@rateAC.s:NADH3_pos2" scaleFactor="0.5" weight="0.1"/>

<operator id="RateAGScaler.s:NADH3_pos2" spec="ScaleOperator" parameter="@rateAG.s:NADH3_pos2" scaleFactor="0.5" weight="0.1"/>

<operator id="RateATScaler.s:NADH3_pos2" spec="ScaleOperator" parameter="@rateAT.s:NADH3_pos2" scaleFactor="0.5" weight="0.1"/>

<operator id="RateCGScaler.s:NADH3_pos2" spec="ScaleOperator" parameter="@rateCG.s:NADH3_pos2" scaleFactor="0.5" weight="0.1"/>

<operator id="RateGTScaler.s:NADH3_pos2" spec="ScaleOperator" parameter="@rateGT.s:NADH3_pos2" scaleFactor="0.5" weight="0.1"/>

<operator id="gammaShapeScaler.s:NADH3_pos3" spec="ScaleOperator" parameter="@gammaShape.s:NADH3_pos3" scaleFactor="0.5" weight="0.1"/>

<operator id="RateACScaler.s:NADH3_pos3" spec="ScaleOperator" parameter="@rateAC.s:NADH3_pos3" scaleFactor="0.5" weight="0.1"/>

<operator id="RateAGScaler.s:NADH3_pos3" spec="ScaleOperator" parameter="@rateAG.s:NADH3_pos3" scaleFactor="0.5" weight="0.1"/>

<operator id="RateATScaler.s:NADH3_pos3" spec="ScaleOperator" parameter="@rateAT.s:NADH3_pos3" scaleFactor="0.5" weight="0.1"/>

<operator id="RateCGScaler.s:NADH3_pos3" spec="ScaleOperator" parameter="@rateCG.s:NADH3_pos3" scaleFactor="0.5" weight="0.1"/>

<operator id="RateGTScaler.s:NADH3_pos3" spec="ScaleOperator" parameter="@rateGT.s:NADH3_pos3" scaleFactor="0.5" weight="0.1"/>

<operator id="gammaShapeScaler.s:NADH4_pos1" spec="ScaleOperator" parameter="@gammaShape.s:NADH4_pos1" scaleFactor="0.5" weight="0.1"/>

<operator id="RateACScaler.s:NADH4_pos1" spec="ScaleOperator" parameter="@rateAC.s:NADH4_pos1" scaleFactor="0.5" weight="0.1"/>

<operator id="RateAGScaler.s:NADH4_pos1" spec="ScaleOperator" parameter="@rateAG.s:NADH4_pos1" scaleFactor="0.5" weight="0.1"/>

<operator id="RateATScaler.s:NADH4_pos1" spec="ScaleOperator" parameter="@rateAT.s:NADH4_pos1" scaleFactor="0.5" weight="0.1"/>

<operator id="RateCGScaler.s:NADH4_pos1" spec="ScaleOperator" parameter="@rateCG.s:NADH4_pos1" scaleFactor="0.5" weight="0.1"/>

<operator id="RateGTScaler.s:NADH4_pos1" spec="ScaleOperator" parameter="@rateGT.s:NADH4_pos1" scaleFactor="0.5" weight="0.1"/>

<operator id="gammaShapeScaler.s:NADH4_pos2" spec="ScaleOperator" parameter="@gammaShape.s:NADH4_pos2" scaleFactor="0.5" weight="0.1"/>

<operator id="RateACScaler.s:NADH4_pos2" spec="ScaleOperator" parameter="@rateAC.s:NADH4_pos2" scaleFactor="0.5" weight="0.1"/>

<operator id="RateAGScaler.s:NADH4_pos2" spec="ScaleOperator" parameter="@rateAG.s:NADH4_pos2" scaleFactor="0.5" weight="0.1"/>

<operator id="RateATScaler.s:NADH4_pos2" spec="ScaleOperator" parameter="@rateAT.s:NADH4_pos2" scaleFactor="0.5" weight="0.1"/>

<operator id="RateCGScaler.s:NADH4_pos2" spec="ScaleOperator" parameter="@rateCG.s:NADH4_pos2" scaleFactor="0.5" weight="0.1"/>

<operator id="RateGTScaler.s:NADH4_pos2" spec="ScaleOperator" parameter="@rateGT.s:NADH4_pos2" scaleFactor="0.5" weight="0.1"/>

<operator id="gammaShapeScaler.s:NADH4_pos3" spec="ScaleOperator" parameter="@gammaShape.s:NADH4_pos3" scaleFactor="0.5" weight="0.1"/>

<operator id="RateACScaler.s:NADH4_pos3" spec="ScaleOperator" parameter="@rateAC.s:NADH4_pos3" scaleFactor="0.5" weight="0.1"/>

<operator id="RateAGScaler.s:NADH4_pos3" spec="ScaleOperator" parameter="@rateAG.s:NADH4_pos3" scaleFactor="0.5" weight="0.1"/>

<operator id="RateATScaler.s:NADH4_pos3" spec="ScaleOperator" parameter="@rateAT.s:NADH4_pos3" scaleFactor="0.5" weight="0.1"/>

<operator id="RateCGScaler.s:NADH4_pos3" spec="ScaleOperator" parameter="@rateCG.s:NADH4_pos3" scaleFactor="0.5" weight="0.1"/>

<operator id="RateGTScaler.s:NADH4_pos3" spec="ScaleOperator" parameter="@rateGT.s:NADH4_pos3" scaleFactor="0.5" weight="0.1"/>

<operator id="gammaShapeScaler.s:NADH4L_pos1" spec="ScaleOperator" parameter="@gammaShape.s:NADH4L_pos1" scaleFactor="0.5" weight="0.1"/>

<operator id="RateACScaler.s:NADH4L_pos1" spec="ScaleOperator" parameter="@rateAC.s:NADH4L_pos1" scaleFactor="0.5" weight="0.1"/>

<operator id="RateAGScaler.s:NADH4L_pos1" spec="ScaleOperator" parameter="@rateAG.s:NADH4L_pos1" scaleFactor="0.5" weight="0.1"/>

<operator id="RateATScaler.s:NADH4L_pos1" spec="ScaleOperator" parameter="@rateAT.s:NADH4L_pos1" scaleFactor="0.5" weight="0.1"/>

<operator id="RateCGScaler.s:NADH4L_pos1" spec="ScaleOperator" parameter="@rateCG.s:NADH4L_pos1" scaleFactor="0.5" weight="0.1"/>

<operator id="RateGTScaler.s:NADH4L_pos1" spec="ScaleOperator" parameter="@rateGT.s:NADH4L_pos1" scaleFactor="0.5" weight="0.1"/>

<operator id="gammaShapeScaler.s:NADH4L_pos2" spec="ScaleOperator" parameter="@gammaShape.s:NADH4L_pos2" scaleFactor="0.5" weight="0.1"/>

<operator id="RateACScaler.s:NADH4L_pos2" spec="ScaleOperator" parameter="@rateAC.s:NADH4L_pos2" scaleFactor="0.5" weight="0.1"/>

<operator id="RateAGScaler.s:NADH4L_pos2" spec="ScaleOperator" parameter="@rateAG.s:NADH4L_pos2" scaleFactor="0.5" weight="0.1"/>

<operator id="RateATScaler.s:NADH4L_pos2" spec="ScaleOperator" parameter="@rateAT.s:NADH4L_pos2" scaleFactor="0.5" weight="0.1"/>

<operator id="RateCGScaler.s:NADH4L_pos2" spec="ScaleOperator" parameter="@rateCG.s:NADH4L_pos2" scaleFactor="0.5" weight="0.1"/>

<operator id="RateGTScaler.s:NADH4L_pos2" spec="ScaleOperator" parameter="@rateGT.s:NADH4L_pos2" scaleFactor="0.5" weight="0.1"/>

<operator id="gammaShapeScaler.s:NADH4L_pos3" spec="ScaleOperator" parameter="@gammaShape.s:NADH4L_pos3" scaleFactor="0.5" weight="0.1"/>

<operator id="RateACScaler.s:NADH4L_pos3" spec="ScaleOperator" parameter="@rateAC.s:NADH4L_pos3" scaleFactor="0.5" weight="0.1"/>

<operator id="RateAGScaler.s:NADH4L_pos3" spec="ScaleOperator" parameter="@rateAG.s:NADH4L_pos3" scaleFactor="0.5" weight="0.1"/>

<operator id="RateATScaler.s:NADH4L_pos3" spec="ScaleOperator" parameter="@rateAT.s:NADH4L_pos3" scaleFactor="0.5" weight="0.1"/>

<operator id="RateCGScaler.s:NADH4L_pos3" spec="ScaleOperator" parameter="@rateCG.s:NADH4L_pos3" scaleFactor="0.5" weight="0.1"/>

<operator id="RateGTScaler.s:NADH4L_pos3" spec="ScaleOperator" parameter="@rateGT.s:NADH4L_pos3" scaleFactor="0.5" weight="0.1"/>

<operator id="gammaShapeScaler.s:NADH5_pos1" spec="ScaleOperator" parameter="@gammaShape.s:NADH5_pos1" scaleFactor="0.5" weight="0.1"/>

<operator id="RateACScaler.s:NADH5_pos1" spec="ScaleOperator" parameter="@rateAC.s:NADH5_pos1" scaleFactor="0.5" weight="0.1"/>

<operator id="RateAGScaler.s:NADH5_pos1" spec="ScaleOperator" parameter="@rateAG.s:NADH5_pos1" scaleFactor="0.5" weight="0.1"/>

<operator id="RateATScaler.s:NADH5_pos1" spec="ScaleOperator" parameter="@rateAT.s:NADH5_pos1" scaleFactor="0.5" weight="0.1"/>

<operator id="RateCGScaler.s:NADH5_pos1" spec="ScaleOperator" parameter="@rateCG.s:NADH5_pos1" scaleFactor="0.5" weight="0.1"/>

<operator id="RateGTScaler.s:NADH5_pos1" spec="ScaleOperator" parameter="@rateGT.s:NADH5_pos1" scaleFactor="0.5" weight="0.1"/>

<operator id="gammaShapeScaler.s:NADH5_pos2" spec="ScaleOperator" parameter="@gammaShape.s:NADH5_pos2" scaleFactor="0.5" weight="0.1"/>

<operator id="RateACScaler.s:NADH5_pos2" spec="ScaleOperator" parameter="@rateAC.s:NADH5_pos2" scaleFactor="0.5" weight="0.1"/>

<operator id="RateAGScaler.s:NADH5_pos2" spec="ScaleOperator" parameter="@rateAG.s:NADH5_pos2" scaleFactor="0.5" weight="0.1"/>

<operator id="RateATScaler.s:NADH5_pos2" spec="ScaleOperator" parameter="@rateAT.s:NADH5_pos2" scaleFactor="0.5" weight="0.1"/>

<operator id="RateCGScaler.s:NADH5_pos2" spec="ScaleOperator" parameter="@rateCG.s:NADH5_pos2" scaleFactor="0.5" weight="0.1"/>

<operator id="RateGTScaler.s:NADH5_pos2" spec="ScaleOperator" parameter="@rateGT.s:NADH5_pos2" scaleFactor="0.5" weight="0.1"/>

<operator id="gammaShapeScaler.s:NADH5_pos3" spec="ScaleOperator" parameter="@gammaShape.s:NADH5_pos3" scaleFactor="0.5" weight="0.1"/>

<operator id="RateACScaler.s:NADH5_pos3" spec="ScaleOperator" parameter="@rateAC.s:NADH5_pos3" scaleFactor="0.5" weight="0.1"/>

<operator id="RateAGScaler.s:NADH5_pos3" spec="ScaleOperator" parameter="@rateAG.s:NADH5_pos3" scaleFactor="0.5" weight="0.1"/>

<operator id="RateATScaler.s:NADH5_pos3" spec="ScaleOperator" parameter="@rateAT.s:NADH5_pos3" scaleFactor="0.5" weight="0.1"/>

<operator id="RateCGScaler.s:NADH5_pos3" spec="ScaleOperator" parameter="@rateCG.s:NADH5_pos3" scaleFactor="0.5" weight="0.1"/>

<operator id="RateGTScaler.s:NADH5_pos3" spec="ScaleOperator" parameter="@rateGT.s:NADH5_pos3" scaleFactor="0.5" weight="0.1"/>

<operator id="gammaShapeScaler.s:NADH6_pos1" spec="ScaleOperator" parameter="@gammaShape.s:NADH6_pos1" scaleFactor="0.5" weight="0.1"/>

<operator id="RateACScaler.s:NADH6_pos1" spec="ScaleOperator" parameter="@rateAC.s:NADH6_pos1" scaleFactor="0.5" weight="0.1"/>

<operator id="RateAGScaler.s:NADH6_pos1" spec="ScaleOperator" parameter="@rateAG.s:NADH6_pos1" scaleFactor="0.5" weight="0.1"/>

<operator id="RateATScaler.s:NADH6_pos1" spec="ScaleOperator" parameter="@rateAT.s:NADH6_pos1" scaleFactor="0.5" weight="0.1"/>

<operator id="RateCGScaler.s:NADH6_pos1" spec="ScaleOperator" parameter="@rateCG.s:NADH6_pos1" scaleFactor="0.5" weight="0.1"/>

<operator id="RateGTScaler.s:NADH6_pos1" spec="ScaleOperator" parameter="@rateGT.s:NADH6_pos1" scaleFactor="0.5" weight="0.1"/>

<operator id="gammaShapeScaler.s:NADH6_pos2" spec="ScaleOperator" parameter="@gammaShape.s:NADH6_pos2" scaleFactor="0.5" weight="0.1"/>

<operator id="RateACScaler.s:NADH6_pos2" spec="ScaleOperator" parameter="@rateAC.s:NADH6_pos2" scaleFactor="0.5" weight="0.1"/>

<operator id="RateAGScaler.s:NADH6_pos2" spec="ScaleOperator" parameter="@rateAG.s:NADH6_pos2" scaleFactor="0.5" weight="0.1"/>

<operator id="RateATScaler.s:NADH6_pos2" spec="ScaleOperator" parameter="@rateAT.s:NADH6_pos2" scaleFactor="0.5" weight="0.1"/>

<operator id="RateCGScaler.s:NADH6_pos2" spec="ScaleOperator" parameter="@rateCG.s:NADH6_pos2" scaleFactor="0.5" weight="0.1"/>

<operator id="RateGTScaler.s:NADH6_pos2" spec="ScaleOperator" parameter="@rateGT.s:NADH6_pos2" scaleFactor="0.5" weight="0.1"/>

<operator id="gammaShapeScaler.s:NADH6_pos3" spec="ScaleOperator" parameter="@gammaShape.s:NADH6_pos3" scaleFactor="0.5" weight="0.1"/>

<operator id="RateACScaler.s:NADH6_pos3" spec="ScaleOperator" parameter="@rateAC.s:NADH6_pos3" scaleFactor="0.5" weight="0.1"/>

<operator id="RateAGScaler.s:NADH6_pos3" spec="ScaleOperator" parameter="@rateAG.s:NADH6_pos3" scaleFactor="0.5" weight="0.1"/>

<operator id="RateATScaler.s:NADH6_pos3" spec="ScaleOperator" parameter="@rateAT.s:NADH6_pos3" scaleFactor="0.5" weight="0.1"/>

<operator id="RateCGScaler.s:NADH6_pos3" spec="ScaleOperator" parameter="@rateCG.s:NADH6_pos3" scaleFactor="0.5" weight="0.1"/>

<operator id="RateGTScaler.s:NADH6_pos3" spec="ScaleOperator" parameter="@rateGT.s:NADH6_pos3" scaleFactor="0.5" weight="0.1"/>

<operator id="CoalescentConstantTreeScaler.t:COX1_pos1" spec="ScaleOperator" scaleFactor="0.5" tree="@Tree.t:COX1_pos1" weight="3.0"/>

<operator id="CoalescentConstantTreeRootScaler.t:COX1_pos1" spec="ScaleOperator" rootOnly="true" scaleFactor="0.5" tree="@Tree.t:COX1_pos1" weight="3.0"/>

<operator id="CoalescentConstantUniformOperator.t:COX1_pos1" spec="Uniform" tree="@Tree.t:COX1_pos1" weight="30.0"/>

<operator id="CoalescentConstantSubtreeSlide.t:COX1_pos1" spec="SubtreeSlide" tree="@Tree.t:COX1_pos1" weight="15.0"/>

<operator id="CoalescentConstantNarrow.t:COX1_pos1" spec="Exchange" tree="@Tree.t:COX1_pos1" weight="15.0"/>

<operator id="CoalescentConstantWide.t:COX1_pos1" spec="Exchange" isNarrow="false" tree="@Tree.t:COX1_pos1" weight="3.0"/>

<operator id="CoalescentConstantWilsonBalding.t:COX1_pos1" spec="WilsonBalding" tree="@Tree.t:COX1_pos1" weight="3.0"/>

<operator id="PopSizeScaler.t:COX1_pos1" spec="ScaleOperator" parameter="@popSize.t:COX1_pos1" weight="3.0"/>

<operator id="CoalescentConstantTreeScaler.t:COX1_pos2" spec="ScaleOperator" scaleFactor="0.5" tree="@Tree.t:COX1_pos2" weight="3.0"/>

<operator id="CoalescentConstantTreeRootScaler.t:COX1_pos2" spec="ScaleOperator" rootOnly="true" scaleFactor="0.5" tree="@Tree.t:COX1_pos2" weight="3.0"/>

<operator id="CoalescentConstantUniformOperator.t:COX1_pos2" spec="Uniform" tree="@Tree.t:COX1_pos2" weight="30.0"/>

<operator id="CoalescentConstantSubtreeSlide.t:COX1_pos2" spec="SubtreeSlide" tree="@Tree.t:COX1_pos2" weight="15.0"/>

<operator id="CoalescentConstantNarrow.t:COX1_pos2" spec="Exchange" tree="@Tree.t:COX1_pos2" weight="15.0"/>

<operator id="CoalescentConstantWide.t:COX1_pos2" spec="Exchange" isNarrow="false" tree="@Tree.t:COX1_pos2" weight="3.0"/>

<operator id="CoalescentConstantWilsonBalding.t:COX1_pos2" spec="WilsonBalding" tree="@Tree.t:COX1_pos2" weight="3.0"/>

<operator id="PopSizeScaler.t:COX1_pos2" spec="ScaleOperator" parameter="@popSize.t:COX1_pos2" weight="3.0"/>

<operator id="CoalescentConstantTreeScaler.t:COX1_pos3" spec="ScaleOperator" scaleFactor="0.5" tree="@Tree.t:COX1_pos3" weight="3.0"/>

<operator id="CoalescentConstantTreeRootScaler.t:COX1_pos3" spec="ScaleOperator" rootOnly="true" scaleFactor="0.5" tree="@Tree.t:COX1_pos3" weight="3.0"/>

<operator id="CoalescentConstantUniformOperator.t:COX1_pos3" spec="Uniform" tree="@Tree.t:COX1_pos3" weight="30.0"/>

<operator id="CoalescentConstantSubtreeSlide.t:COX1_pos3" spec="SubtreeSlide" tree="@Tree.t:COX1_pos3" weight="15.0"/>

<operator id="CoalescentConstantNarrow.t:COX1_pos3" spec="Exchange" tree="@Tree.t:COX1_pos3" weight="15.0"/>

<operator id="CoalescentConstantWide.t:COX1_pos3" spec="Exchange" isNarrow="false" tree="@Tree.t:COX1_pos3" weight="3.0"/>

<operator id="CoalescentConstantWilsonBalding.t:COX1_pos3" spec="WilsonBalding" tree="@Tree.t:COX1_pos3" weight="3.0"/>

<operator id="PopSizeScaler.t:COX1_pos3" spec="ScaleOperator" parameter="@popSize.t:COX1_pos3" weight="3.0"/>

<operator id="CoalescentConstantTreeScaler.t:COX2_pos1" spec="ScaleOperator" scaleFactor="0.5" tree="@Tree.t:COX2_pos1" weight="3.0"/>

<operator id="CoalescentConstantTreeRootScaler.t:COX2_pos1" spec="ScaleOperator" rootOnly="true" scaleFactor="0.5" tree="@Tree.t:COX2_pos1" weight="3.0"/>

<operator id="CoalescentConstantUniformOperator.t:COX2_pos1" spec="Uniform" tree="@Tree.t:COX2_pos1" weight="30.0"/>

<operator id="CoalescentConstantSubtreeSlide.t:COX2_pos1" spec="SubtreeSlide" tree="@Tree.t:COX2_pos1" weight="15.0"/>

<operator id="CoalescentConstantNarrow.t:COX2_pos1" spec="Exchange" tree="@Tree.t:COX2_pos1" weight="15.0"/>

<operator id="CoalescentConstantWide.t:COX2_pos1" spec="Exchange" isNarrow="false" tree="@Tree.t:COX2_pos1" weight="3.0"/>

<operator id="CoalescentConstantWilsonBalding.t:COX2_pos1" spec="WilsonBalding" tree="@Tree.t:COX2_pos1" weight="3.0"/>

<operator id="PopSizeScaler.t:COX2_pos1" spec="ScaleOperator" parameter="@popSize.t:COX2_pos1" weight="3.0"/>

<operator id="CoalescentConstantTreeScaler.t:COX2_pos2" spec="ScaleOperator" scaleFactor="0.5" tree="@Tree.t:COX2_pos2" weight="3.0"/>

<operator id="CoalescentConstantTreeRootScaler.t:COX2_pos2" spec="ScaleOperator" rootOnly="true" scaleFactor="0.5" tree="@Tree.t:COX2_pos2" weight="3.0"/>

<operator id="CoalescentConstantUniformOperator.t:COX2_pos2" spec="Uniform" tree="@Tree.t:COX2_pos2" weight="30.0"/>

<operator id="CoalescentConstantSubtreeSlide.t:COX2_pos2" spec="SubtreeSlide" tree="@Tree.t:COX2_pos2" weight="15.0"/>

<operator id="CoalescentConstantNarrow.t:COX2_pos2" spec="Exchange" tree="@Tree.t:COX2_pos2" weight="15.0"/>

<operator id="CoalescentConstantWide.t:COX2_pos2" spec="Exchange" isNarrow="false" tree="@Tree.t:COX2_pos2" weight="3.0"/>

<operator id="CoalescentConstantWilsonBalding.t:COX2_pos2" spec="WilsonBalding" tree="@Tree.t:COX2_pos2" weight="3.0"/>

<operator id="PopSizeScaler.t:COX2_pos2" spec="ScaleOperator" parameter="@popSize.t:COX2_pos2" weight="3.0"/>

<operator id="CoalescentConstantTreeScaler.t:COX2_pos3" spec="ScaleOperator" scaleFactor="0.5" tree="@Tree.t:COX2_pos3" weight="3.0"/>

<operator id="CoalescentConstantTreeRootScaler.t:COX2_pos3" spec="ScaleOperator" rootOnly="true" scaleFactor="0.5" tree="@Tree.t:COX2_pos3" weight="3.0"/>

<operator id="CoalescentConstantUniformOperator.t:COX2_pos3" spec="Uniform" tree="@Tree.t:COX2_pos3" weight="30.0"/>

<operator id="CoalescentConstantSubtreeSlide.t:COX2_pos3" spec="SubtreeSlide" tree="@Tree.t:COX2_pos3" weight="15.0"/>

<operator id="CoalescentConstantNarrow.t:COX2_pos3" spec="Exchange" tree="@Tree.t:COX2_pos3" weight="15.0"/>

<operator id="CoalescentConstantWide.t:COX2_pos3" spec="Exchange" isNarrow="false" tree="@Tree.t:COX2_pos3" weight="3.0"/>

<operator id="CoalescentConstantWilsonBalding.t:COX2_pos3" spec="WilsonBalding" tree="@Tree.t:COX2_pos3" weight="3.0"/>

<operator id="PopSizeScaler.t:COX2_pos3" spec="ScaleOperator" parameter="@popSize.t:COX2_pos3" weight="3.0"/>

<operator id="CoalescentConstantTreeScaler.t:COX3_pos1" spec="ScaleOperator" scaleFactor="0.5" tree="@Tree.t:COX3_pos1" weight="3.0"/>

<operator id="CoalescentConstantTreeRootScaler.t:COX3_pos1" spec="ScaleOperator" rootOnly="true" scaleFactor="0.5" tree="@Tree.t:COX3_pos1" weight="3.0"/>

<operator id="CoalescentConstantUniformOperator.t:COX3_pos1" spec="Uniform" tree="@Tree.t:COX3_pos1" weight="30.0"/>

<operator id="CoalescentConstantSubtreeSlide.t:COX3_pos1" spec="SubtreeSlide" tree="@Tree.t:COX3_pos1" weight="15.0"/>

<operator id="CoalescentConstantNarrow.t:COX3_pos1" spec="Exchange" tree="@Tree.t:COX3_pos1" weight="15.0"/>

<operator id="CoalescentConstantWide.t:COX3_pos1" spec="Exchange" isNarrow="false" tree="@Tree.t:COX3_pos1" weight="3.0"/>

<operator id="CoalescentConstantWilsonBalding.t:COX3_pos1" spec="WilsonBalding" tree="@Tree.t:COX3_pos1" weight="3.0"/>

<operator id="PopSizeScaler.t:COX3_pos1" spec="ScaleOperator" parameter="@popSize.t:COX3_pos1" weight="3.0"/>

<operator id="CoalescentConstantTreeScaler.t:COX3_pos2" spec="ScaleOperator" scaleFactor="0.5" tree="@Tree.t:COX3_pos2" weight="3.0"/>

<operator id="CoalescentConstantTreeRootScaler.t:COX3_pos2" spec="ScaleOperator" rootOnly="true" scaleFactor="0.5" tree="@Tree.t:COX3_pos2" weight="3.0"/>

<operator id="CoalescentConstantUniformOperator.t:COX3_pos2" spec="Uniform" tree="@Tree.t:COX3_pos2" weight="30.0"/>

<operator id="CoalescentConstantSubtreeSlide.t:COX3_pos2" spec="SubtreeSlide" tree="@Tree.t:COX3_pos2" weight="15.0"/>

<operator id="CoalescentConstantNarrow.t:COX3_pos2" spec="Exchange" tree="@Tree.t:COX3_pos2" weight="15.0"/>

<operator id="CoalescentConstantWide.t:COX3_pos2" spec="Exchange" isNarrow="false" tree="@Tree.t:COX3_pos2" weight="3.0"/>

<operator id="CoalescentConstantWilsonBalding.t:COX3_pos2" spec="WilsonBalding" tree="@Tree.t:COX3_pos2" weight="3.0"/>

<operator id="PopSizeScaler.t:COX3_pos2" spec="ScaleOperator" parameter="@popSize.t:COX3_pos2" weight="3.0"/>

<operator id="CoalescentConstantTreeScaler.t:COX3_pos3" spec="ScaleOperator" scaleFactor="0.5" tree="@Tree.t:COX3_pos3" weight="3.0"/>

<operator id="CoalescentConstantTreeRootScaler.t:COX3_pos3" spec="ScaleOperator" rootOnly="true" scaleFactor="0.5" tree="@Tree.t:COX3_pos3" weight="3.0"/>

<operator id="CoalescentConstantUniformOperator.t:COX3_pos3" spec="Uniform" tree="@Tree.t:COX3_pos3" weight="30.0"/>

<operator id="CoalescentConstantSubtreeSlide.t:COX3_pos3" spec="SubtreeSlide" tree="@Tree.t:COX3_pos3" weight="15.0"/>

<operator id="CoalescentConstantNarrow.t:COX3_pos3" spec="Exchange" tree="@Tree.t:COX3_pos3" weight="15.0"/>

<operator id="CoalescentConstantWide.t:COX3_pos3" spec="Exchange" isNarrow="false" tree="@Tree.t:COX3_pos3" weight="3.0"/>

<operator id="CoalescentConstantWilsonBalding.t:COX3_pos3" spec="WilsonBalding" tree="@Tree.t:COX3_pos3" weight="3.0"/>

<operator id="PopSizeScaler.t:COX3_pos3" spec="ScaleOperator" parameter="@popSize.t:COX3_pos3" weight="3.0"/>

<operator id="CoalescentConstantTreeScaler.t:CYTB_pos1" spec="ScaleOperator" scaleFactor="0.5" tree="@Tree.t:CYTB_pos1" weight="3.0"/>

<operator id="CoalescentConstantTreeRootScaler.t:CYTB_pos1" spec="ScaleOperator" rootOnly="true" scaleFactor="0.5" tree="@Tree.t:CYTB_pos1" weight="3.0"/>

<operator id="CoalescentConstantUniformOperator.t:CYTB_pos1" spec="Uniform" tree="@Tree.t:CYTB_pos1" weight="30.0"/>

<operator id="CoalescentConstantSubtreeSlide.t:CYTB_pos1" spec="SubtreeSlide" tree="@Tree.t:CYTB_pos1" weight="15.0"/>

<operator id="CoalescentConstantNarrow.t:CYTB_pos1" spec="Exchange" tree="@Tree.t:CYTB_pos1" weight="15.0"/>

<operator id="CoalescentConstantWide.t:CYTB_pos1" spec="Exchange" isNarrow="false" tree="@Tree.t:CYTB_pos1" weight="3.0"/>

<operator id="CoalescentConstantWilsonBalding.t:CYTB_pos1" spec="WilsonBalding" tree="@Tree.t:CYTB_pos1" weight="3.0"/>

<operator id="PopSizeScaler.t:CYTB_pos1" spec="ScaleOperator" parameter="@popSize.t:CYTB_pos1" weight="3.0"/>

<operator id="CoalescentConstantTreeScaler.t:CYTB_pos2" spec="ScaleOperator" scaleFactor="0.5" tree="@Tree.t:CYTB_pos2" weight="3.0"/>

<operator id="CoalescentConstantTreeRootScaler.t:CYTB_pos2" spec="ScaleOperator" rootOnly="true" scaleFactor="0.5" tree="@Tree.t:CYTB_pos2" weight="3.0"/>

<operator id="CoalescentConstantUniformOperator.t:CYTB_pos2" spec="Uniform" tree="@Tree.t:CYTB_pos2" weight="30.0"/>

<operator id="CoalescentConstantSubtreeSlide.t:CYTB_pos2" spec="SubtreeSlide" tree="@Tree.t:CYTB_pos2" weight="15.0"/>

<operator id="CoalescentConstantNarrow.t:CYTB_pos2" spec="Exchange" tree="@Tree.t:CYTB_pos2" weight="15.0"/>

<operator id="CoalescentConstantWide.t:CYTB_pos2" spec="Exchange" isNarrow="false" tree="@Tree.t:CYTB_pos2" weight="3.0"/>

<operator id="CoalescentConstantWilsonBalding.t:CYTB_pos2" spec="WilsonBalding" tree="@Tree.t:CYTB_pos2" weight="3.0"/>

<operator id="PopSizeScaler.t:CYTB_pos2" spec="ScaleOperator" parameter="@popSize.t:CYTB_pos2" weight="3.0"/>

<operator id="CoalescentConstantTreeScaler.t:CYTB_pos3" spec="ScaleOperator" scaleFactor="0.5" tree="@Tree.t:CYTB_pos3" weight="3.0"/>

<operator id="CoalescentConstantTreeRootScaler.t:CYTB_pos3" spec="ScaleOperator" rootOnly="true" scaleFactor="0.5" tree="@Tree.t:CYTB_pos3" weight="3.0"/>

<operator id="CoalescentConstantUniformOperator.t:CYTB_pos3" spec="Uniform" tree="@Tree.t:CYTB_pos3" weight="30.0"/>

<operator id="CoalescentConstantSubtreeSlide.t:CYTB_pos3" spec="SubtreeSlide" tree="@Tree.t:CYTB_pos3" weight="15.0"/>

<operator id="CoalescentConstantNarrow.t:CYTB_pos3" spec="Exchange" tree="@Tree.t:CYTB_pos3" weight="15.0"/>

<operator id="CoalescentConstantWide.t:CYTB_pos3" spec="Exchange" isNarrow="false" tree="@Tree.t:CYTB_pos3" weight="3.0"/>

<operator id="CoalescentConstantWilsonBalding.t:CYTB_pos3" spec="WilsonBalding" tree="@Tree.t:CYTB_pos3" weight="3.0"/>

<operator id="PopSizeScaler.t:CYTB_pos3" spec="ScaleOperator" parameter="@popSize.t:CYTB_pos3" weight="3.0"/>

<operator id="CoalescentConstantTreeScaler.t:Mytilidae27sq12PCGs" spec="ScaleOperator" scaleFactor="0.5" tree="@Tree.t:Mytilidae27sq12PCGs" weight="3.0"/>

<operator id="CoalescentConstantTreeRootScaler.t:Mytilidae27sq12PCGs" spec="ScaleOperator" rootOnly="true" scaleFactor="0.5" tree="@Tree.t:Mytilidae27sq12PCGs" weight="3.0"/>

<operator id="CoalescentConstantUniformOperator.t:Mytilidae27sq12PCGs" spec="Uniform" tree="@Tree.t:Mytilidae27sq12PCGs" weight="30.0"/>

<operator id="CoalescentConstantSubtreeSlide.t:Mytilidae27sq12PCGs" spec="SubtreeSlide" tree="@Tree.t:Mytilidae27sq12PCGs" weight="15.0"/>

<operator id="CoalescentConstantNarrow.t:Mytilidae27sq12PCGs" spec="Exchange" tree="@Tree.t:Mytilidae27sq12PCGs" weight="15.0"/>

<operator id="CoalescentConstantWide.t:Mytilidae27sq12PCGs" spec="Exchange" isNarrow="false" tree="@Tree.t:Mytilidae27sq12PCGs" weight="3.0"/>

<operator id="CoalescentConstantWilsonBalding.t:Mytilidae27sq12PCGs" spec="WilsonBalding" tree="@Tree.t:Mytilidae27sq12PCGs" weight="3.0"/>

<operator id="PopSizeScaler.t:Mytilidae27sq12PCGs" spec="ScaleOperator" parameter="@popSize.t:Mytilidae27sq12PCGs" weight="3.0"/>

<operator id="CoalescentConstantTreeScaler.t:NADH1_pos1" spec="ScaleOperator" scaleFactor="0.5" tree="@Tree.t:NADH1_pos1" weight="3.0"/>

<operator id="CoalescentConstantTreeRootScaler.t:NADH1_pos1" spec="ScaleOperator" rootOnly="true" scaleFactor="0.5" tree="@Tree.t:NADH1_pos1" weight="3.0"/>

<operator id="CoalescentConstantUniformOperator.t:NADH1_pos1" spec="Uniform" tree="@Tree.t:NADH1_pos1" weight="30.0"/>

<operator id="CoalescentConstantSubtreeSlide.t:NADH1_pos1" spec="SubtreeSlide" tree="@Tree.t:NADH1_pos1" weight="15.0"/>

<operator id="CoalescentConstantNarrow.t:NADH1_pos1" spec="Exchange" tree="@Tree.t:NADH1_pos1" weight="15.0"/>

<operator id="CoalescentConstantWide.t:NADH1_pos1" spec="Exchange" isNarrow="false" tree="@Tree.t:NADH1_pos1" weight="3.0"/>

<operator id="CoalescentConstantWilsonBalding.t:NADH1_pos1" spec="WilsonBalding" tree="@Tree.t:NADH1_pos1" weight="3.0"/>

<operator id="PopSizeScaler.t:NADH1_pos1" spec="ScaleOperator" parameter="@popSize.t:NADH1_pos1" weight="3.0"/>

<operator id="CoalescentConstantTreeScaler.t:NADH1_pos2" spec="ScaleOperator" scaleFactor="0.5" tree="@Tree.t:NADH1_pos2" weight="3.0"/>

<operator id="CoalescentConstantTreeRootScaler.t:NADH1_pos2" spec="ScaleOperator" rootOnly="true" scaleFactor="0.5" tree="@Tree.t:NADH1_pos2" weight="3.0"/>

<operator id="CoalescentConstantUniformOperator.t:NADH1_pos2" spec="Uniform" tree="@Tree.t:NADH1_pos2" weight="30.0"/>

<operator id="CoalescentConstantSubtreeSlide.t:NADH1_pos2" spec="SubtreeSlide" tree="@Tree.t:NADH1_pos2" weight="15.0"/>

<operator id="CoalescentConstantNarrow.t:NADH1_pos2" spec="Exchange" tree="@Tree.t:NADH1_pos2" weight="15.0"/>

<operator id="CoalescentConstantWide.t:NADH1_pos2" spec="Exchange" isNarrow="false" tree="@Tree.t:NADH1_pos2" weight="3.0"/>

<operator id="CoalescentConstantWilsonBalding.t:NADH1_pos2" spec="WilsonBalding" tree="@Tree.t:NADH1_pos2" weight="3.0"/>

<operator id="PopSizeScaler.t:NADH1_pos2" spec="ScaleOperator" parameter="@popSize.t:NADH1_pos2" weight="3.0"/>

<operator id="CoalescentConstantTreeScaler.t:NADH1_pos3" spec="ScaleOperator" scaleFactor="0.5" tree="@Tree.t:NADH1_pos3" weight="3.0"/>

<operator id="CoalescentConstantTreeRootScaler.t:NADH1_pos3" spec="ScaleOperator" rootOnly="true" scaleFactor="0.5" tree="@Tree.t:NADH1_pos3" weight="3.0"/>

<operator id="CoalescentConstantUniformOperator.t:NADH1_pos3" spec="Uniform" tree="@Tree.t:NADH1_pos3" weight="30.0"/>

<operator id="CoalescentConstantSubtreeSlide.t:NADH1_pos3" spec="SubtreeSlide" tree="@Tree.t:NADH1_pos3" weight="15.0"/>

<operator id="CoalescentConstantNarrow.t:NADH1_pos3" spec="Exchange" tree="@Tree.t:NADH1_pos3" weight="15.0"/>

<operator id="CoalescentConstantWide.t:NADH1_pos3" spec="Exchange" isNarrow="false" tree="@Tree.t:NADH1_pos3" weight="3.0"/>

<operator id="CoalescentConstantWilsonBalding.t:NADH1_pos3" spec="WilsonBalding" tree="@Tree.t:NADH1_pos3" weight="3.0"/>

<operator id="PopSizeScaler.t:NADH1_pos3" spec="ScaleOperator" parameter="@popSize.t:NADH1_pos3" weight="3.0"/>

<operator id="CoalescentConstantTreeScaler.t:NADH2_pos1" spec="ScaleOperator" scaleFactor="0.5" tree="@Tree.t:NADH2_pos1" weight="3.0"/>

<operator id="CoalescentConstantTreeRootScaler.t:NADH2_pos1" spec="ScaleOperator" rootOnly="true" scaleFactor="0.5" tree="@Tree.t:NADH2_pos1" weight="3.0"/>

<operator id="CoalescentConstantUniformOperator.t:NADH2_pos1" spec="Uniform" tree="@Tree.t:NADH2_pos1" weight="30.0"/>

<operator id="CoalescentConstantSubtreeSlide.t:NADH2_pos1" spec="SubtreeSlide" tree="@Tree.t:NADH2_pos1" weight="15.0"/>

<operator id="CoalescentConstantNarrow.t:NADH2_pos1" spec="Exchange" tree="@Tree.t:NADH2_pos1" weight="15.0"/>

<operator id="CoalescentConstantWide.t:NADH2_pos1" spec="Exchange" isNarrow="false" tree="@Tree.t:NADH2_pos1" weight="3.0"/>

<operator id="CoalescentConstantWilsonBalding.t:NADH2_pos1" spec="WilsonBalding" tree="@Tree.t:NADH2_pos1" weight="3.0"/>

<operator id="PopSizeScaler.t:NADH2_pos1" spec="ScaleOperator" parameter="@popSize.t:NADH2_pos1" weight="3.0"/>

<operator id="CoalescentConstantTreeScaler.t:NADH2_pos2" spec="ScaleOperator" scaleFactor="0.5" tree="@Tree.t:NADH2_pos2" weight="3.0"/>

<operator id="CoalescentConstantTreeRootScaler.t:NADH2_pos2" spec="ScaleOperator" rootOnly="true" scaleFactor="0.5" tree="@Tree.t:NADH2_pos2" weight="3.0"/>

<operator id="CoalescentConstantUniformOperator.t:NADH2_pos2" spec="Uniform" tree="@Tree.t:NADH2_pos2" weight="30.0"/>

<operator id="CoalescentConstantSubtreeSlide.t:NADH2_pos2" spec="SubtreeSlide" tree="@Tree.t:NADH2_pos2" weight="15.0"/>

<operator id="CoalescentConstantNarrow.t:NADH2_pos2" spec="Exchange" tree="@Tree.t:NADH2_pos2" weight="15.0"/>

<operator id="CoalescentConstantWide.t:NADH2_pos2" spec="Exchange" isNarrow="false" tree="@Tree.t:NADH2_pos2" weight="3.0"/>

<operator id="CoalescentConstantWilsonBalding.t:NADH2_pos2" spec="WilsonBalding" tree="@Tree.t:NADH2_pos2" weight="3.0"/>

<operator id="PopSizeScaler.t:NADH2_pos2" spec="ScaleOperator" parameter="@popSize.t:NADH2_pos2" weight="3.0"/>

<operator id="CoalescentConstantTreeScaler.t:NADH2_pos3" spec="ScaleOperator" scaleFactor="0.5" tree="@Tree.t:NADH2_pos3" weight="3.0"/>

<operator id="CoalescentConstantTreeRootScaler.t:NADH2_pos3" spec="ScaleOperator" rootOnly="true" scaleFactor="0.5" tree="@Tree.t:NADH2_pos3" weight="3.0"/>

<operator id="CoalescentConstantUniformOperator.t:NADH2_pos3" spec="Uniform" tree="@Tree.t:NADH2_pos3" weight="30.0"/>

<operator id="CoalescentConstantSubtreeSlide.t:NADH2_pos3" spec="SubtreeSlide" tree="@Tree.t:NADH2_pos3" weight="15.0"/>

<operator id="CoalescentConstantNarrow.t:NADH2_pos3" spec="Exchange" tree="@Tree.t:NADH2_pos3" weight="15.0"/>

<operator id="CoalescentConstantWide.t:NADH2_pos3" spec="Exchange" isNarrow="false" tree="@Tree.t:NADH2_pos3" weight="3.0"/>

<operator id="CoalescentConstantWilsonBalding.t:NADH2_pos3" spec="WilsonBalding" tree="@Tree.t:NADH2_pos3" weight="3.0"/>

<operator id="PopSizeScaler.t:NADH2_pos3" spec="ScaleOperator" parameter="@popSize.t:NADH2_pos3" weight="3.0"/>

<operator id="CoalescentConstantTreeScaler.t:NADH3_pos1" spec="ScaleOperator" scaleFactor="0.5" tree="@Tree.t:NADH3_pos1" weight="3.0"/>

<operator id="CoalescentConstantTreeRootScaler.t:NADH3_pos1" spec="ScaleOperator" rootOnly="true" scaleFactor="0.5" tree="@Tree.t:NADH3_pos1" weight="3.0"/>

<operator id="CoalescentConstantUniformOperator.t:NADH3_pos1" spec="Uniform" tree="@Tree.t:NADH3_pos1" weight="30.0"/>

<operator id="CoalescentConstantSubtreeSlide.t:NADH3_pos1" spec="SubtreeSlide" tree="@Tree.t:NADH3_pos1" weight="15.0"/>

<operator id="CoalescentConstantNarrow.t:NADH3_pos1" spec="Exchange" tree="@Tree.t:NADH3_pos1" weight="15.0"/>

<operator id="CoalescentConstantWide.t:NADH3_pos1" spec="Exchange" isNarrow="false" tree="@Tree.t:NADH3_pos1" weight="3.0"/>

<operator id="CoalescentConstantWilsonBalding.t:NADH3_pos1" spec="WilsonBalding" tree="@Tree.t:NADH3_pos1" weight="3.0"/>

<operator id="PopSizeScaler.t:NADH3_pos1" spec="ScaleOperator" parameter="@popSize.t:NADH3_pos1" weight="3.0"/>

<operator id="CoalescentConstantTreeScaler.t:NADH3_pos2" spec="ScaleOperator" scaleFactor="0.5" tree="@Tree.t:NADH3_pos2" weight="3.0"/>

<operator id="CoalescentConstantTreeRootScaler.t:NADH3_pos2" spec="ScaleOperator" rootOnly="true" scaleFactor="0.5" tree="@Tree.t:NADH3_pos2" weight="3.0"/>

<operator id="CoalescentConstantUniformOperator.t:NADH3_pos2" spec="Uniform" tree="@Tree.t:NADH3_pos2" weight="30.0"/>

<operator id="CoalescentConstantSubtreeSlide.t:NADH3_pos2" spec="SubtreeSlide" tree="@Tree.t:NADH3_pos2" weight="15.0"/>

<operator id="CoalescentConstantNarrow.t:NADH3_pos2" spec="Exchange" tree="@Tree.t:NADH3_pos2" weight="15.0"/>

<operator id="CoalescentConstantWide.t:NADH3_pos2" spec="Exchange" isNarrow="false" tree="@Tree.t:NADH3_pos2" weight="3.0"/>

<operator id="CoalescentConstantWilsonBalding.t:NADH3_pos2" spec="WilsonBalding" tree="@Tree.t:NADH3_pos2" weight="3.0"/>

<operator id="PopSizeScaler.t:NADH3_pos2" spec="ScaleOperator" parameter="@popSize.t:NADH3_pos2" weight="3.0"/>

<operator id="CoalescentConstantTreeScaler.t:NADH3_pos3" spec="ScaleOperator" scaleFactor="0.5" tree="@Tree.t:NADH3_pos3" weight="3.0"/>

<operator id="CoalescentConstantTreeRootScaler.t:NADH3_pos3" spec="ScaleOperator" rootOnly="true" scaleFactor="0.5" tree="@Tree.t:NADH3_pos3" weight="3.0"/>

<operator id="CoalescentConstantUniformOperator.t:NADH3_pos3" spec="Uniform" tree="@Tree.t:NADH3_pos3" weight="30.0"/>

<operator id="CoalescentConstantSubtreeSlide.t:NADH3_pos3" spec="SubtreeSlide" tree="@Tree.t:NADH3_pos3" weight="15.0"/>

<operator id="CoalescentConstantNarrow.t:NADH3_pos3" spec="Exchange" tree="@Tree.t:NADH3_pos3" weight="15.0"/>

<operator id="CoalescentConstantWide.t:NADH3_pos3" spec="Exchange" isNarrow="false" tree="@Tree.t:NADH3_pos3" weight="3.0"/>

<operator id="CoalescentConstantWilsonBalding.t:NADH3_pos3" spec="WilsonBalding" tree="@Tree.t:NADH3_pos3" weight="3.0"/>

<operator id="PopSizeScaler.t:NADH3_pos3" spec="ScaleOperator" parameter="@popSize.t:NADH3_pos3" weight="3.0"/>

<operator id="CoalescentConstantTreeScaler.t:NADH4L_pos1" spec="ScaleOperator" scaleFactor="0.5" tree="@Tree.t:NADH4L_pos1" weight="3.0"/>

<operator id="CoalescentConstantTreeRootScaler.t:NADH4L_pos1" spec="ScaleOperator" rootOnly="true" scaleFactor="0.5" tree="@Tree.t:NADH4L_pos1" weight="3.0"/>

<operator id="CoalescentConstantUniformOperator.t:NADH4L_pos1" spec="Uniform" tree="@Tree.t:NADH4L_pos1" weight="30.0"/>

<operator id="CoalescentConstantSubtreeSlide.t:NADH4L_pos1" spec="SubtreeSlide" tree="@Tree.t:NADH4L_pos1" weight="15.0"/>

<operator id="CoalescentConstantNarrow.t:NADH4L_pos1" spec="Exchange" tree="@Tree.t:NADH4L_pos1" weight="15.0"/>

<operator id="CoalescentConstantWide.t:NADH4L_pos1" spec="Exchange" isNarrow="false" tree="@Tree.t:NADH4L_pos1" weight="3.0"/>

<operator id="CoalescentConstantWilsonBalding.t:NADH4L_pos1" spec="WilsonBalding" tree="@Tree.t:NADH4L_pos1" weight="3.0"/>

<operator id="PopSizeScaler.t:NADH4L_pos1" spec="ScaleOperator" parameter="@popSize.t:NADH4L_pos1" weight="3.0"/>

<operator id="CoalescentConstantTreeScaler.t:NADH4L_pos2" spec="ScaleOperator" scaleFactor="0.5" tree="@Tree.t:NADH4L_pos2" weight="3.0"/>

<operator id="CoalescentConstantTreeRootScaler.t:NADH4L_pos2" spec="ScaleOperator" rootOnly="true" scaleFactor="0.5" tree="@Tree.t:NADH4L_pos2" weight="3.0"/>

<operator id="CoalescentConstantUniformOperator.t:NADH4L_pos2" spec="Uniform" tree="@Tree.t:NADH4L_pos2" weight="30.0"/>

<operator id="CoalescentConstantSubtreeSlide.t:NADH4L_pos2" spec="SubtreeSlide" tree="@Tree.t:NADH4L_pos2" weight="15.0"/>

<operator id="CoalescentConstantNarrow.t:NADH4L_pos2" spec="Exchange" tree="@Tree.t:NADH4L_pos2" weight="15.0"/>

<operator id="CoalescentConstantWide.t:NADH4L_pos2" spec="Exchange" isNarrow="false" tree="@Tree.t:NADH4L_pos2" weight="3.0"/>

<operator id="CoalescentConstantWilsonBalding.t:NADH4L_pos2" spec="WilsonBalding" tree="@Tree.t:NADH4L_pos2" weight="3.0"/>

<operator id="PopSizeScaler.t:NADH4L_pos2" spec="ScaleOperator" parameter="@popSize.t:NADH4L_pos2" weight="3.0"/>

<operator id="CoalescentConstantTreeScaler.t:NADH4L_pos3" spec="ScaleOperator" scaleFactor="0.5" tree="@Tree.t:NADH4L_pos3" weight="3.0"/>

<operator id="CoalescentConstantTreeRootScaler.t:NADH4L_pos3" spec="ScaleOperator" rootOnly="true" scaleFactor="0.5" tree="@Tree.t:NADH4L_pos3" weight="3.0"/>

<operator id="CoalescentConstantUniformOperator.t:NADH4L_pos3" spec="Uniform" tree="@Tree.t:NADH4L_pos3" weight="30.0"/>

<operator id="CoalescentConstantSubtreeSlide.t:NADH4L_pos3" spec="SubtreeSlide" tree="@Tree.t:NADH4L_pos3" weight="15.0"/>

<operator id="CoalescentConstantNarrow.t:NADH4L_pos3" spec="Exchange" tree="@Tree.t:NADH4L_pos3" weight="15.0"/>

<operator id="CoalescentConstantWide.t:NADH4L_pos3" spec="Exchange" isNarrow="false" tree="@Tree.t:NADH4L_pos3" weight="3.0"/>

<operator id="CoalescentConstantWilsonBalding.t:NADH4L_pos3" spec="WilsonBalding" tree="@Tree.t:NADH4L_pos3" weight="3.0"/>

<operator id="PopSizeScaler.t:NADH4L_pos3" spec="ScaleOperator" parameter="@popSize.t:NADH4L_pos3" weight="3.0"/>

<operator id="CoalescentConstantTreeScaler.t:NADH4_pos1" spec="ScaleOperator" scaleFactor="0.5" tree="@Tree.t:NADH4_pos1" weight="3.0"/>

<operator id="CoalescentConstantTreeRootScaler.t:NADH4_pos1" spec="ScaleOperator" rootOnly="true" scaleFactor="0.5" tree="@Tree.t:NADH4_pos1" weight="3.0"/>

<operator id="CoalescentConstantUniformOperator.t:NADH4_pos1" spec="Uniform" tree="@Tree.t:NADH4_pos1" weight="30.0"/>

<operator id="CoalescentConstantSubtreeSlide.t:NADH4_pos1" spec="SubtreeSlide" tree="@Tree.t:NADH4_pos1" weight="15.0"/>

<operator id="CoalescentConstantNarrow.t:NADH4_pos1" spec="Exchange" tree="@Tree.t:NADH4_pos1" weight="15.0"/>

<operator id="CoalescentConstantWide.t:NADH4_pos1" spec="Exchange" isNarrow="false" tree="@Tree.t:NADH4_pos1" weight="3.0"/>

<operator id="CoalescentConstantWilsonBalding.t:NADH4_pos1" spec="WilsonBalding" tree="@Tree.t:NADH4_pos1" weight="3.0"/>

<operator id="PopSizeScaler.t:NADH4_pos1" spec="ScaleOperator" parameter="@popSize.t:NADH4_pos1" weight="3.0"/>

<operator id="CoalescentConstantTreeScaler.t:NADH4_pos2" spec="ScaleOperator" scaleFactor="0.5" tree="@Tree.t:NADH4_pos2" weight="3.0"/>

<operator id="CoalescentConstantTreeRootScaler.t:NADH4_pos2" spec="ScaleOperator" rootOnly="true" scaleFactor="0.5" tree="@Tree.t:NADH4_pos2" weight="3.0"/>

<operator id="CoalescentConstantUniformOperator.t:NADH4_pos2" spec="Uniform" tree="@Tree.t:NADH4_pos2" weight="30.0"/>

<operator id="CoalescentConstantSubtreeSlide.t:NADH4_pos2" spec="SubtreeSlide" tree="@Tree.t:NADH4_pos2" weight="15.0"/>

<operator id="CoalescentConstantNarrow.t:NADH4_pos2" spec="Exchange" tree="@Tree.t:NADH4_pos2" weight="15.0"/>

<operator id="CoalescentConstantWide.t:NADH4_pos2" spec="Exchange" isNarrow="false" tree="@Tree.t:NADH4_pos2" weight="3.0"/>

<operator id="CoalescentConstantWilsonBalding.t:NADH4_pos2" spec="WilsonBalding" tree="@Tree.t:NADH4_pos2" weight="3.0"/>

<operator id="PopSizeScaler.t:NADH4_pos2" spec="ScaleOperator" parameter="@popSize.t:NADH4_pos2" weight="3.0"/>

<operator id="CoalescentConstantTreeScaler.t:NADH4_pos3" spec="ScaleOperator" scaleFactor="0.5" tree="@Tree.t:NADH4_pos3" weight="3.0"/>

<operator id="CoalescentConstantTreeRootScaler.t:NADH4_pos3" spec="ScaleOperator" rootOnly="true" scaleFactor="0.5" tree="@Tree.t:NADH4_pos3" weight="3.0"/>

<operator id="CoalescentConstantUniformOperator.t:NADH4_pos3" spec="Uniform" tree="@Tree.t:NADH4_pos3" weight="30.0"/>

<operator id="CoalescentConstantSubtreeSlide.t:NADH4_pos3" spec="SubtreeSlide" tree="@Tree.t:NADH4_pos3" weight="15.0"/>

<operator id="CoalescentConstantNarrow.t:NADH4_pos3" spec="Exchange" tree="@Tree.t:NADH4_pos3" weight="15.0"/>

<operator id="CoalescentConstantWide.t:NADH4_pos3" spec="Exchange" isNarrow="false" tree="@Tree.t:NADH4_pos3" weight="3.0"/>

<operator id="CoalescentConstantWilsonBalding.t:NADH4_pos3" spec="WilsonBalding" tree="@Tree.t:NADH4_pos3" weight="3.0"/>

<operator id="PopSizeScaler.t:NADH4_pos3" spec="ScaleOperator" parameter="@popSize.t:NADH4_pos3" weight="3.0"/>

<operator id="CoalescentConstantTreeScaler.t:NADH5_pos1" spec="ScaleOperator" scaleFactor="0.5" tree="@Tree.t:NADH5_pos1" weight="3.0"/>

<operator id="CoalescentConstantTreeRootScaler.t:NADH5_pos1" spec="ScaleOperator" rootOnly="true" scaleFactor="0.5" tree="@Tree.t:NADH5_pos1" weight="3.0"/>

<operator id="CoalescentConstantUniformOperator.t:NADH5_pos1" spec="Uniform" tree="@Tree.t:NADH5_pos1" weight="30.0"/>

<operator id="CoalescentConstantSubtreeSlide.t:NADH5_pos1" spec="SubtreeSlide" tree="@Tree.t:NADH5_pos1" weight="15.0"/>

<operator id="CoalescentConstantNarrow.t:NADH5_pos1" spec="Exchange" tree="@Tree.t:NADH5_pos1" weight="15.0"/>

<operator id="CoalescentConstantWide.t:NADH5_pos1" spec="Exchange" isNarrow="false" tree="@Tree.t:NADH5_pos1" weight="3.0"/>

<operator id="CoalescentConstantWilsonBalding.t:NADH5_pos1" spec="WilsonBalding" tree="@Tree.t:NADH5_pos1" weight="3.0"/>

<operator id="PopSizeScaler.t:NADH5_pos1" spec="ScaleOperator" parameter="@popSize.t:NADH5_pos1" weight="3.0"/>

<operator id="CoalescentConstantTreeScaler.t:NADH5_pos2" spec="ScaleOperator" scaleFactor="0.5" tree="@Tree.t:NADH5_pos2" weight="3.0"/>

<operator id="CoalescentConstantTreeRootScaler.t:NADH5_pos2" spec="ScaleOperator" rootOnly="true" scaleFactor="0.5" tree="@Tree.t:NADH5_pos2" weight="3.0"/>

<operator id="CoalescentConstantUniformOperator.t:NADH5_pos2" spec="Uniform" tree="@Tree.t:NADH5_pos2" weight="30.0"/>

<operator id="CoalescentConstantSubtreeSlide.t:NADH5_pos2" spec="SubtreeSlide" tree="@Tree.t:NADH5_pos2" weight="15.0"/>

<operator id="CoalescentConstantNarrow.t:NADH5_pos2" spec="Exchange" tree="@Tree.t:NADH5_pos2" weight="15.0"/>

<operator id="CoalescentConstantWide.t:NADH5_pos2" spec="Exchange" isNarrow="false" tree="@Tree.t:NADH5_pos2" weight="3.0"/>

<operator id="CoalescentConstantWilsonBalding.t:NADH5_pos2" spec="WilsonBalding" tree="@Tree.t:NADH5_pos2" weight="3.0"/>

<operator id="PopSizeScaler.t:NADH5_pos2" spec="ScaleOperator" parameter="@popSize.t:NADH5_pos2" weight="3.0"/>

<operator id="CoalescentConstantTreeScaler.t:NADH5_pos3" spec="ScaleOperator" scaleFactor="0.5" tree="@Tree.t:NADH5_pos3" weight="3.0"/>

<operator id="CoalescentConstantTreeRootScaler.t:NADH5_pos3" spec="ScaleOperator" rootOnly="true" scaleFactor="0.5" tree="@Tree.t:NADH5_pos3" weight="3.0"/>

<operator id="CoalescentConstantUniformOperator.t:NADH5_pos3" spec="Uniform" tree="@Tree.t:NADH5_pos3" weight="30.0"/>

<operator id="CoalescentConstantSubtreeSlide.t:NADH5_pos3" spec="SubtreeSlide" tree="@Tree.t:NADH5_pos3" weight="15.0"/>

<operator id="CoalescentConstantNarrow.t:NADH5_pos3" spec="Exchange" tree="@Tree.t:NADH5_pos3" weight="15.0"/>

<operator id="CoalescentConstantWide.t:NADH5_pos3" spec="Exchange" isNarrow="false" tree="@Tree.t:NADH5_pos3" weight="3.0"/>

<operator id="CoalescentConstantWilsonBalding.t:NADH5_pos3" spec="WilsonBalding" tree="@Tree.t:NADH5_pos3" weight="3.0"/>

<operator id="PopSizeScaler.t:NADH5_pos3" spec="ScaleOperator" parameter="@popSize.t:NADH5_pos3" weight="3.0"/>

<operator id="CoalescentConstantTreeScaler.t:NADH6_pos1" spec="ScaleOperator" scaleFactor="0.5" tree="@Tree.t:NADH6_pos1" weight="3.0"/>

<operator id="CoalescentConstantTreeRootScaler.t:NADH6_pos1" spec="ScaleOperator" rootOnly="true" scaleFactor="0.5" tree="@Tree.t:NADH6_pos1" weight="3.0"/>

<operator id="CoalescentConstantUniformOperator.t:NADH6_pos1" spec="Uniform" tree="@Tree.t:NADH6_pos1" weight="30.0"/>

<operator id="CoalescentConstantSubtreeSlide.t:NADH6_pos1" spec="SubtreeSlide" tree="@Tree.t:NADH6_pos1" weight="15.0"/>

<operator id="CoalescentConstantNarrow.t:NADH6_pos1" spec="Exchange" tree="@Tree.t:NADH6_pos1" weight="15.0"/>

<operator id="CoalescentConstantWide.t:NADH6_pos1" spec="Exchange" isNarrow="false" tree="@Tree.t:NADH6_pos1" weight="3.0"/>

<operator id="CoalescentConstantWilsonBalding.t:NADH6_pos1" spec="WilsonBalding" tree="@Tree.t:NADH6_pos1" weight="3.0"/>

<operator id="PopSizeScaler.t:NADH6_pos1" spec="ScaleOperator" parameter="@popSize.t:NADH6_pos1" weight="3.0"/>

<operator id="CoalescentConstantTreeScaler.t:NADH6_pos2" spec="ScaleOperator" scaleFactor="0.5" tree="@Tree.t:NADH6_pos2" weight="3.0"/>

<operator id="CoalescentConstantTreeRootScaler.t:NADH6_pos2" spec="ScaleOperator" rootOnly="true" scaleFactor="0.5" tree="@Tree.t:NADH6_pos2" weight="3.0"/>

<operator id="CoalescentConstantUniformOperator.t:NADH6_pos2" spec="Uniform" tree="@Tree.t:NADH6_pos2" weight="30.0"/>

<operator id="CoalescentConstantSubtreeSlide.t:NADH6_pos2" spec="SubtreeSlide" tree="@Tree.t:NADH6_pos2" weight="15.0"/>

<operator id="CoalescentConstantNarrow.t:NADH6_pos2" spec="Exchange" tree="@Tree.t:NADH6_pos2" weight="15.0"/>

<operator id="CoalescentConstantWide.t:NADH6_pos2" spec="Exchange" isNarrow="false" tree="@Tree.t:NADH6_pos2" weight="3.0"/>

<operator id="CoalescentConstantWilsonBalding.t:NADH6_pos2" spec="WilsonBalding" tree="@Tree.t:NADH6_pos2" weight="3.0"/>

<operator id="PopSizeScaler.t:NADH6_pos2" spec="ScaleOperator" parameter="@popSize.t:NADH6_pos2" weight="3.0"/>

<operator id="CoalescentConstantTreeScaler.t:NADH6_pos3" spec="ScaleOperator" scaleFactor="0.5" tree="@Tree.t:NADH6_pos3" weight="3.0"/>

<operator id="CoalescentConstantTreeRootScaler.t:NADH6_pos3" spec="ScaleOperator" rootOnly="true" scaleFactor="0.5" tree="@Tree.t:NADH6_pos3" weight="3.0"/>

<operator id="CoalescentConstantUniformOperator.t:NADH6_pos3" spec="Uniform" tree="@Tree.t:NADH6_pos3" weight="30.0"/>

<operator id="CoalescentConstantSubtreeSlide.t:NADH6_pos3" spec="SubtreeSlide" tree="@Tree.t:NADH6_pos3" weight="15.0"/>

<operator id="CoalescentConstantNarrow.t:NADH6_pos3" spec="Exchange" tree="@Tree.t:NADH6_pos3" weight="15.0"/>

<operator id="CoalescentConstantWide.t:NADH6_pos3" spec="Exchange" isNarrow="false" tree="@Tree.t:NADH6_pos3" weight="3.0"/>

<operator id="CoalescentConstantWilsonBalding.t:NADH6_pos3" spec="WilsonBalding" tree="@Tree.t:NADH6_pos3" weight="3.0"/>

<operator id="PopSizeScaler.t:NADH6_pos3" spec="ScaleOperator" parameter="@popSize.t:NADH6_pos3" weight="3.0"/>

<operator id="CoalescentConstantTreeScaler.t:atp6_pos1" spec="ScaleOperator" scaleFactor="0.5" tree="@Tree.t:atp6_pos1" weight="3.0"/>

<operator id="CoalescentConstantTreeRootScaler.t:atp6_pos1" spec="ScaleOperator" rootOnly="true" scaleFactor="0.5" tree="@Tree.t:atp6_pos1" weight="3.0"/>

<operator id="CoalescentConstantUniformOperator.t:atp6_pos1" spec="Uniform" tree="@Tree.t:atp6_pos1" weight="30.0"/>

<operator id="CoalescentConstantSubtreeSlide.t:atp6_pos1" spec="SubtreeSlide" tree="@Tree.t:atp6_pos1" weight="15.0"/>

<operator id="CoalescentConstantNarrow.t:atp6_pos1" spec="Exchange" tree="@Tree.t:atp6_pos1" weight="15.0"/>

<operator id="CoalescentConstantWide.t:atp6_pos1" spec="Exchange" isNarrow="false" tree="@Tree.t:atp6_pos1" weight="3.0"/>

<operator id="CoalescentConstantWilsonBalding.t:atp6_pos1" spec="WilsonBalding" tree="@Tree.t:atp6_pos1" weight="3.0"/>

<operator id="PopSizeScaler.t:atp6_pos1" spec="ScaleOperator" parameter="@popSize.t:atp6_pos1" weight="3.0"/>

<operator id="CoalescentConstantTreeScaler.t:atp6_pos2" spec="ScaleOperator" scaleFactor="0.5" tree="@Tree.t:atp6_pos2" weight="3.0"/>

<operator id="CoalescentConstantTreeRootScaler.t:atp6_pos2" spec="ScaleOperator" rootOnly="true" scaleFactor="0.5" tree="@Tree.t:atp6_pos2" weight="3.0"/>

<operator id="CoalescentConstantUniformOperator.t:atp6_pos2" spec="Uniform" tree="@Tree.t:atp6_pos2" weight="30.0"/>

<operator id="CoalescentConstantSubtreeSlide.t:atp6_pos2" spec="SubtreeSlide" tree="@Tree.t:atp6_pos2" weight="15.0"/>

<operator id="CoalescentConstantNarrow.t:atp6_pos2" spec="Exchange" tree="@Tree.t:atp6_pos2" weight="15.0"/>

<operator id="CoalescentConstantWide.t:atp6_pos2" spec="Exchange" isNarrow="false" tree="@Tree.t:atp6_pos2" weight="3.0"/>

<operator id="CoalescentConstantWilsonBalding.t:atp6_pos2" spec="WilsonBalding" tree="@Tree.t:atp6_pos2" weight="3.0"/>

<operator id="PopSizeScaler.t:atp6_pos2" spec="ScaleOperator" parameter="@popSize.t:atp6_pos2" weight="3.0"/>

<operator id="CoalescentConstantTreeScaler.t:atp6_pos3" spec="ScaleOperator" scaleFactor="0.5" tree="@Tree.t:atp6_pos3" weight="3.0"/>

<operator id="CoalescentConstantTreeRootScaler.t:atp6_pos3" spec="ScaleOperator" rootOnly="true" scaleFactor="0.5" tree="@Tree.t:atp6_pos3" weight="3.0"/>

<operator id="CoalescentConstantUniformOperator.t:atp6_pos3" spec="Uniform" tree="@Tree.t:atp6_pos3" weight="30.0"/>

<operator id="CoalescentConstantSubtreeSlide.t:atp6_pos3" spec="SubtreeSlide" tree="@Tree.t:atp6_pos3" weight="15.0"/>

<operator id="CoalescentConstantNarrow.t:atp6_pos3" spec="Exchange" tree="@Tree.t:atp6_pos3" weight="15.0"/>

<operator id="CoalescentConstantWide.t:atp6_pos3" spec="Exchange" isNarrow="false" tree="@Tree.t:atp6_pos3" weight="3.0"/>

<operator id="CoalescentConstantWilsonBalding.t:atp6_pos3" spec="WilsonBalding" tree="@Tree.t:atp6_pos3" weight="3.0"/>

<operator id="PopSizeScaler.t:atp6_pos3" spec="ScaleOperator" parameter="@popSize.t:atp6_pos3" weight="3.0"/>

<operator id="proportionInvariantScaler.s:Mytilidae27sq12PCGs" spec="ScaleOperator" parameter="@proportionInvariant.s:Mytilidae27sq12PCGs" scaleFactor="0.5" weight="0.1"/>

<operator id="proportionInvariantScaler.s:atp6_pos3" spec="ScaleOperator" parameter="@proportionInvariant.s:atp6_pos3" scaleFactor="0.5" weight="0.1"/>

<operator id="proportionInvariantScaler.s:COX1_pos1" spec="ScaleOperator" parameter="@proportionInvariant.s:COX1_pos1" scaleFactor="0.5" weight="0.1"/>

<operator id="proportionInvariantScaler.s:COX1_pos2" spec="ScaleOperator" parameter="@proportionInvariant.s:COX1_pos2" scaleFactor="0.5" weight="0.1"/>

<operator id="proportionInvariantScaler.s:COX1_pos3" spec="ScaleOperator" parameter="@proportionInvariant.s:COX1_pos3" scaleFactor="0.5" weight="0.1"/>

<operator id="proportionInvariantScaler.s:COX2_pos1" spec="ScaleOperator" parameter="@proportionInvariant.s:COX2_pos1" scaleFactor="0.5" weight="0.1"/>

<operator id="proportionInvariantScaler.s:COX2_pos2" spec="ScaleOperator" parameter="@proportionInvariant.s:COX2_pos2" scaleFactor="0.5" weight="0.1"/>

<operator id="RateACScaler.s:COX2_pos2" spec="ScaleOperator" parameter="@rateAC.s:COX2_pos2" scaleFactor="0.5" weight="0.1"/>

<operator id="RateAGScaler.s:COX2_pos2" spec="ScaleOperator" parameter="@rateAG.s:COX2_pos2" scaleFactor="0.5" weight="0.1"/>

<operator id="RateATScaler.s:COX2_pos2" spec="ScaleOperator" parameter="@rateAT.s:COX2_pos2" scaleFactor="0.5" weight="0.1"/>

<operator id="RateCGScaler.s:COX2_pos2" spec="ScaleOperator" parameter="@rateCG.s:COX2_pos2" scaleFactor="0.5" weight="0.1"/>

<operator id="RateGTScaler.s:COX2_pos2" spec="ScaleOperator" parameter="@rateGT.s:COX2_pos2" scaleFactor="0.5" weight="0.1"/>

<operator id="proportionInvariantScaler.s:COX2_pos3" spec="ScaleOperator" parameter="@proportionInvariant.s:COX2_pos3" scaleFactor="0.5" weight="0.1"/>

<operator id="proportionInvariantScaler.s:COX3_pos1" spec="ScaleOperator" parameter="@proportionInvariant.s:COX3_pos1" scaleFactor="0.5" weight="0.1"/>

<operator id="proportionInvariantScaler.s:COX3_pos2" spec="ScaleOperator" parameter="@proportionInvariant.s:COX3_pos2" scaleFactor="0.5" weight="0.1"/>

<operator id="proportionInvariantScaler.s:COX3_pos3" spec="ScaleOperator" parameter="@proportionInvariant.s:COX3_pos3" scaleFactor="0.5" weight="0.1"/>

<operator id="proportionInvariantScaler.s:CYTB_pos1" spec="ScaleOperator" parameter="@proportionInvariant.s:CYTB_pos1" scaleFactor="0.5" weight="0.1"/>

<operator id="proportionInvariantScaler.s:CYTB_pos2" spec="ScaleOperator" parameter="@proportionInvariant.s:CYTB_pos2" scaleFactor="0.5" weight="0.1"/>

<operator id="proportionInvariantScaler.s:CYTB_pos3" spec="ScaleOperator" parameter="@proportionInvariant.s:CYTB_pos3" scaleFactor="0.5" weight="0.1"/>

<operator id="proportionInvariantScaler.s:NADH1_pos1" spec="ScaleOperator" parameter="@proportionInvariant.s:NADH1_pos1" scaleFactor="0.5" weight="0.1"/>

<operator id="proportionInvariantScaler.s:NADH1_pos2" spec="ScaleOperator" parameter="@proportionInvariant.s:NADH1_pos2" scaleFactor="0.5" weight="0.1"/>

<operator id="proportionInvariantScaler.s:NADH1_pos3" spec="ScaleOperator" parameter="@proportionInvariant.s:NADH1_pos3" scaleFactor="0.5" weight="0.1"/>

<operator id="proportionInvariantScaler.s:NADH2_pos1" spec="ScaleOperator" parameter="@proportionInvariant.s:NADH2_pos1" scaleFactor="0.5" weight="0.1"/>

<operator id="proportionInvariantScaler.s:NADH2_pos2" spec="ScaleOperator" parameter="@proportionInvariant.s:NADH2_pos2" scaleFactor="0.5" weight="0.1"/>

<operator id="proportionInvariantScaler.s:NADH2_pos3" spec="ScaleOperator" parameter="@proportionInvariant.s:NADH2_pos3" scaleFactor="0.5" weight="0.1"/>

<operator id="proportionInvariantScaler.s:NADH3_pos1" spec="ScaleOperator" parameter="@proportionInvariant.s:NADH3_pos1" scaleFactor="0.5" weight="0.1"/>

```
    <operator id="proportionInvariantScaler.s:NADH3_pos2" spec="ScaleOperator"
parameter="@proportionInvariant.s:NADH3_pos2" scaleFactor="0.5" weight="0.1"/>

    <operator id="proportionInvariantScaler.s:NADH3_pos3" spec="ScaleOperator"
parameter="@proportionInvariant.s:NADH3_pos3" scaleFactor="0.5" weight="0.1"/>

    <operator id="proportionInvariantScaler.s:NADH4_pos1" spec="ScaleOperator"
parameter="@proportionInvariant.s:NADH4_pos1" scaleFactor="0.5" weight="0.1"/>

    <operator id="proportionInvariantScaler.s:NADH4_pos2" spec="ScaleOperator"
parameter="@proportionInvariant.s:NADH4_pos2" scaleFactor="0.5" weight="0.1"/>

    <operator id="proportionInvariantScaler.s:NADH4_pos3" spec="ScaleOperator"
parameter="@proportionInvariant.s:NADH4_pos3" scaleFactor="0.5" weight="0.1"/>

    <operator id="proportionInvariantScaler.s:NADH4L_pos1" spec="ScaleOperator"
parameter="@proportionInvariant.s:NADH4L_pos1" scaleFactor="0.5" weight="0.1"/>

    <operator id="proportionInvariantScaler.s:NADH4L_pos2" spec="ScaleOperator"
parameter="@proportionInvariant.s:NADH4L_pos2" scaleFactor="0.5" weight="0.1"/>

    <operator id="proportionInvariantScaler.s:NADH4L_pos3" spec="ScaleOperator"
parameter="@proportionInvariant.s:NADH4L_pos3" scaleFactor="0.5" weight="0.1"/>

    <operator id="proportionInvariantScaler.s:NADH5_pos1" spec="ScaleOperator"
parameter="@proportionInvariant.s:NADH5_pos1" scaleFactor="0.5" weight="0.1"/>

    <operator id="proportionInvariantScaler.s:NADH5_pos2" spec="ScaleOperator"
parameter="@proportionInvariant.s:NADH5_pos2" scaleFactor="0.5" weight="0.1"/>

    <operator id="proportionInvariantScaler.s:NADH5_pos3" spec="ScaleOperator"
parameter="@proportionInvariant.s:NADH5_pos3" scaleFactor="0.5" weight="0.1"/>

    <operator id="proportionInvariantScaler.s:NADH6_pos1" spec="ScaleOperator"
parameter="@proportionInvariant.s:NADH6_pos1" scaleFactor="0.5" weight="0.1"/>

    <operator id="proportionInvariantScaler.s:NADH6_pos2" spec="ScaleOperator"
parameter="@proportionInvariant.s:NADH6_pos2" scaleFactor="0.5" weight="0.1"/>

    <operator id="proportionInvariantScaler.s:NADH6_pos3" spec="ScaleOperator"
parameter="@proportionInvariant.s:NADH6_pos3" scaleFactor="0.5" weight="0.1"/>

    <operator id="RateACScaler.s:Mytilidae27sq12PCGs" spec="ScaleOperator"
parameter="@rateAC.s:Mytilidae27sq12PCGs" scaleFactor="0.5" weight="0.1"/>

    <operator id="RateAGScaler.s:Mytilidae27sq12PCGs" spec="ScaleOperator"
parameter="@rateAG.s:Mytilidae27sq12PCGs" scaleFactor="0.5" weight="0.1"/>

    <operator id="RateATScaler.s:Mytilidae27sq12PCGs" spec="ScaleOperator"
parameter="@rateAT.s:Mytilidae27sq12PCGs" scaleFactor="0.5" weight="0.1"/>

    <operator id="RateCGScaler.s:Mytilidae27sq12PCGs" spec="ScaleOperator"
parameter="@rateCG.s:Mytilidae27sq12PCGs" scaleFactor="0.5" weight="0.1"/>

    <operator id="RateGTScaler.s:Mytilidae27sq12PCGs" spec="ScaleOperator"
parameter="@rateGT.s:Mytilidae27sq12PCGs" scaleFactor="0.5" weight="0.1"/>

    <operator id="FrequenciesExchanger.s:atp6_pos1" spec="DeltaExchangeOperator" delta="0.01" weight="0.1">
        <parameter idref="freqParameter.s:atp6_pos1"/>
    </operator>
```

```
    <operator id="FrequenciesExchanger.s:COX1_pos1" spec="DeltaExchangeOperator" delta="0.01"
weight="0.1">
        <parameter idref="freqParameter.s:COX1_pos1"/>
    </operator>

    <operator id="FrequenciesExchanger.s:COX1_pos3" spec="DeltaExchangeOperator" delta="0.01"
weight="0.1">
        <parameter idref="freqParameter.s:COX1_pos3"/>
    </operator>

    <operator id="FrequenciesExchanger.s:NADH5_pos3" spec="DeltaExchangeOperator" delta="0.01"
weight="0.1">
        <parameter idref="freqParameter.s:NADH5_pos3"/>
    </operator>

    <operator id="FrequenciesExchanger.s:NADH2_pos3" spec="DeltaExchangeOperator" delta="0.01"
weight="0.1">
        <parameter idref="freqParameter.s:NADH2_pos3"/>
    </operator>

    <operator id="FrequenciesExchanger.s:NADH3_pos3" spec="DeltaExchangeOperator" delta="0.01"
weight="0.1">
        <parameter idref="freqParameter.s:NADH3_pos3"/>
    </operator>

    <operator id="FrequenciesExchanger.s:NADH1_pos1" spec="DeltaExchangeOperator" delta="0.01"
weight="0.1">
        <parameter idref="freqParameter.s:NADH1_pos1"/>
    </operator>

    <operator id="FrequenciesExchanger.s:NADH6_pos1" spec="DeltaExchangeOperator" delta="0.01"
weight="0.1">
        <parameter idref="freqParameter.s:NADH6_pos1"/>
    </operator>

    <operator id="FrequenciesExchanger.s:NADH1_pos2" spec="DeltaExchangeOperator" delta="0.01"
weight="0.1">
        <parameter idref="freqParameter.s:NADH1_pos2"/>
    </operator>

    <operator id="FrequenciesExchanger.s:NADH4L_pos1" spec="DeltaExchangeOperator" delta="0.01"
weight="0.1">
        <parameter idref="freqParameter.s:NADH4L_pos1"/>
    </operator>

    <operator id="FrequenciesExchanger.s:CYTB_pos1" spec="DeltaExchangeOperator" delta="0.01"
weight="0.1">
        <parameter idref="freqParameter.s:CYTB_pos1"/>
    </operator>

    <operator id="FrequenciesExchanger.s:atp6_pos2" spec="DeltaExchangeOperator" delta="0.01" weight="0.1">
        <parameter idref="freqParameter.s:atp6_pos2"/>
    </operator>

    <operator id="FrequenciesExchanger.s:NADH6_pos3" spec="DeltaExchangeOperator" delta="0.01"
weight="0.1">
        <parameter idref="freqParameter.s:NADH6_pos3"/>
    </operator>

    <operator id="FrequenciesExchanger.s:NADH2_pos2" spec="DeltaExchangeOperator" delta="0.01"
weight="0.1">
        <parameter idref="freqParameter.s:NADH2_pos2"/>
    </operator>
```

<operator id="FrequenciesExchanger.s:NADH6_pos2" spec="DeltaExchangeOperator" delta="0.01" weight="0.1">
<parameter idref="freqParameter.s:NADH6_pos2"/>
</operator>

<operator id="FrequenciesExchanger.s:NADH4L_pos2" spec="DeltaExchangeOperator" delta="0.01" weight="0.1">
<parameter idref="freqParameter.s:NADH4L_pos2"/>
</operator>

<operator id="FrequenciesExchanger.s:NADH4_pos3" spec="DeltaExchangeOperator" delta="0.01" weight="0.1">
<parameter idref="freqParameter.s:NADH4_pos3"/>
</operator>

<operator id="FrequenciesExchanger.s:NADH5_pos1" spec="DeltaExchangeOperator" delta="0.01" weight="0.1">
<parameter idref="freqParameter.s:NADH5_pos1"/>
</operator>

<operator id="FrequenciesExchanger.s:NADH5_pos2" spec="DeltaExchangeOperator" delta="0.01" weight="0.1">
<parameter idref="freqParameter.s:NADH5_pos2"/>
</operator>

<operator id="FrequenciesExchanger.s:NADH3_pos2" spec="DeltaExchangeOperator" delta="0.01" weight="0.1">
<parameter idref="freqParameter.s:NADH3_pos2"/>
</operator>

<operator id="FrequenciesExchanger.s:Mytilidae27sq12PCGs" spec="DeltaExchangeOperator" delta="0.01" weight="0.1">
<parameter idref="freqParameter.s:Mytilidae27sq12PCGs"/>
</operator>

<operator id="FrequenciesExchanger.s:COX3_pos1" spec="DeltaExchangeOperator" delta="0.01" weight="0.1">
<parameter idref="freqParameter.s:COX3_pos1"/>
</operator>

<operator id="FrequenciesExchanger.s:NADH2_pos1" spec="DeltaExchangeOperator" delta="0.01" weight="0.1">
<parameter idref="freqParameter.s:NADH2_pos1"/>
</operator>

<operator id="FrequenciesExchanger.s:NADH3_pos1" spec="DeltaExchangeOperator" delta="0.01" weight="0.1">
<parameter idref="freqParameter.s:NADH3_pos1"/>
</operator>

<operator id="FrequenciesExchanger.s:NADH1_pos3" spec="DeltaExchangeOperator" delta="0.01" weight="0.1">
<parameter idref="freqParameter.s:NADH1_pos3"/>
</operator>

<operator id="FrequenciesExchanger.s:CYTB_pos2" spec="DeltaExchangeOperator" delta="0.01" weight="0.1">
<parameter idref="freqParameter.s:CYTB_pos2"/>
</operator>

<operator id="FrequenciesExchanger.s:COX2_pos2" spec="DeltaExchangeOperator" delta="0.01" weight="0.1">

```
        <parameter idref="freqParameter.s:COX2_pos2"/>
    </operator>

    <operator id="FrequenciesExchanger.s:COX2_pos3" spec="DeltaExchangeOperator" delta="0.01"
weight="0.1">
        <parameter idref="freqParameter.s:COX2_pos3"/>
    </operator>

    <operator id="FrequenciesExchanger.s:COX3_pos2" spec="DeltaExchangeOperator" delta="0.01"
weight="0.1">
        <parameter idref="freqParameter.s:COX3_pos2"/>
    </operator>

    <operator id="FrequenciesExchanger.s:CYTB_pos3" spec="DeltaExchangeOperator" delta="0.01"
weight="0.1">
        <parameter idref="freqParameter.s:CYTB_pos3"/>
    </operator>

    <operator id="FrequenciesExchanger.s:NADH4_pos2" spec="DeltaExchangeOperator" delta="0.01"
weight="0.1">
        <parameter idref="freqParameter.s:NADH4_pos2"/>
    </operator>

    <operator id="FrequenciesExchanger.s:COX3_pos3" spec="DeltaExchangeOperator" delta="0.01"
weight="0.1">
        <parameter idref="freqParameter.s:COX3_pos3"/>
    </operator>

    <operator id="FrequenciesExchanger.s:NADH4L_pos3" spec="DeltaExchangeOperator" delta="0.01"
weight="0.1">
        <parameter idref="freqParameter.s:NADH4L_pos3"/>
    </operator>

    <operator id="FrequenciesExchanger.s:COX2_pos1" spec="DeltaExchangeOperator" delta="0.01"
weight="0.1">
        <parameter idref="freqParameter.s:COX2_pos1"/>
    </operator>

    <operator id="FrequenciesExchanger.s:NADH4_pos1" spec="DeltaExchangeOperator" delta="0.01"
weight="0.1">
        <parameter idref="freqParameter.s:NADH4_pos1"/>
    </operator>

    <operator id="FrequenciesExchanger.s:atp6_pos3" spec="DeltaExchangeOperator" delta="0.01" weight="0.1">
        <parameter idref="freqParameter.s:atp6_pos3"/>
    </operator>

    <logger id="tracelog" spec="Logger" fileName="Mytilidae27sq-no_pat-pt123ps.log" logEvery="1000"
model="@posterior" sanitiseHeaders="true" sort="smart">
        <log idref="posterior"/>
        <log idref="likelihood"/>
        <log idref="prior"/>
        <log idref="treeLikelihood.NADH6_pos1"/>
        <log id="TreeHeight.t:NADH6_pos1" spec="beast.evolution.tree.TreeHeightLogger"
tree="@Tree.t:NADH6_pos1"/>
        <log idref="treeLikelihood.NADH1_pos1"/>
        <log id="TreeHeight.t:NADH1_pos1" spec="beast.evolution.tree.TreeHeightLogger"
tree="@Tree.t:NADH1_pos1"/>
        <log idref="treeLikelihood.COX2_pos1"/>
        <log id="TreeHeight.t:COX2_pos1" spec="beast.evolution.tree.TreeHeightLogger"
tree="@Tree.t:COX2_pos1"/>
        <log idref="treeLikelihood.NADH1_pos2"/>
```

```
<log id="TreeHeight.t:NADH1_pos2" spec="beast.evolution.tree.TreeHeightLogger"
tree="@Tree.t:NADH1_pos2"/>
<log idref="treeLikelihood.NADH4_pos1"/>
<log id="TreeHeight.t:NADH4_pos1" spec="beast.evolution.tree.TreeHeightLogger"
tree="@Tree.t:NADH4_pos1"/>
<log idref="treeLikelihood.NADH4L_pos3"/>
<log id="TreeHeight.t:NADH4L_pos3" spec="beast.evolution.tree.TreeHeightLogger"
tree="@Tree.t:NADH4L_pos3"/>
<log idref="treeLikelihood.NADH4L_pos1"/>
<log id="TreeHeight.t:NADH4L_pos1" spec="beast.evolution.tree.TreeHeightLogger"
tree="@Tree.t:NADH4L_pos1"/>
<log idref="treeLikelihood.NADH2_pos1"/>
<log id="TreeHeight.t:NADH2_pos1" spec="beast.evolution.tree.TreeHeightLogger"
tree="@Tree.t:NADH2_pos1"/>
<log idref="treeLikelihood.NADH3_pos2"/>
<log id="TreeHeight.t:NADH3_pos2" spec="beast.evolution.tree.TreeHeightLogger"
tree="@Tree.t:NADH3_pos2"/>
<log idref="treeLikelihood.NADH2_pos3"/>
<log id="TreeHeight.t:NADH2_pos3" spec="beast.evolution.tree.TreeHeightLogger"
tree="@Tree.t:NADH2_pos3"/>
<log idref="treeLikelihood.NADH3_pos1"/>
<log id="TreeHeight.t:NADH3_pos1" spec="beast.evolution.tree.TreeHeightLogger"
tree="@Tree.t:NADH3_pos1"/>
<log idref="treeLikelihood.NADH2_pos2"/>
<log id="TreeHeight.t:NADH2_pos2" spec="beast.evolution.tree.TreeHeightLogger"
tree="@Tree.t:NADH2_pos2"/>
<log idref="treeLikelihood.CYTB_pos2"/>
<log id="TreeHeight.t:CYTB_pos2" spec="beast.evolution.tree.TreeHeightLogger"
tree="@Tree.t:CYTB_pos2"/>
<log idref="treeLikelihood.atp6_pos3"/>
<log id="TreeHeight.t:atp6_pos3" spec="beast.evolution.tree.TreeHeightLogger" tree="@Tree.t:atp6_pos3"/>
<log idref="treeLikelihood.COX1_pos1"/>
<log id="TreeHeight.t:COX1_pos1" spec="beast.evolution.tree.TreeHeightLogger"
tree="@Tree.t:COX1_pos1"/>
<log idref="treeLikelihood.CYTB_pos1"/>
<log id="TreeHeight.t:CYTB_pos1" spec="beast.evolution.tree.TreeHeightLogger"
tree="@Tree.t:CYTB_pos1"/>
<log idref="treeLikelihood.NADH4_pos3"/>
<log id="TreeHeight.t:NADH4_pos3" spec="beast.evolution.tree.TreeHeightLogger"
tree="@Tree.t:NADH4_pos3"/>
<log idref="treeLikelihood.COX1_pos3"/>
<log id="TreeHeight.t:COX1_pos3" spec="beast.evolution.tree.TreeHeightLogger"
tree="@Tree.t:COX1_pos3"/>
<log idref="treeLikelihood.COX2_pos2"/>
<log id="TreeHeight.t:COX2_pos2" spec="beast.evolution.tree.TreeHeightLogger"
tree="@Tree.t:COX2_pos2"/>
<log idref="treeLikelihood.CYTB_pos3"/>
<log id="TreeHeight.t:CYTB_pos3" spec="beast.evolution.tree.TreeHeightLogger"
tree="@Tree.t:CYTB_pos3"/>
<log idref="treeLikelihood.NADH5_pos1"/>
<log id="TreeHeight.t:NADH5_pos1" spec="beast.evolution.tree.TreeHeightLogger"
tree="@Tree.t:NADH5_pos1"/>
<log idref="treeLikelihood.atp6_pos1"/>
<log id="TreeHeight.t:atp6_pos1" spec="beast.evolution.tree.TreeHeightLogger" tree="@Tree.t:atp6_pos1"/>
<log idref="treeLikelihood.NADH1_pos3"/>
<log id="TreeHeight.t:NADH1_pos3" spec="beast.evolution.tree.TreeHeightLogger"
tree="@Tree.t:NADH1_pos3"/>
<log idref="treeLikelihood.atp6_pos2"/>
<log id="TreeHeight.t:atp6_pos2" spec="beast.evolution.tree.TreeHeightLogger" tree="@Tree.t:atp6_pos2"/>
<log idref="treeLikelihood.NADH4_pos2"/>
<log id="TreeHeight.t:NADH4_pos2" spec="beast.evolution.tree.TreeHeightLogger"
tree="@Tree.t:NADH4_pos2"/>
<log idref="treeLikelihood.COX3_pos2"/>
```

<log id="TreeHeight.t:COX3_pos2" spec="beast.evolution.tree.TreeHeightLogger" tree="@Tree.t:COX3_pos2"/>
<log idref="treeLikelihood.NADH5_pos3"/>
<log id="TreeHeight.t:NADH5_pos3" spec="beast.evolution.tree.TreeHeightLogger" tree="@Tree.t:NADH5_pos3"/>
<log idref="treeLikelihood.NADH6_pos3"/>
<log id="TreeHeight.t:NADH6_pos3" spec="beast.evolution.tree.TreeHeightLogger" tree="@Tree.t:NADH6_pos3"/>
<log idref="treeLikelihood.COX3_pos1"/>
<log id="TreeHeight.t:COX3_pos1" spec="beast.evolution.tree.TreeHeightLogger" tree="@Tree.t:COX3_pos1"/>
<log idref="treeLikelihood.NADH3_pos3"/>
<log id="TreeHeight.t:NADH3_pos3" spec="beast.evolution.tree.TreeHeightLogger" tree="@Tree.t:NADH3_pos3"/>
<log idref="treeLikelihood.NADH5_pos2"/>
<log id="TreeHeight.t:NADH5_pos2" spec="beast.evolution.tree.TreeHeightLogger" tree="@Tree.t:NADH5_pos2"/>
<log idref="treeLikelihood.NADH6_pos2"/>
<log id="TreeHeight.t:NADH6_pos2" spec="beast.evolution.tree.TreeHeightLogger" tree="@Tree.t:NADH6_pos2"/>
<log idref="treeLikelihood.NADH4L_pos2"/>
<log id="TreeHeight.t:NADH4L_pos2" spec="beast.evolution.tree.TreeHeightLogger" tree="@Tree.t:NADH4L_pos2"/>
<log idref="treeLikelihood.COX1_pos2"/>
<log id="TreeHeight.t:COX1_pos2" spec="beast.evolution.tree.TreeHeightLogger" tree="@Tree.t:COX1_pos2"/>
<log idref="treeLikelihood.COX2_pos3"/>
<log id="TreeHeight.t:COX2_pos3" spec="beast.evolution.tree.TreeHeightLogger" tree="@Tree.t:COX2_pos3"/>
<log idref="treeLikelihood.COX3_pos3"/>
<log id="TreeHeight.t:COX3_pos3" spec="beast.evolution.tree.TreeHeightLogger" tree="@Tree.t:COX3_pos3"/>
<log idref="treeLikelihood.Mytilidae27sq12PCGs"/>
<log id="TreeHeight.t:Mytilidae27sq12PCGs" spec="beast.evolution.tree.TreeHeightLogger" tree="@Tree.t:Mytilidae27sq12PCGs"/>
<log idref="clockRate.c:COX2_pos1"/>
<log idref="clockRate.c:COX3_pos1"/>
<log idref="clockRate.c:NADH2_pos2"/>
<log idref="clockRate.c:NADH4_pos3"/>
<log idref="clockRate.c:NADH4L_pos1"/>
<log idref="clockRate.c:NADH5_pos2"/>
<log idref="clockRate.c:NADH6_pos3"/>
<log idref="clockRate.c:COX1_pos1"/>
<log idref="clockRate.c:COX1_pos3"/>
<log idref="clockRate.c:Mytilidae27sq12PCGs"/>
<log idref="clockRate.c:atp6_pos1"/>
<log idref="clockRate.c:NADH4_pos2"/>
<log idref="clockRate.c:NADH1_pos1"/>
<log idref="clockRate.c:NADH6_pos1"/>
<log idref="clockRate.c:COX1_pos2"/>
<log idref="clockRate.c:NADH4L_pos3"/>
<log idref="clockRate.c:COX2_pos3"/>
<log idref="clockRate.c:COX3_pos2"/>
<log idref="clockRate.c:CYTB_pos2"/>
<log idref="clockRate.c:atp6_pos2"/>
<log idref="clockRate.c:COX3_pos3"/>
<log idref="clockRate.c:CYTB_pos3"/>
<log idref="clockRate.c:NADH4L_pos2"/>
<log idref="clockRate.c:NADH6_pos2"/>
<log idref="clockRate.c:NADH1_pos2"/>
<log idref="clockRate.c:NADH4_pos1"/>
<log idref="clockRate.c:atp6_pos3"/>
<log idref="clockRate.c:COX2_pos2"/>

<log idref="clockRate.c:NADH2_pos1"/>
<log idref="clockRate.c:NADH5_pos3"/>
<log idref="clockRate.c:NADH5_pos1"/>
<log idref="clockRate.c:NADH3_pos3"/>
<log idref="clockRate.c:NADH2_pos3"/>
<log idref="clockRate.c:NADH3_pos2"/>
<log idref="clockRate.c:NADH3_pos1"/>
<log idref="clockRate.c:CYTB_pos1"/>
<log idref="clockRate.c:NADH1_pos3"/>
<log idref="proportionInvariant.s:atp6_pos1"/>
<log idref="gammaShape.s:atp6_pos1"/>
<log idref="rateAC.s:atp6_pos1"/>
<log idref="rateAG.s:atp6_pos1"/>
<log idref="rateAT.s:atp6_pos1"/>
<log idref="rateCG.s:atp6_pos1"/>
<log idref="rateGT.s:atp6_pos1"/>
<log idref="rateAC.s:atp6_pos2"/>
<log idref="rateAG.s:atp6_pos2"/>
<log idref="rateAT.s:atp6_pos2"/>
<log idref="rateCG.s:atp6_pos2"/>
<log idref="rateGT.s:atp6_pos2"/>
<log idref="proportionInvariant.s:atp6_pos2"/>
<log idref="gammaShape.s:atp6_pos2"/>
<log idref="gammaShape.s:atp6_pos3"/>
<log idref="rateAC.s:atp6_pos3"/>
<log idref="rateAG.s:atp6_pos3"/>
<log idref="rateAT.s:atp6_pos3"/>
<log idref="rateCG.s:atp6_pos3"/>
<log idref="rateGT.s:atp6_pos3"/>
<log idref="gammaShape.s:Mytilidae27sq12PCGs"/>
<log idref="gammaShape.s:COX1_pos1"/>
<log idref="rateAC.s:COX1_pos1"/>
<log idref="rateAG.s:COX1_pos1"/>
<log idref="rateAT.s:COX1_pos1"/>
<log idref="rateCG.s:COX1_pos1"/>
<log idref="rateGT.s:COX1_pos1"/>
<log idref="gammaShape.s:COX1_pos2"/>
<log idref="gammaShape.s:COX1_pos3"/>
<log idref="rateAC.s:COX1_pos3"/>
<log idref="rateAG.s:COX1_pos3"/>
<log idref="rateAT.s:COX1_pos3"/>
<log idref="rateCG.s:COX1_pos3"/>
<log idref="rateGT.s:COX1_pos3"/>
<log idref="gammaShape.s:COX2_pos1"/>
<log idref="rateAC.s:COX2_pos1"/>
<log idref="rateAG.s:COX2_pos1"/>
<log idref="rateAT.s:COX2_pos1"/>
<log idref="rateCG.s:COX2_pos1"/>
<log idref="rateGT.s:COX2_pos1"/>
<log idref="gammaShape.s:COX2_pos2"/>
<log idref="rateAC.s:COX2_pos3"/>
<log idref="rateAG.s:COX2_pos3"/>
<log idref="rateAT.s:COX2_pos3"/>
<log idref="rateCG.s:COX2_pos3"/>
<log idref="rateGT.s:COX2_pos3"/>
<log idref="gammaShape.s:COX2_pos3"/>
<log idref="gammaShape.s:COX3_pos1"/>
<log idref="rateAC.s:COX3_pos1"/>
<log idref="rateAG.s:COX3_pos1"/>
<log idref="rateAT.s:COX3_pos1"/>
<log idref="rateCG.s:COX3_pos1"/>
<log idref="rateGT.s:COX3_pos1"/>
<log idref="gammaShape.s:COX3_pos2"/>

<log idref="rateAC.s:COX3_pos2"/>
<log idref="rateAG.s:COX3_pos2"/>
<log idref="rateAT.s:COX3_pos2"/>
<log idref="rateCG.s:COX3_pos2"/>
<log idref="rateGT.s:COX3_pos2"/>
<log idref="gammaShape.s:COX3_pos3"/>
<log idref="rateAC.s:COX3_pos3"/>
<log idref="rateAG.s:COX3_pos3"/>
<log idref="rateAT.s:COX3_pos3"/>
<log idref="rateCG.s:COX3_pos3"/>
<log idref="rateGT.s:COX3_pos3"/>
<log idref="gammaShape.s:CYTB_pos1"/>
<log idref="rateAC.s:CYTB_pos1"/>
<log idref="rateAG.s:CYTB_pos1"/>
<log idref="rateAT.s:CYTB_pos1"/>
<log idref="rateCG.s:CYTB_pos1"/>
<log idref="rateGT.s:CYTB_pos1"/>
<log idref="gammaShape.s:CYTB_pos2"/>
<log idref="rateAC.s:CYTB_pos2"/>
<log idref="rateAG.s:CYTB_pos2"/>
<log idref="rateAT.s:CYTB_pos2"/>
<log idref="rateCG.s:CYTB_pos2"/>
<log idref="rateGT.s:CYTB_pos2"/>
<log idref="gammaShape.s:CYTB_pos3"/>
<log idref="rateAC.s:CYTB_pos3"/>
<log idref="rateAG.s:CYTB_pos3"/>
<log idref="rateAT.s:CYTB_pos3"/>
<log idref="rateCG.s:CYTB_pos3"/>
<log idref="rateGT.s:CYTB_pos3"/>
<log idref="gammaShape.s:NADH1_pos1"/>
<log idref="rateAC.s:NADH1_pos1"/>
<log idref="rateAG.s:NADH1_pos1"/>
<log idref="rateAT.s:NADH1_pos1"/>
<log idref="rateCG.s:NADH1_pos1"/>
<log idref="rateGT.s:NADH1_pos1"/>
<log idref="gammaShape.s:NADH1_pos2"/>
<log idref="rateAC.s:NADH1_pos2"/>
<log idref="rateAG.s:NADH1_pos2"/>
<log idref="rateAT.s:NADH1_pos2"/>
<log idref="rateCG.s:NADH1_pos2"/>
<log idref="rateGT.s:NADH1_pos2"/>
<log idref="gammaShape.s:NADH1_pos3"/>
<log idref="rateAC.s:NADH1_pos3"/>
<log idref="rateAG.s:NADH1_pos3"/>
<log idref="rateAT.s:NADH1_pos3"/>
<log idref="rateCG.s:NADH1_pos3"/>
<log idref="rateGT.s:NADH1_pos3"/>
<log idref="gammaShape.s:NADH2_pos1"/>
<log idref="rateAC.s:NADH2_pos1"/>
<log idref="rateAG.s:NADH2_pos1"/>
<log idref="rateAT.s:NADH2_pos1"/>
<log idref="rateCG.s:NADH2_pos1"/>
<log idref="rateGT.s:NADH2_pos1"/>
<log idref="gammaShape.s:NADH2_pos2"/>
<log idref="rateAC.s:NADH2_pos2"/>
<log idref="rateAG.s:NADH2_pos2"/>
<log idref="rateAT.s:NADH2_pos2"/>
<log idref="rateCG.s:NADH2_pos2"/>
<log idref="rateGT.s:NADH2_pos2"/>
<log idref="gammaShape.s:NADH2_pos3"/>
<log idref="rateAC.s:NADH2_pos3"/>
<log idref="rateAG.s:NADH2_pos3"/>
<log idref="rateAT.s:NADH2_pos3"/>

<log idref="rateCG.s:NADH2_pos3"/>
<log idref="rateGT.s:NADH2_pos3"/>
<log idref="rateAC.s:NADH3_pos1"/>
<log idref="rateAG.s:NADH3_pos1"/>
<log idref="rateAT.s:NADH3_pos1"/>
<log idref="rateCG.s:NADH3_pos1"/>
<log idref="rateGT.s:NADH3_pos1"/>
<log idref="gammaShape.s:NADH3_pos1"/>
<log idref="gammaShape.s:NADH3_pos2"/>
<log idref="rateAC.s:NADH3_pos2"/>
<log idref="rateAG.s:NADH3_pos2"/>
<log idref="rateAT.s:NADH3_pos2"/>
<log idref="rateCG.s:NADH3_pos2"/>
<log idref="rateGT.s:NADH3_pos2"/>
<log idref="gammaShape.s:NADH3_pos3"/>
<log idref="rateAC.s:NADH3_pos3"/>
<log idref="rateAG.s:NADH3_pos3"/>
<log idref="rateAT.s:NADH3_pos3"/>
<log idref="rateCG.s:NADH3_pos3"/>
<log idref="rateGT.s:NADH3_pos3"/>
<log idref="gammaShape.s:NADH4_pos1"/>
<log idref="rateAC.s:NADH4_pos1"/>
<log idref="rateAG.s:NADH4_pos1"/>
<log idref="rateAT.s:NADH4_pos1"/>
<log idref="rateCG.s:NADH4_pos1"/>
<log idref="rateGT.s:NADH4_pos1"/>
<log idref="gammaShape.s:NADH4_pos2"/>
<log idref="rateAC.s:NADH4_pos2"/>
<log idref="rateAG.s:NADH4_pos2"/>
<log idref="rateAT.s:NADH4_pos2"/>
<log idref="rateCG.s:NADH4_pos2"/>
<log idref="rateGT.s:NADH4_pos2"/>
<log idref="gammaShape.s:NADH4_pos3"/>
<log idref="rateAC.s:NADH4_pos3"/>
<log idref="rateAG.s:NADH4_pos3"/>
<log idref="rateAT.s:NADH4_pos3"/>
<log idref="rateCG.s:NADH4_pos3"/>
<log idref="rateGT.s:NADH4_pos3"/>
<log idref="gammaShape.s:NADH4L_pos1"/>
<log idref="rateAC.s:NADH4L_pos1"/>
<log idref="rateAG.s:NADH4L_pos1"/>
<log idref="rateAT.s:NADH4L_pos1"/>
<log idref="rateCG.s:NADH4L_pos1"/>
<log idref="rateGT.s:NADH4L_pos1"/>
<log idref="gammaShape.s:NADH4L_pos2"/>
<log idref="rateAC.s:NADH4L_pos2"/>
<log idref="rateAG.s:NADH4L_pos2"/>
<log idref="rateAT.s:NADH4L_pos2"/>
<log idref="rateCG.s:NADH4L_pos2"/>
<log idref="rateGT.s:NADH4L_pos2"/>
<log idref="gammaShape.s:NADH4L_pos3"/>
<log idref="rateAC.s:NADH4L_pos3"/>
<log idref="rateAG.s:NADH4L_pos3"/>
<log idref="rateAT.s:NADH4L_pos3"/>
<log idref="rateCG.s:NADH4L_pos3"/>
<log idref="rateGT.s:NADH4L_pos3"/>
<log idref="gammaShape.s:NADH5_pos1"/>
<log idref="rateAC.s:NADH5_pos1"/>
<log idref="rateAG.s:NADH5_pos1"/>
<log idref="rateAT.s:NADH5_pos1"/>
<log idref="rateCG.s:NADH5_pos1"/>
<log idref="rateGT.s:NADH5_pos1"/>
<log idref="gammaShape.s:NADH5_pos2"/>

<log idref="rateAC.s:NADH5_pos2"/>
<log idref="rateAG.s:NADH5_pos2"/>
<log idref="rateAT.s:NADH5_pos2"/>
<log idref="rateCG.s:NADH5_pos2"/>
<log idref="rateGT.s:NADH5_pos2"/>
<log idref="gammaShape.s:NADH5_pos3"/>
<log idref="rateAC.s:NADH5_pos3"/>
<log idref="rateAG.s:NADH5_pos3"/>
<log idref="rateAT.s:NADH5_pos3"/>
<log idref="rateCG.s:NADH5_pos3"/>
<log idref="rateGT.s:NADH5_pos3"/>
<log idref="gammaShape.s:NADH6_pos1"/>
<log idref="rateAC.s:NADH6_pos1"/>
<log idref="rateAG.s:NADH6_pos1"/>
<log idref="rateAT.s:NADH6_pos1"/>
<log idref="rateCG.s:NADH6_pos1"/>
<log idref="rateGT.s:NADH6_pos1"/>
<log idref="gammaShape.s:NADH6_pos2"/>
<log idref="rateAC.s:NADH6_pos2"/>
<log idref="rateAG.s:NADH6_pos2"/>
<log idref="rateAT.s:NADH6_pos2"/>
<log idref="rateCG.s:NADH6_pos2"/>
<log idref="rateGT.s:NADH6_pos2"/>
<log idref="gammaShape.s:NADH6_pos3"/>
<log idref="rateAC.s:NADH6_pos3"/>
<log idref="rateAG.s:NADH6_pos3"/>
<log idref="rateAT.s:NADH6_pos3"/>
<log idref="rateCG.s:NADH6_pos3"/>
<log idref="rateGT.s:NADH6_pos3"/>
<log idref="popSize.t:COX1_pos1"/>
<log idref="CoalescentConstant.t:COX1_pos1"/>
<log idref="popSize.t:COX1_pos2"/>
<log idref="CoalescentConstant.t:COX1_pos2"/>
<log idref="popSize.t:COX1_pos3"/>
<log idref="CoalescentConstant.t:COX1_pos3"/>
<log idref="popSize.t:COX2_pos1"/>
<log idref="CoalescentConstant.t:COX2_pos1"/>
<log idref="popSize.t:COX2_pos2"/>
<log idref="CoalescentConstant.t:COX2_pos2"/>
<log idref="popSize.t:COX2_pos3"/>
<log idref="CoalescentConstant.t:COX2_pos3"/>
<log idref="popSize.t:COX3_pos1"/>
<log idref="CoalescentConstant.t:COX3_pos1"/>
<log idref="popSize.t:COX3_pos2"/>
<log idref="CoalescentConstant.t:COX3_pos2"/>
<log idref="popSize.t:COX3_pos3"/>
<log idref="CoalescentConstant.t:COX3_pos3"/>
<log idref="popSize.t:CYTB_pos1"/>
<log idref="CoalescentConstant.t:CYTB_pos1"/>
<log idref="popSize.t:CYTB_pos2"/>
<log idref="CoalescentConstant.t:CYTB_pos2"/>
<log idref="popSize.t:CYTB_pos3"/>
<log idref="CoalescentConstant.t:CYTB_pos3"/>
<log idref="popSize.t:Mytilidae27sq12PCGs"/>
<log idref="CoalescentConstant.t:Mytilidae27sq12PCGs"/>
<log idref="popSize.t:NADH1_pos1"/>
<log idref="CoalescentConstant.t:NADH1_pos1"/>
<log idref="popSize.t:NADH1_pos2"/>
<log idref="CoalescentConstant.t:NADH1_pos2"/>
<log idref="popSize.t:NADH1_pos3"/>
<log idref="CoalescentConstant.t:NADH1_pos3"/>
<log idref="popSize.t:NADH2_pos1"/>
<log idref="CoalescentConstant.t:NADH2_pos1"/>

<log idref="popSize.t:NADH2_pos2"/>
<log idref="CoalescentConstant.t:NADH2_pos2"/>
<log idref="popSize.t:NADH2_pos3"/>
<log idref="CoalescentConstant.t:NADH2_pos3"/>
<log idref="popSize.t:NADH3_pos1"/>
<log idref="CoalescentConstant.t:NADH3_pos1"/>
<log idref="popSize.t:NADH3_pos2"/>
<log idref="CoalescentConstant.t:NADH3_pos2"/>
<log idref="popSize.t:NADH3_pos3"/>
<log idref="CoalescentConstant.t:NADH3_pos3"/>
<log idref="popSize.t:NADH4L_pos1"/>
<log idref="CoalescentConstant.t:NADH4L_pos1"/>
<log idref="popSize.t:NADH4L_pos2"/>
<log idref="CoalescentConstant.t:NADH4L_pos2"/>
<log idref="popSize.t:NADH4L_pos3"/>
<log idref="CoalescentConstant.t:NADH4L_pos3"/>
<log idref="popSize.t:NADH4_pos1"/>
<log idref="CoalescentConstant.t:NADH4_pos1"/>
<log idref="popSize.t:NADH4_pos2"/>
<log idref="CoalescentConstant.t:NADH4_pos2"/>
<log idref="popSize.t:NADH4_pos3"/>
<log idref="CoalescentConstant.t:NADH4_pos3"/>
<log idref="popSize.t:NADH5_pos1"/>
<log idref="CoalescentConstant.t:NADH5_pos1"/>
<log idref="popSize.t:NADH5_pos2"/>
<log idref="CoalescentConstant.t:NADH5_pos2"/>
<log idref="popSize.t:NADH5_pos3"/>
<log idref="CoalescentConstant.t:NADH5_pos3"/>
<log idref="popSize.t:NADH6_pos1"/>
<log idref="CoalescentConstant.t:NADH6_pos1"/>
<log idref="popSize.t:NADH6_pos2"/>
<log idref="CoalescentConstant.t:NADH6_pos2"/>
<log idref="popSize.t:NADH6_pos3"/>
<log idref="CoalescentConstant.t:NADH6_pos3"/>
<log idref="popSize.t:atp6_pos1"/>
<log idref="CoalescentConstant.t:atp6_pos1"/>
<log idref="popSize.t:atp6_pos2"/>
<log idref="CoalescentConstant.t:atp6_pos2"/>
<log idref="popSize.t:atp6_pos3"/>
<log idref="CoalescentConstant.t:atp6_pos3"/>
<log idref="proportionInvariant.s:Mytilidae27sq12PCGs"/>
<log idref="proportionInvariant.s:atp6_pos3"/>
<log idref="proportionInvariant.s:COX1_pos1"/>
<log idref="proportionInvariant.s:COX1_pos2"/>
<log idref="proportionInvariant.s:COX1_pos3"/>
<log idref="proportionInvariant.s:COX2_pos1"/>
<log idref="proportionInvariant.s:COX2_pos2"/>
<log idref="rateAC.s:COX2_pos2"/>
<log idref="rateAG.s:COX2_pos2"/>
<log idref="rateAT.s:COX2_pos2"/>
<log idref="rateCG.s:COX2_pos2"/>
<log idref="rateGT.s:COX2_pos2"/>
<log idref="proportionInvariant.s:COX2_pos3"/>
<log idref="proportionInvariant.s:COX3_pos1"/>
<log idref="proportionInvariant.s:COX3_pos2"/>
<log idref="proportionInvariant.s:COX3_pos3"/>
<log idref="proportionInvariant.s:CYTB_pos1"/>
<log idref="proportionInvariant.s:CYTB_pos2"/>
<log idref="proportionInvariant.s:CYTB_pos3"/>
<log idref="proportionInvariant.s:NADH1_pos1"/>
<log idref="proportionInvariant.s:NADH1_pos2"/>
<log idref="proportionInvariant.s:NADH1_pos3"/>
<log idref="proportionInvariant.s:NADH2_pos1"/>

```
    <log idref="proportionInvariant.s:NADH2_pos2"/>
    <log idref="proportionInvariant.s:NADH2_pos3"/>
    <log idref="proportionInvariant.s:NADH3_pos1"/>
    <log idref="proportionInvariant.s:NADH3_pos2"/>
    <log idref="proportionInvariant.s:NADH3_pos3"/>
    <log idref="proportionInvariant.s:NADH4_pos1"/>
    <log idref="proportionInvariant.s:NADH4_pos2"/>
    <log idref="proportionInvariant.s:NADH4_pos3"/>
    <log idref="proportionInvariant.s:NADH4L_pos1"/>
    <log idref="proportionInvariant.s:NADH4L_pos2"/>
    <log idref="proportionInvariant.s:NADH4L_pos3"/>
    <log idref="proportionInvariant.s:NADH5_pos1"/>
    <log idref="proportionInvariant.s:NADH5_pos2"/>
    <log idref="proportionInvariant.s:NADH5_pos3"/>
    <log idref="proportionInvariant.s:NADH6_pos1"/>
    <log idref="proportionInvariant.s:NADH6_pos2"/>
    <log idref="proportionInvariant.s:NADH6_pos3"/>
    <log idref="rateAC.s:Mytilidae27sq12PCGs"/>
    <log idref="rateAG.s:Mytilidae27sq12PCGs"/>
    <log idref="rateAT.s:Mytilidae27sq12PCGs"/>
    <log idref="rateCG.s:Mytilidae27sq12PCGs"/>
    <log idref="rateGT.s:Mytilidae27sq12PCGs"/>
    <log idref="freqParameter.s:atp6_pos1"/>
    <log idref="freqParameter.s:COX1_pos1"/>
    <log idref="freqParameter.s:COX1_pos3"/>
    <log idref="freqParameter.s:NADH5_pos3"/>
    <log idref="freqParameter.s:NADH2_pos3"/>
    <log idref="freqParameter.s:NADH3_pos3"/>
    <log idref="freqParameter.s:NADH1_pos1"/>
    <log idref="freqParameter.s:NADH6_pos1"/>
    <log idref="freqParameter.s:NADH1_pos2"/>
    <log idref="freqParameter.s:NADH4L_pos1"/>
    <log idref="freqParameter.s:CYTB_pos1"/>
    <log idref="freqParameter.s:atp6_pos2"/>
    <log idref="freqParameter.s:NADH6_pos3"/>
    <log idref="freqParameter.s:NADH2_pos2"/>
    <log idref="freqParameter.s:NADH6_pos2"/>
    <log idref="freqParameter.s:NADH4L_pos2"/>
    <log idref="freqParameter.s:NADH4_pos3"/>
    <log idref="freqParameter.s:NADH5_pos1"/>
    <log idref="freqParameter.s:NADH5_pos2"/>
    <log idref="freqParameter.s:NADH3_pos2"/>
    <log idref="freqParameter.s:Mytilidae27sq12PCGs"/>
    <log idref="freqParameter.s:COX3_pos1"/>
    <log idref="freqParameter.s:NADH2_pos1"/>
    <log idref="freqParameter.s:NADH3_pos1"/>
    <log idref="freqParameter.s:NADH1_pos3"/>
    <log idref="freqParameter.s:CYTB_pos2"/>
    <log idref="freqParameter.s:COX2_pos2"/>
    <log idref="freqParameter.s:COX2_pos3"/>
    <log idref="freqParameter.s:COX3_pos2"/>
    <log idref="freqParameter.s:CYTB_pos3"/>
    <log idref="freqParameter.s:NADH4_pos2"/>
    <log idref="freqParameter.s:COX3_pos3"/>
    <log idref="freqParameter.s:NADH4L_pos3"/>
    <log idref="freqParameter.s:COX2_pos1"/>
    <log idref="freqParameter.s:NADH4_pos1"/>
    <log idref="freqParameter.s:atp6_pos3"/>
</logger>

<logger id="screenlog" spec="Logger" logEvery="1000">
    <log idref="posterior"/>
    <log idref="likelihood"/>
```

```
        <log idref="prior"/>
    </logger>

    <logger id="treelog.t:NADH6_pos1" spec="Logger" fileName="$(tree).trees" logEvery="1000" mode="tree">
        <log id="TreeWithMetaDataLogger.t:NADH6_pos1" spec="beast.evolution.tree.TreeWithMetaDataLogger"
tree="@Tree.t:NADH6_pos1"/>
    </logger>

    <logger id="treelog.t:NADH1_pos1" spec="Logger" fileName="$(tree).trees" logEvery="1000" mode="tree">
        <log id="TreeWithMetaDataLogger.t:NADH1_pos1" spec="beast.evolution.tree.TreeWithMetaDataLogger"
tree="@Tree.t:NADH1_pos1"/>
    </logger>

    <logger id="treelog.t:COX2_pos1" spec="Logger" fileName="$(tree).trees" logEvery="1000" mode="tree">
        <log id="TreeWithMetaDataLogger.t:COX2_pos1" spec="beast.evolution.tree.TreeWithMetaDataLogger"
tree="@Tree.t:COX2_pos1"/>
    </logger>

    <logger id="treelog.t:NADH1_pos2" spec="Logger" fileName="$(tree).trees" logEvery="1000" mode="tree">
        <log id="TreeWithMetaDataLogger.t:NADH1_pos2" spec="beast.evolution.tree.TreeWithMetaDataLogger"
tree="@Tree.t:NADH1_pos2"/>
    </logger>

    <logger id="treelog.t:NADH4_pos1" spec="Logger" fileName="$(tree).trees" logEvery="1000" mode="tree">
        <log id="TreeWithMetaDataLogger.t:NADH4_pos1" spec="beast.evolution.tree.TreeWithMetaDataLogger"
tree="@Tree.t:NADH4_pos1"/>
    </logger>

    <logger id="treelog.t:NADH4L_pos3" spec="Logger" fileName="$(tree).trees" logEvery="1000" mode="tree">
        <log id="TreeWithMetaDataLogger.t:NADH4L_pos3" spec="beast.evolution.tree.TreeWithMetaDataLogger"
tree="@Tree.t:NADH4L_pos3"/>
    </logger>

    <logger id="treelog.t:NADH4L_pos1" spec="Logger" fileName="$(tree).trees" logEvery="1000" mode="tree">
        <log id="TreeWithMetaDataLogger.t:NADH4L_pos1" spec="beast.evolution.tree.TreeWithMetaDataLogger"
tree="@Tree.t:NADH4L_pos1"/>
    </logger>

    <logger id="treelog.t:NADH2_pos1" spec="Logger" fileName="$(tree).trees" logEvery="1000" mode="tree">
        <log id="TreeWithMetaDataLogger.t:NADH2_pos1" spec="beast.evolution.tree.TreeWithMetaDataLogger"
tree="@Tree.t:NADH2_pos1"/>
    </logger>

    <logger id="treelog.t:NADH3_pos2" spec="Logger" fileName="$(tree).trees" logEvery="1000" mode="tree">
        <log id="TreeWithMetaDataLogger.t:NADH3_pos2" spec="beast.evolution.tree.TreeWithMetaDataLogger"
tree="@Tree.t:NADH3_pos2"/>
    </logger>

    <logger id="treelog.t:NADH2_pos3" spec="Logger" fileName="$(tree).trees" logEvery="1000" mode="tree">
        <log id="TreeWithMetaDataLogger.t:NADH2_pos3" spec="beast.evolution.tree.TreeWithMetaDataLogger"
tree="@Tree.t:NADH2_pos3"/>
    </logger>

    <logger id="treelog.t:NADH3_pos1" spec="Logger" fileName="$(tree).trees" logEvery="1000" mode="tree">
        <log id="TreeWithMetaDataLogger.t:NADH3_pos1" spec="beast.evolution.tree.TreeWithMetaDataLogger"
tree="@Tree.t:NADH3_pos1"/>
    </logger>

    <logger id="treelog.t:NADH2_pos2" spec="Logger" fileName="$(tree).trees" logEvery="1000" mode="tree">
        <log id="TreeWithMetaDataLogger.t:NADH2_pos2" spec="beast.evolution.tree.TreeWithMetaDataLogger"
tree="@Tree.t:NADH2_pos2"/>
    </logger>
```

```
<logger id="treelog.t:CYTB_pos2" spec="Logger" fileName="$(tree).trees" logEvery="1000" mode="tree">
    <log id="TreeWithMetaDataLogger.t:CYTB_pos2" spec="beast.evolution.tree.TreeWithMetaDataLogger"
tree="@Tree.t:CYTB_pos2"/>
</logger>

<logger id="treelog.t:atp6_pos3" spec="Logger" fileName="$(tree).trees" logEvery="1000" mode="tree">
    <log id="TreeWithMetaDataLogger.t:atp6_pos3" spec="beast.evolution.tree.TreeWithMetaDataLogger"
tree="@Tree.t:atp6_pos3"/>
</logger>

<logger id="treelog.t:COX1_pos1" spec="Logger" fileName="$(tree).trees" logEvery="1000" mode="tree">
    <log id="TreeWithMetaDataLogger.t:COX1_pos1" spec="beast.evolution.tree.TreeWithMetaDataLogger"
tree="@Tree.t:COX1_pos1"/>
</logger>

<logger id="treelog.t:CYTB_pos1" spec="Logger" fileName="$(tree).trees" logEvery="1000" mode="tree">
    <log id="TreeWithMetaDataLogger.t:CYTB_pos1" spec="beast.evolution.tree.TreeWithMetaDataLogger"
tree="@Tree.t:CYTB_pos1"/>
</logger>

<logger id="treelog.t:NADH4_pos3" spec="Logger" fileName="$(tree).trees" logEvery="1000" mode="tree">
    <log id="TreeWithMetaDataLogger.t:NADH4_pos3" spec="beast.evolution.tree.TreeWithMetaDataLogger"
tree="@Tree.t:NADH4_pos3"/>
</logger>

<logger id="treelog.t:COX1_pos3" spec="Logger" fileName="$(tree).trees" logEvery="1000" mode="tree">
    <log id="TreeWithMetaDataLogger.t:COX1_pos3" spec="beast.evolution.tree.TreeWithMetaDataLogger"
tree="@Tree.t:COX1_pos3"/>
</logger>

<logger id="treelog.t:COX2_pos2" spec="Logger" fileName="$(tree).trees" logEvery="1000" mode="tree">
    <log id="TreeWithMetaDataLogger.t:COX2_pos2" spec="beast.evolution.tree.TreeWithMetaDataLogger"
tree="@Tree.t:COX2_pos2"/>
</logger>

<logger id="treelog.t:CYTB_pos3" spec="Logger" fileName="$(tree).trees" logEvery="1000" mode="tree">
    <log id="TreeWithMetaDataLogger.t:CYTB_pos3" spec="beast.evolution.tree.TreeWithMetaDataLogger"
tree="@Tree.t:CYTB_pos3"/>
</logger>

<logger id="treelog.t:NADH5_pos1" spec="Logger" fileName="$(tree).trees" logEvery="1000" mode="tree">
    <log id="TreeWithMetaDataLogger.t:NADH5_pos1" spec="beast.evolution.tree.TreeWithMetaDataLogger"
tree="@Tree.t:NADH5_pos1"/>
</logger>

<logger id="treelog.t:atp6_pos1" spec="Logger" fileName="$(tree).trees" logEvery="1000" mode="tree">
    <log id="TreeWithMetaDataLogger.t:atp6_pos1" spec="beast.evolution.tree.TreeWithMetaDataLogger"
tree="@Tree.t:atp6_pos1"/>
</logger>

<logger id="treelog.t:NADH1_pos3" spec="Logger" fileName="$(tree).trees" logEvery="1000" mode="tree">
    <log id="TreeWithMetaDataLogger.t:NADH1_pos3" spec="beast.evolution.tree.TreeWithMetaDataLogger"
tree="@Tree.t:NADH1_pos3"/>
</logger>

<logger id="treelog.t:atp6_pos2" spec="Logger" fileName="$(tree).trees" logEvery="1000" mode="tree">
    <log id="TreeWithMetaDataLogger.t:atp6_pos2" spec="beast.evolution.tree.TreeWithMetaDataLogger"
tree="@Tree.t:atp6_pos2"/>
</logger>

<logger id="treelog.t:NADH4_pos2" spec="Logger" fileName="$(tree).trees" logEvery="1000" mode="tree">
    <log id="TreeWithMetaDataLogger.t:NADH4_pos2" spec="beast.evolution.tree.TreeWithMetaDataLogger"
tree="@Tree.t:NADH4_pos2"/>
```

</logger>

<logger id="treelog.t:COX3_pos2" spec="Logger" fileName="$(tree).trees" logEvery="1000" mode="tree">
<log id="TreeWithMetaDataLogger.t:COX3_pos2" spec="beast.evolution.tree.TreeWithMetaDataLogger" tree="@Tree.t:COX3_pos2"/>
</logger>

<logger id="treelog.t:NADH5_pos3" spec="Logger" fileName="$(tree).trees" logEvery="1000" mode="tree">
<log id="TreeWithMetaDataLogger.t:NADH5_pos3" spec="beast.evolution.tree.TreeWithMetaDataLogger" tree="@Tree.t:NADH5_pos3"/>
</logger>

<logger id="treelog.t:NADH6_pos3" spec="Logger" fileName="$(tree).trees" logEvery="1000" mode="tree">
<log id="TreeWithMetaDataLogger.t:NADH6_pos3" spec="beast.evolution.tree.TreeWithMetaDataLogger" tree="@Tree.t:NADH6_pos3"/>
</logger>

<logger id="treelog.t:COX3_pos1" spec="Logger" fileName="$(tree).trees" logEvery="1000" mode="tree">
<log id="TreeWithMetaDataLogger.t:COX3_pos1" spec="beast.evolution.tree.TreeWithMetaDataLogger" tree="@Tree.t:COX3_pos1"/>
</logger>

<logger id="treelog.t:NADH3_pos3" spec="Logger" fileName="$(tree).trees" logEvery="1000" mode="tree">
<log id="TreeWithMetaDataLogger.t:NADH3_pos3" spec="beast.evolution.tree.TreeWithMetaDataLogger" tree="@Tree.t:NADH3_pos3"/>
</logger>

<logger id="treelog.t:NADH5_pos2" spec="Logger" fileName="$(tree).trees" logEvery="1000" mode="tree">
<log id="TreeWithMetaDataLogger.t:NADH5_pos2" spec="beast.evolution.tree.TreeWithMetaDataLogger" tree="@Tree.t:NADH5_pos2"/>
</logger>

<logger id="treelog.t:NADH6_pos2" spec="Logger" fileName="$(tree).trees" logEvery="1000" mode="tree">
<log id="TreeWithMetaDataLogger.t:NADH6_pos2" spec="beast.evolution.tree.TreeWithMetaDataLogger" tree="@Tree.t:NADH6_pos2"/>
</logger>

<logger id="treelog.t:NADH4L_pos2" spec="Logger" fileName="$(tree).trees" logEvery="1000" mode="tree">
<log id="TreeWithMetaDataLogger.t:NADH4L_pos2" spec="beast.evolution.tree.TreeWithMetaDataLogger" tree="@Tree.t:NADH4L_pos2"/>
</logger>

<logger id="treelog.t:COX1_pos2" spec="Logger" fileName="$(tree).trees" logEvery="1000" mode="tree">
<log id="TreeWithMetaDataLogger.t:COX1_pos2" spec="beast.evolution.tree.TreeWithMetaDataLogger" tree="@Tree.t:COX1_pos2"/>
</logger>

<logger id="treelog.t:COX2_pos3" spec="Logger" fileName="$(tree).trees" logEvery="1000" mode="tree">
<log id="TreeWithMetaDataLogger.t:COX2_pos3" spec="beast.evolution.tree.TreeWithMetaDataLogger" tree="@Tree.t:COX2_pos3"/>
</logger>

<logger id="treelog.t:COX3_pos3" spec="Logger" fileName="$(tree).trees" logEvery="1000" mode="tree">
<log id="TreeWithMetaDataLogger.t:COX3_pos3" spec="beast.evolution.tree.TreeWithMetaDataLogger" tree="@Tree.t:COX3_pos3"/>
</logger>

<logger id="treelog.t:Mytilidae27sq12PCGs" spec="Logger" fileName="$(tree).trees" logEvery="1000" mode="tree">
<log id="TreeWithMetaDataLogger.t:Mytilidae27sq12PCGs" spec="beast.evolution.tree.TreeWithMetaDataLogger" tree="@Tree.t:Mytilidae27sq12PCGs"/>
</logger>

<operatorschedule id="OperatorSchedule" spec="OperatorSchedule"/>

</run>

</beast>
