# Supplementary material for: Structure, Evolution, and Mitochondrial Genome Analysis of Mussel Species (Bivalvia, Mytilidae)
Source: Int J Mol Sci. 2024 Jun 24;25(13):6902. doi: 10.3390/ijms25136902 (PMC11241113; doi:10.3390/ijms25136902)
Supplement: Supplementary file 1 [file ijms-25-06902-s001.zip › Table S5.DNA Polymorphism estimated via DNAsp-6 among 26 PCG sequences of mussels.pdf]

Table S5. DNA Polymorphism estimated via DNAsp-6 among 26 PCG sequences of mussels (Mytilidae)

DnaSP Ver. 6.12.03 07 - Mart - 2024 12:40:38

===

DNA Polymorphism

Input Data File: D:\...\myt26-pt+pos-Y.nex

Number of sequences: 26 Number of sequences used: 26

Selected region: 1-12354 Number of sites: 12354

Total number of sites (excluding sites with gaps / missing data): 10052

Number of polymorphic (segregating) sites, S: 7552

Total number of mutations, Eta: 15557

Number of Haplotypes, h: 25

Haplotype (gene) diversity, Hd: 0,997

Variance of Haplotype diversity: 0,00014

Standard Deviation of Haplotype diversity: 0,012

Nucleotide diversity, Pi: 0,37673

Theta (per site) from Eta: 0,40557

Theta (per site) from S, Theta-W: 0,19688

Variance of theta (no recombination): 0,0038545

Standard deviation of theta (no recombination): 0,06208

Variance of theta (free recombination): 0,0000051

Standard deviation of theta (free recombination): 0,00227

Finite Sites Model

Theta (per site) from Pi: 0,75695

Theta (per site) from S: 0,35454

Theta (per site) from Eta: 0,74698

Average number of nucleotide differences, k: 3786,883

Stochastic variance of k (no recombination), Vst(k): 2568873,342

Sampling variance of k (no recombination), Vs(k): 217360,380

Total variance of k (no recombination), V(k): 2786233,722

Stochastic variance of k (free recombination), Vst(k): 1262,294

Sampling variance of k (free recombination), Vs(k): 100,984

Total variance of k (free recombination), V(k): 1363,278

Theta (per sequence) from S, Theta-W: 1979,057

Variance of theta (no recombination): 389468,422

Variance of theta (free recombination): 518,627

Sliding Window Option

Sites with alignment gaps were not counted in the window length (and slide)

Window length: 100 Step size: 25

Window	Midpoint	Pi	Theta	S
1-184	131	0,54354	0,25420	97
104-221	159	0,53578	0,25420	97
132-249	184	0,54240	0,25682	98
160-274	221	0,50335	0,24109	92
185-299	249	0,45422	0,22537	86
222-324	274	0,41889	0,20703	79
250-349	299	0,37569	0,19654	75
275-374	324	0,37738	0,20965	80
300-399	349	0,37428	0,20965	80
325-424	374	0,38397	0,22013	84
350-449	399	0,41342	0,22275	85
375-474	424	0,40748	0,20965	80
400-499	449	0,40695	0,21227	81

425-524	474	0,39129	0,20703	79
450-549	499	0,37880	0,19916	76
475-574	524	0,36945	0,19654	75
500-599	549	0,42234	0,20965	80
525-651	574	0,46434	0,22013	84
550-679	599	0,49545	0,23585	90
575-704	651	0,52446	0,24895	95
600-730	679	0,44594	0,22799	87
652-755	704	0,38729	0,20965	80
680-903	730	0,33769	0,19130	73
705-928	755	0,32529	0,17558	67
731-953	903	0,33883	0,17296	66
756-978	928	0,34234	0,17558	67
904-1009	953	0,34151	0,17820	68
929-1034	978	0,30089	0,16510	63
954-1059	1009	0,26702	0,15461	59
979-1084	1034	0,25406	0,14151	54
1010-1109	1059	0,24594	0,13627	52
1035-1134	1084	0,27015	0,14937	57
1060-1159	1109	0,28412	0,15199	58
1085-1184	1134	0,30249	0,16772	64
1110-1209	1159	0,32114	0,17820	68
1135-1234	1184	0,29505	0,16510	63
1160-1260	1209	0,31769	0,17558	67
1185-1285	1234	0,32662	0,17558	67
1210-1310	1260	0,32360	0,17034	65
1235-1335	1285	0,33658	0,18344	70
1261-1360	1310	0,32465	0,18868	72
1286-1385	1335	0,32003	0,18606	71
1311-1410	1360	0,33382	0,19654	75
1336-1435	1385	0,34649	0,19916	76
1361-1460	1410	0,33342	0,18606	71

1386-1485	1435	0,30135	0,16772	64
1411-1510	1460	0,25295	0,14937	57
1436-1535	1485	0,22935	0,13103	50
1461-1560	1510	0,21268	0,12841	49
1486-1585	1535	0,20828	0,12579	48
1511-1610	1560	0,21258	0,12055	46
1536-1635	1585	0,23074	0,14151	54
1561-1660	1610	0,26363	0,15461	59
1586-1685	1635	0,26757	0,16248	62
1611-1710	1660	0,28197	0,16772	64
1636-1735	1685	0,25545	0,14413	55
1661-1760	1710	0,23406	0,13103	50
1686-1785	1735	0,24415	0,14151	54
1711-1811	1760	0,21969	0,12579	48
1736-1836	1785	0,24317	0,13889	53
1761-1861	1811	0,29951	0,16772	64
1786-1886	1836	0,31120	0,16510	63
1812-1911	1861	0,32495	0,17296	66
1837-1936	1886	0,32702	0,17820	68
1862-1961	1911	0,26923	0,14937	57
1887-1986	1936	0,24077	0,13627	52
1912-2011	1961	0,25711	0,14151	54
1937-2036	1986	0,23720	0,13103	50
1962-2061	2011	0,26714	0,14151	54
1987-2086	2036	0,29797	0,16248	62
2012-2111	2061	0,29022	0,16510	63
2037-2136	2086	0,29028	0,16248	62
2062-2161	2111	0,26197	0,14675	56
2087-2186	2136	0,24742	0,13627	52
2112-2211	2161	0,25234	0,15199	58
2137-2236	2186	0,28775	0,17296	66
2162-2261	2211	0,30618	0,18606	71

2187-2286	2236	0,30840	0,18868	72
2212-2311	2261	0,34452	0,19130	73
2237-2342	2286	0,35443	0,18868	72
2262-2367	2311	0,39351	0,19916	76
2287-2395	2342	0,45092	0,22013	84
2312-2534	2367	0,47295	0,22537	86
2343-2559	2395	0,46132	0,23061	88
2368-2584	2534	0,44412	0,22799	87
2396-2612	2559	0,44252	0,22799	87
2535-2637	2584	0,41972	0,22799	87
2560-2662	2612	0,42858	0,22275	85
2585-2687	2637	0,41702	0,21489	82
2613-2712	2662	0,38732	0,20440	78
2638-2737	2687	0,36665	0,19392	74
2663-2762	2712	0,36449	0,19130	73
2688-2787	2737	0,34123	0,18606	71
2713-2845	2762	0,35791	0,18868	72
2738-2870	2787	0,38588	0,19130	73
2763-2910	2845	0,40945	0,20440	78
2788-2935	2870	0,40822	0,19916	76
2846-2960	2910	0,37680	0,19130	73
2871-2985	2935	0,36711	0,19392	74
2911-3010	2960	0,30754	0,16772	64
2936-3035	2985	0,33000	0,17820	68
2961-3060	3010	0,31938	0,17034	65
2986-3088	3035	0,33191	0,17558	67
3011-3113	3060	0,32228	0,19130	73
3036-3138	3088	0,29458	0,18868	72
3061-3163	3113	0,27452	0,18344	70
3089-3188	3138	0,28175	0,18344	70
3114-3405	3163	0,36086	0,19654	75
3139-3430	3188	0,36637	0,19654	75

3164-3455	3405	0,39658	0,20965	80
3189-3480	3430	0,33877	0,18606	71
3406-3505	3455	0,28935	0,16772	64
3431-3530	3480	0,27797	0,16248	62
3456-3555	3505	0,28760	0,17296	66
3481-3581	3530	0,33012	0,18868	72
3506-3606	3555	0,33554	0,19392	74
3531-3631	3581	0,39360	0,21751	83
3556-3656	3606	0,39662	0,20965	80
3582-3681	3631	0,39295	0,22013	84
3607-3709	3656	0,42446	0,23061	88
3632-3734	3681	0,43686	0,23323	89
3657-3759	3709	0,44526	0,23847	91
3682-3784	3734	0,43852	0,22537	86
3710-3809	3759	0,39957	0,21489	82
3735-3834	3784	0,34994	0,19392	74
3760-3859	3809	0,30726	0,16772	64
3785-3884	3834	0,30003	0,16510	63
3810-3909	3859	0,31649	0,16510	63
3835-3934	3884	0,34702	0,18344	70
3860-3959	3909	0,35406	0,19392	74
3885-3984	3934	0,30705	0,17558	67
3910-4009	3959	0,31720	0,17558	67
3935-4325	3984	0,30788	0,17296	66
3960-4350	4009	0,33018	0,18344	70
3985-4375	4325	0,35332	0,19392	74
4010-4400	4350	0,31622	0,18344	70
4326-4425	4375	0,27606	0,15723	60
4351-4450	4400	0,27145	0,14937	57
4376-4475	4425	0,29283	0,16248	62
4401-4500	4450	0,29351	0,17034	65
4426-4525	4475	0,30954	0,18606	71

4451-4550	4500	0,30040	0,17820	68
4476-4575	4525	0,27898	0,16772	64
4501-4600	4550	0,26068	0,15461	59
4526-4625	4575	0,24065	0,13627	52
4551-4650	4600	0,27228	0,15461	59
4576-4675	4625	0,29769	0,15986	61
4601-4700	4650	0,31671	0,16772	64
4626-4725	4675	0,32289	0,17034	65
4651-4750	4700	0,27945	0,15723	60
4676-4775	4725	0,27369	0,15723	60
4701-4800	4750	0,31914	0,17034	65
4726-4825	4775	0,35874	0,19130	73
4751-4850	4800	0,36526	0,19130	73
4776-4875	4825	0,34963	0,18082	69
4801-4900	4850	0,32871	0,17558	67
4826-4925	4875	0,29471	0,16248	62
4851-4950	4900	0,31209	0,17296	66
4876-4975	4925	0,29963	0,17820	68
4901-5000	4950	0,27692	0,16510	63
4926-5025	4975	0,31000	0,18606	71
4951-5050	5000	0,29698	0,18082	69
4976-5075	5025	0,33449	0,18606	71
5001-5100	5050	0,33840	0,19916	76
5026-5125	5075	0,30086	0,17558	67
5051-5150	5100	0,28022	0,15986	61
5076-5175	5125	0,24612	0,15199	58
5101-5200	5150	0,27351	0,15986	61
5126-5225	5175	0,31474	0,17820	68
5151-5250	5200	0,34329	0,19654	75
5176-5275	5225	0,36883	0,20178	77
5201-5300	5250	0,35231	0,19130	73
5226-5325	5275	0,35043	0,19392	74

5251-5350	5300	0,36434	0,20440	78
5276-5375	5325	0,39566	0,22275	85
5301-5760	5350	0,42065	0,22799	87
5326-5785	5375	0,42889	0,22537	86
5351-5810	5760	0,42963	0,21489	82
5376-5835	5785	0,37951	0,18606	71
5761-5860	5810	0,33603	0,17034	65
5786-5885	5835	0,31126	0,15723	60
5811-5910	5860	0,28757	0,14937	57
5836-5935	5885	0,29271	0,15461	59
5861-5960	5910	0,33145	0,17820	68
5886-5985	5935	0,34480	0,18606	71
5911-6010	5960	0,39277	0,20440	78
5936-6035	5985	0,40548	0,20965	80
5961-6060	6010	0,40092	0,20965	80
5986-6085	6035	0,40434	0,20965	80
6011-6110	6060	0,35535	0,19654	75
6036-6135	6085	0,32246	0,17820	68
6061-6160	6110	0,30409	0,15986	61
6086-6185	6135	0,25240	0,13889	53
6111-6210	6160	0,28735	0,14937	57
6136-6235	6185	0,36425	0,18606	71
6161-6260	6210	0,37640	0,19654	75
6186-6291	6235	0,45280	0,23323	89
6211-6316	6260	0,47338	0,23585	90
6236-6341	6291	0,40720	0,21489	82
6261-6366	6316	0,35120	0,18868	72
6292-6391	6341	0,28062	0,14937	57
6317-6416	6366	0,20449	0,12579	48
6342-6441	6391	0,20422	0,11793	45
6367-6466	6416	0,25825	0,13627	52
6392-6491	6441	0,30548	0,15723	60

6417-6516	6466	0,36948	0,18344	70
6442-6541	6491	0,42683	0,21489	82
6467-6587	6516	0,41188	0,22013	84
6492-6792	6541	0,43702	0,23847	91
6517-6817	6587	0,43837	0,23585	90
6542-6842	6792	0,42991	0,23061	88
6588-6867	6817	0,42271	0,21751	83
6793-6892	6842	0,39560	0,20703	79
6818-6917	6867	0,41465	0,21489	82
6843-6942	6892	0,37880	0,19392	74
6868-6967	6917	0,40612	0,20965	80
6893-7013	6942	0,44378	0,22013	84
6918-7038	6967	0,45609	0,21751	83
6943-7063	7013	0,50148	0,23323	89
6968-7088	7038	0,48178	0,22537	86
7014-7113	7063	0,45548	0,21751	83
7039-7138	7088	0,41594	0,21227	81
7064-7163	7113	0,36357	0,19654	75
7089-7197	7138	0,41108	0,21489	82
7114-7222	7163	0,44218	0,22275	85
7139-7247	7197	0,43985	0,21489	82
7164-7272	7222	0,45514	0,22275	85
7198-7297	7247	0,40262	0,19916	76
7223-7322	7272	0,35197	0,18344	70
7248-7347	7297	0,36468	0,18606	71
7273-7372	7322	0,38302	0,19392	74
7298-7398	7347	0,41929	0,20965	80
7323-7423	7372	0,46551	0,22799	87
7348-7448	7398	0,48994	0,24109	92
7373-7473	7423	0,50517	0,24371	93
7399-7498	7448	0,47874	0,22537	86
7424-7523	7473	0,43652	0,20440	78

7449-7551	7498	0,43911	0,20178	77
7474-7576	7523	0,42791	0,19916	76
7499-7602	7551	0,43883	0,21751	83
7524-7627	7576	0,46511	0,23061	88
7552-7661	7602	0,47634	0,23323	89
7577-7692	7627	0,51677	0,24371	93
7603-7726	7661	0,54585	0,25420	97
7628-7862	7692	0,55502	0,26206	100
7662-7920	7726	0,52557	0,25682	98
7693-7948	7862	0,50169	0,25420	97
7727-7973	7920	0,50409	0,24633	94
7863-7998	7948	0,44508	0,22013	84
7921-8023	7973	0,38268	0,19916	76
7949-8048	7998	0,32917	0,17296	66
7974-8073	8023	0,27212	0,15461	59
7999-8098	8048	0,27711	0,15199	58
8024-8123	8073	0,33646	0,17034	65
8049-8151	8098	0,39375	0,19392	74
8074-8176	8123	0,44086	0,20965	80
8099-8201	8151	0,45280	0,22275	85
8124-8226	8176	0,39514	0,19654	75
8152-8326	8201	0,38908	0,19654	75
8177-8351	8226	0,34994	0,18344	70
8202-8376	8326	0,38926	0,19130	73
8302-8405	8351	0,45252	0,22537	86
8327-8430	8376	0,42326	0,21751	83
8352-8455	8405	0,42425	0,22013	84
8377-8480	8430	0,39188	0,20703	79
8406-8505	8455	0,37231	0,19654	75
8431-8569	8480	0,40111	0,20703	79
8456-8594	8505	0,44120	0,22013	84
8481-8622	8569	0,48240	0,23847	91

8506-8650	8594	0,50486	0,24895	95
8570-8678	8622	0,53978	0,24895	95
8595-8703	8650	0,55302	0,25158	96
8623-8729	8678	0,54692	0,24633	94
8651-8754	8703	0,53037	0,23585	90
8679-8782	8729	0,49905	0,23323	89
8704-8807	8754	0,48895	0,23323	89
8730-8832	8782	0,48594	0,23847	91
8755-8857	8807	0,46917	0,23847	91
8783-8882	8832	0,40972	0,23061	88
8808-8907	8857	0,40095	0,23323	89
8833-8951	8882	0,36345	0,23585	90
8858-8976	8907	0,35397	0,24633	94
8883-9001	8951	0,38117	0,25682	98
8908-9047	8976	0,39329	0,25682	98
8952-9072	9001	0,43366	0,25682	98
8977-9100	9047	0,47582	0,25682	98
9002-9128	9072	0,49040	0,25682	98
9048-9153	9100	0,44234	0,23847	91
9076-9178	9128	0,36717	0,20440	78
9101-9203	9153	0,28148	0,16510	63
9129-9228	9178	0,25018	0,13627	52
9154-9253	9203	0,24302	0,12317	47
9179-9278	9228	0,29926	0,14675	56
9204-9303	9253	0,39828	0,18606	71
9229-9328	9278	0,43520	0,20440	78
9254-9353	9303	0,46043	0,22013	84
9279-9378	9328	0,42794	0,21227	81
9304-9403	9353	0,34680	0,18082	69
9329-9428	9378	0,31268	0,17034	65
9354-9456	9403	0,31495	0,18344	70
9379-9481	9428	0,31649	0,17558	67

9404-9506	9456	0,30905	0,17034	65
9429-9531	9481	0,33846	0,18344	70
9457-9556	9506	0,36474	0,18082	69
9482-9581	9531	0,37409	0,18606	71
9507-9606	9556	0,41680	0,20703	79
9532-9631	9581	0,38729	0,19916	76
9557-9656	9606	0,33397	0,18868	72
9582-9682	9631	0,36265	0,19916	76
9607-9707	9656	0,39702	0,20703	79
9632-9732	9682	0,41942	0,21227	81
9657-9757	9707	0,42769	0,21489	82
9683-9782	9732	0,41295	0,20965	80
9708-9813	9757	0,44028	0,21751	83
9733-9838	9782	0,45477	0,22799	87
9758-9863	9813	0,45129	0,22799	87
9783-9972	9838	0,44849	0,23061	88
9814-10000	9863	0,40588	0,22275	85
9839-10025	9972	0,43240	0,22537	86
9864-10050	10000	0,46803	0,23585	90
9973-10075	10025	0,50923	0,24633	94
10001-10100	10050	0,48935	0,24109	92
10026-10125	10075	0,43302	0,23061	88
10051-10150	10100	0,41551	0,22013	84
10076-10175	10125	0,35055	0,19916	76
10101-10200	10150	0,35295	0,19916	76
10126-10225	10175	0,34849	0,18606	71
10151-10250	10200	0,32557	0,18344	70
10176-10275	10225	0,33588	0,18344	70
10201-10300	10250	0,30006	0,16510	63
10226-10325	10275	0,28292	0,16510	63
10251-10350	10300	0,31788	0,17296	66
10276-10375	10325	0,29166	0,16510	63

10301-10400	10350	0,32508	0,18344	70
10326-10428	10375	0,37788	0,20440	78
10351-10453	10400	0,40194	0,20965	80
10376-10478	10428	0,48462	0,23847	91
10401-10503	10453	0,46317	0,23061	88
10429-10528	10478	0,39852	0,19916	76
10454-10553	10503	0,31542	0,16248	62
10479-10578	10528	0,23422	0,12841	49
10504-10603	10553	0,20957	0,10744	41
10529-10628	10578	0,24111	0,12055	46
10554-10653	10603	0,34535	0,16248	62
10579-10678	10628	0,39646	0,19392	74
10604-10703	10653	0,42175	0,21227	81
10629-10728	10678	0,44409	0,22275	85
10654-10753	10703	0,35018	0,18868	72
10679-10778	10728	0,29671	0,16248	62
10704-10803	10753	0,34508	0,18344	70
10729-10828	10778	0,29738	0,16772	64
10754-10853	10803	0,30129	0,16772	64
10779-10878	10828	0,31812	0,17820	68
10804-10903	10853	0,28735	0,16772	64
10829-10928	10878	0,34708	0,18082	69
10854-10953	10903	0,38117	0,19916	76
10879-10978	10928	0,41203	0,20703	79
10904-11003	10953	0,38043	0,18868	72
10929-11028	10978	0,32098	0,17558	67
10954-11056	11003	0,35452	0,19130	73
10979-11081	11028	0,36243	0,19392	74
11004-11106	11056	0,40588	0,21751	83
11029-11131	11081	0,45742	0,23585	90
11057-11156	11106	0,44914	0,23061	88
11082-11181	11131	0,47449	0,23585	90

11107-11206	11156	0,48662	0,23585	90
11132-11231	11181	0,46815	0,22537	86
11157-11256	11206	0,48258	0,22799	87
11182-11281	11231	0,49637	0,23061	88
11207-11306	11256	0,51828	0,23847	91
11232-11331	11281	0,55178	0,25420	97
11257-11410	11306	0,56520	0,25682	98
11282-11444	11331	0,57738	0,25944	99
11307-11469	11410	0,53548	0,24633	94
11332-11497	11444	0,52649	0,23847	91
11411-11522	11469	0,52028	0,23585	90
11445-11547	11497	0,48862	0,22799	87
11470-11572	11522	0,46769	0,21751	83
11498-11597	11547	0,47372	0,21751	83
11523-11622	11572	0,45203	0,20965	80
11548-11647	11597	0,43215	0,20703	79
11573-11672	11622	0,50135	0,23061	88
11598-11697	11647	0,52326	0,24109	92
11623-11923	11672	0,52920	0,24895	95
11648-11948	11697	0,51514	0,24633	94
11673-11973	11923	0,48015	0,24109	92
11899-11998	11948	0,45480	0,23585	90
11924-12023	11973	0,41354	0,22013	84
11949-12048	11998	0,39000	0,20703	79
11974-12073	12023	0,35852	0,18868	72
11999-12098	12048	0,33951	0,18082	69
12024-12129	12073	0,40012	0,19916	76
12049-12172	12098	0,47498	0,22537	86
12074-12212	12129	0,54166	0,24895	95
12099-12240	12172	0,57422	0,25944	99
12130-12265	12212	0,55203	0,24895	95
12173-12290	12240	0,49286	0,23323	89

12213-12315	12265	0,44612	0,22537	86
12241-12340	12290	0,41763	0,20965	80
12266-12354	12304	0,39520	0,20760	61
