# Supplementary material for: Structure, Evolution, and Mitochondrial Genome Analysis of Mussel Species (Bivalvia, Mytilidae)
Source: Int J Mol Sci. 2024 Jun 24;25(13):6902. doi: 10.3390/ijms25136902 (PMC11241113; doi:10.3390/ijms25136902)
Supplement: Supplementary file 1 [file ijms-25-06902-s001.zip › Table S6a.Nucleot-cont-26PCGs-Mytilidae...pdf]

Table S6. Nucleotide content of 26 mitogenome sequences of PCGs among Mytilidae

Species	Mean frequencies of nucleotides (%) combined for the three codon positions				
	T	C	A	G	Length of sequence (bp)
**Aligned PCG sequences**					
Arcuatula senhousia GU001953	40.9	12.8	24.0	22.2	11212.0
Arcuatula senhousia OR453539	41.2	12.8	23.9	22.1	10971.0
Bathymodiolus childressi NC 059707	40.5	14.5	21.9	23.1	10866.0
Bathymodiolus japonicus AP014560	41.3	13.7	22.5	22.5	10842.0
Bathymodiolus securiformis NC 039552	41.4	13.5	22.5	22.5	10872.0
Brachidontes exustus NC 024882	40.9	13.8	25.0	20.3	11100.0
Crenomytilus grayanus NC 044128	36.3	13.8	24.7	25.2	11298.0
Gregariella coralliophaga NC 044129	40.1	12.1	26.9	20.9	11181.0
Modiolus kurilensis NC 036486	40.8	12.0	23.1	24.1	10929.0
Modiolus modiolus KX821782	40.6	12.1	22.8	24.4	10839.0
Mytilisepta keenae NC 044127	45.2	9.5	23.1	22.2	10914.0
Mytilus californianus JX486124	36.8	13.4	26.2	23.6	11301.0
Mytilus chilensis KP100300	35.4	15.0	25.2	24.5	11297.0
Mytilus chilensis NC 030633	35.4	15.0	25.2	24.5	11297.0
Mytilus coruscus KJ577549	36.1	14.0	25.8	24.0	11294.0
Mytilus coruscus OR453540	36.3	13.9	25.8	24.0	10959.0
Mytilus edulis MF407676	35.3	15.0	25.2	24.5	11451.0
Mytilus galloprovincialis FJ890849	35.3	15.0	25.1	24.6	11451.0
Mytilus trossulus GU936625	34.5	15.5	24.9	25.1	11451.0
Mytilus trossulus HM462080	34.5	15.5	25.0	25.1	11451.0
Perna canaliculus MG766134	40.5	12.5	26.3	20.8	11022.0
Perna canaliculus MK775558	40.5	12.5	26.1	20.8	10914.0
Perna perna OK576481	40.4	12.8	25.9	20.9	10911.0
Perna viridis JQ970425	42.4	10.2	24.7	22.7	11004.0

Perna viridis MW727515	42.4	10.2	24.7	22.7	11004.0
Septifer bilocularis NC 044131	44.8	11.1	24.6	19.5	11250.0
**Average, n=26**	**39.18±1.48**	**13.18±0.79**	**24.67±0.32**	**22.96±0.69**	**11118.5**
**Average, n=26; T+C, A+G**	**26.18±1.14**		**23.82±0.50**		**11118.5**

Note. Standard errors for average frequencies and heterogeneity of nucleotide content among four nucleotide types (T, C, A, G) are estimated by ANOVA/MANOVA testing of two subsets as given in the table for aligned sequences. Heterogeneity of nucleotide frequencies between four types of nucleotides is statistically significant for 26 mitogenomes set: Wilk's Lambda=0.0054. F=801. d.f.=6;380. *P*<00001. Average frequencies of 4 nucleotide types for 26 sequences do not differ: Wilk's Lambda=0.9981. F=0. d.f.=6;380. *P*<0.9992.
