# Supplementary material for: Structure, Evolution, and Mitochondrial Genome Analysis of Mussel Species (Bivalvia, Mytilidae)
Source: Int J Mol Sci. 2024 Jun 24;25(13):6902. doi: 10.3390/ijms25136902 (PMC11241113; doi:10.3390/ijms25136902)
Supplement: Supplementary file 1 [file ijms-25-06902-s001.zip › Table S9.Tests of substitution saturation+phylog informativness, Mytilidae PCGs mitogenome.pdf]

Table 8S. Test of substitution saturation for Pleuronectoidei PCGs mitogenome sequences

```
Test of substitution saturation (Xia et al. 2003; Xia and Lemey 2009)

Analysis performed on fully resolved sites only.

Testing whether the observed Iss is significantly
lower than Iss.c.

Part I. For a symmetrical tree.
========================================================
Prop. invar. sites                  0,1600
Mean H                              1,0818
Standard Error                      0,0062
Hmax                                1,8076
Iss                                 0,5985
Iss.c                               0,8432
T                                  39,1625
DF                             8443
Prob (Two-tailed)                   0,0000
95% Lower Limit                     0,5862
95% Upper Limit                     0,6107

========================================================
Part II. For an extreme asymmetrical (and generally very
unlikely) tree.
========================================================
Iss.c                               0,6256
T                                   4,3423
DF                             8443
Prob (Two-tailed)                   0,0000

95% Lower Limit                     0,5862
95% Upper Limit                     0,6107

========================================================

Interpretation of results:
               Significant Difference
               ----------------------
               Yes                 No
```

Iss < Iss.c	Little saturation	Substantial saturation
Iss > Iss.c	Useless sequences	Very poor for phylogenetics

Test the presence of phylogenetic signal by the method of Steel et al. (1993) with modifications.

Test of the null model of no phylogenetic information

Quartet	c	X2	Prob
3,1,11,10	1291	4,74	0,0937
18,2,4,18	5044	9800,18	0,0000
10,7,3,7	1791	3537,14	0,0000
12,1,6,6	895	1793,62	0,0000
1,7,16,23	1426	301,93	0,0000
14,6,20,20	3168	6227,72	0,0000
7,5,20,8	1350	9,30	0,0096
11,19,0,5	1306	3,24	0,1976
12,3,22,23	1607	3173,87	0,0000
7,19,0,18	1452	226,72	0,0000
2,10,8,5	1652	741,62	0,0000
7,0,1,24	1917	3545,85	0,0000
20,6,0,23	1351	468,30	0,0000
9,6,5,5	1657	3271,93	0,0000
9,6,19,0	1362	4,85	0,0883
15,23,20,24	1525	676,08	0,0000
18,18,1,16	565	1125,43	0,0000
15,11,0,10	1654	1335,88	0,0000
22,8,7,3	1623	508,28	0,0000
5,23,3,22	1706	3363,15	0,0000
4,16,9,3	1193	1357,48	0,0000
23,11,9,14	1602	1757,64	0,0000
19,13,8,6	1687	1316,23	0,0000
7,17,1,17	1617	3140,51	0,0000
7,15,14,14	963	1933,88	0,0000

14,11,9,7	1191	817,96	0,0000
7,22,15,4	1405	198,73	0,0000
3,3,19,14	1961	3891,39	0,0000
14,6,11,13	722	1037,95	0,0000
1,2,10,10	1558	3064,89	0,0000
23,20,2,2	3173	6272,55	0,0000
10,9,21,24	1372	32,59	0,0000
15,14,2,18	1027	433,88	0,0000
2,1,5,24	1288	6,83	0,0329
15,21,24,8	1430	9,86	0,0072
24,20,1,13	1326	25,34	0,0000
9,20,20,4	2516	4981,03	0,0000
17,11,14,8	832	48,20	0,0000
6,22,18,13	915	15,40	0,0005
23,4,9,6	1608	462,96	0,0000
0,10,6,3	1383	5,73	0,0570
1,12,17,24	1619	1786,44	0,0000
6,0,1,15	3239	6091,02	0,0000
15,8,3,7	2006	1181,96	0,0000
0,3,5,2	1705	2569,75	0,0000
18,20,24,19	1665	3278,27	0,0000
3,14,22,16	1581	1187,06	0,0000
17,12,20,3	1730	1805,45	0,0000
24,22,18,21	1503	698,95	0,0000
10,2,0,19	1417	23,40	0,0000
17,3,11,2	3504	5931,34	0,0000
24,24,4,10	1731	3447,21	0,0000
22,24,5,8	1395	30,87	0,0000
2,11,18,8	2502	2916,85	0,0000
24,14,5,12	1514	1044,23	0,0000
15,12,7,2	1424	2528,09	0,0000
22,20,5,21	854	157,69	0,0000
0,3,6,23	1309	11,84	0,0027
7,24,22,19	1503	756,46	0,0000
24,24,9,9	5413	10558,24	0,0000
10,3,13,23	1311	5,56	0,0621
17,16,0,12	641	476,14	0,0000
5,17,10,0	1391	142,36	0,0000
15,13,24,17	921	297,46	0,0000
3,23,7,6	1353	186,17	0,0000
20,21,16,0	1384	908,92	0,0000

12,9,0,17	1652	1787,48	0,0000
12,15,21,18	674	607,84	0,0000
24,10,21,21	1702	3355,83	0,0000
10,18,0,20	1305	0,23	0,8911
5,19,3,8	1449	361,42	0,0000
15,21,0,11	1724	1314,05	0,0000
3,0,17,0	1822	3646,44	0,0000
17,15,7,12	677	712,69	0,0000
16,1,11,21	1736	1385,37	0,0000
10,4,18,0	1358	9,76	0,0076
0,13,18,5	1629	1192,89	0,0000
4,23,22,21	943	1866,23	0,0000
9,13,24,10	1433	79,55	0,0000
13,19,14,22	2874	5447,93	0,0000
7,22,0,5	1346	12,14	0,0023
14,15,10,23	1610	1224,28	0,0000
4,20,21,9	2184	2675,83	0,0000
18,24,6,19	1608	964,30	0,0000
22,8,22,0	1447	2883,21	0,0000
22,14,19,2	1338	543,96	0,0000
3,19,15,8	1657	481,24	0,0000
5,13,20,15	1615	1175,18	0,0000
0,8,24,21	1315	2,50	0,2864
9,3,2,21	1196	1438,06	0,0000
13,11,23,4	1699	1791,17	0,0000
7,7,12,13	2489	4883,70	0,0000
1,14,0,6	3216	6143,11	0,0000
11,22,19,1	1374	489,15	0,0000
22,7,18,1	1466	288,38	0,0000
14,11,22,1	1742	1858,11	0,0000
18,1,3,24	1338	18,72	0,0001
23,13,12,20	2590	3106,94	0,0000
1,19,7,9	1254	0,83	0,6607
24,9,17,20	1383	9,30	0,0096
22,0,17,0	1867	3759,16	0,0000
19,4,3,8	1187	1452,64	0,0000
1,20,24,17	1358	27,90	0,0000
14,19,23,19	956	1915,18	0,0000
20,23,6,15	2531	2834,25	0,0000
6,1,20,7	1367	234,18	0,0000
5,12,14,23	1553	1050,36	0,0000

19,13,22,4	1384	544,55	0,0000
20,12,15,7	1530	2706,14	0,0000
19,18,24,10	1383	3,84	0,1467
1,4,0,6	1599	2941,11	0,0000
2,19,3,8	1203	1401,89	0,0000
16,17,22,20	2919	4039,95	0,0000
3,24,5,21	1331	21,28	0,0000
9,11,12,24	1470	1070,26	0,0000
12,0,9,2	1550	438,27	0,0000
6,18,11,3	837	8,45	0,0146
8,20,11,21	1445	1011,71	0,0000
6,4,11,18	837	15,58	0,0004
1,20,15,6	1712	1159,10	0,0000
18,3,20,14	1543	1033,65	0,0000
14,13,20,0	1797	3317,07	0,0000
23,10,16,10	1608	3158,62	0,0000
2,2,4,3	546	1090,73	0,0000
13,14,20,14	186	372,19	0,0000
9,17,17,4	3083	5992,68	0,0000
1,13,0,4	1566	2942,15	0,0000
7,2,14,1	1334	163,75	0,0000
9,0,14,23	1342	7,64	0,0220
11,2,19,8	1610	456,92	0,0000
8,2,19,9	1181	1203,69	0,0000
12,3,13,20	1667	1203,52	0,0000
3,18,10,15	1934	2197,90	0,0000
19,17,22,22	997	1972,12	0,0000
22,1,8,16	1372	6,54	0,0380
20,22,3,7	1267	502,21	0,0000
1,3,22,23	1491	2956,93	0,0000
16,21,6,8	1604	1106,75	0,0000
24,9,10,21	1372	32,59	0,0000
14,8,15,17	1003	468,41	0,0000
11,0,21,8	1354	1,66	0,4364
12,2,5,13	1641	1292,18	0,0000
4,12,0,3	1795	2680,88	0,0000
11,14,11,11	0	9999,00	1,0000
2,7,9,15	1991	1137,55	0,0000
2,4,10,13	1763	2683,39	0,0000
6,0,8,22	1354	10,75	0,0046
6,0,12,6	894	1791,35	0,0000

22,5,20,18	1419	482,85	0,0000
10,3,19,16	1391	11,63	0,0030
6,18,20,18	1031	2046,51	0,0000
3,8,18,2	1178	1319,20	0,0000
24,5,18,24	1469	2857,34	0,0000
17,7,11,12	862	124,40	0,0000
13,18,3,12	814	136,42	0,0000
10,10,10,2	0	9999,00	1,0000
21,2,24,21	1520	3038,97	0,0000
10,3,19,19	1781	3530,89	0,0000
15,10,7,9	1423	290,65	0,0000
19,11,1,18	1737	1381,90	0,0000
2,19,15,1	1335	0,14	0,9321
8,12,11,17	790	144,92	0,0000
7,9,11,18	1284	584,82	0,0000
0,21,11,17	1712	1326,27	0,0000
17,11,24,17	963	1886,69	0,0000
10,9,8,11	1719	2313,75	0,0000
16,11,3,4	3546	6114,47	0,0000
6,7,15,22	1320	559,05	0,0000
21,16,15,23	3238	6040,72	0,0000
23,1,15,17	1986	2404,33	0,0000
23,17,0,15	1941	2324,67	0,0000
22,15,20,14	2471	2882,45	0,0000
22,1,10,8	1371	30,15	0,0000
8,2,15,4	1171	1351,49	0,0000
17,13,0,3	1594	1115,48	0,0000
13,1,14,4	1717	3109,66	0,0000
15,17,11,15	714	1430,04	0,0000
10,6,1,0	1681	3001,85	0,0000
20,12,10,4	1397	12,66	0,0018
0,24,16,8	1392	14,27	0,0008
21,17,1,19	1416	900,15	0,0000
2,15,20,12	1862	3491,60	0,0000
6,11,15,7	889	6,08	0,0479
12,1,2,0	1596	2947,56	0,0000
20,16,19,2	1430	2762,23	0,0000
16,10,24,5	1497	154,97	0,0000
8,13,8,10	1905	3857,91	0,0000
6,5,3,11	1669	1402,76	0,0000
23,4,12,1	1333	5,88	0,0528

14,4,0,23	1289	1,44	0,4870
16,0,13,16	883	1759,49	0,0000
24,14,8,0	1360	6,67	0,0356
22,7,5,10	1406	134,10	0,0000
14,18,13,19	883	1010,47	0,0000
24,14,10,10	1752	3487,33	0,0000
13,15,10,11	803	99,00	0,0000
14,6,9,5	1581	1211,00	0,0000
17,3,10,1	1356	4,43	0,1093
8,0,10,7	1361	19,47	0,0001
0,22,10,17	1326	4,86	0,0882
5,11,11,16	754	1513,61	0,0000
2,14,21,7	1321	127,15	0,0000
1,11,7,4	1342	246,18	0,0000
18,10,14,22	1611	1253,76	0,0000
4,16,9,6	2428	2645,92	0,0000
8,17,5,8	1880	3750,56	0,0000
17,5,0,5	1751	3486,12	0,0000
9,7,12,13	1271	626,35	0,0000
4,21,3,20	3436	6042,61	0,0000
2,5,2,6	1826	3648,47	0,0000
1,22,17,19	1367	475,25	0,0000
20,24,22,7	1511	741,64	0,0000
3,6,3,6	5590	10965,39	0,0000
10,4,11,8	1540	387,83	0,0000
4,9,21,11	1562	509,42	0,0000
2,1,19,3	1873	2758,14	0,0000
5,8,22,22	1704	3400,42	0,0000
12,22,11,4	1698	1347,05	0,0000
8,18,5,4	1551	388,69	0,0000
15,12,10,5	2416	4357,35	0,0000
21,20,8,5	1522	1270,88	0,0000
21,21,10,14	1512	3051,69	0,0000
3,7,18,1	1330	229,27	0,0000
24,11,9,24	1539	2994,92	0,0000
18,16,11,24	896	214,84	0,0000
5,22,0,16	1316	10,53	0,0052
8,17,13,11	754	54,13	0,0000
1,8,9,3	1153	1166,52	0,0000
19,1,15,4	1323	2,06	0,3563
14,0,4,16	1496	1043,60	0,0000

24,19,4,0	1329	12,27	0,0022
24,19,11,15	1611	1248,41	0,0000
3,14,9,2	1130	1364,43	0,0000
3,12,0,3	1913	3815,67	0,0000
10,15,8,15	2277	4385,30	0,0000
2,17,22,15	1844	2258,40	0,0000
6,21,15,4	1569	1051,33	0,0000
10,22,23,24	1687	3353,86	0,0000
3,13,23,9	1565	443,13	0,0000
24,7,4,14	1286	121,94	0,0000
20,21,10,5	1795	1860,42	0,0000
4,0,14,6	1445	1042,35	0,0000
0,5,5,17	1751	3486,12	0,0000
14,23,3,4	1824	2730,71	0,0000
18,22,1,5	1292	7,54	0,0231
9,21,20,5	1504	1282,09	0,0000
0,11,15,4	1631	1246,35	0,0000
23,7,18,9	1446	225,64	0,0000
5,8,3,11	1550	346,30	0,0000
6,18,20,11	857	14,62	0,0007
13,8,21,18	1681	1208,39	0,0000
11,6,6,8	835	1668,44	0,0000
15,0,13,9	1624	1206,83	0,0000
7,3,24,19	1289	13,85	0,0010
0,6,22,12	1772	1330,76	0,0000
17,1,5,14	1498	1035,52	0,0000
16,19,6,19	3254	6365,51	0,0000
6,21,23,7	1757	1253,59	0,0000
9,18,23,19	1407	430,27	0,0000
14,0,9,17	1517	965,64	0,0000
7,24,17,15	1509	1378,77	0,0000
2,24,14,6	1396	981,57	0,0000
14,17,10,7	1228	572,83	0,0000
5,21,20,16	1469	987,49	0,0000
16,24,10,5	1497	154,97	0,0000
1,19,2,7	1205	2,96	0,2274
23,4,1,21	1266	437,12	0,0000
23,11,18,17	946	1797,43	0,0000
4,13,6,1	1591	1301,48	0,0000
16,18,20,4	1829	2015,42	0,0000
0,14,21,15	1576	1156,34	0,0000

8,0,16,5	1343	11,90	0,0026
13,16,6,21	900	18,33	0,0001
16,17,10,0	1846	2116,79	0,0000
16,12,23,24	1882	3318,22	0,0000
8,21,10,23	1503	734,62	0,0000
16,13,20,16	942	1874,89	0,0000
23,16,19,2	1384	496,77	0,0000
20,18,21,12	3165	4689,29	0,0000
1,18,24,19	1335	20,91	0,0000
18,16,12,16	152	304,02	0,0000
21,23,2,24	1375	521,84	0,0000
22,9,5,4	1504	302,84	0,0000
19,13,15,22	2608	3125,80	0,0000
4,18,1,11	1625	1347,29	0,0000
21,0,17,11	1712	1326,27	0,0000
9,21,1,0	1599	2965,11	0,0000
4,9,20,20	2516	4981,03	0,0000
16,18,17,18	35	70,00	0,0000
15,18,6,17	718	1328,50	0,0000
2,17,13,11	781	37,59	0,0000
3,18,11,3	3799	7430,62	0,0000
2,22,16,13	1692	1355,80	0,0000
5,2,16,22	1374	6,75	0,0342
2,19,3,3	347	695,44	0,0000
17,4,11,22	1699	1348,47	0,0000
17,17,13,1	979	1940,54	0,0000
21,10,1,17	1324	3,99	0,1361
16,5,13,23	1656	1207,31	0,0000
6,18,2,4	3373	5667,80	0,0000
22,20,5,21	854	157,69	0,0000
0,4,21,22	1263	425,70	0,0000
8,7,18,23	1478	219,07	0,0000
11,11,23,13	589	1180,94	0,0000
7,6,18,16	1005	326,05	0,0000
11,5,17,13	773	58,28	0,0000
24,16,22,14	1529	1093,81	0,0000
3,20,18,14	1543	1033,65	0,0000
22,6,11,12	843	12,19	0,0023
4,14,11,24	1504	1490,63	0,0000
14,3,13,10	1832	3352,70	0,0000

10,11,12,7	1285	767,53	0,0000
20,6,22,15	2532	2831,24	0,0000
8,12,6,19	1655	1130,58	0,0000
11,21,7,10	1370	166,01	0,0000
15,7,12,21	1516	2704,43	0,0000
13,0,24,12	1508	1047,59	0,0000
13,13,14,17	156	312,07	0,0000
4,3,4,15	310	619,80	0,0000
10,11,6,17	832	18,65	0,0001
17,12,17,23	723	1437,80	0,0000
10,11,24,21	1331	7,41	0,0246
12,15,8,12	69	138,02	0,0000
16,10,13,12	797	1052,63	0,0000
6,22,11,21	2554	3093,92	0,0000
0,7,9,14	1384	134,72	0,0000
13,1,7,14	1314	2261,17	0,0000
10,19,8,5	1355	70,30	0,0000
8,17,19,15	1857	2173,10	0,0000
13,10,24,9	1433	79,55	0,0000
13,16,18,20	943	249,25	0,0000
3,11,0,2	1807	2597,79	0,0000
10,15,13,15	902	1792,47	0,0000
13,6,1,3	1580	1267,85	0,0000
2,17,14,8	2373	2587,01	0,0000
8,16,24,12	1766	3139,91	0,0000
3,16,20,17	1844	2034,06	0,0000
17,15,9,21	1834	2067,53	0,0000
24,19,2,12	1385	9,19	0,0101
23,18,2,3	1826	2662,06	0,0000
11,0,22,21	1329	488,94	0,0000
0,14,18,22	1710	1255,10	0,0000
6,19,17,22	2585	2777,22	0,0000
0,9,7,24	1333	24,11	0,0000
12,4,10,6	1676	1253,26	0,0000
18,10,9,20	1426	32,23	0,0000
5,19,2,11	1359	3,68	0,1589
24,12,22,4	1345	14,36	0,0008
18,21,5,12	1669	1725,55	0,0000
8,15,15,10	2277	4385,30	0,0000
10,3,6,8	1507	392,56	0,0000
10,6,10,5	1384	2697,60	0,0000

21,1,7,7	1777	3545,61	0,0000
14,0,2,20	1258	1,83	0,4011
15,10,10,20	1709	3309,37	0,0000
4,1,16,18	1796	2111,75	0,0000
17,23,2,16	1849	2234,57	0,0000
10,16,19,6	1618	1065,03	0,0000
23,13,23,15	3427	6639,08	0,0000
10,14,14,18	833	1684,45	0,0000
13,0,2,21	1267	1,96	0,3762
2,19,19,8	2585	5110,62	0,0000
16,12,16,14	304	607,94	0,0000
11,17,11,9	671	1345,65	0,0000
19,24,5,9	1336	16,08	0,0003
3,23,10,21	1473	625,29	0,0000
7,18,10,20	1390	141,76	0,0000
23,14,20,2	1321	531,25	0,0000
1,21,11,23	1334	481,82	0,0000
6,22,11,23	3424	6671,84	0,0000
17,20,12,18	727	1381,76	0,0000
17,16,11,14	1232	667,22	0,0000
7,21,5,15	1365	121,46	0,0000
11,18,18,21	963	1900,71	0,0000
21,14,8,24	1369	4,07	0,1308
22,14,9,19	1384	456,68	0,0000
18,9,1,15	1758	2080,10	0,0000
24,17,9,3	1691	634,14	0,0000
3,2,15,9	1155	1333,71	0,0000
20,6,21,22	882	110,20	0,0000
18,6,19,11	860	15,01	0,0006
6,9,20,17	1643	1090,83	0,0000
1,19,8,2	1626	505,26	0,0000
4,20,10,9	1532	385,01	0,0000
0,9,13,22	1360	4,79	0,0910
2,12,16,12	123	246,03	0,0000
10,4,16,12	2020	3431,21	0,0000
18,18,20,22	3321	6405,17	0,0000
12,0,14,23	1701	1334,25	0,0000
11,2,20,23	1369	522,93	0,0000
7,3,2,5	1795	2694,49	0,0000
16,15,1,9	1959	3593,59	0,0000
8,15,13,20	1688	1327,03	0,0000

13,12,11,13	835	1671,26	0,0000
23,20,7,3	1264	506,09	0,0000
8,10,10,1	1475	2914,52	0,0000
23,22,5,1	1544	3049,82	0,0000
22,5,24,3	1350	24,97	0,0000
23,11,5,9	1399	8,03	0,0180
8,6,11,8	3832	7566,31	0,0000
0,21,18,10	1321	0,31	0,8547
6,0,24,19	1354	25,62	0,0000
2,19,3,8	1203	1401,89	0,0000
12,14,22,22	3311	6418,58	0,0000
12,17,9,21	1730	1837,12	0,0000
14,24,8,20	1402	7,65	0,0218
11,13,11,15	730	1458,05	0,0000
0,24,19,18	1370	30,06	0,0000
13,22,19,17	2621	3067,51	0,0000
2,9,7,19	1581	511,98	0,0000
7,21,21,2	1465	2939,30	0,0000
4,11,9,0	1585	453,20	0,0000
14,4,18,9	2305	2552,02	0,0000
8,17,4,11	2518	2932,20	0,0000
4,2,9,23	1234	1628,80	0,0000
16,2,4,4	278	554,25	0,0000
7,13,8,17	1292	607,24	0,0000
0,19,15,24	1363	29,23	0,0000
23,21,3,12	1430	507,98	0,0000
7,9,24,22	1444	31,39	0,0000
14,4,22,16	1580	1155,66	0,0000
2,16,13,16	929	1854,80	0,0000
14,23,16,10	1606	1220,74	0,0000
4,22,20,4	3178	6318,27	0,0000
16,13,1,12	777	1091,52	0,0000
21,14,2,0	1256	1,17	0,5569
10,12,16,13	797	1052,63	0,0000
17,15,23,2	1842	2250,10	0,0000
23,9,14,17	1558	1153,29	0,0000
19,2,16,23	1384	496,77	0,0000
0,22,0,0	0	9999,00	1,0000
11,16,21,5	1592	1204,01	0,0000
1,16,14,1	3462	6788,46	0,0000
14,0,21,10	1347	0,41	0,8145

4,16,24,6	1496	933,60	0,0000
7,11,8,3	2023	1188,05	0,0000
24,15,9,14	1437	915,06	0,0000
7,8,16,19	1446	181,10	0,0000
23,16,0,21	1377	472,95	0,0000
9,16,10,21	1399	32,31	0,0000
22,18,14,11	866	65,10	0,0000
2,9,3,15	1155	1333,71	0,0000
15,0,21,17	1870	2080,44	0,0000
1,3,9,13	1532	450,15	0,0000
5,17,0,6	1655	1073,70	0,0000
13,16,17,19	955	257,12	0,0000
18,2,9,22	1598	418,38	0,0000
22,3,18,16	1906	2171,01	0,0000
15,12,18,18	1688	3373,66	0,0000
7,24,24,6	2039	3975,79	0,0000
3,11,21,21	1503	3005,97	0,0000
20,0,5,21	1396	1074,49	0,0000
24,8,2,20	1653	490,97	0,0000
9,8,22,10	1745	2472,06	0,0000
20,23,18,4	1370	468,65	0,0000
23,11,21,2	1354	503,13	0,0000
13,18,16,21	987	241,74	0,0000
1,0,15,19	1698	2923,47	0,0000
2,16,18,19	1846	2060,62	0,0000
13,7,24,14	1456	2578,70	0,0000
18,9,7,7	1158	2290,17	0,0000
19,15,12,5	1868	3454,96	0,0000
13,24,18,19	1624	1161,20	0,0000
8,20,1,18	1382	0,21	0,8989
4,6,0,23	1322	11,48	0,0032
8,21,4,19	2231	2691,61	0,0000
24,11,1,12	1587	1166,14	0,0000
2,8,20,6	1602	448,71	0,0000
20,14,6,18	979	1,34	0,5109
16,20,15,19	4911	9579,34	0,0000
10,24,2,16	1423	35,44	0,0000
0,13,9,2	1545	426,59	0,0000
21,20,12,23	872	106,18	0,0000
24,19,21,19	752	1504,14	0,0000
3,16,13,2	3457	5843,61	0,0000

10,8,23,11	1376	6,11	0,0472
16,18,16,2	503	1008,37	0,0000
21,5,9,14	1350	14,70	0,0006
18,19,9,22	1412	424,86	0,0000
23,22,1,18	1531	3025,32	0,0000
24,6,18,23	1543	1060,99	0,0000
23,24,19,6	1489	716,73	0,0000
0,24,20,13	1324	27,30	0,0000
9,16,11,16	943	1876,87	0,0000
17,14,3,11	868	28,31	0,0000
7,2,23,4	1959	2995,49	0,0000
24,2,7,13	1307	105,21	0,0000
23,5,5,4	1768	3519,15	0,0000
0,6,9,23	1358	10,75	0,0046
3,21,1,20	1277	1039,70	0,0000
24,5,13,21	1360	1,29	0,5245
23,12,13,11	777	81,35	0,0000
8,23,8,21	3234	6443,38	0,0000
23,18,12,1	1878	2187,72	0,0000
13,18,11,14	734	1228,57	0,0000
24,17,0,22	1383	44,12	0,0000
8,16,2,24	1732	675,90	0,0000
10,14,23,23	1544	3122,24	0,0000
11,5,8,2	1560	354,29	0,0000
23,5,9,7	1419	25,89	0,0000
23,15,23,8	1545	3058,70	0,0000
15,10,5,20	1443	117,40	0,0000
19,12,15,1	1984	3638,07	0,0000
22,16,15,12	273	165,32	0,0000
8,6,13,3	2467	2914,39	0,0000
18,12,12,4	526	1055,47	0,0000
21,16,23,8	1381	455,17	0,0000
7,0,1,19	1720	3077,94	0,0000
23,8,15,22	1543	3036,49	0,0000
7,10,5,22	1406	134,10	0,0000
4,2,8,0	1128	1400,38	0,0000
17,10,4,12	1875	1992,64	0,0000
11,19,8,9	1865	2503,95	0,0000
0,22,23,1	4448	8852,59	0,0000
14,10,24,13	1802	3144,20	0,0000
23,6,19,9	1398	480,91	0,0000

18,22,0,14	1710	1255,10	0,0000
21,0,15,23	1366	473,22	0,0000
2,10,7,11	1501	259,18	0,0000
4,5,21,9	1434	347,88	0,0000
1,1,4,18	1705	3409,50	0,0000
2,0,10,2	1952	3876,54	0,0000
0,5,1,18	1589	2887,46	0,0000
19,4,16,17	1825	2021,44	0,0000
19,1,18,9	1344	1,36	0,5077
5,12,17,10	2177	2611,87	0,0000
23,4,4,11	1952	3891,54	0,0000
8,8,19,18	2051	4088,27	0,0000
14,21,18,6	998	1,35	0,5093
23,4,1,21	1266	437,12	0,0000
15,21,21,0	1447	2888,66	0,0000
18,1,23,5	1292	7,54	0,0230
9,16,12,10	2090	3626,98	0,0000
6,3,12,20	1610	1032,07	0,0000
3,3,15,0	1923	3835,65	0,0000
1,1,3,1	0	9999,00	1,0000
10,24,15,0	1363	10,73	0,0047
9,15,1,21	1330	0,47	0,7886
18,3,2,9	1179	1337,77	0,0000
16,4,17,11	911	267,55	0,0000
1,11,23,15	1830	1561,53	0,0000
22,5,5,19	3108	6197,82	0,0000
1,3,6,5	1279	7,04	0,0296
5,16,1,22	1302	16,44	0,0003
24,3,13,24	1505	2937,79	0,0000
10,16,13,15	880	1440,81	0,0000
20,19,8,21	651	1122,56	0,0000
12,21,2,0	1308	1,22	0,5429
2,5,18,2	1833	3663,54	0,0000
0,0,24,18	2100	4279,26	0,0000
3,23,19,24	1389	575,96	0,0000
17,11,18,18	28	56,01	0,0000
16,4,2,5	1744	2603,96	0,0000
23,14,17,17	1107	2186,33	0,0000
6,16,7,12	920	1311,37	0,0000
4,8,8,8	0	9999,00	1,0000
0,18,12,21	1801	1898,47	0,0000

14,20,0,6	1642	1122,44	0,0000
19,7,21,11	1942	1971,42	0,0000
12,17,8,5	1822	2106,61	0,0000
21,21,24,19	690	1379,65	0,0000
19,15,15,14	1065	2118,84	0,0000
11,11,15,6	861	1718,15	0,0000
13,22,13,0	2110	4138,58	0,0000
3,19,22,23	906	1805,99	0,0000
12,23,10,22	1593	3218,93	0,0000
9,24,2,14	1616	552,30	0,0000
11,16,23,18	932	234,39	0,0000
1,24,21,15	1342	23,08	0,0000
23,2,13,20	1325	544,69	0,0000
21,0,23,7	1333	453,13	0,0000
13,21,21,14	4612	9091,90	0,0000
23,4,15,8	1658	467,43	0,0000
14,15,8,3	2352	2595,52	0,0000
9,21,4,11	1562	509,42	0,0000
11,16,19,23	2636	3191,59	0,0000
13,7,8,20	1416	148,95	0,0000
5,7,13,23	1352	116,58	0,0000
19,2,23,13	1342	557,54	0,0000
2,11,11,12	719	1437,50	0,0000
19,15,7,2	1383	172,44	0,0000
20,3,0,13	1265	3,86	0,1451
3,15,18,2	4033	6965,54	0,0000
12,23,23,6	3301	6398,44	0,0000
20,15,2,10	1414	10,17	0,0062
15,11,21,12	811	1331,04	0,0000
17,12,6,1	891	180,74	0,0000
1,24,12,9	1348	12,20	0,0022
2,24,14,7	1293	108,65	0,0000
3,19,12,16	1824	3264,20	0,0000
1,1,12,12	5412	10571,31	0,0000
9,19,16,7	1427	183,05	0,0000
6,16,20,11	843	7,40	0,0247
0,22,14,0	1906	3824,85	0,0000
0,24,14,21	1246	13,63	0,0011
9,18,3,18	3068	5968,37	0,0000
6,24,3,22	1375	13,55	0,0011
8,21,0,24	1315	2,50	0,2864

15,0,0,18	4369	8521,41	0,0000
2,12,0,14	1472	981,30	0,0000
6,7,3,23	1353	186,17	0,0000
8,23,22,0	1444	2865,51	0,0000
3,8,13,0	1564	493,89	0,0000
23,24,13,1	1338	38,68	0,0000
16,10,16,24	2093	3928,21	0,0000
10,15,16,1	2096	3748,92	0,0000
3,5,0,20	1357	25,81	0,0000
12,9,6,15	840	1402,78	0,0000
5,6,18,21	1600	1020,75	0,0000
10,19,24,1	1282	5,63	0,0600
10,8,19,13	1405	26,39	0,0000
16,0,21,22	1381	470,85	0,0000
19,1,18,14	1667	1134,68	0,0000
6,9,16,9	3765	7409,54	0,0000
1,11,4,19	1253	3,63	0,1625
12,16,10,23	2045	3547,01	0,0000
13,8,5,8	1942	3865,86	0,0000
5,18,23,15	1849	2048,94	0,0000
8,19,24,11	1439	8,63	0,0134
10,3,5,20	1387	71,24	0,0000
24,18,1,17	1985	3702,20	0,0000
19,9,3,13	1624	451,82	0,0000
20,13,2,16	1663	1266,72	0,0000
0,15,19,1	1698	2923,47	0,0000
17,7,6,8	1281	482,90	0,0000
16,2,23,6	1681	1206,32	0,0000
6,10,19,19	1664	3344,90	0,0000
4,14,20,8	1594	488,03	0,0000
15,4,6,8	2451	2734,93	0,0000
21,22,6,1	1335	467,05	0,0000
19,5,11,11	1895	3742,18	0,0000
24,14,10,13	1802	3144,20	0,0000
0,23,13,23	1547	3068,29	0,0000
15,3,4,13	3519	5992,97	0,0000
15,1,20,14	1668	1177,92	0,0000
20,15,19,23	877	1566,62	0,0000
5,5,0,15	1735	3456,21	0,0000
10,6,3,11	1682	1400,42	0,0000
8,6,22,6	2076	4077,13	0,0000

6,20,12,23	2528	2833,81	0,0000
23,0,18,3	1341	1,65	0,4379
24,3,19,23	1389	575,96	0,0000
19,11,20,21	700	1225,34	0,0000
5,14,7,5	2333	4646,58	0,0000
2,14,24,12	1440	910,79	0,0000
8,18,0,6	1632	983,47	0,0000
23,12,2,1	1328	6,40	0,0408
19,14,5,18	1514	1066,28	0,0000
21,8,10,24	1393	29,69	0,0000
17,9,23,24	1413	31,34	0,0000
4,11,9,16	2496	2808,34	0,0000
8,18,12,16	660	401,37	0,0000
16,1,20,0	1673	3015,55	0,0000
13,14,23,24	1779	3154,88	0,0000
24,17,16,21	1783	1890,37	0,0000
11,5,16,11	754	1513,61	0,0000
4,17,7,11	1244	604,75	0,0000
16,3,18,6	979	356,21	0,0000
4,19,4,0	2069	4120,32	0,0000
0,9,13,20	1320	1,52	0,4681
8,11,4,6	2466	2912,62	0,0000
24,1,4,4	1942	3872,00	0,0000
21,14,8,19	1445	910,26	0,0000
3,22,1,16	1319	5,83	0,0543
8,20,1,16	1356	0,14	0,9310
19,17,9,7	1421	171,69	0,0000
6,5,21,24	1368	0,61	0,7384
5,18,14,5	3422	6676,72	0,0000
14,13,18,4	835	859,06	0,0000
12,12,19,9	1909	3758,69	0,0000
24,0,17,19	1381	31,63	0,0000
20,0,13,22	1312	441,30	0,0000
17,14,7,14	966	1946,27	0,0000
23,8,0,14	1327	8,40	0,0150
20,13,2,13	1907	3774,74	0,0000
20,3,19,10	1688	3188,45	0,0000
17,2,2,21	1987	3958,10	0,0000
0,4,24,3	1841	2766,34	0,0000
19,17,11,22	2650	3197,13	0,0000
7,19,6,16	1317	481,05	0,0000

17,16,18,7	638	1159,16	0,0000
1,8,20,16	1356	0,14	0,9310
4,14,12,18	926	225,03	0,0000
17,21,7,22	1818	1252,14	0,0000
4,23,24,8	1659	556,20	0,0000
11,9,23,23	1577	3132,54	0,0000
15,12,5,4	1928	3548,03	0,0000
15,12,8,12	69	138,02	0,0000
18,22,23,19	997	1960,45	0,0000
0,0,23,1	369	737,88	0,0000
20,1,20,15	1465	2923,72	0,0000
21,4,6,5	1338	8,06	0,0178
9,19,24,15	1390	8,37	0,0152
18,8,18,8	5837	11348,12	0,0000
22,3,17,16	1905	2177,57	0,0000
13,11,0,23	1850	2000,71	0,0000
5,1,16,2	1309	8,78	0,0124
20,15,12,7	1530	2706,14	0,0000
3,20,15,4	1873	2783,14	0,0000
2,8,21,22	2062	1903,14	0,0000
18,11,20,17	943	1783,20	0,0000
18,9,14,21	1516	1017,98	0,0000
1,10,12,8	1356	14,76	0,0006
2,23,9,12	1613	386,18	0,0000
14,23,12,6	980	1,80	0,4057
6,10,17,24	1542	1044,89	0,0000
13,14,14,13	623	1245,98	0,0000
9,17,6,12	841	229,85	0,0000
16,17,1,17	568	1131,11	0,0000
8,19,16,0	1374	1,65	0,4383
13,2,7,7	1245	2469,70	0,0000
2,5,14,18	1557	1133,09	0,0000
3,7,3,7	5338	10517,94	0,0000
6,17,16,13	1198	484,78	0,0000
9,3,9,15	1403	2806,97	0,0000
15,11,15,12	229	457,97	0,0000
12,8,11,14	862	44,34	0,0000
12,19,1,9	1329	0,14	0,9302
16,24,19,14	1541	1037,59	0,0000
12,14,20,4	1584	1046,46	0,0000
9,22,4,7	1628	500,30	0,0000

19,19,13,14	4747	9346,56	0,0000
3,7,16,12	1413	2355,21	0,0000
16,23,0,12	2077	3646,66	0,0000
18,3,15,10	1934	2197,90	0,0000
2,18,24,11	1502	1186,78	0,0000
22,21,13,3	1339	508,63	0,0000
5,6,6,8	2069	4084,40	0,0000
17,14,8,5	1596	1243,80	0,0000
17,22,12,15	677	640,72	0,0000
4,20,2,6	1847	2810,17	0,0000
12,11,18,24	761	106,87	0,0000
18,22,5,5	1830	3662,22	0,0000
1,21,13,20	1391	961,39	0,0000
15,12,6,0	898	1379,95	0,0000
3,15,24,19	1379	9,29	0,0096
12,2,7,3	2417	3825,94	0,0000
23,8,18,3	1639	477,06	0,0000
23,12,7,21	1826	1294,85	0,0000
24,5,20,11	1345	1,98	0,3709
22,9,22,24	1559	3130,82	0,0000
7,11,24,23	1370	146,66	0,0000
5,16,15,8	2110	3909,24	0,0000
3,22,5,24	1350	24,97	0,0000
3,5,15,1	1261	8,58	0,0137
9,10,9,18	1904	3879,67	0,0000
19,2,24,8	1682	489,67	0,0000
0,13,11,13	607	1211,79	0,0000
16,8,7,20	1439	172,14	0,0000
17,20,8,9	1895	2551,34	0,0000
3,3,6,0	1918	3826,61	0,0000
4,9,2,12	1175	1368,11	0,0000
2,17,0,0	1814	3614,77	0,0000
23,10,13,22	1560	3155,24	0,0000
3,10,18,19	1374	18,10	0,0001
12,18,21,16	671	462,93	0,0000
9,14,2,17	2280	2465,89	0,0000
3,5,21,16	1363	21,43	0,0000
20,19,21,4	624	1090,27	0,0000
3,7,18,22	1395	215,62	0,0000
18,9,3,1	1525	470,20	0,0000
12,13,11,4	771	74,99	0,0000

10,6,6,3	2081	4044,20	0,0000
9,0,2,10	1526	346,18	0,0000
13,22,24,16	1591	1227,27	0,0000
14,23,16,7	1274	580,56	0,0000
14,6,0,19	1670	1151,04	0,0000
22,0,9,16	1402	8,12	0,0173
11,1,16,5	1609	1292,07	0,0000
0,4,5,2	1721	2600,91	0,0000
0,8,5,12	1321	8,66	0,0132
14,18,9,8	3322	5356,54	0,0000
11,1,13,18	791	68,99	0,0000
21,6,23,21	857	1713,92	0,0000
21,10,10,22	2894	5731,42	0,0000
10,20,3,9	1504	374,09	0,0000
17,10,5,12	2177	2611,87	0,0000
9,12,24,4	1717	605,60	0,0000
9,12,6,22	1691	1275,79	0,0000
22,3,21,23	933	1833,84	0,0000
15,21,12,24	1858	3401,13	0,0000
24,11,19,21	1576	1223,67	0,0000
2,0,18,14	1490	957,52	0,0000
14,22,3,12	1596	1183,33	0,0000
21,8,11,23	1353	516,20	0,0000
11,22,24,24	1532	3014,35	0,0000
21,12,1,8	1377	1,70	0,4281
8,12,17,18	689	1301,93	0,0000
9,1,11,10	1327	9,69	0,0079
19,20,10,21	635	1147,30	0,0000
16,20,15,20	4987	9734,19	0,0000
7,14,14,15	963	1933,88	0,0000
8,13,1,15	1650	1276,96	0,0000
4,14,7,0	1349	186,26	0,0000
21,2,4,21	4425	8757,94	0,0000
20,2,0,7	1215	0,36	0,8354
3,11,2,1	1788	2568,49	0,0000
4,15,23,7	1402	199,09	0,0000
24,16,18,15	606	765,95	0,0000
11,4,14,5	1580	1645,60	0,0000
12,20,16,12	150	300,12	0,0000
11,20,2,20	1496	2980,57	0,0000
20,3,9,12	1596	493,99	0,0000

3,6,24,2	1947	2962,17	0,0000
8,20,1,23	1276	382,52	0,0000
2,4,14,20	1838	2770,46	0,0000
11,7,17,2	1230	564,77	0,0000
24,23,7,22	1809	3605,82	0,0000
20,23,22,16	1031	2028,84	0,0000
5,24,13,22	1400	0,75	0,6872
3,8,7,7	2718	5365,51	0,0000
8,4,19,18	1624	518,08	0,0000
9,23,0,2	1598	528,45	0,0000
10,18,10,22	1644	3226,15	0,0000
11,13,4,5	1701	1853,20	0,0000
19,4,23,2	2955	5060,70	0,0000
17,18,12,2	718	1337,15	0,0000
8,5,20,20	1665	3328,26	0,0000
5,12,14,6	942	3,07	0,2153
5,6,14,17	941	4,59	0,1009
16,14,15,14	2719	5430,13	0,0000
1,13,15,1	3596	7047,19	0,0000
11,2,21,0	1265	0,99	0,6096
18,2,16,11	913	278,58	0,0000
12,23,0,21	1360	453,02	0,0000
18,23,0,3	1341	1,65	0,4379
12,10,20,10	1744	3377,40	0,0000
17,16,11,13	1258	694,64	0,0000
14,13,13,10	241	481,53	0,0000
22,23,9,21	926	1825,97	0,0000
20,19,10,13	1536	3004,21	0,0000
19,1,13,10	1287	1,48	0,4762
15,9,5,24	1434	37,15	0,0000
7,9,9,7	5466	10749,09	0,0000
7,20,19,20	33	66,00	0,0000
18,18,16,19	590	1174,83	0,0000
14,15,10,15	1010	2004,88	0,0000
23,5,21,19	865	138,44	0,0000
24,10,10,21	1745	3460,77	0,0000
19,8,19,13	1543	3069,47	0,0000
13,19,17,12	858	138,75	0,0000
24,19,24,22	2814	5588,31	0,0000
3,14,1,12	1522	1022,10	0,0000
5,18,6,23	1658	1090,52	0,0000

24,13,22,5	1400	0,75	0,6872
24,14,4,10	1354	23,75	0,0000
7,17,15,23	1525	1376,35	0,0000
17,4,14,9	2299	2545,15	0,0000
14,23,2,11	1587	1665,55	0,0000
15,11,1,11	753	1510,65	0,0000
7,10,7,12	1179	2379,04	0,0000
1,24,12,14	1485	975,37	0,0000
7,3,16,5	1422	253,95	0,0000
23,9,12,7	1431	214,23	0,0000
5,12,15,20	1857	3439,16	0,0000
10,21,0,10	1776	3492,37	0,0000
5,16,3,20	1367	8,07	0,0177
4,17,17,12	711	1415,93	0,0000
7,2,5,20	1327	24,60	0,0000
3,17,11,4	3566	6091,99	0,0000
21,4,8,18	1632	531,11	0,0000
19,22,24,22	988	1970,91	0,0000
3,14,1,14	1812	3560,06	0,0000
20,17,7,6	1356	483,75	0,0000
20,18,9,17	2040	3931,72	0,0000
13,18,1,13	792	1592,39	0,0000
12,11,9,12	809	1610,70	0,0000
8,5,8,8	0	9999,00	1,0000
2,11,15,18	906	290,20	0,0000
9,8,20,19	4186	8230,62	0,0000
11,16,16,4	926	1842,55	0,0000
21,23,13,9	1314	451,11	0,0000
20,22,14,1	1328	463,55	0,0000
14,23,8,4	1573	490,56	0,0000
7,6,13,13	830	1663,45	0,0000
16,19,6,13	908	14,27	0,0008
10,18,3,6	1664	1231,72	0,0000
16,1,2,12	1847	3267,88	0,0000
14,14,0,18	854	1716,96	0,0000
9,22,24,7	1444	31,39	0,0000
14,23,8,24	1371	16,78	0,0002
6,13,17,21	923	17,67	0,0001
2,6,6,1	1894	3731,86	0,0000
22,17,10,8	1436	5,31	0,0702
5,19,13,8	1409	10,11	0,0064

11,8,0,13	1645	1690,58	0,0000
22,2,20,4	2930	5017,60	0,0000
8,21,16,14	1560	1053,05	0,0000
12,17,7,12	636	1277,56	0,0000
12,19,6,3	1608	1025,81	0,0000
7,6,7,0	1165	2316,28	0,0000
1,6,24,5	1377	1,76	0,4149
22,10,12,0	1282	5,68	0,0584
19,10,18,11	1648	1325,28	0,0000
21,0,17,16	1893	2055,59	0,0000
15,1,20,18	1967	2187,50	0,0000
1,22,4,15	1338	4,49	0,1058
3,0,4,14	1790	2710,66	0,0000
8,0,5,11	1290	9,83	0,0073
24,17,1,19	1349	21,72	0,0000
22,9,16,7	1446	231,97	0,0000
11,15,10,20	1654	1266,66	0,0000
21,2,8,2	1256	2502,62	0,0000
3,20,15,7	1394	161,49	0,0000
15,7,14,8	1230	528,99	0,0000
2,12,15,17	620	478,06	0,0000
15,9,18,11	908	289,55	0,0000
14,8,20,13	1753	3205,92	0,0000
15,11,17,6	1130	412,32	0,0000
11,4,4,1	1878	3743,59	0,0000
6,3,9,14	2300	2548,03	0,0000
20,9,3,21	2186	2624,30	0,0000
14,19,3,19	1573	3137,50	0,0000
7,11,19,0	1467	249,37	0,0000
13,0,11,24	1568	1577,96	0,0000
19,10,10,24	1751	3471,88	0,0000
11,11,6,22	767	1535,86	0,0000
15,10,5,20	1443	117,40	0,0000
13,22,11,4	1700	1792,92	0,0000
13,2,15,21	1629	1207,82	0,0000
9,0,14,21	1308	0,31	0,8586
16,6,18,1	993	312,63	0,0000
12,20,2,1	1332	0,26	0,8776
11,6,0,16	905	14,56	0,0007
20,3,24,5	1323	15,10	0,0005
22,14,16,14	832	1672,31	0,0000

0,4,7,20	1211	2,45	0,2932
4,4,4,21	0	9999,00	1,0000
9,21,13,18	1655	1189,25	0,0000
2,3,17,9	1176	1318,55	0,0000
22,12,6,19	2548	2865,92	0,0000
14,3,19,3	1961	3891,39	0,0000
1,7,14,8	1377	157,33	0,0000
5,24,17,20	1373	1,92	0,3821
4,18,12,15	632	505,87	0,0000
17,22,23,10	1553	3142,32	0,0000
0,1,16,8	1646	2880,62	0,0000
11,9,20,12	1652	1273,25	0,0000
4,22,16,3	1839	2738,79	0,0000
24,8,21,22	1383	512,90	0,0000
16,18,15,5	529	640,00	0,0000
10,22,11,4	1374	3,64	0,1624
10,9,17,14	1654	1322,09	0,0000
0,15,2,1	1596	2873,43	0,0000
17,9,14,18	1057	2021,18	0,0000
18,6,5,23	1658	1090,52	0,0000
17,18,22,4	2098	3995,04	0,0000
15,3,10,12	2030	3597,70	0,0000
0,4,20,22	1257	459,60	0,0000
10,14,2,2	1875	3772,19	0,0000
2,21,15,17	1796	2039,15	0,0000
24,13,21,6	1597	1210,27	0,0000
20,16,24,23	1525	670,78	0,0000
18,2,3,6	3357	5539,52	0,0000
22,20,15,23	1027	2020,86	0,0000
9,19,15,12	1891	3471,78	0,0000
12,11,20,10	1640	1306,57	0,0000
4,8,18,4	1195	2380,85	0,0000
16,24,21,1	1349	22,84	0,0000
12,0,6,15	898	1379,95	0,0000
19,15,12,1	1984	3638,07	0,0000
11,3,15,23	1708	1387,61	0,0000
4,23,13,11	1699	1791,17	0,0000
4,15,14,9	2341	2566,85	0,0000
13,8,7,20	1416	148,95	0,0000
13,11,15,22	790	78,32	0,0000
12,12,2,17	508	1018,61	0,0000

13,6,14,16	864	1277,64	0,0000
24,1,20,0	1712	3158,81	0,0000
18,16,22,14	1016	406,54	0,0000
22,21,7,17	1818	1252,14	0,0000
22,21,12,17	2781	3786,64	0,0000
13,14,22,3	1748	3224,61	0,0000
11,5,21,19	1466	983,60	0,0000
0,6,0,15	3589	7048,59	0,0000
23,14,21,22	984	1936,05	0,0000
21,19,14,22	828	109,51	0,0000
12,23,3,16	1947	3445,51	0,0000
13,8,12,19	1695	1328,25	0,0000
12,20,12,23	3170	6173,20	0,0000
22,24,6,9	1374	31,58	0,0000
21,21,14,10	1512	3051,69	0,0000
15,24,5,20	1370	1,10	0,5775
16,6,8,20	1647	1120,26	0,0000
20,14,11,18	886	73,63	0,0000
17,7,10,17	1715	3281,49	0,0000
12,5,22,16	1860	3339,83	0,0000
20,19,18,17	5093	9855,20	0,0000
0,7,0,14	2466	4915,37	0,0000
13,18,24,7	1350	689,73	0,0000
17,11,10,10	3519	6623,41	0,0000
14,14,4,1	1837	3608,28	0,0000
5,4,1,14	1254	10,41	0,0055
4,24,24,6	1519	2957,72	0,0000
2,6,1,10	1339	7,54	0,0230
20,1,10,12	1283	3,04	0,2182
8,9,22,19	2901	4668,75	0,0000
13,1,5,23	1285	14,99	0,0006
18,12,5,9	1820	2084,84	0,0000
15,20,11,1	1775	1394,26	0,0000

==

Sequences ranked from the best to the worst.

==

Seq_Name	Mean_Phi	Num_Insignif
Mytilus_chilensis_KP10030	0,1180	9
Mytilus_edulis_MF407676	0,1142	5

Mytilus_coruscus_OR453540	0,1124	11
Mytilus_trossulus_HM46208	0,1106	9
Mytilus_galloprovincialis	0,1042	4
Mytilus_californianus_JX4	0,1039	8
Bathymodiolus_japonicus_A	0,1014	8
Perna_viridis_MW727515	0,1002	6
Bathymodiolus_childressi_	0,0995	10
Mytilus_trossulus_GU93662	0,0992	6
Perna_canaliculus_MG76613	0,0973	14
Perna_canaliculus_MK77555	0,0965	15
Mytilus_coruscus_KJ577549	0,0958	9
Arcuatula_senhousia_GU001	0,0956	26
Bathymodiolus_securiformi	0,0953	7
Perna_viridis_JQ970425	0,0939	9
Perna_perna_OK576481	0,0931	17
Mytilisepta_keenae_NC_044	0,0925	16
Crenomytilus_grayanus_NC_	0,0890	9
Modiolus_kurilensis_NC_03	0,0867	10
Modiolus_modiolus_KX82178	0,0822	8
Arcuatula_senhousia_OR453	0,0817	23
Septifer_bilocularis_NC_0	0,0765	13
Gregariella_coralliophaga	0,0756	4
Brachidontes_exustus_NC_0	0,0726	12

===

Num_Insignif conditional on c > 15.

Please cite:

Xia, X., Z. Xie, M. Salemi, L. Chen, Y. Wang. 2003. An index of substitution saturation and its application. Molecular Phylogenetics and Evolution 26:1-7.

Xia, X. and Lemey, P. 2009. Assessing substitution saturation with DAMBE. Pp. 615-630 in Philippe Lemey, Marco Salemi and Anne-Mieke Vandamme, eds. The Phylogenetic Handbook: A Practical Approach to DNA and Protein Phylogeny. 2nd edition Cambridge University Press.
