# Supplementary figures and images for: Structure, Evolution, and Mitochondrial Genome Analysis of Mussel Species (Bivalvia, Mytilidae)
Source: Int J Mol Sci. 2024 Jun 24;25(13):6902. doi: 10.3390/ijms25136902 (PMC11241113; doi:10.3390/ijms25136902)

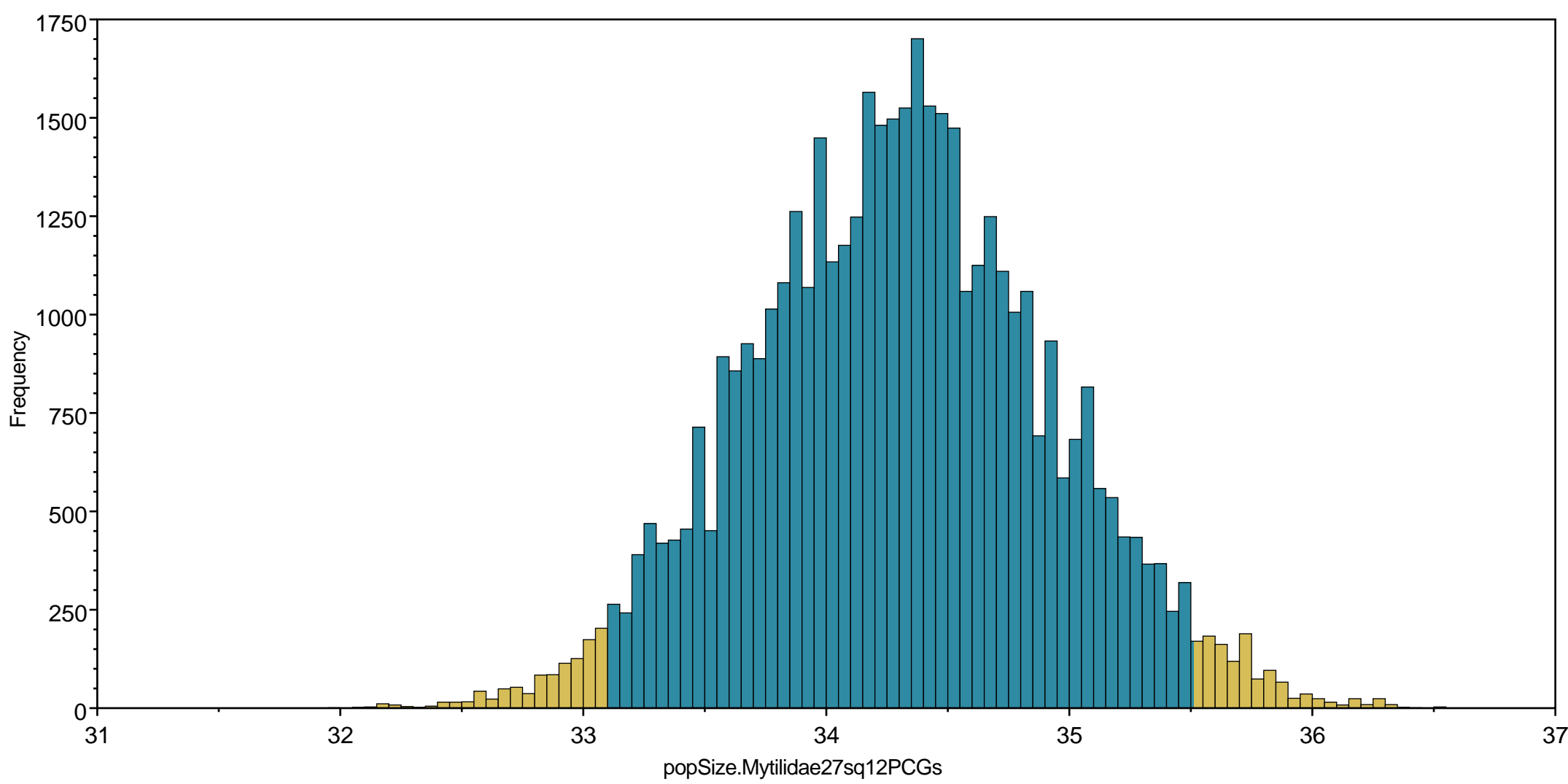

Supplement: Supplementary file 1 [file ijms-25-06902-s001.zip › Tracer_out_for_Fig7/Plot-CA-const-pop-X=34.3,EES=2686.pdf]

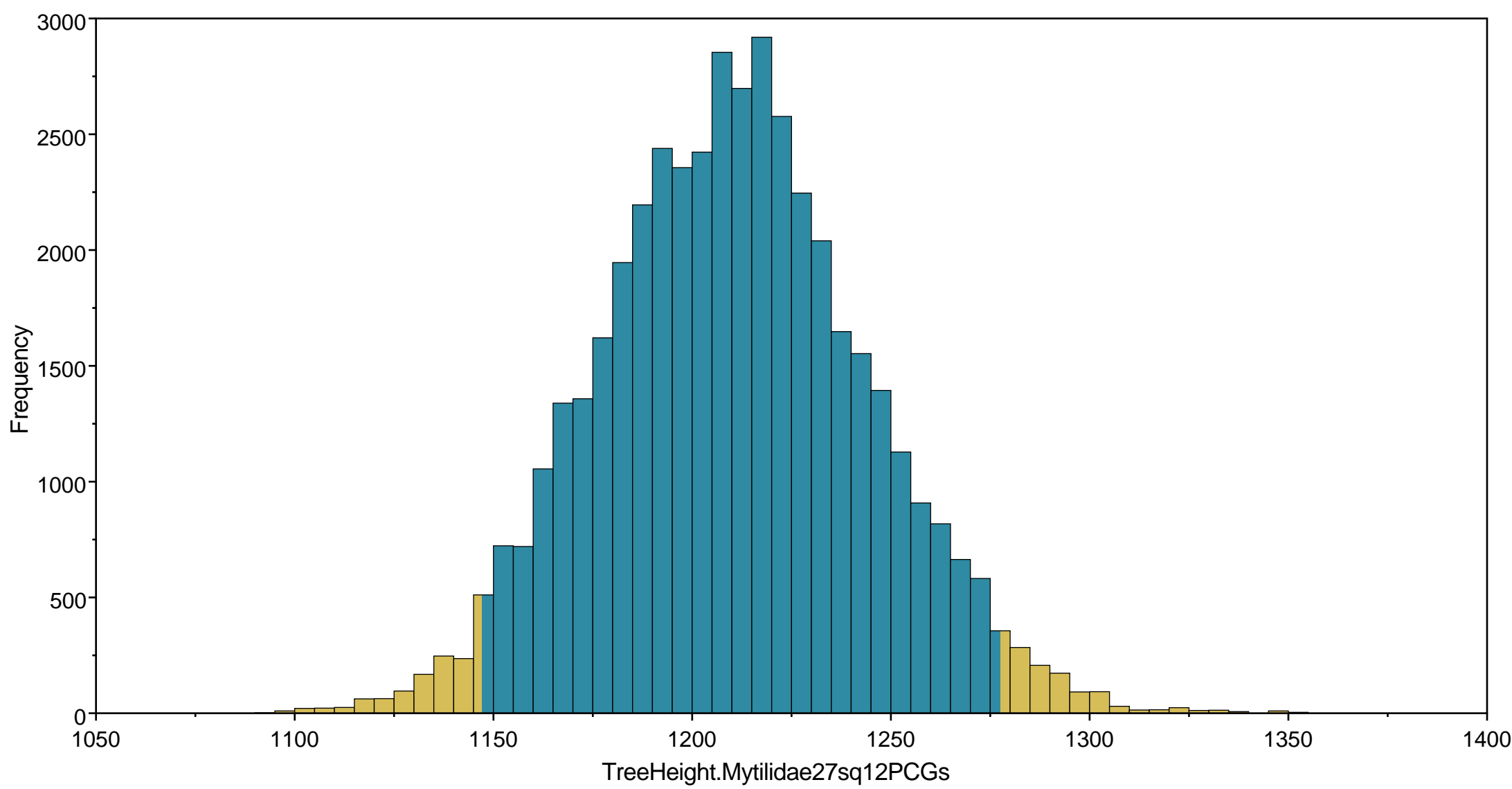

Supplement: Supplementary file 1 [file ijms-25-06902-s001.zip › Tracer_out_for_Fig7/Tracer TreeHeight-Mean=-1210,ESS=852.pdf]

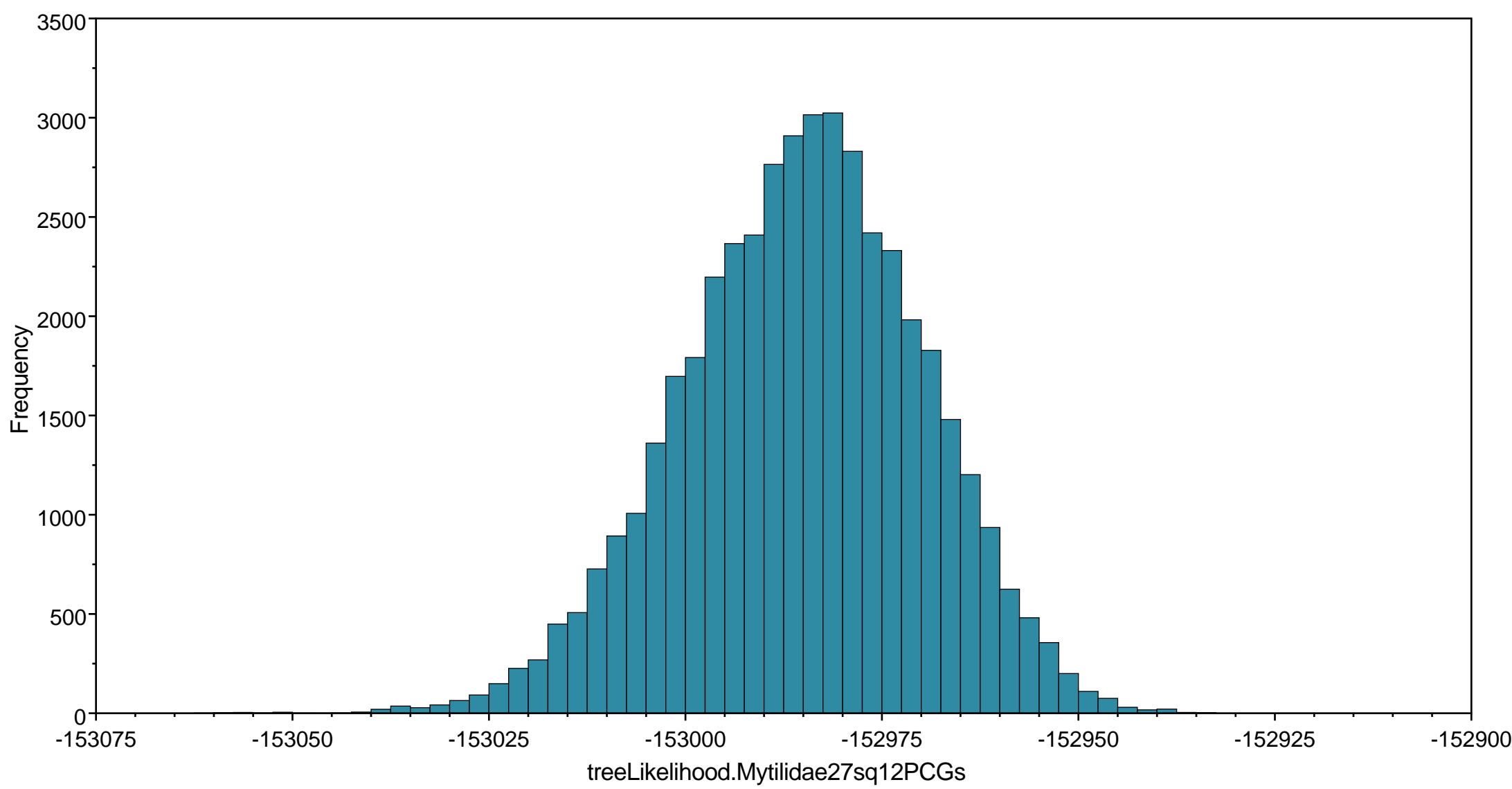

Supplement: Supplementary file 1 [file ijms-25-06902-s001.zip › Tracer_out_for_Fig7/Tracer Treelikelihood-Mean=-1.53E5,ESS=211.pdf]
